# Bioinformatics analysis of IFI6 as a novel prognostic biomarker and its correlation with immune infiltration in breast cancer

**DOI:** 10.1038/s41598-025-20489-6

**Published:** 2025-10-17

**Authors:** Lili Jiang, Chan Xing, Man Li, Zuowei Zhao

**Affiliations:** 1 Department of Breast Surgery & Department of Oncology, The Second Affiliated Hospital of Dalian Medical University, Dalian, 116023 China; 2 International Center for Aging and Cancer, Hainan Academy of Medical Sciences, Hainan Medical University, Haikou, 571199 China

**Keywords:** Bioinformatics analysis, IFI6, Immune infiltration, Breast cancer, Prognosis, Cancer, Immunology

## Abstract

The aim of this study was to identify biomarkers associated with breast cancer prognosis and to explore the underlying pathogenic mechanisms. Interferon alpha-inducible protein 6 (IFI6), known as a proliferative and anti-apoptotic factor, has been implicated in various malignant diseases. However, its biological roles in breast cancer remain poorly understood. To address this, we employed bioinformatics analyses to investigate the expression and prognostic significance of IFI6 in breast cancer. Our findings revealed that IFI6 was upregulated in breast cancer and was associated with histological subtypes and lymph node metastasis status. Kaplan-Meier plotter analysis demonstrated that high IFI6 expression correlated with poor prognosis in breast cancer patients with ER-positive, PR-positive, HER2-positive, and lymph node-positive subtypes. To further enhance clinical applicability, we constructed a prognostic nomogram incorporating IFI6 expression and clinicopathological factors, which showed favorable predictive performance for overall survival. Additionally, IFI6 expression showed significant correlations with infiltrating immune cells, including regulatory T cells (Tregs), M1 macrophages, naïve B cells, and plasma cells. Single-cell RNA sequencing analysis revealed that IFI6 was predominantly expressed in epithelial tumor cells and was associated with altered immune cell composition, suggesting the potential role in shaping the immune microenvironment. Moreover, IFI6 expression was closely associated with several immunomodulators. In conclusion, IFI6 serves as a potential biomarker for immune infiltration and poor prognosis in breast cancer and may offer novel insights into risk stratification and immunotherapeutic strategies.

**Supplementary Information:**

The online version contains supplementary material available at 10.1038/s41598-025-20489-6.

## Introduction

Recent years, the incidence of breast cancer (BC) has surpassed that of lung cancer, making it the most prevalent malignant tumor worldwide. Additionally, it has become the leading cause of cancer-related deaths among women worldwide^1^. Advances in medical care, such as surgery, conventional chemotherapy, precision radiotherapy, endocrine therapy, and monoclonal antibody treatments, have significantly improved the prognosis of breast cancer patients^2^. However, the treatment outcomes for advanced breast cancer remain poor, with many patients continuing to suffer from the threat of recurrence and mortality^3^. Studies have demonstrated that the prognosis and therapeutic responses in breast cancer were significantly correlated with clinical, pathological, and molecular characteristics^4^. Although numerous molecules have been identified as biomarkers for the early diagnosis and prognostic assessment of breast cancer, these biomarkers still exhibit certain limitations, highlighting the urgent need to identify novel and reliable prognostic biomarkers for breast cancer^5^.

Interferon alpha-inducible protein 6 (IFI6), a member of the FAM14 protein family, is primarily localized to the inner mitochondrial membrane and is one of the interferon-stimulated genes^6^. IFI6 has been implicated in several malignancies, including breast and gastric cancers^7^. Previous studies have shown that IFI6 is a target of estrogen signaling and is associated with resistance to tamoxifen treatment^8^. Furthermore, IFI6 has been identified as an anti-apoptotic protein in multiple myeloma and breast cancer^9^. Additionally, Liu et al. reported that higher expression of IFI6 was linked to an immunosuppressive microenvironment in esophageal squamous cell carcinoma^10^. However, its comprehensive role in the tumor microenvironment of breast cancer remains unclear, and few studies have systematically explored its value in predicting immune infiltration and clinical prognosis using integrative computational approaches.

In recent years, machine learning and optimization algorithms have been increasingly applied to improve disease management, early screening, and outcome prediction across various medical domains^11–14^. Among them, prognostic modeling based on molecular and clinical features has become an important strategy in cancer research^15^. Among various statistical approaches, Cox proportional hazards regression remains one of the most widely used and robust methods for evaluating the association between gene expression and patient survival outcomes^16^. Multivariate Cox analysis allows adjustment for potential confounding factors and the identification of independent prognostic markers with clinical relevance. In parallel, nomograms have emerged as effective tools for individualized risk prediction, which also facilitates clinical decision-making and personalized treatment planning^17^. By integrating independent prognostic variables such as gene expression levels and clinicopathological parameters, nomograms provide visual and quantitative representations of patient-specific survival probabilities. Random forest as an ensemble learning method also provides robust variable importance rankings and has shown excellent performance in classification and risk prediction tasks^18^.

While previous studies have associated IFI6 with cancer cell apoptosis, estrogen signaling, and therapy resistance, the broader immunological role of IFI6 within the breast cancer microenvironment remains poorly characterized. Importantly, no studies to date have systematically examined the predictive value of IFI6 in relation to immune cell infiltration and overall prognosis using integrative computational and machine learning approaches. In this study, we comprehensively analyzed the expression and prognostic significance of IFI6 in breast cancer, and developed a nomogram incorporating IFI6 and clinicopathological features for individualized survival prediction. Moreover, we explored the association between IFI6 expression and the immune landscape using CIBERSORT and validated immune associations using multiple algorithms. These findings expand the understanding of IFI6 beyond its previously known roles and underscore its potential as a novel immune-related biomarker for prognostication in breast cancer.

## Results

### Elevated expression of IFI6 in breast cancer

**Fig. 1:**
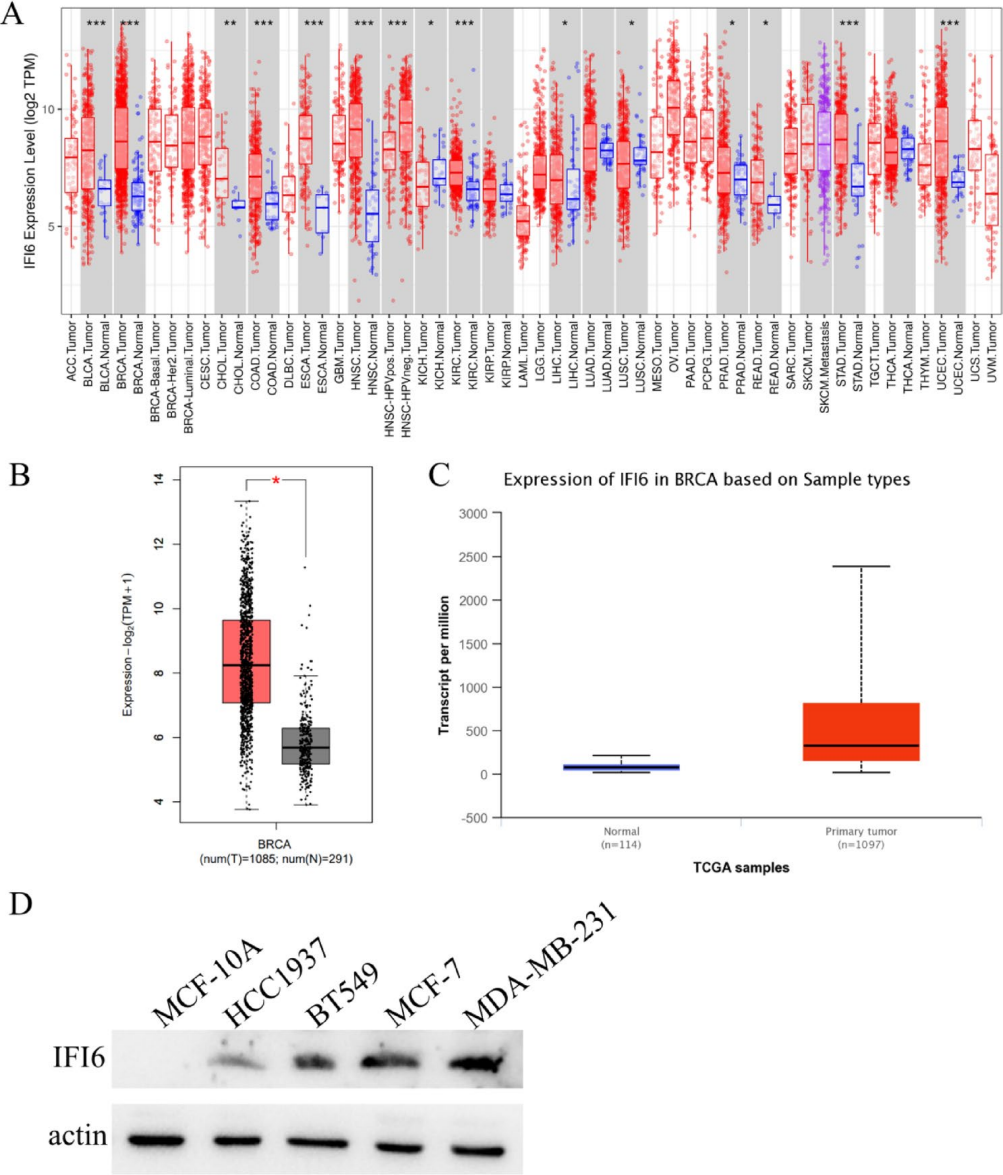
IFI6 was evaluated in breast cancer. (**A**) Differential expression of IFI6 between tumor and adjacent normal tissues across various cancer types (TIMER 2.0). (**B**) IFI6 expression in breast cancer and normal breast tissue (GEPIA). (**C**) IFI6 expression in breast cancer and normal tissue based on the UALCAN database. (**D**) WB analysis showed increased IFI6 protein expression in breast cancer cell lines. (*P < 0.05, **P < 0.01, ***P < 0.001)

The expression of IFI6 in various human cancers was assessed using the TIMER 2.0 database. As depicted in Fig. 1A, IFI6 expression levels were significantly elevated in several cancer types, including bladder urothelial carcinoma (BLCA), breast cancer (BRCA), cholangiocarcinoma (CHOL), colorectal cancer (COAD), esophageal carcinoma (ESCA), head and neck squamous cell carcinoma (HNSC), clear cell carcinoma of kidney (KIRC), hepatocellular carcinoma (LIHC), prostate adenocarcinoma (PRAD), rectum adenocarcinoma (READ), stomach adenocarcinoma (STAD) and uterine corpus endometrial carcinoma (UCEC). In contrast, IFI6 expression was found to be lower in kidney chromophobe (KICH) and lung squamous cell carcinoma (LUSC). Additionally, data from the GEPIA database demonstrated that the mRNA expression levels of IFI6 in BC were significantly higher than those in normal tissues (*P* < 0.05) (Fig. 1B). This higher mRNA expression of IFI6 in breast cancer was further confirmed using the UALCAN cancer database (*P* < 0.001) (Fig. 1C). Furthermore, Western blot (WB) analysis was performed to examine IFI6 expression at the protein level in breast cancer cell lines. As shown in Fig. 1D, IFI6 protein levels were notably higher in HCC1937, BT549, MCF-7 and MDA-MB-231, further substantiating its dysregulation in breast cancer.

### Clinicopathological significance of IFI6 expression in breast cancer

**Fig. 2:**
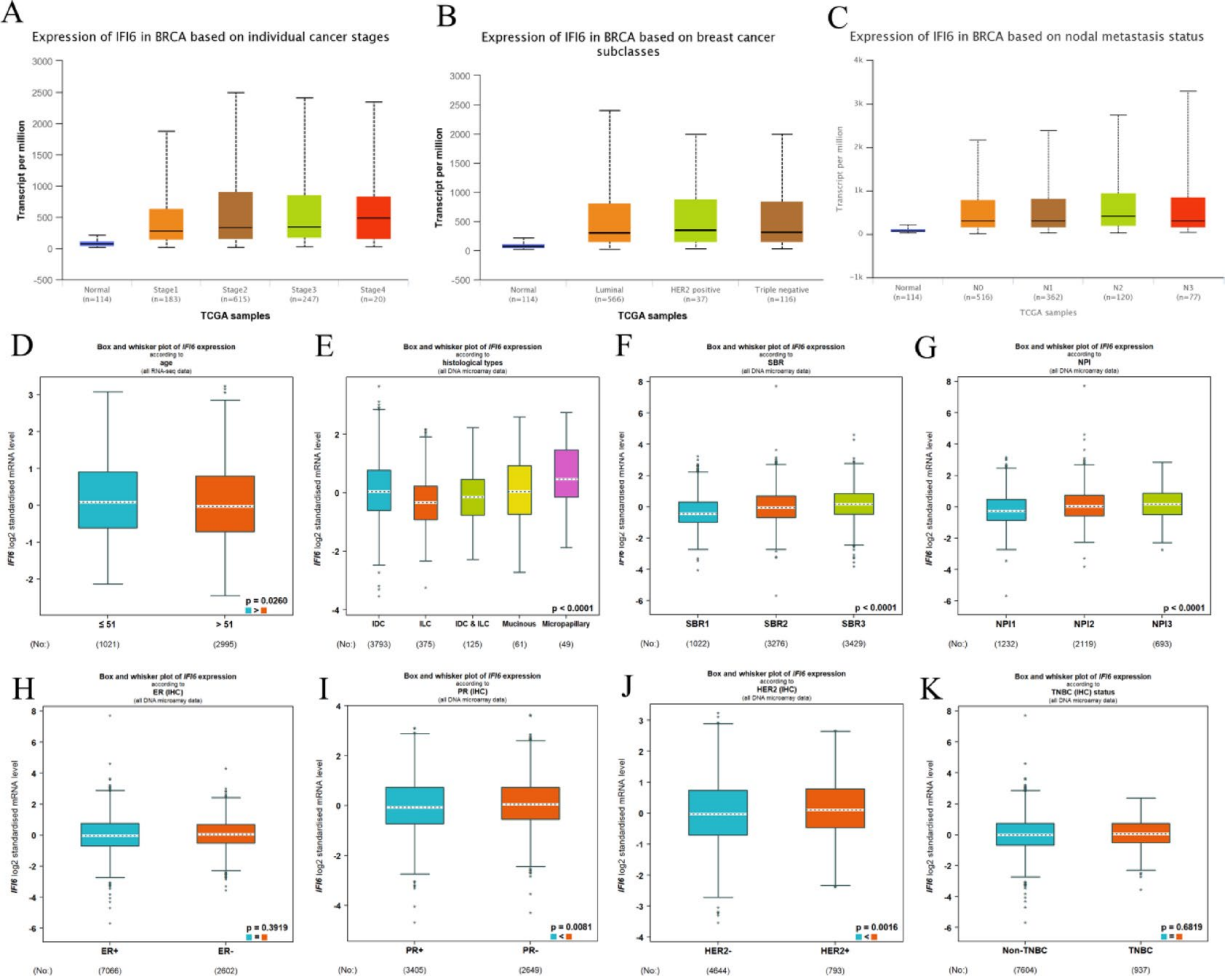
Association of IFI6 expression with clinicopathological features of BC patients. Clinicopathologic features included (**A**) clinical stage; (**B**) molecular subtypes; (**C**) lymph node metastasis status; (**D**) age; (**E**) histological subtype; (**F**) SBR grading; (**G**) NPI; (**H**) ER status; (**I**) PR status; (**J**) HER-2 status; (**K**) TNBC status. (*P < 0.05, **P < 0.01, ***P < 0.001)

The ULCAN database revealed that IFI6 was associated with clinical stages in BC. Specifically, IFI6 expression was significantly upregulated across stages 1 to 4 in BC patients (Fig. 2A). In terms of BC subtypes, IFI6 expression was notably elevated in patients with luminal, HER2-positive, and triple-negative BC (TNBC) subtypes (Fig. 2B). Additionally, IFI6 expression progressively increased according to nodal metastasis status, with higher expression observed in BC patients classified as N0, N1, N2, and N3 (Fig. 2C). Further analysis using the BC-GEM online tool revealed differences in IFI6 expression across various clinical parameters. The results showed that IFI6 expression was significantly associated with age, with higher expression levels in younger BC patients (≤ 51 years, Fig. 2D). Furthermore, IFI6 expression varied across histological types, being higher in micropapillary carcinoma and lower in invasive lobular carcinoma (ILC) (Fig. 2E). BC patients with higher Scarff-Bloom-Richardson (SBR) grades and advanced Nottingham Prognostic Index (NPI) values exhibited significantly elevated IFI6 mRNA levels (Fig. 2F and G). Moreover, IFI6 expression was positively correlated with progesterone receptor (PR) and HER2 status (Fig. 2I and J). However, no significant association was found between IFI6 expression and estrogen receptor (ER) status or TNBC subtypes (Fig. 2H and K).

### Prognostic value of IFI6 expression in breast cancer

**Fig. 3:**
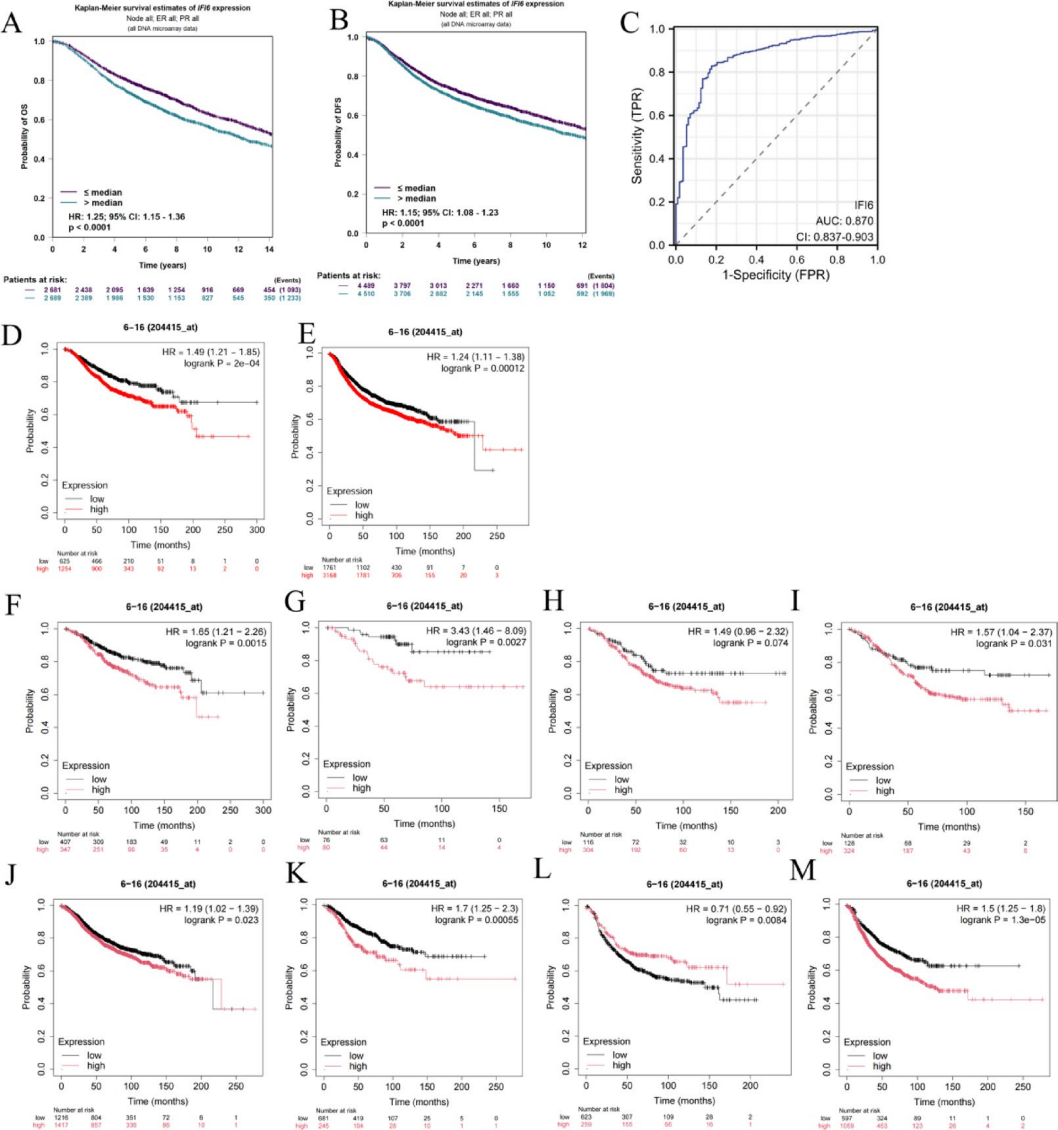
Prognostic significance of IFI6 in BC patients. (**A,B**) The OS (**A**) and RFS (**B**) in BC cohorts obtained through BC-GEM. (**C**) ROC curve. (**D,E**) The OS (**D**) and RFS (**E**) in BC cohorts obtained from the Kaplan-Meier plotter database. (**F–I**) OS curves based on different molecular subtypes: ER-positive (**F**), PR-positive (**G**), HER-2 positive (**H**) and lymph node-positive subtypes (**I**). (**J–M**) RFS curves based on different molecular subtypes: ER-positive (**J**), PR-positive (**K**), HER-2 positive (**L**) and lymph node-positive subtypes (M).

The association between IFI6 expression and prognosis in BC patients was evaluated using the BC-GEM database. Elevated expression of IFI6 was significantly associated with poor overall survival (OS, Fig. 3A) and recurrence-free survival (RFS, Fig. 3B). ROC curve was conducted to assess the diagnostic performance of IFI6, yielding an area under the curve (AUC) value of 0.870 (95% CI: 0.837–0.903; Fig. 3C). Kaplan-Meier plotter analysis further confirmed that higher IFI6 expression correlated with significantly worse OS (Fig. 3D) and RFS (Fig. 3E). Additionally, increased IFI6 expression was linked to poor OS in BC patients with ER-positive (Fig. 3F), PR-positive (Fig. 3G), HER-2-positive (Fig. 3H) and lymph node-positive subtypes (Fig. 3I), as well as poor RFS in patients with ER-positive (Fig. 3J), PR-positive (Fig. 3K), and lymph node-positive subtypes (Fig. 3M). However, no significant association was observed between IFI6 expression and RFS in HER-2-positive patients (Fig. 3L). These findings indicated that IFI6 expression significantly impacted the prognosis of BC patients across various clinicopathological factors.

### Construction and validation of the IFI6-based prognostic model

**Fig. 4:**
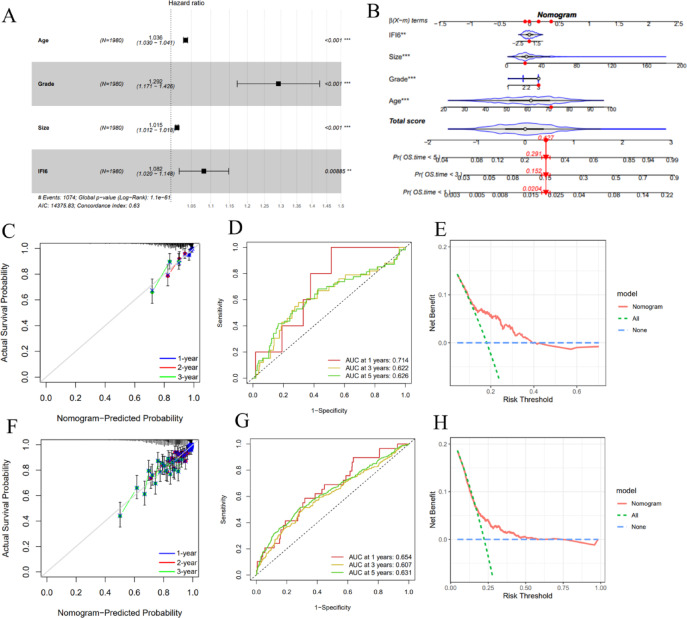
Construction and verification of the predictive model. (**A**) Forest plots of multivariate Cox regression. (**B**) The nomogram predicts prognosis for BC patients. (**C**) The calibration curve evaluates the precision of the nomogram model in forecasting 1-, 3-, and 5-year survival rates. (**D**) Receiver operating characteristic (ROC) curves show the predictive accuracy of the nomogram. (**E**) DCA illustrates the overall improvement of the predictive risk model. (**F–H**) Validation of the prognostic nomogram in external cohorts: calibration curves (**F**), ROC curves (**G**), and DCA curve (**H**).

We further constructed a prognostic model for BC patients via several datasets. The METABRIC cohort was utilized as the training set. Forest plots of multivariate Cox regression revealed that IFI6 (HR: 1.082, 95% CI: 1.020–1.148, *P* = 0.009) was a prognostic predictor independent of BC (Fig. 4A). We also generated a nomogram that combines the clinical factors and IFI6 (Fig. 4B). The calibration curves of internal validation showed good agreement between the nomogram-predicted and observed OS at 1-, 3-, and 5-year time points (Fig. 4C), indicating favorable calibration performance of the model. The AUC values for 1-, 3-, and 5-year OS prediction were 0.714, 0.622, and 0.626 (Fig. 4D), reflecting a moderate discriminatory ability. The decision curve analysis (DCA) curve also indicated that the nomogram provided a certain degree of clinical net benefit (Fig. 4E). In the external validation cohorts (GSE7390 and GSE42568), the nomogram also showed favorable predictive performance. Calibration curves indicated good consistency between the predicted and observed OS at 1, 3, and 5 years (Fig. 4F). The AUCs for predicting 1, 3, and 5-year OS were 0.654, 0.607, and 0.631 (Fig. 4G), indicating moderate discrimination ability. Furthermore, the DCA curve demonstrated that the nomogram provided clinical net benefit across a reasonable range of threshold probabilities, confirming its potential utility in clinical decision-making (Fig. 4H).

### Gene set enrichment analysis (GSEA)

**Fig. 5:**
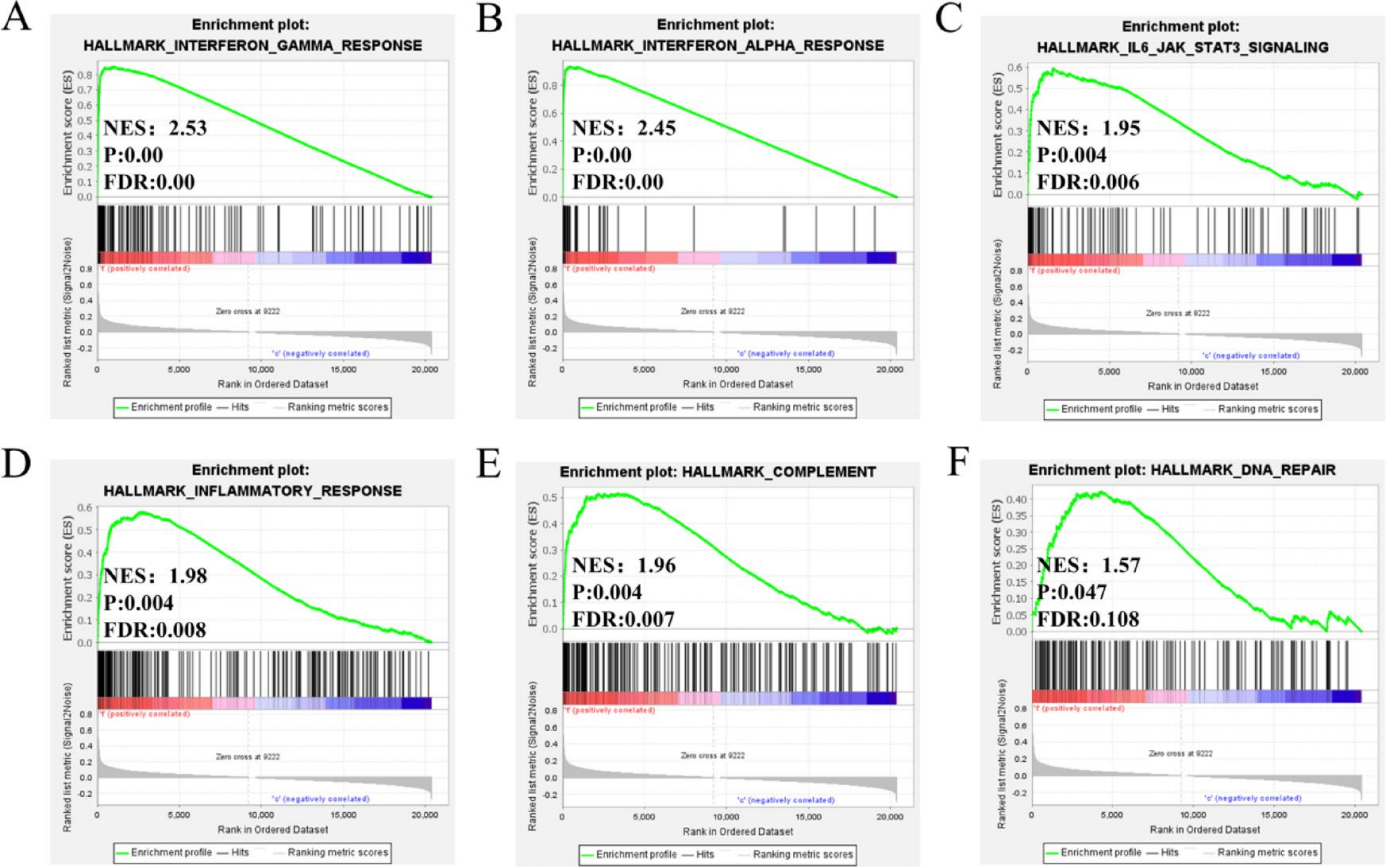
Gene set enrichment analysis of IFI6. The enriched pathways included (**A**) interferon gamma response, (**B**) interferon alpha response, (**C**) IL-6-JAK-STAT3 signaling pathway, (**D**) inflammatory response, (**E**) complement, (**F**) DNA repair.

We further performed GSEA analysis to identify key biological processes involved in IFI6-mediated mechanisms in breast cancer. A detailed enrichment analysis of hallmark pathways was presented in Supplementary Table S1. The crucial pathways included the interferon gamma response (Fig. 5A), the interferon alpha response (Fig. 5B), the IL-6-JAK-STAT3 signaling pathway (Fig. 5C), the inflammatory response (Fig. 5D), the complement (Fig. 5E), and the DNA repair (Fig. 5F). Collectively, these results suggested that the role of IFI6 in tumorigenesis was likely associated with tumor immunology.

### Network construction of IFI6 co-expressed genes in breast cancer

**Fig. 6:**
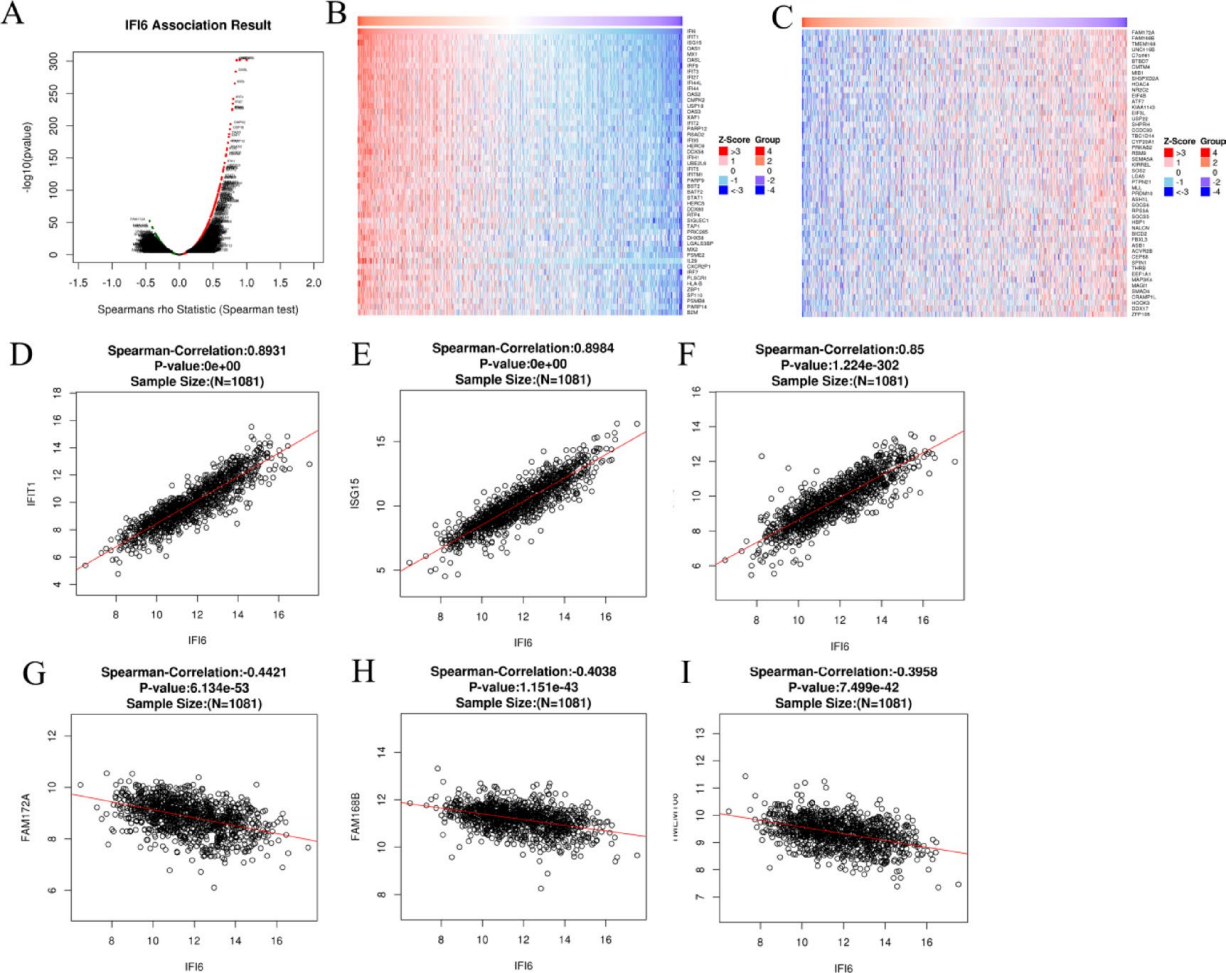
Co-expression network of IFI6. (**A**) Volcano plot of IFI6-associated genes (LinkedOmics). (**B,C**) Heatmaps of the top 50 positively (**B**) and negatively (**C**) genes correlated with IFI6. (**D-F**) Spearman correlations between IFI6 and the top three positively correlated genes: IFIT1, ISG15, and OAS1. (**G-I**) Spearman correlations between IFI6 and the top three negatively correlated genes: FAM172A, TMEM168, and FAM168B. All P-values were adjusted using the Bonferroni correction and Benjamini-Hochberg method.

To investigate the mechanism underlying IFI6 in tumorigenesis and prognosis, co-expressed genes of IFI6 were also identified from the BRCA dataset in TCGA using the LinkedOmics database. The analysis revealed 5785 genes positively correlated and 6534 genes negatively correlated with IFI6 expression, as illustrated in the volcano plot (Fig. 6A, Supplementary Table S2). The top 50 significantly positively and negatively correlated genes were visualized in heat maps (Fig. 6B and C). Among the positively correlated genes, the top three were ISG15 (*r* = 0.898, *P* < 0.05), IFIT1 (*r* = 0.893, *P* < 0.05), and OAS1 (*r* = 0.850, *P* < 0.05) (Fig. 6D and E, and F). Conversely, the top three negatively correlated genes were FAM172A (*r*=-0.442, *P* < 0.05), TMEM168 (*r*=-0.396, *P* < 0.05), and FAM168B (*r*=-0.404, *P* < 0.05) (Fig. 6G and H, and I).

### Enrichment analysis of IFI6 and co-expressed genes

**Fig. 7:**
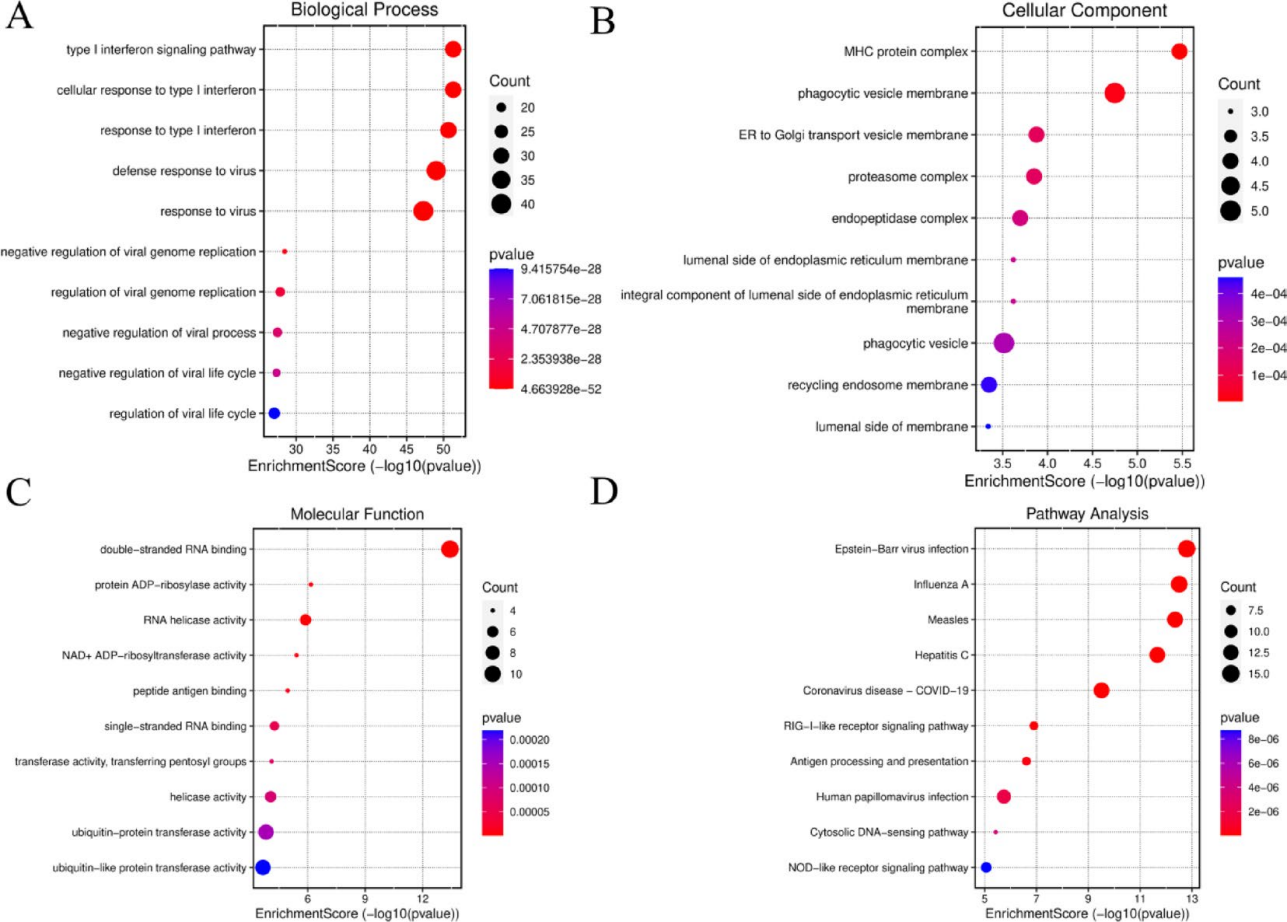
Enrichment analysis of GO and KEGG pathway enrichment of IFI6 co-expressed genes. (**A–C**) GO Enrichment analysis: Biological Process (**A**), Cellular Component (**B**), Molecular Function (**C**); (**D**) KEGG pathway enrichment analysis. Although FDR values were computed, only P-values are shown in the figure for visualization purposes.

We performed Gene Ontology (GO) and Kyoto Encyclopedia of Genes and Genomes (KEGG) pathway enrichment analyses on the top 100 genes most strongly associated with IFI6. The pathways were ranked by P-value. Detailed summaries of the enrichment terms for Biological Process (BP), Cellular Component (CC), Molecular Function (MF), and KEGG pathways are provided in Supplementary Table S3. In the BP analysis, we observed significant enrichment in pathways related to type I interferon signaling, cellular response to type I interferon, and response to type I interferon (Fig. 7A). For CC, the enrichment was mainly observed in the postsynaptic endocytic zone, dendritic spine head, and phagocytic vesicle membrane (Fig. 7B). In the MF category, the most enriched terms included double-stranded RNA binding, single-stranded RNA binding, and RNA helicase activity (Fig. 7C). KEGG pathway analysis revealed significant enrichment of IFI6 co-expressed genes in pathways related to Hepatitis C, Influenza A, Measles, and Epstein-Barr virus infection (Fig. 7D).

### IFI6 is relevant to immune infiltration

**Fig. 8:**
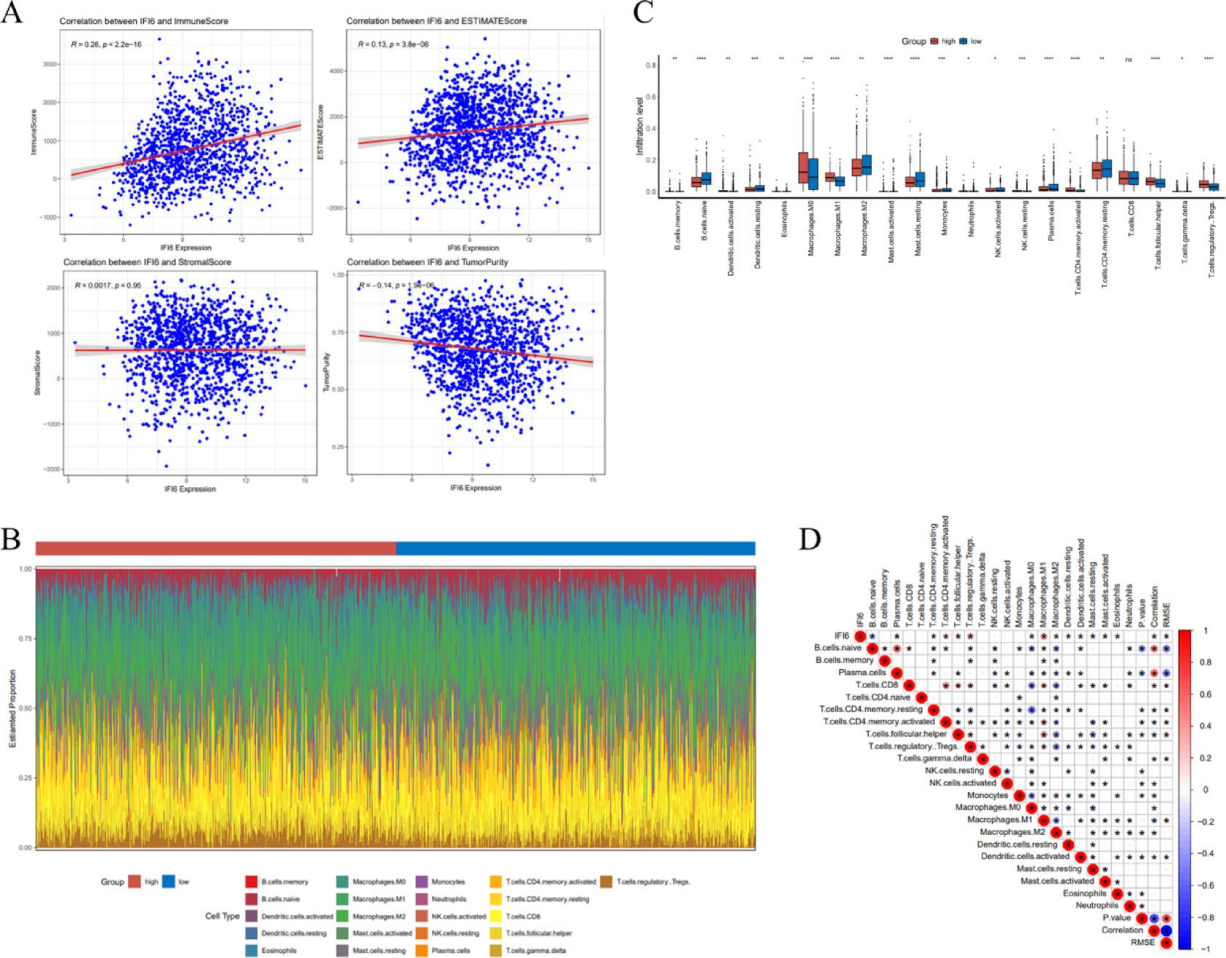
Enrichment analysis of GO and KEGG pathway enrichment of IFI6 co-expressed genes. (**A**) The correlation between IFI6 and tumor microenvironment. (**B**) Heatmap of the proportions of 22 immune cell types in the low and high IFI6 expression groups of cancer samples. (**C**) The box diagram of the proportion of immune cells based on IFI6 expression level. (**D**) The correlation matrix of immune cell and IFI6. All P-values were adjusted using the Benjamini-Hochberg method.

Based on the network of co-expressed genes and enrichment analysis, we hypothesized that IFI6 played a significant role in tumor immunology. For further investigation, the relationship between IFI6 expression and immune infiltration was analyzed via the ESTIMATE algorithm. As shown in Fig. 8A, IFI6 expression was positively correlated with immune score (*r* = 0.26, *P* < 0.05) and ESTIMATE score (*r* = 0.13, *P* < 0.05), but not with stromal score (*r* = 0.0017, *P* > 0.05), indicating its relevance to immune infiltration rather than stromal content. Moreover, IFI6 showed a negative correlation with tumor purity (*r*=-0.14, *P* < 0.05). Additionally, we estimated the proportions of 22 immune cell types in each sample (Fig. 8B). Among these 22 immune cell types, 20 showed significantly different abundances between high and low IFI6 expression groups (Fig. 8C). Furthermore, correlation analyses of infiltrating immune cells were performed, with scores representing the degree of correlation. Moreover, the resulting correlation heatmap indicated that regulatory T cells and M1 macrophages were strongly and positively correlated with IFI6 expression. In contrast, naïve B cells and plasma cells were negatively correlated with IFI6 expression (Fig. 8D and Supplementary Table S4).

### Single-cell RNA sequencing (scRNA-seq) reveals IFI6-associated immune landscape in BC

**Fig. 9:**
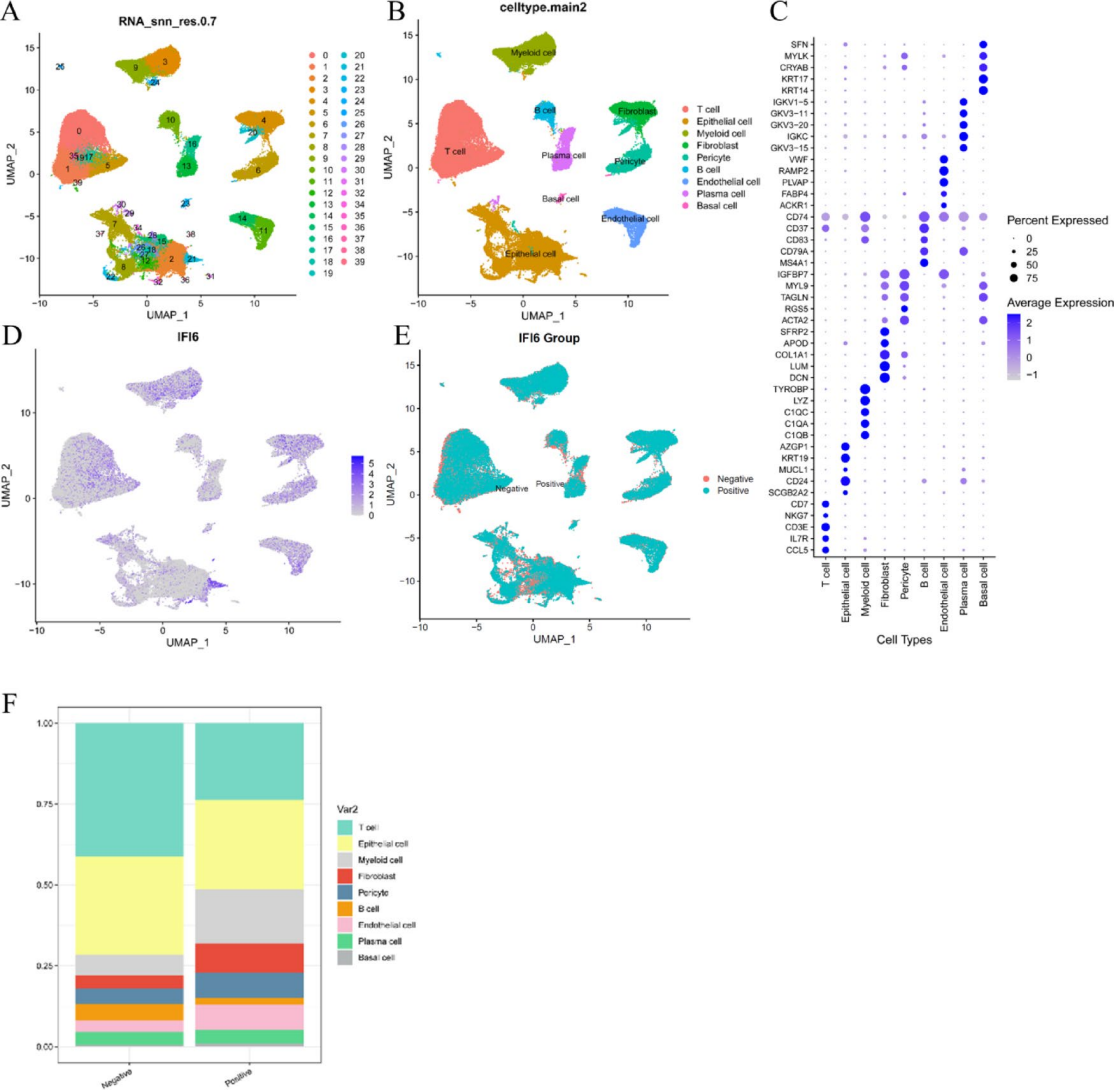
Identification of IFI6-associated immune landscape via scRNA-seq analysis. (**A**) A total of 39 clusters were identified in the dataset. (**B**) UMAP plot displaying the distribution of 9 distinct cell types. (**C**) Heatmap depicting the expression levels of the marker genes specific to each indicated cell type. (**D,E**) UMAP plot illustrating IFI6 heterogeneously expressed among different cell clusters. (**F**) Bar chart highlighting the different proportions of each cell type based IFI6 expression level.

The Seurat package was used to normalize the scRNA-seq data. As shown in the UMAP in Fig. 9A, a total of 794,443 cells were grouped into 39 clusters based on the top 2000 highly variable genes and the top 30 principal components. According to known marker genes, the 39 clusters were annotated into nine major cell types, including T cells, epithelial cells, myeloid cells, fibroblasts, pericytes, B cells, endothelial cells, plasma cells, and basal cells (Fig. 9B). The dot plot (Fig. 9C) visualizes the expression levels and proportions of canonical marker genes across the annotated cell types, further validating the classification.

To explore the immune-related features of IFI6, we mapped its expression across the cell populations. As shown in Fig. 9D, IFI6 was heterogeneously expressed among different cell clusters, with noticeable expression in specific regions. Cells were then dichotomized into IFI6-positive and IFI6-negative groups based on expression levels (Fig. 9E). As visualized in the stacked bar chart (Fig. 9F), IFI6-positive cells were enriched in epithelial cells, while other cell types such as T cells and myeloid cells showed predominantly IFI6-negative status, suggesting high IFI6 expression may be associated with a “cold” immune microenvironment.

### The expression of IFI6 is associated with immunomodulators

**Fig. 10:**
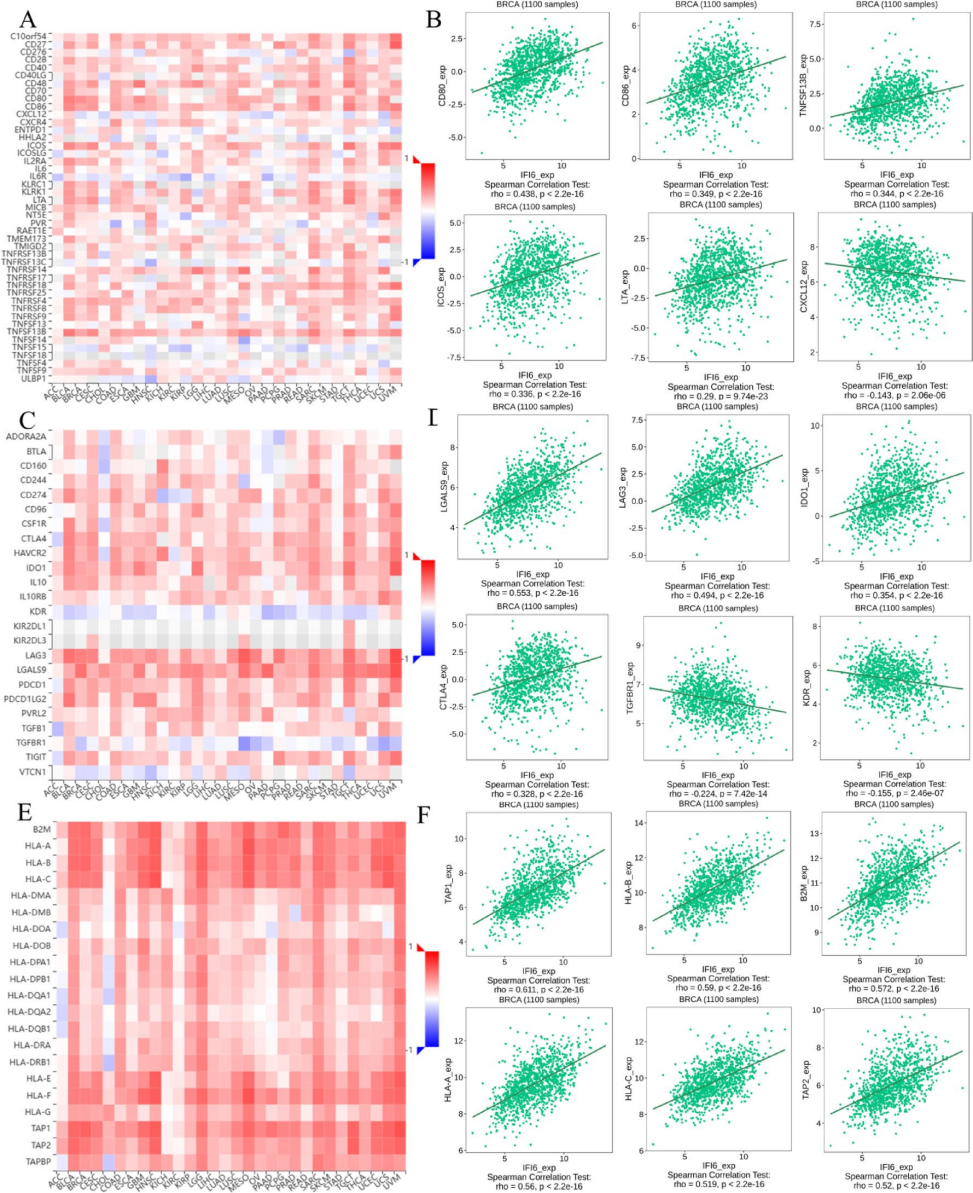
Relationship between IFI6 expression and the abundance of immunomodulators in breast cancer using TISIDB database. (**A,B**) Correlation between IFI6 expression and the abundance of immune-stimulators; (**C,D**) Correlation between IFI6 expression and the abundance of immunosuppressants; (**E,F**) Correlation between IFI6 expression and the abundance of MHC molecules.

To further elucidate the novel function of IFI6, we utilized the TISIDB database to assess the relationship between IFI6 expression and the abundance of immunomodulators. The results indicated that IFI6 was positively correlated with a variety of immunostimulatory molecules (Fig. 10A). The six immune-stimulators with the strongest correlations were CD80 (*r* = 0.438, *P* < 0.05), CD86 (*r* = 0.349, *P* < 0.05), TNFSF13B (*r* = 0.344, *P* < 0.05), ICOS (*r* = 0.336, *P* < 0.05), LTA (*r* = 0.290, *P* < 0.05), and CXCL12 (*r*=-0.143, *P* < 0.05) (Fig. 10B). Additionally, IFI6 expression was associated with a variety of immune-suppressive agents (Fig. 10C), with the six strongest associations being LGALS9 (*r* = 0.553, *P* < 0.05), LAG3 (*r* = 0.494, *P* < 0.05), IDO1 (*r* = 0.354, *P* < 0.05), CTLA4 (*r* = 0.328, *P* < 0.05), TGBR1 (*r*=-0.224, *P* < 0.05), and KDR (*r*=-0.155, *P* < 0.05) (Fig. 10D). Furthermore, IFI6 was positively correlated with several major histocompatibility complex (MHC) molecules (Fig. 10E), particularly TAP1 (*r* = 0.611, *P* < 0.05), HLA-B (*r* = 0.590, *P* < 0.05), B2M (*r* = 0.572, *P* < 0.05), HLA-A (*r* = 0.560, *P* < 0.05), TAP2 (*r* = 0.520, *P* < 0.05), and HLA-C (*r* = 0.519, *P* < 0.05) (Fig. 10F).

## Discussion

IFI6, also referred to as GIP3, belongs to the FAM14 protein family^19^. Due to its role in stabilizing mitochondrial function, IFI6 has been identified as an anti-apoptotic factor in various cancers^20^. Cheriyath et al. demonstrated that ectopic expression of IFI6 could induce tamoxifen resistance in the ER+ breast cancer cell line MCF-7, while silencing IFI6 triggered apoptosis in BT-549 cells and inhibited the growth of MCF-7 cells^21^. However, previous research has primarily concentrated on basic studies involving a limited number of breast cancer cell lines and has not integrated clinical data. Therefore, it is essential to conduct more comprehensive studies that explore the potential role of IFI6 in breast cancer. This study aims to enhance the understanding of the underlying mechanisms of IFI6 in tumor immunology and position IFI6 as a diagnostic and therapeutic target for personalized breast cancer treatment.

In the present study, we found that IFI6 expression was significantly upregulated in pan-cancer including breast cancer compared with adjacent normal tissues, which is consistent with the recent studies^22^. To validate this observation, we performed western blotting analysis and confirmed the overexpression of IFI6 in breast cancer cell lines compared to normal breast epithelial cells. Moreover, the expression of IFI6 was closely associated with advanced clinicopathological features including higher tumor stage, lymph node metastasis, SBR grade, NPI score, and hormone receptor status. Patients with higher IFI6 expression exhibited worse OS and RFS, which suggested that IFI6 may serve as a prognostic marker. Furthermore, we performed subtype-stratified survival analyses based on ER, PR, HER2 status, and lymph node involvement. Within most clinical subgroups, patients with high IFI6 expression consistently exhibited poorer OS and RFS, reinforcing the prognostic relevance of IFI6 across diverse breast cancer subtypes and clinical backgrounds. To enhance the clinical utility of these findings, we constructed a nomogram model integrating IFI6 expression with established clinicopathologic factors to predict individualized OS in breast cancer patients. The nomogram demonstrated favorable predictive accuracy and may serve as a practical decision-support tool for risk stratification in clinical settings^23^. While ER, PR, HER2, and Ki-67 are fundamental in classifying breast cancer into distinct subtypes, they do not fully account for prognostic heterogeneity within each subtype^24^. In the present study, high IFI6 expression remained significantly associated with worse survival outcomes even among ER-positive, PR-positive and HER2-positive patients, which indicates that IFI6 may reflect additional biological features not captured by existing markers. Incorporating IFI6 into multivariate models improved prognostic accuracy and supports its potential as a complementary marker to refine risk stratification.

The potential mechanism by which IFI6 impacts cancer development and progression is complex and not fully understood^25^. Co-expression analysis is a widely used approach for inferring putative gene functions and elucidating the roles of genes in phenotypic variations^26^. In this study, IFI6 exhibited a strong positive correlation with IFIT1, ISG15, and OAS1. IFIT1, an interferon-induced protein, has been implicated in promoting cancer development^27^. ISG15 mediates the ISGylation of KPNA2, thereby maintaining cancer stem cell-like characteristics in anaplastic thyroid carcinoma^28^. Furthermore, OAS1 has been identified as a prognostic biomarker in pan-cancer, potentially contributing to cytotoxic T lymphocyte (CTL) dysfunction and macrophage M2 polarization^29^. GSEA further revealed that IFI6 was associated with key biological processes, including oxidative phosphorylation, proteasome function, and antigen processing and presentation, all of which are critical pathways linked to the development and progression of breast cancer^30,31^.

Based on the co-expression analysis, we hypothesized that IFI6 may be associated with tumor immunity. Although the present study identified a positive correlation between IFI6 expression and immune score, the potential confounding factors such as tumor purity and stromal contamination may influence the estimation of immune cell infiltration^32^. Further analysis revealed a negative correlation between IFI6 expression and tumor purity (*r*=-0.14, *P* < 0.05), which suggests that patients with high IFI6 expression tend to have a lower proportion of tumor cells and a higher level of immune infiltration. Additionally, no significant association was found between IFI6 expression and stromal score. These findings alleviate concerns that tumor purity or stromal contamination may bias immune infiltration estimates, supporting a potential role of IFI6 in modulating the tumor immune microenvironment. Moreover, scRNA-seq revealed that IFI6 was predominantly expressed in epithelial cells and largely absent in immune populations such as T cells and myeloid cells, suggesting a tumor-intrinsic expression pattern^33^. Notably, IFI6-positive cells were enriched in epithelial compartments, whereas IFI6-negative status dominated immune cell populations. These findings are consistent with the negative correlation observed between IFI6 expression and tumor purity, as well as the lack of association with stromal score (*P* > 0.05), which indicates that immune relevance of IFI6 likely stems from the modulation of immune infiltration rather than stromal or purity-related artifacts. Our study also noticed that IFI6 expression was positively correlated with Tregs. As acknowledged, Tregs infiltrated heavily into tumor tissue, which was usually associated with a poor prognosis in cancer patients^34^. This may represent a potential mechanism underlying the poor prognosis observed in patients with elevated IFI6 expression. M1 macrophages are classically activated macrophages that play a critical role in anti-tumor and anti-infection immune responses. The polarization of macrophages toward the M1 phenotype can be induced by interferons, particularly interferon-γ, which in turn activates interferon-stimulated genes (ISGs) such as IFI6, OAS1, ISG15^35,36^. This aligns with the positive correlation between IFI6 expression and M1 macrophages identified in the present study. We also found that IFI6 expression was negatively associated with B cell naïve and plasma cells, which indicated that higher IFI6 expression was associated with reduced recruitment or activity of B cell subtypes within the tumor microenvironment, potentially contributing to an immunosuppressive state that favors tumor progression^37^. Our findings are in line with prior studies that characterized immune cell infiltration patterns in breast cancer using deconvolution algorithms such as CIBERSORT and TIMER^38^. However, compared to earlier studies that often focused on a single algorithm or dataset, our analysis employed a multi-platform approach that integrates TIMER 2.0, CIBERSORT, GEPIA, TISIDB, and single-cell RNA-seq data. In addition, tumor purity and stromal content were adjusted in correlation analyses to reduce potential confounding. This comprehensive strategy provides a more nuanced view of the tumor immune microenvironment and enhances the robustness of our conclusions.

Although existing evidence suggests that IFI6 contributes to the formation of a tumor-suppressive immune microenvironment, its precise role in shaping the tumor immune landscape remains to be fully elucidated. IFI6 is a well-characterized ISG with known mitochondrial localization. Emerging studies indicate its involvement in regulating mitochondrial reactive oxygen species (mtROS)^39^, potentially linking interferon signaling, mitochondrial metabolism, and immune modulation within the tumor microenvironment^40^. Elevated mtROS levels have been shown to promote the differentiation of Tregs and suppress the activity of effector T cells^40,41^. Particularly, moderate mtROS levels facilitate Foxp3 expression and IL-10 production in Tregs, which are crucial for the maintenance of local immune suppression^42^. In this context, IFI6 may enhance the expression of immunosuppressive cytokines such as IL-10, or chemokines that recruit Tregs to contribute to the immunosuppressive tumor microenvironment. Moreover, IFI6 is frequently co-expressed with other ISGs including ISG15 and OAS1, which suggests a role in sustaining a chronic interferon-driven immune state^43^. While acute interferon signaling is typically protective, prolonged activation has been associated with immune exhaustion and tumor immune evasion^44^. These findings raise the possibility that IFI6 may paradoxically promote tumor immune escape under chronic inflammatory conditions. These results support a mechanistic hypothesis in which IFI6 serves as a key mediator connecting interferon responses, mtROS production, and immune suppression. Further experimental validation is warranted to determine whether targeting IFI6 could reshape the immunosuppressive tumor microenvironment and improve anti-tumor immunity. To further elucidate the role of IFI6 in the tumor microenvironment, the correlation between IFI6 immunomodulators was analyzed. IFI6 expression was significantly positively correlated with immune inhibitors, immune stimulators and MHC molecules. The association of IFI6 with both immunostimulatory and immunosuppressive molecules highlights its complex and dualistic role in the tumor microenvironment. On one hand, IFI6 could enhance the anti-tumor immunity response through its correlation with immunostimulatory molecules. On the other hand, its association with immunosuppressive molecules may reduce immune efficacy, ultimately facilitating immune escape and tumor progression. The strong positive correlations identified in this study suggest that IFI6 acts as a significant immunomodulator and a potential target for future immunotherapy strategies. Targeting IFI6 in combination therapies, particularly in pathways involving specific immunosuppressants and MHC molecules, could provide a novel approach to enhance anti-tumor immunity. Further experimental studies are necessary to clarify the precise mechanisms by which IFI6 influences these immune molecules and their associated signaling pathways.

Despite the comprehensive bioinformatics analyses performed in this study, several limitations should be acknowledged. First, although we validated IFI6 expression patterns using independent datasets such as METABRIC and incorporated multiple immune deconvolution algorithms including TIMER and CIBERSORT. We did not employ additional frameworks such as xCell or MCP-counter, which use distinct gene signatures and statistical models to estimate immune cell populations^45^. The use of multiple complementary algorithms would enhance the robustness and reproducibility of immune infiltration findings. Our findings remain based solely on computational analyses and lack experimental validation. Future studies incorporating in vitro and in vivo functional assays, such as IFI6 knockdown or overexpression in breast cancer cell lines and subsequent immunological assays, as well as further validations including qPCR and immunohistochemistry (IHC) in clinical samples, will be crucial for confirming the mechanistic roles proposed. Second, this study is exploratory, and the associations observed between IFI6 expression and clinical or immune parameters should not be interpreted as causal. While approaches such as Mendelian Randomization (MR)-Egger regression could help assess potential pleiotropy and causality^46^, these were beyond the scope of the present work. We suggest that future studies incorporate such frameworks to elucidate the mechanistic role of IFI6 better. Third, while single-cell RNA sequencing data revealed that IFI6 is predominantly expressed in epithelial cells, suggesting a tumor-intrinsic expression pattern, the spatial heterogeneity of immune infiltration and the potential interaction between IFI6 and specific immune subsets remain to be clarified. Spatial transcriptomic profiling and multiplex immunohistochemistry could further unravel the contextual cellular crosstalk in the tumor microenvironment^47^. Fourth, although we stratified IFI6 expression across breast cancer molecular subtypes, the functional significance of IFI6 may differ across these subgroups. Future work should explore whether IFI6 acts as a prognostic or predictive biomarker preferentially in specific subtypes such as triple-negative or HER2-positive breast cancers. Additionally, the ancestry bias inherent in public datasets such as TCGA^48^, which underrepresent non-European populations, may limit the generalizability of our findings.

Our findings indicate that IFI6 is closely linked to immune infiltration patterns and patient prognosis in breast cancer, suggesting its potential as a useful clinical biomarker. Future research should focus on evaluating whether IFI6 adds diagnostic and prognostic value beyond well-established markers like ER, PR, and HER2. Large-scale prospective studies are needed to determine if incorporating IFI6 expression into existing molecular subtyping or risk stratification models can improve the accuracy of predicting patient outcomes and treatment responses. To facilitate clinical application, it is also important to explore how IFI6 testing could be integrated into routine diagnostic workflows, such as standardized IHC or RNA-based assays. Given the association between IFI6 and immune cell infiltration, we propose that IFI6 could serve as a stratification marker for immunotherapy to identify patients more likely to benefit from immune checkpoint inhibitors. Further preclinical studies should investigate whether targeting IFI6 can modulate the tumor immune microenvironment and enhance immunotherapy efficacy. Specifically, IFI6 inhibition may reduce immunosuppressive signaling and reshape macrophage or T cell populations by affecting mtROS and interferon signaling pathways. This raises the possibility that combining IFI6 inhibition with PD-1/PD-L1 blockade could have synergistic effects in breast cancer treatment. Validation of these hypotheses through in vivo models and combination therapy studies will be critical for advancing IFI6 as both a predictive and therapeutic biomarker in clinical practice.

### Methods

#### Data collection and IFI6 gene expression analysis

To analyze IFI6 expression across various cancer types, we utilized the TIMER 2.0 platform (http://timer.cistrome.org/), which processes RNA-seq data using standardized pipelines and applies TPM normalization to estimate gene expression levels. In parallel, we used the GEPIA web server (http://gepia.cancer-pku.cn/), which integrates data from TCGA and GTEx, normalizes expression data using log2(TPM + 1) transformation, and performs differential expression analyses with pre-applied statistical filters, including Benjamini-Hochberg correction for multiple testing. Analyses were conducted using the gene symbol ‘IFI6’, selecting the ANOVA module to compare tumor versus normal expression in breast cancer. Furthermore, the UALCAN database (https://ualcan.path.uab.edu/analysis.html/) was used to validate IFI6 expression levels in breast cancer, with gene expression values log2-transformed prior to comparison. Differentially expressed genes (DEGs) were identified using thresholds of |log2 fold change|>1 and false discovery rate (FDR) < 0.05. For the underlying gene expression analyses based on TCGA data, raw RNA-seq count data were downloaded and normalized using the Trimmed Mean of M-values (TMM) method via the edgeR R package to account for library size differences. Batch effects across sequencing runs were corrected using the ComBat algorithm implemented in the sva package, ensuring consistency for cross-dataset comparisons and reducing technical variability.

#### Cell lines and cell culture

The human breast epithelial cell line MCF-10A and breast cancer cell lines including MCF-7, BT-549, MDA-MB-231, and HCC1937 were used in this study. All cell lines were obtained from the American Type Culture Collection (ATCC) and cultured according to ATCC protocols. MCF-10A cells were maintained in DMEM/F12 medium supplemented with 5% fetal bovine serum (FBS), 20 ng/mL EGF, 0.5 µg/mL hydrocortisone, 100 ng/mL cholera toxin, and 10 µg/mL insulin. MCF-7, BT-549, MDA-MB-231, and HCC1937 cells were cultured in RPMI-1640 or DMEM supplemented with 10% FBS and 1% penicillin-streptomycin. All cells were maintained at 37 °C in a humidified incubator with 5% CO₂.

#### Western blot

Total protein was extracted from cultured cells using RIPA lysis buffer (Beyotime, Shanghai, China) supplemented with protease and phosphatase inhibitors. Protein concentrations were quantified using a BCA Protein Assay Kit (Thermo Fisher Scientific, USA) according to the manufacturer’s protocol. Equal amounts of protein (25 µg) were separated on a 15% SDS-PAGE gel and transferred onto PVDF membranes (Millipore, USA). After blocking with 5% non-fat milk in TBST (Tris-buffered saline with 0.1% Tween-20) for 1.5 h at room temperature, membranes were incubated overnight at 4 °C with primary antibodies against: IFI6 (Abclonal, Cat# A6157, dilution 1:1000), actin (Proteintech, Cat# 20536-1-AP, dilution 1:4000). After washing, membranes were incubated with appropriate HRP-conjugated secondary antibodies for 1 h at room temperature. Protein bands were visualized using an ECL detection reagent (Millipore) and imaged with a Clinx imaging system (Qinxiang, Shanghai, China). Densitometric analysis was performed using Image J (Version 1.53t, National Institutes of Health, Bethesda, MD, USA; https://imagej.net/ij/), with IFI6 expression normalized to actin.

#### Clinicopathological correlation analysis of IFI6

Gene expression data from TCGA were accessed via the UALCAN database to analyze the correlation between IFI6 expression and clinical stage, molecular subtypes, and lymph node metastasis in breast cancer. Analysis of variance (ANOVA) was used to perform difference tests on multiple sample groups. Additionally, the Breast Cancer Gene-Expression Miner (BC-GEM) (http://bcgenex.centregauducheau.fr/BC-GEM/), a comprehensive data mining tool integrating 36 published and annotated genomic datasets, was used to further investigate the relationship between IFI6 expression and various clinicopathological features. The data was last updated in December 2024.

#### Prognostic significance of IFI6

The prognostic value of IFI6 was assessed using the BC-GEM. The diagnostic accuracy of gene signatures is typically evaluated using the AUC value, which represents the area under the ROC curve. We downloaded and collated RNA-seq data from the STAR pipeline of the TCGA-BRCA (breast invasive carcinoma) project from the TCGA database (https://portal.gdc.cancer.gov), extracted data in TPM format, removed duplicate data, and processed the data with log2(value + 1) transformation. Subsequently, using R version 4.2.1, we performed ROC analysis on the data with the pROC package [1.18.0], and visualized the results with ggplot2 [3.4.4]. The Kaplan-Meier Plotter (http://kmplot.com/analysis/), an online database that integrates gene expression and clinical data, was used to evaluate the prognostic significance of IFI6 expression based on OS and RFS in breast cancer patients. Patients were dichotomized into high and low expression groups according to the median IFI6 expression. Log-rank p-values, hazard ratios (HR), and 95% confidence intervals (CIs) were calculated automatically by the platform and reported to evaluate statistical significance.

### The construction and validation of IFI6-based nomogram

To construct and validate an IFI6-based prognostic nomogram, we used the METABRIC cohort as the training set, incorporating clinical variables such as age, tumor stage, lymph node status, hormone receptor status (ER, PR), HER2 status, and IFI6 mRNA expression. Univariate Cox regression identified variables significantly associated with OS, which were then included in a multivariate Cox model to determine independent prognostic factors. Based on the multivariate model, we constructed a nomogram to estimate individualized 1-, 3-, and 5-year OS probabilities using the ‘rms’ R package (version 8.0.0) and ‘regplot’ R package (version 1.1). Model calibration was assessed using calibration curves generated with 1,000 bootstrap resamples. Discrimination ability was evaluated via time-dependent ROC curves, and the AUC at 1, 3, and 5 years was calculated using the ‘timeROC’ R package (version 0.4). To visualize survival curves and perform log-rank tests, we used the ‘survival’ R package (version 3.8-3) and ‘survminer’ R package (version 0.5.0).

In addition, we implemented DCA curve to evaluate the clinical net benefit of the nomogram using the ‘ggDCA’ R package (version 1.2). External validation was conducted on two independent datasets GSE7390 and GSE42568, applying the same statistical procedures and evaluation metrics to assess the model’s generalizability. *P* < 0.05 was considered significant difference.

### Gene set enrichment analysis

Patients with invasive breast cancer from the TCGA BRCA dataset were classified into two groups based on the median IFI6 mRNA level. GSEA analysis was conducted using GSEA software (version 4.1.0) to identify key biological pathways associated with IFI6. *P* < 0.05, FDR < 0.25, and normalized enrichment scores (|NES|) > 1 were considered significant. FDR were calculated using the Benjamini-Hochberg method to account for multiple testing.

### Identification and enrichment analysis of IFI6 co-expressed genes

LinkedOmics (http://www.linkedomics.org) was utilized to analyze genes co-expressed with IFI6 in the TCGA BRCA cohort^49^. The Pearson correlation coefficient was employed to assess statistical correlations between IFI6 expression and other genes. Volcano plots were generated using the “ggplot2” R package (version 3.4.4), and heatmaps of the top 50 positively and negatively correlated genes were constructed using “pheatmap” (version 1.0.12). GO functional annotation and KEGG pathway enrichment analyses were performed using the “cluster Profiler” R package (version 4.8.1). To control for multiple hypothesis testing, both Bonferroni correction and Benjamini-Hochberg FDR adjustments were applied, and terms with *P* < 0.05 were considered statistically significant.

### Immune infiltration analysis

The ESTIMATE algorithm (R package estimate) was used, alongside Pearson correlation analysis, to calculate the correlation between IFI6 expression and the immune-related score including the immune score, stromal score, ESTIMATE score, and tumor purity in breast cancer. The CIBERSORT algorithm was applied to determine the infiltration levels of 22 immune cell types from TCGA expression profiles. Pearson correlation analysis was employed to evaluate the relationship between immune cell proportions and IFI6 expression levels^50^. All correlation analyses were adjusted for multiple comparisons using the Benjamini-Hochberg method, and FDR < 0.05 was considered statistically significant. The significance of differences between two groups was determined using the Dunn test, while comparisons among multiple groups were evaluated with the Kruskal–Wallis test. Furthermore, the association between IFI6 expression and immunomodulators was analyzed using TISIDB database (http://cis.hku.hk/TISIDB/index.php)^51^. Spearman correlation coefficients and FDR-adjusted p-values were reported to identify significant immunoregulatory interactions.

### Single-cell RNA sequencing data processing and analysis

scRNA-seq data were obtained from GSE176078 and processed using the ‘Seurat’ R package (version 5.3.0). Initial quality control steps involved filtering cells with fewer than 200 detected genes and genes expressed in fewer than 3 cells. Cells with high mitochondrial gene content (> 10%) were excluded to remove low-quality or dying cells. The filtered gene expression matrix was normalized using the “LogNormalize” method with a scale factor of 10,000. Highly variable genes were identified for downstream analyses. Principal component analysis (PCA) was performed based on the variable genes, and significant principal components were selected for clustering using the Louvain algorithm. Cell clusters were visualized via Uniform Manifold Approximation and Projection (UMAP). Cluster annotation was conducted by comparing canonical marker gene expression to known cell type signatures. To assess IFI6 expression patterns, cells were dichotomized into IFI6-positive and IFI6-negative groups based on normalized expression thresholds. The distribution of IFI6 expression among cell clusters was visualized using feature plots and stacked bar charts. Differential expression analyses between IFI6-positive and negative cells were performed where relevant. All analyses were conducted in R (version 4.4.3).

## Supplementary Information

Below is the link to the electronic supplementary material.

Supplementary Material 1

## Data Availability

All data generated or analyzed during this study are included in this published article [and its supplementary information files].

## Abbreviations

IFI6: Interferon alpha-inducible protein 6
BC: Breast cancer
BLCA: Bladder urothelial carcinoma
BRCA: Breast cancer
CHOL: Cholangiocarcinoma
COAD: Colorectal cancer
ESCA: Esophageal carcinoma
HNSC: Head and neck squamous cell carcinoma
KIRC: Clear cell carcinoma of kidney
LIHC: Liver hepatocellular carcinoma
PRAD: Prostate adenocarcinoma
READ: Rectum adenocarcinoma
STAD: Stomach adenocarcinoma
UCEC: Uterine corpus endometrial carcinoma
KICH: Kidney chromophobe
LUSC: Lung squamous cell carcinoma
TNBC: Triple-negative BC
ILC: Invasive lobular carcinoma
SBR: Scarff-Bloom-Richardson
NPI: Nottingham prognostic index
PR: Progesterone receptor
ER: Estrogen receptor
AUC: Area under the curve
BP: Biological process
CC: Cellular component
MF: Molecular function
Tregs: Regulatory T cells
MHC: Major histocompatibility complex
mtROS: Mitochondrial reactive oxygen species
CTL: Cytotoxic T lymphocyte
TCGA: The Cancer Genome Atlas
BC-GEM: Breast cancer gene-expression miner
ROC: Receiver operating characteristic
OS: Overall survival
RFS: Recurrence-free survival
HR: Hazard ratios
GSEA: Gene set enrichment analysis
FDR: False discovery rate
NES: Normalized enrichment scores
GO: Gene Ontology
KEGG: Kyoto Encyclopedia of Genes and Genomes
ATCC: American type culture collection
HR: Hazard ratios
CIs: Confidence intervals
PCA: Principal component analysis
UMAP: Uniform manifold approximation and projection
ANOVA: Analysis of variance
