# Supplementary material for: Bioinformatics analysis of IFI6 as a novel prognostic biomarker and its correlation with immune infiltration in breast cancer
Source: Sci Rep. 2025 Oct 17;15:36446. doi: 10.1038/s41598-025-20489-6 (PMC12534504; doi:10.1038/s41598-025-20489-6)
Supplement: Supplementary file 1 — Supplementary Material 1 [file 41598_2025_20489_MOESM1_ESM.pdf]

Table S1 Enrichment analysis of hallmark pathways of IFI6

NAME	SIZE	ES	NES	NOM p-value	FDR q-value	FWER p-value	RANK AT MAX	LEADING EDGE
HALLMARK_INTERFERON_GAMMA_RESPONSE	198	0.85	2.53	0	0	0	1004	tags=59%, list=5%, signal=62%
HALLMARK_INTERFERON_ALPHA_RESPONSE	97	0.93	2.45	0	0	0	481	tags=78%, list=2%, signal=80%
HALLMARK_ALLOGRAFT_REJECTION	200	0.68	2.09	0.004	0.003	0.005	2723	tags=57%, list=13%, signal=65%
HALLMARK_INFLAMMATORY_RESPONSE	200	0.58	1.98	0.004	0.008	0.026	2722	tags=41%, list=13%, signal=46%
HALLMARK_COMPLEMENT	200	0.51	1.96	0.004	0.007	0.028	3379	tags=37%, list=17%, signal=44%
HALLMARK_IL6_JAK_STAT3_SIGNALING	87	0.59	1.95	0.004	0.006	0.032	1555	tags=33%, list=8%, signal=36%
HALLMARK_UV_RESPONSE_UP	158	0.43	1.79	0	0.031	0.131	3365	tags=30%, list=16%, signal=36%
HALLMARK_OXIDATIVE_PHOSPHORYLATION	200	0.51	1.76	0.023	0.037	0.176	3182	tags=43%, list=16%, signal=50%
HALLMARK_REACTIVE_OXYGEN_SPECIES_PATHWAY	49	0.48	1.64	0.024	0.08	0.324	2482	tags=31%, list=12%, signal=35%
HALLMARK_DNA_REPAIR	150	0.42	1.57	0.047	0.108	0.414	4315	tags=41%, list=21%, signal=51%
HALLMARK_APOPTOSIS	161	0.38	1.54	0.035	0.122	0.476	2633	tags=26%, list=13%, signal=30%

Table S2 Co-expressed genes of IFI6

Query	correlation coefficients	P-value	FDR (BH)	Event_SD
ROPN1L	9.34204852040427e-05	0.997552111693189	0.998345039001406	1081
KRT33A	8.48334614802418e-06	0.99977771132256	0.99977771132256	1081
WNT9A	7.57211828158886e-05	0.998015883805428	0.998660340714303	1081
VWA5B1	6.5752217933218e-05	0.998277099371595	0.998822497608065	1081
ETHE1	5.21147163461585e-05	0.99863444144896	0.99908079176546	1081
SPANXN4	4.41000706752672e-05	0.998844448532588	0.999241268097978	1081
PRSS41	4.06331645999914e-05	0.998935291605292	0.999282523060315	1081
JMJD8	3.03416701473537e-05	0.999204958917943	0.999502651442572	1081
FBXO17	1.78782158167587e-05	0.999531538062587	0.999641835842355	1081
P2RX6	1.74563884060747e-05	0.999542591182555	0.999641835842355	1081
ISG15	0.898373082843382	1e-302	1e-299	1081
IFIT1	0.89311166714336	1e-302	1e-299	1081
OAS1	0.850005874542272	1.22377641478662e-302	6.16324396896912e-299	1081
MX1	0.849568711918294	5.18130398370353e-302	2.08754737503415e-298	1081
OASL	0.83681851316041	1.45281183803471e-284	4.87781574620155e-281	1081
IRF9	0.822119267478764	3.13279387271366e-266	9.01573322368811e-263	1081
IFIT3	0.800596472178739	3.05281990562387e-242	7.6873821248491e-239	1081
IFI27	0.793347736284917	8.12272127814143e-235	1.81813577942399e-231	1081
IFI44L	0.785036398840956	1.16055563356379e-226	2.33793932381425e-223	1081
IFI44	0.784712242877097	2.37325120102714e-226	4.34628594951743e-223	1081
OAS2	0.78314200875883	7.45916401024372e-225	1.25220715821966e-221	1081
CMPK2	0.758715195439151	4.60103022364382e-203	7.12982721963883e-200	1081
USP18	0.749128082515377	3.28927047929463e-195	4.7330252718136e-192	1081
OAS3	0.739457126793144	1.21931953372836e-187	1.63754613379719e-184	1081
XAF1	0.733773234342355	2.39368272112859e-183	3.01379615107096e-180	1081
IFIT2	0.721336782299512	2.49411505031379e-174	2.95552633462184e-171	1081
PARP12	0.721206089638674	3.08365882204858e-174	3.45112816500937e-171	1081
RSAD2	0.708297017911025	2.17900866080065e-165	2.31032260378048e-162	1081
IFI35	0.704412771001656	8.06501322632578e-163	8.12348457221664e-160	1081
HERC6	0.694309999087803	2.47870872604463e-156	2.37778987076995e-153	1081
DDX58	0.690259182449148	8.32419444263748e-154	7.62231350213328e-151	1081

IFIH1	0.671975957751154	6.6252284641887e-143	5.80283597439484e-140	1081
UBE2L6	0.660722336356815	1.41125743832269e-136	1.18457421229211e-133	1081
IFIT5	0.655264946941265	1.32069721256681e-133	1.06421781388633e-130	1081
IFITM1	0.654487924593999	3.45794800600897e-133	2.67924471465579e-130	1081
PARP9	0.653204493331518	1.68485418244163e-132	1.25708842612173e-129	1081
BST2	0.651690231428281	1.0806760433236e-131	7.77507817598352e-129	1081
BATF2	0.647253779283284	2.35474320259438e-129	1.63573454538841e-126	1081
STAT1	0.630101473670602	1.13676786265503e-120	7.63339619772852e-118	1081
HERC5	0.625297622832609	2.45899091815019e-118	1.59794748535921e-115	1081
DDX60	0.625213672944331	2.6989856682339e-118	1.69909582145537e-115	1081
RTP4	0.622581436399961	4.93237243252714e-117	3.01098917131088e-114	1081
SIGLEC1	0.608630269640823	1.52843470138058e-110	9.05597560567991e-108	1081
TAP1	0.608469637570638	1.80762917088199e-110	1.04041970421193e-107	1081
PRIC285	0.605966613296107	2.43839414301934e-109	1.36448472253124e-106	1081
DHX58	0.604145795834569	1.59519861728518e-108	8.68520976897567e-106	1081
LGALS3B	0.60346579145611	3.20701346757957e-108	1.70013911327343e-105	1081
MX2	0.60097383457593	4.08602828923866e-107	2.11059076632597e-104	1081
PSME2	0.598694005800052	4.11077351870094e-106	2.07028831335576e-103	1081
IL29	0.59839057706572	5.58158849933157e-106	2.74246586143986e-103	1081
CXCR2P1	0.59489734656776	1.84430178895593e-104	8.84606179488506e-102	1081
IRF7	0.589914238735657	2.51649586260382e-102	1.17894905005009e-99	1081
PLSCR1	0.58885908901237	7.04866749107505e-102	3.22716833199334e-99	1081
HLA-B	0.587733641241426	2.10574762786176e-101	9.42673021406114e-99	1081
ZBP1	0.583668695139581	1.05845478531326e-99	4.63534166307298e-97	1081
SP110	0.581704525261454	6.88878504636125e-99	2.95265052678611e-96	1081
PSMB8	0.580758898117948	1.68968696736848e-98	7.09140499117461e-96	1081
PARP14	0.571573872308418	8.85382809385642e-95	3.64000748879056e-92	1081
B2M	0.567101070819876	5.20215542522439e-93	2.09594842082291e-90	1081
EPSTI1	0.563403932842658	1.43920990386469e-91	5.68487912026554e-89	1081
PSMB9	0.56230186407402	3.84091498997954e-91	1.48798523986803e-88	1081
SP100	0.559672278800662	3.93712740572136e-90	1.49647984128786e-87	1081
ETV7	0.557636378620661	2.35251151775034e-89	8.77617491205196e-87	1081
HLA-A	0.555798951508647	1.16842090996707e-88	4.2796071329612e-86	1081

DDX60L	0.552371235234275	2.26235289860035e-87	8.13841056112573e-85	1081
LGALS9	0.550130530331173	1.54099675527073e-86	5.44620695349628e-84	1081
SAMD9	0.549689364694729	2.24446372602526e-86	7.7956416828929e-84	1081
C19orf66	0.544793696131469	1.40326251185715e-84	4.79130903412919e-82	1081
IFITM3	0.542411925811274	1.02386377241501e-83	3.43762261588341e-81	1081
TRIM21	0.541111919185276	3.00900436349923e-83	9.93711359060525e-81	1081
APOBEC3	0.537305619109484	6.87849434902225e-82	2.23495594614602e-79	1081
LOC40069	0.534281938645982	8.03158237726959e-81	2.55866870544266e-78	1081
OTOF	0.534267058658866	8.12880601381635e-81	2.55866870544266e-78	1081
IL28A	0.531167964846031	9.83380916862742e-80	3.04772439541538e-77	1081
HCP5	0.526261584851621	4.83049675200207e-78	1.47439934953154e-75	1081
LY6E	0.523405297767039	4.52764998888259e-77	1.36133595561253e-74	1081
HLA-C	0.51762916786684	3.91891579521016e-75	1.16097880433101e-72	1081
TAP2	0.517406778766325	4.64526983533118e-75	1.33760419331939e-72	1081
EIF2AK2	0.517406033297061	4.64791727636423e-75	1.33760419331939e-72	1081
HLA-H	0.509733317032998	1.51983772670588e-72	4.31227197246337e-70	1081
IFNB1	0.508456799187869	3.92600177136654e-72	1.09846257894693e-69	1081
PSME1	0.507489881464979	8.03489307459396e-72	2.21730028750267e-69	1081
PARP10	0.503386151452643	1.6366944296869e-70	4.45556882243819e-68	1081
CCL8	0.503219187908079	1.84862149545963e-70	4.96539733680457e-68	1081
HLA-F	0.503034030364549	2.11571170969799e-70	5.60802794629815e-68	1081
RUFY4	0.496995806244447	1.64871680593766e-68	4.31342857865118e-66	1081
LAG3	0.496048944018844	3.23863448160305e-68	8.36439636306326e-66	1081
REC8	0.494355068578525	1.07798950788385e-67	2.74887324510381e-65	1081
DTX3L	0.493830983489822	1.56173060393104e-67	3.93263287702384e-65	1081
LAP3	0.491295176401369	9.3031701118933e-67	2.31373286301346e-64	1081
ISG20	0.490385898033881	1.75773180810901e-66	4.31823259443366e-64	1081
CXCL11	0.487497323136215	1.30996500364134e-65	3.17942710823552e-63	1081
CXCL10	0.480135488184575	2.00921120078444e-63	4.81851900473841e-61	1081
APOL1	0.477816453246377	9.56282264820053e-63	2.26638896762353e-60	1081
IL28B	0.473725354172868	1.45636953914639e-61	3.4114609728028e-59	1081
SECTM1	0.469760288917285	1.96931675811037e-60	4.55998690714176e-58	1081
STAT2	0.465889516504471	2.42218098878087e-59	5.54486772943076e-57	1081

SLC15A3	0.45132085768888	2.30656192669775e-55	5.22086404644115e-53	1081
GMPR	0.450602852588106	3.58191425222327e-55	8.0175180678931e-53	1081
HSH2D	0.444975076383002	1.08800009991703e-53	2.408545276135e-51	1081
TYMP	0.440627516632571	1.45543661603362e-52	3.15266350860186e-50	1081
CD80	0.440164853201386	1.91381461641479e-52	4.10146760081658e-50	1081
APOL2	0.438483237775756	5.15865907685412e-52	1.09390723266554e-49	1081
UNC93B1	0.431727448706192	2.62041824406451e-50	5.49878390902912e-48	1081
IFI30	0.42958718746172	8.928455166347e-50	1.85426525078413e-47	1081
LILRA5	0.428587775319249	1.57790169454206e-49	3.2435540445459e-47	1081
KLHDC7I	0.428257912133316	1.90336544092821e-49	3.87306028358574e-47	1081
LOC4007:	0.426899564144676	4.11096974512047e-49	8.28154855154519e-47	1081
FBXO6	0.426586965888226	4.90556764187807e-49	9.78442179659739e-47	1081
NLRC5	0.424853057239725	1.30290599743967e-48	2.57323934494334e-46	1081
IFIT1B	0.421358769920892	9.1695174928895e-48	1.79339737761416e-45	1081
UBA7	0.414520497032644	3.90944205348262e-46	7.57266443917378e-44	1081
NMI	0.414384265068475	4.20921848904587e-46	8.075686329698e-44	1081
SHISA5	0.411937979963939	1.57710099334423e-45	2.99723580291693e-43	1081
ZNFX1	0.411145681309322	2.41344004350331e-45	4.54380838096955e-43	1081
HCG26	0.407623432771497	1.57793001486094e-44	2.94327779160868e-42	1081
PATL2	0.406226979599934	3.30134609979582e-44	6.10143276884283e-42	1081
CCR1	0.403668571878553	1.26507423757967e-43	2.29593878522905e-41	1081
PML	0.398313249597922	2.0275367579122e-42	3.64685071322691e-40	1081
SP140	0.397516375773359	3.0504932586879e-42	5.43824661028919e-40	1081
TRIM22	0.396824218047736	4.34576477206115e-42	7.67942380115542e-40	1081
KIR2DL4	0.39629527020638	5.69213345450417e-42	9.97113290791187e-40	1081
TREX1	0.39618123113261	6.03277259480041e-42	1.04767417174357e-39	1081
SAMD9L	0.395929579598482	6.85778041827855e-42	1.18076911560873e-39	1081
PSMA6	0.395192168585064	9.97759317010365e-42	1.68906398665326e-39	1081
AIM2	0.394392135661631	1.49703000400348e-41	2.51313911922084e-39	1081
SMTNL1	0.393419186718818	2.44831278463771e-41	4.07613727657246e-39	1081
RNF213	0.392300015694262	4.30255746906504e-41	7.1045098536324e-39	1081
STAC3	0.391210771767523	7.43247745691056e-41	1.21729478349157e-38	1081
HESX1	0.390851774243509	8.89577802023153e-41	1.445205227561e-38	1081

IL27	0.390134881809805	1.27278724020331e-40	2.05122391631166e-38	1081
TAPBP	0.386629954164605	7.24154305066958e-40	1.15778479964872e-37	1081
APOL6	0.382559551664507	5.31246748890608e-39	8.40020687505068e-37	1081
TDRD7	0.382549906033088	5.33743598911138e-39	8.40020687505068e-37	1081
IL4I1	0.382221536795633	6.26062293666851e-39	9.77676349296024e-37	1081
LGALS9C	0.381266307237106	9.94760176304331e-39	1.5414956732039e-36	1081
PSMB10	0.381138872701401	1.05802558657681e-38	1.62701720928167e-36	1081
C1QC	0.375442284871157	1.61970413180592e-37	2.47188937388108e-35	1081
ADAMDE	0.375050051522652	1.95055528414198e-37	2.95443129316092e-35	1081
HLA-E	0.374291168403626	2.7926948620789e-37	4.18434614421765e-35	1081
APOL3	0.37428253594903	2.80410389411459e-37	4.18434614421765e-35	1081
IRF5	0.373462076216517	4.12890749773541e-37	6.11594423102057e-35	1081
C5orf56	0.373243922463451	4.57545775365192e-37	6.72792674797941e-35	1081
WARS	0.372895605951501	5.38981239285549e-37	7.86795439522275e-35	1081
C2	0.3707357922538	1.48165785073102e-36	2.14733794266017e-34	1081
FAM158A	0.370514226079701	1.64290506992251e-36	2.36402304525635e-34	1081
OSCAR	0.370429713300553	1.70889779931265e-36	2.44154228135839e-34	1081
GNLY	0.368764743056458	3.70368790832041e-36	5.25428119106442e-34	1081
GBP1	0.367955737300314	5.38450027016011e-36	7.585367688278e-34	1081
CARD16	0.367737124373885	5.9563495432611e-36	8.33268482979131e-34	1081
MLKL	0.365396958357821	1.74621335627315e-35	2.42603228014639e-33	1081
C1QA	0.364890175221146	2.20164198816632e-35	3.03781355148018e-33	1081
CTSL1	0.364371322003271	2.7900120507783e-35	3.82345528999515e-33	1081
C19orf38	0.363394765912393	4.35200411690517e-35	5.92372452263882e-33	1081
TMEM15(	0.363220270535636	4.71107282108671e-35	6.36943368998603e-33	1081
PSMC4	0.363201516513573	4.75137155585207e-35	6.38109199950933e-33	1081
PKD2L1	0.362516559406186	6.48270104365714e-35	8.64861010095849e-33	1081
HLA-G	0.36184994779409	8.76523896802758e-35	1.16168249349287e-32	1081
CCL7	0.358866008807804	3.35269030753623e-34	4.41437557158937e-32	1081
SOCS1	0.358558951208003	3.84588976330541e-34	5.03087332998621e-32	1081
IDO1	0.358136456212393	4.64409532072183e-34	6.03582582167363e-32	1081
P2RY6	0.356001682376207	1.199106334753e-33	1.53859854226746e-31	1081
PSMA3	0.355683967073988	1.38006568576258e-33	1.75958374934729e-31	1081

OGFR	0.355613990975904	1.42342567569086e-33	1.8034534740121e-31	1081
GRN	0.354896773662262	1.95363055814451e-33	2.45974297461383e-31	1081
CSAG3	0.353440683330808	3.70614749725613e-33	4.63728828150464e-31	1081
APOBEC3	0.353025854474104	4.44507978479126e-33	5.52753902868024e-31	1081
CD86	0.35129835555088	9.44973402130873e-33	1.16788277214273e-30	1081
TNFSF13I	0.350758217909616	1.19512615668678e-32	1.46803758697897e-30	1081
C1QB	0.350455855084387	1.36276875940004e-32	1.65379377458516e-30	1081
APOBEC3	0.348719395139114	2.88812840359786e-32	3.48391297547778e-30	1081
PSMA5	0.348223467146911	3.57602984155479e-32	4.28804292607864e-30	1081
ICOS	0.348060204974861	3.83627646939243e-32	4.57288695123731e-30	1081
SLC25A2	0.34775772144636	4.36905243795956e-32	5.17732713898208e-30	1081
TIMD4	0.347144373104977	5.68482314722296e-32	6.65818385469806e-30	1081
IFI27L1	0.346861575255544	6.4172003796457e-32	7.42956906022774e-30	1081
PILRA	0.346444018056695	7.67280672219126e-32	8.83249665248817e-30	1081
RARRES3	0.345913713038217	9.6238570385466e-32	1.10154886387228e-29	1081
BTN3A2	0.345740043475272	1.03640934914339e-31	1.17957436940642e-29	1081
ADAR	0.345159240496333	1.32742692550241e-31	1.5023042367554e-29	1081
SDS	0.344713171749361	1.60473359514962e-31	1.8059976689547e-29	1081
IL10	0.344620010971022	1.66952750787856e-31	1.86847953590076e-29	1081
ABCD1	0.3445363464226	1.72992366356774e-31	1.92537636478299e-29	1081
GPR84	0.344363373840063	1.86174328123768e-31	2.0607043077216e-29	1081
GBP5	0.343765640915141	2.39869940269892e-31	2.6405354900202e-29	1081
TRANK1	0.343323408880909	2.8923210568089e-31	3.16661998312039e-29	1081
APOBEC3	0.343167950360801	3.08876301523453e-31	3.36341248334592e-29	1081
FPR2	0.343109627423447	3.16582547623786e-31	3.42879323757053e-29	1081
CCL5	0.342756247783851	3.67522721716416e-31	3.95922204758139e-29	1081
LAMP3	0.342150691415298	4.74381216481624e-31	5.08319659894804e-29	1081
RBCK1	0.339828248571921	1.25593717428293e-30	1.33866954369999e-28	1081
IRF1	0.33944702611719	1.4724244783941e-30	1.55298382812822e-28	1081
RAB8A	0.338444551963374	2.23454162502911e-30	2.34452297063601e-28	1081
CNP	0.338317479976624	2.35561242572654e-30	2.45874675213789e-28	1081
CTLA4	0.338183611340947	2.49019479949366e-30	2.58582341421649e-28	1081
RNF31	0.337507960295046	3.29485808564892e-30	3.40384185309731e-28	1081

TYROBP	0.337369388862686	3.48930897564755e-30	3.58633312828673e-28	1081
C1orf200	0.336214085410455	5.62167455064701e-30	5.74866161536975e-28	1081
GBP4	0.335510956072528	7.50747544325102e-30	7.63828751536827e-28	1081
ITGB7	0.335297284291213	8.19607991613601e-30	8.29698642766633e-28	1081
GNGT2	0.333940032145878	1.42888068828937e-29	1.43207967490495e-27	1081
TRIM69	0.333560572072788	1.6682736663569e-29	1.66373133706731e-27	1081
MRPS18C	0.333284173777876	1.867274775038e-29	1.85301725828279e-27	1081
LILRB2	0.332109384843387	3.01069047269503e-29	2.97305684178635e-27	1081
HK3	0.330758571853281	5.2010818978995e-29	5.0862036326789e-27	1081
HPSE	0.330679780720931	5.36915318005312e-29	5.22519762377634e-27	1081
GZMB	0.3303639685835	6.09855770099365e-29	5.90651177339025e-27	1081
TMEM14(	0.33006278099205	6.88535242557133e-29	6.63662318723131e-27	1081
FTSJD2	0.329948903875065	7.20835166740479e-29	6.91486877808903e-27	1081
NCF1	0.329413723743469	8.93905553807525e-29	8.53446795329507e-27	1081
RGS19	0.3281064376654	1.50936383012032e-28	1.4275180449659e-26	1081
HCST	0.327656691142462	1.80636505859317e-28	1.70043103296072e-26	1081
PSMA7	0.327103683737127	2.25189908336601e-28	2.10020865900039e-26	1081
MED8	0.32701205765327	2.33557193758632e-28	2.16820722039984e-26	1081
FPR3	0.326602315001875	2.74904015968666e-28	2.54034009251779e-26	1081
LGMN	0.32532431744945	4.56324611885701e-28	4.19756132714038e-26	1081
SPI1	0.325253063020095	4.69368691849653e-28	4.29792377150512e-26	1081
SIGLEC14	0.324737229971482	5.75476267961177e-28	5.22205829643149e-26	1081
FCGR1B	0.324448658723535	6.4486604604504e-28	5.82548273433961e-26	1081
ZBTB8OS	0.324214542899736	7.07199892714075e-28	6.33179637276669e-26	1081
CASP1	0.32213370539528	1.6001651563103e-27	1.42005846140401e-25	1081
GPBAR1	0.321651280251111	1.93190176752452e-27	1.70693689064831e-25	1081
CTSA	0.32123165625658	2.27527067893427e-27	1.99284034031004e-25	1081
CD72	0.320357260329669	3.19682516575418e-27	2.78788064779731e-25	1081
IL18BP	0.319228606093433	4.95026447443344e-27	4.27996042220866e-25	1081
SIGLEC7	0.318975855982059	5.45815038309459e-27	4.69890766954874e-25	1081
MYD88	0.318897900370878	5.6249671084717e-27	4.82191329362393e-25	1081
FCER1G	0.318793757158827	5.8557234555037e-27	4.99322125784622e-25	1081
LYSMD2	0.318375153706231	6.88166880875709e-27	5.82484109884082e-25	1081

NCF1C	0.31801396022182	7.90863926131369e-27	6.6660894526847e-25	1081
ABHD12	0.31764718497241	9.10664854850717e-27	7.64389312540321e-25	1081
PCK2	0.317432349631368	9.89007509028915e-27	8.24976626406224e-25	1081
HLA-L	0.317423000306927	9.92564353049252e-27	8.24976626406224e-25	1081
OPTN	0.317416272000134	9.95131894845929e-27	8.24976626406224e-25	1081
STAP1	0.316981535744601	1.17574693603283e-26	9.66751919444137e-25	1081
DOK3	0.316056718716618	1.67495635845447e-26	1.36056434843005e-24	1081
CTNNBL1	0.315331374875371	2.20884688359201e-26	1.7870369666651e-24	1081
SLC35B1	0.313853373032854	3.87246653362579e-26	3.12043353279566e-24	1081
ACOT7	0.313830540922417	3.90610069494329e-26	3.13499595616066e-24	1081
FCGR1A	0.313031970878628	5.28320830960048e-26	4.22342188082943e-24	1081
MRPL17	0.311917319421669	8.0407106853279e-26	6.40237615636089e-24	1081
DDIT3	0.31160879835465	9.02904463913258e-26	7.16102772658763e-24	1081
ACP2	0.31081457495666	1.21611995328123e-25	9.60734763092172e-24	1081
HMOX1	0.310357328874254	1.44296991435187e-25	1.13549331736791e-23	1081
ROMO1	0.309921350810382	1.69809282653836e-25	1.33105369613289e-23	1081
FAM26F	0.309634226006474	1.88999514801471e-25	1.47573458359521e-23	1081
HM13	0.309617004454248	1.90216439511671e-25	1.47950199766896e-23	1081
LILRA3	0.309256033738765	2.17585347310419e-25	1.68586800829553e-23	1081
PDE6G	0.308946307858824	2.44150940345828e-25	1.88445237289912e-23	1081
PSMA4	0.308428893545549	2.95869984582094e-25	2.27492398450622e-23	1081
CD68	0.308371161446602	3.02274093539658e-25	2.31532761002145e-23	1081
PTPN6	0.308186315833228	3.23715585883678e-25	2.47017063546465e-23	1081
TNFAIP8I	0.308038151713389	3.41983158767934e-25	2.59971725787926e-23	1081
ODF3B	0.307400759634181	4.32924247968991e-25	3.26638912933907e-23	1081
ATP6V1F	0.306748555265275	5.50728698978746e-25	4.13971255258464e-23	1081
MEFV	0.306482402356167	6.07459007752634e-25	4.54916792237056e-23	1081
CD7	0.304743722152253	1.14972038297164e-24	8.51511658638368e-23	1081
PIR	0.304613102881231	1.20595787433507e-24	8.89890892984616e-23	1081
FOXP3	0.304597816290924	1.212714790531e-24	8.91610929023611e-23	1081
LTA	0.303552907031562	1.77534088325521e-24	1.29580587294117e-22	1081
MOV10	0.303467875812967	1.83114119759634e-24	1.33170900453351e-22	1081
PSMB3	0.302721974764276	2.4012004268245e-24	1.74000656828704e-22	1081

PNPT1	0.302275759556171	2.82279582102196e-24	2.03090077908883e-22	1081
FERMT3	0.301929407404285	3.1998079124841e-24	2.2939548183983e-22	1081
RNF181	0.30184319662283	3.30114237512576e-24	2.35820968606058e-22	1081
BTN3A3	0.300800372429263	4.80938695063306e-24	3.42350177104251e-22	1081
BOLA3	0.300564799881404	5.23497877176649e-24	3.7133326534238e-22	1081
C1orf38	0.300488389723366	5.38086650589712e-24	3.80342300916833e-22	1081
BCL2A1	0.299814287183769	6.85503924571413e-24	4.79495713905941e-22	1081
TACO1	0.299790448681492	6.91390770880455e-24	4.81940037349023e-22	1081
IL22RA1	0.299422045084406	7.88987954199456e-24	5.48074563356829e-22	1081
EDEM2	0.299256508193891	8.37165464386377e-24	5.79542896222116e-22	1081
MRPS15	0.298997178348158	9.18551116985895e-24	6.33705899030167e-22	1081
LOC10013	0.298454087789977	1.11519310174497e-23	7.6674283394718e-22	1081
JAKMIP1	0.298312942388518	1.17277776588214e-23	8.03592112030465e-22	1081
ADPRHL2	0.298288454991651	1.18306326717154e-23	8.05771179492016e-22	1081
ACP5	0.298237963828887	1.20455363000702e-23	8.17028042979507e-22	1081
HAMP	0.298110675527498	1.26045938835667e-23	8.49229243426261e-22	1081
NEDD8	0.298098825760026	1.26579261534812e-23	8.49979741206265e-22	1081
NCF4	0.297577690335064	1.52377666853396e-23	1.01643976780187e-21	1081
CALR	0.29747697062804	1.57932605604131e-23	1.05001727389281e-21	1081
MNDA	0.296852356963615	1.97137931181669e-23	1.29782471361265e-21	1081
ATP5H	0.296450088927147	2.27333398359754e-23	1.487879094785e-21	1081
TMEM149	0.296448216626679	2.27484120721657e-23	1.487879094785e-21	1081
ATOX1	0.296435354368813	2.2852222185617e-23	1.48983176676134e-21	1081
FUT7	0.296077912457745	2.59321640475707e-23	1.68517240238165e-21	1081
DRAP1	0.29600656717615	2.65943601011261e-23	1.72264753774014e-21	1081
CD38	0.295913925779154	2.74792233862331e-23	1.77425947152457e-21	1081
DNTTIP1	0.295778190228596	2.88285183370603e-23	1.85543291341879e-21	1081
PSMB2	0.295306520130645	3.40467572823559e-23	2.17048077675019e-21	1081
CCL2	0.294914620607031	3.90846961637179e-23	2.48185664137085e-21	1081
HAVCR2	0.294247840389003	4.94037270089758e-23	3.11011900186193e-21	1081
LILRB1	0.294027710032885	5.3369512838413e-23	3.33890942897463e-21	1081
SAMHD1	0.293945877268323	5.49226825805539e-23	3.42544099252402e-21	1081
C20orf24	0.29378191849751	5.81702856567356e-23	3.61679137208314e-21	1081

SDHB	0.293721738352213	5.94093325248732e-23	3.68246462681099e-21	1081
ADA	0.293622125604811	6.15178407986893e-23	3.80146289229937e-21	1081
ANKRD58	0.293487439945296	6.4486809519552e-23	3.97274243966782e-21	1081
HIST2H2A	0.293098440111888	7.38805829199925e-23	4.53757421622942e-21	1081
NAGK	0.292889916148507	7.94605487899634e-23	4.86544910447968e-21	1081
ZNF683	0.292742027212307	8.36685026127567e-23	5.10758177313328e-21	1081
SLC7A7	0.292298676033788	9.76486739831454e-23	5.94299860238811e-21	1081
FCGR1C	0.29221299760944	1.00605265697749e-22	6.08880821117184e-21	1081
LILRB4	0.29221175038811	1.0064895181535e-22	6.08880821117184e-21	1081
FAM192A	0.292152027815485	1.0276293389996e-22	6.1980817467506e-21	1081
AUP1	0.291758031882684	1.17851797823177e-22	7.0869387079042e-21	1081
C6orf129	0.291695109804902	1.20456375411193e-22	7.22200500791213e-21	1081
TRIM14	0.291300884907813	1.3811965886011e-22	8.25644073512437e-21	1081
ATP6V0B	0.291112388820941	1.47447168430273e-22	8.76201536291401e-21	1081
SLC25A19	0.290633747854786	1.74023133577124e-22	1.03108706644446e-20	1081
DEF6	0.290576671981921	1.77492482913231e-22	1.04855896430705e-20	1081
PPM1K	0.290046909250859	2.13136868772881e-22	1.25545094193851e-20	1081
VAMP8	0.289868163096133	2.26692549288077e-22	1.33140565755344e-20	1081
IL2RA	0.289662635171767	2.43334942403751e-22	1.42499488800104e-20	1081
GRINA	0.289547326606786	2.53194866122226e-22	1.478437848705e-20	1081
UBE2S	0.289495071022447	2.57792227668706e-22	1.50093191514049e-20	1081
COPE	0.289196715722217	2.85665666345086e-22	1.65365943923039e-20	1081
YIF1A	0.288920896257872	3.14073191445455e-22	1.80270972204809e-20	1081
SLC16A3	0.288920663148346	3.14098343231016e-22	1.80270972204809e-20	1081
IL15RA	0.288252191390047	3.95059204155327e-22	2.25452341861447e-20	1081
UBE2F	0.288225743948566	3.98654913276991e-22	2.26861673106355e-20	1081
BTN3A1	0.288063599178683	4.21417332584925e-22	2.38467757441666e-20	1081
HIST1H1C	0.287282856238015	5.50312725354003e-22	3.08803617054496e-20	1081
ZBED2	0.286935485116936	6.19523545216866e-22	3.45714731811462e-20	1081
CDKN2D	0.285905154738324	8.79495618777305e-22	4.84083039351607e-20	1081
SH3BGRL	0.285845569620302	8.97459386296864e-22	4.92624505094014e-20	1081
CCR5	0.285815389780089	9.0669585956396e-22	4.96342067687934e-20	1081
ADRM1	0.285687835141452	9.46782210998405e-22	5.16881507874332e-20	1081

PREB	0.285634068514908	9.64199416867894e-22	5.2496749331902e-20	1081
GSTO1	0.285313518290816	1.07480577343282e-21	5.83610843822214e-20	1081
GCH1	0.284827693326165	1.26675795966778e-21	6.84151182238806e-20	1081
LSM10	0.284563438973731	1.38500529524398e-21	7.46014215847324e-20	1081
C1orf162	0.284395308239303	1.46584921721442e-21	7.87454199487586e-20	1081
MYO1G	0.284314365361643	1.50641513137388e-21	8.0709395801933e-20	1081
PHF11	0.284178827929128	1.57683974579832e-21	8.40355467701247e-20	1081
TCIRG1	0.284133465470322	1.60112874140171e-21	8.51048509117082e-20	1081
GSDMD	0.283901221034162	1.73139260152521e-21	9.17865893624352e-20	1081
ATP13A1	0.283590850472755	1.92194956584865e-21	1.01355167549793e-19	1081
LAPTM5	0.283273696848572	2.13806239406835e-21	1.12457616001323e-19	1081
SCO2	0.283021277278805	2.32707431759552e-21	1.22080500333234e-19	1081
ATP5G3	0.282979637107584	2.35980236272288e-21	1.23475892459877e-19	1081
TFEC	0.282534548203379	2.73932666058693e-21	1.42963045537626e-19	1081
PTTG1	0.282172615054472	3.09189592697543e-21	1.60531555280722e-19	1081
LCP2	0.281862902510561	3.42892352760301e-21	1.77572402219955e-19	1081
HIST2H2A	0.281720062391934	3.59634907218068e-21	1.85290158718874e-19	1081
CD163	0.281536505424486	3.823401283756e-21	1.96485762401185e-19	1081
PSMG1	0.281303228837125	4.13248234662553e-21	2.11829152348019e-19	1081
IL15	0.280641681271207	5.14966556837599e-21	2.63299525063285e-19	1081
PSMB5	0.280563937757407	5.28436709467255e-21	2.695027218283e-19	1081
PTCRA	0.280093373184145	6.17721125230773e-21	3.11879500445462e-19	1081
MRPL27	0.279740139745715	6.94385897328768e-21	3.49710097542201e-19	1081
ATP5E	0.279678610942768	7.08669197552812e-21	3.56013490890309e-19	1081
RHOG	0.279306917590698	8.01335411041375e-21	4.01564722771853e-19	1081
CCRL2	0.278862723083407	9.27873317891568e-21	4.6382153818674e-19	1081
MRPS11	0.278822124557424	9.40378315848546e-21	4.68908939920024e-19	1081
RNASE1	0.278631822511685	1.0012485931431e-20	4.98028466885622e-19	1081
C15orf63	0.278071997689948	1.20378852985181e-20	5.95830956606013e-19	1081
APOE	0.27801618723268	1.22607304199742e-20	6.05373564486227e-19	1081
TMEM208	0.277204648636432	1.60009255083904e-20	7.88114044905928e-19	1081
CATSPER	0.276859844005417	1.79124981949105e-20	8.80115307649933e-19	1081
HSD17B1(	0.276648144283401	1.91960968109829e-20	9.40888978728104e-19	1081

GTPBP2	0.276490821831319	2.02084168436118e-20	9.88103294452815e-19	1081
RNF19B	0.275755790080538	2.56814306436254e-20	1.25266929858556e-18	1081
GALK1	0.275691671497898	2.62230959080384e-20	1.27600064509042e-18	1081
EIF2S2	0.275604328550905	2.69791449391955e-20	1.3064780644233e-18	1081
WAS	0.275578694840999	2.72050894173471e-20	1.31426025494594e-18	1081
TSPAN33	0.274952071276818	3.33480849180855e-20	1.60333453621678e-18	1081
FCGR3A	0.274387135989239	4.00488716568531e-20	1.9163527779746e-18	1081
MSRB2	0.274341889936663	4.06397459071463e-20	1.94001820213143e-18	1081
PSMC5	0.273151319898654	5.9688729700351e-20	2.81599405108565e-18	1081
WBSCR2[illegible]	0.272727055086524	6.84199799239995e-20	3.21286828803956e-18	1081
TIGIT	0.272390736395117	7.62269770224417e-20	3.56285951767306e-18	1081
MPP1	0.272151322265784	8.23139579230182e-20	3.8295951093746e-18	1081
DYNLRB[illegible]	0.27212482014082	8.30165679336244e-20	3.85338424198816e-18	1081
HIST1H2[illegible]	0.27210608550457	8.35168183490594e-20	3.86769265664782e-18	1081
NUDT5	0.27101466534316	1.18419591252064e-19	5.44649010450418e-18	1081
C11orf48	0.270969685458361	1.20131952797572e-19	5.51266102302297e-18	1081
PRELID1	0.270828181986075	1.25679855868216e-19	5.74109001466036e-18	1081
COQ9	0.270424587027686	1.42930581985354e-19	6.49963109276512e-18	1081
EPHB2	0.270362532149767	1.45782528128762e-19	6.61438970530158e-18	1081
ADAP2	0.270020266649036	1.62551263536984e-19	7.32571633993859e-18	1081
DNAJB11	0.269867725918397	1.70624888554786e-19	7.6724070980718e-18	1081
SLAMF8	0.269711819812476	1.79285405039056e-19	8.02600996558175e-18	1081
ARPC3	0.269059432453016	2.20480151952819e-19	9.80479616134554e-18	1081
TEX19	0.268969316932827	2.26859923137155e-19	1.00662844748854e-17	1081
PLD3	0.268693365101714	2.47550596975889e-19	1.09361990703493e-17	1081
GBP6	0.268579313073351	2.56636195033448e-19	1.12880701942114e-17	1081
ADAM8	0.268466321870742	2.65961530126233e-19	1.16240024353833e-17	1081
TIMM17B	0.26842261690053	2.69657556235558e-19	1.17581200657258e-17	1081
SDF2L1	0.268396973350085	2.71849713330504e-19	1.18281046977171e-17	1081
COX8A	0.268077216271518	3.00706339816628e-19	1.29994189176094e-17	1081
CLEC4E	0.26802649737816	3.05553142592306e-19	1.3152495849406e-17	1081
RENBP	0.2679948711655	3.08614339598635e-19	1.32559400239115e-17	1081
RNF114	0.267928286131133	3.15158562938843e-19	1.3508232447666e-17	1081

NKG7	0.267775622055811	3.3068422180671e-19	1.40837920682795e-17	1081
NDUFS8	0.267613741262518	3.47971747159794e-19	1.47576649400717e-17	1081
IL2RG	0.267530532171263	3.57201690295516e-19	1.51172858214352e-17	1081
SRA1	0.267412790413378	3.70676177407537e-19	1.5654657429507e-17	1081
COX5A	0.267060310409817	4.14084147660502e-19	1.74148750618389e-17	1081
NOD2	0.26609344802494	5.60588330380359e-19	2.33327518915544e-17	1081
SP140L	0.266003095591249	5.76647341322575e-19	2.39023882529697e-17	1081
PDCD1LC	0.265896860335953	5.96110508445422e-19	2.46078815422808e-17	1081
NDUFB3	0.265826483094334	6.09359564836875e-19	2.51033710299363e-17	1081
THAP8	0.265812803874166	6.11968295841743e-19	2.51081493273562e-17	1081
TSPAN17	0.265777254207165	6.18799515113556e-19	2.53368216096801e-17	1081
NCF1B	0.265626153234578	6.48684169732124e-19	2.64529202414041e-17	1081
HMGN2	0.265588252864157	6.56400700963769e-19	2.67135194361922e-17	1081
SIGLEC5	0.265315197846764	7.14735390282246e-19	2.90289202363626e-17	1081
SH3BP1	0.265203028316171	7.40154721313225e-19	3.00008387542352e-17	1081
HIST1H2I	0.265144520579593	7.53765231558453e-19	3.04911658428615e-17	1081
PSMC3	0.264888371661886	8.16317135356214e-19	3.28237698438143e-17	1081
CORO1A	0.264861396887933	8.23195166358337e-19	3.30343956698978e-17	1081
MCOLN2	0.264709042570936	8.63129211528359e-19	3.45680675273137e-17	1081
MANF	0.264665703165054	8.74834035465289e-19	3.4898082464254e-17	1081
TK1	0.264538821422505	9.1000899558354e-19	3.61580497357602e-17	1081
HLA-J	0.264504992465867	9.19620468909514e-19	3.64680203665003e-17	1081
CCR8	0.264415638962405	9.45491698717911e-19	3.73469220993575e-17	1081
RDH16	0.264314371899596	9.7568086058648e-19	3.84639744354494e-17	1081
UBC	0.264207993236753	1.00841784321053e-18	3.96769090849145e-17	1081
FBXO39	0.263998340843927	1.07613970241605e-18	4.20948238935367e-17	1081
IL12RB1	0.263657032095801	1.1961053281549e-18	4.66064638988016e-17	1081
DPP3	0.263538728297002	1.24069171534041e-18	4.82504529064334e-17	1081
PSMD14	0.263491327010427	1.25901276053335e-18	4.88686166877539e-17	1081
ZDHHC12	0.26329971052251	1.33583293259466e-18	5.17506815906141e-17	1081
TSSC1	0.263284705184258	1.34204021231676e-18	5.18913629119407e-17	1081
RABGGT/	0.263143361373312	1.40192377406916e-18	5.41029778326114e-17	1081
PDCD1	0.263084482192783	1.42763986834389e-18	5.49900672041827e-17	1081

CD300LF	0.263016078080591	1.45810065158848e-18	5.60561786760496e-17	1081
RNASEH2	0.262898858595124	1.51179633914578e-18	5.80097852420797e-17	1081
C1orf122	0.262857904506627	1.53101242560916e-18	5.86184494478511e-17	1081
XCL2	0.262852685610337	1.53347842437416e-18	5.86184494478511e-17	1081
NT5C3	0.262696842566128	1.60894864230403e-18	6.12708325126932e-17	1081
TDO2	0.262594048578814	1.66072145589442e-18	6.30927890335581e-17	1081
TRPV2	0.262406993676807	1.7591815226603e-18	6.64891402889151e-17	1081
GPS1	0.262271557571706	1.8340421696782e-18	6.89305587838942e-17	1081
SQSTM1	0.26186726065791	2.07670184076753e-18	7.79053232444354e-17	1081
MRPS23	0.261818367940935	2.10811321021413e-18	7.89366926017913e-17	1081
RNASE6	0.261732803501676	2.16421574962028e-18	8.07372708816676e-17	1081
DNASE2	0.261511333248113	2.31635653422347e-18	8.60941003356676e-17	1081
LAIR2	0.261360265482584	2.42613513613509e-18	9.00082731444593e-17	1081
PPIL5	0.260722083354273	2.94933230311337e-18	1.08618462972978e-16	1081
MRPL38	0.260594717585062	3.06634966319108e-18	1.12516601029115e-16	1081
SPAG4	0.260504143605488	3.15233780998061e-18	1.15461536694653e-16	1081
FAM96B	0.260383212671565	3.27086177284613e-18	1.1958531835569e-16	1081
ALKBH6	0.260231933757314	3.42533091199723e-18	1.25005962359029e-16	1081
HN1	0.260077891407283	3.59000917788664e-18	1.30542842759073e-16	1081
RPL26L1	0.260053622441887	3.61665592494483e-18	1.31274835329754e-16	1081
ICT1	0.259969249704898	3.71082140930116e-18	1.34378566948321e-16	1081
BCAP31	0.259965107886381	3.71550567337873e-18	1.34378566948321e-16	1081
BLVRB	0.259898022590242	3.79219392282219e-18	1.36417404598666e-16	1081
FAM50A	0.259717640213424	4.00622148133011e-18	1.43859771375036e-16	1081
SH2D2A	0.259611531780765	4.13763886961673e-18	1.48314475139553e-16	1081
C16orf61	0.259569765285952	4.19052519787495e-18	1.49943392737462e-16	1081
TUBA1C	0.259525354732855	4.2474906076123e-18	1.5144371378823e-16	1081
RHBDF2	0.259469853942218	4.31975559222597e-18	1.53748191528961e-16	1081
LAIR1	0.259351285506914	4.47822296931595e-18	1.59107234068554e-16	1081
CD74	0.259158547841706	4.74813120202322e-18	1.68399829339362e-16	1081
ZBTB32	0.259145146788995	4.76748318196655e-18	1.68789013533772e-16	1081
PPP1CA	0.258698579403517	5.45877282999256e-18	1.92586652644834e-16	1081
SELL	0.258686711104134	5.47843284042148e-18	1.92929944250757e-16	1081

BTN2A2	0.258681156919593	5.48765738673039e-18	1.92929944250757e-16	1081
LST1	0.258619111004566	5.59175160213831e-18	1.96247101088983e-16	1081
PIK3AP1	0.258382835523855	6.00627338948632e-18	2.10063155262503e-16	1081
C8orf31	0.258308735966455	6.1424068525745e-18	2.14451968882345e-16	1081
RAC2	0.257863358468439	7.02710230584285e-18	2.43231917441932e-16	1081
SNX11	0.257780612717454	7.20478278815991e-18	2.48954286908201e-16	1081
RPN2	0.257198824821713	8.58539077949953e-18	2.93637856117178e-16	1081
PSTPIP1	0.257141476174868	8.73483996502628e-18	2.98242967958397e-16	1081
POP4	0.257079030659025	8.90048815161935e-18	3.03384659584385e-16	1081
ALG3	0.257055160378149	8.96462309842854e-18	3.05054615401762e-16	1081
RPN1	0.257044021982341	8.99470551413293e-18	3.0556212914369e-16	1081
CR1L	0.256867316649588	9.48547451247548e-18	3.20075182669713e-16	1081
CDC25B	0.256795765030646	9.69162054317146e-18	3.26484441207674e-16	1081
MYO7A	0.256725272216915	9.89903214211323e-18	3.32914862275244e-16	1081
PSMD2	0.256548716740067	1.04379066920431e-17	3.50452717185346e-16	1081
CEBPE	0.256393485064982	1.09355125153085e-17	3.66548917838419e-16	1081
HIST1H2I	0.256217160207779	1.15291327374958e-17	3.8452711754446e-16	1081
MRPS7	0.256175650913856	1.16734328927881e-17	3.88054959777586e-16	1081
C19orf10	0.256102629930295	1.19316101908317e-17	3.95332709365634e-16	1081
CD48	0.256071357070101	1.20438938536306e-17	3.98397769591771e-16	1081
CD53	0.255840715471374	1.2904788032197e-17	4.25708550874081e-16	1081
PLEK	0.25564143685607	1.36972487532389e-17	4.50867771460781e-16	1081
GPKOW	0.255593501669967	1.38949136964125e-17	4.56628118130879e-16	1081
GNG5	0.255517843844543	1.42126291031591e-17	4.66308490689155e-16	1081
THOC4	0.255476460520507	1.43894308208533e-17	4.71341599814782e-16	1081
GMIP	0.255418659195396	1.46400077836312e-17	4.7799506774919e-16	1081
CASP5	0.255188497006234	1.56811548092325e-17	5.10334190035523e-16	1081
SOD1	0.254973545084123	1.67192191791267e-17	5.43239790908882e-16	1081
APOBEC3	0.254823896747242	1.74816012670311e-17	5.67096388928086e-16	1081
CD2	0.254694859672216	1.81664087201174e-17	5.86478050748021e-16	1081
PDF	0.254600959359129	1.86812933702145e-17	6.01173570196438e-16	1081
CXCR3	0.254468639534394	1.94313325618366e-17	6.2232781312909e-16	1081
DDX39	0.254456971883095	1.94988754136376e-17	6.22511640582772e-16	1081

RAB4B	0.254335631807397	2.02151584167032e-17	6.42325498902976e-16	1081
CKLF	0.25410668371215	2.16379742020106e-17	6.84296688068293e-16	1081
PARP6	0.25402867316864	2.21449630408979e-17	6.99232414512363e-16	1081
SIRPG	0.253968070648851	2.25468789503856e-17	7.10808883341968e-16	1081
KLRC3	0.253947031806393	2.2688082324452e-17	7.1414284129076e-16	1081
REEP4	0.253795205935416	2.37332259040217e-17	7.45874938902522e-16	1081
EGFL6	0.253784587668047	2.3808069758126e-17	7.4706162815802e-16	1081
CD300A	0.253760695117964	2.39773299957185e-17	7.51202663707231e-16	1081
EFHD2	0.253690715798525	2.44799303731107e-17	7.65758070444589e-16	1081
SLA2	0.253664287887909	2.46724270031049e-17	7.68200992237323e-16	1081
PSMC1	0.253652920107009	2.47556860728803e-17	7.68541602071975e-16	1081
NDUFS3	0.253521372277981	2.57395279339417e-17	7.9772736958347e-16	1081
SASH3	0.253355860201862	2.70322597454172e-17	8.31396752017449e-16	1081
PRDX1	0.253006936443948	2.99707019419544e-17	9.18964673699652e-16	1081
FGD2	0.252863449874727	3.12684207488608e-17	9.54397478766365e-16	1081
TMEM18(	0.252850309657449	3.13899938779413e-17	9.56658739290663e-16	1081
HCK	0.252752905625677	3.23058108639774e-17	9.7778168563904e-16	1081
HTATIP2	0.252750813420845	3.23257683115215e-17	9.7778168563904e-16	1081
NME1	0.252540671356996	3.43934250403254e-17	1.03411275736919e-15	1081
C20orf7	0.25251360999398	3.46689853543601e-17	1.04084457520653e-15	1081
SSR4	0.25248835834928	3.49280787069411e-17	1.04706271659424e-15	1081
GPR65	0.25247758750255	3.50391719440608e-17	1.04883227163908e-15	1081
FIBP	0.252445681492907	3.53703053824695e-17	1.05717329663182e-15	1081
MIR155H(	0.252273408830053	3.72121919597598e-17	1.11057719559905e-15	1081
PSMB6	0.252240337228562	3.75764417467743e-17	1.11978908134433e-15	1081
HIST1H2/	0.252200657085972	3.80181151933276e-17	1.12960904213803e-15	1081
CCDC88B	0.251946790002418	4.09673819505855e-17	1.2136586902861e-15	1081
ZDHHC24	0.251894865297474	4.15978060252304e-17	1.23052540731023e-15	1081
ATPIF1	0.251793070152563	4.28615697508114e-17	1.26605032643709e-15	1081
CARS	0.251671936825926	4.44147598345205e-17	1.30809259775792e-15	1081
PSMD8	0.251559549670418	4.59053171409625e-17	1.35001841431342e-15	1081
APOC1	0.251486576128213	4.68994143016648e-17	1.37585940999084e-15	1081
CFL1	0.251485037849405	4.69205964092184e-17	1.37585940999084e-15	1081

RHOF	0.251258900236928	5.01392482017376e-17	1.46720363963245e-15	1081
BRMS1	0.251256037938431	5.01813505935349e-17	1.46720363963245e-15	1081
DNPEP	0.251164136578812	5.15518198836314e-17	1.50291087055826e-15	1081
NUB1	0.250980664021919	5.43990227642024e-17	1.58133955784251e-15	1081
C6orf226	0.250890451154992	5.58552325865343e-17	1.62133092284688e-15	1081
PTPN7	0.250758439607943	5.80556010417225e-17	1.67554453149785e-15	1081
RHEBL1	0.250700762901102	5.90435502425189e-17	1.70161991364169e-15	1081
ABCF3	0.250609307457997	6.0644143355382e-17	1.74525181127739e-15	1081
HIST1H2/	0.250564286643815	6.14476891475201e-17	1.76585406259172e-15	1081
CCL3	0.250481849749021	6.29463205008113e-17	1.80634419727755e-15	1081
SAMSN1	0.250344362868223	6.55261849244448e-17	1.87503550469168e-15	1081
PSMC2	0.250316983900883	6.60522325506683e-17	1.88682727258445e-15	1081
NSFL1C	0.24999984800666	7.2457092446304e-17	2.05874206957799e-15	1081
SEMA4D	0.249979837133628	7.28811414294428e-17	2.06787407619173e-15	1081
C15orf24	0.249798826125978	7.68296738102247e-17	2.16466262784192e-15	1081
BIRC5	0.24935415241457	8.74463065676565e-17	2.45691191883604e-15	1081
NOL7	0.249227377455438	9.07292222033342e-17	2.54559913828157e-15	1081
LRRC25	0.248895941801753	9.98963122315973e-17	2.79501556931323e-15	1081
CCL4	0.248605936724296	1.08661555895825e-16	3.01929247382261e-15	1081
CTSS	0.248536033463232	1.10885123010597e-16	3.07260082950273e-15	1081
TRAPPC3	0.248457888608136	1.13423912199818e-16	3.13736980464676e-15	1081
CCL11	0.248336016411767	1.17497994732711e-16	3.23401025557722e-15	1081
GTSF1	0.248311739947897	1.18326579260913e-16	3.25196308214338e-15	1081
TNFRSF4	0.248152687849542	1.23899342614244e-16	3.40047991411983e-15	1081
HINT2	0.248146453730304	1.24122953735005e-16	3.40198218094105e-15	1081
PRKD2	0.248137610171957	1.24440844431485e-16	3.40606088460905e-15	1081
PGP	0.248120359517162	1.25063245451508e-16	3.41845193978376e-15	1081
TIFAB	0.247894527425949	1.33499520058123e-16	3.64410275280607e-15	1081
FTL	0.247728297532614	1.4006490011822e-16	3.81814264260019e-15	1081
SIL1	0.247709424801834	1.40830097249986e-16	3.83381393121753e-15	1081
NDUFA8	0.247539072932751	1.47925928432154e-16	4.02154902599965e-15	1081
CTSZ	0.24745543443766	1.51537499089719e-16	4.10864457491573e-15	1081
FMNL1	0.247353467133477	1.56058113421431e-16	4.21419664192324e-15	1081

OR2B6	0.247326516050771	1.57274980764269e-16	4.24137146920508e-15	1081
OTUB1	0.247248515579582	1.60849664708276e-16	4.3319739245297e-15	1081
BOLA2	0.247148728482636	1.65539631125056e-16	4.45233093326334e-15	1081
TRAFD1	0.246811473619107	1.82409004079931e-16	4.89950584958694e-15	1081
PRDX5	0.246777232461313	1.84213560346762e-16	4.94138771396207e-15	1081
BCAS4	0.246601728827782	1.93742361415382e-16	5.16945678240115e-15	1081
MT2A	0.246554466057567	1.96390324414802e-16	5.23317868430713e-15	1081
PRF1	0.246274720605263	2.12808863788539e-16	5.64826687881438e-15	1081
TBX21	0.246120274110797	2.22444895776001e-16	5.88850515822278e-15	1081
CARD17	0.246032140698604	2.28134763802341e-16	6.01541206387194e-15	1081
TRIM5	0.245830405393469	2.41703555036812e-16	6.339997547157e-15	1081
NDUFA13	0.245448947155557	2.6956681871779e-16	7.04335092486367e-15	1081
LILRA6	0.245366746939094	2.75972912906948e-16	7.20139161983221e-15	1081
CD300C	0.245336000866989	2.78407350565037e-16	7.24614480249698e-15	1081
SLC11A1	0.245277146826015	2.83126446694678e-16	7.3594609918249e-15	1081
PSMB7	0.245270666003359	2.8365088664247e-16	7.36359163841824e-15	1081
LIMD2	0.245192313755143	2.90067454640857e-16	7.51080832100265e-15	1081
P4HB	0.245064257439284	3.00863360687111e-16	7.78034968041316e-15	1081
MRPL12	0.245031564716893	3.03682329816767e-16	7.83313768778332e-15	1081
LSM4	0.244987436147808	3.07528634499463e-16	7.92220504091007e-15	1081
MRPL14	0.244921393250519	3.1337476418427e-16	8.06249632757614e-15	1081
C14orf156	0.244813341302724	3.231762749735e-16	8.27240922406753e-15	1081
LILRB3	0.244627229302217	3.40770943119542e-16	8.7117140217553e-15	1081
C6orf125	0.244580578488771	3.45326975980418e-16	8.80583788750065e-15	1081
HIST1H3I	0.244504165109198	3.52919548915501e-16	8.97672261730147e-15	1081
SLC22A1	0.244410488500423	3.62451878306245e-16	9.18439382198656e-15	1081
NFKBIE	0.244077403225159	3.98443527862733e-16	1.00710726082745e-14	1081
ARPC5L	0.244015968724407	4.05455874584003e-16	1.02354744279383e-14	1081
TACC3	0.243985831580395	4.08940097860405e-16	1.03105109779698e-14	1081
LOC6067	0.243925859551985	4.15961490880476e-16	1.0474430292234e-14	1081
TRIM38	0.243876804629391	4.21792834509506e-16	1.06080108004919e-14	1081
PIM2	0.243825547888191	4.27971865578965e-16	1.07499915612073e-14	1081
SNRPG	0.243698300386268	4.43699330483564e-16	1.111731717984e-14	1081

HIST1H2A	0.243517577657373	4.67018026302792e-16	1.16725535234116e-14	1081
TRAF3IP3	0.243464016450714	4.74157833006981e-16	1.18363191399326e-14	1081
ANAPC11	0.243384694350999	4.84929370260634e-16	1.20752807959215e-14	1081
SNRPC	0.24293876534914	5.50119347613788e-16	1.3631304708049e-14	1081
COX6A1	0.242938734542572	5.5012413639334e-16	1.3631304708049e-14	1081
NDUFA12	0.242730091085284	5.83516151795487e-16	1.44055549974511e-14	1081
CTRL	0.242724533671725	5.84432318641582e-16	1.4410512924155e-14	1081
ARRB2	0.242661218459189	5.94970591565852e-16	1.46345330489549e-14	1081
MARS	0.24251823519343	6.19461678290655e-16	1.51813327362108e-14	1081
GINS2	0.24250036402403	6.22591668254069e-16	1.52395007982724e-14	1081
ABI3	0.242409752177245	6.38702650195708e-16	1.55770761358263e-14	1081
COX17	0.242262353545129	6.65791068663204e-16	1.61985037176573e-14	1081
CD4	0.241996528154092	7.17530531146197e-16	1.73942870637065e-14	1081
FANCA	0.241947819041405	7.27430997399653e-16	1.76130978877596e-14	1081
C20orf20	0.24193327709469	7.3041271632142e-16	1.76640626294058e-14	1081
AIF1	0.241727322911196	7.73955947889238e-16	1.86722665511721e-14	1081
C5AR1	0.241665878953297	7.87434213677406e-16	1.89747155915447e-14	1081
CCDC28B	0.241638377006144	7.93541569317091e-16	1.90990381289042e-14	1081
AIFM2	0.241624125750357	7.96724652836138e-16	1.9152766266568e-14	1081
IL23A	0.241613188127512	7.99176138521271e-16	1.91888001317175e-14	1081
SIGLEC9	0.241460025356573	8.34294733093532e-16	1.99843845400347e-14	1081
PFN1	0.241395379709753	8.49568966756398e-16	2.02539252488848e-14	1081
COX7B	0.241308006714686	8.70651121227491e-16	2.07319938973142e-14	1081
FUCA2	0.241024833134767	9.42577467489493e-16	2.23128355846955e-14	1081
TLCD1	0.24092229325715	9.70038789503921e-16	2.29090637919771e-14	1081
GMFG	0.240477555266635	1.09854020008015e-15	2.57926484039798e-14	1081
SELS	0.240256215173933	1.16859420146945e-15	2.73101278290047e-14	1081
LAT2	0.239804794770799	1.32536567047907e-15	3.06829875894707e-14	1081
ITGAL	0.239801382711851	1.32662607051025e-15	3.06829875894707e-14	1081
COQ2	0.239771679223093	1.33764833769045e-15	3.09024378013464e-14	1081
CD97	0.239474667723635	1.45293659571169e-15	3.34125658911095e-14	1081
NUDC	0.239463895164341	1.45729678773112e-15	3.34746223361954e-14	1081
CD14	0.239452976462324	1.46172926389267e-15	3.35381959238245e-14	1081

CHMP5	0.239433213214341	1.46978595596423e-15	3.36846849634806e-14	1081
FLVCR2	0.23934025858664	1.50826953177378e-15	3.45273746790715e-14	1081
PDCD5	0.239199776269609	1.56831778507216e-15	3.58612506019055e-14	1081
ITPKA	0.239177825783033	1.57791039419473e-15	3.60396880850939e-14	1081
BSCL2	0.238882086448243	1.712918386829e-15	3.89466601610274e-14	1081
CCL4L2	0.238867784469493	1.71972774446253e-15	3.90134182569794e-14	1081
ATP6V0E	0.238806627428288	1.7491469804686e-15	3.95652136651293e-14	1081
TMEM93	0.238748111800402	1.77775871702936e-15	4.01040866232435e-14	1081
MXD3	0.238554429188884	1.87578407037904e-15	4.22361498849738e-14	1081
SNRPB	0.23844388231608	1.93409631570921e-15	4.34847882588862e-14	1081
WBP2	0.238398720729067	1.95842822450859e-15	4.398276096179e-14	1081
GBP3	0.238363073196376	1.97784657789538e-15	4.43693978972188e-14	1081
GALT	0.238341057234917	1.98993383367112e-15	4.45908977522856e-14	1081
GPX1	0.238260136164324	2.03498878702441e-15	4.5549832349563e-14	1081
PDIA3	0.238160780360409	2.091681616685e-15	4.67149957517952e-14	1081
SLC25A2[illegible]	0.238057119546522	2.15248645823946e-15	4.79666368376481e-14	1081
TMED9	0.238024705609746	2.17185398589577e-15	4.83447497744424e-14	1081
COX6B1	0.237953795174184	2.21482292391836e-15	4.92468077288471e-14	1081
TAPBPL	0.237776633484329	2.32586324982012e-15	5.16018889511304e-14	1081
NSUN5	0.237742588222472	2.34782053911732e-15	5.20069643664261e-14	1081
CYTH4	0.237703437732962	2.37332247885939e-15	5.24814284704965e-14	1081
COX5B	0.237648412855056	2.40962575330251e-15	5.32257793862708e-14	1081
CD274	0.237623229811727	2.42642216869258e-15	5.35380882675925e-14	1081
ZC3H12D	0.237552924679383	2.47392558292381e-15	5.4466918981421e-14	1081
C17orf60	0.23748605458196	2.51995609841741e-15	5.5419776858754e-14	1081
MAP4K2	0.237444878724776	2.54871767420876e-15	5.58693335657623e-14	1081
YIF1B	0.237381142634994	2.59387542301943e-15	5.67974134747026e-14	1081
NAA10	0.237360120176571	2.6089419208225e-15	5.70034002114635e-14	1081
TIAF1	0.237349182090022	2.6168150947848e-15	5.71134778812998e-14	1081
MS4A4A	0.23721650559458	2.71419554163631e-15	5.90469429657272e-14	1081
SNX20	0.237122883821175	2.78504665820523e-15	6.05229395140715e-14	1081
CRYBB1	0.237003761381903	2.87783016214156e-15	6.23375146412278e-14	1081
HSPA5	0.236980580008059	2.89623603871207e-15	6.26688238451715e-14	1081

SCYL1	0.236783606575723	3.05737459507619e-15	6.60137312087993e-14	1081
ORMDL2	0.236771221056618	3.06779634357979e-15	6.61678344126498e-14	1081
GPR18	0.236731601850142	3.10136891249068e-15	6.68204029327537e-14	1081
SRGN	0.236615439015115	3.20189930204532e-15	6.89126724783152e-14	1081
SLC2A6	0.236357542639984	3.43668254819036e-15	7.36510318439306e-14	1081
PGAM1	0.236024037300135	3.76553709995505e-15	8.01021593226974e-14	1081
TP53I13	0.235989598934297	3.80120826477982e-15	8.07756756265713e-14	1081
AP2S1	0.235894274375128	3.90168677860887e-15	8.28234775079829e-14	1081
CDKN3	0.235877597564295	3.91953165962362e-15	8.31147002980187e-14	1081
PLA2G4C	0.235854879736425	3.94396984494911e-15	8.35449763685593e-14	1081
DOK2	0.235777931140632	4.02786381158057e-15	8.51430393329386e-14	1081
XCL1	0.235743000611903	4.06652379927277e-15	8.57802323940836e-14	1081
TRADD	0.235463048179724	4.38983171714389e-15	9.20959773488876e-14	1081
KIR2DL3	0.235460106362402	4.3933598526821e-15	9.20959773488876e-14	1081
FARSA	0.235452146434011	4.40292021483823e-15	9.22004446236134e-14	1081
PFDN6	0.235430574136214	4.42893281115272e-15	9.26488592738023e-14	1081
UBE2M	0.235340383134313	4.53933419824015e-15	9.4859841725672e-14	1081
ECE2	0.235205637269416	4.70933264104023e-15	9.80056880720614e-14	1081
CD3D	0.235158212697393	4.77064120411627e-15	9.91791197697857e-14	1081
SHKBP1	0.234979749879952	5.00846278458519e-15	1.03695254671602e-13	1081
PTPRO	0.234949860569747	5.04941722559221e-15	1.04328728214928e-13	1081
RASSF4	0.234914828624561	5.09783729695084e-15	1.05113543855757e-13	1081
AURKA	0.234871463584789	5.15840768058734e-15	1.06253704218233e-13	1081
MRPL47	0.234607593897046	5.5425153200256e-15	1.13816484324073e-13	1081
BAX	0.234553913713206	5.62402984418262e-15	1.1537279145729e-13	1081
AGTRAP	0.234494848118201	5.71508425574331e-15	1.17121436756815e-13	1081
ITGB2	0.234419035318083	5.83408204348209e-15	1.19438600371897e-13	1081
MAPK8IP	0.234386326026434	5.88617240047941e-15	1.20382683256505e-13	1081
NCF2	0.23420904561823	6.17654998184432e-15	1.25683433721469e-13	1081
SEC61B	0.234137481977687	6.29771701248997e-15	1.28019686394158e-13	1081
PRR13	0.23404083659651	6.46506598379079e-15	1.31289066777687e-13	1081
TALDO1	0.233902142272461	6.71288812605995e-15	1.35638045435785e-13	1081
MRPS22	0.23385632476554	6.79678963274462e-15	1.37195718588818e-13	1081

HRAS	0.233806995306543	6.8882746188806e-15	1.3869947330867e-13	1081
USMG5	0.233801346787322	6.8988271161721e-15	1.3869947330867e-13	1081
PARVG	0.23379198920285	6.9163438212595e-15	1.389130072575e-13	1081
CMC1	0.233784076945432	6.93118908399787e-15	1.39072514040973e-13	1081
PQLC2	0.233745454630433	7.00410441227098e-15	1.40395704860894e-13	1081
C8orf76	0.233717923818688	7.05653973810842e-15	1.41306156087668e-13	1081
EXOSC4	0.233257233754584	7.99343836743142e-15	1.59115650578988e-13	1081
CAPG	0.233253643860911	8.00119900901043e-15	1.59115650578988e-13	1081
FEN1	0.233233208989802	8.04551642109036e-15	1.59805088633111e-13	1081
PDCL3	0.233205853193271	8.10522118433059e-15	1.6070834720309e-13	1081
VMO1	0.233143140436713	8.24374169082651e-15	1.63294175380236e-13	1081
EME1	0.23312406384575	8.28633786445272e-15	1.63976695755796e-13	1081
HAUS8	0.233069813064274	8.4086603921336e-15	1.66234017271375e-13	1081
LOC38895	0.233036868900739	8.48380581258587e-15	1.67555164798571e-13	1081
BAK1	0.233026946193402	8.50656849508573e-15	1.67840178583254e-13	1081
MAX	0.232964050233429	8.65225417509477e-15	1.70440436883022e-13	1081
METTL5	0.232934439477535	8.72168770081123e-15	1.7141307193448e-13	1081
CCL25	0.232905769292146	8.78943737494525e-15	1.72576233838472e-13	1081
FASLG	0.232628130679288	9.47285760774162e-15	1.8509283851402e-13	1081
CALHM1	0.232580872156385	9.5942766654443e-15	1.87096874590882e-13	1081
TUBG1	0.232577389557449	9.603284602977e-15	1.87096874590882e-13	1081
CCL13	0.232353908698289	1.01990454613576e-14	1.98511855863814e-13	1081
G6PD	0.232294255355117	1.03641182350168e-14	2.01141774416583e-13	1081
TMC6	0.23210732310258	1.08985863684983e-14	2.10904920646876e-13	1081
RDM1	0.232024259741394	1.11446824631818e-14	2.152537183325e-13	1081
ACOT13	0.231878384654267	1.15901569906505e-14	2.22577419043521e-13	1081
ACAP1	0.231793624348583	1.18569875356681e-14	2.27484775148603e-13	1081
RPS6KB2	0.23177314713133	1.1922350960501e-14	2.28521179923208e-13	1081
EMP3	0.231766618338486	1.19432652565048e-14	2.28704447331074e-13	1081
AURKB	0.231755577243896	1.19787162964155e-14	2.29165469887265e-13	1081
CENPM	0.231715854563004	1.21071161915779e-14	2.31402140113223e-13	1081
EBNA1BP	0.231505244096855	1.28108224039161e-14	2.4415706464228e-13	1081
GALR2	0.231386981490292	1.32234302628629e-14	2.51307549665447e-13	1081

DUS2L	0.231294470980728	1.35552679912791e-14	2.57371228731685e-13	1081
CLPTM1L	0.231046044620087	1.44874021708232e-14	2.73266588699657e-13	1081
SSTR3	0.230991883799078	1.46988337543118e-14	2.7699532832611e-13	1081
ABP1	0.230946044700111	1.48801456788778e-14	2.79955901407798e-13	1081
C20orf118	0.230849694798334	1.52684411319957e-14	2.86924203921691e-13	1081
H2AFX	0.230649600687025	1.61069086826368e-14	3.01555460419812e-13	1081
CTSC	0.230499552213391	1.67651902427911e-14	3.13297548646592e-13	1081
IFNG	0.230478033651639	1.68617364959272e-14	3.14809714282162e-13	1081
LOC10014	0.230406535948886	1.71864606508004e-14	3.20575231305902e-13	1081
POLD4	0.230331619299725	1.75333038758712e-14	3.25838013449655e-13	1081
NDUFS6	0.23018735332747	1.82206958312516e-14	3.38300384811579e-13	1081
PSMD11	0.230092257011006	1.86882127511358e-14	3.46342268511161e-13	1081
E2F1	0.230058590508685	1.88565339303773e-14	3.49140511054643e-13	1081
DDX27	0.230044259417036	1.89286356660305e-14	3.49832445405673e-13	1081
C17orf79	0.229884997088853	1.97483631562498e-14	3.64647823815446e-13	1081
PLA2G7	0.229833332922872	2.00216964539973e-14	3.6935629584778e-13	1081
CLEC4C	0.229754875245512	2.04439047196245e-14	3.7680005542254e-13	1081
RALY	0.229630922153532	2.11288269334401e-14	3.89067841475459e-13	1081
NDUFB4	0.229581514625169	2.1408075221299e-14	3.93849931811021e-13	1081
AARS	0.229566562474521	2.14932961712713e-14	3.95056981177245e-13	1081
ATP1A3	0.229460516442816	2.21073511705438e-14	4.04428032260809e-13	1081
SAC3D1	0.229457751714214	2.21235885605069e-14	4.04428032260809e-13	1081
IL2RB	0.22939577316327	2.24906822297415e-14	4.10665968110872e-13	1081
CSAG1	0.229393276261704	2.2505595869665e-14	4.10665968110872e-13	1081
CLEC7A	0.229328736978963	2.28944683329777e-14	4.1738376883967e-13	1081
FLT3LG	0.229272328801043	2.32397478475008e-14	4.23295407222335e-13	1081
ASF1B	0.229258500326985	2.33251708023253e-14	4.24467539126327e-13	1081
NLRC4	0.229178096397237	2.38279964226647e-14	4.32835877308008e-13	1081
TRIM31	0.229073298474469	2.44993858589053e-14	4.44630746060944e-13	1081
C6orf153	0.229040228690322	2.47150793080864e-14	4.48141559551215e-13	1081
MIIP	0.228956560562071	2.52691582171458e-14	4.57776252054319e-13	1081
ARHGAP9	0.228951413531105	2.53036391634606e-14	4.57989048470723e-13	1081
ASPHD2	0.228838540368489	2.60715245139338e-14	4.71041131240535e-13	1081

PEF1	0.228829518079255	2.6133882513926e-14	4.71744680325305e-13	1081
GEMIN6	0.228654802481499	2.73707346047211e-14	4.92306650546523e-13	1081
CTSD	0.228568197220299	2.80049683333492e-14	5.03265019692524e-13	1081
C3AR1	0.228440466907659	2.896679712939e-14	5.19622554026324e-13	1081
CDCA5	0.228184604044744	3.09920912814829e-14	5.53489077008399e-13	1081
SELK	0.228087044324326	3.18004138727129e-14	5.66618948687865e-13	1081
KLRC1	0.228085703403193	3.18116669628183e-14	5.66618948687865e-13	1081
MAP4K1	0.227960553996607	3.28793256695084e-14	5.84086433520499e-13	1081
CXorf21	0.227899043653198	3.34168959461762e-14	5.93113100295788e-13	1081
POMP	0.227859957317468	3.37629666779189e-14	5.98201375309303e-13	1081
TROAP	0.227689471511318	3.53140619724888e-14	6.22398756286777e-13	1081
HPCAL1	0.2275178119921	3.69464885427544e-14	6.50600534697367e-13	1081
NDUFA1	0.227485792891918	3.72590765049553e-14	6.55531961740021e-13	1081
FAM104A	0.22742564725581	3.78532879907715e-14	6.64825184458668e-13	1081
CNPY2	0.227396711412754	3.81424667592711e-14	6.69320551276582e-13	1081
PTRH2	0.227393362158949	3.817607826505e-14	6.69327325195328e-13	1081
ATP5EP2	0.22738192995029	3.82910256578615e-14	6.70758879893582e-13	1081
DDRGK1	0.227248191036506	3.96612360770061e-14	6.93555209002854e-13	1081
POP5	0.227217858540959	3.99786407096489e-14	6.98499320985149e-13	1081
NGDN	0.227214119375559	4.00179402868905e-14	6.9858007545876e-13	1081
BRI3	0.227167182689192	4.05144954104741e-14	7.06024662667821e-13	1081
RBX1	0.227150164073488	4.06960301640822e-14	7.07963322673088e-13	1081
CYC1	0.227128525864465	4.09279951049214e-14	7.11384349774497e-13	1081
GIMAP2	0.227006806187952	4.22572457037749e-14	7.32592267386012e-13	1081
HLA-DMA	0.226891513315528	4.35553612771792e-14	7.544477669207e-13	1081
PRAF2	0.226676151146082	4.60859096039013e-14	7.96910428300938e-13	1081
EBP	0.226612548109928	4.68604885892318e-14	8.08915632073758e-13	1081
PDE6D	0.226589210417447	4.71478966546874e-14	8.1269071006189e-13	1081
SAPS1	0.226588241775628	4.71598629963936e-14	8.1269071006189e-13	1081
RAD23A	0.226530393206793	4.78799417727622e-14	8.2369037319581e-13	1081
SNF8	0.226408695877108	4.94302127941757e-14	8.49634502336749e-13	1081
MRPL22	0.2263256762783	5.05159477432296e-14	8.6607980194669e-13	1081
HSPB11	0.22626274664826	5.13545138860946e-14	8.78960647608646e-13	1081

FCN1	0.226142941157736	5.29889260247996e-14	9.04628741330159e-13	1081
LIN37	0.226117544974621	5.33418908670398e-14	9.09883481385704e-13	1081
NDUFB11	0.226110526166322	5.3439847138323e-14	9.10783181557967e-13	1081
CKS2	0.226030599498347	5.45678571218764e-14	9.28437062263683e-13	1081
SSB	0.225971694430597	5.54141186984222e-14	9.41245717689472e-13	1081
ASPSCR1	0.225753828056701	5.86575463057009e-14	9.9132237443653e-13	1081
MRPL20	0.225718672852841	5.91980876281024e-14	9.9961900693053e-13	1081
NOC4L	0.225521461420328	6.23222769352595e-14	1.05149268748811e-12	1081
CD37	0.225499454959035	6.2680781740222e-14	1.05590561898413e-12	1081
CSK	0.22549322283671	6.27826758906578e-14	1.05660568572874e-12	1081
GLT1D1	0.22536064353003	6.49892608326069e-14	1.09009880056026e-12	1081
OBFC2B	0.225123767167473	6.91228038859379e-14	1.15846828975226e-12	1081
C17orf62	0.225063696251991	7.02114240635145e-14	1.17378351681286e-12	1081
NCR3	0.22498587162166	7.16468383732701e-14	1.19678736237937e-12	1081
CNPY3	0.224964395797837	7.20479889373929e-14	1.20249108296916e-12	1081
CLTA	0.224961064887377	7.21104047665754e-14	1.20253650995253e-12	1081
BCL7B	0.224656094519569	7.80557227438477e-14	1.29524920483922e-12	1081
ARHGDIA	0.224555420304175	8.01220113842307e-14	1.32844273196323e-12	1081
RFXANK	0.224519315437694	8.08760498019793e-14	1.33984212439217e-12	1081
ACOT8	0.224218288995594	8.74400809022528e-14	1.43987212037591e-12	1081
MRPS18A	0.22421626664925	8.74859009848655e-14	1.43987212037591e-12	1081
C1orf135	0.224107802164272	8.99782039965096e-14	1.47606752403069e-12	1081
IL10RA	0.224051359611483	9.13025783285947e-14	1.49657480913714e-12	1081
SPC25	0.224016747879476	9.21241558602168e-14	1.50758823704636e-12	1081
SLC35E4	0.224007385546227	9.23476332835514e-14	1.51001872767625e-12	1081
CD247	0.223942556380894	9.39097608860656e-14	1.53307304136936e-12	1081
RIMBP3	0.223830713071534	9.66659333929772e-14	1.57678965846277e-12	1081
CTSW	0.223824438811054	9.68228810022464e-14	1.57807195614098e-12	1081
SYNGR2	0.223758961720375	9.84757560496547e-14	1.60241850211655e-12	1081
SNRPF	0.223674913559016	1.00638070789456e-13	1.63496285165612e-12	1081
CLIC3	0.223643720864986	1.01452367732538e-13	1.64686377757614e-12	1081
PSMA2	0.223546297973567	1.04037490799297e-13	1.68611042007388e-12	1081
LTB	0.223517316028677	1.04818928841251e-13	1.69740942243329e-12	1081

TMC8	0.223478990714295	1.05861142660941e-13	1.71153508740341e-12	1081
PTPMT1	0.223303692296991	1.10759294585855e-13	1.78356993559716e-12	1081
KPNA2	0.223189807047619	1.14059929439134e-13	1.83090297485583e-12	1081
S1PR4	0.223189729460794	1.14062210645027e-13	1.83090297485583e-12	1081
CXCR6	0.223175281888909	1.14487779176061e-13	1.834810112571e-12	1081
VAMP5	0.22300899276164	1.19499599654572e-13	1.90905585649592e-12	1081
CYTH1	0.222853135742819	1.24392147697024e-13	1.98249985392133e-12	1081
TBCA	0.222804497100121	1.25958832895473e-13	2.00588196733542e-12	1081
PARD6A	0.222781333928375	1.26711725729937e-13	2.01309756690031e-12	1081
EPR1	0.222709591228102	1.29071773022667e-13	2.04736288782805e-12	1081
CST7	0.22265530083948	1.30886329783499e-13	2.07288137852876e-12	1081
CDK5	0.222651304696695	1.31020878294071e-13	2.07338224134647e-12	1081
MS4A6A	0.222645082989086	1.31230630879674e-13	2.07507147493802e-12	1081
EIF2B3	0.222625580101302	1.31890269899357e-13	2.08386626440984e-12	1081
SLC6A12	0.222463287868985	1.37507101315205e-13	2.16751217213991e-12	1081
RRM2	0.222443738783204	1.3819932399472e-13	2.17672039239533e-12	1081
GLRX	0.222267472960279	1.44597217407094e-13	2.26157682039278e-12	1081
VOPP1	0.222162701789857	1.48536863871688e-13	2.31959311836833e-12	1081
CREM	0.221915017827109	1.58273962649958e-13	2.46640637600265e-12	1081
PTPRCAP	0.221516664438183	1.75262948056138e-13	2.72428401897447e-12	1081
C19orf62	0.22148196901243	1.76824648267404e-13	2.74643989155501e-12	1081
GALE	0.221477574944915	1.77023404205763e-13	2.74740868854013e-12	1081
COMTD1	0.221424042960269	1.79462477458134e-13	2.78311902108861e-12	1081
CDT1	0.221402017650682	1.8047556132559e-13	2.79667706377232e-12	1081
BIN2	0.221368409702379	1.82032226311914e-13	2.81863120603651e-12	1081
OSM	0.221338045335664	1.834499765542e-13	2.83622392761655e-12	1081
BTK	0.221296334494738	1.85415183919835e-13	2.86154728780604e-12	1081
KLRC2	0.22129424734141	1.85514060951833e-13	2.86154728780604e-12	1081
CSTB	0.221262140218356	1.87041642253848e-13	2.88290274154841e-12	1081
KCNJ10	0.221224810827553	1.8883320592671e-13	2.90729532380378e-12	1081
PCNA	0.221223159202825	1.88912860702862e-13	2.90729532380378e-12	1081
PSMD7	0.221118654010077	1.94020551142743e-13	2.98362137616074e-12	1081
TIMM10	0.221102143733749	1.94839788278674e-13	2.99165208450754e-12	1081

CHMP1A	0.220956794802809	2.02199970767945e-13	3.10229886604741e-12	1081
NPL	0.220923074235426	2.03946110791679e-13	3.12433034364895e-12	1081
C19orf53	0.220663424954263	2.17896151975966e-13	3.33043852925329e-12	1081
HIST1H2I	0.220624074679171	2.20090409364938e-13	3.36142630527421e-12	1081
SLC25A1(	0.220562865573514	2.23546714926702e-13	3.41162770621091e-12	1081
CDC20	0.220516245916355	2.26214863657616e-13	3.44712437850429e-12	1081
PLAC8	0.220448893720251	2.30124832594525e-13	3.50140842342652e-12	1081
SFRS9	0.220407180804775	2.32579537868125e-13	3.53505677608674e-12	1081
MYLK2	0.22035402685371	2.35744767226888e-13	3.57611320465788e-12	1081
LIN7B	0.220215200379807	2.4421230803208e-13	3.70177347276617e-12	1081
RELL2	0.220182737405549	2.46234981024883e-13	3.72682471280711e-12	1081
SARNP	0.220115483685631	2.50477791716695e-13	3.78535267376806e-12	1081
OSGIN1	0.220086149630443	2.52350762346516e-13	3.81079918101241e-12	1081
ATP5C1	0.220057454478904	2.54196229027632e-13	3.83292143245632e-12	1081
CSNK2B	0.219962631391596	2.60389220445097e-13	3.92043411499736e-12	1081
MDK	0.21994752748445	2.61389241272658e-13	3.93255135581606e-12	1081
TBC1D10	0.219935839577498	2.62165675338289e-13	3.94128920126106e-12	1081
ABCB6	0.219684758414289	2.79402191573621e-13	4.18169178993357e-12	1081
UBE2T	0.219644196291475	2.82289122779062e-13	4.22176271594967e-12	1081
HIST1H3(	0.219496359080567	2.9306092702333e-13	4.36988332707992e-12	1081
NDUFA6	0.219380717873554	3.01766920638405e-13	4.48973014494879e-12	1081
NKAP	0.219276367489085	3.09840289562958e-13	4.60304766463553e-12	1081
DNAJA1	0.219003087013155	3.32000721473116e-13	4.91776068682053e-12	1081
MRPL36	0.218933855678691	3.37857075601336e-13	4.99715916886118e-12	1081
IFI16	0.218911166739035	3.39798309510202e-13	5.02218411231329e-12	1081
COX4NB	0.218885177071705	3.420353928052e-13	5.05154178010319e-12	1081
GADD45(	0.218866576734586	3.43645294455009e-13	5.0716003346492e-12	1081
CYBA	0.218861461208954	3.44089356300712e-13	5.07443637092082e-12	1081
SNRPA1	0.218844058643552	3.45604233109583e-13	5.09304848280363e-12	1081
TOR2A	0.218812694505309	3.4835098574383e-13	5.12977383611803e-12	1081
PPIB	0.218790381022811	3.50318138281075e-13	5.1549736272259e-12	1081
PQBP1	0.218773929599394	3.51775465829514e-13	5.17263997017194e-12	1081
IPO13	0.218677811325296	3.60409580926326e-13	5.28801967061969e-12	1081

GZMA	0.218606333572124	3.66964693417783e-13	5.37636636283726e-12	1081
SNX10	0.218520136948464	3.75025296179474e-13	5.48648118484786e-12	1081
MAD2L1E	0.218517043998344	3.75317734706305e-13	5.48677486622534e-12	1081
FAM58A	0.218436686739912	3.82994386042647e-13	5.59493974389349e-12	1081
PPM1G	0.218385153055201	3.87998294060913e-13	5.66393161873702e-12	1081
RHBDD3	0.218368051730324	3.89672953283506e-13	5.68425897458091e-12	1081
HSPE1	0.218265704471254	3.99844542479683e-13	5.81935836876232e-12	1081
WDR62	0.218263267177443	4.00089915151939e-13	5.81935836876232e-12	1081
TMEM179	0.218234101127778	4.03037665170794e-13	5.85378065239052e-12	1081
CHMP4B	0.217956470735762	4.32184966343996e-13	6.26357276762575e-12	1081
ALDH3B1	0.217920475748255	4.3611214364272e-13	6.31140742362256e-12	1081
ISOC2	0.217877358174212	4.40862501440514e-13	6.37557436577111e-12	1081
ANXA10	0.217867756710763	4.41927202775581e-13	6.38638701572029e-12	1081
TAF10	0.217796972699356	4.49854596182682e-13	6.48433263021128e-12	1081
IRAK1	0.217795753037372	4.49992405908929e-13	6.48433263021128e-12	1081
ORC6L	0.217786874736988	4.50996814112806e-13	6.49416070071657e-12	1081
PSMF1	0.217763855620423	4.53611232316885e-13	6.52248270879633e-12	1081
PSMD3	0.217632536264811	4.68812625586029e-13	6.72665978805595e-12	1081
PSMC3IP	0.217605402451163	4.72015313883996e-13	6.76779252540434e-12	1081
GMPPA	0.217581192762486	4.74890961591e-13	6.79934500444257e-12	1081
NDUFB2	0.217577002929418	4.75390374257036e-13	6.8016612850909e-12	1081
COX7A2	0.217374605835263	5.00138515312004e-13	7.13042490513823e-12	1081
LY96	0.217350616289632	5.03154293248412e-13	7.15622900762127e-12	1081
BLVRA	0.217322581881946	5.06701156927981e-13	7.19449992834548e-12	1081
RELT	0.217253728490289	5.15516735166785e-13	7.30315374819612e-12	1081
APRT	0.21721720228081	5.20254200890579e-13	7.35074943606996e-12	1081
SLC25A39	0.217216574481006	5.20335998800485e-13	7.35074943606996e-12	1081
LRRC59	0.217200273399917	5.22464335217378e-13	7.37564403150251e-12	1081
ARPC1B	0.217194041576351	5.23280241062545e-13	7.38198911498948e-12	1081
ATF5	0.217151041807701	5.28944163963582e-13	7.44624750736992e-12	1081
UBL5	0.217137618068159	5.30724616135138e-13	7.46609454751561e-12	1081
HES4	0.217007955784721	5.48227625986677e-13	7.69619897247499e-12	1081
UPP1	0.216961238379379	5.54671647924575e-13	7.76501761462165e-12	1081

ABCB9	0.216902563171897	5.62870366666692e-13	7.87432190034758e-12	1081
UBB	0.216899636443796	5.63282416199325e-13	7.87461781702666e-12	1081
SDSL	0.216775251814247	5.81070368881085e-13	8.1064145298542e-12	1081
PLEKHO1	0.216684024740839	5.94465459011389e-13	8.2760930696506e-12	1081
IL32	0.216570777121222	6.11515591369663e-13	8.50171262121592e-12	1081
TSIX	0.216567693963933	6.11986420781773e-13	8.50239065286125e-12	1081
IL12B	0.216562644593448	6.12758278628615e-13	8.50724708681837e-12	1081
CDKN2B/	0.216459986978447	6.28659305056216e-13	8.72199841622415e-12	1081
NDUFA2	0.216306283306831	6.53226011170258e-13	9.03173506865123e-12	1081
SLC3A2	0.216300274567183	6.54205272558975e-13	9.03907079266155e-12	1081
EXOSC6	0.21628429100379	6.56817170285387e-13	9.06893892762106e-12	1081
IL21R	0.216265174180168	6.59954505862647e-13	9.10601611000207e-12	1081
TNFRSF1	0.216210957169889	6.68932397857211e-13	9.21096592948293e-12	1081
CLIC1	0.216201670427727	6.70482170151174e-13	9.22599953394494e-12	1081
MRPL52	0.216169745536003	6.75836693351105e-13	9.29333118604643e-12	1081
MYBL2	0.216058079731306	6.94897355506544e-13	9.53590410536739e-12	1081
PLA2G15	0.215929531880736	7.17492685642041e-13	9.82589405320117e-12	1081
PLAC1	0.215878370838947	7.26684440712498e-13	9.94501226776717e-12	1081
CLN6	0.215727269172054	7.54510969017998e-13	1.02769597504176e-11	1081
SLC17A9	0.215723537976002	7.55211146597848e-13	1.02795463163606e-11	1081
CXCL9	0.215666176744665	7.66055768480028e-13	1.04060643668443e-11	1081
HIST1H2I	0.215659592368938	7.67310324136853e-13	1.04137031608016e-11	1081
RNF8	0.215635663661262	7.71886567483456e-13	1.04641015490944e-11	1081
QPRT	0.215627288850354	7.73494518810428e-13	1.04788480709052e-11	1081
NDUFV3	0.215610537774288	7.76720558043482e-13	1.05154809420604e-11	1081
CDC25C	0.215510056725646	7.96350557826749e-13	1.07739973051846e-11	1081
PSMB1	0.21546343500079	8.05622952308895e-13	1.08702440550989e-11	1081
CHAF1B	0.215369256129027	8.24677733229838e-13	1.11124635022843e-11	1081
CARD11	0.215359333266809	8.26710919892553e-13	1.11249776093757e-11	1081
GIMAP4	0.215307121398059	8.37490299288421e-13	1.12625114013119e-11	1081
VPS4A	0.215299542593859	8.39066384164705e-13	1.1268661539332e-11	1081
MRPS12	0.215268787691751	8.45492049704611e-13	1.13322936402524e-11	1081
PSMD9	0.215213607346456	8.57142073777531e-13	1.14734488535873e-11	1081

C19orf28	0.215078613994034	8.86310045376032e-13	1.18243151417882e-11	1081
MYO1F	0.215046610050274	8.93366521600682e-13	1.1910568218164e-11	1081
RPP21	0.214979804075268	9.0827431596621e-13	1.20933153305613e-11	1081
PPIL1	0.214950127220683	9.14974679325565e-13	1.21744814498108e-11	1081
KIF2C	0.214914609255841	9.23057538744653e-13	1.22416024476702e-11	1081
ANKRD3!	0.21476354434929	9.58224402088876e-13	1.26746097045833e-11	1081
FCN3	0.214726653052181	9.67009970205012e-13	1.27824250982808e-11	1081
NOP10	0.214712331774067	9.70441775657076e-13	1.28193767676143e-11	1081
SDHAF2	0.214646351233187	9.86407475683875e-13	1.30132145367725e-11	1081
HMGB3	0.21463511965489	9.89150766990947e-13	1.30408653148119e-11	1081
NFKBIB	0.214613334225281	9.94493149397496e-13	1.30941598004004e-11	1081
CHCHD1	0.21443053560554	1.04044868634948e-12	1.36457283766342e-11	1081
MGAT1	0.214414066721099	1.04468971755538e-12	1.36924361484405e-11	1081
IFI27L2	0.214293744995872	1.0761929380719e-12	1.40778615178301e-11	1081
EIF6	0.214279922932347	1.07987097691038e-12	1.41168078065279e-11	1081
TNFRSF9	0.214249493315862	1.08801173651256e-12	1.42140054682526e-11	1081
IL18RAP	0.214001449088683	1.15665228161178e-12	1.51009463467721e-11	1081
VCP	0.213961557771728	1.16808038640786e-12	1.52402716218823e-11	1081
RNFT2	0.213895167757202	1.18734563611233e-12	1.54516006715005e-11	1081
GALNS	0.213826227234806	1.20768048767719e-12	1.56959505962948e-11	1081
AIFM1	0.213638319525075	1.26485468486407e-12	1.63967166194252e-11	1081
TCL1A	0.213615941537727	1.27183784422499e-12	1.64756139158031e-11	1081
CD3E	0.21360300553702	1.27589180183139e-12	1.6497330133436e-11	1081
SLAMF7	0.21358339941184	1.28206022991349e-12	1.65664549914094e-11	1081
POLA2	0.213430187889938	1.33128018777031e-12	1.71694234203796e-11	1081
ELOF1	0.21342596940851	1.33266126025764e-12	1.71762386998657e-11	1081
ARHGAP!	0.213409636235918	1.33802176743404e-12	1.7234302113145e-11	1081
SAP30BP	0.213253940752674	1.39019285714904e-12	1.78720070882371e-11	1081
H2AFJ	0.21321739912044	1.40272323392182e-12	1.80215940990785e-11	1081
C17orf53	0.213137112029624	1.4306441638792e-12	1.83335411458948e-11	1081
IL1RN	0.212969182991004	1.49081583818851e-12	1.90561453428348e-11	1081
FAM78A	0.212932262508636	1.50437383733442e-12	1.92050766496209e-11	1081
NUTF2	0.212910186948174	1.51253809213156e-12	1.92970740126601e-11	1081

EMD	0.212861078697477	1.53085591495419e-12	1.95060673034486e-11	1081
PTGES2	0.212687655008265	1.59730023571179e-12	2.02757487387611e-11	1081
GPSM3	0.212666003587827	1.60579146427377e-12	2.03706983928181e-11	1081
SEC61G	0.212650141453788	1.61204030835116e-12	2.04242465482604e-11	1081
KIR3DL2	0.212582222494258	1.63906726737305e-12	2.0740584234441e-11	1081
CCR7	0.212574415935224	1.64220202840947e-12	2.07672064421273e-11	1081
KCTD17	0.21249772134679	1.67331308588964e-12	2.11208597213326e-11	1081
MRPS5	0.212474355042628	1.68290582922022e-12	2.12286399058494e-11	1081
CLECL1	0.21236287917337	1.72941752390997e-12	2.17880650526368e-11	1081
CDK5RAI	0.212346318938326	1.73643361887317e-12	2.1862784532625e-11	1081
AQP9	0.212271832925719	1.76833750836371e-12	2.22366785930006e-11	1081
ICAM3	0.212181705828518	1.80770921977428e-12	2.26961751085642e-11	1081
PANK2	0.212180462824808	1.80825818065255e-12	2.26961751085642e-11	1081
SHARPIN	0.21215097043007	1.82133122959327e-12	2.28460259154149e-11	1081
CCDC137	0.212056980040254	1.8636149511304e-12	2.32894064457332e-11	1081
RAD54L	0.212051261651536	1.86621830266684e-12	2.33074815295868e-11	1081
SKA1	0.211899401533151	1.93667255079409e-12	2.41126505165308e-11	1081
PTTG3P	0.211833311977635	1.96814185848557e-12	2.44742084809826e-11	1081
IL10RB	0.211781866768562	1.99298428508359e-12	2.47678398661375e-11	1081
HCLS1	0.211613705591256	2.0763498256205e-12	2.57720685379698e-11	1081
RNF113A	0.211601779277716	2.08239029692154e-12	2.58152323270673e-11	1081
ATG4D	0.211563373603249	2.10195930325971e-12	2.60418020689833e-11	1081
NDUFB7	0.211548233732587	2.10972298679832e-12	2.61219235212367e-11	1081
PLA1A	0.211500835926528	2.13421050394647e-12	2.64088885761681e-11	1081
CCL3L1	0.211435777768119	2.16827586901607e-12	2.67810652246038e-11	1081
C9orf100	0.211396715240712	2.18898484253429e-12	2.70202816500326e-11	1081
CDC45	0.211314194233027	2.23337225686642e-12	2.75344456025544e-11	1081
TPI1	0.211175997569038	2.30968938050225e-12	2.84579159450873e-11	1081
CD70	0.211173029544388	2.31135613333323e-12	2.84610448080671e-11	1081
RLTPR	0.211156498140617	2.32066124601878e-12	2.85407330897731e-11	1081
C12orf62	0.211148780760258	2.32501771836434e-12	2.85768651229101e-11	1081
SPAG5	0.211105583382725	2.34955100125731e-12	2.88607956831272e-11	1081
MRPS26	0.210900622876763	2.46945376437821e-12	3.02598212186125e-11	1081

SSSCA1	0.210768508804513	2.54989606584247e-12	3.11886194574356e-11	1081
RIMBP3C	0.210720557302408	2.57972322473484e-12	3.15342987635215e-11	1081
PDIA3P	0.210648969911597	2.62488942204423e-12	3.20281026087711e-11	1081
PDIA6	0.210569001417682	2.67625947515391e-12	3.25956754092959e-11	1081
H2AFZ	0.210492387777158	2.72639728886187e-12	3.31862679058141e-11	1081
RPS6KA4	0.210484811359469	2.73140517440539e-12	3.32070954969201e-11	1081
CENPA	0.21047018950118	2.74109546006535e-12	3.32847305865078e-11	1081
FXYD2	0.210452560256669	2.75282359219267e-12	3.33946151434702e-11	1081
AK2	0.210426269168028	2.77040549688853e-12	3.35799150028998e-11	1081
GTSF1L	0.210406958960567	2.7833890061992e-12	3.36967376982469e-11	1081
LY86	0.210319488110962	2.84295152559919e-12	3.43765056921943e-11	1081
KIAA0101	0.210290388012856	2.86304231432126e-12	3.45779301091138e-11	1081
CCDC64	0.210231080737819	2.90441960307124e-12	3.5056640445698e-11	1081
CDCA3	0.210213372016713	2.91688769427899e-12	3.51439608859152e-11	1081
NDC80	0.210199032395719	2.9270221108336e-12	3.52449853094698e-11	1081
TMEM51	0.21012281985419	2.98146609651484e-12	3.58791126130773e-11	1081
UTS2	0.209923680716993	3.12844919745688e-12	3.76254382583694e-11	1081
CTSH	0.209844396443683	3.18892284075922e-12	3.82386015637467e-11	1081
COMMD4	0.209686135988924	3.31307762500663e-12	3.96094651369488e-11	1081
PGD	0.209665876364628	3.32930905423894e-12	3.97799115644385e-11	1081
PARL	0.209576294294178	3.40201787695836e-12	4.06245703208809e-11	1081
MCOLN1	0.209510167307193	3.45668469252446e-12	4.12284861639463e-11	1081
C1orf54	0.209458121125404	3.50031501439499e-12	4.17241692100516e-11	1081
SURF1	0.209435473733035	3.51946826630307e-12	4.19028890216757e-11	1081
SIRT7	0.209420458261006	3.53222361698155e-12	4.20299142138767e-11	1081
RAD9A	0.20904471548746	3.86656413922409e-12	4.57112292163552e-11	1081
CHAC1	0.208876861677829	4.02575429126898e-12	4.75374092600314e-11	1081
FCGR3B	0.208742589160479	4.15769987331806e-12	4.90379765503468e-11	1081
C1orf212	0.208620469244351	4.28137381301769e-12	5.03786655743232e-11	1081
RECQL4	0.208543315287672	4.36135845870559e-12	5.12300677263114e-11	1081
PDCD2L	0.208511973595718	4.39426622698086e-12	5.15865344653435e-11	1081
TRPM2	0.208507038562063	4.39946995576716e-12	5.16175435404364e-11	1081
GRAMD1	0.208391220828518	4.52334038789935e-12	5.29783093687398e-11	1081

C16orf75	0.208358776383763	4.55864857728758e-12	5.33298348370838e-11	1081
C7orf33	0.208257649117956	4.67044188387719e-12	5.44479466149919e-11	1081
IRF3	0.208254282647963	4.67420920899035e-12	5.44603496327996e-11	1081
GIPC1	0.208169057134345	4.77057989232041e-12	5.55510589195345e-11	1081
FAM100B	0.208154798753816	4.7868916882766e-12	5.57088001503941e-11	1081
MICB	0.208032095311296	4.92954147256954e-12	5.72366645330913e-11	1081
SNRPB2	0.208022623125661	4.94072483841261e-12	5.73334688190219e-11	1081
NAT8B	0.207948627554258	5.0289469150362e-12	5.82900665151924e-11	1081
DTNBP1	0.207935253573019	5.04505591243095e-12	5.84389260722806e-11	1081
LAGE3	0.207845004379391	5.15508947257341e-12	5.95124798997085e-11	1081
TMEM138	0.207836642836391	5.16540213224814e-12	5.95701644227131e-11	1081
SLFN12L	0.207758160993338	5.26318677172816e-12	6.05867985808364e-11	1081
GPR137	0.207688903955017	5.3509810613878e-12	6.15271195671559e-11	1081
HIST1H3E	0.207662690227371	5.38458372108756e-12	6.18781740224238e-11	1081
PSMB4	0.207489854207038	5.61136541217998e-12	6.4410801269724e-11	1081
CYTIP	0.207445760461546	5.67070243391258e-12	6.50548408491851e-11	1081
HIST1H3D	0.207397697420975	5.73608054196031e-12	6.57674117915712e-11	1081
BLOC1S1	0.207356467191447	5.79275169174844e-12	6.63044491787144e-11	1081
SEC61A2	0.207353868562853	5.79634181941974e-12	6.63073855492394e-11	1081
ATP6V0D	0.207294574449624	5.87885372823531e-12	6.71749905588771e-11	1081
C17orf95	0.207215007093007	5.99138590075236e-12	6.83832685386155e-11	1081
PRDX4	0.207175078610359	6.04864849420197e-12	6.89196967848975e-11	1081
HSPB9	0.207163713540085	6.06504516687877e-12	6.90674589523871e-11	1081
C20orf134	0.207151430788306	6.08281471491546e-12	6.92306793400971e-11	1081
PIF1	0.207103018451983	6.15335073963598e-12	6.99149749858808e-11	1081
MLF2	0.207070460765215	6.20123632895066e-12	7.04158397366607e-11	1081
IL17C	0.207065514893202	6.20854251613931e-12	7.04229104660059e-11	1081
SCNM1	0.207013451305748	6.28596530625484e-12	7.12609854217804e-11	1081
TMIGD2	0.206921633494082	6.42481486026567e-12	7.26714740932352e-11	1081
SIPA1	0.206787212992588	6.63351827791069e-12	7.48639919935635e-11	1081
TSGA10IF	0.206598702945849	6.93741701012132e-12	7.81186504577384e-11	1081
ST6GALN	0.206534606135319	7.04381958927314e-12	7.91398469748508e-11	1081
LPAR5	0.206529790982634	7.05187706579244e-12	7.91862115331041e-11	1081

EXOSC9	0.206488480880492	7.12137531931541e-12	7.99220645167737e-11	1081
STX11	0.20646611757206	7.15927751743057e-12	8.02133735198214e-11	1081
POLR2H	0.20645799460094	7.17309345827176e-12	8.03234951177791e-11	1081
JMJD6	0.206449861072745	7.18695349577859e-12	8.04339878735887e-11	1081
PSAP	0.20638281210677	7.30221200564255e-12	8.16785457266347e-11	1081
CD52	0.206246568780206	7.54201649008947e-12	8.42417698002482e-11	1081
MMP9	0.20621695808938	7.59514531519648e-12	8.47668711216804e-11	1081
SHCBP1	0.206209932168107	7.6078051950684e-12	8.4861149310439e-11	1081
YARS	0.206080330060372	7.84507080867818e-12	8.72192963042117e-11	1081
LOC38878	0.206080265593164	7.84519061321576e-12	8.72192963042117e-11	1081
ENY2	0.206073715140746	7.85737317688329e-12	8.73065541358599e-11	1081
SCAND1	0.206013694181469	7.96986581420937e-12	8.85076884383946e-11	1081
PYHIN1	0.205970460046092	8.05187107861228e-12	8.93199024662139e-11	1081
NUP85	0.205695428796574	8.59321339930148e-12	9.47511132615918e-11	1081
KLF1	0.20544490876739	9.1171994541929e-12	1.00199663395917e-10	1081
PFDN2	0.205405263399472	9.20293716759375e-12	1.01086788026814e-10	1081
C11orf10	0.205302443935826	9.42898887887844e-12	1.03400642876977e-10	1081
E2F2	0.205293432354108	9.44905807523108e-12	1.03564349796262e-10	1081
MRPL34	0.205272237934072	9.49642387347186e-12	1.04026894470414e-10	1081
MGC7208	0.205205922316781	9.64613306095936e-12	1.05494761407723e-10	1081
OVCA2	0.205154551127827	9.76368963151758e-12	1.06664602834556e-10	1081
UCKL1	0.20510125688016	9.88712800413484e-12	1.07896096231471e-10	1081
KRTCAP2	0.205089528791034	9.91449647959309e-12	1.08033620038763e-10	1081
NSDHL	0.205088963078738	9.91581848854174e-12	1.08033620038763e-10	1081
TRIM34	0.205016788958328	1.00859038290547e-11	1.09767980894818e-10	1081
CISD2	0.204967170435616	1.02044868734715e-11	1.10998589668511e-10	1081
MEA1	0.204960818444612	1.02197655994432e-11	1.11044864078093e-10	1081
TNFSF12-	0.204956719443219	1.02296369877258e-11	1.11087914998193e-10	1081
OR52N4	0.204949248298604	1.02476533146056e-11	1.11168000012241e-10	1081
GJD3	0.20482987169034	1.05397705857485e-11	1.14091175953737e-10	1081
TMEM189	0.204801767830713	1.06097171498022e-11	1.14786655200196e-10	1081
C6orf1	0.204509119286592	1.13656026930628e-11	1.22242427256674e-10	1081
MRPL21	0.20449635481382	1.13997442379824e-11	1.22413564858292e-10	1081

GEMIN8P	0.204485716144851	1.14282764633686e-11	1.22654570780266e-10	1081
PPP1R7	0.204466788436926	1.14792121578797e-11	1.23106703252643e-10	1081
APOBEC3	0.204447642626896	1.15309606856314e-11	1.23493462526339e-10	1081
CRTAM	0.204438288503275	1.15563265254869e-11	1.23699361241197e-10	1081
MAGOH	0.204425044785135	1.15923332577634e-11	1.24018881294553e-10	1081
GPI	0.204247348331384	1.20861961438131e-11	1.29096723922118e-10	1081
EBI3	0.204210638545867	1.21907573655157e-11	1.301445718751e-10	1081
TMEM147	0.204091260723394	1.25369377598931e-11	1.33698576587108e-10	1081
TNIP3	0.204073556420574	1.25890899987807e-11	1.34112754111812e-10	1081
BATF3	0.204060128020135	1.2628788006373e-11	1.344645530594e-10	1081
ZNF593	0.203871806530646	1.31985863610007e-11	1.40161055478313e-10	1081
ANKRD22	0.203758872049693	1.35522698187005e-11	1.43387854778215e-10	1081
FKBP3	0.203715700930389	1.36899061787757e-11	1.44768062977132e-10	1081
UFD1L	0.203704564999291	1.37256305221909e-11	1.45069688808781e-10	1081
NDUFA7	0.203626192380586	1.39796435740153e-11	1.47676937492679e-10	1081
KLRK1	0.203623775617716	1.39875491621037e-11	1.47683007269695e-10	1081
HLA-DRB	0.203583657427248	1.41194222057604e-11	1.48934525047568e-10	1081
TRAPPC2	0.203527083352466	1.43074561471338e-11	1.50508461662668e-10	1081
COPS6	0.203523757089952	1.43185874741313e-11	1.50546943980363e-10	1081
UQCR10	0.203520733763795	1.43287123732411e-11	1.50574809994231e-10	1081
HIST1H4I	0.203514344201998	1.43501335925918e-11	1.50721293651075e-10	1081
STAT4	0.203472263947823	1.44919934665283e-11	1.52131948089219e-10	1081
TSEN54	0.203304376480962	1.50717492193773e-11	1.58107253510248e-10	1081
CCND3	0.203302915032778	1.5076894216589e-11	1.58107253510248e-10	1081
SURF2	0.203278770314447	1.51621442037297e-11	1.58918519762817e-10	1081
CCNB2	0.203271803257759	1.51868309516249e-11	1.59094492730361e-10	1081
GPR172A	0.203240508811271	1.52982039482384e-11	1.60177920237662e-10	1081
SF3B2	0.203234698154255	1.53189710891157e-11	1.60312037709213e-10	1081
SRPRB	0.203136910288726	1.56726268700603e-11	1.63672922912061e-10	1081
C12orf63	0.203090650508418	1.58426980982831e-11	1.65192108276353e-10	1081
CDCA8	0.203071074651225	1.59152097909067e-11	1.65862338974556e-10	1081
SHFM1	0.20302331753308	1.60934732321781e-11	1.67633411717802e-10	1081
TUBA1B	0.202906918205763	1.65361773545976e-11	1.71977951888678e-10	1081

PSMA1	0.202837382250314	1.68063038632961e-11	1.74427094964503e-10	1081
RAD51C	0.202755709480052	1.71290918048005e-11	1.77411596096507e-10	1081
POC1A	0.202547905159074	1.79779604417212e-11	1.8572616056332e-10	1081
TNFSF9	0.202541291013658	1.80056435220173e-11	1.85916806125597e-10	1081
MDP1	0.202356862850469	1.87945636940855e-11	1.93368991632969e-10	1081
MEP1A	0.202217245449742	1.94141804688702e-11	1.99388782261331e-10	1081
RASAL3	0.202216121209857	1.94192499774997e-11	1.99388782261331e-10	1081
GRIPAP1	0.202210614891909	1.94440981885685e-11	1.9954220988727e-10	1081
DOLK	0.202192951376253	1.9524017853418e-11	2.00260356240889e-10	1081
MAGEF1	0.202172322069709	1.9617763075421e-11	2.01119510002217e-10	1081
RNF7	0.202038771715586	2.0235387923062e-11	2.07029908435797e-10	1081
HIST2H3C	0.202029643162077	2.02782916706623e-11	2.07258338764836e-10	1081
CBLN3	0.201974772948733	2.05380592078507e-11	2.0959432763027e-10	1081
FTH1	0.201891102602271	2.09404476272735e-11	2.1359256579819e-10	1081
PKMYT1	0.201861652822674	2.10839036537656e-11	2.14729645654756e-10	1081
HIST1H1E	0.201853561184344	2.11234876932264e-11	2.15024082657931e-10	1081
LCK	0.201813218238409	2.13219308557744e-11	2.16824985910941e-10	1081
STK10	0.201808276144613	2.13463655133615e-11	2.16963942112345e-10	1081
LMAN2	0.201726493250393	2.17547133263156e-11	2.2088130488682e-10	1081
SRP68	0.201696788612447	2.19049133272603e-11	2.22090714914354e-10	1081
LRFN1	0.201562225974125	2.25981184295736e-11	2.28763364705407e-10	1081
CDK18	0.201508796506163	2.28792711676524e-11	2.31493178137799e-10	1081
SAMD3	0.201331520267861	2.38368364540831e-11	2.40216643505504e-10	1081
SCARB1	0.201320825427938	2.38958416775372e-11	2.4057058000699e-10	1081
NFKB2	0.201308600882573	2.3963461433672e-11	2.41130834456205e-10	1081
FAM49B	0.201117747588724	2.50437392611842e-11	2.51123010162546e-10	1081
ZNF511	0.201110676143639	2.50846667785797e-11	2.51283248261804e-10	1081
HIGD2A	0.201091110305045	2.51982493296236e-11	2.52295592815739e-10	1081
KCNN1	0.20108791564097	2.52168425040889e-11	2.52356329977582e-10	1081
SLC2A5	0.200946663386239	2.60524833299081e-11	2.6046018693846e-10	1081
CIAPIN1	0.200885314674657	2.64237945497569e-11	2.6404133988336e-10	1081
FKBPL	0.200814969292121	2.68559265667322e-11	2.68226395977601e-10	1081
SEPT9	0.200796174851945	2.6972545405237e-11	2.69257644791129e-10	1081

ATP5J	0.200785873955734	2.70366719492588e-11	2.69764119077671e-10	1081
DAD1	0.200707525679804	2.75293178496457e-11	2.74407772430041e-10	1081
SRP19	0.200607102272885	2.81736165140586e-11	2.80643696418059e-10	1081
ASNA1	0.200574535779444	2.83857048074994e-11	2.82106572938863e-10	1081
NOP56	0.200569111040752	2.84211845495949e-11	2.8231990273747e-10	1081
LPXN	0.200550715559934	2.85418206217378e-11	2.83238904642812e-10	1081
PIK3R5	0.200442348102759	2.92627106121826e-11	2.90106941575993e-10	1081
C20orf29	0.200403096790229	2.95281867120676e-11	2.92594845703198e-10	1081
HIST1H2I	0.200390528341741	2.96136898529861e-11	2.93297827968734e-10	1081
HBXIP	0.200322311370273	3.00820079476202e-11	2.97497324548262e-10	1081
ATP6AP1	0.200284040877725	3.03479011928232e-11	2.99979621947705e-10	1081
CLEC4A	0.200185155429939	3.10456118383607e-11	3.06125232738021e-10	1081
LOC1001	0.200161992135395	3.12112987261919e-11	3.07608421154176e-10	1081
EDF1	0.200153955004462	3.12689899775352e-11	3.07979667442318e-10	1081
SEC13	0.200117820012157	3.15296604700873e-11	3.09987803889657e-10	1081
TXNDC17	0.20004234819381	3.20809675366715e-11	3.15254190744511e-10	1081
OCIAD2	0.19993947767693	3.28476136633328e-11	3.22316209083215e-10	1081
MED27	0.199876024732566	3.3329396615145e-11	3.26884466802384e-10	1081
C2orf47	0.199821371722781	3.37498941166589e-11	3.30525822547445e-10	1081
LIME1	0.199767056436402	3.41729282780279e-11	3.34506141963495e-10	1081
VIL1	0.199704244631127	3.46686009449101e-11	3.39028624288939e-10	1081
CAMK1	0.199669588812168	3.49450842172112e-11	3.4107011703281e-10	1081
CHCHD8	0.199619857070408	3.53456110488088e-11	3.44478633080916e-10	1081
MPV17L2	0.199590084892246	3.55875316772529e-11	3.46668677774787e-10	1081
C4orf33	0.199573121020938	3.57260983566871e-11	3.47850290669629e-10	1081
RPA3	0.199561245104256	3.58234185041513e-11	3.48629355442574e-10	1081
HTRA4	0.199552620712172	3.58942555604456e-11	3.49150061933934e-10	1081
C5orf55	0.199510109325032	3.62454309962558e-11	3.52395853001724e-10	1081
PIGU	0.199499644574262	3.63323912166898e-11	3.52900685178503e-10	1081
CLTB	0.199483498616549	3.64669613559388e-11	3.54037077838741e-10	1081
VAV1	0.199391256253914	3.72451559736052e-11	3.61070099657496e-10	1081
TMEM22	0.199361129249709	3.75028177618279e-11	3.63393104286688e-10	1081
SKA2	0.19929638578387	3.8062445229444e-11	3.68638441897668e-10	1081

TTC9C	0.199264600168051	3.83401710909025e-11	3.70971540166297e-10	1081
SIGMAR1	0.1992144613833	3.87822872450136e-11	3.74889240187523e-10	1081
ATP5I	0.199138571574625	3.94609640139131e-11	3.81083950172713e-10	1081
TMEM52	0.198972523177463	4.0986648736586e-11	3.95628193003606e-10	1081
UROS	0.198941766298917	4.1275507928267e-11	3.98034996273307e-10	1081
XIRP1	0.198886592744394	4.17986737414863e-11	4.02694539704563e-10	1081
FKBP11	0.198843696940597	4.22098912544002e-11	4.06367658009688e-10	1081
UBQLNL	0.198829517145678	4.2346692126622e-11	4.07389738725311e-10	1081
GSS	0.198597677169419	4.46458107288763e-11	4.29098214281113e-10	1081
SIGLEC1(	0.198587286561788	4.47516588142221e-11	4.29910427664522e-10	1081
FTHL3	0.198553143627429	4.51011988710199e-11	4.3285547939814e-10	1081
NCKAP1L	0.198549104889887	4.51427216629553e-11	4.33047679952492e-10	1081
DTYMK	0.198533688445818	4.53015638166177e-11	4.34364589760002e-10	1081
C21orf70	0.198522335064078	4.54188915411915e-11	4.35282383490629e-10	1081
MRPL40	0.198509854880318	4.55482063767565e-11	4.36106757347794e-10	1081
SIT1	0.198500317404828	4.56472724290083e-11	4.36847649920367e-10	1081
GDI1	0.198317405222891	4.75884332154603e-11	4.53915240116216e-10	1081
DNAJC7	0.198267152895581	4.81357099432702e-11	4.58918067585035e-10	1081
GRK6	0.198095866863638	5.00477061104426e-11	4.7602032086632e-10	1081
FBXL6	0.19771419073745	5.45789502013966e-11	5.16436332459904e-10	1081
CREB3L3	0.197677940536432	5.50295879968008e-11	5.19967659566394e-10	1081
WDR8	0.197644364406228	5.54502245995781e-11	5.23451159586927e-10	1081
AP2M1	0.19762770404144	5.56601073291113e-11	5.2517978560485e-10	1081
MRPL23	0.197625694025328	5.56854813627183e-11	5.2517978560485e-10	1081
CD83	0.197591457056792	5.61194212487887e-11	5.28530033219658e-10	1081
AIMP2	0.197525909871383	5.69594404272468e-11	5.35940180946701e-10	1081
ATP5J2	0.197369206268198	5.90177656680492e-11	5.5453026557036e-10	1081
CECR1	0.19733352873976	5.94964520854551e-11	5.58767378676687e-10	1081
TCN2	0.197327479348048	5.95779920999877e-11	5.59272437490332e-10	1081
DDOST	0.197127608010677	6.23343101228584e-11	5.84329770788731e-10	1081
PRR19	0.196969373480088	6.46044098785874e-11	6.04765723514936e-10	1081
ITGAX	0.196912009382321	6.54471601972201e-11	6.11801875718329e-10	1081
CCNE1	0.196872180004147	6.60386092912306e-11	6.17044426795844e-10	1081

BNIP1	0.196755658894985	6.77990317778015e-11	6.32906160872943e-10	1081
CD8A	0.196674282854558	6.90555715795567e-11	6.44039115495449e-10	1081
UBD	0.196639453566874	6.9600299985067e-11	6.48219160055097e-10	1081
CHRNA1	0.196636914591365	6.96401731386432e-11	6.48290798464865e-10	1081
RABAC1	0.196526010944346	7.14037903527851e-11	6.62565341619925e-10	1081
HLA-DMI	0.196377603152159	7.38321885101355e-11	6.83838821855944e-10	1081
CCNB1	0.196365765272096	7.40293284227431e-11	6.84767104412519e-10	1081
TCEB1	0.196268242082643	7.56730928386764e-11	6.99281860199604e-10	1081
PCYT2	0.196144453943104	7.78109595658348e-11	7.18378451170368e-10	1081
OIP5	0.196096309588536	7.86582633730174e-11	7.25203988855576e-10	1081
AATF	0.196040700329925	7.9648160328826e-11	7.32653967956255e-10	1081
METTL11	0.196036983664858	7.97147524045319e-11	7.32931851752303e-10	1081
TCEB2	0.195990539269747	8.05515024594865e-11	7.39949848174353e-10	1081
PGLS	0.1958761922684	8.264831782561e-11	7.57930845461946e-10	1081
C17orf37	0.195639152100906	8.71661491664819e-11	7.97801942280226e-10	1081
CPNE9	0.195624566416864	8.74518832676698e-11	8.00053673218532e-10	1081
SULT1C2	0.195618103988116	8.75787746654483e-11	8.00850846861306e-10	1081
RNF149	0.195547847572042	8.89699301316045e-11	8.12465658432082e-10	1081
FDX1L	0.195506887901076	8.97909090025562e-11	8.19591237814451e-10	1081
SLA	0.195456686012948	9.08072279773157e-11	8.27742808870147e-10	1081
VPS29	0.195367312729175	9.26444239975028e-11	8.43344745336508e-10	1081
LOC3410	0.195341155735456	9.31889491905878e-11	8.47153150471295e-10	1081
TRIM47	0.195325049302304	9.35257991902754e-11	8.49831855971176e-10	1081
POLDIP2	0.195273208459467	9.46180894945242e-11	8.58595231021257e-10	1081
DPPA4	0.195209857275595	9.59698225506238e-11	8.70469191932606e-10	1081
POP7	0.195205814702774	9.60567165084096e-11	8.70865235851445e-10	1081
BUD31	0.195194180494155	9.63072193745745e-11	8.72743560189295e-10	1081
MRPL55	0.194954580721857	1.016103177503e-10	9.18734224003497e-10	1081
B3GAT3	0.19494807733097	1.01758161400917e-10	9.19658215083657e-10	1081
GPR171	0.194918715449811	1.02428272947318e-10	9.24702367192556e-10	1081
ANKRD1	0.194917592241757	1.02453992731387e-10	9.24702367192556e-10	1081
DGUOK	0.194848254013633	1.04054001630149e-10	9.3830253484304e-10	1081
SLC7A5	0.194753150289791	1.06288291815871e-10	9.5759286164165e-10	1081

RNASEK	0.194711505778175	1.0728133496787e-10	9.65675823470844e-10	1081
FYB	0.194661210884958	1.08492731844099e-10	9.76143851272612e-10	1081
MVD	0.194604946239293	1.09863742458986e-10	9.88037987426903e-10	1081
GZMM	0.194596933726677	1.10060355390396e-10	9.89098609956511e-10	1081
DIABLO	0.194596139008672	1.10079875081782e-10	9.89098609956511e-10	1081
CCS	0.194553028576355	1.11143823218979e-10	9.97768412988565e-10	1081
NAPSB	0.194449772523216	1.13733120966295e-10	1.01965007648688e-09	1081
NHP2	0.194430013587184	1.14235274027887e-10	1.02324126068999e-09	1081
ARHGAP1	0.194403823038894	1.14904216588179e-10	1.02877575251949e-09	1081
AURKAIP	0.194359793393562	1.1603742118133e-10	1.03846017312212e-09	1081
NDUFA3	0.194251070594148	1.18882584634533e-10	1.06297810362302e-09	1081
KCNG3	0.194204023882877	1.20134752136423e-10	1.07322154403027e-09	1081
CD101	0.194187279046668	1.20583521724465e-10	1.07675312284546e-09	1081
PSMD12	0.194168245501426	1.21095617013878e-10	1.08084678987354e-09	1081
WBSCR22	0.19409731026654	1.2302288539742e-10	1.09707659421471e-09	1081
HMHA1	0.193960028099578	1.26838166416452e-10	1.12760585280645e-09	1081
ZFAND2A	0.193913968304022	1.2814392615238e-10	1.13871168607838e-09	1081
C7orf30	0.193752684686284	1.32820418553447e-10	1.17715236768992e-09	1081
RNF25	0.193674239347943	1.35154781285627e-10	1.19678816219735e-09	1081
CEBPB	0.193620416154799	1.3677955408145e-10	1.21011160165604e-09	1081
HNRNPA1	0.193601317550437	1.37360657596517e-10	1.2147192481483e-09	1081
CYP2S1	0.193588019205279	1.37766701773935e-10	1.21777543099426e-09	1081
TIMM16	0.193449531826566	1.4206542859361e-10	1.25302454422866e-09	1081
ERI3	0.193314698173602	1.46376382537788e-10	1.28991785924048e-09	1081
NRM	0.193290891237349	1.4715069417727e-10	1.29617434814215e-09	1081
POR	0.193217882109001	1.4955029738422e-10	1.31616021878773e-09	1081
CD180	0.193173246939816	1.51036093172309e-10	1.32865593753545e-09	1081
H2AFY	0.193153831463654	1.51686873678865e-10	1.33379837200382e-09	1081
LAT	0.193140767486958	1.52126299694327e-10	1.33707866812487e-09	1081
H3F3C	0.193068134884302	1.54592149903235e-10	1.35697553803951e-09	1081
DUS1L	0.193064445213109	1.5471844844232e-10	1.3574926584802e-09	1081
GNA15	0.19305908469699	1.54902119924155e-10	1.35792132544478e-09	1081
CD5	0.193028500036761	1.55954142925761e-10	1.36595487358237e-09	1081

FASTK	0.192960832796573	1.58306535767397e-10	1.38535411078811e-09	1081
TRIM26	0.192902770172702	1.60352605375145e-10	1.40082534053873e-09	1081
ARFGAP1	0.192869272180421	1.61544755946064e-10	1.4106281354718e-09	1081
OSTF1	0.192650354725154	1.69551451500042e-10	1.47479015132485e-09	1081
GZMH	0.192639618735335	1.69953913375335e-10	1.47755678699995e-09	1081
CCM2	0.192637959032704	1.7001621406135e-10	1.47755678699995e-09	1081
NDUFB9	0.192563545681478	1.7283250089483e-10	1.49750999162424e-09	1081
RCE1	0.192523264349716	1.74375945341886e-10	1.51023362807923e-09	1081
ZAP70	0.192437721415246	1.77698398767305e-10	1.53768653057017e-09	1081
H3F3B	0.192381246578532	1.79925622948564e-10	1.55562303617975e-09	1081
C21orf119	0.192210580347921	1.86823067595297e-10	1.61387251145251e-09	1081
ORAI1	0.19210899426931	1.91050419805236e-10	1.64897630975856e-09	1081
U2AF1L4	0.192063338358724	1.92980582322395e-10	1.66492241151376e-09	1081
COMMD5	0.192052036858376	1.93461297545131e-10	1.66835523931792e-09	1081
CYBB	0.192036192937487	1.94137194440613e-10	1.67346760034495e-09	1081
C20orf43	0.191843772800191	2.0253193445063e-10	1.74135971809985e-09	1081
CD8B	0.191838977652166	2.02745591630935e-10	1.74245304752781e-09	1081
HNRNPA:	0.191784356120876	2.05194931936213e-10	1.76199995901748e-09	1081
C6orf136	0.191781480317035	2.0532468560764e-10	1.76236292780823e-09	1081
MT3	0.191733287295272	2.07511058586877e-10	1.77961271827698e-09	1081
LHX2	0.19169126917817	2.09435814797527e-10	1.79382843924157e-09	1081
GPS2	0.191621265643341	2.12681265306416e-10	1.81930534590138e-09	1081
TMSB10	0.191523608861626	2.17290839428077e-10	1.85322775625682e-09	1081
PFDN4	0.191489672560845	2.18915397764301e-10	1.86629313921365e-09	1081
AIP	0.191472672954583	2.19733630656555e-10	1.87247630692737e-09	1081
ZRSR2	0.191308376392987	2.27796945317129e-10	1.93954753314183e-09	1081
ADAP1	0.19118165655742	2.34212534576834e-10	1.98828972147085e-09	1081
AVPI1	0.191173655323805	2.34623484705408e-10	1.99093938474745e-09	1081
HIST1H3(	0.191040968939643	2.41541796655877e-10	2.04447877883724e-09	1081
HLA-DRB	0.191028466948203	2.42203818879891e-10	2.04922130673474e-09	1081
HARS	0.191006572379797	2.4336747558878e-10	2.05733856304489e-09	1081
SPOCK2	0.190930263321325	2.47465942282111e-10	2.08760527942761e-09	1081
FFAR2	0.190922020165692	2.47912680254199e-10	2.08962382582462e-09	1081

SERPINB9	0.190871046545271	2.50692718661741e-10	2.10864501772057e-09	1081
GPR82	0.190803913266572	2.54400516377792e-10	2.1380468929623e-09	1081
CRELD2	0.190764886215045	2.56580523303337e-10	2.15546899163708e-09	1081
ENTPD6	0.190644055808458	2.63446133678264e-10	2.21037999289822e-09	1081
GPR37L1	0.190458824682013	2.74320197744719e-10	2.29492540845821e-09	1081
ZDHHC18	0.190403692594539	2.77640431710162e-10	2.3207744800005e-09	1081
COX6C	0.190392950830461	2.7829188214106e-10	2.32525506666597e-09	1081
YDJC	0.190351550513455	2.8081663949624e-10	2.34440580300528e-09	1081
NDUFB8	0.190344770300444	2.81232247200757e-10	2.34690290797815e-09	1081
CCDC22	0.190328090717969	2.82257212541787e-10	2.35436157609996e-09	1081
RBM38	0.190326425019334	2.82359770059941e-10	2.35436157609996e-09	1081
C17orf87	0.19025152464989	2.87009152517748e-10	2.39114945304799e-09	1081
PPP2R5B	0.190236671135691	2.87940003243044e-10	2.39791292489918e-09	1081
C14orf73	0.190211698922011	2.89511619627982e-10	2.40801468926742e-09	1081
RAB33A	0.190134866487559	2.94399681222481e-10	2.44463379151974e-09	1081
ELOVL1	0.190118761398002	2.95434438992225e-10	2.45221539905166e-09	1081
SLC35C2	0.190100576485797	2.96607089537858e-10	2.45992170388643e-09	1081
C15orf27	0.190066323931349	2.98828197516463e-10	2.47732264978154e-09	1081
C16orf59	0.189981068565021	3.04427244836901e-10	2.52117641428796e-09	1081
RFC4	0.189980068401655	3.04493532686156e-10	2.52117641428796e-09	1081
HIST2H4A	0.18991109736722	3.09098837999633e-10	2.55510713643931e-09	1081
GRIP2	0.189738512456326	3.20922243689026e-10	2.64416302622308e-09	1081
E2F4	0.189729841312612	3.21527776833592e-10	2.64806911868877e-09	1081
BLOC1S2	0.189561034484934	3.33540493007092e-10	2.74028272089228e-09	1081
TMEM191	0.189552413208398	3.34165609735093e-10	2.74429931027861e-09	1081
APOB48R	0.18954248549175	3.34886869758007e-10	2.74910187093523e-09	1081
VAC14	0.189444523238215	3.42085815211708e-10	2.80362845705445e-09	1081
DDX49	0.189425292732868	3.43516617516298e-10	2.81420994707842e-09	1081
JAGN1	0.189404897973579	3.45040418675591e-10	2.82554440415438e-09	1081
GLRX2	0.189402679622604	3.45206560360684e-10	2.82575626105891e-09	1081
FAM96A	0.189340744712165	3.4987674495796e-10	2.86165936954044e-09	1081
B3GNT7	0.189312043772409	3.52061763458142e-10	2.87602766620611e-09	1081
NKIRAS2	0.189187385680377	3.61707431096871e-10	2.95362634756646e-09	1081

PLAUR	0.189173720914817	3.62780308671765e-10	2.96118691985118e-09	1081
GM2A	0.18915800610021	3.64017979998121e-10	2.97008594858734e-09	1081
C19orf43	0.189141074311199	3.65356105215387e-10	2.97738622150646e-09	1081
SLAMF6	0.189123640716803	3.66738898854557e-10	2.98744646883342e-09	1081
DHDH	0.189119008144114	3.67107201350616e-10	2.98923790267104e-09	1081
BANF1	0.189102339438935	3.6843539544791e-10	2.99884082476693e-09	1081
NUDT1	0.189061494313248	3.7170984868149e-10	3.02305002086743e-09	1081
PRDX2	0.189021268646008	3.74962363070274e-10	3.04704187335647e-09	1081
RRBP1	0.188826568005829	3.91101465304187e-10	3.174350934147e-09	1081
KYNU	0.188769828738402	3.95930774524741e-10	3.20727878927548e-09	1081
PLA2G2D	0.188696857745978	4.02227155245556e-10	3.25416306924567e-09	1081
TRMT112	0.188687641660315	4.03029284406481e-10	3.25934361074611e-09	1081
FAM54A	0.188679321907016	4.03754739655193e-10	3.26390017269416e-09	1081
SART1	0.188666791552943	4.04849748376167e-10	3.27143930246204e-09	1081
PSEN2	0.18864766369331	4.06526887823559e-10	3.28235837883992e-09	1081
RXFP1	0.188537406732051	4.16327115656352e-10	3.36014012215433e-09	1081
TFG	0.188533318412654	4.16694900565666e-10	3.36176162270538e-09	1081
KARS	0.188528999060881	4.17083813186279e-10	3.36311240866188e-09	1081
SERF2	0.188527751345055	4.17196222846664e-10	3.36311240866188e-09	1081
PDIA4	0.188298227015975	4.38384914087829e-10	3.52686265746778e-09	1081
SPATC1	0.188278081652914	4.40293940756951e-10	3.54080696069812e-09	1081
EXOSC3	0.188058751704318	4.61609706268547e-10	3.69849779513813e-09	1081
ARSA	0.188057456776439	4.61738493659588e-10	3.69849779513813e-09	1081
OR52K2	0.187992052320211	4.68289064767267e-10	3.74947663344061e-09	1081
PYCRL	0.187960193220863	4.71512625668462e-10	3.76901834828318e-09	1081
ATG3	0.187959940624085	4.71538270079291e-10	3.76901834828318e-09	1081
APEX2	0.187959693314016	4.71563379083927e-10	3.76901834828318e-09	1081
NR1H3	0.18789866738348	4.77799245778915e-10	3.81349675365144e-09	1081
APOC4	0.187881104345952	4.79608749472555e-10	3.82490825737317e-09	1081
PLCB2	0.187850901247552	4.82736177891881e-10	3.84832619850888e-09	1081
KIF18B	0.187816420279817	4.86330865750965e-10	3.87238549033723e-09	1081
ARHGAP[illegible]	0.187751308207788	4.93190167552055e-10	3.92390044444555e-09	1081
ATP5G1	0.187625572175059	5.06703811205289e-10	4.02347192618468e-09	1081

KIFC1	0.187612693110348	5.08108209401351e-10	4.03303383703318e-09	1081
TMEM54	0.18759213972721	5.10357315033311e-10	4.04769610682916e-09	1081
C19orf24	0.187586616610321	5.10963347988205e-10	4.05090777065029e-09	1081
UQCRHL	0.18753730590353	5.16405219085326e-10	4.08808069356781e-09	1081
SEPT1	0.187531124791039	5.17091330282689e-10	4.09143945347399e-09	1081
TNFRSF6I	0.187413770947718	5.30287572537818e-10	4.18762961535647e-09	1081
MRPL4	0.187354906919754	5.37029754234219e-10	4.23754970585521e-09	1081
IDO2	0.187335535486301	5.39266737554148e-10	4.25183224487909e-09	1081
AOAH	0.187278793149574	5.45871636333697e-10	4.29889918449661e-09	1081
LLPH	0.187264201629501	5.47582814552794e-10	4.30900617154923e-09	1081
STOML2	0.187254788582788	5.48689474825389e-10	4.31602868815207e-09	1081
SH2D1A	0.187224735143335	5.5223736375537e-10	4.34054689537727e-09	1081
CDK2AP2	0.187222828870903	5.52463156708592e-10	4.34062803896044e-09	1081
MIF4GD	0.187205663416177	5.54500416108844e-10	4.35493601657413e-09	1081
NCRNA0(	0.187149522235124	5.61214713873571e-10	4.40423467510054e-09	1081
C20orf135	0.187146316406004	5.61600502131682e-10	4.4055459951101e-09	1081
RHBG	0.187106054095117	5.6646771125224e-10	4.43854221827163e-09	1081
ZNRD1	0.187082952544502	5.69278931959597e-10	4.45883518053114e-09	1081
NDUFC1	0.187018678361592	5.77172156821331e-10	4.51188711647874e-09	1081
MRPL37	0.186970750721835	5.8312724185087e-10	4.55490433776106e-09	1081
PRMT7	0.186901604737863	5.91824358073658e-10	4.61210897229936e-09	1081
FCHO1	0.186816133171541	6.02749692453119e-10	4.68818245346258e-09	1081
KIAA201:	0.186757897851906	6.10305874835872e-10	4.74329160824408e-09	1081
APOO	0.18673183637378	6.13717238113366e-10	4.76612712482411e-09	1081
C17orf90	0.186716915789997	6.15678649942211e-10	4.77767581012552e-09	1081
CXCL13	0.186653340587943	6.24104716522617e-10	4.83561135167235e-09	1081
TOMM40	0.186522595854516	6.41787845911912e-10	4.96307721915373e-09	1081
KIR3DL1	0.186332172072593	6.68418650781004e-10	5.15714045192774e-09	1081
CRYBA2	0.18626968218922	6.77390210097675e-10	5.21884716016549e-09	1081
SSU72	0.186269249525885	6.77452733871072e-10	5.21884716016549e-09	1081
C11orf83	0.186217921670016	6.84910107358112e-10	5.27024985207378e-09	1081
SLC39A3	0.186145179935898	6.95615821368235e-10	5.34854226010042e-09	1081
ECEL1	0.186035155765521	7.12118969680527e-10	5.45668948049229e-09	1081

EXOG	0.185872487981151	7.37219313568788e-10	5.64044172876689e-09	1081
CLEC4D	0.18583766153809	7.42704128289854e-10	5.68024854381135e-09	1081
C9orf25	0.18570443294979	7.64055553099799e-10	5.83026481711949e-09	1081
MTX2	0.185665399702423	7.70423530386413e-10	5.87440651765113e-09	1081
C11orf59	0.185590181017675	7.8284101041031e-10	5.96006506225083e-09	1081
SLC35A2	0.185487232632967	8.0015270593795e-10	6.08267028721509e-09	1081
IKBKG	0.185433656301374	8.09308997461295e-10	6.14763565379253e-09	1081
PHLDA2	0.18542211341104	8.11295018790321e-10	6.16039885168903e-09	1081
ACRBP	0.185393884785729	8.16171938349677e-10	6.19509559082677e-09	1081
MESDC1	0.18529121786429	8.341511407706e-10	6.32441653399464e-09	1081
FADD	0.185232491528675	8.44608218033873e-10	6.4012914041732e-09	1081
RPUSD3	0.185198997316085	8.50629410570596e-10	6.44450149527818e-09	1081
C11orf17	0.185134596560095	8.62324307640328e-10	6.52819360293665e-09	1081
TTLL6	0.185131021887545	8.62978019639499e-10	6.53068828160695e-09	1081
SRXN1	0.185072041277589	8.73833932463035e-10	6.61035845642803e-09	1081
UNC119	0.185056270295905	8.76759187777789e-10	6.62750988284561e-09	1081
NEURL3	0.185045555560353	8.78752031890761e-10	6.64008240151515e-09	1081
SH2D1B	0.184988916426815	8.89359915638992e-10	6.70877214162016e-09	1081
CHST12	0.184872018929212	9.11649308021692e-10	6.87319435258121e-09	1081
ASGR1	0.184747425102794	9.36005045523054e-10	7.04627116668981e-09	1081
IGF2BP3	0.184742056006618	9.37068720738737e-10	7.05164339905934e-09	1081
HIST1H1T	0.184702367365012	9.44968058089194e-10	7.10577884666175e-09	1081
EXOSC1	0.18464070703503	9.57369274077082e-10	7.19097838414721e-09	1081
TMEM206	0.184587668593948	9.68163119113313e-10	7.26392775960436e-09	1081
RTBDN	0.184425843030628	1.00183373812371e-09	7.50261626352438e-09	1081
AKR1A1	0.18432882763017	1.02256331646963e-09	7.64645063484807e-09	1081
KCP	0.184248076606864	1.04013561770261e-09	7.77208160928006e-09	1081
CHMP4A	0.184179785724678	1.05522564089009e-09	7.88153843927517e-09	1081
GALM	0.184178253356056	1.05556667704961e-09	7.88153843927517e-09	1081
LOC64300	0.184165736530807	1.0583563793821e-09	7.89944026033807e-09	1081
SUV39H1	0.18414457158091	1.06308988324681e-09	7.92921846873192e-09	1081
METTL7E	0.184144320965634	1.06314605621458e-09	7.92921846873192e-09	1081
DHCR7	0.184132368430562	1.06582845863897e-09	7.94343851249802e-09	1081

RHCE	0.184128609158881	1.06667348035142e-09	7.94679632458556e-09	1081
CDKN2A	0.184095099567566	1.07423475853404e-09	7.99721330771184e-09	1081
1-Mar	0.183995684928301	1.0969755724152e-09	8.15746508169223e-09	1081
RNF166	0.183967933889967	1.10340677969211e-09	8.20075446115038e-09	1081
HIST1H2I	0.183967054875838	1.10361108682942e-09	8.20075446115038e-09	1081
CD300E	0.183953970534577	1.10665660688074e-09	8.21732301718113e-09	1081
TARP	0.183943668009106	1.10906038631686e-09	8.2321376132473e-09	1081
AMZ2	0.183927944328831	1.11273882934667e-09	8.25639915918555e-09	1081
CSAG2	0.183906032957764	1.11788465522864e-09	8.29152664933022e-09	1081
TBCC	0.183867948260907	1.1268839282744e-09	8.34905359878182e-09	1081
CLPP	0.183841073256885	1.13327680055227e-09	8.39333130409024e-09	1081
LOC72875	0.183697201576619	1.16810525502253e-09	8.6354056375886e-09	1081
NADK	0.183604767040317	1.19102930839626e-09	8.80164542099876e-09	1081
NDUFA4	0.183571438817409	1.19940176398659e-09	8.8602671564026e-09	1081
CDC123	0.183427584090652	1.23620194462188e-09	9.11675983964041e-09	1081
PLK1	0.183426876681177	1.23638559850571e-09	9.11675983964041e-09	1081
LRRC45	0.183423479708883	1.23726787245245e-09	9.11992729255567e-09	1081
U2AF1	0.183280881340814	1.27486203208048e-09	9.3833012920209e-09	1081
EIF5A	0.18319935350013	1.29685294965027e-09	9.53819009518246e-09	1081
GPRIN1	0.183130790220263	1.31563234357109e-09	9.66219962130501e-09	1081
LRP8	0.183128144552042	1.31636226787551e-09	9.66403713059483e-09	1081
C7orf50	0.182987592629696	1.35571166069824e-09	9.92759411296475e-09	1081
BDKRB1	0.182955341109426	1.36490112633144e-09	9.98762556845144e-09	1081
MRPL53	0.182936481656212	1.37030283734677e-09	1.00235114954069e-08	1081
GNAI2	0.182874586047031	1.38817754573032e-09	1.01505759196869e-08	1081
TMEM55I	0.182863991663622	1.39125973915222e-09	1.01694221499352e-08	1081
VSTM1	0.182802642461811	1.40923928538638e-09	1.02971075096513e-08	1081
COTL1	0.182794038183044	1.41177894694866e-09	1.03081866206164e-08	1081
HMGA1	0.1827786033259	1.41634590776266e-09	1.03340414023465e-08	1081
DSN1	0.18274594439623	1.42605668607428e-09	1.03973622660031e-08	1081
CD6	0.182681410665678	1.44543599512994e-09	1.05234218004671e-08	1081
HSP90B1	0.182667388685695	1.44968053143408e-09	1.05505109486054e-08	1081
FCRL3	0.182574157753915	1.47821216054682e-09	1.07503913264317e-08	1081

STRADA	0.182556461425074	1.48368917498586e-09	1.07863292782715e-08	1081
HAGH	0.182539505704182	1.48895549672972e-09	1.08207101304546e-08	1081
RAE1	0.182523735565478	1.49386991189109e-09	1.08485974675724e-08	1081
AP1B1	0.182495113942602	1.50282956489577e-09	1.0909730300838e-08	1081
HPRT1	0.182483723508464	1.50640973195026e-09	1.09317809978883e-08	1081
PIPOX	0.18242674844022	1.52444273563779e-09	1.10507012988209e-08	1081
POLR2C	0.182307593460871	1.56283782562723e-09	1.13046204658027e-08	1081
SLC26A6	0.182232479809063	1.58752345873782e-09	1.14708249914897e-08	1081
SHD	0.182216880759461	1.59269736770623e-09	1.15040833533316e-08	1081
C18orf56	0.182210422641835	1.59484420287532e-09	1.15154610992557e-08	1081
GLRX3	0.182162553614347	1.61084506514337e-09	1.16226625491809e-08	1081
CDC6	0.182118547420403	1.62569247699157e-09	1.17130096384103e-08	1081
SNRPA	0.18205509884394	1.64733430193897e-09	1.18519819687716e-08	1081
TNFRSF1‹	0.181947624436574	1.68463430109405e-09	1.20944255151602e-08	1081
NDUFB10	0.181911952639427	1.69719522689298e-09	1.21802628591945e-08	1081
KHK	0.181862230090242	1.71485593562455e-09	1.229824593206e-08	1081
TPX2	0.18184466061105	1.72113900925068e-09	1.23389129328665e-08	1081
PAFAH1B	0.181769712352804	1.74819400044863e-09	1.25106103513455e-08	1081
KCNH2	0.181738354661647	1.75963595911039e-09	1.25880207373149e-08	1081
UBE2D2	0.181713986059399	1.76857798822897e-09	1.2643010494277e-08	1081
UQCRH	0.181691046730723	1.77703594025411e-09	1.2698967370138e-08	1081
IDH3G	0.181576384811968	1.81990643404284e-09	1.29915007490407e-08	1081
C1orf127	0.181567057076613	1.82343787265732e-09	1.30104396973529e-08	1081
ACD	0.181551106207627	1.82949225567239e-09	1.3046060704609e-08	1081
NXT1	0.181500343873067	1.84889029185904e-09	1.31750601094801e-08	1081
NDUFA9	0.181418035610092	1.88076918394471e-09	1.33832904311431e-08	1081
XRCC6BP	0.181370315744334	1.89949585651234e-09	1.35070045991674e-08	1081
HARBI1	0.181311860473912	1.92268275162355e-09	1.36594494826982e-08	1081
GIMAP5	0.181311144586851	1.92296841563327e-09	1.36594494826982e-08	1081
LOC15221	0.181289729040522	1.93153308898802e-09	1.37057886853342e-08	1081
ILKAP	0.181252903473559	1.94634751674939e-09	1.38023770905659e-08	1081
METRNL	0.181249712073568	1.94763656658639e-09	1.38054674996069e-08	1081
HJURP	0.181231333838497	1.95507596544348e-09	1.38533258261902e-08	1081

WDR53	0.181185827549865	1.97361581604536e-09	1.39748648907676e-08	1081
EMR1	0.181166344557154	1.98160563015221e-09	1.40265092829994e-08	1081
FDXR	0.181123757098866	1.99918012147993e-09	1.41310468586713e-08	1081
TBCB	0.181089542180307	2.01340929746594e-09	1.42216445643238e-08	1081
RPL39L	0.18107350127365	2.02011420981296e-09	1.42640030692892e-08	1081
MGC2950	0.1810157286976	2.04444295353655e-09	1.44307299576012e-08	1081
MTHFS	0.180998324988262	2.0518275727324e-09	1.44777815946389e-08	1081
MFSD5	0.180986776932962	2.05674187713757e-09	1.45073757405239e-08	1081
DAPP1	0.180960507619735	2.06796353908045e-09	1.45763210268634e-08	1081
LOC28474	0.180930548890003	2.08083394279744e-09	1.46465408028143e-08	1081
RNASEH2	0.180858216056341	2.1122304902899e-09	1.48519662223002e-08	1081
MASTL	0.180854344142092	2.11392405183006e-09	1.4858688075407e-08	1081
AK1	0.180809671439053	2.13355952795049e-09	1.49810235937827e-08	1081
MND1	0.18078889418525	2.14275232048306e-09	1.50403294411607e-08	1081
AHSA1	0.180751644116713	2.15932998478915e-09	1.51461359831398e-08	1081
C1orf151	0.180636269526795	2.21147151686469e-09	1.54956847677354e-08	1081
C15orf53	0.180634354319628	2.2123473125624e-09	1.54964313670269e-08	1081
CYHR1	0.180609508373334	2.22373961828054e-09	1.55654046595766e-08	1081
RAD51	0.180512142393587	2.26893615762921e-09	1.58652269682195e-08	1081
ZCRB1	0.180492248018845	2.27828029831015e-09	1.59250369914843e-08	1081
TM9SF1	0.180453936393028	2.29638035911163e-09	1.60312580059967e-08	1081
MYH6	0.180398717293774	2.32271454297363e-09	1.61962909201121e-08	1081
RTKN	0.180360462469762	2.34113040270779e-09	1.63190560424043e-08	1081
GIYD2	0.180282246441795	2.37922667840996e-09	1.6557678577101e-08	1081
USF1	0.180236929687801	2.40157404339732e-09	1.6699934105709e-08	1081
SDF2	0.180180402457597	2.4297360106196e-09	1.68841089803145e-08	1081
RGS10	0.180178683268354	2.43059752728307e-09	1.68842714438336e-08	1081
DPEP2	0.18013847024852	2.45083395945024e-09	1.69955422076162e-08	1081
ME1	0.180021058675814	2.51086130901334e-09	1.73759192957999e-08	1081
PDZD7	0.180004656505798	2.51936002469556e-09	1.74287457752376e-08	1081
CDK16	0.179997713782709	2.52296578919626e-09	1.74476985318773e-08	1081
CCDC124	0.179955387581812	2.54505709423236e-09	1.75944321082055e-08	1081
UBE2A	0.179921575852351	2.56283950406849e-09	1.77112870701405e-08	1081

GYG1	0.179885474204088	2.58195962270404e-09	1.78373033605531e-08	1081
ABHD8	0.179856332197311	2.59749490908733e-09	1.79384761548043e-08	1081
FCGR2A	0.179833423712766	2.60977095570727e-09	1.80051345227059e-08	1081
EIF4EBP1	0.179795034615294	2.6304692635079e-09	1.81351140702829e-08	1081
SLC9A3R	0.179709961337765	2.67690887523592e-09	1.84300510224291e-08	1081
MFSD2B	0.179688986684776	2.68848042759288e-09	1.85033953583391e-08	1081
C16orf80	0.179681620870709	2.69255561932748e-09	1.85192083168977e-08	1081
FOXD2	0.179681510754042	2.6926165877485e-09	1.85192083168977e-08	1081
FOXM1	0.179614154570978	2.7301624328999e-09	1.87454404263014e-08	1081
RDBP	0.17960719515761	2.73407066142986e-09	1.87658785262366e-08	1081
PEX16	0.179567972229297	2.75619920347791e-09	1.89048801341717e-08	1081
RANBP1	0.179549104409554	2.76690587708157e-09	1.89718580305678e-08	1081
MUTYH	0.179512663764212	2.78769898125259e-09	1.90927642224849e-08	1081
SERTAD1	0.179511561629488	2.7883302230107e-09	1.90927642224849e-08	1081
NDUFS5	0.179490083071387	2.80065973812839e-09	1.91641611496591e-08	1081
UBE2MP1	0.179440939715538	2.82906966407262e-09	1.93322959235899e-08	1081
CD27	0.179280773394701	2.92362090903199e-09	1.99378277631854e-08	1081
FAM125A	0.179205014754219	2.96940724859621e-09	2.02226872964742e-08	1081
NDUFB1	0.179192115417813	2.9772723903009e-09	2.0269399223593e-08	1081
LMF2	0.179144390716329	3.00654817938341e-09	2.04489257328131e-08	1081
ARHGAP4	0.179144164815473	3.00668741725452e-09	2.04489257328131e-08	1081
IDH3B	0.179054455604028	3.06247974536328e-09	2.08213481168894e-08	1081
CENPN	0.178920947079959	3.14738009181038e-09	2.1376929180553e-08	1081
TMEM156	0.178898240450345	3.16204552919289e-09	2.14548356974034e-08	1081
BSG	0.178843074167935	3.19795288818775e-09	2.16838643327305e-08	1081
IAH1	0.178831475947781	3.20555237020041e-09	2.17280795752649e-08	1081
TOMM5	0.178822299176556	3.2115776898499e-09	2.17615985745127e-08	1081
FZD2	0.178796481416126	3.22858834193744e-09	2.18621553439764e-08	1081
CXorf65	0.178740899247376	3.26550781787048e-09	2.20824622326287e-08	1081
CHMP2A	0.17866154900034	3.31892695789386e-09	2.24211212497558e-08	1081
DPM1	0.178655619651254	3.32295257464569e-09	2.24407910211993e-08	1081
LILRP2	0.178558044454217	3.3898852201951e-09	2.28558912158871e-08	1081
MSR1	0.178554116344138	3.39260700942361e-09	2.2865195117042e-08	1081

BIN1	0.178546441528852	3.39793102443623e-09	2.28934182231665e-08	1081
TMEM176	0.178511135903229	3.42252732518916e-09	2.30437209110748e-08	1081
HLA-DRB	0.178485034526271	3.44082251611506e-09	2.31591612386027e-08	1081
ACSL6	0.178456839431062	3.46069207278528e-09	2.32773428401534e-08	1081
RDH12	0.178412355749145	3.49226749209474e-09	2.34583956746411e-08	1081
TRAPPC5	0.178401266431294	3.50018242211249e-09	2.35037249644854e-08	1081
COG4	0.178355609057422	3.53295418745471e-09	2.37079820473934e-08	1081
TNFRSF8	0.178228210235005	3.62598328191103e-09	2.42917968786491e-08	1081
SYNGR4	0.178060819043711	3.75184214959781e-09	2.51015809045659e-08	1081
KLRD1	0.178054922379935	3.75635231587841e-09	2.51234121525135e-08	1081
PCGF1	0.178049587680184	3.76043719459972e-09	2.51340435584643e-08	1081
ZWINT	0.178036764447281	3.77027385440342e-09	2.51914317734517e-08	1081
MRPL46	0.177895363627151	3.88041410043363e-09	2.59187473651311e-08	1081
GAA	0.177884702662039	3.88884378590623e-09	2.59664428462317e-08	1081
NACC1	0.177823640606207	3.93746992673798e-09	2.62737103922281e-08	1081
UBASH3A	0.177818023363349	3.94197272858026e-09	2.62950465620031e-08	1081
GBP2	0.17780189225906	3.95493132100186e-09	2.63727545387562e-08	1081
NEU1	0.177795533871891	3.96005056998806e-09	2.63894206855473e-08	1081
HIST1H4J	0.177726872360858	4.01574307399121e-09	2.67340199026943e-08	1081
NQO2	0.177693506232848	4.04308102839823e-09	2.68893586388519e-08	1081
NDUFAB1	0.177647192700604	4.08132719714143e-09	2.71079249543073e-08	1081
XYLT2	0.177635643159872	4.09091956467699e-09	2.7153731344454e-08	1081
NRL	0.177621238572479	4.10291387590184e-09	2.72243741864435e-08	1081
LSR	0.177610293676807	4.11205023961739e-09	2.72760131962767e-08	1081
SYCE2	0.177590675991863	4.12847581197855e-09	2.73759530060263e-08	1081
AP1M1	0.177543224240295	4.16847051256225e-09	2.76229731827521e-08	1081
NCRNA00	0.177442973407579	4.254208932496e-09	2.81725966289059e-08	1081
IRF8	0.177440122689778	4.25667186842234e-09	2.81796433747513e-08	1081
VPREB3	0.177422091267004	4.27228261973877e-09	2.82458593287291e-08	1081
SF4	0.177418027118671	4.27580884282485e-09	2.82598980113867e-08	1081
ATP6V0C	0.177367707823889	4.31970294471354e-09	2.85125870973965e-08	1081
FAM3A	0.177297362834154	4.38180043966556e-09	2.88846105553216e-08	1081
DHRSX	0.177284130637916	4.39357779603844e-09	2.89459084465072e-08	1081

TRPC4AP	0.177283687104689	4.39397309652118e-09	2.89459084465072e-08	1081
SEPX1	0.177279161362317	4.39800865589428e-09	2.89630220245147e-08	1081
BIRC7	0.177197063121635	4.47184324472415e-09	2.9439634694434e-08	1081
CCDC53	0.1770948328517	4.56546873682366e-09	3.00175122541871e-08	1081
CDK5R2	0.177094709979402	4.5655824049059e-09	3.00175122541871e-08	1081
FAM113B	0.177068302023751	4.59007609176891e-09	3.01687056667813e-08	1081
C4orf48	0.177022537633086	4.63282581987912e-09	3.04298259346152e-08	1081
CLEC2B	0.176972031564266	4.68045400535358e-09	3.0732642091867e-08	1081
C8G	0.176927063759732	4.72325965188591e-09	3.09834144211142e-08	1081
APLP1	0.176889330421071	4.75947200849396e-09	3.12006389883211e-08	1081
MELK	0.17678814994067	4.85791073869662e-09	3.1814893313083e-08	1081
COG1	0.176779498915809	4.86641855445921e-09	3.18499031122744e-08	1081
DNAJC9	0.176721868288055	4.92346578137211e-09	3.21814465171126e-08	1081
TXN	0.176709951060378	4.93534318504619e-09	3.2248617730378e-08	1081
TIMM50	0.176672837311418	4.97251155079417e-09	3.24809485054308e-08	1081
F8A1	0.176661217776751	4.98420398821742e-09	3.25467712617958e-08	1081
C20orf27	0.176611944972551	5.03408345856841e-09	3.28299162424282e-08	1081
IMP4	0.17659767436464	5.04862012257272e-09	3.29034139014e-08	1081
TIMELES:	0.176572584706042	5.07427649299696e-09	3.30385584846231e-08	1081
BIRC3	0.176464263748026	5.18650577188662e-09	3.37256806890433e-08	1081
TAGAP	0.176462402248783	5.18845535912234e-09	3.37274711873248e-08	1081
FXYD3	0.176397063695908	5.25733871928349e-09	3.41201960373601e-08	1081
PSENEN	0.176378930663253	5.27661250114299e-09	3.42122171984312e-08	1081
RAP2B	0.17614216248445	5.5346636934348e-09	3.58276992622892e-08	1081
GPR19	0.176109244903467	5.57149711867494e-09	3.60333225377263e-08	1081
HSPB1	0.17608341162316	5.60057017390173e-09	3.62077940158057e-08	1081
SELPLG	0.17606968541141	5.61607770690369e-09	3.62964021192091e-08	1081
ROM1	0.17594405607891	5.75996232127537e-09	3.71676221288097e-08	1081
TLX1	0.175943932976632	5.7601050526753e-09	3.71676221288097e-08	1081
PANX2	0.17592760071127	5.77907205750062e-09	3.72661352747599e-08	1081
SFXN4	0.175838571164222	5.88353555634718e-09	3.79033654565443e-08	1081
ATP1B3	0.175812393939524	5.91459820014566e-09	3.80912981911555e-08	1081
HAX1	0.175753299534353	5.98530819530254e-09	3.85097520263078e-08	1081

FKBP2	0.175641575091454	6.12124373885311e-09	3.93466672365016e-08	1081
NUDT22	0.175503375567753	6.29354491407143e-09	4.04026329808696e-08	1081
SNAI3	0.175424968255599	6.39338467092492e-09	4.10043725551679e-08	1081
CD96	0.175422042967622	6.39713919238327e-09	4.10153943445452e-08	1081
MRPS24	0.175374071694455	6.45901529390481e-09	4.13857707047431e-08	1081
NDUFB6	0.175269980129555	6.59528427930755e-09	4.21918074965546e-08	1081
RIPK2	0.175213668850172	6.6701625069343e-09	4.26302105654161e-08	1081
MAGIX	0.175181830336967	6.71286351654117e-09	4.28759148829175e-08	1081
RBM42	0.175076043216722	6.85665766342662e-09	4.37388754368997e-08	1081
C3orf1	0.175044561123402	6.90002513039501e-09	4.39876602062682e-08	1081
C15orf48	0.174792173937482	7.25745710239314e-09	4.61057941746168e-08	1081
SUPT4H1	0.174781078056645	7.27357691649175e-09	4.61790756327534e-08	1081
GRHPR	0.174770238017418	7.28935862414571e-09	4.62646910785808e-08	1081
MICALL2	0.174702044796477	7.38940449277876e-09	4.68701364946562e-08	1081
PIN1	0.17466151350668	7.44949814582221e-09	4.71870219005334e-08	1081
SPR	0.174608992155653	7.52807512360861e-09	4.76446978841016e-08	1081
IL20RB	0.174586586031227	7.56184121112194e-09	4.78283488847885e-08	1081
ISCA2	0.174524222017223	7.65660012310575e-09	4.83973045120695e-08	1081
TBL2	0.174515288185018	7.67026869094852e-09	4.84684952255828e-08	1081
SCML4	0.174406329702653	7.83889137979595e-09	4.94874543547444e-08	1081
GRPEL1	0.174359786488119	7.9120132265957e-09	4.99334293389005e-08	1081
PTGIR	0.174276627438778	8.04431261013457e-09	5.07092288449044e-08	1081
MRPS33	0.174224168885346	8.12887217518561e-09	5.11737906153482e-08	1081
C4orf50	0.174220470596125	8.13486601924544e-09	5.11955251351763e-08	1081
C20orf107	0.174187366915911	8.18870885233619e-09	5.15021978864541e-08	1081
C11orf84	0.174158900448268	8.23528587110768e-09	5.17721806606251e-08	1081
TRAF2	0.174157992717672	8.23677532972466e-09	5.17721806606251e-08	1081
RAN	0.17408432427428	8.35853085030356e-09	5.25047096911024e-08	1081
SLC2A4R(	0.174075861279976	8.37262946178332e-09	5.25768767168407e-08	1081
MLX	0.173951999104608	8.58163385072765e-09	5.38389953045495e-08	1081
CBR3	0.173913704318829	8.64727116816684e-09	5.41832901034902e-08	1081
SMS	0.173853262599974	8.75186122868991e-09	5.48215934241164e-08	1081
MAPKAPI	0.173801419652463	8.84254933904179e-09	5.53638924529408e-08	1081

TEX11	0.173793776786002	8.85599581852626e-09	5.54221919118396e-08	1081
APOA1BP	0.173772449332618	8.8936233493854e-09	5.56231115719866e-08	1081
STX5	0.173708202813107	9.00791236038259e-09	5.63029458578676e-08	1081
HIST1H1D	0.173657744645155	9.09867147378192e-09	5.68218697331504e-08	1081
COX4I1	0.173603400731224	9.19741271583711e-09	5.74120169813708e-08	1081
MRPL2	0.17360220201874	9.19960242322487e-09	5.74120169813708e-08	1081
APITD1	0.173597783475412	9.20767821905529e-09	5.74446199203682e-08	1081
PLEKHO2	0.173483818422328	9.4183668509049e-09	5.87065577568372e-08	1081
LYN	0.173478589119408	9.42814578790042e-09	5.87472925757049e-08	1081
HEXA	0.173452231264386	9.47758591641322e-09	5.90006082466454e-08	1081
C12orf44	0.173384405279767	9.60596875775198e-09	5.97628908662488e-08	1081
NME2	0.173303076874954	9.76213595588892e-09	6.07157236280896e-08	1081
CCDC107	0.173298963649813	9.77009931607872e-09	6.0746497136545e-08	1081
NOSIP	0.173287867630258	9.79161309268992e-09	6.08614766282748e-08	1081
SLAMF1	0.173039512881333	1.0285352686398e-08	6.3772985493225e-08	1081
DNAJC30	0.172883293654087	1.06082139778332e-08	6.57343803701781e-08	1081
IGSF6	0.172851171191853	1.06758108073466e-08	6.61125756882869e-08	1081
TOMM34	0.172728353240191	1.09381382702793e-08	6.76954824745855e-08	1081
NCR1	0.172708827157477	1.09804163637503e-08	6.79362677050827e-08	1081
NUP93	0.172607828653044	1.12016436452797e-08	6.92412124069221e-08	1081
NDUFA11	0.172522484718887	1.1391947616478e-08	7.030967669545e-08	1081
WDR77	0.172378651522988	1.17197955325355e-08	7.22666914609515e-08	1081
KIF4A	0.172259479495929	1.19983507499072e-08	7.39390565484489e-08	1081
TCF19	0.172241514556568	1.20408957332352e-08	7.41785457327288e-08	1081
STRA13	0.172234448093691	1.20576707770241e-08	7.42364846586643e-08	1081
UQCR11	0.172228291783354	1.20723036962411e-08	7.42990183549458e-08	1081
NIT2	0.172200566509108	1.21384176551564e-08	7.46423759655453e-08	1081
COPG	0.172130494055263	1.23070830172683e-08	7.55365475233661e-08	1081
KCNAB2	0.172129872338708	1.2308589627537e-08	7.55365475233661e-08	1081
PSMG3	0.172129258953677	1.23100762233413e-08	7.55365475233661e-08	1081
LACTB	0.172056013308043	1.24888519844573e-08	7.65635798012454e-08	1081
ARMC6	0.171989546202182	1.2653261274465e-08	7.75243151989345e-08	1081
POLR2G	0.171896845406229	1.28860718838502e-08	7.89027106687426e-08	1081

FN3K	0.171808639175746	1.31114502343284e-08	8.01608998393156e-08	1081
DCTN3	0.171800984363035	1.31311886356453e-08	8.02572193765394e-08	1081
RAB35	0.171777255291154	1.31925588667966e-08	8.05834136966701e-08	1081
HMOX2	0.171753497094269	1.32542831706213e-08	8.08868629118949e-08	1081
FADS3	0.171682445894209	1.3440552231139e-08	8.19491297506948e-08	1081
SEC61A1	0.171669556060123	1.34746155147932e-08	8.2131960528142e-08	1081
CENPB	0.17166632375544	1.34831704812597e-08	8.21592466258246e-08	1081
FTSJ3	0.171605169299134	1.3646024956301e-08	8.3076208142848e-08	1081
SLC25A11	0.171525158310444	1.38619783414002e-08	8.4238176074663e-08	1081
SCT	0.171491418722741	1.39540335727203e-08	8.47209181200877e-08	1081
UFSP1	0.171442977275472	1.40872391306207e-08	8.54524035791488e-08	1081
LENG1	0.17139755542421	1.42132614059784e-08	8.61908943478131e-08	1081
MRPL11	0.17134572929241	1.43583882451348e-08	8.69924003603732e-08	1081
EMILIN2	0.171339649832678	1.43755062447101e-08	8.70699258267246e-08	1081
SRM	0.171331313607904	1.4399010829223e-08	8.71598777508103e-08	1081
GBAP1	0.171305332038727	1.44725071405935e-08	8.75784488877311e-08	1081
BCL2L14	0.171255296187689	1.46150744773769e-08	8.83880742560063e-08	1081
RNASEH2	0.171250969254361	1.46274670253807e-08	8.8436471556511e-08	1081
HGS	0.171225277484448	1.47012597199147e-08	8.88365704072082e-08	1081
CFDP1	0.171206671542217	1.47549256018745e-08	8.91268294601986e-08	1081
DPF1	0.171152597086107	1.49119756964661e-08	8.9994532775707e-08	1081
LOC10018	0.171144673992174	1.4935122748552e-08	9.01072320364123e-08	1081
PUF60	0.17111027730101	1.50360162408852e-08	9.06887865786326e-08	1081
ATP5B	0.171105465202296	1.50501838545729e-08	9.07470678690126e-08	1081
DCXR	0.171056313292458	1.51956387978884e-08	9.15692921278676e-08	1081
AURKC	0.170970313970658	1.54534225631205e-08	9.29558666867907e-08	1081
DENND1C	0.170968468799189	1.54589996993622e-08	9.29616564010903e-08	1081
WNT10A	0.170877662533618	1.57358921527994e-08	9.44012946450694e-08	1081
C7orf55	0.170823650773974	1.59028635220721e-08	9.52611316241876e-08	1081
GLI4	0.170741807806943	1.61591512011042e-08	9.67388115739209e-08	1081
SEC11C	0.170713858468828	1.62475874494797e-08	9.72104690138905e-08	1081
TMED1	0.170689232940485	1.6325895474709e-08	9.76499894115242e-08	1081
DDX41	0.17062677479834	1.65261545550315e-08	9.87598289857935e-08	1081

LRPAP1	0.170618594733642	1.65525580252762e-08	9.88882803734249e-08	1081
RPP38	0.170601919542434	1.66065086811414e-08	9.91811791822099e-08	1081
MAD1L1	0.170513341832928	1.68959629140099e-08	1.00790397661454e-07	1081
DDA1	0.170480584947519	1.70042402760868e-08	1.0140628193066e-07	1081
RBBP7	0.170477292072797	1.70151619699639e-08	1.01439577575776e-07	1081
ITK	0.170419630599966	1.72075177857681e-08	1.0249717498353e-07	1081
CABLES2	0.170417419327583	1.7214936311546e-08	1.02511052910462e-07	1081
PSMD13	0.170365262321032	1.73908182389183e-08	1.03527787654554e-07	1081
BET3L	0.170317636375424	1.75529418983551e-08	1.04431191536433e-07	1081
C19orf60	0.17027072705902	1.77140583365034e-08	1.05327539902261e-07	1081
TMEM173	0.170267674560439	1.77245921084761e-08	1.0535907584103e-07	1081
ARL9	0.170237156001648	1.78302421553707e-08	1.05955819533906e-07	1081
DUSP2	0.170106721501935	1.82887073974671e-08	1.08328729938834e-07	1081
ADORA3	0.170098509516078	1.83179511234592e-08	1.08446684982499e-07	1081
ANKRD54	0.170098106383333	1.83193878875872e-08	1.08446684982499e-07	1081
PUS1	0.170086344540398	1.83613552041381e-08	1.08635529172919e-07	1081
EVI2B	0.169968631615598	1.87865336819201e-08	1.10951252132008e-07	1081
PHYH	0.169793409872412	1.94371651852446e-08	1.14692938680947e-07	1081
TFPT	0.169746331460979	1.96156669099306e-08	1.15610769426727e-07	1081
SYVN1	0.169739938732055	1.96400277005621e-08	1.15720490794918e-07	1081
GBP7	0.169726264319437	1.96922353430772e-08	1.15994175726985e-07	1081
UGT3A2	0.169713951726962	1.97393587844069e-08	1.16169845957311e-07	1081
NDUFS7	0.169668589648338	1.99139162912777e-08	1.17128713485486e-07	1081
TXNDC11	0.169663874802326	1.99321450843299e-08	1.17171566885912e-07	1081
CSF2	0.169663696456354	1.99328349326393e-08	1.17171566885912e-07	1081
PDZD11	0.169648971164078	1.9989872793627e-08	1.17443368992453e-07	1081
TMEM111	0.169615052941978	2.0121856416961e-08	1.18144796712235e-07	1081
C19orf59	0.169571005726522	2.02945157920826e-08	1.19054461453554e-07	1081
FAM64A	0.169534401672307	2.04390909426911e-08	1.19832795995493e-07	1081
MAGOHB	0.169508166681604	2.05433251398551e-08	1.20393082550329e-07	1081
PRAM1	0.169507342511769	2.05466079825281e-08	1.20393082550329e-07	1081
HEXB	0.169486274166488	2.0630700541993e-08	1.20815541400712e-07	1081
KIR2DS4	0.169472952047507	2.06840467760486e-08	1.21057560227629e-07	1081

LGALS2	0.169445556753537	2.07941672988432e-08	1.21666715142374e-07	1081
KLC2	0.169416532893333	2.09114541650298e-08	1.22317434423498e-07	1081
LYZ	0.169405687029022	2.0955447241524e-08	1.22539182780987e-07	1081
B3GNTL1	0.169355711005105	2.11593222786186e-08	1.23659572759725e-07	1081
HNRNPL	0.169299364160973	2.13914931756082e-08	1.24871524202442e-07	1081
SAE1	0.169297795260854	2.13979928632117e-08	1.24873281062978e-07	1081
UTP11L	0.169279717188271	2.14730255216359e-08	1.25274862187476e-07	1081
C6orf108	0.169200015571717	2.18068774333552e-08	1.27148927900128e-07	1081
BLOC1S3	0.16919367510963	2.18336511847494e-08	1.27268201133327e-07	1081
KIAA031[illegible]	0.169158226017912	2.19839295277467e-08	1.28033032765671e-07	1081
DLGAP5	0.169146411762069	2.20342357710514e-08	1.28288924742148e-07	1081
SNX22	0.169064963539566	2.23840992659535e-08	1.30100311515474e-07	1081
KTI12	0.168978578473447	2.27610523714495e-08	1.32062615213955e-07	1081
MAN2B1	0.168970780051801	2.27953830743802e-08	1.32223723591531e-07	1081
PTP4A3	0.168949376957953	2.28898632348047e-08	1.3261900916455e-07	1081
CCDC101	0.168768273443924	2.37046523186612e-08	1.36993489874708e-07	1081
MRPL28	0.168687820209288	2.40755665761584e-08	1.39008967806452e-07	1081
FSD1	0.168653006686331	2.42378057173961e-08	1.39905614950414e-07	1081
SNRNP25	0.168646261906546	2.42693602645428e-08	1.40047626046753e-07	1081
PLEKHB1	0.168633505912342	2.43291463609495e-08	1.40312239748448e-07	1081
GPR97	0.168613495981645	2.44232186348338e-08	1.40814464624707e-07	1081
UTP6	0.168589982161753	2.45342142525982e-08	1.41413947387293e-07	1081
CEP55	0.168497752917495	2.49743155309174e-08	1.43745024677237e-07	1081
C14orf2	0.168477450410511	2.50722179103787e-08	1.44226107882518e-07	1081
TMEM10[illegible]	0.168452448280681	2.51932938690134e-08	1.44881217525342e-07	1081
ACOT11	0.168184579753871	2.65265577810671e-08	1.52152633518477e-07	1081
BAD	0.16812742946202	2.68197359549497e-08	1.5370798885134e-07	1081
DYNLL1	0.168105892832073	2.69310303618818e-08	1.54301935904468e-07	1081
CHST13	0.168084604470897	2.70414815582256e-08	1.54890715379714e-07	1081
TSR2	0.16803189302891	2.73168586599619e-08	1.56379118415724e-07	1081
ARPC2	0.168028288316481	2.73357894796596e-08	1.56443033826063e-07	1081
ERGIC3	0.168022393863399	2.73667726924743e-08	1.56564244446715e-07	1081
MDH1	0.16799625851288	2.75045592314303e-08	1.57230234312475e-07	1081

CCR3	0.167965043026207	2.76700094785583e-08	1.58131160551931e-07	1081
TMEM171	0.167962512901132	2.76834619735757e-08	1.58163171145117e-07	1081
ZGPAT	0.167959387399792	2.77000887917868e-08	1.58213294219038e-07	1081
CDK1	0.16794596606555	2.77715967001979e-08	1.58490577967155e-07	1081
SERTAD3	0.167915685363649	2.79335877539303e-08	1.59366220703179e-07	1081
TUBB4	0.167901237277042	2.80112021904548e-08	1.59756391140433e-07	1081
S100A14	0.167893774539323	2.8051373463121e-08	1.59902353824158e-07	1081
PFKFB4	0.16788245505779	2.81124118237055e-08	1.60204960732262e-07	1081
NME2P1	0.167836285469818	2.83627090409756e-08	1.61494283106403e-07	1081
ZNF165	0.167812285967703	2.84936680816129e-08	1.62194106669706e-07	1081
CD81	0.167737483105555	2.89056177197116e-08	1.64446108151254e-07	1081
CC2D1B	0.167699035929738	2.91195909069493e-08	1.65569900880749e-07	1081
NDUFB5	0.167678211675928	2.9236125710947e-08	1.66185596062931e-07	1081
GAPDH	0.167663709707546	2.93175470819381e-08	1.6660140647832e-07	1081
FBXO22O	0.167623260892509	2.95458098256022e-08	1.67803873396323e-07	1081
B4GALT7	0.167591239759538	2.97277332684688e-08	1.68694418786846e-07	1081
PIN4	0.167562663458746	2.98910014551259e-08	1.69573141175306e-07	1081
LY9	0.167472388140771	3.04125089800784e-08	1.72337550887111e-07	1081
SMPD3	0.167460264228724	3.04832149941625e-08	1.72689641748426e-07	1081
SIRT6	0.167453817204377	3.05208785326382e-08	1.72854399224064e-07	1081
RBM17	0.167411638666244	3.07684003473176e-08	1.74158309917593e-07	1081
PSMG4	0.16738052870179	3.09522120578286e-08	1.75149525815999e-07	1081
TREML2	0.167369533588374	3.10174302303376e-08	1.75420025825421e-07	1081
ATAD3A	0.167313473923293	3.13520267010414e-08	1.7726258150224e-07	1081
NRSN2	0.167306712206405	3.13926200723099e-08	1.77442292748788e-07	1081
TMSL3	0.167301351253525	3.14248402632369e-08	1.7757458824766e-07	1081
KIAA1539	0.167277350176051	3.15694838101836e-08	1.78341910083048e-07	1081
SUMO2	0.167138398984725	3.24196404899232e-08	1.82888170727948e-07	1081
SPSB2	0.167084547400865	3.27550530178065e-08	1.84573578473766e-07	1081
RNF187	0.167081617987546	3.27733948357071e-08	1.846252905384e-07	1081
UBE2O	0.167072710138026	3.28292303420975e-08	1.84888131182989e-07	1081
C16orf57	0.167065189239495	3.28764440007488e-08	1.85102281832053e-07	1081
ADCK5	0.166982923634188	3.33971931841055e-08	1.87854578747395e-07	1081

PARP15	0.166874646269534	3.40947910048194e-08	1.91480224363559e-07	1081
C9orf142	0.166818840889565	3.44598187901145e-08	1.93476323725434e-07	1081
TLK2	0.16681249319533	3.45015785923767e-08	1.93598833658022e-07	1081
C17orf82	0.16680338282673	3.45615989691841e-08	1.93831684642042e-07	1081
PPPDE2	0.166674771790611	3.54197743068511e-08	1.98313327796419e-07	1081
HS1BP3	0.166672850500169	3.5432749550555e-08	1.98330852930239e-07	1081
DUSP14	0.166668774782573	3.54602897468528e-08	1.98374767273077e-07	1081
IL1R2	0.166663470028332	3.54961657707738e-08	1.9852033854865e-07	1081
UBL7	0.166607563423223	3.58764057827941e-08	2.00368781395727e-07	1081
DPY30	0.166578810558166	3.60734970843053e-08	2.0130210492059e-07	1081
VTN	0.166573510535813	3.6109941255797e-08	2.01424016308799e-07	1081
FBXO44	0.166572725681871	3.61153411222329e-08	2.01424016308799e-07	1081
C15orf42	0.166555519562954	3.62339174804237e-08	2.01945092206976e-07	1081
TNFSF8	0.166554805905961	3.62388437988692e-08	2.01945092206976e-07	1081
CST3	0.166535606455094	3.63716198477928e-08	2.02572928347743e-07	1081
CUTA	0.166518762505118	3.64884940521429e-08	2.03111553655822e-07	1081
SIRT5	0.166451310307771	3.6960176250549e-08	2.05623515760096e-07	1081
LPAR2	0.166407158627741	3.72721136893616e-08	2.07187287602702e-07	1081
TPRA1	0.166323395645402	3.78709318247331e-08	2.10457909409448e-07	1081
BRD7	0.166212401311673	3.86787945182221e-08	2.14710475494512e-07	1081
NAPRT1	0.166070072645855	3.97391711930891e-08	2.20172058219137e-07	1081
MGC1298	0.166037891229976	3.99828022806937e-08	2.21400096741224e-07	1081
SERPING	0.165708329299078	4.25624845058047e-08	2.35038719947762e-07	1081
ZCCHC17	0.165594075782142	4.34938789214555e-08	2.400504632528e-07	1081
ZFPL1	0.165554400408838	4.38219049802757e-08	2.41662270962949e-07	1081
SEMA7A	0.16552335935665	4.40802134111931e-08	2.43020224184041e-07	1081
NRBP1	0.165507073434133	4.42163258475302e-08	2.43570654689225e-07	1081
TRAT1	0.165334590037017	4.56830909718101e-08	2.51169723697356e-07	1081
STIP1	0.165286765442816	4.60980527921053e-08	2.53243870601844e-07	1081
VDAC2	0.165220728260385	4.66770432867454e-08	2.56284828839326e-07	1081
NUAK2	0.165189093574087	4.69568928361063e-08	2.57751118851052e-07	1081
PARK7	0.165176574215284	4.70680906768335e-08	2.58291115958815e-07	1081
PACSIN1	0.165139209258051	4.74014867308546e-08	2.59979022649895e-07	1081

MYL6	0.165088876846689	4.78542030845962e-08	2.62319162214746e-07	1081
MYO1A	0.16504048208503	4.82934363753724e-08	2.64654862835113e-07	1081
ERP44	0.164992432299007	4.87333983050993e-08	2.66920693000605e-07	1081
TAF12	0.16499082726117	4.87481613974078e-08	2.66928978350307e-07	1081
OCEL1	0.164962276358939	4.90114963323911e-08	2.68225100140185e-07	1081
C16orf13	0.164939757963722	4.92201621214559e-08	2.69289098299056e-07	1081
DSCR9	0.164938413173957	4.92326507339501e-08	2.69289098299056e-07	1081
HIST1H4I	0.164814606470218	5.03956366813278e-08	2.75351261444358e-07	1081
KATNB1	0.164770218478573	5.08190387036187e-08	2.77438898288455e-07	1081
SFN	0.164720799325152	5.12944819232835e-08	2.7980702365138e-07	1081
GNL2	0.164714633593119	5.13541013461259e-08	2.80056408126071e-07	1081
GPR31	0.164665415062944	5.1832429530565e-08	2.82511984007909e-07	1081
CCDC90A	0.1646250973075	5.22274661869617e-08	2.84588127221083e-07	1081
CIITA	0.164616720355421	5.23099090891664e-08	2.84883243741892e-07	1081
SYP	0.164542110990082	5.3049765191376e-08	2.88600464428914e-07	1081
RUVBL2	0.164483511967143	5.36379473290452e-08	2.91564071490452e-07	1081
MCM10	0.164468362959593	5.3791027791164e-08	2.92159680467242e-07	1081
POLR2I	0.164440300314422	5.40757181561245e-08	2.93547653531428e-07	1081
TST	0.164321351735236	5.52987029924822e-08	3.00024877937935e-07	1081
RECQL5	0.164212984353079	5.64361657681062e-08	3.05949020290231e-07	1081
SQRDL	0.164118503088662	5.74463229687093e-08	3.10506084304977e-07	1081
NCRNA0(	0.164116268420624	5.74704257196589e-08	3.10553038122996e-07	1081
LOC2853:	0.16407941864714	5.78692951500967e-08	3.12540737479543e-07	1081
CHCHD2	0.163951101347566	5.92792400971062e-08	3.19555871489485e-07	1081
TKT	0.163947589810095	5.93182882187192e-08	3.19680822944381e-07	1081
TIMM8B	0.163843605692417	6.04859331905308e-08	3.25538104227423e-07	1081
TIMP1	0.1638346199047	6.05878727216726e-08	3.25912602397355e-07	1081
MAD2L2	0.163832743388218	6.06091818898313e-08	3.25940194653137e-07	1081
IFITM2	0.163761029046188	6.14290050235642e-08	3.30172707097039e-07	1081
DONSON	0.163714019148506	6.19722230912678e-08	3.328260288386e-07	1081
SPIRE2	0.163589920648579	6.3428653929644e-08	3.40194417841501e-07	1081
SERPINA	0.163487940292232	6.46502413540501e-08	3.46561764789074e-07	1081
PYY2	0.16343633912868	6.52769951435791e-08	3.49735390204096e-07	1081

TSEN34	0.163339434330098	6.64699340757463e-08	3.55465044320655e-07	1081
NTHL1	0.163310180893605	6.68341817705709e-08	3.57318097603013e-07	1081
NAT8	0.163264664695112	6.74047668888255e-08	3.60177461266682e-07	1081
HIST1H2/	0.163177936568062	6.85050510314317e-08	3.65571987557137e-07	1081
LGALS9B	0.163163926473754	6.86844135855141e-08	3.66432074067845e-07	1081
PPDPF	0.163078149933512	6.97925028303795e-08	3.7192727829552e-07	1081
CSF1R	0.163076777294856	6.98103748358822e-08	3.7192727829552e-07	1081
JOSD2	0.163075637732145	6.9825215513212e-08	3.7192727829552e-07	1081
HAUS7	0.163028448009166	7.04424605222365e-08	3.750167460942e-07	1081
DERL2	0.1629413420162	7.1595703985953e-08	3.8065332720956e-07	1081
GPATCH3	0.162890310919079	7.2279793409098e-08	3.84189033832791e-07	1081
RFPL2	0.162873042750805	7.25127075426126e-08	3.85325374161417e-07	1081
PPFIA3	0.162848205499999	7.28489873459952e-08	3.86806233549044e-07	1081
MOGS	0.162781426412167	7.37606285145614e-08	3.91337335113468e-07	1081
LOC72874	0.162772906776433	7.38777258095233e-08	3.91855394005489e-07	1081
JAK3	0.162740968183813	7.43183051609576e-08	3.93984804596708e-07	1081
GUK1	0.16273151880363	7.44491417739555e-08	3.94574575384461e-07	1081
FANCG	0.162627101756143	7.59098187001329e-08	4.01998763857565e-07	1081
NENF	0.162551869476807	7.69793724180681e-08	4.07341596365112e-07	1081
TESK1	0.16253832019233	7.71735408777687e-08	4.08261812232839e-07	1081
PLEKHJ1	0.162518724154246	7.7455200649602e-08	4.09536749891399e-07	1081
NUF2	0.162497858437684	7.77562027061686e-08	4.11020389272046e-07	1081
THAP11	0.162488941949767	7.78851737799849e-08	4.11486185627536e-07	1081
C8orf30A	0.162484976230926	7.79426017136348e-08	4.11681623366852e-07	1081
C11orf31	0.162435363584249	7.86645167283027e-08	4.14842065311952e-07	1081
PPP1R9B	0.162293773119227	8.07605433880856e-08	4.25561377596909e-07	1081
PPA1	0.162239901876141	8.15721222336928e-08	4.29613177097449e-07	1081
PDAP1	0.162215944955212	8.19355653071927e-08	4.31301793340318e-07	1081
NARF	0.162156225435709	8.28483816747557e-08	4.35765182464218e-07	1081
HIST2H2/	0.162135598917577	8.31659379686146e-08	4.3709309167173e-07	1081
PXMP2	0.162118200720821	8.34347073153728e-08	4.38391282959881e-07	1081
PABPN1	0.162089108950653	8.38859977431102e-08	4.40647568327237e-07	1081
MPEG1	0.162071953734008	8.41532259579975e-08	4.41936062806011e-07	1081

ADRBK1	0.162023969601124	8.4905057180446e-08	4.45535914795542e-07	1081
NCAPH	0.161928141984817	8.64260005850087e-08	4.53280859615986e-07	1081
STAG3	0.161893418112936	8.69836016944926e-08	4.55925534348288e-07	1081
CD33	0.161892514951584	8.6998151106221e-08	4.55925534348288e-07	1081
CCL22	0.161826512615914	8.80678039910279e-08	4.61171279282365e-07	1081
IL1B	0.161768763880791	8.90141173243148e-08	4.658844877886e-07	1081
BTLA	0.161729603399146	8.96614156039318e-08	4.68906858084425e-07	1081
KRT36	0.161705716858082	9.00584772268942e-08	4.70616867376338e-07	1081
NAGLU	0.161700323921078	9.014835798816e-08	4.7096438580692e-07	1081
SLC25A41	0.161669221202139	9.06684227239905e-08	4.73435815390044e-07	1081
TIGD3	0.161556534459114	9.25770456656246e-08	4.82901238978251e-07	1081
S100A4	0.161478589079968	9.39199035873904e-08	4.8952560356222e-07	1081
LOC15153	0.16146311632406	9.41887020881029e-08	4.90799638790697e-07	1081
COMMD7	0.16146014625716	9.42403842113039e-08	4.90941954987514e-07	1081
HIST1H1E	0.161449291996341	9.44294924306854e-08	4.91799928908004e-07	1081
MEI1	0.161367188381958	9.58718718112835e-08	4.99053968381992e-07	1081
KIAA1949	0.161326394951405	9.65964193975378e-08	5.02695651966778e-07	1081
C10orf35	0.16131596915831	9.6782443454237e-08	5.03533657899175e-07	1081
ESPNL	0.16130721021522	9.69389940808375e-08	5.04217928158655e-07	1081
TGFB1	0.161256904730809	9.78428669486031e-08	5.0878795939071e-07	1081
HIST1H2A	0.161246867876866	9.80241782427027e-08	5.09599244051418e-07	1081
OAZ1	0.161218680242997	9.85351138339681e-08	5.11859171785788e-07	1081
HMBS	0.161165237654578	9.95109028650158e-08	5.16528507656723e-07	1081
NCAPG	0.161128377817967	1.00189349198852e-07	5.19782240435454e-07	1081
HIST1H2B	0.16110645221145	1.0059503286161e-07	5.21484029078009e-07	1081
CCT7	0.161090384536853	1.00893336438641e-07	5.22895874081919e-07	1081
ZNHIT2	0.161060600320249	1.0144855676819e-07	5.25638162575925e-07	1081
THAP3	0.161036250776906	1.01904660058773e-07	5.27865615038306e-07	1081
NR2F6	0.160958341251137	1.03377377227417e-07	5.34669387483006e-07	1081
HSF1	0.160956920599797	1.03404421728304e-07	5.34671990687032e-07	1081
CD28	0.160867121119138	1.05127854380354e-07	5.43025801664672e-07	1081
MT1H	0.160850859911169	1.05442894352934e-07	5.44513485449846e-07	1081
C1orf31	0.160795404014148	1.06524157640411e-07	5.49956216213759e-07	1081

LSM2	0.160721634914428	1.07979106806074e-07	5.56611849183309e-07	1081
PTPN22	0.160712062642152	1.08169302831911e-07	5.57307060242671e-07	1081
CKM	0.160703905120688	1.08331643996058e-07	5.58000758962055e-07	1081
GLT25D1	0.160693488793259	1.0853927951585e-07	5.58641743956771e-07	1081
CD3G	0.16069101474734	1.08588652798903e-07	5.58753106164469e-07	1081
ELMO2	0.160642876537784	1.09553653776357e-07	5.63574656620202e-07	1081
FAM32A	0.16064047203662	1.09602072220961e-07	5.63679791904838e-07	1081
C6orf81	0.160629632562336	1.0982059909502e-07	5.64659512192234e-07	1081
BUB1	0.160611442206425	1.10188268382853e-07	5.66405375496959e-07	1081
ORC1L	0.160575083487206	1.1092673085852e-07	5.70055865598183e-07	1081
ERP29	0.160572036235364	1.10988838717241e-07	5.7022957305759e-07	1081
NAGA	0.16056613758275	1.11109158177144e-07	5.70644413157502e-07	1081
C12orf59	0.16056530082315	1.1112623642675e-07	5.70644413157502e-07	1081
C1orf182	0.160534201577298	1.11762773855584e-07	5.73474548986435e-07	1081
ZMAT5	0.160503415143083	1.12396378497352e-07	5.76432037889299e-07	1081
RELB	0.160497900810556	1.12510232971567e-07	5.7686908709906e-07	1081
TPD52L2	0.16049184532447	1.12635389043068e-07	5.77363845361985e-07	1081
RHOD	0.160421258933533	1.14104244318495e-07	5.84595626092592e-07	1081
ERH	0.160400475279371	1.14540254106492e-07	5.8653111819402e-07	1081
SCAMP5	0.160338546912094	1.15848984772301e-07	5.92780746313946e-07	1081
LYPLA2	0.160327263982043	1.16088977710921e-07	5.93857911626842e-07	1081
FKBP1A	0.160277669700881	1.17149578951453e-07	5.99131319618438e-07	1081
SIGLEC8	0.16019715596631	1.18891377680016e-07	6.07730729095135e-07	1081
PHKG2	0.160143392795883	1.20068371839336e-07	6.13280261334539e-07	1081
STAP2	0.160128457075433	1.20397340844311e-07	6.14804672068097e-07	1081
TFE3	0.160105664258102	1.20901046735e-07	6.17220371636231e-07	1081
MTCP1NB	0.160086754470762	1.21320485106946e-07	6.19204756138694e-07	1081
C6orf62	0.160064935640154	1.21806196164898e-07	6.20740152224102e-07	1081
DMBX1	0.159995015349183	1.23375389068646e-07	6.282601650121e-07	1081
LOC33967	0.159982381942702	1.23660991180232e-07	6.29555387244321e-07	1081
C9orf23	0.159968230352214	1.23981673260384e-07	6.3086911033858e-07	1081
C16orf70	0.159896783603407	1.25612999126601e-07	6.38685983696461e-07	1081
C18orf8	0.159803747619138	1.27768375275322e-07	6.48826296930012e-07	1081

HPCA	0.159800946793542	1.27833813211072e-07	6.48995001798649e-07	1081
MOBKL2(	0.159794691198043	1.27980084647525e-07	6.49573899023529e-07	1081
NUSAP1	0.159787066460303	1.28158588707633e-07	6.50316062850193e-07	1081
HIST1H2I	0.159728486730927	1.29538054657244e-07	6.56300628901954e-07	1081
C1orf50	0.159709881190867	1.29979181201106e-07	6.58227904800471e-07	1081
NAA20	0.159569846310249	1.33346210016364e-07	6.73923582734482e-07	1081
NFU1	0.159555485592176	1.33696227532253e-07	6.75523075906003e-07	1081
ERAS	0.15949459176193	1.35190294478082e-07	6.82900823034342e-07	1081
MICAL1	0.159478279649003	1.35593252994214e-07	6.84593002899358e-07	1081
CENPW	0.159447331245121	1.36360963302239e-07	6.88296568710501e-07	1081
CTU2	0.159431320351556	1.36759777970477e-07	6.89963868573821e-07	1081
MRPL41	0.159240609193369	1.415976871017e-07	7.12764969181347e-07	1081
FCRLB	0.158997092232188	1.48015891911618e-07	7.42660060413337e-07	1081
IDH3A	0.15897792325472	1.4853287509003e-07	7.45068418498173e-07	1081
PHB	0.158974492772342	1.48625578245991e-07	7.4527723698364e-07	1081
CREB3	0.158971185297403	1.48715010235865e-07	7.45425200597537e-07	1081
DGAT1	0.158946977228197	1.49371164957247e-07	7.48341735405057e-07	1081
TMEM14I	0.158890674327739	1.50908075062302e-07	7.55665715170288e-07	1081
IKBKE	0.158874481532611	1.51352912989457e-07	7.57516629111206e-07	1081
MRPS18B	0.15886827830445	1.51523659297491e-07	7.57994565817718e-07	1081
STMN1	0.158813704663244	1.53033859311322e-07	7.64789654137084e-07	1081
S100A11	0.158724666576139	1.55529037970973e-07	7.76873907742437e-07	1081
NRD1	0.158685150982637	1.56648957039603e-07	7.81886332894649e-07	1081
MRPL51	0.15863897086733	1.57967626182032e-07	7.88272932731494e-07	1081
C14orf72	0.158300627902909	1.67961299067866e-07	8.35451943141275e-07	1081
LRRC33	0.158275856500244	1.6871648436921e-07	8.39001129996973e-07	1081
TMEM17(	0.158264600741315	1.6906071167414e-07	8.40283205793795e-07	1081
ZNRF1	0.158256557737204	1.69307100751244e-07	8.41107655889963e-07	1081
TMEM21	0.158251414344364	1.69464844957859e-07	8.41683752878716e-07	1081
NFKBIL2	0.15824380678517	1.69698423589603e-07	8.42636121077777e-07	1081
HIST1H3I	0.158223060347699	1.70336992476584e-07	8.45598500108622e-07	1081
TXNDC12	0.158088111004981	1.74547675068129e-07	8.65222173781361e-07	1081
FAM129C	0.158072045294261	1.75055600152375e-07	8.67526461271733e-07	1081

LRRC20	0.158051910825301	1.7569417602867e-07	8.70476924765755e-07	1081
XKRX	0.15804256161095	1.75991455575238e-07	8.71735400187651e-07	1081
WIBG	0.158035615668161	1.76212631988593e-07	8.72616389235546e-07	1081
CDC7	0.157947512777973	1.79041445386343e-07	8.8553643930957e-07	1081
LOC38858	0.157844402550491	1.82407809765222e-07	9.01522406212071e-07	1081
FAM83D	0.157841836412702	1.82492363945063e-07	9.01719075710891e-07	1081
SNRPD1	0.157839617132961	1.82565519670252e-07	9.01859341284264e-07	1081
IGFL2	0.157786179222412	1.84335604440646e-07	9.10157046925689e-07	1081
C11orf73	0.157761834040557	1.8514749993171e-07	9.13494094078937e-07	1081
ITIH1	0.157724405733412	1.86402445810504e-07	9.19010590027557e-07	1081
MED19	0.157692738315752	1.87470641955461e-07	9.23824873334827e-07	1081
C3orf37	0.157652849563099	1.88824569166855e-07	9.29814457557146e-07	1081
TBC1D7	0.157648219521775	1.88982334739322e-07	9.30363913324446e-07	1081
PYCR2	0.157606986966268	1.90392929922417e-07	9.36621629618336e-07	1081
CHCHD10	0.157589257116935	1.9100260046321e-07	9.39162164103338e-07	1081
SH3TC1	0.157569480481345	1.91684876498739e-07	9.42286929494166e-07	1081
CFB	0.157525935773898	1.93195431722084e-07	9.4948084216672e-07	1081
AURKAPS	0.157518412835211	1.93457561955351e-07	9.50537215997696e-07	1081
KCNK12	0.157344913879716	1.99599120605765e-07	9.77613490056682e-07	1081
WDR74	0.157321082733572	2.00457261617603e-07	9.81577913292806e-07	1081
PHPT1	0.1573128582287	2.00754245302139e-07	9.82793261631007e-07	1081
CSF2RA	0.157142749695784	2.06992881736453e-07	1.01235047404245e-06	1081
MAMSTR	0.1571326030212	2.07370859468193e-07	1.01395290388028e-06	1081
PRICKLE3	0.157010404345435	2.11975570564856e-07	1.03571376886467e-06	1081
ME2	0.156993270354846	2.12629051967565e-07	1.03865476524893e-06	1081
NASP	0.156953606472849	2.14149279455142e-07	1.04481405537027e-06	1081
DNLZ	0.156904731020938	2.16036995047988e-07	1.0530039354565e-06	1081
VPS28	0.156880988396532	2.1695979090246e-07	1.05724600574022e-06	1081
CD244	0.156873732344392	2.1724256725772e-07	1.05836796067878e-06	1081
RPP25	0.156837703591268	2.18651922861978e-07	1.06497654401706e-06	1081
CUEDC2	0.156818527608433	2.19405633234655e-07	1.0683892872884e-06	1081
RAB37	0.156791044013317	2.20490246601416e-07	1.0734113141096e-06	1081
STXBP2	0.156738472292282	2.22579369571335e-07	1.08305830918226e-06	1081

MED6	0.156722497431503	2.23217961862202e-07	1.08590336675056e-06	1081
EWSR1	0.156660619001876	2.25708268716018e-07	1.0969585219021e-06	1081
LOC7299	0.15661111094698	2.27720011489119e-07	1.10540232083092e-06	1081
TM7SF4	0.156531472100296	2.30992457021622e-07	1.1207473619221e-06	1081
RRP1	0.156483066863011	2.33003600787667e-07	1.13023297324044e-06	1081
SRCRB4D	0.156426127765947	2.35390937822117e-07	1.14126364438665e-06	1081
LOC44095	0.156421411819829	2.35589722372199e-07	1.14195258835129e-06	1081
TWF2	0.15639278653576	2.3679980280388e-07	1.14671442968369e-06	1081
C19orf50	0.156360822440137	2.38158111796394e-07	1.15270067378714e-06	1081
HIST3H2I	0.156359660258669	2.38207639859809e-07	1.15270067378714e-06	1081
SLCO5A1	0.156335197011681	2.39252485928732e-07	1.15747870533965e-06	1081
RPF1	0.156270964245415	2.42017011218271e-07	1.1700102450185e-06	1081
PDXP	0.156222938877668	2.44104091596603e-07	1.17896833498287e-06	1081
TUBB	0.156208748354708	2.44724097566531e-07	1.18139634447107e-06	1081
MAF1	0.156186551613981	2.45696955366736e-07	1.18552459062584e-06	1081
C19orf70	0.156182665094331	2.45867680465317e-07	1.18606427753204e-06	1081
TNF	0.156170530123787	2.46401476231002e-07	1.18822793758198e-06	1081
HLA-DPA	0.156169787744492	2.46434168439687e-07	1.18822793758198e-06	1081
PRKCQ	0.156142425307061	2.47642053657611e-07	1.19319520950313e-06	1081
FTSJ1	0.155984319710825	2.54734091330678e-07	1.22443766877989e-06	1081
SCAMP2	0.155906563239216	2.58293439605661e-07	1.24065840268385e-06	1081
CDC25A	0.155798599543884	2.63315183292419e-07	1.26357417042062e-06	1081
SLC12A3	0.155781960351642	2.64097453706224e-07	1.26672457259807e-06	1081
JMJD4	0.155758979215978	2.65181569392612e-07	1.27131906601956e-06	1081
C11orf21	0.155699199519908	2.68021769257558e-07	1.28371339555242e-06	1081
ASGR2	0.155645191424878	2.7061296401054e-07	1.2955081178689e-06	1081
TRPC2	0.155611803555738	2.72226911172012e-07	1.30230613288059e-06	1081
TM7SF2	0.155601259211556	2.72738543776698e-07	1.30444396115422e-06	1081
TMEM219	0.1555978677567	2.7290330095411e-07	1.30492214519833e-06	1081
RIBC2	0.155512716181515	2.77071574427293e-07	1.32359660109979e-06	1081
VWA5B2	0.155489275800285	2.78229744875769e-07	1.32849922031817e-06	1081
CERKL	0.155444164812861	2.80471795246073e-07	1.33825303534631e-06	1081
KNG1	0.155403255611354	2.82520071005047e-07	1.34675031481228e-06	1081

DNAJB2	0.155386503288015	2.83362996801181e-07	1.35012951053921e-06	1081
LILRA4	0.155324628810159	2.86497404015585e-07	1.36409600659276e-06	1081
BAT5	0.1553196273716	2.86752219876062e-07	1.36498664210852e-06	1081
ERAL1	0.155232776276485	2.9121216108122e-07	1.38523470719744e-06	1081
BCL2L12	0.155221749429274	2.91783170093113e-07	1.38762322037908e-06	1081
DDX28	0.155208819432472	2.92454106223585e-07	1.39015761441107e-06	1081
PKN1	0.155193194582086	2.932668632673e-07	1.3936921350601e-06	1081
MESDC2	0.155148936103716	2.95580894429617e-07	1.40303419375227e-06	1081
FATE1	0.155138858751813	2.96110239026947e-07	1.4052157279618e-06	1081
DLGAP3	0.155129249172727	2.96615863768703e-07	1.4072836965663e-06	1081
RPL36AL	0.155071812423652	2.99655402168711e-07	1.42103532878736e-06	1081
C8orf59	0.155045742988435	3.01044879017777e-07	1.42728855914642e-06	1081
CORO7	0.155017289526802	3.02568512705477e-07	1.43417475022396e-06	1081
TMEM19[illegible]	0.155010966687646	3.0290809813923e-07	1.435446633031e-06	1081
MT1F	0.154990160889763	3.04028126837922e-07	1.44007679641428e-06	1081
DNAJC1	0.154980070895106	3.04572733579898e-07	1.4423172820797e-06	1081
GZMK	0.154951220728374	3.06135114197921e-07	1.44937529389357e-06	1081
NUDCD2	0.154919349255981	3.078700974661e-07	1.45724697214628e-06	1081
PTTG2	0.154838128901415	3.12334451910961e-07	1.47699419432731e-06	1081
ARF1	0.154838112996301	3.12335332233027e-07	1.47699419432731e-06	1081
NPLOC4	0.15476556009428	3.16376010931661e-07	1.49539998597333e-06	1081
C17orf89	0.154704081333877	3.19839343920346e-07	1.51106087787884e-06	1081
LSM12	0.15468519709309	3.20910483350471e-07	1.51505546920441e-06	1081
SFTPB	0.154634073384714	3.23827671950879e-07	1.52804686686454e-06	1081
GTSE1	0.154599220891674	3.25831040218799e-07	1.53648555833514e-06	1081
C9orf95	0.15459666515978	3.25978416413739e-07	1.53682078133742e-06	1081
SLC35A4	0.154589874667021	3.26370302227828e-07	1.53830831501628e-06	1081
SSBP1	0.154572377833609	3.27382155162276e-07	1.54235582688121e-06	1081
CCL18	0.154538831930104	3.29330600054554e-07	1.55117253638041e-06	1081
EHD1	0.154534360672758	3.29591146666625e-07	1.5520368512387e-06	1081
OSGEP	0.154427390822992	3.35884009677691e-07	1.57982334227343e-06	1081
ESPL1	0.15434015775483	3.41101377272889e-07	1.60361429292004e-06	1081
GAL3ST4	0.154335559780791	3.41378536279514e-07	1.60454284025917e-06	1081

SESN2	0.154290153251253	3.44127256877715e-07	1.61686157592938e-06	1081
EIF4E1B	0.154289615095277	3.44159962153645e-07	1.61686157592938e-06	1081
INO80C	0.154233304444941	3.47598730391984e-07	1.63225557663089e-06	1081
HIST1H2I	0.154217062108558	3.48596752938104e-07	1.63579818027908e-06	1081
TMEM8A	0.154099185671223	3.55922971456733e-07	1.66745773488276e-06	1081
UTP18	0.153994786158281	3.62535315214655e-07	1.69725166744114e-06	1081
NHLH1	0.15395482188979	3.65097734527107e-07	1.70885080437932e-06	1081
NAT9	0.153906131659679	3.68243236769968e-07	1.72201724105565e-06	1081
CIR1	0.153905986061855	3.6825268178048e-07	1.72201724105565e-06	1081
LOC1001	0.153904357063275	3.68358371730132e-07	1.72211171930924e-06	1081
HLA-DRA	0.153894913379358	3.68971657202098e-07	1.72457866225899e-06	1081
ZMYND1	0.153882851832468	3.69756381390299e-07	1.72784558179252e-06	1081
NR2C2AP	0.153870444829691	3.70565259067331e-07	1.7312238274377e-06	1081
UBE2L3	0.153808300926948	3.7464247686387e-07	1.74703071676451e-06	1081
FANCI	0.153800383208451	3.75165047517655e-07	1.74865800144451e-06	1081
SMAP2	0.153782497502304	3.76348093898958e-07	1.75376644728071e-06	1081
CCDC56	0.153758771378193	3.77923002636265e-07	1.76029107239481e-06	1081
RAMP1	0.153727068531355	3.80037311811075e-07	1.76931917971031e-06	1081
ARL2	0.153720295689645	3.80490477988841e-07	1.77061230748099e-06	1081
DKFZp76	0.153690412189575	3.82496187762653e-07	1.77953480426759e-06	1081
CEP250	0.153669413724638	3.83911646076081e-07	1.78488347800661e-06	1081
DHRS13	0.153624483990645	3.8695723105563e-07	1.79862792330772e-06	1081
FADS2	0.153618283323517	3.87379370401515e-07	1.80017472127763e-06	1081
KCNH6	0.153534997582443	3.9309262652919e-07	1.82462003719598e-06	1081
GTF3C6	0.153533618278092	3.93187924482728e-07	1.82464195777575e-06	1081
BCAS2	0.15352702828691	3.93643543011872e-07	1.82633559971768e-06	1081
HLA-DQA	0.153508380999056	3.94935536634412e-07	1.8319079865301e-06	1081
CALHM3	0.153056220179793	4.27544449047226e-07	1.97769986821042e-06	1081
OGFOD1	0.15301715805322	4.30480180756464e-07	1.99036567393596e-06	1081
IKZF1	0.153008115227969	4.31162561465654e-07	1.99306328607747e-06	1081
NAPA	0.152992423343472	4.32349160777959e-07	1.99808989306538e-06	1081
DEDD2	0.152958712590562	4.3490897127995e-07	2.00945899688867e-06	1081
PARVB	0.152940585959718	4.36291438559302e-07	2.01353746386647e-06	1081

HIST1H2A	0.152919859327562	4.37877388724036e-07	2.0203939523238e-06	1081
OGFOD2	0.152918209662282	4.38003855326499e-07	2.02051469327967e-06	1081
NOP2	0.152901921173472	4.39254453528426e-07	2.02581981829903e-06	1081
AIM1L	0.15285739617035	4.42690573978479e-07	2.03933263498661e-06	1081
MFSD3	0.152844829069698	4.436650889318e-07	2.04288759234997e-06	1081
NECAB3	0.152822233221842	4.45422483132409e-07	2.0505109512574e-06	1081
C16orf7	0.152796809203881	4.47407855946042e-07	2.05823961133433e-06	1081
WDR34	0.152767997527828	4.49668076841724e-07	2.06722122500606e-06	1081
TRAPPC1	0.152745667997473	4.5142734732545e-07	2.07483548069158e-06	1081
GDF15	0.152728436184398	4.52789516023067e-07	2.08062153291165e-06	1081
FKBP8	0.152672581898876	4.57232047227673e-07	2.09864196659865e-06	1081
PRC1	0.152631328108366	4.60540212837038e-07	2.11286326294742e-06	1081
STARD3	0.152594441025096	4.63517723749971e-07	2.12603928618924e-06	1081
FGR	0.152499074746519	4.71301841990427e-07	2.15764387521533e-06	1081
CDC34	0.152498221072412	4.71372087109589e-07	2.15764387521533e-06	1081
SRP14	0.152491035076293	4.71963791240421e-07	2.15986155714182e-06	1081
ITPK1	0.152466078625338	4.74024299066897e-07	2.16830597291159e-06	1081
CASP4	0.152458019879332	4.74691511158939e-07	2.16906996661695e-06	1081
NUPR1	0.15245349631021	4.75066429727717e-07	2.17011637797389e-06	1081
ROBLD3	0.152431902989836	4.76860042407746e-07	2.17732220179149e-06	1081
PLP2	0.152392257563919	4.80170117316066e-07	2.19094609588497e-06	1081
LHPP	0.152364814714445	4.82474324014539e-07	2.20096133543317e-06	1081
HSBP1	0.152351339193232	4.83609675322123e-07	2.20564113863803e-06	1081
C14orf80	0.152312939910416	4.86859055890013e-07	2.21895377396025e-06	1081
CTPS	0.152288157305806	4.8896733499528e-07	2.22705108828395e-06	1081
SLC29A3	0.152282670416355	4.89435297189949e-07	2.22867858541852e-06	1081
POLR2K	0.152270128699387	4.90506567138858e-07	2.23254740059022e-06	1081
S100A8	0.152197021089355	4.96796256316186e-07	2.2596433920726e-06	1081
RETNLB	0.152191252066288	4.97295877352292e-07	2.26138714035684e-06	1081
PSRC1	0.152190002704555	4.97404140924356e-07	2.26138714035684e-06	1081
NSMCE2	0.152147872000179	5.01068299274814e-07	2.27650448554153e-06	1081
KIF20A	0.152104955517644	5.04827517714129e-07	2.29254967185553e-06	1081
CCDC150	0.15209250135644	5.05923499974746e-07	2.29646719496406e-06	1081

CISD1	0.152077294843858	5.0726479982e-07	2.30102440719971e-06	1081
SLC32A1	0.151947399156276	5.18862741647443e-07	2.35151629482289e-06	1081
RFC2	0.15189541668807	5.23575258994193e-07	2.37180651954981e-06	1081
SH2B2	0.151829066758153	5.29650172511906e-07	2.39716978774486e-06	1081
MRPS16	0.151817286577382	5.30735821612001e-07	2.40154382892492e-06	1081
MUC13	0.15178308948267	5.33899542132937e-07	2.41369081603861e-06	1081
OLR1	0.15176707650616	5.35387206373818e-07	2.41933047833122e-06	1081
TMUB1	0.151760577793559	5.35992098528924e-07	2.42152070528486e-06	1081
TMPRSS4	0.151670576499019	5.44437270351418e-07	2.45705095434707e-06	1081
ELOVL7	0.151670268481114	5.44466391670653e-07	2.45705095434707e-06	1081
C14orf182	0.151644215680513	5.4693496957776e-07	2.46708575059202e-06	1081
C17orf49	0.151550650668174	5.55889676223204e-07	2.50243520167965e-06	1081
TNFAIP2	0.151520923303639	5.58764184219478e-07	2.51481333581353e-06	1081
TMEM160	0.151504983722476	5.60311361303802e-07	2.52065037370815e-06	1081
PADI6	0.151497957553117	5.60994666130025e-07	2.52316087278173e-06	1081
NCRNA00	0.151494538211018	5.61327492091061e-07	2.52409426968179e-06	1081
NDUFV2	0.151473993849951	5.63331209274575e-07	2.53213489699614e-06	1081
SMARCD2	0.151420580209159	5.68572985346473e-07	2.55268615774564e-06	1081
LOC39981	0.151385965059212	5.71995003130542e-07	2.5663339282995e-06	1081
C21orf67	0.15136774114993	5.73804558316305e-07	2.57330650651869e-06	1081
HIST1H2E	0.151315096369513	5.79062965487606e-07	2.59515538147894e-06	1081
PFDN1	0.151311839834126	5.79389762523652e-07	2.59604243016881e-06	1081
PARP2	0.151250648483159	5.85563497286901e-07	2.62195524624241e-06	1081
MARCO	0.151221710653396	5.88505109430522e-07	2.63341205824669e-06	1081
MYPOP	0.151221620210651	5.88514325451805e-07	2.63341205824669e-06	1081
HSD17B3	0.151164972972958	5.94313996400516e-07	2.65759277635702e-06	1081
BTNL8	0.15112827734268	5.98100298555273e-07	2.6727441247551e-06	1081
PA2G4	0.151110066294083	5.99987948356526e-07	2.68058487905128e-06	1081
TNFRSF1	0.151073368359183	6.03809276544553e-07	2.69632928474416e-06	1081
TNIP1	0.15096604269594	6.15119935620806e-07	2.74453845029483e-06	1081
TRPT1	0.150958644671404	6.15907054294774e-07	2.74744189742432e-06	1081
MYL4	0.15091991081741	6.20044024548764e-07	2.76344842356966e-06	1081
CD69	0.150893014738399	6.22932380755706e-07	2.775093500735e-06	1081

TMEM50A	0.150868110946708	6.25618329782698e-07	2.78582697910532e-06	1081
C2orf28	0.150853554717718	6.27193420972893e-07	2.79037355686814e-06	1081
PSORS1C	0.150813996767648	6.31493186340667e-07	2.80764295714693e-06	1081
ACTR1A	0.150784284562234	6.3474140787157e-07	2.82021739337732e-06	1081
ICAM1	0.150766920945573	6.36647085260486e-07	2.82806075690683e-06	1081
C8orf73	0.150727993216272	6.40939480829414e-07	2.84650040593222e-06	1081
FAM72B	0.150715969573082	6.42270897482447e-07	2.85178471011327e-06	1081
C16orf54	0.150699207558545	6.44131451924578e-07	2.85941562340693e-06	1081
SCAMP3	0.150532267373649	6.6294684509548e-07	2.93711550350747e-06	1081
RRN3P2	0.1504931169605	6.67435346686685e-07	2.95440234212333e-06	1081
NUP37	0.150442434329342	6.73289439413526e-07	2.97900631605216e-06	1081
LLGL2	0.150368642973151	6.81901121404928e-07	3.01512249576433e-06	1081
SPATA2L	0.150360029225849	6.82913253794532e-07	3.0182728165184e-06	1081
PTPRC	0.150349162881526	6.84192133000981e-07	3.02326179410063e-06	1081
AP4B1	0.150308903305484	6.88950469343966e-07	3.04362000108206e-06	1081
DOCK2	0.150263998047247	6.94295451353481e-07	3.06656037437314e-06	1081
TMEM222	0.15026003269733	6.94769351474045e-07	3.06798083854551e-06	1081
INSM1	0.150232444451417	6.9807505677959e-07	3.08190269972054e-06	1081
ATP5D	0.150227698601152	6.98645242120901e-07	3.0837441723325e-06	1081
FADS1	0.15020422821898	7.01471661593415e-07	3.09486347411286e-06	1081
TECR	0.150172424448606	7.05319189128132e-07	3.11115722903136e-06	1081
DNASE1L	0.150121902695755	7.11472943971389e-07	3.13515147840492e-06	1081
ASCL2	0.150093035831274	7.1501220158856e-07	3.14908631416737e-06	1081
LOC38933	0.15000894195993	7.25419451747839e-07	3.19143368758686e-06	1081
TRAPPC2	0.149995807239986	7.27058075128261e-07	3.19794430643206e-06	1081
DNAJA2	0.149971133377703	7.30145898609182e-07	3.21082495688321e-06	1081
RAPGEFL	0.149956541068924	7.31977993917639e-07	3.21817911118962e-06	1081
ARL6IP4	0.149937518744905	7.34372928587122e-07	3.2267363943602e-06	1081
DPM2	0.149937265343214	7.34404883005287e-07	3.2267363943602e-06	1081
DEPDC1B	0.149901718261816	7.38900715318767e-07	3.24578170739131e-06	1081
CNTD2	0.149848467992122	7.45685141978759e-07	3.27415588168311e-06	1081
MTCH2	0.149842367828773	7.46466156937896e-07	3.27687093735322e-06	1081
PRPF6	0.149727951986596	7.6126165775092e-07	3.33673109125159e-06	1081

FBXL15	0.149719755522808	7.6233233017404e-07	3.34069714843507e-06	1081
BFSP2	0.149717334152306	7.62648901531649e-07	3.34135757315247e-06	1081
CD40	0.14969917621131	7.65026914348161e-07	3.35104744282316e-06	1081
SDHAF1	0.149666710431249	7.69296509357194e-07	3.36682124288522e-06	1081
GDPD2	0.149494131109359	7.92380069814056e-07	3.46182964788639e-06	1081
TIMM17A	0.149464851867432	7.96361881670823e-07	3.47847140205089e-06	1081
UQCRFS1	0.149452734193054	7.98015441790366e-07	3.48493845108756e-06	1081
SIGLEC16	0.149389391531797	8.06712980057725e-07	3.52215712684501e-06	1081
C5orf32	0.149387665511274	8.0695125017847e-07	3.52243400538359e-06	1081
EXOC3L	0.149385612428366	8.07234758076427e-07	3.52290818922219e-06	1081
LRRC61	0.149382427801527	8.07674709159674e-07	3.52406476413724e-06	1081
JAKMIP2	0.149380944566989	8.0787969433988e-07	3.52419585155411e-06	1081
HIST1H3A	0.149334706281285	8.14295045692108e-07	3.54987528575363e-06	1081
HES6	0.149322719727565	8.15966105934199e-07	3.55639056772921e-06	1081
CD177	0.149307712485203	8.1806293423017e-07	3.56244656508145e-06	1081
GNAS	0.149251363217577	8.2598247321676e-07	3.59615667234745e-06	1081
CDH15	0.14924704417092	8.26592521370819e-07	3.59803507843888e-06	1081
FCAR	0.149241140088311	8.27427150641757e-07	3.59974634571376e-06	1081
DBNDD1	0.149240471378163	8.27521733780115e-07	3.59974634571376e-06	1081
TIPIN	0.149199204795538	8.33378690178509e-07	3.62287736591413e-06	1081
NDUFAF2	0.149120968848101	8.44592323216557e-07	3.66687766189602e-06	1081
MTP18	0.149109916936949	8.46188054066486e-07	3.67301408083804e-06	1081
DBNL	0.149066487319595	8.5248677045006e-07	3.69876071305545e-06	1081
CAPNS1	0.149056203621532	8.53984832305958e-07	3.70446262851066e-06	1081
FAM195B	0.149003874856381	8.61647045091745e-07	3.73528722258946e-06	1081
UQCRB	0.148921519631486	8.73839928938975e-07	3.78570007924207e-06	1081
RILP	0.148873805951316	8.80979779902775e-07	3.81417100067513e-06	1081
EZH2	0.148799383993817	8.92228351673662e-07	3.85872480559595e-06	1081
MEF2B	0.148775238016288	8.95907496267953e-07	3.87380478907875e-06	1081
MED25	0.148772268591379	8.9636095626482e-07	3.87493379054824e-06	1081
NPEPL1	0.148760004982247	8.98236067300338e-07	3.88220673155231e-06	1081
TOR1B	0.148742360761095	9.00940494476443e-07	3.89222523294616e-06	1081
ZFAND2E	0.148720571601733	9.04291044491127e-07	3.90502531431377e-06	1081

P2RY10	0.148714724885537	9.05192139133875e-07	3.90724140622496e-06	1081
CPSF3	0.14871304832082	9.05450690167445e-07	3.90752016997069e-06	1081
AIPL1	0.148704019262608	9.06844325645083e-07	3.91269628188482e-06	1081
TMEM125	0.148670045947206	9.12106611258425e-07	3.93287407615603e-06	1081
VPS16	0.148666659635392	9.12632739108923e-07	3.93430056266836e-06	1081
KIR2DL1	0.148552719621288	9.30506553994799e-07	4.0079227133259e-06	1081
IL18	0.148503991627485	9.38252850991927e-07	4.03956052216978e-06	1081
SSNA1	0.148500789044898	9.38764131501867e-07	4.0408981686122e-06	1081
MTVR2	0.148483185010622	9.41579337139726e-07	4.04955502704521e-06	1081
PLAC8L1	0.148437669750063	9.4889572824647e-07	4.07927965120041e-06	1081
C14orf179	0.148396164954356	9.55615073573989e-07	4.10601793667035e-06	1081
C16orf42	0.148369317629331	9.59985812631562e-07	4.12255685258214e-06	1081
BCMO1	0.148346370236793	9.63736871088548e-07	4.13602029571342e-06	1081
ALG1L2	0.148338260460878	9.65065889146093e-07	4.14084181828499e-06	1081
HOXB13	0.148315594649023	9.68789678954836e-07	4.15504962370559e-06	1081
PUSL1	0.14828035596155	9.74606524836119e-07	4.17821843856642e-06	1081
CAMK2N	0.148259208480468	9.78113441759523e-07	4.19236069877566e-06	1081
MYH7	0.14818767462668	9.90066143522228e-07	4.23998351642332e-06	1081
C2orf48	0.148141091377831	9.97925164887457e-07	4.27091617732267e-06	1081
LOC10012	0.148121894404652	1.00118128827601e-06	4.2839415998981e-06	1081
DHRS4	0.148061870730959	1.01142831315394e-06	4.32503149405354e-06	1081
WNT10B	0.148031614074804	1.01663176178595e-06	4.34359423991048e-06	1081
HDDC3	0.148021360811323	1.01840091727084e-06	4.35023038134459e-06	1081
ANK1	0.147989570198626	1.02390507913106e-06	4.37281488638863e-06	1081
NOL3	0.14792099657185	1.03587530276688e-06	4.42299872281448e-06	1081
LEMD2	0.147889182634226	1.04147431725755e-06	4.44596315345484e-06	1081
NANS	0.147875258887106	1.0439339328999e-06	4.4536317404211e-06	1081
DHPS	0.147840566139474	1.05008668077436e-06	4.4774532986347e-06	1081
TMEM63I	0.14779704881518	1.05785375447535e-06	4.50824283560525e-06	1081
GINS1	0.14773679349577	1.06869947272446e-06	4.55157523848502e-06	1081
LOXL3	0.147723481233021	1.07110999959835e-06	4.56087739207542e-06	1081
SIRPD	0.147703870264317	1.07467059005238e-06	4.57507164763425e-06	1081
EXO1	0.147623957014937	1.08929770559717e-06	4.63636219718042e-06	1081

PRR7	0.147599763758659	1.09376357717299e-06	4.65438683188631e-06	1081
KLHL6	0.147579659135194	1.09748810257373e-06	4.66778576390244e-06	1081
SLC25A1	0.147552907141307	1.10246300419132e-06	4.68744559295781e-06	1081
DCAF15	0.147538856906747	1.10508451132635e-06	4.6976002280374e-06	1081
C5orf20	0.147476345544781	1.11682073560403e-06	4.74448623339166e-06	1081
SNX17	0.147445740816519	1.12261021837806e-06	4.76807565870252e-06	1081
HBD	0.147436596959132	1.12434554587485e-06	4.77154231024905e-06	1081
CCHCR1	0.147408931726406	1.12961158406023e-06	4.79175097091877e-06	1081
MCRS1	0.147374429189586	1.13621230933707e-06	4.8177219472943e-06	1081
TBCD	0.14737239850336	1.1366019552265e-06	4.81835993014265e-06	1081
DNAJC8	0.147283092014095	1.15386555286178e-06	4.8843499815929e-06	1081
GPR132	0.147249939222853	1.16033813085541e-06	4.91071673237023e-06	1081
TREML3	0.147216661720887	1.1668701323413e-06	4.9352506437152e-06	1081
SNRPD2	0.147213882300215	1.16741729735592e-06	4.93652843308878e-06	1081
C6orf26	0.147204018236407	1.16936115691868e-06	4.94371049446523e-06	1081
ANP32B	0.147185670284423	1.17298516741529e-06	4.95799122903505e-06	1081
VRK1	0.147105813289404	1.18888426375588e-06	5.02097976800046e-06	1081
C17orf67	0.147102886648373	1.18947085754961e-06	5.0224041973039e-06	1081
MGC1291	0.147071762744319	1.19572632526156e-06	5.046701617933e-06	1081
C16orf79	0.147069357798845	1.19621099879859e-06	5.04768968805981e-06	1081
RCC1	0.146964178550078	1.21759360294119e-06	5.13146927432014e-06	1081
RABGGT1	0.146945930616057	1.22134053570662e-06	5.14618387195355e-06	1081
MAP7D1	0.146937162151313	1.22314494307278e-06	5.15163179556788e-06	1081
RUSC1	0.146933649612468	1.22386848462467e-06	5.15360171880519e-06	1081
ATG9B	0.146927371299224	1.2251627674823e-06	5.15797365745684e-06	1081
FBXO2	0.146882185135929	1.23451682173139e-06	5.19518307369517e-06	1081
ESRRA	0.146801733645499	1.25134128336486e-06	5.26268687962111e-06	1081
CHPF	0.146763556148463	1.25940202636443e-06	5.29437684079953e-06	1081
NAA38	0.146738466548104	1.26472654533885e-06	5.31454239796645e-06	1081
CALCOC(	0.146668662085838	1.27965447732497e-06	5.37278854641759e-06	1081
CCNA2	0.146666080859028	1.28020971410668e-06	5.37399972716795e-06	1081
MAP2K2	0.146656922342156	1.28218163174534e-06	5.38115603573122e-06	1081
ARTN	0.146566685449718	1.30176714281529e-06	5.45994151405664e-06	1081

ADCK1	0.146515190930147	1.3130722442892e-06	5.50621156561324e-06	1081
9-Mar	0.14644587461524	1.32843892853944e-06	5.568331713572e-06	1081
USF2	0.146442143305892	1.32927100021266e-06	5.57066034934137e-06	1081
SDF4	0.14642990094618	1.3320045294676e-06	5.57979439511851e-06	1081
C6orf47	0.146387017509821	1.34162237906255e-06	5.61657997219766e-06	1081
KIAA0748	0.146382844063776	1.34256194828021e-06	5.61934561564615e-06	1081
TYMS	0.146317700959851	1.35730980860572e-06	5.67635584271584e-06	1081
RBM4	0.146113675555466	1.40451300315861e-06	5.86888912023027e-06	1081
NCLN	0.146037304238161	1.42258450105352e-06	5.94070579886468e-06	1081
FAM136A	0.146026915403004	1.42505996296947e-06	5.94980993865699e-06	1081
PAOX	0.145980957700357	1.43606052033863e-06	5.99325444007081e-06	1081
UQCRC1	0.145966265068116	1.43959455627472e-06	6.00675897600545e-06	1081
CKS1B	0.145883355963083	1.45969383513199e-06	6.08810192727412e-06	1081
TOP1P2	0.145845799214914	1.46888696690598e-06	6.12137524789427e-06	1081
NHLRC1	0.145834908087207	1.47156327595334e-06	6.13126001945811e-06	1081
POLR2F	0.145805640216702	1.47877857352615e-06	6.1575019354453e-06	1081
NOC2L	0.145744573874636	1.49394255901268e-06	6.21935789446381e-06	1081
GIT1	0.145743204299924	1.49428435744469e-06	6.2194955332073e-06	1081
VNN2	0.145680553019761	1.51000047500286e-06	6.28231300473618e-06	1081
ITGA2B	0.145625045039904	1.52405717536907e-06	6.33556165864834e-06	1081
HMGB2	0.145561683396371	1.54025627236612e-06	6.3989405252249e-06	1081
CEL	0.145491945918435	1.558276488409e-06	6.46846895920035e-06	1081
HLX	0.145377717917841	1.58823114811314e-06	6.58873897832358e-06	1081
PNP	0.145357043371908	1.59371150188456e-06	6.61011286915061e-06	1081
STOML1	0.145288500548867	1.61201080573132e-06	6.67776222115101e-06	1081
STIL	0.145256467223159	1.62063193808601e-06	6.71071539419171e-06	1081
TMEM70	0.145193396808142	1.63773561460628e-06	6.77596713005617e-06	1081
CHIT1	0.145159851518239	1.64690300210838e-06	6.8083030940844e-06	1081
TLR2	0.145112720905778	1.65986629371004e-06	6.85907825370026e-06	1081
C1orf170	0.145100427066358	1.66326379036268e-06	6.87029917097727e-06	1081
THAP7	0.145082620441846	1.66819662515635e-06	6.8892622004458e-06	1081
PITX1	0.145065176891065	1.67304249358495e-06	6.90785837943612e-06	1081
MVK	0.145047045535372	1.67809375530827e-06	6.92729481571417e-06	1081

DGCR6L	0.145013546287398	1.68746492911801e-06	6.95884974351737e-06	1081
RDH14	0.144906495204372	1.71774960348575e-06	7.07939152254921e-06	1081
OST4	0.144894721434781	1.72111205725345e-06	7.08889846521584e-06	1081
CCL1	0.144855714815834	1.73229709031016e-06	7.13205086537876e-06	1081
SPIC	0.144775177943418	1.75561191592901e-06	7.2236115290829e-06	1081
TREML1	0.144674446491342	1.78519699220675e-06	7.3392648749031e-06	1081
RNASET2	0.144654362940776	1.79115251040476e-06	7.36082564710402e-06	1081
FAM89B	0.144641738024056	1.7949060211849e-06	7.3747464402957e-06	1081
HIST1H2I	0.144563013567135	1.81848240472848e-06	7.46400326879691e-06	1081
DENND3	0.144559540158788	1.81952943527264e-06	7.46677948127261e-06	1081
RAB39	0.144448278981872	1.85337591431632e-06	7.59639019204522e-06	1081
ATF4	0.144373716111966	1.87639569307547e-06	7.6860494585208e-06	1081
CENPBD1	0.144333195613121	1.88902037017431e-06	7.73461694251248e-06	1081
GIMAP1	0.144294713437732	1.90108540303083e-06	7.78085441772777e-06	1081
AGAP2	0.14428502626142	1.90413418004637e-06	7.79174955454686e-06	1081
SNRNP35	0.144172716795183	1.93982476036523e-06	7.92491782550345e-06	1081
VGF	0.144154181093757	1.94577654351346e-06	7.94601023090992e-06	1081
CYB561	0.144152593595763	1.9462871001698e-06	7.94648431960287e-06	1081
PYCARD	0.144145399672407	1.94860235558863e-06	7.95432511719007e-06	1081
TTC24	0.144088967809295	1.96685597757789e-06	8.02395983562306e-06	1081
MBOAT7	0.144084287867126	1.96837710979963e-06	8.02853955799018e-06	1081
NOP16	0.14406000689152	1.97628734501657e-06	8.05917177436412e-06	1081
HMMR	0.144041355828128	1.98238417170226e-06	8.08239812567135e-06	1081
CALM3	0.143873248518423	2.03815688136715e-06	8.29635691556702e-06	1081
PNPLA1	0.14382840046741	2.05328851497187e-06	8.35626204729462e-06	1081
KIF23	0.143801251126927	2.06250094013836e-06	8.39205846073264e-06	1081
CCNK	0.143793675970188	2.0650784392253e-06	8.40084918380324e-06	1081
C21orf29	0.143790366644092	2.0662054295885e-06	8.40373680174852e-06	1081
DCI	0.143759682331651	2.07668309793499e-06	8.44123910571034e-06	1081
ALDH16A	0.143733746776738	2.08557894829909e-06	8.47568850382996e-06	1081
C2orf70	0.143681676742734	2.10354935822301e-06	8.54182661185295e-06	1081
CD47	0.143668222062002	2.10821692770863e-06	8.5582542585365e-06	1081
FAM131C	0.143556882890871	2.14722470814443e-06	8.70689246086343e-06	1081

OLIG1	0.143555339335456	2.14777032842939e-06	8.7073522371121e-06	1081
EIF5AL1	0.143537732053368	2.15400361647474e-06	8.73086576536894e-06	1081
ZNF622	0.143492017361782	2.17026852716924e-06	8.79325411903142e-06	1081
JUND	0.143489997987062	2.17098971365323e-06	8.79440735603143e-06	1081
RCCD1	0.143434205580033	2.1910061940655e-06	8.87112708824396e-06	1081
PAF1	0.143433529425563	2.19124985808399e-06	8.87112708824396e-06	1081
COL29A1	0.143400800242799	2.20307551117793e-06	8.91721040238685e-06	1081
SPC24	0.143382484747896	2.20971991743243e-06	8.93692185036665e-06	1081
UBA1	0.143298996696861	2.24025152408635e-06	9.05676639629128e-06	1081
PA2G4P4	0.143278498919133	2.24780918931097e-06	9.08367424647332e-06	1081
LSMD1	0.143231902101397	2.26508081070141e-06	9.14796570400561e-06	1081
ECHS1	0.143151707643018	2.29510414270721e-06	9.26364915945438e-06	1081
FAM71E1	0.14314591933217	2.29728588192189e-06	9.26986895622407e-06	1081
E2F8	0.143144802451227	2.2977070872568e-06	9.26986895622407e-06	1081
RTCD1	0.14314395802248	2.2980255928212e-06	9.26986895622407e-06	1081
USP5	0.143120136121057	2.30702831122949e-06	9.29873656056786e-06	1081
PPIH	0.143089241095717	2.31875446750006e-06	9.34226174955776e-06	1081
CBS	0.143067362528241	2.32709298331529e-06	9.3721087862628e-06	1081
HCCS	0.143049748961141	2.33382687462234e-06	9.39547210017326e-06	1081
SLCO4A1	0.142983266448549	2.35941272698015e-06	9.49278397943182e-06	1081
FAM195A	0.142971062879536	2.36413840230891e-06	9.50989778644428e-06	1081
CINP	0.142968510890648	2.36512777384778e-06	9.51197824000071e-06	1081
TMEM229	0.142967096463919	2.36567629890845e-06	9.51228523782651e-06	1081
YIPF3	0.142958293407375	2.36909293159978e-06	9.52254146528419e-06	1081
HLA-DOE	0.142958088821778	2.36917239136284e-06	9.52254146528419e-06	1081
HIST1H2I	0.14292499779318	2.38205842563548e-06	9.56860757416284e-06	1081
FAU	0.142922340534363	2.38309610289439e-06	9.57086742280851e-06	1081
NTSR1	0.142891294069317	2.39525211108748e-06	9.61585368231513e-06	1081
C20orf46	0.142875548299922	2.4014399461722e-06	9.63877420116338e-06	1081
UQCRQ	0.142797129576338	2.43248616634858e-06	9.75561095383079e-06	1081
C1orf35	0.142763378236637	2.44596654596441e-06	9.80381935305473e-06	1081
DHRS11	0.142644064827266	2.49419706312585e-06	9.98124748443986e-06	1081
GPR183	0.142640671778621	2.49558189190141e-06	9.98331097407326e-06	1081

CGA	0.142640373047675	2.49570385035656e-06	9.98331097407326e-06	1081
UBE2V1	0.142543893387231	2.53539249900791e-06	1.01360353031384e-05	1081
IL12RB2	0.142522088311773	2.54444586156573e-06	1.01702106907226e-05	1081
HIST2H2I	0.142519008915867	2.54572690633425e-06	1.017331254277e-05	1081
GCHFR	0.142491485570883	2.55720425084357e-06	1.02131006410079e-05	1081
APBB1IP	0.142465756632394	2.56797816230547e-06	1.02500336991567e-05	1081
C8orf38	0.142443490962288	2.57733694712949e-06	1.02833140819813e-05	1081
C21orf59	0.142363701231976	2.6111436626407e-06	1.04114119579123e-05	1081
PPP1R14E	0.142314775681149	2.63208307086722e-06	1.04851321856081e-05	1081
COPS7A	0.142199830003521	2.68191320518224e-06	1.06773006953352e-05	1081
GTF3C5	0.142167277664846	2.69618845293655e-06	1.07256548942352e-05	1081
HLA-DQE	0.142108780953579	2.72202434322573e-06	1.08183396819101e-05	1081
HYAL3	0.142091993139762	2.7294826221734e-06	1.08431132762144e-05	1081
GPR56	0.142057182590915	2.74501026973147e-06	1.09026482420624e-05	1081
C9orf139	0.142055802965873	2.74562740806455e-06	1.09029497605875e-05	1081
CPLX3	0.142028026505066	2.75808078743745e-06	1.09459293662189e-05	1081
BATF	0.142024033470832	2.7598754799262e-06	1.09508945328173e-05	1081
FBXW5	0.142022112489629	2.76073927475376e-06	1.09521647676082e-05	1081
NLRC3	0.141965574383667	2.78627872439861e-06	1.10491308864193e-05	1081
ECH1	0.141860065542805	2.83454599849734e-06	1.1227276669235e-05	1081
RFNG	0.141853367999467	2.83763682448703e-06	1.12373095791805e-05	1081
PIGP	0.141848746452048	2.83977148909197e-06	1.12435528002669e-05	1081
CHN1	0.141738756215212	2.89103175617893e-06	1.14285389969043e-05	1081
C6orf150	0.141674417238258	2.92142626943114e-06	1.15418968812885e-05	1081
GUCY2E	0.141666214159301	2.92532343856589e-06	1.15550275823353e-05	1081
C1orf228	0.141555547226532	2.97838909267854e-06	1.17600251415149e-05	1081
MAGEA1	0.141544352413989	2.98380816466512e-06	1.17768055401996e-05	1081
NGRN	0.141426088556252	3.04163615350607e-06	1.1990951137452e-05	1081
CARD9	0.14140682961373	3.0511543584554e-06	1.20237684959085e-05	1081
MTERFD1	0.141402454335663	3.05332069383354e-06	1.20299521567136e-05	1081
HS3ST1	0.141271394003157	3.11890010726197e-06	1.22667400743445e-05	1081
TMEM20	0.141211666097163	3.14923206985724e-06	1.23812021950184e-05	1081
TRAPPC4	0.141105884434915	3.20364641833675e-06	1.25853075462937e-05	1081

MRPL13	0.141080788604311	3.21668731961235e-06	1.26340740989648e-05	1081
EMG1	0.141050321035222	3.23258795370832e-06	1.26891045065187e-05	1081
AKT1S1	0.140972296173843	3.27365211497263e-06	1.28452905835847e-05	1081
FMR1	0.140891643001898	3.31662445979376e-06	1.30032040145195e-05	1081
ELMO3	0.140890711104261	3.31712412164883e-06	1.30032040145195e-05	1081
PNPLA3	0.140871616189914	3.32737825759796e-06	1.30383262009942e-05	1081
LGALS1	0.140851546362137	3.33818864938596e-06	1.30755999109236e-05	1081
HSPBP1	0.140844720543507	3.34187296052416e-06	1.30874865454431e-05	1081
LOC40101	0.140842503059721	3.34307070975099e-06	1.30896325457597e-05	1081
ARPC4	0.140789320875275	3.37191994594075e-06	1.31923339116288e-05	1081
CCL26	0.140745064641802	3.39610880775578e-06	1.32792336825e-05	1081
RAB1B	0.140739653108789	3.39907793294678e-06	1.32882641100743e-05	1081
MRPL54	0.140708334354143	3.41631035816206e-06	1.33530407771003e-05	1081
OS9	0.14069947254345	3.42120153094173e-06	1.33695644696064e-05	1081
SLC38A1(	0.140672420176455	3.43617428391797e-06	1.34228681306045e-05	1081
KRT19	0.140653423384476	3.44672597502461e-06	1.34614763022239e-05	1081
AIFM3	0.14064516995333	3.45131997194625e-06	1.34741939602436e-05	1081
PPP4C	0.140625331669064	3.46238628722281e-06	1.35095432415463e-05	1081
VPS25	0.140609861634171	3.47103944864987e-06	1.35406835191812e-05	1081
SLC25A5	0.140594526597345	3.47963753421799e-06	1.35689698271044e-05	1081
KDELR1	0.140517856792645	3.52293170581058e-06	1.37271681264128e-05	1081
GSDMB	0.140501386339488	3.5322993665096e-06	1.37583470105058e-05	1081
TUBB2C	0.140469703815694	3.5503860832594e-06	1.38248888735475e-05	1081
NT5E	0.14041481355524	3.58193157013832e-06	1.39354985477861e-05	1081
C8orf55	0.140374580070319	3.6052240460249e-06	1.4012586997332e-05	1081
HIST1H4I	0.140357554292631	3.61512443031554e-06	1.40456473767997e-05	1081
KIR3DX1	0.140325608162889	3.63377119157848e-06	1.411537228198e-05	1081
ACAT2	0.140324077534385	3.63466691781941e-06	1.41161297589111e-05	1081
LCNL1	0.140298797989248	3.64949115947872e-06	1.41674548656083e-05	1081
MYL12A	0.140297945470142	3.64999209493705e-06	1.41674548656083e-05	1081
C9orf169	0.140278896256344	3.66120249070926e-06	1.4207420649498e-05	1081
SPRYD4	0.140253828779875	3.67600485103749e-06	1.42574350643339e-05	1081
PIH1D1	0.140243817884041	3.68193228606675e-06	1.42776758234485e-05	1081

METTL1	0.140175329303365	3.72273038589182e-06	1.44303258848934e-05	1081
PRKAG1	0.14015094995327	3.73735713908058e-06	1.44842361613656e-05	1081
B3GALT4	0.140121698450947	3.75497962373421e-06	1.45497335103146e-05	1081
SNX8	0.140084545201345	3.77747719371228e-06	1.46312782286741e-05	1081
CIB3	0.140021311098874	3.81606461114714e-06	1.476087204139e-05	1081
CCDC59	0.140018946444862	3.81751489049996e-06	1.47636470472493e-05	1081
HEXDC	0.140002446142706	3.82764947974044e-06	1.47991484489597e-05	1081
SLC4A1A	0.13999271510098	3.83363841427749e-06	1.48174685064505e-05	1081
FLAD1	0.139988435159296	3.83627532813366e-06	1.48248161299161e-05	1081
GTPBP5	0.139972399197157	3.84617068833078e-06	1.48602049321871e-05	1081
TMEM151	0.139963201316383	3.85185747371674e-06	1.48793228778569e-05	1081
C12orf73	0.139934452113226	3.86968421927793e-06	1.49453198997994e-05	1081
CD5L	0.139918917069755	3.87934999519584e-06	1.49769079442737e-05	1081
UROD	0.13985280314228	3.92074459978253e-06	1.51251244662235e-05	1081
POLR2J	0.139703554902579	4.01575048953149e-06	1.54649767944201e-05	1081
C21orf58	0.139693765634296	4.0220583697904e-06	1.54833491036552e-05	1081
ACSF3	0.139618233729563	4.07104848694593e-06	1.56569820102187e-05	1081
CHCHD6	0.139604444089435	4.08005389937566e-06	1.56826341925058e-05	1081
PBK	0.139589154178346	4.09006136111034e-06	1.57151032080045e-05	1081
POLR3K	0.139578830397047	4.09683170168087e-06	1.57381149180704e-05	1081
TTC1	0.139520019799413	4.13560475941031e-06	1.58840339138838e-05	1081
PLOD3	0.139514279124357	4.13940826730138e-06	1.58956118080035e-05	1081
XKR8	0.139492961615887	4.15356158714311e-06	1.59469217024963e-05	1081
NFKBIA	0.13946885344104	4.16962351153557e-06	1.60024891674384e-05	1081
SLC5A5	0.139453091375847	4.18015700682247e-06	1.60368049709462e-05	1081
PTS	0.13944659182928	4.1845079439203e-06	1.60504403142183e-05	1081
YKT6	0.139429628553116	4.19588395236682e-06	1.60910112736397e-05	1081
HMGN1	0.139399414191079	4.21621981910124e-06	1.61581386249966e-05	1081
GPR143	0.139333749487907	4.26074135124175e-06	1.63206797286787e-05	1081
IRF4	0.139332719444933	4.26144330468355e-06	1.63206797286787e-05	1081
MAP6D1	0.139306950384013	4.27904045444603e-06	1.63787326533945e-05	1081
TAZ	0.139267244380688	4.30629102352899e-06	1.64736484369524e-05	1081
PLA2G1B	0.139165324994868	4.37700140590292e-06	1.67314408580482e-05	1081

LYL1	0.139154051882854	4.38489043334122e-06	1.67584173363041e-05	1081
TNFRSF2:	0.139113800227775	4.41317006882204e-06	1.68473206436271e-05	1081
CD22	0.139078666349814	4.43799665649576e-06	1.69388864428016e-05	1081
RHBDD2	0.138988716382919	4.50216827615527e-06	1.71675525124262e-05	1081
MAP3K10	0.138966680384303	4.51802392254871e-06	1.72214932676904e-05	1081
MCM2	0.138913902413558	4.55621694264006e-06	1.73637893131827e-05	1081
CISD3	0.138890147319826	4.57350804164954e-06	1.74197994893231e-05	1081
THAP4	0.138870809795651	4.5876299671855e-06	1.74702846292915e-05	1081
LOC64338	0.138852045016742	4.6013734969949e-06	1.7515999451429e-05	1081
PTMA	0.138836961789568	4.61244916223939e-06	1.75548438264335e-05	1081
EIF1AD	0.138801218219971	4.63879770376866e-06	1.7651790657805e-05	1081
TRAIP	0.13878836093061	4.64831067821228e-06	1.76846494074762e-05	1081
RAET1K	0.138782594062528	4.65258358345398e-06	1.76975634986179e-05	1081
PTRH1	0.138752869895397	4.67466707669078e-06	1.77728823049203e-05	1081
PPP1R16A	0.138671996249565	4.7352606179607e-06	1.79882755324945e-05	1081
RHEB	0.138651420341532	4.75079631942961e-06	1.80438898670644e-05	1081
DENND2I	0.138637356348221	4.76144328096096e-06	1.80777740550144e-05	1081
ACCN5	0.138594171864718	4.79427834401338e-06	1.81918887248351e-05	1081
COMMD1	0.138512374606505	4.85706694753799e-06	1.84058716437458e-05	1081
IL1F5	0.138509908189779	4.85897236122273e-06	1.8409629154943e-05	1081
C9orf167	0.138457075301535	4.89996014713286e-06	1.85544543541337e-05	1081
GFI1	0.13845403978265	4.90232512195974e-06	1.85599209888891e-05	1081
FGL2	0.138442712662509	4.91115972988318e-06	1.85898746257979e-05	1081
NUDT14	0.138410747551135	4.93617316681245e-06	1.86775372737485e-05	1081
SIGIRR	0.138392967289255	4.95013931873151e-06	1.8723348962795e-05	1081
UBE2J2	0.138248390348398	5.06511392565363e-06	1.91294938193274e-05	1081
WBSCR27	0.138240623279054	5.07136240932749e-06	1.91495024809564e-05	1081
PIGT	0.13823648294863	5.07469625053888e-06	1.91584999938354e-05	1081
ORMDL1	0.138204644282927	5.10040322271119e-06	1.92447317702785e-05	1081
AKIRIN2	0.138202465518666	5.10216693150874e-06	1.92477814298209e-05	1081
RRAS	0.138185514400637	5.11590878830455e-06	1.9296008713798e-05	1081
TXNL4A	0.138135010771079	5.15706096355108e-06	1.94366684959283e-05	1081
P2RX4	0.138115177359316	5.17330834489631e-06	1.94931276993281e-05	1081

GPR35	0.138114363176875	5.17397636179238e-06	1.94931276993281e-05	1081
RAB3A	0.138098547354323	5.18696923453825e-06	1.95347719629413e-05	1081
MRPL32	0.138095727289867	5.1892892235927e-06	1.95398563381822e-05	1081
DKFZP43	0.138042808126851	5.23300880107519e-06	1.9690465302792e-05	1081
ATP6V1E	0.138042581381404	5.23319688414734e-06	1.9690465302792e-05	1081
KCNH4	0.137923660871357	5.33273601358166e-06	2.00500125034719e-05	1081
PPP1R2P9	0.137892779566988	5.35887892860787e-06	2.01445448809117e-05	1081
TMEM86I	0.137852501260458	5.39316115996477e-06	2.02620722803973e-05	1081
SLC18A1	0.137839988712048	5.40385363065062e-06	2.02908912189109e-05	1081
LSM3	0.137789780586262	5.44696264904368e-06	2.04451392891718e-05	1081
CLPTM1	0.137771247795432	5.46295794361506e-06	2.0497539164486e-05	1081
CPA6	0.137763208897839	5.46991011601047e-06	2.05159819934893e-05	1081
C1R	0.137754824420343	5.47717016268086e-06	2.05393881100532e-05	1081
ST3GAL5	0.137750014759384	5.48133896350984e-06	2.05511954997033e-05	1081
MCTS1	0.137647612344736	5.57082000160332e-06	2.08677075029584e-05	1081
FUNDC1	0.137645976868433	5.57226037602124e-06	2.08687832821989e-05	1081
TMEM161	0.137620923764226	5.59436935145702e-06	2.09414011627636e-05	1081
C21orf33	0.137620471793272	5.59476897781055e-06	2.09414011627636e-05	1081
C15orf23	0.137618952273761	5.59611271567035e-06	2.09425395982127e-05	1081
RNASE2	0.137542904159285	5.66375890090055e-06	2.11838884252955e-05	1081
GEMIN7	0.137535436594726	5.67044342033833e-06	2.1204953165531e-05	1081
LRRC23	0.137529892131541	5.67541137133086e-06	2.12195920704269e-05	1081
MRPS34	0.137479419122601	5.72082793741432e-06	2.13695677358078e-05	1081
MRPL18	0.137473882408343	5.72583106865182e-06	2.13842912269171e-05	1081
CLEC2D	0.137422723378891	5.77225809340315e-06	2.15403603190033e-05	1081
PEX14	0.1374158370142	5.77853490569114e-06	2.15571454953978e-05	1081
PKP3	0.137397119897783	5.79562824139941e-06	2.16169100024053e-05	1081
ATP8B5P	0.137338909712412	5.84909788735788e-06	2.18042333347196e-05	1081
MDS2	0.137331992437648	5.85548308442414e-06	2.18204184460289e-05	1081
CEACAM	0.137317349180963	5.86902196601849e-06	2.18623238730478e-05	1081
NMRAL1	0.137179263482563	5.99817333504603e-06	2.23145340414593e-05	1081
MVP	0.137173402239513	6.00371500225796e-06	2.23227830787166e-05	1081
SEPT3	0.137149167688798	6.02668018290731e-06	2.24040362215663e-05	1081

EPN1	0.137083646075458	6.0891909437515e-06	2.26280670654628e-05	1081
LOC38963	0.137051332451095	6.12024736594107e-06	2.27267065782273e-05	1081
CLEC6A	0.137030381756247	6.14046376885437e-06	2.27975751241377e-05	1081
WDR54	0.137028295775357	6.14248012860563e-06	2.28008590732929e-05	1081
C6orf64	0.13702498195085	6.14568465282273e-06	2.28085514611485e-05	1081
MAL	0.137017944570165	6.1524952182417e-06	2.28296216930335e-05	1081
PDRG1	0.136999847222948	6.17004242376099e-06	2.28863017173016e-05	1081
SDR39U1	0.136961221394934	6.20765416071359e-06	2.30173372110391e-05	1081
TYSND1	0.136958066655382	6.21073573372955e-06	2.30245254611671e-05	1081
ASB2	0.136955111505348	6.21362367214361e-06	2.30309933533271e-05	1081
ABHD11	0.136939577538706	6.22882544892431e-06	2.3074602550309e-05	1081
DDT	0.136823110596086	6.34393920245471e-06	2.34665176704829e-05	1081
ZMYND1	0.136779676349519	6.38738694684347e-06	2.36184388032018e-05	1081
LSM7	0.136771612603468	6.39548442776801e-06	2.36398227151168e-05	1081
AMPD3	0.136767865667377	6.39925037598459e-06	2.36494035634213e-05	1081
FBF1	0.136648451358222	6.5203864605899e-06	2.40661753844968e-05	1081
TMEM85	0.136481923457548	6.69297837255344e-06	2.46490035310949e-05	1081
CCL17	0.136419862767557	6.75840838927857e-06	2.48672396350716e-05	1081
SF3A3	0.136405874861053	6.77323986102017e-06	2.4908163015745e-05	1081
CACNA1I	0.136374732633901	6.80637192062508e-06	2.50163040213451e-05	1081
TH1L	0.136338062944181	6.84558309733335e-06	2.51404235910981e-05	1081
TMEM198	0.136227324361286	6.96531069310634e-06	2.5558503444923e-05	1081
FAM128B	0.136217774147881	6.97572928956021e-06	2.55920718517921e-05	1081
TMEM134	0.136200522470441	6.99458737089805e-06	2.56519138151723e-05	1081
RPP40	0.136184758447615	7.01186184111549e-06	2.57105855095143e-05	1081
KIAA0415	0.136176464451924	7.02096688697373e-06	2.57299214004158e-05	1081
UNC13D	0.136161829808203	7.03706016611127e-06	2.57842082659715e-05	1081
GTF2B	0.136057581026687	7.15272305021563e-06	2.61794342017794e-05	1081
HIST1H2A	0.13597587721801	7.24463934079857e-06	2.64965975890318e-05	1081
ITPA	0.135953337475627	7.27019426432406e-06	2.65755876346957e-05	1081
CDKN2C	0.135935486143973	7.29049466533206e-06	2.66401260716696e-05	1081
GNGT1	0.135866987829248	7.36889394964885e-06	2.69119595024794e-05	1081
CORO1B	0.135863605377398	7.37278609719219e-06	2.69212934435267e-05	1081

UBE2Z	0.135848733223599	7.38992261350409e-06	2.69719840887878e-05	1081
C17orf66	0.135767206958165	7.48453946489658e-06	2.7294722577904e-05	1081
ZC3H3	0.135764511556078	7.48768733475357e-06	2.73012599744092e-05	1081
FOXK2	0.13568008984326	7.58692296400461e-06	2.76380765117311e-05	1081
GPX4	0.135540194227771	7.75413705395126e-06	2.82165987991055e-05	1081
PIGX	0.135528625344476	7.76812149350676e-06	2.82623817024912e-05	1081
MEIG1	0.135524609006752	7.77298206171268e-06	2.82749591248108e-05	1081
LRFN4	0.13551234394613	7.78784320598357e-06	2.83239034815922e-05	1081
UBAC1	0.135481548105052	7.82527710697114e-06	2.84497757300006e-05	1081
FER1L4	0.135473196806905	7.83545810344513e-06	2.84816498545475e-05	1081
TRNAU1A	0.135466615081395	7.84349073822945e-06	2.85057046584218e-05	1081
ATP5O	0.135462182115877	7.84890535948378e-06	2.8520237818687e-05	1081
TRABD	0.135377062631193	7.95356881781245e-06	2.88589055898472e-05	1081
APOBEC3	0.13531425581394	8.03164933106382e-06	2.91159934810654e-05	1081
KCNJ1	0.135282523578464	8.07137609214838e-06	2.92473087908324e-05	1081
ABT1	0.135180039089219	8.20096539963271e-06	2.96977256831927e-05	1081
CHCHD4	0.135175400341084	8.2068777228162e-06	2.97084549373104e-05	1081
ARL11	0.135172031195748	8.21117442232286e-06	2.97169118294468e-05	1081
ZNF787	0.135159906582617	8.22665481879336e-06	2.97640016746753e-05	1081
MRPL49	0.135086975839143	8.3203603114993e-06	3.00868171737845e-05	1081
C22orf25	0.135020942046871	8.40608228862072e-06	3.03695350976084e-05	1081
GPR114	0.134998946603062	8.43482242114702e-06	3.04624413183949e-05	1081
PPCDC	0.13498758355724	8.44970655010132e-06	3.05107256590412e-05	1081
MAPK12	0.134974198264792	8.46727171908853e-06	3.05522458859105e-05	1081
LRRC24	0.134968942940706	8.4741776662896e-06	3.05662146978342e-05	1081
FLOT1	0.134957223660058	8.48959717306391e-06	3.06163507073707e-05	1081
ALG1L	0.134937416546434	8.51571904521884e-06	3.07050582004535e-05	1081
MAP3K11	0.134928689417639	8.52725282251777e-06	3.07356428895367e-05	1081
KCNK13	0.134926606445373	8.53000788266245e-06	3.07400731299168e-05	1081
TLX1NB	0.134908251839559	8.5543215028623e-06	3.08188177705909e-05	1081
MORN2	0.134907801944252	8.55491829104713e-06	3.08188177705909e-05	1081
GPA33	0.134881066491139	8.59045442183072e-06	3.094093028616e-05	1081
CACNG6	0.134863731902736	8.61357038071355e-06	3.1007929828355e-05	1081

SULT1A3	0.134791699260134	8.71026451747086e-06	3.13392174860601e-05	1081
ARHGAP2	0.13475130703107	8.7649380449458e-06	3.15077938821258e-05	1081
C9orf16	0.134664634566437	8.88336229030964e-06	3.18993464061119e-05	1081
CDCA2	0.134518926622028	9.08589756486003e-06	3.25797781956397e-05	1081
LOC54147	0.134517853808037	9.08740499783069e-06	3.25797781956397e-05	1081
MID1IP1	0.134496331404523	9.11769703111072e-06	3.26825634682786e-05	1081
EEF1E1	0.134493274455072	9.12200738712042e-06	3.26921969068744e-05	1081
FUNDC2	0.134363871482228	9.30626160519564e-06	3.33229008241497e-05	1081
ZNHIT1	0.134298822231668	9.40021971476783e-06	3.36413974336468e-05	1081
RHPN1	0.134295835250547	9.40455581467575e-06	3.36509372800431e-05	1081
CXorf26	0.134290003816055	9.41302661144775e-06	3.36752656877313e-05	1081
DYSFIP1	0.134235138175575	9.49308217199791e-06	3.39435818876283e-05	1081
NDUFAF3	0.134230660170073	9.49964471134172e-06	3.39549933836017e-05	1081
LMNB1	0.134224051934829	9.50933701934142e-06	3.39836072830642e-05	1081
GINS3	0.134194749226375	9.55242912121684e-06	3.4125498252689e-05	1081
POLE4	0.13416949698849	9.58971404475062e-06	3.42465501562668e-05	1081
MT1DP	0.13414774800111	9.62193765602281e-06	3.43494478257274e-05	1081
TMEM53	0.134141199755967	9.63165984362805e-06	3.4378062996082e-05	1081
PI4K2A	0.134083144670807	9.71826508734553e-06	3.46687533530327e-05	1081
C16orf87	0.134044574940441	9.77621267275194e-06	3.48507882308596e-05	1081
SPIB	0.134026007230417	9.80422638668466e-06	3.49382877339046e-05	1081
SERPINB1	0.133975956116255	9.88012204173404e-06	3.51900739976542e-05	1081
TRAF1	0.133964991983207	9.89682230956649e-06	3.52433242754493e-05	1081
GPT2	0.133962201310304	9.9010772830135e-06	3.52465106400785e-05	1081
HCN2	0.13393270035457	9.94616460447938e-06	3.53940091781023e-05	1081
HIST1H2A	0.133796131561773	1.01574512392147e-05	3.6107615178045e-05	1081
RUNX3	0.133764016255079	1.02077551372652e-05	3.62672358448339e-05	1081
PPIA	0.133719621720111	1.02776839949331e-05	3.64968793296854e-05	1081
MRPS9	0.133719532122963	1.02778255863641e-05	3.64968793296854e-05	1081
C19orf46	0.133706725538751	1.02980831300502e-05	3.65623695197146e-05	1081
TCF25	0.133699113540876	1.03101418776445e-05	3.65987327092773e-05	1081
PPCS	0.133682707607138	1.03361775755073e-05	3.66717677454377e-05	1081
SFXN5	0.13361614495418	1.04424550687246e-05	3.70227485673104e-05	1081

ZNF831	0.133517288572109	1.06022194935164e-05	3.75495273728706e-05	1081
MB	0.133476089905007	1.06694875235045e-05	3.77811260609945e-05	1081
ELOVL3	0.133467941097432	1.06828407884775e-05	3.78217623346008e-05	1081
ASIP	0.133430410694323	1.0744546977594e-05	3.80057231779404e-05	1081
CTRC	0.133414401793351	1.07709715302207e-05	3.80869267116547e-05	1081
DCAF11	0.133371371249584	1.08423059176147e-05	3.83257155133091e-05	1081
ST20	0.133251702619286	1.10430639406739e-05	3.89806418582226e-05	1081
SLC37A1	0.133212240559994	1.1110039619367e-05	3.91964532630732e-05	1081
ENHO	0.133161246324801	1.11971616053234e-05	3.94830772867566e-05	1081
RMND5B	0.132990089782514	1.1494363384163e-05	4.04743839143443e-05	1081
C16orf55	0.132964503765524	1.15394327222393e-05	4.06259825567126e-05	1081
AGER	0.13296008504483	1.15472332623944e-05	4.0646341791182e-05	1081
GPR115	0.132937248788839	1.15876271755508e-05	4.07671584791252e-05	1081
ZNF438	0.132891746919963	1.16685150758363e-05	4.10374015717043e-05	1081
KIAA0513	0.132876733641017	1.16953218089238e-05	4.11244995358299e-05	1081
EDC4	0.132865324790798	1.17157319509278e-05	4.11747069350037e-05	1081
MAPRE1	0.13285901927599	1.17270269187642e-05	4.12072138982215e-05	1081
SPN	0.132843279911635	1.17552658966984e-05	4.12992381389935e-05	1081
LONP1	0.132841205497084	1.17589925641266e-05	4.13051273242077e-05	1081
EMR2	0.132792706189542	1.1846442751451e-05	4.15833053193901e-05	1081
CASP7	0.132780117960604	1.18692419909506e-05	4.1656076638972e-05	1081
FAIM3	0.13255544064807	1.22832703624941e-05	4.30117297848851e-05	1081
BTF3L4	0.132549427670851	1.22945377875781e-05	4.30437024210567e-05	1081
DYNLT1	0.13251736628958	1.23547825119513e-05	4.3239592373742e-05	1081
RER1	0.132510461779228	1.23677931496088e-05	4.32776086501423e-05	1081
DDB2	0.13250470620642	1.23786487435895e-05	4.33008283069628e-05	1081
DNAJB12	0.13250466434331	1.23787277349118e-05	4.33008283069628e-05	1081
ALDOA	0.132468328810214	1.24474705407682e-05	4.3526174977222e-05	1081
CCDC135	0.132364474178246	1.2645963493056e-05	4.41895810177994e-05	1081
SPCS2	0.132260553843854	1.28475986615474e-05	4.48164285778135e-05	1081
NUCB1	0.132258996754712	1.28506430261126e-05	4.48192873547505e-05	1081
C10orf125	0.13217342786258	1.30190050560378e-05	4.53829134545565e-05	1081
ZNF80	0.132163048858603	1.3039568713468e-05	4.54467321337048e-05	1081

KCNK7	0.132145948077581	1.30735175695787e-05	4.5557172018537e-05	1081
CHAF1A	0.13212355218242	1.31181057770158e-05	4.56730454334571e-05	1081
C17orf99	0.13208971590664	1.31857456441712e-05	4.58689079609445e-05	1081
VSIG4	0.132063387690211	1.32386063636709e-05	4.60289480835608e-05	1081
DOHH	0.132055142858558	1.32552014681781e-05	4.60786943186279e-05	1081
DGKZ	0.131947599823403	1.347348616508e-05	4.68213522158939e-05	1081
NFE2	0.131921043405484	1.35279137007213e-05	4.6994278582692e-05	1081
C9orf89	0.131914312424401	1.35417421195494e-05	4.70342060341937e-05	1081
EIF2B2	0.131910024433429	1.35505585740372e-05	4.70567147860678e-05	1081
PLOD1	0.131905795603079	1.35592587445099e-05	4.70788120317393e-05	1081
CC2D1A	0.13190049434005	1.35701727973048e-05	4.71085871104094e-05	1081
C8orf85	0.131893335828557	1.35849237675082e-05	4.71516694170317e-05	1081
TIMM44	0.131885878290869	1.36003071606596e-05	4.71969315678705e-05	1081
TREM1	0.131860272841951	1.36532524219226e-05	4.73643482072724e-05	1081
ECM1	0.13185364594315	1.36669870348065e-05	4.74038315799202e-05	1081
GNG3	0.131803635549229	1.37710615678809e-05	4.77401540672793e-05	1081
MTX1	0.131774330994724	1.38323963614801e-05	4.79445328117718e-05	1081
HSP90B3I	0.131702003746583	1.39848937571706e-05	4.84480971174896e-05	1081
SPG7	0.131676407409089	1.40392446057974e-05	4.86280231402664e-05	1081
HMSD	0.131668349515676	1.40563962006431e-05	4.86790616231658e-05	1081
C12orf52	0.131646343415464	1.41033389252238e-05	4.88332352438352e-05	1081
PDCD6	0.131634161948932	1.41293882102314e-05	4.8915024144202e-05	1081
RGS9BP	0.131616343699453	1.41675739440789e-05	4.90387933167473e-05	1081
LOC10013	0.131594754675674	1.42139723996012e-05	4.91824929560231e-05	1081
LSM14B	0.131585743945791	1.42333807535155e-05	4.92327361400362e-05	1081
CTSL2	0.131583561103452	1.42380862084113e-05	4.92405573679735e-05	1081
PTPLAD2	0.131498316528942	1.44230070862892e-05	4.98544059288428e-05	1081
C2orf24	0.131455636584076	1.4516449653036e-05	5.01515826205472e-05	1081
TAF6L	0.131454417325888	1.45191275224242e-05	5.01522331857399e-05	1081
TATDN2	0.131412173423245	1.46121992866594e-05	5.04477728585695e-05	1081
SPOCD1	0.131398211547663	1.46430848082362e-05	5.05284247108459e-05	1081
SPDYC	0.131388254873291	1.46651482559215e-05	5.05958916964443e-05	1081
SULT1A1	0.131381294974041	1.46805898078222e-05	5.06404934381128e-05	1081

CCDC58	0.131335283550338	1.47830630423931e-05	5.097651574615e-05	1081
C7orf11	0.131242607676432	1.49915351280432e-05	5.16423521125907e-05	1081
HSPA6	0.13123597984689	1.50065510305549e-05	5.16852402992869e-05	1081
PKM2	0.131192518865039	1.51053709375398e-05	5.20078102096633e-05	1081
CHST11	0.131173689832492	1.51483757538864e-05	5.21469633564663e-05	1081
CAPN12	0.131169584260638	1.51577681962588e-05	5.21614691345463e-05	1081
NEURL	0.131164435057843	1.51695560232579e-05	5.2193118033908e-05	1081
IDI1	0.131151300054461	1.51996649085314e-05	5.22736945709996e-05	1081
NFAM1	0.131134242812831	1.52388495240029e-05	5.23867958465934e-05	1081
WDFY4	0.131099252538593	1.53195318723673e-05	5.26465024026422e-05	1081
LOC9661(	0.131099213294872	1.53196225904338e-05	5.26465024026422e-05	1081
TCOF1	0.131049237888884	1.54355646272461e-05	5.30087707834765e-05	1081
PEPD	0.131003176470823	1.55431656931766e-05	5.33419204240276e-05	1081
C2orf18	0.13098431917488	1.55874226800376e-05	5.34846925377886e-05	1081
ARAF	0.130931041494107	1.57131115268676e-05	5.38792564610634e-05	1081
SURF4	0.130886300206887	1.58194066657789e-05	5.42068289304501e-05	1081
KIR3DL3	0.130884975016693	1.58225654301982e-05	5.42084320733578e-05	1081
TNIP2	0.130846461726197	1.59146296293014e-05	5.45053066783876e-05	1081
ASNS	0.130765218492306	1.61105128839418e-05	5.50918828801574e-05	1081
GPN2	0.130740133617062	1.61714565525379e-05	5.52909016040184e-05	1081
GET4	0.130729175629425	1.61981477780077e-05	5.5363367320659e-05	1081
TMEM175	0.130674420448509	1.63321486917131e-05	5.57835088834455e-05	1081
SKAP1	0.130657096191255	1.63747651582093e-05	5.59101091715469e-05	1081
C6orf106	0.13064825126745	1.63965638469863e-05	5.59750514654362e-05	1081
RBM34	0.130599000054813	1.65184515232652e-05	5.63816004636016e-05	1081
GOT1	0.130554273639501	1.6629887902223e-05	5.67235170657437e-05	1081
ACYP1	0.130480348290068	1.68156438912837e-05	5.73182988477006e-05	1081
LOC28574	0.130449715056609	1.68931947936013e-05	5.75631612173712e-05	1081
CNIH2	0.130443932772607	1.69078713229904e-05	5.76034276681281e-05	1081
ATP6V1B	0.130432293304875	1.69374512963493e-05	5.76944464600873e-05	1081
CTSB	0.130374365050804	1.70854012505613e-05	5.81394270595537e-05	1081
HIST3H2A	0.130339361358874	1.71753966187768e-05	5.84160670074722e-05	1081
RRP9	0.130337191734089	1.71809895991293e-05	5.84252254345813e-05	1081

SGOL1	0.130288829462346	1.73061113855008e-05	5.88124922949354e-05	1081
P2RY11	0.130059955937357	1.79101009750599e-05	6.07405697209733e-05	1081
TBRG4	0.130017866840787	1.80233338811607e-05	6.10937339787957e-05	1081
FAM20A	0.130006124238846	1.80550464868518e-05	6.11909339632619e-05	1081
KIF4B	0.129916283294301	1.82994378334992e-05	6.19879225081288e-05	1081
HEXIM2	0.129894164001226	1.83600892167903e-05	6.21724655021417e-05	1081
SLC7A11	0.129885229643024	1.83846415206395e-05	6.2224227727366e-05	1081
TMEM14/	0.129850778262403	1.84796093107641e-05	6.24931558780163e-05	1081
VCAM1	0.129819957080455	1.85649655178478e-05	6.27607367607055e-05	1081
REEP2	0.129735323443769	1.88012830589062e-05	6.35169960123537e-05	1081
CHFR	0.129688564079544	1.89330707863148e-05	6.39300554794353e-05	1081
PRMT1	0.129623418837059	1.91181432391237e-05	6.45441587987509e-05	1081
LOC7299	0.129570405708539	1.92700178765139e-05	6.503509970219e-05	1081
POLD2	0.129478903209319	1.95348619614437e-05	6.58737519607103e-05	1081
TM4SF19	0.129462435041007	1.95828933927897e-05	6.60136190424614e-05	1081
SYNGR3	0.129460509386126	1.958851712996e-05	6.60215287908723e-05	1081
LMBR1L	0.129385164837974	1.98097651280204e-05	6.67448935447351e-05	1081
SLFN14	0.129360967824664	1.98813219682766e-05	6.69523956955753e-05	1081
CD63	0.129281629941551	2.01176728295583e-05	6.77143724563828e-05	1081
HRASLS2	0.12927921601825	2.01249057225887e-05	6.77274015672486e-05	1081
ZDHHC16	0.129227823314838	2.02794828495296e-05	6.82134216069082e-05	1081
HMX1	0.129205975607667	2.03455369990314e-05	6.84127596136683e-05	1081
FAM65C	0.129172991251815	2.04456492928064e-05	6.87035204342926e-05	1081
TBXAS1	0.129165130732427	2.04695761245877e-05	6.87724501383954e-05	1081
MYBPC3	0.12915792262151	2.04915404728082e-05	6.88347645197134e-05	1081
EEF1D	0.129153884759059	2.05038543219115e-05	6.88646457677406e-05	1081
C14orf119	0.129124514192765	2.05936346277069e-05	6.91546540381989e-05	1081
SH2D5	0.129107759413812	2.06450181429925e-05	6.93040977321419e-05	1081
FAM178B	0.129103486879565	2.06581406330496e-05	6.93365949771384e-05	1081
C17orf106	0.129097207789782	2.06774403698018e-05	6.93782538723613e-05	1081
E2F6	0.129082427590139	2.07229372517043e-05	6.95077540685287e-05	1081
FOXE3	0.12906225679816	2.07851812270782e-05	6.9704923559096e-05	1081
TOMM6	0.129015628430241	2.0929750322775e-05	7.01547121884031e-05	1081

POLQ	0.128957749997064	2.11105297293704e-05	7.07371293077456e-05	1081
LDHA	0.128890809725784	2.13214644373197e-05	7.14082961080309e-05	1081
LPCAT1	0.128824119050907	2.15336040718379e-05	7.20828271896267e-05	1081
ZDHHC13	0.128799921615136	2.16110697704333e-05	7.2330121369892e-05	1081
EVPL	0.12879831611334	2.16162189860821e-05	7.23353374542565e-05	1081
GTF3A	0.128780907658677	2.16721268608858e-05	7.24983386935478e-05	1081
RAD51AP	0.128779571294351	2.16764243187189e-05	7.25006753944202e-05	1081
WDR45	0.128742798386167	2.17949963362742e-05	7.28488802379699e-05	1081
HMHB1	0.128711206863166	2.1897353385526e-05	7.31667248219308e-05	1081
LTK	0.128680462728138	2.19974034692586e-05	7.34888379582444e-05	1081
NME1-NM	0.128648493738685	2.21019003158665e-05	7.38134585316862e-05	1081
DHRS4L2	0.128593239106094	2.22836241830843e-05	7.43710419430471e-05	1081
FTSJ2	0.128583162932567	2.23169158756771e-05	7.44698145296531e-05	1081
LSM6	0.128542794755629	2.24507670646453e-05	7.48792553836554e-05	1081
RCC2	0.128539211580848	2.24626848101027e-05	7.49066024663996e-05	1081
RPS19BP1	0.128521497775337	2.25216898400655e-05	7.50785109760251e-05	1081
AZI1	0.128377144956958	2.30080535349385e-05	7.65984528939574e-05	1081
IL9R	0.128331207345543	2.31649120109089e-05	7.70951846125491e-05	1081
SNTA1	0.128329111773898	2.31720917266445e-05	7.71063409040722e-05	1081
LOC12653	0.128318546132352	2.32083232829785e-05	7.72141490562515e-05	1081
MRPL10	0.128270142929763	2.33749962179332e-05	7.7742991383567e-05	1081
DAPK2	0.128268949693917	2.33791193697551e-05	7.77438692148758e-05	1081
MRPL15	0.128259830460506	2.34106531413059e-05	7.78230375464697e-05	1081
PRKCB	0.128241726810665	2.34733742929255e-05	7.79929284398785e-05	1081
TMEM126	0.128011656331361	2.42845049168151e-05	8.0608230606235e-05	1081
FAM110A	0.12798470857743	2.43812348528192e-05	8.09026480168082e-05	1081
AGPAT2	0.127832348846395	2.49350578379984e-05	8.25907168935348e-05	1081
MKI67	0.127762522462922	2.51928490492914e-05	8.34171505749467e-05	1081
TCTEX1D	0.127757042416996	2.52131875480988e-05	8.34707745532374e-05	1081
CD40LG	0.127747114710253	2.52500727062265e-05	8.35791512761967e-05	1081
C12orf10	0.12774373562431	2.52626389671094e-05	8.36070087058352e-05	1081
ZMAT2	0.127712806496794	2.53779356267993e-05	8.39609974054641e-05	1081
SNRPD3	0.127678436447544	2.55066455284979e-05	8.43729678442678e-05	1081

KISS1	0.127638461381765	2.56571251972285e-05	8.4856803004132e-05	1081
PCBD1	0.127572239994321	2.59082618998458e-05	8.56592706339066e-05	1081
GRIN2C	0.127560436106976	2.59532711951418e-05	8.57799258779542e-05	1081
SHBG	0.127544652635149	2.60135710591095e-05	8.59510232878071e-05	1081
CCDC23	0.127511547711658	2.61404790580215e-05	8.63137109693236e-05	1081
MAD2L1	0.127499054245215	2.61885255145207e-05	8.64440187596295e-05	1081
ATG4B	0.127497003290996	2.61964209335561e-05	8.64559141065674e-05	1081
TNFSF18	0.127394318801037	2.65946211230838e-05	8.76695536777162e-05	1081
IL24	0.127385453084625	2.66292697401119e-05	8.7769410817172e-05	1081
FOLR3	0.127375614819424	2.6667769410126e-05	8.78675523007832e-05	1081
CD19	0.127372937196349	2.66782567872306e-05	8.78877322941553e-05	1081
SLC6A9	0.12735516962153	2.67479458706224e-05	8.81029054224473e-05	1081
LOC1002	0.127308072287386	2.69335113730999e-05	8.86590548269779e-05	1081
LOC1002	0.127270014407945	2.70843534170463e-05	8.91235379918976e-05	1081
CXCL16	0.127242052513071	2.7195690958925e-05	8.94606783748439e-05	1081
SULT1A2	0.127237965274748	2.72120017187312e-05	8.94851084923018e-05	1081
TINF2	0.127090840676632	2.7805349011135e-05	9.13766322723187e-05	1081
HLA-DPB	0.127075172686631	2.78692561295035e-05	9.15717117483033e-05	1081
WNT3A	0.12706006738905	2.79310001328738e-05	9.17297029143695e-05	1081
ANO9	0.127040238309591	2.80122501037352e-05	9.19815449616537e-05	1081
SIVA1	0.127022959472236	2.80832333301683e-05	9.21470141697098e-05	1081
WDR76	0.12702129320031	2.80900875659562e-05	9.21470141697098e-05	1081
MGAT5B	0.126961219642629	2.83382646122754e-05	9.29157455426901e-05	1081
EPHX1	0.12689292057709	2.86229514599492e-05	9.37728666711136e-05	1081
ADCK4	0.12688512677141	2.86556099485118e-05	9.38493354597252e-05	1081
LRWD1	0.126855219973163	2.87812574039273e-05	9.42455185959226e-05	1081
P2RY8	0.126786028251186	2.90739603052587e-05	9.51730468556121e-05	1081
SELM	0.12677483984949	2.912155537795e-05	9.53133603718609e-05	1081
TSPAN3	0.126730101894602	2.93126092262437e-05	9.58763618871051e-05	1081
LCMT1	0.126709825634926	2.93995904333965e-05	9.61296460445988e-05	1081
IL3RA	0.12667852266933	2.95343552813095e-05	9.65389562132047e-05	1081
NEIL3	0.126637115727222	2.97135205112549e-05	9.70698316383758e-05	1081
CENPT	0.126622113872716	2.9778686797866e-05	9.72586973967268e-05	1081

RAVER1	0.126611762934825	2.98237291121925e-05	9.73900183117392e-05	1081
TMEM18[illegible]	0.126570826823012	3.00024977667326e-05	9.79420381641271e-05	1081
CNO	0.126510787078938	3.02665329222587e-05	9.87200375870486e-05	1081
UXS1	0.126509951172946	3.02702244812708e-05	9.87200375870486e-05	1081
PELO	0.126480876466182	3.03988915154533e-05	9.9107569117787e-05	1081
TRIM65	0.126453717428733	3.05195496805146e-05	9.94526574432168e-05	1081
MEPE	0.126438886142136	3.05856316548422e-05	9.9635761592302e-05	1081
HCG4	0.12643769842819	3.05909294767827e-05	9.96369077299577e-05	1081
TTLL13	0.126434399533828	3.0605648830294e-05	9.96687351578196e-05	1081
FXYD7	0.126396863761524	3.07736038950299e-05	0.000100199490943168	1081
MRPL43	0.126282046602289	3.12928034916797e-05	0.000101774867022907	1081
SULT2B1	0.126263253079185	3.13785748463207e-05	0.000102020882872681	1081
CDK4	0.126258620076933	3.13997535540423e-05	0.000102073266959203	1081
SPINT2	0.126255443018051	3.14142845892867e-05	0.000102104027597803	1081
KAT2A	0.126242420240797	3.1473914070162e-05	0.000102281335528862	1081
PYCR1	0.126190833951578	3.17111774828298e-05	0.000103019137299082	1081
GATA4	0.126184986950637	3.17381767111368e-05	0.000103090224096396	1081
VSIG10L	0.12618262123946	3.17491068627284e-05	0.000103109101684614	1081
PWP2	0.126170814178531	3.18037117240918e-05	0.000103219876378577	1081
CXCR5	0.126124572173716	3.20184291353313e-05	0.000103900008848461	1081
ARL14	0.12605856699658	3.23272976242e-05	0.000104784136868787	1081
SIGLEC1[illegible]	0.126030817546634	3.2457992226261e-05	0.000105173918835134	1081
ARHGAP[illegible]	0.126008205934954	3.25648590861427e-05	0.000105469306477547	1081
EIF4E2	0.125960344114925	3.27921654064182e-05	0.000106171355209305	1081
PVT1	0.125911018036623	3.3028000291992e-05	0.000106883384077458	1081
METT11D	0.125847841123507	3.33324085675916e-05	0.000107819923545682	1081
LOC3449[illegible]	0.125847624555166	3.33334566315466e-05	0.000107819923545682	1081
VAX2	0.125827888977696	3.34290966010604e-05	0.000108077218910024	1081
UHRF1	0.125809179548472	3.35200040859281e-05	0.00010835373592924	1081
SCNN1B	0.125741062488107	3.38529639420691e-05	0.000109390262653864	1081
TTC7A	0.12574025057236	3.38569514840824e-05	0.000109390262653864	1081
EIF4A3	0.125726248214236	3.39257910581665e-05	0.000109595102768885	1081
MEN1	0.125714054616555	3.39858463748265e-05	0.000109771504765252	1081

RUVBL1	0.125686277833727	3.41230279301176e-05	0.000110179259120407	1081
DSCR8	0.125641100973699	3.43472658035419e-05	0.000110867756707635	1081
C2orf39	0.125630979884586	3.43976936670621e-05	0.000110959413758681	1081
SNHG3	0.125446481314261	3.53293483250579e-05	0.000113691648883113	1081
SLC38A6	0.12541668113671	3.54820556195948e-05	0.000114164831569516	1081
SH3KBP1	0.125377715020468	3.56826770977221e-05	0.000114755352831037	1081
C10orf54	0.125367877003746	3.5733499053139e-05	0.000114900453060732	1081
TOR3A	0.125364672694607	3.57500669240038e-05	0.000114935381133747	1081
ZFYVE19	0.125357019886728	3.57896652215946e-05	0.00011504432836908	1081
TMEM187	0.125321699243914	3.59729661121213e-05	0.000115577728801622	1081
COQ10B	0.125320625499431	3.59785523611304e-05	0.000115577728801622	1081
IQSEC2	0.12529742809963	3.6099439887427e-05	0.000115929095573444	1081
INA	0.125275719350636	3.62129184527289e-05	0.000116256452945056	1081
KIFC2	0.125232018118327	3.64423853409676e-05	0.000116955847171227	1081
RAMP3	0.125192257017969	3.66523605486028e-05	0.000117573535549619	1081
KAZALD1	0.125168554368806	3.67780775327028e-05	0.000117939250540639	1081
CD226	0.125127075924925	3.6999060207301e-05	0.000118591259805263	1081
C11orf45	0.125109077780148	3.70953391492301e-05	0.0001188242339261	1081
SUMO1P1	0.125053764421303	3.73927202942158e-05	0.00011971969967053	1081
ORM1	0.124972734303218	3.78324457647374e-05	0.000121012165755896	1081
INPP5D	0.124883597151213	3.83218262588945e-05	0.000122486362532846	1081
ABCB8	0.124883252490292	3.83237301089365e-05	0.000122486362532846	1081
RAB24	0.124815300781851	3.87008405281257e-05	0.000123574010530843	1081
TLR7	0.124737574309392	3.91365100209007e-05	0.000124866169523447	1081
ENO1	0.124698275421894	3.93585520199697e-05	0.000125554715826174	1081
COX7A2L	0.124678002978278	3.9473559317084e-05	0.000125841882013397	1081
CCL20	0.124634291431594	3.97226233205295e-05	0.000126555787884243	1081
RACGAP1	0.124620393383969	3.98021245498766e-05	0.000126789025783881	1081
TMEM11	0.124605912748786	3.98851187607735e-05	0.00012703331500961	1081
PCBP1	0.12459091613021	3.99712431371459e-05	0.000127267376797504	1081
SLC28A1	0.124576807659024	4.00524278245587e-05	0.000127505714052739	1081
UBXN1	0.124575144181594	4.00620103014618e-05	0.000127516068497859	1081
TSC22D4	0.124534735327492	4.02954543890811e-05	0.000128197366991087	1081

ARRDC5	0.124533690011089	4.03015103079946e-05	0.000128197366991087	1081
TCP10L	0.124466646490502	4.06917258152071e-05	0.000129397761096661	1081
LRRC52	0.124412405112761	4.1010045891846e-05	0.000130368845588013	1081
TIGD5	0.124387893740223	4.11546656970362e-05	0.000130802255751287	1081
DSCC1	0.124342464381776	4.14239826467012e-05	0.00013156016560268	1081
C8ORFK2	0.124311877282924	4.16062506060642e-05	0.000132097386675991	1081
ELF4	0.124295772722884	4.17025224940689e-05	0.000132361322773439	1081
ZNF706	0.124255648252915	4.19433025051729e-05	0.000133062650230978	1081
C7orf28A	0.124201482512151	4.22704315638661e-05	0.000133973858378553	1081
EVI2A	0.124184841647444	4.23714169610263e-05	0.000134230570007843	1081
RASGEF1	0.124136826593269	4.26640799641929e-05	0.000135136460829979	1081
ALOXE3	0.124094297858361	4.29249009368366e-05	0.000135905646771258	1081
MIF	0.124093930635244	4.29271596130809e-05	0.000135905646771258	1081
ARMC7	0.124064427581269	4.31089921527737e-05	0.000136438436279281	1081
SNX24	0.124040445588128	4.3257334771044e-05	0.00013686492994545	1081
RBMX2	0.123989606448295	4.3573405130786e-05	0.000137821674730677	1081
GFER	0.123885854116326	4.42252409134561e-05	0.000139707931347275	1081
SPATA12	0.123883410782848	4.42407021822144e-05	0.000139714234405365	1081
CYBASC3	0.123883346814707	4.42411070375687e-05	0.000139714234405365	1081
ENDOG	0.12385142258186	4.44435944816856e-05	0.00014028771714722	1081
ANGPTL6	0.123809967940638	4.47078411261297e-05	0.00014105551440656	1081
GARS	0.12380458398083	4.47422693832376e-05	0.000141119933728718	1081
C7orf59	0.123801178086104	4.47640616750705e-05	0.000141144470565706	1081
HAR1A	0.123797494370062	4.47876428963251e-05	0.000141196723966584	1081
BASP1	0.123771201879614	4.49562956465604e-05	0.000141661907680269	1081
FAM127B	0.123757595783849	4.50438077201606e-05	0.000141915468646017	1081
SS18L2	0.123729108002137	4.52275590576769e-05	0.000142449840090197	1081
LSP1	0.123705019188398	4.53834899817527e-05	0.000142918618990528	1081
LYAR	0.12370003748892	4.54158008272701e-05	0.000142998016202775	1081
LTB4R	0.123671094657643	4.56039527589709e-05	0.000143562908483617	1081
EOMES	0.12364178935651	4.57952130092756e-05	0.000144070231542441	1081
NPC2	0.123635777581151	4.5834542483993e-05	0.000144136256375279	1081
CHKB	0.123630171358852	4.5871247621667e-05	0.000144229168618461	1081

ALMS1P	0.123559508218238	4.63362874238729e-05	0.000145555046024313	1081
STARD5	0.123541045332049	4.64585267614846e-05	0.000145893534155901	1081
FAIM	0.123537589858492	4.64814387072732e-05	0.00014594273422039	1081
CDKN2AI	0.123501680580802	4.67201738296225e-05	0.00014664660358332	1081
N4BP1	0.123422895725824	4.72480324678982e-05	0.000148211089079074	1081
KRTAP1-:	0.123416854168467	4.72887431726511e-05	0.000148315698460697	1081
SLED1	0.123374297563045	4.75764509029381e-05	0.000149171611430302	1081
FPR1	0.12337067540375	4.76010152177076e-05	0.000149225404849163	1081
TEX264	0.12333262980208	4.78597547065909e-05	0.000149966520230872	1081
KLHDC4	0.123318082425012	4.79590395706914e-05	0.00015023088977633	1081
MAPRE3	0.12331069767029	4.80095146114785e-05	0.000150365620623171	1081
BID	0.12326424470684	4.83281772246803e-05	0.000151316619551008	1081
NUDT2	0.123225308255139	4.8596818934699e-05	0.000152070719788424	1081
BAIAP2	0.123197381268771	4.87903708932911e-05	0.000152621431932508	1081
AMZ1	0.123142406078897	4.917351854385e-05	0.000153772202897525	1081
A4GALT	0.123128052757932	4.92740214143659e-05	0.000154062573551514	1081
HDAC11	0.123099206395781	4.9476593867833e-05	0.0001546239502742	1081
CLDN3	0.122998226116669	5.0191951613038e-05	0.000156788512715558	1081
C19orf56	0.12299814083646	5.01925598661025e-05	0.000156788512715558	1081
SIRPB1	0.122945220563294	5.05713572330019e-05	0.00015784939440019	1081
SF3B5	0.12293434667357	5.06495256934209e-05	0.000158068891571489	1081
C19orf52	0.122897300671652	5.09166953555516e-05	0.000158804277432666	1081
CBX8	0.122851194523338	5.12510685142915e-05	0.000159743347097972	1081
C5orf39	0.122809597347382	5.1554523812025e-05	0.000160594693396203	1081
GALNTL4	0.122792906287781	5.16767640458334e-05	0.000160875971519597	1081
IFNA10	0.12274419012011	5.20351169804864e-05	0.000161941515768871	1081
CLNK	0.122723730259304	5.21863185085038e-05	0.000162386992022522	1081
ABHD15	0.122717456429929	5.22327663070844e-05	0.000162506421197871	1081
SLC22A2(	0.122658539772706	5.26708639507462e-05	0.000163818828823187	1081
ADCK2	0.122600048858261	5.31092337219092e-05	0.000165105789093806	1081
STK32C	0.122598295448437	5.31224280866354e-05	0.000165121325999887	1081
TLR8	0.12252602887006	5.36689385507201e-05	0.00016676858971221	1081
FAM24B	0.122500051968658	5.38666834494659e-05	0.000167357239063771	1081

HRSP12	0.122487425763456	5.39630471438804e-05	0.00016763077636291	1081
FAM167B	0.122476109141663	5.40495546320209e-05	0.000167873616722489	1081
FAM111B	0.122443588190524	5.42988849751768e-05	0.000168596029257851	1081
RETN	0.122430224613673	5.4401655441469e-05	0.000168863073785577	1081
AP2A1	0.12233283007151	5.51562297664645e-05	0.000171073479391136	1081
TSTA3	0.122318136982739	5.52709219364683e-05	0.000171376438727128	1081
CCDC86	0.122266345492394	5.56770008460654e-05	0.000172555874160614	1081
C11orf68	0.12219950903396	5.62052142609537e-05	0.0001740322842433	1081
LYPLA2P	0.122191347150307	5.62700420432699e-05	0.000174206238967523	1081
ANKRD5:	0.122103542572866	5.69719395945706e-05	0.00017621675466492	1081
MTG1	0.12207227351459	5.72238937263735e-05	0.000176887423525824	1081
AGMAT	0.12205720862466	5.73456567014349e-05	0.000177209426944379	1081
HTATSF1	0.122046038797388	5.74360957368892e-05	0.00017746167923614	1081
PTK2B	0.121945826007799	5.82535476623459e-05	0.000179821899733061	1081
AEN	0.121910649398216	5.85430888895957e-05	0.000180660313370237	1081
TGM1	0.12186527967746	5.89185403799482e-05	0.000181763245934771	1081
C9orf37	0.121851050978013	5.90367564910069e-05	0.000182100055046905	1081
AGRP	0.121834831080564	5.91717896265597e-05	0.000182460692182312	1081
WIPF1	0.121831691814819	5.91979582082477e-05	0.000182513447827541	1081
TRIM15	0.121793524413725	5.95169942149976e-05	0.00018341285734452	1081
POLD1	0.121790316379256	5.95438836734825e-05	0.000183467656256088	1081
DDX56	0.121742784615093	5.9943639196745e-05	0.000184586458517033	1081
FAM71F1	0.121696921341964	6.03317669214918e-05	0.000185668109476543	1081
DDX54	0.121651278615697	6.07203865139367e-05	0.000186778467907048	1081
BCS1L	0.121579259897223	6.13383986863355e-05	0.000188478041723037	1081
BSND	0.121576238292049	6.13644574488783e-05	0.000188529357222457	1081
HIST1H4I	0.121575122317422	6.1374084422883e-05	0.000188530181564346	1081
MLF1IP	0.121571809690298	6.14026692677533e-05	0.000188589231955922	1081
CCR2	0.121563287120616	6.14762688593287e-05	0.000188741147245368	1081
TLR1	0.121557523471826	6.15260901057363e-05	0.0001888531289319	1081
OR13A1	0.121529240413922	6.17711239269681e-05	0.000189515663091658	1081
FCGRT	0.12148262012266	6.21770395591997e-05	0.000190647863305948	1081
C4orf44	0.121429554686872	6.26421405454003e-05	0.000192015508412521	1081

IL2	0.121345697119058	6.33838355913089e-05	0.000194141305760517	1081
C1orf86	0.121306713891503	6.37314500064078e-05	0.000195146688004117	1081
CDK11B	0.121290908818358	6.38728970049207e-05	0.000195520363191631	1081
HVCN1	0.121288533557817	6.38941799549783e-05	0.00019555579689958	1081
HPS3	0.121193996294531	6.47467218308236e-05	0.000198044748144844	1081
SKA3	0.121187390054644	6.48066974046653e-05	0.000198198105240167	1081
MMP1	0.121114840299135	6.54688134596773e-05	0.000200071184336347	1081
MPDU1	0.121112783130193	6.54876808386613e-05	0.000200098487865135	1081
PNOC	0.121071542670135	6.58670046879453e-05	0.000201196483614656	1081
TTC35	0.121068746297805	6.58928003503317e-05	0.000201244763956554	1081
CXCR1	0.121033283883803	6.6220759425812e-05	0.000202215734217521	1081
CEBPG	0.121019091973645	6.63524389841747e-05	0.000202556430267646	1081
CD79A	0.120981106029586	6.67061099712021e-05	0.000203605240207555	1081
CN5H6.4	0.120973290745593	6.67790953855576e-05	0.00020376626424448	1081
SFRS2	0.120947695323241	6.70186546875385e-05	0.000204435311732354	1081
SUMO3	0.120882334452612	6.76340871154978e-05	0.000206281405744391	1081
C9orf103	0.120743790596084	6.89563057492881e-05	0.000209996187349873	1081
SIGLEC11	0.120727025882948	6.91179492312295e-05	0.000210456633504099	1081
ZNF672	0.120724718505065	6.9140224716813e-05	0.0002104926442376	1081
MCM5	0.120691775626849	6.9458996747178e-05	0.000211399227900272	1081
C9orf140	0.120667027205935	6.96993879165992e-05	0.000212034758317712	1081
MPG	0.120656734898003	6.97995923953948e-05	0.000212278583801017	1081
PMCH	0.120656630190287	6.9800612514169e-05	0.000212278583801017	1081
CCDC85B	0.120648919198323	6.98757758618542e-05	0.00021247509505465	1081
C22orf27	0.120645081487691	6.99132126644296e-05	0.000212556847136271	1081
INSIG1	0.120637795389847	6.99843405707877e-05	0.000212708892697423	1081
STARD10	0.120630631271425	7.00543443507319e-05	0.000212857430911839	1081
JTB	0.120589163906381	7.04608417715777e-05	0.000213995727044842	1081
APOC3	0.120434745848536	7.19942593649586e-05	0.000218521072006493	1081
PTOV1	0.120417210222953	7.2170372861965e-05	0.000218989631164978	1081
CSTF1	0.120383920933064	7.25058233812104e-05	0.000219941245597724	1081
CAPN1	0.120377430430197	7.25713984057597e-05	0.000220107019103287	1081
KCNE2	0.120334568111363	7.30058546292233e-05	0.000221358058625181	1081

ACTL8	0.120325720600581	7.30958391217572e-05	0.000221597543883792	1081
THOC3	0.120295310225462	7.34059303648238e-05	0.000222403739990882	1081
NAGPA	0.120293352650483	7.34259340449389e-05	0.000222430893433879	1081
ADPGK	0.120233288120698	7.40422180269015e-05	0.000224230379156935	1081
KRTCAP3	0.12023087644544	7.40670644175666e-05	0.000224271909317883	1081
SNHG12	0.120212990443213	7.42515816526041e-05	0.000224796830837347	1081
CRABP2	0.12016947113956	7.47023543626837e-05	0.0002260935890379	1081
MBD4	0.120085345972796	7.55810584759193e-05	0.000228615679128738	1081
TREM2	0.12005915742077	7.5856589256437e-05	0.000229414651039022	1081
C1QL1	0.120051202669219	7.59404691376446e-05	0.000229633856316099	1081
PIK3R6	0.120049013385267	7.59635696831954e-05	0.000229663641162112	1081
ABCF1	0.120048108455871	7.59731201143865e-05	0.000229663641162112	1081
NEU4	0.119991984219277	7.65676640873242e-05	0.000231356771117316	1081
ABCA7	0.119978013214395	7.67163453578695e-05	0.000231701765702291	1081
IL21	0.119970827558315	7.67929221761153e-05	0.000231898278704518	1081
CCDC153	0.119864659330773	7.79327910024854e-05	0.000235023364482795	1081
TOE1	0.119844808909329	7.8147678750244e-05	0.000235600866271126	1081
CELF3	0.119830118988966	7.83070623092642e-05	0.00023601073761522	1081
C17orf101	0.119813128180452	7.84917929378969e-05	0.000236532112002084	1081
SHMT2	0.11980439568819	7.85868959557685e-05	0.000236783281338462	1081
PCP4	0.119798672982633	7.86492792621643e-05	0.000236935805403963	1081
LPCAT4	0.119784413982046	7.88049202910974e-05	0.000237333699994641	1081
CCNE2	0.11974698068866	7.92149001203006e-05	0.000238461470849291	1081
RPL28	0.119708650722924	7.96367865057736e-05	0.000239624057379956	1081
REXO4	0.119705074680849	7.96762549150465e-05	0.000239707012434828	1081
TCL1B	0.119701017976087	7.97210507042186e-05	0.000239805967811928	1081
PPP2R2C	0.119585476416833	8.10069346612315e-05	0.00024344043100763	1081
RACGAP1	0.119570873077339	8.11708446168368e-05	0.000243802991621616	1081
PVRIG	0.119562490417147	8.12650741583338e-05	0.000244049630131132	1081
CSF1	0.119535734272753	8.15665313766804e-05	0.000244881933618961	1081
TTC9B	0.119513220381138	8.18210092438313e-05	0.00024560933262062	1081
HDHD1A	0.119509031019138	8.18684448441562e-05	0.000245715110456723	1081
KIF11	0.119471981612682	8.22890817452034e-05	0.000246830487158595	1081

SLC2A8	0.119459900695865	8.24266817151915e-05	0.000247169619403473	1081
QPCT	0.119401056304745	8.3100021776448e-05	0.000249040455026264	1081
AAMP	0.119339743579727	8.38071267039096e-05	0.00025101019438749	1081
LHX3	0.119322795663572	8.40035823675054e-05	0.00025148642692724	1081
MKKS	0.119286924651461	8.44208237188983e-05	0.000252622919461855	1081
CIDECP	0.119267898444417	8.46429241059404e-05	0.000253174715087479	1081
HDAC10	0.119185669766154	8.56091682996033e-05	0.00025598882223475	1081
STUB1	0.119165069919274	8.58528560278038e-05	0.00025663556169953	1081
RRS1	0.119164156827246	8.58636726659139e-05	0.00025663556169953	1081
SAMD1	0.119161424626163	8.58960464496981e-05	0.000256694237610024	1081
S1PR5	0.119153377286021	8.59914661185994e-05	0.000256941276321445	1081
SLC22A1(	0.119129546612834	8.62746203661888e-05	0.000257710887793131	1081
TGFBI	0.119121483001038	8.63706306276844e-05	0.000257959429799066	1081
S100A13	0.11905877110624	8.71207647572693e-05	0.000260084144344278	1081
EED	0.119028407156881	8.7486170568737e-05	0.000261136302579228	1081
PELI3	0.118984577267098	8.80161790281048e-05	0.000262679396521655	1081
DRG1	0.118977695834685	8.80996666789143e-05	0.000262889614167787	1081
CACNB1	0.11895667082807	8.83552116088816e-05	0.000263574076389889	1081
CBR1	0.118948847073692	8.84504825259411e-05	0.000263745773558898	1081
GTPBP4	0.118938062043768	8.85819721848107e-05	0.000264094099402547	1081
KEL	0.118882427161741	8.92631995942504e-05	0.000266006975713931	1081
TRIP13	0.118845506820409	8.97179995778227e-05	0.00026724369384818	1081
FABP5	0.118821955825356	9.00092513921955e-05	0.000268071609889973	1081
TOR1A	0.118800496077777	9.02754174451806e-05	0.00026874512848133	1081
IFNW1	0.118732436069074	9.11244879839538e-05	0.000271192614926392	1081
PRKCG	0.118729610759996	9.11598969437791e-05	0.000271257920817198	1081
TICAM2	0.118712624079974	9.1373060893195e-05	0.000271852061983963	1081
CDK9	0.118672726552746	9.18755809206348e-05	0.000273226096493385	1081
MCM6	0.11866212340537	9.20095681678444e-05	0.000273584169851103	1081
RGS20	0.118659230928883	9.20461510863597e-05	0.000273652555140897	1081
PRPF31	0.118657238285349	9.20713612477892e-05	0.000273687114111954	1081
CLEC12A	0.118623650725995	9.24972794129787e-05	0.000274828118676694	1081
P2RX5	0.118617208529554	9.25791839202295e-05	0.000274953215402185	1081

CDC37	0.118576406326233	9.30995237995468e-05	0.000276457828263837	1081
RGS18	0.118566912140413	9.32209956206167e-05	0.000276777738655464	1081
VRK2	0.118546833277049	9.3478383878872e-05	0.000277460150764679	1081
C11orf24	0.118503109754022	9.40411904720209e-05	0.000278966246805899	1081
CLCN2	0.118496157026596	9.41309794990097e-05	0.000279191487339157	1081
MRPL24	0.11847039834309	9.44643378068983e-05	0.000280138979113789	1081
RNF167	0.11845412752576	9.46754814267771e-05	0.000280723811207304	1081
AGPAT1	0.118448808197049	9.47446059237843e-05	0.000280846701913704	1081
PTMS	0.118429338611865	9.49980186314208e-05	0.00028155584601	1081
DKFZp68	0.118416499354319	9.51654817343493e-05	0.000281969205697671	1081
C7orf47	0.118412092680814	9.52230223445492e-05	0.000282098203695727	1081
S100A9	0.118377422826852	9.56768734400401e-05	0.000283359396567128	1081
CAPS	0.118320957303973	9.64204129956373e-05	0.000285393655568192	1081
C17orf93	0.118281892262012	9.69380074948169e-05	0.000286841386748397	1081
CBX2	0.118177080243124	9.83396942546427e-05	0.000290732776747839	1081
KIF18A	0.1181663712977	9.84839795782516e-05	0.000291116620484795	1081
OLIG2	0.118109270689224	9.92566910393625e-05	0.00029331466055273	1081
C17orf56	0.118101366077664	9.9364109127662e-05	0.000293464006726088	1081
DAZAP1	0.118101263807731	9.93654996216753e-05	0.000293464006726088	1081
TMED3	0.118093796013236	9.94670836813074e-05	0.000293720961706235	1081
KMO	0.11807974835528	9.96584386689289e-05	0.000294156666224992	1081
C15orf56	0.118072027798026	9.9763754609272e-05	0.00029442438274301	1081
MRPL33	0.118054023797109	0.000100009754507271	0.000295107148754793	1081
HAPLN3	0.118005607323785	0.00010067413858808	0.000296980600652639	1081
SCXB	0.117992940267056	0.000100848644792486	0.000297451822744456	1081
ENO3	0.117988895181861	0.000100904431467344	0.000297572796356264	1081
SLC39A7	0.117915435889734	0.000101922599723029	0.000300311652979438	1081
GTF2A2	0.11783715164438	0.000103018297837118	0.000303185333809898	1081
NR1H2	0.117825546647031	0.000103181667854491	0.000303577435216698	1081
C18orf21	0.117763364043517	0.000104061214567929	0.00030598644978411	1081
C19orf22	0.117742071427515	0.000104364010203258	0.000306832017738562	1081
LOC1457	0.117680815593194	0.000105239747366273	0.000309090933181742	1081
SIRT2	0.117651203435992	0.000105665572904854	0.000310251124641931	1081

ILVBL	0.117613267547704	0.000106213467096128	0.000311814382782207	1081
HIST1H4E	0.117603732190987	0.000106351603182068	0.000312151257435089	1081
NTAN1	0.117603207627535	0.000106359207298806	0.000312151257435089	1081
DHODH	0.117602061810528	0.000106375818942126	0.000312154533516261	1081
DGCR6	0.117592189788264	0.00010651904127947	0.000312529287296085	1081
ARF5	0.117566846295638	0.000106887554655443	0.00031351918877896	1081
KLHL17	0.117553704042417	0.00010707912554632	0.000313989662901108	1081
RNF5	0.117547001944717	0.000107176944636782	0.000314185033426656	1081
HLA-DQA	0.117536548073907	0.000107329690072436	0.000314587022626104	1081
C8orf51	0.117528513801215	0.000107447221224186	0.000314884909770509	1081
C1orf123	0.117513266127151	0.000107670608768252	0.000315402706650637	1081
TARBP2	0.117505783530561	0.00010778039328133	0.000315632508017502	1081
CASP10	0.117503250496237	0.000107817581802051	0.000315695521134058	1081
RHOC	0.11748780892325	0.000108044547527609	0.000316314112766122	1081
TUBA4A	0.117482070759631	0.000108129003762295	0.000316515370646822	1081
GCA	0.117478286012691	0.000108184742988638	0.000316632521793711	1081
SMUG1	0.117463261434126	0.000108406282116015	0.000317234827604171	1081
CARM1	0.117436712894776	0.000108798788662206	0.000318337196456085	1081
MRP63	0.117353432419501	0.000110038753615856	0.000321871742644317	1081
PDCD10	0.117196488684115	0.000112411760883146	0.000328717509506601	1081
LOC28583	0.117129492185832	0.000113439365808971	0.000331674314110554	1081
DBI	0.117012079558794	0.000115261635358627	0.000336855599056949	1081
EIF1	0.116976675139536	0.000115816507015056	0.000338428130812056	1081
DRAM1	0.116931210159622	0.000116532741594071	0.000340361653632852	1081
FAM72A	0.116930385113273	0.000116545777451448	0.000340361653632852	1081
FFAR3	0.116905047397513	0.000116946786544838	0.000341334832649342	1081
MUC21	0.116870045933886	0.000117502877255499	0.000342858554796064	1081
TRIAP1	0.116843636273581	0.00011792411170031	0.000343988014799122	1081
EIF3I	0.116783267094476	0.000118892347409195	0.000346711977208778	1081
NEK2	0.116762552213687	0.000119226306101381	0.000347635538632553	1081
PLEKHF1	0.116747538797457	0.000119468899462605	0.000348191692661195	1081
LIPT2	0.116723489770367	0.000119858462955567	0.00034922602491176	1081
FLOT2	0.116692555050687	0.000120361324788814	0.000350589775574127	1081

ITIH4	0.116616649064018	0.000121603650687775	0.000353910127930865	1081
MAP1S	0.116597474985525	0.00012191937041599	0.000354768989893127	1081
CCNF	0.11657320410331	0.000122320120691603	0.000355883713364002	1081
SAP30	0.116560474661323	0.000122530799167519	0.0003564451912245	1081
MYBPH	0.116539108160599	0.000122885193292264	0.000357424519040234	1081
C19orf73	0.116508891092433	0.000123388034412164	0.000358835275477557	1081
UBE2G2	0.116443108640376	0.000124489424078886	0.000361881594237972	1081
USE1	0.116424030483498	0.000124810575042082	0.000362658161578355	1081
AICDA	0.116336897674116	0.000126287259430428	0.000366631624330016	1081
RGS1	0.116299828897543	0.000126920455886311	0.000368416798822728	1081
PSMD6	0.116274679357991	0.000127351752023357	0.000369457102849927	1081
SLC25A42	0.116214134311783	0.000128395719578909	0.000372323559942008	1081
C6orf115	0.116208744517948	0.000128489044221185	0.000372500655799519	1081
CDH24	0.116147691247743	0.000129550652657752	0.000375510488890709	1081
SLIT1	0.11614549963567	0.000129588913960873	0.000375567353149444	1081
C20orf123	0.116107035472172	0.000130262157344818	0.000377247147744588	1081
C7orf61	0.116104586465004	0.000130305133907137	0.000377317367048911	1081
WASH3P	0.116089311833984	0.000130573483055027	0.000378040071305479	1081
C5orf40	0.116051386209993	0.000131242021786041	0.000379866455298822	1081
C12orf45	0.116048418056798	0.000131294478959601	0.000379936356831775	1081
IL26	0.116026483318235	0.000131682751096006	0.000380977886087755	1081
RPUSD1	0.115883745151243	0.000134235907252024	0.000387958445832564	1081
SPATS2L	0.115830285245471	0.000135204076809506	0.000390548627377042	1081
TAF11	0.115816079993392	0.000135462439340461	0.000391238830181159	1081
ALOX5AP	0.115810833372321	0.000135557981329312	0.00039140254176279	1081
CWC15	0.115809436309311	0.000135583432804152	0.000391419927463406	1081
VWA3A	0.115806368803322	0.000135639331920542	0.000391525195807323	1081
EAF2	0.11577117636156	0.000136282196463627	0.000393324476756413	1081
ZNF259	0.115759526222141	0.000136495641698663	0.000393827657121106	1081
TMEM9	0.115737206950643	0.000136905437548637	0.000394896912860436	1081
NFS1	0.1157057698259	0.00013748460647091	0.000396397223036564	1081
CCDC12	0.115687654021648	0.000137819401501278	0.000397305644425192	1081
TSSC4	0.115681602786855	0.00013793140408736	0.000397571631898679	1081

TRAPPC9	0.115649799457022	0.000138521462296196	0.000399044023731855	1081
TGM2	0.115581238766431	0.000139801578893161	0.00040250147303169	1081
C3orf75	0.115551627562217	0.000140357891892261	0.000404045403282309	1081
DUSP13	0.115525228462228	0.000140855613294275	0.000405304432197282	1081
NADSYN	0.115463451150082	0.000142026841136117	0.000408441215515642	1081
DEPDC1	0.115382726520328	0.000143571096050828	0.000412587693287292	1081
CKAP2L	0.115374585286194	0.000143727710628091	0.000412978851890301	1081
SFXN3	0.115309895220802	0.000144977889192497	0.00041627417015149	1081
RTDR1	0.115307117471844	0.000145031799341366	0.00041636961632205	1081
IFT20	0.115228855551689	0.000146558465589899	0.000420273350791247	1081
C14orf1	0.115175709036513	0.0001476038032693	0.000423030106254097	1081
C2orf89	0.115065301492255	0.000149797856564874	0.000429196105888122	1081
TTK	0.115051951760518	0.000150065214761238	0.000429900988533153	1081
CAMK1G	0.115000816217883	0.000151093474202979	0.000432600630730388	1081
OR52K1	0.114985254570988	0.000151407707910412	0.000433377134960963	1081
LPPR2	0.114979060803999	0.000151532948442702	0.000433612392951453	1081
KLKB1	0.114949552808909	0.000152130950656052	0.000435138151493136	1081
LRSAM1	0.114948007293068	0.000152162332782046	0.000435166126333663	1081
RPS27L	0.114925021603656	0.000152629782428167	0.000436317151556043	1081
TERF2IP	0.114863929669337	0.000153878751974977	0.000439700348728497	1081
INO80B	0.11479179928629	0.000155365759278439	0.000443571885014762	1081
TPI1P2	0.114749020443909	0.000156254038003345	0.000445981523884584	1081
SCAF1	0.114725938830723	0.00015673529510019	0.000447228402237016	1081
NUDT18	0.11466604761681	0.000157990546261076	0.000450682463102432	1081
TOM1	0.114632899243039	0.000158689353658113	0.000452547710849757	1081
VPS33B	0.114599753166606	0.000159391016279731	0.000454355741185112	1081
RIN3	0.114562010627165	0.00016019353020797	0.000456449599156939	1081
CCDC84	0.114503232895837	0.000161450877019007	0.000459642159065558	1081
ADK	0.114495503769868	0.000161616903203255	0.000460049811364926	1081
CCDC94	0.114491709230007	0.000161698470858289	0.000460192952209096	1081
RANGAP	0.114449710483209	0.000162603865634101	0.000462663117683469	1081
COPS5	0.11443854223423	0.000162845427496709	0.000463285007332468	1081
MRPS21	0.114366674351197	0.000164407966880032	0.000467532254771067	1081

NHP2L1	0.114360811074277	0.00016453606480587	0.000467830490545414	1081
FCGR2B	0.114347476640655	0.000164827738019351	0.000468593675190491	1081
MTCH1	0.114295333564154	0.00016597296967149	0.000471516778174048	1081
SOD2	0.114248261499026	0.000167013241799737	0.000474204616780226	1081
POLR2D	0.114221451908361	0.000167608456628653	0.000475559487152707	1081
B9D1	0.114218614588633	0.000167671566037558	0.000475671552996282	1081
LSM1	0.114170236875294	0.000168751053937897	0.000478397126594277	1081
IL19	0.114166704469985	0.000168830130330367	0.000478553957437068	1081
IER5L	0.114128351314916	0.000169690947292165	0.000480791017327802	1081
WDR83	0.114048240976322	0.000171502299482663	0.000485786533053748	1081
PPP2R3B	0.114025130530809	0.000172028208822237	0.000487139199708177	1081
CENPE	0.114020997670389	0.000172122417142894	0.000487189750781013	1081
IDH2	0.113961751893976	0.00017347825862946	0.000490762465958498	1081
C12orf32	0.113960458029093	0.000173507980386654	0.000490777627757532	1081
CIB1	0.113900607944863	0.000174888053396874	0.000494334198916799	1081
GRAMD1.	0.113877382214746	0.000175426382797624	0.000495716717836742	1081
SPERT	0.113758480083112	0.000178206774414798	0.000503150030916062	1081
ACCN3	0.113747871903364	0.000178456834941923	0.000503785445614495	1081
RAP2C	0.113739388077532	0.000178657056367996	0.000504212052680393	1081
PFKP	0.113674012103789	0.000180207043965967	0.000508156621037849	1081
ZNF444	0.113667989747299	0.000180350460302395	0.000508489856233975	1081
COMMD9	0.113658941663831	0.000180566132735118	0.000508955469980263	1081
ACTN2	0.11362436843402	0.000181392461537122	0.00051114157751648	1081
POLR2L	0.113613910183195	0.000181643120969605	0.000511776317752823	1081
G6PC3	0.113577450728814	0.00018251951283547	0.000514173624118382	1081
SERINC2	0.113526709191962	0.000183745817962959	0.00051755585890154	1081
DDX24	0.113496455110239	0.000184480665266798	0.000519335243404087	1081
SLC24A4	0.113469695963558	0.000185132921755902	0.000521025804522584	1081
BOP1	0.113465720082258	0.000185230018724423	0.000521226250482399	1081
GSDMC	0.113445905008996	0.000185714645680308	0.00052229813447296	1081
YTHDF1	0.113403059679257	0.000186766607057006	0.000525183319257872	1081
FAM83F	0.113399655229243	0.000186850434360004	0.000525345708329699	1081
GTPBP1	0.113384493232023	0.00018722419519652	0.00052624967381525	1081

THOP1	0.113341175255256	0.000188295903938669	0.00052904058366032	1081
NT5C	0.113338780331185	0.000188355323229551	0.000529133731203361	1081
SGTA	0.113296903078432	0.00018939716796354	0.000531763895278816	1081
NIT1	0.113295321889166	0.000189436611405083	0.000531800520729569	1081
KEAP1	0.113286447710238	0.000189658124685278	0.000532330686632843	1081
TBX1	0.113284581440741	0.000189704740597527	0.000532330686632843	1081
TLR3	0.11324858384606	0.00019060599989015	0.000534785218354747	1081
FMR1NB	0.113243349922212	0.000190737373954516	0.00053507929234281	1081
C17orf42	0.113222940080167	0.000191250483302523	0.000536444024802189	1081
ANXA2P3	0.113219555368353	0.000191335701068241	0.000536608338858376	1081
CSTL1	0.113108951877751	0.000194140082452713	0.000544094596690302	1081
LCP1	0.113039797523412	0.000195913058995261	0.000548834456050555	1081
REM2	0.113024818250174	0.000196299093646649	0.000549839438474937	1081
C17orf96	0.113016097364363	0.000196524170423301	0.000550316849204531	1081
SYT13	0.11298424008287	0.000197348432599969	0.000552548182727781	1081
EID2	0.112977533697578	0.000197522363653829	0.000552812055363404	1081
MYH14	0.112977431993996	0.000197525002457472	0.000552812055363404	1081
POLR2E	0.112966220418579	0.000197816101163484	0.000553549848303706	1081
PTPN1	0.112949338356485	0.000198255188548604	0.00055462446511757	1081
LYG1	0.112931091035994	0.000198730813376122	0.000555800671312228	1081
KRT2	0.112849644274564	0.000200866844034268	0.000561307056883109	1081
VEGFB	0.112842862502963	0.000201045671449422	0.000561728855942941	1081
MRTO4	0.112823873907181	0.000201547173495378	0.000562973905998945	1081
MGST2	0.112821009246831	0.000201622932974448	0.000563029385191331	1081
C17orf55	0.112805490543773	0.000202033808821706	0.000564098555608213	1081
C10orf11	0.112774406896774	0.000202859148237209	0.000566089145482557	1081
CAPZB	0.112751845491318	0.000203460183226735	0.000567609111079156	1081
BLM	0.112686827059311	0.00020520162894962	0.000572316806416231	1081
BCL2L1	0.112661613926671	0.000205880688866962	0.000573964361641981	1081
VRK3	0.112620772573273	0.000206985134602057	0.000576803919844854	1081
SPP1	0.112569043297987	0.000208391992792434	0.000580323015593528	1081
GLTPD1	0.112556322050288	0.000208739338289149	0.000581209947454722	1081
GSK3A	0.112539607120901	0.000209196554196221	0.000582402513029695	1081

NTSR2	0.112527024088606	0.000209541366749657	0.000583201275652368	1081
ANKRD19	0.112521734868839	0.000209686466180955	0.000583524500789519	1081
ELL3	0.112512689394944	0.00020993482952022	0.000584134964182989	1081
PRSS36	0.112492515723538	0.000210489736099048	0.000585194001340784	1081
LOC11511	0.112423088257267	0.000212409971898736	0.000590206742606902	1081
PPP1R14D	0.112391726977162	0.000213282742610415	0.000592386715826114	1081
CHCHD3	0.11236994105164	0.000213891013642561	0.000593994274859305	1081
CPVL	0.112351005153035	0.000214421031936106	0.000595302051867814	1081
TCHH	0.112344140156056	0.000214613488099054	0.000595590125052411	1081
HIST1H4C	0.11231907351013	0.000215317593206726	0.000597215050963721	1081
PPP2R3C	0.112317601520781	0.000215359007650666	0.000597247688480543	1081
TMEM106	0.112311969308878	0.000215517538998513	0.000597605068565045	1081
FUT1	0.112296438531097	0.000215955254959118	0.000598736390194252	1081
H1FX	0.112250130945225	0.00021726533084037	0.000602202819177113	1081
HS6ST1	0.112234555360172	0.000217707647792081	0.000603345792374668	1081
ACCN1	0.112204465954716	0.0002185645215736	0.000605387362450182	1081
BRE	0.112187216050018	0.000219057182253182	0.000606668536773487	1081
MAP1LC3	0.112118645679103	0.000221025879342992	0.000611616255407221	1081
S100A12	0.112066171837573	0.000222543625650979	0.000615562452112999	1081
CKB	0.111828979307832	0.000229526846105974	0.000633486548130544	1081
LDLRAP1	0.111820361372426	0.000229784393706839	0.000634110494688257	1081
FAM173A	0.111751211249133	0.000231860777325703	0.000639227502289078	1081
NDOR1	0.111714693802145	0.000232964375411476	0.000641946236723527	1081
TOP2A	0.111714363413413	0.00023297438256072	0.000641946236723527	1081
MYEOV2	0.111627260938808	0.000235626728929351	0.000648633568499833	1081
EPCAM	0.111607236358066	0.000236240482137694	0.000650234255043563	1081
RING1	0.111539865595232	0.000238316411099168	0.000655589799480094	1081
ADPRH	0.111515152154215	0.000239082194302765	0.000657337355565608	1081
BGLAP	0.111381716769448	0.000243256874930926	0.000668176949206914	1081
CLDN14	0.111329599797201	0.000244905882684308	0.000672156540418989	1081
ADM	0.111281894619308	0.000246424472847946	0.000676140153299083	1081
FARSB	0.111251894680128	0.000247383968104777	0.000678680380971093	1081
SLC27A4	0.111249566222297	0.000247458585887076	0.000678792648787465	1081

CIT	0.111145380857792	0.000250818967916928	0.000687635834061855	1081
ITFG3	0.111141783419181	0.000250935759083202	0.000687862412128331	1081
DPM3	0.111099838799907	0.000252301259189806	0.000691135282346837	1081
LBX2	0.111081781675351	0.000252891245994275	0.000692657260442511	1081
FAM98C	0.111076575110827	0.000253061601748949	0.000692935431185615	1081
RASGRP2	0.11105110963324	0.000253896366729354	0.000695032247283985	1081
ARFRP1	0.111042535992847	0.000254177992654036	0.000695708649730374	1081
TMEM60	0.111014692711599	0.000255094604440357	0.000697933017309655	1081
HDGFRP2	0.111000749963619	0.0002555547674558	0.000699097065507482	1081
CPNE1	0.110991777868042	0.000255851291204111	0.000699623219941199	1081
OR2B2	0.110927750320333	0.000257976745211969	0.000704861187073797	1081
GLYATL3	0.110920647466585	0.000258213547150586	0.000705412517948	1081
TMEM141	0.110859122521481	0.000260273260264455	0.00071084664154385	1081
ZGLP1	0.110788345611802	0.000262661731981897	0.000717078274939058	1081
RNF130	0.110765631837363	0.000263432581167446	0.000718890456193199	1081
DERL3	0.110756325950653	0.000263749011091966	0.000719461588144572	1081
PPP2R5D	0.110698709365144	0.000265716094930861	0.000724631207849221	1081
LOC65356	0.110659649985886	0.000267057420615054	0.000727993469322093	1081
EHD4	0.110629483784364	0.000268097683651249	0.000730730325687244	1081
UPK2	0.110624122042422	0.00026828297659342	0.000731136439863968	1081
CYB5B	0.110607634478427	0.000268853510925648	0.000732394047004352	1081
LOC44245	0.110579522857195	0.000269828903031485	0.000734951764679458	1081
PFKL	0.110568122043942	0.000270225421604411	0.000735932285821395	1081
PIN1L	0.110554502088405	0.000270699836596259	0.000737057447479672	1081
ICAM2	0.110554160568132	0.000270711742561534	0.000737057447479672	1081
LRRN4	0.110545334876871	0.000271019590903966	0.000737696211155303	1081
DTX2	0.110402050325502	0.000276063558925523	0.000751121068956599	1081
VNN3	0.110364856553149	0.0002773871598345	0.000754416678124206	1081
C14orf166	0.110360127829158	0.000277555864052464	0.000754773607091912	1081
IPCEF1	0.1103416617518	0.000278215588707254	0.000756434302928496	1081
EPS15L1	0.110257451069782	0.000281242772119694	0.000763974601449735	1081
ISY1	0.110206306861765	0.000283096305289987	0.000768802247245455	1081
HOXB9	0.110183800051384	0.000283915597965779	0.000770923267424264	1081

ANO7	0.110164120859448	0.000284633777105804	0.000772769196738063	1081
TOX2	0.110101797534906	0.000286919457059295	0.000778555019189049	1081
PIP4K2C	0.11007846307145	0.000287779647701804	0.000780783973461662	1081
LOC15038	0.110076014097248	0.000287870065288796	0.000780924113283436	1081
TBC1D10	0.110070695309575	0.000288066529649757	0.000781351856711238	1081
C10orf113	0.110031460120006	0.000289519671886043	0.000784765041730939	1081
CD84	0.109955503429442	0.000292352366123026	0.00079212352596481	1081
LOC14688	0.109892994031516	0.000294702988578305	0.000798063140867045	1081
DNTT	0.109874716604775	0.000295393631573696	0.000799718412585957	1081
CYLD	0.109807180847827	0.00029795873629118	0.000806229515458135	1081
TBCE	0.10979648932722	0.000298366720573193	0.000807225031687749	1081
LACTB2	0.109743253429107	0.000300405971051886	0.000812305810314127	1081
CENPO	0.109741305203357	0.000300480846313017	0.000812399228154036	1081
TXK	0.10973354143022	0.000300779401219927	0.000813097294360633	1081
SF3B14	0.109683587817603	0.000302707005905704	0.000818088628115161	1081
C4orf19	0.109660530275435	0.000303600639280428	0.000820283647841232	1081
SRP54	0.109595124245497	0.00030614900161706	0.000826836256545875	1081
CD79B	0.109589254381055	0.000306378679814323	0.000827345644083049	1081
CD320	0.109555608938465	0.000307698282535979	0.000830797735114233	1081
ODF3L1	0.109545662865142	0.000308089392498524	0.000831742269081045	1081
LOC92659	0.109538405192778	0.000308375079625688	0.000832401980310798	1081
TCEA2	0.109532065781567	0.000308624823467162	0.000832964505458999	1081
C21orf45	0.109524037559055	0.000308941370159422	0.000833595486453462	1081
HTR3E	0.10950205670829	0.000309809612456546	0.000835714333548087	1081
UBA52	0.109482663456973	0.000310577538604004	0.000837566946595433	1081
MYL6B	0.109479980639469	0.000310683911748771	0.000837624116993975	1081
FBXO43	0.109452806301036	0.000311763290110983	0.000840196853416153	1081
HBE1	0.109449059729658	0.000311912380519434	0.000840373800396417	1081
GSTT2	0.109416935650334	0.000313193459370117	0.00084371252193247	1081
LAPTM4E	0.109396583572719	0.0003140076252541	0.0008457927009953	1081
LRRC29	0.109385975135999	0.00031443278993986	0.000846711476184797	1081
GUCA1A	0.109370802477896	0.000315041814773357	0.000848238085887366	1081
PTGDS	0.109355781304283	0.000315645845249726	0.000849637299913913	1081

TERF2	0.109339364834454	0.000316307221367021	0.00085130380420022	1081
ASS1	0.10931851944902	0.000317148893684291	0.000853455044519109	1081
TMX2	0.109314805516042	0.000317299070392647	0.000853745127963121	1081
WDR67	0.109303646476383	0.000317750697694748	0.000854846127812593	1081
C19orf6	0.109277926873213	0.000318793907925695	0.000857423668246078	1081
MGC7085	0.109268976189532	0.000319157706039769	0.000858172982937954	1081
SH2D6	0.109252181987353	0.000319841349232314	0.000859781689389507	1081
DGCR9	0.109245609372946	0.000320109273761411	0.000860387100723632	1081
VCX3A	0.109228229374286	0.000320818759161631	0.000862074335922247	1081
CSTA	0.109213714219695	0.000321412422147118	0.000863544044298971	1081
HSD17B1	0.109099341050708	0.000326126279809666	0.000875741656460374	1081
SLC37A2	0.109041015724337	0.000328554942692995	0.000882028161054156	1081
KIN	0.109032001352057	0.000328931805146122	0.000882922213813275	1081
IL1F7	0.1090299929086	0.000329015826839489	0.000883030086821411	1081
AMICA1	0.108996940077377	0.000330401450991461	0.000886512683833642	1081
TNFSF14	0.108977561906307	0.000331216346677767	0.000888462490522451	1081
C19orf39	0.10896527843823	0.000331733866313779	0.000889613782866224	1081
SPATA20	0.108902481488976	0.000334391415652811	0.000896382577288873	1081
FAM123C	0.108896039430976	0.000334665163893912	0.000896997036541094	1081
FAM108A	0.108871944772177	0.000335690897537479	0.000899387302951525	1081
FAM179A	0.108846721550086	0.000336767821818294	0.000901912758645246	1081
MAFA	0.108736024310213	0.000341532404315585	0.000913943980464594	1081
RAB42	0.108722686749749	0.000342110705773114	0.000915369925328646	1081
AP1S1	0.108699655267473	0.000343111478918826	0.000917803843157581	1081
EBAG9	0.108685353644336	0.00034373429444664	0.00091922572170816	1081
PES1	0.108676486763373	0.000344120964817047	0.000920015505804832	1081
HPS6	0.10866671861039	0.000344547408571958	0.0009210333792041	1081
RTN4R	0.108661008638788	0.000344796914747429	0.000921455803606655	1081
PSMD1	0.108620318872488	0.000346579810863907	0.000925974839503104	1081
TCEA3	0.108604114859947	0.000347292213779411	0.000927632146192818	1081
ZNF428	0.108351602971825	0.000358572041062752	0.00095585996654878	1081
RNPEPL1	0.10832713089676	0.000359683216609937	0.000958441587117353	1081
AFG3L1	0.108323446720964	0.000359850777982969	0.000958761264709285	1081

SDCBP2	0.108305241660763	0.00036067983967949	0.000960843079918452	1081
TUBA8	0.108298895561792	0.000360969261160613	0.000961486945138245	1081
ACE	0.108259319782513	0.000362779061026551	0.000966179823424097	1081
C7orf43	0.108246112411162	0.00036338491708829	0.000967537556799313	1081
KRT78	0.108226555920283	0.00036428375895519	0.000969674461436614	1081
MRPL16	0.108223182153605	0.0003644390314759	0.000969959610131061	1081
TARS	0.108218763428626	0.000364642490096958	0.000970372914531469	1081
NAE1	0.108198414230711	0.000365580831250726	0.000972484596005002	1081
RABIF	0.108161895393438	0.000367270441778919	0.000976592271599303	1081
SLC2A1	0.108139108843964	0.000368328393899088	0.000979276164061914	1081
SCN1B	0.108102748802716	0.000370022441002397	0.000983390774933152	1081
DVWA	0.108072628841942	0.00037143126275484	0.000987004720775129	1081
C1orf210	0.108033241760355	0.000373281098163701	0.000991527916997332	1081
RNF34	0.10800292794281	0.000374710655676794	0.000994931614420589	1081
COX7C	0.107969449491441	0.00037629539086401	0.000998744486028391	1081
LOC10013	0.107923427099141	0.000378484118983845	0.00100428906440063	1081
C10orf75	0.107896000830435	0.000379794099384584	0.00100736696933541	1081
RET	0.107864167539419	0.000381319881734241	0.0010111476921859	1081
OTUD5	0.107823727391907	0.000383266444938607	0.00101617564270706	1081
ATP6V1H	0.107764860360111	0.000386116556490987	0.00102346289875144	1081
ERCC6L	0.107732534956685	0.000387690026846999	0.00102722814557843	1081
TMEM177	0.107728331429997	0.00038789507667367	0.0010276362861114	1081
CRB3	0.107709946993939	0.000388793067149771	0.00102974445670946	1081
ATXN7L2	0.107690897442606	0.000389725591538368	0.00103180733888033	1081
ENO2	0.107635731060842	0.000392437915054737	0.00103871525407669	1081
OSR2	0.107627818930408	0.000392828365912377	0.00103961211656658	1081
KIAA0895	0.107621266262613	0.000393152003865606	0.00104008885024494	1081
ZNF513	0.107577491670747	0.00039532043309004	0.00104552056250477	1081
SH3BGR	0.107567007347612	0.000395841441000742	0.00104676106969808	1081
FAM86A	0.107562974111903	0.000396042039182745	0.00104705408914276	1081
SF3A2	0.107562689455686	0.000396056200509696	0.00104705408914276	1081
SLC7A9	0.107552236894801	0.000396576530983499	0.00104801708207564	1081
S100A3	0.107544807332145	0.000396946763746995	0.00104885789030472	1081

ALPPL2	0.107515304645131	0.000398420137975028	0.00105247491207801	1081
VPS33A	0.10744498521961	0.000401952513020669	0.00106055446952212	1081
TRMT6	0.107428696652086	0.000402774898241457	0.00106230692917965	1081
LOC10013	0.10738870501389	0.000404800678433015	0.00106681183503834	1081
EPHA8	0.107372920406671	0.000405602862123653	0.00106850655910566	1081
MTA2	0.107364145624889	0.000406049443131632	0.0010694265982281	1081
SRC	0.10736277335022	0.000406119324711092	0.00106944755507254	1081
LILRB5	0.107360129406363	0.000406253996277989	0.00106966236505295	1081
ERAP1	0.107303066174105	0.000409170732841656	0.00107706055312886	1081
RNF121	0.107297667964654	0.000409447666498442	0.00107764871199518	1081
DSCR6	0.107277733260087	0.000410471852812634	0.00108000891936657	1081
P4HA2	0.107277083967386	0.000410505251704632	0.00108000891936657	1081
HSP90AB	0.10727198735879	0.000410767503614728	0.00108055776447097	1081
C1orf68	0.107251536299669	0.000411821409024842	0.00108304729566651	1081
PLEKHA4	0.107247051371497	0.00041205286798331	0.00108351455756739	1081
CYP19A1	0.107228075109141	0.000413033541679181	0.00108580982606383	1081
FPGS	0.10718452494635	0.00041529240582756	0.00109146321140198	1081
GIMAP7	0.107174374554918	0.000415820540375339	0.0010925661648443	1081
C11orf85	0.107149542045015	0.000417115241458457	0.00109582505727447	1081
TXNL4B	0.107118601567626	0.000418733656094646	0.00109964665650197	1081
ZC3H18	0.107108864917618	0.000419244164688597	0.00110084380834877	1081
UCHL1	0.107070469130133	0.000421262981822255	0.00110600062150519	1081
DNAJC5B	0.107047529166145	0.00042247346749958	0.0011088896420559	1081
HOXB7	0.107004330743596	0.000424761751888286	0.00111416998591009	1081
MYBL1	0.106958538510972	0.000427200049632246	0.00112022348326748	1081
LOC10000	0.106951761650543	0.000427562003572758	0.00112093135892416	1081
DGKG	0.1069040731584	0.000430117155962328	0.00112748342314393	1081
CFP	0.106885531241092	0.000431114471379719	0.00112995069294099	1081
LOC72855	0.106850477179874	0.000433005820074278	0.0011347602764923	1081
LIPC	0.10684046049325	0.000433547691734631	0.00113588480296451	1081
HIST2H3I	0.106811828386614	0.000435100088116616	0.00113980380690627	1081
OR7E91P	0.106796279344446	0.000435945307093138	0.00114186948529336	1081
SERPINB6	0.106755628748582	0.000438162240214823	0.0011473779187739	1081

PTGES	0.106743609079931	0.000438819758879008	0.00114867260400258	1081
ATP6V1C	0.106626619336671	0.000445267733033008	0.00116462197896	1081
MGC1627	0.106620830100289	0.000445589092749371	0.00116531121296068	1081
MYOG	0.106611788644335	0.000446091415200628	0.00116632207128055	1081
S1PR2	0.106563400018748	0.000448788758618218	0.00117291768842294	1081
IMP3	0.106558621124785	0.000449055973239278	0.0011733293562146	1081
ARL2BP	0.106558499752028	0.000449062761797695	0.0011733293562146	1081
EIF3K	0.106542776325216	0.000449943005608732	0.00117532440974947	1081
SFXN1	0.106521045113584	0.000451162230227863	0.00117835642784134	1081
MGMT	0.106501754593264	0.000452247099385139	0.00118088370928239	1081
LOC1001[illegible]	0.106485128551853	0.000453184072459783	0.00118317692323773	1081
RGS14	0.10647525272347	0.000453741488311501	0.00118447871997346	1081
NOL12	0.10646557871638	0.000454288132299546	0.00118543868113905	1081
ZNF688	0.106464579941656	0.000454344604471313	0.00118543868113905	1081
BUB1B	0.106439633277615	0.000455757249148881	0.00118866258209531	1081
ST8SIA4	0.106387241105655	0.000458737373913285	0.00119566106837665	1081
PITPNC1	0.106375213622475	0.000459424068340056	0.00119714110163115	1081
SLC47A1	0.106365208792942	0.0004599960114563	0.00119847641629425	1081
DMAP1	0.106356192980041	0.000460511983998852	0.00119966557838573	1081
HNRNPA[illegible]	0.106348644347038	0.000460944404956267	0.00120048158213885	1081
ATP5L	0.106332350287915	0.000461879094816313	0.00120276038845329	1081
ISL2	0.10628969842413	0.000464334137916334	0.00120868474070611	1081
ZNF367	0.106227931829631	0.000467910984805462	0.00121752348087136	1081
ACHE	0.106219093224105	0.000468424914750267	0.00121854595914826	1081
TPST2	0.106216162350369	0.000468595449533642	0.0012188321924926	1081
KIF22	0.106207425508196	0.000469104151604331	0.00121999782262706	1081
LRRC16B	0.106198462903241	0.000469626533254337	0.0012211987236877	1081
ZFPM1	0.106188371466123	0.000470215356994758	0.00122257206590854	1081
TLR9	0.10614771633949	0.000472594510561788	0.00122844082777642	1081
FBXW10	0.10613594302819	0.000473285580310181	0.00123007844347163	1081
TSPAN32	0.106092456946873	0.000475846294294004	0.00123593651348024	1081
KIAA065[illegible]	0.106073553777639	0.000476963442449459	0.00123819955517324	1081
PHF19	0.106048620212916	0.000478440714245594	0.00124184350051658	1081

SLC22A18	0.105967253763004	0.000483291239086678	0.00125365722526412	1081
GDA	0.105961414025566	0.000483641120878743	0.00125440329343405	1081
GSG2	0.105933275121945	0.000485330343539062	0.00125829855477405	1081
GNB1L	0.10591586277787	0.000486378384361641	0.00126052908181722	1081
KRT18	0.105914652195115	0.000486451327076346	0.00126055595368575	1081
C1orf91	0.105912992675313	0.000486551336696472	0.00126065294890681	1081
WDYHV1	0.105896647132388	0.000487537411290456	0.00126304541543804	1081
EIF2B4	0.105888649517047	0.000488020559766025	0.0012641345218576	1081
CSE1L	0.105870991886047	0.000489088864386726	0.00126673890114047	1081
PROCR	0.105846186713252	0.000490593281494371	0.00127047199584832	1081
PLA2G4F	0.105831775557733	0.000491469286335212	0.00127257696313918	1081
VARS2	0.105827775150668	0.000491712715559695	0.00127304365183782	1081
NAPSA	0.105807353997035	0.00049295711594965	0.00127610140077174	1081
CMKLR1	0.105784879254538	0.00049433004494547	0.0012793266643662	1081
MUS81	0.105781321935226	0.000494547679292546	0.00127972549766838	1081
C1orf159	0.105772210327306	0.000495105528194398	0.00128058682404226	1081
HDGF	0.105771824475262	0.000495129164518927	0.00128058682404226	1081
CCK	0.105771732297667	0.000495134811241754	0.00128058682404226	1081
TERT	0.105748189131633	0.000496579011188347	0.00128399232195986	1081
FDPS	0.10568862723764	0.000500250227344883	0.00129298701948456	1081
WNT1	0.105654696913049	0.000502352872823424	0.0012980163765063	1081
RPL38	0.105649470958338	0.00050267745296489	0.00129859416388532	1081
HIST1H3J	0.10563880665079	0.000503340410981495	0.0013001400922198	1081
CHEK1	0.10555944640019	0.000508299553377225	0.00131244482219741	1081
BYSL	0.105556445283902	0.00050848797968342	0.0013127630848036	1081
ARHGAP1	0.105537552486059	0.000509675665612983	0.00131549215679354	1081
SFPQ	0.10552539529849	0.000510441285877843	0.0013172994752766	1081
TOP1MT	0.105481437074095	0.000513218575634847	0.00132395802358356	1081
GABARA	0.105418752577356	0.000517203332663059	0.00133338381577903	1081
C1orf216	0.105397087402888	0.000518587237651487	0.00133678053774654	1081
CABP7	0.10536864164496	0.00052040949917459	0.00134113462464783	1081
CEND1	0.105353580854059	0.000521376718623748	0.00134345535900171	1081
10-Mar	0.10534530133383	0.00052190915034274	0.00134452504387593	1081

C20orf201	0.105225000419809	0.000529702664133155	0.00136386249603303	1081
GNB2	0.105201995701011	0.000531205270305542	0.00136720712537436	1081
NOXO1	0.105168889279818	0.000533374654818685	0.00137208944213031	1081
TNFAIP3	0.10514040740388	0.000535247597935489	0.00137638024769089	1081
PIGZ	0.105120189288321	0.000536580835295358	0.00137963253695277	1081
ZNF707	0.105068978530934	0.000539971649461831	0.00138764241337015	1081
MAP2K3	0.105040635904579	0.000541856857233601	0.00139230948838914	1081
WRAP53	0.105006234778654	0.000544153272261331	0.00139785356665449	1081
FKBP4	0.104989727138743	0.000545258436189381	0.00140051398661674	1081
WDR61	0.104980907130521	0.000545849779233645	0.00140185413088498	1081
PCGF5	0.104974694284455	0.000546266681526917	0.0014027459909955	1081
EIF3B	0.10496441835901	0.0005469568799435	0.00140416036019648	1081
BRI3BP	0.104915880734036	0.000550227964055213	0.0014120181319608	1081
NCR2	0.1049019873425	0.000551167622477634	0.00141406925048547	1081
ZNF385A	0.104898645134044	0.000551393890296351	0.00141446961925633	1081
ADCY7	0.104832375791963	0.000555898211313502	0.00142475438510312	1081
SNRNP27	0.104788871525813	0.000558873775668229	0.00143219847485517	1081
SERINC4	0.104758273859776	0.000560975432527371	0.00143703587083722	1081
LAX1	0.104726522994161	0.000563164068592578	0.00144190901903883	1081
LOC44089	0.104716562751287	0.00056385227813855	0.00144348762779274	1081
IQGAP3	0.104701002050962	0.000564929020643481	0.00144606037113887	1081
BCCIP	0.104679984445097	0.000566386396541341	0.00144923840446149	1081
TNNT1	0.104672420307938	0.000566911754292581	0.00145039843665533	1081
TNFSF4	0.104671323474857	0.000566987971081108	0.00145040922887986	1081
DSCR10	0.104635519893086	0.000569481130666338	0.00145623221341373	1081
CRYBB3	0.104625446187098	0.000570184442259038	0.00145784561356877	1081
CHEK2	0.1045996492274	0.000571989181794191	0.001462088829748	1081
RSAD1	0.104594364696323	0.000572359539261701	0.00146284990084077	1081
SBSN	0.10459290902268	0.000572461596864052	0.00146292513875762	1081
RAX	0.104573193056964	0.000573845555346886	0.00146627583871931	1081
UBASH3E	0.104566838938681	0.000574292243953778	0.00146723110392503	1081
GLRX5	0.104524956624545	0.000577244630156023	0.00147346592428954	1081
SSBP4	0.104508519721754	0.000578407158675866	0.00147610057167626	1081

MRPS28	0.104508291342422	0.000578423326523325	0.00147610057167626	1081
LOC3894:	0.104502451003181	0.000578836929302138	0.00147696896020159	1081
C20orf202	0.104476781683723	0.000580658048678183	0.00148124052052957	1081
RNF135	0.104445894708947	0.000582856397527902	0.00148666018336282	1081
DDTL	0.104423201872282	0.000584476465212591	0.00149060367030101	1081
PNKD	0.104389416956748	0.000586896166555752	0.0014962064382771	1081
OLFML3	0.104377371380963	0.000587761129575144	0.0014980323830075	1081
FAM131A	0.104369228462732	0.000588346523588053	0.00149933468914375	1081
MITD1	0.104356963716026	0.000589229258710821	0.00150120442857335	1081
HHIPL2	0.104346536531612	0.000589980706449452	0.00150254884088802	1081
DCLRE1C	0.104329757052779	0.00059119181051379	0.00150525265707789	1081
PTH2	0.104302399509479	0.000593171371682395	0.00150991120577986	1081
LOC1002	0.104263227461439	0.000596016546337455	0.00151688533237578	1081
SDCCAG:	0.104243170080658	0.000597478275157611	0.00152010606883684	1081
FAM72D	0.104188133547992	0.000601506325504708	0.00152919547290413	1081
WWOX	0.104160353649346	0.000603549070742782	0.00153361453457535	1081
PLEKHN1	0.104138921451218	0.000605129453053094	0.0015372424756311	1081
C12orf48	0.104136089833436	0.000605338540242079	0.00153744413452248	1081
FXYD5	0.104097235398936	0.000608214348777542	0.0015443002339455	1081
DSCR4	0.104032040142622	0.000613068313602861	0.00155564443601582	1081
CCDC159	0.104013332209279	0.000614467802718728	0.00155899922994569	1081
FABP6	0.103983536262973	0.000616702886225788	0.00156427595605874	1081
GMNN	0.103979720061481	0.000616989696076438	0.00156480642420494	1081
KIAA1751	0.103966659539472	0.000617972208861359	0.00156710097526587	1081
THTPA	0.103963967318772	0.000618174919020099	0.00156741771474637	1081
CACNG3	0.103957962916248	0.000618627241754057	0.00156836720175377	1081
NOTO	0.103954734049973	0.000618870605346509	0.00156878675534232	1081
PELP1	0.103931221419657	0.00062064546525162	0.00157269218836401	1081
SERPINI1	0.103923112444759	0.000621258670322362	0.00157404803341014	1081
GALNT14	0.103868437593843	0.000625407927976741	0.00158356494583854	1081
PPP5C	0.103860872190903	0.000625984087591189	0.00158482461285968	1081
CARNS1	0.103851615805149	0.000626689698441971	0.00158621233510661	1081
PACSIN3	0.103846753251602	0.000627060665072873	0.00158695189672023	1081

TGM3	0.103819840902588	0.000629117513416449	0.00159195733045778	1081
RELL1	0.103812786997852	0.00062965766274844	0.00159312404120414	1081
AMN	0.103768661904989	0.000633046309158604	0.00160149665929927	1081
NDUFAF1	0.103747006167517	0.000634715581247309	0.00160511491140184	1081
NT5C3L	0.103724878187729	0.000636425477689573	0.00160879102869087	1081
ARHGEF1	0.103713204835379	0.00063732923630528	0.00161071351968007	1081
RFPL3S	0.103685617027835	0.00063946984249836	0.00161592071965999	1081
STX4	0.103613982669017	0.000645059353036139	0.00162902352600138	1081
KDELR2	0.103605201940641	0.000645747612113515	0.00163055723815828	1081
LCN12	0.103589800419179	0.000646956475838203	0.00163325637430348	1081
NPM3	0.103576043129781	0.000648038060846048	0.00163572569048285	1081
AVEN	0.103570269842751	0.000648492451231132	0.00163666755575685	1081
HKDC1	0.103559232549108	0.000649361972460729	0.00163865676252304	1081
PDHA1	0.103509666186496	0.000653280191152571	0.0016475124500211	1081
LOC28299	0.103485087893778	0.000655231236471684	0.00165201918131691	1081
GML	0.103470640729055	0.00065638058811586	0.00165408893515061	1081
C1orf105	0.103455205556013	0.000657610609707384	0.00165677410362122	1081
OXA1L	0.103367649369912	0.000664628522861837	0.00167361769913146	1081
DGCR14	0.103360029807414	0.000665242533248319	0.00167495448472533	1081
FLJ44054	0.103348851288018	0.000666144289747877	0.00167680578745108	1081
NTNG2	0.103285886627494	0.000671244802251967	0.00168858972794279	1081
KPNA7	0.103179740298815	0.000679925446703463	0.00170850668876653	1081
EXT1	0.10315622745489	0.000681862372395283	0.00171294643869597	1081
POMC	0.103137705183237	0.000683391793282879	0.00171593265308284	1081
AKAP8L	0.103078769489091	0.000688279442733728	0.00172712872120964	1081
TGIF2	0.103050705760873	0.000690618206819309	0.00173256585010896	1081
HSF5	0.103028987974854	0.000692433175671859	0.00173690279216904	1081
FADS6	0.103015158944648	0.000693591182193601	0.00173959093193353	1081
CAPN3	0.102940751608704	0.000699852768974736	0.00175376713907153	1081
CLSPN	0.102915738647439	0.000701969434161357	0.00175863378325837	1081
DPP7	0.102914338436566	0.000702088099243967	0.00175871235504473	1081
EEFSEC	0.102861438501072	0.00070658492392433	0.00176909685464276	1081
MORN3	0.102821380997176	0.000710007827102714	0.00177722510896921	1081

RTN4IP1	0.102803675727043	0.000711525635456324	0.00178080307196765	1081
ABHD14A	0.102786900861037	0.00071296646059843	0.00178374433044652	1081
MAGEA6	0.10269804552585	0.000720643665745779	0.00180183277230343	1081
LYZL2	0.102684526863326	0.000721818393526642	0.00180454598381661	1081
CETP	0.102612591117452	0.000728099309889603	0.00181957084700732	1081
GNAZ	0.102609296405828	0.000728388191229913	0.00182006699483089	1081
TBK1	0.102589754303326	0.000730103835598275	0.00182351303752575	1081
STX10	0.102575555347256	0.00073135274378274	0.00182634201357423	1081
DCUN1D5	0.102567298808621	0.000732079881227056	0.00182770469789553	1081
ZNF653	0.102560199087716	0.000732705676150156	0.0018290403774529	1081
NECAP2	0.102548321075969	0.000733753756435388	0.00183120285225358	1081
FUCA1	0.102491064163277	0.000738825471525011	0.00184294689498159	1081
CLDN7	0.102463708039335	0.000741260089175723	0.00184810451688675	1081
GRB2	0.102455318446897	0.000742008228752895	0.00184974084497303	1081
C11orf82	0.102449918547129	0.000742490133687899	0.0018507131580231	1081
S100A6	0.102430990140874	0.00074418166037316	0.00185447050324311	1081
KLF16	0.10239801196513	0.000747137277401884	0.00186160549823883	1081
MC1R	0.102395768117951	0.000747338773699619	0.00186187726888187	1081
C1orf57	0.102374156079272	0.000749282105376099	0.00186619483226115	1081
GPHA2	0.102357172224484	0.000750812562917501	0.00186960680840211	1081
GYS1	0.102344460923241	0.000751959906208061	0.00187223239532337	1081
WFDC2	0.102325598903302	0.000753665420272663	0.00187624689710737	1081
KRT38	0.102308056357646	0.000755254845555309	0.00187950696278094	1081
FCRLA	0.102305868588398	0.000755453284104345	0.00187976857809808	1081
IK	0.10228897506943	0.000756987215943086	0.00188335278068093	1081
C5orf60	0.102248118808077	0.000760708911654809	0.00189191727791914	1081
RPP30	0.102219775456522	0.000763300735837316	0.00189812286426895	1081
TSPAN10	0.102201140113853	0.000765009281451236	0.00190190200849502	1081
C19orf33	0.102176292648688	0.000767292889683511	0.00190640296776941	1081
C10orf99	0.102125485112301	0.000771982047304626	0.00191734414288641	1081
C19orf63	0.102124355568725	0.000772086597017832	0.00191736741825989	1081
RRP12	0.102099746412177	0.000774367663180153	0.00192278137762146	1081
FKBP10	0.102073118594343	0.000776842878932029	0.00192775311604899	1081

PPME1	0.102032085191166	0.000780671543738539	0.00193677687790799	1081
TSFM	0.101980862466307	0.000785475479535333	0.00194749581972176	1081
PIM3	0.101946175128016	0.000788744184929893	0.00195511893754309	1081
SLC27A3	0.101944879645431	0.000788866506520757	0.00195518156666593	1081
THEMIS	0.101908078258091	0.000792348706866309	0.00196308752918728	1081
POU2F2	0.101896360719866	0.000793460423888359	0.00196560012779525	1081
SLC19A1	0.101891681010127	0.000793904821408685	0.00196645919430443	1081
CRIP1	0.101890461917726	0.000794020627491022	0.00196650424647242	1081
TPP1	0.101862016956116	0.000796727167732648	0.00197296481794397	1081
MAGEA2	0.101858952599834	0.000797019251063774	0.0019734455276155	1081
TPM3	0.101854664322407	0.000797428160730459	0.00197421534938123	1081
AQP8	0.101728212807502	0.000809573726148667	0.00200256172805316	1081
SPNS1	0.101700890009346	0.000812220479094346	0.00200861546174265	1081
WASH5P	0.101611635705561	0.00082092250009843	0.00202913911220649	1081
NAT14	0.101572776211516	0.000824738105074974	0.00203832034434246	1081
CD209	0.101559301015346	0.000826065063518818	0.0020405984922853	1081
NUBP1	0.101542498459613	0.000827722451630506	0.00204419134339788	1081
FHOD1	0.101531427140737	0.00082881619905358	0.00204664161926138	1081
MYO19	0.101525331024149	0.000829419012346711	0.00204787915231333	1081
RBM10	0.101515779711112	0.000830364308818328	0.0020497106973588	1081
MUC20	0.101495750214237	0.000832349876246039	0.00205410857000814	1081
ZNHIT3	0.101494652842338	0.000832458788081547	0.00205412570870931	1081
KRT8	0.101471837940447	0.000834726111206719	0.00205896381905955	1081
HEATR3	0.101461123339239	0.000835792890881057	0.00206134277507332	1081
CHMP6	0.101438476813975	0.000838051807471429	0.00206655932715602	1081
KRT31	0.101369882297559	0.000844928437114968	0.00208208970834019	1081
SYMPK	0.101355362374079	0.000846390751652593	0.00208492806212295	1081
OPALIN	0.101343530144158	0.000847584119103013	0.002087357222412	1081
SRRD	0.101326965774859	0.000849257375607935	0.00209122232387506	1081
C19orf57	0.10131933420887	0.0008500293103235	0.00209286732540539	1081
TBC1D3C	0.101286821589409	0.000853325256447131	0.0021002122530394	1081
SIRPB2	0.101272688879297	0.000854761638095981	0.00210349049590075	1081
GPR158	0.10126919218999	0.00085511737038332	0.0021041088831528	1081

KIF15	0.101249670186341	0.000857105943181331	0.00210848690015727	1081
MMP25	0.101224661090851	0.000859659705636257	0.00211451096093314	1081
RAB1A	0.101191715476263	0.000863034648037087	0.00212125829486422	1081
KIAA1524	0.101186564694624	0.000863563400787526	0.0021222989763163	1081
SUMO1	0.101180697880935	0.000864166023115142	0.0021235209240857	1081
MYL12B	0.101126762976409	0.000869724335528666	0.0021357363280804	1081
PGK1	0.101102215785473	0.000872265014658968	0.00214133301490433	1081
LOC72967	0.101083579308634	0.000874198511203418	0.00214555665304494	1081
BTC	0.101074825903818	0.000875108028051399	0.0021475272536357	1081
RELA	0.101066918503815	0.000875930394411002	0.00214892293081168	1081
IL13	0.101058410342321	0.000876816038770114	0.00215040894826199	1081
MT1E	0.101026222402571	0.00088017410066564	0.00215838189384167	1081
F5	0.100996560433634	0.000883279173932964	0.0021652055194548	1081
SEPT6	0.100977272512237	0.000885303709153158	0.00216964029451221	1081
WFDC3	0.100964830973627	0.000886611899651673	0.00217231777164716	1081
DOK1	0.100905762719239	0.000892847200358911	0.00218679718555991	1081
PRPSAP1	0.100875674986417	0.000896038870287566	0.00219381417622059	1081
TRIM11	0.100871293501429	0.000896504532777627	0.00219468754560764	1081
HSPA1B	0.100852554375082	0.000898498648452421	0.00219903477986563	1081
FAM164C	0.100840886491616	0.000899742354124197	0.00220181119094169	1081
CCDC17	0.100802867099922	0.000903805989286936	0.00221094980621558	1081
PRKRIP1	0.100787780047191	0.000905423244641317	0.00221409944929587	1081
MATK	0.100708087150614	0.000914010445581222	0.00223414947824869	1081
ZC3HAV1	0.1007075736443	0.000914066021331632	0.00223414947824869	1081
PLAC2	0.100632803331641	0.00092219172121955	0.00225198315490038	1081
DDX19B	0.100628020590168	0.000922713755643023	0.00225282615530587	1081
ARL16	0.100624999931722	0.000923043600241165	0.00225335837698234	1081
PIK3CD	0.100611511688616	0.000924517797637982	0.00225668375541223	1081
LDOC1	0.100593808048839	0.000926456023037633	0.00226114085099262	1081
NEK8	0.100581156725654	0.000927843414652116	0.00226425264544723	1081
TUBGCP2	0.100573989317248	0.000928630272364504	0.00226589835716848	1081
MRPS17	0.100571971597489	0.00092885189450529	0.00226616463785989	1081
SLC1A5	0.100537714427272	0.00093262210732845	0.00227426127008009	1081

NDUFC2	0.100513804691606	0.000935261905286139	0.00227979460222438	1081
CENPH	0.100513063246667	0.000935343876266295	0.00227979460222438	1081
SAMD10	0.100495613693207	0.000937274944225309	0.0022833962693698	1081
CD58	0.100412443657465	0.000946529806958672	0.0023040870669518	1081
VBP1	0.100410951288502	0.000946696641802299	0.0023040870669518	1081
ALAS1	0.100410050497272	0.000946797356179051	0.0023040870669518	1081
YIPF2	0.100355691723643	0.000952893402081543	0.00231836202716578	1081
ZDHHC19	0.100343913783268	0.000954219011450876	0.00232130684526964	1081
MAST1	0.100329377685561	0.000955857403585183	0.00232501175986761	1081
QRICH2	0.100291570949162	0.000960130867334108	0.00233427906377572	1081
TPMT	0.100251844411848	0.000964640369328862	0.00234439380385208	1081
ANAPC7	0.100242154609973	0.000965743261183038	0.00234679107316433	1081
PAIP2	0.10015326842532	0.000975914825762899	0.00236979317321524	1081
FITM1	0.10013579500153	0.000977925987248803	0.00237439062469894	1081
SMYD5	0.100132349631131	0.000978322995617249	0.00237478211190619	1081
SNAP25	0.100098093447859	0.000982278434682463	0.002383692807284 7	1081
ADM2	0.100064747973261	0.000986142915633644	0.00239203480258155	1081
POU3F1	0.100023644712809	0.00099092580660945	0.0024030577072526	1081
ZFR2	0.0999985537389167	0.00099385599583861	0.00240958346806701	1081
NFKBID	0.0999565310936388	0.000998781459156596	0.00242094242506433	1081
KCNK15	0.0999302386232368	0.00100187465945305	0.0024278557698402	1081
PFN4	0.0999140396778797	0.001003784804701	0.00243219207249239	1081
PPAN	0.0998204307987953	0.00101488906269279	0.00245732453941663	1081
BCAR1	0.099805205104378	0.00101670589464474	0.00246142774277349	1081
NCAPH2	0.0997908210711365	0.00101842505506853	0.00246499732480544	1081
RUNDC3A	0.0996148668916794	0.00103967345110111	0.00251340713697729	1081
GNL1	0.0996055494925388	0.00104080996996115	0.00251555091120184	1081
C2orf74	0.0995751978693745	0.00104452016985503	0.00252300465488363	1081
DPF2	0.0995713170225638	0.00104499544700665	0.00252385005154645	1081
C17orf28	0.0995679641731193	0.00104540622259149	0.00252453948143198	1081
GAGE4	0.0995611789843878	0.00104623796901542	0.00252563977061901	1081
TMEM140	0.0995014008050715	0.00105359220472958	0.00254187005560208	1081
MAGEB6	0.0994950120868418	0.00105438099808015	0.00254316393753887	1081

FCRL2	0.0994743184969092	0.00105693971279033	0.00254842016925927	1081
DAXX	0.0994716383475596	0.00105727152637628	0.00254891513868479	1081
C17orf88	0.0994283756292476	0.0010626409624471	0.00256124697158372	1081
SLC38A5	0.0993934710759939	0.00106699139708078	0.00257080991438732	1081
RAB39B	0.0993909306600366	0.00106730867096564	0.00257126682332011	1081
NKAIN1	0.0993341621102095	0.00107442129850204	0.00258623695284067	1081
PSMC6	0.0992999504910191	0.00107872884777121	0.00259598526321241	1081
B3GNT9	0.0992988632936176	0.00107886599681573	0.00259600519659016	1081
FAH	0.0992758386782067	0.00108177431791585	0.00260269242020958	1081
INHA	0.0992564055242178	0.00108423462214157	0.00260830027024622	1081
TRMT2A	0.0992357720457434	0.00108685254768685	0.00261397380290731	1081
HIST1H4A	0.099209767549388	0.0010901602503408	0.0026213780124087	1081
AHSP	0.0991897054144509	0.00109271845088154	0.00262710625632384	1081
C12orf60	0.0991473914732423	0.0010981322536467	0.00263952681657472	1081
RRP8	0.0990572311158111	0.00110975042089599	0.00266427389214035	1081
C9orf71	0.099037509730982	0.00111230682306257	0.00267009305893654	1081
AKTIP	0.0989118317082627	0.00112872610473603	0.00270435149618309	1081
APOA1	0.0988797514154373	0.00113295295234439	0.00271318797253658	1081
CORT	0.0988371076598303	0.0011385942478757	0.00272572562370243	1081
CD300LB	0.0988160061216836	0.00114139532674643	0.00273210656574464	1081
KCNF1	0.0987716651740882	0.00114730200982893	0.00274492239710264	1081
UAP1L1	0.0987322492326309	0.00115257630405348	0.00275690449360691	1081
PMFBP1	0.0986602886832908	0.00116226319505011	0.00277842554459291	1081
XAB2	0.0986470085006028	0.00116405908400964	0.00278238849636619	1081
RPS26	0.0986326838636725	0.0011659990859702	0.00278603387342778	1081
SLC8A2	0.0986253723836072	0.00116699043779278	0.00278807191287187	1081
CD200R1	0.0986155213951422	0.00116832734748142	0.0027909349478256	1081
GTF2E2	0.0985321870331416	0.00117969360486663	0.00281547924461643	1081
WBP1	0.0985314591449833	0.00117979333197266	0.00281547924461643	1081
MANEAL	0.0985309739991087	0.00117985980556227	0.00281547924461643	1081
ACOT1	0.0985014359807643	0.00118391355468794	0.00282481802193399	1081
KPTN	0.0984772352883347	0.00118724439135729	0.00283242992229899	1081
SCLT1	0.0984495021895211	0.00119107203720705	0.00284055240790056	1081

MAGEB1(	0.0984233846364103	0.00119468711647543	0.00284883664315785	1081
RAB3IL1	0.0984125913614413	0.00119618402903795	0.00285206856018103	1081
LOC1454	0.0983830610754588	0.0012002884139685	0.00286117738721992	1081
ACP1	0.0983203070723426	0.00120905370501432	0.00288138967082853	1081
CDHR2	0.098303633886426	0.00121139247335249	0.00288593914092786	1081
C7orf27	0.0982851539432804	0.0012139895532425	0.00289178426747904	1081
CDKN1A	0.0982617363030779	0.00121728793008951	0.00289928280165111	1081
RPL22L1	0.0982607620877547	0.00121742532733516	0.00289928280165111	1081
KRTAP10	0.0982552764761393	0.0012181992508177	0.0029007829678159	1081
RINL	0.0982278992535166	0.00122206848444849	0.00290930862907289	1081
LOC6537	0.0982246484258154	0.00122252867730963	0.0029097164702744	1081
GAR1	0.0982193870756397	0.00122327382194368	0.00291114602989431	1081
RNH1	0.0982174860349176	0.00122354316197891	0.00291144306615463	1081
MSLNL	0.0981766505818014	0.00122934196418833	0.00292385996087059	1081
ALOX12P	0.0981628916719623	0.0012313014876137	0.00292782913927974	1081
NUCB2	0.0981372742038102	0.00123495756569439	0.00293617610774383	1081
STAMBPI	0.0980921380984368	0.00124142367704509	0.00294946101828912	1081
ARFGAP2	0.0980808208841368	0.00124304984743791	0.00295297631799961	1081
TAC3	0.0980756814218635	0.00124378898509382	0.00295403549925903	1081
ZDHHC4	0.0980543946714026	0.00124685468108603	0.00296061852315866	1081
MACROD	0.0980095340051327	0.00125333829631129	0.00297426080565331	1081
WASH2P	0.0979879231510487	0.00125647274842379	0.00298134788186068	1081
MMP12	0.097977246427786	0.00125802397319667	0.00298467706277788	1081
C6orf114	0.0979458665853985	0.00126259338842326	0.00299481264686054	1081
SLC12A5	0.0979230394833938	0.00126592699465532	0.00300236629471763	1081
RASGRF1	0.0979115767256859	0.00126760404252595	0.00300563599772661	1081
WFDC10A	0.0978881794674613	0.00127103351506848	0.0030127038664613	1081
PRKACA	0.0978621547595347	0.00127485815538821	0.00302141382827005	1081
KLHL22	0.0978332242931543	0.00127912228207258	0.00303009388197932	1081
DCPS	0.097831409872679	0.00127939015124737	0.00303037208664059	1081
TPRKB	0.09781598822785	0.00128166899050606	0.00303502873819375	1081
SC65	0.0977968692066272	0.00128449938161987	0.00304068625649029	1081
MBLAC1	0.0977482561958507	0.00129172204971426	0.00305670629525359	1081

STMN4	0.0977294388398737	0.00129452786985415	0.00306298613321727	1081
VCX	0.0976979884642504	0.00129922991861612	0.00307266808059658	1081
ENG	0.0976937894592509	0.0012998588886338	0.00307343395675211	1081
FAM83C	0.0976843066674169	0.00130128035105768	0.00307643383077772	1081
NAIF1	0.0976828892731849	0.00130149294027488	0.00307657536750029	1081
CIRH1A	0.0976801653776789	0.00130190157618987	0.00307681924593441	1081
VKORC1	0.0976534315272401	0.00130591844006647	0.00308558843245825	1081
DOM3Z	0.0976518860453039	0.00130615100354054	0.00308577600167986	1081
CYP2C18	0.0976337789786331	0.00130887859205081	0.0030914948102783	1081
C16orf91	0.0976296078425106	0.00130950766159319	0.00309225552019633	1081
ABHD4	0.0976000823841908	0.00131396851371936	0.00310133517385782	1081
C9orf86	0.0975962856779961	0.00131454315387862	0.00310232800314958	1081
C19orf40	0.0975853910460915	0.00131619336726561	0.00310585866036848	1081
MAP1LC3	0.0975532461344539	0.00132107349948338	0.00311627934977665	1081
ATP2A3	0.0975391647984509	0.00132321652762607	0.00312060371681423	1081
C20orf4	0.0975363403500337	0.00132364676385987	0.00312125296242036	1081
C3orf26	0.0975244628590394	0.00132545742275553	0.00312479108032886	1081
GUCY1B2	0.0975143642966946	0.00132699868846997	0.00312805857468144	1081
MPST	0.0974813885051882	0.00133204303690083	0.00313958195605091	1081
FAM127A	0.0974598985354714	0.00133533988686717	0.0031466162148718	1081
CDK5RAI	0.0973196609343117	0.00135703948891864	0.00319475990467057	1081
APOA2	0.0972689554378092	0.00136496500657778	0.00321191683886338	1081
MYT1	0.0972076732440483	0.00137460060919412	0.00323345741151513	1081
ALKBH7	0.0972008231246359	0.00137568156003478	0.00323562230319915	1081
ACOT6	0.097149255065055	0.00138384417939543	0.00325254240974459	1081
LOC67865	0.0971041887074822	0.00139101415363011	0.00326786940231821	1081
EMX1	0.0970496321958095	0.00139973976303705	0.00328721817747509	1081
SOAT1	0.0970443615519755	0.00140058539867989	0.00328882070828842	1081
C11orf51	0.0970304760670857	0.00140281547420941	0.00329367339486581	1081
SIP1	0.0970160080598462	0.00140514258340393	0.00329875274940824	1081
CCDC63	0.0970013202037717	0.00140750868971282	0.00330392246029653	1081
ZNF771	0.0969972146579876	0.00140817071885536	0.00330509135865563	1081
HHLA2	0.0969723524313409	0.00141218594178285	0.0033133573022613	1081

IFT27	0.0969335427057407	0.00141847474528147	0.00332656271754309	1081
YARS2	0.0968878325499429	0.00142591477090787	0.00334284336785047	1081
PDX1	0.0968705904384095	0.00142873050676452	0.00334866504465053	1081
SPRN	0.0968498119952862	0.00143213054649714	0.00335624358529374	1081
EFTUD2	0.0968266526251421	0.00143592896104676	0.00336475385835606	1081
F12	0.0968019491852668	0.00143999084161638	0.00337387944921632	1081
HTR3D	0.0967805307935784	0.00144352113320459	0.00338175755650733	1081
C16orf53	0.0967775586226487	0.00144401165094715	0.00338251333817794	1081
GNG8	0.0967632923558231	0.00144636824703998	0.00338724579593356	1081
IGLL1	0.0967451588181148	0.00144936877374596	0.00339348372235151	1081
DOK7	0.0967142873913097	0.00145449020059438	0.00340468337101716	1081
DNM2	0.096632548087915	0.00146813091154503	0.00343581519668619	1081
C1orf97	0.0966192335835738	0.00147036396278731	0.00343984229826388	1081
SNAPC2	0.0965530198811058	0.00148151553297289	0.00346552838036912	1081
LIPA	0.0965408222517267	0.00148357830063798	0.00346995064046813	1081
MED29	0.096494454095494	0.00149144386105945	0.00348794248677067	1081
BAT4	0.0964693975686568	0.00149571022126313	0.00349710798599648	1081
SAFB	0.0964561405022877	0.00149797203345022	0.00350117723794579	1081
ITLN2	0.0964377140743118	0.00150112102655602	0.00350690978545415	1081
DHRS9	0.0963471550958566	0.00151668584633503	0.00354039818938808	1081
DPP9	0.0963302609146182	0.00151960591230977	0.00354639262088511	1081
PPP1R3F	0.0963085502486803	0.00152336607415364	0.00355475611766767	1081
FLJ45445	0.0962817664903329	0.00152801665745372	0.00356519522404507	1081
WDR18	0.0962776119348399	0.00152873920021339	0.00356646800096107	1081
TSKU	0.096271604222695	0.00152978459195184	0.00356849358555694	1081
C5orf34	0.0962596928881549	0.00153185920736882	0.0035725056416352	1081
TXNDC2	0.0962349025231504	0.00153618528304551	0.00358176533876756	1081
C6orf10	0.0962161780248629	0.00153946027215248	0.00358815540697809	1081
C19orf47	0.0962008179868214	0.00154215159674101	0.00359401248453814	1081
HNRNPA:	0.0961119099654496	0.0015578149014418	0.00362925652706662	1081
GALP	0.0960883351189446	0.00156199265062712	0.00363772739270328	1081
HCFC1R1	0.0960682774212898	0.00156555522711656	0.00364549197503207	1081
TEX14	0.0960245772842124	0.00157334297529249	0.00366035272401745	1081

MTHFD1L	0.0960227755168466	0.00157366482931517	0.00366067875133418	1081
PPFIA4	0.0960030629695996	0.00157719008524716	0.00366760871145147	1081
ENTPD8	0.0959993890616562	0.00157784790375482	0.00366871491472078	1081
STAB1	0.0959898539319355	0.00157955635956233	0.00367226345797844	1081
EXD1	0.0959784142099147	0.00158160831469927	0.00367576136359216	1081
ST14	0.0959586022216134	0.00158516781053087	0.00368325151646752	1081
ASCC2	0.0958946126052986	0.00159671479240692	0.00370915815187238	1081
MRPL48	0.0958892201275049	0.001597691393752	0.00371099886165502	1081
EIF4A1	0.0958763539498455	0.0016000237332638	0.00371555943591922	1081
ACTR5	0.0958258304136543	0.00160921278670393	0.00373560631345363	1081
LIG1	0.0958102642079776	0.00161205367637396	0.00374176994014903	1081
CSF2RB	0.0956351974968826	0.00164432299276718	0.00381183966505119	1081
PWWP2B	0.0956305601300274	0.00164518579340317	0.00381305534802657	1081
C7orf52	0.095630344265466	0.00164522596599886	0.00381305534802657	1081
ATAD3B	0.0956166785134387	0.00164777101077327	0.00381807534069791	1081
C1orf59	0.0956091064372824	0.00164918275540253	0.0038204676411665	1081
NETO1	0.0955891536605676	0.0016529080934052	0.00382865741539011	1081
BNIP3	0.0955817211817876	0.00165429777026864	0.00383143579927129	1081
PMM1	0.0955802397007577	0.00165457489630666	0.00383163711761095	1081
MPI	0.0954572185186817	0.00167773665617117	0.00388259677640071	1081
CPT1B	0.0954307332161683	0.00168276197088705	0.00389333179091762	1081
VCX2	0.0954288146328543	0.00168312653898208	0.00389372808082154	1081
SAR1B	0.0954262161737013	0.00168362041237211	0.00389442337894549	1081
C9orf96	0.0953743398460466	0.00169350813229008	0.00391594597394211	1081
KRT86	0.0953376361679454	0.00170053610838879	0.00393129445759608	1081
KRT34	0.0952963493979053	0.00170847365872747	0.00394873816602396	1081
SAAL1	0.0952747769754262	0.00171263456683234	0.00395790103806786	1081
HNRNPD	0.0952461457961702	0.001718171347902	0.00396978573270855	1081
EXOSC5	0.0952420850632433	0.00171895795403731	0.00397114770459651	1081
FCRL6	0.0952311996701311	0.00172106819957089	0.00397511108465438	1081
TMEM95	0.0952223962555791	0.00172277657191737	0.00397860071549643	1081
CLDN22	0.0951919137497912	0.00172870398264483	0.00399091699866837	1081
PGAM2	0.0951829431445133	0.00173045190588425	0.00399449451633302	1081

MPND	0.0951539764245603	0.00173610716136572	0.00400708968443085	1081
GOLGA7I	0.0951426191097605	0.00173832911012268	0.0040117584973561	1081
SPANXB2	0.0951364402720635	0.00173953903640255	0.00401409093795297	1081
ZSCAN5A	0.0950948824575211	0.00174769690721547	0.00403245380779471	1081
FRG2	0.0950179689989952	0.00176288788522149	0.00406657236319557	1081
HSF4	0.0950106145969173	0.00176434676575948	0.00406947167348577	1081
PPIAL4C	0.0949940970775268	0.00176762735967016	0.0040761049863273	1081
GAB3	0.0949894642258254	0.0017685485092865	0.00407776235774025	1081
UTF1	0.0949667576411366	0.0017730696343809	0.00408771890416608	1081
OTUB2	0.0949240993961377	0.00178159205136464	0.00410689688462533	1081
BLK	0.0948967334191229	0.00178707910596157	0.00411907420933591	1081
SPINT1	0.0948845452797899	0.00178952789559888	0.00412377481775788	1081
HRK	0.0948821058296093	0.00179001838942671	0.00412443331293619	1081
TUBB3	0.0948487229231565	0.00179674301297739	0.00413945425393751	1081
TSGA13	0.0948294093789907	0.00180064409652755	0.0041465449616538	1081
C17orf75	0.0948142833096019	0.00180370479159036	0.00415264377446717	1081
CNOT3	0.0947074315185391	0.00182546215686016	0.00420081507310349	1081
2-Mar	0.0946677484824364	0.00183360363501771	0.00421810497058716	1081
HS3ST6	0.094643950136589	0.00183850213598834	0.00422889078893413	1081
IFITM4P	0.0946400365105675	0.00183930884073701	0.0042302633401812	1081
AARSD1	0.0946308628497512	0.00184120105476876	0.00423413187766172	1081
SLC25A3	0.0946009015156973	0.00184739350811671	0.00424788748099658	1081
PIPSL	0.0945900575165105	0.00184963946760854	0.00425256643174778	1081
DTL	0.0945612760581137	0.00185561270545806	0.00426532610126113	1081
CLCN7	0.094556667378898	0.00185657082231638	0.00426704155340143	1081
POLE2	0.0945531895379048	0.00185729414512342	0.00426821703781785	1081
YRDC	0.0945514206553849	0.00185766213722143	0.00426857576757451	1081
HERPUD1	0.0945412232064159	0.00185978488329998	0.00427296606684285	1081
FJX1	0.0944817794691596	0.00187220331218624	0.00430002687538385	1081
CHTF18	0.0944802415652798	0.00187252560375994	0.00430027682258822	1081
SYK	0.0944567850366058	0.00187744759468177	0.00431108877178437	1081
GNAT1	0.094450816805728	0.00187870182842595	0.00431347712943251	1081
LRCH4	0.0944233990667477	0.00188447359398928	0.00432623596021813	1081

TMEM11(	0.0944001702097408	0.00188937627153746	0.00433699692230198	1081
SNAPC5	0.0943266004144142	0.0019049812016185	0.00436982991421143	1081
SUGT1	0.0942239135408283	0.00192695984533397	0.00441572131546501	1081
SVOP	0.0942213640209985	0.00192750847962335	0.00441647615127529	1081
PDIA5	0.0942117197572064	0.00192958513649788	0.00441972627342237	1081
FOSL1	0.0941857720068105	0.00193518254060921	0.00443204323335295	1081
MPO	0.0941621086006497	0.00194030014561059	0.00444291724149932	1081
ZAR1L	0.0941267933866795	0.00194796072884085	0.00445776086362592	1081
CARHSP1	0.0941127348520049	0.00195101801806263	0.00446323619962205	1081
SNX21	0.0940339435994227	0.00196823420649796	0.00450159832991614	1081
MYL5	0.0939748051192515	0.00198124755937615	0.00452930459414803	1081
SHB	0.0939439432036612	0.00198806996083431	0.00454438549427064	1081
LYPD5	0.0939048225799592	0.00199674899629548	0.00456163625883109	1081
FAM183B	0.0938937487527083	0.00199921205876293	0.00456674531395615	1081
MGAT2	0.0938892008934924	0.00200022440894722	0.00456853976397299	1081
SPAG7	0.0938217029303411	0.00201530474215646	0.00460037552756282	1081
TSG101	0.0938143075804956	0.00201696332205917	0.00460363994140969	1081
NGB	0.0937777554639189	0.00202517937289765	0.00462134554452007	1081
C10orf50	0.0937590461898597	0.00202939663060271	0.00462913048047463	1081
FCRL5	0.0937585434144945	0.00202951007215448	0.00462913048047463	1081
HSPA1A	0.093731181176735	0.00203569260281234	0.00464270660971975	1081
COX6A2	0.0937004768498896	0.00204265082685639	0.00465699421763489	1081
GMPPB	0.0936863307459438	0.00204586394821022	0.00466379192448738	1081
SAT1	0.0936809361723672	0.00204709048143499	0.00466605993986284	1081
C4orf27	0.0936482222755557	0.00205454289410664	0.00468251686862522	1081
HELLS	0.093574563474668	0.00207141386351741	0.0047177650967279	1081
TBC1D3B	0.0935657344313176	0.00207344458135772	0.00472185632957848	1081
FN3KRP	0.0935543331390853	0.00207606962548138	0.00472729994408526	1081
SLC38A8	0.093544389135523	0.00207836162721877	0.00473144931408319	1081
GTF2H5	0.0934917191619395	0.0020905402717869	0.00475756142963703	1081
ICAM4	0.093483068863466	0.00209254667969321	0.00476055856742055	1081
MECR	0.0934829847388692	0.00209256620076986	0.00476055856742055	1081
OR56B1	0.0934610488343908	0.00209766211404898	0.00477053548064085	1081

SMC1B	0.093434368857793	0.00210387543713516	0.00478358585565325	1081
NCRNA0(	0.0934112281464839	0.00210927816896097	0.00479424672387665	1081
CXorf38	0.0933331005302037	0.00212761286181846	0.00483210384456966	1081
NRN1L	0.0933145401204247	0.00213198994727786	0.00484149898409564	1081
C18orf32	0.0932993500605633	0.00213557833988709	0.0048485546779021	1081
SH2D4B	0.0932963678739987	0.00213628347972044	0.00484906261396826	1081
ACSS1	0.0932530885152616	0.00214654093653896	0.0048701505987811	1081
PAQR7	0.0932484894492484	0.00214763358802796	0.00487208092689452	1081
MRM1	0.093213147737976	0.00215604712065974	0.00488951584438707	1081
RWDD2B	0.0931862076426296	0.00216248080125846	0.0049018989244235	1081
ITGB1BP1	0.0931716178308258	0.00216597238934429	0.0049092612267485	1081
PAK6	0.0931617011948814	0.00216834855294061	0.00491298859509489	1081
DHX16	0.0931575212744547	0.00216935083461885	0.00491470676601402	1081
SFTA2	0.093145222168549	0.00217230243458211	0.00492084027264777	1081
MBOAT4	0.0931373115392761	0.00217420280854938	0.00492459136251711	1081
SOX12	0.0931303940490145	0.00217586584743717	0.0049278041030491	1081
PPIE	0.0930579336575866	0.00219335622003596	0.00496406707702781	1081
ACYP2	0.0930560822876727	0.00219380478198258	0.00496452452629062	1081
NFKBIL1	0.0930444094534147	0.00219663488636869	0.00497037063752637	1081
SERPINA(	0.0930305383123111	0.00220000231983446	0.0049772705209631	1081
LRTM2	0.0930066598558569	0.00220581025293318	0.00498833043840804	1081
EGFL7	0.0930045202056895	0.0022063313623633	0.00498894884889535	1081
PRLH	0.0928939687041821	0.0022334098612638	0.00504621373431575	1081
UBL4B	0.0928442969191939	0.00224567520766394	0.00507335730160256	1081
CCT5	0.092803042321443	0.00225590893411195	0.00509419184818801	1081
SUV420H	0.0927658919967003	0.00226516103758953	0.00511393803678596	1081
NME3	0.0927627705023557	0.0022659400090659	0.00511512342925062	1081
B3GNT4	0.0926914662593336	0.00228380091667007	0.00515313278072565	1081
SLC4A9	0.0926880268925306	0.00228466568627742	0.00515450669168537	1081
FAM100A	0.0926729407282356	0.0022884623823298	0.00516249436640916	1081
MMP11	0.0926216606575272	0.00230141109785071	0.00518996155448365	1081
KCTD13	0.0925811400091474	0.00231169031589897	0.00521139227996697	1081
DGCR10	0.092575873841886	0.00231302930829085	0.0052138273934787	1081

IGSF8	0.0925693946535979	0.00231467770050251	0.00521695930595469	1081
GSDMA	0.0925592079761398	0.00231727150018069	0.00522163695426623	1081
TRIB3	0.0925449340624181	0.00232091049149014	0.00522866717189318	1081
IMPDH1	0.0924953967690421	0.00233358010799021	0.00525544676081193	1081
GEN1	0.0924776766138889	0.00233812753714008	0.0052645109238501	1081
PNPO	0.0924607075435631	0.00234248981356245	0.00527256506080621	1081
MAN1B1	0.0924439807127772	0.00234679709949842	0.00528166993290087	1081
PRR5	0.0924322173747063	0.0023498305845578	0.00528768905967965	1081
RGL4	0.0924070918406134	0.00235632187332342	0.00530073748052487	1081
C14orf109	0.0923822295479423	0.00236276128798632	0.00531403663575799	1081
MAGEA1	0.0923549437523452	0.00236984691390914	0.0053281881786495	1081
CCDC87	0.0922381517040709	0.00240039576431679	0.0053926589352249	1081
UROC1	0.0922043067684541	0.00240931548728826	0.00540968128526772	1081
C9orf24	0.0921886993511415	0.00241343896963245	0.00541833590139816	1081
PIM1	0.0921620999771198	0.0024204813963436	0.00543354108862734	1081
PHF5A	0.0921274660598638	0.00242967919072392	0.00545236574547547	1081
CRLS1	0.0921231139378389	0.00243083724841315	0.00545435691348662	1081
TNNC2	0.0921166248614198	0.00243256486589684	0.00545762548429578	1081
DLGAP4	0.0921064249194868	0.0024352827129768	0.0054629938255627	1081
CMAS	0.0921056098926994	0.00243550000235188	0.0054629938255627	1081
STK38	0.0920998678318168	0.00243703136078927	0.00546551531189174	1081
LIN28A	0.092099360352657	0.00243716674344619	0.00546551531189174	1081
CYP27B1	0.0920575840614926	0.00244833520677106	0.00548933920316116	1081
ANKRD2	0.0920405660536784	0.00245289818850937	0.0054983458337066	1081
ABCG5	0.0920328917512776	0.00245495841490732	0.00550173960043476	1081
COX7B2	0.092013543180788	0.00246015971067912	0.00551273033083532	1081
EPN3	0.0920126132523411	0.00246040994810327	0.00551273033083532	1081
PPY2	0.0919738085678921	0.00247087278309752	0.00553494186761921	1081
STK17A	0.0918921339387134	0.00249302751462929	0.00558270779037427	1081
UBL4A	0.0918401013028034	0.00250723615974891	0.00561203027090465	1081
PTAFR	0.0918271710126219	0.00251077851429802	0.00561808654565518	1081
LOC15056	0.0917596352370022	0.00252935488300313	0.00565713934918375	1081
MIER2	0.0916744404134862	0.00255296758978452	0.00570672311335701	1081

DNMT3A	0.091673519607014	0.00255322389777546	0.00570672311335701	1081
PASK	0.0915693860225332	0.00258236171910647	0.0057699286636424	1081
CENPK	0.0915682514574689	0.00258268084936194	0.00577000174230856	1081
AES	0.0915490409416264	0.00258808984968266	0.00578144489042551	1081
C3orf21	0.0915412236538754	0.00259029388025429	0.00578572682312038	1081
SEPW1	0.0915084142335081	0.00259956297415753	0.00580449968015999	1081
SLC5A11	0.0915015007700421	0.00260151997754182	0.00580775709280434	1081
SLCO6A1	0.0915012237860585	0.00260159841178786	0.00580775709280434	1081
PLBD1	0.0914665299182635	0.00261143985005674	0.00582778949588934	1081
SCN1A	0.0914541651457531	0.00261495551118345	0.00583434253768862	1081
TCTEX1D	0.0914157001645747	0.00262591986031225	0.00585685956443647	1081
PAGE2B	0.0913498194852959	0.00264479652686219	0.0058950460316042	1081
LTBR	0.0913447096468285	0.00264626580281248	0.00589766839226214	1081
DNAJC17	0.0913434084094516	0.00264664007782626	0.00589785004068694	1081
RABEPK	0.0913364847426937	0.00264863234531421	0.00590163683180563	1081
LOC1436(	0.0913107568336959	0.00265604748553781	0.00591619599692162	1081
ITGAD	0.0913063250779442	0.00265732669037976	0.00591839095386403	1081
NUBP2	0.0913004003361903	0.00265903771678515	0.005921547071041	1081
WDR64	0.091277402299145	0.00266568892985292	0.00593439092627772	1081
WFDC10E	0.0912160774186613	0.00268349886209591	0.00597271954225193	1081
RNF10	0.0911468417550254	0.00270373669774149	0.00601643386457554	1081
LCN2	0.091131903669754	0.00270812137868701	0.0060241944758889	1081
MOCOS	0.0911022135482162	0.00271685539944464	0.00604176863477041	1081
BTBD6	0.0910844281791519	0.00272209966943387	0.00605183301390386	1081
MAGEA9I	0.0910498229531851	0.00273232999089025	0.00607335183344191	1081
C22orf9	0.090987296448796	0.00275090365455242	0.00611193935380595	1081
SSBP3	0.0909718933040873	0.00275549684627136	0.00612079435088065	1081
LMTK3	0.0909386637590469	0.00276542967551955	0.00614150378275178	1081
CNTNAP4	0.0909354849363722	0.00276638158476876	0.00614258949825961	1081
WDR46	0.0909172871066371	0.00277183674206515	0.00615234146858776	1081
HSPD1	0.0909113835471828	0.00277360855685593	0.00615559594335824	1081
FIS1	0.0909012423099447	0.00277665462283011	0.00616055713486447	1081
C20orf111	0.0909008869388663	0.00277676141894115	0.00616055713486447	1081

SLC25A3:	0.0908452129029082	0.00279353892493702	0.00619436892051253	1081
RAB9A	0.0908076923489794	0.00280489792863073	0.00621750316596237	1081
AMZ2P1	0.0908002967128419	0.00280714185302723	0.00622179256565449	1081
SF3B4	0.0906908925939321	0.00284052816056002	0.00629174159950823	1081
TRMT61A	0.0906470988736337	0.00285399363976669	0.00631868357765687	1081
SLC9A9	0.0906354469815947	0.00285758609535628	0.00632594196603871	1081
TNFSF10	0.0906196940893333	0.00286244950634465	0.00633601201025305	1081
RSPO4	0.0906183591668838	0.00286286198532573	0.00633622881722555	1081
XRCC3	0.0906059818710662	0.00286668903920283	0.00634330521690916	1081
C10orf95	0.0905991529696529	0.00286880252815784	0.00634728467103127	1081
CALCB	0.0905934538653347	0.0028705674415054	0.00635049210510942	1081
MRS2	0.0905879969914725	0.00287225826787369	0.00635353495182996	1081
TTBK1	0.0905386782620475	0.00288758103708909	0.00638392449431077	1081
MGC2328	0.0905304159197194	0.00289015532811642	0.0063875127904449	1081
SLC6A13	0.0905142767026175	0.0028951898454706	0.00639723587112046	1081
UBTD1	0.0904945685965326	0.00290134849342287	0.00640943803048621	1081
CNIH4	0.0904912314271118	0.00290239251736641	0.00641104136648534	1081
TMEM61	0.0904601876353086	0.00291212090050331	0.00643111987948249	1081
FAM25A	0.0904283005129414	0.00292214449686057	0.00645113434402807	1081
ZNF579	0.0903953605688081	0.00293253205309676	0.00647264799053734	1081
PC	0.0903659567689829	0.00294183290663033	0.00649246537073488	1081
ART3	0.0903513206202635	0.002946472548817	0.00650128033909294	1081
RANBP3	0.0902931219146482	0.00296498751762131	0.00653998396391999	1081
CYP11B2	0.0902632505597437	0.00297453171419756	0.00655959949452762	1081
GRAP2	0.0902468594217746	0.00297978073275413	0.00657045565469921	1081
CDCA4	0.0901897196244259	0.00299814495976464	0.00660877888329781	1081
FAM128A	0.0901842895416	0.00299989549429866	0.00661191408453464	1081
PLA2G16	0.0901229697312402	0.00301972832748054	0.00665271513091595	1081
C14orf34	0.0900861930467886	0.00303168033337677	0.00667831605422363	1081
METRN	0.0900671735007833	0.00303787835982794	0.00669123765129389	1081
HIST1H2A	0.090061138890505	0.00303984730910648	0.00669484246659561	1081
C14orf159	0.0899832679556942	0.0030653592177604	0.0067488154581184	1081
DNAJC19	0.089939290085341	0.00307985315951418	0.00677702259949898	1081

BHLHA15	0.0898983622234564	0.00309339793502149	0.00680385428551238	1081
USH1C	0.0898921860248331	0.00309544660619229	0.00680761701765761	1081
B4GALT4	0.0898682679430917	0.00310339199618447	0.00682360093463613	1081
PORCN	0.0898669450988388	0.0031038319755916	0.00682382354559562	1081
ACO2	0.0898163440718847	0.003120704628386	0.00685642869874971	1081
BTN1A1	0.0897938715227693	0.00312822472794105	0.0068722014334103	1081
SARDH	0.0896676354903777	0.00317077518715668	0.00695918626990733	1081
ROGDI	0.0896634617011648	0.00317219100333661	0.00696119692398867	1081
EXOSC7	0.089660716043392	0.00317312268818939	0.00696248301422234	1081
KCNMB3	0.0896038317178315	0.00319248138141173	0.0070022322103414	1081
AGRN	0.0895905154982365	0.00319702861309417	0.00701035609130098	1081
MEMO1	0.0895689857212591	0.00320439308657949	0.00702497537589986	1081
RANGRF	0.0894456813377384	0.00324686877713225	0.0071126763283307	1081
HUS1B	0.0894303704878822	0.00325217863464922	0.00712275914274936	1081
PDSS1	0.0894246335234637	0.00325417026836266	0.00712557174523541	1081
CRYBA4	0.0893936223196652	0.00326495528330418	0.00714841040997312	1081
TOMM40I	0.0893849926105234	0.00326796228465779	0.00715421649906881	1081
PPIF	0.0893696188078993	0.00327332549213078	0.00716206604094434	1081
FAM83A	0.0893645817251791	0.0032750844376828	0.00716435834478444	1081
DULLARI	0.089337227520071	0.00328465152831704	0.00718294670407585	1081
DHRS3	0.0892850082206902	0.00330298576864233	0.00721833893570186	1081
PRAME	0.0892312272873822	0.00332196555814312	0.00725902984800881	1081
STK19	0.0891918071268182	0.00333594023849263	0.00728482559397659	1081
C14orf142	0.0891850289620695	0.00333834851783591	0.00728929448209455	1081
C21orf122	0.0891820810808505	0.0033393963921726	0.00729000220202829	1081
HAGHL	0.0891390927131352	0.0033547113606894	0.00732026217082842	1081
ASZ1	0.0891285771280591	0.00335846733623105	0.00732766430070125	1081
MPV17	0.0891048053543211	0.00336697227832755	0.00734462983723968	1081
GJA9	0.0890867563088128	0.00337344284712336	0.00735476257091993	1081
TIMM13	0.0890528096901376	0.00338564332340843	0.00737736990265687	1081
CPSF4	0.0889956405128232	0.00340628073342003	0.00741912913555482	1081
CCL15	0.0889933368914454	0.00340711470597761	0.00742014332453178	1081
C2orf85	0.0889527630486689	0.00342183398255749	0.00744897834218939	1081

UCHL3	0.0889374851693691	0.00342739141807672	0.00746027013691579	1081
FUK	0.0889075029535156	0.0034383215156934	0.00748163609134191	1081
HEATR7A	0.088905279154044	0.00343913346859678	0.00748259463493704	1081
SHISA4	0.08890276551088	0.00344005145897224	0.00748378365453517	1081
TXNDC9	0.0888746822611839	0.00345032270556359	0.0075020778093447	1081
PNKP	0.0888486258542569	0.00345987757065536	0.00752041796081704	1081
PROCA1	0.0888418977742555	0.00346234865611556	0.00752497720114876	1081
HNRNPC	0.0888376756258857	0.0034639001834535	0.00752753713006157	1081
PPP1R1C	0.0888180395698228	0.00347112421884013	0.0075422573209564	1081
VTI1B	0.0888064901309586	0.00347537960903414	0.00755004014062253	1081
C6orf203	0.0887934733813105	0.003480181312425	0.00755721165665643	1081
LOC10027	0.088775659076129	0.00348676254140469	0.007569870826231	1081
FLYWCH1	0.088755693803944	0.00349415185259549	0.00758509580501467	1081
GPR174	0.0887376790778631	0.00350083146082693	0.00759713990933517	1081
MRPS36	0.0886844758478089	0.00352062626710109	0.00763845085091561	1081
C13orf35	0.0886050657967153	0.00355036081193862	0.00769633256822378	1081
TAF15	0.0885783602683978	0.00356041166245692	0.0077164597030871	1081
SRRT	0.0885652661438917	0.00356534918455817	0.00772549847509135	1081
CCDC136	0.0885254558874391	0.00358039903321495	0.00775635015014558	1081
ATP5L2	0.0885245492813797	0.00358074243714837	0.00775635015014558	1081
CALR3	0.0884690144214548	0.00360183504779574	0.00779784707553414	1081
CACYBP	0.0884639203177205	0.00360377547110941	0.00780120963523523	1081
SYTL1	0.088407359442164	0.00362538427511049	0.00784630062549429	1081
DBP	0.088406086078085	0.00362587210915033	0.0078465134427794	1081
AGT	0.0883482565254626	0.0036480899180161	0.00789289779813493	1081
CTXN2	0.0883039167762695	0.00366520867054971	0.00792823243511479	1081
MTHFSD	0.0882990740428091	0.00366708277136894	0.00793143466064283	1081
SPAM1	0.0882904757960209	0.00367041237116083	0.00793693186099558	1081
HSPB8	0.0882843530388635	0.00367278503118198	0.00794035785073632	1081
SELO	0.0881789449637265	0.00371385119771236	0.00802569538488688	1081
C16orf48	0.0881682428454482	0.00371804388325779	0.00803389402855606	1081
SLC6A8	0.0881277815358789	0.00373393395470553	0.00806476889863224	1081
PGLYRP4	0.0880941065294925	0.00374720586644148	0.00809256670019979	1081

C17orf59	0.0880538164509872	0.00376314114746497	0.00812436806512505	1081
PECR	0.0880347872468054	0.00377068882321712	0.00813804653350213	1081
GHITM	0.0879904657228624	0.00378832164884165	0.00817347537923477	1081
YBX1	0.0879547467577382	0.00380258645817188	0.00819986129307135	1081
CCDC34	0.087928795017383	0.00381298115868894	0.00821963675139525	1081
IBSP	0.0879088954618872	0.00382096917723214	0.00823597518460748	1081
GLIPR2	0.0878622234768955	0.00383976370501447	0.00827294543716754	1081
CEACAM	0.0878235377557516	0.00385540574426768	0.00830398254231502	1081
SUN3	0.0877650461702558	0.00387916581214681	0.00835337202412587	1081
C1orf53	0.0877565475284341	0.00388262910487452	0.00835993622463629	1081
HTR3A	0.0877320415756945	0.00389263126803971	0.00837878586330377	1081
WDR25	0.0876488707070658	0.00392675217189069	0.00844952173710084	1081
SQLE	0.08763621093229	0.00393196957348724	0.0084598448208801	1081
FAM75A2	0.0876315357778556	0.00393389790754174	0.00846308984914869	1081
TMEM82	0.0875878089765871	0.00395197521777738	0.00849952132387528	1081
NUDT7	0.0875875123587799	0.00395209810080614	0.00849952132387528	1081
CEBPA	0.0875500315446727	0.00396765358847983	0.00853206463918939	1081
TMCC2	0.0875151164495983	0.00398219416388841	0.00856089929453413	1081
ZIM3	0.0874887002971227	0.00399322739803235	0.00858177475478201	1081
BEST4	0.0874722560533575	0.00400010965467063	0.00859543562595626	1081
KHDC1	0.0874470362313132	0.00401068556831274	0.0086172419767129	1081
PRIM1	0.0874430478600222	0.00401236040840526	0.00861992112907368	1081
CUTC	0.0874365673014802	0.00401508314700054	0.00862485071404627	1081
SCD	0.0874218698756056	0.00402126432426373	0.0086372075714141	1081
ZNF276	0.0873833014752925	0.00403752576669749	0.0086712107217613	1081
C4orf26	0.0873498544936319	0.00405167612316441	0.00869974584322607	1081
FAM46A	0.0873432161156233	0.00405448995092419	0.00870300480142438	1081
WDR85	0.0873362686223067	0.00405743670109668	0.00870780536240764	1081
MAGEA4	0.0873359051666078	0.00405759091272874	0.00870780536240764	1081
TSPO	0.0873320788911718	0.0040592146961943	0.00871036217030616	1081
TRIM16	0.0873250844080735	0.00406218451680044	0.00871580648534933	1081
NKX6-3	0.087287853943401	0.00407802554193404	0.00874700005773649	1081
C12orf65	0.0872700113071885	0.00408563715392748	0.0087614605562986	1081

FAM71D	0.0872529848493398	0.00409291259040034	0.00877519413938005	1081
TMEM217	0.087241769554875	0.00409771131829668	0.00878454767554395	1081
CYCS	0.0872357864746458	0.0041002734051798	0.00878910488905587	1081
C2orf7	0.087212164102741	0.00411040321293206	0.00880988112826006	1081
OR4C6	0.0871690486581299	0.00412895058140497	0.00884681019595864	1081
LOC44108	0.0871521941685316	0.00413622162611876	0.00886024940505689	1081
ZNF341	0.0871514513382057	0.00413654235068553	0.00886024940505689	1081
TOMM22	0.0871272059268895	0.00414702293296619	0.00888080971453216	1081
ALDH5A1	0.0871185897953884	0.00415075322513345	0.00888785328659793	1081
BTN2A1	0.0871079022509754	0.00415538454493073	0.00889493376449151	1081
LCE3D	0.0870740225819912	0.00417009685717031	0.00892547823923672	1081
H2AFB1	0.0870660304873172	0.00417357431192471	0.00893170575821255	1081
MCM3	0.087040329751069	0.00418477482127111	0.00895308929210987	1081
C5orf58	0.0870236755387192	0.00419204734303882	0.00896674386552528	1081
DPYSL5	0.0870148170349066	0.00419592031621938	0.00897407524899028	1081
CXCR2	0.0869636974359895	0.00421833340031143	0.00901913886109889	1081
VWA3B	0.0869381011440988	0.00422959663708247	0.00904226087806711	1081
HLA-DPB	0.0869338544120335	0.00423146797560859	0.00904530160957503	1081
PTPRU	0.0868704821555721	0.00425948243358999	0.00909746327657659	1081
TNNI2	0.0868547758054786	0.00426645153546004	0.00911041617361061	1081
CLRN1OS	0.0868469101633355	0.00426994549727165	0.00911691065633676	1081
GVIN1	0.0868373001114923	0.00427421785238703	0.00912506556129045	1081
RILPL1	0.0868314478424612	0.00427682150052512	0.00912965657815815	1081
C12orf27	0.0867680668802986	0.0043051115536936	0.00918518028480804	1081
DDX12	0.0867465677268579	0.00431474610472017	0.00920476122837952	1081
CLEC4M	0.0867405608659186	0.00431744148419386	0.00920953607571848	1081
TRMU	0.086727479452888	0.00432331660991345	0.00922109191177411	1081
LGALS4	0.0867124847212108	0.00433005994880745	0.00923254209025467	1081
CNTN2	0.0867098330847234	0.00433125341692686	0.0092341094384582	1081
TCTA	0.0866856896459661	0.00434213378262437	0.00925436786404654	1081
KRT82	0.0866446970846048	0.00436066397838679	0.0092928780117002	1081
LIM2	0.0866358237824181	0.004364684461013	0.00929955753577005	1081
CETN2	0.0866204643650124	0.00437165172671469	0.00931333799012981	1081

GTF2F1	0.0866095630690875	0.0043766028273477	0.00932289985798027	1081
SLC25A4:	0.0865844085823281	0.00438804675126693	0.00934431308713238	1081
SEZ6	0.0865205207019849	0.00441723411832509	0.00940043058837084	1081
BCL11B	0.0865191024839359	0.00441788402427828	0.00940043058837084	1081
IFFO1	0.086494265091993	0.00442927992900797	0.00942215883525508	1081
GLB1	0.0864646609615873	0.00444289765851721	0.00944713672480781	1081
KIAA022(	0.0863943303824145	0.00447540136902627	0.00951123120361158	1081
LOC3887‹	0.0863652077674828	0.00448892344388839	0.00953695030343087	1081
MRPL44	0.0863307805076716	0.00450495621821555	0.00956899441332267	1081
T	0.086320080297307	0.00450994984887366	0.00957657212032887	1081
LOC6493:	0.0863071609718651	0.00451598578092871	0.00958736785296752	1081
FAM92B	0.0863001818462403	0.0045192494804205	0.00959126430500116	1081
PLCB3	0.0862825845244266	0.00452748811971438	0.00960487996885957	1081
RNF126	0.0862818768054538	0.0045278197405956	0.00960487996885957	1081
MGST3	0.0862813877818114	0.00452804889869741	0.00960487996885957	1081
TMPRSS6	0.0862680430752151	0.0045343063235142	0.00961714054402964	1081
FLJ30679	0.0862655753580191	0.00453546430974913	0.00961858390566337	1081
ANAPC13	0.0862248979763112	0.00455459092389313	0.00965508093884321	1081
PPM1M	0.0862093214433283	0.00456193434887189	0.0096696304143544	1081
TDP2	0.0862065998944548	0.00456321849859644	0.00967133473479487	1081
SLMO2	0.0862005516897437	0.00456607348562756	0.00967636759604114	1081
DAND5	0.0861895907163669	0.00457125160729827	0.00968632203944712	1081
EXOC7	0.0861768954326775	0.00457725568007872	0.00969802436634264	1081
ENTHD1	0.0861645886774034	0.00458308281082231	0.00970934937680256	1081
PANK4	0.0861433862642555	0.00459313768896584	0.00972758186966115	1081
RNF220	0.0861298449367275	0.00459956986506502	0.00974018027244137	1081
CST6	0.0860882695195289	0.00461936926066401	0.00978107985664037	1081
SYT5	0.0860643374463478	0.00463080131487642	0.00980322535605143	1081
HMGN3	0.0860356476004192	0.00464453981267053	0.00982817799645461	1081
PNPLA6	0.08602899768764	0.00464772946459651	0.00983286180049324	1081
CLEC18B	0.08601708690878	0.00465344745901366	0.00984392513512866	1081
IL3	0.0860079158608195	0.00465785452731924	0.00985221329828287	1081
CDK10	0.0860035115305924	0.00465997233140815	0.0098546234113182	1081

CRYBB2	0.085931673090037	0.00469463868285031	0.0099224643720386	1081
VASP	0.0859304899938168	0.00469521154391914	0.0099224643720386	1081
PPP1R2P1	0.0859288766843568	0.00469599281821871	0.0099224643720386	1081
CHCHD5	0.0858985566047425	0.004710697741822	0.00994831806363395	1081
PISD	0.0858603185370426	0.00472930215215146	0.00998551429148844	1081
SOX3	0.0858410639073276	0.00473869544976043	0.0100022711114911	1081
IL1F9	0.0858409972523015	0.00473872799642942	0.0100022711114911	1081
SLC6A7	0.085831753108603	0.00474324373348405	0.0100107537989561	1081
SLC25A4	0.0858185728210408	0.00474968899171929	0.0100233065931474	1081
C14orf153	0.0858139520888405	0.00475195043731751	0.0100265495558109	1081
KCTD5	0.0858133990750104	0.00475222115457348	0.0100265495558109	1081
NUMBL	0.0858105540203755	0.00475361411654343	0.0100284382006249	1081
ARRDC2	0.0857658430076499	0.00477555351716572	0.0100726128785785	1081
GLDC	0.0857636329533392	0.00477664034600524	0.0100737637232134	1081
NDUFAF4	0.0857626998675598	0.00477709927266607	0.0100737637232134	1081
AKNA	0.0857534225058136	0.00478166439610145	0.0100823350700716	1081
GKN1	0.0857276112327019	0.00479438613325418	0.01010704360134	1081
GAS8	0.0857178149453585	0.00479922248235282	0.0101151220869426	1081
DGCR5	0.0857137702940143	0.00480122058009334	0.0101182747762298	1081
HCG27	0.0857098532086855	0.00480315637547655	0.0101202784715124	1081
MAST3	0.0857098133102867	0.00480317609660609	0.0101202784715124	1081
RBP5	0.0856910574689593	0.00481245491000926	0.0101378403963955	1081
CA11	0.0856813672835613	0.00481725514218017	0.01014675918436	1081
GOLGA6I	0.0856797208853875	0.00481807114915167	0.0101474169680774	1081
ARID3A	0.0856663404335487	0.00482470755773831	0.0101582079588878	1081
PXDNL	0.0856636280325666	0.00482605385516079	0.0101599806575623	1081
MAP2K1	0.085620314674485	0.00484759843427178	0.0102032045197372	1081
CLEC18A	0.0856088578728077	0.00485331169740838	0.0102141625725336	1081
ADORA1	0.0855739036549369	0.00487078023566522	0.0102455745899004	1081
YJEFN3	0.0855725753322425	0.0048714451902022	0.0102459034617481	1081
UXT	0.0855099607245881	0.00490288308311662	0.0103077206960326	1081
CCDC97	0.0854979911075015	0.00490891367943918	0.0103193223491915	1081
GTPBP3	0.0854734308256297	0.00492130875516793	0.0103432201223639	1081

UBE2R2	0.0854649433535071	0.00492559877822933	0.0103511566229324	1081
C6orf154	0.0854620363595408	0.0049270689050943	0.0103531660679175	1081
CHPF2	0.0854609715126843	0.0049276075193013	0.0103532179261916	1081
NIP7	0.0854229557422786	0.0049468712940063	0.0103882750148814	1081
DUSP23	0.0854217807178967	0.00494746779759216	0.0103884447344688	1081
SENP5	0.0853914307389697	0.00496289755366085	0.0104197572921832	1081
LPIN3	0.0853700638276066	0.00497378642250975	0.010439354811571	1081
PXT1	0.0853411402968115	0.00498856061624087	0.0104692732174364	1081
SPCS1	0.085299580417855	0.00500985886467008	0.0105117807341713	1081
C19orf35	0.0852759518588655	0.00502200440179184	0.0105350701524624	1081
TAF1C	0.0852378234584084	0.0050416592095838	0.0105740994041713	1081
PPP2R4	0.0852221743654304	0.00504974624156618	0.0105899581549397	1081
ACTR10	0.0852187243025122	0.00505153071641866	0.010592597718565	1081
SNAI1	0.0852167676319196	0.00505254301744692	0.0105936177234043	1081
ANAPC5	0.0851721745722821	0.00507566333934472	0.0106386361696337	1081
RSPH10B2	0.0851712828905816	0.00507612662509404	0.0106386361696337	1081
SUPT5H	0.0851551482839086	0.00508451618368018	0.010652894281876	1081
USP2	0.0851385794914241	0.00509314453131718	0.0106694073558966	1081
PKIG	0.0851369638563264	0.00509398659463953	0.0106694073558966	1081
HSP90AB4	0.0851294398943721	0.00509790971264907	0.0106754044866232	1081
AANAT	0.0851264864624898	0.00509945042602808	0.0106765885608466	1081
COQ10A	0.085100861093084	0.00511283603664197	0.0107022113422852	1081
KRTAP10	0.0850221635900772	0.00515414276818389	0.010785312772937	1081
CNR2	0.0849740506048867	0.00517954440745799	0.0108350905595266	1081
C17orf61	0.0849631867629461	0.00518529567098369	0.0108457795154813	1081
NANP	0.0849623104727958	0.00518575982565426	0.0108457795154813	1081
C13orf34	0.084961348795321	0.00518626925155777	0.0108457795154813	1081
APOC1P1	0.0849525084128188	0.00519095435110247	0.0108529562715163	1081
SLC43A3	0.0849518238949909	0.00519131728132693	0.0108529562715163	1081
CYFIP2	0.0848919628295172	0.0052231442087109	0.0109172276493547	1081
ZCCHC2	0.0848862429150088	0.00522619456808129	0.0109213370927383	1081
C8orf56	0.0848648180956701	0.00523763444790253	0.0109429730297652	1081
CEACAM	0.0848120178771517	0.00526592386673128	0.0109997963806825	1081

PADI4	0.084777715685495	0.00528437615498754	0.0110337642663997	1081
GABRG2	0.0847271945869888	0.00531165934783468	0.0110825535649846	1081
CLDN23	0.0847262995951339	0.00531214381849049	0.0110825535649846	1081
TYK2	0.0847007269957965	0.00532600344594313	0.0111103178438982	1081
PTPN2	0.0846911407088115	0.00533120734128701	0.0111188706791828	1081
AIDA	0.0846835705076174	0.00533532004868162	0.011125144641413	1081
CYP2W1	0.084681843148201	0.00533625888215093	0.0111259506500653	1081
TBC1D26	0.0846465430608392	0.00535547741911707	0.0111614000215305	1081
CASP8	0.0846161422619835	0.00537207862065441	0.0111893628407801	1081
YWHAB	0.084598801009067	0.00538156906468302	0.0112076614088741	1081
PNO1	0.0845689901449134	0.00539791916018159	0.0112393882668587	1081
APTX	0.0845533565713105	0.00540651145590725	0.0112549522867884	1081
ZFYVE28	0.0845375529754661	0.00541520972807096	0.0112718950167379	1081
FOLR2	0.084492200559515	0.0054402417211124	0.0113204905972327	1081
CCL14-CC	0.0844740154303285	0.00545030815849069	0.0113381118230091	1081
TADA3	0.0844349773383982	0.00547197455495513	0.0113759471010909	1081
RPL35	0.0843780310499784	0.00550371935716513	0.0114372216267889	1081
PRKCSH	0.0843767741288778	0.00550442189830653	0.0114375017164915	1081
BRF2	0.0843619769245857	0.00551269869900826	0.0114530015355774	1081
IQCD	0.0843614057474821	0.00551301841104462	0.0114530015355774	1081
C19orf23	0.0843599917119935	0.00551380997854308	0.0114534648399413	1081
ARRDC1	0.084356028812142	0.00551602892889472	0.0114568927490034	1081
LOC9145(	0.0843118205109502	0.00554083714821541	0.0115060472477888	1081
UCK1	0.0842605560492122	0.00556973091789071	0.0115648556319221	1081
USP11	0.0842594833702712	0.00557033695063749	0.011564921969555	1081
KBTBD8	0.084248859434312	0.00557634237810197	0.0115761971565194	1081
C5orf49	0.0842416133046733	0.00558044176764774	0.011583513591887	1081
FUT4	0.0841938307476627	0.00560754209428465	0.0116373684443561	1081
SIAH2	0.0841352517425205	0.00564092748355949	0.011701831341397	1081
MAGEA3	0.0841058420693011	0.00565775608874712	0.0117343247614342	1081
RPS26P11	0.0841018942355673	0.00566001853109034	0.011737808658515	1081
EIF2AK1	0.0840977662305694	0.00566238509836077	0.011740928193259	1081
SPRR1A	0.0840734031849733	0.00567637048908744	0.0117668742027852	1081

RIBC1	0.0840617033938884	0.0056830977026876	0.0117783953930701	1081
ULK3	0.0840162469290638	0.00570930272618248	0.011830271900735	1081
HIST2H2I	0.0839951865136887	0.00572148061412586	0.0118520976676938	1081
CCR10	0.083994978719734	0.00572160088450345	0.0118520976676938	1081
SCGB1D4	0.0839795537333712	0.00573053517025542	0.0118693842283359	1081
SYS1	0.0839740816489281	0.00573370766682713	0.01187473434237	1081
CKMT1A	0.0839266400700373	0.00576127877377848	0.0119281563101508	1081
KCNG4	0.0839141194255066	0.00576857514425629	0.0119395814528966	1081
FAM65A	0.083896960855405	0.00577858776747098	0.0119590764922645	1081
COMT	0.0838881189027904	0.00578375346192936	0.0119673082878561	1081
TMEM15(	0.0838737763244894	0.00579214160529084	0.0119834335666616	1081
DPEP3	0.0838487951389588	0.00580677781095249	0.0120112474588395	1081
DAZL	0.0838099081065563	0.0058296275694886	0.0120572738590706	1081
CAMKV	0.0837908293861295	0.005840867669274	0.0120768017240608	1081
CCDC42	0.0837735969622144	0.00585103680093871	0.0120953449312376	1081
CHRNB4	0.0837369367297404	0.00587272363277122	0.0121364400474124	1081
ODF3L2	0.083734006416735	0.00587446021601875	0.0121375385694049	1081
LMO2	0.0837258466204482	0.00587929836928947	0.0121462891651458	1081
GAS2L1	0.0837106435312132	0.00588832223979781	0.0121636845283764	1081
DYNLL2	0.0836989990295726	0.00589524232146284	0.0121767309100655	1081
LOC6471	0.083660232799118	0.00591833305379287	0.0122182724296766	1081
CEACAM	0.083660142701853	0.00591838681403742	0.0122182724296766	1081
BCL7C	0.0836580007943789	0.00591966500079116	0.0122196589241662	1081
C10orf58	0.0836336236715519	0.005934229601789	0.0122472139461161	1081
NUP62	0.083628272071837	0.00593743133613044	0.0122513115094077	1081
ITLN1	0.0836215188041541	0.00594147387357766	0.0122583972944717	1081
INPPL1	0.0836139726160388	0.00594599398697068	0.0122664668579134	1081
GH1	0.0836022275555993	0.00595303535189522	0.0122797355277421	1081
OR4K17	0.0835698160046798	0.00597250557341444	0.012317375591363	1081
SMARCA	0.0835636994350027	0.00597618633118019	0.0123237049484722	1081
TM2D2	0.0834842393665022	0.00602418871974318	0.0124157835683713	1081
PAX7	0.0834274023692813	0.00605873685420251	0.0124811590068422	1081
MIP	0.0834236522729716	0.00606102259023339	0.0124845910102507	1081

SFRS17A	0.083413611147769	0.0060671466157953	0.0124933730527646	1081
PNMA5	0.0833838133000077	0.00608535293601124	0.0125283019822122	1081
APBA3	0.0833469274351402	0.00610795805364821	0.012572270868575	1081
TUT1	0.0833328072938943	0.00611663140487622	0.0125888373162272	1081
MC2R	0.0833122985523612	0.00612924870738039	0.0126122283156464	1081
ETV2	0.0832674333389247	0.00615693214398128	0.0126640185869413	1081
RTN3	0.0832632586635726	0.00615951377686558	0.0126680352256209	1081
DHDPSL	0.0832616088734343	0.00616053428081081	0.012668840658119	1081
TMEM158	0.0832499337225962	0.00616776047737795	0.012681111942925	1081
RIPK3	0.0832470903767865	0.0061695214864134	0.0126834381410142	1081
MSX1	0.0832276736238314	0.00618155922571951	0.012706888836951	1081
UNC45A	0.0832214897839827	0.00618539743199837	0.0127134814067551	1081
MED10	0.0832085483829341	0.00619343687961284	0.0127276052196343	1081
MFSD1	0.0832083944099188	0.00619353258714692	0.0127276052196343	1081
ITGAM	0.0831848590016861	0.00620817752350292	0.0127537972884934	1081
CHCHD7	0.0831690483278297	0.00621803321031276	0.0127727418192873	1081
RNPEP	0.0831354405783312	0.0062390295657093	0.0128132582935278	1081
CD2BP2	0.083117674179297	0.00625015481224998	0.0128347980318834	1081
TNFRSF1'	0.0830971858481832	0.00626300666117382	0.0128559475432389	1081
FOXD4L6	0.0830579294712121	0.00628769764837689	0.0129013718808874	1081
ASCL1	0.0830223562989872	0.00631014755004156	0.012943480541247	1081
PCTP	0.0830136871922481	0.00631562944991668	0.0129534061564418	1081
PTPRQ	0.0830108932565413	0.00631739710384395	0.0129557125783301	1081
FOXB1	0.082981660957411	0.00633591833800318	0.0129910508823485	1081
CNDP2	0.0829553918053748	0.0063526037740506	0.0130226114814541	1081
C9orf119	0.082939604864992	0.00636265018669228	0.0130418791219898	1081
ANKRD1	0.0829347752780366	0.00636572646543396	0.0130468572231323	1081
RASSF7	0.0829011028786059	0.00638721178314989	0.0130855671078567	1081
YIPF4	0.0828585575894558	0.00641445178705794	0.0131346951870586	1081
PAPD5	0.0828439092450822	0.00642385464937587	0.0131526122483664	1081
QRFP	0.0828364102465756	0.00642867309376662	0.0131598028121053	1081
SLC7A6O	0.082807422524447	0.0064473295630609	0.0131953118000469	1081
COQ3	0.0828025830916044	0.00645044895108235	0.0131990141309857	1081

IL1F10	0.0827880656636128	0.00645981469018931	0.0132168359672825	1081
SH3GL1	0.0827856799589688	0.00646135496624896	0.0132186448456469	1081
TREML4	0.0827777657272156	0.00646646697335333	0.0132277596647241	1081
ATP13A2	0.082762855636739	0.00647610765806443	0.0132440935570048	1081
AMIGO3	0.0827623664081885	0.00647642420600917	0.0132440935570048	1081
FLJ35776	0.0826951689257623	0.00652003561784538	0.0133292183399122	1081
CCDC51	0.0826750776805316	0.00653312603510975	0.0133546244522867	1081
MED30	0.082671406502912	0.00653552053386632	0.0133581636723556	1081
DERL1	0.0826558360964654	0.00654568498104349	0.0133748680335857	1081
NCRNA0(	0.0826081517433576	0.00657690196931867	0.0134318420693354	1081
TH	0.0826045780268267	0.0065792469114851	0.0134339072604771	1081
MT1IP	0.0825899828085546	0.00658883155690797	0.0134521142914676	1081
HARS2	0.0825674913202483	0.0066036262147093	0.0134809535970125	1081
SMPDL3E	0.0825466364231599	0.00661737098652679	0.013507643988609	1081
CBFA2T3	0.0825397890104085	0.00662188948798485	0.01351412863291	1081
SLMO1	0.0825218537740935	0.00663373780624115	0.0135369376121078	1081
TARM1	0.082499776065786	0.00664834884597559	0.0135640052159387	1081
TNFRSF1:	0.082491555869399	0.00665379635390415	0.0135723701447346	1081
ATP2A1	0.0824875452445863	0.00665645564136383	0.0135764198537283	1081
ACTB	0.082463861974926	0.00667217854859993	0.0136032387051821	1081
TBXA2R	0.0824558482928481	0.00667750623618196	0.0136124633806806	1081
NFE2L3	0.0824211018044221	0.00670065073902517	0.0136554991540376	1081
GUCA1B	0.0823860214084705	0.00672409068394337	0.0137018821391907	1081
LOC2834(	0.0823532261534431	0.00674607028615813	0.0137452802583853	1081
PKLR	0.0823403343265326	0.00675472811813179	0.0137615289178565	1081
SGK494	0.0823051821615734	0.00677838613404735	0.0138083313449675	1081
TINAG	0.0822848433514385	0.00679210844115621	0.0138334874681116	1081
NOP58	0.0822684394714252	0.00680319406287042	0.0138532643683943	1081
ZNF296	0.0822560944327744	0.00681154745393068	0.0138688723933125	1081
TMEM38/	0.0822410480692865	0.00682174116763452	0.0138882239335015	1081
AP1M2	0.0822360576005832	0.00682512516650324	0.0138937092238488	1081
NPRL2	0.0821889038469862	0.00685717420227772	0.0139561299560401	1081
ZC3H12A	0.0821688367903022	0.00687085410844766	0.0139811470721897	1081

PDHX	0.0820780955279866	0.00693301948877176	0.0141016222698193	1081
RIN1	0.0820600388404723	0.0069454498554689	0.0141215267802201	1081
PRR3	0.0820186505184448	0.00697401736100739	0.0141724583614944	1081
DPCD	0.0820088103508072	0.00698082483811431	0.0141848614448066	1081
SLC4A2	0.0819926218538087	0.0069920370893634	0.0142014562515266	1081
PVR	0.0819919302131536	0.00699251648343966	0.0142014562515266	1081
SNRNP40	0.0819363275152113	0.00703115273408352	0.0142770458449866	1081
HMGN4	0.0819290170017945	0.00703624675240782	0.0142859494887377	1081
RNASEH1	0.0819191855163287	0.00704310262026461	0.0142984281250862	1081
SMAD5O:	0.0818807789287203	0.00706994243453713	0.0143456879878878	1081
PSMD10	0.0818498543593854	0.00709162017502671	0.0143853276030524	1081
DUSP15	0.0818367616339582	0.00710081595616707	0.014402530954187	1081
IL17RC	0.081828463174034	0.00710664998036838	0.0144114620348823	1081
MFNG	0.0817943187139109	0.00713069962985164	0.0144558663624194	1081
TSKS	0.0817229162910625	0.0071812278854111	0.0145465898191661	1081
LOC1001	0.0817208595962245	0.00718268805840046	0.0145480847513048	1081
LOC1477	0.0816510902986585	0.00723237943908542	0.0146384289963203	1081
ELL	0.0816466454150529	0.00723555560715373	0.0146433863478111	1081
SLC39A1	0.0815875027055407	0.00727793618142806	0.0147232400456787	1081
EIF3G	0.0815659259016423	0.007293453013127	0.0147487061784223	1081
TRIM78P	0.0815082570606431	0.007335070813246	0.0148283480444138	1081
DLEU2	0.081507274943573	0.00733578141527068	0.0148283480444138	1081
MKNK1	0.0814887072171502	0.0073492275382946	0.0148525470263789	1081
SKIV2L	0.0814424652826425	0.0073828104097869	0.0149174238420418	1081
LYPD2	0.0814245759616599	0.00739583923404288	0.0149422506639047	1081
SDC4P	0.0814064868825933	0.00740903448093807	0.0149674087062272	1081
CRISP1	0.0813923200874269	0.00741938330170735	0.0149853094659008	1081
CSTF3	0.0813842508099403	0.00742528368167251	0.0149942201049812	1081
DYRK4	0.0813707605460601	0.00743515735747425	0.0150126535999117	1081
NCL	0.081350388576211	0.00745009011971032	0.0150397901053777	1081
KIAA012	0.0813160251216638	0.00747533959049516	0.0150862268133165	1081
DQX1	0.0813015034928089	0.00748603276320125	0.0151041516472201	1081
DAGLB	0.0813009090272988	0.00748647079659927	0.0151041516472201	1081

PATE2	0.0812818948363103	0.00750049356459832	0.0151309275845016	1081
TXNRD1	0.0812669667456802	0.00751151935312014	0.0151501358999405	1081
NOL10	0.0812599423236578	0.00751671256422771	0.015157575035672 4	1081
SCNN1G	0.0812343614240736	0.00753565191046901	0.0151896845843905	1081
SLC43A2	0.0812127306708405	0.00755170001303556	0.0152189872711686	1081
C9orf106	0.0811924518893132	0.0075667728560191	0.0152463131810867	1081
C17orf74	0.0811691781832559	0.00758410496241151	0.015278179446778	1081
C20orf106	0.081148020224916	0.00759989228874503	0.0153076720417021	1081
TLX3	0.0811194610624032	0.00762124878967249	0.0153468669400192	1081
SCG3	0.0811139575530825	0.00762537047450994	0.0153536320048978	1081
TMEM49	0.08111017817809	0.00762820207722625	0.015357798405529	1081
LRMP	0.0810539126577621	0.00767046907507347	0.0154351812523579	1081
POU2AF1	0.0810438194124062	0.00767807331683057	0.0154457408878787	1081
TMEM62	0.0810375147717929	0.00768282665014598	0.0154492456445589	1081
ASAP3	0.0810353353871909	0.00768447039218495	0.0154510086885483	1081
GPR172B	0.0810301066182886	0.00768841533359212	0.0154559145703831	1081
NLRP3	0.080978764213253	0.00772724794543077	0.0155308200998407	1081
CKMT1B	0.0809502370885917	0.00774890008718612	0.0155727845427339	1081
MICA	0.080929184281309	0.00776491400794097	0.0156002984631466	1081
KCNK6	0.0809212460340338	0.0077709599583854	0.0156093317740227	1081
C1orf56	0.080916231022697	0.00777478167632843	0.0156154513329647	1081
CDC42EP4	0.0809097338150131	0.00777973541111896	0.0156222856715502	1081
ACTR3C	0.0808916249188077	0.00779355727679262	0.0156484811463159	1081
UCP3	0.0808663204033664	0.00781290809949315	0.0156810833580043	1081
JSRP1	0.0808556416931708	0.00782108719297921	0.0156959355949956	1081
LENG9	0.0808481321067911	0.00782684356137985	0.0157059232537102	1081
EID2B	0.0808382113278909	0.00783445400677229	0.0157196290803215	1081
SPRR1B	0.0807895929503093	0.0078718460233819	0.0157870756577147	1081
CCT8	0.0807091044055383	0.00793410014720157	0.0159053087337422	1081
BOLA1	0.0807050896814244	0.00793721684612941	0.0159099734691818	1081
CCDC151	0.0806671063628129	0.0079667581742397	0.0159660110843672	1081
LY6K	0.0806369708108678	0.00799026585242126	0.0160051611411978	1081
NDUFV1	0.0806128897500346	0.00800909520166505	0.0160412828432633	1081

EGLN2	0.0805940195932804	0.00802387778157482	0.016066098589586	1081
ESCO2	0.0805172861342226	0.0080842411652953	0.0161805303800173	1081
SNHG6	0.0805008395981126	0.00809723175837468	0.0162033111922577	1081
PFN3	0.0804938677429066	0.00810274423352975	0.0162127316830013	1081
SNX6	0.0804900161900703	0.00810579100038581	0.0162156067232147	1081
ACAD9	0.0804426745483964	0.00814332430046049	0.0162809912696285	1081
DSCAML	0.0804190797919954	0.00816208858675509	0.0163152683647729	1081
C7orf44	0.0803589836772142	0.0082100561194185	0.0163997600917884	1081
DBF4B	0.0803506066348473	0.00821676247683606	0.0164115288613784	1081
ST7OT1	0.0802827808401822	0.00827124188726043	0.0165121561608227	1081
CYLC2	0.0802771643826325	0.0082757676062945	0.0165195539465718	1081
H2AFY2	0.080264728494574	0.00828579626925883	0.0165379338000812	1081
FOXH1	0.0802537358802705	0.00829467007374441	0.016552365392331	1081
SCRT1	0.0802296474494419	0.00831414519173159	0.0165879424470073	1081
INO80E	0.0801955745762551	0.00834176233383319	0.0166413945548692	1081
TMEM132	0.0801926616207524	0.00834412717472752	0.0166444639998897	1081
MUC12	0.0801865398955833	0.00834909896091642	0.0166527325314516	1081
UPK3B	0.0801627762085053	0.0083684238290593	0.0166813673101523	1081
DIRC1	0.08015535557881	0.00837446654046895	0.0166917610030422	1081
HOMER3	0.0801133816142226	0.00840871989490412	0.0167550605621012	1081
RASSF5	0.0801052705838191	0.00841535340660316	0.0167666199560895	1081
MT1G	0.0800668489521248	0.00844683964134606	0.0168243607449987	1081
ORM2	0.0800215349466541	0.00848410930551212	0.0168952532581595	1081
C1S	0.0800187176942619	0.00848643126811927	0.0168982067704124	1081
TMEM120	0.0800089837561634	0.00849445828995125	0.0169125185067274	1081
NCCRP1	0.0800009452091762	0.00850109233031423	0.0169226991447514	1081
BLCAP	0.0800007521770238	0.00850125169247376	0.0169226991447514	1081
TRIM10	0.0799635071576009	0.00853205013811078	0.0169806510602886	1081
MCHR1	0.0799364298859379	0.00855450322632366	0.0170236557832945	1081
LETM2	0.079934276373427	0.00855629123041687	0.0170255320858107	1081
DNASE1L	0.0798998646964773	0.00858490762791145	0.0170757271094269	1081
MTCP1	0.0798631180300844	0.00861556008445494	0.0171316215478575	1081
C9orf30	0.079856471265416	0.00862111494610387	0.0171409751864649	1081

C8orf80	0.07985046644438	0.0086261360684949	0.0171458817069393	1081
TCEB3	0.0798307187422342	0.00864266715227418	0.0171736565183038	1081
COG8	0.0797928636578081	0.00867443526150217	0.0172316831025502	1081
TMCO6	0.0797863484716493	0.00867991332670614	0.0172408651120583	1081
KCNK9	0.0797837608952362	0.00868208985586746	0.0172434881343242	1081
TCEAL3	0.0797672322781158	0.00869600433045102	0.0172694210604235	1081
DCAF8L2	0.0797554995899088	0.00870589346769447	0.0172873557325486	1081
CLK3	0.0797423675843882	0.00871697395049638	0.0173076522996993	1081
PHTF1	0.0797152502653674	0.00873989475349933	0.0173497418022511	1081
OR52A5	0.0797017922583781	0.00875129003537797	0.0173689396810531	1081
CCDC102	0.0796976483975887	0.00875480143017635	0.0173741971048077	1081
RNF208	0.079686233323336	0.00876448075587132	0.0173914704866489	1081
FAM119A	0.0796421819268504	0.00880192339256456	0.0174625513830228	1081
S100P	0.0796362128892041	0.00880700789628674	0.0174709181753517	1081
MDH2	0.07962847232525	0.00881360531150558	0.0174822842654864	1081
UMODL1	0.0796051893939257	0.00883347633884034	0.0175199744851766	1081
SPINK2	0.0795911342714716	0.0088454911468838	0.0175403503449133	1081
LRRC10B	0.0795797173556274	0.00885526145259037	0.0175562682769839	1081
ABCC5	0.0795727376187667	0.0088612392743637	0.0175646625191436	1081
IMPA2	0.0795573952248032	0.00887439198009908	0.0175872726452628	1081
TRYX3	0.0795305506863376	0.00889744712558189	0.0176277608521683	1081
SUCLG1	0.0795123666044993	0.00891309466763639	0.0176552892900231	1081
TAF9	0.0794784149032512	0.00894237607780609	0.0177098079126429	1081
PPP1R12C	0.0794630098184277	0.00895569035942208	0.0177309466624627	1081
LOC40783	0.0794197967784942	0.00899313285816052	0.0178015782084744	1081
STARD3N	0.0793913898581266	0.00901782238648715	0.0178486964016294	1081
MFF	0.0793863176458004	0.00902223719197923	0.0178556801485825	1081
LMCD1	0.0793779549196788	0.00902952023774155	0.0178665833601123	1081
GPSM2	0.0793420882998393	0.00906081574227358	0.0179232259552338	1081
HDAC1	0.0792825036690302	0.00911302027986248	0.0180176458472696	1081
GPR78	0.079257151678365	0.00913531339361791	0.018058177638547	1081
CCDC109	0.0792311266051008	0.0091582489329504	0.0181017390849966	1081
COX16	0.0791706055800677	0.00921178386140976	0.0182039813506082	1081

PAEP	0.0791616383481847	0.00921973966182709	0.0182144413173972	1081
CAMP	0.0791567576259446	0.00922407245100327	0.0182193292994863	1081
PSCA	0.0791505241199162	0.00922960879281798	0.0182284773658155	1081
PBX4	0.0791273541107319	0.00925021340557028	0.0182625750068296	1081
LRFN2	0.0791270325556541	0.0092504996460506	0.0182625750068296	1081
TLL2	0.0790729227625102	0.00929877943047662	0.0183471020202695	1081
BRIX1	0.0790716754111563	0.00929989502977586	0.018347506157559	1081
SCLY	0.0790651934474485	0.00930569425344584	0.0183571495040801	1081
P2RX2	0.0790326589190494	0.00933485064797775	0.0184074555896155	1081
DRD4	0.0790188546128721	0.00934724619541322	0.0184300944119212	1081
SCARNA1	0.0790034808225166	0.00936106831617383	0.0184554245718393	1081
CNTN5	0.0789667019743927	0.00939420900674823	0.0185081995541265	1081
WNT7A	0.0789294536149723	0.00942787925170037	0.0185690875562675	1081
TRAF4	0.0789143874917388	0.00944152861235942	0.0185941532794976	1081
LRRC41	0.0788524694526989	0.00949780910158269	0.0187013355831672	1081
MS4A1	0.078812701459328	0.00953411365985157	0.0187636498317419	1081
MS4A3	0.0787930983716371	0.00955205492442185	0.0187934511624649	1081
ZSWIM3	0.0787894513431899	0.00955539609623666	0.0187981889022156	1081
CDRT1	0.0787693692733497	0.00957381263971048	0.0188304202924175	1081
LOH12CR	0.0787488079358383	0.00959270144125066	0.0188613814657676	1081
TTC32	0.0787428257038502	0.00959820328395534	0.018869503772351	1081
DGKQ	0.0787412498950208	0.00959965301854048	0.0188705123007902	1081
ZNF335	0.0787179639093667	0.00962109872270874	0.0189071343058207	1081
ANKLE1	0.0787061905604982	0.00963195785513502	0.0189266280717611	1081
SDR9C7	0.0786988494965131	0.00963873439737675	0.0189380965995469	1081
LYPD1	0.078664533953223	0.00967046742798911	0.0189948875133425	1081
FAM159A	0.0786487240047394	0.00968511882318411	0.019021811318421	1081
SLC22A6	0.0786286445952266	0.00970375529550535	0.0190546983553909	1081
DEFB126	0.0785689710247508	0.00975932916481539	0.019160090247072	1081
DHRS1	0.0785660321293024	0.00976207345604141	0.0191636103851057	1081
SSR3	0.0785303123015378	0.00979548297414572	0.0192251005534239	1081
OR56A3	0.0785294828617596	0.00979625997423163	0.0192251005534239	1081
SPRR4	0.0785134150522598	0.00981132275470929	0.0192471610569304	1081

GCDH	0.0784914980221797	0.00983190212026868	0.0192818991640199	1081
CCDC21	0.0784727620954377	0.00984952497770289	0.0193142037605116	1081
EVI5L	0.0784544220312062	0.00986680270611689	0.0193428124284473	1081
KIF19	0.0784517358084009	0.00986933559410322	0.0193440129931124	1081
CTRB2	0.0784408525599935	0.00987960352660613	0.0193622544064092	1081
SERPINA	0.0784344896137398	0.0098856111271822	0.0193702593285756	1081
LOC2839	0.0784321284406645	0.00988784126465664	0.0193727448236246	1081
ZNF32	0.0783973354464908	0.00992075532087254	0.0194315620747669	1081
KRT33B	0.0783938566825653	0.00992405157385067	0.0194361286170739	1081
PDLIM2	0.0783353851246354	0.00997960146646684	0.0195326966469448	1081
SLC37A4	0.0783131232225567	0.010000823590807	0.0195693629176111	1081
SNORA76	0.0782352904826022	0.0100753368781958	0.0197017726083531	1081
MAT1A	0.0782303356567188	0.0100800970602595	0.0197091677452129	1081
S100A10	0.0782009979659089	0.0101083232592494	0.0197586039256336	1081
DMRT1	0.0781688340491313	0.0101393493539684	0.0198134826593941	1081
RRP7A	0.0781350814701265	0.0101719989333053	0.0198753558207018	1081
ERCC3	0.0781069703082183	0.0101992627593898	0.0199247622466939	1081
MIOX	0.0780825854796202	0.010222965170008	0.0199691295791537	1081
SYAP1	0.0780579903456635	0.0102469215896529	0.0200139844312156	1081
CLSTN3	0.0780526243436397	0.0102521548678672	0.0200222646450009	1081
FLJ40330	0.0780451189659697	0.0102594785913342	0.0200346254577769	1081
DCTPP1	0.0780312272732113	0.010273046330962	0.0200591759559202	1081
ACAP3	0.0779715172706002	0.0103315456719104	0.0201694919624609	1081
OR10H4	0.0779341192438939	0.0103683360337742	0.0202354320287136	1081
PADI1	0.0779285069848815	0.0103738671364098	0.0202442655684371	1081
FCHSD1	0.0779226583527959	0.0103796339838746	0.020252677777597	1081
NLRP7	0.0778873839512745	0.0104144755656303	0.0203156396116609	1081
CNFN	0.0778846614690124	0.0104171689575746	0.0203189260893048	1081
KLRC4	0.0778444151633713	0.0104570574882293	0.0203868598761617	1081
FBXL8	0.0778050059844168	0.0104962477706451	0.0204573250135105	1081
MRGPRD	0.0777886519185918	0.0105125492694289	0.0204871147366398	1081
CYB561D	0.0777464396279176	0.0105547298670558	0.0205673276428553	1081
SLC9A5	0.0777447552728223	0.0105564160720635	0.0205686238293568	1081

SMPD1	0.0777341963011458	0.0105669920932451	0.0205852485947609	1081
RP9P	0.0777223021089714	0.0105789167615769	0.0206064859951621	1081
KIAA0174	0.077677878804985	0.0106235596403553	0.0206874440749113	1081
FXYD4	0.0776477620701711	0.0106539203213303	0.0207405513020099	1081
ASTL	0.0776444720288659	0.0106572416759945	0.0207450124227373	1081
MFSD10	0.0776321505066911	0.0106696886411895	0.0207652282558944	1081
ZNF346	0.0776285746726797	0.0106733032960277	0.0207702564623723	1081
FAM75A6	0.0775776131474621	0.0107249363892137	0.0208687185898494	1081
COPZ1	0.0775678252014222	0.010734878669788	0.0208860470159242	1081
C1orf93	0.0774946577310735	0.0108094594140385	0.0210088335644771	1081
C10orf91	0.0774860528878465	0.0108182606230172	0.0210239108866179	1081
GMPS	0.0774773526007436	0.0108271659207503	0.0210371583211338	1081
C15orf61	0.077459342125993	0.0108456214685004	0.0210669216548974	1081
MEAF6	0.0774218450779812	0.0108841347487859	0.0211357943977195	1081
NUP210	0.0774217564559608	0.0108842259162046	0.0211357943977195	1081
ASCC1	0.0774149261020362	0.0108912544907405	0.0211453663951395	1081
TIMM9	0.0773888035327513	0.0109181723285248	0.0211894587242902	1081
RHAG	0.0773848984304658	0.0109222013854261	0.0211952361920247	1081
SPANXC	0.0773659481356177	0.0109417719385299	0.0212311689175193	1081
CDSN	0.0773578525749163	0.0109501419556361	0.0212453635458239	1081
ANXA11	0.077351262252189	0.0109569598988946	0.021256544410943	1081
PRR23A	0.0773154054378714	0.0109941212033322	0.0213224772928783	1081
C1orf61	0.0772655236589522	0.0110460036978755	0.0214127929651369	1081
ANP32E	0.0772545926543484	0.0110574021101502	0.021432826470603	1081
PLD4	0.0772421796141956	0.0110703585816178	0.0214558758540206	1081
SETD6	0.0772240469365694	0.0110893092981224	0.0214905373555243	1081
SLC22A1	0.0772005874266205	0.0111138698606932	0.0215360627494868	1081
PPM1N	0.0771677458209362	0.0111483339158704	0.0215945371860779	1081
CCDC106	0.0770778052239478	0.0112432035483873	0.0217615618257361	1081
ZNF414	0.0770650909466851	0.0112566722047902	0.0217813525660839	1081
WDR38	0.0770623784936298	0.0112595474475379	0.0217848236007156	1081
CA5BP	0.0770496756844461	0.0112730212925477	0.0218046100756959	1081
C15orf57	0.0769920568730213	0.0113343171236665	0.0219105477839229	1081

FAM162A	0.0769660232619251	0.011362108890352	0.0219600579100202	1081
OR1S1	0.0769568126935522	0.0113719559442747	0.0219748731412388	1081
STYXL1	0.0769063781164324	0.0114260099961457	0.0220750907617105	1081
TMEM39I	0.0768732057527685	0.0114616870443241	0.0221397723183343	1081
LRDD	0.0768628289216172	0.0114728676360628	0.0221592443459716	1081
SIRPA	0.0768534937536843	0.0114829341388161	0.0221725480222745	1081
L1CAM	0.0768429077479513	0.0114943589436285	0.0221922427563156	1081
SNW1	0.0768372357615384	0.0115004845029678	0.0222019415728114	1081
LOC1002	0.0768356043932097	0.0115022468629127	0.0222032160840722	1081
MAG	0.0768120505682689	0.0115277186434725	0.0222438593939418	1081
FOXD4L3	0.0767762759060702	0.0115665020122626	0.0223165580918523	1081
ZG16	0.0767719767841025	0.0115711704708155	0.0223234274214306	1081
HRH3	0.0767256975450327	0.0116215313774369	0.0224162916122622	1081
HTRA2	0.076697015327429	0.011652840654782	0.0224745308751157	1081
SPRR3	0.0766944602293189	0.0116556334010383	0.0224777651602448	1081
CLEC1B	0.0766910722520375	0.0116593374064711	0.0224827560116168	1081
PMVK	0.0766430410758287	0.0117119611984357	0.0225755868665666	1081
NFYC	0.0766241321364032	0.0117327358115742	0.0226113042116295	1081
ANP32D	0.0765884906967298	0.0117719826217498	0.0226739257974137	1081
DEM1	0.0765740622035525	0.0117879036890926	0.0227024206325785	1081
ASPHD1	0.0765668456548862	0.0117958739153154	0.0227155988934164	1081
TMEM203	0.0765413520453031	0.0118240682363036	0.0227655409175509	1081
TFEB	0.0765331908123477	0.0118331066465054	0.0227785889530675	1081
GRAP	0.0765185689542693	0.0118493153781736	0.0228054321480183	1081
MFSD6L	0.0765110366769984	0.0118576727850805	0.0228193368604745	1081
LRRC14	0.076439834199397	0.011936933450173	0.0229611122305896	1081
TAGLN3	0.0764397362036654	0.0119370428588218	0.0229611122305896	1081
SYT12	0.0764355961294575	0.0119416659113639	0.0229678117036878	1081
LSG1	0.0763502317339194	0.0120373428266178	0.0231452010348588	1081
SMARCB	0.0763477028500966	0.0120401875337375	0.0231483210769673	1081
PRSS22	0.0763467463287803	0.0120412636666935	0.0231483210769673	1081
DDX19A	0.0763199903708421	0.0120713999384374	0.0231974007211506	1081
DUT	0.0762935027941898	0.012101299660008	0.0232482053834503	1081

MANBAL	0.0762860616821046	0.0121097111252543	0.0232621465260082	1081
CIB4	0.0762717848692776	0.0121258641855787	0.0232885572252698	1081
BANP	0.0762671644537277	0.0121310958951812	0.0232943405593771	1081
MED15	0.0762609561134476	0.0121381287618238	0.0233056237044357	1081
RSPH9	0.0762463409909956	0.0121546991580833	0.023335215337805	1081
PGK2	0.0762382418789685	0.0121638904219399	0.0233506358442899	1081
C7orf25	0.076232259521446	0.0121706834393215	0.0233614500128758	1081
GOLGA6I	0.0762008335621767	0.012206423046131	0.0234233560929995	1081
OR7A17	0.0761706220718922	0.0122408690005809	0.0234827450734885	1081
LOC10021	0.0761666713202751	0.0122453798452596	0.0234891617770668	1081
WDR66	0.0761583025490824	0.0122549399042144	0.0235052617700084	1081
DECR2	0.0761446012324285	0.0122706058481383	0.0235308286350067	1081
ZNF695	0.0761407361919533	0.0122750282982707	0.023537068824354	1081
LCN1	0.0760576963951741	0.0123703851713636	0.0236996109631117	1081
PSAPL1	0.0760462845722416	0.0123835407598372	0.0237225588253062	1081
C2orf29	0.0760433206209507	0.0123869596367773	0.0237268519428429	1081
POLR1C	0.0759527744330669	0.0124918065369888	0.0239140399779188	1081
AGAP3	0.0759444299805366	0.01250150827844	0.0239303386800811	1081
ZNF524	0.0758883377373164	0.0125668974574243	0.024048651019266	1081
A2LD1	0.0758760417558194	0.0125812717645687	0.0240738715517892	1081
DDX5	0.0758582108241256	0.0126021423897709	0.0241046485417712	1081
CBX4	0.0758508909204429	0.01261071899767	0.0241164737239474	1081
PAK1	0.0758369688873059	0.0126270454509868	0.0241454039497038	1081
QTRT1	0.0758338519433016	0.0126307032692857	0.0241501060516098	1081
SLC39A13	0.0757751901449762	0.0126997194043835	0.0242751539426231	1081
DNAJC11	0.0757620533001139	0.0127152206255206	0.0243024781310354	1081
LOC55064	0.0757606587322852	0.0127168671698736	0.0243033193375491	1081
DNAJC2	0.0757529931080403	0.0127259212175263	0.0243160090038004	1081
GGA3	0.0757311241303807	0.0127517824969957	0.0243631125191557	1081
CASP14	0.0757141834225239	0.01277184773876	0.0243968208512536	1081
SMARCA	0.0756836758659733	0.012808052468589	0.0244567030312535	1081
MUL1	0.0756596394025914	0.0128366414990629	0.0245089700501017	1081
LHX1	0.0756468250063278	0.0128519060065942	0.0245355893548989	1081

FOXD3	0.0756410621023444	0.0128587760040304	0.0245442526626106	1081
C21orf56	0.0756271431224835	0.0128753823002426	0.024571293713375	1081
S100A16	0.0756078822584019	0.012898393081647	0.0246105444809413	1081
DBF4	0.0755644327791881	0.0129504352607974	0.0246981462017196	1081
GNPTG	0.075553706630464	0.0129633111589154	0.0247196932502032	1081
COX4I2	0.0755529768619907	0.0129641875993247	0.0247196932502032	1081
SLURP1	0.0755286903220029	0.0129933852244169	0.0247730215167404	1081
FGFBP3	0.0754433346875544	0.0130964623154647	0.0249542396327651	1081
BRSK2	0.0754430354713984	0.0130968249211575	0.0249542396327651	1081
SSX2	0.0753879011790731	0.0131637910446331	0.0250741840576905	1081
TTC4	0.0753772298561137	0.0131767873029037	0.0250965661545802	1081
ATP5G2	0.0753605062924733	0.0131971771790725	0.0251306488583434	1081
ACY3	0.0753286257803762	0.013236124051642	0.0252024309092937	1081
OR5T1	0.0753055332706319	0.0132643983865968	0.025251493621054	1081
SUB1	0.0752847226482687	0.0132899244555536	0.0252976970761719	1081
C3orf43	0.0752721647873247	0.0133053487527384	0.0253246646470063	1081
LZTS2	0.0752650069774562	0.0133141474609414	0.0253342307169798	1081
SLC36A4	0.075254378476095	0.0133272219778524	0.0253519250938467	1081
CCDC72	0.0752437620519738	0.0133402929506115	0.0253743934935387	1081
RPL19	0.0752362223454298	0.0133495827300735	0.0253853902590116	1081
KRT80	0.075196732272554	0.0133983323430445	0.0254703600123271	1081
MAP1LC3	0.0751920138902907	0.0134041675756319	0.0254790484818932	1081
TTC15	0.0751905020995732	0.0134060376845561	0.025480198995696	1081
MRPL1	0.0751581157676648	0.0134461554290461	0.0255516273104551	1081
HAR1B	0.0751284488344719	0.0134829975226471	0.0256168051583256	1081
RPIA	0.0751236825626862	0.0134889248490831	0.0256232334827704	1081
SERPINA(	0.0750680988936612	0.0135582185381049	0.0257402989774878	1081
CCDC42B	0.0750578649242788	0.0135710109521358	0.025759668737085	1081
TNNI3	0.0750515945113138	0.0135788541990612	0.0257673340090513	1081
MUSK	0.0750480829027855	0.0135832483857662	0.0257719915438717	1081
UBE2V2	0.0750320119935853	0.0136033744342047	0.0258041410524533	1081
RBM14	0.0750296441639228	0.0136063419558782	0.0258073400528356	1081
HTR3C	0.0750209842800669	0.0136171999627379	0.0258255030360907	1081

MAP3K8	0.0749736612177848	0.0136766700738659	0.0259345653818585	1081
C6orf141	0.0749731761421268	0.013677280844719	0.0259345653818585	1081
C11orf86	0.0749644151631835	0.0136883161536744	0.0259530474273666	1081
C21orf131	0.0749569333987127	0.013697746383226	0.0259684830500741	1081
ACRV1	0.07489159394911	0.0137803459254807	0.0261127898286905	1081
HEMGN	0.0748686354171543	0.013809473302068	0.0261655229185628	1081
PIGW	0.0748564686554166	0.0138249312105749	0.0261923482777232	1081
CDX1	0.0748378970396213	0.013848555925748	0.0262321729312829	1081
RFX5	0.0748131848692172	0.0138800471552963	0.0262844096581542	1081
UCP2	0.0748120349517598	0.0138815140549647	0.0262847166686026	1081
VPS18	0.0748051511293167	0.0138902983063392	0.0262988777613914	1081
FAM27A	0.074746997180176	0.0139647027108353	0.0264298136142218	1081
GGT5	0.0747387806902689	0.0139752434814373	0.0264447942826934	1081
DAOA	0.0747214973536912	0.0139974388237281	0.0264818186611573	1081
CHST2	0.0747186861639126	0.0140010519033747	0.0264861668319545	1081
LARP1	0.0746984355356384	0.0140271033076661	0.026532957383374	1081
OR4N5	0.0746503410610435	0.0140891457024988	0.0266403078821887	1081
LRRC42	0.0746287701609489	0.0141170508689877	0.0266880621017039	1081
SLN	0.0746130042995288	0.0141374771519782	0.0267191553829252	1081
FBXO31	0.0745484597215531	0.0142213730610638	0.0268676320280531	1081
PCSK1N	0.0745304573969812	0.0142448508212072	0.026904417756724	1081
CCDC127	0.0745201047766094	0.0142583676666844	0.0269274225785467	1081
DAK	0.0745154641273001	0.0142644303734968	0.0269363470073204	1081
EPHA10	0.0744871309120343	0.0143014951634622	0.0270003281489486	1081
SEL1L3	0.0744854677773637	0.0143036734676386	0.0270003281489486	1081
VAT1	0.074455499244478	0.0143429751112655	0.027066907130346	1081
ACOXL	0.0744536070287989	0.0143454598098256	0.0270690603099416	1081
L3MBTL2	0.0744330793663064	0.0143724393872787	0.0271098119341506	1081
DHX37	0.0744144305230554	0.0143969883589347	0.0271535746176144	1081
KRT10	0.0743687736789297	0.0144572460813755	0.0272544658721046	1081
EMID1	0.0743138240274169	0.0145300628371301	0.0273840505055652	1081
SSX3	0.0742987068287569	0.014550152011717	0.027416781617813	1081
UNC13A	0.0742864017298667	0.0145665222505092	0.0274450608620004	1081

ERAP2	0.0742697405059914	0.0145887135554177	0.0274817313048336	1081
TACR2	0.0742647648408886	0.0145953464787046	0.0274916554290326	1081
LOC10012	0.0742583424367033	0.0146039119354889	0.0275052174589027	1081
MMP26	0.0742514219857255	0.0146131465824351	0.0275174647507156	1081
RNFT1	0.0742405470635388	0.0146276684606532	0.0275396617887719	1081
CYTSB	0.074203416178891	0.0146773471459812	0.0276254469079503	1081
MON1A	0.0741833365261831	0.0147042742267971	0.0276692518199511	1081
CLCNKA	0.0741770072654375	0.0147127708577695	0.0276817753740326	1081
MYH15	0.0741481800900964	0.0147515241980683	0.0277515725224499	1081
RCSD1	0.0741473637112592	0.0147526229904649	0.0277515725224499	1081
HMG20B	0.0740807886964759	0.0148424714046694	0.027907558936631	1081
PMM2	0.0740727361280667	0.0148533715796428	0.0279254475475411	1081
ARL8A	0.0740348660373447	0.0149047280726984	0.0280115446426447	1081
DIAPH3	0.0740089765078106	0.0149399273044871	0.0280750779429937	1081
HYOU1	0.0739987193326672	0.014953893105852	0.0280987012981427	1081
FAM57B	0.0739826219371629	0.0149758338983717	0.028132056497827	1081
CABP2	0.0739220180683318	0.0150586913497611	0.0282797927883787	1081
MRPL39	0.0739026501283096	0.0150852560351818	0.0283191205692608	1081
TMEM179	0.0739014543012203	0.0150868975582256	0.0283195631112984	1081
CDIPT	0.0738338134193333	0.0151800049741274	0.0284837183498321	1081
SFTPD	0.0738252540553299	0.0151918228474669	0.0285032384522885	1081
MCF2	0.0738141450050422	0.015207173124304	0.0285267252620453	1081
LOC38815	0.073803654265619	0.0152216815501066	0.0285512825723368	1081
SLC25A14	0.0738008677152583	0.0152255373235761	0.0285558560081409	1081
SLC16A13	0.0737966920436638	0.0152313168436091	0.0285640362888201	1081
ZNF394	0.0737862195322759	0.0152458202653227	0.0285885738848483	1081
PROZ	0.073773418270976	0.0152635652739061	0.028616521399985	1081
OSBPL2	0.0737682142974864	0.0152707841713141	0.0286273913205957	1081
C20orf199	0.0737172681275831	0.0153416148542208	0.0287494726733283	1081
KIF25	0.0737144670908698	0.0153455175101667	0.0287533929010141	1081
OR6S1	0.0736911229681471	0.0153780765981962	0.0288097603525213	1081
AIMP1	0.0736611363121483	0.0154199894144604	0.0288855948255817	1081
C7orf36	0.0736531809354354	0.015431125606642	0.0289037680470297	1081

DGAT2L6	0.073614635444785	0.0154851829809396	0.0289888496562613	1081
CXorf50B	0.0736038940385288	0.0155002766774251	0.0290144093724891	1081
MRPS2	0.0735924662982681	0.0155163489955558	0.0290417960155599	1081
FGD1	0.0735909658164833	0.0155184604056827	0.0290430495050612	1081
C1orf230	0.0735737838781236	0.0155426560574327	0.029082928318501	1081
IDUA	0.0735671335621603	0.0155520299450187	0.0290950634511888	1081
SHROOM	0.0735607507156921	0.0155610314953635	0.0291092004340327	1081
ST7OT3	0.0735548601719561	0.0155693428266808	0.0291220437551982	1081
GMDS	0.0735488538848603	0.0155778214835785	0.0291351976405802	1081
CENPI	0.0735333915662542	0.0155996672297013	0.0291733472282151	1081
MAZ	0.0735115081231937	0.0156306309870748	0.0292258271054968	1081
MAGEA1(	0.0734754624429705	0.0156817511916508	0.0293159686113406	1081
GPSM1	0.0734616654855835	0.0157013569552869	0.0293498966191198	1081
TRY6	0.073447743975804	0.0157211615323395	0.0293814638713219	1081
PYGB	0.0734444103644247	0.0157259071440199	0.0293876066248869	1081
SPTBN4	0.0734139595577663	0.0157693141168662	0.0294659895078629	1081
PEBP1	0.0734108787523476	0.0157737115957613	0.0294714728340392	1081
PLAA	0.0734030697458346	0.0157848628381291	0.0294888112061455	1081
KRT72	0.0734023303243716	0.0157859190889587	0.0294888112061455	1081
MAGED1	0.0733825441524255	0.0158142063607127	0.0295389139672284	1081
CRCT1	0.0733264597686632	0.015894629395471	0.0296781267190438	1081
RARS	0.0733056691835088	0.0159245334828921	0.029731207322786	1081
LOC44245	0.0733013992851431	0.0159306811921157	0.0297399288865879	1081
XRCC6	0.073300116502659	0.0159325285222021	0.029740621486264	1081
ABCG8	0.0732809954282936	0.0159600870141495	0.0297810256483922	1081
SUZ12P	0.0732639310218514	0.0159847166413752	0.0298214592276813	1081
MARCKS	0.0732555162437192	0.0159968742520529	0.0298413771467364	1081
FAM82A2	0.0732224117600289	0.0160447821996701	0.0299196646683656	1081
ID3	0.0732195110830915	0.0160489859794796	0.0299247336686984	1081
OR5H1	0.0732069953764743	0.0160671353424113	0.0299530299345619	1081
LEPRE1	0.0731989358547609	0.0160788322063689	0.0299720620706304	1081
WIT1	0.0731959236526602	0.0160832057637671	0.0299774407948824	1081
PRM3	0.0731911307832735	0.0160901669082196	0.0299848670088884	1081

RANBP9	0.0731883320032334	0.0160942330682398	0.0299896702580419	1081
FGA	0.0731780974110032	0.0161091099027114	0.0300146151489199	1081
SLC26A11	0.07308334052056	0.0162474211785714	0.0302639204477413	1081
SLC12A9	0.0730015431431926	0.016367653138008	0.0304737867343041	1081
LHB	0.0729878425710914	0.016387867413018	0.030505783499838	1081
E2F7	0.0729467917748485	0.0164485662942463	0.0306012610678112	1081
CHAC2	0.0729459643221199	0.0164497918145112	0.0306012610678112	1081
KRI1	0.072908411998456	0.0165054940233724	0.030700128209019	1081
OTOA	0.0729018840531046	0.016515193933078	0.0307115832901187	1081
TMEM126	0.072855172273658	0.0165847492316736	0.0308323895599912	1081
ELAVL4	0.0728451343659786	0.0165997294803288	0.030857391379646	1081
SLC35C1	0.0728023642145446	0.0166636913447022	0.0309620053623894	1081
AHCY	0.0727582028529325	0.0167299603997256	0.0310712117627433	1081
IFNA4	0.0727580581581197	0.0167301779096787	0.0310712117627433	1081
VARS	0.0727530866801415	0.0167376527033951	0.0310822284024607	1081
TRMT12	0.0727406269395998	0.0167563992319677	0.0311141729678301	1081
ALPI	0.0727244211909681	0.0167808094243992	0.0311566272676979	1081
MOBKL2A	0.0727120201951281	0.0167995096906569	0.0311884732023117	1081
PLTP	0.0726616047852175	0.0168757225891856	0.0313126491258308	1081
HDAC3	0.0726351214072737	0.0169158787501193	0.0313784877920031	1081
ADAM15	0.0726091019679447	0.0169554129848568	0.0314460315393059	1081
SLC9A3R1	0.0725812634386155	0.0169978008065501	0.0315188417938099	1081
LOC10012	0.0725710886953348	0.0170133163473874	0.0315418054314486	1081
DCAF13	0.0725494388002406	0.01704637173505	0.0316001802339727	1081
LOC10013	0.0725073407868257	0.0171108087530773	0.0317097055618572	1081
C8orf71	0.0724951342277481	0.0171295324995759	0.0317368189279828	1081
FAM75A5	0.0724760874664792	0.0171587843481758	0.0317880918423765	1081
EIF5B	0.0724660457444196	0.0171742239470973	0.0318137693254506	1081
BRIP1	0.0724605116519642	0.0171827380636276	0.0318266144071146	1081
MED24	0.0724249567763612	0.0172375269355124	0.0319196563144431	1081
GGTLC2	0.0724248293588684	0.017237723556457	0.0319196563144431	1081
OR4K13	0.0724157973974671	0.0172516659955489	0.0319425378198835	1081
AMBN	0.0724136300107604	0.0172550132116705	0.0319457992049538	1081

C19orf20	0.0723813616410906	0.0173049143734298	0.03203229808442	1081
KLC3	0.0723569287131281	0.0173427825164122	0.0320964955253214	1081
VWCE	0.0723552570815675	0.0173453759975966	0.0320983464515509	1081
GPRC5C	0.0723488676601065	0.0173552921045012	0.0321082584517074	1081
TMEM97	0.0722939834735463	0.0174406746696907	0.032250999744898	1081
DECR1	0.072267389246165	0.0174821790641379	0.032324781757417	1081
MARCKS	0.0722545278120054	0.0175022823212385	0.032356013339575	1081
DTHD1	0.0722533927736524	0.0175040574314118	0.032356325652027	1081
CLEC3A	0.0722094560472635	0.0175728922448946	0.0324746274904505	1081
ARF3	0.0721866772336239	0.0176086725518619	0.0325377644980057	1081
BCL3	0.0721357194102308	0.0176889466151558	0.0326711130065384	1081
KRT75	0.0721283552592117	0.0177005738390195	0.0326865945537673	1081
SULT2A1	0.0721096635814734	0.0177301160818023	0.0327351469588404	1081
C2orf79	0.0720971611142706	0.017749900337091	0.032768671397608	1081
C1orf144	0.0720926018719278	0.0177571198158083	0.0327789955731199	1081
PROK2	0.0720752660616731	0.0177845942273003	0.0328267042980543	1081
DGKA	0.0720471169655631	0.0178292851604716	0.0329021350429227	1081
SMEK3P	0.0720433665194059	0.0178352469710797	0.0329051241168972	1081
SUPT7L	0.0720167247973556	0.0178776475994059	0.0329712726256553	1081
TFDP2	0.0720103674844779	0.0178877783644766	0.0329839171764193	1081
POLRMT	0.071998713889342	0.0179063621201941	0.0330151624484084	1081
C11orf49	0.0719966889391214	0.0179095929898039	0.0330157955579546	1081
RTEL1	0.0719964443248427	0.0179099833138411	0.0330157955579546	1081
HTR5A	0.0719939407158522	0.0179139786792968	0.0330201391247539	1081
PGAM4	0.0719921057319625	0.0179169075196072	0.0330225161923594	1081
ADSL	0.0719447292749495	0.0179926708324855	0.0331530553297741	1081
RPS21	0.071936599305913	0.0180057002195756	0.0331740288022087	1081
PAPL	0.0719308081170206	0.0180149864241647	0.0331881025619386	1081
DHDDS	0.0719214524238834	0.0180299971819669	0.0332127188396783	1081
LIMK1	0.0719167687711601	0.0180375159849341	0.0332235310886438	1081
SPAG1	0.0719057424551477	0.0180552276771152	0.0332531140570019	1081
MCM7	0.0718914723890036	0.018078172421027	0.0332892855047155	1081
PTGER1	0.0718359920739575	0.0181676213296609	0.0334448260701837	1081

HAUS1	0.0717949191466437	0.0182340908065455	0.0335549245727467	1081
FAM75A3	0.0717754278062038	0.0182657085189769	0.0336100381909746	1081
DNAJB1	0.0717580561929998	0.0182939281200717	0.0336558157058306	1081
ITFG1	0.0717546575355149	0.018299453580564	0.0336629068012476	1081
AAAS	0.0717448663506514	0.0183153800245155	0.0336829770489196	1081
DOLPP1	0.0717285905071625	0.018341881296148	0.0337255566548834	1081
CELA2A	0.0716899943729526	0.0184048597929336	0.033825919216189	1081
CHRNA1(	0.0716889041143987	0.0184066415428978	0.0338261078162448	1081
ATP5F1	0.0716850037098373	0.0184130169954268	0.0338347375146285	1081
GAL	0.0716826570981494	0.01841685360725	0.0338387008316355	1081
RAD54B	0.0716595658428676	0.018454644124734	0.03389994331705	1081
U2AF2	0.0716592060576492	0.0184552334752091	0.03389994331705	1081
CCL19	0.0716356718105418	0.0184938197762741	0.0339638635713148	1081
ALPP	0.0716348979570537	0.0184950897680478	0.0339638635713148	1081
FGF8	0.071623360376238	0.0185140334433737	0.0339955522483604	1081
ORMDL3	0.0715877595120868	0.0185725938006151	0.0340968652249513	1081
SMPD2	0.071563186634352	0.0186131084233603	0.0341681309630574	1081
ANXA9	0.0715375615041017	0.0186554401825245	0.0342333614936196	1081
CPSF4L	0.0715317289368378	0.0186650871091938	0.0342479442403415	1081
ADAM19	0.07150382475313	0.0187113002886281	0.034315906426815	1081
PPP4R4	0.0714950426482077	0.0187258653313765	0.0343344458997524	1081
NQO1	0.0714760949600291	0.0187573236432744	0.0343827374698601	1081
FAM134C	0.0714596927435626	0.0187845930483245	0.0344264580566319	1081
BAT3	0.0714545368453318	0.0187931721059264	0.0344390477643852	1081
CCDC154	0.0714457872561319	0.0188077386483335	0.0344626064281133	1081
SSX1	0.0714347278887583	0.018826164678752	0.0344900952576809	1081
SLC23A1	0.0714144180599131	0.018860043985967	0.0345458798051742	1081
C16orf90	0.0713899126310477	0.0189009929472601	0.034608298756822	1081
SUMO1P3	0.0713876801301613	0.0189047273478491	0.0346119905864237	1081
CXorf49B	0.071373406709048	0.0189286183479418	0.034646285355196	1081
HTR1D	0.0713653457896835	0.0189421224445324	0.0346678528795408	1081
ITGA4	0.0713449745866079	0.0189762869083766	0.0347257964584989	1081
SMPX	0.0713444128568475	0.01897722974493	0.0347257964584989	1081

SELT	0.0713367343727094	0.018990121815195	0.0347462310596824	1081
MYF6	0.0713202780396328	0.0190177775343647	0.0347905129340517	1081
C16orf92	0.0713079868390438	0.0190384564678561	0.0348251798370072	1081
CPN1	0.0713064198150039	0.0190410942673132	0.0348266972248997	1081
SSTR2	0.0712961538531233	0.0190583830376078	0.0348521356474771	1081
TP53RK	0.0712914113509091	0.0190663744496605	0.0348624393109098	1081
DDX4	0.0712824415172214	0.0190814971784859	0.0348849043162354	1081
C14orf68	0.0712567830223188	0.019124814060982	0.0349577514979114	1081
DEF8	0.0712526411810262	0.0191318143923342	0.0349673744269254	1081
IER5	0.0712030348642049	0.0192158304711724	0.0351076668353693	1081
NME4	0.0712025215485046	0.0192167015325401	0.0351076668353693	1081
SAT2	0.0712021766737878	0.0192172867805221	0.0351076668353693	1081
ZNF668	0.0711828022730452	0.0192501898862994	0.0351613995157767	1081
EEF1A2	0.0711790891357498	0.0192565014346922	0.0351697390210222	1081
GAGE2D	0.0711641628727906	0.0192818911518815	0.0352129178909123	1081
LOC10013	0.0711353502545483	0.0193309843323184	0.0352897761100637	1081
TMUB2	0.0710970549571151	0.019396403650016	0.0353931658994179	1081
C3orf10	0.0710371634691093	0.0194991027412533	0.0355580179888249	1081
IP6K3	0.0709688508726439	0.019616820778754	0.0357500320777999	1081
CCL23	0.0709640356044522	0.0196251419113512	0.0357587268274394	1081
CCDC142	0.070951709288837	0.0196464567070314	0.0357943267037304	1081
LSS	0.070950491320119	0.0196485639271394	0.0357949285867447	1081
BASE	0.0709245902026162	0.0196934224070091	0.0358669193010757	1081
LOC10013	0.0708883063147024	0.019756413125171	0.0359751371604963	1081
PRAC	0.0708581023286661	0.0198089827439267	0.0360676027997472	1081
MLST8	0.070830559059055	0.0198570277041588	0.0361518141075716	1081
GCSH	0.0707747866882794	0.0199546251820382	0.0363167604117075	1081
KIF14	0.0707251864844802	0.0200417728623107	0.0364585077037429	1081
NAT15	0.0706740232140763	0.020132014238781	0.0366193613399767	1081
KIF6	0.0706606747629075	0.0201556162955808	0.0366523641699292	1081
TUBBP5	0.0706551991755354	0.0201653049243577	0.0366666727774335	1081
SLC6A17	0.0706501925271071	0.0201741673534958	0.0366794766548892	1081
FRG1	0.0706468384510635	0.0201801064094863	0.0366868891127703	1081

KIF17	0.0706190401796299	0.0202293874203269	0.0367666013697659	1081
CST9L	0.0705542479011415	0.0203446589359325	0.0369561004746943	1081
PDZD3	0.0705301150760614	0.0203877395074283	0.0370310172551747	1081
GAPT	0.0705111795359236	0.0204215977907262	0.0370891712490245	1081
KCNIP3	0.0705004409267255	0.0204408210510796	0.0371105545197214	1081
NAB2	0.0704716100258556	0.0204925094302116	0.0371877851068924	1081
TNFRSF1:	0.0704655767634409	0.0205033403255734	0.0372040885298753	1081
MT1A	0.0704512594479084	0.0205290626841878	0.0372440532936746	1081
KDM2B	0.0704349017293467	0.020558485203525	0.0372891612423866	1081
LCTL	0.0704333209376724	0.0205613305078397	0.0372891612423866	1081
RBKS	0.0704122262565582	0.0205993321560089	0.037351354300882	1081
PITRM1	0.0704099229081223	0.0206034852935624	0.037355522566719	1081
CA9	0.070405644833671	0.020611200968587	0.0373661486242066	1081
PADI3	0.0703894417755191	0.020640446633529	0.0374158010827358	1081
EML2	0.0703552776258227	0.0207022295510603	0.0375142947113528	1081
ASL	0.0702817623530675	0.0208357217937814	0.0377358280621888	1081
RASA4P	0.0702676575125007	0.0208614194687868	0.0377789729592511	1081
C12orf70	0.0702226988462716	0.0209435140595189	0.0379174162603583	1081
TCAM1P	0.0702056911191348	0.0209746434103846	0.0379607766448319	1081
STRN4	0.0701911621155999	0.0210012678192163	0.0380014856928152	1081
KRT37	0.0701709322026099	0.021038388127278	0.0380652352096296	1081
LOC1001:	0.0701670674635905	0.0210454861124174	0.0380746580812436	1081
WDR4	0.0701642517948682	0.021050658685515	0.0380805961942978	1081
RAET1G	0.0700876335021687	0.0211918368690325	0.038298156788971	1081
AKR7A2	0.0700370617464214	0.021285471857166	0.0384535764113182	1081
CALHM2	0.0700207222964621	0.0213158016100084	0.0384980117835413	1081
C6orf222	0.0699586062949862	0.0214314464081251	0.0386895320272139	1081
SNORA75	0.069954511457642	0.0214390891251115	0.0386998611492269	1081
PSMG2	0.0699108356477745	0.0215207544155785	0.0388298788805937	1081
CST11	0.0699064330059389	0.0215290014876855	0.0388378020031722	1081
DDX23	0.0699039292336466	0.0215336928041549	0.0388393089390008	1081
DCTN2	0.069864900690172	0.021606935546732	0.0389540351904752	1081
SPHK2	0.0698529314847999	0.0216294408508285	0.0389910591445136	1081

SLC38A7	0.069849516052743	0.0216358665113137	0.038999152726415	1081
C22orf41	0.0698355323671418	0.0216621921791613	0.0390431118769978	1081
TERC	0.0698312937958648	0.0216701772045663	0.03905400964269	1081
SERPINA4	0.0698210230586068	0.021689536812135	0.0390819068944955	1081
CTU1	0.0698082303654684	0.0217136711483652	0.0391114006870365	1081
ZBTB17	0.069800685652862	0.0217279157309385	0.0391300610048056	1081
HIST1H3I	0.0697940979596388	0.0217403600810387	0.0391483711880933	1081
C2orf54	0.0697932461838095	0.0217419695632856	0.0391483711880933	1081
SNX15	0.0697877390464844	0.0217523781337395	0.0391566131269934	1081
IWS1	0.069772997957445	0.021780260321314	0.039203300944681	1081
TSNAXIP	0.0697097325530227	0.0219002773204375	0.0394052417488579	1081
RPS2P32	0.0696666275307577	0.0219823780291044	0.0395317805210059	1081
UNC93A	0.0696523403717935	0.0220096492453653	0.0395737579478654	1081
COASY	0.0696460207246904	0.022021721510457	0.0395919303728833	1081
BRF1	0.0695793543000895	0.0221494235376983	0.0398022736534336	1081
NCRNA0(	0.0695787558263145	0.0221505728433181	0.0398022736534336	1081
C2orf51	0.0695673595929124	0.0221724679907643	0.0398345106281947	1081
EPYC	0.0695603808178825	0.0221858853220317	0.0398550615134946	1081
GPR55	0.0695465885486612	0.0222124229571056	0.0398956188008999	1081
C11orf67	0.0695389789223124	0.0222270763977052	0.0399183787137177	1081
SERP1	0.0695109679523821	0.0222810879363815	0.0400046806130486	1081
CCDC104	0.069504344596559	0.0222938759247861	0.040024073656966	1081
PPP2R1A	0.0694935674196743	0.0223146974900614	0.040057884596087	1081
CSF3R	0.0694895791080721	0.0223224072096223	0.0400681540798219	1081
GC	0.0694833362812598	0.0223344797276685	0.0400862521484214	1081
ETFB	0.0694765439001679	0.0223476214252602	0.0401062658006117	1081
MED31	0.0694626305398955	0.0223745615451671	0.0401457540949559	1081
GPR108	0.0694328869470898	0.0224322479647708	0.0402402168522091	1081
TAGLN2	0.0694100229013586	0.0224766795303079	0.040316330615088	1081
BAIAP2L1	0.0694078610867551	0.0224808845223213	0.0403202830041099	1081
C20orf152	0.069396996869773	0.022502027085049	0.0403546101333849	1081
CCDC130	0.0693610645873624	0.0225720769330202	0.0404694277158858	1081
TNFRSF1	0.0693518201749193	0.022590129461801	0.0404945860480496	1081

UBE2N	0.069309172806561	0.0226735737697484	0.040640550190549	1081
STRA8	0.0692896246572582	0.0227119112087776	0.0407056451335254	1081
C14orf70	0.0692757494724194	0.022739157064242	0.0407472263884678	1081
GLO1	0.0692746691438794	0.0227412796291714	0.0407474053303974	1081
LOC11323	0.0692513832901445	0.0227870720891661	0.0408221936181638	1081
PLK3	0.0692495028660184	0.0227907734988795	0.0408251940365399	1081
TNFSF13	0.0692450041554621	0.0227996308366626	0.0408374289325658	1081
PTCHD2	0.0692427768463796	0.0228040172049204	0.0408416542134711	1081
CD248	0.0692000200289219	0.022888362717313	0.0409817853471043	1081
RBM4B	0.0691956411396819	0.0228970161222431	0.0409936357787582	1081
FUS	0.0691638763243242	0.0229598736270834	0.0410952158345265	1081
MMP24	0.0691298287556365	0.0230274144821843	0.0412051221125958	1081
CDH1	0.0691208295309668	0.0230452951942358	0.0412334551636806	1081
APBA2	0.0690792354417195	0.0231280957294651	0.0413742552588646	1081
CACNG5	0.0690741832011965	0.0231381706750263	0.0413886031120941	1081
PLIN2	0.0690225196554456	0.0232414143722799	0.0415511441719541	1081
GSTK1	0.0690064098369215	0.0232736895898097	0.0416045039201191	1081
WNT6	0.068977861742623	0.0233309797212292	0.041692769137245	1081
GRIN3B	0.0689620489242995	0.0233627653642971	0.0417421648127507	1081
CEACAM	0.0689089235424434	0.0234698287538138	0.041922300074976	1081
DNAJC4	0.0689003600897857	0.0234871263860362	0.0419457589580407	1081
FAM151A	0.0688924234053671	0.0235031678505138	0.0419706866721568	1081
LOC1003(	0.0688849650332212	0.0235182512132163	0.0419938991925405	1081
RAC3	0.0688628777625175	0.0235629683962055	0.0420697696163337	1081
C14orf53	0.0688587022674277	0.023571430246192	0.0420754813783666	1081
SEMA3B	0.0688582756268085	0.0235722950030403	0.0420754813783666	1081
TBPL2	0.0688471748322017	0.023594804842515	0.0421038294341376	1081
MBD3	0.0688159116580151	0.0236582994401279	0.0422028196423781	1081
GAMT	0.0688029317082219	0.0236847048023084	0.0422461818879495	1081
PGM1	0.0687976407670595	0.0236954755892785	0.0422616516818075	1081
HPN	0.0687867227173748	0.0237177149146041	0.0422912897275337	1081
FGG	0.0687663197030218	0.0237593229432583	0.042354373165581	1081
COPB2	0.0687659497416357	0.0237600779917712	0.042354373165581	1081

SEC23B	0.0687597061402584	0.0237728235978754	0.0423695949198619	1081
TFR2	0.068755020232925	0.0237823932380698	0.0423829009006472	1081
IRAK2	0.0687256335983431	0.0238424832454955	0.0424712021381649	1081
GKN2	0.0687214245152604	0.0238511007679139	0.0424827961953692	1081
OPRM1	0.0687158998174168	0.0238624159262431	0.0424984598469232	1081
GUCA2B	0.0687134129446815	0.023867510818747	0.0424984598469232	1081
AKR1B1	0.0687130115164994	0.0238683333188429	0.0424984598469232	1081
APOC2	0.0686687627116891	0.0239591468590362	0.0426526169561049	1081
WDR45L	0.0686150108564568	0.0240698662853767	0.0428156694321336	1081
GLOD5	0.0686056307357167	0.0240892330288006	0.0428463358083338	1081
HDDC2	0.0685912541209669	0.0241189420113376	0.0428916037092511	1081
IL23R	0.0685728276294396	0.0241570663649254	0.0429556096673513	1081
MKRN3	0.0685601132463668	0.0241834027940468	0.0429986451267497	1081
URM1	0.0685575783272998	0.0241886565676538	0.0430034690327859	1081
MBD2	0.0685447369391137	0.0242152863080604	0.0430366585292397	1081
TBC1D1	0.0685441930210507	0.0242164148144466	0.0430366585292397	1081
NDUFS4	0.0685351342580926	0.0242352163836207	0.0430641645980453	1081
CLDN4	0.0685259267202846	0.0242543396849703	0.0430943440601277	1081
NCRNA0(	0.0684891119956785	0.0243309314313605	0.0432189942407863	1081
FAHD2A	0.0684675839545355	0.0243758166985904	0.0432804265977096	1081
HNRNPM	0.0684478464206821	0.0244170317700953	0.0433413609136108	1081
RAB34	0.0684038806596215	0.024509056031722	0.0434855499171252	1081
PPP2R2D	0.0683984221352581	0.02452050212657	0.0435020268903349	1081
RAB32	0.0683896325838454	0.0245389428428084	0.0435232438429631	1081
B4GALNT	0.0683564077300265	0.0246087579348108	0.0436278648769483	1081
LOC6449	0.0683418073574748	0.0246394918788071	0.0436746646633144	1081
SNX2	0.0683396234867037	0.0246440918044322	0.0436789749604334	1081
ZNF598	0.0683341917156907	0.0246555360617777	0.0436954142662543	1081
LOC2846	0.0683036172202933	0.0247200396091535	0.0437962033341776	1081
ATIC	0.0682964451790506	0.0247351917099745	0.0438173089164119	1081
FEZF1	0.068267916131429	0.0247955435150079	0.0439203573472113	1081
C2orf83	0.0682331116464913	0.0248693431314029	0.0440433333962296	1081
C19orf42	0.0681745052293692	0.0249940417625845	0.0442525023121168	1081

TRAPPC6	0.0681492229521259	0.0250480023165899	0.0443402466316084	1081
PLEK2	0.0681029048730794	0.0251471213966062	0.0445039761516851	1081
KLRB1	0.0680397991911524	0.0252827109223006	0.0447321457517781	1081
ATP10A	0.0679929919959052	0.0253836890613083	0.0449029167667769	1081
C9orf69	0.067986890612637	0.0253968773436377	0.0449223017023077	1081
HIST1H4	0.0679656934286088	0.0254427415801022	0.0449915755908671	1081
CLLU1OS	0.0679515088983211	0.0254734725277812	0.045041964721509	1081
C9orf40	0.0679497509066642	0.0254772834696875	0.0450447494731309	1081
TIGD1	0.0679359965935254	0.0255071168238025	0.0450895812930416	1081
RHO	0.0679334152429144	0.0255127191825243	0.0450955275890104	1081
C16orf86	0.0678949255838266	0.0255963802779639	0.0452354663303433	1081
ADRA2C	0.0678640061703213	0.025663758249231	0.0453438484645881	1081
ZPBP2	0.0678537182641194	0.0256862110590793	0.0453743179397714	1081
TUBB8	0.0678364885803851	0.0257238519333496	0.0454368257077885	1081
TTYH3	0.0678344640774605	0.0257282778952857	0.0454406591443566	1081
KRTAP12	0.0678257173747958	0.0257474074692609	0.0454624878138541	1081
C3orf79	0.067824404684667	0.0257502794627753	0.045463574038353	1081
SLC35D2	0.0677929740428175	0.0258191282470622	0.0455731480361927	1081
FANCB	0.067773918965147	0.0258609457275986	0.0456354854339958	1081
CRYGS	0.0677705705229015	0.0258683001180516	0.0456399462145866	1081
SH3D20	0.0677651056697524	0.0258803067914338	0.045653133127271	1081
NRG4	0.0677577249302017	0.0258965304425444	0.0456777520151526	1081
FRMD8	0.0677462576259275	0.025921754218808	0.0457142378305075	1081
CPSF3L	0.0677356480386866	0.0259451102285948	0.0457514220548881	1081
EMID2	0.0677211420495169	0.025977073204318	0.0457997671714499	1081
KCNA3	0.0677004451261888	0.0260227363425628	0.0458722456791152	1081
PTBP1	0.0676834510397946	0.0260602817905928	0.0459263736043646	1081
HSPA7	0.0676809454160162	0.0260658214798843	0.0459321180644042	1081
FABP3	0.0676543605106121	0.0261246607466038	0.0460237245946947	1081
MTFMT	0.0676331315462571	0.0261717282251108	0.0460985804926866	1081
SP7	0.0676132841565909	0.0262157987495647	0.0461721687191801	1081
RBPJL	0.0675952447730599	0.0262559101986084	0.0462306888341025	1081
TMEM37	0.0675906505919217	0.0262661340425005	0.0462446486878319	1081

C14orf147	0.0675637563411293	0.0263260531848219	0.0463379940068359	1081
LOC64869	0.0675149393064502	0.0264351166671257	0.0465096441274452	1081
KIAA1486	0.0675020433409964	0.0264639929089279	0.0465523172502927	1081
WNT4	0.0674763188391962	0.0265216756997467	0.0466456396866943	1081
SLC35D3	0.0674566598394105	0.0265658307268721	0.0467151414099894	1081
CPSF1	0.067446192993207	0.0265893656081367	0.046752445681759	1081
DDX11	0.0674292653758706	0.0266274657054803	0.046799013840246	1081
SPEM1	0.0674148079073852	0.0266600433510184	0.0468440098827968	1081
OLFM1	0.0674018297517665	0.0266893168708367	0.0468872667971576	1081
GTPBP8	0.0673558727124074	0.0267932005410031	0.0470574563991724	1081
PDK3	0.0673412218193661	0.0268263913911018	0.0471034301903378	1081
VDAC3	0.0673236407844665	0.0268662671210814	0.0471693351188935	1081
SYS1-DBN	0.0673215797350822	0.0268709451539869	0.0471734370481104	1081
CCDC24	0.0673090277070669	0.0268994500786524	0.0472127988107948	1081
DDN	0.0673086984006281	0.0269001982652261	0.0472127988107948	1081
HRH2	0.0673077502643142	0.0269023525378804	0.0472127988107948	1081
LOC28455	0.0673075792994486	0.0269027410051682	0.0472127988107948	1081
LHX9	0.0672742326866752	0.0269786038863712	0.0473418096943335	1081
UBE2Q1	0.0672668869373135	0.0269953400852815	0.0473670521747232	1081
C1GALT1	0.067228071957694	0.0270839229232574	0.0475141493687986	1081
TICAM1	0.0672270540852825	0.0270862492604261	0.0475141493687986	1081
C13orf18	0.0671910577684376	0.0271686292990485	0.0476420645220519	1081
PRSSL1	0.0671426184465346	0.0272798267470477	0.0478245679069948	1081
IGHMBP2	0.0671358610761133	0.0272953701141342	0.0478476532326169	1081
GBA	0.0670980190067193	0.0273825559026584	0.0479879589960029	1081
GCGR	0.0670822797757786	0.027418888706436	0.0480390948852977	1081
FLJ45079	0.0670719148297508	0.0274428380619833	0.04807687388109	1081
PLD6	0.0670666222459811	0.0274550741118693	0.0480899459163209	1081
RCOR2	0.067046268273823	0.027502174746292	0.0481682585866851	1081
ARHGEF1	0.0670271277277466	0.0275465309094483	0.0482417512971257	1081
SLCO2B1	0.0669820143649709	0.0276513201352591	0.048404226983385	1081
PRRT3	0.0669630938770707	0.0276953706858633	0.0484771259420206	1081
PCP2	0.0669572440345722	0.0277090024744134	0.0484967727929678	1081

OR5H15	0.066945520149985	0.0277363398382152	0.0485361853753341	1081
FLJ44082	0.0669259619463851	0.0277819966975636	0.048611858201374	1081
ZNF280A	0.0669044193753329	0.0278323607656463	0.0486872965981196	1081
BST1	0.0668789578159004	0.0278919883259472	0.0487788961564552	1081
RPS19	0.0668703828610034	0.0279120944624761	0.048801348979915	1081
TRAP1	0.0668539080494605	0.027950758815691	0.0488604682698799	1081
TRMT1	0.0667984532787821	0.0280812433130065	0.0490800491532636	1081
GPAA1	0.0667939916426275	0.0280917642474676	0.0490927447994125	1081
KRTAP4-[illegible]	0.0667933061123228	0.0280933810894826	0.0490927447994125	1081
CKAP4	0.0667697713481557	0.028148937070772	0.0491834097961271	1081
C20orf11	0.0667632932663057	0.0281642457651586	0.0491989574808358	1081
TNFRSF2[illegible]	0.0667483849319111	0.0281995036510615	0.049252557746717	1081
SPTA1	0.0667416244492623	0.0282155045584847	0.049276232278342	1081
TEAD4	0.0667402433161584	0.0282187744255194	0.0492776708392933	1081
CES3	0.0667365378421154	0.0282275488387261	0.0492887207554942	1081
MPHOSPH[illegible]	0.0667132184285494	0.0282828221672233	0.0493809544599336	1081
C19orf30	0.0666780133211837	0.0283664442272568	0.0495140818783544	1081
KCNQ1	0.06660404224612	0.0285428396292555	0.0497960945987142	1081
FAR2	0.066590079284496	0.0285762421009661	0.0498414196644123	1081
FCGR2C	0.0665623219763233	0.0286427435380788	0.0499487593987706	1081
GBGT1	0.0665598557241249	0.0286486586604611	0.0499547501700847	1081
BDKRB2	0.0664959441112908	0.0288023126832467	0.0502139843361319	1081
LOC1463[illegible]	0.0664626495510191	0.0288826386179817	0.0503322452386888	1081
ADAM6	0.0664577842920641	0.0288943925782053	0.0503483728473268	1081
VSX1	0.0664564212736169	0.0288976862264898	0.0503497568787958	1081
SPANXE	0.0663904758535693	0.029057425217599	0.0506149443154804	1081
TTC39B	0.0663600631839131	0.0291313488891102	0.0507275057647343	1081
FOXD1	0.0663539625303369	0.0291461971130655	0.0507432495758971	1081
GCAT	0.0663018427704877	0.0292733154056787	0.0509469493604662	1081
REXO1L1	0.0662972906728275	0.02928444040013	0.0509619084191964	1081
AXL	0.0662891441890904	0.029304358879757	0.0509877621033602	1081
AGBL1	0.0662643212116134	0.0293651238443782	0.0510846649261657	1081
FAM193B	0.0662270120051558	0.0294566578594222	0.051230628729868	1081

NARFL	0.0662234819662069	0.029465331098098	0.0512412892758274	1081
STAMBP	0.0661931033818445	0.0295400614141492	0.0513668137408748	1081
FANCE	0.0661514817769599	0.0296427134621929	0.0515337249557458	1081
GPR150	0.0661361628558669	0.0296805718168891	0.0515933315429486	1081
CD1A	0.0661294951538501	0.0296970629804935	0.051617543894913	1081
CPXCR1	0.0661187330730092	0.0297236973311702	0.0516593807899598	1081
SCRIB	0.0661157090348365	0.0297311850105928	0.0516679366837813	1081
ILF2	0.0661063373618235	0.0297544000598524	0.0517038203403543	1081
HCN4	0.0660859273531874	0.029805012598894	0.0517783700245533	1081
ACTR3	0.0660217777809876	0.0299645716266834	0.0520420980618619	1081
GNG4	0.0660176318630387	0.0299749089330566	0.0520552524703534	1081
A2ML1	0.0659916181660467	0.0300398405854921	0.0521593336144404	1081
PHF1	0.0659883074088665	0.0300481130755035	0.0521692008882202	1081
ZNF804A	0.0659763945282882	0.0300778956117109	0.0522164087468042	1081
SLC26A9	0.0659255273471155	0.030205350210139	0.0524241216492849	1081
ZNF133	0.0659159375449412	0.0302294306068834	0.0524613955526936	1081
CRYAA	0.0659147683886942	0.0302323675336851	0.0524619729490986	1081
TMEM86/	0.065886274469766	0.0303040201452987	0.0525772531071434	1081
PHGDH	0.0658823904198262	0.0303137984982995	0.052589689179146	1081
NDRG4	0.0658627531885642	0.0303632779815784	0.0526664572876611	1081
CDX2	0.0658361063680434	0.0304305301556794	0.0527694783495017	1081
SLC10A3	0.0658294145274783	0.0304474393165591	0.0527942558987849	1081
OR2W5	0.0658278979697551	0.0304512725241253	0.0527963581201914	1081
GPN1	0.0658227126974618	0.0304643817963846	0.0528145414189474	1081
NANOS2	0.0658111586974431	0.0304936097347835	0.0528561149636219	1081
EML1	0.0658093473126923	0.0304981941413703	0.0528595131186358	1081
GTF2H4	0.0657802202770169	0.0305719925425829	0.0529737476148574	1081
CCDC57	0.065754050393794	0.0306384290548498	0.05308000367632	1081
UPK3A	0.0657529523596867	0.030641219297688	0.05308000367632	1081
P2RX6P	0.065709939350038	0.0307506923654456	0.0532513279207342	1081
FAM86C	0.0656990723347927	0.0307784031804777	0.0532901531646518	1081
TM9SF4	0.0656955497055167	0.0307873904551641	0.0532992259804506	1081
WWP2	0.0656630909109728	0.0308703084311127	0.0534263198749798	1081

C19orf12	0.0656484697682124	0.0309077215337031	0.0534864745551455	1081
MRPS6	0.065640905256942	0.0309270931282844	0.0535154003667143	1081
LCE3E	0.0656353103715417	0.0309414274972784	0.0535356056800372	1081
TMEM143	0.0656272626078414	0.0309620562476898	0.0535666972784018	1081
LOC72898	0.0655561699999671	0.0311447998235915	0.0538504842885804	1081
MAGEA1	0.0655450251217743	0.0311735313833024	0.053895536364283	1081
RFESD	0.0655260712914399	0.0312224466287799	0.0539569518175148	1081
STMN3	0.0655122044709314	0.0312582751816444	0.0540096023273202	1081
VPS72	0.0655071340270255	0.0312713847908419	0.0540229865887583	1081
OSBP2	0.0654718407413855	0.031362766001077	0.0541622735612256	1081
WASH7P	0.0654451185689189	0.0314321070206677	0.0542727175736137	1081
FXN	0.0654112718194218	0.0315201239995191	0.0544033618786763	1081
NCRNA00	0.0654108305995472	0.0315212727648503	0.0544033618786763	1081
CACNG2	0.0653979008555934	0.0315549527274954	0.0544568253829688	1081
DAPK3	0.0653723816565037	0.0316215167108575	0.0545662581765625	1081
NR1I3	0.0653653139663424	0.0316399732428961	0.0545895221803821	1081
FAM69A	0.0653517150020834	0.031675511466511	0.054646157274374	1081
TLR4	0.0653340784046597	0.0317216521610436	0.0547163869153372	1081
TNFAIP8	0.0653071860145992	0.0317921185522568	0.0548279413704702	1081
RTP3	0.0652755358171393	0.031875223526672	0.0549530490325039	1081
SUSD2	0.0652744702735476	0.0318780245928955	0.0549531752031389	1081
PLEKHH3	0.0652091688469339	0.0320500895131329	0.0552214380124924	1081
DEFB103	0.0652024467918008	0.0320678467244238	0.0552425835696526	1081
MERTK	0.0651913834065817	0.0320970904763374	0.0552835061256684	1081
C2orf50	0.0651837477428825	0.0321172870711837	0.0553135631400356	1081
DPH2	0.065168440001826	0.0321578093369193	0.0553738840150646	1081
C19orf48	0.0651285344714595	0.0322636520095459	0.0555324051377566	1081
CCDC114	0.0651248797162771	0.0322733605131596	0.0555443697170098	1081
LINGO3	0.0650915713798215	0.0323619561488432	0.0556873329305924	1081
RNF112	0.0650842085909474	0.0323815682211054	0.0557163214737075	1081
ATPAF1	0.0650825649464125	0.0323859477430592	0.0557190978976794	1081
TDRG1	0.0650683964382608	0.0324237210171525	0.0557793219377059	1081
ARPM1	0.0650593270123644	0.0324479199060298	0.0558161853391658	1081

GGCT	0.0650538557157799	0.0324625257974178	0.0558365421950974	1081
TMPRSS9	0.0650280562500247	0.0325314745326385	0.0559408070388394	1081
FAM65B	0.0650157314165942	0.0325644566778121	0.0559831865313641	1081
C17orf54	0.0649672969732059	0.0326943479431188	0.056172932990544	1081
GAK	0.0649653331689273	0.0326996237974427	0.0561772063277744	1081
SPIN2B	0.0649337852671502	0.0327844785936314	0.0563181820814109	1081
FAM83H	0.0649298625559879	0.032795042709842	0.0563315258688409	1081
ABLIM2	0.0649255645190299	0.0328066209444369	0.0563418055350111	1081
FTHL17	0.0649136968571184	0.0328386087070854	0.0563919335439635	1081
OAF	0.0649117191222001	0.0328439420297435	0.0563962847075675	1081
CCRN4L	0.0648834648949228	0.0329202156052306	0.0565079876761563	1081
ANXA2	0.0648659156471708	0.0329676667984565	0.0565846168232858	1081
PRAMEF1	0.0648543872151857	0.0329988701932409	0.056633348103837	1081
FSHB	0.0648354042083654	0.0330503055009368	0.0567035014776771	1081
CELA2B	0.0648145209195009	0.0331069688546513	0.0567924626096289	1081
PVRL2	0.0647940311550959	0.0331626451234929	0.0568756586082722	1081
TMEM14I	0.064788209513499	0.0331784786992064	0.0568923459715716	1081
B9D2	0.0647761816585754	0.0332112122647102	0.0569298733043385	1081
AQP6	0.0647303379672728	0.0333362281279056	0.0571247291286711	1081
GATAD2A	0.064706833177462	0.0334004815315834	0.0572163523483261	1081
MOB2	0.0647066226499028	0.0334010575138404	0.0572163523483261	1081
FAM183A	0.0646726389136415	0.0334941449171292	0.0573514279095255	1081
UCN	0.0646262500835508	0.0336215697133493	0.0575549389764975	1081
LOC64284	0.0646044913992387	0.0336814808340095	0.0576477002040035	1081
EIF2B5	0.0645807203045322	0.0337470371867059	0.0577500903946814	1081
OR6K2	0.0645437540602542	0.0338491998287227	0.0579199974984811	1081
GRWD1	0.0645201043016302	0.0339146984712322	0.0580271446155065	1081
SFT2D1	0.0645136239001504	0.0339326649940629	0.0580529542509892	1081
AP1G2	0.0645090783494325	0.0339452721080553	0.0580695912548212	1081
NPW	0.0644848373587755	0.03401257219829	0.0581748401201011	1081
FBXO33	0.0644708127238679	0.034051560680918	0.0582316375141845	1081
FAM27C	0.0644687772675317	0.0340572224182447	0.0582363759965657	1081
FES	0.0643941038194932	0.0342654870680012	0.0585676427104094	1081

CECR5	0.0643100034657807	0.0345013437746892	0.0589437004196784	1081
CA7	0.0643090811928452	0.0345039379173952	0.0589437004196784	1081
BLNK	0.0643039425787029	0.0345183947096496	0.0589599000700264	1081
FGF21	0.0642833847462405	0.0345762830770546	0.0590391749811455	1081
RAB11FIF	0.0642772576377133	0.0345935522838236	0.059063235084128	1081
CAPSL	0.0642427665167248	0.0346909025755072	0.0592177156894673	1081
COL22A1	0.0642420770844688	0.0346928508596224	0.0592177156894673	1081
NGFRAP1	0.0642179539601356	0.0347610797932358	0.0593123090096269	1081
SNORA28	0.0642062287536436	0.034794284231837	0.0593556487298126	1081
CENPQ	0.0641852571558779	0.0348537408445537	0.0594520414321367	1081
LOC34009	0.0641632535922657	0.0349162162998837	0.0595535667903782	1081
A1BG	0.0641498493757907	0.0349543221234615	0.0596084668735404	1081
FGF12	0.0641471903438636	0.0349618854953637	0.0596163182075589	1081
AMDHD1	0.064137454197806	0.0349895909770443	0.0596585112342411	1081
SLC5A10	0.0641327324607022	0.0350030340385679	0.0596763812378936	1081
NR4A3	0.0641223057284935	0.0350327351531646	0.0597219640907591	1081
CHRNB1	0.0641183128287516	0.0350441148335392	0.0597363084550386	1081
C15orf2	0.0641105730983717	0.0350661818769874	0.0597688665633227	1081
CALM2	0.0640788948235352	0.0351566245306825	0.0599128839498011	1081
NRGN	0.0640709747303652	0.0351792677370192	0.0599368199943033	1081
RIMS4	0.064070859974558	0.0351795959102826	0.0599368199943033	1081
GAGE12D	0.0640455754124594	0.0352519671680871	0.060049964366744	1081
APEX1	0.0640434014533242	0.0352581955470344	0.060055495839605	1081
NUDT3	0.063959913846375	0.0354980976273019	0.0604334637625283	1081
PLXND1	0.0639261112909435	0.0355956242398423	0.0605829547449715	1081
SNUPN	0.0638798896560644	0.0357293515799755	0.0607911982752202	1081
RXRG	0.0638700700804249	0.0357578163882416	0.0608344912711027	1081
CDCP2	0.0638494752915863	0.0358175789290558	0.0609155869587024	1081
KRTAP1-3	0.0638333489440513	0.0358644341834321	0.0609901254959683	1081
PPM1J	0.0638074470943091	0.0359398015003777	0.0610976625506421	1081
C6orf126	0.0636960905255183	0.036265356830273	0.0615991242281492	1081
CD200R1L	0.0636528515232449	0.0363924425828492	0.0617897129483383	1081
FAM153A	0.0636526965709091	0.0363928986910913	0.0617897129483383	1081

TBL3	0.0636271824828016	0.0364680667819427	0.0619069019400216	1081
RAC1	0.0636089640935932	0.0365218215313871	0.0619793378961839	1081
LOC14841	0.0636085500642384	0.036523043939717	0.0619793378961839	1081
SEPHS2	0.0635526773569408	0.0366883259647271	0.0622186467688798	1081
FBXO24	0.063552526983846	0.0366887716538855	0.0622186467688798	1081
TMEM194	0.0635457583797002	0.0367088378152581	0.0622474358407723	1081
C22orf34	0.0635372073028832	0.0367342016308096	0.0622842809257493	1081
GPR25	0.0635104179741455	0.0368137594511498	0.0623990899573759	1081
KRTAP19	0.0635080228225369	0.0368208796034291	0.0624059077579572	1081
ZNF581	0.0634875839860266	0.0368816865170212	0.0624984501081251	1081
IL20	0.0634847956725166	0.0368899885538577	0.0625072604438947	1081
FUT5	0.0634423236764755	0.0370166429990696	0.062700771312222	1081
TCEAL6	0.0633729887027939	0.0372241992099772	0.0630099565654139	1081
PFN2	0.0633648818618021	0.0372485317467724	0.0630458470877778	1081
DND1	0.0633511092711474	0.0372899009267713	0.0631091646586554	1081
RASGRP4	0.0633503421474458	0.0372922063090908	0.0631091646586554	1081
C16orf74	0.0633367133404382	0.0373331841944676	0.0631732041661109	1081
PTGES3	0.0632811334130178	0.0375006935125914	0.0634406676865262	1081
PCMT1	0.0632489072480482	0.0375981104140464	0.0635841113407459	1081
NPIPL3	0.0632457843817826	0.0376075619859923	0.0635947566698409	1081
MORN5	0.0632364004610943	0.0376359752233792	0.0636374618830765	1081
LEF1	0.0632262578381032	0.0376667062175654	0.063684078619627	1081
GPR120	0.0632065862625257	0.0377263696307071	0.0637742482345049	1081
MFSD11	0.0632024675093097	0.0377388718696818	0.0637900296874258	1081
FIP1L1	0.0631431233089822	0.0379193988571387	0.0640790445413187	1081
C17orf73	0.0631406233114016	0.0379270200041435	0.0640801126134589	1081
FSCB	0.0631397856969868	0.0379295737305974	0.0640801126134589	1081
ST3GAL4	0.0631355328174794	0.0379425422017449	0.0640933273983873	1081
GHDC	0.0631169801310703	0.0379991596885058	0.064170766361384	1081
HSD11B2	0.0630831163412767	0.0381026872923338	0.0643348114578882	1081
KIR3DP1	0.0630666539271534	0.0381531022670994	0.064398344798552	1081
LPO	0.0630632162391981	0.0381636370882348	0.0644035931509615	1081
RRAGD	0.0630625096695369	0.0381658026823618	0.0644035931509615	1081

PRKCDBI	0.0630499883201436	0.0382041971881862	0.0644521859438916	1081
FAM71B	0.0630362760755917	0.0382462809802024	0.0645015764207767	1081
WRNIP1	0.0630350637669309	0.0382500035291757	0.0645024544697175	1081
EAPP	0.0630309637983213	0.0382625952871963	0.0645182876086523	1081
LOC44056	0.0630098461065221	0.0383275073634745	0.0646169249173315	1081
PEX10	0.0629801983035198	0.0384187972425974	0.0647654117533159	1081
SMC4	0.0629774247242792	0.0384273469278422	0.0647744041386814	1081
CAPN10	0.0629630368574075	0.0384717241104845	0.0648329330940029	1081
C19orf36	0.062943055513289	0.0385334256666951	0.064926986252397	1081
SNCB	0.0629428757920377	0.0385339810192931	0.064926986252397	1081
BRP44L	0.0628511859532013	0.0388181969066343	0.0653675981513123	1081
ANKRD9	0.0628332461259708	0.0388740134811487	0.0654287744655142	1081
TRIM67	0.0628217044434591	0.0389099593499165	0.0654838037680926	1081
PAK4	0.0627801551910889	0.0390395955170774	0.0656635761619374	1081
ATP6V1B	0.0627579524381636	0.039109019498281	0.0657474086449863	1081
CETN4P	0.062740163816979	0.0391647168122211	0.0658270511803188	1081
ZNF628	0.0627396914163801	0.0391661968452371	0.0658270511803188	1081
PTPN18	0.0627250403855798	0.0392121221372126	0.0658987403398804	1081
CD1D	0.0627162716741429	0.0392396304802848	0.0659394691379161	1081
GJA8	0.0626905213334008	0.0393205065312088	0.0660588444726212	1081
CCR4	0.0626868286102952	0.0393321161263267	0.066068295028604	1081
DENR	0.0626866457484246	0.0393326911034027	0.066068295028604	1081
BTG3	0.0626515769867869	0.0394430904869104	0.0662316460664175	1081
ALG1	0.0626063139991123	0.0395859703782617	0.0664549477725069	1081
OTOP2	0.0625972055374928	0.039614775660558	0.0664977631598984	1081
C6orf227	0.0625821559598391	0.0396624084996313	0.0665721729066049	1081
LOC72946	0.0625560957548819	0.0397450054533332	0.0666941386803329	1081
SOCS3	0.062547338057234	0.0397727953882635	0.0667352126517216	1081
MUC17	0.0625215455586845	0.0398547358444718	0.0668671319719234	1081
ZNF534	0.0625011199118714	0.0399197277428489	0.0669705958843847	1081
ADCY3	0.0624875635605868	0.0399629119430056	0.0670318785255494	1081
FAM25B	0.0624810273410179	0.0399837474214924	0.0670612431775842	1081
KPRP	0.0624689343242039	0.0400223205429996	0.0671091765722263	1081

TMEM201	0.0624660473001377	0.0400315339496491	0.0671190388194491	1081
CCDC99	0.062438456206254	0.0401196765278962	0.0672556281646392	1081
SLC7A5P1	0.0624169527491338	0.0401884854599835	0.0673541630275681	1081
FAM189B	0.0623749056222295	0.0403233209307626	0.0675576596931314	1081
ISCU	0.0623552355439169	0.0403865297574013	0.0676523068321012	1081
METTL2A	0.0622747339907504	0.0406460925336153	0.0680474972234422	1081
FAM98A	0.0622333781533192	0.0407799851413652	0.068254636147624	1081
TM2D3	0.0622240057586104	0.0408103807982812	0.0682998356053315	1081
ZNF419	0.0622028604452789	0.0408790276959211	0.0683976754928846	1081
SPACA3	0.0621962233473945	0.0409005947455905	0.0684280774977095	1081
MED26	0.0621676797707592	0.0409934560165401	0.0685685654532553	1081
DDO	0.0621672738238356	0.0409947779756271	0.0685685654532553	1081
IL9	0.0621622962763556	0.0410109902052148	0.0685899873544253	1081
FGF23	0.0621594909017571	0.0410201299018156	0.0685995780235825	1081
LEPROTL	0.0621556607175849	0.0410326111325566	0.0686147548157511	1081
LOC10013	0.0621347126179024	0.0411009305204591	0.0687061858214793	1081
HS3ST3A	0.0621190659601988	0.0411520227372438	0.0687801790460281	1081
CXorf61	0.0620978732128433	0.0412213106834572	0.0688731279520814	1081
CPA5	0.062096752484561	0.0412249775581068	0.0688735422879468	1081
SLC34A1	0.0620758615097921	0.0412933805879118	0.0689821006670109	1081
C4orf52	0.0620517790670624	0.0413723524901353	0.0691025653688563	1081
LOC34802	0.062033774724411	0.0414314763054765	0.0691783746517882	1081
TRIML2	0.0619958099368121	0.0415563817240053	0.0693696809603982	1081
FAM104B	0.0619912140298951	0.0415715239939911	0.0693892079591475	1081
PGS1	0.0619778050733006	0.0416157295424543	0.0694457315799157	1081
MNAT1	0.0619611504966108	0.0416706903159803	0.0695201703035547	1081
SLC1A3	0.0619415270676579	0.0417355271582147	0.0696173765380295	1081
AMPD2	0.0619318886892383	0.0417674040856013	0.0696642122292133	1081
PRPS1	0.0619053613963381	0.0418552438675711	0.0697991628900844	1081
SOLH	0.0618982243931906	0.0418789032529504	0.0698328371848924	1081
OR4N3P	0.0618637021176864	0.0419935052973929	0.0700123449653215	1081
KRTAP5-1	0.0618498492227086	0.042039566584287	0.0700751061283266	1081
LMNB2	0.0618481268708672	0.0420452964382738	0.0700754940637897	1081

ANG	0.0617782405183087	0.0422783489621996	0.0704231304649836	1081
MOBP	0.0617744190790444	0.0422911238587691	0.0704385853770074	1081
PAX4	0.0617654464261374	0.0423211318444556	0.0704827381784522	1081
PTK6	0.061700560930685	0.0425386695633358	0.0708274649436647	1081
GOT2	0.0616405397163747	0.0427407391637298	0.0711227647822019	1081
PDK2	0.0616313505122583	0.042771747296973	0.0711684851158437	1081
TMEM165	0.061599789755212	0.0428783908801134	0.0713164782265427	1081
PLIN3	0.0615967837220908	0.042888559943378	0.0713275026879675	1081
RBM11	0.0615814668662671	0.0429404067208386	0.071401939198621	1081
LRRC3	0.061571710957061	0.0429734575503201	0.0714333115233268	1081
KRTAP5-6	0.0615437403931836	0.0430683345954949	0.0715792096713344	1081
FCRL4	0.06150694358695	0.0431934197594197	0.0717752570365017	1081
CSNK2A2	0.0614800402310223	0.0432850675302489	0.0719038249688188	1081
ELOVL6	0.0614352426093972	0.0434380371717206	0.0721341405345241	1081
LRRC47	0.0614239546476558	0.0434766538303284	0.0721923171292421	1081
DAPK1	0.0613733124503675	0.0436502598209513	0.0724686405219271	1081
OR51I1	0.0613615079483082	0.0436908106312864	0.0725299860047189	1081
FOXJ1	0.0613049223506715	0.0438856348365851	0.0728414034590925	1081
ISYNA1	0.0612820377871624	0.0439646341502417	0.0729665146611154	1081
MST4	0.0612531179532092	0.0440646391546633	0.0731264647640408	1081
SLC8A1	0.0612403603038045	0.0441088161205004	0.0731937480022636	1081
HAND1	0.0612161857609383	0.0441926297081313	0.0733207482680206	1081
NUDT19	0.0612075185472071	0.044222711770931	0.0733585744915518	1081
NRADDP	0.0612044527739701	0.0442333565516341	0.0733701908384249	1081
PAX5	0.0612004397609258	0.0442472935409272	0.0733872656332931	1081
PLA2G2F	0.061188005752833	0.044290499716754	0.0734468321364842	1081
SNCG	0.0611784672720884	0.0443236684966037	0.0734897367789367	1081
SAMD7	0.0611454286929201	0.0444387175679696	0.073668364500226	1081
PSIP1	0.0611368523570138	0.0444686237423595	0.0737058108680131	1081
AOC2	0.0611329355729485	0.0444822874324802	0.073716327766314	1081
NKD1	0.0611108385258452	0.044559439209146	0.0738320367550786	1081
PPP3R2	0.0610966960292123	0.0446088768394379	0.0738957177806493	1081
KIRREL2	0.0610898135529953	0.0446329524784203	0.0739195252211457	1081

WDR49	0.0610894407553011	0.0446342568771415	0.0739195252211457	1081
NIPSNAP	0.0610882962511305	0.0446382616352109	0.0739200806116995	1081
LRRC69	0.0610611381689597	0.044733379888203	0.0740556006171284	1081
PNMA3	0.0610512549662189	0.0447680370831139	0.0740984394905373	1081
DENND1A	0.0610384723840637	0.044812895002672	0.0741665929862659	1081
ALDOC	0.0610242014563688	0.0448630206935764	0.07423125682727	1081
GABARA	0.0609899051817087	0.0449836772249405	0.0744125618078852	1081
ATG4A	0.0609754325077581	0.0450346749592353	0.0744846902343018	1081
ELAVL1	0.0609629019186336	0.0450788686326377	0.0745516631314741	1081
C2orf81	0.0609530936292215	0.0451134865999161	0.0746027899815555	1081
C3orf45	0.0609493431153162	0.0451267298040045	0.0746185645490988	1081
ACOT9	0.060926433771901	0.0452076946255077	0.0747463073071941	1081
MST1R	0.0609140494734924	0.0452515133661152	0.0748064776596415	1081
DKFZp434	0.0608682080477722	0.0454140230184284	0.075062807163295	1081
B4GALT2	0.0608650958284987	0.0454250737412326	0.0750649517961128	1081
YBX2	0.0608566577412461	0.0454550466941692	0.075104205426105	1081
RADIL	0.0608550845485457	0.0454606366767794	0.075104205426105	1081
LRRC46	0.0608454715484562	0.0454948068574045	0.0751531680313582	1081
MAMDC4	0.0608315739471878	0.0455442452075812	0.0752286667519452	1081
ADAMTS	0.0608015659189298	0.0456511478712517	0.0753743442231263	1081
PDE6H	0.0607756597203573	0.0457436076351238	0.0755146255682675	1081
LOC28307	0.0607741832254044	0.045748882018607	0.0755171442367123	1081
EXOSC2	0.0607360254183022	0.045885368160688	0.0757114212136178	1081
SPRR2E	0.060692356117601	0.0460419884340946	0.0759636246523206	1081
HGD	0.0606848644674859	0.0460689024243112	0.0760018048757472	1081
OTOP3	0.0606564423410031	0.0461711301623349	0.0761584620703136	1081
KAT5	0.0606485855655064	0.0461994227757443	0.076191241841876	1081
ZNF205	0.0606478394864495	0.0462021101988202	0.076191241841876	1081
SNN	0.0606317115685539	0.0462602361019173	0.0762612484675224	1081
GJB6	0.0606182365877371	0.0463088478095351	0.0763351394421966	1081
MNX1	0.0606111292681276	0.0463345051378419	0.0763649354497116	1081
GPR83	0.0605931456369368	0.0463994791892796	0.0764532560337018	1081
MGC1612	0.0605715422841641	0.0464776323889495	0.0765695047820892	1081

VDAC1	0.0605350761180984	0.0466098049592431	0.0767746950861776	1081
CALML4	0.060483143406892	0.0467985816231531	0.0770667397039498	1081
UNG	0.0604603970847346	0.0468814671527139	0.0771906134688534	1081
UGT1A4	0.0604434362566689	0.0469433511118806	0.0772735584367408	1081
GAGE8	0.0604342436207326	0.0469769204018088	0.0773224986922492	1081
PNPLA5	0.0604210158879431	0.047025260279772	0.0773957408771248	1081
C2orf62	0.0604074503197753	0.0470748781495421	0.0774647459828889	1081
LOC10013	0.060399581260265	0.047103680430343	0.0775058108526716	1081
LOC72937	0.0603943425268972	0.0471228634149198	0.0775310424284188	1081
DNAJB13	0.0603898366763785	0.0471393680072523	0.0775518634958021	1081
TMCO7	0.0603707913206816	0.0472091833614416	0.0776603788025674	1081
IFT122	0.060339634999213	0.0473235816194534	0.0778167946880979	1081
ZNF34	0.0602931383447511	0.0474947386492695	0.0780702319962967	1081
FAM92A1	0.0602925020997108	0.0474970843061113	0.0780702319962967	1081
CHRNA5	0.0602906247865191	0.0475040059997153	0.0780752387096569	1081
LYZL1	0.0602629987583056	0.0476059617215797	0.0782318736857049	1081
SPANXA2	0.0602623104688271	0.0476085042439889	0.0782318736857049	1081
IRX5	0.0602616459366478	0.0476109591157479	0.0782318736857049	1081
AGXT2L2	0.0602544060238875	0.0476377112190053	0.0782633961625346	1081
PRR14	0.0602445077438126	0.0476743066377343	0.0783167990880826	1081
C1orf128	0.0602123569745924	0.0477933356818031	0.0784867324781872	1081
SLFNL1	0.0602063369278501	0.047815650888282	0.0785041798813725	1081
PSPH	0.060204332145393	0.0478230841890027	0.0785099854117398	1081
IFNA13	0.06016901923466	0.0479541759211491	0.0787123664899819	1081
KHDC1L	0.0601474569590809	0.0480343695029076	0.0788311506017168	1081
MRPL9	0.0601374330957653	0.0480716881659594	0.078879543708011	1081
MSI1	0.0601263672076175	0.0481129145176413	0.0789407609511226	1081
ZNF784	0.0601160391235411	0.0481514189138389	0.078997502566926	1081
GPR119	0.0601046866804042	0.0481937720724077	0.0790605487295321	1081
SYCN	0.0600986069820957	0.0482164667711476	0.0790855021405572	1081
ZSWIM1	0.0600985084071298	0.0482168348121282	0.0790855021405572	1081
SLBP	0.060095450722833	0.0482282521988019	0.079097788858167	1081
FAM166A	0.0600722309712255	0.0483150287301747	0.0792272082840349	1081

TUFM	0.0600696204069166	0.0483247930797086	0.079236770030175	1081
GPR155	0.0600430428972354	0.0484242957494499	0.079393459581075	1081
CFLAR	0.0600323313103662	0.0484644470647435	0.0794528227636115	1081
PATL1	0.0599925263924917	0.0486138965722813	0.0796783781993822	1081
SAR1A	0.0599711361784348	0.0486943666790423	0.0798037761755049	1081
OR56A1	0.0599661258476992	0.0487132317070854	0.0798281991978552	1081
IFNA2	0.0599357723460001	0.0488276505613943	0.0800017705366315	1081
RPS6KA1	0.0599126763608666	0.0489148627512016	0.0801260294456786	1081
CCT3	0.0598617746906855	0.0491075323927894	0.0803958748519092	1081
TUBG2	0.0598563048371465	0.049128274314299	0.080416761685346	1081
C11orf75	0.0598508115609236	0.0491491124443865	0.0804443345947487	1081
NCRNA0(	0.059832015480299	0.0492204693173479	0.0805545823704585	1081
BRP44	0.0598267083573313	0.049240632827788	0.0805744901564283	1081
TMEM14:	0.0597166798315294	0.0496602292410525	0.0812083219466679	1081
TPCN2	0.0596854825133126	0.0497797444463701	0.0813971551844258	1081
OR5D14	0.0596274272650189	0.0500027921021106	0.0817435066044222	1081
USP10	0.059626128297637	0.0500077922756465	0.0817435066044222	1081
KIAA019:	0.0596069849374223	0.050081530228965	0.0818573976845842	1081
MED7	0.0596027631117264	0.0500978044452401	0.0818707123022115	1081
ZNF408	0.0595967367266918	0.050121042513383	0.0819020442433567	1081
LOC1491:	0.059578732104844	0.0501905230685735	0.0819956278660623	1081
S100A7	0.0595650879018156	0.0502432302752251	0.0820551174620519	1081
CRHBP	0.0595625127994959	0.0502531830244811	0.082064718873879	1081
GUSB	0.0595509026193351	0.0502980767691361	0.0821313736333183	1081
YWHAZ	0.059514172733637	0.0504403234280773	0.0823369512566742	1081
LOC4010:	0.0595026020997528	0.0504852035793023	0.0823901835794754	1081
ZDHHC22	0.0594862545626225	0.050548669323028	0.0824870752136411	1081
TOX	0.0594847409319322	0.0505545490387988	0.0824899878816298	1081
MAGEC2	0.0594359084081566	0.0507445467846208	0.0827731898766142	1081
OSBPL7	0.0593792550415287	0.0509657220022737	0.0831137755796813	1081
ATP6V0D	0.0593399843115789	0.0511195079031624	0.0833397041840245	1081
SLC25A4:	0.0593385971063595	0.0511249473470427	0.0833397041840245	1081
NDRG3	0.0593230009036077	0.0511861357580969	0.0834259469940827	1081

TRPM5	0.0593192787251667	0.0512007479867004	0.0834430117459816	1081
PECAM1	0.0592447536590556	0.0514940472415366	0.0838870759890632	1081
PRR22	0.0592258265665209	0.0515687595058238	0.0839905133347982	1081
ZNF358	0.0592254998154777	0.0515700501086185	0.0839905133347982	1081
FSTL4	0.0592110611049769	0.0516271071517792	0.0840630515335105	1081
NR5A1	0.0592061035444604	0.051646709965537	0.0840881736244843	1081
TBC1D22.	0.0592020140637539	0.0516628849645426	0.084107711137119	1081
TPI1P3	0.0591940688205795	0.0516943226456791	0.0841476086179082	1081
HSD3B7	0.0591937091782714	0.051695746053871	0.0841476086179082	1081
PAG1	0.0591908549751716	0.05170704370619	0.084159198146659	1081
SPINK13	0.0591717730315891	0.0517826278440128	0.0842686031115306	1081
DNAH8	0.0591675083236082	0.0517995330929788	0.0842893048592938	1081
C14orf169	0.0591486121535586	0.0518744927637565	0.0844044630260783	1081
CPLX2	0.0591453414222443	0.0518874767026392	0.0844187706488989	1081
CAPN14	0.0591043497324533	0.0520504332452644	0.0846565459168296	1081
ANLN	0.0590953282754607	0.0520863540788111	0.0847081297261363	1081
APOA5	0.0590901903608268	0.0521068210219265	0.0847345745468768	1081
RAB7A	0.0590848098829723	0.0521282614184183	0.0847625979719135	1081
RRAGA	0.0590665709621571	0.052200995610181	0.0848671662147603	1081
UPB1	0.0590566235985919	0.0522406999695814	0.08491643058459	1081
C20orf141	0.0590344782675938	0.0523291824265172	0.0850412536287665	1081
OR4C13	0.0590265686678171	0.0523608158288964	0.08508579776342	1081
CYP11B1	0.058977015500727	0.052559360764402	0.0853808823964905	1081
LOC3395(	0.0589705935211111	0.0525851376878335	0.0854089815948887	1081
ACTL9	0.0589538997652636	0.052652193453188	0.0855041061760962	1081
CCDC18	0.0589191471887724	0.0527920171738918	0.0856981267520738	1081
SMARCD:	0.0588923392246401	0.0529000881061141	0.0858582238879849	1081
SEMA4F	0.0588744248281157	0.0529724092612447	0.0859617516165437	1081
TTC16	0.0588731590166117	0.052977522513502	0.0859631245295608	1081
NSMCE1	0.0588668201318064	0.0530031346677933	0.0859908309481112	1081
RPS15	0.0588656793429758	0.0530077451021347	0.0859913854954504	1081
SLC48A1	0.0588460885483898	0.0530869724338113	0.0861129768644116	1081
TEKT5	0.0588340829934469	0.0531355730264869	0.0861848726746037	1081

PLSCR2	0.0588294223769325	0.0531544500192365	0.0862085496850109	1081
LCN15	0.0588114414683442	0.0532273309082117	0.0863198020565066	1081
PIGH	0.0587893838572737	0.0533168494721845	0.0864580159878578	1081
SF3B3	0.058764006138555	0.0534199976730835	0.0866183075599056	1081
C10orf114	0.0587273832911953	0.0535691452516387	0.0868391752711242	1081
KREMEN:	0.0586916327246154	0.0537150752161864	0.0870547216597003	1081
NIF3L1	0.0586644809517361	0.0538261271510519	0.0872066375629677	1081
NDUFS2	0.0586335920799084	0.0539526966422017	0.0873932838746976	1081
ADAT3	0.0586276882357471	0.0539769163075923	0.0874157873636503	1081
DIRC3	0.0586164493933463	0.0540230470761575	0.0874834632917357	1081
TEX13B	0.0585959045239618	0.0541074601471712	0.0876026630761891	1081
NTN5	0.0585953593773456	0.0541097014969978	0.0876026630761891	1081
SCNN1D	0.0585812127859023	0.0541678917382581	0.0876677194865061	1081
C12orf5	0.0585811106555956	0.0541683120280738	0.0876677194865061	1081
USH1G	0.058555655930937	0.0542731488799197	0.0878108251695432	1081
LOC28333	0.0585471552536028	0.0543081970781805	0.0878604746337894	1081
GPR139	0.0585425381857842	0.0543272410990606	0.0878842264466856	1081
SARS	0.0585156227497863	0.054438369998284	0.0880569265790454	1081
SNHG1	0.0585046904217082	0.0544835616251749	0.0881229505370654	1081
R3HDM1	0.0585018762960581	0.054495199603258	0.0881330739834625	1081
CALM1	0.0585010612899397	0.0544985704945144	0.0881330739834625	1081
RNPS1	0.0584830282883213	0.0545732000144009	0.0882325131854019	1081
POMGNT	0.0584449158195934	0.0547312084425269	0.0884666768895695	1081
ALOX5	0.0583849322589235	0.0549806626354225	0.0888485038336745	1081
PTCD1	0.0583812190132337	0.0549961360081885	0.0888663800340865	1081
AGFG2	0.0583614572269263	0.0550785458557361	0.0889924050580529	1081
HIST1H4(	0.0583474833773534	0.0551368810976864	0.0890652289080983	1081
CCDC69	0.0583442174610591	0.0551505224065039	0.0890801213822179	1081
OR10T2	0.0583135738545383	0.0552786536768006	0.0892656094845008	1081
C10orf62	0.0582868888246035	0.0553904345367099	0.0894246116158055	1081
XCR1	0.0582640011927249	0.0554864582866093	0.0895652806236975	1081
C12orf57	0.0582491492517414	0.0555488428316931	0.0896444315344434	1081
MCOLN3	0.0582450724088512	0.0555659775319407	0.0896649004630683	1081

NFE2L1	0.0582056847370153	0.0557317479028023	0.0899179930723973	1081
LRRFIP2	0.0581959792285239	0.055772658465345	0.0899767922466866	1081
FAM134B	0.0581902532418153	0.05579680630215	0.0900085412361317	1081
LYPLAL1	0.0581866456111562	0.0558120249972716	0.0900258822619935	1081
BEYLA	0.0581798211720559	0.0558408231580257	0.0900651226996339	1081
CXorf42	0.0581359362812962	0.0560263065846793	0.0903208983793506	1081
PLK4	0.0581184321943617	0.0561004319836813	0.0904186896800752	1081
KRTAP10	0.0581166330018409	0.0561080557307882	0.090418773053176	1081
C9orf153	0.0581070318476511	0.0561487534210471	0.0904603836905785	1081
MED18	0.0580827636037128	0.0562517319036795	0.0905973088583005	1081
WFS1	0.0580508158141518	0.0563875367141009	0.0908015129580786	1081
UBXN11	0.0579977445994168	0.0566137361362335	0.0911407730458565	1081
FAM103A	0.0579973469609364	0.0566154337878761	0.0911407730458565	1081
SPHK1	0.0579727530197501	0.0567205158097168	0.0913012218127643	1081
C7orf29	0.0579506466284574	0.056815107598815	0.0914461763005854	1081
CDK3	0.0579213731115882	0.0569405687221302	0.0916334683581492	1081
CLEC12B	0.0579143586334585	0.05697066569743	0.0916630124082235	1081
CST9	0.0579141884359833	0.0569713961268191	0.0916630124082235	1081
KRT26	0.0579118615348113	0.0569813831716421	0.0916698581690408	1081
THNSL2	0.0579107576021611	0.0569861217521286	0.0916701607200057	1081
XAGE2	0.0578951501479239	0.0570531510766672	0.091756005783128	1081
PRAMEF5	0.0578756407587415	0.0571370301295644	0.0918762349904275	1081
C12orf42	0.0578556118496228	0.0572232493516877	0.0920001882034916	1081
RGS9	0.0578500838800311	0.0572470648357264	0.0920311324807045	1081
IL11RA	0.0578113307608256	0.0574142514572444	0.0922925387492969	1081
GHRLOS	0.0578098007787013	0.0574208603345316	0.0922957976094422	1081
LOC39085	0.0577979075945114	0.0574722554164546	0.0923563006831907	1081
ADAM11	0.0577913451883939	0.0575006304787321	0.092380954582825	1081
IVL	0.0577911777407735	0.0575013546544573	0.092380954582825	1081
FLJ25363	0.057776362675636	0.0575654566752318	0.0924765649698999	1081
ZNF600	0.057768260334875	0.0576005390802855	0.0925181677381877	1081
INHBC	0.0577467602656591	0.0576937184954292	0.0926495518331956	1081
IL22	0.0577401799366516	0.0577222620615487	0.0926841199768769	1081

PCYT1A	0.0577378993911732	0.0577321571439251	0.0926926202011932	1081
SFRS7	0.0576870754309265	0.0579530433661824	0.093032437533807	1081
FBXO45	0.0576776769176635	0.0579939670279795	0.0930907144046731	1081
PRAMEF1	0.0576550851372068	0.0580924358492299	0.0932413449273155	1081
GNG13	0.0576371692692324	0.0581706227768819	0.0933445273092469	1081
C3orf34	0.0576360560454625	0.058175483894453	0.0933448923181008	1081
RAPSN	0.0576137615015274	0.0582729085350498	0.0934937673175038	1081
TUBB4Q	0.0576106243677722	0.0582866283125075	0.0935083321936342	1081
SUPV3L1	0.0575579051588858	0.0585175889473866	0.0938435378278651	1081
C14orf181	0.0575576145158326	0.0585188643431939	0.0938435378278651	1081
ZC3H15	0.0575543933199327	0.0585330010940766	0.0938584072964356	1081
AP3S2	0.0575533786071629	0.0585374549154836	0.0938584072964356	1081
POMT1	0.0574784242423758	0.0588672259321477	0.0943270972321919	1081
SLC24A5	0.0574309955295457	0.0590766890149677	0.0946401511098627	1081
IL22RA2	0.0574200503192982	0.0591251148267031	0.0947101970566106	1081
RIMKLA	0.0574065841629771	0.059184739408921	0.0947981693084768	1081
OR1S2	0.0573863956343875	0.0592742222343877	0.0949264016942317	1081
IL1A	0.0573833990566093	0.0592875136969067	0.0949401401768033	1081
NUDT16P	0.057361014935088	0.0593868776826757	0.0950765835585713	1081
AP3B2	0.0573550892355782	0.05941320518166	0.0951111743789367	1081
RAP1A	0.057328184663288	0.0595328621204528	0.0952800117118076	1081
PRDM12	0.057305865047932	0.0596322791169605	0.0954284246494461	1081
ENSA	0.0573041767648132	0.0596398047325156	0.0954284246494461	1081
FLJ41941	0.0572624290133312	0.0598261480631393	0.0957037840651109	1081
FAM70B	0.0572304846963132	0.0599690586806847	0.0958867122090955	1081
RDH11	0.0572224635208912	0.0600049877181201	0.0959365458398039	1081
C9orf78	0.0571916612556643	0.0601431251813136	0.0961421406743027	1081
ZNF691	0.0571812107626667	0.0601900517916071	0.096209521014197	1081
SFTPC	0.0571052645639282	0.0605319899360548	0.0967253856795291	1081
TAAR2	0.0570141977545707	0.0609441248160737	0.097360776718462	1081
CYLC1	0.0569637567062581	0.0611733996556669	0.097672833166633	1081
DTD1	0.0569601109399366	0.061189998736415	0.0976915933226406	1081
PRRT2	0.0569349158809296	0.0613048130477095	0.0978593865963635	1081

CALML6	0.0569270074632592	0.0613408885563294	0.0979014577695496	1081
GRK7	0.0569185634822355	0.0613794264990663	0.0979552045332877	1081
IL18R1	0.0568972456891681	0.0614768090115922	0.0981028451789072	1081
SPNS3	0.056893662145058	0.0614931916526582	0.098121215512301	1081
SUSD4	0.0568779666901861	0.0615649880110982	0.098219145026088	1081
OR4E2	0.0568770482246439	0.0615691915308731	0.098219145026088	1081
SPPL2B	0.0568601085538307	0.0616467614797534	0.0983117487341381	1081
IFNAR2	0.056842174285133	0.0617289739048585	0.0984272739681315	1081
HMGCL	0.0568386287005166	0.0617452379328668	0.0984454149709222	1081
IGFBP6	0.0568152652301739	0.0618524976035637	0.0986008201490694	1081
TRPA1	0.0568131978426678	0.0618619962123422	0.0986081590202274	1081
OR2Y1	0.0567498579692347	0.0621535959487626	0.0990259562154241	1081
PCCB	0.0566754618800336	0.0624975448739089	0.0995345909941414	1081
IRGM	0.0566573703024886	0.0625814232815587	0.0996524205206704	1081
C10orf78	0.0566521912165358	0.0626054523092505	0.0996646604975121	1081
CATSPER	0.0566510852754208	0.0626105844507582	0.0996646604975121	1081
POLG2	0.0566503823062314	0.0626138467737162	0.0996646604975121	1081
FAM132A	0.0566470024680592	0.0626295338075876	0.0996817538558784	1081
POU4F1	0.0565969838082555	0.0628620679062446	0.100036050080678	1081
PDCL2	0.0565045641867961	0.0632935970316991	0.100667101863538	1081
ABTB1	0.056490396638295	0.0633599642699329	0.100764701998721	1081
C11orf74	0.0564891181792871	0.0633659559768514	0.100766275904142	1081
MCART6	0.0564781198626031	0.0634175206988829	0.100839779544345	1081
NCAPG2	0.056477124418261	0.0634221894677015	0.100839779544345	1081
NMB	0.0564741118431514	0.063436320581004	0.100854287594059	1081
FH	0.0564599485005251	0.0635027915225178	0.100944033395496	1081
RASA4	0.0564549047195005	0.0635264767041326	0.100973715733372	1081
SPG21	0.0564369067621187	0.0636110530472951	0.101092194985623	1081
RRN3P1	0.056373182581279	0.0639112539408223	0.101537240586582	1081
C11orf35	0.056353024599297	0.0640064599213317	0.101680477495089	1081
PRMT5	0.0563470113968716	0.0640348828626452	0.101717608836776	1081
LYZL6	0.0563415630079719	0.0640606450546594	0.101742486173614	1081
CTSE	0.0563338089256439	0.0640973242362355	0.101792715548992	1081

KRT3	0.056278791885068	0.0643580692667814	0.102167672389311	1081
OR5A2	0.0562786407132344	0.0643587869258059	0.102167672389311	1081
PGCP	0.0562745217799163	0.0643783433032619	0.102190664710756	1081
GABRD	0.0562680245687724	0.0644092015175569	0.102223537742944	1081
LRP11	0.0562663080535929	0.0644173560654705	0.102228425865677	1081
CASP3	0.0562528467729182	0.0644813352904509	0.10231383895921	1081
COBRA1	0.0562284155507178	0.064597586604182	0.102482153263604	1081
MYO15A	0.056221725707503	0.0646294490538281	0.102524628017117	1081
ORAOV1	0.0562066360199183	0.0647013659181814	0.102630631214312	1081
CCT2	0.0561818529679995	0.0648196240630444	0.102785841211432	1081
RPUSD2	0.0561670213423401	0.0648904817849249	0.102890102759332	1081
C21orf90	0.0561519723445717	0.0649624432092797	0.102996097784585	1081
RPL8	0.0561428831411817	0.0650059378737709	0.103056946444252	1081
CAMK1D	0.0561329565849287	0.0650534669351309	0.103124180941786	1081
NPHS1	0.0560684045644054	0.0653632454131425	0.103598944047817	1081
GNPDA1	0.0560660077831233	0.0653747706669041	0.103600908990307	1081
ONECUT3	0.0560533804774573	0.0654355184454365	0.103680865115882	1081
CASS4	0.0560360688921852	0.065518877014918	0.103796616661334	1081
USP9Y	0.0560338926655127	0.0655293621429737	0.103805064116553	1081
OGDH	0.056026483965731	0.0655650678475421	0.103845293795797	1081
YWHAH	0.0560238147157281	0.0655779360334997	0.10385750954362	1081
LOC14780	0.056018325665974	0.0656044047249439	0.103891261157456	1081
OR4D11	0.0560133027213999	0.0656286335213006	0.103921460641927	1081
TTR	0.0560008661537278	0.0656886545484994	0.104000152929859	1081
UGT1A7	0.0559597027882412	0.0658876384479975	0.10428240701877	1081
HCRTR2	0.0558899849131752	0.0662257864634122	0.104776444538242	1081
PRRX2	0.0558849603322935	0.0662502119156164	0.10480395218706	1081
CALCA	0.0558842679882577	0.0662535781137857	0.10480395218706	1081
FOXRED1	0.0558686383502348	0.0663296073503736	0.104915981475603	1081
ATP8B3	0.0558562665758969	0.06638983982535	0.105003008815394	1081
CHIA	0.0558256710719669	0.0665389884856035	0.105214122687793	1081
AQP3	0.0558173614307193	0.0665795443402333	0.105262327562383	1081
MYADML	0.0558172835707915	0.0665799244378203	0.105262327562383	1081

STK11	0.0557789154442447	0.066767447690423	0.105525673444498	1081
GPR111	0.0557617413787603	0.0668515260956801	0.105650270118261	1081
RPL30	0.0557516214186066	0.0669011107401968	0.105720338552029	1081
PRCC	0.0557281472932896	0.0670162430443895	0.105855485858462	1081
TMEM42	0.0557205928815687	0.067053329394134	0.105902729960394	1081
ACOT4	0.0557163980144921	0.0670739302483119	0.105924975495311	1081
HPD	0.055711497293631	0.067098004116448	0.105948369095928	1081
POLR2J3	0.0556954828359912	0.0671767216535727	0.106047728055107	1081
KRTAP10	0.0556757885621981	0.0672736310299329	0.106184070915772	1081
TSPY2	0.0556394849131426	0.0674525710568076	0.106433151401221	1081
ITGB1BP2	0.0556247421644462	0.0675253493703163	0.106539643097198	1081
LOC10013	0.0555979355464476	0.0676578468842557	0.10673197537066	1081
RPPH1	0.0555794346050427	0.0677494163072217	0.106859686291823	1081
CGB2	0.0555783642770181	0.0677547169523681	0.106859686291823	1081
IPO4	0.0555673327963964	0.0678093686209065	0.106929137445649	1081
NPB	0.0555610294778501	0.0678406124926243	0.106970032769561	1081
OPRL1	0.0555599021427018	0.0678462016390983	0.106970472882495	1081
TUBB2A	0.0555456881272545	0.0679167048962831	0.107073252475788	1081
SGOL2	0.0555357198972541	0.0679661844253531	0.107126107913992	1081
CSH1	0.0555057094457877	0.0681153267386848	0.107344383724541	1081
GPNMB	0.0555018044066539	0.0681347532746212	0.107350192725892	1081
ORC4L	0.055501754924675	0.0681349994635523	0.107350192725892	1081
ILK	0.0554902333815283	0.0681923427750942	0.10743213773397	1081
PPAP2C	0.0554680410397865	0.0683029068698353	0.107589495573761	1081
ABCG1	0.055437537877659	0.068455116287689	0.10781239290247	1081
SDHA	0.0553710200697805	0.0687880028778645	0.108277411937379	1081
SFRS4	0.0553581198978301	0.0688527149810888	0.108353874173427	1081
SCFD1	0.055343377726513	0.0689267283955314	0.108461876544913	1081
PYGL	0.0553209679648807	0.0690393621484167	0.108630629577431	1081
UPK3BL	0.0553143679076884	0.0690725634796848	0.108674382325699	1081
FAS	0.0552818897107015	0.0692361346546622	0.108906217897882	1081
NINJ1	0.0552610005205891	0.069341507463245	0.109054935424082	1081
PLXNA3	0.0552598374777998	0.0693473781447974	0.109055654389301	1081

PLEKHA2	0.0552519621918191	0.0693871408869914	0.109103473313716	1081
DAP	0.0552173141656622	0.0695623027798872	0.109350962895109	1081
CD1B	0.055215233541114	0.0695728328342627	0.109358982322583	1081
ANXA6	0.0552129640265425	0.0695843203525694	0.109367708324175	1081
BRS3	0.0552081922991679	0.069608478301669	0.109389405990102	1081
OR5F1	0.0551948429870043	0.0696760987442538	0.109487130203042	1081
HSPA8	0.0551720216077056	0.069791824315347	0.109651871847814	1081
C13orf16	0.0551562261411163	0.0698720142203718	0.109769299420525	1081
HNF4A	0.0551367272404487	0.0699711097413107	0.109916407184865	1081
PRR24	0.055101144036261	0.0701522443222691	0.110192355701529	1081
ULK1	0.0550931688544993	0.0701928942655693	0.110247610711047	1081
OR4K5	0.0550517016087639	0.0704045663084339	0.11054559534555	1081
OR13J1	0.0550419076464683	0.0704546364416447	0.110598461006801	1081
ITGAE	0.0550276691159356	0.0705274807773469	0.110686826134283	1081
FOXI3	0.0550241459676305	0.0705455147254962	0.110706504178945	1081
CDH16	0.0550188195405522	0.0705727862931054	0.110726813523583	1081
CUX2	0.0549840761564248	0.0707508860660718	0.110967088870919	1081
HS3ST2	0.0549825335674544	0.0707588021338403	0.110967088870919	1081
PDZK1IP1	0.0549808496111768	0.0707674444809979	0.110967088870919	1081
HYLS1	0.0549799309181723	0.0707721597326166	0.110967088870919	1081
ENOPH1	0.0549488555353913	0.0709318077626029	0.11120009862861	1081
TGFA	0.054935788623499	0.0709990262957952	0.11129681617997	1081
CDK2	0.0549325020019602	0.0710159414713436	0.111314670163416	1081
FGB	0.0549067933687706	0.0711483693486549	0.111487546711936	1081
TSPAN31	0.05487695201302	0.0713023387589582	0.111711433683249	1081
FBP1	0.054846903527694	0.0714576523372291	0.111911249812134	1081
ALG6	0.0548398462183613	0.0714941700354895	0.111959736890931	1081
OR1F2P	0.0548226261996915	0.0715833382681116	0.112081948500786	1081
OPLAH	0.0548180298045534	0.071607154577206	0.112110525293994	1081
DKFZp779	0.0548147716038282	0.0716240409331072	0.112125577346987	1081
ABCC10	0.0548140270002404	0.0716279004655437	0.112125577346987	1081
CDH26	0.0548118705628812	0.0716390789703766	0.112134362537548	1081
GIMAP6	0.0547945332357451	0.0717290038053054	0.112257674149928	1081

XPO6	0.0547834602672456	0.0717864851259131	0.112338906460151	1081
OTOS	0.0547355541856175	0.0720356066248863	0.112660829208127	1081
OR5M8	0.0547254719604177	0.0720881262378688	0.112732130341707	1081
CD99	0.0547175011132859	0.0721296695721878	0.112788340722792	1081
PIGS	0.0546607438663149	0.0724260495992421	0.113207849874048	1081
CTAGE5	0.054652724881259	0.072468004007993	0.113264639672668	1081
FLJ46321	0.0546511403388306	0.0724762965024464	0.113268812493544	1081
C6orf218	0.0546442179720809	0.0725125328965829	0.113307863419304	1081
DUSP9	0.0546211681779503	0.0726332980368372	0.113478966104551	1081
ZYG11A	0.0546030393351405	0.0727283962201688	0.113609921049574	1081
C20orf30	0.0545608212880334	0.07295025324343	0.113929714478136	1081
OPTC	0.0545351977183902	0.0730851752945729	0.114111809508439	1081
PLCG2	0.0545050591646405	0.0732441320610736	0.114326905344051	1081
C1orf85	0.0544891319028039	0.0733282496257606	0.114449336694115	1081
TUSC2	0.0544872690329365	0.0733380932600864	0.114455832718039	1081
IL28RA	0.0544825811635567	0.0733628693163989	0.114485630364773	1081
FFAR1	0.0544756576463841	0.0733994735939641	0.114533880367963	1081
LOC84989	0.0544691078983204	0.0734341155034755	0.114579061019093	1081
FAM122C	0.0544469450751831	0.0735514347179525	0.11475322586688	1081
SNAR-B2	0.0544201263123164	0.0736936048469951	0.114948329047055	1081
OR51F1	0.0544069345648609	0.0737636184915038	0.115048629181739	1081
TGM7	0.0543658226936203	0.0739821633472012	0.115380559002041	1081
LOC28479	0.0543636756750725	0.0739935910992462	0.115389448265545	1081
UGT1A6	0.0543603505353583	0.0740112923799451	0.115399186145046	1081
FLJ45244	0.0542987043349594	0.0743400906164769	0.115867011641696	1081
KRTAP4-4	0.054292066348181	0.074375566179372	0.115913335964989	1081
IFNA14	0.0542782298697364	0.0744495572338309	0.116019674361841	1081
KRT77	0.0542654817869694	0.0745177812026277	0.116099048903862	1081
HHLA1	0.0542607592652483	0.0745430676865587	0.116129463966107	1081
OTP	0.0542461078439298	0.074621562473164	0.116242760286258	1081
CD163L1	0.0542250885293469	0.07473429090647	0.116391363765817	1081
RNASE3	0.0542088690310017	0.0748213724218924	0.11651797676554	1081
MT1M	0.0541882316908698	0.0749322926915426	0.116663655326619	1081

GP1BA	0.0541490411000313	0.0751433009298451	0.116956022346576	1081
UTP14A	0.0541251823801341	0.0752719970757469	0.117138229516487	1081
IL16	0.0541116521456274	0.075345060226653	0.117242873340485	1081
ANAPC10	0.0541061990039176	0.0753745234867337	0.117270603617566	1081
C12orf34	0.0541002693984454	0.0754065717331021	0.117311405325766	1081
CBWD1	0.054094039598815	0.0754402544291897	0.1173495184656	1081
SAFB2	0.0540818955370807	0.0755059490574626	0.117418975665117	1081
C9orf53	0.0540805402471932	0.0755132835375567	0.117418975665117	1081
HOXD11	0.054080396670929	0.0755140605693661	0.117418975665117	1081
MSC	0.054079942665075	0.0755165176826637	0.117418975665117	1081
DDX55	0.0540709075355338	0.07556543002691	0.117485960321996	1081
MS4A10	0.0540623073837162	0.0756120115700885	0.117540240225282	1081
GDPD3	0.054057624741953	0.0756373842956122	0.117570610079869	1081
LCT	0.0540477113471212	0.0756911225627918	0.117645063191686	1081
FLI1	0.0540400501917143	0.0757326732604903	0.117700563403223	1081
NF1P1	0.0540118153783883	0.0758859668332977	0.117911515762189	1081
C1orf174	0.0539919039891115	0.0759942224751013	0.118062126869106	1081
ATP4B	0.0539918311049058	0.0759946189680663	0.118062126869106	1081
AGBL2	0.0539700175282517	0.0761133614439878	0.118219249521136	1081
POLR1D	0.0539633280872912	0.0761498057650362	0.118266736345436	1081
C1GALT1	0.0539336429822315	0.0763117028436085	0.118463377805694	1081
COPS7B	0.0539120949696003	0.0764293972318314	0.118613321645857	1081
C3orf71	0.0538894361585687	0.0765533181573135	0.118792681734639	1081
REPIN1	0.0538759905780278	0.07662692928036	0.118875557788512	1081
S100A5	0.0538401864584388	0.0768232287878983	0.119156447792748	1081
CHRAC1	0.0538380541905981	0.0768349320532973	0.119165425068417	1081
HSD17B8	0.0537966724712208	0.0770623492357563	0.119481338055438	1081
C3orf67	0.0537609921638459	0.0772588732797518	0.119767603095083	1081
HORMAD	0.0537555247095715	0.07728902354486	0.119795905155898	1081
FTCD	0.0537295109744866	0.077432607395341	0.120009222648034	1081
DDX51	0.0536765739084324	0.0777254657700974	0.12041678904396	1081
COL20A1	0.053672257373496	0.0777493853684662	0.120444583839415	1081
STAU1	0.0536677800511392	0.0777742022774399	0.120473764312113	1081

EFCAB10	0.053661878257009	0.0778069245928196	0.120505919575794	1081
TTC7B	0.0536330529213798	0.0779669066970324	0.120716573315788	1081
HSD17B7	0.053543352722015	0.0784664593486671	0.121434034230537	1081
KRTAP12	0.0535240436015991	0.0785743340073288	0.121580527460352	1081
GRIN3A	0.0535209830693295	0.0785914434053932	0.12159009503123	1081
PSMA8	0.0535056308170652	0.0786773133125269	0.12170425222152	1081
UGT1A10	0.0534695970183915	0.0788791609986606	0.121979020366778	1081
MMADHC	0.0534580028998957	0.078944196241902	0.122070220547522	1081
MMAB	0.0534405593788123	0.0790421247768436	0.122193508067647	1081
CCDC9	0.0534332572989837	0.079083148194687	0.122228787815097	1081
MIB2	0.0534252413910043	0.0791282018418086	0.122279658338695	1081
C11orf80	0.0534117157249134	0.0792042705095146	0.122387821539785	1081
ZNF280B	0.0534016217036506	0.0792610782151009	0.122466208056696	1081
THOC7	0.0533278864618072	0.0796770539272833	0.123080611254131	1081
ALKBH2	0.0532994337849302	0.0798380421739112	0.123298790738164	1081
FOXR2	0.0532686054599101	0.0800127699737583	0.123542366147111	1081
SLC27A5	0.0532451322970422	0.0801460184038345	0.123738622068152	1081
HTR1E	0.0532418923689653	0.0801644244053639	0.123757554574761	1081
LCE3C	0.053236949813804	0.0801925096223298	0.123791425773321	1081
E4F1	0.0532001142761909	0.0804020727532083	0.124086398193012	1081
MRPS25	0.0531983146104614	0.0804123227042889	0.124089023565712	1081
GLMN	0.0531883059148319	0.0804693463205629	0.124161686705556	1081
PLOD2	0.053182884634176	0.0805002472572276	0.124199853028785	1081
C19orf76	0.0531309551625491	0.0807967296751227	0.124609563566479	1081
LDHC	0.0531170210713512	0.0808764344698682	0.124703847868006	1081
C1orf51	0.0531048320654275	0.080946209417058	0.124801881884788	1081
MKI67IP	0.0530476671003601	0.0812740969382553	0.125278650456894	1081
LIN28B	0.0530414033240001	0.0813100901314217	0.125324542134467	1081
PRDX6	0.0529846891050402	0.0816365722898715	0.125789257211218	1081
OR1F1	0.0529712436400302	0.0817141282550784	0.125897708632458	1081
PRDM1	0.0529511921889598	0.0818298996640659	0.126040812186002	1081
HAUS5	0.052950982066342	0.0818311135557571	0.126040812186002	1081
C11orf20	0.0529475593435519	0.0818508888965336	0.126061632784455	1081

HHLA3	0.0529275849694284	0.0819663713378043	0.126229841036623	1081
WDR63	0.052904532704547	0.0820998126969046	0.126396692913958	1081
LOC64794	0.0529015861967971	0.0821168816556989	0.126413310480976	1081
TMEM89	0.0528919145874456	0.0821729289608988	0.126489925415856	1081
MCHR2	0.0528870683637282	0.082201024640062	0.126523505606208	1081
SIRT4	0.0528754255080192	0.0822685551621895	0.126616079026484	1081
SND1	0.0528739609506741	0.082277053021075	0.126616079026484	1081
TNK2	0.0528727769968289	0.0822839232427132	0.126616079026484	1081
MAP2K6	0.0528610612588806	0.0823519321155223	0.126707375885374	1081
LOC28537	0.0527824315565062	0.0828095494472468	0.12738228265232	1081
GDI2	0.0527605796717337	0.08293709010925	0.127558992231703	1081
AFM	0.0527590503223574	0.0829460222547932	0.127562990939981	1081
SAA4	0.0527378191568295	0.0830701030378341	0.127724563097021	1081
EPS8L1	0.0527180381562834	0.0831858437596422	0.127848277718339	1081
C11orf71	0.052713476982839	0.083212550167554	0.127875263034966	1081
HNRNPH1	0.0527071895102101	0.0832493757065878	0.127922095622365	1081
AMDHD2	0.0526688445633364	0.0834742463751523	0.128228510997975	1081
CHRNA2	0.0526663418785188	0.0834889402183411	0.128241303903811	1081
SPRYD3	0.052647459501298	0.0835998704795269	0.128392116399334	1081
PCSK2	0.0526432055839507	0.0836248778529031	0.128410943238565	1081
CH25H	0.0526411860260236	0.0836367522835891	0.128419388319581	1081
PDIA2	0.0526049565625106	0.0838500030268525	0.128727199434228	1081
C20orf144	0.0525792021396024	0.0840018636456075	0.128921037874506	1081
C3orf16	0.0525589861113171	0.0841212228814938	0.129094388279705	1081
PRKCZ	0.0525497265370366	0.0841759389056882	0.129168266809037	1081
SNORA69	0.0525139595407311	0.0843875609243743	0.129453808621803	1081
SEC24C	0.0524901301399091	0.084528790438768	0.129621107055567	1081
OR52B6	0.0524778709392086	0.0846015213025286	0.129703017248055	1081
C19orf25	0.0524723834285695	0.0846340937485115	0.129743080325985	1081
CYP21A2	0.052446742682822	0.084786424885802	0.129956823352696	1081
PLUNC	0.0524429201277868	0.0848091535636958	0.129981771039307	1081
PPIAL4G	0.0524355335394261	0.0848530877087455	0.13003921277236	1081
GNB1	0.0524318390640097	0.0848750687026312	0.130053111661558	1081

STRA6	0.0524290605784065	0.0848916028683555	0.130068553375648	1081
ULBP1	0.0524266722862413	0.0849058171565424	0.130080438559476	1081
HIST1H3I	0.0524088554623456	0.0850119173947637	0.130213281319762	1081
SOST	0.0523567834132237	0.0853226234082579	0.130639526378305	1081
RPL29P2	0.0523160945406069	0.085566045285495	0.130986263767938	1081
ASPM	0.052292393828098	0.0857080933585316	0.131179329423263	1081
PRSS1	0.0522867472716869	0.0857419635391865	0.131211778752424	1081
ATP6V0E	0.0522572219934894	0.0859192432721978	0.131433150255784	1081
PRB1	0.0522259077126761	0.0861075878416943	0.131701264677772	1081
NKX2-3	0.0522188799122683	0.0861499033623013	0.131755982632369	1081
ZACN	0.0521842888218386	0.0863584264514806	0.132064867597744	1081
GAGE2A	0.0521316223788531	0.0866766934944857	0.132491235332454	1081
SNAR-C4	0.052128160136918	0.0866976491539368	0.132503159	1081
GSTZ1	0.05212364422183	0.0867249884428467	0.132534887891151	1081
OR2G3	0.0520465806863333	0.0871926023463487	0.133209083442074	1081
RPA2	0.0520175528465472	0.0873692663891297	0.133468861106318	1081
UBE2E1	0.0520007598337116	0.0874716006394211	0.133615058756532	1081
ZNF101	0.0519993502854796	0.0874801946187826	0.133618054484447	1081
CDCA7	0.0519971556633205	0.08749357652784	0.133628362331565	1081
SLC22A8	0.0519672107537939	0.0876763331787725	0.133897333931193	1081
PABPN1L	0.0519653805564527	0.087687513012582	0.13390425634009	1081
DYSF	0.0519548259839433	0.0877520084500673	0.133988910151791	1081
HSD17B1	0.0519145816466625	0.0879982787264893	0.134338081611483	1081
MAPKAP	0.0518970423915548	0.088105782038784	0.134481813848409	1081
OR8H3	0.0518584703897684	0.0883425733250006	0.134833028231846	1081
USP15	0.0518549373343202	0.0883642881358939	0.134855953371029	1081
SARS2	0.0518337826584972	0.0884943985812072	0.13502383241827	1081
VNN1	0.0518047852165357	0.0886729959522821	0.135265599232071	1081
TREX2	0.0517942418267576	0.0887380053281675	0.135335894370835	1081
CDV3	0.0517839549670525	0.0888014699281155	0.135410310476261	1081
PIP4K2A	0.0517484487583281	0.0890208055035566	0.135713949361976	1081
SLC22A1	0.05172467961201	0.0891678801013568	0.135907312146617	1081
DNAI1	0.0517177351384746	0.0892108867850597	0.13595228945344	1081

OR3A3	0.0516822386366644	0.0894309750368372	0.136246463897534	1081
RXFP4	0.0516657764575398	0.0895331932644544	0.136391876762888	1081
LOC2857[illegible]	0.0516478409112466	0.089644666952554	0.136551366030941	1081
POU2F1	0.0516436826422388	0.0896705275127517	0.13658043072315	1081
PLEKHM[illegible]	0.0515531844446625	0.0902348292549518	0.137346477925274	1081
CUEDC1	0.0515390876012021	0.0903229864859429	0.137470275216026	1081
UGT1A3	0.0515325217199567	0.090364071061846	0.137522415316226	1081
AAGAB	0.0515090197786271	0.0905112524337907	0.137736000927535	1081
PPAPDC1	0.0514967995939327	0.0905878576516221	0.137842162730714	1081
ALOX15	0.0514884980384067	0.0906399276867044	0.137910977586757	1081
XRCC1	0.0514840520794553	0.090667824060264	0.13794300397961	1081
DNMT3L	0.051465844109879	0.0907821427667561	0.13810649947412	1081
FUZ	0.0514516858282326	0.0908711155003684	0.138225733921211	1081
OR5AR1	0.0514476379486944	0.0908965658424839	0.138248443921722	1081
ZSWIM7	0.0514466308968241	0.0909028983959854	0.138248443921722	1081
IGFBP2	0.0514337307607436	0.0909840485010278	0.138354463744299	1081
ECSIT	0.0514115896743783	0.0911234656929237	0.138542054066713	1081
SNORA70	0.0514002307516685	0.0911950566902702	0.138640435969021	1081
PAAF1	0.0513759183719407	0.0913484401971691	0.138852661870669	1081
IQCG	0.0513551478984471	0.0914796422725342	0.139041602050717	1081
PASD1	0.0513188319827017	0.0917094045487755	0.13936979138768	1081
DKC1	0.0513157944734653	0.0917286431247216	0.139388512917517	1081
ZBTB48	0.0513077296302767	0.0917797388811988	0.139445119523475	1081
WT1	0.0512328841006312	0.0922550217153658	0.140156667606036	1081
C1orf131	0.051208503921021	0.0924102658861868	0.140362061246541	1081
SLC35B2	0.0512083698323168	0.0924111202935154	0.140362061246541	1081
ALDH8A1	0.0512071056441475	0.0924191759563636	0.140363713784751	1081
CELA1	0.0512055306344304	0.0924292130248625	0.140368375151591	1081
ROR2	0.0511915164768481	0.0925185595812612	0.140493470734548	1081
PMPCA	0.0511672396178297	0.0926734993612558	0.140707540294882	1081
PRSS53	0.0511380902126121	0.0928598114892169	0.140969171247195	1081
PRSS38	0.0511369733985144	0.0928669557163771	0.140969393633217	1081
WDR55	0.0511292303799307	0.0929164996976493	0.141033526973229	1081

ARHGEF1	0.0511198873645794	0.0929763095188664	0.141101373876148	1081
UNC50	0.0511190114985182	0.0929819180047587	0.141101373876148	1081
C5orf25	0.0511121666995451	0.0930257570586833	0.141157266943897	1081
ANKRD16	0.0510763855992458	0.0932551948748151	0.14149475790865	1081
NAP1L4	0.0510495203414204	0.0934277598326986	0.141735237730982	1081
KRTAP7-1	0.0510464811177248	0.0934472979286278	0.141754203070196	1081
CLDN9	0.0510270067143506	0.0935725696935444	0.141933545401435	1081
TAF4	0.051024306991172	0.0935899466449208	0.141949215115339	1081
C7orf40	0.0510212453867826	0.0936096559971765	0.141968419789439	1081
GRB14	0.0510186273737859	0.0936265123271844	0.141983295003849	1081
KLRF1	0.0510118853141143	0.0936699329241635	0.142024990953075	1081
WBSCR16	0.051011074192087	0.0936751578452969	0.142024990953075	1081
CENPF	0.0509838503497774	0.0938506583105203	0.142257332520763	1081
NR0B2	0.0509781180312762	0.0938876456554189	0.142293606810744	1081
CTNS	0.0509528667023024	0.0940507169140553	0.142508589111218	1081
ARHGAP1	0.0509510604180225	0.0940623904537514	0.142515557738479	1081
NT5M	0.0509450145252669	0.0941014719074073	0.142564048399994	1081
LRP3	0.0509318647268625	0.0941865188413928	0.142671435601162	1081
KIF21B	0.0509295982295399	0.0942011837340014	0.14268292077605	1081
FGF5	0.0509154037781508	0.0942930675146577	0.142800619837827	1081
FOXL2	0.0508784023806095	0.0945329229372718	0.143153103252751	1081
TPRN	0.0508471465962704	0.0947359136234796	0.143417372807169	1081
C2orf52	0.0508354039816936	0.0948122661086661	0.143511390845223	1081
CUX1	0.0508317542116114	0.0948360075647591	0.143536541877691	1081
COMMD3	0.050822470984917	0.0948964156323245	0.143599971106796	1081
TMEM8C	0.0508220277187254	0.0948993008361765	0.143599971106796	1081
IFNA7	0.0508146530285094	0.0949473127300516	0.143661830775641	1081
GPHB5	0.0508086142420167	0.0949866418789392	0.143710544547595	1081
ESRP2	0.0508012074559552	0.0950348982715902	0.143772756509551	1081
C9orf122	0.0507500232246436	0.0953689079744461	0.144223905948894	1081
NOMO3	0.050720241456239	0.0955636846150079	0.144453397356444	1081
GATA5	0.0506098163119064	0.0962886570383715	0.14541978206317	1081
KRT13	0.0505895274208498	0.0964223360459539	0.145601912118283	1081

CCDC83	0.050542173303339	0.096734918761569	0.146004715550446	1081
ITGA5	0.0505147852751213	0.0969160751873838	0.146259386800699	1081
CSH2	0.0505144686631845	0.0969181709805179	0.146259386800699	1081
FAM74A3	0.0504865768862839	0.0971029409513849	0.146505298492035	1081
CDC26	0.0504811565329121	0.0971388808753475	0.146548547534927	1081
SNAP47	0.0504670095174147	0.097232733404184	0.1466681703053	1081
PRKCD	0.0504326242165727	0.0974611502992636	0.146988824992771	1081
POLR2J2	0.0504317145321812	0.0974671990197785	0.146988824992771	1081
ANKRD1:	0.0504195988505989	0.0975477878106065	0.147088337233882	1081
TSHR	0.0504121030802929	0.0975976733760033	0.147152543234757	1081
MMEL1	0.0504102144362209	0.0976102458189027	0.147160485108651	1081
VSTM2L	0.0504082818928109	0.0976231118315549	0.147168868356407	1081
NMT1	0.0504036938524945	0.0976536623797209	0.147203908159195	1081
SCARNA:	0.0503776576584831	0.0978271750429603	0.147443396770944	1081
C6orf191	0.0503459880527208	0.0980385618739069	0.147728093114295	1081
LOC28437	0.0503449643236217	0.098045401089011	0.147728093114295	1081
SUCNR1	0.050324847211791	0.0981798744875936	0.147919644869686	1081
VANGL1	0.0502794212215412	0.0984840669656382	0.148344663453176	1081
MIAT	0.0502585570453135	0.098624034339484	0.148522177750535	1081
SCGB1C1	0.0501995928496742	0.0990204534108384	0.149074585902499	1081
P2RY14	0.0501788934207627	0.0991599174440018	0.149251086140871	1081
C3	0.0501626630253751	0.0992693804661162	0.149404682068727	1081
TMEM184	0.0501612385644076	0.0992789920848111	0.149407985622928	1081
TXLNA	0.0501581206985782	0.0993000326219727	0.149428487127037	1081
MPP3	0.0501555012925632	0.0993177121102839	0.149443928179091	1081
ACOT2	0.0501453902960571	0.0993859790649391	0.149524312790381	1081
TMPRSS1	0.0501434554372213	0.0993990470153983	0.149532805774416	1081
CCNI2	0.0501366358344068	0.0994451172258661	0.149590941346705	1081
PPP1R11	0.0500895535279139	0.0997636487357194	0.150032991538522	1081
KIAA1279	0.050088033475527	0.0997739460521378	0.150032991538522	1081
FOXN1	0.0500783491212047	0.0998395708152704	0.150117043892642	1081
PCYOX1L	0.0500582835074239	0.099975652230688	0.150310434673275	1081
C1orf43	0.0500141656604134	0.100275370988258	0.150727305518464	1081

TDH	0.0499982386698006	0.100383748073863	0.150878952842495	1081
FAM169B	0.049986046611243	0.100466773364721	0.150992476084177	1081
C20orf151	0.0499522959419661	0.100696893250945	0.151293176800438	1081
IFRD2	0.0499506048951447	0.100708434225854	0.151299232417021	1081
BEND4	0.0499187825902224	0.100925809902806	0.151569288839424	1081
MT1X	0.0499028234198117	0.101034966216122	0.151699291527449	1081
GSTA2	0.0498900788422659	0.101122203035193	0.151808101611795	1081
LOC72915	0.0498592022143267	0.1013338024436	0.152089997055073	1081
OR8D2	0.0498254527464062	0.101565492266381	0.152359583119088	1081
ARGFX	0.0497973214344464	0.101758935207606	0.152638402811409	1081
LOC64649	0.0497918773159958	0.10179640510265	0.152683239	1081
XAGE1D	0.0497747058467859	0.101914661968752	0.15284923059563	1081
CFHR5	0.0497297016610372	0.102225116024831	0.153280607541513	1081
TRH	0.0497196288829661	0.10229470427051	0.153362120825291	1081
ZDHHC11	0.0497084618292474	0.102371896362697	0.153455007978758	1081
RASL10A	0.0497008477810779	0.102424554889309	0.153522519214668	1081
FAM53A	0.0496853765291803	0.102531619891524	0.153671563329719	1081
TNP2	0.0496835505850744	0.102544261753541	0.153679076999337	1081
DAGLA	0.0496791399347005	0.102574803849979	0.153701980330097	1081
C11orf36	0.0496670373331751	0.102658646939515	0.153804733199207	1081
CRNN	0.0496633357521764	0.10268430121931	0.153831728122481	1081
PTGER2	0.0496592330860074	0.102712741220985	0.153862892020876	1081
CCDC43	0.0496569098319497	0.102728848973756	0.153875579045008	1081
KRTAP21	0.049654920742167	0.102742641468945	0.153884796460364	1081
RSPH6A	0.0496301046022021	0.102914842091844	0.154131253731336	1081
CCDC123	0.0496160332414751	0.103012585858855	0.15426617173109	1081
SNHG11	0.0496011579726167	0.103115993947975	0.154402344129289	1081
AWAT2	0.0496007479777133	0.103118845267583	0.154402344129289	1081
TAS2R38	0.0495732758930104	0.103310043234273	0.154677132735372	1081
OR4C15	0.049492800220438	0.103871751349772	0.155437262735192	1081
CCDC78	0.0494654274552079	0.104063360522006	0.155712426481157	1081
ERGIC2	0.0494564497359005	0.104126265528263	0.15579498061994	1081
NKIRAS1	0.0494453600551928	0.104204010247938	0.155888146921484	1081

CYP2F1	0.0494387620884186	0.104250287442326	0.155922640175637	1081
OR5I1	0.0494374533520528	0.104259468669025	0.155924795570714	1081
GAGE10	0.0494161357396063	0.104409109232045	0.15613699840246	1081
RBMXL3	0.0494097226792715	0.104454159499254	0.156192773390178	1081
MAP7D2	0.0494034008311479	0.104498584108844	0.15624760460719	1081
LRRC28	0.0493752457408742	0.104696616294891	0.156519108710782	1081
SNRPE	0.0493598718671469	0.104804875803439	0.156659065300903	1081
PSPN	0.0493518817999129	0.104861175174658	0.156731590287393	1081
NSUN2	0.0493369652617437	0.104966343795613	0.156865504136693	1081
ATP6V1G	0.0493165520614275	0.105110401957102	0.157045839014005	1081
HDAC6	0.0493015519838679	0.105216358972537	0.157180834371654	1081
OR2T1	0.0492922358802625	0.105282208335081	0.157267543149207	1081
FAM138E	0.049280758924744	0.1053633762231	0.157377119746004	1081
FAM113A	0.0492728950016893	0.105419020468129	0.157448559262341	1081
CGB1	0.0492661027423171	0.105467100479325	0.15750869146386	1081
PTP4A2	0.0492455130373911	0.105612953915914	0.157714822582363	1081
ATP6V1D	0.0491595447655148	0.106223663524505	0.158509311237123	1081
HOXD9	0.0491261403379948	0.10646171807816	0.158817484499744	1081
FAM22A	0.0491167166347122	0.106528951828168	0.158894249561561	1081
TNNC1	0.0491149515257773	0.106541548797786	0.158898071958899	1081
LMNA	0.0490799159484459	0.106791829602358	0.15923918633157	1081
SPRR2B	0.0490742456528838	0.106832379741844	0.159272196559409	1081
PCP4L1	0.0490735514903746	0.106837344762173	0.159272196559409	1081
VWA1	0.0490692007679568	0.106868467607211	0.159294508320183	1081
CIB2	0.0490627067539772	0.106914935810924	0.159335953903131	1081
LOC2854(	0.049049434158839	0.107009958181665	0.159458214924894	1081
SCARNA2	0.0490480630083171	0.107019778454124	0.159461053029462	1081
FBXO5	0.0490430572153802	0.107055636334098	0.159502684265248	1081
LOC64371	0.0490029035254324	0.107343612373321	0.159884441498007	1081
IGFBP3	0.0490010555058455	0.107356880817875	0.159892382380311	1081
GLTPD2	0.0489761823896814	0.107535591527678	0.160134867779795	1081
PLLP	0.0489189168925551	0.107947932428399	0.16070138181866	1081
PAGE5	0.048911027764374	0.108004836019546	0.160774212784582	1081

MLYCD	0.0488894606582814	0.108160518691011	0.160970275489836	1081
RNF5P1	0.0488748580569274	0.108266028583365	0.161103497253057	1081
EML3	0.0488596641694391	0.108375897097952	0.161255074011245	1081
NGEF	0.0488506384048932	0.108441205004665	0.161340330488847	1081
TBX4	0.0488260695870668	0.108619135640729	0.161569259948496	1081
HECTD3	0.048821870599971	0.108649568328302	0.161590664745193	1081
HHIPL1	0.0488147869773413	0.108700923042218	0.161655108126789	1081
MYT1L	0.0488050711338428	0.108771391967966	0.161747965689428	1081
EIF2S1	0.0488004675733268	0.108804794149333	0.161785693691933	1081
IFT57	0.0487848068138731	0.108918485025006	0.161930839913561	1081
CP	0.0487816607719257	0.108941335350478	0.161952859614448	1081
MTHFD2	0.0487782135814551	0.108966377324453	0.161978133943411	1081
THOC6	0.0487574607562642	0.109117231459429	0.162190410075274	1081
GHSR	0.0487459239142628	0.109201165090969	0.162267276739512	1081
TRIM41	0.0487347180586439	0.109282739507888	0.162376514779939	1081
CXorf30	0.0487157392296518	0.109421007994602	0.162518334181808	1081
MTHFD1	0.0487148724421662	0.109427326190385	0.162518334181808	1081
CHI3L1	0.0487078826064119	0.109478287096165	0.162571140612727	1081
SNORA34	0.0486979999689648	0.109550370566052	0.162666191129441	1081
TLR6	0.0486244287680951	0.110088173564588	0.163404528179976	1081
TECTB	0.0486188221376739	0.11012924311758	0.16345344452985	1081
C16orf5	0.0486173551081265	0.110139991361887	0.163457354205482	1081
DNAJC6	0.0486102222568799	0.110192262246074	0.163522881985058	1081
CATSPER	0.0485959201060228	0.110297130077555	0.163654392385089	1081
CHIC2	0.0485925119481614	0.110322131366262	0.163667378774089	1081
GORASP2	0.0485857056116104	0.110372074117831	0.163729114147184	1081
ZNF76	0.0485651911445735	0.110522710696915	0.163904594154104	1081
CYP2B6	0.0485610062616599	0.110553459960772	0.1639381266772	1081
C17orf70	0.0485163310265036	0.110882140817347	0.164372748931323	1081
C9orf70	0.0485156238730735	0.110887349614132	0.164372748931323	1081
C14orf48	0.0485143891147533	0.110896445139899	0.164374136365482	1081
IL17RE	0.0485055281566725	0.110961734339234	0.164458809466147	1081
NMUR1	0.0484840536861527	0.111120087977849	0.164681392798776	1081

COQ5	0.0484601458536167	0.111296594834094	0.164918712977773	1081
YIPF1	0.0484271101310402	0.111540854661012	0.16520618217743	1081
EIF2B1	0.0484261443937135	0.111548001488081	0.16520618217743	1081
CSMD3	0.0484146771098197	0.111632891375072	0.165319752756805	1081
TRIM55	0.0483976388947958	0.111759115682515	0.165470188550952	1081
ABHD6	0.0483668581033003	0.11198743422451	0.165747326607358	1081
CPB1	0.0483624182144635	0.112020397689281	0.165783934135363	1081
PLCXD2	0.0483509510509101	0.112105569738753	0.165885610576405	1081
SOX2	0.0483467916637111	0.112136476077462	0.165919156120489	1081
APIP	0.0483231686714129	0.112312134090457	0.166166858200078	1081
KCNK5	0.048288022518192	0.112573877980916	0.166541879409969	1081
PODXL2	0.0482864865339844	0.112585327856341	0.166546587580114	1081
PTPRN2	0.0482676292879323	0.112725972468646	0.166730155314308	1081
OR56A4	0.0482643449078532	0.11275048277702	0.166754164565236	1081
FBXO18	0.0482398057580105	0.112933743608041	0.1670086311957	1081
CTBP1	0.0482390862481397	0.112939120515216	0.1670086311957	1081
CXorf59	0.048218481699472	0.113093184102483	0.167224177462163	1081
CRYGA	0.0481644269127925	0.113498146650014	0.167747373511416	1081
C17orf65	0.0481642194559446	0.113499703049932	0.167747373511416	1081
RHBDL2	0.0481454008686637	0.1136409552564	0.167898573057586	1081
OR13C3	0.0481148620505226	0.113870473636311	0.168188599459073	1081
PGA5	0.0481148381820029	0.113870653165664	0.168188599459073	1081
LILRA2	0.0481073551518797	0.11392694846538	0.168249709739965	1081
TLX2	0.0481071182265389	0.113928731226749	0.168249709739965	1081
FOXN4	0.0480952490927806	0.114018069434992	0.168369301331763	1081
FRS3	0.0480801191844363	0.114132031128817	0.168500532582632	1081
SNORA78	0.0480604702097167	0.114280165030152	0.168698310106796	1081
TOP3B	0.0480601285661811	0.114282742021715	0.168698310106796	1081
TSPAN1	0.0479803757991394	0.114885562060506	0.169501219255083	1081
LOC40089	0.047975634163666	0.114921480757806	0.169537680181568	1081
C18orf2	0.0479609480454977	0.115032786586576	0.169643886221565	1081
GKAP1	0.0479459247964062	0.11514673512549	0.169799500702949	1081
FANCD2	0.0479445779864216	0.115156954756307	0.169802141235969	1081

RAET1L	0.0479300499225747	0.115267239394668	0.169927445122985	1081
TCP10	0.0479240247869663	0.11531300139839	0.169970030962944	1081
NLRP2	0.0478879938583671	0.115586960710626	0.170348915320474	1081
GPR3	0.0478564957014124	0.115826873715943	0.170674687313219	1081
HNF1A	0.0478556364437179	0.115833423923869	0.170674687313219	1081
SLC9A4	0.0478458624892367	0.115907952228919	0.170772010359949	1081
ANXA7	0.0478316927539852	0.11601606604579	0.170906299853194	1081
TRIP6	0.0478146236853075	0.11614640659137	0.171085797073936	1081
C9orf116	0.04778602473549	0.116365047599765	0.171370267117279	1081
FIBCD1	0.0477558845237055	0.116595821016848	0.171672475835726	1081
RPF2	0.0477438524691876	0.116688046592613	0.171795709903397	1081
CRTC2	0.0477341256891774	0.116762644077956	0.171892974638294	1081
MGC3771	0.0476941121966449	0.117069912584864	0.172332728463434	1081
RBP1	0.0476691837047153	0.117261661823218	0.172577160829101	1081
PLAU	0.0476640004391848	0.117301562253201	0.172623272086401	1081
DOCK10	0.0476622972992868	0.117314675232628	0.172629958550862	1081
FCRL1	0.0476525275003408	0.11738991801672	0.172722365519064	1081
MTIF3	0.0476519170839463	0.117394620436189	0.172722365519064	1081
TXNDC3	0.0476082084558425	0.117731719324604	0.173167748670718	1081
NSUN5P1	0.0476043663523601	0.11776138743555	0.173198740592038	1081
C1orf65	0.0476002762685947	0.117792976848088	0.173225487483284	1081
KRTAP19	0.0475905433892901	0.117868174609361	0.17331783777413	1081
FAM163A	0.0475867996321888	0.11789710948189	0.173335080317667	1081
RNF17	0.0475830165948197	0.117926353597833	0.173365423135689	1081
GSTA3	0.0475702302569587	0.118025238238918	0.173498133707166	1081
FOXR1	0.0475621752073535	0.118087566251226	0.173577090268584	1081
RPL10L	0.0475443895794974	0.118225278425453	0.173754679419304	1081
MESP1	0.0475439186936167	0.118228926151951	0.173754679419304	1081
NCKIPSD	0.0475432303087704	0.118234258896988	0.173754679419304	1081
ZNF815	0.0475327197007647	0.118315705346392	0.173836327343233	1081
RPL19P12	0.0474996219106456	0.118572466399763	0.174188167113193	1081
F11	0.0474845998767036	0.118689145929618	0.174334148359617	1081
FTLP10	0.0474824720936819	0.118705680166621	0.174345722291963	1081

HYAL4	0.0474775766037153	0.118743728106838	0.174363466922681	1081
C10orf82	0.0474293054812283	0.119119404563933	0.174864126279999	1081
CHD5	0.0473917889465412	0.119412023915898	0.175255370959185	1081
PIWIL4	0.0473780653546542	0.119519204804456	0.175387119812483	1081
RAD51L3	0.0473488548104842	0.1197475889618	0.175671073378639	1081
ATG12	0.0473414373222316	0.119805637291206	0.175730636612156	1081
PTPRH	0.0473361235134536	0.11984723601948	0.175778854722419	1081
MS4A12	0.0473329742659981	0.119871894983074	0.175802222221464	1081
ZFP41	0.0473205753370598	0.119969018566605	0.175909106000023	1081
KCMF1	0.0473174147850487	0.119993785717635	0.175909106000023	1081
FAM186A	0.0473169833025796	0.119997167269909	0.175909106000023	1081
LOC10012	0.0473002572038352	0.120128308087744	0.176062914982001	1081
HSPA14	0.04729311383775	0.120184349720791	0.17613223665978	1081
MFSD4	0.0472276816393169	0.12069863460858	0.176847333929002	1081
ATAD2	0.0472161207586267	0.120789679501893	0.176954991896272	1081
STK25	0.0472114957867126	0.12082611736535	0.176995501332532	1081
C17orf86	0.047183907313854	0.121043651749665	0.17727483737271	1081
PCSK7	0.0471828485584905	0.12105200610072	0.17727483737271	1081
NCK1	0.0471794004336723	0.12107921744002	0.177301798017678	1081
BRSK1	0.04717709873337	0.121097384276106	0.177315511429143	1081
NDUFA4L	0.0471739569702742	0.121122184988135	0.177326047717005	1081
LOC26102	0.0471718380906396	0.121138913417612	0.177337650664763	1081
C17orf81	0.0471649165033003	0.121193571518953	0.177404773888193	1081
P2RX1	0.0471630360337107	0.121208424460103	0.177413624264242	1081
XPNPEP1	0.0471147004718092	0.121590692470725	0.177947293848365	1081
LMF1	0.0470563527995195	0.122053395988038	0.178546631484934	1081
C15orf21	0.047051124061028	0.122094927477141	0.178581450230669	1081
FAM134A	0.047046261056337	0.122133563869081	0.178612024983132	1081
TAF7L	0.0470141056160334	0.122389277868998	0.178947017177454	1081
C2orf65	0.0469679322060275	0.122757199310768	0.179445887824934	1081
DLL3	0.0469593545462792	0.122825643212693	0.179519885548844	1081
USP36	0.046956853683501	0.12284560400365	0.179536033999821	1081
PSME3	0.0469537114731319	0.122870687336919	0.179559666042963	1081

SLC15A1	0.0469478291558792	0.122917654870091	0.179615273274189	1081
SLC39A4	0.0469314839646241	0.123048237043859	0.179793046728696	1081
HCRT	0.0469301473304156	0.123058920234227	0.179794952588349	1081
ALK	0.0469290875101714	0.123067391473852	0.179794952588349	1081
C19orf69	0.0469264850836498	0.12308819483528	0.179812304927971	1081
TUBA3C	0.0469120157442725	0.123203910326622	0.179942792942567	1081
HAPLN1	0.0469091657300053	0.123226712711636	0.179949411205212	1081
MOSPD3	0.0469030859633919	0.123275366686675	0.180007412431362	1081
PPARD	0.0468992777442878	0.123305849991537	0.180038874253788	1081
C5orf52	0.046893917709008	0.123348764935623	0.180088481637058	1081
GGTLC1	0.0468830201070024	0.123436052129643	0.18020286036319	1081
HABP4	0.0468692285205515	0.123546588477805	0.180351161223579	1081
SPANXN5	0.0468408254727536	0.12377447536329	0.180618385091886	1081
IGF2BP1	0.0468040947457448	0.124069663551408	0.181009803899414	1081
AP3S1	0.0468002576828603	0.124100531903058	0.18104172751011	1081
ZSCAN5B	0.046790215515475	0.124181347320024	0.181133389454918	1081
FAM38A	0.0467835684001738	0.124234863240869	0.181198328988366	1081
NAAA	0.0467661051242005	0.124375545674889	0.181377252614785	1081
ZSCAN10	0.0467632591711569	0.12439848414804	0.181397572433026	1081
PSAT1	0.0467589508273618	0.124433215808091	0.181425056233075	1081
FAM90A1	0.0467417258660246	0.124572150092329	0.18159823168174	1081
IRF2	0.0467398979934204	0.124586900561534	0.181606592750514	1081
REXO1	0.0467239447074546	0.124715697293574	0.181768030818915	1081
HIRIP3	0.0467203247003264	0.124744937368209	0.181797494269158	1081
MS4A6E	0.0467161118242469	0.124778972975797	0.181829535267647	1081
STK24	0.0467152805323932	0.124785689793759	0.181829535267647	1081
TBC1D22I	0.0466874960244243	0.125010349949592	0.182131590347475	1081
CRELD1	0.0466813457557584	0.125060122373326	0.182182152502502	1081
SPATA8	0.0466809727414083	0.125063141571462	0.182182152502502	1081
CPNE5	0.0466787313748351	0.125081284507509	0.182195406826013	1081
SERPINA1	0.0466576727305221	0.125251845607125	0.182417468894993	1081
ZMIZ2	0.0466429071306856	0.125371545134757	0.182565402395524	1081
NMU	0.0466263980541497	0.12550548381568	0.182747233210472	1081

SERPINA:	0.0465901024170723	0.125800343016842	0.183110398126755	1081
ALS2CR1:	0.0465842020203377	0.125848327661303	0.183167008217393	1081
LCAT	0.0465825399227774	0.125861847146339	0.183169082423477	1081
KRTAP9-4	0.0465817911716129	0.125867937849997	0.183169082423477	1081
XPOT	0.0465421685152088	0.126190574914047	0.18354578567823	1081
NOB1	0.0465394234133501	0.126212951372188	0.183565078723032	1081
MBD3L1	0.0465339474040231	0.12625759780172	0.183603501603671	1081
CCL3L3	0.0465316211220272	0.12627656791871	0.183604580348062	1081
ZCCHC16	0.0465277946674437	0.126307776305023	0.183636702776032	1081
DEDD	0.0465177228840535	0.126389949975086	0.183742912769582	1081
KLRG1	0.0464955398102389	0.12657108385072	0.183953140767098	1081
FAM58B	0.0464626378715964	0.126840113227159	0.184277663418514	1081
NSF	0.046458173750721	0.126876649235632	0.184317451420769	1081
HSD11B1	0.0464484213251746	0.126956495130485	0.184420146697693	1081
FDX1	0.046442528929391	0.12700475678149	0.184476950419144	1081
DDC	0.046417789783065	0.127207538302228	0.184744853226039	1081
DNAJC15	0.046412020244819	0.127254866138264	0.184800265164024	1081
NOL11	0.0464046786096402	0.127315109751203	0.184874424128738	1081
OR8G2	0.0464006264016089	0.127348370640987	0.184909393582433	1081
FASTKD5	0.0463789320320931	0.127526554820964	0.185141427419164	1081
UCN3	0.0463727162146522	0.127577643392038	0.185201222197028	1081
HOXB6	0.0463698905351624	0.127600873256316	0.185209279612975	1081
CACNA1A	0.0463664002091314	0.127629571704271	0.185224243352967	1081
DHRS4L1	0.046328489416633	0.127941608014731	0.185646190962256	1081
TCAP	0.0463211404972986	0.128002164021499	0.185711457991581	1081
PABPC1L	0.0463174339465418	0.128032714901406	0.185742405421924	1081
EFNA2	0.0462503970591582	0.128586237573507	0.186438013237246	1081
TCHHL1	0.0462246845699418	0.128799037363176	0.186733113183244	1081
UQCC	0.0462162486662824	0.12886891346242	0.186807531244185	1081
LOC1487(	0.0462082971147824	0.128934804511267	0.186889598278851	1081
DHX38	0.0462020078021367	0.128986939820595	0.18695171614403	1081
ALG12	0.0461793952110946	0.129174522200007	0.187196658493571	1081
C17orf108	0.0461691538923531	0.129259548481174	0.187306402255305	1081

RARA	0.0461628244659321	0.129312118842704	0.187369102645923	1081
HEXIM1	0.0461587749396155	0.129345761719478	0.187404370673108	1081
ZDHHC5	0.0461132162862498	0.129724724339627	0.187912890761615	1081
TBC1D29	0.0461058765431144	0.129785857678726	0.187987928022572	1081
SLC22A9	0.0460991693815619	0.129841741720593	0.188055351711939	1081
SPANXN2	0.0460972453520873	0.129857776168698	0.18806042813528	1081
LOC1513(	0.0460965084944423	0.129863917388428	0.18806042813528	1081
NUDT9	0.0460914050982832	0.129906456990122	0.188108508917912	1081
GGH	0.0460818511875803	0.12998612310606	0.188210339249018	1081
BRCA1	0.0460802389616374	0.129999570526038	0.188216282035866	1081
KRTAP2-1	0.0460736991830847	0.130054129242301	0.188281741544101	1081
MAST2	0.0460638329943542	0.130136472428971	0.188387412840013	1081
LOC36003	0.0460477971533575	0.1302703937174	0.188554180301554	1081
C11orf94	0.0460430873606693	0.130309747235472	0.188597590204654	1081
KRTAP9-9	0.046034010246296	0.130385618679523	0.188693842550214	1081
PFKFB1	0.0460285814708573	0.130431011683248	0.188733854571131	1081
SLC4A5	0.0460284627947267	0.130432004136971	0.188733854571131	1081
SLC26A1(	0.0459920000351295	0.130737208681797	0.18910756579987	1081
DBNDD2	0.0459594832887794	0.131009850251595	0.189474724195447	1081
PRB4	0.0459513568663539	0.131078056190709	0.189559758934805	1081
GPC2	0.0459471428934448	0.131113435344167	0.189597312110276	1081
ST7	0.0459384375325014	0.131186546154493	0.189685389469756	1081
NEUROG1	0.0459376482917554	0.131193176047759	0.189685389469756	1081
PF4	0.0459219542902584	0.131325064927422	0.189862453922989	1081
C2orf27A	0.0459161024597021	0.131374268668313	0.189919959980134	1081
ELMO1	0.0459089310375798	0.131434587345591	0.189993524833304	1081
VASN	0.045905325945691	0.131464917792563	0.190023733151409	1081
NPPB	0.0458987241939237	0.131520473856717	0.19007675915371	1081
FAM9A	0.0458883048949071	0.131608192897379	0.190162605502632	1081
DCAF4	0.0458812087917405	0.131667960200922	0.190232686089558	1081
ACTN3	0.0458803041882555	0.131675580780978	0.190232686089558	1081
CST5	0.0458783622808632	0.131691940991782	0.190242678471096	1081
ECHDC3	0.04582997309319	0.132100119182864	0.190745894061561	1081

CSNK1G2	0.0457971914447519	0.132377198512825	0.191068185429594	1081
PITX3	0.0457934995625975	0.132408431439127	0.191099573817253	1081
SYN1	0.0457794270057751	0.132527536029915	0.191257770135585	1081
TRIM28	0.0457780962112215	0.132538803639006	0.191260329463307	1081
SLC30A3	0.0457473522856383	0.13279931349456	0.191595084892066	1081
RCN3	0.0457416504991278	0.132847671297961	0.191637403386854	1081
MGAT4B	0.0457241642637174	0.132996059884584	0.191823986996129	1081
OR7E24	0.0457196373438483	0.133034496300394	0.191851952750478	1081
BAI2	0.0457079475923669	0.133133789409024	0.19195392124569	1081
CTHRC1	0.0456677294911369	0.133475840813008	0.192419552	1081
COCH	0.0456644917502149	0.133503407022438	0.192445519460967	1081
B3GALN1	0.0456492188042275	0.133633500601537	0.192619266572551	1081
PPY	0.0456003988920567	0.134050001211341	0.193122883101084	1081
CMTM2	0.0455489526056926	0.134489992605065	0.193715208138784	1081
SLC6A18	0.0455378603641913	0.134585004313379	0.193838200607207	1081
XAGE3	0.0455337497664655	0.134620227207369	0.193861210743616	1081
GGNBP1	0.0455091575734776	0.134831101473003	0.19413712666526	1081
OGG1	0.0454865496595517	0.135025185689162	0.194402684799041	1081
RNF122	0.0454848008735819	0.135040207629908	0.194410418259452	1081
NYX	0.045441750056932	0.135410417722347	0.1948876805756	1081
C10orf76	0.0454388878722359	0.135435058449664	0.194909219350513	1081
BUB3	0.0454294265044135	0.135516536573594	0.195007601054671	1081
CGB	0.045412087566292	0.135665951818606	0.195185730566049	1081
GAST	0.0453876092151802	0.135877105957606	0.195447647234272	1081
LOC40264	0.0453623658433351	0.136095124620792	0.19573329660069	1081
SLC22A14	0.045309837633651	0.136549657805193	0.196316932378362	1081
PALM3	0.0453025740297509	0.136612602644938	0.196393411852014	1081
DNASE2E	0.0452988070020911	0.136645255705753	0.196426336248922	1081
MAGEB18	0.0452846189266296	0.13676829368147	0.19658382529257	1081
ATOH7	0.0452446752232328	0.137115141073168	0.19697529180054	1081
OR10C1	0.0452292119744623	0.137249596511695	0.19715438688877	1081
LOC10013	0.0452228563606066	0.137304889034557	0.197205688692511	1081
FCER2	0.0452151986475471	0.137371532335813	0.197287340051682	1081

C17orf76	0.0452140536752543	0.137381498904546	0.19728758878187	1081
SPAG16	0.045196380679704	0.137535406565041	0.197487137334497	1081
LCE1E	0.0451950233748319	0.137547232324717	0.197487137334497	1081
INCENP	0.0451820014367692	0.137660728063187	0.197584736090429	1081
C1orf172	0.045172508748814	0.137743509226882	0.197652467652649	1081
ALOX12B	0.0451478254972817	0.137958939364445	0.19793339744297	1081
PRRG2	0.045141862769859	0.138011019644006	0.197994017285893	1081
TMPRSS1	0.0451365439180811	0.138057488885493	0.198046579334776	1081
MPZ	0.0451243291447723	0.138164251343771	0.198185619718049	1081
MURC	0.04511550304989	0.1382414348046	0.198282214605814	1081
LRRC10	0.0451127130742699	0.138265839808341	0.198303100024138	1081
KLRG2	0.0451050462409081	0.138332921659863	0.19838518593564	1081
GSC	0.0450831309704434	0.1385248099721	0.198617957073876	1081
PRDM9	0.0450773423227607	0.138575529069063	0.198662399167113	1081
C2orf69	0.0450571583196836	0.138752489452111	0.198877212171984	1081
OR6V1	0.0450569342897922	0.138754454577262	0.198877212171984	1081
PCSK1	0.0450568735700739	0.138754987196686	0.198877212171984	1081
CEACAM	0.045051605237748	0.138801205785603	0.198929303539483	1081
TRIM8	0.0450356816005303	0.138940974355416	0.199115453396163	1081
BCAR4	0.0450268616819066	0.139018437115862	0.199212293050153	1081
ACSL5	0.0450162452611151	0.139111722069607	0.199331790389944	1081
SOX18	0.0449636987433414	0.139574149558174	0.199937512824391	1081
COMMD8	0.0449346833330908	0.139830001013531	0.200232824169575	1081
FAF1	0.0449293330919998	0.139877217582892	0.20026557117988	1081
C1orf106	0.0449191446305549	0.139967165949992	0.200372268196603	1081
SNAPIN	0.0449142284331508	0.140010584267539	0.200408317168517	1081
RPLP0P2	0.044914040524606	0.140012244022323	0.200408317168517	1081
S100A7A	0.0448943001170241	0.140186690919964	0.200629503309368	1081
C7orf53	0.0448868048987633	0.140252970227763	0.20069584353163	1081
KCNJ4	0.0448680213390814	0.140419176721858	0.200909103419964	1081
OR11G2	0.0448666799556268	0.140431051750013	0.200909103419964	1081
XPA	0.0448665815641707	0.140431922822363	0.200909103419964	1081
BMP1	0.0448446083879419	0.140626558173398	0.201173271865012	1081

TMEM40	0.0448408338698241	0.140660013170245	0.201192556469369	1081
MYO9B	0.0448361744613894	0.140701319716447	0.201237350776559	1081
CEP70	0.0448288222419374	0.140766517490224	0.201315475188409	1081
OR51Q1	0.0448227942771281	0.14081998938511	0.201364188398853	1081
C18orf26	0.0448051650420664	0.140976461449712	0.201559319794496	1081
LASS5	0.0447698270983093	0.141290512837903	0.201979660879901	1081
HSPA9	0.0447602461300383	0.141375752190208	0.202087172913627	1081
UPRT	0.044757087305816	0.14140386406625	0.202113015582135	1081
TCF7	0.0447381159306188	0.141572789591098	0.202329717928242	1081
CACNB3	0.0447378046755745	0.141575562368652	0.202329717928242	1081
ULBP2	0.0447140114816714	0.141787644490077	0.202604064282352	1081
RP9	0.0446939664719701	0.141966506016656	0.202830869766349	1081
GPR4	0.0446917056772018	0.141986689932822	0.202840678871636	1081
FOXS1	0.0446376038520867	0.142470356849062	0.203393084251149	1081
ST8SIA5	0.0446296827740024	0.142541276670257	0.203478884532478	1081
ALG5	0.044605440805425	0.142758490549616	0.203774519387942	1081
FAM177A	0.0445869193548255	0.142924618107895	0.203997196527104	1081
SYF2	0.0445679138637374	0.143095241223814	0.204226258197218	1081
BPESC1	0.0445660486416076	0.143111994791846	0.204235699566573	1081
GPR128	0.044564564529339	0.143125326275591	0.204240256274123	1081
CCRL1	0.0445598897126888	0.143167325438169	0.20428571829947	1081
CHST1	0.0445577377396314	0.143186662218495	0.204298839180649	1081
IL7R	0.0445339894063122	0.143400188276809	0.204545549305127	1081
CRYGC	0.0444998933743542	0.143707178973343	0.204881891041613	1081
STK40	0.0444892274351619	0.143803315246567	0.204954043142137	1081
C6orf27	0.0444888374744003	0.143806831048118	0.204954043142137	1081
ANAPC2	0.0444886349238558	0.143808657225818	0.204954043142137	1081
MS4A15	0.0444842070253057	0.143848583203028	0.204996442319256	1081
CACNA2I	0.0444829735946296	0.143859706451856	0.204997792068519	1081
PRPSAP2	0.0444366540469289	0.144277899509315	0.205535555166901	1081
IKZF3	0.0444218942114116	0.144411353052282	0.20568203529682	1081
MCM8	0.0444140390221135	0.144482415581371	0.20575461911907	1081
PLA2G12I	0.0444091095317025	0.144527024232264	0.205803131629247	1081

RPL36	0.0444008896454991	0.144601432244666	0.205894532977721	1081
LOC29034	0.0443984081453071	0.144623901027976	0.20591197160284	1081
ZNF775	0.0443955689297973	0.14464961202733	0.205934023624775	1081
CTSF	0.0443894186875218	0.144705318587347	0.205983096905238	1081
APOL4	0.0443882938859263	0.144715508388632	0.205983096905238	1081
LOC10027	0.0443872482435211	0.144724981563502	0.205983096905238	1081
CPNE6	0.0443639949460367	0.144935771383763	0.206239394965452	1081
IL17A	0.0443454579099376	0.145103976743875	0.206449580585166	1081
C3orf47	0.0443409393061692	0.145145001252497	0.206478783294369	1081
ATP6V1G	0.0443349222186255	0.145199644301796	0.206541931539308	1081
PIGF	0.0443263582878606	0.145277443160103	0.206638006951936	1081
OR51V1	0.0442999401726778	0.14551763886526	0.206965040591688	1081
CHKA	0.0442841729070401	0.1456611407889	0.207154513321029	1081
OR5AS1	0.0442777296668297	0.145719813498712	0.207208699296362	1081
ARAP1	0.0442611938349498	0.145870472915613	0.207393653531303	1081
TCEAL8	0.0442536317883519	0.145939411049682	0.207462383430657	1081
SILV	0.0442446850929058	0.146021004259889	0.207563726419381	1081
STX3	0.0442240946407777	0.146208920134173	0.20778685686793	1081
POU6F2	0.0442100038438825	0.146337624282716	0.207955096019703	1081
ENGASE	0.044142582453234	0.146954643887315	0.208655293283758	1081
MSL3L2	0.0441076237413088	0.147275356315279	0.209051723010942	1081
UBE2I	0.044099125279974	0.147353402173841	0.209119026054186	1081
FAM46C	0.0440990696808797	0.147353912873625	0.209119026054186	1081
FKBP15	0.0440930476405847	0.14740923570694	0.209182801726987	1081
RAET1E	0.0440862880277327	0.147471353302946	0.209256209923776	1081
TRIM64	0.044075207853179	0.147573218002568	0.209386003427365	1081
EXTL1	0.0440592137609707	0.147720353111614	0.209580006580285	1081
SPRR2C	0.0440356390456465	0.147937429044817	0.20985842603393	1081
TECRL	0.0439610109766544	0.148626209067539	0.210731628777138	1081
ITM2C	0.043901564054848	0.149176622326573	0.211422756209991	1081
HYAL2	0.0438931014897893	0.149255102553524	0.211519102422846	1081
C22orf40	0.043883523221973	0.149343967555501	0.211630150985198	1081
OR4D1	0.0438705460564722	0.149464431075904	0.211771062317069	1081

TBR1	0.0438550543786636	0.149608333069779	0.211945138515521	1081
THG1L	0.0438321949095684	0.149820867151222	0.21222887232647	1081
WIPI2	0.0438312469290073	0.14982968588866	0.21222887232647	1081
SLC27A2	0.0438227077020098	0.149909141207396	0.212326488759262	1081
PRDM7	0.0438064850827715	0.150060176919901	0.212468918080627	1081
RFWD3	0.0438062425673625	0.150062435663994	0.212468918080627	1081
ZNF580	0.0437879782657891	0.150232620442216	0.212694928583066	1081
BAAT	0.0437654117321843	0.150443095881744	0.212962979870545	1081
STX16	0.0437579618684518	0.150512629164737	0.213031470141476	1081
MAPK3	0.0437541088519727	0.150548600881458	0.213067413570111	1081
CCDC70	0.0437328780630388	0.150746928675319	0.213303145196621	1081
LOC44230	0.0437184775289018	0.150881564670027	0.213478657134266	1081
PRAMEF1	0.0437143817392887	0.150919874454139	0.213517864378021	1081
NOTCH2N	0.043712100239452	0.150941217570349	0.213520476922966	1081
C20orf71	0.0437119183278508	0.150942919427151	0.213520476922966	1081
LCE1A	0.0436445300530383	0.151574369273866	0.214323413281536	1081
EIF4EBP3	0.0436266090495398	0.151742632583438	0.214531218569258	1081
REG1P	0.0436219991857336	0.15178593832959	0.214577384396462	1081
CLPB	0.0435787699751961	0.152192497384726	0.215076665016857	1081
TP73	0.0435410599302588	0.152547825748448	0.215548569103071	1081
MARK4	0.0435358358863269	0.152597099737379	0.215603070150757	1081
KRTAP8-1	0.0435281404949518	0.152669705886401	0.215683870142116	1081
RNMTL1	0.0435088661540309	0.152851674833872	0.21588704967597	1081
GZF1	0.0434825719253646	0.153100184178258	0.216207725921556	1081
DNASE1	0.0434634436879269	0.153281159783706	0.216446140258738	1081
TNFSF12	0.0434512714165591	0.153396408336531	0.216580505042011	1081
IFNA17	0.0434229213526828	0.153665085300225	0.216944645271079	1081
SH3GLB2	0.0434125884932813	0.153763099841651	0.217022184986341	1081
C17orf98	0.0434109651245954	0.153778502964496	0.217028719505379	1081
WBSCR26	0.0433887814020692	0.153989107721296	0.217310723295657	1081
CLCF1	0.0433660204019415	0.154205420420434	0.217585500761339	1081
RPS4Y1	0.0433606474492159	0.154256516735743	0.217642353946038	1081
SLC9A11	0.0433510799731961	0.154347534410156	0.217755520743231	1081

DPPA2	0.0433420538270052	0.15443343963536	0.217861459485597	1081
FBRS	0.0433124764031822	0.15471519329453	0.21821309031144	1081
TRIB1	0.0432997502709098	0.154836541957443	0.218353667324654	1081
RPL13P5	0.0432941953894156	0.154889532557159	0.218413106073356	1081
MMP7	0.0432678373562006	0.155141161585124	0.218737311039496	1081
RPA4	0.0432631003048747	0.155186417056739	0.218779722219425	1081
RABL5	0.0432533152668982	0.155279930215052	0.218887005400757	1081
DNAJC5	0.0432347467900617	0.155457501946351	0.21912198269726	1081
IL12A	0.0432204981491832	0.155593867109697	0.219298849291601	1081
IGFL1	0.0432169182696506	0.155628142227948	0.219331812311601	1081
FAM194A	0.0432104743972609	0.155689852715792	0.219388331282714	1081
ADIPOR1	0.0431976287062758	0.155812926289042	0.219510269402299	1081
PPP1R8	0.0431968753359442	0.155820146560083	0.219510269402299	1081
FAAH	0.0431879984164195	0.155905241696258	0.219592763591709	1081
REN	0.0431862185647341	0.155922307789318	0.219592763591709	1081
HDX	0.0431831570293748	0.155951666607095	0.219618757343581	1081
C14orf176	0.0431813167927154	0.155969315706864	0.219628258417083	1081
DNAJA4	0.0431713701135025	0.156064737207744	0.219747265747536	1081
TM4SF5	0.0431456994355243	0.156311208150484	0.22006354659246	1081
CAMK2B	0.0431398678554315	0.156367239762766	0.220127047171273	1081
WNT3	0.0431348934544249	0.156415047357296	0.220163578047284	1081
LOC80154	0.043122831172401	0.156531020588475	0.220311423863259	1081
PSTK	0.0431168850476426	0.156588213769758	0.220361129332293	1081
GRIN2D	0.0430218216944618	0.157504736948732	0.22149619028497	1081
FOXF1	0.0430178754349411	0.157542871122781	0.221518890121339	1081
C13orf27	0.0429960100752012	0.157754290831109	0.221772535965306	1081
C2orf64	0.0429958035350186	0.15775628892444	0.221772535965306	1081
OR10K1	0.0429616178138163	0.158087269403053	0.222206813	1081
RCN1	0.0429019188721517	0.158666523620887	0.222912136016652	1081
CBWD6	0.0428925095347794	0.158757967874198	0.223025053195657	1081
NCRNA0(	0.0428867602169755	0.158813861982843	0.223055127512998	1081
TFAP2C	0.0428859399617502	0.158821837608081	0.223055127512998	1081
SP6	0.0428857519375837	0.15882366587473	0.223055127512998	1081

OR9I1	0.0428745809882078	0.158932315961925	0.223176600101281	1081
FBXO22	0.0428542725579324	0.159129982230751	0.223407449441667	1081
TAF6	0.0428401282882698	0.159267761117827	0.223585299492588	1081
GNRH2	0.0428180027048578	0.15948346646799	0.223856914158142	1081
BANK1	0.042811992953665	0.159542094451072	0.22392360431386	1081
OR51A4	0.0428075401879476	0.159585543798093	0.223968982848863	1081
NKX2-8	0.0427832456360341	0.159822763417989	0.224249752661321	1081
PDE1B	0.0427696653671308	0.159955481395154	0.224378746097443	1081
IFNA1	0.0427581163755188	0.160068413459437	0.224521528278816	1081
C10orf116	0.0427378391850264	0.160266840021177	0.224784201923456	1081
PDYN	0.0426685400807037	0.160946382210258	0.225676447626711	1081
CHST7	0.0426388615630731	0.161238071607773	0.226051983613236	1081
GRM4	0.0426174468775132	0.161448789094926	0.226299414167614	1081
AG2	0.0426034528239513	0.161586600758233	0.226446057201712	1081
KRTAP4-[illegible]	0.0425955357872916	0.161664606004714	0.226539613798341	1081
C3orf62	0.0425903252618109	0.161715959935771	0.226580053756163	1081
DPRXP4	0.042569800969952	0.161918363023245	0.22681631479753	1081
GJA10	0.0425594288944838	0.16202072125744	0.226943918073364	1081
SPANXN[illegible]	0.0425372837396559	0.162239426937713	0.227187074632297	1081
C19orf45	0.0425339068363995	0.162272796793973	0.227218008717216	1081
DRD3	0.0425291150442994	0.162320157164074	0.227268526971801	1081
SNHG10	0.0425005952260922	0.162602252408612	0.227620315048365	1081
RPTOR	0.0425002863886938	0.162605309201341	0.227620315048365	1081
EMB	0.0424957779280132	0.162649937708493	0.227651723752978	1081
WDR41	0.0424897773195391	0.162709351052353	0.227707254719072	1081
ATG2A	0.0424894461999148	0.162712630016433	0.227707254719072	1081
RASGRP1	0.04245015992085	0.163102021957926	0.228204627888764	1081
TMOD1	0.0424412059023933	0.163190869186866	0.228313081447976	1081
LCE2D	0.0424336879466571	0.163265495065383	0.22840162486751	1081
WDR72	0.0424280307055016	0.163321667774578	0.228448479191701	1081
SLC39A8	0.0424137346760975	0.163463682849782	0.228614279809203	1081
TPK1	0.0424126716293014	0.163474246743686	0.228614279809203	1081
PSG1	0.0424098938762417	0.163501852741391	0.228637013985515	1081

CXXC5	0.0424070193771293	0.163530423919831	0.228661094597418	1081
LBP	0.0423869134669244	0.163730372359303	0.22892478839382	1081
SCARNA1	0.0423834033729675	0.163765298255822	0.228957730124474	1081
LTV1	0.0423765840199713	0.163833167742187	0.229036722010157	1081
RAB25	0.0423535186282229	0.164062882507966	0.229326031648832	1081
KCNE1L	0.0423484580894094	0.164113314247534	0.229379670819983	1081
AACSL	0.0423473830207603	0.164124029545755	0.229379670819983	1081
ANP32C	0.0423438984096513	0.164158764571135	0.229412300540097	1081
TRIM40	0.0423217465930164	0.164379705900188	0.229705131476088	1081
TMEM109	0.0423173820108846	0.164423264431275	0.229750063256436	1081
PIGY	0.0422985824713328	0.164610983077258	0.229970220959711	1081
NUDT16L	0.0422981733504949	0.16461507005406	0.229970220959711	1081
ZNF639	0.042294236074456	0.164654405991838	0.230002636887144	1081
INPP5E	0.0422519891516561	0.165076925038861	0.230535504672988	1081
SAP18	0.0422393383821151	0.165203606089187	0.23065603869041	1081
MRFAP1	0.0422376535100415	0.165220483410405	0.23065603869041	1081
LOC15837	0.0422280928487781	0.165316276855833	0.230757788058534	1081
FBXW4	0.0422179238453289	0.165418211415385	0.230884076003806	1081
PRPS1L1	0.0422138430842856	0.165459130430218	0.230911012904475	1081
OR4N2	0.0422034806053938	0.165563072295081	0.231022240866135	1081
KLHDC8E	0.0421947998846305	0.165650182879088	0.231111776030143	1081
FBXL7	0.0421900055410832	0.165698308628035	0.23114743654236	1081
TMEM115	0.0421839345353479	0.165759264618302	0.231208107826841	1081
AACS	0.0421830054501391	0.165768594593251	0.231208107826841	1081
KRTAP13	0.0421492084391474	0.166108256129588	0.231654608496404	1081
SPATA24	0.0421296244147541	0.166305315540535	0.231881269488101	1081
TRIM25	0.042127965563135	0.166322015386619	0.231888504392238	1081
MAGEB10	0.0421250258434544	0.166351612968753	0.231903468078548	1081
NOTUM	0.0421246128356052	0.166355771516659	0.231903468078548	1081
LEPREL2	0.0421228301377803	0.166373722275303	0.23191244362275	1081
DHRS7B	0.0421102669385353	0.166500267828657	0.232040670730425	1081
MLN	0.0420864410030222	0.166740458199092	0.232334407839095	1081
OR5B17	0.042085927515281	0.166745637554611	0.232334407839095	1081

SERPINA'	0.0420760735552757	0.166845054097553	0.232441796771161	1081
TELO2	0.0420757120035208	0.166848702636957	0.232441796771161	1081
FAM36A	0.0420748575406224	0.166857325545186	0.232441796771161	1081
FLJ36000	0.0420248605808267	0.167362458043828	0.233064891282518	1081
CYP2D7P	0.0419950426751907	0.167664261872256	0.233452899876735	1081
SLC45A1	0.041990791854436	0.167707320048037	0.233496714518468	1081
EPB41L3	0.0419895978801642	0.167719415758075	0.233497417446194	1081
PPIAL4E	0.0419729269509504	0.167888371104042	0.233700334154983	1081
CDHR5	0.0419461851036253	0.168159658997928	0.234045621840076	1081
CPA4	0.0419405792210568	0.168216570614115	0.234107972938528	1081
C13orf15	0.0419394831266653	0.168227699987994	0.234107972938528	1081
CELA3B	0.0419145213901332	0.168481302516279	0.234428501118278	1081
DBR1	0.0419110444592844	0.168516649643388	0.234461489541132	1081
ATG7	0.0418906895237369	0.168723693231542	0.234717132805015	1081
ZNF777	0.0418876408409493	0.168754719817843	0.234744084431048	1081
KCNE3	0.0418826945171402	0.168805067879735	0.234797907369831	1081
NTRK1	0.0418494814726699	0.169143431204581	0.235236066387041	1081
COL13A1	0.0418338376626509	0.169302980972972	0.235429781699037	1081
SAA3P	0.0418295770461544	0.169346454129693	0.23543715103124	1081
ACCSL	0.0418218106670183	0.169425719807878	0.235531096924278	1081
OR52E2	0.0418071043656896	0.16957589224564	0.235707331076273	1081
LOC2027ε	0.0417893700137578	0.169757117962856	0.235926674119472	1081
LRRC37B	0.0417756560816742	0.169897358766417	0.236056437847402	1081
SLC6A16	0.0417507098921231	0.170152684721831	0.236378583112977	1081
MOGAT3	0.0417431098270824	0.170230528950762	0.23647041826735	1081
PRSS54	0.0417318411845241	0.170345997906248	0.236598188625301	1081
FAM125B	0.0417247547398119	0.170418642197917	0.236682767809517	1081
MYOD1	0.041717058233067	0.170497566590628	0.236743415975201	1081
ANKRD3'	0.0417019215994638	0.170652866084584	0.236942724327931	1081
LOC1001:	0.0416585178285856	0.171098768739711	0.237529094911549	1081
PAQR6	0.0416327311865729	0.171364096507264	0.237847884950153	1081
CYP2C9	0.0416317046027298	0.171374665750334	0.237847884950153	1081
LOC3897ʢ	0.0416191912581945	0.1715035366661	0.238009007036276	1081

DNMT3B	0.0416150049641735	0.17154666613115	0.238052461886892	1081
C3orf38	0.041608487057622	0.171613833267232	0.238129265130761	1081
GHRL	0.0416016161413393	0.171684659455426	0.238211134701396	1081
KLHDC3	0.0415974942606128	0.171727158754869	0.238253692363418	1081
HIST1H4F	0.0415960002982272	0.171742564430225	0.238258657148054	1081
GDAP1	0.0415666138895154	0.172045805821529	0.238646475127363	1081
NDNL2	0.0415645726578395	0.172066884352519	0.238659280176363	1081
ZBBX	0.0415243046449476	0.172483101679538	0.239170710581931	1081
NRAP	0.0415182242411699	0.172546015103152	0.239241480780026	1081
PNLIPRP2	0.0415150941517432	0.17257840855522	0.239253460900482	1081
IL17REL	0.0415115225124498	0.172615377176837	0.239288244785809	1081
LRTM1	0.0414937278490064	0.172799650818229	0.239510731094896	1081
CABP5	0.0414849821895521	0.172890270807096	0.239619849013343	1081
SPPL2A	0.0414402121268442	0.173354721021515	0.240213980945001	1081
PSORS1C	0.0413983893761526	0.173789436124215	0.24073356190073	1081
HPDL	0.0413954462388677	0.173820058359237	0.240738820593302	1081
C2orf68	0.0413948365633017	0.173826402307162	0.240738820593302	1081
CUL1	0.0413772159157863	0.174009827700348	0.240972570222281	1081
RPS29	0.0413747736549066	0.174035262255038	0.240991226156705	1081
XRCC4	0.0413661153739214	0.174125454947899	0.241099545668116	1081
RAD21	0.0413562082097594	0.174228699929185	0.241225921654531	1081
SPRR2G	0.0413431959488674	0.17436437323381	0.241397175369054	1081
RAB17	0.0413326755631785	0.174474122399719	0.24149460580353	1081
QSOX2	0.0413148168369507	0.174660543398222	0.241690936032229	1081
SRD5A3	0.0412966556158409	0.174850274278849	0.241920245559575	1081
NPSR1	0.0412871963910199	0.174949155938465	0.242023811727811	1081
MRPL42	0.0412749001527799	0.17507775650726	0.242112020729331	1081
CCL27	0.0412748329389188	0.175078459658937	0.242112020729331	1081
IGSF5	0.0412742049474754	0.175085029435835	0.242112020729331	1081
SH2D3C	0.0412655543716726	0.175175546742755	0.242203938856061	1081
OSTC	0.0412603124633555	0.175230413631181	0.242263172232527	1081
OR6C68	0.0412432828711706	0.175408750206183	0.242476447739213	1081
ZFP57	0.0412026745684226	0.175834552352125	0.242998357490125	1081

C2orf80	0.0411868978971039	0.176000187573645	0.243177213900622	1081
LOC10012	0.0411800976129685	0.176071617831837	0.243225861703515	1081
SLC25A2(	0.0411720932080338	0.176155723842251	0.243325360449956	1081
CYP2J2	0.0411511856311203	0.176375550587445	0.243578903584292	1081
C1orf223	0.0411225545296068	0.17667691532354	0.243961646390617	1081
RAG2	0.0411194245263693	0.176709884277518	0.243990446797163	1081
GMPR2	0.0410938539213675	0.17697939610224	0.244345825130534	1081
PFDN5	0.0410813082572063	0.177111738023901	0.244495029294284	1081
LRRC39	0.0410780914620421	0.177145683264219	0.244525132887329	1081
FABP5L3	0.0410620894839453	0.177314616104955	0.244741551314445	1081
CHRNB3	0.041055972495936	0.177379224777973	0.244813954724053	1081
DMKN	0.0409816512199919	0.178165617994517	0.245781440423169	1081
PGBD5	0.0409556744149218	0.178441088716399	0.24612774612748	1081
LOC64567	0.0409111442980649	0.178914043861664	0.246661881576322	1081
LOC1001(	0.0408750092798905	0.179298518331956	0.247158112207284	1081
AKNAD1	0.0408687332635451	0.179365357256037	0.247216415019353	1081
OR52A4	0.0408654592847401	0.179400232132632	0.247247566279802	1081
TMOD4	0.0408411522805323	0.179659311162625	0.247553818288035	1081
C9	0.0408393809490863	0.179678201955465	0.247562914875375	1081
PWP1	0.0408314234538264	0.179763084778372	0.247631036021608	1081
KRTAP26	0.0408093644746693	0.179998544121467	0.247919504398123	1081
NECAP1	0.0408070810603295	0.180022930537223	0.247931302769524	1081
CCDC155	0.0408051313437632	0.180043755066705	0.247931302769524	1081
MGC3403	0.0408051054001287	0.180044032177482	0.247931302769524	1081
PAGE2	0.040789522902665	0.180210530113841	0.248143617849851	1081
C17orf91	0.0407789621481232	0.180323435991148	0.248282114554143	1081
ANKRD4:	0.0407742756960293	0.180373556034706	0.248334150240511	1081
CYP4B1	0.0407729085999703	0.180388178618258	0.248337310070717	1081
OR5R1	0.0407671791637252	0.180449470753522	0.248369312610998	1081
STAG3L4	0.0407664032145603	0.180457772859217	0.248369312610998	1081
PSG9	0.0407661258004984	0.180460741061662	0.248369312610998	1081
RND1	0.0407193316180664	0.18096193560966	0.249025083192609	1081
OR7A10	0.0407036223416255	0.181130422822208	0.249222892408537	1081

PITPNM1	0.040637417580813	0.181841768765182	0.250082088460854	1081
TMIE	0.040620787422317	0.18202077854223	0.250294101278719	1081
SSX7	0.0405656530910322	0.182615187349078	0.251042919963639	1081
NPDC1	0.0405608744949745	0.182666773474098	0.251088389108068	1081
P2RX7	0.0405602800967303	0.182673190904336	0.251088389108068	1081
ALPK2	0.040556255322572	0.18271664883493	0.251130987977053	1081
BCAT2	0.040535307136533	0.182942962711308	0.251390585526555	1081
TTC13	0.040525113055913	0.183053169551602	0.251507713860116	1081
OC90	0.0405221607253842	0.18308509597501	0.251534423952573	1081
NPM1	0.0405142420024948	0.183170749181749	0.251617779902239	1081
IQCC	0.0405050491652734	0.183270221089851	0.251737256501776	1081
C20orf196	0.0405024795278926	0.183298033224954	0.251758292719486	1081
WHAMM	0.0404822273769341	0.183517339044011	0.252042323087102	1081
IL7	0.0404797067470917	0.183544647933853	0.252062644531151	1081
CECR6	0.0404662918933793	0.183690037088871	0.252227918830026	1081
NCDN	0.0404416843752561	0.183956952894051	0.252508368496229	1081
C22orf28	0.0404360977816974	0.184017590161559	0.252574392164926	1081
PCNXL3	0.0404247096753804	0.18414124321418	0.252726893619679	1081
RGS4	0.0404217956529864	0.184172893792774	0.252753112981499	1081
MOCS3	0.0403899833710263	0.184518683741059	0.253158679014073	1081
PPIAL4D	0.0403868840200366	0.184552398391239	0.25318769174554	1081
PRNT	0.0403589145278178	0.184856855679455	0.253570840096869	1081
ZNF282	0.0403523180006972	0.18492871514227	0.253652139071358	1081
TRIM62	0.0403500986979422	0.184952895821699	0.2536680341999	1081
RBM28	0.0403408399369077	0.18505380096633	0.253765713180378	1081
CBFB	0.0403398635672635	0.185064444146796	0.253765713180378	1081
C4orf11	0.0403268882507257	0.185205928022804	0.253928634044741	1081
RARRES2	0.0403243298325422	0.185233834644649	0.253949612012825	1081
HTR6	0.0402705159801421	0.185821543422916	0.254686011175305	1081
C1orf88	0.040268329693477	0.185845449239995	0.254701447373271	1081
FBXO47	0.0402618907015151	0.185915869192465	0.254780624821919	1081
TMPRSS1	0.0402207199660494	0.1863665981031	0.255328830167774	1081
CHST5	0.0402190442566626	0.186384960494828	0.255336622180776	1081

SMCP	0.0402147630496772	0.186431879942544	0.255383531989837	1081
STIM1	0.0401755075688875	0.186862502744174	0.255938612848883	1081
OR8A1	0.0401603498264954	0.187028975581187	0.256096976147567	1081
CALY	0.0401412226161288	0.187239199931359	0.256349985226127	1081
RAB13	0.0401329931260111	0.187329702635851	0.256427744835115	1081
HSPC157	0.0401325857797711	0.187334183208657	0.256427744835115	1081
DPYS	0.0401270494688621	0.187395087258498	0.256493683436775	1081
SORD	0.0401129738899511	0.187549996134327	0.256670833704214	1081
ADAM18	0.0400738143093623	0.187981463764351	0.257191428112799	1081
TTTY15	0.0400581235010607	0.188154553153051	0.257393282172226	1081
SLC41A1	0.0400451147575469	0.188298144999475	0.25755473458816	1081
MYH3	0.0400403394100378	0.188350876027337	0.257609369106573	1081
PTH	0.0400193273687372	0.188583027448754	0.257909374606596	1081
RGS11	0.0400116838223241	0.188667529382937	0.258007425118408	1081
MED11	0.0399996926884686	0.188800151517829	0.25815374006154	1081
MAGED2	0.0399953091058101	0.188848651144367	0.258202530019226	1081
TPD52	0.0399541129269589	0.189304890843709	0.258738518627214	1081
TSPYL2	0.0399472568871922	0.189380898835043	0.258824844439074	1081
SGPP2	0.0399420839810775	0.189438261997117	0.258868117482833	1081
ADAM3A	0.039926975358645	0.189605877082226	0.259055584518085	1081
HNRNPCI	0.0399237936910051	0.189641188415422	0.259057553443323	1081
C5orf27	0.0399099544139084	0.189794838172228	0.259232287950338	1081
KCNQ2	0.0398492043380713	0.190470395860363	0.259978733288638	1081
SKP1	0.0398181393986543	0.190816529074096	0.260398250792418	1081
GDPD4	0.0398168892621568	0.190830468074892	0.260399632823187	1081
SEC24D	0.0398129685883349	0.190874188376277	0.260424011164246	1081
OR9G9	0.0398062081372908	0.190949592954392	0.260509247600314	1081
GRPEL2	0.03976035267527	0.191461632639979	0.261137074443628	1081
HIST2H2I	0.0397443250421729	0.191640841040881	0.261363803585983	1081
NKX2-1	0.0397402190662916	0.191686770656775	0.261408745930187	1081
CACNG1	0.0397354093639843	0.191740582467881	0.261451165086912	1081
NEUROD	0.0397351188754017	0.191743832861456	0.261451165086912	1081
MESP2	0.0396959805564426	0.192182137396393	0.261995611954411	1081

LOC28595	0.0396933029471647	0.192212150429273	0.262018796210428	1081
NACA2	0.0396823724974102	0.192334704355622	0.262168118224779	1081
GPR141	0.0396766463878635	0.192398929291195	0.262218813908986	1081
LOC40075	0.0396755751685432	0.19241094600162	0.262218813908986	1081
ALKBH4	0.0396240742124382	0.19298932291343	0.262953663178291	1081
SMPD4	0.0396197808711072	0.193037596441417	0.263001648878151	1081
NIPAL4	0.0396007672376549	0.193251488677616	0.263275257957028	1081
C9orf7	0.0395951618535598	0.193314579132493	0.263325593118133	1081
STAR	0.0395903784582248	0.193368429777459	0.263363330259408	1081
FLJ43859	0.0395720739749871	0.193574600121712	0.263608485057249	1081
FOXQ1	0.039556843171682	0.193746273273673	0.263824433898753	1081
MRS2P2	0.0395546282449505	0.193771247991541	0.263840607691085	1081
CRADD	0.0395458772359178	0.19386994431042	0.263957152482659	1081
PLB1	0.0395074320737702	0.194303976193912	0.26451233953415	1081
OR8B12	0.0395018723730059	0.194366802082327	0.264579987022669	1081
FYN	0.0394730042403386	0.19469325790991	0.264988560272626	1081
BRDT	0.0394673934207209	0.194756754567362	0.265057074770943	1081
CYP2D6	0.0394413486853539	0.195051696904982	0.265440548142327	1081
KIAA1609	0.0393743438474415	0.195811993048153	0.26638523770631	1081
CACNA1H	0.0393630272574545	0.195940614894681	0.266542216696154	1081
FLJ23867	0.0393461195570428	0.196132899238372	0.266770794873623	1081
RDH8	0.0393459232618559	0.196135132433519	0.266770794873623	1081
C5orf48	0.0393381150805864	0.196223979005985	0.26685560366405	1081
RHCG	0.0393263294849803	0.196358138948402	0.267020028966893	1081
TMEM41A	0.0393116889302718	0.19652489141937	0.267210713933802	1081
ADAM28	0.0393090904616605	0.196554498185087	0.26723293284326	1081
FLJ39582	0.0392840976177669	0.196839431468803	0.267566150265792	1081
AMHR2	0.0392724644595969	0.196972159354193	0.267699061463046	1081
RDH13	0.0392654181789579	0.197052585324619	0.267747493009878	1081
MARK3	0.0391965911786554	0.197839436890919	0.268599235487771	1081
OR8H1	0.0391702432063665	0.198141261848817	0.268972757408654	1081
TIMM8A	0.0391538123187036	0.198329653425571	0.269192215891263	1081
PWRN2	0.0391510010931064	0.198361899186668	0.269217844042002	1081

SLC4A10	0.0391252437304199	0.198657523586498	0.269565448976177	1081
UCK2	0.0390914655290156	0.199045692699374	0.270050591251284	1081
ZSCAN12	0.0390858791241138	0.199109943322857	0.270105710992522	1081
ITPKC	0.0390693278629791	0.199300392317344	0.270345862449188	1081
MYEOV	0.0390488641222867	0.19953604442886	0.270629072579235	1081
EHMT2	0.0390356418752224	0.199688414506845	0.270799266929679	1081
EIF3M	0.0390264887482681	0.199793942697645	0.270905901302016	1081
C5orf45	0.0390229929014884	0.19983425773096	0.270942328845753	1081
OR5A1	0.0389926993069492	0.200183859997705	0.271379802130132	1081
TEX101	0.0389743133985247	0.200396259327268	0.271631183161608	1081
COX19	0.0389587312407178	0.2005763974928	0.271857063008306	1081
LOC10024	0.038956269564038	0.200604866597553	0.271877357212575	1081
ATXN2L	0.0389505885550391	0.200670578277847	0.271938057582878	1081
LDHB	0.0389500634878531	0.200676652470939	0.271938057582878	1081
TUBB6	0.0389447492108756	0.200738137753399	0.272003079642311	1081
MATN1	0.0389167011188529	0.201062876864986	0.272424781708713	1081
C14orf23	0.0389098758609831	0.201141957154298	0.272513600569866	1081
ANKS4B	0.0388909792504331	0.201361019382557	0.272773702875503	1081
CAND2	0.0388480846300385	0.201858927505771	0.273356284928997	1081
CDCA7L	0.0388279745657492	0.202092667587065	0.273599246541762	1081
NPTXR	0.0388223267868792	0.20215834736614	0.273669774053551	1081
PRDM14	0.0387751670231547	0.202707389527826	0.274320862692332	1081
C17orf80	0.0387657923188784	0.202816660597016	0.27445030078101	1081
LOC10028	0.0387065792339061	0.203507835406068	0.275311620727636	1081
EIF3IP1	0.0386996827306321	0.203588447213239	0.275402180305581	1081
SP8	0.0386911749984032	0.203687924320728	0.2755182458498	1081
C21orf71	0.0386781183607161	0.203840658453971	0.275687819036942	1081
BTNL3	0.038658322956927	0.204072380159644	0.275945636885221	1081
MSH5	0.038643450368771	0.204246602086019	0.276144147585426	1081
KNTC1	0.0386058616174176	0.20468740953901	0.276665852466678	1081
PATE1	0.0386017479151396	0.204735693293325	0.276712548902653	1081
PRPF19	0.0385990737981992	0.204767084639466	0.276736409503692	1081
GPR22	0.0385881221728069	0.204895681777492	0.276891628725269	1081

IMMP1L	0.0385761586109003	0.205036228373039	0.277007230941913	1081
TMEM92	0.0385676412462867	0.205136332070659	0.277105108236122	1081
MRC1	0.0385664834616652	0.205149942131225	0.277105108236122	1081
XKR7	0.0385533130040241	0.205304810443072	0.277295702740576	1081
TAS2R16	0.0385500127298658	0.205343630882955	0.27731095020025	1081
FAM174A	0.0385462371142424	0.205388049199629	0.277352342882861	1081
SSX8	0.0385284259927825	0.205597682591062	0.27761083937798	1081
UTY	0.0385276316749295	0.205607035170984	0.27761083937798	1081
TMEM129	0.0385135803780665	0.205772531158129	0.277797054026304	1081
FURIN	0.0384387442109182	0.206655578821519	0.278877052207897	1081
CAPZA1	0.0384311792529773	0.206744996056407	0.278960344645433	1081
WHAMMI	0.0384226511864623	0.206845830789052	0.279077708207452	1081
CSTT	0.0384046427081871	0.207058877768649	0.279290330944053	1081
CLCA3P	0.0383976349667972	0.207141824917442	0.279383507161346	1081
CDK11A	0.0383820556286588	0.207326316060108	0.279557472358158	1081
GSTM2	0.0383744688441635	0.207416202157979	0.279659955322434	1081
PSKH2	0.0383181977560362	0.208083767660684	0.280447414165016	1081
NEU2	0.0383167074355785	0.208101469026154	0.280452508264108	1081
HRCT1	0.0383149122081508	0.2081227933891	0.280462483967049	1081
TAF5	0.0383004928410152	0.208294129256342	0.280637054164594	1081
C10orf67	0.0382895763882667	0.208423910192778	0.280755578123271	1081
POPDC2	0.0382866508564308	0.208458700505347	0.280764412573152	1081
BTBD16	0.0382855090358388	0.2084722801325	0.280764412573152	1081
C6orf147	0.0382674973137088	0.208686577554093	0.281034233894458	1081
ACBD6	0.0382512461685032	0.208880064988967	0.281257195989756	1081
C18orf10	0.0382396709389872	0.209017959730583	0.281367644421824	1081
RPL36A	0.0381696296033158	0.209853761438175	0.282436132026458	1081
SERPINA	0.0381584996173157	0.209986797482923	0.282559055327951	1081
LOC1001	0.0381584681366968	0.209987173855287	0.282559055327951	1081
LOC1001	0.0381454971013173	0.210142292964769	0.282730013475942	1081
STRC	0.0381393081219785	0.210216335430576	0.282810743772469	1081
DGAT2	0.0381017152504811	0.210666486574768	0.283321741908586	1081
CTXN1	0.0380837389402052	0.210881987604159	0.28356410807887	1081

TMEM105	0.0380752947114737	0.210983272445439	0.283672029861401	1081
ARPC5	0.0380661488761653	0.211093012380474	0.283762760870455	1081
C6orf58	0.0380595120274785	0.211172673049326	0.283850904022064	1081
C3orf33	0.0380381752732624	0.211428920597167	0.284176381467169	1081
HIP1R	0.0380208860312833	0.211636723416045	0.284436706465823	1081
HHEX	0.0380138550755843	0.211721272015897	0.284531355887942	1081
EXOSC8	0.0380101193366213	0.211766204914829	0.284572756854729	1081
LOC44004	0.0380066842822937	0.211807527298225	0.284609300788604	1081
GBX2	0.0379782367715865	0.212149963588225	0.285006230527154	1081
CYorf15B	0.0379774437602394	0.212159515164318	0.285006230527154	1081
TOLLIP	0.0379699215773505	0.212250132957921	0.285108950352559	1081
FLJ37543	0.0379419321793386	0.212587559245714	0.285543164488925	1081
RCN2	0.0379224667671845	0.212822452568426	0.2857824494728	1081
MGC4210	0.0379185917139441	0.212869235998434	0.285826218702157	1081
DEFB136	0.0379140713998134	0.212923819018158	0.285880454153611	1081
C2orf34	0.0379011984038078	0.21307931637675	0.286070165172251	1081
OXER1	0.0378790369010049	0.213347204562391	0.286372563693321	1081
LOC65036	0.0378708541793183	0.213446178614268	0.286486326083312	1081
TCF21	0.0378411571374862	0.213805656927809	0.286854143110937	1081
RBM45	0.0377929098349498	0.214390613001962	0.287581495467075	1081
OR4B1	0.0377883304377764	0.214446193984413	0.287636898449697	1081
FAM187B	0.0377852747339445	0.214483287392559	0.287667498303801	1081
C19orf54	0.0377601656605789	0.214788263190319	0.28799983772424	1081
GINS4	0.0377585092820631	0.214808392598635	0.288007658495807	1081
SFTPA1	0.0377489988106003	0.214923996425978	0.288124509082112	1081
C17orf77	0.0377489861942594	0.214924149812703	0.288124509082112	1081
POP1	0.0377420166164437	0.21500889646671	0.288199761748744	1081
TRIT1	0.0377312591444924	0.215139749261072	0.288336787230676	1081
CDR2L	0.0377227562107043	0.215243218624405	0.288456269238201	1081
IGFBP4	0.0377138395080035	0.215351761460049	0.288574489371086	1081
NIPA1	0.0377063087562919	0.21544346381331	0.288667015531702	1081
YAF2	0.0376796360312365	0.215768484911535	0.289064050578098	1081
MCAT	0.0376735488150347	0.215842710221066	0.289144261032276	1081

FAM55C	0.0376717141428494	0.215865085150011	0.289155006339981	1081
OR52L1	0.0376593273189705	0.21601619355293	0.289338179463016	1081
RNF151	0.0376537377307251	0.216084406533137	0.28939106299761	1081
HMGB3L1	0.0376443801861979	0.216198636728086	0.28952479803811	1081
SLC36A2	0.0376391023068434	0.216263084440606	0.28959185296836	1081
EPS8L2	0.0375947718207459	0.216804945259093	0.290240272610607	1081
DAO	0.037591990570043	0.216838973562008	0.290266537471371	1081
AKIRIN1	0.037541434335342	0.21745819264557	0.290979428153105	1081
COL23A1	0.0375127953450142	0.217809529486175	0.291375895437443	1081
PSG8	0.0375102097648631	0.217841268859932	0.291375895437443	1081
FLJ35220	0.0374956868625918	0.218019606923891	0.291595072465926	1081
CROCC	0.0374831690536326	0.218173406949994	0.291762034187973	1081
RGS16	0.0374529720748156	0.218544742489424	0.292219821946731	1081
C1orf158	0.0374503407602061	0.21857712151622	0.292243718918447	1081
SERPINH	0.0374288332778004	0.218841905848059	0.292520084487369	1081
PRAMEF1	0.0374034105339397	0.21915518860672	0.292898512648844	1081
AMAC1	0.0374023198380064	0.219168636369753	0.292898512648844	1081
UBXN2A	0.0373909056812711	0.219309403000172	0.293067192267892	1081
RMRP	0.0373830769588502	0.219405989246118	0.293176814364755	1081
RALYL	0.0373691717537603	0.219577618795764	0.293369164777509	1081
KCTD19	0.0373685044216631	0.219585857980138	0.293369164777509	1081
LDHD	0.0373601270090473	0.219689308163701	0.293477527384466	1081
BSDC1	0.0373550273501225	0.219752299440308	0.293522747130686	1081
MYBPHL	0.0373453652882824	0.219871681269636	0.293662734149494	1081
ZNF645	0.037297234279486	0.220467067428506	0.294360357459388	1081
HPS1	0.0372870907034388	0.220592691823321	0.294489050813838	1081
LOC4015	0.0372819098316653	0.220656874756325	0.294555214496466	1081
STARD7	0.0372747317605404	0.220745821976497	0.294654425107111	1081
CSRP2BP	0.037264804341141	0.220868880106712	0.294779658845766	1081
SFRS8	0.0372637288672402	0.220882214395185	0.294779658845766	1081
FAM50B	0.0372636218053952	0.220883541835535	0.294779658845766	1081
MMP19	0.0372462373907517	0.221099163797729	0.295008786243558	1081
ZNF114	0.0372347571596757	0.221241637706765	0.295121164981667	1081

THY1	0.0372347289991592	0.221241987269949	0.295121164981667	1081
ZDHHC7	0.0371994118446507	0.221680698679661	0.29566721894212	1081
BPIL2	0.0371981433563699	0.221696467473348	0.295668273444575	1081
C11orf9	0.0371969869772158	0.221710843318627	0.295668273444575	1081
KRT84	0.0371814341061413	0.221904257918688	0.295852023862169	1081
MYLK3	0.0371811834899299	0.221907375555094	0.295852023862169	1081
OR7C2	0.0371740602114508	0.221996001409046	0.295931011671866	1081
UPF3B	0.0371486352443188	0.222312538232944	0.296302894359265	1081
GMEB2	0.0371481085467579	0.222319098944665	0.296302894359265	1081
BEST2	0.0371259728616408	0.22259495323897	0.296592058535555	1081
PPIAL4B	0.0371058772955278	0.222845595449328	0.296867115482523	1081
C1orf177	0.0370919674149357	0.223019204707057	0.297078746202715	1081
C12orf24	0.0370777742414259	0.223196449346309	0.297275535344224	1081
TJP3	0.037070146340039	0.2232917483041	0.297382802431978	1081
MCEE	0.037064349307229	0.223364192798967	0.297459619484048	1081
ADORA2/	0.037033032698836	0.223755841286867	0.297961490132466	1081
TXNL1	0.0370215361789493	0.223899740850052	0.29813340468136	1081
UBXN6	0.0370199201946629	0.223919973087172	0.298140638323932	1081
APOH	0.0370117486207282	0.22402230175934	0.298257172	1081
CCDC60	0.0370048471322931	0.224108751767552	0.298332835812947	1081
SCARNA1	0.036980958909432	0.224408166328754	0.298711676403644	1081
GSTA4	0.0369708252049852	0.22453526821002	0.298841370116995	1081
OR4S1	0.0369608934857757	0.224659886534116	0.298987475340541	1081
HIST1H2/	0.0369420659038514	0.224896261207254	0.299282281808702	1081
TSPAN4	0.036937146719402	0.224958049324297	0.299344732389058	1081
HDGFL1	0.0369198458993572	0.22517545492909	0.299574662498119	1081
VPREB1	0.0369157549810385	0.225226884135815	0.299623296633164	1081
MMP13	0.0369121934099063	0.22527166544615	0.299663081115471	1081
KDM1A	0.0369061095479824	0.225348175393107	0.299725273556988	1081
DUSP5	0.0369035725541109	0.225380085810205	0.299747925572494	1081
PQLC1	0.0368660601882315	0.225852294057801	0.300316466257056	1081
RARG	0.0368362286832938	0.226228318187983	0.300776760156871	1081
PRSS30P	0.0368344886899023	0.226250264449332	0.300786087067366	1081

ELMOD3	0.0368071959307907	0.226594702238065	0.30120424127917	1081
MKS1	0.0368053955848772	0.226617435937715	0.301214584782612	1081
ASB10	0.036791765173435	0.226789605398323	0.301403654885158	1081
OR8J3	0.0367855240084668	0.226868470349282	0.301488576765373	1081
PAGE1	0.0367675191382812	0.227096093916744	0.301771161738312	1081
ACSL1	0.0367653868662652	0.227123061575404	0.301787090260306	1081
GPR26	0.0367631893152942	0.227150857227781	0.301804116795519	1081
POTEB	0.0367510321219429	0.227304670855908	0.301968649063062	1081
SEC22B	0.036743026505235	0.227405998937639	0.302083339835063	1081
CDK7	0.0366960219551145	0.228001589987354	0.302774355297106	1081
C9orf135	0.036695132632558	0.228012869212357	0.302774355297106	1081
CLC	0.036662588987535	0.228425891412324	0.303291086513517	1081
SCRT2	0.0366126772189501	0.229060371546765	0.304050014997593	1081
GTF2E1	0.0366121080796233	0.229067613681731	0.304050014997593	1081
GREM1	0.0365936768883876	0.229302233294193	0.304321331425753	1081
ATHL1	0.036590772564626	0.229339219415371	0.304350367267632	1081
UPK1B	0.0365719084199121	0.229579554621012	0.304649241014445	1081
MPHOSPI	0.0365583320956366	0.229752632003926	0.304838752006789	1081
GCG	0.0365449588920605	0.229923210505334	0.305004812039375	1081
DEFB115	0.0364983089469091	0.230518946115419	0.305694435487796	1081
CYP27A1	0.0364748588232493	0.230818825629522	0.306071961710553	1081
ZNF821	0.0364526922382376	0.231102545724762	0.306428011822901	1081
PBX2	0.0364414633462199	0.231246363745873	0.306598525579875	1081
WDR5	0.036414883164321	0.231587051856771	0.306989613716829	1081
GPR148	0.0364015281344123	0.2317583625495	0.307196487271988	1081
AP3D1	0.036390153458986	0.231904341228225	0.307369758144785	1081
RASGRP3	0.0363546161637018	0.232360834627159	0.307893771858457	1081
C6orf221	0.0363521809412575	0.232392139511822	0.307906404358245	1081
LCE2B	0.036286222686046	0.23324117263324	0.308897733396662	1081
IFNA8	0.0362710087769149	0.233437321701287	0.309123332203691	1081
ERN2	0.036267658293888	0.233480534316091	0.309153763888369	1081
SYNC	0.0362440736582395	0.233784875711777	0.309516056862102	1081
FUT3	0.0362026411837458	0.234320209308334	0.310122897084054	1081

BDH1	0.0361960684596038	0.23440521266847	0.310194640294708	1081
HDAC8	0.0361793120534425	0.234622017670139	0.3104611498926	1081
OR2M2	0.0361733387846165	0.234699337886923	0.310543064809987	1081
UGT1A8	0.036160917976973	0.234860175139571	0.310735467502079	1081
TNP1	0.0361463384345679	0.235049065300989	0.310944537725796	1081
ALDH1B1	0.0361381545917879	0.235155140879826	0.311064437128117	1081
MEPCE	0.036114577911718	0.23546092120251	0.311428025581023	1081
C5orf46	0.0361015093310564	0.235630536585688	0.311631903854956	1081
GGT1	0.0360927122191305	0.235744761749543	0.311742039217838	1081
APBB3	0.0360899573399598	0.235780540228017	0.31176888630741	1081
ACBD4	0.0360799496145898	0.235910545973788	0.311899845681037	1081
YSK4	0.0360719235984975	0.236014844833818	0.31201726271015	1081
LOC10013	0.0360485250784145	0.236319096766234	0.312398989720833	1081
PRR11	0.0360346273586989	0.236499940456366	0.312576518861927	1081
UGT3A1	0.0360285497672142	0.236579055647534	0.312660570492658	1081
EPHX3	0.0360209815496453	0.236677601218977	0.312770288412247	1081
CFC1B	0.0360067976582921	0.236862367353551	0.312993369334141	1081
CXorf66	0.0360044452557361	0.236893020711722	0.312993369334141	1081
ATG9A	0.0359943078303755	0.237025150088364	0.313147406120808	1081
OCM2	0.0359793848171054	0.237219748578131	0.313383948790507	1081
MUC16	0.0359673820403256	0.237376348496776	0.313554332268145	1081
DNAJB5	0.0359646792950866	0.237411621202726	0.313575734928463	1081
4-Mar	0.0359584392008767	0.237493072897885	0.313662751821143	1081
LDLRAD1	0.0359420947605086	0.237706509645704	0.313903483239115	1081
SAMD14	0.0359119046155377	0.238101108633339	0.314342147809071	1081
FSCN1	0.0359006639776544	0.238248146833066	0.314474440961349	1081
RYR1	0.0358940370805635	0.23833486291752	0.314568290209882	1081
PROC	0.0358795726458624	0.238524214164045	0.314756340517142	1081
KRTAP4-2	0.035871938080835	0.238624199585784	0.314847033053158	1081
ANXA2P1	0.0358666627642378	0.238693304567893	0.314917585992547	1081
CUZD1	0.0358604011104	0.238775348559523	0.314984163498184	1081
SLC17A3	0.0358580384523744	0.238806310702793	0.314984163498184	1081
FAM111A	0.0358564515636204	0.238827108138785	0.314990971157249	1081

LOC64662	0.0358545106520815	0.238852547059214	0.315003899214918	1081
PRAMEF1	0.0358421720334197	0.239014310121703	0.315196601034414	1081
RFT1	0.0358128992830962	0.23939839354095	0.315620460594401	1081
HOXD1	0.0358064160660753	0.239483517632443	0.315705584012213	1081
GPRC5D	0.0358016191100869	0.239546514977364	0.315753748885625	1081
STX1A	0.0357841423429932	0.23977613213148	0.31599438582943	1081
OR10A7	0.0357589112412244	0.240107902274073	0.316373289399825	1081
LYPD6B	0.0357388077013065	0.240372479979007	0.316676712391413	1081
SPINK1	0.0357311682378531	0.240473074832005	0.316788522919876	1081
SPAG11A	0.0357224400095507	0.240588042559897	0.316898529970519	1081
SLC6A19	0.0356677561681512	0.241309215060024	0.31774456744782	1081
CCDC92	0.0356592318932801	0.241421770285508	0.31785122295285	1081
TCP1	0.0356457318839645	0.241600101014823	0.318065222516247	1081
STARD4	0.0355961663690709	0.242255639252732	0.318865191748687	1081
NPPC	0.0355938055002703	0.24228689451959	0.318865191748687	1081
LEO1	0.0355324846346789	0.243099706722968	0.319830433120049	1081
C9orf172	0.0355275769185234	0.243164841452907	0.319877786241418	1081
MFSD7	0.0355144198270266	0.243339521521122	0.320062330963894	1081
SPACA1	0.035510441705834	0.243392354363359	0.320110921110522	1081
DEXI	0.0354875756061769	0.243696191774354	0.32046868485504	1081
GDPD5	0.0354716338727239	0.243908177783915	0.320684652229277	1081
C7orf26	0.0354428667416779	0.24429103737873	0.321125151582024	1081
OR7E37P	0.0354281605947283	0.244486923071614	0.321361677233306	1081
ZP3	0.0354101036221219	0.244727592290945	0.321615065999157	1081
SFTA1P	0.0354085581841551	0.244748198103314	0.321621164435177	1081
SLC22A2	0.0353841171637702	0.245074239637255	0.322007602236662	1081
UBE2DNI	0.0353636954326329	0.245346897402941	0.322344828029886	1081
C13orf31	0.03535261001973	0.245494991623513	0.322518364826899	1081
HDHD3	0.0353220645281831	0.245903384295228	0.323033823060148	1081
FAM102A	0.0353122226240335	0.246035072	0.323143598786531	1081
SLFN13	0.0353046399440482	0.246136563618361	0.323255823332152	1081
DCAF4L1	0.0353021903930447	0.246169356411944	0.323277815183742	1081
FCAMR	0.0352998602126107	0.246200553997974	0.323297709425017	1081

RAB20	0.035238961165836	0.247016881412244	0.324221727655047	1081
HIST4H4	0.0352303495424653	0.247132469391304	0.324344996942222	1081
KCNS3	0.0352295652411733	0.247142998414649	0.324344996942222	1081
TMEM59	0.0352004798581774	0.24753368302565	0.324836560781169	1081
ST6GALN	0.035184252229361	0.247751845526988	0.325059328392677	1081
TMCO5A	0.0351829184436589	0.247769782774158	0.325061691565316	1081
ZNF467	0.035180913627176	0.247796746000903	0.325075895297486	1081
MYBPC2	0.0351750199923997	0.247876022693625	0.32515872091965	1081
RPTN	0.0351461564150736	0.248264529557662	0.325604749214785	1081
SSTR5	0.0351300692075091	0.248481249645165	0.325867767339486	1081
SH2B3	0.0351025097332199	0.248852826316239	0.326291342498088	1081
TMEM15	0.0350487389768984	0.249578918957472	0.327158206819252	1081
TRIM27	0.0350467680079289	0.249605561967724	0.327171842399623	1081
FLJ42289	0.0350323426020744	0.249800621011107	0.327384913816196	1081
MGST1	0.0350067431010257	0.250147036937718	0.32779627002604	1081
OSTalpha	0.0349802275555347	0.250506202013025	0.328224223710724	1081
LOC2864	0.0349709778600606	0.250631577878968	0.328345785027757	1081
LOC1002	0.0349635636905568	0.250732105516571	0.32843476595561	1081
KIF1A	0.0349174340106923	0.251358202560533	0.329169277161928	1081
C3orf22	0.0348841328919385	0.251810859849665	0.329673017869623	1081
ANKRD2	0.0348694695394474	0.252010356588062	0.32987320555338	1081
LOC2851	0.0348673170563035	0.252039650649812	0.329890115154341	1081
CYP4F3	0.0348644900006358	0.252078128858689	0.329919042740273	1081
PLA2G4D	0.0348186682445168	0.252702365498494	0.33060716670783	1081
PITPNM2	0.0348119260618931	0.252794305962734	0.330684499585667	1081
ZWILCH	0.0348044306648337	0.252896545029829	0.330796759926362	1081
RBPMS2	0.0347957675185671	0.253014748319672	0.330929886047254	1081
DRGX	0.0347771173387113	0.253269348942313	0.331219880189748	1081
GRSF1	0.0347719832868878	0.253339467081035	0.331268568372546	1081
C11orf52	0.0347640290802099	0.253448128047518	0.331367636261503	1081
CDK2AP1	0.0347557846800244	0.253560787494056	0.331450396734005	1081
ARID3B	0.0347543027449765	0.253581041777128	0.331455365079175	1081
ARL3	0.0347495144596628	0.253646493148524	0.331519405986961	1081

UIMC1	0.0347202299647958	0.254047040311881	0.331999846064407	1081
SLC38A3	0.0347116439578374	0.254164561203563	0.332131881515683	1081
SNORA20	0.0347042633345884	0.254265613608127	0.332199285695293	1081
RAB19	0.034655884819497	0.254928683222488	0.332979207904884	1081
HAL	0.0346495754625856	0.255015246876731	0.333070678704081	1081
CDX4	0.0346434431328336	0.255099401296043	0.333158991190197	1081
CDK5R1	0.0346418863066343	0.255120768806896	0.333165298043233	1081
CRYGD	0.0346222148890691	0.255390866968506	0.333435053823703	1081
KRT28	0.0346127962950102	0.255520259107084	0.333578874973249	1081
UTP3	0.034611038464362	0.255544413127443	0.333588789687165	1081
FBXO27	0.0345898475885646	0.255835717669961	0.333925782847049	1081
PIK3CG	0.0345625238371262	0.256211668967032	0.334356945603806	1081
KRT39	0.0345622205854337	0.25621584359821	0.334356945603806	1081
TGIF2LX	0.0345553849431244	0.256309957109773	0.334458095995361	1081
OR4X1	0.0345451678200541	0.256450671737247	0.334598366719355	1081
STK16	0.0345225921775796	0.256761783167052	0.334917516310558	1081
OR10J1	0.0345166976444791	0.256843057875181	0.335001838840759	1081
TRPC7	0.0344631680402515	0.257581948495229	0.335878591005009	1081
C21orf63	0.0344345502827562	0.25797757438949	0.336329163608353	1081
ALS2CL	0.0344326534769573	0.258003811623651	0.336341602611691	1081
LOC44145	0.034418642437459	0.258197674111867	0.336572547235898	1081
LASP1	0.0343957881316431	0.258514112044328	0.336963234366418	1081
FRMPD1	0.034381617401593	0.258710453013416	0.33719733928282	1081
OPN1LW	0.0343104076770987	0.259698653550826	0.33835012458534	1081
MMP17	0.0343094124348819	0.259712483319092	0.33835012458534	1081
MSLN	0.0343051265074712	0.259772045878177	0.338405837054829	1081
ACVRL1	0.034286203473557	0.260035136706712	0.338688702143462	1081
SNORA57	0.0342858810837387	0.260039620553633	0.338688702143462	1081
DPY19L2l	0.0342530034547601	0.260497168294107	0.33924076897568	1081
DIRC2	0.0342405819672942	0.26067017896183	0.339422193470756	1081
MS4A5	0.0341959990191939	0.261291798957002	0.340121691004704	1081
ABCF2	0.0341929111688054	0.261334890717154	0.340155803676234	1081
MLXIPL	0.0341765310244469	0.261563561961621	0.340431448230834	1081

FMO3	0.0341485536962691	0.261954452352279	0.340874132332321	1081
ZNF692	0.0341379264427445	0.262103038560339	0.341001402208604	1081
CTAG2	0.0341328527987217	0.262173996819835	0.341071692989059	1081
CA5A	0.0340956541800956	0.262694648813592	0.341628824835038	1081
KRTAP13	0.0340949863768081	0.262704002259597	0.341628824835038	1081
C12orf36	0.03408167944451	0.262890430881313	0.341810365006092	1081
PDE4DIP	0.0340728447963062	0.263014253673847	0.341943990981584	1081
WFDC13	0.0340698992543196	0.263055546112773	0.341975605087881	1081
SLC17A4	0.0340553315239146	0.263259831314281	0.342219094136039	1081
KRT35	0.0340389285753804	0.26348998285221	0.342429888688328	1081
ACBD7	0.0340369637378135	0.263517560994922	0.342443637352774	1081
CRX	0.0340334809704997	0.263566449441185	0.342462985293645	1081
SNORA65	0.0340235539269338	0.263705832039159	0.342599895938918	1081
KCNH3	0.0340170616090698	0.263797016157965	0.342696259141176	1081
ACTL6B	0.0339937287023379	0.264124904365661	0.343048235775193	1081
TAAR6	0.0339900968909287	0.264175966020825	0.34307792905425	1081
ZNF485	0.033979278406704	0.264328109383628	0.343183287004057	1081
HSCB	0.0339792652063928	0.264328295060128	0.343183287004057	1081
SBK2	0.0339777245515644	0.264349966635766	0.343183287004057	1081
NEURL1E	0.0339738332546174	0.264404708896001	0.343197993602444	1081
C4BPB	0.0339037220322572	0.26539236165978	0.344435583406536	1081
NAA11	0.0338753156945632	0.26579324153512	0.344889201334944	1081
ACPT	0.0338582330073319	0.266034518588175	0.345180044889783	1081
STK4	0.0338527526882422	0.266111954881646	0.34525828112905	1081
CSHL1	0.0338456810261388	0.266211899565843	0.345365708188686	1081
TMEM31	0.0338392951605869	0.266302173966561	0.345438331909619	1081
ZC3H8	0.0338332755236169	0.266387290403055	0.345526493153663	1081
ODC1	0.0338159759786313	0.266632006491969	0.345777119272609	1081
POU4F3	0.0338138614466304	0.266661928851158	0.345793663128843	1081
NLRP6	0.0338100555086162	0.266715791803288	0.345841247803633	1081
CLIC2	0.0337849854008385	0.267070779413782	0.346256973309991	1081
ANKK1	0.0337669641556445	0.267326156936622	0.346543464059734	1081
FLJ36031	0.0337504476114636	0.267560358676319	0.346824749085286	1081

GPR179	0.0337444159099955	0.267645922259617	0.34691333830395	1081
SLC29A2	0.033726105089213	0.267905788129882	0.347205487768687	1081
OR10X1	0.0337184378779397	0.268014652060778	0.34732423066995	1081
C9orf163	0.0337105731101748	0.268126352557246	0.347424285860019	1081
PAK1IP1	0.0337079041275054	0.268164266425587	0.347451064261863	1081
ALG14	0.0336695313595533	0.268709772133761	0.348113077790008	1081
ELAVL3	0.0336439267486033	0.269074189228009	0.348517941361683	1081
IFNA6	0.0336424728638327	0.269094891772469	0.348522347611958	1081
TMEM114	0.0336356520334681	0.269192031339447	0.348588472142176	1081
SMOC1	0.0336329465456127	0.269230568483558	0.348588472142176	1081
OR51M1	0.0336315985739395	0.269249770490557	0.348588472142176	1081
TUBD1	0.0336205492906329	0.269407204146006	0.34873697018758	1081
HBG2	0.0336178759811029	0.269445303774651	0.34873697018758	1081
ACTRT2	0.0336084941139462	0.269579042031259	0.348857827565987	1081
ZP1	0.0335935462562655	0.26979221727661	0.349044005975037	1081
CNDP1	0.0335894239949787	0.269851026214617	0.349097670375896	1081
OR5T3	0.0335668055014482	0.270173862074199	0.349470428373234	1081
FLJ40504	0.0335425848719228	0.27051985851086	0.349873045050158	1081
HLA-DQE	0.0335242052945453	0.270782616717148	0.35016791717595	1081
ARF6	0.0335116251656841	0.270962565482485	0.350378129638916	1081
CETN3	0.0334861844051819	0.271326725414958	0.350803984563528	1081
PRCP	0.0334715400020512	0.271536497667738	0.351030144742127	1081
CNOT10	0.0334475815841551	0.271879926695998	0.351429010156617	1081
RNF126P1	0.0334458070178016	0.271905375809337	0.351439355554927	1081
OR10P1	0.0334283940524943	0.272155182106668	0.351694537400656	1081
CLDN25	0.0333936887580432	0.272653531849477	0.352225561056029	1081
TXN2	0.0333776993975569	0.272883340304477	0.352499832666476	1081
UBE2B	0.0333731142660877	0.272949264903564	0.352562384039645	1081
NXF3	0.0333542890350142	0.273220046360844	0.352844274244451	1081
NPRL3	0.0333478782253193	0.273312301077707	0.352940788795538	1081
GADD45E	0.0333369922561772	0.273469004267705	0.353120510927051	1081
LOR	0.033320422508206	0.273707642997006	0.353360706753057	1081
FAM176B	0.0333077333022304	0.273890489952761	0.353574105741645	1081

KCNK3	0.0333054289374424	0.273923703973275	0.353594323756352	1081
SEZ6L2	0.033303197093042	0.273955875339495	0.353610862084394	1081
MSN	0.0333021047200617	0.273971622507482	0.353610862084394	1081
CXorf40A	0.0332977437761922	0.274034494109404	0.353646693391027	1081
KRTDAP	0.0332956204429458	0.274065109706972	0.35366354718128	1081
MRPS30	0.0332773465893178	0.27432869046888	0.353935664755705	1081
GLYCTK	0.0332340977561801	0.274953197321085	0.354718678196175	1081
LOC1501[illegible]	0.0331900267990152	0.275590571880861	0.355427149202301	1081
LRRIQ4	0.0331632283065087	0.275978635897148	0.355849320987954	1081
OR4M1	0.0331560124504123	0.276083190945959	0.355948536422805	1081
CCND1	0.0331471805307221	0.276211198862374	0.356090784659063	1081
HAO1	0.0331344781218663	0.276395375660648	0.356292235288283	1081
PHLDB3	0.0331227130806626	0.276566035799227	0.356388587678336	1081
OR2Z1	0.0331190199900882	0.276619621446698	0.356434839071493	1081
KLHL30	0.0331088968964643	0.276766540764276	0.356601340818442	1081
HYI	0.0331033934599894	0.276846435974048	0.356681469218291	1081
C10orf53	0.0330433078254207	0.277719740223111	0.357592226986965	1081
LOC4403[illegible]	0.0330425442371577	0.277730850505736	0.357592226986965	1081
PGAM5	0.0330168805175689	0.278104435883741	0.358050352200291	1081
PTDSS1	0.0329489178878502	0.279095413754016	0.359142581288704	1081
FAM127C	0.0329310651700976	0.27935612534672	0.359432148247408	1081
ACSS2	0.0329116694441947	0.279639557567338	0.359750870885371	1081
VCX3B	0.0329082478691305	0.279689577728683	0.359792244147147	1081
NUDT8	0.0328840996211804	0.280042774742406	0.360200593614211	1081
CXorf27	0.0328776000153441	0.280137890922319	0.360276928794057	1081
FAM194B	0.0328758528889838	0.280163462361784	0.360286814508658	1081
C1orf100	0.0328578152113601	0.280427559530012	0.360580403825371	1081
HPR	0.0328439249080438	0.280631048311998	0.360795996441713	1081
SCARF1	0.0328303306962284	0.280830296378619	0.360983047508121	1081
DLEU7	0.0328155663194006	0.281046803992364	0.361238299395532	1081
BOD1	0.0328054232553801	0.28119560935534	0.361406504431755	1081
PRPF3	0.03278778537041	0.281454495781222	0.361693086087823	1081
XK	0.0327823492714599	0.281534318637612	0.361772587163022	1081

FZR1	0.0327773088479823	0.28160834515944	0.361821551963576	1081
CCDC140	0.032767858024568	0.281747180863314	0.361976846842568	1081
PCOLCE	0.0327508653568184	0.281996925365141	0.362274603754912	1081
TUBAL3	0.0327313932670644	0.282283294709034	0.362619370738011	1081
C2orf49	0.0327094285962898	0.282606557847361	0.362988338933632	1081
TMEM12(	0.0326861189603662	0.282949889369014	0.36339467660903	1081
CPT2	0.0326581578674343	0.283362105195339	0.363869422047337	1081
TFAMP1	0.0326268207732028	0.283824574183545	0.364413387312142	1081
CHRNA4	0.0326225785524261	0.283887219554398	0.364470590652179	1081
OR52B4	0.0326123784797394	0.284037883481277	0.364640782738359	1081
GAGE1	0.0325900775530705	0.284367475777441	0.365037860972267	1081
PANX1	0.0325889985236365	0.284383429640047	0.365037860972267	1081
SPATS1	0.0325688566891302	0.284681345389988	0.365327156509193	1081
SMG5	0.0325634476372182	0.284761386008058	0.365406594122704	1081
SLC20A1	0.0325372315471313	0.285149534824339	0.365881361722058	1081
OGDHL	0.0325124154481733	0.285517284753407	0.36623659352801	1081
OR2K2	0.0325051566739576	0.285624913069937	0.366351322666107	1081
PINK1	0.0325016070267489	0.285677554866777	0.366395514279698	1081
HULC	0.0324757921755776	0.286060589937481	0.366863418913328	1081
FASTKD1	0.0324567697628874	0.286343061954339	0.367202303333768	1081
SNORD11	0.0324540095701268	0.286384064873865	0.367231507758371	1081
TPRXL	0.0324491178561813	0.286456741466702	0.367301321166489	1081
CNIH	0.0324344839702763	0.286674232567899	0.3675497795705	1081
CLEC4GP	0.0324336249080866	0.28668700354387	0.3675497795705	1081
NLRP10	0.0324209446646091	0.286875554992254	0.36774470603366	1081
RIMBP2	0.0324169453069649	0.286935041590229	0.36779755744688	1081
C22orf13	0.0324147475069578	0.286967735291675	0.367816060790913	1081
INPP1	0.0324112547278509	0.287019697814272	0.367859257696176	1081
KLHL35	0.0323996252366925	0.287192756961067	0.368057642914988	1081
KRTAP5-4	0.0323961185667433	0.287244953722094	0.368101119130508	1081
RHOH	0.0323869671958942	0.28738120193577	0.368252293937796	1081
CBWD2	0.0323833923716633	0.287434436763345	0.36829708234306	1081
INF2	0.0323627153747041	0.287742480555794	0.36866833751806	1081

OR4D9	0.0323512159719398	0.287913893685655	0.368817587962452	1081
EFCAB2	0.0323404770034879	0.288074033675487	0.368952343339862	1081
C1orf92	0.0323116707588703	0.288503889923045	0.369455906331431	1081
HLA-DOA	0.0323013408642991	0.288658141131754	0.369606448426822	1081
POM121L	0.0322933606799758	0.288777343278003	0.369712080097577	1081
LOC1447	0.0322730429640653	0.289080983958168	0.37006953573029	1081
CEMP1	0.0322722199679843	0.289093287852448	0.37006953573029	1081
FUNDC2P	0.0322346135957681	0.28965588370916	0.370742599740837	1081
OR52E6	0.0322274452487013	0.28976320653058	0.370856403783896	1081
LOC1001	0.0322196808090126	0.289879484072481	0.370909364635373	1081
C16orf68	0.0322061734219393	0.290081840737164	0.371075608436003	1081
SURF6	0.0321852240058502	0.290395875606151	0.371406571905651	1081
CD276	0.0321654290189418	0.290692815245079	0.371739145757133	1081
CHID1	0.0321581666635521	0.290801807144803	0.371844599914597	1081
HIST1H2I	0.0321574745835701	0.290812195167758	0.371844599914597	1081
DNAI2	0.0321499988144702	0.290924421295827	0.371936873567212	1081
C6orf223	0.0321489779003416	0.290939749499734	0.371936873567212	1081
HMX2	0.0321304694489812	0.291217733053712	0.372221383945627	1081
SLC12A7	0.032124953209801	0.291300617462112	0.372303701229175	1081
TAF7	0.0321149961865346	0.291450267090215	0.37247133353628	1081
C16orf93	0.0321031866789546	0.291627825626675	0.372650970329804	1081
EHBP1L1	0.0320986314000145	0.291696334644587	0.372714871331677	1081
PLCG1	0.0320889056556546	0.291842640910955	0.372878163325375	1081
C1orf185	0.032085547582886	0.291893168488196	0.372919068949437	1081
DVL1	0.0320796425709476	0.29198203307585	0.372989035717185	1081
LCLAT1	0.0320723621303127	0.29209162148898	0.373077968228221	1081
C2orf56	0.032069949616819	0.292127941795563	0.373100702939937	1081
PSG11	0.0320590161682506	0.29229258246146	0.373287312900095	1081
PRSS33	0.0320436073001478	0.292524721608862	0.373536417140626	1081
C8orf33	0.0320159714598312	0.292941373348122	0.374021039808462	1081
TNFRSF1	0.0320068129491347	0.293079539353077	0.374163896395745	1081
WFDC8	0.0320060925772149	0.293090408792497	0.374163896395745	1081
AAA1	0.0319994772260093	0.293190238141595	0.374220195613155	1081

CR2	0.0319808207699572	0.293471897240165	0.374555966162134	1081
PPP1R10	0.0319352382393062	0.294160825378444	0.375363896069472	1081
HOXB4	0.031865915281291	0.295210637257042	0.376655791218134	1081
OR2H2	0.0318503266227263	0.295447053243165	0.376933558428344	1081
PRSS21	0.0318309327763754	0.295741355016204	0.377285136900857	1081
DNA2	0.0318052613481741	0.296131220474589	0.377686827252966	1081
CBX1	0.0317899983452201	0.296363178566827	0.377934812447219	1081
HBZ	0.0317781537005849	0.296543270110391	0.37811660082118	1081
POLR3F	0.0317486713863974	0.296991850476402	0.378616683194983	1081
OR1D4	0.0317393948382285	0.297133089014545	0.378755388578575	1081
CLDN15	0.0317390555460564	0.297138255700958	0.378755388578575	1081
C10orf122	0.0317260019803597	0.297337078839338	0.378945390201017	1081
NXF2	0.0317221484093731	0.297395790817058	0.378963767854364	1081
CHGB	0.0317144210571762	0.297513546006002	0.379089840878616	1081
TAAR1	0.0317115653008643	0.297557072037165	0.37912132162347	1081
MLLT11	0.0317092727304296	0.297592017341809	0.379141866263012	1081
LOC64681	0.0316839254033936	0.297978565361586	0.379538328225161	1081
GAGE12F	0.0316506331347051	0.298486782838482	0.380089522141669	1081
AKAP7	0.0316483751989673	0.298521271890216	0.380109412946615	1081
KLC4	0.0316412890357364	0.298629527411547	0.380183020909198	1081
NIPA2	0.0316410005007422	0.298633935923536	0.380183020909198	1081
CHST8	0.0316408852178268	0.298635697337659	0.380183020909198	1081
CDCP1	0.0316219044595217	0.298925799863919	0.380528293096913	1081
APH1A	0.0316142595473084	0.299042697996813	0.380653048852887	1081
PRPF40B	0.0316059430847703	0.299169899380654	0.380790903078491	1081
BEST1	0.0315888155608119	0.299431980678878	0.381028188413619	1081
SLC45A2	0.0315833062060741	0.2995163160186	0.381111431669701	1081
ZNF768	0.0315724171296521	0.299683048858557	0.381299502258298	1081
EFR3B	0.03156742404889	0.299759523259738	0.381372716689871	1081
OR6P1	0.0315642738436346	0.299807778727009	0.38141002225801	1081
CIDEB	0.0315561326183203	0.299932511586802	0.381520518148395	1081
DHX30	0.0315286680702145	0.300353554686789	0.381959621183345	1081
LIPF	0.0315191104897987	0.300500168655943	0.382097828540931	1081

OXT	0.0315151565379225	0.300560836487252	0.38215084896716	1081
CLEC9A	0.0315069063087062	0.300687450899075	0.382263580610997	1081
TFF3	0.031496470726259	0.300847654266487	0.382418980009993	1081
CLRN2	0.0314932496263151	0.300897114919731	0.382457718471701	1081
KRT79	0.0314867514349579	0.300996912450997	0.382556250377273	1081
SCRN2	0.0314604590397711	0.301400928964597	0.383025593867765	1081
SLC17A6	0.0314270831390213	0.301914311848923	0.383629608439293	1081
PTDSS2	0.0314163289489542	0.302079854849343	0.383815747457585	1081
MAFB	0.0314119400667956	0.302147431771923	0.383877397391864	1081
EPDR1	0.0313962540287704	0.302389036462367	0.384135902354293	1081
CPNE4	0.0313343770019337	0.303343350700737	0.385275316806402	1081
TTC38	0.0313330228726914	0.303364257431277	0.385277579495213	1081
SUV39H2	0.0313263828869332	0.303466787627163	0.385383498502755	1081
KRTAP12	0.0312953912979676	0.303945643036979	0.38588758515497	1081
GPER	0.0312947147612488	0.303956101888278	0.38588758515497	1081
CLNS1A	0.0312937876633708	0.303970434652301	0.38588758515497	1081
RPLP2	0.0312928985604813	0.303984180441853	0.38588758515497	1081
NSUN5P2	0.0312713935070414	0.304316780421425	0.386268133173058	1081
OR7G3	0.031266810707562	0.304387689778769	0.386333796030324	1081
FZD9	0.0312582420593033	0.304520301303383	0.386477759072428	1081
CYP2B7P	0.0312455852071188	0.304716253425903	0.386673822389512	1081
TAAR5	0.0312406779380195	0.304792249755568	0.386725443807137	1081
ID1	0.0312383209301033	0.304828755979393	0.386742785084047	1081
SLC23A3	0.0312373165582353	0.304844312948602	0.386742785084047	1081
DNM1P35	0.0312301701391772	0.304955020827873	0.386858872454503	1081
RPH3A	0.0312190205222524	0.305127796962643	0.387022560243555	1081
CRYZL1	0.0312186585717736	0.305133406888216	0.387022560243555	1081
SLC5A6	0.0312122350739844	0.305232977026421	0.387073815460125	1081
DHRS7	0.0312028049553543	0.305379191552571	0.387226273923745	1081
HNF4G	0.0311699808147432	0.305888493475413	0.387847664971185	1081
TMEM30(	0.0311511112773634	0.306181529113468	0.388152024777797	1081
TYRO3	0.0311456956856043	0.306265665183144	0.388228154109894	1081
RTKN2	0.031092378091344	0.307094818630409	0.389107813152373	1081

MAGEB2	0.0310900353133225	0.307131285694168	0.389123317267077	1081
KRTAP10	0.031083884529066	0.307227040805061	0.389201907748583	1081
LOC33924	0.0310689104547581	0.307460238986111	0.389453038590097	1081
FAM108C	0.0310515259449275	0.307731122743628	0.389747396560504	1081
ABTB2	0.031051275548452	0.307735025549336	0.389747396560504	1081
C7orf70	0.0310465169054231	0.307809202377046	0.389788971007289	1081
PAGE4	0.0310462030875068	0.307814094526653	0.389788971007289	1081
LGI4	0.0310242758740168	0.308156048027341	0.390099782408859	1081
VAPB	0.0310234983817377	0.308168177575308	0.390099782408859	1081
TMBIM1	0.0310076006074072	0.308416265683048	0.390340244530063	1081
LOC72789	0.0309938848473888	0.308630408965093	0.390537662600615	1081
TCP11L1	0.0309883141512238	0.308717411917303	0.390623218583887	1081
C9orf57	0.0309692824935752	0.309014769887616	0.390925806291511	1081
GSG1	0.0309639554685174	0.30909803523883	0.39098817534348	1081
LMAN1L	0.0309636450295036	0.30910288808738	0.39098817534348	1081
PCBP4	0.0309575472274474	0.30919822041839	0.391084206085796	1081
AHSG	0.0309560969043151	0.309220897458403	0.391088333707906	1081
DVL3	0.0309542331959846	0.309250039740258	0.391100637238213	1081
ACTL6A	0.0309454305084853	0.309387709380666	0.39122562334276	1081
C8orf77	0.0309368621044369	0.309521753778785	0.391370557988553	1081
BEX1	0.0309277797431369	0.309663880367702	0.3915256932158	1081
GGNBP2	0.0308921649951109	0.310221618027069	0.392132427380015	1081
HOMEZ	0.0308883164898948	0.310281926402204	0.392184051159016	1081
LOC28537	0.0308747580576608	0.31049445671283	0.392403439804263	1081
LOC10013	0.0308609072299299	0.3107116694207	0.392653320398972	1081
TCF15	0.0308486118997497	0.310904572245092	0.392872450625855	1081
MANBA	0.0308397946254644	0.311042956030895	0.393022665071968	1081
SERPINC1	0.0308277694561369	0.311231751914473	0.393187234561461	1081
DYNC1LI	0.0308020941775536	0.311635107912406	0.393647432210648	1081
TTC25	0.0307930934297371	0.311776590180092	0.393801455212111	1081
OR2H1	0.0307363819976862	0.312669006544537	0.394755398398076	1081
DEFB134	0.0307129162740328	0.313038756300867	0.395172687409511	1081
LOC25845	0.0306988830638894	0.313260015121881	0.395386884331198	1081

AGXT	0.0306984201840566	0.313267315006714	0.395386884331198	1081
PROK1	0.0306814686335376	0.313534727875527	0.395663148142032	1081
MCCC1	0.0306808098873794	0.313545122707341	0.395663148142032	1081
ACOT12	0.0306653174929512	0.313789653694238	0.395922120360167	1081
HIST1H2/	0.0306571360382895	0.313918839807807	0.396060313642405	1081
LOC2226§	0.0306542290332714	0.313964750164378	0.396093430114065	1081
CTAGE1	0.0306494545396073	0.314040163356736	0.396138953714555	1081
KHDRBS1	0.0306422978743507	0.31415322526432	0.396256760562878	1081
GIMAP8	0.0306300671341732	0.314346509931787	0.396426095065472	1081
GPR89C	0.0306168488274734	0.314555489082286	0.396664809237098	1081
CMTM7	0.0305810967092757	0.315121180798665	0.397328421304945	1081
CHI3L2	0.0305773435750555	0.315180603859048	0.397353605653703	1081
PXN	0.0305542221899839	0.315546844972739	0.397790437545422	1081
PGLYRP3	0.0305448182053625	0.315695883248485	0.397903620599432	1081
C12orf56	0.0305402174052977	0.315768815464181	0.397970644865235	1081
MYF5	0.0305326647552999	0.315888564572862	0.398096661452631	1081
PCDHA1	0.0305129140703906	0.316201857229548	0.398466559107297	1081
LOC81691	0.0305080859386476	0.316278473806677	0.398538178197004	1081
ZFAND3	0.0304334927716644	0.317463722655781	0.399856614536745	1081
CERCAM	0.0304283457116862	0.317545614022569	0.399934754265999	1081
AIRE	0.0304226202822836	0.317636723676444	0.400020050790624	1081
C17orf72	0.0304215942440789	0.317653052990698	0.400020050790624	1081
SCPEP1	0.0304144763200324	0.317766349298351	0.400120903239604	1081
TTPAL	0.0304140671243493	0.317772863287686	0.400120903239604	1081
PRR15L	0.0303985314860022	0.318020239882227	0.400407358276717	1081
HBQ1	0.0303857186812624	0.31822435512515	0.400633342066012	1081
TMED2	0.0303847681772015	0.318239500607611	0.400633342066012	1081
OTUD6A	0.030368929586021	0.318491944625553	0.400875990283147	1081
CRISP3	0.0303436486807329	0.318895156623482	0.401333349795717	1081
MXD1	0.0302985983155681	0.31961450392217	0.40211305155584	1081
GAGE12J	0.0302965042506236	0.319647966926758	0.402130037703087	1081
OR5W2	0.0302924740789732	0.319712375215679	0.402164403979387	1081
STX8	0.0302922966369113	0.319715211205256	0.402164403979387	1081

MSL3	0.0302794334813128	0.319920841944946	0.402397937124184	1081
HGC6.3	0.0302723136992811	0.320034696092543	0.402490882306422	1081
DCAF4L2	0.0302624668754258	0.320192202618746	0.402663831809391	1081
TRPV4	0.0302547996958623	0.32031487931968	0.402792961541507	1081
PISRT1	0.0302224165447268	0.320833355898356	0.403339546267819	1081
WNT2	0.0302213993049939	0.320849651473904	0.403339546267819	1081
GPR61	0.0301785455962338	0.32153663298532	0.404077072457223	1081
DGKE	0.0301713898956383	0.32165143834904	0.404177821829492	1081
PIP5K1C	0.0301710476956704	0.32165692924152	0.404177821829492	1081
SCGB1A1	0.0301489615253222	0.322011450076425	0.404547593501066	1081
PPP2CA	0.0301204324697874	0.322469767034561	0.404997098311173	1081
ARL6IP1	0.0300827471921992	0.323075829049283	0.405682389590339	1081
KISS1R	0.0300569673803923	0.323490851928821	0.406178210677268	1081
GOLGA6(	0.0300476264074044	0.323641315562758	0.406341807542023	1081
APEH	0.0300317750151268	0.323896752692298	0.406637173313371	1081
C13orf29	0.0300274804901592	0.323965979352373	0.406694377847611	1081
FABP12	0.0300212139379137	0.324067011862309	0.406759974781089	1081
UGT1A9	0.0300204186525385	0.324079835318772	0.406759974781089	1081
TSEN15	0.0299755991555469	0.324803053783886	0.407597179248514	1081
SCARNA2	0.0299471248404274	0.325263066843104	0.40809818644459	1081
OR51G1	0.0299383732828867	0.32540453647436	0.408224834180844	1081
OR8U1	0.0299363255626829	0.325437643805201	0.408240944919097	1081
SOAT2	0.0298888300838959	0.326206159709612	0.409128569751596	1081
LCE6A	0.0298869852356343	0.326236034617392	0.409140566355436	1081
C5orf47	0.0298809654570719	0.326333529418076	0.409211886095682	1081
RGSL1	0.029878899023628	0.326367001206842	0.409228385367349	1081
PKD1L2	0.0298134429989449	0.327428401297319	0.410436179265643	1081
PI4KAP2	0.0298132145844881	0.327432109058236	0.410436179265643	1081
RNF215	0.0297877731742426	0.327845259684241	0.410902927663724	1081
LOC15052	0.0297839821221235	0.327906852612598	0.410954556792384	1081
MSTO1	0.0297782098927336	0.328000647971666	0.411020966247152	1081
LGSN	0.0297720275859845	0.328101126125127	0.411095732416388	1081
FAM71A	0.0297682653252656	0.328162282155496	0.411146786119938	1081

DDX53	0.0297056991186855	0.329180386213825	0.412294135804371	1081
TIMM22	0.0297000072263335	0.329273108445306	0.412384629756337	1081
SPAG6	0.0296898780639108	0.329438156616316	0.412522170202358	1081
NPC1L1	0.0296894972924351	0.329444362085656	0.412522170202358	1081
OR5AC2	0.0296548059735943	0.330010047134706	0.413127790177023	1081
HTA	0.0296471222505293	0.330135424691991	0.413259064836896	1081
NELL1	0.0296371891140726	0.330297552217315	0.413410636186258	1081
C12orf61	0.0296344257934345	0.330342663971691	0.413441411885544	1081
TWIST2	0.029624851618828	0.330498995061497	0.413611372026705	1081
GLA	0.0296056632184778	0.330812455124843	0.41396422353938	1081
TPD52L3	0.0296036443485436	0.330845446374929	0.413967796100804	1081
PRAP1	0.0295687242582907	0.331416427407156	0.414579230633207	1081
GAGE2C	0.0295475323702867	0.331763247606259	0.414935780904457	1081
ADSSL1	0.029527497990375	0.332091339705331	0.415294558219871	1081
GSTM1	0.0295164724078602	0.332271988929233	0.415468885666898	1081
HES5	0.0295086995976908	0.332399381029787	0.415602379024644	1081
OR6C74	0.0294925618161737	0.332663971020285	0.415881574792333	1081
CCDC77	0.029426853118098	0.333742712091666	0.417178390114583	1081
DHTKD1	0.0294162035958142	0.333917757483414	0.417371298908195	1081
NVL	0.0294071106782362	0.334067263870756	0.417532263970492	1081
LOC11643	0.0293867295561941	0.334402528271836	0.417925363362252	1081
PAX8	0.0293663465698982	0.334738039889068	0.418318722925886	1081
GALR3	0.0292969477243008	0.335881994705066	0.419540103133281	1081
OTX2	0.0292909400221773	0.335981142349991	0.419637926259567	1081
HRNBP3	0.0292357597450699	0.336892685470244	0.420672109886447	1081
SSR2	0.0292321814973792	0.336951850626478	0.420719910181629	1081
NSMCE4A	0.0291993239792263	0.337495450832907	0.421346421481713	1081
FKSG73	0.0291627488706093	0.338101216994541	0.421998080319394	1081
FANCL	0.0291597290219119	0.33815126363458	0.422034397244198	1081
RWDD1	0.0291520896050129	0.338277889580463	0.422166279618289	1081
HS3ST3B1	0.029143885317376	0.338413912307943	0.42230987198436	1081
PMF1	0.029127061130347	0.338692958056305	0.422579563981436	1081
TAS1R3	0.0290666784740256	0.339695678116768	0.423725661650916	1081

PSG7	0.0290593427408919	0.339817625350155	0.423785374831563	1081
C1orf14	0.0290501417257769	0.339970620142958	0.4239111254506	1081
PRAMEF6	0.0290436171098765	0.340079138394252	0.423993949928964	1081
SLC25A18	0.029033016039334	0.340255504034317	0.424187581457473	1081
DDX52	0.0289563267971425	0.341533094148614	0.425588070165891	1081
NKD2	0.0289558792164372	0.341540559523355	0.425588070165891	1081
COQ4	0.0289198697701132	0.342141516955387	0.426195947258612	1081
ID2B	0.0289075461934628	0.342347338710293	0.426399600489604	1081
RAB26	0.0289044672659159	0.342398773645879	0.426437297996676	1081
HSPBAP1	0.0288821455089603	0.342771816670885	0.426828232616577	1081
CKAP5	0.0288675035896493	0.343016654465339	0.427101211706796	1081
RARRES1	0.028860942276472	0.343126406983481	0.427185060792425	1081
MMP8	0.0288578684429297	0.343177831374967	0.427222680326827	1081
KRTAP10	0.0288301689950238	0.343641457331572	0.427773413949485	1081
ANKDD1A	0.0288107310867116	0.343967042241038	0.428152253209696	1081
NR0B1	0.0288087316988741	0.344000543132914	0.42816749715246	1081
ADAMTS	0.0288049480100267	0.344063946705986	0.428193501352449	1081
PRB3	0.0287884099115738	0.344341164362354	0.428512030891995	1081
C21orf57	0.0287406530004755	0.345142481919117	0.429350086344363	1081
COL19A1	0.0287196509000239	0.345495253684564	0.42973585363519	1081
C15orf54	0.0287177463818729	0.34552725517542	0.429749123634552	1081
PPP1R14A	0.0287130959922462	0.345605403288354	0.429819783259902	1081
C9orf21	0.0287039629609719	0.345758913308327	0.429973525238221	1081
MGC1614	0.0287032017100677	0.345771710541533	0.429973525238221	1081
C14orf86	0.0286795635545045	0.346169236723337	0.43044128595714	1081
GOLT1A	0.0286721790484501	0.346293482569925	0.430567827503399	1081
AGPAT3	0.0286709745759976	0.346313750758877	0.430567827503399	1081
SI	0.0286574149847946	0.346541976032126	0.430729186319567	1081
OR2T34	0.0286569121104478	0.346550441889677	0.430729186319567	1081
DHRS2	0.0286525960792487	0.346623107407341	0.430792923605459	1081
TAS1R2	0.0286399361606562	0.346836308069225	0.431031303272951	1081
PROKR2	0.028600992249179	0.347492669489463	0.431703385570815	1081
MAP3K14	0.028584983255844	0.347762714059877	0.431916145174859	1081

PTGDR	0.028559566101559	0.348191732037886	0.432404758693787	1081
LOC40139	0.0285583891715691	0.348211605659025	0.432404758693787	1081
WFDC1	0.0285578611986381	0.348220521235508	0.432404758693787	1081
CEBPZ	0.0285528230258721	0.348305605261744	0.432469380556877	1081
CPT1C	0.0285522373096794	0.348315497619028	0.432469380556877	1081
PSME4	0.0285491692832794	0.348367317459581	0.432481016470531	1081
AATK	0.0285476439221645	0.348393083064622	0.432481016470531	1081
NOMO1	0.028545940441823	0.348421858796272	0.432481016470531	1081
FLJ32063	0.0285459114462168	0.348422348612333	0.432481016470531	1081
DKFZP434	0.0285453276089086	0.348432211333667	0.432481016470531	1081
GUCY2F	0.0285415362570428	0.348496262676901	0.432533868007281	1081
ZFYVE27	0.0285274206645479	0.348734798096681	0.43280325946634	1081
PRPH	0.0285111288242135	0.349010238050928	0.433065059780471	1081
OBSL1	0.0285089434327211	0.34904719612738	0.433084242792934	1081
CCDC79	0.0284949502737202	0.34928389916107	0.433351243985943	1081
DEGS1	0.0284848147613269	0.349455411233646	0.433485722417147	1081
S100G	0.0284650249283675	0.349790446567616	0.433815979307679	1081
LOC64543	0.0284595170323909	0.349883729626638	0.433881670257225	1081
LOC38824	0.0284337562668959	0.350320229438424	0.434342751233201	1081
TOP1P1	0.0284194879475726	0.350562145410639	0.434589195033682	1081
TMEM55A	0.028418011517376	0.350587183972725	0.434593490931668	1081
KDM5D	0.0283771431685911	0.351280713742224	0.43542640772441	1081
ALKBH3	0.0283695806694453	0.351409143340452	0.435558800996333	1081
C3P1	0.0283673128223191	0.351447662680565	0.435579744352159	1081
SPEG	0.0283572827174752	0.351618055850422	0.435737311460799	1081
NECAB2	0.0283496386055456	0.351747950438447	0.435871468387926	1081
GFRA4	0.0283111020291646	0.352403255447811	0.43662977926048	1081
IER3	0.0282957530415202	0.352664475936187	0.436872824236532	1081
JAKMIP3	0.0282737986494714	0.353038324702794	0.43730904821606	1081
KCNQ1DN	0.0282632077983885	0.353218759706526	0.437478752799752	1081
C12orf47	0.028261622918356	0.353245766117736	0.437485304219955	1081
BCAT1	0.0282549622338246	0.353359278669526	0.437598983758383	1081
OR4C3	0.0282499039026759	0.353445499019408	0.437678852824317	1081

PRKRA	0.0282373729227605	0.353659149488168	0.437916501717324	1081
TBC1D3H	0.0282357641627487	0.353686584398107	0.43792355517516	1081
LOC1003(	0.0282214531237978	0.353930696121456	0.438171944036796	1081
USP20	0.0282194186422356	0.353965408087432	0.4381879890568	1081
ACR	0.0282023743125639	0.354256299817231	0.438521147832009	1081
ZNRF4	0.0281869363589172	0.354519906002066	0.438820491945414	1081
REG1B	0.0281662739458883	0.354872914413027	0.439230453480612	1081
ZNF90	0.0281156604771664	0.355738559493345	0.440220731064158	1081
CDH18	0.0280934542862692	0.35611877265988	0.440610040242801	1081
VN1R4	0.0280694410770347	0.356530213304008	0.441064919369272	1081
PLEKHG6	0.0280634956899203	0.356632127334241	0.441120684163936	1081
CDS2	0.0280632611084116	0.356636148835493	0.441120684163936	1081
BMS1	0.028034916215473	0.357122283871232	0.441634647549783	1081
KRTAP13	0.0280099153266316	0.35755141260528	0.442111048792866	1081
OR52N5	0.0279861714574644	0.357959265171534	0.442561028408037	1081
ANPEP	0.0279718410693886	0.358205562145391	0.442811183690408	1081
AGPS	0.027938037134199	0.358786973426467	0.443502704772423	1081
REG4	0.0279213473721805	0.359074247587229	0.443829592466142	1081
CCDC134	0.0279105291797139	0.359260533879332	0.444006346932462	1081
B3GNT8	0.0279061472317255	0.359336007070533	0.444072379758044	1081
PRG2	0.0279042188365116	0.359369224250274	0.444086187125614	1081
OSCP1	0.0278654651995741	0.360037175518402	0.444802459267644	1081
OR14I1	0.0278538389012174	0.360237716175285	0.444995633575614	1081
SCARNA1	0.0278415717854861	0.360449386119917	0.445229804610076	1081
AKR1C4	0.027825465723058	0.36072741569307	0.445520729042756	1081
LOC44046	0.0278097390236826	0.360999026166201	0.445799385895796	1081
EXT2	0.0277522196092325	0.361993515066445	0.446972685321087	1081
DISP2	0.0277063659795222	0.362787533750258	0.447898196200218	1081
RAD21L1	0.0277035631774349	0.36283610341232	0.447930708618776	1081
ACER1	0.0276908195581527	0.363056988415302	0.448148470075138	1081
C21orf2	0.0276715621439853	0.363390936377106	0.448533203438318	1081
TCEB3C	0.0276618830203992	0.363558857095817	0.448685485278149	1081
SNORA67	0.0276469281734406	0.363818400322034	0.448895796808193	1081

NXF2B	0.0276371668367069	0.363987871933328	0.449049891609829	1081
TMEM18(	0.0276352482021641	0.364021188134765	0.44906349265002	1081
CFHR1	0.0276247159501576	0.364204109732805	0.449234128739123	1081
CLCNKB	0.0276172544939066	0.364333733214595	0.449338989568263	1081
CYP2A13	0.0276059518244338	0.364530142671529	0.44955370211925	1081
FLJ43860	0.0275623460632095	0.365288510491365	0.450351104274697	1081
CENPP	0.0275188250748207	0.366046384153713	0.451202619395249	1081
NOL6	0.0275099967801636	0.366200239347113	0.451337034056139	1081
GAGE13	0.0274867853416495	0.366604949088788	0.451808191569413	1081
HNRNPH:	0.0274704681637715	0.366889618602484	0.452048401635905	1081
AKT1	0.0274561149451155	0.367140138603893	0.452329404450824	1081
CFH	0.0274495355707093	0.367255010086755	0.452443259429897	1081
OR8K1	0.0274475021076186	0.367290517518012	0.452459333174363	1081
SEC22A	0.0274446788801984	0.367339819005551	0.452492396592077	1081
PAQR4	0.0273979193889009	0.36815696994506	0.453443516724336	1081
C16orf11	0.0273780275594346	0.368504934280838	0.453788856353535	1081
BMP7	0.0273763692589467	0.368533951871151	0.453796849660412	1081
PRG1	0.02736872559149	0.368667722108386	0.453933822008034	1081
SLC46A3	0.0273545161173057	0.368916479325984	0.454212350325262	1081
C3orf65	0.0273395560866755	0.369178488749352	0.454479384982627	1081
MORF4L2	0.027330473450396	0.369337618066878	0.454619718682467	1081
RAB5C	0.0273209456923136	0.369504591754795	0.454797458355248	1081
LAPTM4A	0.0272814226736391	0.370197731778465	0.455567092649797	1081
TRIM7	0.0272213218108202	0.371253303010327	0.456810272974776	1081
LOC6429:	0.0272167066313951	0.371334437956587	0.456882199513556	1081
KCND1	0.0272068546562679	0.371507672628636	0.457067427940875	1081
CCDC96	0.0272002188742012	0.371624382775429	0.45718309563426	1081
XKR3	0.0271812922122389	0.37195738967841	0.457536888018048	1081
MTBP	0.0271325342780948	0.372816116361284	0.458369280689537	1081
ESRP1	0.0271148896391212	0.373127176405964	0.458695732513465	1081
ZNF16	0.0271104042333491	0.373206275889078	0.458764976066968	1081
ZFAT	0.0270879735337791	0.373601993589334	0.459195372840582	1081
PRIM2	0.0270801113417689	0.373740758090189	0.459309881144879	1081

OR10S1	0.0270726052599445	0.373873267105831	0.459444699923563	1081
TFF1	0.0270497828934918	0.374276341250637	0.459799786223569	1081
DMRTA2	0.0270289830589446	0.374643928392738	0.460170746117131	1081
OR6X1	0.0270288188753562	0.374646830829837	0.460170746117131	1081
PRSS27	0.0270262644929115	0.374691988984911	0.460198153767896	1081
C9orf123	0.0270172194772015	0.37485192009768	0.460366514074728	1081
HIST1H1A	0.0270087542472674	0.375001637785548	0.460522311216158	1081
RNF216L	0.0270058228648712	0.375053491380503	0.460535704462287	1081
P4HTM	0.0270055529623804	0.375058265942332	0.460535704462287	1081
ZNF716	0.0269985334980636	0.37518245309454	0.460660115657311	1081
OR52N2	0.026983719600661	0.375444621273558	0.460945961210609	1081
TMEM106	0.0269827931144149	0.37546102146959	0.460945961210609	1081
TSPY1	0.0269455080767901	0.376121389236357	0.461672275540239	1081
TATDN1	0.0269287732204197	0.376418018506781	0.461923792813055	1081
TFAP4	0.026922603496716	0.376527414671762	0.462029893924751	1081
PLCD4	0.0269135019369235	0.376688831358354	0.462171661350512	1081
UNK	0.0269066829614929	0.376809794207972	0.462291918655274	1081
EIF3J	0.0268981714621501	0.37696081456422	0.462449035344754	1081
OR12D2	0.0268930285523201	0.377052083773937	0.462504671961637	1081
NPFF	0.0268746553699389	0.377378256584561	0.462848400541612	1081
OOEP	0.0268573164552448	0.377686227638167	0.463145244091331	1081
FAM99A	0.0268465717402247	0.37787715115433	0.463319245891904	1081
CD99L2	0.0268083978287191	0.378555945881032	0.464010315167227	1081
B4GALNT	0.0267863837730947	0.378947732451164	0.464434025079315	1081
KRTAP4-1	0.0267707812981311	0.379225562241835	0.464699274108759	1081
NDRG1	0.0267703434086531	0.37923336144691	0.464699274108759	1081
GFRA3	0.0267659714452789	0.379311235446233	0.464766427715125	1081
OR5K4	0.0267587077386532	0.379440639280555	0.464882539547329	1081
LPIN1	0.0267448704666382	0.379687227011287	0.465142251772219	1081
OR51A7	0.026735147572855	0.379860553125858	0.465326290223194	1081
C3orf32	0.0267224506983191	0.380086968317807	0.465575336055103	1081
DDX1	0.0266825242906503	0.380799489839762	0.466326508545983	1081
GLE1	0.0266816022607034	0.380815953938444	0.466326508545983	1081

SNORA45	0.0266708109937357	0.381008679138805	0.466534150331341	1081
VAX1	0.026668761089922	0.381045295881863	0.466550628185749	1081
MYO18B	0.0266340352102256	0.381665920418899	0.46722532613264	1081
GSTTP1	0.02663020378815	0.381734433909701	0.467280802765445	1081
MTTP	0.0266067304285417	0.382154349007704	0.467652737259155	1081
CCDC81	0.0266016122784713	0.382245945179308	0.467732542450263	1081
SGSM3	0.0266004917440449	0.382266000441853	0.467732542450263	1081
LOC14648	0.0265709789963898	0.382794449881352	0.468265374839679	1081
AKAP14	0.026509861596496	0.383890226596059	0.46932505860018	1081
LOC44092	0.026509668785009	0.383893686553177	0.46932505860018	1081
SLC26A8	0.0264603341888671	0.384779610638723	0.470293972595381	1081
PDE6B	0.0264520502456516	0.384928491543851	0.470447398055626	1081
INTS8	0.026436823481067	0.385202242128732	0.47075340740617	1081
PPP1R2	0.0264149059431607	0.385596489663422	0.47120662931572	1081
MAGEH1	0.0264036864331002	0.385798398806123	0.471405847432817	1081
PCGEM1	0.0264032466509196	0.38580631455075	0.471405847432817	1081
CELA3A	0.0263926622893964	0.385996854885872	0.471581456830366	1081
ANXA2P2	0.0263884664773592	0.386072404035874	0.471645153384031	1081
ZNF705D	0.0263818931978257	0.386190779628003	0.471761157941066	1081
GPR50	0.0263744901665212	0.386324124383761	0.471895433283463	1081
MFAP1	0.0263385602259838	0.386971699637138	0.472600478277669	1081
OR5B3	0.0263334620517714	0.38706363898008	0.472684105677359	1081
EPB41L1	0.0263098616709813	0.38748941633957	0.473146702155451	1081
CT47B1	0.0262891553616549	0.387863216142961	0.473488334092835	1081
PNCK	0.026251469401934	0.38854410329934	0.474207585182632	1081
GLTP	0.0262501075382583	0.388568722229898	0.474207585182632	1081
LGI3	0.0262178025302089	0.389152990308547	0.474832646260793	1081
DKKL1	0.0262006117228461	0.389464120616735	0.475079541166092	1081
MUC4	0.0261934215174416	0.389594298394874	0.475198422206632	1081
DMRT3	0.0261825100658538	0.389791899353222	0.47532432278878	1081
NRBF2	0.026177314228806	0.38988601476376	0.475381538	1081
OR52A1	0.0261501411083677	0.390378443043988	0.475953140175582	1081
BAGE	0.0261473077961244	0.390429809735345	0.475986959399571	1081

LECT2	0.0261390902065366	0.39057881417914	0.476139801006884	1081
HAPLN2	0.026130688670478	0.390731189675142	0.476296733390157	1081
C19orf51	0.0261265575184778	0.390806128047429	0.47635925754919	1081
LOC34050	0.0261224597755173	0.390880468997701	0.476421045980075	1081
PEX11G	0.0261183587575512	0.390954877949949	0.47648290981316	1081
LOC40079	0.0261124632149653	0.391061861843644	0.476555635281605	1081
SUPT6H	0.0261044666087994	0.391207001209146	0.476703667998927	1081
CLTC	0.0261005050964257	0.391278915232451	0.476749065838851	1081
RASA3	0.0260998070009952	0.391291588710824	0.476749065838851	1081
RPL27A	0.0260771140653169	0.39170370004195	0.47722318557308	1081
MSH6	0.0260017262976082	0.393074654374506	0.478747818160485	1081
CHTF8	0.0259903989081255	0.39328089772782	0.478970055300583	1081
CHGA	0.0259803866346874	0.393463250608364	0.479163171533399	1081
INSL4	0.0259474609794986	0.394063283910874	0.479806881498008	1081
ZNF7	0.0259143705618539	0.394666876710268	0.480483727039846	1081
DDHD1	0.0259092969842031	0.394759471730548	0.480538374404006	1081
CPA2	0.0259056203559778	0.394826580008308	0.480591024427031	1081
C10orf111	0.025900311944679	0.39492348483578	0.480679934868998	1081
CIZ1	0.0258615374801585	0.395631746543356	0.481396565240149	1081
LOC10013	0.0258389378895868	0.396044908226088	0.481811986002449	1081
KIRREL3	0.0258372979315023	0.396074899769912	0.481819375354159	1081
EIF4G1	0.025834631669186	0.39612366328295	0.481849598263089	1081
CLSTN1	0.0258225210932674	0.396345200266574	0.482060862124624	1081
CATSPER	0.0257982916087053	0.396788650813399	0.4825263091296	1081
CLP1	0.0257976857184429	0.396799743710149	0.4825263091296	1081
LOC72841	0.0257887383299794	0.396963578059938	0.482696401280706	1081
C15orf39	0.0257822619190297	0.397082192115327	0.482782350181862	1081
FNDC7	0.0257559468920215	0.39756436566906	0.483310249616995	1081
KLHL36	0.0257459376414228	0.397747858926686	0.483504140603312	1081
YEATS2	0.0257350776092122	0.397947006675145	0.483717036714584	1081
KLHL34	0.0257315940891768	0.398010899044287	0.483765509909929	1081
LAMP2	0.0257176528145654	0.398266662256523	0.483993963916256	1081
CHRDL2	0.0257174200831109	0.398270932729699	0.483993963916256	1081

DSCR3	0.0257145849376053	0.398322958046177	0.484027988288107	1081
DPPA3	0.0256875929152776	0.398818470129912	0.484571657464842	1081
CPLX4	0.025682467654317	0.398912600101003	0.484627567786438	1081
APC2	0.0256780578791424	0.398993600227846	0.484696742241449	1081
OPN5	0.0256646937029793	0.399239137959837	0.484936535073918	1081
UCN2	0.0256573773461002	0.399373598591453	0.485056587013187	1081
FA2H	0.0256456079722368	0.399589954229406	0.485274875087496	1081
KIAA0391	0.0256348604718305	0.399787586321289	0.485456354818708	1081
MED16	0.0256292129082079	0.399891460951469	0.485553220472988	1081
POM121L	0.0256124463899529	0.40019993999333	0.485839929562832	1081
KRTAP24	0.0256069445811217	0.400301196225403	0.485933570229632	1081
TMEM20(	0.0256040985078	0.400353581880277	0.485967878222353	1081
POU5F1B	0.0255786116726407	0.400822883366623	0.486449604519587	1081
ACAN	0.0255700423196457	0.400980749127602	0.486611878986478	1081
RAVER2	0.0255658349150358	0.401058272142013	0.486676639497672	1081
NR1I2	0.0255492275995462	0.401364355928689	0.487018729682174	1081
OR10J5	0.0255466897261322	0.401411142943114	0.487046164824974	1081
CSNK1D	0.025542129128962	0.401495228136433	0.487118849121202	1081
PRSS50	0.0255356245711188	0.401615173021559	0.487205688336704	1081
AWAT1	0.0255255764062254	0.401800504682934	0.487359631368026	1081
BCAN	0.0255249565020296	0.401811940078015	0.487359631368026	1081
SETD8	0.0255248096191952	0.401814649659546	0.487359631368026	1081
LOC3744(	0.0255155094670982	0.401986234001613	0.487479694435498	1081
VAV2	0.0255018410311665	0.402238491198905	0.487707314764124	1081
GJD2	0.0254933498015838	0.402395248429735	0.487858225783402	1081
LOC15391	0.0254899543180389	0.40245794294503	0.487904872156685	1081
OR8G5	0.0254859293170453	0.402532268502435	0.487942231171707	1081
PDZK1	0.0254856621509163	0.402537202275632	0.487942231171707	1081
OR6C1	0.0254828257403513	0.402589584675008	0.487947005792554	1081
IL11	0.025452433334441	0.403151123093123	0.488568812772121	1081
POU5F1	0.025425028515578	0.403657863576414	0.489065234964025	1081
CRH	0.0254107605573069	0.403921842030725	0.489292518172153	1081
DNAJB3	0.0254068434821088	0.403994331798959	0.489305717963652	1081

APCS	0.0254064245408309	0.404002085226578	0.489305717963652	1081
LOC15077	0.0253899353942599	0.404307323700078	0.489587102424746	1081
RIPPLY1	0.0253823373018547	0.404448021983469	0.48969860577335	1081
RGS22	0.0253655646100923	0.404758715177687	0.490045334290192	1081
MAGEB3	0.0253460927109245	0.405119586547244	0.490393827123797	1081
HGFAC	0.025305884652212	0.405865367228806	0.491149025219217	1081
MAGEB1	0.0253011590960856	0.405953070774288	0.491225649372178	1081
IL4	0.0252921629837418	0.406120064569907	0.491398204142037	1081
PPM1H	0.0252897483387924	0.406164894317066	0.491422930691729	1081
RPL23AP8	0.0252715076639116	0.40650364184386	0.491735102627553	1081
CXorf22	0.0252506286746807	0.406891592103042	0.492124827264397	1081
VSX2	0.0252354849200855	0.407173114540248	0.492435756283442	1081
RAB11B	0.0252241928515276	0.407383109601304	0.49263057463943	1081
ADH7	0.0252113418822385	0.407622173485244	0.492811544916064	1081
TMEM102	0.0251970132255361	0.407888824914834	0.493064529127473	1081
FBXO7	0.0251621257221425	0.408538503375475	0.493709333731568	1081
TMEM194	0.0251617888300744	0.408544780010188	0.493709333731568	1081
PMCHL1	0.0251564876745356	0.40864355336662	0.493799075194683	1081
C22orf42	0.025149453715702	0.408774634957277	0.493873816289993	1081
RHOBTB3	0.0250966546979833	0.409759370661738	0.494969270371213	1081
WFDC11	0.025078750210809	0.410093620441183	0.495343325565873	1081
SLC25A3	0.0250764179200963	0.410137172702758	0.495366229635893	1081
PSG10	0.0250594523892186	0.41045406283016	0.495630108236742	1081
ENPP7	0.0250401225639943	0.410815291342746	0.495977109199305	1081
SLC9A6	0.025035187584626	0.410907544586437	0.496014346883761	1081
GRHL3	0.0250332042525098	0.410944623950855	0.496014346883761	1081
USP39	0.0250285729675041	0.411031215815725	0.496070580819286	1081
NINJ2	0.0249886539351668	0.411778036642887	0.496881589596106	1081
FSHR	0.0249761667197109	0.41201181729712	0.497093972537012	1081
C20orf56	0.0249654850005116	0.412211858538618	0.49730446814511	1081
ATP8B4	0.0249305927187175	0.4128657022314	0.497975067144746	1081
INSL6	0.0249279032375911	0.412916125663436	0.498006067861457	1081
CEP72	0.0249237270723783	0.412994429244203	0.498070688285708	1081

ZCCHC3	0.0249012849569186	0.41341537192475	0.498518656017245	1081
TTC9	0.0248759128802713	0.413891576758178	0.499063016328096	1081
SPATS2	0.0248405759051486	0.414555351190039	0.49977364151546	1081
RXFP2	0.0248374696557409	0.414613729433455	0.499814109235651	1081
SNORA16	0.024817414045259	0.414990767485723	0.500230714085214	1081
SFRS16	0.0247632056032078	0.416010878298375	0.501228417662725	1081
OR5AN1	0.0247404275290926	0.416439963672046	0.501625392739379	1081
BTBD17	0.0247188263707323	0.416847119530516	0.502025780052744	1081
CLU	0.0247151418146952	0.416916592379207	0.502049742497069	1081
ST8SIA3	0.0247090181478383	0.417032070105054	0.502128447329288	1081
WDR73	0.0247044796472049	0.417117667533679	0.502201494887997	1081
C9orf114	0.0246772420830297	0.417631593117995	0.502790201599355	1081
PARS2	0.0246619021616087	0.4179211947554	0.503108788594928	1081
GTDC1	0.0246566112449	0.418021109108223	0.503168931822726	1081
ANGPT2	0.02465017967548	0.418142582610491	0.503285076578141	1081
GYPC	0.0246336666733203	0.418454559890341	0.503630488050599	1081
LOC65311	0.024608831919772	0.418924016125073	0.504105149324388	1081
XPO5	0.0245912583331653	0.419256399964009	0.504384603002387	1081
NXPH4	0.0245847644336595	0.419379263659161	0.504502285107716	1081
VPS37D	0.0245598757008035	0.419850350858658	0.505008676740367	1081
SAMM50	0.0245314592123769	0.420388589992745	0.505595710173364	1081
COL9A3	0.0245168693655132	0.420665094691227	0.505837660869979	1081
RPL3L	0.0245131817116446	0.420734999460424	0.505891522271114	1081
C16orf73	0.0245103233303706	0.420789188863116	0.505926482223066	1081
SPPL3	0.0245026625624918	0.420934442441385	0.506040719877167	1081
PI4K2B	0.0244858279616018	0.421253741617926	0.506333927499589	1081
BARHL2	0.0244294821764371	0.422323474963953	0.507559145874528	1081
CNNM2	0.0244169646668194	0.422561337420367	0.507814719461511	1081
BFSP1	0.0244134923371385	0.422627333826995	0.507863734189025	1081
MCF2L2	0.0243972446495062	0.422936223607907	0.508204606297721	1081
KCNG2	0.0243907298759533	0.423060115039834	0.508313226979047	1081
LUC7L3	0.024388717713255	0.42309838462128	0.508313226979047	1081
VSIG1	0.0243700506844096	0.423453511720064	0.508613820271923	1081

RLN1	0.0243624002525762	0.42359910611826	0.508758361220554	1081
SNORA46	0.0243303164580201	0.424210007211038	0.509400965383069	1081
OR10K2	0.0242967637178709	0.424849428159236	0.51004718297186	1081
DEFB109I	0.024262637083076	0.425500362412793	0.510767775045032	1081
KIAA1875	0.0242424682283866	0.425885338021991	0.51113906090931	1081
ARHGAP1	0.0242411159050708	0.425911157926219	0.51113906090931	1081
LOC10028	0.024227410323236	0.426172890015928	0.51142270026633	1081
IL17F	0.0242194741792593	0.42632448741005	0.511574148134111	1081
CNKSR1	0.024207589499508	0.426551569043925	0.511785667563422	1081
PRSS3	0.0241628728996206	0.427406603875419	0.512703285320097	1081
TLR10	0.0241303737637904	0.428028651308825	0.513343881682222	1081
DPP4	0.0241241013774244	0.428148768034736	0.513457371833538	1081
ECT2	0.0241209672596593	0.428208793992425	0.513498788914662	1081
WDHD1	0.0240923728266387	0.428756672559892	0.514063990520118	1081
GTF2IRD1	0.0240870205551277	0.428859269023517	0.514154440394367	1081
SGSH	0.0240857744212012	0.428883157924395	0.514154440394367	1081
GHRHR	0.0240718483140208	0.429150179701645	0.514399502465094	1081
ACTL7B	0.0240540876252785	0.429490866443013	0.514760441723852	1081
ABHD1	0.0240203377149853	0.430138691790487	0.515475547062424	1081
HIGD2B	0.0240037778787505	0.430456762875645	0.515764675437451	1081
ENDOD1	0.0239870896290052	0.430777438313005	0.516056820576563	1081
CCDC47	0.0239404327656458	0.431674713951947	0.517070229073729	1081
THEM5	0.0239373529622023	0.431733980838236	0.517110472916717	1081
PIWIL3	0.0239134741049519	0.432193658732281	0.517537969158997	1081
NCRNA00	0.0238951530970662	0.432546537556934	0.517929743169545	1081
KRTAP6-3	0.0238913098980579	0.432620582100867	0.517987615240532	1081
IMMT	0.0238738033095098	0.432957963435826	0.518329956225989	1081
FAM116B	0.0238637089248015	0.433152568252748	0.518532118341551	1081
DEFB4A	0.0238455464038922	0.433502842038382	0.518920598541994	1081
HIATL1	0.0238409971759115	0.433590601906026	0.518963975723183	1081
DHX34	0.023832664399181	0.433751377405092	0.519125564271957	1081
PSTPIP2	0.0238300325617931	0.433802164143805	0.519155503871974	1081
ACTR1B	0.0238280867882945	0.433839714069606	0.51916959961579	1081

SPO11	0.0238242029897174	0.43391467000033	0.519228454241559	1081
ANKRD3	0.0238105268843697	0.434178673463075	0.519451798130041	1081
LRFN3	0.0237738340429986	0.434887449606758	0.520268880119255	1081
HDC	0.023765453282277	0.435049429840607	0.520402615420999	1081
FSCN2	0.0237640406493946	0.435076736109231	0.520402615420999	1081
TBC1D28	0.0237583704590191	0.435186351130243	0.520502822746468	1081
KIF3C	0.0237326814516413	0.435683165390139	0.520973310784374	1081
SNORA11	0.0237193549774756	0.435941022251908	0.521189498126204	1081
PSG2	0.0237179895250804	0.435967447650765	0.521189498126204	1081
SLC44A2	0.0236988391288009	0.436338159605445	0.521539825872296	1081
EDDM3A	0.023649722882271	0.437289777136031	0.522615244447398	1081
RASAL1	0.0236365796398601	0.437544627039742	0.522888800600084	1081
SPINK6	0.0235687235575428	0.438861726113623	0.524214021497713	1081
DPAGT1	0.0235496389488479	0.439232571331938	0.524625883403408	1081
RNU4AT	0.0235243741191714	0.439723784692074	0.525025820449373	1081
SDC1	0.023484157705007	0.44050634447599	0.52586668500556	1081
CCT6A	0.0234587640578257	0.441000882703505	0.52639466623583	1081
SCEL	0.0234435964893666	0.441296420771145	0.52668501667366	1081
LOC3897	0.0234304973276924	0.441551747355205	0.526927311798507	1081
OR51G2	0.0234188573473394	0.44177870270175	0.527166921331996	1081
KIAA040	0.023413390613795	0.441885315619128	0.527262908437323	1081
HBEGF	0.0233925080988996	0.442292704388105	0.527624002481103	1081
AADACL	0.0233890758137614	0.44235968401643	0.527643190622126	1081
RLN3	0.0233889994745392	0.442361173810726	0.527643190622126	1081
PLAC4	0.0233656157682973	0.442817652263081	0.528043478346324	1081
VSTM2B	0.0233486306119942	0.443149391785274	0.52839564945335	1081
TNFAIP8	0.0233410130656842	0.443298217234314	0.528541819731608	1081
ARHGDI	0.0232782460239284	0.444525594058447	0.529723045980918	1081
C10orf120	0.0232742778431951	0.444603254788523	0.529784252201278	1081
OR2AG1	0.0232452048362253	0.445172474414772	0.53036841309868	1081
MINPP1	0.0232421566449067	0.445232178892808	0.530408175268812	1081
CCT4	0.0232247130893547	0.44557393093771	0.530689774077105	1081
RAD9B	0.0232222947283211	0.445621322955961	0.530714841912376	1081

PRR23B	0.023200283081481	0.446052811551281	0.531197321393979	1081
OCM	0.0231875492095919	0.446302538785009	0.531461709198421	1081
LHFPL2	0.0231862723076701	0.446327584820992	0.531461709198421	1081
OR6C4	0.0231840875599459	0.446370439824597	0.531481323380017	1081
PRODH2	0.0231719702056457	0.446608170719442	0.531701530591759	1081
INSM2	0.0231447402912837	0.447142658029583	0.532274942150088	1081
TP53INP2	0.0231284105642514	0.44746336321806	0.532625233516179	1081
NCRNA0(	0.0231259734267631	0.447511238205446	0.532650747039806	1081
KRTAP10	0.0231157285007303	0.447712520738686	0.532827360446673	1081
OR5B21	0.0231069425329385	0.447885179971764	0.532969871258266	1081
ADI1	0.0230567050038348	0.448873158347432	0.534050896226614	1081
CXCR4	0.0230344088797069	0.449312032498613	0.534541480817608	1081
NHEJ1	0.0230155155205257	0.449684117209426	0.534952553512689	1081
LBX1	0.0229999661257678	0.449990476905614	0.535285394583028	1081
ARPC1A	0.0229850458239857	0.450284553006274	0.535508726625621	1081
CEBPD	0.022976646663988	0.450450146552706	0.5356601683063	1081
ERO1L	0.0229758896178803	0.450465073778954	0.5356601683063	1081
IRF2BP1	0.0229544567824088	0.450887796586235	0.536099549207915	1081
C3orf20	0.0229280408851731	0.451409109402829	0.536585555773176	1081
ABCA12	0.0228146239491513	0.453651237008178	0.538971701434876	1081
C11orf88	0.0228116982258348	0.453709158216288	0.538985031399425	1081
TBX2	0.0228113542953262	0.453715967360211	0.538985031399425	1081
CTTN	0.0227966493733873	0.454007149575454	0.53926733656825	1081
C9orf79	0.0227900970345653	0.454136930800866	0.539389686397232	1081
UGT2B7	0.0227862386093697	0.454213363839819	0.539448662572406	1081
GPAT2	0.0227815183165467	0.454306879796753	0.539527919206837	1081
SLC18A3	0.022779428640043	0.454348282828492	0.53954528163051	1081
GFI1B	0.0227666170752373	0.454602166445311	0.539783133504703	1081
CYP2C19	0.0227624328516546	0.454685101460871	0.539849788939073	1081
OR4K2	0.0227529273705318	0.454873540143695	0.540009868948951	1081
OR9Q2	0.0227201595646097	0.455523472730874	0.54062214906112	1081
CEACAM	0.0227140007333042	0.455645688226166	0.540682216470579	1081
PDLIM7	0.0227135514965709	0.455654603575132	0.540682216470579	1081

CLEC10A	0.0227031098806672	0.455861850776494	0.540836480434672	1081
RNASE10	0.0227022940875411	0.455878045025477	0.540836480434672	1081
OR2T6	0.022694678633587	0.456029234427957	0.540889746000154	1081
OR6N2	0.022693920073297	0.456044295647189	0.540889746000154	1081
APLN	0.0226530342926862	0.456856496317827	0.541759775474408	1081
CYP4F11	0.0226529320220337	0.456858528953052	0.541759775474408	1081
ZNF334	0.0226470681240405	0.456975082756152	0.541866092302236	1081
DOCK8	0.022630097226121	0.457312498894574	0.54217045022547	1081
SPRR2A	0.0226192450256447	0.457528336034168	0.54239441707811	1081
SEPT5	0.0225825050112085	0.45825947426874	0.543165280603893	1081
OR51F2	0.022575249387628	0.458403940877266	0.54327258436125	1081
FHL3	0.0225629693319764	0.458648506975979	0.543469205220907	1081
GOT1L1	0.0225608029026988	0.458691660544623	0.543485678253819	1081
CES7	0.0225486431191194	0.458933916054093	0.54373456920789	1081
UPF1	0.0225328303841915	0.459249055223826	0.544018124043513	1081
ACCN4	0.0225305550034137	0.459294412280497	0.544039862138568	1081
VENTXP1	0.0225280868846958	0.459343614175753	0.544066151668071	1081
SLC13A3	0.0225240775016293	0.459423547427758	0.544128835494866	1081
OR51T1	0.0225094363573908	0.459715507344072	0.544442615840467	1081
OR4F17	0.022494531019292	0.460012842088457	0.544762724347303	1081
LOC2837(	0.0224735518726163	0.460431520442619	0.545226485969702	1081
C15orf55	0.0224721673438142	0.460459158805356	0.545227164764233	1081
ATG16L2	0.0224695070379777	0.460512267197455	0.545258000628466	1081
MSMB	0.0224588099114655	0.460725851306728	0.545446772130585	1081
POM121L	0.0224409836400317	0.461081902297556	0.545804143952537	1081
EPO	0.0224319418231618	0.461262556673945	0.545985910112029	1081
IRX3	0.0224257712224382	0.461385867178553	0.546099782274498	1081
TAS2R41	0.0224030547661236	0.461839981041431	0.546506039166144	1081
ZP4	0.0224018162680389	0.461864746428573	0.546506039166144	1081
JUP	0.0223937916192055	0.462025227704545	0.546638219567923	1081
HOXB2	0.0223850744751772	0.462199593091154	0.546773786060326	1081
NAA40	0.0223827551587875	0.462245991582924	0.546796564911215	1081
KRTAP10	0.0223772346734974	0.46235644068276	0.546895102903775	1081

ARSI	0.0223575352364124	0.462750689832732	0.547329300533137	1081
AGAP7	0.022353863700563	0.46282418977682	0.547384095758471	1081
OR8B3	0.0223503358095076	0.462894820240482	0.547435491002966	1081
HINT1	0.0223337349321909	0.463227260169047	0.547796487003548	1081
XKR9	0.0222638723602779	0.464627744435757	0.549162515351932	1081
LOC15482	0.0222479688579543	0.464946878778641	0.549507472748356	1081
SIM2	0.0222464468453781	0.464977427267703	0.549511338279237	1081
KRT25	0.0222435186262245	0.465036203025228	0.549548560447189	1081
DDIT4	0.0222413172817513	0.465080391535805	0.549568541030548	1081
ITGB3BP	0.0221819295264399	0.466273386316059	0.550849012862831	1081
TPST1	0.0221631360799935	0.466651266444008	0.551198461595692	1081
IQCF2	0.0221459764488367	0.46699644357959	0.551541499437817	1081
FKSG83	0.0221278801569125	0.467360615411878	0.551906887711606	1081
KRTAP3-1	0.0221260468764971	0.467397517323513	0.551918111751593	1081
DNAJB6	0.022099653368741	0.467928968244796	0.552480897039704	1081
TMEM202	0.0220700555075573	0.468525338391199	0.553152607506928	1081
SLC25A35	0.0220508890547332	0.468911749108509	0.553516435739924	1081
LOC73227	0.0220501371463251	0.468926911760025	0.553516435739924	1081
HOXC10	0.0220493189867459	0.468943410711009	0.553516435739924	1081
SCARNA2	0.0220304240098279	0.469324534762923	0.553933838340701	1081
RABEP2	0.0220282657967772	0.469368078214613	0.553952776122408	1081
LOC93622	0.0220068492472376	0.469800292899629	0.554281635127021	1081
RFTN1	0.0220062786973357	0.469811810364551	0.554281635127021	1081
TMPO	0.0220015290624388	0.469907695410838	0.554352031315351	1081
LOC28562	0.0220005969174476	0.469926514706986	0.554352031315351	1081
PCGF6	0.0219908850404034	0.470122614830798	0.554550888614968	1081
MBD3L5	0.0219611854154755	0.470722583253363	0.555193585458958	1081
ATP6AP2	0.0219361237752113	0.471229186918757	0.555758560416741	1081
HOXD12	0.021928353189988	0.471386324881249	0.555878798497498	1081
JPH1	0.0219257837178693	0.471438291487505	0.555907538165289	1081
OXNAD1	0.0219159084520244	0.471638044381332	0.556110529942168	1081
MTIF2	0.0218883283168644	0.472196170948749	0.556605726375807	1081
DRP2	0.0218609326460024	0.472750924129072	0.557194439888847	1081

ETNK2	0.0218464244074389	0.473044856166187	0.557466241153801	1081
PLEKHG7	0.021827226151392	0.473433960707542	0.55783629516602	1081
MOCS2	0.0218241962118482	0.473495386678423	0.557855392346831	1081
SMAP1	0.0218224339872569	0.473531114274437	0.557855392346831	1081
LOC28366	0.0218223289217537	0.473533244434391	0.557855392346831	1081
PRR15	0.0218099555590256	0.473784146086338	0.558118333600916	1081
C2CD2L	0.0218066978234167	0.473850217163526	0.558163526181688	1081
CDC42EP	0.0218013254982511	0.473959185890011	0.558259241054451	1081
DCP1B	0.021782953872938	0.47433192783055	0.558632954466321	1081
SCARNA	0.0217578232715608	0.474842062997783	0.559168373127394	1081
OR5L2	0.0217516665074628	0.474967087235606	0.559282906965238	1081
CGB7	0.0217336484105918	0.475333080957774	0.559681157045669	1081
SLC25A1	0.021727407896636	0.475459877805458	0.559765018899594	1081
B3GALT6	0.0217257473283876	0.475493620906852	0.559772030923827	1081
SNRNP70	0.0217192116148275	0.475626440742761	0.559895672807977	1081
COLEC11	0.0216986216145868	0.476045006892128	0.560290176667558	1081
IRX6	0.0216889730331802	0.476241218528702	0.560399170376752	1081
HSP90AA	0.0216885752514821	0.476249308688148	0.560399170376752	1081
ZSCAN16	0.021688034187273	0.476260313075135	0.560399170376752	1081
RPSAP9	0.0216858605909712	0.476304521975217	0.560399170376752	1081
IGFBP7	0.0216778148041203	0.476468185234416	0.56051959755405	1081
OR2T10	0.0216777431651557	0.476469642615573	0.56051959755405	1081
SRPR	0.0216762995690471	0.476499010809369	0.56051959755405	1081
SPATA9	0.0216753575965423	0.476518174619542	0.56051959755405	1081
TGM4	0.0216512189423752	0.47700940344608	0.560999149537117	1081
KRT83	0.0216120952812322	0.477806169267181	0.561805012542308	1081
LOC1001	0.0216044626930407	0.477961694038855	0.561955079164978	1081
IFNA21	0.0215924736704523	0.478206043114272	0.562209555794398	1081
DNMT1	0.0215875995817011	0.478305401735755	0.562293552635784	1081
FAM20C	0.0215787073052003	0.478486700403594	0.562473862381421	1081
DIO1	0.0215734193853245	0.478594530034483	0.562567791314311	1081
HEATR6	0.0215627202233561	0.478812744585215	0.562791454557976	1081
SEMA4G	0.0215507096581806	0.47905777046704	0.563046603620684	1081

NANOS1	0.0215490028245469	0.479092596891305	0.563054685512826	1081
KRT1	0.0215440385880787	0.479193895571863	0.563140883578065	1081
HMX3	0.0215387398888513	0.479302032076925	0.563235106818507	1081
BARX1	0.0215125145512192	0.479837437753069	0.56383138028089	1081
RAB9BP1	0.0215011822060828	0.480068894986208	0.564070451414241	1081
POLE3	0.0214915240863807	0.480266205009739	0.564236467015874	1081
TMEM183	0.0214650955298802	0.480806351337638	0.564739312442232	1081
USP50	0.0214491390920123	0.481132628616022	0.565089599082892	1081
BACE2	0.0214278063219542	0.481569029139053	0.565503240571625	1081
PTTG1IP	0.0214051699333167	0.482032332649003	0.566013624901286	1081
KIF21A	0.0213555625488323	0.483048502864744	0.5670752966323	1081
GART	0.0213537982040401	0.48308466553127	0.56708470293849	1081
MPP2	0.021348670093958	0.483189781515422	0.567175046534679	1081
OR7G1	0.0213436587977838	0.483292515028399	0.567262583187502	1081
GABBR2	0.0212782719908972	0.484634054530116	0.568648569255358	1081
PARP3	0.0212778507002737	0.484642704668416	0.568648569255358	1081
HSFY2	0.0212724457935876	0.484753688179846	0.568738461604111	1081
DNHD1	0.0212713699126542	0.484775781792216	0.568738461604111	1081
PKDCC	0.0212587424903656	0.485035131291056	0.569009592351405	1081
C20orf70	0.0212368701103085	0.485484536737769	0.569443194971225	1081
VPS52	0.0212366264470192	0.48548954448403	0.569443194971225	1081
KLHL25	0.0212349963606027	0.485523046593304	0.569449334747445	1081
C1QBP	0.0211876360355208	0.486496957990606	0.570458743813781	1081
TPRX1	0.0211788876196939	0.48667697456335	0.570588344034116	1081
SLC17A8	0.0211731017457714	0.486796050515206	0.570676585057543	1081
CACNA1S	0.0211689529840189	0.486881443620749	0.570743478716322	1081
PRDXDD1	0.021147666286616	0.487319710397393	0.571157526527547	1081
PMS2L5	0.0211194264501092	0.487901461845641	0.57177283006867	1081
FLJ12825	0.0211149519457163	0.487993672677591	0.571847625855975	1081
C4orf17	0.0210930570007109	0.488445020240535	0.57227666236743	1081
TOX4	0.0210886852762242	0.488535166910752	0.572348993161797	1081
OR4F6	0.0210748259078521	0.488821011704608	0.572617275152022	1081
CFD	0.0210679569564104	0.488962714857774	0.572749964577849	1081

CPLX1	0.021059189191283	0.489143621724319	0.572861941726434	1081
NEURL2	0.0210489587399601	0.489354753958947	0.573042580858164	1081
ZSCAN1	0.0210362395371443	0.489617316437363	0.573250077858345	1081
SNORA32	0.0210325904628205	0.489692658247915	0.573304968931496	1081
ZDHHC9	0.0210250202388039	0.489848979232604	0.573446499417666	1081
WFDC5	0.0210239791401292	0.489870479447933	0.573446499417666	1081
KRTAP19	0.0210048248520387	0.490266134073157	0.57377628948491	1081
SCCPDH	0.0209906570718626	0.490558896821136	0.574052220532197	1081
MRPS10	0.0209787389144486	0.490805246068417	0.574240427578596	1081
IL1F6	0.0209759612153742	0.490862670864141	0.574271755541468	1081
KIF2B	0.020974685963342	0.49088903601013	0.574271755541468	1081
LIX1	0.0209504634002324	0.491389968662682	0.574791018389834	1081
KRTAP10	0.0209276517565836	0.491861973424648	0.575201576045906	1081
SRRM4	0.0209252214768907	0.491912273622182	0.575201576045906	1081
NELF	0.0209073387994364	0.492282481285155	0.575567648606468	1081
AXIN1	0.020866205999398	0.49313457963777	0.576530445522772	1081
KRTAP9-2	0.0208632115936387	0.493196641906591	0.576569542201038	1081
C20orf114	0.0208327153689989	0.493828946727331	0.57724173911002	1081
LRIT2	0.0208052601765066	0.494398569557751	0.577773476258319	1081
MBD3L2	0.0208011849398501	0.49448314986583	0.577838798888981	1081
C1orf63	0.0207861098648251	0.494796095644378	0.5781374251352	1081
OPRK1	0.0207828577508076	0.494863620633708	0.578152639476271	1081
3-Mar	0.0207827183160947	0.494866515884306	0.578152639476271	1081
TLE6	0.0207700269220094	0.495130080413346	0.578427016349273	1081
KPNB1	0.0207669310442022	0.495194384440488	0.578468592319723	1081
TPPP3	0.0207648967031352	0.495236641867944	0.578484410902802	1081
SLC22A4	0.0207593302924819	0.49535227744075	0.578585935469584	1081
KRT20	0.0207497653464535	0.495551011464839	0.578750949386004	1081
C8orf74	0.0207327363478607	0.495904933747666	0.57913071828097	1081
AGPAT9	0.0207270291372311	0.496023579721241	0.579235697263022	1081
MAPKAPI	0.0207218224670633	0.496131833258766	0.579328528924058	1081
METTL12	0.0207027555728908	0.496528366543339	0.579690753057987	1081
HBA1	0.020695088346871	0.496687869035234	0.579843365885187	1081

RAB38	0.0206614987840806	0.497386959290306	0.580558566249679	1081
BTG4	0.0206525719715206	0.497572838541715	0.580741879051149	1081
FAM82B	0.0206363470464202	0.49791077785779	0.581001657781811	1081
FRAT2	0.0206341903468941	0.497955707652232	0.581020430388312	1081
HNF1B	0.0205862069309962	0.498955886934431	0.582018896484893	1081
DEFA5	0.020582792821661	0.499027092154251	0.582068251487893	1081
UBE2QL1	0.0205594113163353	0.499514885505856	0.582603483587046	1081
TESC	0.0205486483861071	0.499739510565511	0.582831728150421	1081
ZNF319	0.020545439088336	0.499806499778639	0.582876110804717	1081
RHOT2	0.0205421772340525	0.499874590905953	0.582921773302485	1081
ZNF446	0.0205301029221196	0.500126684644467	0.583181990169181	1081
LOC10012	0.0204935946059111	0.500889333719796	0.583936089570908	1081
IL5	0.0204707915189686	0.501365996267999	0.584424140424652	1081
RASSF1	0.0204679260658722	0.501425911006088	0.584426533446815	1081
OR6C65	0.0204554998034897	0.501685779781228	0.584633409211871	1081
SCG5	0.0204538803973592	0.501719651449731	0.584633409211871	1081
POU3F2	0.0204501729869553	0.50179720058623	0.58468995348543	1081
GPR6	0.0204358986871901	0.502095840009056	0.584936427075089	1081
CSPG5	0.0204062307301694	0.502716837749196	0.585592153143145	1081
KC6	0.0203667701944484	0.503543437655645	0.58642729587685	1081
TRIM60	0.0203515154624686	0.503863178245777	0.586690002	1081
TMEM130	0.0203465542026202	0.50396718976457	0.586777195573186	1081
HOXC5	0.0203133578123661	0.504663434453517	0.587485980182958	1081
OR5M3	0.0203031391879789	0.504877856715375	0.58769094899417	1081
OXSM	0.0203008217498517	0.504926491292121	0.58769094899417	1081
LOC64416	0.0203007962564803	0.504927026318744	0.58769094899417	1081
SLC16A10	0.0202988951307617	0.504966925878977	0.587703433002022	1081
C6orf127	0.020294811399731	0.505052638114905	0.587769231359027	1081
MRPL3	0.0202674853838574	0.505626372793998	0.588402939167875	1081
OR10AD1	0.0202234281535279	0.506552115678873	0.589378096936057	1081
BEX2	0.0202009349466048	0.507025092170302	0.589860272682533	1081
C19orf77	0.0201878335575902	0.50730068871735	0.590113834081296	1081
RBM23	0.0201870314658258	0.507317563810074	0.590113834081296	1081

ZNF560	0.0201453782427866	0.508194304411808	0.590981600321877	1081
SRRM5	0.0201145987489063	0.50884267817234	0.591633138161248	1081
SYCE1L	0.0200797744766052	0.50957677732552	0.592384106366357	1081
SPRR2F	0.0200765755334577	0.509644239122884	0.59242834374671	1081
RFC5	0.020074071245837	0.509697054742847	0.592455551773969	1081
PRR5-ARI	0.0200629018245145	0.509932653535118	0.592695205715725	1081
IL27RA	0.0200601801938468	0.509990070044821	0.592727742517332	1081
OCIAD1	0.0200465965512042	0.510276685997973	0.593026643557699	1081
HOXC13	0.0200031516334185	0.511193941751961	0.594058376498024	1081
LOC14983	0.0199987792560478	0.511286303685537	0.594131436764256	1081
CT45A4	0.0199940569542845	0.511386067183285	0.594178816669009	1081
GAGE2E	0.019983071553809	0.511618184369046	0.594386590474592	1081
C10orf47	0.0199649685279864	0.512000813961345	0.594653006471681	1081
SMOX	0.0199610376012106	0.512083918602951	0.594698513002861	1081
OR5D18	0.0199495821417976	0.512326141216477	0.594893660430337	1081
GGA2	0.0199262514991888	0.512819645820057	0.59536374856184	1081
MYO7B	0.0199022148748576	0.513328342384955	0.595816977261173	1081
SMNDC1	0.0198996833012085	0.513381934345565	0.595844850342307	1081
CPNE7	0.0198809793900202	0.513777975532647	0.596254700357652	1081
GSTO2	0.0198802105375583	0.51379425879913	0.596254700357652	1081
COL11A2	0.019877872586952	0.513843775112477	0.596277813919404	1081
TEKT4	0.0198725513951307	0.513956483828953	0.596345672730921	1081
PEMT	0.0198633204196866	0.514152036539232	0.596491991354883	1081
NCRNA0(	0.0198629803612732	0.514159241211873	0.596491991354883	1081
LOC25502	0.0198564101878557	0.514298450967471	0.596564881369246	1081
ARAP2	0.0198502388803198	0.514429227319804	0.59661351665846	1081
CCDC141	0.0198471553693009	0.514494576556318	0.596647246647374	1081
CAMLG	0.0198326157676328	0.514802774218141	0.596909283217707	1081
C22orf33	0.0198067617668449	0.515351041063752	0.597441832435361	1081
GRK1	0.019797845905038	0.515540183386879	0.59762671160828	1081
KRT76	0.0197786119689466	0.515948337263405	0.598065438412526	1081
AZU1	0.0197475223071987	0.516608429430094	0.598692792433368	1081
FAM18A	0.0197372034331627	0.516827615154431	0.598912350856306	1081

MIXL1	0.019734425685329	0.516886626192522	0.598946280393923	1081
ZNF576	0.0196996628578592	0.517625429400346	0.599733379839534	1081
ALG8	0.0196934158055835	0.517758253815343	0.599819340651283	1081
TMEM169	0.0196897201402289	0.517836839098531	0.599874820220811	1081
SIX3	0.0196841125738791	0.5179560911502	0.599978463355804	1081
EGFL8	0.0196805572311105	0.518031707396529	0.600031551604363	1081
YEATS4	0.0196778582831092	0.518089113352389	0.600063542142464	1081
RBM15	0.019675841856259	0.518132004396243	0.600078718440975	1081
YPEL5	0.0196725201243644	0.518202664340992	0.600126051920051	1081
BBC3	0.0196640471890281	0.518382923313079	0.600300298352609	1081
TG	0.0196235089755893	0.519245808417661	0.601161310952516	1081
BTF3L1	0.0196152720564006	0.51942122765261	0.601234181829836	1081
SMTNL2	0.0196149479310974	0.519428131078008	0.601234181829836	1081
SREBF1	0.0195987470068654	0.519773248836528	0.601529964830922	1081
PPP3CC	0.019584952306829	0.520067201446503	0.601801009428445	1081
RNF40	0.0195789746937239	0.520194605565807	0.601809903319055	1081
CLEC4G	0.0195773852713869	0.520228484492574	0.601809903319055	1081
OR4D2	0.0195761821128868	0.520254130866287	0.601809903319055	1081
GSX1	0.0195724064667489	0.520334616285277	0.601868445398881	1081
MMD2	0.0195709300926681	0.520366089882835	0.60187029228281	1081
SOX14	0.0195179036902469	0.521497165126679	0.603109270995864	1081
ADIPOR2	0.0194975815651691	0.521930979119465	0.60357632459022	1081
COPS4	0.0194676858042405	0.522569497035535	0.604280036609888	1081
LOC10013	0.0194122057991487	0.523755507896493	0.605512465226677	1081
NCRNA00	0.0194092988905919	0.523817687640426	0.605549599306576	1081
CTAG1B	0.0193893479033214	0.524244547894836	0.606008286988091	1081
GTF2F2	0.0193719286139062	0.524617386856304	0.606334897201391	1081
ACOX1	0.0193681005116761	0.524699340958859	0.606394826666067	1081
C18orf20	0.0193592637560772	0.524888548045621	0.606578694376953	1081
SAA2	0.0193423488350995	0.525250817025621	0.606892899855529	1081
KDELR3	0.0193409093174957	0.525281653252582	0.606893720163642	1081
NCAN	0.0192824055024755	0.526535658743706	0.608237906152415	1081
PPIC	0.0192602866286505	0.527010165252236	0.60871623066374	1081

RND2	0.0192579350243994	0.52706062600184	0.608717532525779	1081
NKX3-2	0.0192574177515328	0.52707172597901	0.608717532525779	1081
SPRR2D	0.0192310755722748	0.527637151036241	0.609198569032612	1081
CLEC18C	0.0192309686997925	0.527639445643138	0.609198569032612	1081
ABRA	0.0192111919851111	0.528064149278004	0.609653979437526	1081
B3GNT1	0.0192030356983837	0.528239355644532	0.609821307705392	1081
POLL	0.0191549318172258	0.529273283210122	0.610944894010309	1081
ACBD5	0.0191373268503172	0.529651934826818	0.611311918590939	1081
PDE4A	0.0191138434710177	0.530157234643356	0.611789969175139	1081
C6orf15	0.0190885012374277	0.530702806258103	0.612174074213781	1081
ZDHHC8I	0.0190850287689065	0.530777584270879	0.612225275415795	1081
LOC10012	0.0190736057010686	0.531023612453355	0.612438923276626	1081
NLRP9	0.0190621237087432	0.531270967949267	0.612651280289971	1081
DCBLD1	0.0190608237851556	0.531298975759037	0.612651280289971	1081
HIGD1C	0.0190578455642855	0.531363146536886	0.612690205883211	1081
AMFR	0.0190410480274759	0.531725151192605	0.612933352259511	1081
PCIF1	0.0190314663623001	0.531931702082321	0.613100133793819	1081
MFN2	0.0190228694647676	0.532117058987749	0.613243601447837	1081
EDDM3B	0.0190053872038722	0.532494093116984	0.613572813924478	1081
FGFR1OP	0.0189673270512809	0.533315390564169	0.614308339122602	1081
RPAIN	0.0189532843817886	0.533618577799953	0.614587287735409	1081
ARL4C	0.0189503293361865	0.533682389572733	0.614607283603093	1081
TCL6	0.0189438691134545	0.533821906163186	0.614716034049238	1081
SLC25A1	0.0189342853043516	0.534028914397009	0.614919261533452	1081
SEC14L4	0.0189313715975696	0.534091857866295	0.614956588746943	1081
TRHR	0.0189132462778189	0.534483494823878	0.615302017442541	1081
GPR180	0.0189081608204235	0.534593403185875	0.615358214226584	1081
CTDP1	0.0189039415283894	0.534684600376918	0.615428023916867	1081
CELF6	0.018894015940719	0.534899166254551	0.615639816271378	1081
FAM57A	0.0188914893207226	0.534953792255164	0.615667512853078	1081
PGAP3	0.0188825593534325	0.535146882340167	0.615854552684529	1081
DDAH2	0.0188710077704217	0.535396710899951	0.616106862851566	1081
SNORD17	0.0188240685948709	0.536412477953649	0.617205241511095	1081

C6orf52	0.0188181629111963	0.536540345909625	0.617281854274665	1081
AKAP4	0.0188165475963566	0.536575322851725	0.617286841348181	1081
NSMAF	0.018807534644198	0.536770504284899	0.617440861578216	1081
TRIM50	0.0188010308104324	0.536911371246169	0.617532376463264	1081
TM6SF2	0.0187747189284643	0.5374814513976	0.61811747664581	1081
HCN3	0.0187634441221217	0.537725827218079	0.618363214368547	1081
PRR25	0.018750549513998	0.538005379720228	0.618649373506707	1081
CKAP2	0.0187398024790052	0.538238428917131	0.61888202914016	1081
CYSLTR2	0.0186817544657389	0.539498072525454	0.620118034407467	1081
AMAC1L2	0.0186672913934421	0.539812150891704	0.620443645787264	1081
SFTPA2	0.0186647423954116	0.539867514103288	0.620448432790102	1081
NPM2	0.018660076221926	0.539968868866107	0.620482166636685	1081
TYRP1	0.0186553510629349	0.540071514552724	0.620564547838765	1081
NOS3	0.0186539399608616	0.540102170129415	0.620564547838765	1081
SAPS2	0.0186435077961493	0.540328831323986	0.620779330525187	1081
KRT73	0.0186353705420733	0.540505663629926	0.620921908863188	1081
DHCR24	0.0185683013933787	0.541964256522724	0.622455527232057	1081
ACAA1	0.018555992881031	0.542232149908257	0.622727704230194	1081
LY6G6F	0.018554421545011	0.542266354605913	0.622731485208991	1081
SGTB	0.0185344477811126	0.54270123526796	0.623171529990403	1081
LOC25431	0.0185339806318211	0.542711408396706	0.623171529990403	1081
OVOL1	0.0185072807312633	0.543293010857789	0.623732700959147	1081
PAH	0.0184900989689811	0.543667444035272	0.624091438833583	1081
PDILT	0.0184808893673516	0.543868196847826	0.624250745000254	1081
FKBP1AP	0.0183964680003482	0.54571014539115	0.626104749819097	1081
HSPB3	0.0183845297715756	0.54597086891505	0.626271674882911	1081
SFRS3	0.018360589603469	0.546493893132572	0.626800243518313	1081
PIP5KL1	0.018316436562809	0.547459160466955	0.627692930427251	1081
CACNG4	0.0182725816056841	0.548418744038786	0.628650028374949	1081
OR2B11	0.0182652938182146	0.548578287338439	0.628750197679662	1081
ZNF764	0.0182652721968186	0.548578760705286	0.628750197679662	1081
FLJ43663	0.0182390671188719	0.549152628144836	0.629348031287844	1081
NCAPD2	0.0182305985000524	0.549338146424386	0.629524828472567	1081

C1D	0.0182253596095869	0.549452927927129	0.629548926919122	1081
TRIM16L	0.0182110768559032	0.549765915977694	0.629835894982407	1081
KRT4	0.0182004134317065	0.549999648305064	0.630032009274736	1081
PRKY	0.0181858627040266	0.550318665551907	0.630361603317403	1081
FLJ45983	0.0181558693659031	0.550976541057363	0.630971657074674	1081
OR56A5	0.0181519707753707	0.551062081315174	0.631006413460686	1081
TAS2R60	0.0181516309677896	0.55106953745415	0.631006413460686	1081
PACS1	0.0181376933915803	0.551375401761747	0.631320760969103	1081
MAPK11	0.0181206146107969	0.551750314340486	0.631678227005518	1081
OR8K5	0.0181077337968819	0.552033156059396	0.631930215298132	1081
RNF139	0.0181058123148295	0.55207535477291	0.631942611619994	1081
ZG16B	0.0180957086050721	0.552297273866322	0.632124798706724	1081
SENP3	0.0180862581090143	0.552504885264949	0.63232649208399	1081
CT45A5	0.0180297588249592	0.553746876004165	0.633675915536463	1081
ZIC2	0.0180002377182067	0.554396363871743	0.634275031246948	1081
FBRSL1	0.0179881981398205	0.554661350983496	0.634398053458385	1081
TP53I3	0.0179386332945099	0.55575290770475	0.635538279161682	1081
LY6G5B	0.0179362916898005	0.555804502291935	0.635561202172392	1081
YME1L1	0.0179025687639124	0.556547807294531	0.636357812194955	1081
PMS2L1	0.0179018196997915	0.556564323309154	0.636357812194955	1081
OR8B2	0.0178941525539668	0.556733388901152	0.636420723498371	1081
ARSB	0.0178935936497241	0.556745714083485	0.636420723498371	1081
TCP10L2	0.0178877298596978	0.556875032756694	0.63653242935109	1081
VPS35	0.0178850371122372	0.556934422884955	0.636564195688931	1081
CAMK4	0.0178804946613606	0.557034616292997	0.636642593056985	1081
HABP2	0.0178615286526681	0.557453046632601	0.637048454979085	1081
SNORA71	0.0178615005928198	0.557453665805492	0.637048454979085	1081
GPR68	0.017859760501255	0.557492063584527	0.637048454979085	1081
STARD6	0.0178453458614719	0.557810194063723	0.63727592351918	1081
ETFA	0.0178364799480996	0.558005908188279	0.637463367384194	1081
SLCO3A1	0.0177899220240474	0.559034216654881	0.638457072085298	1081
OR8B4	0.0177618574856451	0.559654511782726	0.639093029865824	1081
LOC1001[illegible]	0.01775534431359	0.559798516510008	0.639221239944117	1081

TMEM79	0.0177518598634928	0.559875564262846	0.639272983170381	1081
GATS	0.0177234153987852	0.560504716917283	0.639882552550078	1081
TBC1D3P:	0.0177047514900371	0.560917722788651	0.640281478103886	1081
ESR2	0.0176989018500572	0.561047197310397	0.640392984861349	1081
FAM165B	0.0176630941351795	0.561840071311453	0.641152678670436	1081
GP9	0.0176543869709883	0.562032951815054	0.641275479881121	1081
PDXK	0.0176424744921696	0.562296887975539	0.641528618013662	1081
LOC7291:	0.0176056513008936	0.56311313053678	0.642350736957159	1081
LOC6535	0.0176037924671729	0.563154349567025	0.642361382256255	1081
DEFA1B	0.0175960436648824	0.563326192378775	0.642521013784986	1081
BCKDK	0.0175894247782278	0.563472997389372	0.642652071132248	1081
SLC22A1	0.0175755687168431	0.563780380481074	0.642929847992711	1081
LRRN2	0.0175647721066018	0.564019948569379	0.643095831912577	1081
PLA2G10	0.0175493863170896	0.564361431427678	0.643373948056736	1081
OR6C2	0.0175447243192216	0.564464922649267	0.643455515321949	1081
PGPEP1L	0.0175276698939993	0.564843589810285	0.643777884963406	1081
CST4	0.0175200586242751	0.565012625661605	0.643934110882158	1081
BCAS3	0.017513228241529	0.565164339761539	0.644070578972461	1081
FAM66D	0.0174829315684753	0.565837514628459	0.64471164359262	1081
NNMT	0.0174757613439329	0.565996889192051	0.644836971653313	1081
LOC2847	0.0174506920492032	0.566554280421515	0.645289533504349	1081
LOC1001	0.0174420965415187	0.566745453626138	0.645390876727878	1081
ABCC13	0.0174409297221545	0.566771407306671	0.645390876727878	1081
C2CD4C	0.017405917949263	0.567550442278515	0.645994803203711	1081
RPL23A	0.01740523914241	0.567565551235556	0.645994803203711	1081
CT45A2	0.0174041240883449	0.567590370648085	0.645994803203711	1081
CDKL4	0.0174017992653863	0.567642119350359	0.646017202096661	1081
OR2B3	0.0173792422298108	0.568144338026175	0.646515714259577	1081
KRTAP10	0.0173464886941621	0.56887395319734	0.647236292056953	1081
CPZ	0.017342698551585	0.568958410962607	0.647256150217442	1081
TBC1D3G	0.0173413798953532	0.568987796684074	0.647256150217442	1081
ORAI3	0.01732854187954	0.569273924507877	0.647508509356399	1081
GAS6	0.017298693736046	0.569939431309616	0.648165499340278	1081

SLC2A3	0.01729121093206	0.570106329133774	0.64823570584184	1081
ASAP1IT1	0.0172800057875464	0.570356294222235	0.648410132455244	1081
USP35	0.0172461661177158	0.5711115082178	0.649122169546806	1081
MAGEA5	0.0172111696245595	0.571893039121005	0.64990044977112	1081
OR52E8	0.017199417050844	0.572155607798461	0.650125484748153	1081
UBOX5	0.017195319923702	0.57224715670367	0.65015616557416	1081
ARMC5	0.0171890311176579	0.572387691442727	0.650279158815347	1081
C19orf18	0.0171813049609305	0.572560368786478	0.650438652749315	1081
HR	0.0170908568993353	0.57458369133399	0.652266607832649	1081
KRTAP4-[illegible]	0.0170843136873244	0.574730193896692	0.652388558970466	1081
RPL23AP[illegible]	0.0170634439430828	0.575197585479971	0.652815456916779	1081
SIRT3	0.0170631802283575	0.575203492691627	0.652815456916779	1081
ACADS	0.0170588670933829	0.575300111008685	0.652888329461437	1081
RPGRIP1	0.0170203148133715	0.576164058690195	0.653647855060763	1081
DNAJC25[illegible]	0.0170059280393955	0.576486619554008	0.653940140262149	1081
OR52J3	0.0169804865452043	0.57705724256807	0.654513718345463	1081
FAM23A	0.0169785276467837	0.57710118939012	0.654526712096834	1081
HTR2C	0.0169735682596104	0.577212457587071	0.6545792027748	1081
SNTG1	0.0169664063420493	0.577373159291162	0.654687734657236	1081
PHB2	0.016955049771372	0.577628025270298	0.654866147170362	1081
ZNF837	0.0169513177228264	0.577711792045983	0.654909513188483	1081
SNORA56	0.0169504488004999	0.577731296047283	0.654909513188483	1081
STON2	0.0169112200619383	0.578612154228986	0.655649783268248	1081
ANKRD2[illegible]	0.0168729417311792	0.579472278638138	0.656439805047814	1081
PLBD2	0.0168613077569831	0.579733815847504	0.656662227733931	1081
RG9MTD[illegible]	0.0168590254018824	0.579785130749737	0.656683428480459	1081
DMRTB1	0.0168483007618363	0.580026284658614	0.656919632565794	1081
OR8K3	0.0168454375030309	0.58009067574448	0.656955625302032	1081
LOC73081	0.0168437632718054	0.580128328647253	0.656961334566245	1081
FKBP5	0.0168152361118337	0.580770070712784	0.657540216630643	1081
ZNF222	0.0167989492358842	0.581136606244382	0.657888974656446	1081
GADL1	0.0167977500486263	0.581163598180305	0.657888974656446	1081
HOOK2	0.0167896706773227	0.5813454681743	0.658043740876062	1081

EN1	0.0167813772221301	0.58153218493074	0.6582181068339	1081
CCDC38	0.016758260625628	0.58205277389559	0.658733321917228	1081
MAN1C1	0.0167566904188551	0.582088143054322	0.658736343004849	1081
LIPM	0.0167502458936602	0.582233317515982	0.658863621017833	1081
NELL2	0.0167369592673716	0.582532675829671	0.659165351603029	1081
ASB5	0.0167043268513906	0.583268214578158	0.659960580918725	1081
PMAIP1	0.0166926591111411	0.583531311797739	0.660179648702524	1081
MR1	0.0166908439374985	0.583572247320681	0.660179648702524	1081
CARS2	0.0166687909318648	0.584069690080085	0.660622068815508	1081
NKX2-5	0.0166682281973948	0.584082386084538	0.660622068815508	1081
CHRNA9	0.0166585590925707	0.584300553409131	0.660831722907419	1081
ZNF75A	0.0166492080940311	0.584511579205518	0.66099616947879	1081
TARS2	0.0166476713386657	0.584546262848604	0.660998285999727	1081
C7orf65	0.0166425637460588	0.584661545005738	0.661021286825056	1081
LOC15202	0.0166409550772883	0.58469785604049	0.661021286825056	1081
AMBP	0.01663276251536	0.584882795624903	0.661193261384044	1081
PDZK1P1	0.0166286690065264	0.584975213011732	0.661224934433401	1081
ZC3HC1	0.0166257062421113	0.585042106296398	0.661224934433401	1081
KCNJ3	0.0166222077422812	0.585121099984047	0.661277114119418	1081
SMYD2	0.0166171228155729	0.585235922908626	0.661337779877325	1081
RAB10	0.0166119154579106	0.585353521284374	0.661354272926176	1081
OR51B6	0.0166085263947326	0.585430062787708	0.661403657386483	1081
C1orf194	0.016579372291778	0.58608869563251	0.662073502692588	1081
15-Sep	0.0165601405073988	0.586523356936993	0.662415934601992	1081
OR3A4	0.0165577379714187	0.586577667603761	0.662440134201018	1081
CRABP1	0.0165501837475983	0.58674845021324	0.662558718023863	1081
ZMYND8	0.0165275750695274	0.587259714801285	0.662987388179325	1081
KIAA1045	0.0165164768279003	0.58751076182028	0.663233639499554	1081
OR6C6	0.0165133989605881	0.587580393290808	0.663275076927229	1081
SLC5A12	0.0164720503910943	0.588516203242582	0.664182572230914	1081
RTL1	0.0164648875304271	0.588678384522546	0.664291175005976	1081
FOLH1	0.0164572253558669	0.588851894064178	0.6644125353379	1081
TNNT2	0.0164540743383511	0.588923255531394	0.664455837730604	1081

LILRA1	0.0164391607024779	0.589261060479083	0.664762505647708	1081
TRIM59	0.0164333532933041	0.589392626759371	0.664837553598792	1081
FLJ25328	0.0164166493138311	0.589771129161143	0.665226170042062	1081
NCRNA00	0.016398268872028	0.590187748447422	0.665658820473283	1081
NR1D1	0.0163960482340968	0.590238091579236	0.665678331366236	1081
IL1F8	0.0163893055770383	0.590390963570603	0.665752287549039	1081
OR5M11	0.0163772673998186	0.590663942786347	0.665897651095806	1081
XRRA1	0.0163482517013517	0.5913221446555	0.666565083324103	1081
MATN4	0.0163233469554474	0.591887360624709	0.667052916351595	1081
C17orf64	0.016320431629219	0.591953540469478	0.667090180843456	1081
C19orf21	0.0163105659228518	0.592177523728321	0.667305264614142	1081
SNORA68	0.016305672789638	0.592288627993468	0.667393131834009	1081
MRPS14	0.0162892903144932	0.592660680594544	0.667737662783954	1081
FDFT1	0.0162844996465668	0.592769498808737	0.667822915580896	1081
SNORA16	0.0162575813142421	0.593381108585602	0.668437199153216	1081
CNGA2	0.0162408335751037	0.593761778501556	0.668828619319719	1081
C2orf55	0.0162347479525007	0.593900130090428	0.668909656752302	1081
TFF2	0.0162066157241071	0.594539884321245	0.669480490198517	1081
MAT2B	0.0161622500429411	0.595549441576928	0.67042992459163	1081
WDR24	0.0161541082089907	0.595734796745421	0.670601110887154	1081
OR4F5	0.0161154831003756	0.596614485009122	0.67144201740402	1081
LDLR	0.0161154540976204	0.596615145769767	0.67144201740402	1081
PLCH1	0.0160424337344099	0.598279799472543	0.673165022362287	1081
OR4A15	0.0160273001536159	0.598625064670017	0.673440661628273	1081
MYCBPAP	0.0160220232172074	0.598745476596056	0.673500900442657	1081
PNLIPRP1	0.0159973448805201	0.599308745476009	0.674096855254841	1081
FGFBP2	0.015988244771644	0.599516510723385	0.674292898694801	1081
CD9	0.0159798003896216	0.599709334155327	0.674396814589654	1081
NPY6R	0.0159587326585143	0.600190527924387	0.674711952289998	1081
C21orf129	0.0159369916685209	0.600687281904071	0.67519502811949	1081
NLRP1	0.0158991891478583	0.601551462901997	0.67606124466593	1081
HRG	0.0158988774509097	0.60155859074891	0.67606124466593	1081
ADAD2	0.0158922486496956	0.601710186356618	0.676193891785901	1081

IFT52	0.0158707743481805	0.602201405310415	0.676708167009444	1081
GAGE2B	0.0158597090456705	0.602454591604263	0.676837534542131	1081
CLDN6	0.0158584011571869	0.602484520695433	0.676837534542131	1081
C21orf96	0.0158565364246016	0.602527193495688	0.676847728376214	1081
IMPA1	0.0158295503774888	0.603144898072925	0.677466070347314	1081
LZTR1	0.0157931932462026	0.603977554170793	0.678325685943616	1081
OR10G3	0.0157729192622671	0.60444209559051	0.678593745390371	1081
IL31	0.0157714974304584	0.604474680284839	0.678593745390371	1081
SULT6B1	0.0157489985467928	0.604990401041321	0.67904678119999	1081
C4orf10	0.015739712927569	0.605203303971724	0.67921180157311	1081
LCE3A	0.0157396452116982	0.605204856700786	0.67921180157311	1081
DOCK3	0.0157168562574098	0.60572751003903	0.67976049745063	1081
TMC4	0.0156968075360594	0.606187484284278	0.68020090630573	1081
GBX1	0.015674647174555	0.606696086978732	0.680695776785661	1081
RNF157	0.0156643046372351	0.606933523826893	0.68092425025021	1081
HOXB5	0.0155449517249587	0.609676539260606	0.683620944194863	1081
ADAD1	0.0155347903390865	0.609910326155739	0.683768908698723	1081
CCDC112	0.0155200994724224	0.610248394837729	0.684071777530802	1081
SLC39A5	0.0154982013853127	0.610752471417911	0.684560648568065	1081
OR52I2	0.0154894083125719	0.610954932849817	0.684719006026006	1081
CCR9	0.0154887906645247	0.610969155378383	0.684719006026006	1081
FGF17	0.0154861608667678	0.611029713146166	0.684719006026006	1081
RPL18A	0.0154785902688576	0.611204060209376	0.684838188604365	1081
IHH	0.0154365259628454	0.612173180702813	0.685809627697596	1081
DOC2A	0.0154279702799722	0.612370378308938	0.685954252170461	1081
B4GALNT	0.0154190743437497	0.612575448059148	0.686107661578535	1081
TMCO2	0.0154098058385758	0.612789138529462	0.68630884503675	1081
BEND2	0.0153924616962215	0.613189105568357	0.686642275245945	1081
PRB2	0.0153749555778625	0.613592924538619	0.68697990689882	1081
KRTAP20	0.0153302893011263	0.614623785496838	0.687866453268545	1081
C2orf42	0.0153252490495851	0.614740158153034	0.687958473751062	1081
OR1I1	0.0152956783585451	0.615423100787042	0.688619957901595	1081
SNORA3	0.0152956242234609	0.615424351355958	0.688619957901595	1081

SEPT12	0.0152952155931754	0.615433791117415	0.688619957901595	1081
TMEM72	0.0152861761074078	0.615642628380306	0.688815370659331	1081
OR10G2	0.0152741819458295	0.615919774861794	0.689087185637612	1081
CRNKL1	0.0152648403972241	0.616135665886718	0.689252165109281	1081
KRTAP4-1	0.015261418645308	0.616214753744085	0.68930236071823	1081
GNG10	0.0151558495524713	0.618656982617438	0.691803769904429	1081
C22orf46	0.0151484537668121	0.618828234435538	0.691956859608343	1081
OR11H1	0.0151113601901941	0.619687459898447	0.692763811301566	1081
H1FOO	0.0150908252561247	0.620163348737759	0.693180417262506	1081
CLTCL1	0.0150705283973632	0.620633876416518	0.693555938142175	1081
LOC34234	0.0150683059731787	0.620685406722094	0.693571528645251	1081
SELE	0.0150642256027371	0.620780021194391	0.693624452396811	1081
GDEP	0.0150433609898078	0.621263921818469	0.694063980980095	1081
USP14	0.0150250640697642	0.621688406140936	0.69444957468619	1081
FGF6	0.0150237386113274	0.621719161356061	0.69444957468619	1081
SLPI	0.0150146569192296	0.621929905733925	0.694615398958248	1081
PRAMEF8	0.0149993519500227	0.622285133843423	0.694935086272839	1081
UNCX	0.0149964736799503	0.622351948266329	0.694952209137263	1081
RBL1	0.0149957192502644	0.622369461655267	0.694952209137263	1081
KCNK18	0.0149818885065464	0.622690567422279	0.695177695829533	1081
ABCD2	0.0149668425973635	0.623039966862722	0.695431381264907	1081
GFOD1	0.014924766933303	0.624017507048594	0.696393419582035	1081
RPS28	0.0149019250081381	0.62454846860407	0.696777962449903	1081
IZUMO1	0.0149015086667612	0.624558148285401	0.696777962449903	1081
YPEL3	0.0148950168253298	0.624709087979711	0.696869404582273	1081
PPIL2	0.0148718692803303	0.625247411969687	0.69739267560652	1081
RIT2	0.014860760885827	0.625505821955652	0.697642275678032	1081
TKTL1	0.0148473612013097	0.625817594424697	0.697912723631838	1081
PTPRVP	0.0148034599515685	0.626839519262996	0.698936299084135	1081
CASR	0.0147549410985523	0.627969764274196	0.700006648709123	1081
FABP1	0.0147547935793938	0.627973202058638	0.700006648709123	1081
LOC10027	0.0147468278272881	0.628158847803969	0.700174845848002	1081
LRRC50	0.0147374570738161	0.628377267866612	0.700379554120444	1081

PRAMEF9	0.0147313361513062	0.628519956147412	0.700499834942718	1081
ACADVL	0.0147207589802509	0.628766559221903	0.700735911458577	1081
RPL13AP1	0.0147161217968533	0.628874686588933	0.700817644594461	1081
NCRNA00	0.0147136980391835	0.628931205639715	0.700841859586904	1081
COL6A4P	0.0147096361022277	0.629025929887078	0.700907010937353	1081
PRDM13	0.0147082069868353	0.629059258264946	0.700907010937353	1081
C3orf54	0.014706703927199	0.629094311909626	0.700907301223352	1081
TRAF7	0.0146743824313404	0.629848300380691	0.701630945098929	1081
OR11H12	0.014670818913777	0.629931452899993	0.70168477294279	1081
PL-5283	0.0146629874367317	0.630114212117025	0.701849541252763	1081
CRLF3	0.0146177508357016	0.631170317969404	0.702909284951829	1081
PHOX2B	0.0145811600938938	0.632025125906189	0.703656451212643	1081
TFIP11	0.0145707257277344	0.632268976539356	0.703845403341797	1081
MUC2	0.0145698087678284	0.632290407757583	0.703845403341797	1081
FBXO40	0.0145638070243239	0.632430688327094	0.703962659760708	1081
ACRC	0.0145608357269988	0.632500142248597	0.704001069981656	1081
LOC38842	0.0145518044210461	0.632711268429075	0.704163581578431	1081
CHADL	0.0145515995495849	0.632716058086432	0.704163581578431	1081
CGB5	0.0145017704141879	0.633881461964873	0.705343683787139	1081
DUSP8	0.0144768428189244	0.634464809301491	0.705914811851239	1081
POTEG	0.0144676660584018	0.634679617950055	0.706078312412608	1081
SEC11A	0.0144675702592236	0.634681860569249	0.706078312412608	1081
SLFN11	0.0144591403106818	0.634879215291359	0.706219867037241	1081
SLC31A2	0.0144306699903746	0.635545929487711	0.706883433609206	1081
PPP2R2B	0.0144262393597327	0.635649711960387	0.706900138850425	1081
LUZP4	0.0144240366210014	0.635701311264051	0.706900138850425	1081
LOC34007	0.0143922013303846	0.636447252201598	0.707612445256427	1081
FBXO41	0.0143674124347207	0.63702834228131	0.708102188117696	1081
OR12D3	0.0143367126319416	0.637748302213831	0.708785145542184	1081
CBX3	0.014323056946329	0.63806865983286	0.709062949709452	1081
GOLGA8I	0.0143194330215404	0.638153687180019	0.709118320273676	1081
ANKZF1	0.0143010246628499	0.638585671810795	0.7094026887961	1081
GCET2	0.0142939943259845	0.638750683413476	0.709546871649544	1081

TSNARE1	0.0142408254294385	0.639999207918907	0.710620296727465	1081
GYPA	0.0142313229659528	0.640222454389783	0.710828998218816	1081
SLC39A2	0.014198253348325	0.640999630086354	0.711535020282654	1081
C1QL4	0.0141892093988473	0.641212242249826	0.711692596150014	1081
ZNF479	0.0141860243537012	0.641287125755176	0.711736496520193	1081
FRMD5	0.0141696917544219	0.641671178282027	0.712045052687641	1081
GPR173	0.0141649619584904	0.641782414842783	0.712129261746508	1081
CANX	0.0141545793843386	0.642026623051325	0.712361000295712	1081
KCNQ5	0.0141445448106831	0.642262682692289	0.712544429058055	1081
OBFC2A	0.0141199621631596	0.642841132883592	0.713068367487471	1081
LOC84931	0.0140757848920381	0.643881204405936	0.714082276852739	1081
CSTF2	0.0140751152653963	0.643896974883594	0.714082276852739	1081
ATXN3L	0.0140731953819285	0.643942191235227	0.714093110339846	1081
NPVF	0.0140666050922867	0.644097413195103	0.714186613210885	1081
CT45A6	0.0140590400695573	0.644275612281433	0.71430557013811	1081
RMND1	0.0140451032050892	0.644603957739753	0.714590949189265	1081
MRPL50	0.014043317750327	0.644646027153025	0.714598262091988	1081
KIFC3	0.0140353850979233	0.644832952393309	0.714658972002607	1081
OR5K3	0.0140349715802441	0.64484269715023	0.714658972002607	1081
AKR1C3	0.0140062828580935	0.645518910303398	0.715143967363335	1081
PICK1	0.0140049414078279	0.645550536388317	0.715143967363335	1081
HOXD10	0.0140043571501862	0.645564311069855	0.715143967363335	1081
FSIP1	0.014000100771577	0.645664664743008	0.715200019738751	1081
CA1	0.0139891792462796	0.645922193667786	0.715422398913435	1081
RHD	0.013985709740976	0.646004013271804	0.715434351146811	1081
INHBE	0.0139840161051129	0.646043954990677	0.715439254207421	1081
CES8	0.0139780430384937	0.646184828438367	0.71551659258456	1081
RIPPLY2	0.0139591340965288	0.646630875153625	0.715892447789062	1081
GCKR	0.0139533532843802	0.646767265180538	0.715986063036424	1081
OR2AT4	0.0139525373683174	0.646786516512129	0.715986063036424	1081
SLC22A25	0.01394913872319	0.64686670921756	0.716035488608591	1081
SEPHS1	0.0139309375093712	0.647296245497415	0.716392861528702	1081
FAM156A	0.0139077892999018	0.647842697007913	0.716918871194485	1081

C20orf72	0.0138968557082823	0.648100868319848	0.71706501634332	1081
FKRP	0.0138961665924302	0.648117141602341	0.71706501634332	1081
CHKB-CP	0.0138942591880564	0.648162185310628	0.717075469442726	1081
LOC2855(	0.0138888006001301	0.648291097982924	0.717178702299066	1081
ZNF530	0.0138722969091511	0.648680921213666	0.717531141986015	1081
FAM5B	0.0138614347542569	0.648937541766257	0.717772465192452	1081
FOLR4	0.0138597039219571	0.648978436866307	0.717772465192452	1081
NOXA1	0.01385853824821	0.649005979323927	0.717772465192452	1081
HAO2	0.0138390445324195	0.649466646477665	0.71820308466227	1081
BMPR1B	0.0138353660940878	0.649553588779742	0.718222027787746	1081
ZPLD1	0.0138197404447852	0.649922964363631	0.718510488261735	1081
LOC64532	0.0138089899828149	0.650177145028958	0.718752048872763	1081
PSG6	0.0137829077661139	0.650793993957798	0.719394480261185	1081
DKFZp43	0.0137781758552078	0.650905930082709	0.719439260480423	1081
CLN3	0.0137432282098953	0.651732880407624	0.720247479664519	1081
CD82	0.0137427407361174	0.651744418307496	0.720247479664519	1081
SYNPR	0.0137280725069337	0.652091636040308	0.72059166253604	1081
OR5AP2	0.0136892696867905	0.653010516696531	0.721448769269036	1081
UMPS	0.0136765132832781	0.653312713080133	0.721743054839555	1081
GALK2	0.0136626766522714	0.653640564029174	0.722027564647987	1081
C3orf77	0.0136550771696468	0.65382065777398	0.722145794772566	1081
AHRR	0.0136382619769523	0.654219218422177	0.722546390083046	1081
SLC29A4	0.0136263280607353	0.654502140785128	0.722790677285618	1081
OR5AU1	0.0136231854830074	0.654576651440648	0.722803808361637	1081
GUCA1C	0.0136213712182898	0.654619669301144	0.722803808361637	1081
HIST1H4I	0.0136049683697543	0.655008647669327	0.723180379661219	1081
OR5M1	0.0135844230655722	0.655495992452653	0.723639125819744	1081
TMEM69	0.0135797620712365	0.655606573944577	0.723721542666091	1081
OR11H6	0.0135602193159937	0.656070305359908	0.724193769943855	1081
GABRA3	0.0135366170917649	0.656630540692017	0.724633624672152	1081
KRTAP1-:	0.0135358547912182	0.65664863829189	0.724633624672152	1081
C7orf10	0.0135312325149038	0.656758378985646	0.724715027643834	1081
C1orf125	0.0135151106823753	0.65714119653846	0.725058024113664	1081

ATP2C2	0.0134817849052096	0.657932811475032	0.725851943437268	1081
LOC40004	0.0134791107725037	0.657996348994848	0.72588228741587	1081
CACNA2I	0.0134764602927406	0.658059326954037	0.725912010814208	1081
COIL	0.0134710008871471	0.658189055339709	0.72601536000758	1081
ANKRD33	0.0134640581126487	0.65835404692331	0.726138677676453	1081
N4BP3	0.0134375357503457	0.658984490581563	0.726693812281891	1081
GK2	0.0134256246111773	0.659267700344689	0.726846969321572	1081
OR4S2	0.0133847537144507	0.660239853590891	0.72775945779101	1081
BIN3	0.0133825358877878	0.660292623116659	0.727777802302626	1081
ZNF552	0.0133795453854792	0.660363779855574	0.727816410175651	1081
LOC14882	0.0133527100112742	0.661002444449117	0.728321076597247	1081
LRRC56	0.0133313778757898	0.661510311432717	0.728840802002411	1081
BTN2A3	0.0133220950092073	0.661731362823008	0.729004610306764	1081
C4orf51	0.013307217767606	0.662085693565801	0.729315195586345	1081
SERPINB4	0.0133013299642894	0.662225944047461	0.729429801675111	1081
FGF22	0.0132858909643942	0.662593765089802	0.729795046349593	1081
RNF13	0.0132827933929672	0.662667571886862	0.729836435168162	1081
C8B	0.0132742847693215	0.662870326338869	0.729979922598617	1081
CCDC103	0.0132446894604401	0.663575753559009	0.730636945531604	1081
SSX5	0.0132084234401159	0.664440588536769	0.731509217186207	1081
CWC25	0.0131966819758005	0.664720682673396	0.731777591805867	1081
MYO18A	0.0131909792239596	0.664856739420451	0.731865228457677	1081
CT47A1	0.0131902998623266	0.664872948424123	0.731865228457677	1081
C9orf43	0.0131584435877995	0.665633188215242	0.732622006042509	1081
TBC1D21	0.0131438769180654	0.665980931561253	0.732964699863497	1081
MAPK9	0.0131353353821196	0.666184873049099	0.73310276056189	1081
LYSMD4	0.0131345159182679	0.666204440226293	0.73310276056189	1081
CRYGN	0.0131231574241577	0.66647568197692	0.73330889799689	1081
EXOSC10	0.0130964096456188	0.667114593069145	0.733891627204998	1081
LY6D	0.013093236814857	0.667190396930152	0.733934939450549	1081
ONECUT1	0.0130837550386113	0.667416951523448	0.734063905243495	1081
LOC34911	0.013059295624614	0.668001516851339	0.734586525299974	1081
MYADMI	0.0130498172393025	0.66822809867645	0.734755474473941	1081

NCRNA0(	0.0130447954210705	0.668348158020499	0.734807271916332	1081
ADAM5P	0.0130047650444373	0.66930548875659	0.735699190866017	1081
KRTAP4-:	0.0129911356100987	0.669631560744879	0.735889371486446	1081
SLC22A1:	0.0129737000264827	0.6700487821083	0.736194857680486	1081
CGB8	0.0129694092431717	0.670151473155985	0.736260385826585	1081
C14orf64	0.0129681538847534	0.670181518734281	0.736260385826585	1081
PPP1R3D	0.0129621534401066	0.670325139840905	0.736337856049677	1081
KIAA125	0.0129502902813787	0.670609120870679	0.736599079009841	1081
DNAH3	0.0129389122053254	0.670881534117858	0.736788339137777	1081
OR2S2	0.0129238181236202	0.67124298242734	0.737145109081921	1081
STYK1	0.0129205830777886	0.671320459885408	0.737155887538421	1081
MARK1	0.0129173338214093	0.671398281189293	0.737171405348642	1081
CORO1C	0.0128993898132354	0.671828112444818	0.737546448239829	1081
C7orf16	0.0128867785774542	0.672130266470148	0.737837949868733	1081
ACTG1	0.0128784859179667	0.672328980069119	0.738015873119682	1081
MMP15	0.0128697743412201	0.67253775663305	0.738164602123395	1081
UBE2D3	0.0128676622424427	0.672588377757957	0.738179943880907	1081
C11orf65	0.0128650663975605	0.672650594959586	0.738208010212512	1081
SNRPN	0.0128609830571171	0.672748468979292	0.738275203333215	1081
FAM76A	0.0128439834908634	0.673155992873945	0.738601714403356	1081
UGT1A1	0.0128032625483638	0.674132569591119	0.73955239418467	1081
FAM163B	0.0127851367240664	0.674567442788399	0.739948877482836	1081
MT4	0.0127674756907856	0.674991269190082	0.740373468247534	1081
BAALC	0.0127628657569299	0.675101914529025	0.740454514519911	1081
DNAJB9	0.0127612394685212	0.675140949567742	0.74045701377625	1081
OR9Q1	0.0127344677466917	0.675783664018098	0.741081214569656	1081
EDN1	0.0127255798626905	0.675997089565449	0.741194218107874	1081
NUDCD1	0.0127194054357048	0.676145371936777	0.741316453557547	1081
TNFRSF1.	0.0127166676036027	0.676211126544658	0.741348198326102	1081
PGAP2	0.0127073721402296	0.676434394405755	0.741552616200693	1081
LCE2A	0.0126787060801477	0.67712310371556	0.742186458694704	1081
POTEE	0.0126658363295372	0.677432390459392	0.742417712869239	1081
CHODL	0.0126646470810412	0.677460973304079	0.742417712869239	1081

PGA3	0.0126460589020959	0.677907788818951	0.742844452010322	1081
WBSCR17	0.0126337581519643	0.678203531754899	0.743087683411424	1081
FAM73B	0.0126137064554517	0.678685734293104	0.743454275004599	1081
FAAH2	0.012607538431503	0.678834089162829	0.743535924651218	1081
UBAC2	0.0126031954952479	0.678938554018368	0.743609915223184	1081
ZCCHC9	0.0125342879174022	0.680596877777389	0.745304637031175	1081
C10orf40	0.0125324071656095	0.680642161457754	0.745313711070634	1081
GNB5	0.0125126947388212	0.681116855295938	0.745792969341051	1081
GPATCH4	0.0125101347495997	0.681178511509822	0.745819942081927	1081
CR1	0.0125050129335044	0.68130187458111	0.745914470838938	1081
DNM1L	0.0125003578374583	0.681414003706882	0.745996690651331	1081
PPP1CC	0.0124969903832931	0.681495121278546	0.74604495262234	1081
LOC14448	0.0124875778695117	0.681721875940334	0.746252632223986	1081
EPHX4	0.0124794221213472	0.681918377373858	0.746427174103259	1081
FBLN7	0.012441803860026	0.68282501585444	0.747297764132542	1081
LOC72397	0.0124344135385122	0.683003183912548	0.747411545435292	1081
SLC10A5	0.012417183682399	0.683418634859445	0.747825551289708	1081
UFC1	0.0124054968058422	0.683700486493968	0.748052699349391	1081
RNF26	0.0124000156049662	0.683832691421918	0.748122296658119	1081
LOC72923	0.0123782993214322	0.684356576802623	0.748607908323677	1081
GPRC6A	0.0123547035998226	0.684925973870438	0.749190082186023	1081
RNF4	0.0123417271265741	0.68523919016983	0.749491990768337	1081
DEFB107A	0.012333619369065	0.685434916522187	0.749624668476626	1081
TJAP1	0.0123174687383134	0.685824865698385	0.750010418516583	1081
AKR1B10	0.0123152919723725	0.68587742905306	0.750027185336765	1081
GLRA2	0.0123113739158824	0.685972043965191	0.750089932458274	1081
TPD52L1	0.012297167894679	0.68631513828893	0.750383634237747	1081
SSX6	0.012200427123436	0.688653270449785	0.752501392718664	1081
1-Dec	0.0121905873129841	0.688891256272432	0.752688547770799	1081
PRAMEF1	0.0121584788955051	0.689668044787847	0.753395301895297	1081
SF1	0.0121424234987005	0.690056590364465	0.75373447326306	1081
TRIM36	0.0121410111668883	0.690090773137943	0.75373447326306	1081
GPR89A	0.0121051793715666	0.690958223822973	0.754559192221705	1081

C18orf18	0.012047571406755	0.692353702398895	0.755796322468068	1081
C17orf71	0.0120402044224884	0.692532233434228	0.755950248850313	1081
CARD18	0.012035746438624	0.692640276221052	0.756027219574831	1081
TAAR8	0.0120104118215268	0.693254399351726	0.756525645498635	1081
ITGB1BP3	0.0120091627303365	0.69328468312486	0.756525645498635	1081
GCLM	0.0120028945800502	0.693436659594612	0.756650498728928	1081
OTOP1	0.0119502423166978	0.694713742848198	0.757961890688743	1081
CASP9	0.0119343322723921	0.695099812253524	0.758342037251408	1081
TMIGD1	0.0119268399333715	0.695281646951479	0.758499338126153	1081
CALML5	0.0119073507963416	0.695754718897098	0.758892133422602	1081
SPZ1	0.0118996009806195	0.695942867868715	0.759056257347876	1081
C19orf61	0.011894972668522	0.696055242315069	0.759137721641333	1081
OR5M9	0.0118843066412806	0.696314236589051	0.759379076228152	1081
SNORA4	0.0118807625113884	0.696400303578621	0.759390945236013	1081
RAP1GDS	0.0118772083058679	0.69648661916892	0.759443731700021	1081
OR5D13	0.0118263924306478	0.697721135870455	0.760625157319677	1081
SCARNA3	0.011818597382858	0.697910578913163	0.760790509318488	1081
DNAH17	0.0117992092071862	0.698381851247257	0.761263048177912	1081
DDX3Y	0.0117839849207646	0.698751992491699	0.761558690031073	1081
C2orf61	0.0117833889171608	0.698766484315431	0.761558690031073	1081
TRUB2	0.0117770517926822	0.698920578234551	0.761676140073594	1081
KRTAP5-1	0.0117566617121956	0.699416468746624	0.762022901674738	1081
UGT1A5	0.0117407072458037	0.699804573621381	0.762360108998633	1081
PRRT1	0.0117173514089887	0.700372863834071	0.762877095273292	1081
PNLDC1	0.011696689926317	0.700875735161861	0.763238103942683	1081
NCRNA00	0.0116784728062687	0.701319222764821	0.763647003172545	1081
SC4MOL	0.0116562811005232	0.701859607182524	0.76418559002767	1081
ZNF735	0.0116315051103078	0.702463098736635	0.764718677333127	1081
FAM138D	0.0116033389664317	0.70314939492985	0.765300370677066	1081
JDP2	0.0115869462629115	0.703548930779057	0.76562374414022	1081
KCNK4	0.0115864706595445	0.703560523778791	0.76562374414022	1081
ING1	0.0115713907495143	0.703928137569491	0.765858302621376	1081
EZR	0.0115653298228002	0.704075908602813	0.765977705827276	1081

KCNN4	0.0115433756545302	0.704611265024803	0.766395186639883	1081
ENKUR	0.0115406980839187	0.70467656808023	0.766421364032035	1081
TMPRSS7	0.011539245972797	0.704711984411288	0.766421364032035	1081
SNORA15	0.0115308646699663	0.704916413072772	0.766560925309095	1081
RBMXL2	0.0115292930584917	0.704954748675163	0.766561233512964	1081
LOC1001(	0.0115189086111746	0.705208070566677	0.766795303155703	1081
CRYL1	0.0115067032518118	0.705505854159402	0.767077689553171	1081
DNALI1	0.0114797690936281	0.706163146993568	0.767750909179418	1081
KPNA4	0.0114740297061442	0.706303237537441	0.767861776588869	1081
PHC2	0.0114574028416832	0.70670913247884	0.768178679856808	1081
F13B	0.0114524164723032	0.706830875946794	0.768269558430353	1081
ADC	0.0114440245364738	0.707035784085845	0.768450815776064	1081
SPDEF	0.0114391229144787	0.707155478107103	0.768464718964072	1081
LCA5L	0.0114388142323482	0.707163016140778	0.768464718964072	1081
CREG1	0.0114342834198105	0.707273662116862	0.768543498750968	1081
FAM3B	0.0114209122879944	0.707600231576065	0.768815417997941	1081
PTP4A1	0.0114161103904437	0.707717523698433	0.768859920989318	1081
FLJ40292	0.0114085792834645	0.707901493991686	0.768977033287647	1081
HIST1H2I	0.0113860983756879	0.708450760071099	0.769359202173776	1081
TRA2B	0.0113847942062641	0.70848262891664	0.769359202173776	1081
C10orf96	0.0113814106596554	0.708565312056955	0.76940751462847	1081
CSNK2A1	0.0113382263253228	0.709620901060326	0.770470682971881	1081
FAM74A1	0.0113084520993038	0.710349020568707	0.771095000504182	1081
SLC4A1	0.0113037763528286	0.710463388516289	0.771177593709825	1081
FAM170B	0.0112504687401199	0.711767743186043	0.772468547919558	1081
PSG3	0.0112311425651856	0.712240833098839	0.772815446664662	1081
MCM4	0.0112246411858345	0.712400006743851	0.772927544547438	1081
CLEC5A	0.0112237886248844	0.712420880970803	0.772927544547438	1081
OR2V2	0.0112069110035212	0.712834159390229	0.773250990302954	1081
GGA1	0.0112022177385929	0.712949097254043	0.773292390253739	1081
KRTAP19	0.0111961621199363	0.713097408721532	0.773411612937185	1081
UPK1A	0.0111880183372548	0.713296879632985	0.773586306336823	1081
BPIL1	0.0111744478344724	0.713629314179016	0.773807138631676	1081

OR10Z1	0.0111742092494907	0.713635159245005	0.773807138631676	1081
EIF2C2	0.011162628011785	0.713918906830315	0.774010891668731	1081
RSPH1	0.0111607991829389	0.713963717897222	0.774017818159484	1081
OR51S1	0.0111572554827069	0.714050550580593	0.774070297661629	1081
ZMPSTE2	0.0111188279922938	0.714992391787564	0.775007895214446	1081
GGT3P	0.0111119094719169	0.715162007830109	0.775150040231225	1081
BCO2	0.0111069177310946	0.715284395223914	0.775240982503133	1081
NBPF4	0.0111015271321786	0.715416570020258	0.775342522221761	1081
GUCA2A	0.0110944784088469	0.715589414054055	0.77547360006395	1081
OR10R2	0.0110858934833908	0.715799947463728	0.775632829953031	1081
DNAJC10	0.0110806729937298	0.715927983298055	0.775715126589698	1081
LOC10012	0.0110796567257673	0.715952908844987	0.775715126589698	1081
ALDH3A1	0.0110713955678233	0.716155537762607	0.775866381332944	1081
PCOTH	0.0110708247736243	0.716169538887371	0.775866381332944	1081
OR14C36	0.0110670157085311	0.716262974644632	0.775925877834809	1081
FOXRED2	0.0110501151030106	0.716677594950311	0.776215567488631	1081
CCDC116	0.0110490232200846	0.716704384838558	0.776215567488631	1081
STK33	0.0110189931063479	0.717441324898691	0.776948056163729	1081
SAGE1	0.0110175587920492	0.717476529601084	0.776948056163729	1081
DRG2	0.011004332042921	0.717801203393428	0.777216083975308	1081
HRASLS	0.0110005234314924	0.71789470189323	0.777267302214842	1081
TGIF2LY	0.0109992618582387	0.717925673482828	0.777267302214842	1081
LXN	0.0109945615382775	0.718041070287016	0.77735046006728	1081
AKT2	0.0109630278268242	0.718815415386167	0.777979720784094	1081
LOC20072	0.0109385041965576	0.719417819593856	0.778505118958208	1081
CXorf40B	0.010936972999454	0.719455438056339	0.778505118958208	1081
CASKIN2	0.0109178312083659	0.719925770969279	0.778972212706849	1081
DLX5	0.0109006483961988	0.72034806000585	0.779387274763298	1081
NEUROD4	0.0108730961545986	0.721025368502582	0.780036305702406	1081
SCARNA4	0.0108636321191968	0.721258070510659	0.780246151349867	1081
MRGPRX	0.0108457785406957	0.721697125025793	0.780553451285547	1081
LGALS3	0.0108296106405252	0.722094804428947	0.780857793505885	1081
GAL3ST2	0.0108208920429921	0.722309285594447	0.781005880430472	1081

RPL27	0.0108165299452942	0.722416603213751	0.781079995263043	1081
SMN2	0.0108093104414073	0.722594231568931	0.781230118336076	1081
GCNT3	0.0107945530589827	0.722957368000798	0.78158077591371	1081
PROSC	0.0107783174860493	0.723356950405676	0.781970795058886	1081
C10orf41	0.0107601707792322	0.723803657893817	0.782411713257724	1081
PNLIPRP3	0.0107519082809898	0.724007082402142	0.782589616085805	1081
WNT7B	0.010732168357332	0.724493162411116	0.782946985503564	1081
CABP1	0.0107066580200993	0.725121498578252	0.783499924311247	1081
IL31RA	0.0106903349225171	0.725523644089165	0.783808323600377	1081
DCST1	0.0106587232568785	0.726302662692521	0.784565775105412	1081
TBC1D10.	0.0106115883022459	0.727464752699407	0.785652572944275	1081
CACNG8	0.0106099154418939	0.727506007801811	0.785655008425403	1081
SPDYE4	0.0105686188760748	0.728524689310565	0.786493551464896	1081
DEFB124	0.0105673588377595	0.728555778798035	0.786493551464896	1081
KNDC1	0.0105650846831626	0.728611891157509	0.786511978746545	1081
SELENBP	0.0105364343956944	0.729318930997463	0.787233020679628	1081
TFAP2D	0.0105313941725736	0.729443338837927	0.787303045189973	1081
COL4A4	0.0105306394747747	0.729461967658022	0.787303045189973	1081
PLXNB3	0.0105150173852109	0.729847616211776	0.787634875908621	1081
CCL24	0.0105095248709583	0.729983221217552	0.787739018182322	1081
PPP1R2P3	0.0105023883877392	0.730159426863457	0.787831107202221	1081
SPOP	0.0104984197620694	0.730257421654159	0.787866096787866	1081
DEFB135	0.0104654138660438	0.731072585838363	0.788703327891278	1081
LOC12195	0.0104557151796873	0.731312177160862	0.788913907226419	1081
ASB18	0.0104543419983841	0.731346101635809	0.788913907226419	1081
VEPH1	0.0104477987315634	0.731507760393748	0.789046039469482	1081
LARP7	0.0104326427406437	0.731882251885705	0.78940771881124	1081
LOC33953	0.0104095870623814	0.732452060145472	0.789846150118102	1081
FBXO16	0.0103982159515596	0.732733145086942	0.790040529107892	1081
CYorf15A	0.0103977982056685	0.732743472119725	0.790040529107892	1081
LOC10013	0.010385469342351	0.733048273826463	0.790284570064974	1081
H2BFXP	0.010335327334232	0.734288347957314	0.791494396147472	1081
OR4Q3	0.0103222702558506	0.734611379467895	0.79180022682615	1081

UCKL1AS	0.0102916062122097	0.735370190784392	0.792448512536192	1081
C1orf96	0.0102835842537913	0.735568744477681	0.792620077962176	1081
PBXIP1	0.0102507726676309	0.736381056585952	0.79332565297203	1081
PARP1	0.01024667354864	0.736482558633553	0.793392574527964	1081
GREB1	0.0102226961627583	0.737076376632288	0.793904914038253	1081
FSCN3	0.0102173876455364	0.737207867266226	0.793961640528101	1081
FAM83E	0.0102146474640966	0.737275743876388	0.793992294471818	1081
KRTAP5-8	0.0102083522457029	0.737431689506718	0.794117783990637	1081
C8orf75	0.0101818268680269	0.738088896685903	0.794783024574381	1081
FAHD2B	0.0101726098280614	0.738317307866651	0.794923667751894	1081
EGR4	0.0101717794198731	0.738337887679608	0.794923667751894	1081
PF4V1	0.0101662402617788	0.73847516813544	0.795028979376252	1081
OR4K15	0.0101395394188199	0.739137028741964	0.795613969757246	1081
ELANE	0.010132002813042	0.739323881109545	0.795772578806999	1081
LCE1F	0.0101160271632045	0.739720010479415	0.79611388028143	1081
VSTM2A	0.0101048284069956	0.739997733964533	0.796370230819783	1081
SPACA4	0.010086082827214	0.740462690597573	0.796742917534885	1081
ST6GALN	0.0100543540737408	0.741249891534676	0.797291850337234	1081
SLC25A44	0.0100516953124255	0.741315868583528	0.797320244133218	1081
CDA	0.0100430427074482	0.741530595753078	0.797508614139435	1081
LOC28562	0.0100105355700535	0.742337487843144	0.798248569050931	1081
AFG3L2	0.00999140088185207	0.742812581340589	0.798621587560724	1081
TRIP10	0.00999017773927566	0.742842953969365	0.798621587560724	1081
SERPINB1	0.0099601891902999	0.74358774358423	0.799265788579816	1081
FOXI1	0.00995806374371105	0.743640539853635	0.799265788579816	1081
OR2J2	0.00995431496490833	0.743733662763221	0.799323230706631	1081
TP53TG1	0.00995056661416968	0.743826778781729	0.799338016566623	1081
RPL39	0.00992511262189691	0.744459202282732	0.799889627712712	1081
SYT4	0.00991460615884051	0.744720293431411	0.800127483262708	1081
C7orf72	0.00991129197682279	0.744802658682066	0.800130242649101	1081
WDR69	0.00990598899777731	0.744934456297727	0.800186873313305	1081
SFI1	0.00989393508274769	0.745234066069523	0.80044617122217	1081
CPNE2	0.00989308012022209	0.745255318314372	0.80044617122217	1081

POLE	0.00988830909525228	0.745373917699446	0.800530872317286	1081
NR2C1	0.00988595319848149	0.745432483419734	0.800551091720361	1081
C17orf46	0.00986191886021569	0.746030041850594	0.801107419673786	1081
PDE4C	0.00985313653540352	0.746248432197131	0.801299220010192	1081
DERA	0.00981564917733132	0.747180860197424	0.802129412665979	1081
OR2J3	0.00980506200407478	0.747444263344393	0.802241178809356	1081
OR13C5	0.00977238787731693	0.748257363543553	0.802942768262126	1081
MADCAM	0.0097594669775834	0.748578979152033	0.80321776538381	1081
TMEM65	0.00975888865439168	0.748593375283248	0.80321776538381	1081
CYP11A1	0.00972546417240384	0.749425556	0.804067842779665	1081
CDHR4	0.0097092594865593	0.749829113825992	0.804415139952317	1081
LIN9	0.00967773862511271	0.750614298966879	0.80510736949814	1081
ASRGL1	0.00963603533939993	0.751653524851539	0.806009417490191	1081
DCLRE1B	0.00963516169808916	0.751675300391572	0.806009417490191	1081
LOC15762	0.00961305136808986	0.752226466818664	0.806428725137672	1081
FAM49A	0.00960985227261849	0.752306224344655	0.806471311697694	1081
SPSB4	0.00958762375482582	0.752860482501365	0.806979590294242	1081
NKX2-6	0.00957162434384146	0.753259499433144	0.807278428264121	1081
FBP2	0.00956255118197344	0.753485809	0.807478009049154	1081
MAFK	0.00952318044782049	0.754468065798818	0.808272664620144	1081
GATA3	0.00950316231511296	0.754967648788184	0.808764864920924	1081
CMBL	0.00948901024321541	0.755320897079147	0.809049039707376	1081
TRIM74	0.00948693392692975	0.755372728078814	0.809049039707376	1081
TRIP4	0.00948609918415273	0.755393566039019	0.809049039707376	1081
RPL23P8	0.00947559710166757	0.755655748179028	0.809263734056307	1081
ZNF207	0.00946783899135701	0.755849445717674	0.809408201359905	1081
LOC28635	0.00945639171461335	0.756135279040013	0.809671248405946	1081
CHMP4C	0.00945000744137991	0.756294705934449	0.809755878344378	1081
SNORA79	0.00939655350573971	0.757629953669635	0.811013094036601	1081
NCBP2	0.00938391720025678	0.757945706850817	0.811245441031762	1081
SPATA17	0.00938268617964357	0.757976469475809	0.811245441031762	1081
KRTAP2-4	0.009382565546644	0.757979484058212	0.811245441031762	1081
RAP1B	0.00938142091716604	0.758008088187682	0.811245441031762	1081

ERCC1	0.00936083753820731	0.758522520361228	0.811709756848709	1081
OR2T11	0.00934279863195264	0.758973447341037	0.812062890199978	1081
FXR2	0.00933226059282275	0.759236909525105	0.812301638025559	1081
FBXW9	0.00929258758043642	0.760229027100336	0.813233525432045	1081
GJB7	0.00923421288530321	0.761689540514551	0.814493115009588	1081
CSRP3	0.00922539566807334	0.761910217904075	0.814685846055074	1081
UBQLN3	0.00921870801831312	0.762077609355549	0.814814607851488	1081
DCAF8L1	0.00921735273207316	0.762111533439451	0.814814607851488	1081
SDHAP3	0.00921213329847019	0.76224218501427	0.814911044797137	1081
CLYBL	0.00920236992661598	0.762486597494914	0.815129086528075	1081
CFHR4	0.00919779163671946	0.762601216784032	0.815153450355112	1081
USP17	0.00919753533767304	0.762607633485195	0.815153450355112	1081
CA12	0.00915481950104537	0.763677294192479	0.816062390702315	1081
OR4N4	0.00915451467241245	0.763684929123874	0.816062390702315	1081
C10orf131	0.00913555450477766	0.764159862591781	0.816441285171649	1081
DYRK1B	0.00912853229995597	0.764335784381483	0.816525706258448	1081
CT47A2	0.00912754677300362	0.764360474987432	0.816525706258448	1081
LYG2	0.0091231543903356	0.76447052114702	0.816599216466112	1081
LETM1	0.00912156426868814	0.76451036101022	0.816599216466112	1081
SNAPC4	0.00911253264781299	0.764736656470591	0.816797622	1081
ACTRT1	0.00909651186611762	0.765138121043021	0.81709645082759	1081
MORC2	0.00908883645435054	0.765330481428045	0.817220855125452	1081
SLC2A7	0.00908697617939115	0.765377105625467	0.817220855125452	1081
FAM153B	0.0090855613975753	0.765412564966359	0.817220855125452	1081
DUSP3	0.00907546518605904	0.765665625304025	0.817360988288167	1081
FAM101A	0.00907001201580901	0.765802318755839	0.817415763860349	1081
CAPNS2	0.00905509830663986	0.766176194692649	0.817771508004843	1081
COQ6	0.00904498808254686	0.766429681658404	0.817998725139525	1081
TMEM45A	0.00904167730590315	0.766512696000988	0.818000649517397	1081
DGKK	0.00901080341606249	0.767286955994986	0.818683237620907	1081
PRAMEF2	0.00900548837270538	0.767420270873163	0.818752322674498	1081
TMPRSS1	0.00899689399484637	0.767635854386778	0.818895588149844	1081
ZFP64	0.00899498766551431	0.767683675659876	0.81890323781669	1081

POPDC3	0.00897003337199975	0.768309748616535	0.819397526914082	1081
IFNA16	0.00894498231935	0.768938400983062	0.820024567909147	1081
WBP5	0.00894293624045915	0.76898975373094	0.820035921280492	1081
KRT85	0.00893245091740236	0.769252931579031	0.82018631876043	1081
FOXD4L1	0.0089170810006154	0.769638758954301	0.820510838226841	1081
SCGB2A1	0.00890852617315217	0.769853533439489	0.820696376733794	1081
HFE2	0.00890197962865208	0.770017900588891	0.820828162099863	1081
MFAP2	0.00887067282584179	0.770804077631672	0.821573276904596	1081
PANX3	0.00886916464763157	0.770841956976493	0.821573276904596	1081
TTF2	0.00886531802531127	0.770938571062769	0.821592208329867	1081
LBR	0.00886243696079341	0.771010935941338	0.821625862491444	1081
GLIPR1	0.00885975328636488	0.771078344682431	0.821633771656074	1081
RGS3	0.00885889395276085	0.771099929855038	0.821633771656074	1081
CANT1	0.00884963156423215	0.771332598408869	0.821794753276215	1081
C14orf105	0.00884443291969742	0.77146319587626	0.821890426829935	1081
SSX4	0.0088259025518694	0.771928758099712	0.822299446455434	1081
CYP2E1	0.00881128632773387	0.77229603813419	0.822560203458457	1081
PLCXD1	0.0087966857187546	0.772662976656473	0.822864012727038	1081
TPTE2	0.00878178137970195	0.773037600799398	0.823175941859809	1081
CCNA1	0.00877100052069012	0.773308613204932	0.823377484831573	1081
CDKAL1	0.00874658995511951	0.77392235514483	0.823900324704994	1081
SERPINI2	0.00873793702781372	0.774139945024952	0.824073531187034	1081
TRIM54	0.00871804482821068	0.774640229192048	0.824403160075746	1081
LOC15165	0.00871069923823265	0.774824992699346	0.82449148165547	1081
C22orf43	0.00870970079999191	0.774850107383719	0.82449148165547	1081
RNU11	0.00869869132982791	0.775127054845253	0.824692916592013	1081
ZBED1	0.00869708506221802	0.775167463514439	0.824692916592013	1081
C9orf46	0.00865228244475298	0.776294801863886	0.82572779890955	1081
OR5B12	0.00864088245882988	0.77658172732357	0.825907756556031	1081
IGF2BP2	0.00864067374844771	0.776586980624688	0.825907756556031	1081
SPAG17	0.00863320957267683	0.776774862809493	0.826063960898339	1081
LOC65029	0.00862614988187636	0.77695257562641	0.826209334670293	1081
METTL4	0.00861673058073804	0.777189704804564	0.826417872963206	1081

MAPK13	0.00859633456577288	0.777703241756338	0.826920289516596	1081
LOC1002ﾐ	0.00857881197727986	0.77814450818173	0.827302148898087	1081
ONECUT2	0.0085725620715831	0.778301915096234	0.827425831421902	1081
RWDD2A	0.00855275999675428	0.778800699937056	0.827814368369035	1081
DFNB31	0.00854710295419352	0.778943209152645	0.827845473404381	1081
GAD2	0.00851329493365455	0.779795037432565	0.828619634406532	1081
RAB7L1	0.00850352126047389	0.780041344560459	0.828793928595488	1081
ANKRD27	0.00849351815874819	0.780293456367505	0.829018072808575	1081
GBA3	0.00848515613123327	0.780504225290753	0.829198271199358	1081
RNF32	0.00847192796042233	0.780837680585318	0.82950878423199	1081
DDR1	0.00846102251906426	0.781112614865948	0.829757099054763	1081
AGPAT6	0.00844259807690005	0.781577170807381	0.830137846747351	1081
SLC25A34	0.00842405249504019	0.782044859785602	0.830528425345614	1081
ST3GAL2	0.00841043743675936	0.782388259202189	0.830805517981556	1081
RBBP8	0.00833411768228552	0.784313980746921	0.832674842800881	1081
LOC21934	0.00831021902591214	0.784917270235805	0.83322750745114	1081
UGT2A3	0.00830410011010558	0.785071754815337	0.83330367778887	1081
TRIM37	0.00830146048878094	0.7851384000803	0.833330509463522	1081
VAMP3	0.00829881338645948	0.785205235805423	0.83335754045099	1081
FECH	0.00827711317397001	0.785753196166763	0.833895171045171	1081
CAGE1	0.00826932506502343	0.785949882549066	0.834059968600902	1081
TOMM20L	0.00825883713536296	0.786214773659154	0.8342971247031	1081
C3orf27	0.00825378484445493	0.786342386974136	0.834318406008642	1081
CXorf41	0.00822990838954689	0.786945548024978	0.83480874486378	1081
RAB40AL	0.00822153126049656	0.787157199242702	0.834989299075574	1081
APOBEC1	0.00821231431229601	0.787390086937752	0.835174908335002	1081
OR8H2	0.0082113247256651	0.787415092281295	0.835174908335002	1081
KRTAP9-8	0.00820858670393681	0.787484279051119	0.835202329688606	1081
YIPF7	0.00820537948125132	0.787565324138236	0.835202329688606	1081
OR1M1	0.00819405042201319	0.787851622797049	0.835417988274901	1081
SPINK4	0.00818826975280756	0.787997718142952	0.835499148815041	1081
HOXB8	0.00818509769498804	0.788077888996097	0.835499148815041	1081
PHEX	0.00818388373074999	0.788108571422448	0.835499148815041	1081

ORC3L	0.00818044052414153	0.788195598781362	0.835499148815041	1081
DUSP12	0.00817983511995135	0.788210900700064	0.835499148815041	1081
VN1R5	0.00816977886709379	0.788465089869386	0.835628642435753	1081
GJA4	0.00815215539789071	0.788910607745614	0.836056825347751	1081
ZNF773	0.00814713033108682	0.789037653095923	0.836085234811976	1081
C1orf49	0.00814617031158993	0.789061925254172	0.836085234811976	1081
OSTBETA	0.00809680261048541	0.790310363158749	0.837226885665768	1081
NCRNA0(	0.00807056843635668	0.790974006498795	0.837648995481658	1081
KRTAP10	0.00806973361505901	0.790995127378882	0.837648995481658	1081
ITM2B	0.00806806303614139	0.791037393288076	0.837649720762631	1081
ZNF642	0.00802305410326777	0.792176356938347	0.838679457143315	1081
C21orf7	0.00801587199900293	0.792358143149708	0.83878375164219	1081
TRPM8	0.00801071623675273	0.792488647577816	0.838833743456027	1081
ARFIP2	0.00799933803102304	0.792776677175063	0.83900636520576	1081
C16orf81	0.00798529272346384	0.793132261123923	0.839294500201788	1081
C11orf53	0.00797989212903082	0.7932689989874	0.839395103718939	1081
PKD1L1	0.00797474835912032	0.793399240202311	0.839444731821195	1081
GH2	0.00796358400640734	0.793681943492753	0.839655642876877	1081
TMSB4Y	0.0079589049037696	0.793800435594665	0.839736899388464	1081
GTF2H2	0.00791245943974783	0.794976863018677	0.840804793695136	1081
SLC34A3	0.00790265880734407	0.795225164997192	0.841009033882672	1081
OLIG3	0.00790154177405237	0.795253466688063	0.841009033882672	1081
INGX	0.00789775995895607	0.795349286565166	0.841066214060644	1081
MC4R	0.00788500357343593	0.795672517926527	0.841288084701885	1081
LOC10012	0.00787005094274455	0.796051444059375	0.841632011156509	1081
DLX4	0.00786240659501972	0.796245183923455	0.841716348279418	1081
ZNF671	0.00782907206013867	0.797090167427984	0.842553333132371	1081
ARF4	0.00782495692318662	0.797194496845208	0.84261939970338	1081
FLJ25758	0.00779037336729745	0.798071422683256	0.843413535303441	1081
ATPAF2	0.00778824075375188	0.798125507127767	0.843426446051978	1081
UHRF1BP	0.00778063158727401	0.798318488343031	0.843586128182457	1081
C2CD4D	0.00776724400391827	0.79865804976094	0.843812157782249	1081
LOC15117	0.00776288948319147	0.798768505619423	0.843881433627719	1081

GPX5	0.00775970482193986	0.798849289614327	0.843881433627719	1081
PGLYRP2	0.00775538318375235	0.798958918275098	0.843949816380957	1081
FAM109A	0.00775385005189078	0.798997810772778	0.843949816380957	1081
DBH	0.0077508958727787	0.799072753809251	0.84398472319443	1081
TSPAN15	0.00774692452411885	0.799173503804309	0.844046882360166	1081
ZNF18	0.00770020332599943	0.800359034134807	0.845135820369179	1081
KRTAP3-:	0.00768750853503567	0.800681238493813	0.845373351648735	1081
LELP1	0.00768334786275857	0.800786847190546	0.845440544869428	1081
C6orf48	0.00765367189988112	0.801540205757243	0.846147222395832	1081
PAX3	0.00764792707736316	0.801686066022178	0.846212512445207	1081
C14orf149	0.00762208598399526	0.80234225316476	0.846743967249316	1081
HAAO	0.00760429756201653	0.802794038425529	0.847115703948577	1081
RLN2	0.00757429379238925	0.80355621467402	0.847831139402301	1081
RSPO3	0.00748242753425151	0.805891021356672	0.85002746872769	1081
C14orf183	0.00747569331799228	0.8060622416175	0.850163552742646	1081
TSTD1	0.00746368967709033	0.806367462219122	0.850440946882583	1081
DCK	0.00746152634189659	0.806422473202149	0.850454440511846	1081
BOLL	0.00744680732869372	0.806796785184811	0.850804650450087	1081
LOC6453:	0.00740873481590107	0.807765193315225	0.851692129139287	1081
C22orf15	0.00740001579256626	0.807987011	0.851881422197931	1081
NEUROG:	0.00738517177552544	0.808364687647153	0.852179384041233	1081
WFDC12	0.00738392047227351	0.808396526633307	0.852179384041233	1081
CXorf48	0.00737703618969581	0.8085717005303	0.852301831564414	1081
SRGAP2	0.00737603017671975	0.808597299819264	0.852301831564414	1081
B3GAT1	0.00734380320755848	0.809417464445978	0.852987801907524	1081
KLF17	0.00733934320682457	0.80953098633045	0.853062808998636	1081
STK11IP	0.00733060531578547	0.809753406381793	0.853207927797543	1081
GRXCR1	0.00731700312569048	0.810099676045497	0.85342486041883	1081
HS3ST5	0.00731626294640086	0.810118519785036	0.85342486041883	1081
OR6C75	0.00730914278472819	0.810299792927984	0.853515781884143	1081
BEGAIN	0.00730712236282627	0.81035123296903	0.853525336618274	1081
TERF1	0.00721772906163275	0.81262800645708	0.855744442764134	1081
SLC17A2	0.00720773394577188	0.812882672514551	0.855967876107137	1081

PP14571	0.00719145561487336	0.81329747121033	0.856359897424843	1081
CPPED1	0.00717534877126452	0.813707951607531	0.856702554883125	1081
DUS3L	0.00713854058289034	0.814646194188465	0.857600730660881	1081
FAM19A1	0.00713517661807174	0.814731955085369	0.857646195077325	1081
TMED7-T	0.00712613594055383	0.814962449041686	0.857821221020589	1081
RAB11FIF	0.00712531468587869	0.814983387893425	0.857821221020589	1081
ERMN	0.0071221130893996	0.815065017612861	0.857862318694414	1081
KRT24	0.00710999228762837	0.815374074603204	0.858097938192537	1081
MYLPF	0.00710424026105582	0.815520750190293	0.858162636470092	1081
KCNU1	0.00710013657995076	0.815625397226539	0.858227925156888	1081
BPIL3	0.00708968178297419	0.815892017457517	0.858373965515024	1081
DCAF10	0.00708697667396977	0.815961007131938	0.858401717513859	1081
TCEAL4	0.00707940301331249	0.816154169357669	0.858515259866861	1081
YPEL4	0.0070738980551491	0.816294577417426	0.858618121453323	1081
LOC72792	0.00705978661587321	0.816654527077411	0.858907040199146	1081
AKR1CL1	0.00705576570592456	0.816757098116786	0.858970072647489	1081
LRRC19	0.00703706575801889	0.817234163906378	0.859337208053763	1081
CEP152	0.0070131531520697	0.817844311496939	0.859799272263117	1081
RPLP1	0.00700807324969388	0.817973943119182	0.859890679128316	1081
FAHD1	0.00699922224530888	0.818199819580331	0.860083248040376	1081
IGFBPL1	0.00699167848851592	0.818392347153682	0.860240742677324	1081
RNU6ATA	0.00698988114808919	0.81843821949214	0.860244074489678	1081
CCDC45	0.00695796035603935	0.819253016151853	0.860946851323954	1081
ROBO2	0.0069569898085645	0.819277792994798	0.860946851323954	1081
LOC73178	0.0069492101118978	0.819476405267465	0.86111064545997	1081
UGT2A1	0.00694727551768343	0.819525796412471	0.861117628245839	1081
TBC1D9B	0.00694239550298829	0.819650388771958	0.861164012079275	1081
GK	0.00692825514231803	0.82001143402305	0.861448182018896	1081
VCY	0.00691919594599075	0.820242762140027	0.861646266011934	1081
JKAMP	0.00690580078093752	0.820584838568893	0.861960661850576	1081
GOLGA8C	0.00690181139041234	0.820686723445314	0.861977791647854	1081
MTNR1B	0.00689030545424871	0.820980589982008	0.862241488201218	1081
ZNF256	0.00687725210786501	0.821314008419082	0.862458612469183	1081

LOC34737	0.00687493149880794	0.821373286546032	0.862458612469183	1081
RNF212	0.00686632359874525	0.821593177453953	0.86257007295236	1081
CTBS	0.00686150191218194	0.821716354731731	0.862654435669953	1081
TEX13A	0.00684369635923231	0.822171262528517	0.863037130094735	1081
C12orf75	0.00684083519386631	0.822244367005998	0.863037130094735	1081
ACMSD	0.00683902161995841	0.822290705696473	0.863037130094735	1081
PECI	0.00683884986215927	0.822295094318111	0.863037130094735	1081
SLC39A1	0.00683528057224657	0.822386295292983	0.863060180415636	1081
DNTTIP2	0.0068303530687442	0.822512204700416	0.863130091352288	1081
PTGER4	0.00681570646963689	0.822886486998557	0.863432901744149	1081
CELSR3	0.00681179732636175	0.822986388881773	0.863492750209548	1081
POTEC	0.00679084062106679	0.82352200627925	0.86396473369938	1081
OTX1	0.00675402218821165	0.824463220884479	0.864792645744003	1081
NBPF22P	0.00675325339141872	0.824482876850798	0.864792645744003	1081
SNAP29	0.00673687916663915	0.824901545691659	0.865054614098744	1081
RAB22A	0.00673657059015446	0.824909436091757	0.865054614098744	1081
EFEMP2	0.00673508820719351	0.824947341347777	0.865054614098744	1081
IPPK	0.00673193456935678	0.825027982762728	0.865094144948738	1081
SPTBN2	0.0067262678281025	0.82517289117249	0.865201056194754	1081
PRTFDC1	0.0067189768181339	0.825359343703049	0.865306478214828	1081
RESP18	0.0067120182086626	0.825537304952563	0.865402976961513	1081
C7orf23	0.00669684556180671	0.825925365113784	0.865674634766763	1081
CENPV	0.00668958273285083	0.826111136439161	0.865824298609172	1081
GSTA5	0.00667522644580923	0.82647837544969	0.866085785495236	1081
KRTAP17	0.00666625082533724	0.826707994328012	0.866269573250341	1081
NRTN	0.00666383827282481	0.826769715994957	0.866289188011984	1081
C21orf128	0.00661923620098299	0.827910987634257	0.867304568169116	1081
STK31	0.00659103432375687	0.828632800816346	0.867925324829475	1081
POU4F2	0.00658905088950624	0.82868357128724	0.867933375459158	1081
ROS1	0.00656030972735889	0.829419346227419	0.868478233263233	1081
LHFPL5	0.00655704217430974	0.829503005192642	0.868486164354188	1081
DNAJA3	0.00655664617298441	0.829513144122061	0.868486164354188	1081
ARHGEF1	0.00654785868450894	0.829738139867232	0.868676583911516	1081

HSBP1L1	0.0065421422790474	0.82988451077864	0.868784673368794	1081
C12orf11	0.00653773243899724	0.829997430574348	0.868857734302652	1081
G0S2	0.00651629135178962	0.830546507941401	0.869387342295637	1081
SERGEF	0.00649324568675441	0.831136768517502	0.86993540317216	1081
NPFFR1	0.00648323484609508	0.831393202344992	0.87013799154405	1081
C22orf32	0.00647537240459019	0.831594616179244	0.870258365866539	1081
CNTNAP1	0.00646748025658831	0.831796802118584	0.870381494667806	1081
AMELX	0.0064585816937885	0.832024784347375	0.870572860368663	1081
LRRC66	0.00641010817679776	0.833266928910494	0.871520557037133	1081
TMEM21(	0.0064097301728956	0.833276616981496	0.871520557037133	1081
MRFAP1L	0.00637545087933634	0.834155284873906	0.872213132657783	1081
RDH10	0.00635794292002902	0.834604139502331	0.872637171862483	1081
MXRA7	0.00634544869846088	0.834924488970065	0.872795783295375	1081
OR4C16	0.00633852647390261	0.83510198527261	0.872885876268185	1081
CEACAM	0.00629355483347586	0.836255329997038	0.873955365365757	1081
OR4A16	0.00627597743215117	0.836706216537839	0.874378232767972	1081
MMP10	0.00627439680832512	0.836746764522262	0.874378232767972	1081
FAM19A5	0.00626437188972069	0.837003945370766	0.874601612090569	1081
OR4A47	0.00620894854131526	0.838426097933781	0.875815076114909	1081
PIGQ	0.00619691322478804	0.838734991689545	0.87598159146847	1081
OR52H1	0.00619596069721959	0.838759439952113	0.87598159146847	1081
LPCAT3	0.00619110195746001	0.83888415030402	0.87606641824129	1081
CCDC19	0.00618159973458164	0.839128057642957	0.876184871259907	1081
WHAMMI	0.0061672910311391	0.839495368997198	0.87647754384289	1081
C11orf66	0.00615538327412017	0.839801073512848	0.876705841629079	1081
C14orf115	0.00614997367539285	0.839939960603206	0.876805394670514	1081
ACY1	0.00612451933145683	0.840593547115451	0.877302129652435	1081
JMJD7-PL	0.00612296125432215	0.840633557144748	0.877302129652435	1081
CTRB1	0.00611996883545591	0.840710400941603	0.877336874583951	1081
JUNB	0.0061157463933228	0.84081883368946	0.877404579366701	1081
CLCA1	0.00604567586603694	0.842618690801485	0.879100602112793	1081
PDHB	0.00603318345301276	0.84293966201429	0.879389927047016	1081
HOXD8	0.00603110268602552	0.842993126300425	0.87940016204868	1081

TCP11	0.00602437628679591	0.843165962768707	0.87948937606667	1081
HSPA4	0.00601709812513627	0.843352985403938	0.879638909131321	1081
GYPB	0.00600023676993135	0.843786295596026	0.879999737253155	1081
TMEM189	0.00599448970944852	0.843933996767799	0.880062654772094	1081
C1orf141	0.00599017603750281	0.844044863026724	0.880110756568196	1081
IL6	0.00597914765353867	0.84432831901915	0.880337145419015	1081
FAM155B	0.00595961615772624	0.844830374813378	0.880678287224605	1081
ATP6V0A	0.00595423004924727	0.844968835452682	0.880731473596227	1081
OR4A5	0.00593014281281907	0.845588104270547	0.881285755110464	1081
NCRNA00	0.00591537174320784	0.845967907018849	0.881635979663462	1081
TUBA3E	0.00591031543267701	0.846097926174554	0.881725866369376	1081
TTTY14	0.00590367541874316	0.846268675407102	0.88176702733049	1081
INPP4A	0.00590367302448155	0.846268736977299	0.88176702733049	1081
DEFB131	0.00589706073444502	0.846438780451273	0.881852980564278	1081
DEGS2	0.00587603610945498	0.846979502921854	0.882233820390938	1081
TRIML1	0.00587113281209525	0.847105618883392	0.88230424747762	1081
HORMAD	0.00586802968349668	0.847185435367309	0.88231146127666	1081
AGR3	0.00581524139470274	0.848543457639749	0.883634425130666	1081
OR52I1	0.00581020193759032	0.848673125374457	0.883723772907497	1081
NMNAT2	0.00576593355065736	0.849812348312225	0.88477284390665	1081
ATRIP	0.00576027675402877	0.849957945525391	0.884872303332148	1081
DKFZp566	0.0057564966275081	0.85005524299538	0.884888531942018	1081
RIC8A	0.00573720720602149	0.850551772940486	0.885313912673664	1081
IAPP	0.00572100843651283	0.850968791990566	0.885656453536369	1081
CIAO1	0.00570743587601744	0.85131823430649	0.885882836412224	1081
LOC28600	0.0056961759116341	0.851608158112497	0.886138757498773	1081
RAB40C	0.00568891098365314	0.851795227557752	0.886287632826348	1081
PRG3	0.00568527409149567	0.851888879432625	0.88629702935291	1081
SNORA11	0.00568514319384774	0.851892250154401	0.88629702935291	1081
NINL	0.00567575184757343	0.852134092068656	0.886457076412243	1081
EFR3A	0.00564743124073695	0.852863476143792	0.886946876002203	1081
NGLY1	0.00564721305877822	0.852869095807331	0.886946876002203	1081
AADAC	0.00563762153426813	0.853116149908571	0.887148989781759	1081

KCNA10	0.00563624836979428	0.85315152042899	0.887148989781759	1081
TUBA4B	0.00561953582373207	0.853582032493806	0.887550843635167	1081
CAMK2G	0.00561468614918222	0.853706967362003	0.887634934581035	1081
PKHD1L1	0.00560398417912509	0.853982679073237	0.887874826685794	1081
C3orf39	0.00560059827997784	0.854069912708727	0.887874826685794	1081
HOXD4	0.00558966100947576	0.854351710548902	0.888045355833411	1081
TMEM121	0.00558909876851061	0.854366197141094	0.888045355833411	1081
C9orf50	0.00558698303711049	0.854420711107344	0.888056197774092	1081
C9orf173	0.00557321470791774	0.85477548302153	0.8883332699891	1081
DLX3	0.00556525490722411	0.854980598537836	0.888454769294579	1081
KDM2A	0.00554575017746946	0.85548325582263	0.888803071806741	1081
GOLT1B	0.0055453749965864	0.855492925201092	0.888803071806741	1081
PPIEL	0.0055431541134027	0.855550163529178	0.888803071806741	1081
PI3	0.00554197705891563	0.855580499800265	0.888803071806741	1081
SERPINA	0.00552062432271788	0.856130861661033	0.889328943854046	1081
POLR3D	0.00548785968746147	0.856975498381543	0.890068643787182	1081
CST1	0.00547451650937448	0.857319517952985	0.890334142136451	1081
SPATA2	0.00543084787080749	0.858445590528659	0.891187956869826	1081
LOC2545	0.00542461117510888	0.858606438213752	0.89130303503123	1081
KIAA154	0.00541963581983876	0.858734759464342	0.891344380122072	1081
MARVEL	0.0053402003542428	0.860784011792848	0.893142799782903	1081
NEB	0.00533872544247581	0.860822070021586	0.893142799782903	1081
CNGB1	0.00531659768106032	0.861393087555532	0.893677388977026	1081
MAPK8IP	0.00531532088198559	0.861426038180983	0.893677388977026	1081
ALG2	0.00527143299460439	0.862558808275973	0.894806488115736	1081
IDI2	0.00526637204135558	0.862689452411205	0.894895932998132	1081
SAG	0.00525523320712092	0.862977004998187	0.895084474681505	1081
ALDH7A1	0.00522583021819226	0.863736139604225	0.895704958937872	1081
C10orf110	0.00519598001568837	0.864506949013385	0.89636586998171	1081
CXXC1	0.00517773137743147	0.864978239508385	0.896705467389804	1081
BET1L	0.00517347118281304	0.865088270477802	0.896737841348941	1081
MGC1443	0.00516523470060505	0.865301007159556	0.896859360710916	1081
TTC36	0.005154531618639	0.865577467184721	0.89692187009085	1081

MOSC1	0.00514900457570234	0.865720236837188	0.897015472808885	1081
LOC15332	0.00514758701553756	0.865756854694621	0.897015472808885	1081
KAAG1	0.00514495819195696	0.865824762194529	0.897039695248343	1081
RPL37A	0.00508910267427338	0.867267849189333	0.8981652694797	1081
GRK5	0.00503442301131795	0.868680984765956	0.899536261854127	1081
FAM171A	0.00502291887257379	0.868978350045867	0.899751689025185	1081
LOC28573	0.00500949919893522	0.869325252504273	0.900018358089145	1081
LCE2C	0.00500062505686977	0.869554665453247	0.90011709241846	1081
NKX2-4	0.00497893975881972	0.87011531652346	0.900512358148734	1081
COPZ2	0.00496817759510966	0.870393585419193	0.900666868964686	1081
TPRG1	0.00496594507508707	0.870451311960904	0.900675005365063	1081
FBXO10	0.0049572627504386	0.870675818435322	0.900861035561354	1081
C20orf96	0.00495531521809949	0.870726178914817	0.900866872489291	1081
SOHLH1	0.00492817171215614	0.8714281266508	0.901546816525285	1081
MCART1	0.00491998170437155	0.871639945169451	0.901719647483109	1081
DUSP26	0.0049134470511902	0.871808957776026	0.901801871856126	1081
OR2W1	0.00490374964367215	0.872059782793968	0.901982748777122	1081
DEFA4	0.00487519618237948	0.87279839720928	0.902547287704992	1081
IKBIP	0.00482827742609027	0.874012321085734	0.903524308937861	1081
CCDC105	0.00482379424688425	0.874128329442096	0.903571880474976	1081
CRLF2	0.00482303232024857	0.874148045598253	0.903571880474976	1081
OR10AG1	0.00481631162263705	0.874321958533616	0.903705277304243	1081
KIAA009(	0.00480975812467954	0.874491550646404	0.903834194642236	1081
POU5F2	0.00479396401748978	0.874900295865143	0.904163877299713	1081
SLC22A24	0.0047828184038149	0.875188759986459	0.904415592999241	1081
AP1S2	0.00477236519447844	0.875459318763426	0.904628093520581	1081
OR6K3	0.00475648317012716	0.875870418519745	0.904899777828592	1081
LOC10013	0.00475603983083945	0.875881894657717	0.904899777828592	1081
LOC64516	0.00473948710180982	0.876310391899918	0.9052960433243	1081
SYTL3	0.00471924343322593	0.876834484973604	0.905744574904792	1081
OR4F15	0.00471069841126482	0.877055725246221	0.90588020842315	1081
GNAO1	0.00468411621507908	0.877744029591622	0.906544653992475	1081
LOC10012	0.00464088483136952	0.878863635491726	0.907561407472874	1081

A1CF	0.00463183548840068	0.879098025974298	0.907756919024667	1081
PRSS45	0.00462762051378457	0.879207203252249	0.907823121963945	1081
TMSB15B	0.00462004865130772	0.879403337199924	0.907979100399389	1081
C9orf98	0.00460368335374014	0.879827272720284	0.908323702605961	1081
DPF3	0.00459700316474613	0.880000329843242	0.908455808378195	1081
LPHN1	0.00458550358047377	0.880298251729166	0.90871679636608	1081
RPL13	0.00458035486060339	0.880431646066542	0.908780401037504	1081
SNORD89	0.00457618628676191	0.880539649014801	0.908780401037504	1081
OR4L1	0.00457267828484063	0.880630539024727	0.908780401037504	1081
DPCR1	0.0045555178373546	0.881075177477619	0.909192677506742	1081
DNAH11	0.00454655865925619	0.881307330544013	0.909385656600376	1081
MMP23A	0.00449989923124576	0.882516548653175	0.910540121516937	1081
SERF1A	0.0044944311694755	0.882658276004037	0.910639709623135	1081
ARV1	0.00448106753431556	0.883004665449773	0.910874915117249	1081
PON1	0.00447545643604389	0.883150113469334	0.910876020451975	1081
TFAP2E	0.00446515086550596	0.883417259442036	0.911002850855379	1081
JPH3	0.00444941546098874	0.883825185723211	0.911283568758014	1081
SSRP1	0.00443478794890719	0.884204418475344	0.911627924161206	1081
SDHC	0.00443054170872489	0.884314511427772	0.911671667753824	1081
SLCO4C1	0.0044205311203227	0.884574066388846	0.911869029137412	1081
OR4M2	0.00437936848632734	0.885641462626887	0.912735829775343	1081
LTB4R2	0.00437677157583092	0.885708810586385	0.912758542524183	1081
DAD1L	0.00434325361710869	0.886578135053122	0.913607679709711	1081
GDAP1L1	0.00429171567265512	0.887915092892359	0.914798217476427	1081
ZBTB8B	0.00428027782785193	0.888211848014494	0.915057158548225	1081
LPA	0.00426751837223558	0.888542910819084	0.915351415926896	1081
PPIL3	0.00426563119940589	0.888591877937756	0.915355051189205	1081
CSRP2	0.00426209731387905	0.888683574015528	0.915402699726073	1081
DUSP11	0.00425577569490743	0.888847608976463	0.91552485340172	1081
PHLDA3	0.00421772719506062	0.889835001182528	0.916495020135079	1081
MYH16	0.00419380632110842	0.890455856484194	0.916931840321337	1081
C19orf29	0.0041864166474409	0.89064766565217	0.917050714263376	1081
TTLL10	0.00418153919215261	0.890774270230532	0.9171341957372	1081

VPS45	0.00417611684286849	0.890915021939295	0.917232233708136	1081
ATP10B	0.00416599459202018	0.891177781458537	0.917408983978856	1081
ATF3	0.00414926129244454	0.891612180742725	0.917809268322034	1081
ZNF574	0.004140616927209	0.891836602658313	0.917918333436695	1081
OR6M1	0.00413815980866223	0.891900395070234	0.917918333436695	1081
CPN2	0.00412698168168179	0.892190613600461	0.918170110394957	1081
ZUFSP	0.00411514363634078	0.892497981360551	0.918415499672177	1081
BECN1	0.00410316004800255	0.892809144483033	0.918618939456086	1081
GOLGA2I	0.00409844159791293	0.892931667206466	0.918648113970895	1081
SLC25A2	0.00409499487356175	0.893021168983002	0.918648113970895	1081
ISLR	0.00409328738179001	0.893065508265377	0.918648113970895	1081
PLSCR3	0.00408595385863583	0.893255945277439	0.918797090508757	1081
PLEKHG5	0.00408196147435415	0.893359622141901	0.918809903918343	1081
TRIM77	0.00404740762362651	0.894257015140357	0.919592014803598	1081
FAM92A3	0.00404112894781229	0.8944200922571	0.919712763948715	1081
RPS20	0.0040179106289055	0.895023184268148	0.920211472724616	1081
C11orf90	0.00401718149339318	0.895042124426216	0.920211472724616	1081
ZNF547	0.00400618579639588	0.895327757910785	0.920322657537373	1081
LOC1001	0.00397370712564941	0.896171530205006	0.920949623832059	1081
MYST1	0.00396191534281242	0.896477901237378	0.921193121831232	1081
C1orf146	0.0039610673885242	0.896499933159658	0.921193121831232	1081
CA14	0.00393216740605172	0.897250872786474	0.921870700886597	1081
TBC1D3	0.00392186977355926	0.89751846959843	0.922033860540171	1081
ANKRD2	0.0038740025584931	0.898762511153105	0.92309425854896	1081
MOS	0.00387106195372846	0.898838943922046	0.923125695911782	1081
H2BFM	0.00386493033392927	0.898998321185463	0.9232423113928	1081
OR10A2	0.0038232525015352	0.900081747436626	0.923996827834669	1081
LSM5	0.0038225518465979	0.900099962741502	0.923996827834669	1081
ARL5C	0.00381672206354939	0.900251524796958	0.924105323161005	1081
FLYWCH	0.00380560915781321	0.900540446948523	0.924354799947925	1081
CRYGB	0.00380155152855634	0.900645943699216	0.9244159849096	1081
EXOC3L2	0.00378834594068153	0.900989295868614	0.924680790618269	1081
OR51E1	0.0037862893348538	0.901042770358907	0.924680790618269	1081

TMEM207	0.00378456732213582	0.901087545327736	0.924680790618269	1081
RRAD	0.00378265807417641	0.901137189067078	0.924684630896306	1081
TTC39A	0.00377686806918419	0.901287741541773	0.92476979285207	1081
FLJ46361	0.0037759353835481	0.901311993688635	0.92476979285207	1081
CACNA1H	0.00375208361185418	0.901932230284484	0.925284431395123	1081
HTR4	0.00373278699433735	0.902434059348584	0.925678279653002	1081
SLFN12	0.00373128429223373	0.902473140351698	0.925678279653002	1081
CNPY4	0.0037252426723229	0.902630268064375	0.925771422955052	1081
HOPX	0.00370776337703481	0.903084883083345	0.926113758485152	1081
TMEM80	0.0037061224032265	0.903127564429924	0.926113758485152	1081
OR1A2	0.00370250802914904	0.903221574443756	0.926115876071128	1081
MYL10	0.00366286040136273	0.904252899490516	0.926978651396368	1081
RPS6KL1	0.0036361198597853	0.904948573043661	0.927461793038489	1081
HTR3B	0.00362512889015887	0.905234531823105	0.927660476323962	1081
PLCH2	0.0035956634754329	0.906001211986063	0.928162856766642	1081
LOC10013	0.00357855453003751	0.906446421174116	0.928477304853438	1081
LCE1D	0.00356149309871426	0.906890423186619	0.928846681552054	1081
ZBTB22	0.00355358289944599	0.907096285911843	0.928989873306399	1081
SLC22A7	0.00354349350165644	0.907358871263166	0.929175704635852	1081
SNTB1	0.00349852082105745	0.908529446967245	0.930185287108922	1081
SNORA2A	0.00349119412527533	0.908720169425124	0.930311723650616	1081
LGALS14	0.00346767594028777	0.909332410726506	0.930865461359086	1081
SNORA36	0.00346305873538694	0.90945261525467	0.93094120601145	1081
SMYD3	0.00344092962942959	0.910028754121381	0.931350722516039	1081
GP6	0.00344058955523642	0.910037608439102	0.931350722516039	1081
NCRNA00	0.00343687796728982	0.910134245661835	0.93140230525058	1081
SNORA52	0.00343291422849552	0.910237449507587	0.93146060247538	1081
ELK1	0.00341369206218982	0.91073795836963	0.931846146701434	1081
TUBA3D	0.00340980371139743	0.910839207979316	0.931874500141014	1081
NCALD	0.00340849798666188	0.910873208353274	0.931874500141014	1081
TRAF3	0.00338745892130651	0.911421076792321	0.932245625386205	1081
NCRNA00	0.00337853541262621	0.911653462415247	0.932435976866122	1081
FSTL3	0.00337322132412527	0.911791855261719	0.932503199528843	1081

CLIP2	0.00336584731246725	0.911983898352325	0.932631891583714	1081
NPAS1	0.00336145133229128	0.912098386323588	0.932666802760575	1081
CTNNBIP	0.00335809575598542	0.912185779464466	0.932696301254272	1081
ABCB4	0.00335363313533684	0.912302006280009	0.932720458613011	1081
RAD23B	0.00334320754118195	0.912573543178572	0.932950724553785	1081
MMD	0.00333223065334535	0.912859449658264	0.933195656823594	1081
MDGA2	0.00330162322987737	0.913656716840121	0.933915900179838	1081
VSIG10	0.00329599689044431	0.913803282117012	0.934018322504806	1081
OR10A4	0.00328710888558241	0.914034819239439	0.934207581612304	1081
OR6W1P	0.00328138314446608	0.9141839816159	0.934312633029897	1081
SPEF1	0.00325552628179722	0.914857621205259	0.934953671833398	1081
OR1J1	0.00325102741584105	0.914974834801161	0.93500930187149	1081
C1QTNF2	0.00324056903357725	0.915247324954295	0.935162170886807	1081
CGRRF1	0.00323609496049931	0.915363898685849	0.935233845870387	1081
ITGA3	0.0032312785487418	0.91548939421788	0.935314628589065	1081
HSPH1	0.00322518455832175	0.91564818111685	0.93542521814464	1081
CASC5	0.00322356010659482	0.915690508901082	0.93542521814464	1081
SCTR	0.00322139621835272	0.915746892969262	0.93543538151543	1081
ABCC8	0.00320897316355152	0.916070606579601	0.935542881538595	1081
IFNGR2	0.0032084464143003	0.916084332649805	0.935542881538595	1081
WSB2	0.00319743641191753	0.916371237803295	0.935741006972191	1081
SNORD94	0.00318547297893456	0.916683000346003	0.93596447247695	1081
MARK2	0.00315797983061231	0.917399509817689	0.936553647457423	1081
P4HA1	0.00313637535042954	0.917962599495189	0.93699032840808	1081
OR52D1	0.00313621431539074	0.91796679679632	0.93699032840808	1081
CYP24A1	0.00310672561865771	0.918735443916805	0.937589510315633	1081
SHC3	0.00309599792973647	0.919015088798919	0.937775248903567	1081
PIGO	0.00306192007742769	0.919903482112171	0.93858669201528	1081
TBX6	0.00303963393127582	0.920484525351488	0.939036854368042	1081
TRPM1	0.00303067451664995	0.920718126706548	0.939227600896466	1081
KLK9	0.00301538468889255	0.921116798482505	0.939444000882445	1081
PARD6B	0.00299787136662729	0.92157347067168	0.939814597888073	1081
OR51L1	0.0029814434699112	0.92200186310201	0.940162072907007	1081

C6orf192	0.00298122574306626	0.922007540945685	0.940162072907007	1081
OR11A1	0.00295997261973461	0.92256179534529	0.940679625815199	1081
CHORDC	0.00295306659611643	0.922741903746277	0.940768037399097	1081
TSPYL5	0.00289849858018636	0.924165170493199	0.941980740719769	1081
PSD2	0.00288104575503737	0.924620434069653	0.942397098119562	1081
DUSP5P	0.00287472038759468	0.924785439778069	0.942422232108924	1081
NAT10	0.00286410785690523	0.92506228915544	0.942608994184944	1081
LOC39059	0.00284987446486114	0.925433610264714	0.942891972424776	1081
IGFL3	0.00284052504944112	0.925677526894901	0.943092792155858	1081
SLC13A5	0.00283535327832884	0.925812456081308	0.943182559308079	1081
C1orf52	0.00280808408879566	0.926523932142418	0.943716483618618	1081
IER2	0.00280428174053199	0.926623143276913	0.943722104212003	1081
ATP5SL	0.00280028026052839	0.926727551400951	0.943780725088325	1081
OR6C70	0.00279734183591903	0.926804222685547	0.943811094227092	1081
POM121L	0.00278027577478137	0.927249534840482	0.944142190220507	1081
PLCL2	0.00277918386784928	0.92727802721897	0.944142190220507	1081
HYAL1	0.00277769715666144	0.927316821826902	0.944142190220507	1081
CLLU1	0.00277243010740154	0.927454262895481	0.944234402690123	1081
CHERP	0.00276679191757703	0.92760139109045	0.944288747461576	1081
LOC10024	0.0027225973054549	0.928754731837657	0.945367291843242	1081
ATAD3C	0.00272063787079079	0.928805870414234	0.945371577379483	1081
NCRNA00	0.0026831439043716	0.929784468199971	0.946080821895566	1081
SNORA2B	0.00267932305797471	0.929884198805277	0.946134511082999	1081
OR13C4	0.00267189762177207	0.930078018493729	0.946236133657702	1081
C20orf186	0.0026534050276943	0.930560732532996	0.946655864810915	1081
OR1A1	0.00265067781689122	0.930631923353068	0.946656235909288	1081
AQP12B	0.00259197372431121	0.932164462161553	0.94788012166193	1081
FOXB2	0.0025888045704363	0.932247203816269	0.947916410300764	1081
RRM1	0.00257994681624813	0.93247846980931	0.948103708388863	1081
C3orf66	0.00257096507880464	0.932712978677648	0.948246427224892	1081
OR7G2	0.0025286831945628	0.933817016466712	0.94917724389333	1081
RAB11FIP	0.00252507490582238	0.933911239735269	0.949225122324268	1081
LARP6	0.00252090916464745	0.934020020912881	0.949239901185046	1081

AQP11	0.00249927865506893	0.934584884262781	0.949766054253833	1081
FLJ33360	0.00249296488094132	0.934749769606705	0.94988569959277	1081
TFPI2	0.00249086265710627	0.93480467017988	0.94989357280069	1081
NOX1	0.00247921557930641	0.935108844746911	0.950154730022522	1081
PVALB	0.00242089969582698	0.936631961141446	0.951523166683432	1081
DNAH14	0.00240567450966921	0.937029656688576	0.951818396227882	1081
PHOSPHC	0.00240195855831591	0.937126723063426	0.951868997938421	1081
PRHOXNI	0.00238654183801311	0.937529441501615	0.952182031714144	1081
C1orf180	0.0023843166507461	0.93758756957804	0.952193062570559	1081
ESM1	0.00237419285632713	0.937852035416429	0.952413633788575	1081
RLBP1	0.0023443351948885	0.938632052637833	0.953061628043808	1081
SRPK1	0.00232737180723663	0.939075239325114	0.953382352280232	1081
C13orf36	0.00232550648030619	0.939123974143326	0.953382352280232	1081
FOXN2	0.00232499930284932	0.939137225050828	0.953382352280232	1081
LOC64259	0.00230746753671002	0.93959528374871	0.953736678218666	1081
CETN1	0.00228098613479864	0.940287210559279	0.95434688564158	1081
RIMS2	0.00227190695025244	0.94052444899292	0.954453933049336	1081
ZIC3	0.00226787367030026	0.940629839997453	0.954512801065318	1081
PARN	0.00225039913272791	0.941086467697384	0.954783787860788	1081
NEUROD(	0.00223558050446761	0.941473708906274	0.955080464594465	1081
C9orf27	0.00222520636438227	0.941744814733287	0.955259279597284	1081
HPYR1	0.0021876893427925	0.942725297396507	0.956061272455327	1081
COX8C	0.00216621160351316	0.943286643082153	0.95653247569781	1081
OR10A5	0.00213129624609935	0.944199256448676	0.957315115061582	1081
NKX6-2	0.00212627500861458	0.944330506987381	0.957361015450114	1081
IL4R	0.00210156194523918	0.944976505704592	0.957813922385862	1081
HEBP2	0.00209455844209983	0.945159583651066	0.957951288621993	1081
PDXDC2	0.00205303097818441	0.946245208453649	0.958949470418569	1081
ANKRD5	0.00201663570384301	0.947196747884144	0.959678040845248	1081
OR4K1	0.00200847769348564	0.947410046066745	0.959845874975587	1081
PYGM	0.00197445516890681	0.948299634581655	0.960669768920469	1081
SLC12A8	0.00194734100137974	0.949008636106773	0.961272072323559	1081
ANKS3	0.00194514657448727	0.949066019393001	0.961281862369752	1081

SPINK9	0.00192963320868866	0.949471694509108	0.961596062836759	1081
EEPD1	0.00192111973488563	0.949694327743618	0.961687934576897	1081
NFATC3	0.00191492131588698	0.949856423160133	0.961792291759783	1081
RPS16	0.00190551194663579	0.9501024923856	0.961944750922647	1081
LEUTX	0.00190121955485798	0.950214746496955	0.962010054687966	1081
LOC64575	0.00188492722175868	0.95064083058515	0.96239306156781	1081
CXCR7	0.00187886085185277	0.950799484501836	0.96250530730098	1081
IGLL3	0.00187191141614548	0.950981235652551	0.962640922175802	1081
EVPLL	0.00186975183125484	0.951037716590375	0.962649723681696	1081
DEPDC7	0.00182494831086771	0.952209545311817	0.963610239507865	1081
C1orf189	0.00180670072360186	0.952686837678221	0.96402834765058	1081
PRRT4	0.00179677235090873	0.95294653593481	0.964194272546798	1081
TMED6	0.00178314692185107	0.9533029468638	0.964458008488656	1081
FAM117A	0.00177956647335086	0.953396605014475	0.964504324211148	1081
PTX4	0.00176867650656782	0.953681471010466	0.964744061138186	1081
SOX30	0.00176662995144257	0.953735006633753	0.964749771962689	1081
INCA1	0.00174377205521282	0.954332958081956	0.965257691447558	1081
MOSPD1	0.00173224677186264	0.954634463644474	0.965465699589233	1081
RTN1	0.00172778896485653	0.95475108322201	0.965535169252379	1081
SH3GL3	0.00171767499979731	0.955015675723368	0.965632521464088	1081
C21orf91	0.00171642272563771	0.955048436944111	0.965632521464088	1081
WDR13	0.00171494797233559	0.955087018623577	0.965632521464088	1081
ASMT	0.00170351928986706	0.955386013043475	0.965886341100111	1081
MFSD2A	0.00169708156176	0.955554438088521	0.966008137466415	1081
CCNYL1	0.00167661710827388	0.95608984606936	0.966403911142361	1081
TATDN3	0.00166125157148051	0.956491864986813	0.966713256078635	1081
FYTTD1	0.00164562479149156	0.956900730321613	0.967029457827275	1081
PTPRN	0.00163208478920639	0.957255005528813	0.967308066230298	1081
PYDC1	0.00163141789373919	0.95727245511875	0.967308066230298	1081
F9	0.001614736111123	0.957708947346512	0.96760834447173	1081
KCNJ15	0.00161455392012498	0.957713714590312	0.96760834447173	1081
STRBP	0.00158390363135657	0.958515737154742	0.96837008650864	1081
GPR32	0.00157870666872635	0.958651729655566	0.968442909280968	1081

DTX3	0.00157747475794348	0.958683966089901	0.968442909280968	1081
LOC10013	0.00156388315788921	0.959039633262816	0.968705044729214	1081
GDF2	0.00156201121461065	0.959088619214659	0.968705953074922	1081
LOC28391	0.00155526029266319	0.959265282010668	0.968835811997639	1081
FOXA2	0.00155074088884711	0.959383550013243	0.968858111841627	1081
CALCR	0.0015419133152212	0.959614560820967	0.969042825592179	1081
ZFAND6	0.00153124504804505	0.959893745679793	0.969276165750347	1081
NTF4	0.00152873957525675	0.95995931374882	0.96929378855546	1081
TBC1D17	0.00152066012949957	0.960170754188396	0.969375454685736	1081
UBE2E2	0.00152013285566546	0.960184553129768	0.969375454685736	1081
CPT1A	0.00146537722141135	0.961617591382401	0.970579005881982	1081
LOC10013	0.00146068208358507	0.961740476112781	0.970605775827462	1081
CATSPER	0.00145129196024739	0.96198624430701	0.97080451305836	1081
NPY	0.00144763455872576	0.962081970647659	0.97080451305836	1081
MPPED1	0.00143068220389504	0.962525677163488	0.971203594613496	1081
TRIM42	0.00142811109405437	0.962592973737778	0.971222851645174	1081
IGFBP1	0.00141893163039305	0.962833240420373	0.971319326369293	1081
GSTM4	0.00141286830490848	0.96299194599767	0.971430783781826	1081
SNORA5E	0.00140475045461338	0.963204430386759	0.971547829468319	1081
PLEKHA3	0.00138882014884518	0.963621413039755	0.971871100715223	1081
CCDC115	0.00136851960981846	0.964152802824504	0.972309682263698	1081
LOC34892	0.00134798139017355	0.964690430350086	0.972754465882595	1081
ODF3	0.0013308176668154	0.965139736231052	0.973158816075607	1081
SNORA14	0.00132407075111843	0.965316357638818	0.973179565772176	1081
MYL2	0.00131957105064792	0.965434152175451	0.973179565772176	1081
RANBP10	0.00131827624911887	0.965468048026006	0.973179565772176	1081
PRKACG	0.00131780439408759	0.965480400457882	0.973179565772176	1081
PRSS42	0.00131711391571375	0.965498476124235	0.973179565772176	1081
CCNL2	0.00130842274984466	0.965725999046953	0.973360196667878	1081
PI4KAP1	0.00128821478239323	0.966255026939419	0.973844682694346	1081
PET112L	0.0012802446560804	0.96646368235305	0.974006247486227	1081
FAM69B	0.00126495505196025	0.966863965812461	0.974360910019611	1081
COL18A1	0.00124710532253779	0.967331284166068	0.974783088366037	1081

FAM19A4	0.00124238428299407	0.967454886150938	0.974858877626583	1081
FAM40B	0.00123958164062631	0.9675282627666	0.974884052089889	1081
MBOAT1	0.00122831749792375	0.967823174605015	0.975113545468966	1081
MAPK7	0.00119669104708678	0.968651225008373	0.975820319437599	1081
C16orf58	0.0011927772129708	0.968753700203369	0.975874752005044	1081
DMRTC2	0.00118838336809322	0.968868744045192	0.975941839531496	1081
LOC28483	0.00115830792171093	0.96965622534373	0.976686232977472	1081
OR8J1	0.00115067880225054	0.969855987336598	0.976838601314723	1081
SNORA6	0.00112718647885854	0.970471125616375	0.977360437211512	1081
LGALS13	0.00108785697466655	0.971500994028485	0.978202005533254	1081
TAF1D	0.00106008464466118	0.97222825907553	0.978836437556927	1081
FGFR3	0.00105212633361709	0.972436665076955	0.97889949120404	1081
UNC45B	0.00104362733045533	0.972659232373945	0.979048311927815	1081
KLHL1	0.00104073124297895	0.972735074032599	0.979053113484222	1081
GOSR2	0.00102642292178172	0.9731097786111	0.979332391972853	1081
MAB21L2	0.0009853417587365	0.974185639772813	0.980317184336046	1081
VIPAR	0.000951561463973879	0.975070336107557	0.981109431141638	1081
OR2T12	0.000927941656464265	0.97568895042379	0.981682844185758	1081
FAM43A	0.000911874509401438	0.976109765235198	0.982018193099991	1081
AGR2	0.000867174217689954	0.977280545597476	0.983011080971675	1081
C11orf16	0.000866133231304928	0.977307811479654	0.983011080971675	1081
SPAG8	0.000864476348331223	0.977351209196868	0.983011080971675	1081
KAT2B	0.000862030197389754	0.977415279847138	0.983011080971675	1081
KRTAP4-9	0.000835189663529814	0.978118308261115	0.98353132218469	1081
LALBA	0.000827492899823812	0.978319911116801	0.983642174558193	1081
LOC40138	0.000810464617888078	0.978765941172777	0.983992408669807	1081
TBX10	0.000780661420985146	0.97954660665069	0.984595559317826	1081
OR2M1P	0.000778872042525226	0.979593478292333	0.984595559317826	1081
PLK5P	0.000778231178206843	0.979610265346597	0.984595559317826	1081
SNORA62	0.000770252814609463	0.979819254521164	0.984707352573153	1081
NCRNA00	0.000766306407593173	0.979922629156994	0.98476211535307	1081
CYB5R2	0.000751597531268241	0.98030792554883	0.985051035523802	1081
GOLGA5	0.000749412678722325	0.980365157765073	0.985057346262055	1081

TLE3	0.0007395758419715	0.980622834966739	0.985121777809054	1081
MSL1	0.000717726792572978	0.981195180804443	0.985549307803425	1081
GRASP	0.000705282309529562	0.981521174066651	0.985778445087879	1081
KRT6A	0.00070030508358201	0.981651557377192	0.985860243449999	1081
NFATC4	0.000686549131347064	0.982011910559594	0.9861238192624	1081
LOC1501	0.000684575642626504	0.982063608696371	0.986126577469265	1081
TMEM44	0.000678901995847092	0.982212237766071	0.986214738253146	1081
AQP12A	0.000673443180152409	0.982355239618731	0.986271931329147	1081
GNA13	0.000658276026382283	0.982752568884963	0.986621673486873	1081
ARMS2	0.000624660336761065	0.983633203861493	0.987456565096416	1081
FLT3	0.000585536080390043	0.984658172545622	0.988407553704023	1081
NEUROD	0.000582882008031151	0.984727704285914	0.988407553704023	1081
MAN2C1	0.000557587560031165	0.985390377333374	0.988929417772847	1081
GSTM2P1	0.000552915025259664	0.985512791195136	0.988929417772847	1081
PNPLA4	0.000550785847696338	0.985568572776078	0.988929417772847	1081
ZNF503	0.000505437204060224	0.986756662379019	0.989901546916256	1081
C19orf75	0.000500688391156352	0.986881078321297	0.989927765910598	1081
WDR59	0.00049253917641467	0.987094583416007	0.990092630099356	1081
PRKAR1E	0.000468215468459074	0.987731857064418	0.990583873179803	1081
ZYX	0.000447672626718081	0.988270079288733	0.9910249762194	1081
ERGIC1	0.000434430205122845	0.988617033427895	0.991323550941013	1081
ATAD5	0.000391183338933615	0.989750124747153	0.992262180901333	1081
KSR1	0.000373775371256676	0.990206228885437	0.992620644949101	1081
MYOT	0.000361137720914076	0.990537348504528	0.992888529199148	1081
THPO	0.000289404842408937	0.992416853889516	0.994490251286091	1081
RPS6KB1	0.000222026644266015	0.99418229880327	0.99606119308658	1081
CREB3L1	0.000211508219706721	0.994457905562766	0.996238227040724	1081
RALB	0.000189293451611698	0.995039985380758	0.996573556005537	1081
STK17B	0.000185688358331452	0.995134447665287	0.996618615372468	1081
F2RL3	0.000143206217596936	0.996247588701044	0.997485470893764	1081
EPB49	0.000135005701388592	0.996462464163362	0.997601448194559	1081
SNORA47	0.000128520284898502	0.996632399607112	0.997721994239689	1081
CAPN9	0.000102401249209979	0.997316790414136	0.99829373374338	1081

IFI6	1	1e-302	1e-299	1081
UBE2C	0.263890323	1.11276276051872e-18	4.34430345167627e-17	1081
PSMD4	0.179639417	2.71602112298733e-09	1.86674327951484e-08	1081
INSL3	0.176485385	5.16443493083525e-09	3.36038571323243e-08	1081
RAG1AP1	0.166599026	3.59348173844137e-08	2.00639383649948e-07	1081
LMO1	0.148605679	9.22157349205823e-07	3.97281005127273e-06	1081
MTMR14	0.14000161	3.8281639398128e-06	1.47991484489597e-05	1081
LAS1L	0.106655936	0.00044364367095918	0.00116052483462832	1081
C7orf13	0.032966354	0.278840944725206	0.358883901832947	1081
SCNN1A	-1.35E-05	0.999647035577193	0.999696660628602	1081
VHLL	-2.59E-05	0.99932098840893	0.999519453428226	1081
NCRNA0(	-2.71E-05	0.999290459876429	0.999519453428226	1081
TBL1Y	-5.67E-05	0.998514225328777	0.999010135050818	1081
C10orf27	-6.68E-05	0.99824924520238	0.998822497608065	1081
CNNM1	-8.28E-05	0.997830052886955	0.998523988644762	1081
BRD9	-8.60E-05	0.997747552112833	0.998491030169548	1081
NCRNA0(	-9.55E-05	0.997497640639549	0.998340121754954	1081
CT45A3	-9.92E-05	0.997401736364011	0.99829373374338	1081
BSX	-9.98E-05	0.997386122282799	0.99829373374338	1081
FAM24A	-0.000103456	0.997289147291356	0.99829373374338	1081
CALB2	-0.000137563	0.996395443753961	0.997583927957037	1081
RGS13	-0.000156479	0.995899814314972	0.997186826351961	1081
SLC30A8	-0.000169794	0.995550922484495	0.996887033176765	1081
CROCCL1	-0.000174569	0.9954258000214	0.996811291019094	1081
SLC2A14	-0.000180551	0.995269047220907	0.996703865393973	1081
DIS3L2	-0.000190058	0.995019947812606	0.996573556005537	1081
CCDC102	-0.000192207	0.994963631629303	0.996573556005537	1081
TMEM81	-0.000205433	0.994617084173638	0.99629860079946	1081
SPATA19	-0.000208119	0.994546717145457	0.996277653749141	1081
LOC10013	-0.000213466	0.994406616685775	0.996236388160679	1081
TTLL2	-0.000233686	0.993876802059834	0.995804644260188	1081
ANO10	-0.00027322	0.992840928851505	0.994816240323978	1081
EFEMP1	-0.000281496	0.992624069298909	0.994648422006891	1081

HES3	-0.000309834	0.991881570824146	0.99400329540605	1081
C6orf174	-0.000310176	0.991872620868613	0.99400329540605	1081
FICD	-0.000310862	0.991854640630722	0.99400329540605	1081
C6orf211	-0.000336435	0.991184593679465	0.99345308919214	1081
CELP	-0.000359814	0.990572035733159	0.992888529199148	1081
SUMF2	-0.000374725	0.990181339029372	0.992620644949101	1081
MLC1	-0.000398394	0.989561209464463	0.992122160188206	1081
ACTA1	-0.000408834	0.989287671130436	0.991897279261528	1081
ERLEC1	-0.000429027	0.988758590096557	0.991416146408598	1081
NBPF6	-0.000464182	0.987837528911524	0.990640532652461	1081
C3orf31	-0.000477371	0.98749199167561	0.990392620347763	1081
OBFC1	-0.000481444	0.987385281586147	0.990334901546076	1081
C20orf191	-0.000501746	0.986853366146647	0.989927765910598	1081
C7orf69	-0.000517386	0.986443600197905	0.98963676922245	1081
SLC29A1	-0.000522895	0.986299279893863	0.98954126168942	1081
KRTAP3-[illegible]	-0.000548046	0.985640349964915	0.988929417772847	1081
EIF3H	-0.000549838	0.985593398104624	0.988929417772847	1081
COL2A1	-0.000553081	0.985508454226664	0.988929417772847	1081
QPCTL	-0.000554438	0.985472879615462	0.988929417772847	1081
FUT6	-0.00057027	0.985058106599544	0.988689928625769	1081
PINX1	-0.000584737	0.984679097581466	0.988407553704023	1081
PDK1	-0.000677487	0.982249317861063	0.986214738253146	1081
ACOX2	-0.000688967	0.981948577423201	0.986109376480079	1081
ASCL3	-0.000711454	0.981359509711781	0.985665220279396	1081
HES1	-0.000723894	0.981033617083684	0.985436161363791	1081
TMEM9B	-0.000736196	0.980711361082472	0.985161582178438	1081
LOC2842[illegible]	-0.000742415	0.980548463100244	0.985096189365371	1081
KCNJ5	-0.000745758	0.980460900963041	0.985057346262055	1081
PSD	-0.00074719	0.98042338838775	0.985057346262055	1081
LYNX1	-0.000754438	0.980233522343066	0.985025405676713	1081
RGS7	-0.000773928	0.979722986526578	0.98465972677998	1081
STX17	-0.000779272	0.979582995547812	0.984595559317826	1081
CMIP	-0.000793823	0.979201844076113	0.984381513494351	1081

PCDP1	-0.00081049	0.978765265991514	0.983992408669807	1081
SLC13A4	-0.000833566	0.978160835937963	0.98353132218469	1081
KLHDC9	-0.000849011	0.977756286660356	0.983222702279881	1081
IFNK	-0.000855309	0.977591333147518	0.983105900871443	1081
ADAMTS	-0.000860771	0.977448248346668	0.983011080971675	1081
ANGPTL4	-0.000885983	0.976787893820041	0.98259223614325	1081
OR7E5P	-0.00090728	0.976230096351003	0.982080164344118	1081
MT1B	-0.000911493	0.976119746767646	0.982018193099991	1081
LITAF	-0.000960227	0.974843374252946	0.980930058657622	1081
EIF4E	-0.001024183	0.973168428054558	0.97934249091613	1081
MTMR9L	-0.001038687	0.972788612882574	0.979058083858886	1081
HDAC9	-0.001042769	0.972681703396539	0.979048311927815	1081
SET	-0.00105472	0.972368748618619	0.97888003802519	1081
EMILIN1	-0.001055933	0.97233697101859	0.97888003802519	1081
C6orf164	-0.001071007	0.971942241640592	0.978597383938911	1081
FASTKD3	-0.001094461	0.971328052115546	0.978076757466144	1081
SNORA66	-0.001117195	0.970732763637249	0.977526194624963	1081
LOC80054	-0.001118115	0.970708658702198	0.977526194624963	1081
JPH4	-0.001136195	0.970235230845029	0.977171719096746	1081
LOC91149	-0.001224504	0.967923013034491	0.975135481975386	1081
HOXC4	-0.001227185	0.967852834035839	0.975113545468966	1081
AIF1L	-0.001326873	0.965242992614851	0.973165511547278	1081
THEG	-0.001327063	0.965238029608498	0.973165511547278	1081
EXD3	-0.001359853	0.964379653056211	0.97248976877496	1081
FAM167A	-0.001385571	0.963706456672739	0.971908213750805	1081
KLK12	-0.001390677	0.963572811196556	0.971870739576159	1081
CLCN5	-0.001405592	0.963182412955691	0.971547829468319	1081
CER1	-0.001421312	0.962770939116549	0.971305116611723	1081
OR13D1	-0.001421335	0.962770322932643	0.971305116611723	1081
BAG1	-0.001449026	0.962045548297877	0.97080451305836	1081
SERPINB1	-0.001462617	0.961689843535052	0.970603301503688	1081
GIF	-0.001467073	0.961573213456782	0.970579005881982	1081
E2F5	-0.001477907	0.961289652699747	0.970345244958481	1081

LYPD3	-0.001496396	0.960805774353564	0.969905408115482	1081
C6orf105	-0.001513429	0.960360002186691	0.969503996193981	1081
RNASE8	-0.001524667	0.960065891509598	0.969352815981398	1081
MUC15	-0.00155121	0.959371276347892	0.968858111841627	1081
BMP10	-0.001575599	0.95873305621733	0.96844393609277	1081
SETD3	-0.0016171	0.957647094014556	0.96760834447173	1081
PROX2	-0.001646464	0.956878776374621	0.967029457827275	1081
TTC27	-0.001668383	0.956305268868984	0.966573159468451	1081
OR2M7	-0.001677829	0.956058145767594	0.966403911142361	1081
NETO2	-0.001690399	0.955729280845576	0.966136409204844	1081
OR5M10	-0.001720263	0.954947964429478	0.965632521464088	1081
PRKAG3	-0.001724782	0.954829754684972	0.96556625712207	1081
SP5	-0.001739438	0.954446344186118	0.965323908205109	1081
CCDC120	-0.001758461	0.953948703741626	0.964917485281937	1081
USP13	-0.001794642	0.953002252616447	0.964202218821672	1081
LIPN	-0.001803554	0.952769156000284	0.964063220032434	1081
FBXO42	-0.001824326	0.952225814737308	0.963610239507865	1081
WISP2	-0.001848992	0.951580679959131	0.9630541470875	1081
CYP1A1	-0.001853138	0.951472239723948	0.962992778800187	1081
MAN2B2	-0.001855549	0.951409179316287	0.962977335945666	1081
DYTN	-0.001907674	0.950045943810422	0.961935843288146	1081
TARDBP	-0.001920688	0.949705622758639	0.961687934576897	1081
LOC49414	-0.001922511	0.949657948308531	0.961687934576897	1081
OR2D3	-0.001942932	0.949123939082085	0.961292194711342	1081
STAT5A	-0.001971905	0.948366328841964	0.960669768920469	1081
PRR23C	-0.001972935	0.948339388529569	0.960669768920469	1081
OR4C12	-0.002018304	0.94715312427061	0.959678040845248	1081
KRT81	-0.002029467	0.946861264963634	0.959434645274001	1081
SLCO1B1	-0.002043141	0.946503779265685	0.959120655598955	1081
KRTAP5-[illegible]	-0.002051423	0.94628724360639	0.958949470418569	1081
LIG3	-0.002091459	0.945240602805649	0.957985206194084	1081
RNF207	-0.002107701	0.944816026541584	0.957699449264376	1081
HSPC072	-0.002117045	0.944571771975872	0.957500042593164	1081

SLC6A3	-0.00212411	0.944387098487201	0.957361015450114	1081
NPBWR2	-0.002125612	0.944347825452594	0.957361015450114	1081
FRG2B	-0.002143743	0.943873913500916	0.957033419945438	1081
ABCC6P2	-0.002164462	0.943332375015557	0.95653247569781	1081
C1orf126	-0.002172063	0.943133704948307	0.956427308642519	1081
C4orf6	-0.002197375	0.942472152508111	0.955852666378487	1081
HSP90AB2	-0.002206504	0.942233581775035	0.955658821108553	1081
ALKBH5	-0.002215035	0.942010637225563	0.955480805946779	1081
REG3A	-0.00222833	0.941663178126875	0.955224569382441	1081
NLK	-0.002243505	0.941266628658271	0.954918478839748	1081
SLC2A2	-0.002253817	0.940997146853487	0.95474125023236	1081
BIRC8	-0.002255208	0.940960810925152	0.95474125023236	1081
LAMB4	-0.002265233	0.940698842188215	0.95453473912666	1081
LASS4	-0.002277725	0.940372433369993	0.954347741573728	1081
PHLDA1	-0.00227957	0.940324216088345	0.95434688564158	1081
APCDD1	-0.002306204	0.939628287545588	0.953736678218666	1081
OR1G1	-0.002307159	0.939603337183565	0.953736678218666	1081
OR5D16	-0.002332736	0.938935091669116	0.95332127522173	1081
KCNV1	-0.002349205	0.938504825380981	0.952980478214621	1081
BCKDHA	-0.002368294	0.938006144712616	0.952522118420992	1081
KIAA1984	-0.002390915	0.937415213475364	0.952114020140224	1081
OR52N1	-0.002408787	0.936948359833843	0.951783808625524	1081
KIAA0664	-0.002420418	0.936644546802306	0.951523166683432	1081
OR1D2	-0.002447571	0.935935313039301	0.95089857177611	1081
DSPP	-0.002461017	0.935584137997964	0.950589724112018	1081
TTLL8	-0.002522309	0.933983475582336	0.949239901185046	1081
ZNF239	-0.002529088	0.933806437850659	0.94917724389333	1081
C7orf66	-0.002547498	0.933325727666854	0.948773617795265	1081
THUMPD2	-0.002563171	0.932916494468962	0.948405469372085	1081
INTS5	-0.002577584	0.932540164524703	0.948118583544471	1081
SPATA16	-0.002603916	0.931852683059502	0.947610918739711	1081
DAZ1	-0.002608092	0.931743660030497	0.947547883856548	1081
SPATA5L	-0.002608291	0.931738449841028	0.947547883856548	1081

DPYSL4	-0.002617615	0.931495038199357	0.94739069745676	1081
CCDC113	-0.002633824	0.931071895848129	0.947008146110297	1081
CACNA1E	-0.002644502	0.930793132061004	0.946772413298102	1081
OR8D4	-0.002652492	0.930584566435867	0.946655864810915	1081
OR4C46	-0.002676395	0.929960634300468	0.946164493837521	1081
C13orf37	-0.002683347	0.929779155388579	0.946080821895566	1081
ST3GAL1	-0.002695103	0.929472322600254	0.945858756252885	1081
C21orf54	-0.002696824	0.929427399314796	0.945858756252885	1081
ATXN7L3	-0.002697868	0.929400163933265	0.945858756252885	1081
C13orf28	-0.002708412	0.929124962358547	0.945648581150555	1081
LCE5A	-0.002750735	0.928020404581111	0.944667561914426	1081
ATP2B3	-0.002769556	0.927529265100734	0.944263040502035	1081
RFX2	-0.002782442	0.927193003105193	0.944142190220507	1081
NCRNA0(	-0.002805074	0.926602478628649	0.943722104212003	1081
HOXC11	-0.002811734	0.926428704493221	0.943667201902004	1081
METTL6	-0.002812499	0.926408737305105	0.943667201902004	1081
H2BFWT	-0.002825367	0.926072998080847	0.943400280472246	1081
TWIST1	-0.002858159	0.925217490838703	0.942719455411748	1081
SST	-0.002866448	0.925001232339505	0.942594457255264	1081
SNORA37	-0.002875511	0.924764811394543	0.942422232108924	1081
RPL9	-0.002877773	0.924705810877631	0.942422232108924	1081
OR51B5	-0.002899475	0.924139705314128	0.941980740719769	1081
OPN4	-0.002901975	0.924074488901735	0.941980740719769	1081
RAB3B	-0.002923271	0.92351901920266	0.941465039311653	1081
ST18	-0.002942941	0.92300598002076	0.940989649165902	1081
SERPINB[illegible]	-0.002955755	0.922671791710987	0.940744166616957	1081
GPT	-0.003004975	0.921388244776749	0.939673274491349	1081
AADAT	-0.003015574	0.921111866934354	0.939444000882445	1081
C8A	-0.00302122	0.920964632027376	0.939383924667924	1081
MSH2	-0.00302178	0.920950053875172	0.939383924667924	1081
SLC9A1	-0.00304465	0.920353749546422	0.938950991826834	1081
C11orf34	-0.003058569	0.91999085725288	0.938628301816119	1081
PRMT8	-0.003079069	0.919456411583604	0.938178058620863	1081

CRYM	-0.003097838	0.918967122152269	0.937773804556885	1081
SLC24A6	-0.003106552	0.918739981813383	0.937589510315633	1081
TMPRSS1	-0.003119088	0.918413187117664	0.937350980569731	1081
KCTD2	-0.00311959	0.918400122722258	0.937350980569731	1081
ASF1A	-0.003151107	0.91757863807918	0.936689047537503	1081
OR4F4	-0.003170306	0.917078261294071	0.936273138747672	1081
GPR151	-0.003175776	0.916935696822178	0.936175034842774	1081
LINGO1	-0.003191691	0.916520950566382	0.935846446812295	1081
DPH3	-0.003201818	0.916257053236299	0.935671837453503	1081
C12orf12	-0.003210829	0.916022239876223	0.935542881538595	1081
TRAF5	-0.003215015	0.915913163327649	0.935510352138899	1081
ZFP42	-0.003216068	0.915885737755695	0.935510352138899	1081
NNT	-0.003247449	0.915068062486668	0.935026432604307	1081
C14orf143	-0.003249874	0.915004883449717	0.93500930187149	1081
GPR15	-0.0033165	0.913269204298255	0.933567164996618	1081
L1TD1	-0.003354683	0.912274662670514	0.932720458613011	1081
KCNA7	-0.003360981	0.912110631977467	0.932666802760575	1081
MTA3	-0.003372457	0.911811765913088	0.932503199528843	1081
OR8D1	-0.003399396	0.911110220456531	0.931974986853702	1081
HSD17B6	-0.003399711	0.911102023512699	0.931974986853702	1081
LEFTY1	-0.003402705	0.911024047331199	0.931974986853702	1081
SPESP1	-0.003413115	0.910752980015117	0.931846146701434	1081
ANXA8L1	-0.003419621	0.910583567843523	0.931767459450793	1081
C7orf57	-0.003442296	0.909993169518806	0.931350722516039	1081
PSMB11	-0.003459242	0.909551985214356	0.930995617201525	1081
C2orf53	-0.003490229	0.908745301464225	0.930311723650616	1081
CNTLN	-0.00349972	0.908498228353032	0.930185287108922	1081
PHF20L1	-0.003520892	0.907947119972424	0.929683578928763	1081
DPEP1	-0.003536161	0.907549699994598	0.92932388077015	1081
GTF2A1L	-0.003552238	0.907131288052131	0.928989873306399	1081
ARHGAP[illegible]	-0.003561154	0.906899249414115	0.928846681552054	1081
OLA1	-0.003591531	0.906108736098068	0.928178607174595	1081
CXorf51	-0.003593219	0.906064820930562	0.928178607174595	1081

KRTAP15	-0.0035972	0.905961223079992	0.928162856766642	1081
ASB6	-0.00360998	0.905628697930567	0.927875603692975	1081
MT1L	-0.003610117	0.905625118112514	0.927875603692975	1081
IFITM5	-0.003613198	0.905544970577142	0.927875603692975	1081
PPFIA1	-0.003620356	0.90535872609186	0.927740553289614	1081
PYROXD2	-0.003628613	0.905143886663978	0.927614773202718	1081
LOC10012	-0.003644375	0.904733796416923	0.927288849087709	1081
SPINK14	-0.003645068	0.904715771551853	0.927288849087709	1081
TAS2R20	-0.003650721	0.904568708534092	0.927213994475107	1081
RPL18	-0.003661315	0.90429309790228	0.926978651396368	1081
FOXA3	-0.003670453	0.904055377794057	0.926829291891159	1081
CT45A1	-0.003683384	0.903719017476181	0.92653161011032	1081
MAP2K7	-0.00369317	0.903464469338377	0.926317779663152	1081
ZNF746	-0.003703028	0.903208049163944	0.926115876071128	1081
NCKAP5L	-0.003710077	0.90302471566588	0.926102575833078	1081
INS	-0.003724259	0.902655859502762	0.925771422955052	1081
FLJ13224	-0.003732534	0.902440631979927	0.925678279653002	1081
TUSC1	-0.003751348	0.901951371521769	0.925284431395123	1081
SNORD22	-0.00375221	0.901928947046432	0.925284431395123	1081
CDKL2	-0.003784674	0.901084784411118	0.924680790618269	1081
KRT27	-0.003824424	0.900051279372875	0.923996827834669	1081
CYMP	-0.003826428	0.899999198089366	0.923996827834669	1081
MAP3K12	-0.003826767	0.899990379305585	0.923996827834669	1081
AKR7L	-0.003837304	0.899716443519157	0.923838511376391	1081
GANAB	-0.003839003	0.899672295472169	0.923838511376391	1081
GDNF	-0.00384824	0.899432165907351	0.923640769852861	1081
BRCA2	-0.003900175	0.898082272227458	0.922442633662476	1081
OR52E4	-0.003901505	0.898047697801422	0.922442633662476	1081
ZNF512B	-0.00390963	0.897836559758546	0.922284304540101	1081
RHBDL1	-0.003920772	0.897546984621135	0.922033860540171	1081
C6orf182	-0.003928539	0.897345169955655	0.921920565522066	1081
ESX1	-0.0039328	0.897234428381473	0.921870700886597	1081
tAKR	-0.003978244	0.896053654285848	0.920875465033589	1081

ABCA2	-0.003991005	0.89572211979198	0.920581710280569	1081
FRMD1	-0.003993224	0.895664479687545	0.920569435882939	1081
IRGC	-0.0039944	0.895633929112367	0.920569435882939	1081
CCBP2	-0.004005984	0.895333007814219	0.920322657537373	1081
SMG7	-0.004006428	0.895321462431569	0.920322657537373	1081
KCNJ6	-0.00401185	0.89518061134234	0.920306885199869	1081
SPDYA	-0.004025817	0.894817818417819	0.920074772969935	1081
ZIC5	-0.004052044	0.894136599424284	0.919515125601215	1081
EFCAB5	-0.004053036	0.894110829489857	0.919515125601215	1081
AFARP1	-0.004084172	0.893302218099977	0.918797773084042	1081
C4orf47	-0.0040956	0.893005449292772	0.918648113970895	1081
MGC1588	-0.004096419	0.892984192731051	0.918648113970895	1081
KCTD11	-0.004104609	0.892771529366672	0.918618939456086	1081
TMEM225	-0.004114286	0.892520240113289	0.918415499672177	1081
SNORD15	-0.00413825	0.891898058625808	0.917918333436695	1081
AKR1C2	-0.004147138	0.891667302113595	0.917819109962617	1081
DDX26B	-0.004167346	0.891142698156693	0.917408983978856	1081
HCRTR1	-0.004192618	0.89048669764441	0.916931840321337	1081
ALDH3A2	-0.004204152	0.890187324127108	0.916717290897689	1081
C8orf86	-0.004204928	0.890167179836579	0.916717290897689	1081
GABBR1	-0.004208895	0.890064231319819	0.916684250508065	1081
SCN10A	-0.00431276	0.887369139766147	0.914282493892646	1081
SH2D3A	-0.00432426	0.887070809028606	0.914021863223429	1081
NANOG	-0.004330001	0.886921901311506	0.913915176568813	1081
SPAG11B	-0.004401528	0.885066801988274	0.912190255093307	1081
LOC72885	-0.004407215	0.884919338902939	0.91208493641339	1081
TAS1R1	-0.004409572	0.884858219070832	0.912068605361334	1081
SMARCD	-0.004412616	0.884779290447406	0.912033915267001	1081
C22orf26	-0.004429661	0.884337357139612	0.911671667753824	1081
PLVAP	-0.004455666	0.88366315207745	0.911163136540934	1081
ELAC2	-0.004461019	0.883524373235262	0.911066671725243	1081
C10orf140	-0.004466842	0.883373426913136	0.911002850855379	1081
RPL13AP6	-0.004473164	0.883209538566664	0.910881900088334	1081

SEPT7L	-0.004475128	0.883158621567038	0.910876020451975	1081
FAM123A	-0.004478658	0.883067118006447	0.910874915117249	1081
MSI2	-0.004480212	0.88302683040623	0.910874915117249	1081
CUL2	-0.004481376	0.88299665934486	0.910874915117249	1081
TMEM174	-0.00452293	0.881919641700631	0.909970865706782	1081
LOC15222	-0.004573065	0.880620531639169	0.908780401037504	1081
GPR113	-0.004573995	0.880596426945844	0.908780401037504	1081
NFIL3	-0.004576358	0.880535205205779	0.908780401037504	1081
CXorf64	-0.004609107	0.879686759105237	0.908225182563294	1081
ENTPD2	-0.004644562	0.878768385766763	0.907509567399981	1081
OR2G2	-0.004645391	0.878746914504614	0.907509567399981	1081
CELF4	-0.004714164	0.876965992281963	0.905833969877462	1081
CAMK2A	-0.004728414	0.876597071030964	0.905545766674466	1081
TMEM99	-0.004757249	0.875850599114866	0.904899777828592	1081
RPAP3	-0.004771404	0.875484205077054	0.904628093520581	1081
PACS2	-0.004796102	0.874844954783689	0.90415306864957	1081
OR2F2	-0.004844084	0.873603319107114	0.90314784272877	1081
NR2E1	-0.00484566	0.87356255381596	0.90314784272877	1081
SLC5A8	-0.004850501	0.873437298133199	0.903068896063092	1081
NPPA	-0.004864796	0.873067453614087	0.902732836475686	1081
GUCY2D	-0.004869197	0.872953599658098	0.90266144467264	1081
HOXC9	-0.004876006	0.872777459507953	0.902547287704992	1081
C2orf78	-0.004885133	0.872541336044118	0.902374106196866	1081
TRPV3	-0.004896206	0.872254906746289	0.902124196344799	1081
VPS8	-0.004903224	0.872073367978755	0.901982748777122	1081
UPF0639	-0.004914001	0.871794625012748	0.901801871856126	1081
BPGM	-0.004967978	0.87039873938965	0.900666868964686	1081
SNORA25	-0.004972961	0.870269914045404	0.900626087457344	1081
SIX2	-0.004981453	0.870050331870105	0.900491365368027	1081
BAZ1A	-0.004984933	0.869960368400785	0.900444516335294	1081
PBX3	-0.004993779	0.869731654722277	0.900254042975042	1081
GRTP1	-0.005003706	0.869475012734404	0.900080890623565	1081
PILRB	-0.005005373	0.869431917008125	0.900080890623565	1081

SLFN5	-0.005015181	0.869178362823174	0.899912531175044	1081
CLK2P	-0.00502935	0.868812102402184	0.899625793003958	1081
CTPS2	-0.00505963	0.868029478168707	0.898907820783869	1081
CLEC2A	-0.005094736	0.867122286179002	0.898060688657447	1081
GADD45(	-0.005095579	0.867100506698072	0.898060688657447	1081
C21orf125	-0.005097061	0.867062220148288	0.898060688657447	1081
TRIM6-TF	-0.005111241	0.866695837223233	0.897757488732107	1081
C6orf70	-0.005126992	0.866288895896971	0.897382105612408	1081
ZBTB45	-0.005128279	0.86625564307497	0.897382105612408	1081
GPX7	-0.005140738	0.865933792686604	0.897106518574011	1081
TEC	-0.005155158	0.865561285010127	0.89692187009085	1081
RANBP17	-0.005159186	0.865457250993956	0.896889568458935	1081
OR51B4	-0.005159362	0.865452696683458	0.896889568458935	1081
SNORA38	-0.005163761	0.865339061510949	0.896859360710916	1081
TRIM61	-0.005167814	0.865234382361359	0.896843150639032	1081
CAMTA1	-0.005176404	0.865012526571659	0.896705467389804	1081
C21orf88	-0.00517844	0.864959936593443	0.896705467389804	1081
LHFPL4	-0.005191558	0.864621159283339	0.896438149962062	1081
ATCAY	-0.005210335	0.864136257117686	0.896027635352882	1081
SNORA51	-0.005214546	0.864027518079372	0.895961000242392	1081
NCRNA0(	-0.005233855	0.863528932703791	0.895536182719066	1081
ATP6V1E:	-0.005238422	0.863411031082905	0.89546000932687	1081
PCBP3	-0.005254168	0.863004504926228	0.895084474681505	1081
DMXL2	-0.005258844	0.862883800618837	0.895051447580788	1081
C6orf208	-0.005348974	0.86055763759968	0.89296042080177	1081
CEACAM	-0.005352545	0.86046548719407	0.892910793773479	1081
POLG	-0.005363905	0.860172400488209	0.892652637947402	1081
SCAMP4	-0.005392496	0.859434807576189	0.89193314093155	1081
TCEANC	-0.005409783	0.858988900649055	0.891516301060031	1081
MUSTN1	-0.005414903	0.858856835826502	0.891425161405785	1081
ART4	-0.00542282	0.858652640725101	0.891305067625453	1081
OR52M1	-0.005430625	0.858451342916802	0.891187956869826	1081
C6orf146	-0.005435041	0.858337439978104	0.891161558952683	1081

IFRD1	-0.005451925	0.857902041137421	0.890755417931829	1081
TXNDC15	-0.00546764	0.857496827550102	0.890380577856647	1081
SCGB1D1	-0.005468636	0.857471132199038	0.890380577856647	1081
PLEKHA8	-0.005469516	0.857448456139944	0.890380577856647	1081
KLK1	-0.005474915	0.857309250609049	0.890334142136451	1081
LOC14474	-0.005506177	0.856503276273338	0.889624052618015	1081
SRGAP3	-0.005518023	0.856197915143768	0.889352737989646	1081
LRP5L	-0.005547225	0.855445252818253	0.888803071806741	1081
OIT3	-0.005554065	0.855268977203423	0.888708595747818	1081
PQLC3	-0.005567872	0.854913154648784	0.888430513304088	1081
SARM1	-0.005577039	0.854676936695246	0.888276680066333	1081
C4A	-0.005592181	0.854286771687198	0.888045355833411	1081
RFX4	-0.005600909	0.854061895487848	0.887874826685794	1081
SNX5	-0.005652789	0.852725473510401	0.886889083786826	1081
NME6	-0.00565919	0.85256063031618	0.88676341892397	1081
THRSP	-0.005662274	0.852481188571482	0.886726573231399	1081
THYN1	-0.005665934	0.852386931879674	0.88667431285325	1081
PRKAG2	-0.005680898	0.852001560048855	0.886364977648429	1081
PPP3R1	-0.005714558	0.851134869099452	0.885737779626432	1081
SBF1P1	-0.005717329	0.851063528373611	0.88570929271511	1081
DMPK	-0.005732987	0.850660423825456	0.885381257450985	1081
CCDC157	-0.005746274	0.850318371515877	0.885116705119999	1081
C17orf50	-0.005758809	0.84999572806058	0.884872303332148	1081
GOLGA6I	-0.005776931	0.849529302506272	0.884523868047801	1081
SERPINF2	-0.005784567	0.849332786598097	0.88436496542196	1081
CELF2	-0.005848495	0.847687941174142	0.882789163304027	1081
SLC6A20	-0.005870002	0.847134711080274	0.88230424747762	1081
CNKSR3	-0.005883021	0.846799862802552	0.882092312744061	1081
TMEM88I	-0.005888266	0.846664963307344	0.881997398170775	1081
CHRNA6	-0.005888641	0.846655320575347	0.881997398170775	1081
ST3GAL6	-0.005900436	0.846351982839196	0.881808155898403	1081
LMO3	-0.00590649	0.846196301313421	0.88176702733049	1081
OR10H2	-0.005952423	0.845015282459986	0.880734316284997	1081

ADAM29	-0.005955242	0.84494282963909	0.880731473596227	1081
OR13F1	-0.005960201	0.844815329525518	0.880678287224605	1081
NUP62CL	-0.005971866	0.844515478077961	0.880441148159215	1081
PTGS1	-0.005973818	0.844465316072365	0.880434416327388	1081
RQCD1	-0.005989295	0.844067501459297	0.880110756568196	1081
SULT4A1	-0.005997156	0.843865458975623	0.880036738161408	1081
OR6Y1	-0.006011804	0.843489033724709	0.879735261940682	1081
OR10G8	-0.00602825	0.843066432665347	0.879431093933483	1081
CD55	-0.006064781	0.842127866476004	0.878634030979858	1081
LOC64395	-0.006074985	0.841865741106659	0.878406036908564	1081
SLC25A6	-0.006102613	0.841156113850778	0.877711069798193	1081
C3orf18	-0.006125353	0.840572147080233	0.877302129652435	1081
PREX1	-0.006134869	0.840327790015834	0.877119343516527	1081
SOD3	-0.00613813	0.840244065507286	0.877077397774199	1081
OTOL1	-0.006161197	0.839651825561767	0.876595461543418	1081
BCAS1	-0.006176543	0.839257851319327	0.876274977445208	1081
C20orf160	-0.006182139	0.839114219710235	0.876184871259907	1081
HAVCR1	-0.006182803	0.839097161182722	0.876184871259907	1081
ZNF750	-0.00619839	0.838697081646461	0.87598159146847	1081
MOBKL1I	-0.006199013	0.838681108824089	0.87598159146847	1081
LARGE	-0.006212421	0.838336977887489	0.87576739367058	1081
C10orf105	-0.006238574	0.837665836924093	0.875111667522473	1081
WHSC2	-0.006249372	0.837388777408074	0.874867592619316	1081
SEPT14	-0.006257067	0.837191365586115	0.874706709181696	1081
GALNT12	-0.006259636	0.837125436245936	0.874683190517344	1081
PIGC	-0.006304888	0.835964647361569	0.873696903818355	1081
DDX43	-0.006332378	0.835259658001209	0.87300538603478	1081
NOLC1	-0.006343578	0.834972466402009	0.872795783295375	1081
LOC22059	-0.006343872	0.834964928531639	0.872795783295375	1081
PDGFB	-0.006350351	0.834798804093783	0.872750112017711	1081
SDHAP2	-0.006355459	0.834667827904014	0.872658469645337	1081
THOC1	-0.006383314	0.833953712065811	0.872047626761783	1081
DEFB1	-0.00638336	0.833952537863818	0.872047626761783	1081

ACSM2B	-0.0064054	0.833387595706117	0.871546130690948	1081
GPX6	-0.006406029	0.833371465396657	0.871546130690948	1081
PCDHB2	-0.006412941	0.833194335826747	0.871520557037133	1081
LOC64417	-0.006413653	0.833176072093735	0.871520557037133	1081
CPM	-0.006419757	0.833019650293556	0.871432770169999	1081
DKFZP686	-0.006420669	0.832996276483981	0.871432770169999	1081
ACE2	-0.006427042	0.832832943813107	0.871327948746561	1081
HOXC8	-0.00644418	0.832393781837505	0.870913718454168	1081
ZNF517	-0.006467407	0.831798686291616	0.870381494667806	1081
CHAT	-0.006479711	0.831483476125027	0.870187263054635	1081
PABPC1P2	-0.006492477	0.831156450973173	0.86993540317216	1081
ITIH5L	-0.006561161	0.829397548935054	0.868478233263233	1081
C22orf24	-0.006573426	0.829083545685461	0.86821687517979	1081
ARL4D	-0.00657423	0.829062980445424	0.86821687517979	1081
OR3A1	-0.006577926	0.828968362140566	0.868186517042979	1081
PON3	-0.00659138	0.828623958180056	0.867925324829475	1081
SNX32	-0.006593552	0.828568346260912	0.867925324829475	1081
ZNF200	-0.006621885	0.827843210658223	0.867278666530235	1081
HOXC12	-0.006634944	0.827509005518818	0.866973627843592	1081
HAPLN4	-0.006656851	0.826948493996198	0.866431445964187	1081
DIAPH1	-0.006674787	0.826489607364627	0.866085785495236	1081
CTSK	-0.006683584	0.826264586449434	0.865940073562785	1081
C15orf58	-0.006699866	0.825848101550973	0.865638691177707	1081
CXorf1	-0.006709849	0.825592788614102	0.865416106079253	1081
TNFRSF11	-0.006713522	0.82549885234082	0.865402976961513	1081
BTNL2	-0.00672213	0.825278703584783	0.86526696594751	1081
XG	-0.006739097	0.824844835924082	0.865054614098744	1081
PRR5L	-0.006740453	0.824810160841173	0.865054614098744	1081
C8orf46	-0.006766663	0.824140045670321	0.864523079568247	1081
GPX3	-0.00677763	0.823859696939369	0.864273998585825	1081
LOC33865	-0.00680809	0.823081127359942	0.863547175181815	1081
UPF3A	-0.006819345	0.822793505004787	0.863380308278021	1081
R3HCC1	-0.006834637	0.822402741288585	0.863060180415636	1081

S100Z	-0.00685057	0.821995643351008	0.862902669896095	1081
FOXP4	-0.006872155	0.821444199426468	0.862458612469183	1081
NAPG	-0.006872184	0.821443468508849	0.862458612469183	1081
UTS2D	-0.006872946	0.821424008626293	0.862458612469183	1081
GNB3	-0.006875352	0.821362546473538	0.862458612469183	1081
LHX5	-0.006903847	0.82063473525053	0.861968128766981	1081
PLEC	-0.006931184	0.81993665610681	0.861414546924208	1081
DPPA5	-0.006942198	0.819655436466022	0.861164012079275	1081
GJC2	-0.006965071	0.819071502471226	0.860819877779781	1081
SNORA61	-0.006967826	0.81900116128724	0.860790858983223	1081
SVOPL	-0.007022553	0.817604445173756	0.859591960128663	1081
C14orf79	-0.007025327	0.817533680699199	0.859562421591094	1081
HMGN5	-0.007026614	0.817500840635691	0.859562421591094	1081
ZNF233	-0.007038246	0.817204050287318	0.859337208053763	1081
ERCC2	-0.007041799	0.817113399859528	0.859299929012852	1081
GPC1	-0.007068367	0.816435659003221	0.858721680709022	1081
SPINK7	-0.007083684	0.816044979362118	0.858445227637068	1081
C1orf227	-0.007090843	0.815862404021107	0.858373965515024	1081
DCAF7	-0.007093348	0.815798507361623	0.85836524239005	1081
C17orf63	-0.00710746	0.815438639369294	0.858121056788091	1081
CHST10	-0.007115198	0.815241347961486	0.858003080021114	1081
APOA4	-0.007147719	0.814412205769841	0.857399210098429	1081
COLQ	-0.007178037	0.813639434184994	0.85667518954982	1081
IGSF1	-0.007249277	0.811824316912355	0.854942802247864	1081
COPS3	-0.007262301	0.811492588563695	0.854638132403578	1081
CCDC163	-0.00730522	0.810399660411854	0.853531717415005	1081
SBNO2	-0.007314197	0.810171110977896	0.85342486041883	1081
MAS1	-0.007314852	0.810154443248264	0.85342486041883	1081
SNORA11	-0.00732214	0.809968899694342	0.853390349599504	1081
EID3	-0.007336448	0.809604689685538	0.853095850701703	1081
COL4A3	-0.007359094	0.809028291090541	0.852622282187755	1081
MS4A13	-0.007363565	0.808914509367609	0.852546970346891	1081
KIF7	-0.007368214	0.808796197846598	0.852466876242334	1081

SEMA4A	-0.007389327	0.808258960090105	0.852123547781828	1081
CARD8	-0.007427475	0.807288477515828	0.851234042374057	1081
KRTAP20	-0.007428354	0.807266112597705	0.851234042374057	1081
OR2T3	-0.007486146	0.805796494184602	0.849972268056802	1081
GABARA	-0.007489703	0.805706044479285	0.849921362833701	1081
GSTTP2	-0.007489974	0.8056991532005	0.849921362833701	1081
TRIM49L	-0.007497963	0.805496073124408	0.849788865833527	1081
RHOXF1	-0.007500083	0.805442166908503	0.849776497976945	1081
MNS1	-0.007599503	0.802915822689685	0.847199834909056	1081
CLIP3	-0.007607699	0.802707655365192	0.847068921808894	1081
CWF19L1	-0.007621478	0.802357686315323	0.846743967249316	1081
MAGED4	-0.007631668	0.80209890997565	0.84655957151252	1081
PRAMEF1	-0.00763969	0.801895218464372	0.846388933038079	1081
HBA2	-0.007651938	0.801584228775228	0.846149354887706	1081
KRTAP5-9	-0.007660743	0.801360679662697	0.84600203814092	1081
CYP46A1	-0.007698025	0.800414312078608	0.845135820369179	1081
GJB3	-0.007698282	0.800407804606257	0.845135820369179	1081
RTP2	-0.007706558	0.800197772050008	0.845040056508042	1081
DNAJC14	-0.007743999	0.799247720874846	0.844081013736502	1081
KCNK17	-0.007759768	0.798847690514264	0.843881433627719	1081
PTPRA	-0.007768332	0.798630444050542	0.843812157782249	1081
C8orf47	-0.007769687	0.798596081632933	0.843812157782249	1081
LOC72861	-0.007790836	0.7980596803742	0.843413535303441	1081
RPL13AP3	-0.007813106	0.797494973294294	0.84289277214132	1081
ZNF789	-0.007861958	0.796256562380783	0.841716348279418	1081
C17orf100	-0.00786201	0.796255243835586	0.841716348279418	1081
EFNA3	-0.007884535	0.795684382120874	0.841288084701885	1081
SRPK3	-0.007891063	0.795518983665176	0.841201507843943	1081
C9orf91	-0.007925391	0.794649272357163	0.840502446268773	1081
GABRR2	-0.007949135	0.794047863884694	0.839910434127443	1081
TRIM53	-0.007951288	0.793993336115992	0.839896857595918	1081
CLRN1	-0.007966495	0.793608241385346	0.839621764755411	1081
AMPH	-0.007976743	0.793348734196656	0.839435382656213	1081

HNRNPU	-0.007995218	0.792880972859832	0.839072662232681	1081
CLRN3	-0.008002905	0.792686378106464	0.838954874800605	1081
SHOX	-0.008008004	0.792557307207409	0.838862341916317	1081
C9orf66	-0.008012666	0.792439299019884	0.838825583456233	1081
TMX1	-0.008018038	0.792303314965299	0.838769787165692	1081
D2HGDH	-0.008036842	0.79182739434779	0.838354068383677	1081
NRCAM	-0.008058288	0.791284727088031	0.837823548154546	1081
KRTAP5-5	-0.008061613	0.791200595206473	0.837778501468299	1081
ANKRD7	-0.008085714	0.790590864597199	0.837308919999505	1081
YWHAQ	-0.008087238	0.790552301978988	0.837308919999505	1081
C9orf11	-0.008088043	0.790531938973624	0.837308919999505	1081
SNORA38	-0.008089096	0.790505309	0.837308919999505	1081
SYCP3	-0.008095348	0.790347167272569	0.837226885665768	1081
HK1	-0.00809983	0.790233798823544	0.837194839721288	1081
ZCWPW1	-0.008121431	0.789687470960604	0.836660045361385	1081
IQCF3	-0.008131979	0.78942074015385	0.836421438510456	1081
ATPBD4	-0.008150299	0.788957552304698	0.836062592907846	1081
FBXO15	-0.008171585	0.788419445794394	0.835624229785241	1081
PCNAP1	-0.008177894	0.788259956434781	0.835499148815041	1081
PHF21B	-0.008178786	0.788237409439401	0.835499148815041	1081
C9orf9	-0.008184264	0.788098959025924	0.835499148815041	1081
TMED10	-0.008196728	0.787783960369288	0.83539021327785	1081
PGF	-0.008206058	0.787548169217165	0.835202329688606	1081
SYCE1	-0.008238047	0.786739946914645	0.834634590057166	1081
ANO5	-0.008247567	0.78649943578533	0.834423379707999	1081
RILPL2	-0.008253124	0.786359075447311	0.834318406008642	1081
SNORA84	-0.008256275	0.786279499702819	0.834318406008642	1081
SLC7A5P2	-0.008308156	0.784969344328459	0.833238878780525	1081
CYB5R1	-0.008326442	0.78450773047252	0.832836647890436	1081
USP29	-0.00836786	0.783462430147633	0.831814622922108	1081
OR51B2	-0.008385886	0.783007611339861	0.831375551069493	1081
SORBS3	-0.008407801	0.78245475326667	0.830832332097674	1081
OR2M5	-0.008414899	0.782275727502726	0.830729811836712	1081

VN1R2	-0.008427694	0.781953030490853	0.830474683637613	1081
C10orf81	-0.008441904	0.781594665636982	0.830137846747351	1081
POMZP3	-0.008453147	0.78131117441039	0.829924260927883	1081
LRIT1	-0.008507402	0.779943541053857	0.828733721954214	1081
C14orf145	-0.008541066	0.779095295314552	0.82791975123235	1081
OR6Q1	-0.008543377	0.779037074933419	0.827901554892051	1081
C12orf54	-0.008549896	0.778872848749897	0.827814368369035	1081
NKX2-2	-0.008550796	0.778850174514023	0.827814368369035	1081
FAM22F	-0.008564467	0.778505818197323	0.827555258697961	1081
C11orf64	-0.008570326	0.778358224486127	0.827442028088286	1081
CSN1S2A	-0.008589324	0.777879790238294	0.827064357119883	1081
GPR135	-0.00864512	0.776475059437528	0.825875927791394	1081
MOXD1	-0.008692575	0.775280935935445	0.824692916592013	1081
SPANXN1	-0.008693777	0.775250682140864	0.824692916592013	1081
OSTM1	-0.008694985	0.775220283645941	0.824692916592013	1081
C7orf45	-0.008708237	0.774886930860412	0.82449148165547	1081
C10orf68	-0.008711388	0.774807677909241	0.82449148165547	1081
DYNC2LI	-0.008726814	0.774419681055031	0.82421198620317	1081
FMNL2	-0.008732658	0.774272696320971	0.824099089522162	1081
LRRC67	-0.008733476	0.774252122629382	0.824099089522162	1081
IGSF21	-0.008736866	0.774166869084865	0.824073531187034	1081
PRAMEF2	-0.008752013	0.773785985327096	0.823798682719287	1081
RPL24	-0.008757904	0.773637884430613	0.823684540027203	1081
TRIM71	-0.008779476	0.773095543608303	0.823194128970309	1081
ASB16	-0.00878929	0.772848862676574	0.823018466914393	1081
OR4K14	-0.008809388	0.772343732038571	0.822567511600159	1081
OSTN	-0.008813298	0.772245497774979	0.822549862669959	1081
GBA2	-0.008816094	0.772175235291051	0.822518512845718	1081
ABCB11	-0.008827692	0.771883803335238	0.822295040623393	1081
POLR3GL	-0.008857041	0.771146464141837	0.821639896341953	1081
SLC30A2	-0.008867649	0.770880023829768	0.821573276904596	1081
SLC12A4	-0.008879712	0.770577063758461	0.82138075821018	1081
SLC25A32	-0.008920247	0.76955928283203	0.820469529116234	1081

TRIM48	-0.008934089	0.769211808712498	0.820185882946768	1081
C17orf58	-0.00893432	0.769206011228965	0.820185882946768	1081
TMSB15A	-0.008974656	0.768193770995411	0.819317212870741	1081
HBG1	-0.008979648	0.768068521183732	0.819227000542505	1081
MADD	-0.008986561	0.767895068733197	0.819085362682953	1081
HES7	-0.008998705	0.767590418420468	0.818890482395823	1081
OR10V1	-0.00900969	0.76731487761332	0.818683237620907	1081
UNC5A	-0.00902345	0.766969780853859	0.818401728656232	1081
LUC7L	-0.009026229	0.766900084690305	0.81837070696505	1081
LOC55908	-0.00904239	0.76649483691024	0.818000649517397	1081
SNORA63	-0.009071976	0.765753088453379	0.817406526435635	1081
ASH2L	-0.009075296	0.765669854057384	0.817360988288167	1081
EBPL	-0.009076101	0.765649676331451	0.817360988288167	1081
NCRNA00	-0.009085389	0.76541688232847	0.817220855125452	1081
DEFB129	-0.009098693	0.765083466003059	0.817081398644522	1081
C20orf3	-0.009102451	0.764989278541443	0.817024123434279	1081
GNS	-0.009129485	0.764311925067876	0.816525706258448	1081
COPS8	-0.009147605	0.763857991102006	0.816162046820298	1081
WIZ	-0.009152946	0.763724212058116	0.816062390702315	1081
XPNPEP2	-0.009159476	0.763560661359012	0.816017481330361	1081
C20orf195	-0.009167223	0.7633666373306	0.815853409147697	1081
LOC39974	-0.009172439	0.763236018472894	0.815757087868021	1081
NKPD1	-0.009196611	0.762630780781474	0.815153450355112	1081
AMH	-0.009240107	0.761542043939616	0.814378621677649	1081
G6PC	-0.009256076	0.761142430560378	0.813994492946797	1081
APOD	-0.009260265	0.76103762094586	0.813925614459245	1081
BCL2L13	-0.009265528	0.760905936155595	0.81382798427685	1081
DTNB	-0.009282243	0.760487791546319	0.813423943968387	1081
FCHSD2	-0.00928761	0.760353534512512	0.813323525341399	1081
HTRA3	-0.00931579	0.759648745449927	0.812655938457266	1081
GNAT3	-0.009327333	0.759360110812912	0.812390304425179	1081
FABP2	-0.009345637	0.758902490829106	0.812030099206052	1081
C1orf175	-0.009347849	0.758847188513196	0.812014055699476	1081

NAALADL	-0.009363899	0.758446011896585	0.811671000300505	1081
OR10G4	-0.00939922	0.757563327597836	0.810984867385398	1081
CD160	-0.009403885	0.757446782468316	0.810903195664783	1081
SDK1	-0.009438558	0.75658064910829	0.810018982583254	1081
HEPHL1	-0.009453408	0.756209794158539	0.809707999538841	1081
C20orf112	-0.009474851	0.755674365913784	0.809263734056307	1081
AGTR1	-0.009489153	0.755317342866522	0.809049039707376	1081
PTPN20B	-0.009530246	0.754291753646016	0.808126755156039	1081
SLC16A14	-0.009535936	0.754149789804936	0.80801763193386	1081
MPHOSPH	-0.009553337	0.753715661056303	0.807595446624074	1081
CCDC61	-0.00955551	0.753661449360874	0.807580313690149	1081
LOC12784	-0.009559044	0.753573297526864	0.807528808908914	1081
BHLHE23	-0.009580601	0.753035615731238	0.807081425777069	1081
HERC2P4	-0.009583571	0.752961542931097	0.807044973788079	1081
FHAD1	-0.009588286	0.752843976893982	0.806979590294242	1081
LOC15444	-0.009615885	0.75215582329052	0.80639590527874	1081
CDH9	-0.009627693	0.751861473371234	0.806123230670259	1081
GPRIN3	-0.009632532	0.7517408377254	0.806036788161495	1081
EPHA1	-0.009639961	0.75155567330378	0.805966943769212	1081
CAMK2D	-0.009647646	0.751364142375937	0.80580444251295	1081
TBC1D2	-0.009648756	0.751336493660895	0.80580444251295	1081
H3F3A	-0.009676939	0.750634232510006	0.80510736949814	1081
FLJ35390	-0.009687281	0.750376572646941	0.804916722895241	1081
PIK3R2	-0.009695556	0.750170448380521	0.804738467576846	1081
LECT1	-0.009712006	0.749760710019831	0.804384593031341	1081
TEKT1	-0.009784612	0.747953133170275	0.802659059648156	1081
MGC1670	-0.009795244	0.747688555427534	0.802417875930301	1081
PAIP1	-0.009800985	0.747545713887101	0.802307320525075	1081
WDR16	-0.009806414	0.747410630792202	0.802241178809356	1081
PYDC2	-0.009812161	0.747267650516095	0.802137092750399	1081
CTCFL	-0.009813802	0.747226824762204	0.802136011128349	1081
TAS2R42	-0.009820357	0.747063749816729	0.802046431467598	1081
CTSL3	-0.009822496	0.747010537329405	0.80203204575499	1081

TM4SF1	-0.009838125	0.746621780221047	0.801657379946327	1081
GTF2H2B	-0.009883898	0.745483564764491	0.800563271612595	1081
ATP4A	-0.009909708	0.744842017393824	0.800130242649101	1081
FBL	-0.009910892	0.744812604406576	0.800130242649101	1081
LOC34884	-0.009937714	0.744146083011453	0.799595841810632	1081
PLG	-0.009938363	0.744129963304378	0.799595841810632	1081
TMEM5	-0.009951909	0.743793436917752	0.799338016566623	1081
SNORD15	-0.009959271	0.743610563292025	0.799265788579816	1081
MST1P9	-0.009977844	0.743149252114527	0.798865618134853	1081
KRT23	-0.00998191	0.743048258477937	0.798799678053153	1081
CRISP2	-0.009996967	0.74267436006454	0.798525564875115	1081
DEFB118	-0.010007019	0.742424804798485	0.798299850155616	1081
CHRNG	-0.010015982	0.74220228333972	0.798145785398957	1081
PAICS	-0.010036495	0.741693096583272	0.797640798135277	1081
TAAR9	-0.010054691	0.741241531504679	0.797291850337234	1081
C10orf128	-0.010059871	0.741113004255513	0.797229746928355	1081
EIF1AY	-0.010060399	0.741099890151218	0.797229746928355	1081
CYP51A1	-0.010071036	0.740835973923387	0.797016859529326	1081
HBM	-0.010075143	0.740734068447046	0.79694978684393	1081
FAM63A	-0.010075902	0.740715254205838	0.79694978684393	1081
WRB	-0.010089683	0.740373375547824	0.796689367577102	1081
RFPL4A	-0.010098044	0.740166009906465	0.79650877508364	1081
IL34	-0.010122148	0.739568234889201	0.795993059349413	1081
KCNE4	-0.010154318	0.738770668085024	0.795262109039906	1081
OR5L1	-0.010161099	0.738602585333251	0.795123661707814	1081
TEX2	-0.010174308	0.738275224860862	0.794923667751894	1081
NUPL2	-0.010217693	0.737200302666416	0.793961640528101	1081
KDM5C	-0.010238687	0.736680330050735	0.793520759751474	1081
MEP1B	-0.010240385	0.736638295307634	0.793517911286685	1081
MORN1	-0.010254292	0.736293915547215	0.793274196635931	1081
PPOX	-0.010258417	0.73619177281988	0.793206571292533	1081
HMP19	-0.010276723	0.735738584337409	0.792760685787179	1081
SLC6A10I	-0.010313657	0.734824494260374	0.791902821209824	1081

DMRTC1I	-0.01031706	0.734740302245539	0.791854450499486	1081
TMEM18	-0.010317645	0.734725813002346	0.791854450499486	1081
CCIN	-0.010352085	0.733873839969533	0.791089924346438	1081
PPM1D	-0.010372424	0.7333708292989	0.790590001403454	1081
WDR33	-0.010394184	0.732832808276282	0.790094563699529	1081
CACNA1I	-0.010408264	0.73248477421228	0.789846150118102	1081
FGFR4	-0.010409068	0.732464879427418	0.789846150118102	1081
ST6GAL1	-0.010416051	0.732292298161079	0.789765423548099	1081
JAK2	-0.010417571	0.732254727147737	0.789765423548099	1081
LOC2856	-0.010501317	0.730185882480648	0.787831107202221	1081
PHF23	-0.010504257	0.73011328220256	0.787831107202221	1081
EVX2	-0.010527172	0.729547565648382	0.787353247079538	1081
HCG4P6	-0.010580187	0.728239274703695	0.786236142821476	1081
FARS2	-0.010582441	0.728183673498208	0.786218249684929	1081
LOC2016	-0.010583604	0.728154985858068	0.786218249684929	1081
FAM45A	-0.010589628	0.728006375978659	0.78611108726898	1081
CXCL1	-0.01059468	0.727881768961882	0.786018667152888	1081
ENPP2	-0.010637158	0.726834256106478	0.785013729855511	1081
ACCN2	-0.010638989	0.72678911344853	0.785007060769966	1081
FTMT	-0.010646835	0.726595694787435	0.78484022903447	1081
PKIB	-0.010666405	0.726113325815019	0.78440331126896	1081
FMO1	-0.010700502	0.725273159817617	0.783579738524396	1081
SPNS2	-0.010703295	0.725204354275524	0.783547423806942	1081
KLC1	-0.010724804	0.724674522044135	0.783058962966212	1081
RFPL3	-0.010728921	0.724573140595992	0.782991412794028	1081
LOC1585	-0.010737593	0.724359573768824	0.782844614462068	1081
GNAI3	-0.010738726	0.724331666912228	0.782844614462068	1081
LOC2846	-0.010740536	0.724287094867969	0.782844614462068	1081
RAMP2	-0.01082497	0.722208966170015	0.780939324932632	1081
CARTPT	-0.010836214	0.721932382317876	0.780724062797596	1081
ODF1	-0.010842306	0.721782530894417	0.780603912861332	1081
ACSF2	-0.010854989	0.721470611348593	0.780350360570062	1081
PLSCR5	-0.010855922	0.721447657523182	0.780350360570062	1081

ZNF48	-0.010861776	0.721303700485436	0.780253613611078	1081
C14orf138	-0.010893405	0.720526091113519	0.77953802929548	1081
POC5	-0.010947615	0.719194008617963	0.778305844942727	1081
LOC28326	-0.010948287	0.719177492808441	0.778305844942727	1081
A4GNT	-0.010970973	0.718620295545975	0.777810329560158	1081
PDCD2	-0.01097523	0.718515736511245	0.777738945355921	1081
SSR1	-0.010981013	0.718373731857339	0.777627019251267	1081
OR9K2	-0.010985669	0.718259401237794	0.777545039386069	1081
DNASE1L	-0.011015029	0.717538620051198	0.776973527248515	1081
ZNF696	-0.011048263	0.716723046455995	0.776215567488631	1081
TIAL1	-0.011058248	0.716478070097338	0.776075423277281	1081
PDLIM4	-0.011065276	0.716305649743505	0.775930382001555	1081
ANP32A	-0.011093455	0.715614506090287	0.77547360006395	1081
AKR1E2	-0.011148135	0.714274045777614	0.774270913268943	1081
C1QTNF1	-0.011164993	0.71386095122554	0.77398971272543	1081
INVS	-0.011173434	0.713654148852713	0.773807138631676	1081
OR2M3	-0.011184541	0.713382066838313	0.773637044382958	1081
C15orf60	-0.011202558	0.712940758000025	0.773292390253739	1081
MC5R	-0.011210936	0.712735594618165	0.773185705631822	1081
PRAMEF1	-0.011222063	0.71246313345892	0.772931758496954	1081
CNTNAP2	-0.011242345	0.711966597327727	0.772559499227959	1081
STRAP	-0.01124479	0.711906735971867	0.772536155793647	1081
SEMA6B	-0.011248804	0.711808498465783	0.772471163152141	1081
CNBP	-0.011269715	0.711296715626883	0.771998940590677	1081
CCDC89	-0.011295447	0.710667150959634	0.771357206685444	1081
C10orf10	-0.011313785	0.710218581916137	0.770994952454631	1081
LOC14583	-0.01132965	0.709830611861351	0.770615309115484	1081
OR10Q1	-0.011332279	0.709766315077639	0.770587034073782	1081
HPGDS	-0.011377924	0.708650518375736	0.769458561563046	1081
LRRIQ3	-0.011397368	0.708175401090456	0.769108493987128	1081
STX18	-0.011400044	0.708110003627353	0.769078931586857	1081
TFB1M	-0.011404336	0.708005162396864	0.769006523776612	1081
APOM	-0.01140701	0.707939839133914	0.768977033287647	1081

NDST4	-0.011407754	0.707921656782964	0.768977033287647	1081
OR1L1	-0.011418207	0.707666303525849	0.76884573856802	1081
GRIA2	-0.011423869	0.707528017451253	0.7687784202565	1081
RNASE9	-0.011439052	0.707157200311518	0.768464718964072	1081
SBDSP1	-0.011464096	0.706545738256344	0.768042515496117	1081
C1orf87	-0.011466645	0.706483498129256	0.768016300783221	1081
TDRD6	-0.011537442	0.704755989749437	0.766427845686807	1081
SIPA1L1	-0.011543353	0.70461182684325	0.766395186639883	1081
CDC42EP	-0.011545646	0.704555888765674	0.766395186639883	1081
NFASC	-0.01156077	0.704187098839642	0.766057301335165	1081
C14orf93	-0.011575065	0.703838558959201	0.76580220201097	1081
C17orf97	-0.011575663	0.703823988320543	0.76580220201097	1081
LMAN2L	-0.011584548	0.703607392589127	0.765633388630042	1081
AEBP1	-0.01160072	0.703213219649356	0.765328487835563	1081
ADCY8	-0.011613124	0.702910950942164	0.765082186445315	1081
RHOXF2E	-0.01161974	0.70274974828105	0.764948056363632	1081
B3GALNT	-0.011627676	0.702556391241355	0.764778909626991	1081
SNORA14	-0.011642425	0.702197086090318	0.76447040095598	1081
TAOK2	-0.011647008	0.702085466988161	0.764390192535076	1081
YWHAG	-0.011678152	0.701327039250199	0.763647003172545	1081
B4GALT3	-0.011703107	0.70071953779661	0.763109259861212	1081
ALDH18A	-0.011711108	0.700524799629722	0.762938427233646	1081
S100A1	-0.011712753	0.700484761160618	0.762936067992033	1081
C1QTNF8	-0.011716534	0.700392746442271	0.762877095273292	1081
SNORA72	-0.011728155	0.700109965006555	0.762651557078735	1081
SEMA3F	-0.011749875	0.69958153986357	0.762158353823569	1081
PRCD	-0.011756542	0.699419382078228	0.762022901674738	1081
MKL1	-0.01176889	0.699119060459366	0.76177809784476	1081
P2RY13	-0.011773817	0.698999248187583	0.761688746402275	1081
UCA1	-0.011775847	0.698949869714592	0.761676140073594	1081
DDI1	-0.0117945	0.698496327853538	0.761346635894899	1081
MTFR1	-0.011841228	0.697360632519157	0.76027329484243	1081
ABCC2	-0.011847817	0.69720055521269	0.760139913663454	1081

MTL5	-0.011859695	0.696911984416332	0.759866417301743	1081
LOC40112	-0.011880754	0.696400512399608	0.759390945236013	1081
LQK1	-0.011913444	0.695606802382488	0.75877187751761	1081
PCSK4	-0.011917256	0.695514277728269	0.75871203361867	1081
EXOC3	-0.011997319	0.693571859110898	0.756757033081787	1081
C20orf173	-0.012009455	0.693277605230042	0.756525645498635	1081
SAA1	-0.012023991	0.692925202140092	0.756256268128299	1081
MRAS	-0.012025679	0.692884282177236	0.756252579750795	1081
CXCL17	-0.012047917	0.69234533059507	0.755796322468068	1081
CES2	-0.012051538	0.692257583341064	0.755773304596019	1081
SOX11	-0.012059892	0.692055166970537	0.755593265330956	1081
CECR2	-0.012062602	0.691989502340426	0.755562521661132	1081
CDC16	-0.012088532	0.691361387596232	0.754917619010575	1081
FKBP1B	-0.012091376	0.691292499055533	0.75488331491076	1081
ALPL	-0.012113113	0.690766112596196	0.754390292651544	1081
SYCP1	-0.01213914	0.69013606681989	0.753743077586701	1081
C3orf35	-0.012149255	0.689891256460197	0.753598273581535	1081
C2orf14	-0.012171004	0.689364980627492	0.753105072382908	1081
PRTN3	-0.01218831	0.688946345611604	0.752688547770799	1081
SLC17A1	-0.012188665	0.688937741576556	0.752688547770799	1081
HAS1	-0.012200026	0.688662977223196	0.752501392718664	1081
OR6K6	-0.012203591	0.68857675344494	0.752488808622562	1081
KCNH7	-0.012206185	0.688514023863267	0.752461075827348	1081
TSPAN19	-0.012214728	0.688307433040804	0.752276108865397	1081
SLC25A2	-0.012215822	0.688280980201177	0.752276108865397	1081
ZCCHC8	-0.012235479	0.687805766021918	0.751809395361451	1081
LOC34001	-0.012263086	0.687138513738231	0.751120807382748	1081
RPL31P11	-0.012277736	0.686784560376667	0.750774634729106	1081
ACSS3	-0.012282904	0.686659690881132	0.750678866489413	1081
ADRA2B	-0.012286573	0.686571051551642	0.750622698008674	1081
C8orf41	-0.012308231	0.686047953005983	0.75013221956717	1081
CLVS1	-0.012339678	0.685288645898584	0.749505389631738	1081
DYRK3	-0.012391817	0.684030454711117	0.748291800714388	1081

ATF7IP2	-0.01239978	0.683838370348107	0.748122296658119	1081
SLC36A1	-0.012415333	0.683463256755329	0.747833757391565	1081
CXCL14	-0.012438939	0.682894068616672	0.747332736434314	1081
OSGIN2	-0.012450104	0.682624923192433	0.747119367473191	1081
OR2G6	-0.012468806	0.682174183214455	0.746666608033426	1081
MRO	-0.012534534	0.680590958232347	0.745304637031175	1081
MST1	-0.012587304	0.679320853818143	0.743988180937615	1081
GALNT9	-0.012607664	0.678831068126951	0.743535924651218	1081
IQCF1	-0.012621725	0.678492889141147	0.743283444001762	1081
CLEC11A	-0.012622139	0.678482927156934	0.743283444001762	1081
C12orf43	-0.01262623	0.678384562429796	0.743245608862144	1081
POLR3H	-0.012638655	0.678085798810838	0.742999098017097	1081
C13orf1	-0.012663793	0.677481499909418	0.742417712869239	1081
ZCCHC12	-0.012674496	0.677224275466531	0.742256965684073	1081
C10orf28	-0.012698856	0.67663896489341	0.741696155608757	1081
CDH3	-0.012702703	0.676546564033749	0.741635225143378	1081
C17orf105	-0.012728191	0.675934376408643	0.741165796470287	1081
RASD2	-0.012728344	0.675930704055688	0.741165796470287	1081
SEMA3A	-0.012758572	0.67520497513007	0.740486919483656	1081
EPS8L3	-0.012798727	0.674241370101937	0.739631474662574	1081
PSG4	-0.012811385	0.673937737634209	0.739378919760436	1081
WDSUB1	-0.012841675	0.673211342259537	0.738622215011077	1081
PRR18	-0.012846507	0.67309548317609	0.738575549244639	1081
C12orf41	-0.012851211	0.672982726948981	0.738492048937097	1081
HAT1	-0.012873038	0.672459529982804	0.738118957745523	1081
GUCY2GI	-0.01291108	0.671548080298327	0.737279202006093	1081
SCUBE3	-0.012916707	0.671413300835785	0.737171405348642	1081
ADCY9	-0.012920352	0.671325982267554	0.737155887538421	1081
POU3F4	-0.012946678	0.670695597375513	0.736624294467872	1081
LOC73066	-0.012949164	0.670636073870415	0.736599079009841	1081
ASAP2	-0.012963264	0.67029854952424	0.736337856049677	1081
MFRP	-0.012975609	0.670003093437561	0.73618481058687	1081
LY6G5C	-0.012988372	0.669697684162869	0.735889371486446	1081

NBL1	-0.0129887	0.669689846039069	0.735889371486446	1081
GSC2	-0.012989669	0.669666659932761	0.735889371486446	1081
LOC28507	-0.012996053	0.669513920929151	0.735847997005714	1081
ASB13	-0.012998582	0.669453414448717	0.735821640881133	1081
BUD13	-0.013005821	0.669280228775847	0.735699190866017	1081
VAT1L	-0.013007123	0.669249089896949	0.735699190866017	1081
C12orf74	-0.013027523	0.668761163134128	0.735221219784819	1081
RAB8B	-0.013045529	0.668330626728567	0.734807271916332	1081
C1orf74	-0.013050618	0.668208948491604	0.734755474473941	1081
ARID3C	-0.013069942	0.667747038824626	0.734346768049028	1081
WDR81	-0.013079801	0.667511431758307	0.734127738863957	1081
GABRR3	-0.013085836	0.667367224902503	0.734049289962377	1081
TMEM163	-0.013091561	0.667230440810752	0.733938911768735	1081
ACTL7A	-0.013100714	0.667011769815853	0.733818584618008	1081
ATOH1	-0.01312052	0.666538661871304	0.733338139999859	1081
VPS37C	-0.013132049	0.666263345381494	0.733115309848711	1081
OR4D10	-0.013134051	0.666215549148995	0.73310276056189	1081
HSPA12A	-0.013168598	0.665390814958687	0.73239525556457	1081
CCBL1	-0.013241481	0.663652239190381	0.730681224161897	1081
RDH5	-0.013261195	0.663182291722075	0.730243633054999	1081
FAM120A	-0.013266572	0.663054132148105	0.730142423314943	1081
CRIP2	-0.013278484	0.662770248634528	0.729909613970181	1081
PPM1E	-0.013308726	0.662049766509048	0.729315195586345	1081
C20orf200	-0.013326506	0.661626329092977	0.728928761256659	1081
FAM177B	-0.01335296	0.660996484384153	0.728321076597247	1081
SPATA22	-0.013368612	0.660623945755829	0.727983665404035	1081
ELSPBP1	-0.013369514	0.660602498383272	0.727983665404035	1081
SLC45A4	-0.013377285	0.66041756470685	0.727835868538733	1081
DSE	-0.013406776	0.659715954328218	0.72722177291064	1081
DSCAM	-0.013409762	0.659644946689153	0.727183290524953	1081
C14orf177	-0.013423433	0.659319824823705	0.726864656655915	1081
C14orf4	-0.013428436	0.659200856452855	0.726813050913621	1081
PDE3B	-0.01343205	0.659114924720873	0.726758082019814	1081

R3HDML	-0.013432577	0.659102389495003	0.726758082019814	1081
SPA17	-0.013440419	0.658915940158031	0.726657996084936	1081
DBN1	-0.013454001	0.658593075656569	0.726341700925303	1081
CACNA2I	-0.013463263	0.658372943547303	0.726138677676453	1081
VSIG8	-0.013509904	0.65726484691488	0.725154736902363	1081
INE2	-0.013528492	0.656823439985537	0.72474712157028З	1081
C15orf50	-0.013541424	0.656516428639168	0.724567109786668	1081
SDR42E1	-0.013541671	0.656510563980068	0.724567109786668	1081
ELP4	-0.013551254	0.656283095614001	0.724388962859243	1081
CLDN2	-0.013595866	0.655224531631728	0.723379086409885	1081
CDC42SE:	-0.013620864	0.65463169439355	0.722803808361637	1081
CCDC13	-0.013625905	0.654512163566356	0.722790677285618	1081
AGAP6	-0.013661106	0.653677772352941	0.722027564647987	1081
WIPF3	-0.013661518	0.653668020001848	0.722027564647987	1081
CDHR1	-0.013701727	0.652715450641352	0.721162329466903	1081
OXGR1	-0.013716254	0.652371453545398	0.720821793093025	1081
CYTH2	-0.013721299	0.652252006722893	0.720729344272541	1081
SDC3	-0.013762938	0.651266455625124	0.71979825251375	1081
VWC2L	-0.013778194	0.650905496654395	0.719439260480423	1081
C1orf77	-0.013825711	0.649781810128406	0.718393862303756	1081
SNPH	-0.013826544	0.649762133186308	0.718393862303756	1081
LRP10	-0.013835303	0.649555081869692	0.718222027787746	1081
TAS2R39	-0.013848592	0.649241012030848	0.717992983495906	1081
PRAMEF4	-0.013876384	0.648584373615963	0.717463742051155	1081
SNORA29	-0.013896961	0.648098372087446	0.71706501634332	1081
TMEM64	-0.013898242	0.648068136902823	0.71706501634332	1081
TARSL2	-0.013913062	0.647718216642265	0.716820495207297	1081
RIPK1	-0.013935505	0.647188443301911	0.716312905352288	1081
GPR77	-0.01393756	0.647139936216772	0.716298572257521	1081
LOC72887	-0.01396895	0.646399318028155	0.715675419712954	1081
OR7D4	-0.013972682	0.646311275146408	0.71561727150843	1081
FAM74A4	-0.013980902	0.646117406569623	0.715481264036118	1081
ECD	-0.013986468	0.645986136206229	0.715434351146811	1081

TAS2R30	-0.0139992	0.64568591506521	0.715200019738751	1081
11-Mar	-0.014005453	0.645538476568354	0.715143967363335	1081
PEX5L	-0.014022042	0.645147429027591	0.714839390449416	1081
GLG1	-0.014027337	0.645022616769769	0.71474040785627	1081
MBIP	-0.014028342	0.644998941460847	0.71474040785627	1081
NEAT1	-0.014029244	0.644977688167481	0.71474040785627	1081
USP32	-0.014036079	0.644816605063648	0.714658972002607	1081
OR1E1	-0.014039964	0.644725047721776	0.714646532758621	1081
HOXD13	-0.014055123	0.644367888612469	0.714368560679004	1081
SSX2IP	-0.014061697	0.644213023349549	0.714275488765296	1081
PLA2G2C	-0.014068678	0.644048586487254	0.714171782616048	1081
EYA1	-0.014102587	0.643250105646122	0.713443449773778	1081
ATP2B2	-0.014108963	0.643100024772318	0.713316264675605	1081
C3orf24	-0.014130899	0.642583764029762	0.712822132509887	1081
SLC43A1	-0.014133506	0.642522397446522	0.712793308913497	1081
PLA2G5	-0.014151434	0.642100602568187	0.712403846380797	1081
C15orf37	-0.014174803	0.641550968117008	0.711950876037961	1081
SGPP1	-0.014175734	0.641529081774814	0.711950876037961	1081
PAX2	-0.014192131	0.641143551043326	0.71165556426072	1081
NOMO2	-0.014202084	0.640909584477332	0.711474270088491	1081
AQP10	-0.014213945	0.640630802725447	0.711203985501165	1081
NHEDC1	-0.014220947	0.640466259918461	0.711060501849402	1081
ARHGAP1	-0.014244429	0.639914554407094	0.710565466791473	1081
ATXN10	-0.014245851	0.639881141999922	0.710565466791473	1081
PIWIL1	-0.014250104	0.639781251631164	0.71049577255291	1081
FAM3C	-0.014256161	0.639638988696013	0.710376946208787	1081
SIK1	-0.014271518	0.639278345154631	0.710015561977067	1081
CD1E	-0.014280455	0.63906852450942	0.709821658832346	1081
DKK1	-0.014284688	0.638969144918661	0.709750409372872	1081
FOXG1	-0.014305357	0.638484000541571	0.709328858485079	1081
CEACAM	-0.014308544	0.638409192954099	0.709284866096422	1081
ATP6V0A	-0.014309607	0.638384262831659	0.709284866096422	1081
HDAC2	-0.014315483	0.638246368000706	0.70918218882373	1081

C17orf47	-0.014329607	0.637914978527211	0.708931276131222	1081
NCRNA0(	-0.014346519	0.637518293353978	0.708568607978808	1081
PWRN1	-0.01435083	0.637417193283229	0.708495329876995	1081
PLEKHF2	-0.014368818	0.636995386943127	0.708102188117696	1081
PJA1	-0.014379159	0.636752953329497	0.707874192639629	1081
GABRE	-0.014389712	0.636505594522367	0.70763825616187	1081
GRAPL	-0.014403165	0.636190307001141	0.707365809390549	1081
HSFYL1	-0.014413996	0.635936527865089	0.707122666768351	1081
CD300LD	-0.014424846	0.635682349438402	0.706900138850425	1081
LOC1431ε	-0.014428965	0.635585862401259	0.70688882007803	1081
FAM138B	-0.01444732	0.635155986471765	0.706488727705466	1081
MTSS1L	-0.014464486	0.634754063705434	0.706119642903858	1081
CBARA1	-0.014484015	0.634296942518065	0.70576702054827	1081
NANOS3	-0.014506547	0.633769707796568	0.705258286668611	1081
IFNA5	-0.014530005	0.633221001011175	0.704686612825661	1081
LARP4B	-0.014580061	0.632050805891922	0.703656451212643	1081
OR14J1	-0.014591725	0.631778268623633	0.703430786570668	1081
MBL1P	-0.014597973	0.631632284013663	0.703307117038206	1081
ST7OT2	-0.014610536	0.631338823871881	0.703019214355151	1081
ATP6V1C	-0.014613271	0.631274944351765	0.702986940517761	1081
CEACAM	-0.014619718	0.631124368714069	0.702896970795274	1081
COL6A2	-0.014649388	0.630431626164758	0.702164267655722	1081
CXCL3	-0.014680633	0.629702450006525	0.70150726402596	1081
LRRC2	-0.014684309	0.629616689957955	0.701450515385632	1081
UGT2B10	-0.014760456	0.627841247213508	0.699937018545441	1081
LINGO4	-0.014782762	0.62732156216104	0.699396362263222	1081
QDPR	-0.014787719	0.627206114350941	0.699306352313466	1081
CORO6	-0.014819716	0.626461036265537	0.698552948941063	1081
TIGD7	-0.01483612	0.626079207195827	0.69816582501854	1081
PSPC1	-0.014856732	0.625599555362366	0.69770819037673	1081
SMC6	-0.014889529	0.624836702307745	0.696973165447925	1081
DUSP10	-0.01490003	0.624592526479045	0.696777962449903	1081
PYY	-0.014903822	0.624504356709415	0.696777962449903	1081

EME2	-0.014911264	0.624331365098068	0.696640929982307	1081
APOBEC4	-0.014913383	0.624282113792898	0.696624560037552	1081
C1orf94	-0.014923782	0.624040407559936	0.696393419582035	1081
IER3IP1	-0.014932745	0.623832108496148	0.696238106684482	1081
SCARNA5	-0.014952049	0.623383597776199	0.695776086054714	1081
LHX8	-0.014966831	0.623040236737108	0.695431381264907	1081
DYDC1	-0.014969477	0.622978786835187	0.695431381264907	1081
SNX7	-0.014981076	0.622709432675301	0.695177695829533	1081
GFRA1	-0.014986471	0.622584160821944	0.695114887754701	1081
CRYBA1	-0.014988642	0.622533772163665	0.695097153322084	1081
TKTL2	-0.015008438	0.622074229778362	0.694738072895283	1081
SFRS1	-0.015022541	0.621746960984866	0.69444957468619	1081
RAPGEF1	-0.015037179	0.621407335453824	0.694185702474202	1081
B3GNT5	-0.01505948	0.620890059906026	0.693684779369247	1081
PDHA2	-0.015063294	0.620801632003698	0.693624452396811	1081
TMEM190	-0.015070393	0.620637026403027	0.693555938142175	1081
SLC7A13	-0.015077645	0.620468869684143	0.693444958656702	1081
HMGCS1	-0.01507896	0.620438388879453	0.693444958656702	1081
MAPK15	-0.015097474	0.620009251835348	0.693046631	1081
RBM43	-0.015103611	0.619867024317473	0.692926097601437	1081
SCML1	-0.015121494	0.619452677708978	0.692539774263132	1081
LOC28305	-0.015122548	0.619428247826822	0.692539774263132	1081
RGAG4	-0.015128319	0.619294559449841	0.692439856808405	1081
KIF20B	-0.015172563	0.618270044568075	0.691409461964243	1081
SNX3	-0.015175469	0.618202786469431	0.691372627181852	1081
SFT2D2	-0.015181241	0.618069185246466	0.691261588762495	1081
GCM1	-0.015211162	0.617376853923807	0.690525607811621	1081
DCST2	-0.01524287	0.616643551316583	0.689743716894645	1081
MDGA1	-0.01527021	0.616011571662141	0.689151613879816	1081
DUPD1	-0.015339229	0.61441739624315	0.687673673388424	1081
EPHA6	-0.015342226	0.614348211270412	0.687634443607204	1081
MIA2	-0.01534281	0.614334731554612	0.687634443607204	1081
ELL2	-0.015344563	0.614294266873685	0.687634443607204	1081

FLJ46111	-0.015345514	0.614272319476106	0.687634443607204	1081
UBE2NL	-0.015357905	0.613986355219345	0.687382189946299	1081
TAS2R40	-0.015388747	0.613274791295583	0.686661886985856	1081
PRDM8	-0.015390579	0.613232526055869	0.686652728441747	1081
BLID	-0.015406273	0.61287060931717	0.686323777013419	1081
OR13C9	-0.015406951	0.612854964233038	0.686323777013419	1081
C5orf38	-0.015422583	0.612494561181949	0.686055209063685	1081
TEX15	-0.015429075	0.612344917078884	0.685954252170461	1081
KLHL26	-0.01544544	0.611967763129305	0.685617629066228	1081
M6PR	-0.015464848	0.611520601705832	0.685154756471857	1081
MAGEB4	-0.015480134	0.611168501703303	0.684836437	1081
OR1J2	-0.015486306	0.61102637924681	0.684719006026006	1081
TM2D1	-0.015501473	0.610677140881262	0.684514300192134	1081
BRCC3	-0.01552987	0.610023545056757	0.683857780476815	1081
MST1P2	-0.01553916	0.609809797435865	0.683694254749861	1081
PAPD7	-0.015542998	0.609721484622685	0.683633289237157	1081
CCNH	-0.015550757	0.609543002172549	0.683509255706429	1081
KIAA0913	-0.015557806	0.609380863424338	0.683365480610292	1081
H1F0	-0.015561771	0.609289651629532	0.683301232092463	1081
CYB5R4	-0.015563066	0.609259874649454	0.683301232092463	1081
FLJ40434	-0.015564776	0.609220554205445	0.683299819857953	1081
LOC38949	-0.015630895	0.607700797154326	0.681633215961799	1081
ASMTL	-0.015639767	0.607496999442871	0.681442566611539	1081
CCDC152	-0.015647744	0.607313788312863	0.681274989729515	1081
ELF3	-0.01566089	0.607011927615616	0.680974287565662	1081
ZNF416	-0.015687762	0.606395054246907	0.680395921120861	1081
FIGLA	-0.015698809	0.606141558102065	0.680187259802034	1081
OR6C3	-0.015767295	0.60457099524189	0.678613846277811	1081
KNCN	-0.015767935	0.604556327107282	0.678613846277811	1081
SNHG9	-0.015771016	0.604485716606116	0.678593745390371	1081
ZNF267	-0.015773557	0.604427472657926	0.678593745390371	1081
NPTX2	-0.015777618	0.604334416502568	0.678575153026265	1081
C5orf23	-0.015779188	0.604298447842475	0.67857258817094	1081

LOC54147	-0.015783932	0.604189728513482	0.678488326044044	1081
C7orf49	-0.015791695	0.604011870840809	0.678326409749587	1081
SNORA10	-0.015802672	0.603760420516044	0.678119629309528	1081
ZNF536	-0.015853504	0.60259659422184	0.676887944161869	1081
FAM66E	-0.015864288	0.602349803997436	0.676761673258692	1081
ARHGAP8	-0.015864851	0.602336942861084	0.676761673258692	1081
SLC10A2	-0.015868303	0.602257957206161	0.676733966305115	1081
SNORA42	-0.015920864	0.601055894643793	0.675571667555611	1081
ZNF404	-0.015954199	0.600294099802631	0.674790728225211	1081
DLX6	-0.015961253	0.60013295128125	0.674684876586907	1081
CCDC64B	-0.015962784	0.600097975347818	0.674683207577955	1081
SCARNA7	-0.015967818	0.599982998762419	0.674591589555669	1081
LOC37519	-0.015971863	0.599890598545024	0.674525346488586	1081
ISX	-0.015974368	0.599833391559978	0.674498669995856	1081
HTN1	-0.015980546	0.599692301908282	0.674396814589654	1081
POLM	-0.01598522	0.599585568552135	0.674332920862146	1081
SLC4A3	-0.01602528	0.598671166656647	0.673454916925293	1081
SEC14L3	-0.016035373	0.598440886645442	0.673271063412958	1081
FCN2	-0.016037263	0.59839776343057	0.673260147685498	1081
MOV10L1	-0.016067635	0.59770504180526	0.672555888240348	1081
LOC15100	-0.016074255	0.597554115033621	0.672423620117992	1081
CNPY1	-0.016097045	0.597034623540962	0.67187657065151	1081
CYB5R3	-0.016119995	0.596511685103179	0.671400597631219	1081
KCNG1	-0.016137947	0.596102790593605	0.670977857546414	1081
SNORA41	-0.016164917	0.595488726876922	0.670399038948004	1081
C7orf68	-0.016168158	0.595414960178779	0.670353455138965	1081
KRTAP21	-0.016171875	0.59533035116332	0.670295658628721	1081
C20orf132	-0.016190632	0.594903518502212	0.669852516920634	1081
EFCAB3	-0.016211612	0.594426243159172	0.669389941776596	1081
HPCAL4	-0.016220848	0.594216185217725	0.669190801163409	1081
ZNF488	-0.016223271	0.594161085466545	0.669166157920476	1081
CITED1	-0.016237699	0.593833031980306	0.668871480527999	1081
SEMG1	-0.016271743	0.593059296962937	0.668112042127187	1081

LOC44104	-0.016292583	0.592585891069234	0.667690741964859	1081
DNAL4	-0.016332316	0.591683774348248	0.666860782938651	1081
ACER3	-0.016339293	0.591525425772673	0.66671961630339	1081
LOC38894	-0.016343593	0.591427865987755	0.666646953912451	1081
SNORA22	-0.016355849	0.591149783572544	0.666408080026239	1081
SNORA5C	-0.016378319	0.590640099131272	0.665897651095806	1081
FGF11	-0.016381856	0.590559886645535	0.665854867435736	1081
OR2L2	-0.01638412	0.59050854248911	0.665834243168203	1081
PCDHA7	-0.016388783	0.590402810477219	0.665752287549039	1081
HOXB3	-0.016394352	0.590276541024373	0.66568442696837	1081
PPT1	-0.016433311	0.589393589958839	0.664837553598792	1081
DHX36	-0.016449104	0.589035832048062	0.664545633770621	1081
AVP	-0.016459337	0.588804076728433	0.664395794863289	1081
RPS6KC1	-0.016465153	0.588672362939652	0.664291175005976	1081
TLE4	-0.016492749	0.588047658081956	0.663690967116421	1081
ARHGDIE	-0.016496217	0.587969182312473	0.663639577413983	1081
NOBOX	-0.016501263	0.587854989364535	0.663547865789688	1081
CEPT1	-0.016534261	0.587108505650157	0.662853827625535	1081
NCRNA0(	-0.016535968	0.587069894839487	0.662847384348249	1081
CCND2	-0.016545534	0.586853591942659	0.662640300974433	1081
NSUN3	-0.016554089	0.58666016360045	0.662496159859356	1081
SLC25A25	-0.016563402	0.586449622091976	0.662369793509916	1081
PAQR8	-0.016568451	0.586335503769253	0.662278033273429	1081
PRAMEF2	-0.016577428	0.5861326417331	0.662086019272922	1081
OR51I2	-0.016587376	0.585907848866091	0.661906326570626	1081
WHSC1	-0.016613439	0.585319111214228	0.661352487262921	1081
AP4M1	-0.016614662	0.585291484870325	0.661352487262921	1081
C2orf27B	-0.016616923	0.585240436925941	0.661337779877325	1081
HHATL	-0.016625783	0.585040377267259	0.661224934433401	1081
C10orf4	-0.016627321	0.585005639531197	0.661224934433401	1081
SNORA24	-0.016641079	0.58469506002131	0.661021286825056	1081
MUC5B	-0.016645621	0.58459253627924	0.661013507147805	1081
CCDC48	-0.016652855	0.584429277575536	0.660940200794879	1081

HSDL1	-0.016676838	0.583888162983415	0.660476559228531	1081
C18orf22	-0.016689928	0.583592910602857	0.660179648702524	1081
KCTD14	-0.016692093	0.583544079984823	0.660179648702524	1081
GSTT1	-0.016760232	0.582008378181806	0.658720084188577	1081
KTELC1	-0.016797195	0.581176083047214	0.657888974656446	1081
BREA2	-0.016818433	0.580698129182265	0.657495717871893	1081
ARX	-0.016822302	0.580611093373126	0.657434122646373	1081
ZNF541	-0.01683861	0.580244233071885	0.657055653470102	1081
KRTAP5-2	-0.016864853	0.579654103430511	0.656608857040466	1081
METTL11	-0.016875077	0.579424285916967	0.656422350680311	1081
FMO9P	-0.016881916	0.579270572952649	0.656285118504646	1081
C1orf129	-0.016889502	0.579100098971569	0.6561288804152	1081
LACE1	-0.01690327	0.578790755002289	0.655815274172964	1081
CLEC3B	-0.016916552	0.57849239927197	0.655550958166948	1081
TSPAN16	-0.01692603	0.578279528229652	0.655346596320114	1081
MOG	-0.016928059	0.578233965151423	0.655331827171613	1081
TMCO1	-0.016930669	0.578175361725694	0.655302276469231	1081
CCDC74B	-0.016945547	0.577841339290434	0.654960545772002	1081
RAB2A	-0.016946754	0.577814234215492	0.654960545772002	1081
C1orf104	-0.016958759	0.577544785470595	0.654808628056345	1081
NR4A2	-0.016964342	0.57741949328043	0.654703421631917	1081
GOLGA2	-0.016970888	0.577272595013588	0.654610550326413	1081
C1orf103	-0.016974561	0.577190174967742	0.6545792027748	1081
SPSB3	-0.016980928	0.577047337071973	0.654513718345463	1081
EPHB6	-0.017016912	0.576240345617648	0.65369758770512	1081
BRD2	-0.017028227	0.575986691387569	0.653483436472324	1081
RPS2	-0.017036314	0.575805456317782	0.653314610955884	1081
OMP	-0.017046521	0.575576710942502	0.653091857718639	1081
PHF21A	-0.017049224	0.575516155468591	0.653059930823791	1081
PPHLN1	-0.017054767	0.575391954448659	0.652955775257337	1081
LOC64392	-0.017070327	0.575043405388038	0.652707313586997	1081
TUBA1A	-0.017090558	0.574590380868612	0.652266607832649	1081
CDRT15	-0.017092195	0.574553742490226	0.652266607832649	1081

C15orf40	-0.017095966	0.574469303747549	0.652239425350526	1081
FGL1	-0.017103709	0.574295991280954	0.652079401665811	1081
FAM176A	-0.01710845	0.574189859094715	0.651995643507302	1081
UVRAG	-0.017113502	0.57407679867323	0.651904008414443	1081
C21orf94	-0.01713608	0.573571634123648	0.651367076465465	1081
HTN3	-0.01713896	0.573507212728396	0.651330634818668	1081
NONO	-0.017146925	0.573329047765206	0.651165003508489	1081
TRNP1	-0.017151156	0.573234430109241	0.651094248677868	1081
GIP	-0.017154003	0.57317075822642	0.651058636846418	1081
WBP11	-0.017154453	0.573160683349518	0.651058636846418	1081
IQCF5	-0.017197553	0.57219726413561	0.650136146983185	1081
PSG5	-0.017202614	0.572084174714228	0.650080984861131	1081
SHH	-0.01721239	0.571865775916697	0.64990044977112	1081
CYP3A43	-0.017217028	0.571762175430643	0.649825050722161	1081
L3MBTL4	-0.017255461	0.570904021995963	0.648922954528504	1081
DYNLRB2	-0.017265177	0.570687167558377	0.64871306796431	1081
CDC27	-0.01726571	0.57067529141165	0.64871306796431	1081
ZNF251	-0.017284811	0.570249100212546	0.648324856017932	1081
SGCA	-0.01728945	0.570145613722068	0.648243785327411	1081
CTAGE4	-0.017295422	0.570012409347846	0.648165499340278	1081
VMAC	-0.017296643	0.569985179593681	0.648165499340278	1081
AMTN	-0.017298061	0.56995354716659	0.648165499340278	1081
SLC13A1	-0.017314768	0.569580997450971	0.647821205603535	1081
LRRC8D	-0.017330245	0.569235967491909	0.647501895263948	1081
KCNT1	-0.017341519	0.568984692886711	0.647256150217442	1081
ADRB3	-0.017356455	0.568651888643521	0.647020180554856	1081
UBQLN4	-0.017375593	0.568225596952607	0.646571658981601	1081
PLAC1L	-0.017379833	0.568131176486972	0.646515714259577	1081
ADAMTS	-0.017408725	0.567487964824527	0.645987740938583	1081
CGREF1	-0.017418065	0.567280096968267	0.645787610387983	1081
LOC14962	-0.0174227	0.567176966742177	0.645706696525638	1081
ARID5A	-0.017423366	0.567162140803602	0.645706696525638	1081
ABCC12	-0.017424539	0.567136044894431	0.645706696525638	1081

ILF3	-0.017430409	0.567005446640577	0.645620886421797	1081
GLTSCR1	-0.017444311	0.566696196647167	0.645378194440453	1081
PRM1	-0.017447893	0.566616528327401	0.645323946356597	1081
PRR21	-0.017455231	0.566453338555429	0.645211042926559	1081
TM4SF4	-0.017467456	0.566181527273172	0.644937905960873	1081
ADRA1B	-0.01746778	0.566174307351686	0.644937905960873	1081
BCORL2	-0.017470557	0.566112591066421	0.64493231618125	1081
ARMC8	-0.01748215	0.565854880633463	0.64471164359262	1081
C21orf84	-0.017483574	0.565823241782104	0.64471164359262	1081
AMT	-0.017484691	0.565798415296887	0.64471164359262	1081
RIOK1	-0.017527822	0.564840208806296	0.643777884963406	1081
GLS2	-0.017541303	0.564540868472066	0.643505675061946	1081
NXN	-0.017552183	0.564299353616812	0.643339585659914	1081
PI4KB	-0.017561689	0.564088374900049	0.643135452621058	1081
KCNK10	-0.017564693	0.564021700582348	0.643095831912577	1081
HSD11B1I	-0.017570104	0.563901622813301	0.643031710153625	1081
SAP130	-0.017576624	0.563756959694341	0.642929847992711	1081
DMC1	-0.017628296	0.562611105955465	0.641814413583603	1081
PCDHB1	-0.017637459	0.562408021981435	0.641619073667233	1081
UMOD	-0.017653925	0.562043180579849	0.641275479881121	1081
C2orf82	-0.017660226	0.561903599563384	0.641188853132682	1081
PMCHL2	-0.01766954	0.561697298460249	0.641026063759444	1081
CNTD1	-0.017674801	0.561580790437615	0.640929410422399	1081
DCTN1	-0.017680633	0.561451637581757	0.64081831382915	1081
ZNF157	-0.017719287	0.560596057283891	0.639950562360967	1081
LOC38764	-0.017724787	0.560474381891868	0.639882552550078	1081
GOLGA6A	-0.017725445	0.560459817139473	0.639882552550078	1081
RGS21	-0.017778807	0.559279855116099	0.63870139916745	1081
SRD5A1	-0.017802557	0.558755070474918	0.638174446916727	1081
PRNP	-0.017815158	0.558476729985378	0.637892709959485	1081
POLB	-0.017815536	0.558468377141429	0.637892709959485	1081
RPL13AP2	-0.017816773	0.558441061634469	0.637892709959485	1081
RBP3	-0.017846741	0.557779389072493	0.63727592351918	1081

AQP2	-0.017851329	0.557678134209208	0.637197323671062	1081
LOC40143	-0.017858666	0.557516220465687	0.637048454979085	1081
BRAP	-0.01789781	0.556652725838515	0.636386650134322	1081
POM121L	-0.017899511	0.556615233304428	0.636379902095216	1081
C19orf41	-0.017979676	0.554848955569803	0.634540573940033	1081
SDC2	-0.017982079	0.554796047425803	0.634516088077256	1081
MGAT4C	-0.01798987	0.554624555641809	0.634391986906896	1081
OR10G9	-0.017993831	0.554537359493263	0.634328266821406	1081
CPSF2	-0.017994986	0.554511950043374	0.634328266821406	1081
EGLN3	-0.017997046	0.554466607022574	0.634319370689406	1081
CLPS	-0.018000753	0.55438503126275	0.634275031246948	1081
OR11H4	-0.018001073	0.554377987491101	0.634275031246948	1081
LONRF3	-0.018027589	0.553794590902341	0.633694520518469	1081
FMO6P	-0.01806608	0.552948291028686	0.632798007315393	1081
SPIN2A	-0.018100607	0.552189670493054	0.632037551822874	1081
LOC91948	-0.018112175	0.551935629492342	0.631854478383999	1081
ECE1	-0.018136094	0.551410503170168	0.631325068847004	1081
SNORA18	-0.018158195	0.550925521940606	0.630949098322542	1081
OR2T2	-0.018166309	0.550747505232386	0.630781084365593	1081
DYNLT3	-0.018181317	0.55041833712922	0.630439925032303	1081
SHISA9	-0.018208405	0.549824466455613	0.629867152502038	1081
STAT6	-0.018213056	0.549722535418807	0.629822014218954	1081
DDX11L2	-0.018225521	0.54944938936188	0.629548926919122	1081
LOC28520	-0.018229008	0.549372992146101	0.629528949191308	1081
TADA2A	-0.018264313	0.548599762949388	0.628750197679662	1081
SEMA4C	-0.018290569	0.548025059039045	0.628234496918088	1081
ESF1	-0.018294937	0.54792948878287	0.628160684699005	1081
ZNF389	-0.018301722	0.547781034240286	0.628026232699935	1081
MED12L	-0.018318734	0.547408919721288	0.627671050588272	1081
ABCA5	-0.018324981	0.547272295562277	0.627550113507631	1081
SCARNA2	-0.018335773	0.547036335098814	0.627315248509456	1081
RASGEF1	-0.018345415	0.546825549823913	0.627109228122665	1081
THRA	-0.018352568	0.546669199329816	0.626965614602855	1081

C2orf76	-0.018375258	0.546173392985922	0.626468314166224	1081
SCIN	-0.018390847	0.545832904107647	0.626149071991831	1081
PLXNA2	-0.01839264	0.545793747004628	0.626139808280651	1081
C13orf38	-0.018395462	0.545732107325566	0.626104749819097	1081
ZNF839	-0.018421964	0.545153542799688	0.625512224166982	1081
UPF2	-0.018429682	0.54498510410615	0.625354575200409	1081
GFPT2	-0.018448238	0.544580225942233	0.624925585394833	1081
SLITRK2	-0.018453156	0.544472949788658	0.624838075281561	1081
GRIK5	-0.018462174	0.544276268263725	0.624647947597148	1081
SNORA21	-0.018466668	0.544178265275325	0.62457105480694	1081
FAM41C	-0.018482656	0.543829688246047	0.624242112234565	1081
OR5B2	-0.018503237	0.54338112068874	0.623798306147405	1081
UTP23	-0.018513064	0.5431670098989	0.623623584544246	1081
HYALP1	-0.018520564	0.543003617668817	0.623471523393464	1081
SH2D7	-0.01859034	0.54148476154654	0.621940277174015	1081
GPR162	-0.018602079	0.541229427576301	0.6216824505944	1081
TPSG1	-0.0186264	0.540700635186949	0.621110469056343	1081
SUSD3	-0.0186425	0.540350735207701	0.620779330525187	1081
GNASAS	-0.018662846	0.539908713170041	0.620448432790102	1081
KANK3	-0.018663115	0.539902871994791	0.620448432790102	1081
GRIA1	-0.018683308	0.539464347909493	0.620114652703951	1081
DNAJC5G	-0.01868599	0.539406111260921	0.620083092407627	1081
WNT5B	-0.018697716	0.539151554681057	0.619825832851104	1081
PGA4	-0.018714259	0.538792536416368	0.619448444590101	1081
OR1N1	-0.018717195	0.538728833221505	0.619410555633081	1081
CCDC108	-0.018793335	0.53707808448738	0.617688856588164	1081
SERPINE3	-0.018802958	0.536869617357397	0.617519609550346	1081
CST8	-0.018810624	0.536703606301421	0.617399163370382	1081
CCDC74A	-0.01882142	0.536469827632721	0.617235974508034	1081
OR2C3	-0.018863834	0.53555188798609	0.616250230392402	1081
LOC10013	-0.018908731	0.5345810724706	0.615358214226584	1081
ILDR1	-0.018913975	0.534467750941034	0.615302017442541	1081
CA2	-0.018924726	0.534235443609026	0.615086758387371	1081

LOC10013	-0.018949655	0.533696957660407	0.614607283603093	1081
C12orf71	-0.018959963	0.53347436371185	0.614456321153529	1081
AGAP4	-0.018975869	0.533131003687331	0.614131065260823	1081
CCDC37	-0.018988348	0.532861703186855	0.613855950746223	1081
ZNF232	-0.018989603	0.532834617154969	0.613855950746223	1081
GAPDHS	-0.018991338	0.532797181006656	0.613851827931317	1081
CENPL	-0.018999633	0.532618225854115	0.613680745815097	1081
POM121L	-0.019005435	0.532493053690306	0.613572813924478	1081
CLEC1A	-0.019020602	0.532165943133748	0.613264854666744	1081
LYZL4	-0.019026996	0.532028080374442	0.613176135885527	1081
LRRC8E	-0.01903873	0.531775114147157	0.612954721891313	1081
RNASE11	-0.019041002	0.531726148626816	0.612933352259511	1081
CXCL5	-0.019045318	0.531633114603957	0.612896251213043	1081
NPEPPS	-0.019045561	0.531627881385298	0.612896251213043	1081
C12orf40	-0.019054158	0.531442606624407	0.612746755405716	1081
GSTP1	-0.019070708	0.531086032591401	0.612475848783706	1081
ODF2	-0.019073877	0.53101776408351	0.612438923276626	1081
HESRG	-0.019092455	0.530617661920548	0.612110908743597	1081
LIF	-0.019098038	0.530497457211707	0.612007288714342	1081
ACTR6	-0.019104348	0.530361628438246	0.611885631114396	1081
LOC33978	-0.019105564	0.530335446703525	0.611885631114396	1081
LBH	-0.019109429	0.530252255609291	0.611829525703028	1081
MTMR4	-0.019111633	0.530204815836013	0.611809830164766	1081
DRAM2	-0.019124985	0.529917478519979	0.611548327496847	1081
HNRNPUI	-0.019125868	0.529898459926068	0.611548327496847	1081
SIDT1	-0.01914927	0.529395050274447	0.611050437619821	1081
LOC90110	-0.01918117	0.528709209851325	0.610328750928597	1081
RPL35A	-0.019239566	0.52745487775094	0.609055285583669	1081
ALAS2	-0.019240636	0.527431905756438	0.609055285583669	1081
OR8I2	-0.019241497	0.527413426476502	0.609055285583669	1081
EVX1	-0.019277157	0.526648224174813	0.608333054816606	1081
GJB5	-0.019294623	0.526273654970194	0.607970110068503	1081
FBXL12	-0.019320408	0.525720920098373	0.607366401065649	1081

C8orf45	-0.019354404	0.524992626232515	0.606629371082598	1081
EFNB1	-0.019356774	0.524941870563726	0.606605517266463	1081
SELV	-0.019378497	0.524476786165301	0.606207175242412	1081
C16orf78	-0.019381829	0.524405457753625	0.606159510353843	1081
OR13C8	-0.019419317	0.523603417395158	0.605371375311379	1081
NR1H4	-0.019436163	0.523243189781325	0.604989614770406	1081
PDE7A	-0.019439591	0.523169910224089	0.604939607476999	1081
C18orf54	-0.019543863	0.520943274001952	0.602503287103533	1081
SCO1	-0.01958006	0.520171469224396	0.601809903319055	1081
SLC36A3	-0.019581037	0.520150643595096	0.601809903319055	1081
AP3M2	-0.019583159	0.520105419898463	0.601809903319055	1081
MYO16	-0.019590309	0.519953047240426	0.601703477519439	1081
RWDD3	-0.019607685	0.519582828081518	0.60134413832599	1081
PPP1R3G	-0.019613386	0.519461396657119	0.60123814051466	1081
VENTXP7	-0.019615948	0.519406826746407	0.601234181829836	1081
RALGDS	-0.019618495	0.519352589670609	0.601234181829836	1081
HAP1	-0.019625693	0.519199310557185	0.601142026046008	1081
KRT9	-0.01964415	0.518806349226046	0.600721571741504	1081
MGC2889	-0.019649242	0.518697980778149	0.60063061578294	1081
NCS1	-0.019693373	0.517759171734185	0.599819340651283	1081
ABCD4	-0.019714605	0.517307810923751	0.599399853391174	1081
CRP	-0.019759296	0.516358397639947	0.598437459467077	1081
ZNF679	-0.019762433	0.516291792336374	0.598394692860955	1081
GGT7	-0.019764724	0.516243149882801	0.598372741909611	1081
LOXL4	-0.019810312	0.515275731148779	0.597388904465479	1081
MCCC2	-0.019821736	0.515033466722935	0.597142399259484	1081
CHRNE	-0.019841514	0.514614151284063	0.596724922443875	1081
KRTAP2-2	-0.019846072	0.514517546227758	0.596647246647374	1081
SFTA3	-0.019851738	0.514397456363736	0.596611017240339	1081
BPI	-0.01985228	0.514385965433941	0.596611017240339	1081
DLX6AS	-0.019858881	0.514246106886215	0.596538513372267	1081
HEBP1	-0.019862171	0.514176392647185	0.596491991354883	1081
DUSP7	-0.019872317	0.513961457927737	0.596345672730921	1081

RBPJ	-0.019903799	0.51329480292712	0.595812377122836	1081
ZNRF3	-0.019916869	0.51301817403554	0.59552559156079	1081
PHACTR3	-0.019923038	0.512887642949831	0.595408377065888	1081
INTS7	-0.019932862	0.512679786627926	0.595235681033921	1081
IDH1	-0.01994514	0.512420084139337	0.594968449278786	1081
ABCC6P1	-0.019958926	0.512128558132223	0.594698513002861	1081
PRSS23	-0.019959232	0.512122084221039	0.594698513002861	1081
B3GALT2	-0.019966727	0.511963649937669	0.594644126383438	1081
SHC1	-0.019971356	0.511865790942365	0.594564744192696	1081
KRT6C	-0.019973553	0.511819342466267	0.59454507288565	1081
NAT6	-0.019978731	0.511709911641763	0.594452232859888	1081
INTS1	-0.019982801	0.511623900661674	0.594386590474592	1081
OR9G4	-0.019996318	0.511338296119227	0.594157580626511	1081
FLJ43390	-0.020088129	0.509400608728538	0.592213484697392	1081
TAF3	-0.020097908	0.509194443111284	0.592007967708017	1081
S100A2	-0.020120417	0.508720076691702	0.591524729867494	1081
PPBP	-0.020127676	0.508567148309956	0.59138104379497	1081
AVPR1B	-0.020151663	0.508061974837291	0.590861822139316	1081
SIX1	-0.020152036	0.508054113188471	0.590861822139316	1081
CFI	-0.020152397	0.508046514695654	0.590861822139316	1081
TGM6	-0.020186396	0.507330925413451	0.590113834081296	1081
FRAT1	-0.020212663	0.506778452364684	0.589607387980743	1081
WNK2	-0.020262163	0.505738170028751	0.588465051419696	1081
ANXA5	-0.020264265	0.505694015799606	0.588447663371249	1081
RNASE13	-0.020322659	0.504468306480676	0.587292766646626	1081
FGF4	-0.020330936	0.504294689670969	0.587124575126954	1081
SLC26A1	-0.020354182	0.5038072749826	0.586658818180606	1081
KRTAP5-[illegible]	-0.020360004	0.503685237954121	0.586550616716907	1081
LOC10012[illegible]	-0.020366446	0.503550229043324	0.58642729587685	1081
AKR1C1	-0.020376297	0.503343802906391	0.58625467793416	1081
DEK	-0.020379471	0.503277316657288	0.586211132932123	1081
OR1L6	-0.020417865	0.502473265773046	0.585342273694444	1081
HECW1	-0.020439016	0.502030617906828	0.584894268563591	1081

LEMD1	-0.020442522	0.501957265521476	0.584842632384623	1081
SPP2	-0.020454731	0.501701865452754	0.584633409211871	1081
ASAP1	-0.020458358	0.501626000411794	0.584625732039085	1081
CCL21	-0.020467918	0.50142607118862	0.584426533446815	1081
NMUR2	-0.020487491	0.501016904324725	0.584051011956576	1081
ZAR1	-0.020494513	0.500870144890796	0.583936089570908	1081
GNB4	-0.020507041	0.50060837784333	0.583676106705284	1081
EFTUD1	-0.020519645	0.500345074767549	0.583402878462249	1081
SIAH1	-0.020594067	0.49879198330077	0.581861399246859	1081
NRAS	-0.020596694	0.498737209897924	0.581831196050132	1081
SNORA40	-0.020615639	0.498342263243699	0.581404117278295	1081
LOC10013	-0.020625503	0.498136708767362	0.581197961202277	1081
SNORA13	-0.020637283	0.497891278134874	0.581001657781811	1081
SGPL1	-0.020640244	0.497829595201029	0.580974232147186	1081
FAM47C	-0.020643378	0.497764316459855	0.580931704714894	1081
FIZ1	-0.020683373	0.496931648120157	0.580060728437859	1081
MORC1	-0.020685655	0.496884143423749	0.580038886786314	1081
EPT1	-0.020712289	0.496330089557231	0.579492851172506	1081
C4orf7	-0.020718578	0.496199287246064	0.579373711329737	1081
OR52R1	-0.02074983	0.495549665092427	0.578750949386004	1081
NUP107	-0.020789177	0.494732417166533	0.57809655126565	1081
CWH43	-0.020810678	0.494286133007722	0.57767559026748	1081
HAS2	-0.020821413	0.494063407032751	0.57744878943344	1081
C15orf44	-0.020823765	0.494014607278192	0.577425254634127	1081
ZDHHC14	-0.020841886	0.493638749751038	0.577052899305672	1081
KHSRP	-0.020907765	0.492273654315749	0.575567648606468	1081
F7	-0.020925833	0.491899626703722	0.575201576045906	1081
GGCX	-0.020936713	0.49167446375632	0.575023632648538	1081
DEFB123	-0.02094317	0.491540855184322	0.574900750562481	1081
F11R	-0.020948156	0.491437690959549	0.574813463646294	1081
ESR1	-0.020971004	0.490965155421163	0.574327452294253	1081
FAM124A	-0.020979341	0.490792808754264	0.574240427578596	1081
GPR137C	-0.020988161	0.490610493433896	0.574079251290999	1081

C2CD4A	-0.020995951	0.490449487062417	0.573957529735819	1081
USP1	-0.021009745	0.490164495295368	0.573690666844364	1081
NCRNA00	-0.021016206	0.490031025398592	0.573567776808706	1081
FANCF	-0.021020534	0.489941625922027	0.573496458698387	1081
NCRNA00	-0.021036516	0.489611616756644	0.573250077858345	1081
UBTFL1	-0.021045406	0.489428084877104	0.57309513891242	1081
CUL7	-0.021055267	0.489224558309625	0.572923423273305	1081
GLYCAM	-0.021063348	0.489057801334852	0.572794733016895	1081
RPS9	-0.021066153	0.488999933556726	0.572760257078914	1081
TBPL1	-0.021085544	0.488599944490956	0.572391595822884	1081
ELP2P	-0.021101838	0.488263982317713	0.572097826079819	1081
LOC28584	-0.02110866	0.488123354086875	0.571966319688232	1081
SNORA9	-0.021140893	0.487459213409358	0.571287791851272	1081
PAQR5	-0.021154513	0.487178717471036	0.571025499706407	1081
SLC46A2	-0.02116064	0.487052567384213	0.570910855926625	1081
HCG9	-0.021178135	0.486692455474719	0.570588344034116	1081
TPSD1	-0.021178963	0.486675413803982	0.570588344034116	1081
ZBTB2	-0.021195127	0.486342850614126	0.5703112361384	1081
C2CD4B	-0.021205701	0.486125354402944	0.570089373876314	1081
GOLGA6I	-0.021215134	0.48593135859169	0.569895046796856	1081
ZNF786	-0.021249073	0.485233784428504	0.569209490905038	1081
TNFAIP6	-0.021290723	0.48437844972954	0.568404722421016	1081
SNORA80	-0.021305506	0.484075050572108	0.568081783395964	1081
LOC10021	-0.021318218	0.483814252519369	0.567808803786933	1081
XRCC2	-0.021341795	0.483330730291458	0.56727438602432	1081
POTEA	-0.021386902	0.482406407889653	0.56635451290501	1081
MTSS1	-0.021389523	0.482352722977254	0.56632449028889	1081
GLIPR1L1	-0.021403826	0.482059854178772	0.566013624901286	1081
GRIK3	-0.021430533	0.481513243054028	0.565470693792899	1081
GABPB1	-0.021435667	0.481408204047582	0.565380299104444	1081
ST7OT4	-0.021468249	0.480741885886533	0.564696518436397	1081
MAS1L	-0.021487628	0.480345814935713	0.564264181111431	1081
FOXD4L5	-0.021488339	0.480331289931434	0.564264181111431	1081

RPL23AP1	-0.021497351	0.480147163104477	0.564129511299411	1081
TMEM27	-0.021637087	0.477297124374364	0.561239234795795	1081
C9orf171	-0.021639353	0.477250971969801	0.561217724028465	1081
PATE3	-0.021644678	0.477142563644032	0.561122997350205	1081
GRRP1	-0.021655311	0.476926099548305	0.560933925467107	1081
GADD45A	-0.021672112	0.476584207170305	0.560564538649255	1081
TTC31	-0.021686826	0.47628489176596	0.560399170376752	1081
CCDC33	-0.021691974	0.476180182057375	0.560399170376752	1081
LOC33858	-0.02170692	0.475876298089086	0.560124336839301	1081
RAB3D	-0.021713098	0.475750698630646	0.560009221918568	1081
SERPINA1	-0.021727553	0.475456925479556	0.559765018899594	1081
SNORA50	-0.021761709	0.474763160497037	0.559108141483269	1081
SH3GL2	-0.021783376	0.474323362805854	0.558632954466321	1081
ALLC	-0.021840546	0.473163989376794	0.557550805217333	1081
C13orf26	-0.021845452	0.473064551626916	0.557466241153801	1081
TRIM9	-0.02184659	0.473041490730058	0.557466241153801	1081
FAM157B	-0.021885471	0.472254014573445	0.556641338925871	1081
XAGE5	-0.021893692	0.472087590601591	0.55651029976412	1081
LCN9	-0.021900169	0.47195652086987	0.556388349304981	1081
PFKM	-0.021905824	0.471842085771578	0.556285996246764	1081
KIF24	-0.021913492	0.471686937097495	0.556135628457745	1081
FLJ42875	-0.021933128	0.471289764692885	0.555797465738097	1081
CECR4	-0.021975515	0.470433056771163	0.554884590939463	1081
TFRC	-0.022015422	0.469627255962219	0.554128804039061	1081
OR2T27	-0.022020844	0.46951783916496	0.554032150303311	1081
RBP7	-0.022021777	0.469499010689584	0.554032150303311	1081
BTBD12	-0.022025913	0.469415557310663	0.553976356298963	1081
SETD4	-0.022061043	0.468707013731751	0.553334668988872	1081
YTHDF2	-0.022115077	0.467618353148941	0.552146516862166	1081
CDHR3	-0.022141096	0.467094636560645	0.551625129177758	1081
THBS3	-0.022147894	0.466957858515111	0.551528263355237	1081
LMO4	-0.022166731	0.46657896727334	0.551145379132252	1081
SRI	-0.022168257	0.466548283463688	0.551141451379581	1081

NCOA7	-0.022195902	0.465992543258259	0.55054951521539	1081
GRIA3	-0.022207265	0.465764239940581	0.550312059448856	1081
SLC24A3	-0.022238638	0.465134170071988	0.549599850788914	1081
EML4	-0.02226729	0.46455917480264	0.549113687519754	1081
GABRQ	-0.022270941	0.464485945075278	0.549059345355092	1081
PTPRR	-0.022282246	0.464259191666476	0.548823508956116	1081
HELT	-0.022290323	0.464097225066798	0.548664237028793	1081
ARPP21	-0.02230745	0.463753902964465	0.548290532027651	1081
ADAM2	-0.022316398	0.463574575995036	0.548110683966428	1081
C19orf34	-0.022317937	0.463543737343294	0.548106391311889	1081
PRIMA1	-0.02232637	0.463374783033645	0.547938776955435	1081
EREG	-0.022392123	0.462058608135065	0.54663910388072	1081
OR4X2	-0.022393517	0.462030725469498	0.546638219567923	1081
ABHD10	-0.022411713	0.461666873085927	0.546336084022557	1081
KCNK16	-0.022412726	0.461646619281998	0.546336084022557	1081
DFFB	-0.022420199	0.461497227821036	0.546199497941059	1081
CDYL	-0.022446554	0.460970624937953	0.545704486065409	1081
EIF5A2	-0.022463563	0.460630943156495	0.545366461938736	1081
HOXA1	-0.022539277	0.459120567431953	0.543897902435559	1081
DPH5	-0.02254755	0.45895570190176	0.54373456920789	1081
EIF4A2	-0.022562855	0.458650779744882	0.543469205220907	1081
OR6B1	-0.022571523	0.458478146888981	0.54332856456724	1081
CATSPER	-0.022577397	0.458361183838409	0.543253871178722	1081
FAM118B	-0.022589445	0.45812131411797	0.543033472957135	1081
FAM89A	-0.02261609	0.45759109779398	0.542436899203232	1081
NBN	-0.022632711	0.457260520310403	0.542140732249607	1081
TXNRD2	-0.02264458	0.457024544353366	0.541892845556125	1081
CYP26C1	-0.022685496	0.456211568815803	0.541056284810688	1081
CCDC40	-0.0226964	0.455995065241016	0.540889746000154	1081
PCDH15	-0.022700239	0.45591884391867	0.540836480434672	1081
HOXC6	-0.022700951	0.455904708331119	0.540836480434672	1081
TTL	-0.022702196	0.455879987129564	0.540836480434672	1081
LTF	-0.022716348	0.455599102924445	0.540680054692957	1081

DIRAS1	-0.022720904	0.455508710838438	0.54062214906112	1081
CCDC27	-0.022721435	0.455498174277079	0.54062214906112	1081
CWC27	-0.022732644	0.455275780552773	0.540423699206624	1081
AMY2A	-0.022734352	0.455241903758967	0.540415330066257	1081
SNORA26	-0.022754469	0.454842969712253	0.540005399861701	1081
LOC44160	-0.022774387	0.45444819181366	0.539632114593939	1081
HNRNPR	-0.022804179	0.453858031011653	0.539122002165796	1081
ISM2	-0.022818676	0.45357103115053	0.538908193602326	1081
ELOVL4	-0.022821215	0.453520772603447	0.538880262126722	1081
TNFRSF10	-0.022824806	0.453449695729255	0.538827589244726	1081
C14orf128	-0.022866502	0.452624887070767	0.537879208945293	1081
SLC45A3	-0.022876531	0.45242662307529	0.537675318379548	1081
CAP1	-0.022895942	0.452043023417015	0.537251133140753	1081
SNORA27	-0.022900304	0.451956856284927	0.537180415945475	1081
HIBCH	-0.022926996	0.451429734387877	0.536585555773176	1081
CGN	-0.022928808	0.451393971881828	0.536585555773176	1081
CYP4V2	-0.02294423	0.451089586493581	0.536276171136807	1081
C1orf84	-0.022950832	0.450959309031029	0.536152932036714	1081
PRO1768	-0.022974275	0.450496912969164	0.535666409618924	1081
FAM174B	-0.02298874	0.450211726814009	0.53545372751613	1081
BCAM	-0.022988825	0.450210049078151	0.53545372751613	1081
SCARNA1	-0.022991644	0.450154494064623	0.535448883026207	1081
SEC62	-0.02306104	0.448787862222721	0.533980951182843	1081
DTX1	-0.023099799	0.448025584202046	0.533105457398123	1081
FAM160B	-0.023113356	0.447759136590198	0.532851359086103	1081
CAPRIN2	-0.023120125	0.447626143936321	0.532756036251753	1081
LOC72966	-0.023160883	0.446825751017913	0.531929130968907	1081
LOC25688	-0.023173514	0.446577880687468	0.531696891634104	1081
SLC7A6	-0.023233697	0.445397897559719	0.530511479119053	1081
SPATA3	-0.023235642	0.445359798044458	0.530497465208468	1081
PCGF2	-0.023239703	0.445280246969402	0.530434071030608	1081
TP53I11	-0.023253147	0.445016929487277	0.530214457329145	1081
MDC1	-0.02325324	0.445015107115498	0.530214457329145	1081

MMP14	-0.023281508	0.44446175676653	0.529678306321685	1081
GPR101	-0.023283126	0.444430104821442	0.529671919873865	1081
KIAA0182	-0.023286123	0.444371463468238	0.529633364783319	1081
VGLL2	-0.023291029	0.444275472782765	0.529550286918454	1081
IGLON5	-0.023292088	0.444254747462989	0.529550286918454	1081
METTL10	-0.023293492	0.44422729426927	0.529550286918454	1081
GRID2IP	-0.023325317	0.443604958781725	0.528844945831332	1081
SPINT3	-0.023338843	0.443340628930755	0.528561103734986	1081
PCBD2	-0.023365095	0.442827824432008	0.528043478346324	1081
OR9A4	-0.023366857	0.442793422501143	0.528043478346324	1081
PNRC2	-0.023370382	0.442724589438244	0.528014140917259	1081
IVD	-0.02338573	0.44242498542076	0.527688059875738	1081
DBX1	-0.023404925	0.44205044776916	0.527366236545643	1081
CHRM1	-0.023408674	0.441977315902497	0.527310217877158	1081
OR5H2	-0.023411634	0.441919575037822	0.527272556215169	1081
OR9A2	-0.023433783	0.441487687278587	0.526882077027674	1081
RPL10	-0.02345339	0.441105590101576	0.526488453169585	1081
POFUT2	-0.023462004	0.440937774578129	0.526350525531903	1081
RGS2	-0.023510481	0.439994045618903	0.525286242087993	1081
SNORA19	-0.023517548	0.439856558788054	0.525153225673285	1081
RRM2B	-0.023528818	0.439637357882012	0.524953741599973	1081
WDR1	-0.023534911	0.439518891756207	0.524843395046164	1081
FAM186B	-0.023534995	0.439517241356599	0.524843395046164	1081
KCNC2	-0.023544281	0.439336715894911	0.524688056776321	1081
OR4C11	-0.023544332	0.439335719554254	0.524688056776321	1081
ACTC1	-0.023586801	0.438510621164714	0.523825691613091	1081
LTC4S	-0.023587941	0.438488486934371	0.523825691613091	1081
SFXN2	-0.023592844	0.438393275303063	0.523747629639439	1081
PIRT	-0.023596944	0.438313684187075	0.523683599308975	1081
C12orf50	-0.023621351	0.437840014730438	0.523148700874536	1081
CRIP3	-0.023622709	0.437813679583516	0.523148263551215	1081
CCAR1	-0.023625592	0.437757741433374	0.523112451131529	1081
CBLC	-0.023653355	0.437219365027318	0.522562094836862	1081

KCNJ8	-0.023699936	0.436316924962404	0.521539825872296	1081
GTF3C1	-0.023709776	0.436126429274806	0.521348618427544	1081
SENP2	-0.02371838	0.435959897341745	0.521189498126204	1081
BMP6	-0.023720403	0.435920748002469	0.521189498126204	1081
RPL21	-0.023748831	0.435370795322592	0.520630694038562	1081
CYB5A	-0.023749004	0.435367464222039	0.520630694038562	1081
C4orf35	-0.023756326	0.435225872888024	0.520519188395229	1081
PCNT	-0.023764648	0.435064996922034	0.520402615420999	1081
C6orf168	-0.023812453	0.434141479246442	0.519438148091677	1081
OVOL2	-0.02381694	0.434054860144915	0.519365357425714	1081
AKAP5	-0.023844049	0.433531724849915	0.518924337320353	1081
NPTN	-0.023889558	0.432654327966401	0.517997232668676	1081
TMCO4	-0.023931372	0.431849085392333	0.517156094711006	1081
OR10H3	-0.023932316	0.431830917584937	0.517156094711006	1081
LRRC30	-0.023935839	0.431763110423416	0.517114617091541	1081
ZCCHC7	-0.023965877	0.431185243515594	0.516514641768546	1081
ZBTB42	-0.023998757	0.430553223744822	0.515818893389202	1081
UCMA	-0.02400219	0.430487266278502	0.515770547114335	1081
TAS2R7	-0.024012988	0.430279844915873	0.515583361636346	1081
RBM22	-0.024015084	0.430239586950135	0.515565789013769	1081
EPHB3	-0.024020428	0.430136955487389	0.515475547062424	1081
CYP4F12	-0.024071118	0.429164181580086	0.514399502465094	1081
SLC27A1	-0.02407687	0.429053872652785	0.514328489413291	1081
LAD1	-0.024102837	0.428556131345625	0.513854131656308	1081
RASL10B	-0.024116388	0.428296507770188	0.513573401728003	1081
OFD1	-0.024136559	0.427910222233259	0.513232402172482	1081
MORF4L1	-0.024142212	0.427802004340693	0.513133157335115	1081
SLC10A4	-0.024162269	0.427418166972733	0.512703285320097	1081
PIGB	-0.024169796	0.427274168799734	0.512591599003731	1081
ZNF337	-0.024174562	0.427182996026885	0.512512742240581	1081
RTP1	-0.024214044	0.426428238605391	0.511668167651772	1081
C16orf71	-0.024242013	0.425894038723326	0.51113906090931	1081
CFHR2	-0.024249858	0.425744252457934	0.511030087932139	1081

TCTN3	-0.024265702	0.425441876360114	0.510728001863685	1081
UBAP1	-0.024298419	0.424817868673426	0.510039690352593	1081
BAG3	-0.024301016	0.424768369471301	0.51001065699126	1081
PITPNB	-0.024321058	0.424386398433984	0.509582404270883	1081
PRH1	-0.024339329	0.424038345364454	0.509225184343781	1081
NOX3	-0.024345761	0.423915865653565	0.509108448407719	1081
PLA2G4A	-0.024376855	0.423324050978661	0.508488641521979	1081
OVGP1	-0.0243833	0.423201430524835	0.508371664753894	1081
SCGB1D2	-0.024383349	0.42320050995878	0.508371664753894	1081
PNRC1	-0.024388511	0.423102317695938	0.508313226979047	1081
TRIM6	-0.024483404	0.42129972492638	0.506358985659681	1081
KDM4D	-0.024490468	0.421165720877257	0.506258335644868	1081
AP2A2	-0.02449704	0.421041067421005	0.506138698126038	1081
MLLT3	-0.024503177	0.420924680212165	0.506040719877167	1081
RIC3	-0.024517971	0.420644222322607	0.505837660869979	1081
PLGLA	-0.024524356	0.42052319043028	0.505727399630947	1081
TTYH2	-0.024547465	0.420085371756445	0.50526119852132	1081
PARP8	-0.02458335	0.419406036414996	0.504504365174664	1081
RSPO1	-0.02459468	0.419191674892456	0.50433685443792	1081
ARR3	-0.024596383	0.41915945706295	0.504328212538562	1081
OMG	-0.024601069	0.419070831203298	0.504251696009463	1081
HAUS4	-0.02460899	0.418921020160078	0.504105149324388	1081
PON2	-0.024609731	0.41890701792789	0.504105149324388	1081
C15orf26	-0.024657423	0.41800576976255	0.503168931822726	1081
MAEL	-0.024710391	0.417006184893092	0.502127291970792	1081
CSF3	-0.024715128	0.416916859877561	0.502049742497069	1081
ST8SIA1	-0.024723145	0.416765699110637	0.501957730992692	1081
TLE1	-0.024736345	0.416516899754615	0.501688068493675	1081
C6orf57	-0.024749752	0.416264282254845	0.501443758059191	1081
HLTF	-0.024758276	0.416103724504021	0.501280321141819	1081
MYCNOS	-0.02476094	0.416053535984904	0.501249834484534	1081
CACNG7	-0.024777536	0.415741067754242	0.500933298038711	1081
BAT1	-0.0247863	0.415576100846379	0.500764478499241	1081

GAL3ST1	-0.024792837	0.415453076362332	0.500646181929723	1081
NIPSNAP:	-0.024795827	0.415396817630802	0.500608332805247	1081
MAL2	-0.02480373	0.415248134106324	0.500459088338133	1081
BCAP29	-0.024806223	0.415201237840325	0.500432507855292	1081
EFNA4	-0.024816445	0.415008981112245	0.500230714085214	1081
DZIP1L	-0.024859621	0.414197529076959	0.499372148139047	1081
INTS2	-0.024861293	0.414166113046817	0.499364157728522	1081
LOC64414	-0.024916586	0.413128354140983	0.498202376184981	1081
HSD17B7I	-0.0249354	0.412775591763691	0.497896191610056	1081
TTLL12	-0.024935908	0.412766060513518	0.497896191610056	1081
PMP22	-0.024964214	0.412235657163326	0.49730446814511	1081
AKR7A3	-0.024986887	0.411811120372131	0.496881589596106	1081
C4orf37	-0.024987134	0.411806493210842	0.496881589596106	1081
ACIN1	-0.025004893	0.411474126306725	0.496564205023002	1081
LPPR3	-0.025028078	0.411040463392183	0.496070580819286	1081
FUT2	-0.025034447	0.410921393769675	0.496014346883761	1081
LOC10027	-0.025037067	0.410872412678996	0.496014346883761	1081
UOX	-0.02504411	0.410740762057999	0.495916850563883	1081
AIG1	-0.025057348	0.410493379065451	0.495647873487983	1081
DMP1	-0.025070707	0.410243820399848	0.495405932259617	1081
SLC6A5	-0.025071334	0.410232126162436	0.495405932259617	1081
NR2E3	-0.025074778	0.410167793535914	0.49537351323627	1081
RPS11	-0.02510393	0.409623606551439	0.494834945669149	1081
EVL	-0.025116468	0.40938967079014	0.494582004082001	1081
MAEA	-0.025149222	0.408778953230049	0.493873816289993	1081
KIAA1211	-0.025154963	0.408671966207779	0.493803788343073	1081
MAGEC3	-0.025166366	0.408459504802088	0.493665510213466	1081
PPAPDC1	-0.025171098	0.40837135971395	0.493588590714437	1081
C12orf77	-0.025187958	0.408057389906749	0.493238696728157	1081
OR13C2	-0.025199888	0.40783532584181	0.493029443055885	1081
SERPINB:	-0.025210888	0.407630616675919	0.492811544916064	1081
NUDT9P1	-0.025213256	0.407586564894145	0.492811544916064	1081
LANCL3	-0.025217454	0.407508456487832	0.492752572385796	1081

EBF4	-0.025229625	0.407282086126809	0.492537977249644	1081
NUP88	-0.025262847	0.406664537606251	0.491879742424373	1081
LOC28613	-0.025270598	0.406520545999467	0.491735102627553	1081
C20orf54	-0.025281474	0.406318535780858	0.491549778002966	1081
IGFALS	-0.025288411	0.406189728273799	0.491423462619403	1081
ACSM1	-0.025308187	0.405822639423675	0.491126821530094	1081
SNORA55	-0.02531181	0.405755414505931	0.49107496697038	1081
C10orf55	-0.025312911	0.405734980478761	0.49107496697038	1081
NUP210L	-0.025342585	0.405184623870107	0.490443084051151	1081
CFL2	-0.025346509	0.405111872604909	0.490393827123797	1081
RNF44	-0.025351135	0.405026115108682	0.490339608705793	1081
TPRG1L	-0.025386161	0.404377219427494	0.48964230843102	1081
AMACR	-0.025396607	0.404183808812606	0.489466956929964	1081
POTEH	-0.025402361	0.404077303220291	0.489367396499504	1081
DMWD	-0.025409638	0.403942609566695	0.489292518172153	1081
COX11	-0.025412207	0.403895069541398	0.489292518172153	1081
C7orf28B	-0.02541834	0.403781598337265	0.489185728801071	1081
BZW1	-0.025425412	0.403650761946813	0.489065234964025	1081
JAZF1	-0.025426785	0.403625369733501	0.489065234964025	1081
SPACA5	-0.0254305	0.4035566604462	0.489030854468762	1081
PSORS1C2	-0.025467912	0.402865085406621	0.488251542865864	1081
C20orf26	-0.025483642	0.402574514191163	0.487947005792554	1081
LOC65434	-0.025501404	0.402246564150207	0.487707314764124	1081
GPX2	-0.025510841	0.402072374788558	0.48755480588187	1081
REM1	-0.025516501	0.401967947054894	0.487479694435498	1081
SNX31	-0.025518323	0.401934315107264	0.48747542310872	1081
UBA6	-0.025536268	0.401603314850541	0.487205688336704	1081
AP4S1	-0.025586186	0.400683385757606	0.486309603933424	1081
ZNF57	-0.025597241	0.400479819614674	0.486091821783311	1081
CLEC17A	-0.025617179	0.400112847714985	0.485763473586354	1081
SFRS15	-0.025619442	0.400071206369996	0.485742192160293	1081
OR13H1	-0.025643462	0.399629407540881	0.485293532757312	1081
PCA3	-0.025656696	0.399386130989711	0.485056587013187	1081

CDC42	-0.025664827	0.399236693483662	0.484936535073918	1081
PCDH8	-0.025683164	0.398899804256985	0.484627567786438	1081
SNORA49	-0.025712179	0.398367117555334	0.484052450880765	1081
NAA50	-0.025724184	0.398146828882293	0.483901530487711	1081
LOC73010	-0.025763758	0.397421208277852	0.483165373612391	1081
DEFA6	-0.025784187	0.397046927347399	0.482768611263481	1081
TRA2A	-0.025803516	0.396693015546771	0.482454769270085	1081
JAG2	-0.025823059	0.396335354928598	0.482060862124624	1081
TMEM71	-0.025840812	0.396010643112888	0.481799396395044	1081
FBLL1	-0.025846215	0.395911838443596	0.481708279606586	1081
SNORA53	-0.025867911	0.395515278444466	0.481283919315238	1081
ANO8	-0.025869789	0.395480962775437	0.481271233243396	1081
ALG11	-0.025886308	0.395179198682327	0.480933060922823	1081
TEKT3	-0.025890259	0.395107033454881	0.480874286427536	1081
GLDN	-0.025913025	0.394691433342269	0.480484585731207	1081
CDC42EP	-0.025924127	0.394488845724966	0.480296010947023	1081
RPS10	-0.025949122	0.394033004110529	0.479799012802624	1081
ACSL4	-0.025950197	0.394013398389204	0.479799012802624	1081
REG1A	-0.026037984	0.392414936541221	0.477973208575059	1081
RPRM	-0.026056475	0.392078749328698	0.477592599179261	1081
CHST14	-0.026072026	0.391796136634647	0.47727720702092	1081
TMCC3	-0.026074751	0.391746625043604	0.477245752388933	1081
TULP2	-0.026115353	0.391009416542383	0.476520550287133	1081
RBP2	-0.026177897	0.389875455083395	0.475381538	1081
HAS2AS	-0.026182861	0.389785534466849	0.47532432278878	1081
DHX40P1	-0.026185675	0.389734568787781	0.475311955940782	1081
DEFB125	-0.026189502	0.389665280806259	0.475256225818374	1081
XDH	-0.026200107	0.389473250055001	0.475079541166092	1081
BCYRN1	-0.026205265	0.389379884468055	0.475023180076846	1081
SOX15	-0.026210918	0.389277576027428	0.474927129909916	1081
OR3A2	-0.026215338	0.389197586476694	0.474858299289746	1081
PAGE3	-0.026236301	0.388818367807442	0.474453087375427	1081
C1orf70	-0.026247398	0.388617704170882	0.474236954841435	1081

CHRND	-0.026250031	0.3885700972256	0.474207585182632	1081
TOMM7	-0.026269593	0.388216568683521	0.473862262247305	1081
PAR4	-0.026271536	0.388181469883291	0.473848131297273	1081
RAB12	-0.026295322	0.387751866904584	0.473381089557775	1081
LOC10013	-0.026303147	0.387610616587265	0.47323732552427	1081
RGL3	-0.026304925	0.38757850734541	0.473226803471319	1081
C10orf71	-0.026322425	0.387262732870104	0.47289857269008	1081
GLIS1	-0.026351699	0.386734822970447	0.472339821070671	1081
CS	-0.026359908	0.386586855618354	0.472187728517052	1081
POLR3G	-0.026399789	0.385868551223581	0.471453297210034	1081
UBE2D1	-0.026479964	0.384426952293353	0.469891447967332	1081
REP15	-0.026487527	0.384291142382904	0.469753948015995	1081
KRTAP11	-0.026491268	0.384223977277809	0.469700347245674	1081
C22orf31	-0.026510121	0.383885566086928	0.46932505860018	1081
UBE2CBP	-0.026517923	0.383745576053612	0.469229416060698	1081
CYP4F2	-0.026525578	0.383608267699779	0.469089993493507	1081
LOC64785	-0.026535746	0.383425907617563	0.468895459779991	1081
OR51D1	-0.026539724	0.383354587235147	0.468836702273679	1081
LCE1B	-0.026542747	0.383300383465348	0.468798872254838	1081
TAB1	-0.026545067	0.383258791253013	0.468776463253913	1081
OR5E1P	-0.026547592	0.383213526985425	0.468749559847069	1081
PRSS8	-0.026574187	0.38273698263384	0.468223508541854	1081
ZNF354A	-0.026579814	0.382636204639919	0.468128649488107	1081
ALDH2	-0.026590403	0.382446602942139	0.467925102719064	1081
LOC10013	-0.026611364	0.382071445348699	0.467579689359671	1081
PIGR	-0.026612514	0.382050858707447	0.467579689359671	1081
GPBP1	-0.026616265	0.381983753007092	0.467529175790015	1081
UBAP2L	-0.026627529	0.381782266436381	0.467310958643875	1081
AKAP8	-0.026645408	0.381462601267828	0.467004807203913	1081
SERPINB1	-0.026661864	0.381168513079884	0.466673130910068	1081
OR5J2	-0.026689129	0.380681561215495	0.466218618194793	1081
THNSL1	-0.026693054	0.38061150514777	0.4661611607005	1081
TMEM17	-0.026696865	0.380543477346092	0.466106180527575	1081

FANCC	-0.026758062	0.379452151788371	0.464882539547329	1081
ALB	-0.026772141	0.379201339696201	0.464699274108759	1081
KBTBD13	-0.026792744	0.378834513784556	0.464323514248594	1081
DFFA	-0.026808407	0.378555775736931	0.464010315167227	1081
MYOZ1	-0.026808431	0.37855535808138	0.464010315167227	1081
C14orf19	-0.026811762	0.378496089375024	0.464010315167227	1081
GAP43	-0.026840584	0.377983571662292	0.463421523409218	1081
LIPT1	-0.026851491	0.377789740776588	0.463240265867938	1081
ASB9	-0.026857146	0.377689256387808	0.463145244091331	1081
RFPL1	-0.026859222	0.377652376478087	0.463145244091331	1081
ZNF275	-0.026890927	0.377089377313969	0.462522254383214	1081
TMEM50I	-0.026894537	0.377025305003974	0.46249998595208	1081
RIOK2	-0.026917534	0.376617315157325	0.462112060777458	1081
FXR1	-0.026929166	0.376411052945122	0.461923792813055	1081
ASB17	-0.026930485	0.376387662865309	0.461923792813055	1081
TRIM46	-0.026943405	0.376158666471901	0.461689900449427	1081
TMEM127	-0.026976187	0.375577968106044	0.461033341508516	1081
TMEM91	-0.026977967	0.375546459114145	0.461022755566999	1081
NUDT6	-0.027032561	0.374580681294538	0.460145607944294	1081
MAFF	-0.02705392	0.374203247120633	0.459738026056301	1081
COG2	-0.027055511	0.37417515864721	0.459731554705296	1081
ACCS	-0.027057038	0.374148169002902	0.459726432727262	1081
EPB41	-0.027070108	0.373917354103066	0.459470848993917	1081
SBDS	-0.027083927	0.373673400770027	0.459255119182002	1081
ANKRD49	-0.027097529	0.373433379173325	0.459016134202614	1081
GSTM3	-0.027117068	0.373088757287774	0.458676493077152	1081
XPO1	-0.027133004	0.372807837731879	0.458369280689537	1081
GJB4	-0.027144263	0.372609432186974	0.458171092681841	1081
ZFATAS	-0.027149609	0.372515263816395	0.458083261480972	1081
ALDH4A1	-0.027165241	0.37223994290225	0.457772642071048	1081
AADACL2	-0.027170234	0.372152037191601	0.457692477974652	1081
SPRYD5	-0.027173639	0.372092089467899	0.457646690416437	1081
IGFBP5	-0.027178915	0.371999223818696	0.457560408097914	1081

FERD3L	-0.027191561	0.371776683354709	0.457342530909906	1081
STK35	-0.027223474	0.371215464706141	0.456791615448367	1081
UGP2	-0.027287776	0.370086262404595	0.455457740615833	1081
FABP9	-0.027296973	0.369924915836683	0.455286988607648	1081
DIRAS2	-0.027335747	0.369245212448177	0.454533749145648	1081
SYT3	-0.02735175	0.368964915515818	0.454244223129387	1081
SLC46A1	-0.027379566	0.368478014199953	0.453783445167994	1081
BRD7P3	-0.027384584	0.368390225836642	0.453703068990595	1081
UGT2B4	-0.027417743	0.367810396751314	0.453044356010713	1081
FBXL19	-0.027479706	0.366728434078783	0.451877442321676	1081
SMC1A	-0.027482595	0.366678046891992	0.451842993310446	1081
SRR	-0.027482623	0.36667755089991	0.451842993310446	1081
PRLHR	-0.027513589	0.36613763641361	0.45128748687911	1081
TRIM39	-0.027522226	0.365987131475544	0.451157187833486	1081
C1QL2	-0.027560727	0.365316683311382	0.450358275828149	1081
SCUBE1	-0.027571394	0.365131071987705	0.450184555064099	1081
ICA1L	-0.02757844	0.365008506304568	0.450060984178328	1081
SNORA71	-0.027581107	0.364962109457278	0.450031321235041	1081
FGD3	-0.027592062	0.364771602885289	0.449823943445406	1081
TRAPPC6	-0.027619492	0.36429486590961	0.449318562036925	1081
SEZ6L	-0.027630533	0.3641030726203	0.449137003118972	1081
LOC1001(	-0.027643149	0.363884011929773	0.448949254062058	1081
OR7E156I	-0.02765049	0.363756575708853	0.448847005859049	1081
LOC72827	-0.027650739	0.363752258625517	0.448847005859049	1081
DLK1	-0.027656138	0.36365855597154	0.448781034675734	1081
E2F3	-0.027663158	0.36353673766577	0.448685485278149	1081
RNF222	-0.027695584	0.362974390727178	0.448073969066671	1081
CXCL6	-0.027729013	0.362395235334694	0.447441285598027	1081
ZNF174	-0.027769535	0.361693959880294	0.446630184000768	1081
CCDC55	-0.027821806	0.360790608270227	0.445569323418571	1081
C4orf21	-0.027825366	0.360729144274827	0.445520729042756	1081
LOC25735	-0.027860907	0.360115796377706	0.444872307477088	1081
FAM9C	-0.02787228	0.359919652808786	0.44468453884287	1081

GRM8	-0.027874418	0.359882801952301	0.444666280994179	1081
NOX4	-0.027897136	0.359491237626824	0.44420971489863	1081
FLJ26850	-0.027920114	0.35909548412041	0.443829592466142	1081
DDX42	-0.027971868	0.358205094785022	0.442811183690408	1081
PNMA1	-0.028003234	0.357666156704031	0.442225785724097	1081
DUXA	-0.028023484	0.357318476014515	0.44185014420922	1081
C7orf54	-0.028035926	0.357104963956898	0.441634647549783	1081
RNLS	-0.028047533	0.356905850658151	0.441421191153515	1081
CCKBR	-0.028062979	0.356640982029189	0.441120684163936	1081
B3GNT3	-0.028084402	0.356273830324812	0.440774814953837	1081
COL7A1	-0.028100005	0.3560065903034	0.440498296275535	1081
MRC2	-0.028106218	0.355900197954805	0.44039370318179	1081
PYGO2	-0.028143526	0.35526180258445	0.439657759740984	1081
PHF15	-0.028148425	0.355178037561749	0.439581100121732	1081
GRM2	-0.028232883	0.3537357238058	0.437957479937793	1081
SCARNA1	-0.028263811	0.353208485111065	0.437478752799752	1081
LOC28545	-0.028305706	0.352495075369358	0.436689828012774	1081
IFLTD1	-0.028307342	0.35246723661743	0.436682194443919	1081
GPR62	-0.028317597	0.352292753567403	0.43651971463989	1081
ABHD12E	-0.028363388	0.351514334793683	0.43563557517187	1081
CDC42BP	-0.028426472	0.350443715998839	0.434469115563826	1081
CPXM2	-0.028442004	0.350180438003394	0.434196154587208	1081
TRAPPC2	-0.028446665	0.350101456492829	0.434124944050722	1081
RINT1	-0.028463916	0.34980922153755	0.433815979307679	1081
SNORA64	-0.028466272	0.349769328299198	0.433815979307679	1081
ZNF572	-0.028478699	0.349558935363114	0.433585663006584	1081
ABHD14E	-0.02848473	0.349456844480241	0.433485722417147	1081
HPVC1	-0.028493611	0.349306557875437	0.433352667101902	1081
SNORA1	-0.028517604	0.348900744675508	0.432955864327221	1081
GJB2	-0.02852332	0.348804106000845	0.432862607982938	1081
PLA2G4E	-0.028590693	0.347666383962889	0.431823127500611	1081
TCEAL1	-0.028594371	0.347604350332628	0.431772699312541	1081
ALKBH1	-0.028598886	0.347528184457982	0.431704709619908	1081

CARD6	-0.02860022	0.347505688777182	0.431703385570815	1081
PRH2	-0.028606872	0.347393529424336	0.43161728446116	1081
ADAMTS4	-0.0286097	0.347345836718408	0.431584646931001	1081
GPRC5A	-0.028615761	0.347243666911239	0.43148431223334	1081
ATP5A1	-0.028618884	0.34719102569852	0.431445513089673	1081
CLDN17	-0.028661297	0.346476626747199	0.430690586561911	1081
C11orf2	-0.028663255	0.346443669265615	0.430676193604185	1081
FLJ37453	-0.02866771	0.346368687206991	0.430609553430315	1081
FAM109B	-0.028737552	0.345194562921843	0.429388358756439	1081
SLC30A1(	-0.028747211	0.345032376039629	0.429239623004898	1081
FAM81B	-0.028772621	0.344605948390267	0.428735599698736	1081
MON1B	-0.028779535	0.344489993259523	0.428617807066462	1081
ESPN	-0.028780016	0.344481921573793	0.428617807066462	1081
TPM2	-0.028784132	0.344412901522855	0.428574828660072	1081
DAP3	-0.028805873	0.344048453787059	0.428193501352449	1081
ZNF433	-0.028862129	0.343106549098518	0.427185060792425	1081
C14orf165	-0.028881882	0.342776229698237	0.426828232616577	1081
SLC39A6	-0.028900767	0.342460589588035	0.426487918969521	1081
GUCY2C	-0.02891399	0.342239713428842	0.426291907934461	1081
ZNF705A	-0.028925378	0.34204955389929	0.426107739985232	1081
KIAA1383	-0.028936363	0.341866181722457	0.425905641978905	1081
TM7SF3	-0.028944151	0.341736221139676	0.425770064621113	1081
BIK	-0.028951752	0.341609412079472	0.425638397225444	1081
KLK15	-0.028955439	0.341547894285031	0.425588070165891	1081
HIPK4	-0.028966969	0.341355613529711	0.425427421093543	1081
HEATR4	-0.02897174	0.341276083083706	0.425354618184821	1081
LOC10013	-0.028986641	0.341027706880804	0.425071349778109	1081
SCRN1	-0.02899962	0.340811478489535	0.424828119186417	1081
LRRC27	-0.029018893	0.340490550418358	0.424454340233776	1081
LRRK2	-0.029048338	0.340000611333134	0.423922282311443	1081
KCNN3	-0.029056143	0.339870820313931	0.423812917067418	1081
FOXD4	-0.029058737	0.339827696452175	0.423785374831563	1081
C3orf50	-0.029063015	0.339756571005371	0.423749140843437	1081

CRAT	-0.029064678	0.339728932760956	0.423740904617018	1081
MSGN1	-0.029068912	0.339658549589132	0.423705584337919	1081
C7orf4	-0.029082755	0.339428521184889	0.423444857522268	1081
SLC1A2	-0.029094692	0.339230247635428	0.4232237157748	1081
LINS1	-0.029134335	0.338572289560469	0.422455173316547	1081
PDE6A	-0.029135511	0.33855278783279	0.422455173316547	1081
VEGFA	-0.029179714	0.337820151093838	0.421673396355745	1081
NCOA3	-0.029183481	0.337757761032089	0.421621644317229	1081
ZNF682	-0.029190085	0.337648406621843	0.421511256825744	1081
GLB1L	-0.029212126	0.337283577125391	0.42110800503198	1081
NRG2	-0.029243393	0.336766496978653	0.420540607589571	1081
F2	-0.029246846	0.336709416344463	0.420495393482067	1081
ZNF326	-0.029252604	0.336614267857574	0.420402630253616	1081
CA6	-0.029298855	0.335850518573977	0.419526799570457	1081
SLC25A23	-0.029317035	0.335550620210191	0.419178174633157	1081
RTN4	-0.029318628	0.335524352541281	0.419171353919014	1081
ADAM32	-0.029319498	0.335510005654209	0.419171353919014	1081
MCART3P	-0.029320754	0.335489297840088	0.419171353919014	1081
BMP2	-0.029323342	0.335446621238756	0.419152225831456	1081
SEC14L1	-0.029328626	0.335359503135865	0.419069362364121	1081
LOC28385	-0.029443181	0.333474454324134	0.416868934679471	1081
BCR	-0.029500069	0.332540860757511	0.415753468625337	1081
SEC14L5	-0.029521009	0.33219765675784	0.415401725349887	1081
CD151	-0.029545203	0.331801379424336	0.414957709740703	1081
C3orf30	-0.02955887	0.331577666512103	0.414729423313444	1081
UBE2J1	-0.029564826	0.331480213589374	0.414633275551564	1081
OR1B1	-0.029588538	0.331092369826271	0.414199577106765	1081
UBE2U	-0.029592303	0.331030813790757	0.414148288648292	1081
RBM8A	-0.029594781	0.330990314253003	0.414123338961974	1081
KRT222	-0.029605077	0.330822041934002	0.41396422353938	1081
P704P	-0.02964043	0.330244651112569	0.413370106664764	1081
SMAGP	-0.029671231	0.329742134763927	0.412818053869822	1081
MYH13	-0.029681445	0.329575600669407	0.412635206680249	1081

MYCBP	-0.029684047	0.329533193017854	0.412607755195766	1081
TMEM119	-0.029690055	0.329435278810249	0.412522170202358	1081
EPHA2	-0.029731972	0.328752617672454	0.4117839633782	1081
LOC28609	-0.029732947	0.328736747301746	0.4117839633782	1081
ASTN2	-0.029739742	0.328626169070439	0.411676772335302	1081
GPR34	-0.029741794	0.328592782416559	0.411660547374477	1081
SLC41A3	-0.029776112	0.328034737034335	0.411038115168046	1081
C6orf134	-0.029779964	0.327972137968728	0.411010806804356	1081
DNAJB8	-0.029801638	0.327620058511805	0.410646221921373	1081
ACSM2A	-0.029823761	0.327260940049654	0.410272676414232	1081
IFT74	-0.029870714	0.326499595390583	0.409343686155296	1081
PGLYRP1	-0.029873127	0.326460508647307	0.409320156015435	1081
C7orf51	-0.029885451	0.326260886627576	0.409146262519455	1081
FAM27L	-0.029903919	0.32596188490113	0.40884765402735	1081
CCL28	-0.029905971	0.32592867621363	0.408831455935465	1081
LY6G6D	-0.029945386	0.325291166529594	0.408108024521309	1081
HP	-0.029952001	0.325184262074966	0.408024724976654	1081
KLF2	-0.029964025	0.324989985094312	0.407806356654099	1081
C7orf71	-0.029997453	0.3244502872456	0.407179855255583	1081
ZNF526	-0.030019446	0.324095525202843	0.406759974781089	1081
VMA21	-0.030026444	0.323982694251599	0.406694377847611	1081
ETS2	-0.030086601	0.323013817254714	0.405629806046392	1081
ZNF252	-0.030113523	0.322580823467101	0.405111320288308	1081
TBC1D25	-0.030125952	0.322381055965979	0.404910927890432	1081
CFHR3	-0.030134468	0.322244231287903	0.404764312214416	1081
SLC15A4	-0.030143115	0.322105343553777	0.404615086730114	1081
NPHS2	-0.03014435	0.322085505780294	0.404615086730114	1081
LOC28569	-0.030153152	0.321944162274336	0.404488284209585	1081
CNOT1	-0.030155246	0.321910552222266	0.404471282636908	1081
CDK20	-0.030191515	0.321328626184994	0.403840861843952	1081
CHMP7	-0.030197926	0.32122582575102	0.403736851744092	1081
SIAH3	-0.030204935	0.321113474162258	0.403620823422892	1081
GATA2	-0.030220108	0.320870342667114	0.403340387684326	1081

TNPO2	-0.030230901	0.320697461483559	0.403198549683973	1081
WDR88	-0.030248778	0.32041125549731	0.402863858569049	1081
CHAD	-0.030248812	0.320410709741862	0.402863858569049	1081
GGPS1	-0.030274145	0.320005407332634	0.40247917404732	1081
MLH1	-0.030308613	0.319454507968371	0.401936859847781	1081
MYOZ2	-0.030312524	0.319392024924956	0.401883344291894	1081
EDC3	-0.030315802	0.319339669048061	0.401842565617664	1081
NPR3	-0.030336252	0.319013190151536	0.401456816317009	1081
FAM105B	-0.030362507	0.31859434086119	0.400979819858096	1081
AVIL	-0.030374638	0.318400950038278	0.400786499532687	1081
PER4	-0.030382131	0.318281530248438	0.400661215200574	1081
ZC3H10	-0.03043396	0.317456286525585	0.399856614536745	1081
DOK4	-0.030439392	0.317369876411987	0.399788404222078	1081
MORC4	-0.030469537	0.316890632790309	0.399209667785678	1081
CCNO	-0.030469976	0.316883666354111	0.399209667785678	1081
RCAN1	-0.030479983	0.316724674777998	0.399050508061965	1081
NAT8L	-0.030480372	0.316718494893005	0.399050508061965	1081
SLC6A2	-0.030545462	0.315685673204425	0.397903620599432	1081
HOXD3	-0.030546385	0.31567104975474	0.397903620599432	1081
C17orf78	-0.030579516	0.315146207256914	0.39733510734701	1081
LAMA1	-0.030583989	0.31507539520389	0.397295558111064	1081
MDM2	-0.030636269	0.314248496589348	0.39632730005587	1081
TMEM88	-0.030637824	0.314223922585056	0.396321119488853	1081
SRRM3	-0.030650406	0.31402514044992	0.396138953714555	1081
LOC72767	-0.030676409	0.313614571886808	0.395725997535844	1081
ZNF212	-0.030697018	0.313289435854987	0.395390031656354	1081
DLEU1	-0.030709104	0.313098861032567	0.395223795695285	1081
BLMH	-0.030717841	0.312961132618481	0.395099455824986	1081
SCARNA1	-0.030739783	0.31261544363185	0.39471251093473	1081
RP1	-0.030749271	0.312466031204366	0.394548589608371	1081
BCL2L10	-0.030755144	0.312373575763055	0.394456571412696	1081
SNHG7	-0.030767075	0.312185808735327	0.394244177342851	1081
BARX2	-0.03077066	0.312129396874238	0.394197649052192	1081

C3orf52	-0.030771896	0.312109963268449	0.394197649052192	1081
RGR	-0.03081885	0.311371840943543	0.39333954573322	1081
PFKFB3	-0.03083125	0.311177101030251	0.393142847303506	1081
GLRA1	-0.030832004	0.311165260316807	0.393142847303506	1081
TRAM1	-0.030882234	0.310377258180814	0.392279933876184	1081
C14orf50	-0.030905339	0.310015234156428	0.391896140316343	1081
C3orf72	-0.030909187	0.309954964837927	0.391844541365551	1081
C3orf14	-0.030911365	0.309920862523748	0.391826018296781	1081
CCDC138	-0.030946907	0.309364615612432	0.39122097812382	1081
SCARNA9	-0.030972056	0.308971421767646	0.390895515387127	1081
C1orf201	-0.030977425	0.308887518831194	0.39081390948715	1081
UBAP2	-0.031000674	0.308524405096375	0.390428050798823	1081
RNF145	-0.03100313	0.308486053856074	0.390404042903041	1081
ZNF226	-0.031011619	0.308353541843104	0.390285379519309	1081
TSNAX	-0.031021666	0.308196766804224	0.390111458829475	1081
NTS	-0.03102837	0.308092180608838	0.390052600450292	1081
RNF24	-0.031029986	0.308066978142634	0.39004520612679	1081
LOC64348	-0.031043179	0.30786124149323	0.389809221237028	1081
PAQR9	-0.031045446	0.307825899218414	0.389788971007289	1081
PGM3	-0.031068673	0.307463942702254	0.389453038590097	1081
ADPRHL1	-0.03108911	0.307145687161271	0.389123317267077	1081
BAG2	-0.031097831	0.307009950037408	0.389024747987393	1081
FAM53B	-0.031100804	0.306963692753849	0.388990601404435	1081
SLC5A7	-0.031103865	0.306916055087556	0.388954701166257	1081
RASGEF1	-0.031122299	0.306629334179321	0.388615787168444	1081
MORN4	-0.031123324	0.306613387780871	0.388615787168444	1081
MINA	-0.031134586	0.306438312044075	0.388422563149052	1081
GSG1L	-0.031150802	0.306186340319879	0.388152024777797	1081
MTRR	-0.031153466	0.306144949249672	0.388148404722428	1081
RNF113B	-0.031211799	0.305239743479749	0.387073815460125	1081
BHLHE22	-0.031215617	0.305180545629256	0.387047474924538	1081
RAB31	-0.031218124	0.305141688972369	0.387022560243555	1081
C10orf88	-0.031244544	0.304732370526478	0.386673822389512	1081

OR1J4	-0.031251293	0.304627878076914	0.386589933467269	1081
RMST	-0.031292017	0.303997814664153	0.38588758515497	1081
GPR52	-0.031304115	0.303810796621213	0.385771730093561	1081
ABCC6	-0.031316951	0.303612469390457	0.385544200445711	1081
AGA	-0.031335348	0.303328362572142	0.385275316806402	1081
LAMC3	-0.031357175	0.302991517829877	0.384876986359977	1081
MGC2738	-0.031404143	0.302267505796052	0.38400573275282	1081
PARM1	-0.031430203	0.301866302374717	0.383592800185371	1081
FAM122B	-0.031485729	0.301012614779358	0.382556250377273	1081
C2orf66	-0.031496959	0.300840158983843	0.382418980009993	1081
RPL11	-0.031510848	0.300626956087239	0.382210793965126	1081
TXLNB	-0.031525863	0.300396572874042	0.381990213391464	1081
FIGF	-0.031529016	0.300348218255163	0.381959621183345	1081
TSGA14	-0.031543304	0.300129136057101	0.381722422240691	1081
SLC3A1	-0.03154507	0.300102060026889	0.381712084811319	1081
GABRA5	-0.031561566	0.299849262262875	0.381438708530286	1081
NOX5	-0.031593802	0.299355668433228	0.38095514469914	1081
AZI2	-0.031597942	0.299292305121307	0.380898571398618	1081
SNORA58	-0.031604572	0.299190878819567	0.380793546488512	1081
MYBBP1A	-0.031658939	0.298359932980908	0.379952010234553	1081
SDHAP1	-0.03167294	0.298146202667879	0.379703834412974	1081
ADRB1	-0.031679775	0.298041893193099	0.379594988833216	1081
USO1	-0.03168789	0.297918078448864	0.379485279187629	1081
RAB21	-0.031698363	0.297758347016992	0.37930579870098	1081
MTDH	-0.031699165	0.29774611910861	0.37930579870098	1081
TEAD2	-0.031723445	0.297376032320958	0.378962561431282	1081
COL6A1	-0.031725564	0.297343746979771	0.378945390201017	1081
KCNC1	-0.03173471	0.297204428729626	0.378815768222608	1081
AMD1	-0.031751322	0.29695149713039	0.378589197499633	1081
FRMD7	-0.031763695	0.296763210873018	0.378373093863098	1081
SCARNA1	-0.031782269	0.296480686546233	0.378060731135198	1081
RPS17	-0.031801694	0.296185419038823	0.377732037638458	1081
CCNJ	-0.031811951	0.296029592563741	0.3775811157526	1081

GPHN	-0.031813326	0.296008710485633	0.377578387433235	1081
GATSL3	-0.031825806	0.295819181380725	0.377360524880618	1081
ATP6V1G	-0.031929486	0.294247836807617	0.375451144697837	1081
DDX10	-0.031941961	0.294059146442821	0.375257918731194	1081
RAB30	-0.031948873	0.293954643860433	0.375148324394578	1081
TMEM185	-0.032002583	0.293143370684396	0.374184083287109	1081
GPD2	-0.032003996	0.293122038832426	0.374180563480085	1081
CX3CL1	-0.032019281	0.292891451520316	0.373981003414892	1081
FAM151B	-0.032045539	0.292495620239183	0.373522933104174	1081
LOC28576	-0.032078187	0.292003939166434	0.372989623645159	1081
ADAM7	-0.032079448	0.291984963676347	0.372989035717185	1081
FAM91A1	-0.032105531	0.291592571232014	0.372629557692775	1081
C10orf84	-0.032135372	0.291144088331789	0.372150866715983	1081
DLG3	-0.032144678	0.291004320456762	0.37199581417612	1081
TNFAIP8L	-0.032153625	0.290869975353434	0.371894875190082	1081
PCDHB17	-0.032174754	0.290552913463633	0.371583826925146	1081
PPID	-0.032202568	0.290135871068605	0.371097595090606	1081
C2orf60	-0.032203112	0.290127713802413	0.371097595090606	1081
SLC7A8	-0.032213462	0.289972635401995	0.370959467846142	1081
NKX1-2	-0.032217306	0.289915058602561	0.370909364635373	1081
C14orf126	-0.032217527	0.289911740367352	0.370909364635373	1081
DIO3OS	-0.03222195	0.289845499754936	0.370891036814024	1081
RUFY1	-0.03222357	0.289821242800859	0.370883555852071	1081
EMILIN3	-0.032224743	0.289803675573811	0.370883555852071	1081
TCTE3	-0.032255508	0.289343214978306	0.370365933774176	1081
FOLH1B	-0.032299181	0.288690403251772	0.369624264237127	1081
OR2A5	-0.032308117	0.288556944432095	0.369500358859938	1081
HSD3B1	-0.032319516	0.288386771441727	0.369329403095588	1081
POU2F3	-0.032341723	0.28805545170265	0.368952001179418	1081
ACAT1	-0.032343213	0.288033222395047	0.368946987038101	1081
RBM24	-0.032353833	0.287874873592901	0.368791054278474	1081
DDB1	-0.032355227	0.287854102891897	0.368787897656911	1081
INSC	-0.032426819	0.286788197944613	0.367656118594516	1081

FAM21B	-0.032515864	0.285466163930697	0.366194337263366	1081
SPSB1	-0.032517509	0.285441778110758	0.366186373307089	1081
EP400NL	-0.032526503	0.285308487934621	0.366038688666599	1081
GGN	-0.03253037	0.285251185135942	0.365988480005321	1081
SNORA77	-0.032582752	0.284475791876997	0.365086629761235	1081
TAS2R46	-0.032584572	0.284448887585032	0.365075359352731	1081
FBXO32	-0.032587658	0.284403256375626	0.365040050951704	1081
NYNRIN	-0.032649936	0.283483399199305	0.363998538904328	1081
FAM107B	-0.032657977	0.283364779998939	0.363869422047337	1081
C15orf43	-0.032685501	0.282958992171221	0.36339467660903	1081
SYT6	-0.032712161	0.282566335948415	0.36295981876432	1081
HEATR2	-0.032780029	0.281568394409353	0.361793296681747	1081
TOP1	-0.032788626	0.28144215224532	0.361693086087823	1081
KCNJ9	-0.032831482	0.280813411818691	0.360983047508121	1081
PPAN-P2F	-0.032835124	0.280760030234839	0.360938788071527	1081
PCDHA4	-0.032853347	0.280493011404163	0.360641544213484	1081
NKAIN4	-0.032860705	0.280385241593248	0.360549003695646	1081
BEX5	-0.032878308	0.280127534367049	0.360276928794057	1081
LY6G6C	-0.032888236	0.279982261035322	0.360145753691116	1081
TMEM211	-0.032917792	0.279550065697793	0.359658709508369	1081
SPTBN5	-0.032932443	0.279336001782139	0.359429212819442	1081
OR52B2	-0.032949407	0.279088271115462	0.359142581288704	1081
SLC7A4	-0.032956236	0.278988593298337	0.359050994186099	1081
SUSD1	-0.032968742	0.278806107718468	0.358861992204239	1081
SPATA21	-0.032980882	0.278629042252213	0.358657000394303	1081
COL4A2	-0.032983299	0.278593799084569	0.358634550614011	1081
COL5A3	-0.033006858	0.278250426034486	0.358215416185117	1081
L3MBTL	-0.033049544	0.277629008155167	0.357506799366264	1081
NTF3	-0.033067062	0.277374265480107	0.357201596758726	1081
TEX12	-0.033067292	0.277370921996482	0.357201596758726	1081
WDFY1	-0.033081155	0.277169433009685	0.356983455532262	1081
FAF2	-0.033089573	0.277047135941495	0.356848756620295	1081
MSX2P1	-0.033093774	0.27698611786216	0.356792975531249	1081

CCT6B	-0.033097752	0.276928349188396	0.356741373219097	1081
PROP1	-0.033097869	0.276926656032007	0.356741373219097	1081
ZNF586	-0.033123376	0.276556414487416	0.356388587678336	1081
TAL2	-0.033126563	0.276510180759159	0.356362202763307	1081
CCDC65	-0.033129587	0.276466308906201	0.356328457640142	1081
C12orf69	-0.033130719	0.276449892555857	0.356328457640142	1081
C2orf84	-0.033133964	0.276402832121384	0.356292235288283	1081
SEPT4	-0.033162542	0.275988572405847	0.355849320987954	1081
GOLGA7	-0.033163033	0.275981465072415	0.355849320987954	1081
RGPD8	-0.033186744	0.275638090079706	0.355465675991018	1081
TCTEX1D	-0.033190849	0.275578672750623	0.355427149202301	1081
OR6T1	-0.033196475	0.275497248235268	0.3553522900307	1081
SERPINB8	-0.033209936	0.275302506120608	0.355123838496487	1081
ANKRD44	-0.033212595	0.275264054812593	0.355096976447213	1081
RPSAP58	-0.033282153	0.274259353525359	0.3538688706058	1081
GPR44	-0.033282491	0.274254463769531	0.3538688706058	1081
RIMS1	-0.033299193	0.274013594834036	0.353642377342024	1081
NCRNA00	-0.033327829	0.273600952642231	0.353245606035874	1081
CRIPT	-0.033330496	0.27356254054331	0.353218650124663	1081
SH3BP5L	-0.033357404	0.273175224558512	0.35280900748373	1081
ARMC4	-0.033359462	0.273145626463939	0.352793399058541	1081
PTK2	-0.033399403	0.272571432985244	0.352142084107468	1081
KRT71	-0.033416495	0.272325976731143	0.351847537278661	1081
ING2	-0.033420495	0.272268556672179	0.351795912652238	1081
SLC9A2	-0.033421579	0.272252996014127	0.351795912652238	1081
PRUNE2	-0.03343784	0.27201965581603	0.351541953195658	1081
CDC5L	-0.033442331	0.27195523239316	0.35148124440625	1081
ADAMTS1	-0.033464082	0.271643370041629	0.351145770629403	1081
PPP1R3A	-0.033478402	0.271438194455599	0.350925582550895	1081
SOX1	-0.033509699	0.270990126544982	0.350391277230338	1081
RFX1	-0.033537905	0.270586743390377	0.349937083238053	1081
PEX6	-0.033542744	0.270517581353526	0.349873045050158	1081
PPT2	-0.033586491	0.269892878935365	0.349129393575608	1081

CA10	-0.033593729	0.26978960875324		0.349044005975037	1081
INSL5	-0.033594843	0.269773715808561		0.349044005975037	1081
AGBL5	-0.033606614	0.269605854734585		0.348870114570158	1081
LRRC8C	-0.03361101	0.26954316980943		0.348833814455285	1081
KIAA175[illegible]	-0.033617474	0.269451027101995		0.34873697018758	1081
LENEP	-0.033617752	0.269447068660943		0.34873697018758	1081
HIVEP3	-0.033620313	0.269410569611878		0.34873697018758	1081
MZF1	-0.033632555	0.269236140204799		0.348588472142176	1081
HMGA2	-0.033632679	0.269234377889764		0.348588472142176	1081
LOC2842[illegible]	-0.033641134	0.269113961439832		0.348524638586011	1081
ZNF385B	-0.033648149	0.269014069821503		0.348462476630284	1081
MMP3	-0.03365319	0.268942306427359		0.348391920968372	1081
SERP2	-0.033703938		0.268220617	0.347501725893073	1081
PRKACB	-0.033713314	0.268087425879763		0.347396191582905	1081
C16orf88	-0.033729849	0.267852643487002		0.347158946345342	1081
C4orf3	-0.03377396	0.267226994367408		0.346437209700202	1081
MPPE1	-0.033799809	0.266860840347689		0.346007055982764	1081
B3GALT5	-0.033819875	0.26657683581398		0.345727828331463	1081
RIPK4	-0.033821961	0.266547328922786		0.345711816968165	1081
ADAT2	-0.033843273	0.266245940211893		0.345387627379006	1081
TCTN1	-0.033880182	0.265724543683757		0.3448222708393	1081
ART1	-0.033881221	0.265709873926181		0.3448222708393	1081
LOC1536[illegible]	-0.033904889	0.265375897649055		0.344435583406536	1081
C1orf213	-0.03397585	0.264376343063587		0.343183287004057	1081
AHSA2	-0.033976424	0.26436826823173		0.343183287004057	1081
KRTAP4-[illegible]	-0.033983176	0.264273293600292		0.343160081189756	1081
KRBA1	-0.033988469	0.264198850948492		0.343085531641679	1081
CARD10	-0.033992934	0.264136072728172		0.343048235775193	1081
SNORA12	-0.03399588	0.264094657187711		0.343038678770161	1081
FETUB	-0.033996693	0.264083233226241		0.343038678770161	1081
SNORA71	-0.034026497	0.263664507399306		0.342568300648759	1081
MRPS31	-0.034034553	0.263551396668683		0.342462985293645	1081
C2CD2	-0.034041453	0.263454550826673		0.342405930735698	1081

C21orf99	-0.034052396	0.263301012122969	0.34222845920493	1081
RPS7	-0.034052687	0.263296929761382	0.34222845920493	1081
RPRML	-0.034081389	0.262894504611784	0.341810365006092	1081
SPDYE2	-0.034087377	0.262810592225924	0.341745376994012	1081
MRGPRE	-0.03409616	0.262687568674734	0.341628824835038	1081
C15orf41	-0.034103816	0.262580352036196	0.341534167856997	1081
KRTAP10	-0.034124665	0.262288537557489	0.341176637766876	1081
C15orf32	-0.034129107	0.262226393606178	0.341117828954957	1081
C6orf41	-0.034144604	0.262009668538277	0.340901942304695	1081
LOC28554	-0.03414593	0.261991131058827	0.340899840794475	1081
PPAT	-0.034161792	0.261769440538464	0.340655386282129	1081
IFNAR1	-0.034162627	0.261757772223325	0.340655386282129	1081
FAM110C	-0.034199043	0.261249321187139	0.340088373202902	1081
GCNT2	-0.034200038	0.26123543669283	0.340088373202902	1081
ESRRB	-0.034204739	0.261169859554175	0.3400288774458	1081
KLHL38	-0.034234977	0.260748270022131	0.339501932497144	1081
NCOA5	-0.034245137	0.260606731361045	0.339361512816305	1081
KPNA1	-0.034282498	0.260086675496108	0.338728088820086	1081
AFMID	-0.034288329	0.260005576954929	0.338688156983967	1081
OR5H6	-0.034320227	0.259562229626447	0.338198118868428	1081
WDR70	-0.034321776	0.2595407138467	0.338191958631421	1081
MORF4	-0.034322919	0.259524835570917	0.338191958631421	1081
SH3BP2	-0.034328615	0.259445743167371	0.338111948253765	1081
CAV3	-0.034370344	0.258866730103813	0.337379198935195	1081
ZNF814	-0.034445216	0.257830071173225	0.336158616515735	1081
EML6	-0.03445572	0.257684870797618	0.335991049981749	1081
KIF9	-0.03446413	0.257568658059581	0.335878591005009	1081
OR1K1	-0.034474451	0.257426076019783	0.335718799858777	1081
HDAC5	-0.03447536	0.257413531994832	0.335718799858777	1081
FBLN2	-0.034526696	0.256705207386981	0.334865401982175	1081
COPB1	-0.034531908	0.256633370542401	0.334793371945128	1081
LASS3	-0.034537494	0.256556395536684	0.334714629109935	1081
ECSCR	-0.034549286	0.256393942240796	0.334546017646275	1081

TPSB2	-0.034562913	0.256206312976394	0.334356945603806	1081
ACSBG1	-0.034600807	0.255685030147492	0.3337507245721	1081
PHF7	-0.03462202	0.255393540853797	0.333435053823703	1081
ABCA3	-0.034629613	0.255289269535698	0.333342126963744	1081
HSPA1L	-0.034633731	0.255232718857985	0.333289889245744	1081
ABCC1	-0.034665745	0.254793442351876	0.332824140589971	1081
NPBWR1	-0.034677066	0.254638230747831	0.332642964685498	1081
CDNF	-0.034678811	0.254614314648703	0.332633292386389	1081
CAPN2	-0.034704967	0.254255980546935	0.332199285695293	1081
BTBD11	-0.034705815	0.254244363502814	0.332199285695293	1081
PRSS48	-0.034731633	0.253891017579264	0.331817474317781	1081
FAM120C	-0.034757191	0.253541574060071	0.331446788412727	1081
OR8B8	-0.0347628	0.253464923196424	0.33136808863599	1081
UNC80	-0.0347679	0.253395241651165	0.331319993708231	1081
PPBPL2	-0.034774872	0.253300008863758	0.33123847312953	1081
PREP	-0.034781437	0.253210370475762	0.331164248083764	1081
CD164L2	-0.034815031	0.25275196346187	0.330650581462391	1081
MGC1602	-0.03481986	0.252686110663666	0.33060716670783	1081
FNBP1	-0.034822421	0.252651195453061	0.330583160067674	1081
KLK10	-0.034822909	0.252644553165667	0.330583160067674	1081
PIK3R3	-0.034860538	0.252131931252084	0.329946586661896	1081
PIP5K1P1	-0.034861162	0.25212343070682	0.329946586661896	1081
NOVA1	-0.034881149	0.251851450148026	0.329686624422119	1081
C9orf150	-0.034883116	0.251824690939576	0.329673017869623	1081
OR6C76	-0.034890092	0.251729818584266	0.329591654450802	1081
CCDC62	-0.034891528	0.251710292088348	0.329587509530047	1081
PIP	-0.03489492	0.251664169059343	0.329548536511991	1081
CITED4	-0.034930499	0.251180766553802	0.328958298155399	1081
TPH2	-0.034932861	0.251148695602374	0.328937681094196	1081
CELSR2	-0.034957106	0.250819687499537	0.328528127742405	1081
SLC28A2	-0.034967651	0.250676682704713	0.32838352016429	1081
TCFL5	-0.034974867	0.250578860876699	0.328298071823693	1081
SNORA11	-0.03500182	0.250213691188543	0.327862287562977	1081

OR1L4	-0.035025218	0.249896997060337	0.32748991710776	1081
NWD1	-0.035043975	0.249643319913468	0.327200044219702	1081
SCARNA8	-0.0350646	0.249364588315155	0.326898524865543	1081
LPIN2	-0.035084067	0.24910170393253	0.32657515460893	1081
C6orf94	-0.035091176	0.249005751499566	0.326470606180199	1081
SYT11	-0.03511935	0.248625729392203	0.326014796498466	1081
PAK2	-0.035124436	0.248557172132541	0.32594611591004	1081
MRGPRF	-0.035169799	0.247946263650895	0.325208508447638	1081
ZNF563	-0.035173244	0.247899912545313	0.325168885155966	1081
TMEM116	-0.035189985	0.247674759078762	0.324979353979135	1081
DUOX1	-0.035196994	0.247580537940083	0.324876884888157	1081
USHBP1	-0.035262187	0.246705321143393	0.323833888996785	1081
TMC1	-0.035264458	0.246674874850801	0.323815023711024	1081
CPOX	-0.035266991	0.246640912972476	0.323791540686251	1081
ESYT3	-0.03528867	0.246350406541184	0.323431239557622	1081
MYL9	-0.035293147	0.246290454278403	0.323373603691484	1081
GALNT2	-0.035294833	0.246267867276598	0.323365023222988	1081
RCL1	-0.035315605	0.245989814858301	0.323105223989076	1081
DISC2	-0.035318966	0.245944844223894	0.323067220063273	1081
PPIG	-0.035393723	0.244946058538783	0.321860175413462	1081
ATG10	-0.035412886	0.244690501907226	0.321587301730236	1081
BRMS1L	-0.035419402	0.244603640704651	0.321494117700475	1081
MYO1H	-0.035450024	0.244195739404524	0.321020828132611	1081
ENAM	-0.035456668	0.244107307097217	0.32092551729253	1081
GOLGA6L	-0.035473869	0.243878446208424	0.320666490364122	1081
ANXA13	-0.035479416	0.243804673035744	0.320590413727485	1081
LFNG	-0.035500717	0.243521543793426	0.320259922948071	1081
C8orf4	-0.035525159	0.24319693828995	0.319895678867191	1081
LAMP1	-0.03552738	0.243167456862799	0.319877786241418	1081
SNORA74	-0.03553639	0.24304788541169	0.319783139678564	1081
OR5T2	-0.035541092	0.242985506275001	0.319721948001952	1081
FKBP6	-0.03555131	0.242849967353403	0.319564477910661	1081
ARHGAP1	-0.03555261	0.242832731680155	0.31956267178578	1081

CC2D2B	-0.035593878	0.242285935726556	0.318865191748687	1081
KCNC3	-0.035596344	0.242253286890594	0.318865191748687	1081
C21orf49	-0.035606944	0.242112989585707	0.31871960891355	1081
ZNF71	-0.035665589	0.241337828389141	0.317761474045703	1081
MOGAT1	-0.035669749	0.241282905905879	0.317730692866645	1081
CXorf58	-0.035670344	0.241275046398676	0.317730692866645	1081
KCNH5	-0.035674218	0.241223918857962	0.317694550561823	1081
STATH	-0.035695673	0.24094086242663	0.317342508897317	1081
GANC	-0.035729331	0.240497274710084	0.316799686067786	1081
RPS13	-0.035741519	0.240336791066476	0.316650402618324	1081
CDRT15P	-0.035758694	0.240110758085575	0.316373289399825	1081
ATMIN	-0.035779657	0.239835088369783	0.316051406764524	1081
C11orf1	-0.035793348	0.239655171261006	0.31585563788374	1081
FAM9B	-0.035795899	0.239621652341166	0.315832124209159	1081
APOF	-0.035805595	0.239494303046644	0.315705584012213	1081
LYPD4	-0.035812962	0.239397569068577	0.315620460594401	1081
ANGPTL3	-0.03582818	0.239197840480773	0.315397335808691	1081
DPRX	-0.035840099	0.239041496767461	0.315211818575669	1081
ARNTL2	-0.035858271	0.238803256657483	0.314984163498184	1081
PRM2	-0.035861418	0.238762016883362	0.314984163498184	1081
CABC1	-0.035872335	0.23861900349223	0.314847033053158	1081
OR1E2	-0.035881623	0.238497370010115	0.314741534153539	1081
NEIL2	-0.03588432	0.238462050889895	0.314715540826582	1081
SNORA71	-0.035902845	0.238219617525832	0.314457387789652	1081
LOC10013	-0.035910104	0.238124654322853	0.314352631804316	1081
C16orf46	-0.035912966	0.238087231065093	0.314342147809071	1081
FAM118A	-0.035928334	0.237886313278952	0.314099743134593	1081
OAZ3	-0.035931067	0.237850593318144	0.314073164813451	1081
CCNB3	-0.035957194	0.237509326830018	0.313663654712908	1081
GDF11	-0.035967114	0.237379852143037	0.313554332268145	1081
CLK2	-0.0360047	0.236889697317218	0.312993369334141	1081
RPL41	-0.036039784	0.236432830266175	0.312508323975599	1081
LOC1002	-0.036040448	0.236424186884912	0.312508323975599	1081

LOC41505	-0.036083152	0.235868941730503	0.311865307899775	1081
LPAL2	-0.036100164	0.235647996795028	0.31163453656114	1081
CDKN2B	-0.036119397	0.235398394737931	0.311365769008248	1081
ICMT	-0.036149188	0.235012136677426	0.310916102539355	1081
ERICH1	-0.036197199	0.234390591289012	0.310194640294708	1081
MMP27	-0.036212649	0.234190822128344	0.309972017856471	1081
SSTR4	-0.036233256	0.233924558407924	0.309639938834853	1081
OR10A3	-0.036239398	0.233845244641642	0.309555293291227	1081
FGF19	-0.036241794	0.233814310321378	0.309534683671168	1081
C18orf19	-0.036244218	0.233783014984152	0.309516056862102	1081
SPON2	-0.03627063	0.233442206642579	0.309123332203691	1081
HIRA	-0.036290087	0.233191371253956	0.308852082439905	1081
LYSMD1	-0.036314653	0.232874945558015	0.308453269660479	1081
LONP2	-0.036324512	0.232748031684679	0.308305437814826	1081
NOVA2	-0.036340343	0.23254435769389	0.308055900949788	1081
GPN3	-0.036347973	0.232446236254703	0.307946167917335	1081
SYT8	-0.036351497	0.232400937119242	0.307906404358245	1081
ANO3	-0.036359085	0.232303395684883	0.307837909885013	1081
ACTN4	-0.036359362	0.232299831076089	0.307837909885013	1081
WNT16	-0.036377872	0.232062029678383	0.30755852551783	1081
ECHDC1	-0.036418102	0.231545775227075	0.306955096206201	1081
MPP4	-0.036424304	0.23146625579382	0.306869873179764	1081
FAM13AC	-0.036506407	0.230415451274326	0.305577305195609	1081
PDZD9	-0.036511984	0.230344196647591	0.305502919314354	1081
PATE4	-0.036514282	0.230314837570567	0.305484092893012	1081
LPAR6	-0.036536828	0.23002696572301	0.305122356257986	1081
ERVFRDI	-0.036551213	0.229843425872912	0.304919052631531	1081
LY75	-0.036554781	0.229797912560079	0.304878750561301	1081
C2orf43	-0.036559161	0.229742056279395	0.304838752006789	1081
TMBIM6	-0.036600384	0.229216833223838	0.304228034345382	1081
NXF4	-0.03661356	0.229049133383022	0.304050014997593	1081
TTC21A	-0.036619891	0.228968589183488	0.303978662785117	1081
MFI2	-0.036661823	0.228435624505813	0.303291086513517	1081

GATA1	-0.036694857	0.228016368538714	0.302774355297106	1081
CNBD1	-0.036699169	0.227961680434031	0.302761606826447	1081
RAB5B	-0.036703084	0.227912040974926	0.302715636938081	1081
MRRF	-0.036704286	0.227896795967245	0.302715347142302	1081
KCTD6	-0.036754396	0.227262102030981	0.301932008534862	1081
MEG8	-0.036802252	0.226657137029059	0.301247478092656	1081
C22orf36	-0.036832482	0.226275573268568	0.300799882769915	1081
SNORA31	-0.036850513	0.226048205821767	0.300557131956933	1081
CLUAP1	-0.036891101	0.225536997058142	0.299917011402487	1081
TIMP2	-0.036901025	0.22541213460244	0.299770758619366	1081
LY6G6E	-0.036907419	0.225331701891287	0.299723151838889	1081
OLFM3	-0.036927478	0.225079525949161	0.299466815286035	1081
JOSD1	-0.036932549	0.225015813861664	0.299401821020028	1081
RPS3	-0.036975695	0.224474183913876	0.298779810700036	1081
KLK6	-0.037006523	0.224087763215854	0.298324609435857	1081
ANGPT1	-0.037082141	0.223141903530715	0.29722253680417	1081
C1orf229	-0.037108907	0.222807796287299	0.296836390199566	1081
TOPBP1	-0.037116376	0.222714619884047	0.296731879468527	1081
MYH2	-0.037135593	0.222475038876072	0.296451889016965	1081
GSX2	-0.037135733	0.222473295371195	0.296451889016965	1081
IRAK4	-0.037136025	0.222469651409132	0.296451889016965	1081
GOLGA9I	-0.037155218	0.222230552961445	0.296224077906988	1081
CDK17	-0.037178772	0.221937377313396	0.295872441663581	1081
C1orf163	-0.037185097	0.22185869854789	0.295826282912843	1081
C2orf15	-0.037185172	0.221857764287306	0.295826282912843	1081
MCM3AP	-0.037226027	0.221350030094489	0.295245736360556	1081
RAB18	-0.037238495	0.221195242789463	0.295097891787664	1081
FAM47B	-0.037241418	0.221158968382316	0.295069038880837	1081
LOC12183	-0.037247541	0.221082983654638	0.295006736816763	1081
PLA2G6	-0.037253072	0.221014370736611	0.294934717705951	1081
TMPRSS3	-0.037288861	0.22057076527746	0.294479293956818	1081
EIF2S3	-0.037310737	0.220299925940856	0.294156691726556	1081
COMP	-0.037323767	0.220138709743882	0.293960911294611	1081

ATP12A	-0.037334405	0.22000716378765	0.293804727510919	1081
CFTR	-0.03733961	0.219942812286907	0.293738262630584	1081
GOSR1	-0.037358128	0.219714004116936	0.293491055827576	1081
GRPR	-0.037367875	0.21959362798114	0.293369164777509	1081
MYO15B	-0.037403459	0.21915458774117	0.292898512648844	1081
VPS26A	-0.037432749	0.21879368343851	0.292475033368864	1081
RPE65	-0.037441264	0.2186888362069	0.292354277350056	1081
UBTF	-0.037441454	0.218686496112149	0.292354277350056	1081
AGPHD1	-0.037474327	0.218282086750448	0.291887994529556	1081
LOC72380	-0.037492905	0.21805377738401	0.29162141309174	1081
TMEM90A	-0.037511014	0.217831396991312	0.291375895437443	1081
OR10W1	-0.037512126	0.217817741689445	0.291375895437443	1081
RAB11A	-0.037534896	0.217538364608749	0.29104804111332	1081
ITGA11	-0.037536772	0.217515362175644	0.291036594781373	1081
SCGB3A2	-0.037550544	0.217346528577104	0.290849330290007	1081
KCNH1	-0.037557624	0.21725976522163	0.290752539054656	1081
DCDC2B	-0.037564403	0.217176713167189	0.290660702016545	1081
SLC35F2	-0.037568016	0.217132453788581	0.290620774803731	1081
OR10H1	-0.037580375	0.216981131020772	0.29043753384807	1081
FLJ10661	-0.037599033	0.216752819708355	0.290189775571531	1081
LRRC4	-0.037619382	0.216504009424374	0.289875931799416	1081
LASS2	-0.037620065	0.216495664256213	0.289875931799416	1081
KIAA0284	-0.037657373	0.216040039884276	0.289350881156089	1081
TGIF1	-0.037690154	0.215640278084956	0.288911505854047	1081
GIT2	-0.037713156	0.215360083058074	0.288574489371086	1081
KLK14	-0.037738537	0.215051210757138	0.288237301443949	1081
SLC16A8	-0.037743429	0.214991724874681	0.288195920787893	1081
ORAI2	-0.037769454	0.214675405233981	0.287867672131968	1081
TMEM57	-0.037774894	0.214609338669741	0.287798237751427	1081
RPH3AL	-0.03777701	0.214583641217573	0.287782934047533	1081
FGF3	-0.037804966	0.21424433409115	0.287404415679977	1081
CMTM8	-0.03782912	0.213951493765223	0.287030690057301	1081
ARMC9	-0.037847512	0.213728695027648	0.286769985435724	1081

PLEKHA9	-0.037847564	0.213728066711799	0.286769985435724	1081
THADA	-0.037848436	0.213717507849941	0.286769985435724	1081
REXO2	-0.037859885	0.213578911596316	0.286626285664365	1081
TMX4	-0.037861567	0.213558551219699	0.286618055584333	1081
LOC61920	-0.037892881	0.213179830887266	0.286166968296393	1081
C1orf112	-0.037895461	0.21314864990144	0.286144179145976	1081
AARS2	-0.037931553	0.212712781235497	0.2856542215845	1081
ID2	-0.03793222	0.212704738998215	0.2856542215845	1081
ZMYND1	-0.037932728	0.212698601825393	0.2856542215845	1081
CCDC110	-0.037985236	0.212065675455984	0.284918169405148	1081
POU6F1	-0.037985427	0.212063377991204	0.284918169405148	1081
KCTD15	-0.03806857	0.211063962926372	0.283742644854973	1081
SRMS	-0.038074038	0.21099835004409	0.283673369036185	1081
SAMD13	-0.03808316	0.210888928629319	0.28356410807887	1081
TM6SF1	-0.038098193	0.210708697790189	0.283359593924122	1081
BEST3	-0.03811319	0.210529007132263	0.283155751681095	1081
GRID2	-0.038130908	0.210316863302438	0.282889311025413	1081
CLDND2	-0.038131251	0.210312756623788	0.282889311025413	1081
SLC22A10	-0.038136753	0.210246909003646	0.282832987103736	1081
EPOR	-0.03814779	0.210114865696737	0.282711993685598	1081
C12orf4	-0.038160156	0.209966990233191	0.282559055327951	1081
AHNAK2	-0.038185589	0.20966310832544	0.282198390941136	1081
LOC33904	-0.038193482	0.209568858843646	0.282090382293549	1081
ZNF826	-0.038242853	0.208980048466027	0.281335410074051	1081
OR52W1	-0.038244072	0.20896551881065	0.281334650567436	1081
IL25	-0.038247949	0.208919340234596	0.28129127850728	1081
ST6GALN	-0.038265297	0.208712772785621	0.281050722444274	1081
VPS39	-0.038287613	0.208447257764785	0.280764412573152	1081
CHRD	-0.038292456	0.208389666562478	0.280728222074437	1081
LOC10013	-0.038298245	0.208320849163169	0.280654283848862	1081
ATP8A1	-0.038303232	0.208261578997344	0.280611966350178	1081
USP6	-0.038307898	0.208206129721671	0.280556018946024	1081
PSEN1	-0.038338898	0.207838015179817	0.28013494017111	1081

SNORA81	-0.038345701	0.207757292581206	0.280044875145426	1081
C9orf6	-0.038351207	0.207691982209396	0.279975574251089	1081
SLC25A31	-0.03835588	0.207636562151566	0.279919597439824	1081
BAIAP3	-0.038360637	0.207580147440243	0.279862272131154	1081
CCDC90B	-0.03838313	0.207313594970984	0.279557472358158	1081
OR4P4	-0.038385342	0.207287392861881	0.279542410577225	1081
TMEM184	-0.03838688	0.207269174567666	0.279536554975271	1081
PABPC1L	-0.038404947	0.20705527597389	0.279290330944053	1081
TMC7	-0.038405571	0.207047891528253	0.279290330944053	1081
NCSTN	-0.038407949	0.20701974979361	0.279290330944053	1081
CBWD5	-0.038432768	0.206726220654986	0.27895369516342	1081
STC1	-0.038473298	0.20624751713604	0.27834502798322	1081
DUSP21	-0.038481958	0.2061453284438	0.278225756498751	1081
MRPL19	-0.038482059	0.206144139718747	0.278225756498751	1081
TAX1BP3	-0.038489636	0.206054764531331	0.278140795462589	1081
OSBPL10	-0.038506078	0.205860939137237	0.277897783215147	1081
TEX261	-0.038517653	0.205724551140904	0.277750893554957	1081
EIF3C	-0.038550191	0.205341535940019	0.27731095020025	1081
LOC64367	-0.038570344	0.205104558206622	0.277080963322988	1081
GJC3	-0.0385798	0.204993445596692	0.276968005469172	1081
LCMT2	-0.038580725	0.204982578078538	0.276968005469172	1081
C3orf15	-0.038583593	0.204948877042598	0.276944937484782	1081
DNAJC28	-0.038609442	0.204645393890034	0.276627622620596	1081
C6orf130	-0.038641053	0.204274693434821	0.276145061014929	1081
NFKBIZ	-0.038642129	0.204262081811761	0.276145061014929	1081
PDCD7	-0.038649305	0.204178005721969	0.276069932563867	1081
PPP1R15A	-0.038664901	0.203995358776434	0.27586000554147	1081
C9orf72	-0.038675431	0.203872102974129	0.275711836359683	1081
C12orf23	-0.03868968	0.20370541353068	0.275523402415439	1081
ZNF564	-0.038709051	0.203478944453687	0.275291023238383	1081
SNORA5A	-0.03872077	0.203342034930898	0.27512427252891	1081
FZD5	-0.038759036	0.202895435271501	0.274538456713084	1081
VENTX	-0.03878684	0.202571395052563	0.274155240398648	1081

AFF3	-0.038787411	0.202564734852668	0.274155240398648	1081
CBY1	-0.038812577	0.202271765790701	0.273786516283927	1081
OR4D6	-0.038818295	0.202205240461055	0.27371486151646	1081
OTOR	-0.038830248	0.202066233346748	0.273581844933815	1081
CCDC11	-0.038843767	0.201909089797324	0.273387458930441	1081
LOC73177	-0.038844391	0.201901838221112	0.273387458930441	1081
LOC28504	-0.038856398	0.201762355538494	0.273243875786417	1081
SCG2	-0.038869788	0.201606892513083	0.273051690848195	1081
PCBP2	-0.038881631	0.201469457163711	0.272883897973707	1081
CYP8B1	-0.038884657	0.201434354635807	0.272854698368634	1081
FAM27B	-0.038903988	0.20121019041621	0.272587712571255	1081
APPBP2	-0.038984212	0.200281881807742	0.271494415518266	1081
DUSP28	-0.039001857	0.200078133488265	0.271254727715263	1081
RAB6B	-0.0390327	0.199722326541464	0.270827023975349	1081
CCDC7	-0.03904323	0.199600964189683	0.270698897509166	1081
SEMG2	-0.039065755	0.199341525438556	0.270383452057615	1081
B3GNT2	-0.039090577	0.199055906154893	0.270050591251284	1081
FAM18B2	-0.039109253	0.198841216062166	0.269795668995241	1081
TSPAN13	-0.039125188	0.198658161106991	0.269565448976177	1081
MTPAP	-0.0391269	0.198638503407796	0.269565448976177	1081
ANGPTL1	-0.039164009	0.198212730174025	0.269051644050652	1081
CDRT4	-0.039188171	0.197935854099906	0.268712027821458	1081
NXNL1	-0.039223468	0.197531895618429	0.268199773352648	1081
OR5C1	-0.039234792	0.197402428955016	0.268042055223699	1081
FAM166B	-0.039235914	0.197389603599589	0.268042055223699	1081
FAM196A	-0.03924038	0.197338561425676	0.267991460153717	1081
NSA2	-0.039244182	0.197295123183206	0.267950533036182	1081
TMEM196	-0.039244855	0.197287426678684	0.267950533036182	1081
IGFL4	-0.039246492	0.197268729187139	0.267950533036182	1081
TFDP1	-0.039247146	0.197261254999437	0.267950533036182	1081
PODNL1	-0.039253347	0.197190421076682	0.267880574050156	1081
AK3L1	-0.039253625	0.197187244290723	0.267880574050156	1081
MBD6	-0.039257634	0.197141465623087	0.26785019390147	1081

GJA5	-0.039269705	0.197003652826491	0.267699061463046	1081
TCHP	-0.039270742	0.196991822551153	0.267699061463046	1081
GK3P	-0.039271697	0.196980916527301	0.267699061463046	1081
INPP5K	-0.039279351	0.196893577254863	0.267621693124568	1081
GP2	-0.039287687	0.196798488589444	0.267528547988012	1081
RAB3IP	-0.039292782	0.196740387403797	0.267467613999831	1081
GNG2	-0.039321507	0.196413054739106	0.267076678212575	1081
CMTM3	-0.039340036	0.196202120835527	0.266843891725067	1081
ZBTB9	-0.039384177	0.195700281366088	0.266251243879235	1081
N6AMT2	-0.039394911	0.195578385264723	0.266103375061317	1081
SERPIND1	-0.039400972	0.195509590174797	0.266027740227712	1081
TECPR1	-0.039432543	0.195151490964646	0.265558415663523	1081
C20orf85	-0.039490443	0.194496004684179	0.264737973943431	1081
GLB1L3	-0.039510323	0.194271316538565	0.264485751954409	1081
LAYN	-0.039579615	0.193489640686176	0.263510601110264	1081
KIAA0922	-0.039593783	0.193330097686239	0.263328926158843	1081
ACAA2	-0.039597451	0.193288809356371	0.263308294866384	1081
SSBP2	-0.039659996	0.192585774795756	0.262421566102578	1081
LMOD3	-0.039660428	0.192580920727962	0.262421566102578	1081
ACPP	-0.039680035	0.192360920859353	0.262186113038678	1081
BCDIN3D	-0.039721265	0.191898896232754	0.261627183582081	1081
DFNB59	-0.039723102	0.191878332524905	0.261616853381673	1081
TMEM38B	-0.039780004	0.191242079310311	0.260855283885586	1081
C5orf62	-0.039790241	0.191127776698263	0.260717027463875	1081
OR2D2	-0.039802644	0.190989358171778	0.260545853617558	1081
ZNF575	-0.039814645	0.190855495756684	0.26041614481295	1081
TRIL	-0.039829834	0.190686171116011	0.260237986391981	1081
RPS27	-0.039841736	0.190553564627187	0.260074631396659	1081
CCDC144	-0.039849967	0.190461908559459	0.259978733288638	1081
C11orf93	-0.039855809	0.190396865191618	0.25991359011216	1081
UTS2R	-0.039855992	0.190394822102371	0.25991359011216	1081
OR4D5	-0.039874713	0.190186517098126	0.259661632459623	1081
LMLN	-0.039876606	0.190165461988499	0.259650483377953	1081

MINK1	-0.039879397	0.190134422402963	0.259625699132901	1081
DOCK11	-0.039879633	0.190131798297132	0.259625699132901	1081
KIAA0319	-0.039890828	0.19000733637072	0.259487342633595	1081
PIAS4	-0.039900967	0.189894674405561	0.259351065484748	1081
MAPK1IP	-0.039911275	0.189780169068093	0.259229828171733	1081
TBC1D20	-0.039925082	0.189626887530587	0.259055584518085	1081
RBM19	-0.0399261	0.189615587638323	0.259055584518085	1081
COL9A1	-0.039933711	0.189531139226387	0.258977467253311	1081
C8orf12	-0.03994554	0.189399935150985	0.258833301242561	1081
PPARG	-0.039957927	0.189262619722546	0.258698295176461	1081
MAP1A	-0.039959518	0.189244982404367	0.258691739874872	1081
B4GALT1	-0.039974549	0.189078462928586	0.258481652802413	1081
TMBIM4	-0.039986861	0.188942149034817	0.258312832867756	1081
GPR45	-0.040007166	0.188717489774303	0.258058229127297	1081
ZNF702P	-0.040047944	0.188266904279986	0.257529489150561	1081
PCDHGA8	-0.040068545	0.188039578855107	0.25725346798208	1081
GNPAT	-0.04008987	0.187804470126659	0.256966722183083	1081
AMPD1	-0.040089894	0.187804207324042	0.256966722183083	1081
SAMD11	-0.040098747	0.187706661689714	0.256867787496724	1081
OTUD6B	-0.040120498	0.187467178400776	0.256574924171726	1081
DCAKD	-0.040135964	0.187297028947958	0.256411732800314	1081
TXNRD3I	-0.04015227	0.187117760994038	0.256201134726085	1081
ZNF592	-0.040164173	0.18698698123056	0.256056878314841	1081
SNORA70	-0.040165354	0.186973998798945	0.256056506172995	1081
NCEH1	-0.040171225	0.186909522124357	0.255985609028157	1081
C20orf203	-0.040209742	0.186486916933539	0.255441554472437	1081
PLEKHB2	-0.040239995	0.186155474416358	0.255056929342144	1081
C1orf204	-0.040251773	0.186026558270657	0.254897634088041	1081
MTERFD3	-0.040254051	0.186001633809297	0.254880818521753	1081
TMEM136	-0.040295488	0.185548651905389	0.254329291190995	1081
CHD1L	-0.040298153	0.185519548215268	0.254306702422194	1081
AMY1A	-0.040305026	0.185444493452916	0.254221117428309	1081
PLCD1	-0.040338941	0.185074502757315	0.253765713180378	1081

C3orf51	-0.040345802	0.184999713881001	0.253714972845854	1081
SLC9A8	-0.040375826	0.184672722823852	0.253335512515254	1081
TMEM41I	-0.04040818	0.18432083194578	0.252904451678772	1081
CRMP1	-0.040414781	0.184249094777011	0.252823241896525	1081
ZMYND1(	-0.040418245	0.184211465686531	0.252788826720379	1081
GRAMD4	-0.04044536	0.183917065019119	0.252470819407847	1081
CNGB3	-0.040447717	0.1838914924417	0.252452917761895	1081
CLCC1	-0.04045092	0.1838567413308	0.252422412193073	1081
PHYHIP	-0.040462222	0.183734163177479	0.252271313877475	1081
GALNTL5	-0.040470062	0.183649170346442	0.252188993635246	1081
PM20D1	-0.040517094	0.183139901777352	0.251592561463773	1081
FAM136B	-0.040526539	0.183037746589227	0.251503676764203	1081
OR5AK2	-0.040549746	0.182786954549715	0.251193341933557	1081
CCDC117	-0.040552502	0.182757182019903	0.251169561453878	1081
IFT46	-0.040574359	0.182521228900684	0.250930878059392	1081
UFSP2	-0.040607963	0.182158915617459	0.250449860436371	1081
SUPT3H	-0.040618819	0.18204197936525	0.250306168474027	1081
IGJ	-0.040635757	0.181859637172107	0.25008958910725	1081
SMTN	-0.040641175	0.181801338715343	0.250043556251832	1081
DNAJC12	-0.040657133	0.181629718768973	0.249824572210909	1081
NMNAT3	-0.040659023	0.181609395843086	0.249813675606622	1081
LGR5	-0.040667525	0.181518022708043	0.249705037384153	1081
TMEM229	-0.040689661	0.18128025622628	0.249394984748918	1081
BTBD10	-0.040697261	0.1811986831708	0.249299786400476	1081
ARL6IP6	-0.040712009	0.181040463989492	0.249116130264229	1081
C7orf34	-0.040724652	0.180904895399306	0.248963595970693	1081
VSNL1	-0.040770257	0.180416539848923	0.248359381936351	1081
SPINK5	-0.040829638	0.179782135256978	0.247638366820637	1081
NPY1R	-0.040831289	0.179764520763465	0.247631036021608	1081
ARMC1	-0.040834428	0.179731033392959	0.247618770872737	1081
GNMT	-0.040849565	0.179569613903567	0.247447149058578	1081
GPR149	-0.040851493	0.179549059349523	0.247435750485439	1081
MAF	-0.04087155	0.179335360794487	0.247191983797806	1081

RTN2	-0.040902933	0.179001357567383	0.246765369752612	1081
TK2	-0.040912049	0.178904426397457	0.246661881576322	1081
APBB2	-0.040914758	0.178875628864225	0.246642679224492	1081
RAB41	-0.040916216	0.178860130952776	0.24663819139186	1081
FAM71F2	-0.040924613	0.178770890934383	0.246532009712017	1081
LOXL1	-0.04094161	0.17859036157684	0.24629991332686	1081
ARGFXP2	-0.040947937	0.178523199221657	0.246224144072319	1081
PPAPDC3	-0.040975774	0.178227916925133	0.245850546867763	1081
SLC7A2	-0.040982565	0.178155931828391	0.245781440423169	1081
TMEM25	-0.040989591	0.178081482933512	0.245699025662324	1081
C9orf3	-0.040998378	0.177988408467043	0.245587430723876	1081
CREG2	-0.041002862	0.177940926505405	0.245538733094828	1081
FAM181B	-0.041003437	0.177934831072304	0.245538733094828	1081
NPS	-0.041018139	0.17777921382873	0.245349199327243	1081
C8orf40	-0.041088691	0.177033845493801	0.24440425010092	1081
C1QTNF9	-0.041128656	0.176612662074235	0.243889640628288	1081
MEG3	-0.041152366	0.176363139091958	0.243578461333298	1081
ZNF565	-0.041152657	0.176360070937018	0.243578461333298	1081
WIPI1	-0.041180871	0.176063496989328	0.243225861703515	1081
PKNOX2	-0.04118513	0.176018757234148	0.243186191926611	1081
HMGB4	-0.041190525	0.175962099497873	0.243141264447813	1081
LGALS7	-0.041199603	0.175866792607004	0.243026240709843	1081
CLEC14A	-0.041217199	0.175682171669545	0.242804428394826	1081
SKP2	-0.04122784	0.175570582674951	0.242666853378175	1081
BSN	-0.041232177	0.175525120358832	0.242620663484882	1081
LOC39219	-0.04124933	0.175345406852853	0.242405518875289	1081
TDGF1	-0.041272252	0.17510546610887	0.242123660838986	1081
C15orf52	-0.041278162	0.175043640565767	0.242104643954505	1081
FLII	-0.041281417	0.175009589177492	0.242074167397732	1081
CYP17A1	-0.041282789	0.174995239600027	0.242070940173216	1081
RHBDL3	-0.041290121	0.174918575068644	0.241998124768755	1081
TUSC3	-0.041306929	0.174742932890221	0.241788335948451	1081
FAM154A	-0.041318347	0.174623684820996	0.241656531614959	1081

VGLL1	-0.041321948	0.174586083920971	0.241621095121459	1081
CMYA5	-0.041331854	0.17448269979997	0.24149460580353	1081
ENPP5	-0.041334998	0.174449894139333	0.241482382837688	1081
RPL37	-0.041336154	0.174437833773253	0.241482281566944	1081
REG3G	-0.041394579	0.173829083363126	0.240738820593302	1081
AKAP3	-0.041410788	0.1736604776397	0.24057147036527	1081
ACTR2	-0.041417414	0.173591591094679	0.240492579781467	1081
DIAPH2	-0.041417609	0.173589564553669	0.240492579781467	1081
PABPC4	-0.041420472	0.173559801157857	0.240481614576313	1081
GPR87	-0.04146208	0.173127740852618	0.239915961991882	1081
TMEM212	-0.041462408	0.173124346011863	0.239915961991882	1081
CASQ1	-0.04150839	0.172647807559797	0.239316733179117	1081
STX2	-0.04151524	0.172576899709621	0.239253460900482	1081
ARHGAP2	-0.04152664	0.172458941102198	0.239153670303832	1081
SLC2A11	-0.041535762	0.172364595824375	0.239039293878703	1081
RNF148	-0.041541638	0.172303852882931	0.238971505426963	1081
TPBG	-0.041574134	0.171968162941466	0.238555201931954	1081
FLVCR1	-0.041631611	0.171375629190939	0.237847884950153	1081
DEFB132	-0.041643024	0.171258149679554	0.23773397362835	1081
ZNF544	-0.04169701	0.170703278826285	0.23699638538632	1081
SLC16A4	-0.041719267	0.170474909294802	0.23672827240255	1081
CDKN1C	-0.041719624	0.170471256023108	0.23672827240255	1081
GPR133	-0.041735284	0.170310711001545	0.236565488045654	1081
ADAMTS	-0.041755902	0.170099514517094	0.236321015168748	1081
CCT6P1	-0.04177754	0.16987808362598	0.236045937001335	1081
TRPV5	-0.041780896	0.169843765446838	0.236014530932369	1081
ZFY	-0.041781597	0.169836593112244	0.236014530932369	1081
C15orf59	-0.041803489	0.169612821844702	0.235742396582139	1081
ABCA4	-0.041812082	0.16952504787596	0.235652918124567	1081
PIGG	-0.041831242	0.169329466718161	0.235429781699037	1081
MTA1	-0.041832132	0.169320388747873	0.235429781699037	1081
GABRG3	-0.041832674	0.169314851573706	0.235429781699037	1081
C9orf84	-0.041870409	0.1689301646886	0.234955686802807	1081

PRPF40A	-0.041906028	0.16856765484239	0.234516257375688	1081
PKN3	-0.041932083	0.168302852314759	0.23419637769433	1081
DUS4L	-0.041970496	0.167913021700878	0.23371849804216	1081
EN2	-0.041986824	0.167747521838213	0.233520408225471	1081
CTAGE9	-0.04200143	0.167599582773067	0.233378972486585	1081
PGRMC2	-0.042046148	0.167147249355105	0.232781288507334	1081
ANXA1	-0.042059675	0.167010593514634	0.232607052430331	1081
ASPDH	-0.042062572	0.166981342154328	0.232582392152315	1081
SGSM1	-0.042062968	0.166977345770273	0.232582392152315	1081
LCN10	-0.042097998	0.166623920886452	0.232196934577863	1081
LOC84856	-0.042111802	0.16648480249159	0.23203516993172	1081
PTPN20A	-0.042114163	0.166461013959242	0.232018067266929	1081
LOC22171	-0.042132417	0.166277200157972	0.231858115676773	1081
FAM181A	-0.042136908	0.166232008822035	0.23181114618025	1081
LOC10013	-0.042182207	0.165776615013695	0.231208107826841	1081
SCGB2A2	-0.042189967	0.165698691045333	0.23114743654236	1081
ANKFN1	-0.042202274	0.16557517735473	0.231023129783283	1081
FHL2	-0.042211436	0.165483269550935	0.230926881761124	1081
TOMM70A	-0.042213713	0.165460435407103	0.230911012904475	1081
CDR2	-0.042230634	0.165290809729281	0.230738227565406	1081
PHGR1	-0.04223862	0.165210806485095	0.23065603869041	1081
OR4C45	-0.042247371	0.165123158908441	0.230568103986313	1081
B3GNT6	-0.042249863	0.165098214489675	0.230549253493311	1081
PKD1L3	-0.042280579	0.164790901644001	0.23015201841503	1081
IFNE	-0.042287819	0.164718527108772	0.230066888206768	1081
OR2A14	-0.042293565	0.164661108423251	0.230002636887144	1081
CHN2	-0.042299174	0.1646050755541	0.229970220959711	1081
CADM4	-0.042370917	0.163889589616077	0.229099700424389	1081
SCARNA1	-0.04242424	0.163359319036169	0.228485279593392	1081
C20orf197	-0.042429665	0.163305436972437	0.228441637928598	1081
GOLGA1	-0.042479585	0.162810304190152	0.22781229269366	1081
FGFBP1	-0.04248323	0.162774195774232	0.227777589182543	1081
OPN1MW	-0.042495736	0.162650347976005	0.227651723752978	1081

KIF5A	-0.042510736	0.16250190164477	0.227507179695176	1081
ZNF30	-0.042548699	0.162126657553293	0.22704494378944	1081
C5orf22	-0.042550618	0.162107710455792	0.227034192653777	1081
LOC28579	-0.042553808	0.162076214344361	0.227005863725728	1081
LRP5	-0.042571591	0.161900707085652	0.226807353563315	1081
TRIM49	-0.042576866	0.161848673294688	0.226750227659885	1081
AGTPBP1	-0.042593237	0.161687257567946	0.226555596000992	1081
NKRF	-0.042605147	0.161569914125478	0.226438424937926	1081
OR5V1	-0.04261636	0.1614594926697	0.226299414167614	1081
IYD	-0.042622664	0.161397430846576	0.226243910959869	1081
ARMC3	-0.042629399	0.161331156154046	0.226166746048939	1081
EAF1	-0.042654212	0.161087154806295	0.225856120098331	1081
C2orf77	-0.042668395	0.160947804569519	0.225676447626711	1081
SCD5	-0.04270322	0.160606040884344	0.225228589879228	1081
TSSK6	-0.042709923	0.160540323274263	0.225152103338904	1081
SNORA44	-0.042772688	0.159925931371077	0.22435291695476	1081
KIAA1161	-0.042779911	0.159855342904516	0.224269509214532	1081
TUBGCP5	-0.042782492	0.15983012900031	0.224249752661321	1081
PHACTR2	-0.04279096	0.159747413042697	0.224164923080602	1081
ELAC1	-0.042799267	0.159666296078203	0.22406670390076	1081
RPL23	-0.042827108	0.15939467208043	0.223747869072556	1081
FAM153C	-0.042854652	0.159126287094401	0.223407449441667	1081
IQSEC3	-0.042863043	0.159044595395009	0.223318698977657	1081
FRMPD4	-0.042875531	0.158923076806898	0.223176600101281	1081
NEIL1	-0.042889859	0.158783737658969	0.223045700797707	1081
DNAJC22	-0.042903818	0.158648074091413	0.22290176123389	1081
TTLL4	-0.042915435	0.158535237222546	0.222758760818036	1081
C1orf173	-0.042943899	0.158259022525018	0.222386161325788	1081
STK3	-0.042945882	0.15823979736284	0.222374657682205	1081
KLF14	-0.042951331	0.158186966398853	0.222315922848116	1081
VIM	-0.042954342	0.158157781181127	0.222290413862681	1081
KCNK1	-0.042972234	0.157984432310597	0.222077760721301	1081
POU1F1	-0.042996253	0.157751941529202	0.221772535965306	1081

PIGV	-0.04301803	0.157541373349898	0.221518890121339	1081
CSRNP1	-0.043035574	0.157371894165155	0.221324826023252	1081
LASS1	-0.043039361	0.157335335075974	0.221288858835823	1081
C2orf57	-0.043050236	0.157230368205739	0.221156665794206	1081
ITPRIP	-0.043051619	0.157217023124123	0.22115333641753	1081
LYRM4	-0.043057372	0.157161512014201	0.22109068851439	1081
DHRS7C	-0.043077392	0.156968477628709	0.220834554216799	1081
ZNF518B	-0.043078745	0.156955445203085	0.220831641543243	1081
THUMPD1	-0.043092695	0.156821048940914	0.220657961228939	1081
KCNA5	-0.043100613	0.156744810087331	0.22056609382574	1081
ARSF	-0.043120952	0.156549091406906	0.220321464747249	1081
SLC12A1	-0.04313717	0.156393165623567	0.220148160260413	1081
CYSLTR1	-0.043166904	0.156107594627434	0.219792248655972	1081
ANKRD30	-0.043186352	0.155921030508881	0.219592763591709	1081
ERF	-0.043194647	0.155841502543532	0.219525003058489	1081
ENPP4	-0.043197569	0.155813497121995	0.219510269402299	1081
KIAA1683	-0.043199336	0.155796565921613	0.219510269402299	1081
RAGE	-0.043210456	0.155690026508696	0.219388331282714	1081
PPAPDC2	-0.043262415	0.155192962547311	0.218779722219425	1081
PEBP4	-0.043291731	0.154913042450955	0.218430968025093	1081
PLEKHM1	-0.043309166	0.154746753198608	0.218242323101789	1081
ZNF703	-0.043316798	0.154674001561424	0.218170267571411	1081
VPS37B	-0.043323188	0.154613106852182	0.218099645510623	1081
ZNF648	-0.043372501	0.154143803887003	0.21751379443147	1081
SYTL2	-0.043415114	0.153739139806376	0.21700357142653	1081
THOC5	-0.043415879	0.153731878343207	0.21700357142653	1081
SUOX	-0.043421307	0.153680391116671	0.21695104968783	1081
LGI1	-0.043462457	0.153290498042761	0.216446140258738	1081
C3orf17	-0.04349613	0.152972005228702	0.216041856795583	1081
ATP6V1A	-0.043525236	0.152697113723845	0.215683870142116	1081
USP27X	-0.043525304	0.152696479319824	0.215683870142116	1081
PAPSS2	-0.043526414	0.152686003396686	0.215683870142116	1081
NOS1	-0.043577339	0.152205968837047	0.215080614634001	1081

TDGF3	-0.043579631	0.152184393373535	0.215076665016857	1081
RGS8	-0.043583844	0.152144737173226	0.21503934121621	1081
HOMER2	-0.043612995	0.151870550081913	0.214666870011236	1081
BAZ1B	-0.043620139	0.15180341476415	0.214587031816982	1081
SLC1A6	-0.043630267	0.151708273046052	0.214497695151089	1081
TDRD12	-0.043659421	0.15143466330706	0.214140902170484	1081
TPSAB1	-0.043662189	0.15140870907368	0.214119230908339	1081
CHD7	-0.043663417	0.15139718882621	0.214117970296546	1081
TCF3	-0.043677646	0.151263810807693	0.213944356436213	1081
SYNPO2L	-0.043693833	0.15111219071186	0.213744915172759	1081
SNHG3-R(	-0.043735217	0.150725072900569	0.213287201010253	1081
CBWD3	-0.043752905	0.150559839686972	0.21306835057914	1081
WTAP	-0.043758685	0.150505875195938	0.213031470141476	1081
DEFB119	-0.043771842	0.150383095582619	0.212893004955155	1081
KIAA1598	-0.043806846	0.150056817398105	0.212468918080627	1081
ITPR3	-0.043813362	0.149996141363639	0.212419843077013	1081
LOC10027	-0.043821122	0.149923902195676	0.212332466938407	1081
FLJ42393	-0.043864536	0.149520246955247	0.211835246846715	1081
OR10G7	-0.043871273	0.149457679765335	0.211771062317069	1081
NOL4	-0.043923349	0.148974736126401	0.211151485208355	1081
BPHL	-0.043929333	0.148919319257616	0.21108779105296	1081
TOX3	-0.043931678	0.148897606195382	0.21107186523158	1081
RPLP0	-0.043934183	0.148874415007705	0.21105384168404	1081
GABRA6	-0.043938491	0.148834539984177	0.211012161867917	1081
TSPYL6	-0.043964141	0.148597274189212	0.210705433134489	1081
UBIAD1	-0.043966265	0.148577641626541	0.210692425071565	1081
EMR4P	-0.043970035	0.148542792428541	0.21065783551376	1081
REPS2	-0.043985068	0.148403905435796	0.210475688186716	1081
BAI1	-0.044005752	0.14821297895409	0.210219704360356	1081
TMPRSS5	-0.044032852	0.147963106566778	0.209880071946751	1081
ProSAPiP1	-0.044057369	0.147737330023539	0.209589331924239	1081
RAB3C	-0.044103207	0.147315917129081	0.209094564261631	1081
SNAPC1	-0.044115728	0.147200958396869	0.208960841864909	1081

DPY19L2I	-0.044128694	0.147081990716618	0.208806673924332	1081
SART3	-0.044131925	0.147052358168261	0.208779318859653	1081
DUSP1	-0.044145138	0.146931217133241	0.208636735684017	1081
DENND2A	-0.044146387	0.146919772088287	0.20863519023816	1081
PHRF1	-0.04415592	0.146832424710089	0.208525850954159	1081
MFSD6	-0.044158327	0.1468103746269	0.208509235537148	1081
PRR4	-0.044168836	0.146714143004642	0.208387253107842	1081
MRPL35	-0.044169053	0.146712159343019	0.208387253107842	1081
DAB2	-0.044176548	0.146643550351245	0.20831636145729	1081
C13orf30	-0.044184678	0.146569164214957	0.20822537469043	1081
C3orf55	-0.044191963	0.146502537718557	0.208145399699579	1081
OR10H5	-0.04419238	0.146498724489429	0.208145399699579	1081
ARHGEF4	-0.044199994	0.146429107960138	0.208070422505254	1081
PRICKLE4	-0.044224292	0.146207119344732	0.20778685686793	1081
FAM86B1	-0.044240276	0.146061225539657	0.207606250511281	1081
OAZ2	-0.04425796	0.145899951496462	0.20742092610418	1081
LOC64453	-0.044265324	0.145832834440596	0.207354774831013	1081
C16orf82	-0.044280827	0.145691603299154	0.2071832096895	1081
NSUN6	-0.044342756	0.145128503118312	0.206469893737174	1081
ADAMTS	-0.044349219	0.145069836453627	0.206415585206831	1081
DMRTA1	-0.044374367	0.14484172027574	0.206120122559677	1081
FLJ90757	-0.044382515	0.144767871298562	0.20602958440901	1081
MSTN	-0.044392968	0.144673167696355	0.205953004257161	1081
MAP3K3	-0.044414003	0.14448274222181	0.20575461911907	1081
GBF1	-0.044430669	0.144332003958443	0.205583555097421	1081
LRRC26	-0.044432981	0.144311104393307	0.205568321171205	1081
ATF6B	-0.044448031	0.144175096722382	0.205403629665657	1081
NKX6-1	-0.044449267	0.144163930002772	0.205402246969789	1081
KLHL10	-0.044464124	0.144029779427993	0.205225626437751	1081
WWP1	-0.044490378	0.143792945319189	0.204954043142137	1081
TEKT2	-0.044491846	0.143779713033827	0.204954043142137	1081
SIX5	-0.044503957	0.143670561913189	0.204844183575709	1081
SALL3	-0.044510207	0.143614268676969	0.204778414672815	1081

OR1C1	-0.044514092	0.143579279438342	0.204743015805577	1081
RUNX2	-0.044515395	0.143567548938336	0.204740781067731	1081
GEFT	-0.044529744	0.143438381924517	0.20457105868102	1081
KRT17	-0.044532483	0.143413739070784	0.204550394617739	1081
CBLN1	-0.044540654	0.143340240591925	0.204474518249846	1081
ZCCHC10	-0.044548846	0.14326657750181	0.204383910755184	1081
LOC28410	-0.044555535	0.143206455911845	0.204312610081028	1081
MOBKL3	-0.044637524	0.142471075297491	0.203393084251149	1081
TCEAL5	-0.044642298	0.142428344110136	0.203360903827251	1081
NR6A1	-0.044647892	0.142378284923549	0.203303838232556	1081
ZNF772	-0.044654531	0.142318897352445	0.203233443479479	1081
MEGF6	-0.044670698	0.142174350082515	0.203041420843065	1081
DEAF1	-0.044678617	0.142103586338471	0.202954749860936	1081
BTBD2	-0.044683853	0.142056812261806	0.202902331467248	1081
CDC37L1	-0.04468465	0.142049692236567	0.202902331467248	1081
SERPINE1	-0.044690942	0.141993509726871	0.202840678871636	1081
PGRMC1	-0.044712648	0.141799807830222	0.202607073461936	1081
ABHD3	-0.044731068	0.141635582738086	0.202401136004735	1081
DEFB108I	-0.044770173	0.14128743784267	0.201979660879901	1081
POLR3C	-0.044819641	0.140847964336542	0.201389895773983	1081
TUBB1	-0.044827761	0.140775929460368	0.201315475188409	1081
C11orf58	-0.044843441	0.1406369007139	0.201173781501208	1081
POTEF	-0.044892375	0.140203712036532	0.200639609219005	1081
PCDHGB1	-0.044912377	0.140026936305692	0.200415107060616	1081
NRBP2	-0.044928713	0.139882693078783	0.20026557117988	1081
LOC39132	-0.044929249	0.139877962462582	0.20026557117988	1081
C9orf156	-0.044944998	0.13973900602112	0.200116746733168	1081
EGF	-0.044956067	0.139641413421364	0.199991203851371	1081
FAM86D	-0.044957179	0.139631604189132	0.199991203851371	1081
FRMPD2L	-0.044959588	0.139610377009766	0.199975188058997	1081
ADAL	-0.0449768	0.139458739293055	0.199786396178253	1081
STT3A	-0.044989595	0.139346102266383	0.199639231217999	1081
SNORA8	-0.04501511	0.139121695998754	0.19933190369096	1081

C6orf103	-0.045077507	0.138574090359427	0.198662399167113	1081
CCR6	-0.045086062	0.138499135961081	0.19859528037127	1081
C5orf54	-0.045103418	0.138347169889997	0.198391496115746	1081
NCRNA00	-0.04515767	0.137872984292348	0.197824164428017	1081
FBXL20	-0.045175531	0.137717150182279	0.197628721357887	1081
WFIKKN1	-0.045178971	0.137687154951225	0.197599753258704	1081
ZNF474	-0.045181295	0.13766688288733	0.197584736090429	1081
KCNA2	-0.045183981	0.13764346922801	0.197579285135974	1081
GPR142	-0.04518541	0.13763101299353	0.197575483271906	1081
RNF175	-0.045189363	0.137596561216437	0.197540103029157	1081
KATNA1	-0.045194721	0.1375498646781	0.197487137334497	1081
NLRP4	-0.045226418	0.137273898220253	0.197175235625455	1081
TMEM101	-0.045244723	0.137114724169878	0.19697529180054	1081
C1orf66	-0.045261573	0.136968327576854	0.196792451254241	1081
DHX57	-0.045263672	0.136950099584664	0.196780296443156	1081
APOBEC2	-0.045265397	0.136935119818332	0.196772807528376	1081
CCNL1	-0.045273867	0.136861594775549	0.196681183246786	1081
ZDHHC8	-0.045279137	0.136815852167847	0.196629474346956	1081
ZDHHC1	-0.045283923	0.136774330866252	0.19658382529257	1081
HBBP1	-0.04531509	0.136504158392192	0.196265525002549	1081
OR1N2	-0.045320787	0.13645481132984	0.196208577747297	1081
MYH4	-0.045322059	0.136443797682274	0.196206745971119	1081
FAM19A3	-0.04535778	0.136134758534371	0.195776321435959	1081
SLAMF9	-0.045364039	0.136080663483227	0.195726471931288	1081
ZNF410	-0.045400869	0.135762691436744	0.195297016494802	1081
CNTNAP5	-0.045408961	0.135692904996422	0.195210567103687	1081
BCL11A	-0.045426247	0.135543929088134	0.195024101955608	1081
ABCA1	-0.045428702	0.135522780579071	0.195007601054671	1081
SMARCE1	-0.045459087	0.135261236708786	0.19468688293073	1081
LYRM2	-0.045475193	0.135122757708165	0.194501461524186	1081
NAPB	-0.045480195	0.135079779026334	0.194453490673538	1081
S1PR3	-0.045516921	0.134764504573135	0.194055106835297	1081
FAM169A	-0.045536086	0.134600209546087	0.193846241157129	1081

SNORA7E	-0.045563563	0.134364928594431	0.193548908583112	1081
RMI1	-0.045564272	0.134358855074545	0.193548908583112	1081
LRTOMT	-0.045605427	0.134007057262912	0.193074822526203	1081
FOXF2	-0.045606445	0.133998361793114	0.193074822526203	1081
ARHGEF1	-0.045626541	0.133826846644325	0.192842762922027	1081
SEL1L2	-0.045633029	0.13377150795426	0.19277681005355	1081
LOC72864	-0.045633851	0.133764496805709	0.19277681005355	1081
LOC22144	-0.04563882	0.133722132777018	0.192733230649855	1081
HEPH	-0.04567327	0.133428677010468	0.192365325869597	1081
ACTBL2	-0.045716382	0.133062142011867	0.191864351215308	1081
MALAT1	-0.045716982	0.133057045661778	0.191864351215308	1081
CD1C	-0.045721053	0.133022476577879	0.191848352710579	1081
STXBP5L	-0.045731355	0.13293502404823	0.191749682045796	1081
EIF2C3	-0.045742029	0.132844463709612	0.191637403386854	1081
DHFR	-0.045753552	0.132746746527848	0.191532961524387	1081
C17orf104	-0.045755214	0.132732658689156	0.19152635264616	1081
LOC65443	-0.045798128	0.132369278052248	0.191068185429594	1081
CHP	-0.04579857	0.132365537013245	0.191068185429594	1081
SGCG	-0.045808962	0.132277662898288	0.190965566797048	1081
TIGD2	-0.045823789	0.132152352826088	0.190798333525517	1081
ETS1	-0.04582921	0.132106563114763	0.190745894061561	1081
GNA14	-0.045832687	0.132077200173243	0.190730838529747	1081
LOC10013	-0.045851984	0.131914325180951	0.190509289610026	1081
LOC28331	-0.045854016	0.131897183809124	0.190498190983281	1081
NACAP1	-0.045873868	0.131729814281794	0.190270101721283	1081
NEFH	-0.045875734	0.131714090111329	0.19026103149955	1081
RPSAP52	-0.045893082	0.131567971293587	0.190118125077779	1081
RFFL	-0.045894655	0.131554727384558	0.190112624330124	1081
MPL	-0.045898927	0.13151877041613	0.19007675915371	1081
ZNF673	-0.045983495	0.130808474168124	0.189197064339235	1081
HCFC1	-0.045992825	0.130730300803275	0.18910756579987	1081
TACR3	-0.046002767	0.13064703239572	0.18900427056458	1081
BBS4	-0.046013368	0.130558283728502	0.188889444535383	1081

CDK12	-0.046026922	0.130444893256903	0.188738948118963	1081
ADCY1	-0.046048949	0.130260771128329	0.188553799984205	1081
SPG20	-0.0461381	0.129517633976828	0.18762640129895	1081
DENND4I	-0.046151916	0.129402756156839	0.187473464421397	1081
NSUN4	-0.046199834	0.129004960609518	0.186964383559622	1081
PCDH10	-0.046221112	0.128828628360991	0.186762573282395	1081
PMS2L4	-0.046255773	0.128541780430151	0.186386969464146	1081
ASFMR1	-0.046264728	0.128467754633712	0.186293040389875	1081
SLC38A9	-0.046265281	0.128463180485171	0.186293040389875	1081
EGLN1	-0.046293705	0.128228432760087	0.185972770190925	1081
C21orf130	-0.046301718	0.12816231638625	0.185890263057168	1081
KBTBD11	-0.04631178	0.128079331700597	0.185783276001478	1081
PEG10	-0.046315726	0.128046797467301	0.185749458844875	1081
MRPL45	-0.046327718	0.127947963034002	0.185646190962256	1081
SCML2	-0.046342111	0.127829419287306	0.185500911363116	1081
CYP39A1	-0.046367608	0.127619636387751	0.185223168229917	1081
COX7A1	-0.046371684	0.127586128649806	0.185201222197028	1081
IL17B	-0.04639943	0.127358189577494	0.184910322813594	1081
CADM3	-0.046424368	0.127153590680793	0.184679818620373	1081
TTLL3	-0.04646613	0.126811540912339	0.184249440438447	1081
BZRAP1	-0.046482378	0.126678654164917	0.184069639941738	1081
HCG22	-0.046489057	0.126624054798235	0.184003576708537	1081
C19orf71	-0.046492079	0.126599363853482	0.183980968462589	1081
MTMR11	-0.046497653	0.126553818083961	0.183941317842813	1081
C1orf186	-0.046503731	0.126504179843866	0.183882437618492	1081
NHLH2	-0.046515127	0.126411134424765	0.183760449053752	1081
LYRM5	-0.04653218	0.126272008151413	0.183604580348062	1081
PCDHB13	-0.046537669	0.126227255967979	0.183572630051613	1081
CABYR	-0.046545826	0.126160769925997	0.183515684176418	1081
ANKLE2	-0.046551522	0.126114356521974	0.183461417687404	1081
ATP6V0A	-0.046565044	0.126004226360704	0.183314446453122	1081
STS	-0.046568929	0.125972599360291	0.183281670815619	1081
CNTFR	-0.046573973	0.125931552114221	0.183235183628817	1081

MYCN	-0.046580632	0.125877367593391	0.183169573112457	1081
PPP1R3E	-0.046592301	0.125782467980626	0.183097609471039	1081
PLA2G2A	-0.046592779	0.12577857999266	0.183097609471039	1081
TMEM19	-0.046602837	0.125696827349375	0.182999391989099	1081
C10orf46	-0.046611932	0.125622937377981	0.182905035666336	1081
MUCL1	-0.046647354	0.12533548776487	0.182526089859272	1081
FAM26D	-0.046666902	0.125177070022553	0.182321746482852	1081
VTI1A	-0.046714252	0.124794001221667	0.181829535267647	1081
HOTAIR	-0.046730205	0.124665143755655	0.181707497356029	1081
MSTO2P	-0.046748941	0.124513938377626	0.181526508077672	1081
STEAP3	-0.046758687	0.124435343855667	0.181425056233075	1081
LYRM1	-0.046776673	0.124290393778913	0.181266197254485	1081
LOC28457	-0.046791215	0.124173301036342	0.181133389454918	1081
BANF2	-0.0468164	0.123970714525026	0.180878543065593	1081
GFRAL	-0.046816594	0.123969147910824	0.180878543065593	1081
GYPE	-0.046845899	0.123733742263834	0.180572025348083	1081
SV2A	-0.046851456	0.123689147037351	0.180520022246427	1081
LRRTM4	-0.046851783	0.123686521572879	0.180520022246427	1081
NGF	-0.046851876	0.123685778299471	0.180520022246427	1081
JMJD5	-0.046910849	0.123213248242729	0.179942792942567	1081
SMU1	-0.046911877	0.123205017822442	0.179942792942567	1081
MRPS35	-0.046922222	0.123122275990772	0.179849050093111	1081
SDCBP	-0.046959496	0.1228245129735	0.179519885548844	1081
SCGBL	-0.046978932	0.122669471834113	0.179330661110175	1081
LOC10013	-0.046984884	0.122622019238886	0.179274299845225	1081
QRSL1	-0.047014988	0.122382256246216	0.178947017177454	1081
SERINC3	-0.047029586	0.122266118894191	0.178792898165176	1081
AQP5	-0.047046536	0.122131375629312	0.178612024983132	1081
HPS4	-0.047051569	0.122091393336987	0.178581450230669	1081
TRPM4	-0.047067918	0.121961573745467	0.178425265294295	1081
RXRB	-0.04706971	0.121947352805542	0.178417417551576	1081
NLE1	-0.04707177	0.121931000194046	0.178406449659286	1081
HDAC7	-0.047087426	0.121806812871061	0.178237687607142	1081

ZBTB46	-0.047110722	0.121622200690036	0.177980476020687	1081
RRP1B	-0.047142679	0.121369307092656	0.177636202512464	1081
PDZD4	-0.047175277	0.121111763742008	0.177323677635203	1081
LOC64136	-0.047189453	0.12099989839054	0.177224294974366	1081
FAM22G	-0.047189877	0.12099655164741	0.177224294974366	1081
RNF123	-0.04722617	0.12071053398714	0.176851905976068	1081
B4GALT5	-0.047248651	0.120533632866189	0.176618419703912	1081
MMP20	-0.047288193	0.120222963685811	0.176176009562135	1081
ARL17B	-0.047306549	0.120078960472959	0.176003394843405	1081
MFGE8	-0.047307559	0.120071047310934	0.176003394843405	1081
ZCCHC13	-0.047317432	0.119993650405028	0.175909106000023	1081
HNRNPA(	-0.047317849	0.119990379643863	0.175909106000023	1081
DTNA	-0.047330994	0.119887406087816	0.175812171190148	1081
DUSP22	-0.047343201	0.119791833208218	0.175723183570928	1081
SERPINF1	-0.047351069	0.119730261629244	0.175658445890403	1081
LRRTM3	-0.047361951	0.119645155262332	0.175546369465381	1081
ATRNL1	-0.047364204	0.11962753905411	0.175533307177874	1081
EFCAB7	-0.047383929	0.119473404506568	0.175332682580666	1081
TAS2R8	-0.047396236	0.119377309722858	0.175217187932021	1081
PHACTR1	-0.047418016	0.119207397293548	0.174980546377042	1081
C6orf138	-0.04745582	0.118912933673128	0.174573753741813	1081
GPR37	-0.047463181	0.118855664417612	0.174502394846789	1081
ZNF79	-0.047466876	0.118826930037318	0.174472923148817	1081
C14orf33	-0.047477731	0.118742529562248	0.174363466922681	1081
CLEC2L	-0.047480527	0.11872079968782	0.174355216863099	1081
POTED	-0.047487516	0.118666490279742	0.17431358077041	1081
SFMBT1	-0.047516673	0.118440139243625	0.174006461862808	1081
DEFB109I	-0.047537114	0.118281650298408	0.173798967561009	1081
POLD3	-0.047537841	0.118276018592295	0.173798967561009	1081
SERPINB	-0.047587183	0.117894146699034	0.173335080317667	1081
SSC5D	-0.047599785	0.11779677106148	0.173225487483284	1081
SLC2A9	-0.047614506	0.117683104028518	0.173108881391346	1081
WDR75	-0.04761635	0.117668868638282	0.173100581182867	1081

SENP8	-0.047634422	0.11752946040387	0.172908126768127	1081
C1orf25	-0.047681711	0.117165269300084	0.172447895817212	1081
ZNF492	-0.047692567	0.11708178749684	0.17233761574776	1081
RPSA	-0.047761798	0.116550511946113	0.171618307371862	1081
FAM150A	-0.047779009	0.116418735014573	0.171436799478697	1081
ADIG	-0.047803323	0.116232763996579	0.171187968322203	1081
LRG1	-0.04781116	0.116172870779183	0.1711122674451	1081
RFPL1S	-0.047844136	0.115921123000572	0.170778925175261	1081
C11orf63	-0.047870756	0.115718207288736	0.170529867288338	1081
SNAR-G1	-0.047895344	0.115531035273613	0.170278951242826	1081
SMAD6	-0.047926467	0.115294448664065	0.169955119884209	1081
PPA2	-0.047930878	0.115260951906163	0.169927445122985	1081
OXCT2	-0.04793418	0.115235877240879	0.169906078241786	1081
DHH	-0.047962187	0.11502339461618	0.169642454392192	1081
FAM175B	-0.047962445	0.115021438831712	0.169642454392192	1081
C4orf40	-0.047971143	0.114955510884068	0.169567164586626	1081
FNDC3B	-0.047974892	0.114927106505807	0.169537680181568	1081
NAGS	-0.048000695	0.114731737432819	0.169286665977011	1081
OBP2A	-0.048010894	0.11465459550573	0.169185234871296	1081
GNRH1	-0.048019492	0.114589587952214	0.169101695794986	1081
C12orf76	-0.048019671	0.114588237277507	0.169101695794986	1081
TCERG1L	-0.04803157	0.114498315366804	0.168991762258354	1081
SOHLH2	-0.048041508	0.114423267850014	0.168893371251358	1081
BET1	-0.048091648	0.114045187560356	0.168384660173217	1081
C20orf166	-0.048093131	0.114034020429616	0.168380513197582	1081
SLC9A10	-0.048123763	0.113803542928594	0.168114128642408	1081
ZNF766	-0.048142639	0.113661700851304	0.167916908451857	1081
FASN	-0.048151067	0.113598407989584	0.16784802178012	1081
TMEM213	-0.048153195	0.11358243937502	0.16783673741728	1081
FGGY	-0.048158047	0.113546015105967	0.167795222587274	1081
MARVEL[illegible]	-0.048163472	0.113505308926985	0.167747373511416	1081
OR2L8	-0.048168488	0.113467679795766	0.167728676950815	1081
ARG2	-0.048178902	0.113389589699836	0.167625543736934	1081

CYP1A2	-0.048199125	0.113238064977734	0.167413827900811	1081
TMEM154	-0.048216	0.113111749846008	0.167239354175987	1081
TREH	-0.048281391	0.112623321462863	0.166590558107745	1081
TTLL1	-0.048357386	0.112057771298724	0.16582706257348	1081
TOB1	-0.048367535	0.111982412290438	0.165747326607358	1081
SNHG4	-0.048373103	0.111941084756725	0.165703075348977	1081
FDPSL2A	-0.048375025	0.111926823214531	0.165694139745497	1081
OPN1SW	-0.048392699	0.11179573076152	0.165512236068996	1081
ZNF385D	-0.048403595	0.111714979505155	0.1654169983191	1081
UBA5	-0.048406793	0.111691285776315	0.165394071740949	1081
LOC22042	-0.048434151	0.111488763194423	0.165142730481739	1081
CSMD2	-0.048437421	0.111464573546328	0.16511904067143	1081
CELF5	-0.048438149	0.111459191150363	0.16511904067143	1081
MFN1	-0.048446019	0.111400997467126	0.165049135395695	1081
SIKE1	-0.048458916	0.111305678854615	0.16492004269831	1081
TPPP	-0.04848004	0.111149708446828	0.164713173213282	1081
HOXB1	-0.048544023	0.110678315098377	0.164087036919105	1081
AADACL4	-0.048552398	0.11061672997038	0.164007803433672	1081
IL6R	-0.048556939	0.110583348205492	0.163970377565113	1081
MAPK8IP	-0.048575534	0.110446740049699	0.163803988684473	1081
COL4A1	-0.048580964	0.110406874551132	0.163756920028903	1081
NODAL	-0.048584626	0.110379999961921	0.163729114147184	1081
RHOT1	-0.048592911	0.11031920720768	0.163667378774089	1081
CST2	-0.04860084	0.110261049888062	0.163612908809297	1081
TP53TG5	-0.048627255	0.110067473692808	0.163385841687541	1081
RSU1	-0.048652714	0.109881164759739	0.163121301701175	1081
SSH3	-0.048663696	0.109800871187919	0.163014116742621	1081
ELF5	-0.048691398	0.109598547967802	0.162725733255555	1081
PRL	-0.048713867	0.109434658881917	0.162518334181808	1081
WDR12	-0.048715647	0.10942167853675	0.162518334181808	1081
ZNF219	-0.048722618	0.10937087682421	0.162471522278867	1081
HIC1	-0.048723453	0.109364790004468	0.162471522278867	1081
OR5P3	-0.048730084	0.109316491344694	0.162414685311517	1081

CCDC144(	-0.04874848	0.109182561538121	0.162251600928405	1081
DCTN6	-0.048755171	0.109133882993022	0.162191226329356	1081
SLITRK1	-0.048756339	0.109125392012693	0.162190572679334	1081
C11orf70	-0.048795328	0.108842091643638	0.161829207776299	1081
KCNJ11	-0.048822229	0.108646967347802	0.161590664745193	1081
REEP3	-0.048827107	0.108611619914596	0.161569259948496	1081
TM4SF20	-0.048844286	0.108487187031708	0.161396823185419	1081
OR2T4	-0.048883215	0.108205639356939	0.161025530386758	1081
DYNC1LI	-0.048890069	0.10815612759252	0.160970275489836	1081
CNGA4	-0.04889412	0.108126870771445	0.160943978992963	1081
EFS	-0.04892719	0.107888283131148	0.160624452270858	1081
IL8	-0.048927563	0.107885597170865	0.160624452270858	1081
MMP2	-0.048955259	0.107686107713226	0.160347153513412	1081
NPFFR2	-0.048981345	0.107498478900742	0.160091436198377	1081
H1FNT	-0.049004122	0.107334864655547	0.159883233398847	1081
C1orf110	-0.049021927	0.107207100144578	0.159704727679696	1081
C1orf89	-0.049023348	0.107196913625409	0.159701362593097	1081
ZNF878	-0.049061999	0.106920001234178	0.159335953903131	1081
SCAP	-0.049064422	0.106902662643127	0.159333688883234	1081
LOC72960	-0.04907097	0.106855807121945	0.159287423003669	1081
PROM2	-0.049073504	0.106837686379116	0.159272196559409	1081
ULBP3	-0.049080636	0.106786682003922	0.15923918633157	1081
C14orf178	-0.049114147	0.106547289948911	0.158898071958899	1081
SLC22A11	-0.049119555	0.106508695895312	0.15887579998601	1081
PIK3IP1	-0.049134449	0.10640246911114	0.158740853161809	1081
SYT1	-0.04914331	0.106339306738078	0.158658371666315	1081
SLC34A2	-0.049156018	0.106248778163012	0.158535044522174	1081
NEK6	-0.049171543	0.106138262833638	0.158393607288216	1081
CA4	-0.04917215	0.106133940112337	0.158393607288216	1081
LDLRAD2	-0.049175876	0.106107433573939	0.158371063891754	1081
HSD17B2	-0.049178733	0.106087109075912	0.158352460902063	1081
LOC33879	-0.049199375	0.105940366127912	0.158145140840814	1081
VWC2	-0.049200004	0.105935898785213	0.158145140840814	1081

NRARP	-0.049208123	0.105878224291459	0.158075804369039	1081
TSG1	-0.049214653	0.105831856488963	0.158018288539147	1081
SYCP2	-0.049243773	0.105625286657969	0.157721547678065	1081
CHP2	-0.049306809	0.105179213617558	0.157136996316057	1081
ZSWIM4	-0.049319516	0.105089474830093	0.157026217953733	1081
GFPT1	-0.049323558	0.105060944860096	0.156995232861556	1081
TAC4	-0.049339576	0.104947934027568	0.156849627641913	1081
SH3BP5	-0.049374271	0.104703475253735	0.156519108710782	1081
C12orf72	-0.049401657	0.104510838533957	0.156254329988613	1081
POLR2J4	-0.049442124	0.104226705358458	0.155898944122819	1081
SP9	-0.049443449	0.104217411351452	0.155896617782356	1081
PRPF18	-0.049450163	0.104170332450949	0.155849338820971	1081
KRT40	-0.049504263	0.103791594008757	0.155328850851082	1081
C6orf118	-0.049538347	0.103553546661515	0.154984115712943	1081
C6orf165	-0.049558611	0.10341222104285	0.154784099331913	1081
FUBP1	-0.049558661	0.103411873560722	0.154784099331913	1081
LOC10030	-0.049568214	0.103345300767789	0.154706924572127	1081
ICA1	-0.049569558	0.103335938956686	0.154704406233831	1081
SUPT16H	-0.049671012	0.102631103579451	0.153774903801267	1081
BOK	-0.049679838	0.102569967644695	0.153701980330097	1081
GALNT3	-0.049715316	0.102324512240537	0.153395393591727	1081
LOC10019	-0.049724492	0.102261103079823	0.153323155815944	1081
ACSM4	-0.049731564	0.102212256930824	0.153272734544548	1081
FBXL21	-0.04976548	0.101978241576558	0.152933200071448	1081
OR2T5	-0.049830201	0.101532872592779	0.15232199273023	1081
FAM123B	-0.049835303	0.101497824470733	0.152280753255599	1081
POM121L	-0.049846382	0.101421765692111	0.152177973325457	1081
EMP2	-0.049848093	0.101410018272996	0.152171681050988	1081
ENPP6	-0.049853385	0.101373705425865	0.152128523227357	1081
ISOC1	-0.049858228	0.101340483021606	0.152089997055073	1081
SLC5A2	-0.049878272	0.101203072700728	0.151906407835195	1081
CD34	-0.049878909	0.101198708887129	0.151906407835195	1081
PPPDE1	-0.049890034	0.10112250759636	0.151808101611795	1081

C9orf170	-0.049914089	0.100957902019626	0.151594881945838	1081
LOC4008(	-0.04991479	0.100953111922827	0.151594881945838	1081
NUDCD3	-0.049933771	0.10082337610604	0.151426743581315	1081
ZNF273	-0.049935376	0.10081241269039	0.151421566779593	1081
AKAP1	-0.049938779	0.100789169320466	0.151397943178048	1081
ANKRD11	-0.049940124	0.10077998475248	0.151395435707585	1081
PGC	-0.049957949	0.100658320067867	0.151246502406741	1081
DIO2	-0.049968112	0.100589004090252	0.151153624302411	1081
MAP2	-0.049969983	0.100576244245202	0.15114572475342	1081
CDH2	-0.05003421	0.100139109201149	0.15053371799546	1081
C8orf84	-0.050040024	0.100099614078622	0.150485576538346	1081
ADCY10	-0.050087698	0.0997762217742159	0.150032991538522	1081
LOC10028	-0.050116976	0.0995780249591424	0.149768501777059	1081
PAX1	-0.050118511	0.0995676450181135	0.149764071447017	1081
C1QTNF4	-0.050149676	0.0993570417389891	0.149491941581293	1081
FAM75C1	-0.050193085	0.0990642816350475	0.149118280918929	1081
FAM162B	-0.050196227	0.099043122613154	0.149097571741293	1081
ZFP36	-0.050216491	0.0989067183925432	0.148914487445275	1081
FAM7A3	-0.050219476	0.0988866340431691	0.148895376545305	1081
NRXN2	-0.050236798	0.0987701745814361	0.148731138207731	1081
NDP	-0.050264099	0.0985868434369492	0.148477269814394	1081
C21orf121	-0.050272315	0.098531722575885	0.148405349629249	1081
PADI2	-0.05029825	0.098357888622389	0.148165682068199	1081
ZBTB7A	-0.050299348	0.0983505333538269	0.148165681604311	1081
LOC9131(	-0.050370646	0.0978739434345001	0.147491815566128	1081
ABCA17P	-0.050371155	0.0978705464293691	0.147491815566128	1081
BAP1	-0.050385834	0.0977726604375094	0.147372259222868	1081
ZNF250	-0.050425128	0.0975110067388664	0.147043882832133	1081
GPR39	-0.050449743	0.097347381015252	0.14683011309915	1081
DDX47	-0.050479127	0.0971523432048533	0.146557881822807	1081
PDGFRL	-0.050487711	0.0970954226111695	0.146504927608569	1081
LTBP3	-0.050512386	0.0969319569466475	0.146269233909379	1081
SS18L1	-0.050547904	0.0966970497871694	0.145958494527388	1081

MYST2	-0.050552981	0.0966635074048653	0.145918797802249	1081
GJA3	-0.050555839	0.0966446284299288	0.145901231993474	1081
ZNF767	-0.050559021	0.0966236164192086	0.145880443136098	1081
C4BPA	-0.050564785	0.0965855564968696	0.145833910630298	1081
RBM6	-0.050589172	0.0964246765733437	0.145601912118283	1081
TMPRSS1	-0.050609669	0.096289623873945	0.14541978206317	1081
KLK5	-0.050615586	0.0962506670561927	0.145382746333283	1081
ATOH8	-0.05062735	0.096173249278501	0.145276702663123	1081
CXADRP2	-0.05062962	0.096158317974825	0.145265040540146	1081
ZER1	-0.05062966	0.0961580532195374	0.145265040540146	1081
MFAP5	-0.050631135	0.0961483511370586	0.145265040540146	1081
C9orf131	-0.050662859	0.0959398723018625	0.144967651329209	1081
AP1S3	-0.050663839	0.0959334373337987	0.144967651329209	1081
CPXM1	-0.050676595	0.0958497143201337	0.144853150411035	1081
DPH3B	-0.050695264	0.0957272835031488	0.144678980131363	1081
DMRTC1	-0.050696857	0.0957168452361699	0.144674058169466	1081
KHNYN	-0.050723438	0.0955427662975089	0.144432614967981	1081
LIN54	-0.050724255	0.0955374152115985	0.144432614967981	1081
USP17L6P	-0.050725034	0.0955323195973005	0.144432614967981	1081
KIAA0408	-0.05073222	0.0954853067517585	0.144378255986953	1081
SLC22A5	-0.050737268	0.0954522874424336	0.144339163078203	1081
ZNF684	-0.050772282	0.0952235412372195	0.144014882749534	1081
PCSK9	-0.050774594	0.0952084520201298	0.144002873034426	1081
UBFD1	-0.05077738	0.0951902730754408	0.143986187948998	1081
OR8S1	-0.050799053	0.0950489381337981	0.143783198821458	1081
MED9	-0.050825732	0.0948751914436846	0.143585059847722	1081
PLK1S1	-0.050840323	0.0947802737466211	0.143473746214734	1081
LIPG	-0.050851835	0.0947054449045448	0.143382022215696	1081
SBK1	-0.050857219	0.0946704634008211	0.143339833536982	1081
LRRC36	-0.05086375	0.0946280397363758	0.143286369549706	1081
ENPEP	-0.050927704	0.0942134427183694	0.142690760360992	1081
DLG4	-0.05094344	0.0941116513975646	0.142568748488791	1081
NDUFA10	-0.050968232	0.09395146091631	0.142368901772158	1081

DLD	-0.050974439	0.0939113893867492	0.142318885067032	1081
TMED5	-0.050982922	0.0938566496169503	0.142257332520763	1081
C20orf79	-0.050991486	0.0938014094127759	0.142195002830941	1081
NUP188	-0.050992276	0.0937963138332367	0.142195002830941	1081
NAP1L5	-0.051016654	0.0936392213429662	0.141991879108322	1081
RNF14	-0.05105728	0.0933778887159131	0.141670249147618	1081
MLPH	-0.051128182	0.0929232069255733	0.141033526973229	1081
MIR17HG	-0.051145448	0.0928127572529239	0.140908357439155	1081
SEPN1	-0.051174343	0.0926281422987556	0.140649274636951	1081
ZNF322B	-0.051210362	0.0923984296413377	0.140362061246541	1081
ZFAND1	-0.051310685	0.0917610146114819	0.139427186555159	1081
TANC2	-0.051328418	0.0916487137117028	0.139288067727066	1081
LCE1C	-0.051393112	0.0912399456498812	0.138698211976823	1081
C2orf58	-0.05141565	0.0910978864172263	0.138513617773041	1081
NDE1	-0.051433366	0.0909863457773381	0.138354463744299	1081
JARID2	-0.051451188	0.090874242742741	0.138225733921211	1081
FAM54B	-0.051574106	0.0901041221527327	0.137157891851806	1081
VWF	-0.051578049	0.0900795001375685	0.137130773843521	1081
NUDT15	-0.051616907	0.0898371928028391	0.136772237682376	1081
RHOU	-0.05161942	0.0898215383567986	0.136758740095058	1081
LRRC43	-0.051632417	0.0897406217762843	0.136645867398583	1081
KIAA0802	-0.051633132	0.089736167271255	0.136645867398583	1081
CNNM4	-0.051635569	0.0897210034913084	0.136636650690385	1081
CCDC68	-0.051637928	0.0897063250237657	0.136624625206302	1081
ARHGAP1	-0.051694588	0.0893543547032576	0.13614002991205	1081
FXYD6	-0.051702178	0.0893072896426981	0.136078613558139	1081
SNORA59	-0.051711126	0.0892518352614595	0.136004404034955	1081
AGBL3	-0.051723532	0.0891749852541154	0.135907858824645	1081
NOC3L	-0.051730911	0.0891293059186051	0.135858797497753	1081
GALR1	-0.051734359	0.0891079616315051	0.135836540829865	1081
SNX14	-0.051750063	0.0890108222888861	0.135708999849361	1081
PAPOLB	-0.051755981	0.0889742365272995	0.135663487348051	1081
FSTL5	-0.05179053	0.0887609014334891	0.135358694880972	1081

C6orf195	-0.051794043	0.0887392320051802	0.135335894370835	1081
OLFML1	-0.051803565	0.0886805168771715	0.135266829142926	1081
C6orf163	-0.051810829	0.0886357459337387	0.135219015663398	1081
FZD6	-0.051817083	0.0885972203811747	0.135170478989606	1081
TNKS1BP	-0.051843156	0.0884367279109147	0.134946059973139	1081
NUDT17	-0.05185108	0.0883880036771131	0.134881928192973	1081
KRT32	-0.051911665	0.0880161477588867	0.134355178949971	1081
GAD1	-0.051934174	0.0878783179075653	0.134165116653877	1081
LOC2857	-0.051954132	0.087756251205894	0.133988910151791	1081
TRIM56	-0.052055846	0.0871362714468842	0.133133120083237	1081
PCDHAC2	-0.052105268	0.0868363082824847	0.132684877908879	1081
DDX20	-0.05211845	0.0867564448494362	0.13257290309428	1081
LOC1001	-0.052130316	0.0866846020015865	0.132493270661757	1081
FBXW12	-0.052133698	0.0866641319629142	0.132482086689399	1081
DYRK2	-0.052135124	0.0866555008074335	0.132478945417451	1081
PPP1R14C	-0.052162066	0.0864926047233874	0.132239945518567	1081
CMPK1	-0.052179837	0.0863852903343656	0.132085895543514	1081
S100B	-0.052180846	0.0863792052979382	0.132085895543514	1081
DNAJC25	-0.052245873	0.0859874668821788	0.131527526221829	1081
ELFN1	-0.05225945	0.0859058544059162	0.131422648618407	1081
EPRS	-0.052261056	0.0858962077345692	0.131417870799187	1081
ASAH2B	-0.052264548	0.0858752279834745	0.131395751764172	1081
SULT1C3	-0.052272479	0.0858275953249528	0.13133284525797	1081
AMIGO2	-0.052291368	0.0857142473665133	0.131179329423263	1081
ASPG	-0.05229765	0.0856765747695976	0.131141600086129	1081
TMED8	-0.05231567	0.0855685892869727	0.130986263767938	1081
ITPRIPL1	-0.052352486	0.0853483044439057	0.130668915718383	1081
UBA2	-0.052366512	0.085264507528649	0.130560467023764	1081
TTC22	-0.052386382	0.0851459005276949	0.130388762153585	1081
ANTXRL	-0.052395693	0.0850903698665109	0.130313630907774	1081
WNT11	-0.05240408	0.0850403734166841	0.130246964379161	1081
TCF7L1	-0.05241644	0.0849667408593245	0.130153980276108	1081
OR6N1	-0.052419525	0.0849483652594806	0.13013572761614	1081

MBL2	-0.05243386	0.0848630420442666	0.130044574926346	1081
SELP	-0.052453055	0.0847489002674132	0.129909191590857	1081
KIF12	-0.052485751	0.0845547666716731	0.129641203638089	1081
KIAA1467	-0.052486449	0.0845506218408279	0.129641203638089	1081
GOLGA2I	-0.052498246	0.0844806670668474	0.129557174030271	1081
GDF1	-0.052501651	0.084460488050309	0.12953608921001	1081
SLU7	-0.052503494	0.0844495638786013	0.129529196309915	1081
AP2B1	-0.052503844	0.0844474890237241	0.129529196309915	1081
KBTBD10	-0.052522589	0.0843364661859239	0.129385279972236	1081
SEMA5B	-0.052532619	0.0842771027748263	0.129304054485824	1081
TMEM20	-0.052548669	0.0841821878846287	0.129168266809037	1081
SYT14	-0.052586211	0.083960516251965	0.128867398087302	1081
HNRPDL	-0.052592851	0.0839213555427577	0.128817106629751	1081
INPP5A	-0.052597914	0.0838915070639804	0.128781102629268	1081
TMEM68	-0.052619752	0.083762864762999	0.128603224651369	1081
RPS5	-0.052645666	0.083610412673968	0.128398518319644	1081
NXF1	-0.052650404	0.0835825657691203	0.128375326884639	1081
CHRNA3	-0.052669801	0.0834686303943838	0.128228510997975	1081
TULP1	-0.052684439	0.0833827344425659	0.128107472951914	1081
PCDHA11	-0.052685243	0.0833780209815145	0.128107472951914	1081
PDPN	-0.05271756	0.0831886435508557	0.127848277718339	1081
C9orf47	-0.052720533	0.083171237984053	0.127841033815714	1081
OR10J3	-0.052722432	0.0831601262728874	0.127833708032607	1081
EID1	-0.052724618	0.0831473284904743	0.127823789105663	1081
SLC6A11	-0.052734086	0.0830919389919639	0.127748386704809	1081
PHTF2	-0.05274091	0.0830520290457046	0.127706520504215	1081
RBM18	-0.052748882	0.0830054304098813	0.127644610351684	1081
FLJ44606	-0.052768391	0.0828914813146566	0.127498579146656	1081
MGRN1	-0.052834286	0.0825075285392065	0.126927389264782	1081
WBP11P1	-0.052843309	0.0824550682539432	0.12685637314615	1081
TDG	-0.052872366	0.082286309586236	0.126616079026484	1081
PMS2L3	-0.052911332	0.0820604360847463	0.126345726454235	1081
NR4A1	-0.052911492	0.0820595104701075	0.126345726454235	1081

DCDC2	-0.052913997	0.0820450035136408	0.12634127776963	1081
TCEB3B	-0.052954462	0.0818110134188768	0.126029124823987	1081
LOC15738	-0.05297032	0.0817194558489957	0.125897708632458	1081
MTMR7	-0.052989738	0.0816074662207659	0.125754027921466	1081
AGXT2L1	-0.052993566	0.0815854002665204	0.125729642623092	1081
TDP1	-0.053008219	0.081500988375995	0.125609166156715	1081
OR6A2	-0.053048237	0.0812708226273369	0.125278650456894	1081
TUFT1	-0.0530747	0.0811189065552835	0.125058572936113	1081
CYP2C8	-0.053118572	0.0808675622791004	0.124699712347863	1081
VAMP2	-0.053121406	0.0808513475164826	0.124684252906648	1081
CDH4	-0.053134502	0.0807764535810264	0.124587830747246	1081
KRT16	-0.053141475	0.0807365945420176	0.124535887982308	1081
PPRC1	-0.053145107	0.0807158420241017	0.124513411254731	1081
LIPK	-0.053173586	0.080553270405749	0.124272142159888	1081
ABCC11	-0.053197653	0.0804160934549701	0.124089023565712	1081
VIP	-0.053203708	0.0803816072871719	0.124064318020233	1081
MS4A7	-0.053208125	0.0803564605816403	0.124035008690303	1081
RAD51AP	-0.053279382	0.0799516535205936	0.123457462837066	1081
TSC22D3	-0.053298598	0.0798427761320102	0.123298790738164	1081
CLINT1	-0.053301137	0.0798283956571031	0.123295486507118	1081
CYP1B1	-0.053308802	0.0797850067576973	0.123237920651266	1081
IL5RA	-0.0533589	0.0795018770963941	0.12281942592844	1081
TPTE2P3	-0.053384613	0.0793568757728139	0.122604821109237	1081
CXCL2	-0.053425512	0.0791266795498786	0.122279658338695	1081
CCDC73	-0.053435371	0.0790712703672825	0.122219806763516	1081
KRT6B	-0.053437409	0.0790598201057417	0.12221148526935	1081
C14orf129	-0.053442008	0.0790339894706033	0.122190308356508	1081
CDC20B	-0.05345585	0.0789562781213945	0.122079532025136	1081
ZNF490	-0.053484041	0.0787981981605439	0.121863173801947	1081
SYNGR1	-0.053493105	0.0787474330826905	0.121794014545167	1081
POMT2	-0.053494189	0.0787413617937656	0.121793975225385	1081
SPATA1	-0.053507735	0.0786655390489179	0.121695383515624	1081
SCARF2	-0.053523169	0.0785792240026699	0.121580527460352	1081

CLIC5	-0.053538003	0.0784963343657847	0.121470936841199	1081
ASB15	-0.053543736	0.0784643219593493	0.121434034230537	1081
ADARB2	-0.053551401	0.078421530231686	0.121383152248737	1081
TNFRSF1(	-0.05358866	0.0782138119985038	0.121070942270621	1081
PHKA1	-0.053592956	0.0781898898156979	0.12104321296682	1081
TEX10	-0.053626357	0.0780041084348021	0.120764891209583	1081
PPP2R5A	-0.053648772	0.0778796334229027	0.120590716011097	1081
MYEF2	-0.053651741	0.0778631558524316	0.120574469570854	1081
KCNT2	-0.053660729	0.0778132979919999	0.120506525834013	1081
LOC40093	-0.053665516	0.0777867541155913	0.120483942923157	1081
PUS3	-0.053678832	0.0777129534906427	0.120406664210814	1081
TRPS1	-0.053685894	0.0776738422014679	0.120355322755832	1081
C6orf123	-0.053701194	0.0775891518846653	0.120233343439737	1081
ANKRD53	-0.05370177	0.0775859611033359	0.120233343439737	1081
PTHLH	-0.05375655	0.0772833683723358	0.119795905155898	1081
UBQLN1	-0.053779766	0.0771554191230886	0.119616432063616	1081
FLJ39609	-0.053814964	0.0769617581780543	0.119334561152779	1081
IP6K2	-0.05382817	0.0768892018346232	0.119231234774727	1081
PTK7	-0.05382881	0.0768856886429375	0.119231234774727	1081
RNF103	-0.053851449	0.0767614347114042	0.119069769943885	1081
ZP2	-0.05387537	0.0766303297811671	0.118875557788512	1081
DAZAP2	-0.053885651	0.0765740329252528	0.118806522895812	1081
ADH4	-0.053887116	0.0765660163804486	0.118803234998393	1081
ZNF3	-0.053911647	0.0764318455341211	0.118613321645857	1081
RPS24	-0.053913842	0.0764198509353521	0.118612982286206	1081
FAM138F	-0.053931634	0.0763226682666483	0.118471270783759	1081
FBN3	-0.053935142	0.0763035227918148	0.118459807848421	1081
ZNF835	-0.053946348	0.0762423769305557	0.118374002563857	1081
IRX4	-0.053946886	0.0762394417924797	0.118374002563857	1081
LOC34035	-0.053952144	0.0762107684864964	0.118343168978684	1081
ZNF853	-0.053957747	0.076180221092941	0.118304853061771	1081
ZNF681	-0.053978451	0.0760674370055746	0.118157029722978	1081
ARL1	-0.053986252	0.0760249738619275	0.118100177240016	1081

NUDT16	-0.054013483	0.0758769044952689	0.117906528930669	1081
KLK7	-0.054024085	0.0758193230607049	0.117826140789779	1081
HUS1	-0.054066462	0.0755895037232146	0.117514319532656	1081
PCDHB9	-0.054081754	0.0755067160125374	0.117418975665117	1081
NEURL4	-0.054093583	0.075442720955472	0.1173495184656	1081
RHOV	-0.054109251	0.0753580338333259	0.11725400413782	1081
GALNT6	-0.054136415	0.0752113873886055	0.117052951092665	1081
CBLN2	-0.054161823	0.0750744287489869	0.116857855597925	1081
ANO4	-0.054173576	0.0750111460927228	0.116768374780767	1081
LDB3	-0.054186269	0.0749428493241582	0.116671074160368	1081
GAB4	-0.054195594	0.0748927076078768	0.116611036849643	1081
YWHAE	-0.054206368	0.0748348093722287	0.116529893700514	1081
DSG4	-0.054226274	0.074727928032502	0.116390452312877	1081
MAGEC1	-0.054229785	0.0747090926444929	0.116370112991828	1081
SEBOX	-0.054274377	0.0744701727090354	0.11603384865214	1081
CYP2A7	-0.054275939	0.0744618117436811	0.116029795604614	1081
PTPN23	-0.054314535	0.0742555396264693	0.115744184909875	1081
SNORA23	-0.054329202	0.0741772797720555	0.115631146096731	1081
SRP72	-0.054340339	0.0741178942973911	0.115547514364722	1081
CYP2A6	-0.054343694	0.0741000162305737	0.115528583466056	1081
CEP164	-0.054362036	0.0740023207022613	0.115394128844884	1081
PITX2	-0.054427938	0.0736521723704698	0.114892598141793	1081
TMEFF1	-0.0544402	0.0735871719461817	0.114800091292173	1081
GCM2	-0.054507964	0.0732288014016423	0.114311832951266	1081
F2RL1	-0.054524331	0.0731424550419173	0.114185892499955	1081
COL6A6	-0.054534408	0.0730893362167976	0.114111809508439	1081
GGT8P	-0.054542713	0.0730455838379015	0.114061180250719	1081
RNF43	-0.054559779	0.072955736746982	0.113929714478136	1081
PABPC1L	-0.054577023	0.0728650496892303	0.113805739338622	1081
OSBPL5	-0.054593209	0.0727800072054654	0.113681727933171	1081
PIK3CB	-0.054604016	0.0727232688599305	0.113609921049574	1081
TMED10P	-0.054622914	0.0726241473090874	0.113473469909374	1081
HINFP	-0.054650023	0.0724821449452516	0.113269165303087	1081

STBD1	-0.054664342	0.0724072291870428	0.113187214400014	1081
ESAM	-0.054681522	0.0723174354170715	0.113055621331438	1081
LOC28400	-0.054683254	0.072308383000799	0.113050242572844	1081
ZNF830	-0.054689693	0.0722747567913354	0.113006440201913	1081
PLAGL2	-0.054735298	0.0720369392419898	0.112660829208127	1081
C12orf66	-0.054747073	0.0719756396148833	0.112582441186569	1081
HSFX2	-0.054749026	0.0719654790481951	0.112575289286061	1081
ZNF257	-0.054750127	0.0719597520721847	0.112575072260166	1081
KCNMB1	-0.054752719	0.0719462635410161	0.112562711947326	1081
NFATC1	-0.054763345	0.0718910037126526	0.112484991828457	1081
RTN4RL2	-0.054773558	0.0718379209556563	0.112410666277124	1081
TRPV6	-0.054797318	0.0717145515393906	0.112243775989513	1081
NT5C2	-0.054825859	0.0715665939094386	0.112064441065343	1081
LCN8	-0.05485118	0.0714355314104888	0.111885304016817	1081
GSN	-0.054853905	0.0714214394214625	0.111871930421069	1081
RAB15	-0.054867911	0.0713490409895589	0.111767218564126	1081
MLLT1	-0.054874538	0.0713148052446946	0.111722276355422	1081
F10	-0.054891827	0.0712255547765548	0.111599813406992	1081
FER1L6	-0.054918836	0.0710863124858692	0.111398970441683	1081
KIAA0020	-0.054923162	0.0710640284752729	0.111372713056976	1081
MLF1	-0.054930528	0.0710261007537028	0.111321932598097	1081
SH2B1	-0.054967637	0.0708352850641046	0.11105742218199	1081
PLD1	-0.054980857	0.0707674084581503	0.110967088870919	1081
PTF1A	-0.054998877	0.0706749698218146	0.11085784217554	1081
FLJ35024	-0.055007988	0.0706282699626292	0.110793217442545	1081
LOC25372	-0.055010344	0.0706161973246843	0.110782905934566	1081
VIPR2	-0.055018398	0.0705749459241896	0.110726813523583	1081
GDPD1	-0.055019464	0.0705694882723079	0.110726813523583	1081
NOD1	-0.055038048	0.07047437664426	0.11061210109066	1081
LOC10014	-0.055039296	0.0704679931685872	0.110610699889449	1081
KILLIN	-0.055041894	0.0704547058873309	0.110598461006801	1081
GJD4	-0.0550447	0.0704403572587197	0.110593172549054	1081
LOC64699	-0.05506645	0.0703292245909879	0.110435905322741	1081

CILP2	-0.055068901	0.0703167068769005	0.110424856566508	1081
PHF13	-0.055074071	0.0702903162728041	0.110392018501258	1081
PPARGC1	-0.055182112	0.0697406391569527	0.109579999673724	1081
TEPP	-0.055211992	0.0695892422585889	0.109367708324175	1081
CDKN1B	-0.055223549	0.0695307570474183	0.109309903287049	1081
LGALS7B	-0.05522645	0.0695160798935907	0.109295358577725	1081
ZW10	-0.05525167	0.0693886175276909	0.109103473313716	1081
C1QTNF6	-0.055273973	0.0692760560482249	0.108960505082096	1081
PCLO	-0.055296163	0.069164211289118	0.108801580229524	1081
USP26	-0.055311812	0.0690854248684627	0.108686129166355	1081
GRK4	-0.055359929	0.0688436379407892	0.108348053618531	1081
SULF1	-0.055366232	0.0688120176609243	0.108306750197619	1081
TUB	-0.055373413	0.0687760049290299	0.10826698595728	1081
PDGFA	-0.05537351	0.0687755194169883	0.10826698595728	1081
BMF	-0.055374994	0.0687680778724117	0.10826698595728	1081
AS3MT	-0.055390536	0.0686901989229638	0.1081572656951	1081
NES	-0.055428616	0.0684996903635113	0.107865728318059	1081
GLOD4	-0.055430009	0.068492728259716	0.107863196590993	1081
RAP1GAP	-0.055447477	0.068405491531345	0.107742660429941	1081
ASNSD1	-0.055474009	0.0682731594663318	0.107551047657902	1081
TRPM3	-0.055503151	0.0681280532489046	0.107350192725892	1081
C6orf162	-0.055529213	0.0679984990925066	0.107168656252429	1081
CCDC88C	-0.055537833	0.0679556929633135	0.107117952640528	1081
C1orf116	-0.055539914	0.0679453619796705	0.107110049071169	1081
HSPA13	-0.05557174	0.0677875286269064	0.106903065929938	1081
SLC22A3	-0.05559408	0.0676769203515632	0.106753704524488	1081
TTC29	-0.055600547	0.0676449291934216	0.106719954467968	1081
ADCY4	-0.055650473	0.0673983690160213	0.106355956746651	1081
CSNK1A1	-0.055658979	0.0673564356362532	0.106298111703276	1081
SGCZ	-0.055660478	0.0673490506093381	0.106294784121366	1081
ZNF266	-0.055680416	0.0672508500134751	0.106156431086151	1081
CISH	-0.055696328	0.0671725665661142	0.106047728055107	1081
TAS2R43	-0.055697167	0.06716844022356	0.106047728055107	1081

FAM70A	-0.055715584	0.0670779306226697	0.105924975495311	1081
ICAM5	-0.055727756	0.0670181616599068	0.105855485858462	1081
GLRA3	-0.055732688	0.0669939574063069	0.105833851313524	1081
HBB	-0.055733552	0.0669897222940495	0.105833851313524	1081
C2orf44	-0.055734777	0.0669837104541201	0.105833851313524	1081
KRT74	-0.055743981	0.0669385642261023	0.105771227259772	1081
MCAM	-0.055803139	0.0666490046758296	0.105346739834805	1081
SLC16A1	-0.055805534	0.0666373025517628	0.105336508153269	1081
METTL3	-0.055813439	0.066598694281296	0.105283739801986	1081
SMOC2	-0.055826894	0.066533019677523	0.105212943041346	1081
RABGAP1	-0.055841625	0.0664611806030936	0.105107590143611	1081
TBX19	-0.055891859	0.0662166786090441	0.104770263162048	1081
KIF3B	-0.055906878	0.0661437189830386	0.104663044451599	1081
DUOXA2	-0.055915976	0.0660995553095802	0.10460137798205	1081
TTLL9	-0.055926137	0.0660502635133215	0.104531586022143	1081
LRRC7	-0.055966473	0.0658548770422023	0.104238744245711	1081
DYX1C1	-0.055968315	0.065845966198182	0.104232829566429	1081
ART5	-0.055977192	0.065803036847865	0.104173059119862	1081
MC3R	-0.056004589	0.0656706819421454	0.103979870134757	1081
CCDC71	-0.056032333	0.0655368758981818	0.103808803661651	1081
PPYR1	-0.056040268	0.0654986490988334	0.103772731899017	1081
HK2	-0.056055971	0.0654230512120751	0.103669265056812	1081
ALDOB	-0.056066718	0.0653713536860871	0.103600908990307	1081
OPRD1	-0.056090368	0.0652577081204014	0.103439808803642	1081
LIAS	-0.056188108	0.0647897609870637	0.102746574437881	1081
TBP	-0.056189823	0.0647815751175095	0.102741680896097	1081
AP4E1	-0.056199734	0.0647342811912267	0.1026747574677	1081
OR5K1	-0.056248546	0.0645017878751728	0.102338230821876	1081
SREBF2	-0.056261661	0.0644394372158138	0.102255412580746	1081
C19orf44	-0.056268588	0.0644065251189466	0.102223537742944	1081
FAM40A	-0.056299693	0.0642589084436006	0.102025197871716	1081
HSPA4L	-0.056316413	0.0641796768536046	0.10190743203404	1081
SOX21	-0.056331342	0.0641089961388318	0.101803226171903	1081

LACRT	-0.056345506	0.064041998551557	0.10172089102114	1081
HIGD1B	-0.056374149	0.0639066904513322	0.101537240586582	1081
ADRA1D	-0.056419359	0.0636936012997122	0.101207414275335	1081
ZMAT4	-0.056434398	0.0636228510912812	0.101102968780773	1081
MDFI	-0.056439474	0.0635989810760775	0.101080984124464	1081
GOLGA3	-0.056460014	0.0635024839797493	0.100944033395496	1081
NLRP14	-0.056544777	0.0631055337619989	0.100375916118079	1081
SPTLC3	-0.05654634	0.0630982361361343	0.100372233651486	1081
ZFYVE26	-0.056549957	0.0630813435156647	0.100353286355766	1081
NPAS4	-0.056550066	0.0630808352290858	0.100353286355766	1081
SMO	-0.056577647	0.0629521545531415	0.100163585299189	1081
MAGI2	-0.056586985	0.0629086361828552	0.100102249281486	1081
HELQ	-0.056633708	0.0626912680757034	0.0997721279337213	1081
C17orf102	-0.05665132	0.0626094944474663	0.0996646604975121	1081
DAB1	-0.056654357	0.0625954008920537	0.0996646604975121	1081
CES1	-0.056672519	0.0625111811328754	0.0995484382546858	1081
C15orf5	-0.056687872	0.0624400618123958	0.0994509049028079	1081
C21orf15	-0.056717643	0.062302341074713	0.0992393975606938	1081
ZSCAN18	-0.056722937	0.0622778784305164	0.0992082762124587	1081
DACH1	-0.056731692	0.0622374353221488	0.0991516911478598	1081
C19orf26	-0.056754093	0.0621340638675237	0.099002666820475	1081
SPIRE1	-0.056761275	0.0621009517891652	0.0989577340446711	1081
C5orf44	-0.056763995	0.0620884158325963	0.0989455847597225	1081
ZNF365	-0.05677487	0.0620383127309927	0.0988735609150197	1081
APOB	-0.056805067	0.0618993640875169	0.0986599168876516	1081
MS4A14	-0.056817692	0.0618413482542667	0.0985908484158122	1081
PENK	-0.056854914	0.0616705638268345	0.0983419226067902	1081
RS1	-0.056864447	0.0616268871500498	0.0982878348220848	1081
FBXL14	-0.056872176	0.0615914928756585	0.0982391626270895	1081
VPS11	-0.056872333	0.0615907743024097	0.0982391626270895	1081
TTC39C	-0.056878894	0.0615607455815521	0.098219145026088	1081
WFIKKN2	-0.056927396	0.0613391138555809	0.0979014577695496	1081
FAM180B	-0.056958635	0.0611967176540811	0.0976945777907492	1081

OR4F29	-0.05696655	0.0611606850858871	0.0976602727532654	1081
MYOM2	-0.056976038	0.0611175087324789	0.0975990656691072	1081
LOC25516	-0.056979573	0.0611014316824616	0.0975811274174083	1081
LOC59510	-0.056986604	0.0610694591703959	0.0975377986987731	1081
ARL17A	-0.05699338	0.0610386627639865	0.097496341688908	1081
SNORA39	-0.05699388	0.0610363928674336	0.097496341688908	1081
ABAT	-0.057058663	0.0607426035396837	0.0970465340873128	1081
RALA	-0.057083182	0.0606317157653093	0.0968770553689845	1081
RPGRIP1L	-0.057126655	0.0604355206034275	0.0965788959666862	1081
PNPLA2	-0.057147409	0.0603420440686605	0.0964371660264313	1081
C8orf58	-0.057154972	0.0603080083235308	0.0963904179369666	1081
TSSK3	-0.05720387	0.0600883424261566	0.0960621901575212	1081
PNMAL1	-0.05723114	0.0599661237539577	0.0958867122090955	1081
TWSG1	-0.057248536	0.0598882667920542	0.0957727343435685	1081
ZNF556	-0.057252741	0.0598694578993411	0.0957502563815677	1081
TRIM3	-0.057255305	0.059857996109669	0.0957395261317413	1081
DARC	-0.057259079	0.0598411238340638	0.0957201397202807	1081
YOD1	-0.057279165	0.059751387796318	0.0955917810639157	1081
GPR21	-0.057282278	0.0597374910571416	0.0955771390156554	1081
LRRC14B	-0.057304671	0.0596376002706564	0.0954284246494461	1081
LOC72839	-0.057332608	0.0595131779478548	0.0952560757794005	1081
OR7A5	-0.057348124	0.059444163181101	0.095153171814325	1081
CNN2	-0.057365362	0.0593675721484335	0.0950532300850574	1081
SMURF2	-0.057380777	0.0592991462903462	0.0949512202542742	1081
UBE2W	-0.057391328	0.0592523508702462	0.0948989194053991	1081
SEC16A	-0.057437418	0.0590482885793075	0.0946021769866509	1081
FAM55A	-0.05746679	0.0589185498410512	0.0944018282468763	1081
RGL2	-0.057492273	0.0588061802805712	0.0942367752567105	1081
SMCR7	-0.057501127	0.0587671806363932	0.0941817703993747	1081
FEV	-0.057511317	0.0587223215665959	0.0941173655787313	1081
LOC64728	-0.057524443	0.0586645771946142	0.094032296911641	1081
SETD1B	-0.057530247	0.0586390603022877	0.0939988756098979	1081
UBE2D4	-0.057533167	0.0586262279274015	0.0939857839883418	1081

FLRT3	-0.05753593	0.0586140831711686	0.0939737927165293	1081
EPB42	-0.057596918	0.0583466013763482	0.0935821882744056	1081
FRG2C	-0.057607287	0.058301228066745	0.0935168595751714	1081
ASPRV1	-0.057607616	0.058299786344904	0.0935168595751714	1081
TMC2	-0.057640086	0.0581578887803018	0.0933315278801226	1081
MEOX1	-0.057649351	0.0581174494531682	0.0932740614431225	1081
MIA	-0.057722042	0.0578009991625835	0.0927957545529363	1081
TMEFF2	-0.057746203	0.0576961344128785	0.0926495518331956	1081
CLEC4F	-0.057764784	0.057615596193138	0.0925349745125381	1081
TRIM43	-0.057771232	0.0575876688114139	0.0925048710793345	1081
LOC10012	-0.057792855	0.0574941014803726	0.092380954582825	1081
ABLIM3	-0.057802081	0.0574542179613191	0.0923346805608913	1081
BTBD1	-0.057807035	0.057432810951976	0.0923076413457442	1081
ZIK1	-0.057864911	0.0571832038928076	0.09194314330119	1081
FAM71C	-0.057889896	0.0570757310087303	0.0917849925098484	1081
RAB43	-0.057896974	0.0570453131093038	0.0917507251566407	1081
MBTPS2	-0.057898082	0.0570405555917396	0.0917503986262851	1081
MGC5734	-0.057913913	0.0569725777296285	0.0916630124082235	1081
HDGFRP3	-0.057938786	0.0568659142811594	0.0915206393859515	1081
TRIM17	-0.057997141	0.0566163134224794	0.0911407730458565	1081
PTCD2	-0.058041985	0.0564251250546523	0.0908547793322652	1081
C3orf19	-0.0580527	0.0563795196202924	0.090795860800287	1081
SLC41A2	-0.058094863	0.0562003702419709	0.0905218244602626	1081
LOC44094	-0.058098158	0.0561863883149943	0.0905065402691157	1081
NKAPL	-0.058101015	0.0561742712431865	0.0904942578323863	1081
TRAPPC1	-0.058111158	0.0561312621082079	0.0904394365488161	1081
SLC9A7	-0.058114542	0.056116916561375	0.0904235549615181	1081
GPX8	-0.058116301	0.0561094605082032	0.090418773053176	1081
HRC	-0.058126086	0.0560680079687734	0.0903736614283037	1081
UFM1	-0.058129412	0.0560539263292371	0.0903581936386719	1081
HEMK1	-0.05813885	0.0560139773737549	0.0903082492352374	1081
RPL12	-0.058152885	0.0559546127569424	0.0902197594036022	1081
GIPR	-0.058153132	0.055953566906031	0.0902197594036022	1081

PRSS55	-0.058177411	0.0558509986869627	0.090067112435868	1081
SLC1A4	-0.058178573	0.0558460930842894	0.0900664114308711	1081
F8	-0.05823367	0.0556139263777608	0.0897350858534235	1081
TACSTD2	-0.058254206	0.0555275952598357	0.0896173214636588	1081
STX12	-0.058261808	0.0554956683213026	0.0895729699809824	1081
MAN2A2	-0.058273907	0.0554448816148586	0.0895053401820119	1081
SIPA1L2	-0.058298197	0.0553430421921708	0.0893552604761786	1081
PBLD	-0.058303726	0.0553198851077169	0.0893250308989225	1081
GAS2L3	-0.058322978	0.0552393059858847	0.0892092207059201	1081
AKR1B15	-0.058331857	0.0552021773685396	0.0891564068860122	1081
NHLRC4	-0.058353347	0.0551123983893384	0.0890328200122873	1081
WDR89	-0.058357903	0.055093379208606	0.0890092328300078	1081
KCNAB3	-0.058387159	0.0549713872207529	0.0888406414410001	1081
GPR12	-0.058398659	0.0549234937926947	0.0887703612366683	1081
NPC1	-0.05845035	0.0547086546986797	0.0884373173571579	1081
GNPNAT1	-0.05846922	0.0546304021620102	0.0883179079972471	1081
FLJ44635	-0.058492479	0.0545340760025115	0.0881763352653177	1081
C12orf35	-0.058492911	0.0545322901636544	0.0881763352653177	1081
TBC1D9	-0.058561007	0.0542510954385277	0.0877821941854731	1081
SLCO1B3	-0.058561694	0.0542482659462725	0.0877821941854731	1081
SLC25A27	-0.058580301	0.0541716441880381	0.0876677194865061	1081
INSRR	-0.058588365	0.0541384636435078	0.0876351426354732	1081
KIF2A	-0.058589399	0.0541342122387854	0.0876351426354732	1081
MGC2188	-0.058602048	0.0540822079024364	0.0875722271677984	1081
TRERF1	-0.058630793	0.0539641803709608	0.087402187938013	1081
TTC5	-0.05863319	0.0539543445792809	0.0873932838746976	1081
PNPLA7	-0.058650075	0.0538851241395521	0.0872952011090693	1081
PCDHGA1	-0.058677651	0.0537722389646145	0.0871263374842885	1081
DGCR11	-0.058683018	0.0537502896934291	0.0870977787865291	1081
OR1L3	-0.058686861	0.0537345766883922	0.0870793216465015	1081
ABCA13	-0.05869238	0.0537120194950713	0.0870547216597003	1081
ELFN2	-0.05869493	0.0537016007224169	0.0870468898095501	1081
RHBDF1	-0.05873917	0.0535211072607348	0.0867682847068648	1081

CRTC1	-0.058744324	0.0535001090163504	0.0867412230289238	1081
GPR137B	-0.058866853	0.0530030008656669	0.0859908309481112	1081
ZIC1	-0.058882526	0.0529396943095863	0.0859155838126655	1081
FAM135A	-0.058911612	0.0528223748850999	0.0857390010523195	1081
RHOA	-0.058918918	0.0527929388430497	0.0856981267520738	1081
LPAR3	-0.058927361	0.0527589422801557	0.0856567450220612	1081
NFE2L2	-0.058944325	0.0526906873853942	0.0855528248068644	1081
SNORA54	-0.058946758	0.0526809020840606	0.0855438314108819	1081
SLC26A7	-0.058968206	0.0525947225482998	0.085417662506893	1081
XPNPEP3	-0.058973511	0.0525734263128217	0.085396845111417	1081
C4orf22	-0.058999312	0.0524699487178652	0.0852425094291447	1081
GNG11	-0.059020822	0.0523838097226506	0.0851094319592545	1081
REEP5	-0.059023186	0.0523743483831767	0.0851009233891834	1081
SFRP5	-0.059043531	0.0522929961882559	0.0849893028005176	1081
TMEM66	-0.059055811	0.0522439434433065	0.08491643058459	1081
RPS25	-0.059059488	0.0522292641216021	0.0849062722506193	1081
SEH1L	-0.059067984	0.0521953564105106	0.0848648470451764	1081
PART1	-0.059128439	0.0519546179873755	0.0845075316395381	1081
ADAMTS	-0.059129796	0.0519492245613455	0.0845055821049988	1081
RERG	-0.059131282	0.0519433194020689	0.0845027997540724	1081
MUC1	-0.059179919	0.0517503485205867	0.084222876954857	1081
C6orf225	-0.059213483	0.0516175313366436	0.0840542533971938	1081
MTRF1L	-0.059214615	0.0516130573992523	0.0840537624339481	1081
NOS2	-0.059232375	0.0515429017710545	0.0839598735487905	1081
NAA15	-0.059255458	0.0514518343347722	0.083825087155195	1081
SLC38A4	-0.059291378	0.0513103910300676	0.0836014095196305	1081
PALM	-0.059293984	0.0513001387955457	0.0835914661519265	1081
ESYT1	-0.059295491	0.051294215046885	0.0835885748357465	1081
MYL1	-0.059326584	0.0511720747230887	0.0834097779186521	1081
FN1	-0.0593389	0.0511237586429814	0.0833397041840245	1081
LOC38838	-0.059340771	0.0511164242138496	0.0833397041840245	1081
FBXO25	-0.059362839	0.0510299594025558	0.0832117963545804	1081
ALOX15B	-0.059406209	0.0508603916099627	0.0829487199629776	1081

SLC7A10	-0.059413511	0.0508318916919852	0.0829089513509061	1081
KLK13	-0.059437156	0.0507396840251263	0.0827719600523256	1081
ANKRD32	-0.059460069	0.0506504665541329	0.0826331105225954	1081
PTGR2	-0.059481794	0.0505659965462609	0.0825019843220561	1081
CRLF1	-0.05951024	0.0504555721321679	0.0823484971726907	1081
P2RX3	-0.059511553	0.0504504838209208	0.0823468640878665	1081
KGFLP1	-0.059516162	0.0504326095650813	0.0823310307689273	1081
EI24	-0.059537616	0.0503494943679697	0.0822020069732352	1081
MBOAT2	-0.059545234	0.0503200092425292	0.0821605273294497	1081
OSBPL11	-0.059572093	0.0502161639878279	0.0820175631210307	1081
CNNM3	-0.059574962	0.0502050806854387	0.0820061096576796	1081
ZCCHC5	-0.059576387	0.0501995797177739	0.0820037733874923	1081
ICOSLG	-0.059586085	0.0501621382019548	0.0819559021961378	1081
ANKRD40	-0.05959267	0.0501367278243468	0.0819210302556141	1081
OR14A16	-0.059605686	0.0500865368078661	0.0818589391525607	1081
IQCJ	-0.059626303	0.0500071200177024	0.0817435066044222	1081
SNORD97	-0.059682811	0.0497899896506646	0.0814072998549337	1081
STK39	-0.059749298	0.0495355287869223	0.0810109780331669	1081
ARNT2	-0.05977489	0.0494378722104701	0.0808578335373809	1081
DLAT	-0.059792046	0.0493724956134088	0.0807574637976713	1081
INTS12	-0.059795204	0.0493604714259818	0.0807443521621116	1081
WEE2	-0.059810274	0.0493031157006227	0.0806570785925812	1081
ANXA8	-0.05981241	0.0492949901158825	0.080650335083607	1081
NLGN2	-0.059814304	0.0492877875588736	0.0806451007450869	1081
NCAM1	-0.059827905	0.0492360858587804	0.0805735946080529	1081
SEC31B	-0.059859222	0.0491172111570756	0.0804051859872653	1081
TRIB2	-0.059871975	0.0490688721008112	0.0803391115467199	1081
MTF2	-0.059886275	0.0490147157680389	0.080256965711383	1081
LHCGR	-0.059895216	0.0489808792104809	0.0802080809376637	1081
CSAD	-0.059907721	0.0489335908549573	0.0801371585865471	1081
DAAM2	-0.059909646	0.0489263169446993	0.0801317605569891	1081
HAUS6	-0.059910252	0.0489240248175033	0.0801317605569891	1081
METAP2	-0.059933743	0.0488353087857308	0.0800022196867973	1081

HPX	-0.059934868	0.0488310633168737	0.0800017705366315	1081
LOC64466	-0.059949621	0.0487754185961638	0.079923605630366	1081
ZCCHC18	-0.060028827	0.0484775882906165	0.0794614333697696	1081
RAB27A	-0.060029326	0.0484757163916363	0.0794614333697696	1081
RFC3	-0.060075534	0.0483026756833819	0.0792133996777702	1081
ENTPD1	-0.060145425	0.0480419319680241	0.0788371390921999	1081
MGLL	-0.060157264	0.0479978828758108	0.0787776886535122	1081
USP16	-0.060182434	0.047904341489711	0.0786369751719547	1081
AGK	-0.060207303	0.0478120709642407	0.0785041798813725	1081
SVIP	-0.060209693	0.0478032084061769	0.0784965465717668	1081
CTDSP1	-0.06021368	0.0477884344736151	0.0784850817276191	1081
BGN	-0.060224184	0.0477495198389193	0.0784275643827989	1081
LUM	-0.060230987	0.0477243325390519	0.0783925863502284	1081
PRRG4	-0.060254351	0.047637913315711	0.0782633961625346	1081
FAM71E2	-0.060307307	0.0474425285187267	0.0779932868459074	1081
CDKL3	-0.06030883	0.0474369183935597	0.0779904285512332	1081
GPR1	-0.060337232	0.047332414834099	0.0778249670937744	1081
ZNF879	-0.060339682	0.0473234091486884	0.0778167946880979	1081
FOLR1	-0.060359709	0.0472498499869885	0.077708239692047	1081
RNASE12	-0.060365557	0.0472283862816015	0.0776792816494825	1081
MEIS3	-0.060368511	0.0472175495623198	0.0776677991290056	1081
PION	-0.060417397	0.0470384938418928	0.0774111966706095	1081
NID2	-0.060449625	0.0469207634754707	0.0772426885848947	1081
C12orf68	-0.060455724	0.0468985114495881	0.0772123662268676	1081
ZNF229	-0.060470718	0.0468438443449329	0.077134971745028	1081
EPHA4	-0.060505668	0.0467166259483938	0.0769380665247215	1081
CT62	-0.060520039	0.0466643975251652	0.0768583344080167	1081
WDR86	-0.060537748	0.0466001106407768	0.0767650035864296	1081
PTN	-0.060591856	0.0464041399903889	0.0764546822692717	1081
DGCR2	-0.06059879	0.0463790771845052	0.0764258903788841	1081
SRD5A2	-0.060600861	0.0463715942050456	0.0764198106397778	1081
FERMT1	-0.060614706	0.0463215907837297	0.0763498974258088	1081
KCTD21	-0.060643213	0.0462187789001141	0.076199140759702	1081

MEST	-0.060644988	0.0462123820247435	0.0761948302413209	1081
GPR110	-0.060646621	0.046206499526373	0.0761913671898816	1081
ORC5L	-0.060647692	0.0462026413671064	0.076191241841876	1081
NCRNA0(	-0.060656361	0.0461714220533502	0.0761584620703136	1081
L2HGDH	-0.060736687	0.0458829977940073	0.0757114212136178	1081
KRT12	-0.06073947	0.0458730325579834	0.0757034685738163	1081
C20orf108	-0.060763989	0.0457853122983363	0.0755648956455829	1081
PRKAR1A	-0.060767072	0.0457742911062553	0.0755528958898412	1081
PARP4	-0.060776987	0.045738865009554	0.0755129843974321	1081
C21orf82	-0.060802711	0.045647065014228	0.0753737807140675	1081
USP42	-0.060814213	0.0456060661487608	0.0753122553132868	1081
MAP6	-0.060828836	0.0455539898698559	0.0752324254737044	1081
STAT3	-0.060829471	0.0455517307300134	0.0752324254737044	1081
NBR2	-0.060854859	0.0454614386183135	0.075104205426105	1081
MKRN1	-0.060864694	0.0454264992477742	0.0750649517961128	1081
HSF2BP	-0.06086506	0.0454252017842997	0.0750649517961128	1081
ADORA2I	-0.060893996	0.0453225428775808	0.0749177505759305	1081
PLEKHG2	-0.060923478	0.0452181489869566	0.0747574568192237	1081
GLT6D1	-0.060981972	0.0450116246387862	0.0744526790662902	1081
GAS2L2	-0.061013728	0.0448998363284445	0.0742799706690083	1081
SCRN3	-0.061016943	0.0448885328538914	0.0742673697718169	1081
ARHGEF1	-0.061027892	0.0448500539277931	0.0742158975172821	1081
KLK2	-0.061037105	0.0448176969524896	0.0741684469816728	1081
C1orf187	-0.061058858	0.0447413719563969	0.074060389323058	1081
SOX17	-0.061060732	0.0447348028746506	0.0740556006171284	1081
EIF3CL	-0.061078137	0.0446738222415093	0.0739668076810393	1081
LOH3CR2	-0.061079374	0.0446694921652326	0.0739657175463266	1081
RGS12	-0.061094653	0.0446160214978722	0.0739014761613745	1081
SNORA74	-0.061102505	0.0445885652278514	0.0738681452726206	1081
SLC20A2	-0.061106876	0.0445732870132824	0.0738489075485297	1081
WSCD2	-0.061114266	0.0445474662432691	0.0738182699243774	1081
DYDC2	-0.061134601	0.0444764783368043	0.0737127647959624	1081
THEM4	-0.061137401	0.0444667102318765	0.0737058108680131	1081

NCRNA00	-0.061169025	0.0443565216967467	0.0735381556728634	1081
C5orf13	-0.061181813	0.0443120317468366	0.0734764902082496	1081
C6orf142	-0.061197559	0.0442573020938172	0.0733978225635916	1081
LARP4	-0.06120844	0.0442195137693789	0.0733585744915518	1081
ETV5	-0.061233279	0.0441333534308998	0.0732284329845544	1081
ANKRD34	-0.061356269	0.0437088170251533	0.0725538990583152	1081
GCNT1	-0.061417728	0.0434979687289382	0.0722217571947959	1081
IFNGR1	-0.061442371	0.0434136655660487	0.072099611939658	1081
C3orf57	-0.061451246	0.0433833393603153	0.0720551876835314	1081
C1orf152	-0.061478972	0.0432887108824062	0.0719039479490495	1081
STIM2	-0.061487276	0.0432604015254116	0.0718687769033001	1081
CD59	-0.061489889	0.0432514982595992	0.0718599119537836	1081
DCAF12	-0.061496127	0.0432302457106934	0.0718305262159286	1081
CHRM2	-0.061514649	0.0431671995170592	0.0717376038831181	1081
DAPL1	-0.061546955	0.0430574232040065	0.0715669794096297	1081
HIGD1A	-0.06157508	0.0429620429758385	0.0714202307103702	1081
ATP1B4	-0.061577921	0.0429524183594064	0.0714101236156014	1081
SMPDL3A	-0.061579108	0.0429483975778144	0.0714093322222739	1081
FLJ36777	-0.061586849	0.0429221823117079	0.071377527048816	1081
MYH8	-0.061613706	0.0428313387139742	0.0712441019232938	1081
FAM149A	-0.061622071	0.0428030792286369	0.0712029753146895	1081
GPR112	-0.061623419	0.0427985252777946	0.0712012793559478	1081
EPX	-0.061624484	0.0427949298206108	0.0712011778358279	1081
SYT16	-0.061653462	0.0426971656135766	0.0710561256741429	1081
ZBTB25	-0.061657317	0.0426841732067445	0.0710403725421239	1081
PCDHGA1	-0.06166347	0.0426634464215041	0.0710117432174833	1081
LY6H	-0.061673556	0.0426294863039586	0.0709610809447403	1081
SLC2A10	-0.061680579	0.0426058526772334	0.0709276012050961	1081
KIAA0892	-0.06169487	0.0425577929719514	0.0708534495388397	1081
KLRAQ1	-0.061716793	0.0424841594498585	0.0707425518364523	1081
TMEM185	-0.061717733	0.0424810075452868	0.0707425518364523	1081
PHOX2A	-0.061795713	0.0422199822765691	0.0703317243828235	1081
VAPA	-0.061812334	0.0421645211102003	0.0702451437119571	1081

NAT1	-0.061824651	0.0421234612902149	0.0701825430230237	1081
LRRC6	-0.061827074	0.0421153881379416	0.0701748961157018	1081
C10orf129	-0.061837391	0.04208102645029	0.0701234409662578	1081
IMPG2	-0.061843967	0.0420591388133267	0.0700927656679737	1081
TMEM14(	-0.061849242	0.0420415851410749	0.0700751061283266	1081
PDE8A	-0.061851882	0.0420328043885537	0.070072065905943	1081
SGK1	-0.06189051	0.0419044884519958	0.0698697169231464	1081
NUDT21	-0.061926409	0.0417855370072767	0.06968868639884	1081
NCRNA0(	-0.061941424	0.0417358677810763	0.0696173765380295	1081
ADAM30	-0.06196208	0.0416676203317785	0.0695201703035547	1081
KIAA019(	-0.061974134	0.0416278401792703	0.0694601872286423	1081
TRIM24	-0.061979028	0.0416116953543464	0.06944475212603	1081
OR2M4	-0.061987557	0.0415835767594085	0.0694035752956324	1081
CLDN18	-0.062004209	0.0415287222799779	0.0693292541916097	1081
CYP2R1	-0.062017088	0.0414863373116192	0.0692642354668132	1081
MIDN	-0.062042211	0.0414037641405056	0.0691378339365455	1081
NPIP	-0.062042826	0.041401744691029	0.0691378339365455	1081
TAS2R1	-0.062044652	0.0413957492920516	0.0691359119124838	1081
MTERFD2	-0.062072432	0.0413046178342592	0.0689951514321021	1081
C6orf145	-0.062099663	0.0412154565568523	0.068869058753964	1081
MS4A8B	-0.062111393	0.0411770964175151	0.0688106683808247	1081
DNAH9	-0.062115868	0.0411624728883499	0.0687919376419286	1081
SPATA4	-0.06213112	0.0411126563874977	0.0687200848760488	1081
C18orf62	-0.062140244	0.0410828806485937	0.0686817120884581	1081
PHLPP2	-0.06214369	0.0410716414749959	0.0686686212560206	1081
TRPC4	-0.062145023	0.0410672936559524	0.0686670510208467	1081
GSTA1	-0.062177546	0.0409613368324344	0.0685240101718478	1081
METTL13	-0.062215809	0.0408369786012049	0.0683329955911016	1081
HIF1A	-0.062217617	0.040831111152928	0.0683288531463478	1081
EXD2	-0.062241495	0.0407536760576561	0.0682162695622336	1081
RIT1	-0.062273472	0.0406501731810145	0.0680486736522799	1081
ASB4	-0.062278759	0.0406330802770592	0.0680313665376794	1081
C9orf128	-0.062311278	0.0405280883396465	0.0678612201481322	1081

CALU	-0.062325311	0.0404828508548301	0.0677911081022903	1081
RPS8	-0.062326046	0.04048048401124	0.0677911081022903	1081
FBLN1	-0.062334822	0.0404522164116176	0.0677510724652508	1081
DCTD	-0.062348431	0.0404084152734624	0.067683339626166	1081
SLC35F4	-0.062363359	0.0403604152431194	0.0676141842056249	1081
KTN1	-0.062375663	0.04032088859891	0.0675576596931314	1081
DARS2	-0.06239654	0.0402538975232795	0.0674525674269228	1081
GDF9	-0.062397501	0.0402508147998254	0.0674525674269228	1081
TMOD2	-0.062417322	0.0401873020762519	0.0673541630275681	1081
SMARCA	-0.062424714	0.0401636386494865	0.0673237227986275	1081
CYP26A1	-0.062463939	0.0400382642418735	0.0671247364474485	1081
CCDC46	-0.062469619	0.0400201358009703	0.0671091765722263	1081
ACAD11	-0.06247536	0.0400018201231266	0.0670859695621366	1081
PLXNB2	-0.06249076	0.03995272594618	0.0670203734020982	1081
PLCD3	-0.062568968	0.0397041895254474	0.0666311977665894	1081
CNTROB	-0.062575297	0.0396841346757454	0.066603090314329	1081
TRNT1	-0.062643302	0.0394691782870587	0.0662644050831568	1081
ROPN1B	-0.062648522	0.039452719016334	0.0662422924307426	1081
GCLC	-0.062655283	0.0394314121399046	0.0662175556484144	1081
LGR6	-0.062656625	0.0394271814297784	0.0662159708130793	1081
GABRP	-0.062671078	0.0393816687164664	0.066145048882209	1081
VDR	-0.062697736	0.0392978333922478	0.0660262596903113	1081
GFRA2	-0.062714128	0.0392463585852896	0.0659452743098389	1081
LRP2	-0.062745977	0.0391465074074863	0.06580493922929	1081
RGL1	-0.0627592	0.0391051163096726	0.0657463335051205	1081
ADAM20	-0.062762802	0.0390938469843546	0.0657328726733847	1081
NR2F2	-0.062763662	0.0390911568158832	0.0657328726733847	1081
SEPP1	-0.062768245	0.039076822341019	0.0657152171349718	1081
TINAGL1	-0.062775782	0.0390532601669952	0.0656810758109967	1081
WDR5B	-0.062780739	0.0390377724948173	0.0656635761619374	1081
SDHD	-0.062798915	0.0389810177460975	0.065576000208362	1081
BDNF	-0.06281694	0.0389248058006679	0.0654869060342788	1081
SYBU	-0.062817745	0.0389222971911487	0.0654869060342788	1081

SDAD1	-0.062818919	0.0389186391681017	0.0654869060342788	1081
HUNK	-0.062820581	0.038913460819551	0.0654842258967384	1081
ROPN1	-0.062834239	0.038870923808063	0.0654287744655142	1081
TXNDC5	-0.062834836	0.0388690641985124	0.0654287744655142	1081
MICALL1	-0.062836133	0.0388650279233815	0.0654287744655142	1081
HOXA11A	-0.062845465	0.0388359899592082	0.0653866291456957	1081
SLC39A12	-0.062849929	0.0388221054153998	0.0653687156129412	1081
SCARB2	-0.062859364	0.0387927752100985	0.0653302505105697	1081
CYGB	-0.062880388	0.0387274859615628	0.0652257507479043	1081
NCRNA00	-0.062882869	0.0387197867333021	0.0652182360988604	1081
DNM1	-0.062908203	0.038641250595234	0.065091395036457	1081
PDE9A	-0.062919321	0.0386068261064227	0.0650388452846533	1081
ANKRD20	-0.062919715	0.0386056065673871	0.0650388452846533	1081
C1orf198	-0.06296426	0.0384679491195534	0.0648319948978	1081
MCART2	-0.062974231	0.0384371924820204	0.0647855791959757	1081
ANKFY1	-0.063027972	0.0382717853247536	0.0645283826052193	1081
CPB2	-0.063040198	0.0382342388178554	0.064486666191033	1081
RFWD2	-0.063047763	0.0382110243707004	0.0644529084775817	1081
FNTB	-0.063048207	0.0382096613726619	0.0644529084775817	1081
RAB40B	-0.063050536	0.038202517464761	0.0644521859438916	1081
C3orf74	-0.063056841	0.038183179837871	0.0644275197113588	1081
TTC18	-0.06306352	0.0381627055689268	0.0644035931509615	1081
DHX35	-0.063066817	0.0381526020554851	0.064398344798552	1081
RXFP3	-0.063070928	0.0381400065445294	0.0643870302387954	1081
ATAD1	-0.063075338	0.0381265003916401	0.064369623733623	1081
TRAF3IP2	-0.063091198	0.0380779595282219	0.0642984488429195	1081
RASIP1	-0.063121477	0.0379854304530717	0.0641529591278613	1081
PLIN5	-0.063134091	0.0379469404755803	0.0640933273983873	1081
SRP9	-0.063134673	0.0379451650545469	0.0640933273983873	1081
CENPJ	-0.063141133	0.0379254652296009	0.0640801126134589	1081
AQP7P3	-0.063146297	0.0379097270572169	0.0640680747959425	1081
NTN3	-0.063193998	0.0377645926078151	0.0638281498518697	1081
OR2AE1	-0.063219673	0.0376866679703785	0.0637124812238399	1081

ZEB2	-0.063250561	0.0375931063444233	0.0635809862571075	1081
BNIP2	-0.063257281	0.0375727753279968	0.0635519361026445	1081
PCDHAC1	-0.063261924	0.0375587371726632	0.0635335259336049	1081
CPSF7	-0.063303219	0.0374340557807714	0.0633332538593802	1081
MDH1B	-0.063311445	0.0374092612670532	0.0632966208823103	1081
OLFML2E	-0.063393944	0.0371613647716682	0.0629088817920383	1081
TTC30A	-0.063408483	0.0371178218770226	0.0628404506019514	1081
NXPH3	-0.063410554	0.0371116242492995	0.0628352387377827	1081
DOT1L	-0.063414297	0.0371004230810093	0.0628215535821579	1081
EFNA1	-0.063418944	0.0370865218585472	0.0628032937828206	1081
CIRBP	-0.063437486	0.0370310940400099	0.0627147027688945	1081
AMY2B	-0.063440634	0.0370216883791752	0.0627040450982415	1081
GJB1	-0.063458328	0.0369688733901558	0.0626251223044643	1081
INTS4L1	-0.063461089	0.0369606364959454	0.0626164344639493	1081
RFK	-0.063469069	0.0369368440106005	0.062581389620988	1081
ZNF75D	-0.063504744	0.0368306278916984	0.0624171783358513	1081
RARB	-0.063523181	0.0367758377647781	0.062340058210321	1081
VAMP7	-0.063533617	0.0367448556615795	0.0622927810571841	1081
ARHGEF5	-0.063536348	0.0367367498614918	0.0622842809257493	1081
MAGED4I	-0.063562025	0.0366606299051117	0.062181391718319	1081
MALL	-0.063569796	0.0366376186968371	0.0621475941939865	1081
HDLBP	-0.063575072	0.0366220032852092	0.0621263373625718	1081
ACTR3B	-0.06357952	0.0366088400666084	0.0621092372529752	1081
ABI1	-0.063591281	0.0365740598876022	0.0620554566188618	1081
MIS12	-0.063595767	0.0365608033843133	0.062038189368008	1081
HSPA12B	-0.063619916	0.0364894986565057	0.061932846106269	1081
GPR126	-0.06362541	0.0364732928688992	0.0619105565254445	1081
MAT2A	-0.063637627	0.0364372785526088	0.06185984969175	1081
NRP2	-0.063666668	0.0363517922935532	0.0617303258664443	1081
SMAD3	-0.063671642	0.0363371695891674	0.0617106964570712	1081
COL11A1	-0.063693038	0.0362743159277972	0.0616091471516292	1081
IMPDH2	-0.063696304	0.0362647305230147	0.0615991242281492	1081
C10orf119	-0.063703857	0.0362425687203044	0.0615708000396805	1081

GDE1	-0.063710827	0.0362221291541594	0.0615412660715646	1081
WISP1	-0.063723769	0.0361842047699834	0.0614820179732891	1081
KCNIP1	-0.063723868	0.036183913605837	0.0614820179732891	1081
LIN7A	-0.063763159	0.0360689800916512	0.0612965753286919	1081
LOC44117	-0.063765183	0.0360630671199825	0.0612916972185985	1081
WDR91	-0.063780266	0.0360190355887571	0.0612220276692129	1081
BAIAP2L1	-0.063791262	0.0359869652424344	0.0611726786607748	1081
IL17RB	-0.063808086	0.0359379414503901	0.0610976625506421	1081
ZNF382	-0.063820937	0.0359005337491815	0.0610412096874798	1081
TMEM139	-0.06382147	0.0358989821054785	0.0610412096874798	1081
RABL2A	-0.063855606	0.0357997800996108	0.0608904567803664	1081
ROBO3	-0.06386553	0.0357709834070922	0.0608466149401226	1081
LOC10027	-0.063866904	0.0357669987900639	0.0608449747192904	1081
ZNF620	-0.063893677	0.0356894169574315	0.0607283811645796	1081
FAM182B	-0.063919943	0.0356134468723768	0.0606042310562621	1081
SULT1B1	-0.063925311	0.0355979367245583	0.0605829547449715	1081
GLYATL1	-0.063925566	0.0355972004560584	0.0605829547449715	1081
VN1R1	-0.063954981	0.0355123169670407	0.0604525625571266	1081
OR6B2	-0.063980793	0.0354379704520623	0.0603361996920889	1081
ZC3H12C	-0.063987056	0.0354199527652677	0.0603106202735455	1081
C4orf45	-0.063992399	0.0354045873529547	0.0602895530198877	1081
TBL1XR1	-0.064029523	0.0352979791843387	0.0601130941473077	1081
COL21A1	-0.064040636	0.035266120048362	0.0600639151483136	1081
SLC44A4	-0.064060631	0.035208857446183	0.0599816011207913	1081
TBX22	-0.064073023	0.0351734097012682	0.0599364184090718	1081
COL16A1	-0.064092019	0.0351191317648735	0.0598540532490166	1081
PIGM	-0.064150376	0.0349528256738176	0.0596084668735404	1081
KCNB2	-0.064214322	0.034771362854096	0.0593215705196276	1081
LNP1	-0.064217279	0.0347629899467195	0.0593123090096269	1081
ENPP1	-0.064225913	0.0347385571587846	0.0592806636140377	1081
FAM170A	-0.064229195	0.0347292729919655	0.059269841106671	1081
SIX6	-0.064240333	0.0346977793374829	0.0592211102900612	1081
XKR6	-0.064271935	0.0346085595482458	0.0590838501779162	1081

LAMA5	-0.064283297	0.0345765294826287	0.0590391749811455	1081
ERCC5	-0.064289308	0.0345595964894007	0.059020267148099	1081
LOC28466	-0.064301037	0.0345265705689665	0.0589688651218169	1081
ELP2	-0.064308354	0.0345059845643717	0.0589437004196784	1081
NCK2	-0.064352855	0.0343809950690689	0.058745135340661	1081
NDUFA5	-0.064362311	0.0343544867164912	0.0587048210114272	1081
OLFM2	-0.064368639	0.0343367554733738	0.0586794994071187	1081
XRN1	-0.06437316	0.0343240945202573	0.0586628390693631	1081
SSPO	-0.064399565	0.0342502194574195	0.0585465142952665	1081
KIAA1274	-0.064414725	0.034207866884431	0.0584790799717297	1081
RPL32	-0.064419704	0.0341939666518999	0.0584602782145908	1081
PRMT2	-0.064460955	0.0340789884313704	0.0582686489517872	1081
LOC44090	-0.064474282	0.0340419116094962	0.058220078900866	1081
NUP205	-0.064489231	0.0340003668886622	0.0581589021798505	1081
ARCN1	-0.064585559	0.0337336842242902	0.0577321441422416	1081
PMS2L2	-0.064624129	0.0336274071473149	0.057560040528733	1081
LRRC37B	-0.064631196	0.0336079634341241	0.0575365363627459	1081
GPATCH1	-0.064658202	0.0335337585131778	0.057414377464556	1081
PPEF2	-0.064674617	0.033488721501698	0.0573470158663471	1081
RORC	-0.064677956	0.0334795659693749	0.0573362115491846	1081
C7orf46	-0.064687855	0.0334524388876588	0.0572946251821023	1081
TAS2R9	-0.064695938	0.033430300375211	0.0572615764865765	1081
CTSG	-0.064707643	0.0333982655058367	0.0572163523483261	1081
FAM90A7	-0.064724903	0.0333510747560296	0.0571453092591832	1081
ALDH3B2	-0.064746903	0.0332910101269314	0.0570520968955366	1081
TMCC1	-0.06474862	0.0332863250406481	0.0570489210433772	1081
FAM3D	-0.064759151	0.0332576091210444	0.0570045550704875	1081
BMPER	-0.064784997	0.0331872176733967	0.0568935835274084	1081
ATG5	-0.064785141	0.0331868282414508	0.0568935835274084	1081
SDC4	-0.064787339	0.033180847496649	0.0568923459715716	1081
C6orf186	-0.064788877	0.0331766637348274	0.0568923459715716	1081
ANAPC16	-0.064798275	0.0331511059638351	0.0568607092074463	1081
WDR90	-0.064813958	0.033108497437949	0.0567924626096289	1081

MGC2327	-0.064835146	0.0330510059245909	0.0567035014776771	1081
LOC22072	-0.06483802	0.0330432135018639	0.0566997901188287	1081
C15orf29	-0.064838703	0.0330413630975488	0.0566997901188287	1081
KLF4	-0.06488592	0.0329135817325441	0.0565014149128334	1081
TAF5L	-0.064888765	0.0329058949998624	0.0564930334730039	1081
RAB2B	-0.064908867	0.0328516336984529	0.0564046842968835	1081
LOC14584	-0.064926098	0.0328051827340794	0.0563418055350111	1081
CSNK1A1	-0.064968804	0.0326902985061113	0.0561707662406698	1081
USP21	-0.064975544	0.0326722000537387	0.0561444570572863	1081
EIF1B	-0.064982596	0.0326532705229369	0.0561167151240883	1081
COL9A2	-0.065001782	0.0326018219313163	0.0560330776219065	1081
GYLTL1B	-0.065012597	0.0325728491171414	0.0559880584867588	1081
FLRT1	-0.065012654	0.0325726958191534	0.0559880584867588	1081
GOLM1	-0.065018421	0.0325572578686406	0.0559755875875878	1081
SEC31A	-0.065020762	0.0325509915770406	0.0559695907578938	1081
HRH1	-0.065040489	0.0324982316448552	0.0558884135637364	1081
TSPAN12	-0.065042793	0.0324920767087113	0.0558825992740535	1081
GIN1	-0.065096331	0.0323492843505907	0.0556702830379847	1081
OGFRL1	-0.06514241	0.0322268164231572	0.0554737432149451	1081
GRM5	-0.065159795	0.0321807143270163	0.0553991189640867	1081
KRT7	-0.065160852	0.0321779124415133	0.0553990296670613	1081
CMA1	-0.065167229	0.032161017323908	0.0553746747000108	1081
SFRS13A	-0.065174018	0.032143039134164	0.0553531820275034	1081
CYP26B1	-0.065200441	0.0320731473276417	0.055246990416019	1081
LINGO2	-0.065204956	0.0320612182592629	0.05523588829495	1081
INPP5B	-0.065222981	0.0320136301285959	0.0551633375195076	1081
CLDN5	-0.065232693	0.0319880139165547	0.0551239127757909	1081
PRPF38A	-0.065249861	0.0319427748028678	0.0550506628799531	1081
PLAT	-0.065256164	0.0319261805617456	0.055026771681756	1081
FBXO4	-0.065265492	0.031901635077561	0.0549891707570348	1081
CHST15	-0.065277172	0.0318709229889761	0.0549503375225029	1081
GRXCR2	-0.065277358	0.0318704347540396	0.0549503375225029	1081
OR1Q1	-0.065306282	0.0317944904983784	0.0548279413704702	1081

EIF2AK4	-0.065322913	0.0317508931514964	0.0547621354911726	1081
GOLGA8I	-0.065338322	0.0317105457392843	0.0547019133342937	1081
FBXO46	-0.065371514	0.03162378079977	0.0545662581765625	1081
ATG4C	-0.065414463	0.0315118176491439	0.0543963638853475	1081
SLAIN1	-0.065435561	0.0314569400100877	0.0543062864429872	1081
SLC47A2	-0.065436563	0.0314543366998578	0.0543062864429872	1081
OR2A9P	-0.06546629	0.0313771592997349	0.0541824853500052	1081
ATP11B	-0.065472266	0.0313616643610438	0.0541622735612256	1081
DNER	-0.06547401	0.0313571428705686	0.0541618488491472	1081
SASS6	-0.065499625	0.0312908068987504	0.0540519040452175	1081
FEM1A	-0.065510395	0.0312629541513353	0.0540130541491124	1081
SNORD10	-0.065512846	0.0312566173191304	0.0540096023273202	1081
ATXN8OS	-0.065531836	0.031207561908227	0.0539358557516501	1081
PCDH11Y	-0.065532175	0.0312066880695369	0.0539358557516501	1081
ADH5	-0.065539705	0.0311872558439356	0.0539100110671086	1081
PRO0628	-0.065543636	0.0311771138501218	0.0538971044804516	1081
C3orf42	-0.065558681	0.0311383284141309	0.053843916386495	1081
ZNF263	-0.065584902	0.0310708322622656	0.0537318152565319	1081
LOC5542(	-0.06558778	0.0310634326959078	0.053723630808642	1081
NTM	-0.065588073	0.0310626786099425	0.053723630808642	1081
BTF3	-0.065589295	0.0310595361319193	0.053723630808642	1081
KRTAP5-1	-0.065604261	0.0310210801273006	0.0536642043078119	1081
KCNA4	-0.065681374	0.0308235786701895	0.0533500294106854	1081
CARD14	-0.06568174	0.0308226447407211	0.0533500294106854	1081
BLZF1	-0.065694944	0.0307889348589975	0.0532992259804506	1081
TYR	-0.065703947	0.0307659691925035	0.0532732034883086	1081
OVCH1	-0.06571363	0.0307412861367186	0.0532396156485726	1081
RPRD1B	-0.065745963	0.0306589863710222	0.0531016490795497	1081
RPS27A	-0.065748629	0.0306522068867035	0.0530944718600724	1081
RPL13A	-0.0657531	0.030640843402653	0.05308000367632	1081
PARD3	-0.065783248	0.0305643133918263	0.0529649972712552	1081
PFKFB2	-0.065804851	0.0305095771137844	0.0528746929591523	1081
ZFP82	-0.065817629	0.0304772381449105	0.0528322831451013	1081

SIN3B	-0.065851346	0.030392053307868	0.0527072928621729	1081
NFATC2	-0.065859585	0.0303712683099348	0.0526757813261847	1081
TEAD3	-0.065870584	0.0303435398134304	0.0526367527375833	1081
SDK2	-0.065887713	0.030300399553461	0.0525754994835894	1081
MAPK6	-0.065933557	0.030185201185732	0.052393665163413	1081
AHDC1	-0.065969373	0.0300954618482552	0.0522424023208186	1081
MAPKAP	-0.066016668	0.0299773136713115	0.0520552524703534	1081
AMN1	-0.066045303	0.0299059724140625	0.0519448020590869	1081
DIO3	-0.066077047	0.0298270574036713	0.0518121989649873	1081
GPC5	-0.066087088	0.02980213240545	0.051777831591875	1081
TMPRSS1	-0.066088092	0.0297996411276482	0.051777831591875	1081
RPS14	-0.066151073	0.0296437232458269	0.0515337249557458	1081
HN1L	-0.066182032	0.0295673379965268	0.0514098070032826	1081
DCLRE1A	-0.066232834	0.0294423570196913	0.0512101780488414	1081
PLA2G3	-0.066237654	0.0294305242692748	0.0511940170455522	1081
FXYD1	-0.066267879	0.0293564080355674	0.0510739131079114	1081
RRP7B	-0.066289719	0.0293029520850914	0.0509877621033602	1081
C1orf190	-0.066302613	0.0292714321672457	0.0509469493604662	1081
NOP14	-0.066306807	0.0292611867572299	0.0509346415989282	1081
IDS	-0.066337441	0.0291864402918235	0.050808921506981	1081
NCRNA0(	-0.066354554	0.0291447574756118	0.0507432495758971	1081
OR10A6	-0.066359747	0.0291321178551606	0.0507275057647343	1081
PPP2R1B	-0.066383041	0.0290754819193271	0.0506376401197237	1081
SNORD11	-0.066387169	0.0290654559475142	0.0506245555994011	1081
ITGB4	-0.066392825	0.0290517210556925	0.0506093843537639	1081
PTCHD3	-0.066445298	0.0289245775209166	0.0503922523703939	1081
CALML3	-0.0664721	0.0288598184736451	0.050296828718019	1081
PMS2L11	-0.066482074	0.0288357515322706	0.0502592329657026	1081
GTF2H2C	-0.066489995	0.0288166505514701	0.0502302868702402	1081
CPNE8	-0.06649137	0.028813337330653	0.0502288577817587	1081
RRAGB	-0.066517329	0.0287508217334316	0.0501285532127384	1081
PRDX3	-0.066572646	0.0286179943854903	0.049909920950195	1081
PRMT3	-0.066591528	0.0285727745902187	0.0498396869096852	1081

NCOA6	-0.066591693	0.0285723805360883	0.0498396869096852	1081
ARHGAP2	-0.066614968	0.0285167261681296	0.0497548457177353	1081
DALRD3	-0.066630639	0.0284793079560788	0.0496938639043055	1081
OR2A25	-0.066631073	0.0284782713001812	0.0496938639043055	1081
PLEKHM1	-0.06665334	0.0284251781160489	0.0496080059904535	1081
RAF1	-0.066669198	0.028387415642811	0.0495463947430624	1081
SLC13A2	-0.066705964	0.0283000362709954	0.0494024463326866	1081
LOC10012	-0.066706948	0.0282977016300647	0.0494024463326866	1081
TIE1	-0.066762393	0.0281663726297582	0.0491989574808358	1081
ADRB2	-0.066767846	0.0281534871090554	0.0491849794303982	1081
SLC33A1	-0.066769259	0.0281501471804093	0.0491834097961271	1081
DIS3L	-0.066846622	0.0279678725371855	0.0488861424955837	1081
SYTL5	-0.06685753	0.0279422536461519	0.0488498394256469	1081
NHEG1	-0.066876404	0.0278979748507667	0.0487808960479729	1081
SULT1E1	-0.066877173	0.0278961724621286	0.0487808960479729	1081
RNGTT	-0.066894794	0.0278548900565904	0.0487182462397997	1081
WIPF2	-0.066902778	0.0278362023998147	0.0486897887769617	1081
SUZ12	-0.066907355	0.0278254939127729	0.0486795114956848	1081
RPAP1	-0.066913916	0.02781014910269	0.0486568919292765	1081
CHD4	-0.066952302	0.0277205231924797	0.0485127217194426	1081
SGEF	-0.066993665	0.0276242260501214	0.0483610005891802	1081
C20orf12	-0.066996694	0.0276171852600615	0.0483528765047748	1081
BMP15	-0.067001943	0.0276049880225758	0.0483357221829457	1081
CPA1	-0.067003579	0.0276011877172617	0.0483332689989775	1081
SLC31A1	-0.067070815	0.027445381013483	0.0480771478710099	1081
SYNJ1	-0.067087408	0.0274070464691457	0.0480225233644377	1081
KLHL32	-0.067091055	0.0273986275015616	0.0480119477225955	1081
SYNE1	-0.067120109	0.0273316316879296	0.0479028815341345	1081
NCRNA00	-0.067133102	0.027301717977602	0.0478546166065251	1081
PCDHGC3	-0.067148533	0.0272662289315481	0.0478048896280276	1081
KCNJ14	-0.067156814	0.0272471988746583	0.0477756829428142	1081
FMN2	-0.06719536	0.0271587710288357	0.0476289233373288	1081
UBE2E3	-0.067221076	0.0270999146015276	0.0475298432568146	1081

SAMD4A	-0.06722399	0.0270932533032041	0.0475222975875531	1081
RPE	-0.067236552	0.0270645490827312	0.0474843530109406	1081
TCEA1	-0.067350495	0.0268053782977225	0.0470706368381817	1081
OR51E2	-0.067353331	0.0267989559847306	0.0470634616260481	1081
MED1	-0.067365274	0.0267719207770359	0.0470241820606319	1081
EIF4H	-0.067394307	0.0267062976611184	0.0469130071837487	1081
OR56B4	-0.067408114	0.0266751386178078	0.046866445792407	1081
FARP2	-0.067424507	0.0266381830456215	0.0468096822622161	1081
PHOSPHC	-0.067424766	0.0266376009297461	0.0468096822622161	1081
ZNF331	-0.067431828	0.0266216947067996	0.0467929534829839	1081
COX15	-0.067438659	0.0266063179558594	0.0467700065637685	1081
VEZT	-0.067442214	0.0265983176336933	0.0467600234515011	1081
LEKR1	-0.067443715	0.0265949403749626	0.0467581666829832	1081
RND3	-0.067457693	0.0265635093157311	0.0467151370724926	1081
SNORA48	-0.067499025	0.0264707562056792	0.0465601487613209	1081
CREB3L4	-0.067502214	0.0264636108174583	0.0465523172502927	1081
STOX1	-0.067538684	0.0263820195663539	0.0464202798641103	1081
GDAP2	-0.067544799	0.0263683604795466	0.0464002989046529	1081
C11orf87	-0.067554475	0.0263467582756698	0.0463663357616291	1081
DRD1	-0.067559544	0.0263354476788099	0.046350479948421	1081
EFNA5	-0.067565234	0.0263227576661344	0.0463362419769554	1081
SLC4A11	-0.06758586	0.0262767983996916	0.046259381609874	1081
OLAH	-0.067596389	0.026253365451463	0.0462302488653603	1081
NT5C1B	-0.067597187	0.0262515896606901	0.0462302488653603	1081
LST-3TM1	-0.067634154	0.0261694601988703	0.0460985804926866	1081
LEAP2	-0.06766266	0.0261062785932198	0.0459953631502898	1081
LGALS12	-0.067678257	0.0260717664359787	0.0459385756015737	1081
PCDHA5	-0.067685677	0.0260553620394382	0.0459217207597972	1081
MEX3A	-0.067696025	0.0260324968951131	0.0458854361669485	1081
LCN6	-0.067708367	0.0260052509631715	0.0458454345543966	1081
GDF6	-0.067731446	0.0259543646895741	0.0457637353760586	1081
ACSM5	-0.067753691	0.0259054001728061	0.0456893964700735	1081
BCL8	-0.067766055	0.0258782213168237	0.045653133127271	1081

C9orf144	-0.067772753	0.0258635064383187	0.0456354854339958	1081
TMEM22	-0.067772955	0.0258630625844576	0.0456354854339958	1081
RCVRN	-0.067783627	0.0258396340129597	0.0456053466962566	1081
ESPNP	-0.06780609	0.0257903793299151	0.0455263925342744	1081
SRBD1	-0.067815511	0.025769745933097	0.0454939559917833	1081
LOC72902	-0.0678313	0.0257351955564165	0.0454449083523853	1081
ADAM21	-0.067832351	0.0257328985051804	0.0454448356611606	1081
RGS6	-0.067858136	0.0256765682664553	0.0453612617493415	1081
AKR1D1	-0.067863684	0.0256644606698056	0.0453438484645881	1081
OLFM4	-0.067872802	0.0256445746716704	0.0453166628737544	1081
C1orf113	-0.067917153	0.0255480378289212	0.0451539938641531	1081
ARRB1	-0.067948299	0.0254804311743608	0.0450463612117156	1081
COL4A6	-0.067974892	0.025422829361281	0.0449603105506984	1081
SLC1A1	-0.067983107	0.0254050582627032	0.0449328269273183	1081
C6orf122	-0.068006659	0.0253541695013356	0.0448546363927641	1081
LOC22093	-0.068078834	0.0251987658581273	0.0445875395882279	1081
CHST4	-0.068082199	0.0251915400387438	0.044578669543262	1081
C13orf39	-0.068115859	0.025119366071964	0.0444587620382811	1081
RP1-177G	-0.068135004	0.0250783950981901	0.044390147548813	1081
GPR123	-0.068149617	0.0250471612108559	0.0443402466316084	1081
PRPS2	-0.068175601	0.0249917057084914	0.0442522555592476	1081
DHRS12	-0.068223394	0.0248899827762802	0.0440760111663294	1081
SH3BGRL	-0.068235517	0.0248642377230863	0.0440381632610844	1081
ACLY	-0.068303114	0.0247211034059535	0.0437962033341776	1081
NHEDC2	-0.068311995	0.0247023504303395	0.0437706789884062	1081
KLHL5	-0.068319455	0.0246866085103454	0.0437466333955761	1081
CSPG4	-0.068348532	0.0246253319975798	0.0436534066430169	1081
PTCD3	-0.068358437	0.0246044879313232	0.0436241339004142	1081
RAX2	-0.068366296	0.0245879616439949	0.0435986697753963	1081
GALNT8	-0.06836707	0.024586334646444	0.0435986697753963	1081
MALT1	-0.068371185	0.0245776854367229	0.043588121588413	1081
PEX11A	-0.068390318	0.0245375051676805	0.0435232438429631	1081
LOC38869	-0.068394943	0.0245278004535781	0.0435111430201948	1081

TDRKH	-0.068409264	0.0244977723326238	0.043469358199657	1081
LOC10013	-0.068416108	0.0244834333313354	0.0434477417600205	1081
CDC23	-0.0684287	0.0244570697579035	0.0434047811006049	1081
CLCN4	-0.068436143	0.0244414995655496	0.0433809699337442	1081
SLC35B3	-0.068449513	0.0244135491312007	0.043338997818826	1081
GDF3	-0.068455768	0.0244004820536359	0.0433196184868683	1081
SLITRK5	-0.068467372	0.0243762581373846	0.0432804265977096	1081
FAM46B	-0.068471469	0.0243677110897697	0.0432728790464924	1081
PCDHGB4	-0.068484794	0.0243399291595917	0.043227353691261	1081
GPR146	-0.068487351	0.0243346011196784	0.0432217016007689	1081
CEACAM	-0.068500629	0.0243069486686991	0.0431802011402949	1081
KIAA1908	-0.068502066	0.0243039576235829	0.0431786953282544	1081
C2orf71	-0.068543622	0.0242176004511026	0.0430366585292397	1081
ROBO4	-0.068546742	0.0242111269282772	0.0430365438957155	1081
TULP4	-0.068556744	0.0241903852608354	0.0430034690327859	1081
YY2	-0.068593319	0.0241146732368613	0.0428877983893857	1081
FGFR2	-0.068616492	0.0240668099906629	0.042814013357639	1081
TNK1	-0.06862003	0.0240595085005871	0.0428048042695688	1081
KLB	-0.068620254	0.0240590467205252	0.0428048042695688	1081
RASSF2	-0.068624335	0.0240506289095503	0.0427965656199003	1081
LOC72872	-0.068635771	0.0240270525100367	0.0427583898246191	1081
GIGYF1	-0.068649286	0.0239992154134253	0.0427126243045721	1081
DCD	-0.068657764	0.0239817655088859	0.0426853389447346	1081
KCNN2	-0.068660168	0.0239768213824817	0.042680309865697	1081
MOBKL2I	-0.068693453	0.023908436634904	0.0425661030499462	1081
KIAA0467	-0.068715025	0.0238642087149623	0.0424984598469232	1081
ZNF557	-0.068742135	0.0238087253475465	0.0424148189004532	1081
ARIH2	-0.068748893	0.023794911105951	0.0423939580993529	1081
AFP	-0.068750874	0.0237908626722568	0.0423904942979491	1081
SNTG2	-0.068752658	0.0237872193003556	0.0423877516855962	1081
TMPRSS1	-0.06876105	0.0237700800601454	0.0423684536198574	1081
HSD17B12	-0.068781249	0.023728871340535	0.0423062318041489	1081
CYCSP52	-0.068786391	0.0237183912306615	0.0422912897275337	1081

KIAA1826	-0.068794071	0.0237027452092663	0.042270874844252	1081
CYP4A11	-0.06882147	0.0236469990878861	0.0421863971506789	1081
TPPP2	-0.068843157	0.0236029572584208	0.0421115555726585	1081
LIPJ	-0.068846321	0.0235965368235996	0.0421038294341376	1081
C19orf2	-0.06885121	0.0235866193554412	0.0420935902653582	1081
GPR89B	-0.068855946	0.02357701641876	0.0420801803628884	1081
SLC6A6	-0.068861915	0.0235649183594296	0.0420697696163337	1081
KDELC1	-0.068902974	0.0234818450562223	0.0419400450977568	1081
ALOX12	-0.068928756	0.0234298110703899	0.0418545308160862	1081
MGAT4A	-0.06893787	0.0234114414173955	0.0418254245613189	1081
C15orf34	-0.068969617	0.0233475486778445	0.0417186773208425	1081
SCARNA9	-0.068977924	0.0233308540386898	0.041692769137245	1081
C7orf64	-0.068987642	0.0233113383187358	0.0416650617009079	1081
LOC72881	-0.069005561	0.0232753913715434	0.0416045039201191	1081
MYO5A	-0.069027769	0.0232309066246619	0.0415360445507957	1081
ATP5S	-0.069031854	0.0232227312722797	0.0415251128599391	1081
G3BP1	-0.069041558	0.023203322086764	0.0414940899634141	1081
ZNF846	-0.069044299	0.0231978414055	0.0414879718673471	1081
PIGL	-0.069066598	0.023153303124732	0.0414119942686431	1081
ZFP91-CN	-0.069117289	0.0230523338947479	0.0412423859955325	1081
GFM2	-0.069132825	0.0230214631060399	0.0411981322085079	1081
KDM4A	-0.069160255	0.022967049896296	0.041104408329858	1081
MCTP2	-0.069167444	0.0229528053641972	0.0410862150401415	1081
DOPEY2	-0.069191275	0.0229056471475736	0.041005444040511	1081
WDR3	-0.069212983	0.0228627631143793	0.0409395878168153	1081
NDFIP1	-0.069223887	0.0228412483044243	0.0409046979369391	1081
GPR160	-0.069264705	0.0227608648798232	0.0407788707758839	1081
ZNF486	-0.069282607	0.0227256876074134	0.0407267126457916	1081
DPY19L1	-0.06935996	0.0225742328080323	0.0404696911914044	1081
CHD1	-0.069366555	0.0225613606163966	0.0404538148302011	1081
ZBTB3	-0.069367726	0.0225590757407229	0.0404533185683516	1081
MDFIC	-0.069451019	0.0223970672163044	0.0401806856418606	1081
LOC10012	-0.069462093	0.0223756032255232	0.0401457540949559	1081

C4orf42	-0.069464879	0.0223702058559381	0.0401432208237906	1081
FAM160A	-0.06952269	0.02225847204895	0.0399676369931453	1081
ZNF26	-0.069528703	0.022246877471355	0.0399503785577149	1081
NFIX	-0.069556688	0.0221929888583972	0.0398642675481419	1081
NCBP1	-0.069570667	0.0221661111588991	0.0398266419279364	1081
KIAA1324	-0.069589736	0.0221294949765841	0.0397714940051107	1081
MID1	-0.069590076	0.0221288433239916	0.0397714940051107	1081
KPNA6	-0.069617116	0.0220770105765535	0.0396842489573187	1081
TRPV1	-0.069629496	0.0220533150902525	0.0396451929763642	1081
MFHAS1	-0.069660968	0.0219931774056246	0.0395476710556376	1081
ASB8	-0.069673058	0.0219701131287164	0.0395132514041596	1081
LOC10013	-0.06967498	0.021966449058761	0.0395101889543518	1081
ADRBK2	-0.0696783	0.0219601198621504	0.0395023318709725	1081
PCDHA8	-0.069681861	0.0219533320081255	0.0394936482678771	1081
HSD3B2	-0.069707825	0.0219039042140193	0.0394082477798892	1081
JPH2	-0.069717061	0.0218863455214456	0.0393836918740081	1081
FIGNL2	-0.069726801	0.0218678420885984	0.039353910923246	1081
CYB5D2	-0.069733192	0.0218557060094239	0.0393355845224555	1081
LOC10013	-0.069789051	0.0217498976136102	0.0391556467762446	1081
FAM66C	-0.069791757	0.0217447841656374	0.0391499398531384	1081
YIPF5	-0.069802486	0.0217245153940094	0.0391274351910881	1081
OXTR	-0.069808839	0.0217125216557541	0.0391114006870365	1081
ETF1	-0.06981282	0.0217050105337179	0.0391027935254647	1081
STRADB	-0.069818192	0.0216948767264305	0.0390880325242772	1081
LIMS2	-0.069823301	0.0216852418389887	0.0390776631940628	1081
CABP4	-0.069864883	0.0216069689361316	0.0389540351904752	1081
ARL8B	-0.069871577	0.0215943916337063	0.0389383297047095	1081
ARSG	-0.069894961	0.0215505034396512	0.0388626704674401	1081
FAM105A	-0.069902637	0.0215361136751488	0.0388401978501229	1081
FGFRL1	-0.069903934	0.0215336834025599	0.0388393089390008	1081
GOLGA8I	-0.069906989	0.0215279596896812	0.0388378020031722	1081
GLYATL2	-0.069914035	0.0215147633354641	0.0388225463447622	1081
PI16	-0.069926375	0.0214916672595034	0.038784344436325	1081

NUFIP2	-0.069928665	0.0214873846147977	0.0387800898642805	1081
PTPLA	-0.069940369	0.0214655037115432	0.0387440706270977	1081
ZMYND1:	-0.069991358	0.021370403081421	0.038582789933252	1081
HTRA1	-0.070007168	0.0213409898097947	0.0385331397076557	1081
KLK11	-0.070013148	0.0213298737751755	0.0385165209036312	1081
NUP35	-0.070016889	0.021322922831648	0.0385074209272567	1081
OR2A12	-0.070029037	0.021300363351344	0.0384735783836479	1081
CRYAB	-0.07003229	0.02129432457136	0.0384661198430817	1081
ZNF883	-0.07006363	0.0212362357009133	0.0383680688964034	1081
DUSP27	-0.070083226	0.0211999823654072	0.0383060045520789	1081
KIF16B	-0.07008442	0.0211977752972705	0.0383054524007458	1081
RSPO2	-0.070088667	0.0211899278560414	0.0382981425318458	1081
ZSCAN4	-0.070091643	0.0211844280768521	0.0382916378293571	1081
LOC25303	-0.07011047	0.0211496712163152	0.0382322439566286	1081
RAI14	-0.070110478	0.0211496570704959	0.0382322439566286	1081
KCNK2	-0.070125254	0.0211224149337944	0.0381898266775524	1081
PDE6C	-0.070136871	0.0211010163856673	0.038154561986291	1081
ING4	-0.070137851	0.0210992126478182	0.038154561986291	1081
MBNL1	-0.07014596	0.0210842894769659	0.0381311618200447	1081
C6orf35	-0.070155989	0.0210658443241123	0.0381012240895352	1081
SDR16C5	-0.070157574	0.0210629303560562	0.0380993743398359	1081
SYDE1	-0.070192755	0.0209983480268251	0.0379996156126833	1081
RAB3GAI	-0.070205496	0.0209750014809443	0.0379607766448319	1081
CAPN6	-0.07021611	0.0209555688011113	0.037932422814124	1081
GATA6	-0.070219015	0.0209502524171849	0.0379262073098661	1081
OSBPL9	-0.070223782	0.0209415329593863	0.0379172372341217	1081
PCDHGA1	-0.070247883	0.0208974926699926	0.0378408979628765	1081
SHOX2	-0.070307565	0.0207887825839813	0.0376542011467634	1081
FNTA	-0.070308451	0.0207871735006367	0.0376542011467634	1081
SCRG1	-0.070320765	0.0207648064790592	0.0376175383561734	1081
ATP1A4	-0.070325675	0.02075589340218	0.0376047731438902	1081
KDELC2	-0.070335823	0.0207374831931786	0.0375747975289245	1081
FAM53C	-0.070355462	0.0207018957654241	0.0375142947113528	1081

ECHDC2	-0.070368887	0.0206775983924205	0.0374764030243195	1081
MED13	-0.070381915	0.0206540447910743	0.0374370822670678	1081
TFDP3	-0.070414163	0.020595840663989	0.0373483851090159	1081
FABP7	-0.070433486	0.0205610331732091	0.0372891612423866	1081
GEMIN8	-0.070438772	0.020551520507161	0.037281439046984	1081
ODF4	-0.070451464	0.0205286954666303	0.0372440532936746	1081
DRD5	-0.070475735	0.0204851075859919	0.0371777020107934	1081
HLCS	-0.070493803	0.0204527111206628	0.0371222511510724	1081
RPL29	-0.070498429	0.0204444246244661	0.0371105545197214	1081
TUBB2B	-0.070498861	0.0204436503368942	0.0371105545197214	1081
TBCCD1	-0.070499282	0.0204428960917744	0.0371105545197214	1081
NEDD1	-0.070501872	0.0204382579055949	0.0371105545197214	1081
KCNMB2	-0.070508906	0.0204256665486464	0.0370932166792105	1081
GOLGA8I	-0.070559921	0.0203345425723186	0.0369410551104119	1081
C10orf2	-0.070560975	0.0203326649180107	0.0369409753583447	1081
CNTN6	-0.07058702	0.0202862825573551	0.0368600308575735	1081
PDCD11	-0.070588375	0.0202838731740677	0.0368589775475008	1081
C5orf28	-0.070590887	0.0202794036785313	0.0368541801627436	1081
MGAT3	-0.070644729	0.0201838425245701	0.0366871341385423	1081
C20orf185	-0.070645833	0.0201818865796833	0.0366868891127703	1081
CAPN13	-0.070661501	0.0201541550961668	0.0366523641699292	1081
ANKRD2(	-0.070662458	0.0201524624268756	0.0366523641699292	1081
KLHL3	-0.070749047	0.0199998087428643	0.0363854553531113	1081
MMP23B	-0.070753205	0.0199925041707342	0.0363754512752385	1081
CAP2	-0.07076054	0.019979622433239	0.0363552970750248	1081
OSBPL3	-0.070767649	0.0199671449383942	0.0363358748675656	1081
GSR	-0.070774665	0.0199548384709451	0.0363167604117075	1081
KCNS1	-0.070777844	0.0199492637267798	0.0363131759081937	1081
C10orf25	-0.07079549	0.0199183475354137	0.0362601763149203	1081
RFX8	-0.070900435	0.0197353376834417	0.0359400088259748	1081
C1RL	-0.070927746	0.0196879513382231	0.035860197080335	1081
MAP3K6	-0.07092915	0.0196855193462303	0.0358590096057337	1081
COL1A1	-0.070966306	0.0196212176485259	0.0357548104504345	1081

STX6	-0.070983184	0.0195920700953955	0.0357081563441367	1081
GABRA1	-0.071003196	0.0195575585138753	0.0356484813845474	1081
MBP	-0.07102052	0.0195277267783775	0.0355973265722934	1081
HSD17B1	-0.071022705	0.019523967079035	0.0355936938287023	1081
TP53TG3I	-0.071025203	0.0195196695076935	0.035589079756764	1081
SPINLW1	-0.071030061	0.0195113128488317	0.0355770634811472	1081
COPA	-0.071042754	0.0194894960852096	0.0355437170592565	1081
DLL4	-0.07104386	0.0194875966068401	0.0355434706785689	1081
NOG	-0.07104763	0.019481120646823	0.0355348764424347	1081
CCDC82	-0.071053188	0.0194715777980061	0.0355206859314347	1081
CTSO	-0.071064438	0.0194522753317268	0.0354886874259768	1081
CAST	-0.071086709	0.0194141102103634	0.035422267021807	1081
CCPG1	-0.071110723	0.0193730320113832	0.0353537213397331	1081
NET1	-0.071115982	0.0193640472652704	0.035340526559057	1081
APBB1	-0.071124874	0.0193488610490906	0.0353160103138471	1081
CPNE3	-0.071125929	0.0193470607451775	0.0353159241311707	1081
PRRX1	-0.071141758	0.0193200566578449	0.0352730235066418	1081
LOC8474(	-0.071144598	0.019315215163358	0.035267380537102	1081
PRKX	-0.071147724	0.0193098884189766	0.0352608504532526	1081
C17orf69	-0.071183919	0.0192482926461484	0.0351611221759757	1081
LIMS3-LC	-0.071219564	0.0191878004195665	0.0350633381215682	1081
OR2AG2	-0.071230164	0.0191698437743407	0.0350337025160204	1081
GPC3	-0.071270319	0.0191019522975832	0.0349191314913624	1081
CSN1S1	-0.071290756	0.0190674785965552	0.0348624393109098	1081
PCDH9	-0.07130544	0.0190427436054738	0.0348266972248997	1081
OR2F1	-0.071333322	0.018995852788355	0.0347535604778322	1081
LHFPL1	-0.071380461	0.0189168081844651	0.0346278147093185	1081
SERPINB2	-0.071384853	0.0189094579623998	0.0346175055118634	1081
GIPC3	-0.07139397	0.0188942070199695	0.0345990183089979	1081
FMO4	-0.071398006	0.0188874599211179	0.0345898072828109	1081
F13A1	-0.071413126	0.0188622006117342	0.0345466889102087	1081
HNRPLL	-0.071426404	0.0188400435266922	0.0345123830904078	1081
CCNJL	-0.071442594	0.0188130578833951	0.0344692179227826	1081

MAP3K5	-0.071464735	0.0187762069795038	0.0344142197800112	1081
FBXL16	-0.071479324	0.0187519587849124	0.0343760314607389	1081
ZCCHC11	-0.071493859	0.0187278288585907	0.0343349210371595	1081
MAP7D3	-0.071495379	0.018725307123234	0.0343344458997524	1081
CNOT2	-0.071503193	0.0187123470885363	0.034315906426815	1081
UGT2B15	-0.071504121	0.0187108083862498	0.034315906426815	1081
AMBRA1	-0.071509287	0.0187022470022511	0.0343067534019622	1081
NFKB1	-0.071521638	0.0186817872880801	0.0342723435860462	1081
GAL3ST3	-0.071523307	0.0186790245854091	0.0342703961997327	1081
LTA4H	-0.071538154	0.0186544609379383	0.0342333614936196	1081
TPH1	-0.0715483	0.0186376903722901	0.0342070219159789	1081
ARHGAP1	-0.071549416	0.0186358466055261	0.0342067544299157	1081
SMCHD1	-0.07161099	0.018534363504126	0.0340297806043218	1081
C1QTNF9	-0.071648794	0.0184722965873491	0.033928192446403	1081
ANKS6	-0.07165926	0.018455144302838	0.03389994331705	1081
SRPX2	-0.071693276	0.0183994983310829	0.0338191509014293	1081
CYR61	-0.071697786	0.0183921303835614	0.0338086929990733	1081
LIMS3	-0.071708001	0.0183754541363573	0.0337811209688736	1081
SR140	-0.071708473	0.0183746836570694	0.0337811209688736	1081
SCARA3	-0.071734246	0.0183326689527595	0.0337116947561242	1081
ABCC3	-0.071750494	0.0183062240279832	0.0336692123659018	1081
GPR27	-0.071752561	0.0183028629502463	0.0336661042853097	1081
ILDR2	-0.071773367	0.0182690543182988	0.0336131244170362	1081
BRD8	-0.071809425	0.018210591585933	0.0335147421431227	1081
C16orf89	-0.071809487	0.0182104905484971	0.0335147421431227	1081
KRT5	-0.071817139	0.0181981052898572	0.0334978829554251	1081
SAMD12	-0.071836512	0.0181667814940172	0.0334448260701837	1081
CHMP2B	-0.071887173	0.0180850909195071	0.0332989814983522	1081
OR5P2	-0.071904367	0.0180574374364139	0.0332541436289019	1081
LRRC4B	-0.071951623	0.0179816295356889	0.0331357415840151	1081
TMEM39A	-0.071982003	0.0179330405108655	0.0330492270690134	1081
C1orf168	-0.072011731	0.0178856049031931	0.0329829284854289	1081
SORT1	-0.072027671	0.0178602151430156	0.032942138258199	1081

DACT2	-0.072030447	0.0178557972909455	0.032937005441452	1081
TMEM178	-0.072034109	0.0178499696942813	0.0329292710156865	1081
C6orf59	-0.072045413	0.0178319935665738	0.0329021350429227	1081
ALDH1A1	-0.072046202	0.0178307387558323	0.0329021350429227	1081
FAM157A	-0.07207273	0.0177886168167001	0.0328311210052609	1081
EYA2	-0.072116703	0.0177189845307581	0.0327175933429993	1081
THAP6	-0.07212862	0.0177001559748108	0.0326865945537673	1081
MMACHC	-0.07214984	0.0176666706049754	0.0326329616117027	1081
MCF2L	-0.072155186	0.0176582426804681	0.0326203850342072	1081
CTCF	-0.072159463	0.0176515026977308	0.0326109246006774	1081
ATP2A2	-0.072164521	0.0176435361342641	0.0325991961317758	1081
PSD4	-0.072214315	0.017565268338217	0.0324635165755396	1081
SFRS12IP	-0.072241369	0.0175228712555766	0.0323881311536462	1081
JUN	-0.072258443	0.0174961602405081	0.0323476641010495	1081
TBX5	-0.072301125	0.017429543988411	0.0322333758970476	1081
HNRNPK	-0.072305278	0.0174230742568594	0.0322243693448799	1081
EFHD1	-0.072330959	0.0173831113684607	0.0321534091008761	1081
ANGPTL5	-0.072346492	0.017358980258005	0.0321117224331965	1081
LOC44120	-0.072348725	0.0173555138387015	0.0321082584517074	1081
PAPLN	-0.072349822	0.0173538101231976	0.0321082584517074	1081
FST	-0.07237855	0.0173092682258969	0.0320374134886708	1081
GATC	-0.072403605	0.0172705028772748	0.0319715383626816	1081
TCERG1	-0.072430277	0.0172293185180795	0.0319099587704984	1081
C14orf166	-0.0725057	0.0171133243419465	0.0317097055618572	1081
KIAA1522	-0.072506208	0.0171125453954936	0.0317097055618572	1081
DDX25	-0.072512973	0.0171021760874524	0.0316977953152754	1081
ARG1	-0.07252816	0.0170789147780934	0.0316575946084553	1081
TDRD1	-0.072579923	0.0169998438968979	0.0315197289740458	1081
GCC1	-0.072586231	0.0169902308644403	0.0315077051241968	1081
CASKIN1	-0.072626743	0.0169286006342811	0.0313991952654076	1081
SMC2	-0.072637095	0.0169128827684836	0.0313758194466435	1081
LOC10028	-0.072640388	0.0169078870158504	0.0313694404065487	1081
GCK	-0.072671373	0.016860932441347	0.0312880880647508	1081

GABRG1	-0.072696456	0.016823005106593	0.0312205838666343	1081
ATG16L1	-0.072699291	0.016818724305024	0.0312155151211266	1081
PHF14	-0.072701936	0.016814729966291	0.0312109771649251	1081
TMEM170	-0.072706441	0.0168079288717193	0.031201228079689	1081
CAMK2N	-0.072771028	0.0167106914211752	0.0310407449220446	1081
LOC14814	-0.072791952	0.0166792954397456	0.030985282795433	1081
CRTAP	-0.072795219	0.0166743978101991	0.0309790412142821	1081
TSPAN5	-0.072810995	0.0166507667208659	0.0309408445338847	1081
BEND6	-0.07281253	0.0166484694378257	0.0309394295964022	1081
FOXC1	-0.072839009	0.0166088763725093	0.0308686977141987	1081
AOX1	-0.072842511	0.016603646773666	0.0308618254526206	1081
HSPB2	-0.072884052	0.0165417162399728	0.0307552259948548	1081
CCDC88A	-0.07289601	0.0165239258750728	0.0307249849320049	1081
DPT	-0.072905984	0.0165091010476791	0.0307030872050863	1081
C6orf97	-0.072908081	0.016505986032856	0.030700128209019	1081
MYL7	-0.072954287	0.0164374696352973	0.030583986866451	1081
HIST3H3	-0.072954704	0.016436851027673	0.030583986866451	1081
ZNF497	-0.072964913	0.0164217478309892	0.0305603796817809	1081
ZNF213	-0.072965599	0.0164207335100889	0.0305603796817809	1081
UNC13C	-0.072978384	0.0164018359251747	0.0305289646782449	1081
SLC10A1	-0.07299496	0.0163773638939581	0.0304890486686799	1081
SHROOM	-0.073043635	0.0163056860572776	0.0303612205956056	1081
HFE	-0.073044564	0.0163043208250396	0.0303612205956056	1081
LANCL2	-0.073056312	0.0162870638895269	0.030332153282289	1081
EGFLAM	-0.073059404	0.0162825239816026	0.0303265019979091	1081
C10orf18	-0.073085422	0.0162443723530343	0.0302610394906489	1081
BPNT1	-0.073095989	0.0162288992090478	0.0302350110576406	1081
SYNRG	-0.073191803	0.016089190086902	0.0299848670088884	1081
KDM1B	-0.07321574	0.0160544533296736	0.0299321575498636	1081
PNN	-0.073237303	0.0160232158400109	0.0298822146914479	1081
SCHIP1	-0.073238472	0.0160215241663406	0.0298818261578494	1081
C14orf180	-0.073250279	0.016004444661634	0.0298527349730202	1081
LOC44186	-0.073278987	0.0159629839641443	0.0297836724977018	1081

DSTN	-0.073282636	0.0159577205556505	0.0297793682810171	1081
KCNC4	-0.073285909	0.0159530011047655	0.0297733191824626	1081
ZFP1	-0.073289244	0.0159481936978367	0.0297671047941184	1081
SLC16A5	-0.073333819	0.0158840565188225	0.0296611344615943	1081
FHIT	-0.073367472	0.0158357840787701	0.0295737341491448	1081
GLIS2	-0.073377981	0.0158207364763822	0.0295483716221694	1081
IARS2	-0.073448678	0.0157198320395698	0.0293814638713219	1081
TTC26	-0.073482542	0.0156716990383913	0.029299895789178	1081
IGDCC3	-0.073516594	0.0156234302297769	0.0292150749075332	1081
RBP4	-0.073569965	0.0155480379730132	0.0290902967369138	1081
TRIM72	-0.073579456	0.0155346652436384	0.0290706763895119	1081
C2CD3	-0.073627559	0.015467040343435	0.028957576925511	1081
EHD3	-0.073633423	0.0154588134190439	0.0289448644229612	1081
TMEM132	-0.073646227	0.0154408656981845	0.0289139467828524	1081
NPNT	-0.073648611	0.0154375252618252	0.0289103789531904	1081
SCUBE2	-0.07365133	0.0154337180473208	0.0289059362275268	1081
KLF10	-0.073713718	0.0153465614530506	0.0287533929010141	1081
XRCC5	-0.073732376	0.0153205799141837	0.0287127251252425	1081
C13orf23	-0.073754598	0.0152896865619091	0.0286574930954279	1081
RRAGC	-0.07376654	0.0152731077341588	0.0286290830282524	1081
EDN2	-0.073780019	0.015254412575784	0.028602023579595	1081
OXCT1	-0.073821073	0.015197598205344	0.028511418871918	1081
LRRC1	-0.073848476	0.0151597786832484	0.0284484156100642	1081
KCTD4	-0.073853356	0.0151530524552157	0.028438442492111	1081
PIP5K1A	-0.073853912	0.0151522861544048	0.028438442492111	1081
TIMP4	-0.073906158	0.0150804420958557	0.0283127219031698	1081
PMP2	-0.073911424	0.0150732170971201	0.0283017949875556	1081
RAD51L1	-0.073920182	0.0150612078245616	0.0282818821425981	1081
C14orf162	-0.073943399	0.015029413548981	0.0282274413522489	1081
BFAR	-0.07395867	0.0150085329253694	0.028190852753526	1081
KIAA1529	-0.073983182	0.0149750702120741	0.028132056497827	1081
MTUS2	-0.073990687	0.0149648378506516	0.0281166441430121	1081
INTS10	-0.074041369	0.0148958983599067	0.027997562274708	1081

LBXCOR1	-0.074055191	0.0148771461283706	0.0279649257027178	1081
SBF1	-0.074056561	0.0148752879907815	0.0279640422335101	1081
PRKRIR	-0.074089523	0.0148306563246383	0.027887946575174	1081
MXRA8	-0.074096402	0.0148213575081238	0.0278730626401377	1081
PLEKHG4	-0.074135448	0.0147686695764402	0.0277765706859666	1081
DCLK1	-0.074146066	0.01475436958766	0.0277522666053605	1081
ABCC4	-0.074182989	0.0147047411260559	0.0276692518199511	1081
ANXA8L2	-0.074184214	0.0147030965095138	0.0276692518199511	1081
UNC5CL	-0.074219953	0.0146552042159169	0.0275863473116843	1081
HADHA	-0.074236671	0.0146328473600829	0.0275468376851574	1081
CCDC85C	-0.074241522	0.0146263665207637	0.0275396617887719	1081
LOH12CR	-0.074252094	0.0146122498786571	0.0275174647507156	1081
CASC3	-0.074270353	0.0145878971528216	0.0274817313048336	1081
ST3GAL3	-0.07430428	0.0145427426087574	0.0274053835223029	1081
KLK3	-0.074320283	0.0145214868173956	0.027370448347346	1081
MYLIP	-0.074364374	0.0144630649977312	0.0272628842873861	1081
ZNF274	-0.074370049	0.0144555601431314	0.0272538380049959	1081
OSGEPL1	-0.074380757	0.0144414093534847	0.0272297071720283	1081
SGIP1	-0.074390001	0.0144292025314967	0.0272092375734345	1081
CMAH	-0.074401373	0.0144141984425599	0.0271834888246929	1081
DLEU2L	-0.074446414	0.0143549084363873	0.0270792799373557	1081
STK36	-0.074449922	0.0143503000948401	0.0270731218777444	1081
ZKSCAN2	-0.074452471	0.0143469518626964	0.0270693401961242	1081
SHMT1	-0.074462912	0.0143332454163738	0.0270510800930158	1081
STOM	-0.074482824	0.01430713712478	0.0270043359297941	1081
FAM83B	-0.074486476	0.0143023525243998	0.0270003281489486	1081
ZNF44	-0.074488972	0.0142990842397165	0.0269992550388124	1081
HTR2B	-0.074542992	0.0142284999959638	0.0268760555479316	1081
MCL1	-0.0745448	0.0142261430253194	0.0268741233350581	1081
SLC22A13	-0.074551693	0.0142171596718109	0.0268621911075437	1081
ARVCF	-0.074566343	0.0141980845332195	0.0268286664404565	1081
CWC22	-0.074586339	0.0141720845647511	0.0267820491141568	1081
NFX1	-0.074614148	0.0141359950500489	0.0267188609760963	1081

CNGA3	-0.074619178	0.0141294753516219	0.0267090439108964	1081
SLC14A2	-0.074629171	0.0141165313317555	0.0266880621017039	1081
MRVI1	-0.074660006	0.0140766582212545	0.0266191945806037	1081
LOC28623	-0.074668719	0.0140654099221243	0.0266004208487789	1081
KLHL12	-0.074691427	0.0140361300130827	0.0265475391149704	1081
DISC1	-0.074722679	0.0139959209484136	0.0264814339725549	1081
SNURF	-0.074743879	0.0139687024473872	0.026434900028428	1081
LOC64730	-0.074749419	0.0139615974828025	0.0264264193640005	1081
NTN1	-0.074751024	0.0139595394999298	0.0264250068808575	1081
FGF18	-0.074791004	0.0139083662948245	0.0263306116914989	1081
PRRG1	-0.07482395	0.0138663211968399	0.0262608856360196	1081
YIPF6	-0.0748357	0.013851352861907	0.0262350040807744	1081
GPRIN2	-0.074845115	0.013839369642815	0.0262172373005932	1081
AGAP8	-0.07489812	0.0137720762550084	0.0260995744268245	1081
KIAA0040	-0.074910019	0.0137570089840394	0.0260734731379692	1081
ZNF747	-0.074913521	0.0137525776871606	0.0260675270519242	1081
PI15	-0.074935914	0.0137242706149025	0.0260163198962276	1081
COG7	-0.075036711	0.0135974871364614	0.0257954024262186	1081
MLNR	-0.075047588	0.0135838672729129	0.0257719915438717	1081
C17orf48	-0.075054906	0.0135747121081942	0.0257619006518673	1081
ZNF804B	-0.075056868	0.013572257333106	0.025759668737085	1081
ADAMTS2	-0.075064896	0.0135622212928026	0.0257454719132594	1081
MED22	-0.075071902	0.013553467910478	0.0257337050948708	1081
RPS15A	-0.07507494	0.0135496736930037	0.0257289260576454	1081
C14orf184	-0.075084053	0.0135382971728408	0.0257097470349621	1081
COPS2	-0.075099286	0.0135193006466744	0.0256760923472477	1081
STOML3	-0.075110184	0.0135057238738549	0.0256527255740908	1081
DNAJC3	-0.075125202	0.013487035009799	0.0256220596258394	1081
FOSB	-0.075145973	0.0134612237053922	0.0255778486648864	1081
GUSBL2	-0.07518467	0.0134132536470674	0.0254915089358654	1081
ZNF578	-0.075212228	0.0133791843785702	0.0254363598816814	1081
MAPK4	-0.075234979	0.0133511149066383	0.0253853902590116	1081
IQCB1	-0.075234988	0.0133511044280302	0.0253853902590116	1081

C17orf44	-0.07523781	0.013347626207103	0.0253853902590116	1081
GDF5	-0.075254822	0.0133266761531594	0.0253519250938467	1081
HIATL2	-0.075256868	0.0133241590644875	0.0253508863198055	1081
HERC2P2	-0.075265282	0.0133138095397348	0.0253342307169798	1081
RAB6A	-0.075267704	0.0133108320990506	0.0253327078540741	1081
LRRC31	-0.075322926	0.0132430975252545	0.0252133257391789	1081
BDH2	-0.075367074	0.0131891667456662	0.025117769341222	1081
EFHC2	-0.075413848	0.0131322383779961	0.0250164484278705	1081
A2M	-0.075426981	0.0131162931637543	0.0249884363328759	1081
ADCYAP1	-0.075442805	0.0130971047722624	0.0249542396327651	1081
ORC2L	-0.075466503	0.0130684131377855	0.0249066397976053	1081
PLGLB2	-0.075504097	0.0130230104889558	0.0248224568360312	1081
C14orf148	-0.075507874	0.0130184568388091	0.0248161253801863	1081
HAS3	-0.075515369	0.0130094252376168	0.0248012559299509	1081
LSAMP	-0.075564508	0.0129503450141867	0.0246981462017196	1081
ARC	-0.075565063	0.0129496785439789	0.0246981462017196	1081
AGBL4	-0.075566817	0.0129475747565629	0.0246981462017196	1081
PEX2	-0.0755969	0.0129115302470369	0.0246332774719725	1081
C5orf15	-0.075613949	0.0128911415467819	0.0245990382172892	1081
SYN3	-0.075631156	0.0128705923573404	0.0245644796815369	1081
C4orf38	-0.075645891	0.0128530193329485	0.0245355893548989	1081
ZNF687	-0.075692777	0.0127972423661139	0.0244383778050397	1081
PDXDC1	-0.075697769	0.0127913156761855	0.0244293756443645	1081
CYP27C1	-0.075711309	0.0127752549629444	0.0244010155711117	1081
GPR152	-0.075715292	0.0127705339155466	0.0243966245356744	1081
LOC72917	-0.075757238	0.0127209064109515	0.0243087326549628	1081
KAZ	-0.075794047	0.012677497839451	0.0242349776025565	1081
DPY19L2	-0.075818349	0.012648910292508	0.0241826229327677	1081
C4orf46	-0.075852266	0.0126091075027457	0.0241156812534712	1081
C6orf132	-0.075869574	0.0125888389701189	0.0240814890374176	1081
CD207	-0.07586999	0.0125883515623024	0.0240814890374176	1081
GFOD2	-0.075871052	0.0125871087306865	0.0240814890374176	1081
NPTX1	-0.075897399	0.0125563137265662	0.0240306802224658	1081

MFSD9	-0.075922104	0.0125274983381253	0.0239778103583405	1081
ACP6	-0.07597708	0.0124635859491492	0.0238622827357546	1081
MYH7B	-0.07600068	0.0124362378402887	0.0238121862268431	1081
PRPH2	-0.076002741	0.0124338518654906	0.0238098807823487	1081
SYT9	-0.076009635	0.0124258743962113	0.0237968665948928	1081
CHUK	-0.076028429	0.0124041491746956	0.0237575190268344	1081
PPP2R5E	-0.076063314	0.0123639133097923	0.0236894648683438	1081
TGM5	-0.076090786	0.01233230867017	0.0236311574394154	1081
C3orf49	-0.076098727	0.0123231869769607	0.0236159248145807	1081
MYADM	-0.07610127	0.0123202670528086	0.0236125753761612	1081
TRPM6	-0.076106542	0.0123142148280014	0.0236032214757459	1081
GABARA[illegible]	-0.076126351	0.0122915006564053	0.023561926037043	1081
C1orf130	-0.076131161	0.012285990020781	0.0235536038226716	1081
EDAR	-0.076131414	0.0122857005413444	0.0235536038226716	1081
PAMR1	-0.076156711	0.0122567589547081	0.0235065126754184	1081
ANO2	-0.07618189	0.0122280118974353	0.0234603142546509	1081
CPAMD8	-0.07619458	0.0122135456402604	0.0234347915918702	1081
FTO	-0.076209321	0.012196761923044	0.0234070466742613	1081
SDCCAG[illegible]	-0.076213498	0.0121920094668374	0.0234001553648475	1081
BBS2	-0.076270845	0.0121269280363902	0.0232885572252698	1081
USP43	-0.076273642	0.0121237613870636	0.0232869158221201	1081
LOC6536[illegible]	-0.076305005	0.0120883073070229	0.0232254602479711	1081
CACNB2	-0.076318285	0.0120733225788294	0.0231988824256504	1081
ZIC4	-0.076320032	0.0120713525278791	0.0231974007211506	1081
MYH1	-0.076331184	0.0120587839063437	0.0231775786464358	1081
DYNC1I1	-0.07633196	0.0120579103999222	0.0231775786464358	1081
DEPDC5	-0.076364473	0.0120213346489597	0.0231166271958089	1081
ITIH3	-0.076368945	0.012016310881658	0.0231091725738426	1081
ZNF732	-0.076456295	0.0119185680377892	0.0229299544571925	1081
ENC1	-0.076463535	0.011910498911445	0.0229166189657173	1081
MAPRE2	-0.076488948	0.011882211340887	0.022864375533687	1081
EXOC2	-0.076531514	0.0118349640898943	0.0227799877308352	1081
MMP21	-0.076539605	0.0118260022406845	0.0227670886026939	1081

LYVE1	-0.076547755	0.0118169809976665	0.0227540701775943	1081
KCNV2	-0.076591882	0.0117682431639753	0.0226688906615301	1081
PGR	-0.076593861	0.0117660618304102	0.02266856227753	1081
PTPRJ	-0.076608669	0.0117497490631479	0.0226375951489207	1081
TMEM107	-0.076612439	0.0117455989082256	0.0226317637499956	1081
FAM26E	-0.076620325	0.0117369223127678	0.0226172087230445	1081
ATP6AP1I	-0.076625	0.0117317817129682	0.0226113042116295	1081
ARFGAP3	-0.07665496	0.0116988828832818	0.0225525354721256	1081
ZNF93	-0.076675417	0.0116764661837598	0.0225114758610242	1081
CDC14A	-0.076687025	0.0116637636779902	0.0224891385234603	1081
COQ7	-0.076748765	0.0115964051811506	0.0223699686272411	1081
SLCO1C1	-0.076816204	0.0115232236790509	0.0222373159320319	1081
SKINTL	-0.076818788	0.0115204276698588	0.0222340501445971	1081
PRKAB1	-0.076822344	0.0115165810110097	0.0222287558174562	1081
ENOSF1	-0.076853379	0.0114830575088801	0.0221725480222745	1081
C1orf157	-0.076856792	0.0114793760909697	0.0221696895170726	1081
URGCP	-0.076881294	0.011452978700407	0.0221250724894225	1081
RAPGEF3	-0.076944186	0.0113854669942537	0.0219988713408057	1081
EMR3	-0.076961058	0.011367416712204	0.0219682089089936	1081
KATNAL2	-0.076981	0.0113461134452349	0.0219312469155878	1081
CTR9	-0.076992319	0.0113340377729404	0.0219105477839229	1081
ARSE	-0.076997067	0.0113289759694778	0.0219044266153306	1081
LOC10013	-0.077011347	0.0113137628962191	0.0218771120699112	1081
CPA3	-0.077041998	0.0112811721136299	0.0218161862560309	1081
WDR87	-0.077048121	0.0112746708368932	0.0218057069901319	1081
PEA15	-0.077052872	0.0112696298914864	0.0218001434764733	1081
ZNF587	-0.077060454	0.0112615873927791	0.0217866780012999	1081
LGI2	-0.077066556	0.0112551192280013	0.021780439658798	1081
SNIP1	-0.077067176	0.0112544621480016	0.021780439658798	1081
MARVEL	-0.077097763	0.0112220903118359	0.0217227836390828	1081
NMT2	-0.077101707	0.0112179217998611	0.021716801331751	1081
PITPNA	-0.077115471	0.0112033866925106	0.0216907472292769	1081
SLC26A4	-0.077126673	0.0111915696948374	0.0216699511248077	1081

CAD	-0.077136395	0.0111813217451571	0.0216521894219158	1081
SYCP2L	-0.077156338	0.0111603275377698	0.0216136125983822	1081
TCF23	-0.077162772	0.0111535612172746	0.0216025853977499	1081
SULF2	-0.077184591	0.0111306447681589	0.0215623462693106	1081
EPHA7	-0.077189189	0.0111258208720339	0.0215550741937991	1081
LOC33929	-0.077192711	0.0111221266766552	0.0215499896028873	1081
ZNF138	-0.077267589	0.0110438510212364	0.0214106802832072	1081
RIC8B	-0.077268527	0.0110428742337406	0.0214106802832072	1081
GGTA1	-0.077271159	0.0110401314534363	0.0214075895783497	1081
ZNF77	-0.077311613	0.0109980583247286	0.0213280597758624	1081
USP6NL	-0.077319733	0.010989630136898	0.0213158192863287	1081
DLST	-0.077331951	0.0109769597479374	0.0212932936083004	1081
TSPO2	-0.077393635	0.010913188899167	0.0211818277650755	1081
LENG8	-0.07739405	0.0109127610247368	0.0211818277650755	1081
ZPBP	-0.077404977	0.0109014992225411	0.0211632169064363	1081
LOC44129	-0.077414993	0.0108911858864818	0.0211453663951395	1081
KRR1	-0.07743224	0.0108734463457012	0.0211189333430534	1081
CPO	-0.077459454	0.0108455069819764	0.0210669216548974	1081
ZDHHC23	-0.077469743	0.0108349606631356	0.0210502731757033	1081
FLJ43950	-0.077484784	0.0108195593770849	0.0210244066413983	1081
ATXN7L1	-0.077503958	0.0107999536737396	0.0209923839017256	1081
HRASLS5	-0.077513332	0.0107903813439229	0.0209758016185783	1081
GPC6	-0.077515541	0.010788126476307	0.0209734421796183	1081
VASH2	-0.077519236	0.0107843550043272	0.0209681335355827	1081
NCOR2	-0.077534902	0.0107683814368529	0.0209390969155793	1081
TMEM108	-0.07754257	0.0107605697443304	0.0209259269716706	1081
PMS2CL	-0.077557154	0.0107457272308061	0.0208990804271664	1081
GRB7	-0.077557296	0.0107455830709087	0.0208990804271664	1081
DUOXA1	-0.07755768	0.0107451923270477	0.0208990804271664	1081
C1orf9	-0.077560221	0.0107426083048685	0.0208990675327451	1081
PDE4B	-0.077640094	0.0106616632138145	0.0207516140523955	1081
TFAP2A	-0.077650449	0.0106512084425539	0.0207372759326614	1081
SYT7	-0.077653949	0.0106476772669913	0.0207324046533481	1081

ZKSCAN5	-0.077692054	0.010609296046783	0.02066166558995	1081
SPDYE8P	-0.077707189	0.0105940856404406	0.0206340380186287	1081
YY1	-0.077738744	0.0105624363349234	0.0205783636331752	1081
HSPA2	-0.077807722	0.010493542167089	0.0204540306682155	1081
ANKRD42	-0.077818845	0.010482470699817	0.0204344273512496	1081
SFRS14	-0.07785424	0.0104473073011663	0.0203698224527677	1081
PTX3	-0.077865169	0.0104364709275903	0.0203506637146748	1081
PEX7	-0.077877243	0.0104245108998019	0.0203293099783649	1081
NLGN4Y	-0.077881682	0.0104201173871649	0.0203227093391846	1081
ROBO1	-0.077912531	0.0103896260365243	0.0202691280753226	1081
ZNF74	-0.077922096	0.0103801885357999	0.02025267777597	1081
FAM190B	-0.077945207	0.0103574158573224	0.0202160781363976	1081
C1orf124	-0.077956583	0.0103462231314272	0.0201961884673063	1081
RG9MTD3	-0.077978349	0.0103248377234263	0.0201583500618746	1081
PNMAL2	-0.07812547	0.0101813131705757	0.0198916258191492	1081
FAM198A	-0.078180179	0.0101283958379531	0.0197939982688753	1081
GPR63	-0.078190847	0.0101181064309035	0.0197758080964927	1081
PTRF	-0.078203799	0.0101056254134597	0.0197552473512029	1081
PCDHA2	-0.078211149	0.0100985487102586	0.0197433291700465	1081
C9orf130	-0.078238617	0.0100721421033172	0.0196974374013518	1081
RUNDC1	-0.078239952	0.0100708600115305	0.0196968422264352	1081
FEZF2	-0.078245802	0.0100652451448746	0.0196877719626662	1081
FAM95B1	-0.078248338	0.0100628115088403	0.019684923076868	1081
SPDYE7P	-0.078270045	0.0100420039484568	0.0196461269827776	1081
GRM3	-0.078306326	0.0100073112725849	0.0195801559427179	1081
ATL1	-0.078319985	0.00999427785298493	0.0195584541818906	1081
ZDHHC2	-0.078334813	0.0099801462689006	0.0195326966469448	1081
INTS9	-0.078336911	0.00997814857702751	0.019532582167352	1081
BHLHE40	-0.078351125	0.00996462092594429	0.0195079969439405	1081
CLDND1	-0.078360996	0.00995523599021682	0.0194915180311904	1081
SGK2	-0.078375808	0.00994116878343842	0.0194658675293903	1081
MAOB	-0.078382476	0.00993484184731755	0.0194553697884915	1081
GLP1R	-0.078414914	0.00990411408280076	0.0194008535781818	1081

FOXA1	-0.078420348	0.00989897502095562	0.019392672546645	1081
HFM1	-0.078436627	0.00988359294055845	0.019368188695287	1081
CDADC1	-0.07845285	0.00986828454678307	0.0193438349902642	1081
SCAND2	-0.078457693	0.00986371917917782	0.0193386494272056	1081
RGPD1	-0.078471947	0.0098502918558201	0.0193142037605116	1081
ASB11	-0.078494364	0.00982920877871121	0.0192784939000231	1081
CAPN11	-0.078497181	0.00982656238866407	0.0192751800700718	1081
POLN	-0.078519898	0.00980524313652129	0.0192371078092347	1081
CCDC147	-0.078523484	0.00980188095586264	0.0192323845189299	1081
SOX8	-0.078524106	0.0098012982338486	0.0192323845189299	1081
FMN1	-0.078557183	0.00977034122007054	0.0191779717312989	1081
CTNNA2	-0.078597515	0.009732711419926	0.0191096950832758	1081
LZIC	-0.078635621	0.00969727677487409	0.0190438331672683	1081
OTC	-0.078672625	0.00966297661065151	0.0189820247510068	1081
BCL6	-0.078691809	0.00964523718373116	0.0189490250698522	1081
SSH2	-0.078728367	0.00961151237910839	0.018890138231916	1081
CLIP4	-0.078729692	0.00961029254543256	0.0188895836986768	1081
C8orf48	-0.078748336	0.00959313549259145	0.0188613814657676	1081
MOGAT2	-0.07875287	0.00958896705463197	0.0188568665868373	1081
MIPEP	-0.078768529	0.00957458401862659	0.0188304202924175	1081
LMOD2	-0.078782212	0.00956203173344148	0.0188094062367131	1081
AMOTL2	-0.078798262	0.00954732644544956	0.018785982735259	1081
TTYH1	-0.078802953	0.00954303234847683	0.018779367652639	1081
PCOLCE2	-0.078830991	0.00951740170149882	0.0187325898658225	1081
C7orf31	-0.078839707	0.00950944706335639	0.0187187620765404	1081
KY	-0.078844777	0.00950482230492634	0.0187114868887659	1081
CERK	-0.078846198	0.00950352585198864	0.0187107631243463	1081
SMN1	-0.078858523	0.00949229367247276	0.0186923026424207	1081
SYT10	-0.078933248	0.00942444401489423	0.0185641365679128	1081
LAMB2L	-0.078933307	0.00942439073176195	0.0185641365679128	1081
FGF13	-0.078980833	0.0093814634614374	0.0184848964623099	1081
DFNA5	-0.078984613	0.00937805655606924	0.0184799911300024	1081
PDE4D	-0.078987231	0.00937569751922985	0.0184771499241719	1081

PCDHA13	-0.078989149	0.00937396992233813	0.0184755526940125	1081
GAN	-0.078992589	0.00937087173615743	0.0184712535347252	1081
RRN3P3	-0.079002528	0.00936192522708494	0.0184554245718393	1081
SPCS3	-0.079032688	0.00933482483457221	0.0184074555896155	1081
CEP78	-0.079039586	0.00932863566930744	0.0183988021889758	1081
TRIM63	-0.079062621	0.00930799632553132	0.0183598928794505	1081
PNLIP	-0.079074342	0.00929751002648916	0.018346394307339	1081
PTPRE	-0.079082265	0.00929042809222402	0.0183342157051188	1081
LOC20218	-0.079082403	0.0092903048356026	0.0183342157051188	1081
ANKRD46	-0.079087066	0.0092861385990738	0.0183293417674252	1081
LIPI	-0.079091881	0.00928183826105253	0.0183226488749538	1081
NLRP11	-0.079138365	0.00924041649297105	0.0182462448785436	1081
KRT15	-0.079139032	0.009239823316123	0.0182462448785436	1081
OSTCL	-0.07915958	0.00922156671151175	0.0182161660525009	1081
LOXL2	-0.079161582	0.00921978943229088	0.0182144413173972	1081
ZNF343	-0.079168682	0.00921348959866013	0.0182055662545374	1081
C6orf176	-0.079196061	0.00918923333814026	0.0181611994110503	1081
FAM184A	-0.079265244	0.00912819259491012	0.0180458724067188	1081
KLK8	-0.079295126	0.00910193860703045	0.0179975022809804	1081
ABCG4	-0.079302816	0.00909519416824076	0.0179859317285963	1081
CADPS	-0.079339933	0.00906269984635075	0.0179234329869169	1081
NFATC2IP	-0.079340323	0.00906235815959899	0.0179234329869169	1081
HSPB7	-0.079344308	0.00905887656343357	0.0179211497957743	1081
SRGAP1	-0.079371314	0.00903530738028973	0.0178762784498072	1081
RAB11FIP	-0.079383468	0.00902471874033948	0.0178588368393064	1081
LMX1B	-0.079447914	0.00896875479163802	0.0177550673425263	1081
NAMPT	-0.079471241	0.00894857443922237	0.0177185995752049	1081
ZSWIM2	-0.079475811	0.00894462548184923	0.0177125214127448	1081
STYX	-0.079505755	0.00891878992033945	0.0176648336392919	1081
TGS1	-0.079521914	0.0089048756917291	0.017640743515575	1081
TF	-0.079530741	0.008897283314901	0.0176277608521683	1081
MIPOL1	-0.079535257	0.00889340112700089	0.0176232112633713	1081
C1orf133	-0.07956054	0.00887169448176571	0.0175836565658373	1081

SERHL2	-0.079574424	0.00885979472112455	0.0175635273230717	1081
KBTBD5	-0.079580146	0.0088548946921451	0.0175562682769839	1081
LOC10027	-0.079601505	0.00883662406089603	0.0175244921940097	1081
RUSC2	-0.079685348	0.0087652320601115	0.0173914704866489	1081
RSRC2	-0.079708974	0.00874520758836573	0.0173585778764043	1081
SNORD1C	-0.079717504	0.00873798794439882	0.0173476660234468	1081
LOC10013	-0.079814369	0.00865637481064326	0.0171975020276537	1081
PLS1	-0.079819233	0.00865229487538913	0.0171910918497597	1081
FAM81A	-0.079833853	0.00864004133650078	0.0171701324577102	1081
COMMD6	-0.079839637	0.00863519816541665	0.0171622007737094	1081
FILIP1L	-0.079852948	0.00862406034757368	0.0171434473753574	1081
MCCD1	-0.079853321	0.00862374918655582	0.0171434473753574	1081
PRPF4	-0.079865939	0.00861320364018969	0.0171286265875243	1081
VAMP1	-0.079893533	0.00859018218871234	0.0170845315620111	1081
PTPLAD1	-0.079900213	0.00858461759203653	0.0170757271094269	1081
MCFD2	-0.079918838	0.00856911881606069	0.0170476889738833	1081
OR7C1	-0.07992511	0.00856390531950255	0.0170389997690251	1081
HIF3A	-0.079995175	0.00850585682274198	0.0169301932313148	1081
ZNF480	-0.080032659	0.00847494678237675	0.0168786755245655	1081
CDS1	-0.080085394	0.00843162912975029	0.0167957251872658	1081
LIN7C	-0.080092898	0.00842548145766782	0.0167851388414476	1081
SCGN	-0.080148386	0.00838014583419735	0.0166997762221689	1081
PALM2-A	-0.080151976	0.00837721990797385	0.0166955970564042	1081
TESK2	-0.080164153	0.00836730312695609	0.0166807839181129	1081
CPE	-0.080167141	0.00836487135250663	0.0166775864406419	1081
LOC10012	-0.08017291	0.00836017784733626	0.016669878524655	1081
NDN	-0.080182382	0.00835247707137564	0.0166561721048171	1081
TRAM1L1	-0.080183075	0.00835191422242686	0.0166561721048171	1081
TMEM159	-0.080230317	0.00831360291464708	0.0165879424470073	1081
BEND5	-0.080254743	0.00829385702081775	0.016552365392331	1081
HSPC159	-0.080298467	0.00825861349389781	0.016488579666459	1081
KCNIP2	-0.080322821	0.00823904139827886	0.0164511338059597	1081
TRPC5	-0.080339971	0.00822528373391778	0.016425291516631	1081

ANAPC4	-0.080339992	0.00822526703596696	0.016425291516631	1081
OBSCN	-0.080371825	0.00819978516937541	0.016380867933069	1081
GPR109A	-0.080377764	0.0081950387112229	0.0163730095048681	1081
FPGT	-0.080381531	0.00819202967064678	0.0163686210786728	1081
ANXA4	-0.080398876	0.0081781871940446	0.0163425831786557	1081
PEX12	-0.080400824	0.00817663335880206	0.0163410991084392	1081
SUGT1P1	-0.080412479	0.00816734476867165	0.0163241552103275	1081
IARS	-0.080435406	0.00814910097230996	0.0162909237954931	1081
FAM135B	-0.08044605	0.00814064304122593	0.0162772460610915	1081
THBS1	-0.080457774	0.00813133649846708	0.0162602515149513	1081
WSB1	-0.080466407	0.00812448967927893	0.0162481727974858	1081
GRP	-0.080468121	0.00812313061965916	0.0162470677455355	1081
HPS5	-0.080476302	0.00811664760132623	0.0162357130303562	1081
CLCN1	-0.080490366	0.00810551447953972	0.0162156067232147	1081
KIAA2022	-0.080501475	0.00809672950185664	0.0162033111922577	1081
ANKHD1	-0.080530595	0.00807374219360852	0.0161611224652468	1081
EGR2	-0.080561833	0.00804914832534389	0.0161134942873947	1081
HNRNPA1	-0.080570641	0.00804222636250419	0.0161012373357828	1081
TMED4	-0.080599783	0.00801936059234382	0.0160586500131974	1081
SYT15	-0.080609548	0.00801171138675512	0.0160449275162722	1081
OR2A7	-0.080641237	0.00798693433058003	0.0160000787678535	1081
GLUL	-0.080642801	0.0079857129540866	0.0159992230194008	1081
CDH7	-0.080650527	0.00797968355400082	0.0159887333593939	1081
FAM107A	-0.080652317	0.00797828723936018	0.015987525757178	1081
DPYD	-0.080702895	0.00793892130860391	0.0159118067616979	1081
ENOX1	-0.080751644	0.00790114290985278	0.0158408164728288	1081
MGC4473	-0.080757359	0.00789672455726232	0.0158335340107544	1081
PSIMCT-1	-0.08076163	0.00789342443142834	0.0158284924518339	1081
ADHFE1	-0.08078941	0.00787198684446483	0.0157870756577147	1081
PATZ1	-0.080800401	0.00786352001618156	0.0157732361571221	1081
P4HA3	-0.080801147	0.00786294548012757	0.0157732361571221	1081
TIFA	-0.080829945	0.00784080042577467	0.0157307961933304	1081
DCAF5	-0.080870421	0.00780976928214306	0.0156763453755253	1081

TBL1X	-0.080874241	0.00780684655570997	0.0156720402456181	1081
FER1L5	-0.080875282	0.00780604983233388	0.0156720025784698	1081
SGCE	-0.080912864	0.00777734842843828	0.015619049356085	1081
FAM129B	-0.080924919	0.00776816219072504	0.0156052679828636	1081
HTT	-0.08093211	0.00776268714998273	0.0155973800754441	1081
C3orf48	-0.08094461	0.00775317769997944	0.0155798269093352	1081
PICALM	-0.081009452	0.00770401638925583	0.0154856725365754	1081
ZNF35	-0.081030068	0.00768844477090144	0.0154559145703831	1081
DZIP3	-0.081039778	0.00768112025879378	0.0154473562557054	1081
TMEM204	-0.081041631	0.00767972272721361	0.015446087693662	1081
MKX	-0.081042877	0.00767878356873197	0.0154457408878787	1081
ABI3BP	-0.081043152	0.00767857618465127	0.0154457408878787	1081
ZNF295	-0.081051894	0.00767198958724369	0.0154366989847207	1081
LOC10013	-0.081063071	0.00766357529223311	0.015422849576627	1081
MOCS1	-0.081075834	0.00765397712727647	0.0154050723577764	1081
DPP10	-0.081094781	0.00763974770118175	0.0153779693685358	1081
HMGCLL	-0.081096853	0.00763819324461374	0.0153763768274951	1081
LRRC37A	-0.08114	0.00760588467134795	0.0153174594326007	1081
C20orf165	-0.081147521	0.00760026486776396	0.0153076720417021	1081
GNAT2	-0.081181078	0.00757523859799108	0.015261844340087	1081
MED4	-0.081196145	0.00756402593857813	0.0152423029441489	1081
TCTE1	-0.081221041	0.00754553094068215	0.0152080761180632	1081
PCDHA9	-0.081243844	0.00752862641284548	0.0151770418379638	1081
TGFBR3	-0.081245931	0.00752708060721352	0.0151754442386225	1081
TAF2	-0.08125062	0.00752360996216284	0.0151699652374908	1081
PPP1R16E	-0.081264664	0.00751322163338794	0.0151520522379217	1081
TSEN2	-0.081275799	0.00750499411326167	0.0151384906790484	1081
KANK4	-0.08131206	0.00747825820011723	0.0150906051729301	1081
KIAA1804	-0.081331178	0.00746419630247455	0.0150652474214357	1081
ST7L	-0.081334843	0.00746150320920739	0.0150613208566616	1081
SIDT2	-0.081364775	0.00743954230925631	0.0150200019863669	1081
WFDC6	-0.081386	0.0074240043906352	0.01499313969417	1081
PCDHGC4	-0.081394871	0.00741751882023857	0.0149830458872662	1081

YY1AP1	-0.081454562	0.00737401197902678	0.0149011406678197	1081
R3HDM2	-0.081497081	0.0073431605442458	0.0148417747731345	1081
ATP13A4	-0.081512092	0.00733229637817688	0.0148242784562799	1081
CDAN1	-0.081518446	0.00732770222203497	0.0148164770915281	1081
MEX3D	-0.081578898	0.00728412064035486	0.0147313131512849	1081
SNHG8	-0.08157907	0.00728399727889903	0.0147313131512849	1081
NDUFS1	-0.081581542	0.00728221954587845	0.0147304260218618	1081
C21orf62	-0.081587996	0.0072775820626294	0.0147232400456787	1081
DLG5	-0.081636437	0.0072428550791611	0.0146552145007734	1081
RHOB	-0.081638929	0.00724107227235163	0.014653078947918	1081
TNN	-0.08166244	0.0072242752783309	0.0146234953257612	1081
BEX4	-0.081692535	0.00720282478254427	0.0145815400707823	1081
CTXN3	-0.08170266	0.00719562096371785	0.01456842053408	1081
GCOM1	-0.081717675	0.00718494944720036	0.0145482768734397	1081
LOC10013	-0.081718517	0.00718435176786114	0.0145482768734397	1081
SFRS6	-0.081718869	0.00718410186273137	0.0145482768734397	1081
LOC40092	-0.081726816	0.0071784597728388	0.0145424449038453	1081
RPS12	-0.081745586	0.00716515062243285	0.0145169425011475	1081
CRB2	-0.081757998	0.00715636216655247	0.0145005950357272	1081
MOAP1	-0.081761998	0.00715353182534817	0.0144963181391851	1081
ATP11A	-0.081763323	0.00715259438457202	0.014495876647606	1081
MYC	-0.081768615	0.00714885199563368	0.0144897498191006	1081
RBM39	-0.081788551	0.00713476922609318	0.0144626611048146	1081
ZNF20	-0.081810195	0.00711950807700259	0.0144346306573286	1081
DCUN1D1	-0.081821791	0.00711134364066972	0.0144195287006836	1081
FAM45B	-0.081829351	0.00710602540084842	0.0144114620348823	1081
GSTM5	-0.081852849	0.00708951823328768	0.0143825120654159	1081
PCDH12	-0.081874879	0.00707407343494141	0.0143526245691303	1081
PLXDC1	-0.08188466	0.00706722611938831	0.0143416208497106	1081
BIRC2	-0.081891054	0.00706275285993882	0.0143339871411916	1081
ACVR1C	-0.081898088	0.00705783524603715	0.0143254499779767	1081
MYO1D	-0.081913688	0.00704693881461061	0.0143047745284493	1081
FAM86B2	-0.081953983	0.0070188640062939	0.0142535297789104	1081

DCTN5	-0.081996171	0.0069895777631589	0.0141983507148166	1081
TMEM56	-0.082003708	0.00698435678489722	0.014189175820064	1081
ODF2L	-0.082004955	0.006983493424814	0.0141888527526856	1081
MRAP	-0.082031957	0.0069648214449065	0.0141551985479864	1081
LOC14457	-0.082037387	0.00696107190615348	0.0141490055039312	1081
PLEKHH1	-0.082046537	0.00695475784143326	0.0141375980540538	1081
CD24	-0.082051861	0.00695108642910983	0.014131560814857	1081
PLAG1	-0.082061373	0.00694453103079113	0.0141210838412524	1081
ZNF285	-0.082076292	0.00693426012434002	0.0141016222698193	1081
ZNF584	-0.082076407	0.00693418118837021	0.0141016222698193	1081
WBP4	-0.082080339	0.0069314763082275	0.0141002312662065	1081
ABR	-0.082106217	0.00691370040859979	0.0140654912877442	1081
ZNF23	-0.082121716	0.00690307293338955	0.0140452887832676	1081
FAM102B	-0.082175562	0.00686626640478828	0.0139732232270391	1081
FAM22D	-0.08221575	0.00683891080930101	0.0139203655909234	1081
NPAS3	-0.082278642	0.00679629756855851	0.0138406201494755	1081
TRIM29	-0.082295384	0.00678499388643708	0.0138203945239914	1081
CCDC67	-0.082427783	0.00669619505696747	0.0136477994154805	1081
C4orf23	-0.082435401	0.00669111739338809	0.0136388303035316	1081
MTNR1A	-0.08246367	0.00667230586477561	0.0136032387051821	1081
SIM1	-0.082472623	0.00666635826320647	0.013593864481455	1081
CORIN	-0.082479927	0.00666150976084381	0.0135853527163594	1081
AP3M1	-0.082493347	0.0066526091032252	0.0135713225705794	1081
TLN1	-0.082516235	0.00663745335483502	0.0135431477598654	1081
CTNNA3	-0.0825421	0.00662036422080503	0.0135123847242267	1081
PPEF1	-0.082604724	0.00657915085683875	0.0134339072604771	1081
OR2A2	-0.082610386	0.00657543599763171	0.0134302096899818	1081
ACTN1	-0.082613024	0.00657370589526958	0.0134280374427303	1081
FAM82A1	-0.082624638	0.00656609394577549	0.0134138487514093	1081
PLK2	-0.082640194	0.00655591048840333	0.0133944033254447	1081
PRR16	-0.082665745	0.00653921451849742	0.0133630022798875	1081
ST6GALN	-0.082668995	0.00653709392720925	0.0133600240604271	1081
ADRA1A	-0.082697478	0.00651853278429238	0.0133274985222338	1081

LOC38764	-0.082700803	0.00651636886183373	0.0133244265856314	1081
LASS6	-0.082776377	0.00646736441596351	0.0132282522245492	1081
METTL8	-0.082805455	0.00644859742177572	0.0131965659347493	1081
MTHFD2I	-0.082832041	0.00643148196198874	0.0131642150095776	1081
ANKRD29	-0.082840839	0.0064258270751249	0.0131553136614219	1081
GJA1	-0.082876578	0.00640290163535774	0.0131123770910116	1081
ZNF675	-0.082880516	0.00640037952866506	0.0131085446934687	1081
PNMA6A	-0.082886027	0.0063968524369066	0.0131026530087934	1081
P2RY2	-0.082889099	0.00639488684508703	0.0130999588666136	1081
TRMT2B	-0.082906085	0.0063840284893613	0.0130803757036395	1081
SPATA5	-0.082912086	0.00638019653458846	0.0130738540524143	1081
FAM7A2	-0.082928954	0.0063694359306348	0.0130531319249886	1081
TSPAN8	-0.0829623	0.00634821178875453	0.0130149324734846	1081
CCDC25	-0.083005922	0.00632054347392996	0.0129608457127768	1081
RRP15	-0.083037789	0.00630039927133497	0.0129248007455237	1081
RSPH4A	-0.083039956	0.00629903135085236	0.0129233105777493	1081
VTA1	-0.083066442	0.00628233642378378	0.0128916845530329	1081
MFSD8	-0.083073274	0.00627803596566812	0.012884172221718	1081
C9orf44	-0.083090958	0.00626691804480522	0.0128626657170251	1081
MGAM	-0.083102187	0.00625986708830394	0.0128508124420547	1081
PIH1D2	-0.083112277	0.00625353813239586	0.0128391281774475	1081
DCC	-0.083115881	0.00625127853891898	0.0128357971834189	1081
LOC10012	-0.083154025	0.00622741082553655	0.0127907005587718	1081
LOC64258	-0.083191159	0.00620425404391923	0.0127470369928356	1081
VEZF1	-0.083203471	0.0061965935801086	0.0127325966616981	1081
ZNF330	-0.083252176	0.00616637180109541	0.0126795508760914	1081
FAM18B	-0.083285686	0.0061456559441307	0.0126421156943238	1081
ATN1	-0.083301096	0.00613615039152512	0.012623851065898	1081
AGXT2	-0.083304263	0.00613419877912473	0.0126211249520445	1081
PNMT	-0.083316516	0.00612665213582698	0.0126081731817586	1081
GLT8D1	-0.083373727	0.00609152700563628	0.0125397314049196	1081
MAP1D	-0.083404591	0.00607265288211832	0.0125034333923011	1081
SIGLEC6	-0.083417821	0.00606457815274132	0.0124893607531153	1081

TAGLN	-0.083420973	0.00606265628527451	0.0124866793647741	1081
LRRC18	-0.083463721	0.00603664007055224	0.0124369108428385	1081
VWDE	-0.083473097	0.00603094751169202	0.0124264536793531	1081
AK7	-0.08348265	0.00602515265149653	0.0124157835683713	1081
UPP2	-0.083483287	0.00602476636856949	0.0124157835683713	1081
KALRN	-0.08349786	0.00601593561622441	0.0124005958240909	1081
GOLGA8A	-0.083534967	0.00599350408465353	0.0123556221638708	1081
FAM47A	-0.083535998	0.00599288189153294	0.0123556038997985	1081
SUMO4	-0.083544338	0.00598785079239928	0.0123464948017281	1081
ZNF418	-0.083595115	0.0059573028945185	0.0122872803122837	1081
SNORA36	-0.083630519	0.00593608678344536	0.0122497918717995	1081
LOC16263	-0.083648166	0.00592553703468732	0.0122305270044852	1081
IGBP1	-0.083660178	0.00591836599666907	0.0122182724296766	1081
TMC3	-0.083682658	0.00590496570660488	0.012194314111692	1081
PLXDC2	-0.083694281	0.00589804805533282	0.0121812772272585	1081
ARMCX1	-0.083735385	0.00587364305602532	0.0121370950214001	1081
ODAM	-0.083738305	0.00587191262157994	0.0121360090039733	1081
KCNJ12	-0.083772519	0.00585167353763456	0.0120954200098141	1081
CXADRP3	-0.083780223	0.00584712488517595	0.0120884986465383	1081
B4GALNT	-0.08379218	0.00584007154439838	0.0120763951202941	1081
RETSAT	-0.083798629	0.00583627018139822	0.0120697734117921	1081
ATP2C1	-0.08387021	0.00579422889991643	0.0119865209682498	1081
FNDC5	-0.083894188	0.00578020732745918	0.0119611994464987	1081
PPP4R1L	-0.08392205	0.00576395241504453	0.0119312393548163	1081
LOC28490	-0.083922512	0.00576368350802562	0.0119312393548163	1081
ASAH2	-0.083942369	0.00575212455162814	0.0119104274943518	1081
ZNF876P	-0.083963103	0.00574007715004159	0.0118867037610596	1081
SYNGAP1	-0.084005242	0.00571566334742993	0.011842233686514	1081
NPHP1	-0.084054869	0.00568703044949173	0.011785333649317	1081
GNA12	-0.08406216	0.0056828352475026	0.0117783953930701	1081
AHCYL1	-0.084082617	0.0056710778706438	0.0117571126586518	1081
ADO	-0.084097237	0.00566268842520248	0.011740928193259	1081
NUDT4	-0.084122007	0.00564850072861526	0.0117163351707119	1081

HIC2	-0.084135335	0.00564087987020839	0.011701831341397	1081
PCDHGB5	-0.084152415	0.00563112717081851	0.0116839073906828	1081
RSRC1	-0.084179706	0.00561557548727983	0.0116528397395192	1081
ABCB5	-0.084238983	0.00558193047509982	0.0115854099959701	1081
PURA	-0.084316237	0.00553835444136515	0.0115020773424022	1081
POF1B	-0.084379549	0.00550287086764833	0.0114366381542119	1081
ERBB2	-0.084397538	0.00549282641411502	0.0114169405811336	1081
PDLIM1	-0.084427893	0.00547591464904464	0.01138296363688	1081
HSDL2	-0.084441521	0.00546833721387633	0.0113695585894869	1081
C22orf39	-0.084442284	0.00546791315785045	0.0113695585894869	1081
PRG4	-0.08445106	0.00546303892430205	0.0113608876979524	1081
SNX1	-0.08445888	0.00545869920691437	0.0113530348465094	1081
BBOX1	-0.084472837	0.00545096118172467	0.0113381118230091	1081
SRRM1	-0.084473837	0.00545040712790695	0.0113381118230091	1081
ARMC10	-0.084484879	0.00544429267571479	0.0113277500467129	1081
FAM5C	-0.084521179	0.00542423525526804	0.011288349092704	1081
INMT	-0.084525681	0.00542175242983145	0.0112843478354122	1081
SLC6A4	-0.084567308	0.00539884310398572	0.0112401503027896	1081
WDR43	-0.084591764	0.00538542467279102	0.0112145317379962	1081
FGD6	-0.084615871	0.00537222722243859	0.0111893628407801	1081
TP53	-0.084618657	0.00537070368806243	0.0111885031846968	1081
KIAA0146	-0.084620772	0.00536954757510391	0.0111872516186233	1081
TIPRL	-0.084626392	0.005366476207046	0.0111820090185087	1081
C9orf4	-0.084642342	0.00535776858178405	0.0111650199731085	1081
CASP6	-0.084663365	0.00534631128492815	0.0111434496466505	1081
CDC42EP2	-0.084669379	0.00534303750012451	0.0111377783981797	1081
HADH	-0.084670273	0.00534255115662033	0.0111377783981797	1081
TMEM128	-0.084689309	0.00533220238768911	0.0111197947308486	1081
ZNF460	-0.084693455	0.00532995051153216	0.0111174003991318	1081
SLC7A14	-0.08472737	0.00531156422663667	0.0110825535649846	1081
FIGNL1	-0.084744296	0.00530240967981707	0.0110656835180685	1081
NME5	-0.084752178	0.00529815153488501	0.0110579426720119	1081
ZNF618	-0.084752952	0.00529773374235296	0.0110579426720119	1081

CTF1	-0.084753358	0.00529751459666915	0.0110579426720119	1081
DKK4	-0.08476701	0.00529014718514575	0.0110446694004313	1081
NAP1L1	-0.08478443	0.00528075993307058	0.0110273565721682	1081
ERI1	-0.084792409	0.00527646489149554	0.011019529881731	1081
RPL7A	-0.084807545	0.00526832644597554	0.011003674054347	1081
ANTXR2	-0.084847933	0.00524666630663275	0.0109607064966418	1081
ASB12	-0.08488053	0.00522924298038575	0.0109265739902366	1081
CAND1	-0.084889179	0.00522462883915911	0.0109191978384542	1081
CXorf56	-0.084898018	0.00521991705892181	0.0109116145223596	1081
LOC10013	-0.084953503	0.00519042722104112	0.0108529562715163	1081
SLC16A6	-0.084988956	0.00517166298349758	0.0108197269501048	1081
TAT	-0.08499328	0.00516937875477291	0.0108160713559306	1081
RASSF10	-0.085058817	0.00513486661395469	0.0107460926592684	1081
FOS	-0.08506512	0.00513155851922264	0.0107402853371159	1081
SEMA4B	-0.085121758	0.00510191780598585	0.0106804670270794	1081
PPP3CA	-0.085126324	0.00509953512695289	0.0106765885608466	1081
DVL2	-0.085131027	0.00509708203719794	0.0106747809168679	1081
GCNT4	-0.085137064	0.00509393418237633	0.0106694073558966	1081
LOC22112	-0.085155841	0.00508415557074347	0.010652894281876	1081
UBLCP1	-0.085161891	0.00508100862331648	0.0106477601910653	1081
EGOT	-0.085196304	0.00506314109920756	0.0106136292865282	1081
CCDC126	-0.085196618	0.00506297844854751	0.0106136292865282	1081
CD109	-0.085269295	0.00502543107235684	0.0105411608655382	1081
HADHB	-0.085282872	0.00501844445510692	0.0105286985573973	1081
ZFAND5	-0.085303465	0.00500786461166295	0.0105086908960365	1081
OR11L1	-0.085377185	0.00497015493095168	0.0104328197440889	1081
ZBTB12	-0.08538514	0.00496610133144765	0.0104253971782006	1081
KIF5C	-0.085427429	0.00494460104381615	0.0103845900779479	1081
ARHGAP3	-0.085428126	0.00494424732601948	0.0103845900779479	1081
CFLP1	-0.085435702	0.00494040487238947	0.0103779412048265	1081
IPO9	-0.085452127	0.00493208304868934	0.0103615406211124	1081
WNT9B	-0.085479742	0.00491812090822537	0.0103375986744783	1081
CCDC80	-0.085510056	0.00490283503552109	0.0103077206960326	1081

RAB36	-0.085518844	0.00489841185786402	0.0103004704464166	1081
AZGP1	-0.085522273	0.00489668702731321	0.0102979183803345	1081
CRHR2	-0.085586458	0.0048644994593197	0.0102334316633245	1081
TP53AIP1	-0.085594408	0.00486052618251147	0.0102261409865999	1081
ZNF350	-0.085603549	0.00485596138486891	0.0102176041464575	1081
FUT8	-0.085604046	0.00485571297013985	0.0102176041464575	1081
LOC28392	-0.085630649	0.00484245020380859	0.0101934335794905	1081
LOC90246	-0.085667016	0.00482437246687325	0.0101582079588878	1081
STC2	-0.085672898	0.00482145411915932	0.0101534803711546	1081
DES	-0.085690931	0.00481251763269945	0.0101378403963955	1081
C14orf39	-0.085718466	0.00479890110052695	0.0101151220869426	1081
PGBD1	-0.08574692	0.00478486625539861	0.0100880304254322	1081
SNTB2	-0.085803668	0.00475698700862596	0.0100345029621749	1081
PCNXL2	-0.085844253	0.00473713864395698	0.0100010121549479	1081
SCARNA6	-0.085865844	0.00472660950436424	0.00998087510119682	1081
FLJ10038	-0.085911247	0.00470453786153281	0.0099363509352672	1081
LDHAL6B	-0.085916521	0.00470198004832595	0.00993198994165107	1081
AZIN1	-0.085918204	0.00470116436004683	0.00993130830884474	1081
DYNC1H1	-0.085921514	0.00469955973375768	0.00992895971017813	1081
C5orf30	-0.085929136	0.00469586741750191	0.0099224643720386	1081
PTH2R	-0.085949868	0.00468583667746808	0.00990516053175179	1081
CARKD	-0.085953876	0.00468389983319727	0.00990210537724409	1081
GABRR1	-0.085959889	0.00468099490916631	0.00989700277552007	1081
PARP16	-0.085965892	0.00467809721865485	0.00989191439800587	1081
OTUD1	-0.086005959	0.00465879541695638	0.00985316888972034	1081
SYT14L	-0.086029287	0.00464759059109969	0.00983286180049324	1081
HES2	-0.086043592	0.00464073177279255	0.00982115154563568	1081
DRD2	-0.086047398	0.00463890833096958	0.00981832405204688	1081
MRGPRX1	-0.08606105	0.00463237386707566	0.00980552396261839	1081
KCND2	-0.086084447	0.00462119357247337	0.00978391429505791	1081
STRN3	-0.086149858	0.0045900662819872	0.00972209917470636	1081
LOC38933	-0.086160788	0.00458488379400946	0.00971214343115884	1081
NKX3-1	-0.086227866	0.00455319268447447	0.00965313266983143	1081

SLITRK4	-0.086233702	0.0045504451878032	0.00964832315633044	1081
IGSF22	-0.086254893	0.00454048008922189	0.00962820751551316	1081
CALB1	-0.086285446	0.00452614740812551	0.00960388029668089	1081
NLRX1	-0.086297915	0.00452030986225826	0.00959250417941563	1081
ARL5B	-0.086301304	0.00451872467886567	0.00959116095835517	1081
CDK6	-0.086303873	0.00451752299462132	0.009589620729889	1081
CYP2U1	-0.086310775	0.00451429639715161	0.00958479141237554	1081
ELP3	-0.086320093	0.00450994373554466	0.00957657212032887	1081
BCORL1	-0.086326292	0.00450705037443586	0.00957243329393889	1081
GRIK4	-0.086352427	0.00449486959431902	0.00954857618660304	1081
MEGF11	-0.086384342	0.00448003517678135	0.00951907062928596	1081
LAMB2	-0.086386153	0.00447919447126017	0.00951828825142787	1081
SLC4A4	-0.086410132	0.00446808000575679	0.00949667352985551	1081
RBBP4	-0.086417075	0.00446486645076381	0.00949084463972111	1081
C4orf49	-0.086440691	0.00445395158227535	0.0094686423200651	1081
ZNF300	-0.086457467	0.00444621235864765	0.00945318712031208	1081
NRXN3	-0.086466655	0.0044419792815871	0.00944618100153828	1081
CDR1	-0.086469227	0.00444079493512757	0.00944465941386665	1081
ADCY2	-0.086489188	0.0044316128221693	0.00942612610100312	1081
ITIH2	-0.086509556	0.00442226118980107	0.00940822174131825	1081
UBE2K	-0.086518561	0.00441813238077414	0.00940043058837084	1081
PZP	-0.086526402	0.00441453985507557	0.00939576390707842	1081
SCN11A	-0.086553882	0.00440197103847076	0.00937000280748029	1081
S1PR1	-0.086554608	0.00440163953791734	0.00937000280748029	1081
TMEM132	-0.086573037	0.00439322900414875	0.00935337119938455	1081
CHURC1	-0.086581267	0.00438947803444803	0.00934637300538586	1081
COX10	-0.086585811	0.00438740801306497	0.00934394063042539	1081
MIMT1	-0.08659862	0.00438157816434512	0.00933251132593916	1081
NME7	-0.086635742	0.00436472159348254	0.00929955753577005	1081
CABLES1	-0.086691016	0.00433973143752688	0.00925022641085378	1081
DOK5	-0.086699995	0.00433568383676932	0.00924257681393841	1081
LRP1B	-0.086712507	0.00433004995209521	0.00923254209025467	1081
NBEAL2	-0.086717333	0.00432787858549768	0.00922984481313263	1081

IQCA1	-0.086786017	0.00429708236896477	0.00916902068878247	1081
WDR93	-0.086801583	0.00429013051554729	0.00915515669869704	1081
DHX8	-0.086813663	0.00428474272620129	0.00914462784397977	1081
MRGPRX1	-0.086827808	0.00427844154723608	0.00913214716773372	1081
GRIK2	-0.086857563	0.00426521389550294	0.00910873888740662	1081
STXBP1	-0.086876755	0.00425670180513679	0.0090924883749847	1081
C9orf117	-0.086884914	0.00425308771528716	0.00908573192199997	1081
C2orf73	-0.08688966	0.00425098707333401	0.00908220750793441	1081
FBN2	-0.086897243	0.00424763219762552	0.00907600239936	1081
CES4	-0.086897872	0.00424735367506362	0.00907600239936	1081
TSPAN14	-0.086912827	0.00424074476544822	0.00906320849776729	1081
SLC2A12	-0.086923464	0.00423604970584829	0.00905413488852136	1081
C6orf89	-0.086965499	0.00421754147077794	0.00901840281592417	1081
LOC10012	-0.086990836	0.0042064213304946	0.00899557937397174	1081
COX6B2	-0.087028676	0.00418986273794344	0.00896302270955407	1081
NARS	-0.087048708	0.00418112038311413	0.00894622093657293	1081
ZNF107	-0.087065298	0.00417389317487282	0.00893170575821255	1081
MITF	-0.087109033	0.0041548944133589	0.00889482975102179	1081
SLC35E3	-0.087110502	0.00415425766206862	0.00889441179746756	1081
INPP5J	-0.087135397	0.00414347938018323	0.0088741645879004	1081
TMEM98	-0.08716474	0.00413080819019276	0.00884984908980466	1081
STON1-G	-0.087173966	0.00412683129013061	0.00884320990742274	1081
POLDIP3	-0.087195827	0.00411742237064945	0.00882398655922693	1081
COLEC10	-0.08725613	0.00409156791862274	0.00877324488777594	1081
EIF4G2	-0.087279025	0.00408179039032766	0.00875414323572349	1081
C1orf161	-0.087304215	0.00407105736884852	0.00873298378186067	1081
RNF183	-0.087308705	0.00406914677138713	0.00872981487855099	1081
GPR64	-0.087345857	0.00405337016403754	0.00870152834127625	1081
FBXO36	-0.087348056	0.0040524381278904	0.0087004546612333	1081
TMEM33	-0.087352902	0.00405038483394817	0.00869790027501183	1081
TSNAX-D	-0.087488464	0.00399332621252552	0.00858177475478201	1081
VCPIP1	-0.087496076	0.00399014392031651	0.00857676582103886	1081
FNBP4	-0.087514773	0.00398233741817222	0.00856089929453413	1081

PLXNC1	-0.087523576	0.00397866657251981	0.00855483382467837	1081
ZNF583	-0.087605054	0.00394483682039663	0.00848571679091193	1081
KLHL21	-0.087656007	0.00392381404592835	0.00844410148010112	1081
EHD2	-0.087682463	0.00391293819057193	0.00842159613772131	1081
CNOT8	-0.087734453	0.00389164583399557	0.00837755987666604	1081
GHRH	-0.087738053	0.00389017542325365	0.00837528950533769	1081
WDR17	-0.087766893	0.0038784134395412	0.00835264472306579	1081
FZD10	-0.087825299	0.00385469231711279	0.008303333696347	1081
PCDHB8	-0.087832753	0.00385167462885526	0.00829772060723871	1081
ZNF362	-0.087883495	0.00383118739161378	0.00825535030527968	1081
NXNL2	-0.087889161	0.00382890587746229	0.00825131674170708	1081
OR5K2	-0.087893789	0.00382704311911604	0.00824818483305794	1081
ZSCAN29	-0.087930418	0.00381233031972593	0.00821911325886975	1081
LOC10012	-0.087945862	0.0038061423050688	0.00820665061924552	1081
C10orf57	-0.087958724	0.00380099548968633	0.00819730801196136	1081
ZNF442	-0.087964703	0.00379860540835918	0.00819303061578113	1081
TMED7	-0.08798189	0.0037917419213048	0.00817910279523345	1081
ANKRD34	-0.08798379	0.00379098396156403	0.00817834353241672	1081
APLNR	-0.087991175	0.00378803901861789	0.00817347537923477	1081
IL17RA	-0.08800219	0.00378364982339227	0.00816514469118771	1081
KIAA1967	-0.08803497	0.00377061608998201	0.00813804653350213	1081
MTMR8	-0.088041421	0.00376805608242463	0.00813410734895459	1081
CYP4F22	-0.088054927	0.00376270108699391	0.00812428868140325	1081
C8orf22	-0.088062688	0.00375962716381588	0.00811852172956061	1081
KIFAP3	-0.08812779	0.00373393060337504	0.00806476889863224	1081
ANKRD26	-0.088163189	0.00372002511977189	0.00803645104963054	1081
RCAN2	-0.088166752	0.00371862834780359	0.00803429515942764	1081
LIN52	-0.088201039	0.00370520922960119	0.00800787897546572	1081
KCNA1	-0.088261483	0.00368165974188686	0.00795783642707198	1081
XBP1	-0.088263494	0.00368087865888381	0.00795700188681344	1081
OR2T33	-0.088285759	0.00367224011915568	0.00794003189872182	1081
SLC19A2	-0.088292841	0.00366949622428765	0.00793580262353996	1081
OMA1	-0.088321623	0.00365836387848417	0.0079142762384089	1081

KRT14	-0.088362316	0.00364267701808714	0.00788203313956664	1081
CCT8L2	-0.088421297	0.00362004853219041	0.00783559446448649	1081
BMP4	-0.088498644	0.00359056731861619	0.00777428833120412	1081
PLRG1	-0.088498672	0.00359055672787129	0.00777428833120412	1081
FAM120A	-0.088503434	0.00358874908609676	0.00777202218226395	1081
SHOC2	-0.088507549	0.0035871872991551	0.00776947512541442	1081
RAI2	-0.088551959	0.00357037328901755	0.00773555279708093	1081
ST5	-0.088568609	0.00356408806949112	0.00772359661788926	1081
LRRCC1	-0.088586526	0.0035573356339896	0.00771062258949006	1081
ITGA1	-0.088636872	0.00353842377213658	0.00767128141301027	1081
ADARB1	-0.08865922	0.0035300586371487	0.00765396956682386	1081
ARHGAP2	-0.08865925	0.00353004722831625	0.00765396956682386	1081
LOC37444	-0.088662622	0.00352878654066545	0.00765285874278237	1081
TACR1	-0.088672635	0.00352504545572527	0.00764556855141964	1081
ZNF100	-0.088677465	0.00352324223675159	0.00764248033373111	1081
CCDC148	-0.08867954	0.00352246772035575	0.00764162311291909	1081
CMTM5	-0.088720742	0.0035071219445481	0.00760997108713072	1081
C6orf124	-0.08874148	0.00349942132506435	0.00759489793077153	1081
UBXN4	-0.088746506	0.00349755724652486	0.00759167015744461	1081
FAM188B	-0.088785765	0.00348302759339359	0.00756257715767556	1081
GABRB3	-0.088796549	0.00347904620738682	0.00755556121688309	1081
CATSPER	-0.088799763	0.00347786043035952	0.00755380036329838	1081
CD36	-0.088804745	0.00347602310778168	0.00755062384152059	1081
MYO3B	-0.088817229	0.00347142267956852	0.0075422573209564	1081
MYCL1	-0.088849715	0.00345947757128272	0.00752035995181723	1081
PHKB	-0.088864941	0.00345389188510258	0.00750902784647005	1081
CSNK2A1	-0.088876338	0.0034497164989074	0.0075015693944829	1081
SCGB3A1	-0.088879478	0.00344856681545724	0.00749987892663134	1081
BNC1	-0.088883875	0.00344695760714369	0.00749718861972681	1081
TANK	-0.088889106	0.00344504373175286	0.00749383500444446	1081
MREG	-0.088912762	0.00343640207519821	0.00747826723613135	1081
COL24A1	-0.088913922	0.0034359786455235	0.00747815360999037	1081
EFHB	-0.088972093	0.00341481430991532	0.00743450062393213	1081

ANGPT4	-0.08898714	0.00340935889916607	0.00742342574834636	1081
ACTG2	-0.088987308	0.00340929829975575	0.00742342574834636	1081
ATL3	-0.08903211	0.00339310262322004	0.007391225383301	1081
PRX	-0.089038298	0.00339087115059853	0.00738716333176245	1081
ATP13A5	-0.089048098	0.00338733975716142	0.00738026816007104	1081
OSR1	-0.0890549	0.00338489085570135	0.00737652815751878	1081
LOC33952	-0.089055661	0.003384616982381	0.00737652815751878	1081
SSFA2	-0.089062224	0.00338225579340129	0.00737238075720287	1081
NRIP3	-0.089068752	0.00337990849183411	0.00736806152667441	1081
SNORD11	-0.089088679	0.00337275284779895	0.00735405413128151	1081
KLHDC2	-0.08909108	0.00337189163208783	0.00735297217237598	1081
MYO10	-0.089096012	0.00337012324102652	0.00734991151786069	1081
SCN8A	-0.089099157	0.00336899588307363	0.00734824838290583	1081
SCOC	-0.089117249	0.00336251766079944	0.00733570698254329	1081
EPHX2	-0.089147662	0.00335165347797038	0.00731438189943812	1081
IL1RL1	-0.089171713	0.00334308426821764	0.00729647156914889	1081
ADH1C	-0.089180184	0.00334007073972304	0.00729068426175324	1081
ZNF37A	-0.089183772	0.00333879521205243	0.00728947973846279	1081
ACBD3	-0.089202304	0.00333221383737799	0.00727747698980699	1081
PPP2R5C	-0.08920264	0.00333209458825548	0.00727747698980699	1081
TACC2	-0.089202783	0.0033320437573615	0.00727747698980699	1081
ZNF597	-0.089215399	0.00332757030779766	0.00726970001633054	1081
ZNF99	-0.089215794	0.00332743027237602	0.00726970001633054	1081
CCBL2	-0.08930274	0.00329674955057947	0.00720549199266827	1081
ANKRD13	-0.089304716	0.00329605539837995	0.00720475651045618	1081
C11orf46	-0.089311179	0.00329378563350611	0.00720057640661754	1081
ZNF195	-0.089311553	0.00329365441325398	0.00720057640661754	1081
TEP1	-0.089335449	0.00328527436189916	0.00718352892873749	1081
BTG1	-0.089338043	0.00328436599407769	0.00718294670407585	1081
OBP2B	-0.089362589	0.00327578054047146	0.00716510303884882	1081
WTIP	-0.089368391	0.00327375415310253	0.00716222604411929	1081
MTMR15	-0.089374662	0.00327156519119166	0.00715899204611731	1081
DIRAS3	-0.089375309	0.00327133968884667	0.00715899204611731	1081

GABRB1	-0.089375537	0.00327125981780071	0.00715899204611731	1081
PIP4K2B	-0.089380346	0.00326958235398545	0.00715698538748634	1081
SH3GLB1	-0.089426136	0.00325364846298783	0.00712520364027502	1081
PCDHB14	-0.089434725	0.00325066755147791	0.00712022374954035	1081
CSNK1A1	-0.089505215	0.00322629717751991	0.00706838027636092	1081
IGSF9	-0.089507225	0.00322560480914065	0.00706763202959956	1081
C18orf34	-0.089507541	0.00322549574996578	0.00706763202959956	1081
RAP2A	-0.089512346	0.00322384106114933	0.0070653044143661	1081
AP3B1	-0.089517418	0.00322209528061608	0.00706224670090425	1081
CYP4F8	-0.089552078	0.00321018733708847	0.007036912285707	1081
FAM114A	-0.08958088	0.00320032275248588	0.00701681561262822	1081
PTPRS	-0.089600732	0.00319353933109847	0.00700346721369244	1081
KCNQ3	-0.0896034	0.00319262858535546	0.0070022322103414	1081
HERC3	-0.089617787	0.00318772215624271	0.00699299388408031	1081
KIAA0907	-0.089656027	0.00317471440980586	0.00696521692284242	1081
TAS2R13	-0.089667181	0.0031709293011407	0.00695918626990733	1081
ZC4H2	-0.089671229	0.00316955677098814	0.00695769000234892	1081
TAF9B	-0.089677365	0.00316747695978474	0.00695388223134956	1081
REEP6	-0.089731524	0.00314917474441469	0.00691445506552959	1081
INTS4L2	-0.08974117	0.00314592525155483	0.00690807327148157	1081
BBS5	-0.089768785	0.00313663901419834	0.00688843267644452	1081
TMEM135	-0.08977492	0.00313457949738015	0.00688466026763226	1081
CHMP1B	-0.089775085	0.00313452415248096	0.00688466026763226	1081
GP5	-0.089827516	0.00311697216211329	0.00684897515333467	1081
CLSTN2	-0.089833045	0.00311512670054593	0.00684566678111681	1081
ALPK1	-0.089841489	0.0031123098184934	0.0068402227027656	1081
BBS12	-0.089843695	0.00311157429538489	0.006839352338301	1081
TPTE	-0.089862615	0.00310527258160322	0.00682624576128295	1081
SFT2D3	-0.089876118	0.00310078214239835	0.00681860673055504	1081
SLC8A3	-0.089909487	0.00308971102111947	0.00679648706272676	1081
CDH23	-0.089914748	0.00308796870583495	0.00679339626286393	1081
LOC93432	-0.089931176	0.00308253428564318	0.00678218143122345	1081
KIAA0562	-0.089958646	0.00307346632730417	0.0067637075774025	1081

PEX3	-0.089968416	0.00307024692782833	0.00675736090474181	1081
LOC28444	-0.089972872	0.00306877997519605	0.00675487025790259	1081
CNR1	-0.089974993	0.00306808165735728	0.00675407113839606	1081
CAPN8	-0.089997125	0.00306080513445402	0.00673952556930552	1081
GALNT5	-0.090047722	0.00304422893460221	0.00670375949798442	1081
OSBPL6	-0.090153738	0.00300976204267632	0.00663148379631571	1081
NLN	-0.090154632	0.00300947290615248	0.00663148379631571	1081
LHFPL3	-0.090160639	0.00300753090563326	0.00662801773263123	1081
NAA35	-0.090205717	0.00299299314723774	0.00659814477468857	1081
MAPK14	-0.090230058	0.00298517001119528	0.0065816186796026	1081
GPR182	-0.090287451	0.00296679726917041	0.00654325935925529	1081
FGF2	-0.090297011	0.00296374691731389	0.00653796338691286	1081
CDK15	-0.090328237	0.00295380356968759	0.00651674218720365	1081
MKRN2	-0.090355474	0.00294515541165138	0.00649908596425864	1081
AGGF1	-0.09042519	0.00292312406180294	0.00645258976824679	1081
ZFP90	-0.090454706	0.00291384195015946	0.00643351009271837	1081
UBP1	-0.090458717	0.0029125825846081	0.0064314344148778	1081
ZNF137	-0.090460811	0.00291192541328576	0.00643111987948249	1081
AP1G1	-0.090503651	0.00289850893888231	0.00640386735838825	1081
KLF11	-0.09052098	0.00289309775239634	0.00639331441663277	1081
ADAM9	-0.090534473	0.00288889090394135	0.00638541883474856	1081
AAK1	-0.090537153	0.00288805615274614	0.00638427424526183	1081
C9orf64	-0.09054329	0.00288614513304867	0.00638145030241087	1081
D4S234E	-0.090552327	0.00288333310984996	0.00637593254642453	1081
ZNF569	-0.09057602	0.0028759725624727	0.00636035429476481	1081
NFYB	-0.090577127	0.00287562917587066	0.00636029312120273	1081
PTENP1	-0.090609521	0.00286559429846513	0.00634157938510161	1081
ARHGEF1	-0.090664083	0.00284876440156321	0.00630779939211813	1081
FAM48A	-0.090684755	0.00284241182072792	0.00629442520925183	1081
CTNNAL1	-0.090688808	0.00284116784707761	0.0062923621679176	1081
TSPYL3	-0.090690739	0.00284057532129697	0.00629174159950823	1081
HSD17B1[illegible]	-0.090701306	0.00283733486004013	0.00628594641543039	1081
P2RY12	-0.090725989	0.00282977869717126	0.00626989571651068	1081

RGN	-0.090730944	0.00282826411559644	0.00626722919466399	1081
OCA2	-0.090776336	0.00281442317527946	0.0062372447597365	1081
ZNF398	-0.090807947	0.00280482059408715	0.00621750316596237	1081
FGF16	-0.090810318	0.00280410181761445	0.00621710666033931	1081
POLR1E	-0.090868104	0.00278662951474727	0.0061797282666869	1081
PPP2R3A	-0.090877714	0.00278373357262077	0.00617398577787576	1081
FLJ37307	-0.090879661	0.00278314698834903	0.00617336446600871	1081
TMEM209	-0.090888804	0.00278039471356243	0.00616793871872207	1081
TPTE2P1	-0.090908831	0.00277437490659837	0.00615661847250762	1081
FAM200B	-0.090919836	0.00277107193585009	0.00615132166916806	1081
ST8SIA2	-0.090924957	0.0027695364873414	0.00614859075793394	1081
STAC	-0.090934995	0.00276652839502157	0.00614258949825961	1081
RIN2	-0.090960504	0.00275889775886131	0.00612767313696373	1081
PCSK6	-0.090985393	0.00275147085961216	0.00611252541540439	1081
DDX59	-0.090994509	0.00274875515551532	0.00610783946700376	1081
MLL4	-0.091004088	0.00274590451600477	0.00610217832045406	1081
BAHD1	-0.091012883	0.0027432893085383	0.00609703917922596	1081
LOC14582	-0.091083585	0.00272234851188865	0.00605183301390386	1081
ARL5A	-0.091093208	0.00271950961075601	0.00604685663451212	1081
TMEM84	-0.091101989	0.00271692142280393	0.00604176863477041	1081
CASP2	-0.091108019	0.00271514548198633	0.0060391526702677	1081
CCDC30	-0.091137199	0.00270656626595767	0.00602140004723549	1081
INHBA	-0.091143704	0.00270465727462559	0.0060178176272733	1081
CAMKK1	-0.091184159	0.00269281158439061	0.00599278495001644	1081
RPS18	-0.091249985	0.00267363807566621	0.00595142972754651	1081
ADD1	-0.091284132	0.00266374111071485	0.00593071006579914	1081
RRAS2	-0.091295835	0.0026603568650587	0.0059238298935125	1081
RNASEL	-0.091318172	0.00265390841983535	0.00591208505115373	1081
HNRNPF	-0.091331673	0.00265001759709052	0.005904070392987	1081
BMP8A	-0.091371565	0.00263855206598101	0.00588177839650188	1081
LOC40094	-0.091375033	0.0026375575751249	0.00588021219022699	1081
BAZ2A	-0.091375474	0.00263743121772602	0.00588021219022699	1081
MMRN2	-0.091394624	0.00263194537846861	0.00586899929701684	1081

GDF7	-0.09140468	0.002629068799901	0.00586323380648795	1081
MMGT1	-0.091422929	0.00262385609712775	0.00585290455947719	1081
PRDM6	-0.091451451	0.00261572784587945	0.00583541943025931	1081
TNFSF11	-0.091456116	0.00261440044154718	0.00583375020989897	1081
THAP10	-0.091466555	0.00261143260789878	0.00582778949588934	1081
LOC64621	-0.091470145	0.00261041286708963	0.0058267886102516	1081
FNDC1	-0.091510703	0.00259891527297642	0.0058036967269826	1081
HNRNPUI	-0.091514324	0.00259789125100235	0.00580205313208896	1081
MYOZ3	-0.091603338	0.00257282849402125	0.00574926566966811	1081
ULK2	-0.091662909	0.00255617895586569	0.00571269414975752	1081
GLT25D2	-0.091678566	0.00255181939250923	0.00570484981268432	1081
GAPVD1	-0.091680374	0.00255131659250353	0.00570435879644657	1081
TMEM45I	-0.091683767	0.00255037271519488	0.00570288137946506	1081
GNB2L1	-0.091701168	0.00254553817460166	0.00569270276724583	1081
ATXN2	-0.091777504	0.00252442773473834	0.00564674624875681	1081
LOC28444	-0.091777807	0.00252434434305706	0.00564674624875681	1081
ACACA	-0.091780187	0.00252368857116871	0.00564634676434848	1081
NRSN1	-0.091827771	0.00251061397845772	0.00561808654565518	1081
ZNF680	-0.091837738	0.0025078832599274	0.00561285504624347	1081
TTLL7	-0.091841098	0.00250696340040552	0.00561203027090465	1081
MTM1	-0.091875833	0.00249747088364789	0.00559141486453509	1081
SLC19A3	-0.091890876	0.00249337024416968	0.00558285468142695	1081
WWC3	-0.091905475	0.00248939619323084	0.00557519581018736	1081
RTN4RL1	-0.091950474	0.00247718403554066	0.00554846257460159	1081
C3orf58	-0.091992697	0.00246577493797637	0.00552413657979694	1081
TAPT1	-0.092038918	0.00245334041981612	0.00549872527338626	1081
CEP135	-0.09205293	0.00244958239318711	0.00549152429454199	1081
RPL14	-0.092072996	0.00244420946378141	0.00548069898128635	1081
COL1A2	-0.092137498	0.00242701182666618	0.00544698677007466	1081
FAM155A	-0.092147231	0.00242442633811275	0.0054417903711734	1081
CASC2	-0.092218898	0.00240546635939427	0.0054016408215358	1081
HIVEP2	-0.092225427	0.00240374582615371	0.00539837900422145	1081
PPAP2A	-0.092225699	0.00240367415880703	0.00539837900422145	1081

NAP1L6	-0.092231743	0.00240208253116985	0.00539584663140239	1081
C16orf62	-0.092293246	0.00238594041424973	0.00536078180292893	1081
TMEM63(	-0.092302107	0.00238362303811947	0.00535617245989032	1081
LIPH	-0.092306582	0.00238245328823451	0.00535414117486436	1081
FRS2	-0.092307244	0.0023822804333048	0.00535414117486436	1081
UBE3C	-0.092307894	0.0023821105108753	0.00535414117486436	1081
POLR3A	-0.092352383	0.00237051296028847	0.00532909090336026	1081
PCDHGB8	-0.092362181	0.00236796570849345	0.00532455287393689	1081
PLIN1	-0.092364058	0.00236747804321583	0.00532405058948236	1081
VEGFC	-0.092393135	0.00235993485762369	0.00530827241032037	1081
CLDN1	-0.09242192	0.00235248890655118	0.00529270594398855	1081
FOXL1	-0.092431574	0.00234999653270349	0.00528768905967965	1081
PRODH	-0.092464608	0.00234148651260891	0.00527089571980183	1081
ACACB	-0.092470483	0.00233997584757025	0.0052680837560687	1081
DARS	-0.092490732	0.00233477650057723	0.00525755338745006	1081
CYS1	-0.09251011	0.00232981056766832	0.00524754403909642	1081
LRIT3	-0.092537269	0.00232286680264786	0.00523248929211015	1081
HEY1	-0.092554784	0.00231839882724335	0.00522359292862289	1081
DPH1	-0.092568059	0.00231501762113189	0.00521714173595503	1081
CSDA	-0.092604765	0.00230569206228723	0.00519845177333813	1081
ABHD2	-0.09261936	0.00230199348152412	0.00519069383090479	1081
MED14	-0.092633754	0.00229835139180646	0.00518364182578829	1081
DDHD2	-0.092658468	0.00229211001285606	0.00517014401623395	1081
GPM6A	-0.092696681	0.00228249022172685	0.00515075227026856	1081
ADAMTS	-0.092738954	0.00227189163385957	0.0051274094739078	1081
C8orf44	-0.092753999	0.00226813031007553	0.0051194941284562	1081
LOC9224(	-0.09276593	0.00226515154448048	0.00511393803678596	1081
SHC2	-0.092810182	0.00225413467897194	0.00509075595379929	1081
NXF5	-0.092810694	0.00225400774117409	0.00509075595379929	1081
RABL2B	-0.092818339	0.00225210948010339	0.00508732288368275	1081
NEBL	-0.092914266	0.00222841556284083	0.00503549428081082	1081
PCDH17	-0.092929169	0.00222475509894151	0.00502778679248113	1081
TTC8	-0.092933824	0.00222361273752746	0.00502576894395722	1081

GLP2R	-0.092937046	0.00222282256455117	0.00502454674179571	1081
ADAMTS:	-0.092941978	0.00222161325551617	0.00502237672902852	1081
PRUNE	-0.092998688	0.0022077524797066	0.00499160198694606	1081
PUS7	-0.093024528	0.00220146279216675	0.00497905781387665	1081
SPDYE6	-0.093028797	0.00220042547826743	0.0049772705209631	1081
OSBP	-0.093028873	0.002200406878515	0.0049772705209631	1081
SFRS5	-0.093058617	0.00219319079225426	0.00496406707702781	1081
ZKSCAN4	-0.093063962	0.0021918962887706	0.00496187782192197	1081
NCRNA0(	-0.093067508	0.00219103785777532	0.00496049198076913	1081
NSL1	-0.093099523	0.00218330171536733	0.00494353299495052	1081
GLS	-0.093128434	0.00217633719952973	0.00492831754547284	1081
PRR12	-0.093163447	0.0021679299491156	0.00491259266872146	1081
SCMH1	-0.093165994	0.00216731967902758	0.00491176228304765	1081
C16orf63	-0.093190767	0.00216139081884293	0.00489997952347409	1081
BNIPL	-0.09319361	0.00216071129858997	0.00489899033315644	1081
UST	-0.09319444	0.00216051303523346	0.00489899033315644	1081
LPPR4	-0.09322576	0.00215304108544238	0.00488324844249456	1081
AMOT	-0.093232344	0.00215147337982953	0.00488024222910325	1081
MEGF10	-0.093258052	0.00214536238554161	0.00486802492191211	1081
PLAC9	-0.093274954	0.0021413531826179	0.00485947503253776	1081
ANGPTL2	-0.09327739	0.00214077576907859	0.00485871201758542	1081
PPP1R3C	-0.093298285	0.00213583007453871	0.00484857976691259	1081
PARP11	-0.093309384	0.00213320744146245	0.00484371775341085	1081
ASPA	-0.093343221	0.0021252297442579	0.0048272356746054	1081
STAM	-0.093346368	0.00212448899204292	0.00482609728740467	1081
H19	-0.093348679	0.00212394538260517	0.00482540653350414	1081
FBLN5	-0.093371509	0.00211858125387036	0.00481376261664994	1081
RIMKLB	-0.093377535	0.00211716731782335	0.00481109256825171	1081
GCN1L1	-0.093398235	0.00211231736786814	0.0048006129710857	1081
NOTCH1	-0.093424076	0.00210627706777313	0.00478796564322837	1081
TUBGCP6	-0.093425841	0.00210586496138903	0.00478756908330685	1081
PTPRZ1	-0.093434805	0.00210377370882121	0.00478358585565325	1081
FBXL5	-0.09346145	0.00209756885281914	0.00477053548064085	1081

SH3YL1	-0.093469434	0.00209571281592048	0.00476717871236654	1081
SERHL	-0.09348958	0.00209103638695632	0.00475815294422627	1081
IDE	-0.093500917	0.00208840880171037	0.00475324769070787	1081
USH2A	-0.093520609	0.00208385219707134	0.00474341271299458	1081
CCDC109.	-0.093546125	0.00207796129568312	0.00473107259284995	1081
CCBE1	-0.093575715	0.00207114913095747	0.00471769552726574	1081
TMEM59I	-0.093585961	0.0020687949816291	0.00471286609803441	1081
APCDD1L	-0.093586514	0.00206866798657102	0.00471286609803441	1081
FRMD3	-0.093592125	0.00206738015199494	0.00471070842234341	1081
PRELP	-0.093596681	0.00206633467564025	0.00470885882814173	1081
VILL	-0.093721197	0.00203795272339774	0.00464680901107497	1081
C5orf43	-0.093723018	0.00203754052495173	0.00464639505039082	1081
HELB	-0.093766494	0.00202771696843292	0.00462608814598881	1081
PLEKHG1	-0.09377025	0.00202687028217584	0.00462468023948719	1081
ZMYM6	-0.093791254	0.00202214163505119	0.00461493635868428	1081
THBD	-0.093824711	0.00201463051268685	0.00459935762444204	1081
FLCN	-0.093866888	0.0020051980536137	0.00457834237674805	1081
AKAP2	-0.093881248	0.00200199575208265	0.00457154890338981	1081
PRELID2	-0.093887083	0.00200069596127489	0.00456909875749719	1081
SLC35F3	-0.093913116	0.00199490625798199	0.00455794335568189	1081
ZFYVE21	-0.093923831	0.00199252751729725	0.00455302482258995	1081
GLIPR1L2	-0.093931981	0.00199072016957885	0.00454941098311581	1081
PAM	-0.093935795	0.00198987474271973	0.00454799485955174	1081
NUS1	-0.093996575	0.00197644788464952	0.00451884492523716	1081
MYO1C	-0.094003753	0.00197486778384319	0.00451574477928728	1081
RBL2	-0.094029855	0.00196913138757947	0.00450313903993512	1081
ANKRD35	-0.094085806	0.00195688654009877	0.00447615298629383	1081
NUP214	-0.094116004	0.0019503067084231	0.00446211568894758	1081
FLJ10357	-0.094118625	0.00194973666757933	0.00446131816996655	1081
AGPAT4	-0.094128482	0.00194759371829757	0.00445742734095712	1081
ELOVL2	-0.094132657	0.0019466869697324	0.00445585831215307	1081
MMS19	-0.094139122	0.00194528326780676	0.00445315129885991	1081
AREG	-0.094157655	0.00194126475760474	0.00444445715898937	1081

GTF2H3	-0.094161779	0.00194037160043242	0.00444291724149932	1081
TRIM52	-0.094212321	0.0019294556799119	0.00441972627342237	1081
CKMT2	-0.094214003	0.00192909327495817	0.00441960468827845	1081
PUS7L	-0.094257221	0.00191980555094016	0.00439982739746183	1081
C1orf220	-0.094262149	0.00191874917162806	0.00439790670866392	1081
HEATR7E	-0.094264707	0.00191820095598816	0.00439715046180945	1081
FAM46D	-0.094271058	0.00191684047840781	0.0043945318581456	1081
PPP6C	-0.094282041	0.00191449005257157	0.00438964285329551	1081
TCF20	-0.094285656	0.00191371715195957	0.00438837017942237	1081
LARS2	-0.094290894	0.00191259734337146	0.00438630162593557	1081
SV2C	-0.094295791	0.00191155121308026	0.00438440159256539	1081
STAU2	-0.094351294	0.00189973019629889	0.0043582809252296	1081
FREM2	-0.09435461	0.00189902619787692	0.00435716204512878	1081
NUDT13	-0.094369302	0.0018959093114389	0.00435050610307969	1081
ADAMTS:	-0.09437836	0.00189399010630305	0.00434659725352871	1081
C6orf167	-0.094381608	0.00189330247585985	0.00434551422766284	1081
ESD	-0.09448906	0.00187067833651617	0.0042970142632974	1081
PHAX	-0.094524175	0.00186333862755064	0.00428064279302175	1081
TXNDC6	-0.094587245	0.00185022238829672	0.00425342120418093	1081
N4BP2L1	-0.094689754	0.00182908478328008	0.0042081901506598	1081
LOC1001:	-0.094699769	0.00182703158885187	0.00420394647143586	1081
BCL2L15	-0.094712488	0.00182442715741122	0.00419891295396424	1081
SNTN	-0.094716442	0.00182361808751213	0.00419753043566405	1081
CASP12	-0.094755214	0.00181570303756686	0.0041797894745497	1081
ZFHX3	-0.094827043	0.00180112257305949	0.00414717273223037	1081
SMEK1	-0.094834265	0.00179966250514719	0.00414475833613698	1081
PPP4R1	-0.094838014	0.00179890508600301	0.00414348764664197	1081
GALNTL1	-0.094843449	0.00179780751494004	0.00414143309187731	1081
ADAT1	-0.094887579	0.00178891798601678	0.00412284095965084	1081
C11orf92	-0.095005381	0.00176538557004199	0.00407140152358282	1081
EARS2	-0.095083616	0.00174991465381049	0.00403710841743154	1081
SLC30A1	-0.095207593	0.00172565271029804	0.00398432938096894	1081
APLF	-0.095214319	0.00172434537032403	0.00398176724956185	1081

GRIN1	-0.095232462	0.0017208234530583	0.0039750015436142	1081
AHCTF1	-0.095256214	0.00171622243662634	0.00396573766756568	1081
FITM2	-0.095312587	0.0017053477946347	0.00394196572838967	1081
UCHL5	-0.095346345	0.0016988661701353	0.00392788465481185	1081
HOXA10	-0.095384446	0.00169157765640395	0.00391193110874269	1081
AVPR2	-0.095389265	0.00169065798224654	0.00391025316330156	1081
KAL1	-0.095440723	0.00168086488170867	0.00388938927659328	1081
IL1RAPL1	-0.095490258	0.00167148706994767	0.00386857847243747	1081
ZNF155	-0.095492583	0.00167104819033217	0.00386800710033799	1081
RNASE4	-0.095547501	0.00166070986439299	0.00384451852656823	1081
G6PC2	-0.095568982	0.00165668208281493	0.00383563504865037	1081
ADH6	-0.095578415	0.00165491636917333	0.00383198738586169	1081
RAD1	-0.095611316	0.00164877064220225	0.0038199522239407	1081
GPC4	-0.09562414	0.0016463810588541	0.00381529350403955	1081
F2RL2	-0.095653327	0.00164095391550818	0.00380446732971714	1081
PRSS37	-0.095653798	0.00164086646089507	0.00380446732971714	1081
LIMK2	-0.095664738	0.00163883665192408	0.00380043333176133	1081
PTGR1	-0.095665484	0.00163869820388182	0.00380043333176133	1081
MTOR	-0.095701501	0.00163203243209539	0.00378552600397947	1081
PVRL4	-0.095720015	0.0016286156150668	0.00377803564780294	1081
LIX1L	-0.095746127	0.00162380767161315	0.00376731608253447	1081
GUF1	-0.095780992	0.00161740846452071	0.00375290181038582	1081
ITGB5	-0.095795294	0.00161479020668615	0.00374725823219588	1081
NAA25	-0.095804681	0.00161307384632913	0.00374370652468899	1081
RRN3	-0.095839112	0.00160679249299743	0.00373041774477737	1081
HMGB1	-0.095844091	0.00160588604860674	0.00372874302088322	1081
PXMP4	-0.095881558	0.00159907998339721	0.00371379597250828	1081
URB2	-0.09595643	0.00158555856732275	0.00368366709015302	1081
NAV1	-0.095958441	0.00158519685518855	0.00368325151646752	1081
ENOX2	-0.095985059	0.00158041608283236	0.00367341432890941	1081
TFB2M	-0.095985364	0.00158036150456444	0.00367341432890941	1081
INSIG2	-0.096007491	0.00157639762420003	0.00366618911793	1081
FLJ37201	-0.096010296	0.00157589581401167	0.00366544523418371	1081

OLFML2A	-0.096038851	0.00157079530822097	0.00365484771126258	1081
HMGCR	-0.09604272	0.00157010538395531	0.00365366442876052	1081
TMEM218	-0.096052196	0.00156841694737082	0.00365015704768776	1081
MYOC	-0.096055284	0.00156786699743713	0.00364929874793425	1081
PLCL1	-0.096056047	0.00156773123481879	0.00364929874793425	1081
KIAA1841	-0.096060791	0.00156688679997044	0.0036478602317583	1081
CECR7	-0.096067527	0.00156568858614929	0.00364549197503207	1081
TAOK3	-0.09610223	0.00155952913172497	0.00363241003105556	1081
SETX	-0.096104888	0.00155905822037487	0.00363173310007536	1081
PHF12	-0.096118868	0.0015565837939844	0.00362680783365901	1081
TCTN2	-0.096123332	0.00155579442987505	0.00362538794561399	1081
LOC10027	-0.096223989	0.00153809331793549	0.00358538415758047	1081
OPN3	-0.096233525	0.00153642597384312	0.00358191195961922	1081
IL1RAPL2	-0.09624783	0.00153392793778647	0.00357691611375257	1081
LLGL1	-0.096262347	0.00153139670947226	0.00357184053633423	1081
RNF182	-0.096345495	0.00151697250746885	0.00354065706904876	1081
UAP1	-0.096360835	0.00151432510184384	0.00353529715803038	1081
TFPI	-0.096400352	0.00150752480282317	0.00351982929449151	1081
ZNF193	-0.096404887	0.00150674619009621	0.00351841914912347	1081
AQP7P1	-0.096411701	0.00150557690149476	0.00351609629963041	1081
KLKP1	-0.096415134	0.00150498817864546	0.00351512891116669	1081
TSHB	-0.096425614	0.00150319221210979	0.00351134127005469	1081
FRMPD2	-0.096438528	0.00150098175034983	0.00350690978545415	1081
IMPAD1	-0.096438764	0.00150094149677591	0.00350690978545415	1081
NPR2	-0.096442523	0.00150029865232792	0.00350620839340439	1081
DACH2	-0.096464125	0.00149660934351542	0.00349839814633536	1081
ALX1	-0.096464539	0.00149653879078647	0.00349839814633536	1081
MKNK2	-0.096469713	0.00149565636996057	0.00349710798599648	1081
CHD8	-0.0966236	0.00146963136425892	0.00343852773902391	1081
KIAA1731	-0.096627393	0.00146899512387274	0.0034374383517733	1081
TTC12	-0.096709993	0.00145520395288645	0.00340595836306465	1081
ARFGEF1	-0.096734568	0.00145112382551997	0.00339719807845437	1081
RAB28	-0.096752305	0.00144818558962319	0.00339110760234328	1081

ISM1	-0.096763881	0.00144627090548759	0.00338724579593356	1081
C14orf106	-0.096882442	0.00142679448025097	0.00334451650042539	1081
NCRNA0(	-0.096892472	0.00142515801703012	0.00341458130013	1081
EIF2AK3	-0.096896646	0.00142447740514696	0.00334025111473466	1081
FLNA	-0.096945613	0.00141651614450448	0.00332235623833306	1081
EPHB4	-0.09695882	0.00141437581387675	0.00331772249307723	1081
LOC2850:	-0.096967528	0.00141296623971681	0.00331480201456798	1081
HELZ	-0.096983546	0.00141037686717272	0.00330949819326668	1081
PAPOLA	-0.096992225	0.00140897575912741	0.00330659560433617	1081
DLK2	-0.097056972	0.00139856299280918	0.00328483752945562	1081
SNAPC3	-0.097084817	0.00139410659696787	0.00327475249485981	1081
C10orf108	-0.097114184	0.00138942099291389	0.00326450733639495	1081
KIAA0317	-0.0971459	0.00138437676405648	0.00325303509995541	1081
IKZF2	-0.097147988	0.00138404532866432	0.00325263569131391	1081
CPEB2	-0.097162358	0.00138176594433647	0.00324803675013514	1081
TNFSF15	-0.097162541	0.00138173689553424	0.00324803675013514	1081
PLEKHG4	-0.097167744	0.00138091246271111	0.0032467882307791	1081
HCG2P7	-0.097181295	0.00137876737922913	0.00324212312998376	1081
USP4	-0.09718442	0.00137827321068965	0.00324133946174913	1081
ASCL4	-0.097251844	0.00136764920741388	0.00321748140644081	1081
IL13RA2	-0.097266713	0.00136531646360491	0.00321236862407392	1081
SLC39A14	-0.097296883	0.00136059449479186	0.00320200655345584	1081
TUSC5	-0.097298316	0.00136037064903939	0.00320185380592342	1081
MMP28	-0.097298513	0.00136033977330709	0.00320185380592342	1081
LOC6438:	-0.09736524	0.00134995143254286	0.00317844455453201	1081
GNL3	-0.097371768	0.00134893900999991	0.00317643206971925	1081
RBM44	-0.097389755	0.00134615330431321	0.0031702429641559	1081
RASD1	-0.097395218	0.00134530812169966	0.00316862295237222	1081
ZNF549	-0.097399811	0.00134459800619992	0.00316732072437995	1081
C16orf3	-0.097407515	0.00134340780907912	0.00316488718441104	1081
ZNF324	-0.097423339	0.00134096588439818	0.00315950382938026	1081
LOC10027	-0.097476485	0.00133279456534861	0.00314098578836543	1081
CEP63	-0.097533117	0.0013241379768031	0.00312204582662668	1081

C11orf41	-0.097548779	0.00132175299187327	0.00311751715505058	1081
PKD2L2	-0.097570101	0.00131851259870967	0.00311060268193071	1081
LMO7	-0.097572173	0.00131819811409543	0.003110224995133 8	1081
NUP155	-0.09760617	0.00131304761446925	0.00309952474730292	1081
GXYLT1	-0.097614795	0.00131174396704153	0.00309681029134555	1081
ZNF135	-0.097626485	0.00130997882866526	0.00309300556768187	1081
C13orf33	-0.09763153	0.00130921774580611	0.0030919333516136	1081
GSPT1	-0.097644626	0.00130724397821292	0.00308799600622647	1081
USP24	-0.09766735	0.00130382568169367	0.00308100508594944	1081
HIBADH	-0.097681202	0.00130174608524765	0.00307681272877086	1081
MAP7	-0.09769394	0.00129983634159268	0.00307343395675211	1081
LOC10012	-0.097701052	0.00129877121599306	0.00307194389411533	1081
TNS4	-0.097701461	0.00129870999476741	0.00307194389411533	1081
PRDM16	-0.097715659	0.00129658612528597	0.00306749588888854	1081
MXD4	-0.097757909	0.00129028488205986	0.00305366411526032	1081
PITPNM3	-0.097760243	0.00128993770623532	0.00305320116227358	1081
TMEM231	-0.097800346	0.0012839841777699	0.0030398238642819	1081
EIF3F	-0.097815048	0.00128180811638384	0.00303502873819375	1081
CYP4Z1	-0.097820726	0.00128096848607962	0.00303375383871079	1081
PAIP2B	-0.097841216	0.00127794307326614	0.00302765649899405	1081
CDK8	-0.09784529	0.00127734229040133	0.00302658908964183	1081
CYP4A22	-0.09784891	0.00127680868758836	0.00302568062715768	1081
LOC64285	-0.097891094	0.00127060583017067	0.0030120445338654	1081
DDIT4L	-0.097908353	0.00126807606099723	0.00300640134739192	1081
SCFD2	-0.097919696	0.00126641593176332	0.00300317233023802	1081
CSN2	-0.0979707	0.00125897595676956	0.00298658392005685	1081
FGFR1OP	-0.098011017	0.00125312349186121	0.00297410140710934	1081
ZBTB49	-0.098014845	0.00125256910857134	0.00297313593639326	1081
ADAM33	-0.098026443	0.00125089079600637	0.00296950213122182	1081
MEF2A	-0.098040761	0.00124882188790664	0.00296494012161217	1081
DYRK1A	-0.098060873	0.00124592087952981	0.00295874998445456	1081
GRM1	-0.098076895	0.00124361437973816	0.00295396906966457	1081
STEAP4	-0.098092511	0.00124137006097626	0.00294946101828912	1081

TRIP12	-0.098093682	0.00124120195056145	0.00294946101828912	1081
NEK3	-0.098099463	0.00124037225135628	0.00294800601741061	1081
CLDN11	-0.09812489	0.00123672862319013	0.00293969299282185	1081
ASAH1	-0.098130141	0.00123597742460032	0.00293825409707028	1081
REPS1	-0.098170142	0.00123026855760877	0.0029257183441186	1081
MIA3	-0.098188579	0.00122764539940199	0.00292016962698703	1081
USP48	-0.098203227	0.00122556508713351	0.00291556550310636	1081
C8orf34	-0.098211447	0.00122439918487443	0.00291313588984238	1081
GRAMD2	-0.098225191	0.00122245190009175	0.0029097164702744	1081
PCDHGA	-0.098244775	0.00121968215248473	0.00290397080271892	1081
DGCR8	-0.098303894	0.00121135593721182	0.00288593914092786	1081
LOC3890	-0.098307446	0.00121085733179586	0.0028853466937577	1081
IKZF4	-0.098373266	0.00120165274182363	0.00286409068670575	1081
DSG3	-0.098399061	0.0011980629681632	0.00285621047262103	1081
TAF8	-0.098450067	0.00119099398257438	0.00284055240790056	1081
KIF26B	-0.098469326	0.00118833478853139	0.00283469559679868	1081
TULP3	-0.098543248	0.0011781790667648	0.00281246798198565	1081
ATL2	-0.098547973	0.00117753267327238	0.00281125808284807	1081
ZNF516	-0.098554684	0.00117661511933769	0.00280940044791487	1081
ZSWIM5	-0.098567614	0.00117484917212908	0.00280551642633242	1081
SYNPO2	-0.098610595	0.0011689964559363	0.00279187120389291	1081
ELAVL2	-0.098611806	0.00116883198101187	0.00279180937366424	1081
RNF133	-0.098638779	0.00116517319910695	0.00278439075872	1081
NDST2	-0.098640916	0.00116488378094761	0.00278402939461261	1081
FAM190A	-0.098665315	0.0011615841674196	0.00277713186003654	1081
TGFB1I1	-0.098692727	0.00115788726223295	0.00276862182761813	1081
TNMD	-0.098710775	0.00115545925652337	0.00276314419784701	1081
PAXIP1	-0.098717974	0.00115449194911279	0.00276115876942623	1081
AIM1	-0.098770701	0.00114743070270545	0.00274492239710264	1081
FKBP7	-0.098798366	0.00114374185639718	0.0027367477963085	1081
ACSBG2	-0.098809144	0.00114230759686802	0.00273364059621124	1081
CA13	-0.098812982	0.00114179731717068	0.00273274408392579	1081
ATXN7L3	-0.098851504	0.00113668689626367	0.00272148294809027	1081

UBXN8	-0.098875436	0.00113352269118734	0.00271422971757624	1081
DHX29	-0.098886913	0.00113200810643457	0.00271124756915045	1081
TNNT3	-0.098897105	0.00113066461789486	0.00270835181064112	1081
PROKR1	-0.098906125	0.00112947695393468	0.00270582866417103	1081
STAG3L3	-0.098912752	0.00112860499936099	0.00270435149618309	1081
SGCB	-0.098940135	0.00112500896938547	0.00269608680564718	1081
NEXN	-0.09895455	0.00112312018625628	0.00269188056539354	1081
ISCA1P1	-0.098959114	0.00112252280781688	0.00269076891521551	1081
GALNT13	-0.098961641	0.00112219212646382	0.00269029636886988	1081
VASH1	-0.098966556	0.00112154936754458	0.00268907545931748	1081
GABPB2	-0.098979215	0.00111989513909998	0.00268542882718356	1081
IMMP2L	-0.098983372	0.0011193524657497	0.00268444707411044	1081
LRRTM1	-0.098987379	0.0011188295982666	0.00268351259162765	1081
KIAA1199	-0.098989414	0.00111856413421549	0.00268319534219707	1081
FLJ11235	-0.098993366	0.00111804875301573	0.00268227844819601	1081
CASC4	-0.098999663	0.0011172279481268	0.00268062851536618	1081
APLP2	-0.099006245	0.00111637065456168	0.00267889062967779	1081
COL5A2	-0.099012527	0.00111555302488207	0.00267724752040139	1081
SALL1	-0.099034756	0.00111266424142287	0.00267063280632237	1081
FAM178A	-0.099065485	0.00110868213255117	0.00266202640765714	1081
ZNF85	-0.099079084	0.00110692399600199	0.00265812181421625	1081
OCRL	-0.099081057	0.00110666925931563	0.00265782692285568	1081
TBKBP1	-0.099085701	0.00110606955921937	0.00265670338267248	1081
STXBP6	-0.099104558	0.00110363771233772	0.00265117835857899	1081
ZDHHC6	-0.099115058	0.0011022857780736	0.00264824651154356	1081
POU3F3	-0.099136901	0.00109947832272116	0.00264181665210136	1081
CTAGE6	-0.099138306	0.00109929786471693	0.00264169813726858	1081
LOC15062	-0.099143893	0.00109858099278628	0.00264029039604863	1081
EPGN	-0.099188797	0.00109283447148145	0.00262710625632384	1081
TNIK	-0.099209523	0.00109019136202333	0.0026213780124087	1081
PEX11B	-0.099245343	0.0010856375310921	0.0026113633509075	1081
UBXN10	-0.099308998	0.00107758808542059	0.00259354981849435	1081
ZNF630	-0.099351771	0.00107221032472401	0.00258122334985244	1081

SRL	-0.099355566	0.00107173446528513	0.00258038613638926	1081
RABL3	-0.099356298	0.00107164272343444	0.00258038613638926	1081
LRGUK	-0.09936146	0.00107099568023568	0.00257922390655681	1081
MGC8704	-0.099363024	0.00107079969427136	0.00257906023925113	1081
CNOT4	-0.099363782	0.00107070478891715	0.00257906023925113	1081
COL10A1	-0.09941853	0.00106386643600654	0.00256358724322389	1081
DMBT1	-0.099421533	0.00106349257826619	0.00256299294044412	1081
ZNF610	-0.099457997	0.00105896181561312	0.00255268466860433	1081
MAP4	-0.099479033	0.00105635629088616	0.00254731834808495	1081
MRGPRX4	-0.099479339	0.00105631838201376	0.00254731834808495	1081
BAMBI	-0.099498846	0.00105390758161513	0.00254232645571031	1081
C8orf37	-0.099513735	0.00105207090489513	0.00253850381831506	1081
CCDC36	-0.099519748	0.001051329963251	0.00253701989814225	1081
MAP3K2	-0.099526155	0.0010505410845411	0.00253541992908596	1081
BRPF1	-0.099534295	0.00104953955385889	0.00253330629193473	1081
COX18	-0.099565422	0.00104571779794848	0.0025246866059051	1081
ADAMTS	-0.099566229	0.0010456188869379	0.0025246866059051	1081
CCDC91	-0.099579425	0.00104400269143375	0.0025220571074389	1081
GPCPD1	-0.099579734	0.00104396490472657	0.0025220571074389	1081
TNRC6C	-0.09958713	0.00104306008819549	0.00252038448802903	1081
SYT17	-0.099597322	0.00104181450744835	0.00251767673375084	1081
C14orf104	-0.099612292	0.00103998746736786	0.00251386459444752	1081
PCDHB10	-0.099627298	0.00103815887280442	0.00251004686661607	1081
CD44	-0.09962764	0.00103811726487815	0.00251004686661607	1081
GPBP1L1	-0.099631311	0.00103767037748104	0.00250946815778579	1081
DDX21	-0.099653272	0.00103500100971442	0.00250331316372878	1081
C15orf38	-0.099666632	0.00103338011876039	0.002499692902549	1081
PUS10	-0.099685805	0.00103105804715207	0.0024943754485263	1081
EIF4E3	-0.099692201	0.00103028458714579	0.00249280362815901	1081
NCRNA00	-0.099727335	0.00102604493013987	0.00248284385797811	1081
MAPK10	-0.099752963	0.00102296261104235	0.00247568258042384	1081
LSM14A	-0.099803989	0.00101685109190931	0.00246148344706959	1081
SIK3	-0.09982889	0.00101388095840575	0.00245517873627645	1081

MASP1	-0.099877855	0.00100806384479554	0.0024413856880748	1081
PCDH20	-0.099882542	0.00100750852958273	0.0024403341743951	1081
TBX20	-0.099906858	0.00100463270464268	0.00243366111532309	1081
LRRC15	-0.099907382	0.00100457087782891	0.00243366111532309	1081
GAS5	-0.099951508	0.000999371672120455	0.00242208160910329	1081
FBXO48	-0.099976176	0.000996476111035844	0.00241564515725837	1081
PDE7B	-0.10001253	0.000992222858381852	0.0024059135149377	1081
SLC2A4	-0.100025667	0.000990689995689189	0.00240277509790016	1081
NR3C1	-0.10007245	0.000985249092015157	0.00239015449887347	1081
CYP3A7	-0.100083665	0.000983948871243548	0.00238728772867654	1081
MAGEE1	-0.100097485	0.000982348855104371	0.0023836928072847	1081
ZNF589	-0.10012476	0.000979198051260697	0.00237661984851166	1081
BTBD18	-0.100133389	0.000978203224060814	0.00237477753057425	1081
FABP4	-0.100163509	0.000974737907515484	0.0023672206325376	1081
ACPL2	-0.100174347	0.000973493829524745	0.00236448434962334	1081
TAF1B	-0.100183845	0.000972404744451663	0.0023621239089568	1081
TRDN	-0.100184721	0.000972304316254427	0.0023621239089568	1081
ADD2	-0.100229538	0.000967181034722552	0.00235000144065684	1081
CRISPLD2	-0.100262853	0.00096338884168475	0.00234163467853997	1081
QSOX1	-0.100286551	0.000960699606429509	0.00233537994105496	1081
DCUN1D2	-0.100296187	0.000959608170872244	0.00233328987353305	1081
KIF1B	-0.100310392	0.000958001208574746	0.00232966373089549	1081
CIDEA	-0.100313036	0.000957702431497234	0.00232921833665481	1081
MET	-0.100398519	0.000948087486043114	0.00230694798965316	1081
C6orf72	-0.10041164	0.000946619646178764	0.0023040870669518	1081
CCNB1IP1	-0.100418364	0.000945868197728123	0.0023029387047659	1081
ZFP92	-0.100429344	0.000944642425552022	0.00230023228124568	1081
DKK3	-0.100432263	0.00094431670393943	0.00229971711809234	1081
CSRNP2	-0.100450439	0.000942291394287732	0.00229506228242369	1081
IFFO2	-0.100488275	0.000938088184565197	0.0022851011460781	1081
XKR4	-0.100495743	0.000937260565754393	0.0022833962693698	1081
TNC	-0.100499933	0.000936796559815558	0.00228278295602811	1081
ITGB6	-0.100509988	0.000935683883753338	0.00228034742780196	1081

PAPPA	-0.100521364	0.000934426564070497	0.00227811002459157	1081
CCNC	-0.100531627	0.000933293509471471	0.00227562306321748	1081
GLYAT	-0.100544779	0.000931843426045974	0.00227263750819566	1081
CLIC4	-0.100564095	0.000929717511005317	0.00226772723806782	1081
KLHL14	-0.100568729	0.000929208128528622	0.0022667592333748	1081
KIF26A	-0.100632208	0.000922256690391071	0.00225198315490038	1081
PTH1R	-0.100644446	0.000920922119693291	0.00224926965339735	1081
HIAT1	-0.100645733	0.000920781791513581	0.00224919961077253	1081
C1orf192	-0.100658789	0.000919360116882402	0.00224599921835993	1081
CHSY3	-0.100672678	0.00091785003381202	0.00224258204137576	1081
IVNS1AB	-0.100676539	0.000917430653288319	0.00224182927104478	1081
RAB40A	-0.100684872	0.00091652613488493	0.00223989069358934	1081
CELSR1	-0.100733611	0.000911252036665143	0.00222781216973535	1081
ANKIB1	-0.100773335	0.000906974242004167	0.0022176230252669	1081
BARD1	-0.100790168	0.000905167106034252	0.00221374181753794	1081
PTER	-0.100797538	0.000904376910753792	0.00221207781291102	1081
TSR1	-0.100826927	0.000901232459989559	0.0022049220192482	1081
RASSF3	-0.100837936	0.000900057061950992	0.00220231379970882	1081
USP30	-0.100855008	0.000898237296411825	0.00219866225227414	1081
ETV4	-0.100886716	0.000894866399485467	0.00219120987208396	1081
TBC1D23	-0.100892153	0.000894289576697184	0.00219006364242217	1081
ZNF287	-0.100923999	0.000890917808842073	0.00218233697216969	1081
SNHG5	-0.100950076	0.000888165673159471	0.00217586008583212	1081
AQP7	-0.100973585	0.00088569123426284	0.00217032598397092	1081
ME3	-0.100992075	0.00088374957712043	0.0021660950518422	1081
MAK16	-0.101002318	0.000882675676459305	0.00216398947332028	1081
MYPN	-0.101007873	0.000882093717591389	0.00216282594217119	1081
FAM106A	-0.101064791	0.000876151793744424	0.00214904150553774	1081
UGDH	-0.10106628	0.000875996778745373	0.00214892293081168	1081
PKP1	-0.101070518	0.000875555957058154	0.00214836476917619	1081
C6orf201	-0.101098728	0.00087262654139661	0.00214195950730288	1081
CDH22	-0.10110443	0.000872035569716383	0.0021410306583713	1081
NF2	-0.10112629	0.000869773186178784	0.0021357363280804	1081

COL17A1	-0.101128593	0.000869535149110009	0.00213567246754708	1081
MGC2752	-0.101128989	0.000869494326706417	0.00213567246754708	1081
APH1B	-0.101131944	0.000869188990189078	0.00213534295211695	1081
GYS2	-0.101140933	0.000868260875650512	0.00213332300768137	1081
LNPEP	-0.1011966	0.000862533525140906	0.00212028527931221	1081
MRPL42P	-0.101200671	0.00086211600144039	0.00211951755540843	1081
GUSBP3	-0.101206245	0.000861544690214472	0.00211837151035891	1081
BCL7A	-0.101218195	0.000860321119356196	0.00211562120964729	1081
CD2AP	-0.101222347	0.000859896332442072	0.00211483477195038	1081
CD200	-0.101254978	0.000856564816585429	0.00210741307158201	1081
ZNF664	-0.101302309	0.00085175372608317	0.00209660053909402	1081
EGR3	-0.101307061	0.000851272140082831	0.00209567117951468	1081
DMRT2	-0.101346571	0.000847277258162578	0.00208685662864472	1081
S100PBP	-0.101366252	0.000845293780214603	0.00208248051882392	1081
TMEM167	-0.101366262	0.000845292775416913	0.00208248051882392	1081
HILS1	-0.10137208	0.000844707262292618	0.0020817993392323	1081
DLX1	-0.101397263	0.000842177227539199	0.0020758179675489	1081
PLIN4	-0.101405366	0.000841364663478294	0.00207406891162142	1081
WISP3	-0.101409547	0.000840945646983252	0.00207328969018206	1081
SETDB2	-0.101437862	0.000838113164699166	0.00206655932715602	1081
PEX19	-0.10147483	0.000834428451325623	0.00205848164976178	1081
HRH4	-0.10148913	0.000833007105606664	0.0020552269617203	1081
CMTM6	-0.1015051	0.000831422460536791	0.00205207124081275	1081
MOSC2	-0.101523217	0.000829628139600317	0.00204814446963828	1081
SALL4	-0.101543715	0.000827602344301914	0.00204414531951472	1081
AXIN2	-0.101562979	0.000825702724337836	0.0020399535665668	1081
ASTE1	-0.1015678	0.00082522786038845	0.00203903044861098	1081
NAF1	-0.101569849	0.000825026171221748	0.00203878216624903	1081
RBM20	-0.101622989	0.000819810805999492	0.00202663991739597	1081
TNFAIP1	-0.101638004	0.000818342679831053	0.00202325887152633	1081
ALG9	-0.101665956	0.000815616206130552	0.0020167654931263	1081
DKFZP434	-0.101702929	0.000812022672657619	0.00200837283495245	1081
MUDENG	-0.101736965	0.000808727583208154	0.00200071437599512	1081

ADAMTS	-0.101742185	0.000808223300715389	0.00199971240394393	1081
PI4KA	-0.101774896	0.000805070064896451	0.0019921553196584	1081
AUTS2	-0.101774911	0.000805068539063275	0.0019921553196584	1081
RBMS1	-0.101781317	0.000804452357060056	0.00199111595195661	1081
GCNT7	-0.101832621	0.000799533221723327	0.00197918367554883	1081
LOC10013	-0.101928974	0.000790369754679976	0.00195842542534171	1081
AQPEP	-0.101931565	0.000790124718604046	0.00195805910398309	1081
KBTBD12	-0.101975925	0.000785939949342982	0.001948407615003	1081
VGLL3	-0.101994504	0.000784193479017642	0.00194455657740158	1081
C1orf83	-0.101994678	0.000784177121132427	0.00194455657740158	1081
MICAL3	-0.102017237	0.000782061251327101	0.00193974684905004	1081
PHKG1	-0.102021264	0.000781684164852993	0.00193905030180563	1081
EIF2C1	-0.102053957	0.000778628634976751	0.00193194652686373	1081
FKBP9	-0.102077584	0.000776427292274312	0.00192695919710066	1081
FAM161A	-0.102085914	0.000775652550546303	0.00192527361147798	1081
PAN2	-0.10209405	0.000774896540235976	0.00192363411004975	1081
USP9X	-0.102098778	0.000774457587392432	0.00192278137762146	1081
OR13G1	-0.102128972	0.000771659385086319	0.00191677907676497	1081
NEK9	-0.102170861	0.000767792947023646	0.00190741015141094	1081
LPGAT1	-0.10217637	0.000767285738849967	0.00190640296776941	1081
OTUD7A	-0.102179729	0.000766976653611221	0.0019060874274609	1081
C10orf26	-0.102181333	0.000766829113818089	0.00190595589116168	1081
C16orf72	-0.102190302	0.000766004545810988	0.00190414135925004	1081
PEX13	-0.102206717	0.000764497647365764	0.00190086461443882	1081
PPWD1	-0.102248092	0.000760711340339789	0.00189191727791914	1081
ZRANB2	-0.102270631	0.000758656094208747	0.00188727179770748	1081
FLJ34503	-0.102312481	0.000754853663139647	0.00187874067753252	1081
CTGF	-0.102316173	0.000754519086381324	0.00187813999692966	1081
STXBP4	-0.102373405	0.000749349714478057	0.00186619483226115	1081
EPB41L5	-0.102388015	0.000748035365833487	0.00186338227336659	1081
HECW2	-0.102443536	0.000743060147208293	0.00185190482067439	1081
ANK2	-0.102470461	0.000740658368781986	0.00184683288019719	1081
ARL13A	-0.102472294	0.000740495185908332	0.0018466545580742	1081

TMEM200	-0.102487005	0.000739186241806414	0.00184361852682806	1081
C1orf64	-0.102500767	0.000737963688337867	0.00184102520143236	1081
MEF2C	-0.102539006	0.000734576673994002	0.0018328024644054	1081
ARHGAP2	-0.10254392	0.000734142460773901	0.00183194597699619	1081
GGT6	-0.102556867	0.000732999588250413	0.00182954735538404	1081
FEZ2	-0.102570173	0.000731826698439055	0.00182729906297159	1081
FAM21A	-0.102589464	0.000730129370100905	0.00182351303752575	1081
CAPN5	-0.102597335	0.000729437905099885	0.00182223792140838	1081
TYW1B	-0.102598808	0.000729308530188944	0.0018221406846901	1081
RPL31	-0.102636308	0.00072602295761018	0.00181460700757532	1081
CDYL2	-0.102643536	0.000725391197149897	0.0018132529676864	1081
ZSCAN21	-0.102725947	0.000718224747459504	0.00179600763872538	1081
GTF3C2	-0.10272601	0.000718219306912666	0.00179600763872538	1081
DNAL1	-0.102729795	0.000717891681653874	0.00179562055213773	1081
LOC10013	-0.102756556	0.000715579711056898	0.00179006001232351	1081
CADM1	-0.102795092	0.000712262601775299	0.00178220470907507	1081
PIK3C2B	-0.102799838	0.000711855014614349	0.00178140612042311	1081
ZNF788	-0.102853415	0.000707269337947148	0.00177059038311735	1081
PDP2	-0.102876862	0.00070527106411546	0.0017660267975893	1081
LOC57255	-0.10289586	0.00070365586915365	0.00176220132820739	1081
C1orf109	-0.102911777	0.000702305262124185	0.00175903761102719	1081
SIX4	-0.10291795	0.000701782044557095	0.00175838299597048	1081
ZNF384	-0.102945428	0.000699457683979027	0.00175299515349061	1081
CRYZ	-0.102952777	0.000698837227825107	0.00175165807571691	1081
PKIA	-0.102959983	0.000698229409700125	0.00175035234674079	1081
KPNA3	-0.102971849	0.000697229457004607	0.00174806315013787	1081
ZNF184	-0.103009273	0.000694084587852408	0.00174039507372252	1081
SSH1	-0.103013159	0.000693758803226297	0.00173979473310018	1081
INS-IGF2	-0.103056249	0.000690155673056266	0.00173162112762716	1081
PAR1	-0.103107449	0.000685896949246743	0.00172136464962946	1081
COBLL1	-0.103113422	0.000685401733465767	0.00172033614760377	1081
DOCK6	-0.103113554	0.000685390793620069	0.00172033614760377	1081
ABCA9	-0.103132471	0.000683824582953382	0.00171680536186389	1081

EPB41L4E	-0.103142793	0.000682971404920816	0.00171509086912613	1081
MYL3	-0.103143416	0.000682919911633105	0.00171509086912613	1081
RNF138	-0.103144649	0.000682818053477126	0.00171509086912613	1081
CRCP	-0.103169965	0.000680730049716326	0.00171031514736036	1081
NPR1	-0.103182205	0.000679722695604652	0.00170821029228489	1081
CHRNB2	-0.103199391	0.00067831055923602	0.00170487413796751	1081
TMEM150	-0.10320246	0.000678058706127511	0.00170445378524316	1081
LOC34919	-0.103208055	0.000677599771725829	0.00170351271701196	1081
NRN1	-0.103209736	0.000677461887559641	0.00170337864763966	1081
NCAM2	-0.103234793	0.00067541028361461	0.00169843217618479	1081
ZNF606	-0.10325342	0.000673888938271261	0.00169481806010918	1081
ADAM21I	-0.103283844	0.000671410873631593	0.00168879660997733	1081
NLRP12	-0.103290198	0.000670894393987491	0.00168791901672012	1081
ZSWIM6	-0.103319139	0.000668546660143253	0.0016822223917794	1081
CEP76	-0.103337737	0.00066704196427538	0.00167864589260806	1081
GRHL2	-0.103346535	0.000666331250985587	0.0016770668479641	1081
AJAP1	-0.103358072	0.000665400421543755	0.00167514265083716	1081
MAOA	-0.103387977	0.000662993012354621	0.00166970799273457	1081
B4GALT6	-0.103392149	0.000662657819428919	0.00166907248967187	1081
LDLRAD3	-0.10342137	0.000660314407811625	0.00166337798491499	1081
GLTSCR2	-0.103462032	0.000657066369790862	0.00165561000868504	1081
NDRG2	-0.10347308	0.000656186437587491	0.00165380655388465	1081
MAP9	-0.103481484	0.000655517801081833	0.00165232809094013	1081
SLC16A11	-0.103482095	0.000655469177315535	0.00165232809094013	1081
CYP3A5	-0.103490374	0.000654811181755489	0.0016511667613549	1081
OR4F21	-0.103517905	0.000652627421841405	0.0016460722941023	1081
SUN2	-0.103544394	0.000650532686670073	0.00164099436175415	1081
USP25	-0.103549614	0.000650120641005516	0.00164016033976908	1081
LOC10019	-0.103552495	0.000649893254268194	0.00163979203497404	1081
MAMLD1	-0.103589518	0.000646978697788126	0.00163325637430348	1081
PDZRN4	-0.103621241	0.000644490954062154	0.0016277921601783	1081
PTPN5	-0.103633693	0.000643516881791775	0.00162553574717183	1081
SIGLEC15	-0.103641796	0.000642883719438492	0.00162414002107956	1081

SMURF1	-0.103642696	0.000642813461007412	0.00162414002107956	1081
FAM193A	-0.103724061	0.000636488681988892	0.00160879102869087	1081
MBD1	-0.10372685	0.000636272941483901	0.00160864939836762	1081
LEP	-0.103728364	0.000636155810264295	0.00160855513967293	1081
RPAP2	-0.103748651	0.000634588644876868	0.00160499538619517	1081
PRSS16	-0.103755945	0.000634026040069081	0.00160377380426816	1081
ADRA2A	-0.103858981	0.000626128229524421	0.00158499034729448	1081
ZNF177	-0.103894201	0.0006234495570193	0.00157880469216264	1081
DDX18	-0.103900475	0.000622973471570544	0.0015777974081957	1081
KHDRBS2	-0.103916162	0.000621784744419927	0.00157498474491883	1081
EFCAB4A	-0.103918798	0.000621585172028005	0.00157467722466099	1081
ZNF816A	-0.103942302	0.00061980844064516	0.00157076878057576	1081
OPA3	-0.10395185	0.000619088051399021	0.00156914051276211	1081
SLC9A3	-0.103983712	0.00061668968357015	0.00156427595605874	1081
C9orf68	-0.104034593	0.000612877558535147	0.00155535631351607	1081
TAX1BP1	-0.104051716	0.000611599615706021	0.00155230871341789	1081
ZNF799	-0.104083023	0.000609269418312806	0.00154658926813401	1081
C18orf45	-0.104091341	0.000608651747894586	0.00154521606318039	1081
SPAST	-0.104126871	0.000606019749228763	0.00153892195237784	1081
TUBGCP4	-0.104135779	0.000605361473071845	0.00153744413452248	1081
RICH2	-0.104155835	0.00060388191672722	0.0015342667691348	1081
ZDHHC15	-0.104171416	0.000602734837353827	0.00153173877866694	1081
NKAIN3	-0.104174139	0.000602534619479351	0.00153142315284021	1081
CHML	-0.104184116	0.000601801371365569	0.00152975250803273	1081
KIAA1239	-0.104200482	0.000600600394845739	0.00152708506300232	1081
LPAR1	-0.104204366	0.000600315709236272	0.00152655389580468	1081
NACC2	-0.104205485	0.000600233677356168	0.00152653799145815	1081
TRIOBP	-0.104227052	0.000598655309971298	0.00152271606305199	1081
FKBP9L	-0.104238337	0.000597830979532422	0.00152081135025643	1081
METTL14	-0.104261572	0.000596137065098986	0.00151688533237578	1081
KCNJ13	-0.104262415	0.000596075708400797	0.00151688533237578	1081
TOP3A	-0.104307902	0.000592772742781477	0.00150908718606506	1081
C17orf107	-0.104345384	0.000590063808365014	0.00150257052452448	1081

RARS2	-0.104353117	0.000589506407288506	0.00150153073395207	1081
GATAD1	-0.104353263	0.000589495851310613	0.00150153073395207	1081
TMPRSS2	-0.104366787	0.000588522140798589	0.00149959252800248	1081
C22orf45	-0.104382481	0.000587394090268159	0.00149728634043427	1081
TDRD10	-0.104390014	0.000586853328413265	0.0014962064382771	1081
A2BP1	-0.104410928	0.000585354445539035	0.00149265383612454	1081
FLT4	-0.104494945	0.000579368925923825	0.00147813918601006	1081
CCDC146	-0.104525055	0.000577237687351537	0.00147346592428954	1081
TBC1D24	-0.10453377	0.000576622193621764	0.00147225020158561	1081
UNC5D	-0.104534379	0.00057657916838167	0.00147225020158561	1081
MGC4580	-0.10454775	0.000575636102814565	0.00147010513326565	1081
MGP	-0.104550519	0.000575441016963085	0.00146979324036026	1081
FOXI2	-0.104558603	0.000574871715564676	0.00146852532463231	1081
FBXO9	-0.10462117	0.000570483224839144	0.00145842443710464	1081
GFAP	-0.104643991	0.000568890324535299	0.00145490613022262	1081
DHX40	-0.104661054	0.000567702019264714	0.00145205144465308	1081
SYTL4	-0.104680206	0.000566370983016775	0.00144923840446149	1081
USP17L2	-0.104680409	0.000566356911068403	0.00144923840446149	1081
PCDHGC5	-0.104736236	0.000562493680426611	0.00144037564410755	1081
GPR156	-0.104751515	0.000561440694263203	0.00143786203736743	1081
ABCB1	-0.104755353	0.000561176446377298	0.00143736802444636	1081
NDEL1	-0.104768841	0.000560248801616461	0.0014353570022337	1081
MATN2	-0.10478701	0.000559001443193574	0.00143234343336741	1081
C4orf32	-0.104847445	0.000554870996090546	0.0014223026105413	1081
SNAI2	-0.104856596	0.000554248013958861	0.00142088651580571	1081
CCDC3	-0.104859085	0.000554078679476908	0.00142063319308417	1081
ISL1	-0.104864669	0.000553698978888231	0.00141984036783394	1081
CEP290	-0.104873709	0.000553084765842971	0.00141844590807214	1081
SRCIN1	-0.104877894	0.000552800651181158	0.00141789777413349	1081
PRKAR2B	-0.104902179	0.000551154634422702	0.00141406925048547	1081
ZNF623	-0.104937535	0.00054876635350619	0.00140844670548888	1081
FLJ33630	-0.104951784	0.000547806595121185	0.00140616257119218	1081
SNX4	-0.104971427	0.00054648605078761	0.00140313044775891	1081

MS4A2	-0.105037811	0.000542045067615427	0.001392615468322	1081
OR2L1P	-0.105070264	0.000539886323135894	0.00138760015049408	1081
ZNF91	-0.105071198	0.000539824258997089	0.00138760015049408	1081
ROD1	-0.105097461	0.000538083278796191	0.00138331899583324	1081
ZBTB7B	-0.105143637	0.000535034931311799	0.00137600902480227	1081
ARL4A	-0.105149744	0.000534632983495516	0.00137515084940209	1081
CSRP1	-0.105176485	0.000532876193452863	0.00137098223717854	1081
CIDEC	-0.105183628	0.000532407820739973	0.00136995217126156	1081
BCL2	-0.105189335	0.000532033923817799	0.00136916497129657	1081
GRM6	-0.105202398	0.000531178990871379	0.00136720712537436	1081
MTMR1	-0.105212307	0.000530531266374173	0.00136582138800099	1081
ITM2A	-0.105226814	0.000529584342469215	0.00136373214611304	1081
JRKL	-0.105243652	0.000528487313902011	0.00136108117342828	1081
FRMD4A	-0.105271136	0.000526701138554534	0.00135665444779198	1081
ADD3	-0.10534505	0.000521925333487701	0.00134452504387593	1081
MAP3K15	-0.105379994	0.000519681517714984	0.0013394299097196	1081
SPHKAP	-0.105433226	0.000516280711559194	0.00133117559635991	1081
CYP3A4	-0.10545873	0.000514658730647116	0.00132716335495214	1081
THAP9	-0.105464698	0.000514279826800259	0.00132635605055578	1081
PRKCH	-0.105472907	0.000513759105904459	0.00132518273859735	1081
ATP10D	-0.10550632	0.000511644729875694	0.00132006699325639	1081
RNF41	-0.105518376	0.000510883843192637	0.00131827270668831	1081
SLC30A7	-0.105542693	0.000509352284256865	0.00131482595673431	1081
TMEM164	-0.105582209	0.000506872483520312	0.00130892785290561	1081
CDON	-0.105604273	0.00050549284983874	0.00130553249487198	1081
POLA1	-0.105654109	0.000502389361510034	0.0012980163765063	1081
DLEC1	-0.105655014	0.000502333213989879	0.0012980163765063	1081
CUL9	-0.105690835	0.000500113672498593	0.00129279994000823	1081
MACC1	-0.105740014	0.000497081397991686	0.00128512638122979	1081
PALM2	-0.105769717	0.000495258278503853	0.0012807417227805	1081
SPINK8	-0.10577609	0.000494867936291185	0.00128038974782763	1081
GREM2	-0.105800921	0.00049334971407425	0.00127695361557571	1081
PGGT1B	-0.105921089	0.000486063580695408	0.00125987530019416	1081

BCL10	-0.105921798	0.000486020927515198	0.00125987530019416	1081
LHX4	-0.105944025	0.000484684352809908	0.00125678546625764	1081
LIPE	-0.105956114	0.000483958840911307	0.00125506576340863	1081
PTCH2	-0.105974288	0.000482870075805585	0.00125272603697405	1081
UBR7	-0.105991513	0.000481840288128468	0.00125021543075064	1081
PTEN	-0.106040477	0.000478924081671345	0.0012428089173347	1081
SKAP2	-0.106047783	0.000478490407099017	0.00124184350051658	1081
KLF15	-0.106080727	0.000476539219915263	0.00123725771171452	1081
CDC42SE	-0.106081412	0.000476498719020443	0.00123725771171452	1081
HAUS3	-0.106083906	0.000476351342825017	0.0012370887973714	1081
AOX2P	-0.106100233	0.000475387420078983	0.00123490387846436	1081
MFAP4	-0.106106833	0.000474998302044048	0.00123405220462695	1081
LAMC2	-0.106115914	0.000474463379875116	0.0012328214610582	1081
INTS4	-0.106132643	0.000473479435812636	0.0012304235338552	1081
GPD1	-0.106162259	0.000471742171947378	0.00122638354031229	1081
NT5DC2	-0.106226112	0.000468016748732559	0.00121764140555565	1081
ZNF805	-0.106250923	0.000466576619916286	0.00121420824289027	1081
HS3ST4	-0.10626658	0.000465669924665764	0.00121200524966302	1081
ISG20L2	-0.106319843	0.00046259773975582	0.00120432042742065	1081
EXOC8	-0.106320301	0.000462571419408026	0.00120432042742065	1081
RBMXL1	-0.106351272	0.000460793858553757	0.00120024467036016	1081
PPM1B	-0.106382645	0.000458999648321278	0.00119618989850351	1081
RAB5A	-0.106396978	0.000458182159811235	0.00119436847947688	1081
FMNL3	-0.1064097	0.000457457642269457	0.00119263416636705	1081
LOC6130[illegible]	-0.106417435	0.000457017721310346	0.00119164146981581	1081
RAB23	-0.106437195	0.000455895544508041	0.00118886935198893	1081
CAPZA3	-0.106453509	0.000454971035461304	0.00118676570107056	1081
CCDC125	-0.106454928	0.000454890675713487	0.00118670974647089	1081
SATB2	-0.106465644	0.000454284432938915	0.00118543868113905	1081
SIPA1L3	-0.106472338	0.000453906117629471	0.00118475495460556	1081
CLIP1	-0.106515969	0.00045144746181802	0.0011789485504698	1081
ZNF738	-0.106556914	0.000449151482401388	0.0011734089758755	1081
RNASE7	-0.106571599	0.000448330653245673	0.00117187245486364	1081

GPATCH2	-0.106580011	0.000447861104106365	0.00117079703376885	1081
C1orf156	-0.106619722	0.00044565061178296	0.00116532081702593	1081
BPTF	-0.106656129	0.00044363303272065	0.00116052483462832	1081
RAB9B	-0.106656616	0.000443606091457214	0.00116052483462832	1081
TIPARP	-0.106682605	0.000442171081809128	0.00115712346623082	1081
LOC72802	-0.106706892	0.000440834006457695	0.0011537743354671	1081
PTPRD	-0.106743464	0.000438827717071426	0.00114867260400258	1081
TMEM195	-0.106745473	0.00043871774624499	0.00114867260400258	1081
SEC24A	-0.106785896	0.000436510572755693	0.00114320144151891	1081
ZFX	-0.106847685	0.000433156782743404	0.00113500824510482	1081
SAPS3	-0.106957858	0.000427236387289355	0.00112022348326748	1081
TMEM74	-0.106969635	0.000426607990839433	0.00111886707140482	1081
PTGFRN	-0.107020015	0.000423929574915942	0.00111213195555172	1081
LRRK1	-0.107030482	0.000423375109604727	0.00111082203477302	1081
SYT2	-0.107036963	0.000423032082243804	0.00111006660112041	1081
SP2	-0.107038798	0.000422935022186974	0.00110995649061446	1081
KCTD9	-0.10705124	0.000422277462029102	0.0011085196081022	1081
TAS2R19	-0.107140893	0.000417567069431957	0.00109672602525512	1081
CLCA2	-0.107141162	0.000417553008808416	0.00109672602525512	1081
TRPC6	-0.107181765	0.00041543593450774	0.00109169800426017	1081
SFRP4	-0.107210044	0.000413967362920838	0.00108812271999482	1081
WNK4	-0.107244149	0.000412202719045119	0.00108376713327642	1081
THBS2	-0.107256209	0.000411580403173372	0.00108255480113952	1081
NICN1	-0.107290981	0.000409790973699477	0.00107841138669836	1081
BRD4	-0.107350411	0.000406749374009979	0.00107082673019224	1081
PPP1R13L	-0.107363972	0.000406058279962606	0.0010694265982281	1081
DCT	-0.107374266	0.00040553440857951	0.00106846595093307	1081
FOSL2	-0.107386911	0.00040489176242807	0.00106691230269633	1081
PDCD4	-0.107392599	0.000404602999289373	0.00106643038344687	1081
PAFAH2	-0.107395885	0.000404436281316237	0.00106613044845794	1081
EIF1AX	-0.107398057	0.000404326110958215	0.00106597951907515	1081
FAM133A	-0.107409904	0.000403725669157979	0.00106453581219732	1081
EPN2	-0.10741296	0.000403570900528257	0.00106426702332003	1081

TFCP2L1	-0.107437327	0.000402338982333943	0.00106129616329937	1081
MYO1E	-0.107437574	0.00040232649528005	0.00106129616329937	1081
CREB5	-0.107454771	0.000401459220634688	0.00105939166880872	1081
MARS2	-0.107456741	0.000401359958273353	0.00105926848675707	1081
RGMA	-0.107460661	0.000401162552680798	0.00105888621904542	1081
FLJ39739	-0.107486907	0.000399843122805334	0.00105554183054822	1081
LHX6	-0.107487933	0.000399791657820924	0.00105554183054822	1081
ASCC3	-0.107493154	0.000399529689797228	0.0010549909032593	1081
FAM21C	-0.107496891	0.000399342288207428	0.00105463429417129	1081
KCTD8	-0.107509717	0.000398699777293309	0.00105307552295447	1081
ELOVL5	-0.10753829	0.000397271798048113	0.00104957906513826	1081
C6orf217	-0.107554494	0.000396464131417533	0.00104785750818764	1081
LOC28339	-0.107557163	0.000396331233091887	0.00104764370694608	1081
MAGEA8	-0.107606446	0.000393884895520835	0.00104186071694685	1081
SPARC	-0.107621036	0.000393163395116167	0.00104008885024494	1081
LOC28559	-0.107625152	0.00039296005777954	0.00103982403309718	1081
TMEM144	-0.107670227	0.00039073980533655	0.00103435655433703	1081
KCNJ2	-0.107693477	0.000389599201593725	0.00103160829601809	1081
PLXNA1	-0.10769734	0.000389409955876181	0.00103124274498826	1081
TDRD5	-0.107725543	0.000388031175023187	0.00102786167269456	1081
KIAA1377	-0.107755436	0.000386574676028993	0.00102440763596475	1081
PCDHA12	-0.107763398	0.000386187595927334	0.00102351652676702	1081
TXNIP	-0.107808009	0.000384025533402157	0.00101805426640169	1081
SNED1	-0.107875317	0.000380784853912187	0.00100986188547407	1081
MSRA	-0.107915691	0.000378853191729471	0.00100500362752044	1081
KDM4B	-0.107920889	0.000378605184344978	0.00100447799797571	1081
LEPREL1	-0.107931374	0.000378105352264882	0.00100341619304124	1081
LOC44166	-0.107971756	0.0003761859941411	0.000998585696662597	1081
CBLN4	-0.107980919	0.000375751749582082	0.000997564443243416	1081
ANKRD52	-0.108013407	0.000374215882087588	0.000993748872219149	1081
PRKG2	-0.108027143	0.000373568280481153	0.000992159922253505	1081
ANKRD36	-0.108055796	0.000372220766589421	0.000988841796511127	1081
FAM83G	-0.108063533	0.000371857687152377	0.000988007532007997	1081

STX1B	-0.108104013	0.000369963418240939	0.000983363644341433	1081
TADA2B	-0.108133744	0.000368577907660222	0.000979810233546474	1081
ARMC2	-0.108166048	0.000367077944408698	0.000976209266021546	1081
LOC44242	-0.108173927	0.000366712982962014	0.000975367446761258	1081
EZH1	-0.108205207	0.000365267343628351	0.000971779006523129	1081
LOC28386	-0.108210318	0.000365031633301821	0.000971280181332082	1081
XYLB	-0.10823773	0.000363769921521038	0.000968434659579926	1081
C10orf137	-0.108254834	0.000362984741506278	0.000966599817269526	1081
LPCAT2	-0.108330673	0.000359522170536337	0.000958139188444834	1081
WHSC1L1	-0.108348694	0.000358703972520892	0.000956085145069246	1081
SETD1A	-0.108449679	0.000354150885774645	0.000944199258063821	1081
NEK10	-0.108471144	0.000353190091833262	0.00094176232958055	1081
CG030	-0.108473375	0.000353090371111546	0.000941621065136628	1081
C7orf58	-0.108498192	0.000351982864417196	0.000938791844788085	1081
IL17D	-0.108500762	0.000351868358453911	0.000938610709885334	1081
C14orf174	-0.108501739	0.000351824834387911	0.000938610709885334	1081
C16orf45	-0.108506018	0.000351634280976007	0.000938234780167107	1081
NACAD	-0.108528478	0.000350635670654853	0.000935694209212083	1081
SFRS13B	-0.108567678	0.000348899123784882	0.000931183472263706	1081
KCTD12	-0.108567799	0.000348893809077273	0.000931183472263706	1081
DCTN4	-0.10856825	0.000348873860307328	0.000931183472263706	1081
DCX	-0.108578049	0.000348441048470797	0.000930330672159602	1081
SLC16A2	-0.108581056	0.000348308338356053	0.000930099612431427	1081
DNAH2	-0.108597421	0.000347586900679405	0.000928296183771259	1081
LOC10012	-0.108614448	0.000346837749917596	0.000926541104905181	1081
USP7	-0.108657635	0.0003449444288228	0.000921727751510186	1081
SLC26A5	-0.108662352	0.000344738202077539	0.000921421133189867	1081
PALMD	-0.108678715	0.000344023775485544	0.00091987774849433	1081
DNAH12	-0.108686997	0.000343662684311609	0.000919156236784035	1081
HOXA11	-0.108712716	0.000342543620968252	0.000916406539761679	1081
DCAF6	-0.108771805	0.000339985497169738	0.000909925314266557	1081
HMGCS2	-0.108776223	0.000339794954916744	0.000909536190113978	1081
LOC40065	-0.108788965	0.000339245958482534	0.0009081873533064	1081

TMEM48	-0.108836877	0.000337189027937945	0.000902800766588237	1081
P2RY4	-0.108841292	0.000337000073389564	0.000902414791762962	1081
CHRM5	-0.108865664	0.000335958776340126	0.000899865649431171	1081
SH3RF3	-0.108866143	0.000335938338425858	0.000899865649431171	1081
EDARADI	-0.108872835	0.000335652954226798	0.000899387302951525	1081
PROM1	-0.10888439	0.0003351607286398	0.000898205784016067	1081
NAT2	-0.108904023	0.00033432593374389	0.000896326315580338	1081
PDC	-0.108929494	0.000333245807831488	0.000893549420839256	1081
REEP1	-0.108975148	0.000331317997803928	0.000888616837406487	1081
RABEP1	-0.108984819	0.000330910933612481	0.000887761453938397	1081
TASP1	-0.109020583	0.000329409771111313	0.000883969606905209	1081
BEND7	-0.109076547	0.000327073424984645	0.000878167952327825	1081
COPG2	-0.109107637	0.000325782192598473	0.000874934311411311	1081
ITIH5	-0.109147053	0.000324152044116321	0.000870672390496439	1081
MSH4	-0.109150764	0.000323998958862714	0.000870377253805756	1081
OR2A4	-0.109228135	0.000320822600963469	0.000862074335922247	1081
BHMT2	-0.109259854	0.000319528854928196	0.000859056290207995	1081
PABPC5	-0.109275683	0.000318885077371632	0.0008575543830799	1081
SESN3	-0.109280696	0.000318681455470432	0.000857235668373863	1081
CYP7B1	-0.109361316	0.000315423166087242	0.000849151367209339	1081
C5orf4	-0.109388603	0.000314327404185258	0.000846540849908024	1081
PCDHGB2	-0.10944944	0.000311897231510906	0.000840373800396417	1081
LOC28423	-0.109455117	0.000311671368808404	0.000840061509853531	1081
SMC3	-0.109470363	0.000311065525501979	0.000838540748191807	1081
FAM84A	-0.109481564	0.000310621129710324	0.000837566946595433	1081
CHRFAM7	-0.109481858	0.000310609475900254	0.000837566946595433	1081
MAP4K4	-0.109520105	0.000309096537705308	0.000833902471149516	1081
CRHR1	-0.109528023	0.000308784199651529	0.000833283014330885	1081
GAS1	-0.109637281	0.00030450419764889	0.00082250429895909	1081
GPR144	-0.109655292	0.000303804020064573	0.000820723076867482	1081
LOC10012	-0.109675296	0.000303028084916976	0.000818846515178067	1081
CLIC6	-0.109685222	0.000302643770179364	0.000818027472194189	1081
PCK1	-0.109787928	0.000298693790706418	0.000807784456139186	1081

BZW2	-0.10978979	0.000298622629173831	0.000807700438333356	1081
TRPC3	-0.109795068	0.0002984210118848	0.000807263499989162	1081
CD300LG	-0.109825094	0.000297276348895684	0.000804491140314825	1081
TECTA	-0.109849824	0.000296336677919748	0.000802055942052038	1081
FGFR1	-0.109873438	0.000295441998798027	0.000799741879304791	1081
SATL1	-0.109880889	0.000295160232003336	0.000799193934638065	1081
FLG2	-0.109902572	0.00029434167703491	0.000797191863924209	1081
ZNF14	-0.109947154	0.000292665312614655	0.000792758198550789	1081
EPM2AIP1	-0.109950272	0.00029254843428269	0.000792548172219578	1081
LNX1	-0.109973054	0.000291695543898414	0.000790450192606075	1081
COLEC12	-0.110006598	0.000290444030037132	0.000787164669056643	1081
IL1RAP	-0.110037847	0.000289282661620204	0.000784228127888442	1081
SOX10	-0.110039715	0.000289213361732026	0.000784145783592417	1081
GPR124	-0.110043305	0.000289080248819441	0.000783890377233496	1081
GRAMD1	-0.110054933	0.000288649495991752	0.000782827692077793	1081
RNASEN	-0.110124652	0.000286079306414153	0.00077637985015669	1081
KCNB1	-0.110131551	0.000285826116277762	0.000775797239614056	1081
ZBTB39	-0.110133399	0.000285758343157997	0.000775717803923709	1081
SLC25A3	-0.110237739	0.000281955826431183	0.000765808294924657	1081
ARHGEF2	-0.11026563	0.000280947412800922	0.000763275203084904	1081
BAT2	-0.110271551	0.00028073375995589	0.000762797625345482	1081
CROCCL2	-0.110280968	0.00028039430992574	0.000761978061979499	1081
CDH10	-0.110309969	0.000279351282755498	0.000759246032259782	1081
NUMA1	-0.110319524	0.000279008413257567	0.000758416473495301	1081
TOM1L1	-0.110340933	0.000278241657220162	0.000756434302928496	1081
CAPZA2	-0.110372587	0.000277111578649006	0.000753768937602515	1081
SCP2	-0.110378375	0.000276905390041327	0.00075330980180723	1081
WNT8B	-0.110501549	0.000272551724274938	0.000741666146902423	1081
RNF39	-0.110504614	0.000272444206024112	0.00074147372741904	1081
METAP1	-0.110548012	0.000270926177883902	0.000737541601820434	1081
XPC	-0.110618563	0.000268475197270225	0.000731462381526736	1081
TSPAN18	-0.110619424	0.000268445431046155	0.000731462381526736	1081
NCAPD3	-0.110670708	0.00026667704652477	0.000727054960379144	1081

USP38	-0.11068245	0.000266273691606771	0.000726053535113481	1081
KLF6	-0.110701126	0.000265633323422008	0.000724503560836224	1081
TAF13	-0.110757775	0.000263699727999152	0.000719424569412637	1081
KLHL13	-0.110759539	0.000263639701706678	0.000719358227127324	1081
ATP9A	-0.110777393	0.000263033159506432	0.000717897710101215	1081
CTNND2	-0.110785571	0.000262755792883915	0.00071723786553475	1081
PTPN4	-0.110790152	0.000262600532443366	0.000717008366233615	1081
ARNTL	-0.110843671	0.000260792937888283	0.000712169409483456	1081
APOL5	-0.110906831	0.000258674754954362	0.00070657666963466	1081
UBA3	-0.110937572	0.000257649620534601	0.000704062887367001	1081
FOXE1	-0.11095265	0.000257148213907579	0.00070278805713854	1081
FBXL2	-0.110957713	0.000256980068636571	0.000702423810404846	1081
TTC30B	-0.110986652	0.000256020850529526	0.000699896869849004	1081
ATP8B2	-0.110988738	0.000255951821602719	0.000699803127875511	1081
MUC7	-0.110993505	0.000255794174111123	0.000699561992596875	1081
ZNF469	-0.110996072	0.000255709333590315	0.000699424918557624	1081
CSDC2	-0.111015641	0.000255063325512443	0.000697933017309655	1081
RPS10P7	-0.111028064	0.000254654033853491	0.000696916928675259	1081
TRIM35	-0.111074453	0.000253131057869407	0.000693031416251591	1081
MAST4	-0.111079695	0.000252959510053936	0.000692750044866306	1081
LOC1444	-0.111108064	0.000252032955176434	0.000690494204002347	1081
IBTK	-0.111108777	0.000252009708837419	0.000690494204002347	1081
CLN5	-0.111128029	0.000251382781956507	0.000688900305062417	1081
PTGER3	-0.111137486	0.000251075336219461	0.000688151380699461	1081
FASTKD2	-0.111184386	0.000249555921778737	0.000684266237135247	1081
RASL12	-0.111199376	0.000249072117284159	0.000683032643981675	1081
XKR5	-0.111204536	0.000248905775471733	0.000682669414142691	1081
FRAS1	-0.111308198	0.000245586069215186	0.000673931530355525	1081
IQCF6	-0.111331218	0.000244854514324637	0.000672107125094673	1081
FSD2	-0.111336673	0.000244681457871005	0.00067172362616672	1081
AFAP1L1	-0.111338414	0.00024462626379295	0.000671663634197762	1081
DACT1	-0.111363582	0.000243829499441477	0.000669567239128755	1081
SMR3A	-0.111372693	0.000243541659726976	0.000668867993892287	1081

KIAA0649	-0.111399388	0.000242700118328489	0.000666738563170245	1081
SLC6A1	-0.111433206	0.000241637961608744	0.000663911175205695	1081
ZNF880	-0.111456946	0.000240894932583366	0.000661959953197642	1081
RB1	-0.111466927	0.000240583175442872	0.000661193461022737	1081
DNAJC24	-0.111484745	0.000240027567815663	0.000659756495244443	1081
HSPB6	-0.111506668	0.00023934561310241	0.000657971803486361	1081
NACA	-0.111516992	0.000239025115230738	0.000657270126443246	1081
ZNF512	-0.111524	0.000238807775179404	0.000656762133923427	1081
CCDC14	-0.111533911	0.000238500707091162	0.000656007201577205	1081
ZNF217	-0.111543002	0.000238219388769332	0.000655412399174842	1081
RCBTB2	-0.111579305	0.000237099065770174	0.000652419161308585	1081
ITSN2	-0.11159485	0.000236620866155485	0.000651192260751673	1081
TMPPE	-0.111648173	0.000234987362804174	0.000646961927523585	1081
TMEM131	-0.111653814	0.00023481519268564	0.000646576278930046	1081
ZAN	-0.111658459	0.000234673494332868	0.00064627444201444	1081
IL1R1	-0.111661832	0.000234570655801808	0.00064607955443361	1081
OPA1	-0.111669324	0.000234342357177952	0.000645539010713774	1081
C1orf101	-0.111689791	0.000233719781604792	0.000643912062421845	1081
GPR157	-0.111727293	0.000232583062276926	0.000641043342395494	1081
HSF2	-0.111737153	0.000232285037815193	0.000640309535685149	1081
ZIM2	-0.111770016	0.000231294381078877	0.000637753258531888	1081
NAIP	-0.111792302	0.000230624837319787	0.000635994161233006	1081
BCL9L	-0.111793477	0.000230589575463133	0.000635983981065828	1081
ARRDC4	-0.111796123	0.000230510214902461	0.000635852153801189	1081
HTR1A	-0.111812076	0.000230032248631954	0.000634620603764819	1081
ARSK	-0.111818013	0.000229854633877128	0.000634217449589748	1081
STARD8	-0.11183274	0.000229414539047223	0.000633263344629529	1081
SGMS1	-0.111859298	0.000228622924651835	0.000631164700165989	1081
MTMR2	-0.111915088	0.000226968287023954	0.000626682585265563	1081
RP1L1	-0.111929281	0.000226549157103196	0.000625611071945701	1081
CXCL12	-0.111930097	0.000226525075930904	0.000625611071945701	1081
RUNDC2C	-0.111934518	0.000226394677156936	0.000625355926412516	1081
THSD1	-0.111935771	0.00022635773605058	0.000625339631478186	1081

FBXL4	-0.1119672	0.000225432897028497	0.000622870074151567	1081
CCNDBP1	-0.111972586	0.000225274779326379	0.000622518577438944	1081
PLCE1	-0.111978616	0.000225097835944963	0.000622114954741565	1081
EDA2R	-0.112012783	0.000224097858724803	0.000619436246434022	1081
LAMB3	-0.112031682	0.000223546521992787	0.000617997075002703	1081
RBM46	-0.112032295	0.000223528669916034	0.000617997075002703	1081
VPS26B	-0.112038171	0.000223357517631829	0.000617644089594124	1081
RALBP1	-0.112061796	0.000222670618668516	0.000615829161597645	1081
SORCS3	-0.112095218	0.00022170230684488	0.000613319551138437	1081
RPL21P44	-0.112111911	0.000221220124131036	0.000612069688314752	1081
PCID2	-0.112126358	0.000220803642687447	0.000611085229006542	1081
RNF170	-0.112148294	0.000220172635297737	0.000609422607594519	1081
CYP7A1	-0.112167048	0.00021963449592518	0.000608016616794386	1081
PANK1	-0.112168372	0.000219596562381973	0.00060799515519308	1081
SLC25A37	-0.112181456	0.0002192219267714	0.000607041335369053	1081
CNIH3	-0.11220488	0.000218552703728334	0.000605387362450182	1081
EXOC4	-0.112221898	0.000218067700238902	0.000604177392561228	1081
DNAJC18	-0.112225487	0.00021796556993481	0.000603977497432839	1081
EPS15	-0.112276896	0.000216507212125561	0.000600184090858599	1081
EPHB1	-0.112323011	0.000215206850019598	0.000596990084500798	1081
CXorf36	-0.112327383	0.000215083955442696	0.000596731343119833	1081
C3orf23	-0.11234115	0.000214697353228568	0.000595740796251998	1081
CACNA1C	-0.112348048	0.000214503909783126	0.000595368043893781	1081
ZNF674	-0.112348895	0.000214480164382105	0.000595368043893781	1081
DCN	-0.112352746	0.000214372262871971	0.000595248688567313	1081
FSD1L	-0.112395954	0.000213164917175607	0.000592141099903835	1081
SLITRK6	-0.112401731	0.000213003966485718	0.000591775604034587	1081
MED17	-0.112425287	0.000212348907960642	0.000590118464735429	1081
C1orf226	-0.112445091	0.000211799648514096	0.000588673278051388	1081
ADAM17	-0.112467784	0.000211171892167987	0.000587009489129861	1081
IGF2AS	-0.112498529	0.000210324197983654	0.000584814488389331	1081
KCNE1	-0.112499919	0.000210285931380887	0.000584788802825507	1081
C4orf43	-0.112500716	0.000210264016171314	0.000584788802825507	1081

ERLIN2	-0.112504808	0.000210151450338775	0.000584576217491663	1081
LOC64874	-0.112505326	0.000210137207510029	0.000584576217491663	1081
ADSS	-0.112535765	0.000209301791077655	0.000582614975992726	1081
WDR65	-0.112583098	0.000208008881591203	0.00057933622558479	1081
KIAA1324	-0.112584697	0.000207965314128398	0.000579294974158818	1081
CHRDL1	-0.112593499	0.000207725766483221	0.000578707725875326	1081
VPS37A	-0.112604529	0.000207425940881016	0.000577952362247312	1081
SLC10A7	-0.112639653	0.000206473866774889	0.000575458777833445	1081
EDEM1	-0.112643566	0.000206368063916964	0.000575243482441847	1081
GLRB	-0.112679178	0.000205407416525384	0.000572724208429599	1081
GLRA4	-0.112681208	0.000205352794166325	0.000572651168117471	1081
FNDC4	-0.112686721	0.000205204482141694	0.000572316806416231	1081
ZNF8	-0.112753506	0.000203415890212289	0.0005675641424275	1081
TMLHE	-0.112780026	0.000202709723647277	0.000565750537943252	1081
NEGR1	-0.112795273	0.00020230474571166	0.000564698503860522	1081
BCL2L2	-0.112800396	0.000202168872329925	0.000564397440837906	1081
TCEAL2	-0.112821796	0.000201602112666009	0.000563029385191331	1081
EIF4G3	-0.112830588	0.000201369714529689	0.000562556219553541	1081
ARMCX6	-0.112863981	0.000200489296474138	0.000560329755476069	1081
CSNK1G1	-0.112885656	0.000199919753741309	0.000558815518123861	1081
CXorf57	-0.112886721	0.000199891808002651	0.000558814942022399	1081
PRDM11	-0.112920775	0.000199000181515653	0.000556399535965696	1081
IRS1	-0.112929054	0.000198783975400376	0.000555872179961212	1081
ACN9	-0.11294506	0.000198366616466007	0.000554859134783075	1081
WNK3	-0.112950679	0.000198220287160292	0.000554603845117233	1081
GOLPH3L	-0.112979137	0.000197480770234992	0.000552812055363404	1081
C8orf39	-0.113016253	0.000196520163017272	0.000550316849204531	1081
NCRNA0(	-0.113060449	0.000195382019932112	0.000547422919545534	1081
MESTIT1	-0.113089291	0.000194642617682283	0.00054542711548332	1081
ZNF19	-0.113117625	0.00019391879932892	0.000543550050435661	1081
LOC90586	-0.11315947	0.000192854426552415	0.000540641862357139	1081
WDTC1	-0.11317882	0.000192364117035269	0.000539342399119763	1081
NBR1	-0.113216831	0.000191404311268803	0.000536726037097721	1081

MLLT10	-0.113285312	0.000189686497577975	0.000532330686632843	1081
YTHDC2	-0.113305671	0.000189178583417681	0.000531224221208416	1081
SLC17A5	-0.11331528	0.00018893930673689	0.00053062628387211	1081
FAM171A	-0.11332984	0.000188577302665371	0.000529683458197699	1081
PNPLA8	-0.113347802	0.000188131585259522	0.00052865264124049	1081
CNN1	-0.113354846	0.000187957071248655	0.000528235937542432	1081
ETV6	-0.113392382	0.000187029638002299	0.000525776173256533	1081
FAM55D	-0.113447806	0.000185668112063165	0.000522240172788672	1081
WARS2	-0.113450731	0.000185596514373266	0.000522111685804977	1081
TIAM2	-0.113461154	0.000185341590319476	0.000521467365500815	1081
C3orf36	-0.113471472	0.000185089570456975	0.000520976581927589	1081
BARHL1	-0.113509207	0.00018417059307385	0.000518534814461595	1081
DCAF12L	-0.113512199	0.000184097915903354	0.000518402644097437	1081
SECISBP2	-0.113516159	0.000184001768037355	0.000518204336238294	1081
SRF	-0.113636693	0.000181097491906884	0.000510381781542275	1081
ID4	-0.113663343	0.000180461196452816	0.000508730870772736	1081
ITCH	-0.113676774	0.000180141314391485	0.000508042388130543	1081
TMEM87I	-0.113680908	0.000180042954264812	0.00050783608424316	1081
RHOQ	-0.113701957	0.000179542962865131	0.000506496707312429	1081
ACSM3	-0.113723875	0.000179023717944062	0.000505102632770747	1081
LOC72860	-0.113727135	0.000178946604828276	0.000504955785721477	1081
LYPLA1	-0.113739348	0.000178658011021725	0.000504212052680393	1081
LOC10030	-0.113799564	0.000177241429242611	0.000500494616217044	1081
CAMKK2	-0.113815402	0.000176870595984768	0.000499517475972684	1081
RGAG1	-0.113837182	0.000176361839788913	0.000498150485494624	1081
CSGALNA	-0.113843679	0.000176210337812116	0.000497792351034228	1081
COL5A1	-0.113870761	0.000175580125162992	0.000496081573830081	1081
PCDHB16	-0.113887351	0.000175195130841716	0.000495132703536246	1081
POLR2A	-0.113937473	0.000174036774729737	0.000491997028758146	1081
ADAM23	-0.113956304	0.000173603438572508	0.000490840880006059	1081
ZBTB47	-0.113956377	0.000173601763924588	0.000490840880006059	1081
MMAA	-0.113958422	0.000173554763281157	0.000490840880006059	1081
PCDHB6	-0.11400534	0.000172479768224962	0.00048800631051852	1081

LRPPRC	-0.11400551	0.000172475877627089	0.00048800631051852	1081
FBXO28	-0.114020104	0.000172142797024534	0.000487189750781013	1081
CA8	-0.114021214	0.00017211747867136	0.000487189750781013	1081
ZNF154	-0.11402153	0.00017211027711857	0.000487189750781013	1081
NRIP1	-0.114041719	0.000171650572376521	0.000486138166810771	1081
NT5C1A	-0.11408792	0.000170602871998773	0.000483306828352592	1081
TSN	-0.11415182	0.000169163719064381	0.000479364625200726	1081
FAP	-0.114155281	0.000169086086697596	0.000479212045093286	1081
TTF1	-0.114170267	0.000168750375305765	0.000478397126594277	1081
DLL1	-0.114184836	0.000168424603077379	0.000477606085162414	1081
DKFZp68(	-0.114200585	0.000168073104250996	0.00047667643040072	1081
FAM185A	-0.11420527	0.000167968681519239	0.000476447351338366	1081
PARG	-0.114222894	0.00016757639504214	0.000475535494875885	1081
G2E3	-0.114231104	0.00016739394130774	0.000475084664362414	1081
PKDREJ	-0.11424133	0.000167166944268282	0.000474507269590608	1081
KIF3A	-0.114247078	0.00016703946965557	0.000474212248620555	1081
TRIM45	-0.114252976	0.000166908768848256	0.000473974788334947	1081
PTCHD1	-0.114254044	0.000166885120161474	0.000473974446024657	1081
COMMD1	-0.114261895	0.000166711325806954	0.000473547611164847	1081
UGT8	-0.114298619	0.000165900581158672	0.000471377603306269	1081
SIGLECP3	-0.114307735	0.000165699919447942	0.000470873871812496	1081
LYPD6	-0.114324413	0.000165333379148137	0.000469898550076074	1081
FAM150B	-0.114340004	0.000164991410281352	0.000468992798097621	1081
LAMA4	-0.114390311	0.000163892507308535	0.000466132226419658	1081
FAM196B	-0.114416376	0.000163325863464658	0.000464586207214845	1081
KIAA153(	-0.114491039	0.000161712877075611	0.000460192952209096	1081
SHC4	-0.114510376	0.000161297574776227	0.000459270621041286	1081
MEX3B	-0.114520802	0.000161074076086623	0.000458699075878573	1081
SAMD4B	-0.114534951	0.000160771225801179	0.000457901363461722	1081
NEK11	-0.114540577	0.000160650968094928	0.000457623550943484	1081
ABCE1	-0.114559242	0.000160252541632057	0.000456553168035325	1081
ZNF323	-0.114565835	0.000160112031602301	0.000456281917757582	1081
DHX32	-0.114586659	0.000159669000730735	0.000455083760571684	1081

IRX2	-0.11461451	0.000159078278273119	0.000453528434165296	1081
SMEK2	-0.114623603	0.00015888586024911	0.000453043970943853	1081
KLHDC7A	-0.11465559	0.000158210697399137	0.000451246566488123	1081
DBC1	-0.114675914	0.000157783101164799	0.000450154450214541	1081
C12orf39	-0.11473906	0.000156461552931042	0.000446510551607288	1081
RSPRY1	-0.114780693	0.000155595926108318	0.000444166066522894	1081
C1orf203	-0.114800827	0.000155178906370598	0.000443101214576285	1081
COL4A5	-0.114807062	0.000155049987913186	0.00044279586142772	1081
FAM154B	-0.114820955	0.000154763087600665	0.000442039188957239	1081
GCFC1	-0.114832077	0.000154533752413157	0.000441446744521136	1081
LRRC34	-0.114857322	0.000154014423547402	0.000440025608050263	1081
LARP1B	-0.11489188	0.000153306135408386	0.000438126272918418	1081
DNAH1	-0.114899253	0.000153155428417978	0.000437757676713984	1081
KDM4DL	-0.114940992	0.000152304853342331	0.000435450080979458	1081
TRIM68	-0.114945213	0.000152219083881157	0.000435266635171882	1081
TMEM123	-0.114964997	0.000151817685047289	0.000434303786605742	1081
FAM114A	-0.114969015	0.000151736291497269	0.000434132593695851	1081
KIAA0368	-0.114984132	0.000151430391818844	0.000433380486317747	1081
SMAD7	-0.114994901	0.000151212845149433	0.000432880881843873	1081
OVCH2	-0.115008258	0.000150943418051816	0.000432232431649444	1081
LRRC32	-0.115028325	0.00015053949470768	0.000431137065806968	1081
FAM180A	-0.115044573	0.000150213182497047	0.000430263694213424	1081
UGT2B11	-0.115106253	0.000148980504202192	0.000426914972568016	1081
RFPL4B	-0.115202754	0.000147070979006286	0.000421563015378718	1081
CDKL1	-0.115208515	0.000146957714721376	0.000421298301275384	1081
ZNF860	-0.115211542	0.000146898247124301	0.00042118775808697	1081
ANKRD20	-0.115243103	0.000146279426573709	0.000419532894124056	1081
CNTF	-0.115250624	0.00014613232047326	0.0004191706672268	1081
MAP1LC3	-0.115260783	0.00014593383518488	0.000418660938450499	1081
ABCB10	-0.115262419	0.000145901889320753	0.000418628907615235	1081
DIMT1L	-0.115263876	0.000145873459268965	0.000418606956833804	1081
ANKMY1	-0.115298859	0.000145192197180923	0.000416711328139293	1081
ACVR1B	-0.115303263	0.000145106629521701	0.000416525085738767	1081

SDCCAG1	-0.115320204	0.000144777991340001	0.000415759463370538	1081
CNOT6	-0.115324363	0.000144697410540158	0.000415587301872182	1081
ZNF215	-0.115330202	0.000144584349359942	0.000415321790653934	1081
PCDH19	-0.11534756	0.000144248763487982	0.000414416905371563	1081
ZNF689	-0.115401489	0.000143210770717906	0.000411610925397662	1081
CYP4Z2P	-0.115407902	0.000143087800896419	0.00041131617423778	1081
SLCO1A2	-0.115425655	0.000142747915728803	0.000410397711196909	1081
TAS2R14	-0.115457612	0.000142138015002511	0.000408702585244875	1081
SERPINE2	-0.115474227	0.000141821881414899	0.00040791002300159	1081
EYA4	-0.115507757	0.000141185929941007	0.000406138877432755	1081
DPYSL3	-0.115511649	0.000141112285910646	0.000405985004237356	1081
MBTPS1	-0.11553701	0.000140633284564219	0.000404722502506598	1081
CD3EAP	-0.115547629	0.000140433170307018	0.000404204345740087	1081
TAF1A	-0.11562128	0.00013905260931344	0.000400402346286341	1081
PGPEP1	-0.115623406	0.000139012943602543	0.000400345353663077	1081
CHRM4	-0.115635719	0.000138783462044432	0.00039974161322349	1081
THAP1	-0.115652127	0.000138478199075149	0.000398976447421179	1081
IMP5	-0.115658116	0.000138366927514388	0.000398712881530159	1081
C8orf42	-0.115674297	0.000138066743874169	0.000397904800478559	1081
PABPC1	-0.115713277	0.000137346087881924	0.000396054529112706	1081
AKAP10	-0.115718624	0.000137247522885954	0.000395826964715469	1081
HS6ST3	-0.115750541	0.000136660472777429	0.000394246774180338	1081
CSN3	-0.115762445	0.000136442135733598	0.000393729669725445	1081
MGC2664	-0.115813655	0.000135506582294223	0.000391310220802339	1081
SPATA7	-0.115846061	0.000134917702572451	0.000389777300777574	1081
PRKD3	-0.115849044	0.000134863611313844	0.000389676914790215	1081
GPR17	-0.115882986	0.000134249606646751	0.000387958445832564	1081
LAMB1	-0.115896903	0.000133998625040441	0.000387344282026072	1081
ZFP30	-0.11593571	0.000133301063821412	0.000385383170304586	1081
SLCO2A1	-0.115979154	0.000132524215806482	0.000383192238757225	1081
RECQL	-0.11600127	0.000132130396434428	0.00038210836005908	1081
FCGBP	-0.116012172	0.000131936665998761	0.000381602891104816	1081
LRRTM2	-0.116019286	0.000131810388174189	0.000381292399449891	1081

NCRNA00	-0.116047886	0.00013130389259185	0.000379936356831775	1081
ACADSB	-0.116070695	0.000130901243887297	0.000378934553543556	1081
LOC72864	-0.116113654	0.000130146083063661	0.000376965182360525	1081
LOC55011	-0.116124246	0.000129960504723016	0.000376481789997867	1081
ZNF833	-0.116131131	0.000129840032087981	0.000376186890034861	1081
FBXL22	-0.116138569	0.000129709987254265	0.000375864167611792	1081
LDB1	-0.116208472	0.000128493773003269	0.000372500655799519	1081
JAK1	-0.116225364	0.000128201489630211	0.000371813850935876	1081
GYG2	-0.116266361	0.00012749471318206	0.000369817278193319	1081
LOC90784	-0.116274653	0.000127352202640352	0.000369457102849927	1081
XRN2	-0.116290703	0.000127076807645991	0.00036876437482404	1081
ARHGAP1	-0.116296666	0.000126974616264486	0.000368520911201279	1081
SNX33	-0.116345581	0.000126139361465454	0.000366255035560905	1081
FSTL1	-0.116355534	0.000125970044055088	0.000365816136296633	1081
C14orf28	-0.116357003	0.000125945075182058	0.000365796358065536	1081
MUM1	-0.116380944	0.000125538747616286	0.000364668791741901	1081
GPM6B	-0.116394728	0.000125305352538906	0.000364043312214631	1081
MLLT6	-0.116427314	0.000124755242480435	0.000362549676827519	1081
FAM69C	-0.11642885	0.000124729368333618	0.00036252678186131	1081
EXOC5	-0.116446742	0.000124428343774402	0.000361756239765525	1081
RNF165	-0.116449588	0.000124380526496062	0.000361669414876324	1081
GOLPH3	-0.116616467	0.000121606647085503	0.000353910127930865	1081
MAGT1	-0.116643177	0.000121168110838911	0.000352735779313565	1081
FBXO11	-0.116667522	0.00012076970134651	0.000351626771733696	1081
SEMA3G	-0.116667622	0.000120768077368161	0.000351626771733696	1081
PIP5K1B	-0.116668348	0.000120756201145917	0.000351626771733696	1081
POLR3E	-0.116694459	0.000120330313112185	0.000350550131257408	1081
GRID1	-0.116737835	0.000119625947266964	0.000348598974062344	1081
KCNQ1OT	-0.116754848	0.000119350733379248	0.00034789763043336	1081
KCNQ4	-0.11675964	0.000119273325704176	0.000347722307714995	1081
USP28	-0.116824397	0.00011823187575869	0.000344835838592559	1081
CLUL1	-0.116849146	0.000117836117676591	0.000343781113771895	1081
PRDM15	-0.116902609	0.000116985444817896	0.000341398201630671	1081

ACTA2	-0.116910799	0.00011685564590608	0.000341118241816835	1081
FNIP1	-0.116915924	0.000116774484872204	0.000340930724311675	1081
CHRNA7	-0.116917775	0.00011674518427593	0.000340894584322163	1081
NTNG1	-0.116958745	0.000116098473727314	0.000339153676513448	1081
GRM7	-0.116970333	0.00011591617479858	0.000338670245296214	1081
API5	-0.117028831	0.000114999972777613	0.000336139647650177	1081
ZNF98	-0.117094759	0.000113975584499107	0.000333193752682413	1081
VAV3	-0.117305899	0.000110752428632333	0.000323912264053186	1081
PRMT6	-0.117388862	0.000109509617728046	0.000320370497985984	1081
GAS2	-0.117510615	0.000107709499792067	0.00031547075796906	1081
POSTN	-0.117521363	0.000107551924285294	0.000315100860198843	1081
TPM1	-0.117527464	0.000107462583999615	0.000314884909770509	1081
LMX1A	-0.117548413	0.000107156337070777	0.000314170340604104	1081
EBF1	-0.117558893	0.000107003454339855	0.000313813449945608	1081
WDR44	-0.117573842	0.000106785713450946	0.000313266083802143	1081
SEC63	-0.1176715	0.00010537353587177	0.000309438758037434	1081
SENP1	-0.117682358	0.000105217617504088	0.000309070998048973	1081
IGSF9B	-0.117700293	0.000104960537559858	0.000308360803433474	1081
SLC22A23	-0.117705648	0.000104883895398801	0.000308180582381686	1081
FAM182A	-0.117713238	0.000104775367237004	0.000307906604374829	1081
LOC49375	-0.117717749	0.000104710913708355	0.000307762088802861	1081
UBR4	-0.117740524	0.000104386046452979	0.000306852021858348	1081
DLGAP1	-0.117773101	0.000103923019536517	0.000305624704899727	1081
IRX1	-0.117784875	0.000103756148849049	0.00030517851052184	1081
MXRA5	-0.117811114	0.000103385183384923	0.000304131793120514	1081
ULK4	-0.117834795	0.000103051459232516	0.000303238627846778	1081
SEC14L2	-0.117851064	0.000102822772329909	0.000302654112885156	1081
NXT2	-0.117866658	0.000102604022963572	0.000302054368347386	1081
UNC5C	-0.117868274	0.000102581370735801	0.000302031820150938	1081
ZNF700	-0.117872223	0.000102526059396775	0.000301913092610441	1081
TEX9	-0.117902194	0.000102107157207532	0.000300723491512534	1081
LOC28541	-0.11790528	0.000102064120859252	0.00030064069523463	1081
SLC6A14	-0.1179096	0.000102003903472614	0.000300507258768032	1081

GPR85	-0.117917003	0.000101900772026276	0.000300291259869708	1081
SFRP2	-0.117928033	0.000101747310431522	0.000299882892266717	1081
KITLG	-0.117945825	0.000101500233638894	0.000299198449905696	1081
FLJ14107	-0.117981601	0.000101005095973208	0.000297782475981307	1081
LOC64685	-0.117985085	0.000100957006676825	0.000297684265149976	1081
IGFN1	-0.118016396	0.000100525733420466	0.000296586247767325	1081
DMGDH	-0.118083765	9.96036925584271e-05	0.000294038157472086	1081
RNF6	-0.11809013	9.95169935047928e-05	0.000293825272483373	1081
SLC4A8	-0.118102479	9.93489724803156e-05	0.000293464006726088	1081
OR2T8	-0.11810449	9.93216428858019e-05	0.000293463551765104	1081
ZNF302	-0.118164771	9.85055574109639e-05	0.000291137683985309	1081
GMFB	-0.118230546	9.7622307557234e-05	0.000288654247136427	1081
FAR1	-0.118236528	9.75423553321824e-05	0.0002884601802946	1081
SUSD5	-0.118256016	9.72823006244856e-05	0.000287733364569118	1081
HECA	-0.118258609	9.72477481735177e-05	0.000287673404839282	1081
C9orf85	-0.118267782	9.71256049597167e-05	0.000287354282848214	1081
FAM115C	-0.118308184	9.6589368014109e-05	0.000285851743593981	1081
ZFP91	-0.118346824	9.60791220852996e-05	0.000284425262954939	1081
BTG2	-0.11837246	9.57420086144577e-05	0.000283468954076756	1081
KIF13A	-0.118374492	9.57153268548491e-05	0.000283431612449057	1081
ZBTB33	-0.118399461	9.53881467913798e-05	0.00028254583401152	1081
MFAP3L	-0.118416519	9.51652299427841e-05	0.000281969205697671	1081
TBC1D15	-0.118448793	9.47448094418233e-05	0.000280846701913704	1081
FAM199X	-0.118519921	9.38244153949866e-05	0.000278364189710163	1081
TRMT11	-0.118526115	9.37446772674338e-05	0.000278168584998152	1081
HUWE1	-0.118531855	9.36708271706612e-05	0.000277990396781522	1081
OR2AK2	-0.118564768	9.32484478276853e-05	0.000276818447021621	1081
ZNF607	-0.118621594	9.252342024648e-05	0.000274828118676694	1081
PAPSS1	-0.118621831	9.25204141476132e-05	0.000274828118676694	1081
PLCB1	-0.118624133	9.24911521657622e-05	0.000274828118676694	1081
MAPT	-0.118645347	9.22219358236542e-05	0.000274094260426013	1081
F3	-0.118685151	9.17188103686642e-05	0.00027280015279444	1081
FAM48B2	-0.118693929	9.16082030874273e-05	0.000272511407441852	1081

NRK	-0.11879078	9.03961676722712e-05	0.000269064834184087	1081
GATSL2	-0.118806421	9.02018501193785e-05	0.000268565810028803	1081
LOC72979	-0.118818611	9.0050687944613e-05	0.000268155374522429	1081
ZNF211	-0.118848508	8.96809496963491e-05	0.000267172838159265	1081
ZNF280C	-0.11886848	8.9434745765145e-05	0.000266478768442368	1081
DCUN1D3	-0.118913457	8.88826477639129e-05	0.000264912108182279	1081
PCDH7	-0.118927027	8.87166982550169e-05	0.000264456627160005	1081
GPR109B	-0.118948719	8.84520449820757e-05	0.000263745773558898	1081
DEPDC4	-0.118952381	8.84074412202715e-05	0.0002636908355615	1081
RYR3	-0.118971288	8.81774704868021e-05	0.000263082811456847	1081
TAS2R50	-0.119107326	8.65394399289478e-05	0.00025838698938323	1081
INTS3	-0.119115049	8.64473138925283e-05	0.000258150183570261	1081
NXPH1	-0.119130139	8.62675737339258e-05	0.000257710887793131	1081
FBXO8	-0.11918322	8.56381196845382e-05	0.00025603738810404	1081
STXBP3	-0.119210723	8.53136750713616e-05	0.000255143109310062	1081
ZNF92	-0.119275968	8.45486570907318e-05	0.000252930308448588	1081
NECAB1	-0.11928045	8.44963477939486e-05	0.000252811365856096	1081
EXOC1	-0.119307471	8.41815931700337e-05	0.000251944465073589	1081
SPDYE3	-0.119315902	8.40836128447105e-05	0.000251688615268454	1081
INE1	-0.119334358	8.38695112391252e-05	0.000251122369784807	1081
SLC15A2	-0.119335988	8.38506255449138e-05	0.000251103144284568	1081
CASC1	-0.119343184	8.37672993448588e-05	0.000250928214914822	1081
EXOC6	-0.119347115	8.37218111485202e-05	0.000250829251277058	1081
RPUSD4	-0.119348662	8.37039256348614e-05	0.000250812967709993	1081
ZNF208	-0.119406179	8.30412014859132e-05	0.000248901205763089	1081
RASSF8	-0.119407922	8.30211946350706e-05	0.000248878268738616	1081
ZNF473	-0.119416796	8.29194094224092e-05	0.000248610135855698	1081
WASF1	-0.1194661	8.23560459432981e-05	0.000246994572804487	1081
RFX6	-0.119480268	8.21948287206709e-05	0.000246584486162013	1081
GLB1L2	-0.119495289	8.20242268393214e-05	0.000246109331200198	1081
PLEKHH2	-0.119500915	8.19604209354664e-05	0.00024595451806122	1081
VLDLR	-0.11953888	8.15310293073278e-05	0.000244811832671951	1081
CDH19	-0.119580668	8.10608719727497e-05	0.000243508986861175	1081

MPV17L	-0.119581277	8.10540429304598e-05	0.000243508986861175	1081
DENND1I	-0.119584857	8.10138818304866e-05	0.00024344043100763	1081
FLNC	-0.119589078	8.09665531729217e-05	0.000243370816721651	1081
COL27A1	-0.119598903	8.08565086057422e-05	0.000243076311873254	1081
SHISA7	-0.119614105	8.06865047122019e-05	0.000242601438421986	1081
ZNF235	-0.119619452	8.06267918995728e-05	0.000242458086702029	1081
KCNRG	-0.119642375	8.03712682132724e-05	0.000241725768610984	1081
FAM35B	-0.11972576	7.94482112848833e-05	0.000239092353799518	1081
ZNF546	-0.119743333	7.92549546176743e-05	0.000238546400832668	1081
PXDN	-0.119748083	7.92027931625556e-05	0.000238460658834207	1081
BTNL9	-0.119763583	7.90328189493354e-05	0.000237984474997662	1081
KLF3	-0.119786014	7.87874463694926e-05	0.000237316553097104	1081
LOC14869	-0.11983609	7.82422378776577e-05	0.000235850648218676	1081
ADCY5	-0.119858059	7.80041839726436e-05	0.000235203455490032	1081
CHD6	-0.119880417	7.77626043870676e-05	0.000234545241110567	1081
SOX6	-0.119896549	7.75887390936747e-05	0.000234055877364791	1081
C6orf25	-0.119896949	7.75844360746067e-05	0.000234055877364791	1081
SPRED3	-0.119902164	7.75283104087676e-05	0.00023394365086648	1081
RUFY3	-0.119913453	7.74069470687969e-05	0.000233612426771672	1081
MAFG	-0.119932537	7.72022044288459e-05	0.000233029428861118	1081
RPL34	-0.119942501	7.70954958771794e-05	0.000232742209567778	1081
ALDH9A1	-0.119945168	7.70669596605953e-05	0.000232690932608317	1081
HOMER1	-0.119979314	7.67024897944454e-05	0.000231694655406973	1081
CNRIP1	-0.119988112	7.6608847163166e-05	0.000231446494616373	1081
UNKL	-0.120002203	7.64590873059533e-05	0.000231063353402105	1081
CUL4B	-0.120015747	7.63154023772429e-05	0.0002306637330667	1081
KIAA1407	-0.120124966	7.51660126576236e-05	0.000227394402310832	1081
PALB2	-0.120144508	7.4962090001202e-05	0.000226811550476752	1081
ARHGEF6	-0.120154758	7.48553396566269e-05	0.000226522580348918	1081
ATR	-0.120174581	7.46492882235082e-05	0.000225966928814812	1081
FOXP1	-0.120290588	7.34541938220495e-05	0.000222483045338323	1081
WAC	-0.120300362	7.33543298398025e-05	0.000222280832524492	1081
ZNF439	-0.120313269	7.32226578376921e-05	0.000221915216208862	1081

C3orf59	-0.120314751	7.32075469291544e-05	0.000221902803624408	1081
HYMAI	-0.120335242	7.29990027559458e-05	0.000221358058625181	1081
COL8A2	-0.120395705	7.23869078575166e-05	0.000219613593191216	1081
COL3A1	-0.120431345	7.20283828883011e-05	0.000218591710347217	1081
HCFC2	-0.12050257	7.13168986598038e-05	0.000216497728074404	1081
DNAH10	-0.120528128	7.10632172177025e-05	0.000215760137279671	1081
LRRC37A	-0.120565011	7.06986326301188e-05	0.000214685552356609	1081
WLS	-0.120590101	7.04516292784038e-05	0.000213995727044842	1081
MYO3A	-0.120594983	7.04036630718034e-05	0.000213886561993889	1081
TEF	-0.120631321	7.00476041009114e-05	0.000212857430911839	1081
TACC1	-0.120639615	6.99665679623647e-05	0.00021268696417713	1081
KIAA1107	-0.120680422	6.95691769443657e-05	0.000211670604069513	1081
GPRC5B	-0.12068052	6.95682304808828e-05	0.000211670604069513	1081
ZNHIT6	-0.120713566	6.92479870046593e-05	0.0002107888634344	1081
ZNF321	-0.120762767	6.87737668199947e-05	0.000209471958359358	1081
BSPRY	-0.120774259	6.86634461245869e-05	0.000209167567243279	1081
XIST	-0.120779179	6.86162667129782e-05	0.000209055458701293	1081
GALNTL6	-0.12080773	6.83430857384338e-05	0.000208254645621048	1081
BHMT	-0.120820051	6.82255103339257e-05	0.000207927822341442	1081
ERC2	-0.120840733	6.80285787564006e-05	0.000207359013322392	1081
SLC5A9	-0.120844306	6.79946103548514e-05	0.000207286838014298	1081
ADAMTS(	-0.120853199	6.79101370394823e-05	0.000207060649411287	1081
NXPH2	-0.120860679	6.78391669807526e-05	0.000206875570515783	1081
PCDHA6	-0.120967105	6.68369181808983e-05	0.000203911815349719	1081
ABHD5	-0.12097862	6.67293196531422e-05	0.000203645227149303	1081
LPL	-0.121030956	6.62423394907302e-05	0.000202250974392355	1081
LOC40237	-0.121103315	6.55745852207428e-05	0.000200333639562005	1081
BRAF	-0.121115973	6.54584279461649e-05	0.000200069796840463	1081
COL15A1	-0.121178095	6.48911732999259e-05	0.000198366113221094	1081
KLHL23	-0.121180806	6.48665213747705e-05	0.000198320848853354	1081
C21orf81	-0.121184156	6.48360784619516e-05	0.000198257862874319	1081
C14orf102	-0.121210244	6.45994377764774e-05	0.000197624248140795	1081
LTBP1	-0.121221071	6.45014647442721e-05	0.000197354496851969	1081

PDE2A	-0.121238016	6.43484241436301e-05	0.000196916148317397	1081
SOX2OT	-0.121294858	6.38375252972162e-05	0.000195441785275444	1081
ENDOU	-0.121317886	6.36316482873418e-05	0.000194870713704545	1081
NPHP4	-0.121377208	6.31041642216182e-05	0.000193314079720879	1081
SYNM	-0.121393106	6.29635027922499e-05	0.000192912511596939	1081
WBP2NL	-0.121413933	6.27796811419633e-05	0.000192378563523707	1081
ALX3	-0.121415731	6.27638435640624e-05	0.000192359292347184	1081
CLDN10	-0.121464128	6.23387469941166e-05	0.000191114603286635	1081
EMX2	-0.121509058	6.19465377116602e-05	0.000189970010991231	1081
APOLD1	-0.121523535	6.18206666876193e-05	0.000189612870039904	1081
LETMD1	-0.121528268	6.1779566121763e-05	0.000189515663091658	1081
CSMD1	-0.121548868	6.16009843600406e-05	0.000189025412023308	1081
ZFYVE1	-0.121551982	6.15740256989947e-05	0.000188971472837637	1081
CCDC28A	-0.121562828	6.14802386807698e-05	0.000188741147245368	1081
SYN2	-0.121564956	6.14618511917381e-05	0.000188741147245368	1081
BCL6B	-0.12159503	6.12025605172762e-05	0.000188089333580554	1081
PMS1	-0.121604294	6.11229033205348e-05	0.000187873190020167	1081
KIAA171:	-0.121612406	6.10532276565855e-05	0.000187687665365774	1081
RALGAP/	-0.121617963	6.10055419927996e-05	0.00018756969527548	1081
IGDCC4	-0.121628972	6.09111697016119e-05	0.000187308122979541	1081
5-Mar	-0.12165008	6.07306205190211e-05	0.000186781427535218	1081
SLC7A1	-0.121663608	6.06151744302191e-05	0.0001864833061846	1081
SLC25A1:	-0.121692091	6.03727840227592e-05	0.000185765959086373	1081
AMIGO1	-0.121709113	6.02283605591827e-05	0.00018537820068216	1081
TP63	-0.12171443	6.01833170166342e-05	0.000185267866946836	1081
SERBP1	-0.121726824	6.00784402012217e-05	0.000184973281041359	1081
C1orf115	-0.121755185	5.98391029343863e-05	0.000184292727199696	1081
PRPF38B	-0.121765803	5.97497343534023e-05	0.000184045626689494	1081
PHLDB1	-0.121768262	5.97290493845602e-05	0.000184010047385222	1081
OR6B3	-0.12181457	5.93408766021477e-05	0.000182898096565218	1081
BTD	-0.121817539	5.93160690672047e-05	0.000182849611531574	1081
RNF214	-0.121840483	5.91247011613158e-05	0.000182343402463978	1081
BEAN	-0.121887809	5.8731819149903e-05	0.000181214963512758	1081

ZSCAN2	-0.12191381	5.8517015996856e-05	0.000180607520646034	1081
ABCD3	-0.121956603	5.81651147596264e-05	0.000179576434763628	1081
TP53INP1	-0.12196614	5.80869591529599e-05	0.000179362629082829	1081
TSPAN2	-0.121973819	5.80241014882342e-05	0.000179196002526518	1081
SOX4	-0.12201673	5.7674040268659e-05	0.000178142217297169	1081
NCRNA0(	-0.122021368	5.76363269501038e-05	0.000178053029659537	1081
ELK4	-0.122058162	5.733794466712e-05	0.000177209426944379	1081
ZNF434	-0.12207626	5.71917142117223e-05	0.000176815083301895	1081
ZBTB7C	-0.122080245	5.71595659427737e-05	0.000176742817485368	1081
LOC1001ε	-0.122090288	5.70786133130657e-05	0.000176519598586384	1081
DCAF12L	-0.122146443	5.66279749934748e-05	0.000175179753722904	1081
SLTM	-0.122153356	5.65727285860717e-05	0.000175035726826358	1081
LOC15078	-0.122156748	5.65456393189668e-05	0.000174978787109153	1081
CABIN1	-0.122176237	5.63902437749354e-05	0.00017452472896698	1081
QTRTD1	-0.122182206	5.63427281518902e-05	0.000174404465061437	1081
ZNF124	-0.122212965	5.60984906434684e-05	0.00017372853097812	1081
VIT	-0.122220997	5.60348787724447e-05	0.000173558215386055	1081
ZNF714	-0.122223294	5.60167011637309e-05	0.000173528593717262	1081
STAB2	-0.122243225	5.58591901684134e-05	0.000173067269446738	1081
ZNF710	-0.122253773	5.57759992569993e-05	0.000172836102912206	1081
POLR1B	-0.12230614	5.53647354293577e-05	0.000171614493802802	1081
MYCT1	-0.122316779	5.52815371164452e-05	0.000171382974024437	1081
COL12A1	-0.122322752	5.52348731931591e-05	0.000171291028398428	1081
CAPS2	-0.122335508	5.51353486811698e-05	0.000171035047610435	1081
ZC3H4	-0.122349832	5.50237995498526e-05	0.000170715299850883	1081
PMS2	-0.122377479	5.48090844725103e-05	0.000170075324506888	1081
GRIK1	-0.122426097	5.44334333642667e-05	0.000168935682502412	1081
TSC2	-0.122432193	5.43865081685655e-05	0.000168842072284751	1081
DUSP18	-0.122468563	5.41073121434667e-05	0.00016802710083708	1081
SS18	-0.12254219	5.35462647433691e-05	0.00016641306745683	1081
BMX	-0.122633249	5.28599870154286e-05	0.000164356295481681	1081
UNC13B	-0.122647541	5.27530363244614e-05	0.00016404907637485	1081
EIF3E	-0.122681403	5.25004457901029e-05	0.000163314002538855	1081

DIDO1	-0.122775159	5.18070398615555e-05	0.000161256615885512	1081
CCDC6	-0.122794803	5.16628608624936e-05	0.00016085754746135	1081
NISCH	-0.122799449	5.16288160849334e-05	0.000160776395119954	1081
KLK4	-0.122800349	5.1622222836986e-05	0.000160776395119954	1081
ZNF484	-0.122810838	5.15454494892375e-05	0.00016059125888659	1081
PEX5	-0.122825799	5.14361313524153e-05	0.000160275462659614	1081
PIK3CA	-0.12285032	5.12574333899872e-05	0.000159743347097972	1081
ZNF462	-0.122855364	5.12207475557938e-05	0.000159678421465717	1081
ERLIN1	-0.122870568	5.11103175576548e-05	0.000159358821730221	1081
SEMA3D	-0.122871814	5.11012776655022e-05	0.000159355300088474	1081
ZRANB3	-0.122902367	5.0880077235619e-05	0.000158714641671035	1081
RAD18	-0.12291624	5.07799423921972e-05	0.000158426814231193	1081
RAD50	-0.122921522	5.07418644452543e-05	0.000158332537058496	1081
TLR5	-0.122960254	5.04634718121564e-05	0.000157537058679047	1081
GABRA4	-0.122962693	5.0445992125494e-05	0.000157506898848121	1081
UBQLN2	-0.122988556	5.02609672126521e-05	0.000156953524182123	1081
VPS41	-0.122991118	5.02426713434581e-05	0.00015692071538201	1081
SLIT3	-0.123075061	4.9646756630787e-05	0.000155131675558741	1081
NLGN3	-0.123109314	4.94055243917954e-05	0.000154425801221523	1081
SPRY3	-0.123123701	4.93045308441347e-05	0.000154134043118419	1081
STX19	-0.12314625	4.91466350177172e-05	0.000153711995409395	1081
GPR88	-0.123205718	4.87325144136793e-05	0.000152464125308832	1081
CNGA1	-0.123224961	4.85992203523393e-05	0.000152070719788424	1081
NARS2	-0.123252154	4.84114455363657e-05	0.000151530231561542	1081
WNT8A	-0.123253262	4.84038092487852e-05	0.000151529873708901	1081
ANUBL1	-0.123281507	4.82095289313218e-05	0.00015096859324133	1081
CEP57	-0.123321313	4.7936971397112e-05	0.000150185114898106	1081
ADAMTS[illegible]	-0.123349017	4.77481486747662e-05	0.000149640083237891	1081
PEAR1	-0.123358068	4.76866106986302e-05	0.000149470479620959	1081
PDLIM3	-0.123388008	4.74835819184693e-05	0.000148903604879758	1081
SYNJ2BP	-0.123456399	4.70228721206482e-05	0.000147527761854923	1081
TXLNG	-0.123464849	4.69662452462472e-05	0.00014737305459278	1081
SGK3	-0.123474794	4.68996823051782e-05	0.000147187116379158	1081

PHACTR4	-0.123523335	4.65760735890866e-05	0.00014621708001436	1081
UHRF1BP	-0.123553595	4.63754034775829e-05	0.000145655207835346	1081
CLDN16	-0.12356781	4.6281423187719e-05	0.000145405375875951	1081
C20orf103	-0.123585788	4.61628201284025e-05	0.000145055375368378	1081
TYW1	-0.123590382	4.61325585508713e-05	0.000144982900469158	1081
KCNMA1	-0.123611488	4.59937721138924e-05	0.000144569283700166	1081
MYO1B	-0.123615276	4.59689041702521e-05	0.000144513666434103	1081
C15orf17	-0.123638432	4.58171745649472e-05	0.000144104134521602	1081
HBS1L	-0.123641173	4.57992436236182e-05	0.000144070231542441	1081
HOXA13	-0.123649992	4.57416039114732e-05	0.000143933866103816	1081
MUC6	-0.123664312	4.56481492668464e-05	0.000143662235116485	1081
FAM13A	-0.123670249	4.5609462114428e-05	0.000143562908483617	1081
RAD17	-0.123732931	4.52028590299547e-05	0.000142394307296081	1081
THAP5	-0.123774237	4.49367945355374e-05	0.000141622610437797	1081
SERTAD2	-0.123787809	4.48496967796956e-05	0.000141370230265525	1081
BRWD1	-0.123801498	4.47620157842363e-05	0.000141144470565706	1081
VSIG2	-0.123805006	4.47395705610645e-05	0.000141119933728718	1081
FLT1	-0.123814382	4.46796361069438e-05	0.000140988607358143	1081
ING3	-0.123830371	4.45775964092956e-05	0.000140688654185377	1081
C2orf72	-0.123851476	4.44432548929389e-05	0.00014028771714722	1081
RASL11A	-0.123859684	4.43911085799299e-05	0.00014016596901923	1081
ARHGEF3	-0.123899811	4.4137018260146e-05	0.000139451103019235	1081
ASB7	-0.123914026	4.40473402660294e-05	0.000139189595240653	1081
ZNF629	-0.123915434	4.40384685279213e-05	0.000139183393237367	1081
NLRP5	-0.123948104	4.38330468530431e-05	0.000138555896572188	1081
ELTD1	-0.123971054	4.36892919507388e-05	0.000138123161699252	1081
PGBD2	-0.123973288	4.36753211541823e-05	0.000138100666245645	1081
SPAG9	-0.123982504	4.36177349479785e-05	0.000137940230851967	1081
C20orf177	-0.124023735	4.33609837902442e-05	0.000137171328274885	1081
COMMD2	-0.124049791	4.31994701529473e-05	0.000136703318603695	1081
CCDC52	-0.124081809	4.30017766558136e-05	0.000136120488801283	1081
BIVM	-0.124098527	4.28988991711136e-05	0.000135858878132697	1081
ZHX1	-0.124186359	4.23622001455466e-05	0.000134222479070783	1081

MTRF1	-0.124194532	4.23125844388768e-05	0.000134086363618243	1081
SLC44A3	-0.124208372	4.22286905387368e-05	0.000133862623273462	1081
LOC64282	-0.124240093	4.20369989594995e-05	0.000133275943348933	1081
TMEM63A	-0.124243268	4.20178602457303e-05	0.000133236234007593	1081
ALDH1L1	-0.124250499	4.19742970986334e-05	0.00013311905148803	1081
ZFYVE9	-0.124252276	4.19636010759086e-05	0.000133106084659767	1081
ZNF613	-0.124260883	4.19118161553423e-05	0.000132983703961155	1081
SOX5	-0.124276805	4.18161818798123e-05	0.000132701163196096	1081
LAMA3	-0.124296325	4.16992198572372e-05	0.000132361322773439	1081
LRRN4CL	-0.124315864	4.15824487984738e-05	0.00013204262784446	1081
UGCG	-0.124360447	4.1317179250525e-05	0.000131241654998711	1081
CDH12	-0.124375594	4.12274204280373e-05	0.000130977193585052	1081
C10orf90	-0.12438555	4.11685217653628e-05	0.000130810705199248	1081
C3orf70	-0.124387098	4.11593695312686e-05	0.000130802255751287	1081
RNF20	-0.124441181	4.08408809836006e-05	0.00012985156998337	1081
CCDC129	-0.124470295	4.06703971488624e-05	0.000129350355314782	1081
NR5A2	-0.124559424	4.01526729585928e-05	0.000127764270534015	1081
RPS23	-0.124563319	4.01301921098595e-05	0.000127712909960998	1081
LOC40146	-0.124592481	3.99622504451476e-05	0.000127258857922463	1081
DDX31	-0.124646305	3.96540216774467e-05	0.000126357207638748	1081
ITGB8	-0.124669224	3.95234607444777e-05	0.000125961100569135	1081
NAA30	-0.124675543	3.94875338676674e-05	0.00012586651420319	1081
TRO	-0.124683519	3.94422332537881e-05	0.000125761916571314	1081
NID1	-0.12468733	3.9420607830372e-05	0.000125712861285871	1081
IL1RL2	-0.124690092	3.94049362704254e-05	0.000125682780425541	1081
ZNF676	-0.124737972	3.91342684129029e-05	0.000124866169523447	1081
FMO2	-0.124746827	3.90844017010592e-05	0.000124739428432801	1081
GEMIN4	-0.12475564	3.90348387380148e-05	0.000124600986591239	1081
NT5DC3	-0.124772006	3.89429436152306e-05	0.000124327353269227	1081
CILP	-0.124827963	3.86303045141228e-05	0.000123368339321022	1081
OR2W3	-0.124841925	3.85526691648443e-05	0.000123139927116821	1081
PCDHB5	-0.124865204	3.84235546568489e-05	0.000122746988354301	1081
AMAC1L3	-0.124870571	3.83938436391155e-05	0.000122671527376682	1081

CCDC8	-0.124872321	3.83841612162035e-05	0.000122660045637757	1081
PPP3CB	-0.124896639	3.82498533419714e-05	0.000122289048654819	1081
LRRC17	-0.1249529	3.79408270830645e-05	0.000121320311363228	1081
FAM43B	-0.124955148	3.79285280002117e-05	0.000121300237587595	1081
PAK7	-0.124974209	3.78244020311749e-05	0.000121005650137846	1081
CTH	-0.12498439	3.77688928487989e-05	0.000120847259599596	1081
GPR20	-0.124988462	3.7746714219235e-05	0.00012079548180246	1081
TTPA	-0.125024327	3.75519062234306e-05	0.000120191158384336	1081
FRYL	-0.125027861	3.7532760063917e-05	0.0001201489673427	1081
NPY5R	-0.125064349	3.73356406488979e-05	0.00011955594990813	1081
GNRHR2	-0.125093313	3.71798648715437e-05	0.000119076053710214	1081
ANKRD13	-0.125111648	3.70815754654986e-05	0.00011879903590211	1081
TGDS	-0.125112518	3.70769187222179e-05	0.00011879903590211	1081
PRDM4	-0.125117621	3.70496061087222e-05	0.000118734380378652	1081
PBOV1	-0.125140151	3.69292673740883e-05	0.000118386392624285	1081
CASQ2	-0.125164777	3.67981479680534e-05	0.00011798483062493	1081
STAC2	-0.125176412	3.67363538530669e-05	0.000117824207669166	1081
LTBP4	-0.125201132	3.66053948014713e-05	0.000117441579594782	1081
POFUT1	-0.125226514	3.64713838836698e-05	0.000117030268929042	1081
TMEM170	-0.125262482	3.62822796950812e-05	0.000116460567950512	1081
TMTC1	-0.12528675	3.6155214138956e-05	0.000116089701757933	1081
IL20RA	-0.125313509	3.60155952797848e-05	0.000115678279163148	1081
SEC22C	-0.125320632	3.59785168401352e-05	0.000115577728801622	1081
ZBTB5	-0.125339809	3.58788722376132e-05	0.000115312680476503	1081
GAB2	-0.125400399	3.55657564982737e-05	0.000114397599338611	1081
HACL1	-0.125407603	3.5528700655669e-05	0.000114296658369283	1081
PCDHGA9	-0.125447015	3.53266210285368e-05	0.000113691648883113	1081
ZFP62	-0.125457946	3.52707629722513e-05	0.00011353939278939	1081
MLANA	-0.125479909	3.51587952597573e-05	0.00011319704818728	1081
HEPACAM	-0.125492406	3.50952309950425e-05	0.000113010458503058	1081
ELF1	-0.125500854	3.50523286460676e-05	0.000112890353409278	1081
OR2L3	-0.125509476	3.50085903262633e-05	0.00011276751712865	1081
PRKCE	-0.125512997	3.49907430739871e-05	0.000112728053610342	1081

FHDC1	-0.125527572	3.49169577070916e-05	0.000112508335414165	1081
GPR116	-0.125542327	3.48424150194143e-05	0.000112286106313566	1081
PXK	-0.125550662	3.48003720398537e-05	0.000112168559158857	1081
C1QTNF3	-0.125585141	3.4626965390121e-05	0.000111627495244677	1081
FAM189A	-0.125587383	3.46157170538269e-05	0.000111609094118013	1081
TMEM215	-0.125594034	3.45823697276757e-05	0.00011151942342949	1081
SIN3A	-0.125628498	3.44100682953909e-05	0.000110981560328314	1081
SETDB1	-0.125633455	3.43853529897612e-05	0.000110937369631444	1081
EIF3D	-0.125634252	3.43813834055205e-05	0.000110937369631444	1081
TTN	-0.125634695	3.43791762612826e-05	0.000110937369631444	1081
LOC13446	-0.125672251	3.4192502885389e-05	0.000110385892728552	1081
RASGRF2	-0.125706679	3.40222225821548e-05	0.000109871380878087	1081
FZD3	-0.125788123	3.36225986374705e-05	0.000108667936716163	1081
SCARA5	-0.12583153	3.34114309985933e-05	0.000108037444216158	1081
CCDC160	-0.125832811	3.34052185395123e-05	0.000108034696978404	1081
LMOD1	-0.125855243	3.3296606471548e-05	0.000107735325629511	1081
SOX13	-0.125926405	3.29542594091581e-05	0.000106661882358208	1081
ANKHD1-	-0.12593554	3.29105552882676e-05	0.000106537543995203	1081
ATP13A3	-0.125991813	3.26425428352528e-05	0.000105703910209961	1081
SPATA18	-0.12601523	3.25316250789084e-05	0.000105378611869209	1081
POLR2B	-0.126027179	3.24751647181877e-05	0.000105212639634592	1081
SHF	-0.126053162	3.23527171826658e-05	0.000104849660174518	1081
EVC2	-0.126092433	3.21684699179904e-05	0.000104286100176684	1081
PDIK1L	-0.126093349	3.21641863213788e-05	0.000104286100176684	1081
UEVLD	-0.126100571	3.21304150320843e-05	0.000104196267034987	1081
SLC35A5	-0.126106975	3.21004985908392e-05	0.000104116010966423	1081
LGALS8	-0.126110712	3.20830523715158e-05	0.000104076181968468	1081
FAIM2	-0.126121516	3.20326667251259e-05	0.000103929468703118	1081
GPR161	-0.126171499	3.18005407537005e-05	0.000103219876378577	1081
DPP6	-0.126178848	3.17665478256307e-05	0.000103132490885952	1081
FNDC8	-0.126181002	3.17565891453104e-05	0.000103116777616421	1081
ADH1B	-0.1262049	3.16463168345924e-05	0.000102825008489172	1081
EEF1DP3	-0.126269566	3.13497404468731e-05	0.00010194358697373	1081

LOC10012	-0.126285793	3.12757320559211e-05	0.000101735769783067	1081
SEC23A	-0.12630332	3.1195984007216e-05	0.00010149274835681	1081
MPZL2	-0.126310462	3.1163543320394e-05	0.000101403582650515	1081
GEM	-0.126315793	3.11393488463934e-05	0.000101341224961324	1081
AHCYL2	-0.126332938	3.10616571647887e-05	0.00010110471539581	1081
CTNNA1	-0.126351083	3.09796377083211e-05	0.00010085404034165	1081
KIAA121(	-0.126450716	3.05329104453584e-05	9.94801036587004e-05	1081
ZNF329	-0.126454109	3.05178056884132e-05	9.94526574432168e-05	1081
MICALCL	-0.126476765	3.04171261429562e-05	9.91509718689083e-05	1081
TMEM90I	-0.126508316	3.02774473458796e-05	9.87276103565467e-05	1081
TDRD9	-0.126542265	3.01278316859314e-05	9.82874768118361e-05	1081
GNL3L	-0.126544794	3.01167140483839e-05	9.82671209110291e-05	1081
KIAA1024	-0.126549149	3.0097576510044e-05	9.82205862295214e-05	1081
OR2A1	-0.126559219	3.00533722625614e-05	9.80922203871192e-05	1081
CLGN	-0.126599122	2.98788242531557e-05	9.75541190566973e-05	1081
AUH	-0.126636535	2.9716041286367e-05	9.70698316383758e-05	1081
NTRK2	-0.126660379	2.96127338985243e-05	9.6763750914156e-05	1081
ZNF540	-0.12666869	2.95768088546268e-05	9.66620399702234e-05	1081
KCNA6	-0.126702556	2.94308341460084e-05	9.62161885542581e-05	1081
WDFY2	-0.126711862	2.93908419424506e-05	9.61166413848486e-05	1081
MRPL30	-0.126734411	2.9294157253438e-05	9.58315683453244e-05	1081
QRFPR	-0.126747042	2.92401270464596e-05	9.56703523389521e-05	1081
PTPRK	-0.126767538	2.91526574317798e-05	9.53996562643282e-05	1081
RFXAP	-0.126844114	2.88280519739601e-05	9.43834076085528e-05	1081
FGF9	-0.126891219	2.86300766280521e-05	9.37809583206682e-05	1081
PCMTD2	-0.126894421	2.86166673958697e-05	9.37675284140852e-05	1081
FOXJ3	-0.126901764	2.85859393949686e-05	9.36820805452485e-05	1081
FAM110B	-0.126907053	2.85638225184474e-05	9.36248299111816e-05	1081
MLXIP	-0.126941467	2.84203221401272e-05	9.31696321420442e-05	1081
C20orf117	-0.12696351	2.83287626245033e-05	9.28997107391535e-05	1081
LOC16847	-0.12696532	2.83212601374239e-05	9.28902288291119e-05	1081
SYNCRIP	-0.127022233	2.8086223426211e-05	9.21470141697098e-05	1081
MTF1	-0.127026953	2.80668139330041e-05	9.21156674291898e-05	1081

LOC65125	-0.127033134	2.80414141551211e-05	9.20473013125167e-05	1081
CEP170L	-0.12703483	2.80344500535081e-05	9.20394387757366e-05	1081
PRKCI	-0.12706087	2.79277174769452e-05	9.17297029143695e-05	1081
CRB1	-0.12706785	2.78991700404326e-05	9.16550522610101e-05	1081
ZSCAN22	-0.127143962	2.75897088856203e-05	9.06827680699659e-05	1081
C3orf64	-0.127173983	2.74685471242149e-05	9.02992626986469e-05	1081
POLR1A	-0.127177603	2.74539712410324e-05	9.0266076489407e-05	1081
COL6A3	-0.127238061	2.72116176928302e-05	8.94851084923018e-05	1081
ERP27	-0.127244456	2.7186102103655e-05	8.94437411200604e-05	1081
FANCM	-0.127306735	2.69387974482964e-05	8.86590548269779e-05	1081
ASAM	-0.127307281	2.69366404661205e-05	8.86590548269779e-05	1081
MLH3	-0.127334152	2.68306069915872e-05	8.83462860159404e-05	1081
ALAD	-0.127349149	2.67715981386759e-05	8.81663960280571e-05	1081
INPP5F	-0.127382257	2.66417724054193e-05	8.77962547206234e-05	1081
TNPO3	-0.127398743	2.65773459639018e-05	8.7626945080655e-05	1081
TTC23	-0.127399662	2.65737578754125e-05	8.7626945080655e-05	1081
GEMIN5	-0.127415146	2.65133923804703e-05	8.7444710134999e-05	1081
PPP1CB	-0.127438964	2.64207921988618e-05	8.71535711226576e-05	1081
LOX	-0.127481554	2.62559657879184e-05	8.66240469698028e-05	1081
TMEM192	-0.127489011	2.62272081013823e-05	8.65433427030869e-05	1081
GTF3C3	-0.12750649	2.61599201684593e-05	8.63637482454299e-05	1081
RCAN3	-0.127516814	2.6120251553681e-05	8.62610602539186e-05	1081
EDNRB	-0.127523865	2.60931919531399e-05	8.61858258560424e-05	1081
RHOBTB2	-0.127535044	2.60503465131258e-05	8.60584175970678e-05	1081
FUT11	-0.12754832	2.5999548266174e-05	8.59187827792117e-05	1081
HMGXB3	-0.127570058	2.59165759968363e-05	8.56726983026367e-05	1081
RALGPS1	-0.127609113	2.57681371403314e-05	8.52099676119462e-05	1081
SUDS3	-0.127742463	2.52673739882667e-05	8.36089436586124e-05	1081
LOC72908	-0.127823122	2.4968977207913e-05	8.26894699742574e-05	1081
MTAP	-0.127856418	2.48467810463107e-05	8.23118572895788e-05	1081
PPFIA2	-0.127876456	2.47735140386909e-05	8.20826382087874e-05	1081
SCYL2	-0.127935997	2.45570169773255e-05	8.13786983069948e-05	1081
DDX50	-0.127939589	2.45440129915792e-05	8.13489867909448e-05	1081

NEFL	-0.127942429	2.45337365084464e-05	8.13283070532585e-05	1081
NFYA	-0.127948208	2.45128388008372e-05	8.127240580034e-05	1081
SEMA6C	-0.127950184	2.4505698843472e-05	8.12621075229207e-05	1081
C10orf79	-0.127958288	2.44764264805442e-05	8.11784016217587e-05	1081
ZNF320	-0.127959931	2.44704987307954e-05	8.11721055379341e-05	1081
RP2	-0.127965473	2.44505069275995e-05	8.11191472425053e-05	1081
FIG4	-0.127998967	2.43300096664126e-05	8.07459711251866e-05	1081
PGM2	-0.128031277	2.42143051134983e-05	8.03884602029372e-05	1081
HAND2	-0.128094636	2.39889303022249e-05	7.96533708485777e-05	1081
C1orf150	-0.128126081	2.38778203638857e-05	7.92975092697787e-05	1081
MSX2	-0.128182876	2.36783710660823e-05	7.86481096663195e-05	1081
EXTL2	-0.128192267	2.36455464251597e-05	7.85520337623419e-05	1081
TRAF3IP1	-0.128251535	2.34393744724797e-05	7.7892807447724e-05	1081
PER1	-0.128255417	2.34259281162943e-05	7.78609671510888e-05	1081
ALG10	-0.128263935	2.33964540005442e-05	7.77886723619347e-05	1081
ATP11C	-0.12827995	2.33411347746064e-05	7.76431902302587e-05	1081
KIAA1033	-0.12833748	2.31434332605911e-05	7.7036428128653e-05	1081
HHAT	-0.128410479	2.28948626836705e-05	7.62342163243871e-05	1081
CBX7	-0.128419682	2.28637062394224e-05	7.61430587193197e-05	1081
TPM4	-0.128441433	2.27902291200942e-05	7.59109070145994e-05	1081
ACAD10	-0.128445827	2.27754144210994e-05	7.58741067493051e-05	1081
LRRC55	-0.128468983	2.26974831493784e-05	7.56269927297762e-05	1081
ZNF83	-0.128495968	2.26069893858697e-05	7.53379323702804e-05	1081
AFAP1L2	-0.128501545	2.2588328573331e-05	7.52881997203431e-05	1081
DPYSL2	-0.128523866	2.25137936563812e-05	7.50646099317775e-05	1081
C14orf21	-0.128550281	2.24258883196573e-05	7.48086637190755e-05	1081
AASDHPI	-0.128569692	2.23614962027217e-05	7.46062174567454e-05	1081
USP33	-0.128598747	2.2265444487554e-05	7.43226808950747e-05	1081
ZNF582	-0.1286115	2.22234096822222e-05	7.41946615923708e-05	1081
KLHL29	-0.128621423	2.21907535446471e-05	7.40979164854825e-05	1081
ADAMTS	-0.128655547	2.20788060619886e-05	7.37485571412303e-05	1081
AMMECR	-0.128725342	2.18515004582152e-05	7.30256265313113e-05	1081
TBC1D2B	-0.128764642	2.17244890391638e-05	7.26252624782533e-05	1081

USP37	-0.128768758	2.17112273458494e-05	7.25929750841719e-05	1081
CDH5	-0.128775817	2.16885027575036e-05	7.2529031880795e-05	1081
SMARCC2	-0.128788003	2.16493221235265e-05	7.24340797506129e-05	1081
ELMOD1	-0.128838389	2.14880443121088e-05	7.19422723396099e-05	1081
MED20	-0.128866451	2.13987156460443e-05	7.16551074949405e-05	1081
C6orf204	-0.128914762	2.12457585579795e-05	7.11665790073988e-05	1081
ZNF132	-0.128921482	2.12245667143116e-05	7.11074166738412e-05	1081
DHFRL1	-0.128985607	2.10233352940118e-05	7.04566776739091e-05	1081
CCKAR	-0.129018726	2.09201166920123e-05	7.01340906574451e-05	1081
KIAA1310	-0.129059481	2.07937618842717e-05	6.97220927361273e-05	1081
CAB39	-0.12908392	2.07183384170415e-05	6.95039013174523e-05	1081
PTGS2	-0.12909765	2.06760797337652e-05	6.93782538723613e-05	1081
FMOD	-0.129117881	2.06139640572235e-05	6.92113843221279e-05	1081
ERCC4	-0.129190204	2.03933469995654e-05	6.85392017527936e-05	1081
DCLK2	-0.129197773	2.03703904474875e-05	6.84734716443576e-05	1081
BMP8B	-0.129204288	2.03506482335709e-05	6.84185261457421e-05	1081
LOC72826	-0.129212627	2.03254057462222e-05	6.83564772550326e-05	1081
CSDAP1	-0.129256449	2.01932441193497e-05	6.7934686503724e-05	1081
TMEM161	-0.129266353	2.01634891507943e-05	6.78459143047857e-05	1081
FAM189A	-0.129282511	2.01150345898146e-05	6.77143724563828e-05	1081
STX7	-0.12934458	1.99299233411459e-05	6.71048480206224e-05	1081
GOLGA8C	-0.129370309	1.98536697927499e-05	6.6870452762907e-05	1081
TGFBR2	-0.129372636	1.98467865741688e-05	6.68584474141521e-05	1081
DNAJB4	-0.129413545	1.97261510838336e-05	6.64742913321895e-05	1081
ARMCX3	-0.129473733	1.95499292542101e-05	6.59135271675419e-05	1081
LRRC48	-0.129494243	1.94902214306295e-05	6.57342224543834e-05	1081
BBS1	-0.129496716	1.94830349507113e-05	6.57209877900332e-05	1081
BOC	-0.129500682	1.94715141954706e-05	6.5693125685439e-05	1081
PRINS	-0.129525373	1.93999310638078e-05	6.54625814540046e-05	1081
ARPP19	-0.129592159	1.92075596468846e-05	6.4835169082857e-05	1081
CBFA2T2	-0.129729562	1.88174742506528e-05	6.35503803486004e-05	1081
TNXB	-0.129730106	1.88159452334939e-05	6.35503803486004e-05	1081
MTO1	-0.129737392	1.87954722419492e-05	6.35080154837416e-05	1081

LOC40109	-0.129751308	1.87564336484854e-05	6.33867397833819e-05	1081
SCAND3	-0.1298161	1.85756750445809e-05	6.27864049954834e-05	1081
DET1	-0.129822685	1.85573969089556e-05	6.27456798809853e-05	1081
USP3	-0.129866622	1.84358779240637e-05	6.23557355238856e-05	1081
ZNF134	-0.129867182	1.84343332009364e-05	6.23557355238856e-05	1081
EFCAB1	-0.129877388	1.84062176454752e-05	6.22763275895362e-05	1081
DCAF8	-0.129880611	1.83973470970181e-05	6.22567709170888e-05	1081
PPIP5K1	-0.129888477	1.83757152229293e-05	6.22044670082188e-05	1081
C7orf42	-0.129892921	1.83635036144202e-05	6.21735765231085e-05	1081
YES1	-0.129903998	1.83331000643093e-05	6.20915098849212e-05	1081
RNPC3	-0.12996587	1.8164161881183e-05	6.15400338204559e-05	1081
KIF1C	-0.129987257	1.81061105141694e-05	6.13536747364076e-05	1081
PAQR3	-0.130041967	1.79584132977706e-05	6.08839171800049e-05	1081
HYDIN	-0.130044201	1.79524068024012e-05	6.08737981879099e-05	1081
BDNFOS	-0.130076073	1.78669202977224e-05	6.06043289101898e-05	1081
ITPKB	-0.130088743	1.78330458649165e-05	6.04996141712263e-05	1081
WDR6	-0.130100651	1.78012620575651e-05	6.04019579163971e-05	1081
INO80	-0.130111859	1.77713979964351e-05	6.03107838002334e-05	1081
HTR7P1	-0.130117074	1.77575166835896e-05	6.02738287432033e-05	1081
COBL	-0.130128872	1.77261561036434e-05	6.01775218584253e-05	1081
ADH1A	-0.130145038	1.76832663381198e-05	6.00420361337304e-05	1081
DLGAP2	-0.13018538	1.75766711260902e-05	5.96901618063193e-05	1081
FAM124B	-0.130189589	1.75655856636931e-05	5.96625734606471e-05	1081
DISP1	-0.130190794	1.75624127399717e-05	5.96618557583018e-05	1081
FAM78B	-0.130283349	1.73203445454164e-05	5.88494418734043e-05	1081
ZNF571	-0.13028866	1.73065502270726e-05	5.88124922949354e-05	1081
TBX15	-0.130290424	1.73019731907248e-05	5.88124922949354e-05	1081
ABO	-0.13033319	1.71913110584087e-05	5.84504575985895e-05	1081
CEP97	-0.130339547	1.71749175765505e-05	5.84160670074722e-05	1081
PLAGL1	-0.130361703	1.71179050027788e-05	5.82401952847458e-05	1081
TSPAN7	-0.130385443	1.70570126112508e-05	5.8052630352027e-05	1081
UBE2H	-0.130396648	1.70283457661818e-05	5.79648572929592e-05	1081
FAM84B	-0.130421023	1.69661400760613e-05	5.77628683170955e-05	1081

C9orf144E	-0.130425323	1.69551899591117e-05	5.77353451193891e-05	1081
SAMD8	-0.130425651	1.69543534473578e-05	5.77353451193891e-05	1081
TIAM1	-0.130468372	1.68459233533528e-05	5.74117959657068e-05	1081
NHLRC3	-0.130521657	1.67116022666943e-05	5.69732996551966e-05	1081
NRXN1	-0.130533098	1.66828959430914e-05	5.68850607267393e-05	1081
NCRNA00	-0.130542153	1.66602093737045e-05	5.68173214547615e-05	1081
NUDT10	-0.130555351	1.66271943718595e-05	5.67235170657437e-05	1081
PPP1R15E	-0.130573096	1.65829058063513e-05	5.65824250455534e-05	1081
ADIPOQ	-0.130588057	1.65456504710759e-05	5.64648701913981e-05	1081
WDR47	-0.130659409	1.63690688274306e-05	5.59001341801303e-05	1081
FAT2	-0.130704955	1.62572932401682e-05	5.55372515386107e-05	1081
ZNF140	-0.13072523	1.62077689552361e-05	5.53774602447815e-05	1081
ITGB1	-0.130725288	1.62076284205481e-05	5.53774602447815e-05	1081
SRPX	-0.130734706	1.6184671442853e-05	5.53266937410952e-05	1081
ZNF224	-0.130775278	1.60861342073952e-05	5.50178563001657e-05	1081
ZSCAN23	-0.130782728	1.6068102415156e-05	5.49655159030936e-05	1081
GRIN2B	-0.130783771	1.606558147877e-05	5.49655159030936e-05	1081
STH	-0.130801308	1.60232174595019e-05	5.48305955022363e-05	1081
CBR4	-0.130806939	1.60096375551491e-05	5.47934333245803e-05	1081
SLC35A1	-0.13080707	1.60093204303268e-05	5.47934333245803e-05	1081
C2orf88	-0.130808969	1.60047435626662e-05	5.47934333245803e-05	1081
KIAA010(	-0.130822658	1.59717856988111e-05	5.46917597998556e-05	1081
BMP3	-0.130852478	1.59002146123292e-05	5.44651969674159e-05	1081
DENND5E	-0.130900625	1.57853000999948e-05	5.40991613668587e-05	1081
ITSN1	-0.130903701	1.57779856155322e-05	5.40832942359869e-05	1081
ZNF468	-0.130906839	1.57705266765515e-05	5.40669264634328e-05	1081
HTR7	-0.130962151	1.56396028746188e-05	5.36363295725562e-05	1081
AQP1	-0.130963291	1.56369160720587e-05	5.36362462577257e-05	1081
CAB39L	-0.13096422	1.56347258711562e-05	5.36362462577257e-05	1081
ZNRF2	-0.131006694	1.55349230362225e-05	5.33227167430059e-05	1081
ZNF550	-0.131008702	1.55302197314475e-05	5.33156572068864e-05	1081
RGNEF	-0.131031217	1.54775767149372e-05	5.31439889078592e-05	1081
ANGPTL7	-0.131064291	1.54005548543275e-05	5.28975579779075e-05	1081

RNF19A	-0.131086041	1.53501022695909e-05	5.27332554946981e-05	1081
LOC15116	-0.13109676	1.53252939177596e-05	5.26570093763033e-05	1081
ST13	-0.131149692	1.52033545044173e-05	5.22736945709996e-05	1081
AGAP11	-0.131150622	1.52012213518358e-05	5.22736945709996e-05	1081
BEND3	-0.131162552	1.51738692668826e-05	5.21990430979082e-05	1081
HPSE2	-0.131171688	1.51529546842471e-05	5.21538137902199e-05	1081
PDP1	-0.131195308	1.50990112511966e-05	5.19948002829666e-05	1081
PGBD3	-0.131261653	1.49484651826858e-05	5.15027930742611e-05	1081
ASPN	-0.13126325	1.49448590485643e-05	5.14991764511336e-05	1081
SHANK3	-0.131285366	1.48950067534808e-05	5.13361695549821e-05	1081
SCN2A	-0.131315161	1.48280919930629e-05	5.11142904175655e-05	1081
TUG1	-0.131333273	1.47875557026081e-05	5.09832807853911e-05	1081
PMPCB	-0.131373799	1.46972389121771e-05	5.06892446303387e-05	1081
RPS6KA6	-0.131402516	1.46335563999575e-05	5.0504196278421e-05	1081
LOC44145	-0.131410452	1.46160045829131e-05	5.04522639346788e-05	1081
FOXD4L2	-0.131420305	1.45942407735998e-05	5.03944087048626e-05	1081
ARAP3	-0.13142371	1.45867254181066e-05	5.03770930135022e-05	1081
LOC64698	-0.131481443	1.44598813990223e-05	4.99647188307556e-05	1081
SMCR5	-0.13148488	1.445236187478e-05	4.99473031338897e-05	1081
ABCA10	-0.131559586	1.42898652612864e-05	4.94026661555887e-05	1081
IRS2	-0.131566935	1.4273975224361e-05	4.93562016640494e-05	1081
UBR2	-0.131590735	1.42226280504409e-05	4.92039914264351e-05	1081
ZNF324B	-0.131613417	1.41738553536247e-05	4.90521072150439e-05	1081
FIBIN	-0.131719101	1.39487008294311e-05	4.83310248037306e-05	1081
LOC40002	-0.131728293	1.39292811370842e-05	4.82720399976882e-05	1081
SOCS4	-0.131834206	1.37073525793735e-05	4.75274729279655e-05	1081
IQCK	-0.131842898	1.36892899688767e-05	4.74730153938752e-05	1081
SMYD1	-0.131872058	1.36288593965148e-05	4.72878698833604e-05	1081
TPO	-0.131937677	1.34937990368465e-05	4.68838533282636e-05	1081
HCG18	-0.131975795	1.34159280792232e-05	4.66293773560992e-05	1081
COL28A1	-0.132087347	1.31904929985985e-05	4.58695807796938e-05	1081
MED28	-0.132088031	1.31891223202651e-05	4.58695807796938e-05	1081
PCDHGA4	-0.132099998	1.31651556802866e-05	4.58051919135361e-05	1081

HGF	-0.132109431	1.31462933951289e-05	4.574746596042e-05	1081
DNAJC13	-0.1321149	1.31353709961155e-05	4.571735465044e-05	1081
SPOCK3	-0.132119926	1.31253390219087e-05	4.5690332572378e-05	1081
SORCS1	-0.132124766	1.31156846825201e-05	4.56725095815672e-05	1081
RFC1	-0.132126523	1.31121838070978e-05	4.56682127928745e-05	1081
CCL14	-0.132136263	1.30927813493371e-05	4.56085215774505e-05	1081
HCN1	-0.132139437	1.30864665873502e-05	4.55944084057712e-05	1081
LPPR5	-0.132187933	1.29903190852912e-05	4.52907542355818e-05	1081
ZNF311	-0.132208408	1.29499279507329e-05	4.51577459871064e-05	1081
IFT140	-0.13226765	1.28337323859643e-05	4.47758120739956e-05	1081
PTGFR	-0.132281455	1.28068008838211e-05	4.46895901272434e-05	1081
ACTR8	-0.132282602	1.28045647318576e-05	4.46895281571848e-05	1081
KDM4C	-0.132288418	1.27932352730326e-05	4.46577238910488e-05	1081
ZNF271	-0.132296455	1.27775953956851e-05	4.46108594880548e-05	1081
NNAT	-0.132316622	1.27384335010432e-05	4.44818413725976e-05	1081
VIPR1	-0.132324279	1.27235933183148e-05	4.44377231965069e-05	1081
C18orf25	-0.132342079	1.2689159051629e-05	4.43251446324025e-05	1081
TP53BP2	-0.132344886	1.26837371534504e-05	4.43138891703535e-05	1081
ARL6	-0.13237087	1.26336527184455e-05	4.41542217232972e-05	1081
ICK	-0.132431306	1.25178872104544e-05	4.37572163551281e-05	1081
GUCY1B3	-0.13243966	1.25019653179401e-05	4.37091446251133e-05	1081
ZNF501	-0.132499263	1.23889230459085e-05	4.33289678402476e-05	1081
ZNF167	-0.132532598	1.23261266111947e-05	4.31467976685522e-05	1081
CSPP1	-0.132585692	1.22267328771606e-05	4.2821198506676e-05	1081
TBC1D8	-0.132590635	1.22175190658218e-05	4.27963696019786e-05	1081
ZNF781	-0.132594776	1.22098037929205e-05	4.27767821579799e-05	1081
DSC3	-0.132599395	1.22012053843054e-05	4.27540933148083e-05	1081
RNF216	-0.132628297	1.21475282729152e-05	4.25734093698464e-05	1081
NBPF9	-0.13266042	1.20881337321297e-05	4.23726211995394e-05	1081
ST6GALN	-0.132673369	1.20642689366796e-05	4.22963274851045e-05	1081
EIF5	-0.132674967	1.20613280044216e-05	4.22933773105435e-05	1081
RTF1	-0.132689901	1.20338667690346e-05	4.22044300247567e-05	1081
LRCH2	-0.132699191	1.20168146713057e-05	4.21519644007407e-05	1081

DCHS2	-0.13272969	1.19609952630645e-05	4.19634708419427e-05	1081
HOOK1	-0.132773895	1.18805285670349e-05	4.16884250100887e-05	1081
CD164	-0.13280294	1.18279392252573e-05	4.15255900475441e-05	1081
NUP50	-0.132805509	1.18232969986931e-05	4.15165274601136e-05	1081
CORO2A	-0.132836302	1.17678069915704e-05	4.13288828181983e-05	1081
SPEF2	-0.132867252	1.17122816441944e-05	4.11697633436219e-05	1081
ZNF652	-0.132868243	1.17105085545299e-05	4.11697633436219e-05	1081
C12orf29	-0.132908227	1.16391571255633e-05	4.09412991608996e-05	1081
MAP2K5	-0.132942759	1.15778683238081e-05	4.07399401542556e-05	1081
PDGFC	-0.132944814	1.15742302917071e-05	4.07342538830259e-05	1081
ANKRD1(	-0.132992266	1.14905380846662e-05	4.04679877125177e-05	1081
EMCN	-0.133008648	1.14617783949663e-05	4.03737586582611e-05	1081
USP45	-0.133076775	1.13429135296803e-05	3.9962048453202e-05	1081
UBTD2	-0.133133967	1.12440350105282e-05	3.96206201306786e-05	1081
FGD5	-0.133138935	1.1235483489839e-05	3.95974133839761e-05	1081
EDA	-0.133143769	1.12271709201874e-05	3.95750408026555e-05	1081
ZNF238	-0.133156694	1.12049707500468e-05	3.95036989428935e-05	1081
FAM184B	-0.133182969	1.11599690040813e-05	3.93588192554651e-05	1081
HSD17B4	-0.133210962	1.1112216491233e-05	3.91972686422498e-05	1081
MME	-0.133229365	1.1080928328266e-05	3.91005957563353e-05	1081
RBBP9	-0.133246686	1.10515570118924e-05	3.90037869664634e-05	1081
OR7D2	-0.133258483	1.10315951350808e-05	3.89469828244311e-05	1081
FRG1B	-0.133258783	1.10310870346424e-05	3.89469828244311e-05	1081
QKI	-0.133268413	1.10148181298847e-05	3.89013869611725e-05	1081
C9orf152	-0.133287116	1.09832874258635e-05	3.87968306494862e-05	1081
WDR26	-0.13329598	1.09683741408096e-05	3.87509465216782e-05	1081
ZBTB10	-0.133303531	1.09556852066692e-05	3.87129062424751e-05	1081
RBM12	-0.133366469	1.08504620086874e-05	3.83478170464926e-05	1081
SV2B	-0.133385748	1.08184223398158e-05	3.82480024632485e-05	1081
ZNF554	-0.133429456	1.0746120586823e-05	3.80057231779404e-05	1081
LOC10019	-0.133435237	1.0736592556623e-05	3.79853630230366e-05	1081
SALL2	-0.133435981	1.0735366649498e-05	3.79853630230366e-05	1081
RPL26	-0.133443737	1.07225977471126e-05	3.79491798340801e-05	1081

N6AMT1	-0.133447678	1.07161141837894e-05	3.793289759839e-05	1081
KCNH8	-0.133518963	1.05994943100246e-05	3.75464766793468e-05	1081
RALGPS2	-0.133535608	1.05724376650924e-05	3.74572206759211e-05	1081
AGAP5	-0.133546463	1.05548293936067e-05	3.74014139198252e-05	1081
ZAK	-0.133549149	1.05504767135676e-05	3.73925674515868e-05	1081
WIF1	-0.133587269	1.04888827211381e-05	3.71808098569994e-05	1081
GPIHBP1	-0.133618842	1.04381280702587e-05	3.70139218404085e-05	1081
PABPC3	-0.133650208	1.03879392825452e-05	3.68424360645903e-05	1081
NEK4	-0.133667841	1.03598247125319e-05	3.67491933146602e-05	1081
LRRC70	-0.1336948	1.031698113386e-05	3.661010832158e-05	1081
CDH6	-0.133697153	1.03132502120487e-05	3.66033166881117e-05	1081
HHIP	-0.133758196	1.02168970057713e-05	3.62933151439363e-05	1081
HTR2A	-0.133773958	1.01921577407935e-05	3.62182073890076e-05	1081
RORA	-0.133789842	1.0167284765802e-05	3.61361947083771e-05	1081
EPHA5	-0.133812859	1.0131344323659e-05	3.60211668549437e-05	1081
GTF2H1	-0.133826447	1.01101834303311e-05	3.59522762937371e-05	1081
SLC25A21	-0.13387553	1.00340974775565e-05	3.56880108907797e-05	1081
EPB41L2	-0.133879744	1.00275913405441e-05	3.56711685599966e-05	1081
ZNF737	-0.133904351	9.98967650706921e-06	3.55425703346714e-05	1081
THSD4	-0.133954915	9.91219555047837e-06	3.52793603117291e-05	1081
ITGA7	-0.13396211	9.90121636694983e-06	3.52465106400785e-05	1081
PRSS12	-0.13398203	9.87088152259409e-06	3.51633790048909e-05	1081
ZNF28	-0.134014082	9.82225890559106e-06	3.49963575615727e-05	1081
MDM1	-0.134034662	9.79115872829564e-06	3.48978932380601e-05	1081
LOC44035	-0.134048913	9.76967934923313e-06	3.48336620336817e-05	1081
PODXL	-0.134059197	9.75420568109161e-06	3.47846474500957e-05	1081
MAGEL2	-0.134060908	9.75163312364679e-06	3.47816305375114e-05	1081
SLC25A26	-0.13408557	9.71463166542497e-06	3.46619296670184e-05	1081
SLC28A3	-0.134096719	9.69794923743595e-06	3.46085362955088e-05	1081
NUBPL	-0.134150726	9.61751938059224e-06	3.43397603548442e-05	1081
MIOS	-0.134190535	9.55864140459088e-06	3.41416367190573e-05	1081
IQCE	-0.134202852	9.54049484717719e-06	3.40889089564357e-05	1081
ANKRD43	-0.134231496	9.49841974173824e-06	3.39549933836017e-05	1081

TES	-0.134275028	9.43481423162235e-06	3.37412271784187e-05	1081
FAM119B	-0.134287146	9.41718102011154e-06	3.36841462446994e-05	1081
FGF20	-0.134332268	9.35179732622318e-06	3.34740506639598e-05	1081
KIAA0586	-0.134350069	9.32612310649728e-06	3.33880842332304e-05	1081
BICD1	-0.134393801	9.26333258522379e-06	3.31750817652148e-05	1081
NUFIP1	-0.13441257	9.23650843936492e-06	3.30848973170353e-05	1081
MYB	-0.134420785	9.22479018499552e-06	3.30487992667143e-05	1081
ABCA6	-0.134485562	9.13289139887053e-06	3.27253819335195e-05	1081
ATP7B	-0.134522154	9.08136425409848e-06	3.25697138862051e-05	1081
RBM12B	-0.134533884	9.06490537266818e-06	3.25164741332622e-05	1081
TM9SF3	-0.134546733	9.04690996480293e-06	3.24577028033758e-05	1081
FAM98B	-0.13457018	9.01415718261475e-06	3.23459559037717e-05	1081
TMEM167	-0.134582043	8.99762992348956e-06	3.22924024244962e-05	1081
UQCRC2	-0.134591385	8.98463553141639e-06	3.22515115431902e-05	1081
BTRC	-0.134596252	8.97787203158796e-06	3.22329766666083e-05	1081
PPAP2B	-0.134666935	8.88019895177763e-06	3.18936722915957e-05	1081
HEPACAM	-0.134677506	8.86567924673374e-06	3.18472019303586e-05	1081
CACNB4	-0.134684588	8.85596506231303e-06	3.18179804138213e-05	1081
DPY19L2P	-0.134701577	8.83270017486193e-06	3.17400544100238e-05	1081
C9orf93	-0.134727367	8.79749712539767e-06	3.16191935042169e-05	1081
ZNF823	-0.134754658	8.76038959655416e-06	3.14970637912874e-05	1081
PPP2R2A	-0.134759048	8.75443470216364e-06	3.14812722376092e-05	1081
SYPL2	-0.134779525	8.7267089480017e-06	3.13871722473655e-05	1081
CDH20	-0.134788935	8.71399551218122e-06	3.13470427844448e-05	1081
ALDH1L2	-0.134813211	8.68128053560966e-06	3.12405138245546e-05	1081
PCDHA3	-0.134824943	8.66551094622729e-06	3.11893367896639e-05	1081
KIF5B	-0.134873466	8.60058272863545e-06	3.09667093955963e-05	1081
SMARCA	-0.134879991	8.5918870201429e-06	3.094093028616e-05	1081
MEIS1	-0.134934036	8.52018507633735e-06	3.07156636297093e-05	1081
MYRIP	-0.134970434	8.47221772517871e-06	3.05646178498791e-05	1081
KLHL15	-0.134974499	8.46687672469519e-06	3.05522458859105e-05	1081
RNF115	-0.134979328	8.46053602978474e-06	3.05388816197838e-05	1081
DUOX2	-0.134980093	8.45953161520235e-06	3.05388816197838e-05	1081

MYOF	-0.135008521	8.42230033147414e-06	3.04226717191226e-05	1081
CCDC149	-0.135031991	8.39168020115892e-06	3.03229412829321e-05	1081
LMBR1	-0.135044094	8.37593214093834e-06	3.02714662682459e-05	1081
ZNF182	-0.135079631	8.32985339436423e-06	3.01103349415875e-05	1081
RC3H2	-0.135082767	8.32579869169072e-06	3.0101079440795e-05	1081
CC2D2A	-0.135087531	8.31964294260346e-06	3.00868171737845e-05	1081
ZNF286B	-0.135139903	8.25225600560653e-06	2.98512654395661e-05	1081
SUCLG2	-0.135171255	8.21216421715216e-06	2.97169118294468e-05	1081
EPPK1	-0.13517574	8.20644490159856e-06	2.97084549373104e-05	1081
TGFB3	-0.135201789	8.17329784069862e-06	2.96028559872121e-05	1081
DLX2	-0.135251723	8.11011607645573e-06	2.93793001906493e-05	1081
ZCCHC4	-0.135281845	8.07222818947771e-06	2.92473087908324e-05	1081
DENND5A	-0.135283993	8.06953229320626e-06	2.92473087908324e-05	1081
IGF2R	-0.13531948	8.02512672764676e-06	2.90975842203823e-05	1081
ZNF506	-0.135321355	8.02278731425802e-06	2.90943385140824e-05	1081
NLRP13	-0.135351656	7.98506673947831e-06	2.89627600768438e-05	1081
ZNF778	-0.135375813	7.95511581514524e-06	2.88593207448408e-05	1081
POM121	-0.135392595	7.93437185428908e-06	2.87944372193575e-05	1081
FREM1	-0.135397844	7.92789366310545e-06	2.87761113231098e-05	1081
LDHAL6A	-0.135404477	7.91971563319045e-06	2.87516077546624e-05	1081
ANKRA2	-0.135420509	7.89998194016332e-06	2.86851362985923e-05	1081
SH3TC2	-0.135428439	7.89023797541492e-06	2.86549205002224e-05	1081
THOC2	-0.135437769	7.87878819721541e-06	2.86184976979633e-05	1081
AKAP6	-0.135457175	7.85502542319301e-06	2.85373286114018e-05	1081
KHDRBS3	-0.135502342	7.79998286304034e-06	2.83629340750808e-05	1081
TSSK1B	-0.135612911	7.66678571118045e-06	2.79037756371689e-05	1081
DCDC1	-0.135614944	7.66435718312428e-06	2.78999774944052e-05	1081
HEYL	-0.135646699	7.62651845097162e-06	2.77672536047033e-05	1081
USP40	-0.135662786	7.60741756352857e-06	2.77027163444113e-05	1081
BACE1	-0.135675613	7.59221971564971e-06	2.76523713924721e-05	1081
CAV1	-0.135685811	7.58015830796267e-06	2.76184281269502e-05	1081
C2orf3	-0.135697422	7.56644778956343e-06	2.75734606947821e-05	1081
CCDC144B	-0.135734303	7.52305290377122e-06	2.7420282385828e-05	1081

DNAH6	-0.135745092	7.51040452018461e-06	2.7379134827926e-05	1081
ZNF185	-0.135775016	7.47542662856097e-06	2.7266425752736e-05	1081
ING5	-0.135785967	7.46266558259747e-06	2.72248095185487e-05	1081
SLC39A9	-0.13584397	7.39541929872304e-06	2.69843727174019e-05	1081
USP44	-0.135848072	7.39068514123151e-06	2.69719840887878e-05	1081
PDDC1	-0.135857951	7.37929668896579e-06	2.69401833633954e-05	1081
PAPPA2	-0.135886272	7.34674189333777e-06	2.68359230174596e-05	1081
SLC4A7	-0.135905909	7.32424915768842e-06	2.67586143057006e-05	1081
HKR1	-0.135947282	7.2770744233988e-06	2.65959115129479e-05	1081
CYP4X1	-0.135954367	7.26902537695968e-06	2.65755876346957e-05	1081
CRY1	-0.135966874	7.2548371874066e-06	2.65290788056464e-05	1081
LRRC37A	-0.136002679	7.21436367132326e-06	2.63906584635568e-05	1081
ACVR1	-0.136007618	7.20879761365082e-06	2.63750868011253e-05	1081
JUB	-0.136053968	7.15676358395817e-06	2.61894645592802e-05	1081
ATP2B4	-0.136057925	7.15233895439839e-06	2.61794342017794e-05	1081
ADAMTS	-0.136067576	7.14155525306755e-06	2.61480608093504e-05	1081
CSTF2T	-0.136074915	7.13336644635854e-06	2.61228262246669e-05	1081
ITPRIPL2	-0.136081713	7.12578822999211e-06	2.60998188896711e-05	1081
DACT3	-0.136093463	7.11270942750435e-06	2.60566523762639e-05	1081
PHYHIPL	-0.136177825	7.01947217247806e-06	2.5729124256654e-05	1081
KIAA137(	-0.136182702	7.01411867613822e-06	2.57141802967797e-05	1081
ERRFI1	-0.136210659	6.98350147586253e-06	2.56159208359888e-05	1081
ZFP36L2	-0.136283688	6.90412374352724e-06	2.53385995287586e-05	1081
TET1	-0.13629624	6.89056859699182e-06	2.52934592540817e-05	1081
SEC23IP	-0.136304511	6.88164922175939e-06	2.52653223204561e-05	1081
XPO7	-0.136337318	6.84638192210298e-06	2.51404235910981e-05	1081
SLC35F5	-0.136339202	6.84436159136286e-06	2.51404235910981e-05	1081
DLC1	-0.136353827	6.82870037308374e-06	2.50892155782914e-05	1081
LGR4	-0.136355492	6.82691972661937e-06	2.50872487947368e-05	1081
TMEM8B	-0.136386674	6.79364891420685e-06	2.4974098061441e-05	1081
PDGFRB	-0.136388884	6.79129744919294e-06	2.49700104241635e-05	1081
OAT	-0.136415904	6.76260292964139e-06	2.48735870034007e-05	1081
ISPD	-0.13641804	6.76033904533735e-06	2.48698009620747e-05	1081

C2orf63	-0.136426962	6.75089290849008e-06	2.48441245234806e-05	1081
MID2	-0.13644593	6.73085236118407e-06	2.47748987421986e-05	1081
RALGAPI	-0.136468282	6.70730839998713e-06	2.46927499484175e-05	1081
FTSJD1	-0.136470424	6.7050561869346e-06	2.46889703684514e-05	1081
GLCCI1	-0.136531311	6.6413418914342e-06	2.44633081738786e-05	1081
CLCA4	-0.136549222	6.62270998478758e-06	2.43991391081832e-05	1081
ELN	-0.13656823	6.60299119170681e-06	2.43309415688556e-05	1081
LUZP6	-0.136572246	6.59883190954266e-06	2.4320063815905e-05	1081
SPTY2D1	-0.136578171	6.59270115062393e-06	2.43019148544042e-05	1081
BAGE2	-0.136586815	6.58376599952142e-06	2.42734198499925e-05	1081
RD3	-0.136589303	6.58119544620128e-06	2.4268384086349e-05	1081
BBS7	-0.136609789	6.56007306630944e-06	2.41949234567565e-05	1081
LOC65227	-0.136642675	6.52630166437934e-06	2.40747751380556e-05	1081
GALC	-0.136642896	6.52607485049226e-06	2.40747751380556e-05	1081
PKHD1	-0.136644908	6.52401432680581e-06	2.40747751380556e-05	1081
ZMYM3	-0.136677575	6.49064222158311e-06	2.3960782032947e-05	1081
OR1L8	-0.136683148	6.48496569550652e-06	2.39442144310812e-05	1081
UGGT1	-0.136684313	6.48377974235976e-06	2.39442144310812e-05	1081
RORB	-0.13669318	6.4747589768287e-06	2.39152951206847e-05	1081
PRKDC	-0.136714037	6.45358622485984e-06	2.38414624059786e-05	1081
MCC	-0.13672708	6.44038022316968e-06	2.37970395443421e-05	1081
GLYR1	-0.136778541	6.38852683239745e-06	2.36184388032018e-05	1081
MEIS3P1	-0.136790429	6.37660487242993e-06	2.35830191215533e-05	1081
EIF4ENIF	-0.13682579	6.34126872931632e-06	2.34609473924843e-05	1081
FAM60A	-0.136831639	6.33544153199543e-06	2.34436939129405e-05	1081
OCLN	-0.136835656	6.33144268126032e-06	2.34332009579256e-05	1081
PPARGC1	-0.136848073	6.31909652307235e-06	2.339180438392e-05	1081
ANKRD31	-0.136884212	6.28329685445404e-06	2.32635572749452e-05	1081
SOX7	-0.1368941	6.27353546239816e-06	2.32316860091932e-05	1081
BCOR	-0.136911536	6.25635737980988e-06	2.3172333042153e-05	1081
COL8A1	-0.136941717	6.2267295401365e-06	2.3071080850846e-05	1081
C2orf40	-0.136948573	6.22001772693426e-06	2.30504520068231e-05	1081
HAUS2	-0.136981163	6.18820863797483e-06	2.2949459317379e-05	1081

CEP110	-0.13700937	6.16080336366877e-06	2.28562401033347e-05	1081
CRIPAK	-0.137051979	6.1196244124004e-06	2.27267065782273e-05	1081
HEPN1	-0.137058507	6.11333884546958e-06	2.27094248648321e-05	1081
OXSR1	-0.137073087	6.09932251008065e-06	2.26615366959747e-05	1081
FLJ45340	-0.137128158	6.04665723740577e-06	2.24741531453024e-05	1081
GPR98	-0.137176579	6.00071065522661e-06	2.23157312441462e-05	1081
KIAA1429	-0.137176925	6.00038407833396e-06	2.23157312441462e-05	1081
STAG3L1	-0.137207006	5.97200957557227e-06	2.2221302715165e-05	1081
GJC1	-0.137223792	5.95623242853414e-06	2.21666917186071e-05	1081
CDH11	-0.137231108	5.9493676851693e-06	2.21452350365365e-05	1081
CCDC54	-0.137240017	5.94101969470708e-06	2.21182483366982e-05	1081
MCTP1	-0.137240694	5.94038585122854e-06	2.21182483366982e-05	1081
ZNF253	-0.137294556	5.89015543268829e-06	2.19369904217981e-05	1081
ATXN7	-0.137323945	5.86292004287945e-06	2.18436331170347e-05	1081
SYNJ2	-0.137331859	5.85560596273181e-06	2.18204184460289e-05	1081
ODZ1	-0.137345964	5.8425929928813e-06	2.17840155176002e-05	1081
LRRC8B	-0.137381204	5.81020125471394e-06	2.16672536609056e-05	1081
PRDM5	-0.137421946	5.77296626271028e-06	2.15403603190033e-05	1081
FNDC3A	-0.137431881	5.76392101778645e-06	2.15145801191973e-05	1081
FHL1	-0.137456553	5.7415176956131e-06	2.14349284614763e-05	1081
THRAP3	-0.13745748	5.74067743476097e-06	2.14349284614763e-05	1081
KBTBD4	-0.137481513	5.71893728073958e-06	2.13664672701222e-05	1081
WSCD1	-0.137489042	5.71214248824754e-06	2.13450399602572e-05	1081
RPS6	-0.137491714	5.70973320900761e-06	2.13399954537028e-05	1081
PHKA2	-0.137493656	5.70798187165832e-06	2.13374085738647e-05	1081
RB1CC1	-0.13754438	5.6624385158527e-06	2.11828828044295e-05	1081
CUBN	-0.137613894	5.60058776406556e-06	2.09553938534734e-05	1081
WDR92	-0.137622439	5.59302971143914e-06	2.09414011627636e-05	1081
ZNF701	-0.137647479	5.57093725246514e-06	2.08677075029584e-05	1081
PDE12	-0.137684896	5.53808078049524e-06	2.07523506925366e-05	1081
C18orf55	-0.13769338	5.53065607350602e-06	2.07283844838658e-05	1081
IGSF11	-0.137747952	5.48312748721101e-06	2.05540757777942e-05	1081
ODZ2	-0.137768932	5.464960155712e-06	2.05012332098358e-05	1081

NGFR	-0.137774047	5.4605387124839e-06	2.04922787561453e-05	1081
ZNF43	-0.137798446	5.43949926830499e-06	2.04209304435341e-05	1081
CYB5D1	-0.137841866	5.40224848313837e-06	2.02886457294598e-05	1081
SLC5A1	-0.137848828	5.3962983109157e-06	2.02700782161844e-05	1081
THSD1P1	-0.137862417	5.38470225306661e-06	2.02340658250376e-05	1081
C1orf183	-0.13786785	5.38007263023981e-06	2.02204408836158e-05	1081
C1orf21	-0.137926287	5.33051806793886e-06	2.0045414687069e-05	1081
PCNX	-0.137956304	5.30523389419332e-06	1.99540584015169e-05	1081
METTL2E	-0.137994349	5.27335211079837e-06	1.98378484168129e-05	1081
KLHDC8A	-0.138085381	5.19780965758133e-06	1.95646254768266e-05	1081
OSMR	-0.138093621	5.1910227267728e-06	1.9542730859809e-05	1081
ZNF366	-0.138109564	5.17791552213877e-06	1.95043209037931e-05	1081
CACNA1C	-0.13815434	5.14127345684151e-06	1.9380792250762e-05	1081
C20orf94	-0.138169731	5.12873580623236e-06	1.93371481969962e-05	1081
SEPT8	-0.138178364	5.12171564734517e-06	1.9314294592993e-05	1081
SPG11	-0.138206528	5.09887898029219e-06	1.92425846867715e-05	1081
PCDHB7	-0.138216092	5.09114567601352e-06	1.92170001205345e-05	1081
UBR5	-0.138248945	5.0646679274243e-06	1.91294938193274e-05	1081
RRH	-0.138257363	5.05790512138994e-06	1.91094333590398e-05	1081
GPR75	-0.138276198	5.04280333694216e-06	1.90559507076908e-05	1081
FGF7	-0.138285021	5.03574407484655e-06	1.90328451008975e-05	1081
C17orf51	-0.138321701	5.00649791119907e-06	1.89258585890608e-05	1081
KDR	-0.138376163	4.96337338429052e-06	1.87663582632381e-05	1081
PHYHD1	-0.138388367	4.95375878211748e-06	1.87335218069752e-05	1081
PLS3	-0.138407388	4.93880916860001e-06	1.86840020096614e-05	1081
TMEM10C	-0.138419868	4.929023597085e-06	1.86539884206796e-05	1081
C15orf51	-0.13848507	4.87820073241671e-06	1.84755318207435e-05	1081
WDR52	-0.138499257	4.86720871497183e-06	1.84373673492116e-05	1081
TNRC18	-0.138529346	4.84397522014179e-06	1.83597141692862e-05	1081
NOM1	-0.138531349	4.84243256455259e-06	1.83573210411953e-05	1081
L3MBTL3	-0.138547408	4.8300793704704e-06	1.83139373081359e-05	1081
ADNP	-0.13855801	4.8219403438326e-06	1.8286518867942e-05	1081
ZNF2	-0.138579125	4.8057697290771e-06	1.82286257187457e-05	1081

WDR60	-0.138592472	4.79557500124567e-06	1.81933819962512e-05	1081
PPP1R1A	-0.138597338	4.79186348552793e-06	1.81861510768576e-05	1081
SLC27A6	-0.138605702	4.78549006006076e-06	1.81653848238033e-05	1081
SLC6A15	-0.138637265	4.76151249123387e-06	1.80777740550144e-05	1081
C12orf51	-0.138693999	4.71870140993534e-06	1.79287513962934e-05	1081
CNST	-0.138718109	4.70061998580013e-06	1.78634200365863e-05	1081
GBE1	-0.138719175	4.69982151501255e-06	1.78634200365863e-05	1081
LRRC37A	-0.138752381	4.67503119055709e-06	1.77728823049203e-05	1081
ACOX3	-0.138765296	4.66542295806321e-06	1.77430518199327e-05	1081
MMRN1	-0.138867183	4.59028291726285e-06	1.74770836076848e-05	1081
NMD3	-0.138891859	4.57226015599944e-06	1.74183397962573e-05	1081
ZXDB	-0.138901535	4.56521128202752e-06	1.73947761067608e-05	1081
SLC44A5	-0.138974718	4.51223437426722e-06	1.72026800661645e-05	1081
ZNF658	-0.138994419	4.49807360845054e-06	1.71551860738804e-05	1081
MYNN	-0.139024601	4.47646171741882e-06	1.70759934287828e-05	1081
ZNF780A	-0.139041401	4.46447544652346e-06	1.70334958087529e-05	1081
WNK1	-0.139050777	4.45779887299414e-06	1.70112442311928e-05	1081
SNX25	-0.139119687	4.40902327368059e-06	1.68346804109734e-05	1081
UBE3B	-0.139123654	4.4062306757096e-06	1.68272070070464e-05	1081
LRRIQ1	-0.13913966	4.3949814485782e-06	1.67874291394781e-05	1081
C14orf167	-0.139150253	4.38755219278991e-06	1.67622300253656e-05	1081
SNRNP20(	-0.139152015	4.38631697500185e-06	1.67606895791753e-05	1081
CLDN20	-0.139173574	4.37123723252388e-06	1.67125781076473e-05	1081
LHFP	-0.139192966	4.35771522084018e-06	1.66640419749099e-05	1081
ACO1	-0.139254225	4.31526270517019e-06	1.65048352374508e-05	1081
PAWR	-0.139278564	4.29850522798105e-06	1.64469872398249e-05	1081
BVES	-0.139288018	4.29201346201986e-06	1.64252680836607e-05	1081
KIAA0087	-0.139314566	4.27383246154463e-06	1.63619070577378e-05	1081
SERPINB:	-0.139328427	4.26436945498384e-06	1.63287821080877e-05	1081
PHF6	-0.139373357	4.2338330638086e-06	1.62211044257178e-05	1081
TRUB1	-0.139398856	4.2165964135819e-06	1.61581386249966e-05	1081
RNF168	-0.139401352	4.21491308182111e-06	1.61578352109013e-05	1081
NUDT12	-0.139423613	4.19992516451037e-06	1.61034435552077e-05	1081

PSMD5	-0.139466892	4.17093284877295e-06	1.60044651882916e-05	1081
TM4SF18	-0.139470423	4.16857623566403e-06	1.60015183436456e-05	1081
ALDH1A2	-0.139602141	4.08155951429645e-06	1.56854285416829e-05	1081
ENTPD3	-0.139605942	4.07907451451081e-06	1.56818618501565e-05	1081
CPSF6	-0.139612851	4.07456126249014e-06	1.5667500788865e-05	1081
CORO2B	-0.139645477	4.0533128425288e-06	1.55917485607681e-05	1081
CHST6	-0.139666738	4.03952332461102e-06	1.55416725313768e-05	1081
WNT2B	-0.139669877	4.03749110241658e-06	1.55368210617348e-05	1081
DNAJB7	-0.139671279	4.03658419239227e-06	1.55362989216168e-05	1081
ZNF670	-0.139701368	4.01715861661824e-06	1.5467442723963e-05	1081
GNAI1	-0.139718407	4.00619868332611e-06	1.54311419647427e-05	1081
FAM55B	-0.139761851	3.97838215053751e-06	1.53269283653812e-05	1081
FUT9	-0.139771301	3.97235570215266e-06	1.5306638412369e-05	1081
ANO1	-0.139775012	3.96999215903924e-06	1.53004576322643e-05	1081
FAM133B	-0.139779109	3.96738327870687e-06	1.52933287695273e-05	1081
PBX1	-0.139818488	3.94239620490287e-06	1.51999179995729e-05	1081
FBXW11	-0.139823059	3.93950556363039e-06	1.51916806239154e-05	1081
RPA1	-0.139843917	3.92634028638519e-06	1.51438110413995e-05	1081
C4orf14	-0.139862485	3.91465627545595e-06	1.51045299117142e-05	1081
KERA	-0.139863223	3.91419257108486e-06	1.51045299117142e-05	1081
STT3B	-0.139895547	3.89393426543156e-06	1.50303325880665e-05	1081
VPS54	-0.13992164	3.87765406905784e-06	1.49732300596454e-05	1081
TLE2	-0.140030427	3.81047854771991e-06	1.47420953224155e-05	1081
NPAS2	-0.140036121	3.80699355583775e-06	1.47314416408666e-05	1081
KLHL33	-0.140042885	3.80285763325537e-06	1.47182645575273e-05	1081
AGL	-0.140051153	3.79780747266071e-06	1.47015433391141e-05	1081
PTPN12	-0.140081284	3.77945831484471e-06	1.46333245728516e-05	1081
GABRA2	-0.140081956	3.77904981993199e-06	1.46333245728516e-05	1081
RNF152	-0.140105991	3.76447516771176e-06	1.45837215872218e-05	1081
TAF4B	-0.140200973	3.70740430156189e-06	1.43736835363673e-05	1081
VANGL2	-0.140276238	3.66276965049118e-06	1.42088377833901e-05	1081
MYH9	-0.140278054	3.66169908226328e-06	1.4207420649498e-05	1081
NFXL1	-0.140318096	3.63816922378667e-06	1.41270082908987e-05	1081

EEF1G	-0.140368127	3.60897354131451e-06	1.40244544733373e-05	1081
FAM198B	-0.140397771	3.591780434632e-06	1.39630291114747e-05	1081
NEUROG2	-0.140401857	3.58941694251635e-06	1.39565343190488e-05	1081
MED21	-0.140409735	3.5848636312134e-06	1.39415208206166e-05	1081
ZNF646	-0.14040993	3.58475132121774e-06	1.39415208206166e-05	1081
ZNF808	-0.140420062	3.57890353040488e-06	1.39264074985525e-05	1081
ANKMY2	-0.140449374	3.56203857596105e-06	1.38634596431096e-05	1081
IGF1	-0.140456539	3.55792730566075e-06	1.38501344101518e-05	1081
C2orf16	-0.140469058	3.55075577223802e-06	1.38248888735475e-05	1081
HNRNPA1	-0.140509794	3.52751428570006e-06	1.37423661352596e-05	1081
BCL2L11	-0.140569898	3.49348913398088e-06	1.36150780816492e-05	1081
UGT2B28	-0.140584702	3.48515669170968e-06	1.35852324989341e-05	1081
RSL1D1	-0.140587675	3.48348570095645e-06	1.35813469026065e-05	1081
KIAA0528	-0.140597165	3.47815694676197e-06	1.35658222057154e-05	1081
IQCH	-0.140635204	3.45687487778225e-06	1.34906517653862e-05	1081
C1orf111	-0.140637688	3.45548934821725e-06	1.34878575702067e-05	1081
SLC30A6	-0.140652063	3.44748287904089e-06	1.34618225621785e-05	1081
ZNF525	-0.140689172	3.4268950647465e-06	1.33892166561905e-05	1081
ATAD2B	-0.140779469	3.37729037706184e-06	1.32082148409844e-05	1081
GLT8D2	-0.14078409	3.3747704302922e-06	1.32009223918906e-05	1081
SF3A1	-0.140789905	3.37160191811637e-06	1.31923339116288e-05	1081
KRAS	-0.140803871	3.364003294187e-06	1.31664749099275e-05	1081
EDN3	-0.140815283	3.35780664108485e-06	1.3144775511981e-05	1081
BTBD19	-0.140853502	3.3371336601595e-06	1.30740096429236e-05	1081
FLJ39653	-0.140874018	3.32608665619633e-06	1.30358007177189e-05	1081
UTP15	-0.140915501	3.30385694146662e-06	1.2956238677408e-05	1081
ANKS1B	-0.140940039	3.29077444314014e-06	1.29074476551904e-05	1081
PTPDC1	-0.140965929	3.27702485565746e-06	1.28560205875793e-05	1081
LEFTY2	-0.141003126	3.25736713620141e-06	1.27838809582656e-05	1081
FKSG29	-0.141053693	3.23082424054566e-06	1.26846529576676e-05	1081
POLR3B	-0.141064788	3.22502871115861e-06	1.26643671318304e-05	1081
TIA1	-0.141126605	3.19291725918145e-06	1.25456052635479e-05	1081
BACH2	-0.141140975	3.1854968907077e-06	1.2518890921441e-05	1081

PEG3AS	-0.141171919	3.16957346468956e-06	1.24587429163261e-05	1081
ZNF542	-0.141255389	3.12700032114559e-06	1.2296197827343e-05	1081
RG9MTD2	-0.14128049	3.11430536463073e-06	1.2251060646453e-05	1081
CAPRIN1	-0.141306757	3.1010736951713e-06	1.22013924978957e-05	1081
SOS1	-0.141309956	3.09946574083389e-06	1.21974482025979e-05	1081
MPZL1	-0.141333754	3.08753016085653e-06	1.21528517175566e-05	1081
CMTM1	-0.141354007	3.07740693371535e-06	1.21153728121352e-05	1081
KCND3	-0.141377782	3.06556420983335e-06	1.20711084845764e-05	1081
USP19	-0.141381872	3.06353121435193e-06	1.20654616447937e-05	1081
NCKAP5	-0.141400431	3.05432303349281e-06	1.2031548202916e-05	1081
ZNF548	-0.141412289	3.04845310118712e-06	1.20154740214077e-05	1081
TMEM87A	-0.141444301	3.03266132062739e-06	1.19579100223212e-05	1081
C4orf29	-0.141445355	3.03214266711734e-06	1.19579100223212e-05	1081
NIN	-0.141480383	3.01495543097483e-06	1.18927505692164e-05	1081
CLK1	-0.141509234	3.0008690988479e-06	1.18395041120821e-05	1081
SERAC1	-0.141510845	3.00008447853343e-06	1.18387270950159e-05	1081
QRICH1	-0.141546226	2.98290070821952e-06	1.17755310145174e-05	1081
C10orf93	-0.141570166	2.97132691153466e-06	1.17344404298894e-05	1081
SKIL	-0.141724579	2.89770289306928e-06	1.1450416787148e-05	1081
EFCAB4B	-0.141732502	2.89397308547359e-06	1.143792187696e-05	1081
MPPED2	-0.141743122	2.8889803285792e-06	1.14226709949417e-05	1081
UHRF2	-0.141748384	2.88650960621668e-06	1.14151425239959e-05	1081
TBC1D19	-0.1417533	2.88420317580389e-06	1.14082609417965e-05	1081
ZNF461	-0.141756167	2.88285898103477e-06	1.14051834589445e-05	1081
SMG6	-0.141782882	2.87036174374845e-06	1.13579723684566e-05	1081
ZNF425	-0.14181501	2.85540064399637e-06	1.13009913503555e-05	1081
GPLD1	-0.141831588	2.84771049422295e-06	1.1272770270411e-05	1081
PIGA	-0.141863574	2.83292806151953e-06	1.12230748867868e-05	1081
PLP1	-0.141878171	2.82620678758562e-06	1.11986498300378e-05	1081
C17orf68	-0.141880095	2.82532203015256e-06	1.11973465074608e-05	1081
IGF1R	-0.141889001	2.82122956908305e-06	1.11833273650488e-05	1081
NCKAP1	-0.141913477	2.81001254006386e-06	1.11410554260158e-05	1081
C1orf27	-0.142007818	2.76717512194886e-06	1.09755351115692e-05	1081

CADM2	-0.142053299	2.74674777675997e-06	1.09031002882423e-05	1081
FHL5	-0.142053806	2.74652115552464e-06	1.09031002882423e-05	1081
SHISA2	-0.142095311	2.72800695071422e-06	1.08393885645243e-05	1081
C4orf39	-0.142108447	2.72217244217436e-06	1.08183396819101e-05	1081
MRPS27	-0.142112409	2.72041491362576e-06	1.08156223475411e-05	1081
STL	-0.142138295	2.70895961754717e-06	1.07722051905819e-05	1081
GRHL1	-0.142138465	2.70888426115036e-06	1.07722051905819e-05	1081
ARHGEF1	-0.142176555	2.69211247812989e-06	1.0711555574151e-05	1081
ASB3	-0.14218163	2.68988561659336e-06	1.07048095113143e-05	1081
PCMTD1	-0.142186404	2.6877919650249e-06	1.06985910166818e-05	1081
LOC64647	-0.142245257	2.66211301002001e-06	1.06005666311234e-05	1081
TTC21B	-0.142312374	2.63311510564843e-06	1.04871695933744e-05	1081
SHISA3	-0.142330136	2.62549177393223e-06	1.04609437867612e-05	1081
FBLIM1	-0.142330397	2.62537978007572e-06	1.04609437867612e-05	1081
AGTR2	-0.142341122	2.62078711428871e-06	1.0446330909645e-05	1081
SEPT7P2	-0.142362843	2.61150978522368e-06	1.04114119579123e-05	1081
PGM2L1	-0.142378654	2.60477604894945e-06	1.03886781837431e-05	1081
PTPRT	-0.142439442	2.57904245881902e-06	1.02880812540414e-05	1081
ZBED5	-0.142456835	2.57172430105203e-06	1.02629528614685e-05	1081
NRG3	-0.142470627	2.5659354565419e-06	1.02439099825677e-05	1081
ZNF841	-0.142488415	2.55848769410057e-06	1.02162011095453e-05	1081
MIER1	-0.142501371	2.55307633674322e-06	1.0198636288656e-05	1081
HOXA4	-0.142512312	2.54851511142365e-06	1.01824349305096e-05	1081
PID1	-0.142561127	2.5282591707006e-06	1.01095238177379e-05	1081
STEAP1	-0.142618478	2.50465803283041e-06	1.00171403754951e-05	1081
IRAK1BP1	-0.142651428	2.49119454549312e-06	9.97121281918514e-06	1081
MGEA5	-0.142660873	2.4873480307921e-06	9.95779532597513e-06	1081
SFRP1	-0.142663338	2.48634501792867e-06	9.95575837530771e-06	1081
MBD5	-0.142681443	2.47899033471415e-06	9.92828236437704e-06	1081
OXR1	-0.142702301	2.47054337238921e-06	9.89642001129064e-06	1081
TMTC3	-0.142733696	2.45788069543691e-06	9.84765445695634e-06	1081
ZNF443	-0.14274351	2.45393535339408e-06	9.83380300261063e-06	1081
NDST3	-0.142780797	2.43900036868496e-06	9.77784326908626e-06	1081

NCRNA0(	-0.142790527	2.43511762605741e-06	9.76422065623536e-06	1081
PLEKHG3	-0.142799454	2.43156030716828e-06	9.753839583414e-06	1081
IKBKB	-0.142805966	2.42896856604014e-06	9.7453837408641e-06	1081
TSGA10	-0.142871868	2.40288863007558e-06	9.64266762009413e-06	1081
NUP160	-0.142918728	2.38450765744526e-06	9.57462761794594e-06	1081
LRRFIP1	-0.142931618	2.37947524696156e-06	9.56013738532919e-06	1081
TRIM33	-0.142948406	2.37293618074145e-06	9.53576687832365e-06	1081
ESCO1	-0.142989481	2.35700961837392e-06	9.48500974073963e-06	1081
TLL1	-0.143033614	2.34001190762195e-06	9.4184894863225e-06	1081
NUP98	-0.143061198	2.32944777222563e-06	9.37971724395067e-06	1081
WDR7	-0.143083027	2.32112003521269e-06	9.34992263734446e-06	1081
GTPBP10	-0.14309982	2.31473274771734e-06	9.32792382531823e-06	1081
LRP1	-0.143128054	2.30403238354971e-06	9.28851958507281e-06	1081
FGF1	-0.143131098	2.30288157911944e-06	9.28573847305067e-06	1081
ANKH	-0.143138372	2.3001337806475e-06	9.27651551774654e-06	1081
TMEM182	-0.143176674	2.28571656493368e-06	9.22760725462707e-06	1081
PPIL6	-0.14322483	2.26771325910516e-06	9.15676159644688e-06	1081
KIAA0141	-0.143240063	2.26204661736243e-06	9.13754343428238e-06	1081
RNF185	-0.143259504	2.25483474415934e-06	9.11023785019855e-06	1081
NCRNA0(	-0.143288292	2.24419525443619e-06	9.07088952660857e-06	1081
TMEM232	-0.143361162	2.21747936137337e-06	8.96650376051115e-06	1081
SUN1	-0.14338327	2.20943470743936e-06	8.93692185036665e-06	1081
ZNF595	-0.143388488	2.20753991381567e-06	8.93169141671353e-06	1081
ZFC3H1	-0.143392279	2.20616448201038e-06	8.92791954401346e-06	1081
PRRC1	-0.143483189	2.17342306096275e-06	8.8024944839354e-06	1081
KIAA1586	-0.143511565	2.16329923692762e-06	8.76677994928726e-06	1081
ZCWPW2	-0.143611848	2.12788186352389e-06	8.63019531723149e-06	1081
SLC7A3	-0.143621561	2.12448117468221e-06	8.61813799113436e-06	1081
ZBTB43	-0.143628835	2.12193796900142e-06	8.60955496183961e-06	1081
PTCH1	-0.143661338	2.11060908062041e-06	8.56531424840819e-06	1081
PAX9	-0.143667567	2.10844457111525e-06	8.5582542585365e-06	1081
PKD2	-0.143693348	2.09950835656811e-06	8.52713625868236e-06	1081
ZNF792	-0.143721366	2.08983824271889e-06	8.48957277668322e-06	1081

TJP2	-0.143730521	2.08668781909254e-06	8.47848449286391e-06	1081
FANK1	-0.143766132	2.07447668753063e-06	8.4339723249858e-06	1081
ZNF655	-0.143788761	2.0667524733905e-06	8.40426495285659e-06	1081
PCF11	-0.143880741	2.03563950294077e-06	8.28778451631808e-06	1081
DAB2IP	-0.143929624	2.01928745901267e-06	8.22287161144336e-06	1081
CCDC41	-0.143930456	2.01901026988438e-06	8.22287161144336e-06	1081
SLC5A4	-0.143943696	2.01460373796789e-06	8.2071167444617e-06	1081
SEPSECS	-0.143966967	2.00688115442198e-06	8.17731004365508e-06	1081
RPS6KA3	-0.144023159	1.98834977713912e-06	8.10344047349131e-06	1081
ATF7IP	-0.144027977	1.98676866109561e-06	8.09863510274608e-06	1081
SPARCL1	-0.144096314	1.96447062789011e-06	8.01585189362897e-06	1081
CPD	-0.144123014	1.95582378514251e-06	7.98218601128361e-06	1081
LOC39995	-0.144171159	1.9403243436088e-06	7.92535156163813e-06	1081
MAN2A1	-0.144186143	1.93552463560491e-06	7.90895411445453e-06	1081
TTC14	-0.144192825	1.93338768615278e-06	7.90182490110523e-06	1081
C15orf62	-0.144204925	1.92952434406571e-06	7.88763553392931e-06	1081
ATP1B2	-0.144217588	1.9254890190837e-06	7.8727372213195e-06	1081
ARHGEF3	-0.14425543	1.91347800900944e-06	7.82521609652766e-06	1081
NOL9	-0.144261309	1.91161849903894e-06	7.81919891637348e-06	1081
SNX19	-0.14427464	1.90740812916219e-06	7.80356148699679e-06	1081
CALN1	-0.144303092	1.89845235919014e-06	7.77165673153532e-06	1081
CRTC3	-0.144363964	1.87942672868494e-06	7.69690007102219e-06	1081
KSR2	-0.144397733	1.86895104410381e-06	7.65711181278653e-06	1081
ALCAM	-0.144423937	1.86086105974791e-06	7.62551791062279e-06	1081
RGS17	-0.144502018	1.83695348112385e-06	7.53061210363042e-06	1081
CEP120	-0.144512943	1.83363171505671e-06	7.51852450637442e-06	1081
ZNF440	-0.144520818	1.83124100680467e-06	7.51025042387622e-06	1081
MED13L	-0.14453225	1.82777602407512e-06	7.49756628079686e-06	1081
GOLGA6L	-0.144546823	1.82336798914053e-06	7.48100776807249e-06	1081
STAM2	-0.144563338	1.81838463138679e-06	7.46400326879691e-06	1081
GLI1	-0.144565951	1.81759739807278e-06	7.46341206363149e-06	1081
USP51	-0.144570978	1.81608388547602e-06	7.45871760915685e-06	1081
FLJ22536	-0.144638888	1.79575437807919e-06	7.3767275584024e-06	1081

MPP6	-0.144673282	1.78554168041202e-06	7.3392648749031e-06	1081
LONRF2	-0.144700268	1.77756779288836e-06	7.30947197136887e-06	1081
ZNF761	-0.144739122	1.76614726493707e-06	7.26399278320891e-06	1081
ZNF502	-0.144757951	1.76063780219993e-06	7.24281162452882e-06	1081
EVC	-0.144817261	1.74339174078724e-06	7.1747960404819e-06	1081
SCN5A	-0.144820628	1.74241758399182e-06	7.17225219238153e-06	1081
KLHL7	-0.144876989	1.72618821616582e-06	7.10835274216281e-06	1081
LOC44017	-0.144898878	1.71992437837397e-06	7.08545533790259e-06	1081
ZBTB6	-0.144904573	1.71829816601307e-06	7.08020383602644e-06	1081
VPS13B	-0.1449422	1.70759113753762e-06	7.03896530912529e-06	1081
MANSC1	-0.14499931	1.69146267889353e-06	6.97390824116047e-06	1081
SLC18A2	-0.145020625	1.68548040044684e-06	6.95208899815757e-06	1081
USP31	-0.14503517	1.68141019122773e-06	6.93672093022375e-06	1081
GMEB1	-0.145035845	1.68122156360357e-06	6.93672093022375e-06	1081
ASB14	-0.145045016	1.67866005963761e-06	6.92821284601508e-06	1081
VTCN1	-0.145100438	1.66326070650905e-06	6.87029917097727e-06	1081
TOMM20	-0.145121222	1.65752091207194e-06	6.85079170572203e-06	1081
IRAK3	-0.145161854	1.6463544561973e-06	6.80743237276161e-06	1081
ATXN3	-0.145181232	1.64105429727588e-06	6.7869100428295e-06	1081
NRG1	-0.145190464	1.63853523116648e-06	6.777883415164e-06	1081
SESTD1	-0.145220164	1.63045565742851e-06	6.74723278942014e-06	1081
NFIB	-0.145224428	1.62929896944029e-06	6.74383146483967e-06	1081
IL13RA1	-0.145233013	1.62697226832692e-06	6.73558494563211e-06	1081
PPARA	-0.145280914	1.61404866486151e-06	6.6848294312572e-06	1081
TIGD6	-0.145298789	1.60925123068138e-06	6.66770177747354e-06	1081
AK5	-0.145300623	1.60875971134142e-06	6.6670364914571e-06	1081
ZNF449	-0.145323527	1.6026346414219e-06	6.64301951675808e-06	1081
CASK	-0.145325452	1.60212067013925e-06	6.64225579336391e-06	1081
G3BP2	-0.145355426	1.59414086353385e-06	6.61053266691835e-06	1081
ECT2L	-0.145395666	1.58348820286999e-06	6.57041603435961e-06	1081
ZNF677	-0.145411064	1.57942984345359e-06	6.55492669888187e-06	1081
TIGD4	-0.145507568	1.55422212585339e-06	6.45296882220044e-06	1081
IRF2BP2	-0.145519847	1.55104276705925e-06	6.4410959683382e-06	1081

MRAP2	-0.145521786	1.55054111048737e-06	6.44034034448828e-06	1081
TCF4	-0.145579488	1.53568772251483e-06	6.38127664398953e-06	1081
KIAA1614	-0.145596772	1.53126521670773e-06	6.36421245937222e-06	1081
ERBB4	-0.145626938	1.52357571734422e-06	6.33486745632595e-06	1081
UBXN7	-0.145649917	1.51774314500541e-06	6.31191900415648e-06	1081
TMEM132	-0.145654701	1.51653156544982e-06	6.30818261118864e-06	1081
ANKAR	-0.145730641	1.49742322624736e-06	6.23127264878186e-06	1081
ZNF500	-0.145810269	1.47763514211056e-06	6.15401280500668e-06	1081
RDX	-0.145818764	1.47553896730094e-06	6.1465534524974e-06	1081
PTBP2	-0.145852363	1.4672763159288e-06	6.11592828147852e-06	1081
ZNF638	-0.145865248	1.46411939720631e-06	6.10403254485122e-06	1081
ZNF117	-0.145879899	1.46053770268539e-06	6.09036059213354e-06	1081
ISCA1	-0.1458916	1.4576832764207e-06	6.08097527510769e-06	1081
TBX3	-0.14598502	1.43508485599667e-06	5.99042362703126e-06	1081
C10orf32	-0.146062835	1.41651860379409e-06	5.91660113486043e-06	1081
EEF1B2	-0.146081379	1.412128275493e-06	5.89948654288812e-06	1081
TMOD3	-0.146127189	1.401338493896e-06	5.85683899575412e-06	1081
SLC35F1	-0.146164543	1.39259886312851e-06	5.82151983766837e-06	1081
LRRC3B	-0.146194746	1.38557075446511e-06	5.79334222679528e-06	1081
C11orf30	-0.146339467	1.35236502632881e-06	5.65685080053858e-06	1081
ZNF662	-0.146342277	1.35172780721711e-06	5.65535964203295e-06	1081
DNAJC21	-0.146343713	1.3514023810551e-06	5.65517261453158e-06	1081
GRAMD3	-0.146400203	1.33865803681776e-06	5.60533488914857e-06	1081
ANGEL1	-0.146427009	1.33265096223142e-06	5.58134171188192e-06	1081
PCDHGA1	-0.146434336	1.33101357901593e-06	5.57680294286104e-06	1081
NOSTRIN	-0.146487704	1.31914517475478e-06	5.53052644025702e-06	1081
ZNF17	-0.146577764	1.29934717046544e-06	5.45092643669852e-06	1081
C14orf132	-0.14664679	1.28436665183636e-06	5.38920354118797e-06	1081
TP53BP1	-0.146696094	1.27376794032373e-06	5.3491880670881e-06	1081
DCLK3	-0.146711584	1.27045558877387e-06	5.33639029104453e-06	1081
RAD52	-0.146723111	1.26799594031723e-06	5.32716959701575e-06	1081
OR6F1	-0.146750309	1.26221054546084e-06	5.30507645280797e-06	1081
CNN3	-0.146766038	1.25887642990542e-06	5.29327190157475e-06	1081

FAM48B1	-0.146813589	1.24884820546074e-06	5.25329862163429e-06	1081
FGF10	-0.146837053	1.2439281293587e-06	5.23369510566645e-06	1081
IKBKAP	-0.146924904	1.22567170940415e-06	5.15903815000973e-06	1081
MATN3	-0.146941142	1.22232556759395e-06	5.14925733148894e-06	1081
EPAS1	-0.146970116	1.21637673596974e-06	5.12741354804571e-06	1081
MUTED	-0.146977763	1.21481144287741e-06	5.12188709015603e-06	1081
C14orf37	-0.147018633	1.20647771978695e-06	5.08781529518697e-06	1081
LOC34459[illegible]	-0.147019871	1.20622615260409e-06	5.08781529518697e-06	1081
SMR3B	-0.147023129	1.20556433162407e-06	5.08609287132293e-06	1081
ZNF566	-0.14708629	1.19280267573954e-06	5.03541699555175e-06	1081
RYR2	-0.147133751	1.18329866661385e-06	4.99843817130133e-06	1081
CLASP1	-0.147139826	1.18208728349318e-06	4.99436835695684e-06	1081
NLRP8	-0.147170993	1.17589189586952e-06	4.96923478965629e-06	1081
DNAJC27	-0.147221006	1.16601534138872e-06	4.93267094755896e-06	1081
KIAA049[illegible]	-0.147235057	1.16325508393961e-06	4.92202765510678e-06	1081
FAM19A2	-0.147300203	1.15053844316114e-06	4.87128981451894e-06	1081
MYST4	-0.147310765	1.14848945834545e-06	4.86363677493571e-06	1081
GNG7	-0.147328712	1.14501562126322e-06	4.8499452671042e-06	1081
PCDHGB[illegible]	-0.147334431	1.14391089546717e-06	4.84628496092244e-06	1081
CRISPLD[illegible]	-0.147351761	1.14056918891256e-06	4.83314394418247e-06	1081
KIAA066[illegible]	-0.147359925	1.13899809877064e-06	4.82750193556374e-06	1081
KLHDC5	-0.147378947	1.13534581729381e-06	4.81506136618607e-06	1081
C1orf114	-0.147420744	1.12736027294053e-06	4.78320823470659e-06	1081
DENND4[illegible]	-0.147436448	1.12437385687527e-06	4.77154231024905e-06	1081
ERO1LB	-0.147438757	1.12393546168962e-06	4.77154231024905e-06	1081
LOC7296[illegible]	-0.147442686	1.12318970599488e-06	4.76953132952504e-06	1081
BAG4	-0.147496243	1.11307206047912e-06	4.72955845989282e-06	1081
AVL9	-0.147536228	1.10557576498559e-06	4.69869700118874e-06	1081
ADAM12	-0.147579016	1.09760740449768e-06	4.66778576390244e-06	1081
QARS	-0.147595418	1.0945676560914e-06	4.65682480083659e-06	1081
CCDC66	-0.147787827	1.05950674409093e-06	4.51337774576267e-06	1081
NHSL1	-0.147793673	1.0584586032637e-06	4.50986644728158e-06	1081
ACAP2	-0.147817443	1.05420694766731e-06	4.49365191721497e-06	1081

SPDYE5	-0.147840016	1.05018450414738e-06	4.4774532986347e-06	1081
H2AFV	-0.147866655	1.04545651531377e-06	4.45918304065126e-06	1081
PCDHA10	-0.14788579	1.04207304344523e-06	4.44663449697184e-06	1081
SLC37A3	-0.147886034	1.04202999276915e-06	4.44663449697184e-06	1081
CEP170	-0.148032147	1.01653990844449e-06	4.34359423991048e-06	1081
ABCA11P	-0.148034832	1.01607709960824e-06	4.34306666064248e-06	1081
GTF2IP1	-0.148042493	1.01475792920643e-06	4.33834857467391e-06	1081
ZNF143	-0.148064556	1.0109678027303e-06	4.32398012441652e-06	1081
CADPS2	-0.148087917	1.00696954124302e-06	4.30779388582304e-06	1081
ZNF230	-0.148174293	9.92317586361441e-07	4.24781933218258e-06	1081
TRIM2	-0.148178855	9.91549481502067e-07	4.24543343355136e-06	1081
CAV2	-0.148210165	9.86293120478591e-07	4.22472356199048e-06	1081
LOC72819	-0.148220701	9.84530349176349e-07	4.21806973291313e-06	1081
KDM6A	-0.148222263	9.84269215055542e-07	4.21784797645052e-06	1081
PRPF39	-0.148297005	9.71854163440701e-07	4.1673056880615e-06	1081
DR1	-0.148335022	9.65597105311244e-07	4.14223885998616e-06	1081
CROT	-0.148356832	9.6202501066774e-07	4.12955334325626e-06	1081
CLPX	-0.148364789	9.60725012825642e-07	4.12485195724053e-06	1081
CSNK1E	-0.148377223	9.5869674788273e-07	4.11789893093765e-06	1081
CLEC16A	-0.148395477	9.55726885333695e-07	4.10601793667035e-06	1081
MECOM	-0.148425035	9.5093638971891e-07	4.08718019434338e-06	1081
PSKH1	-0.148480972	9.41933804722141e-07	4.05021483375187e-06	1081
GATM	-0.148492809	9.40039290048708e-07	4.0437948960135e-06	1081
PLCZ1	-0.148494397	9.39785425289265e-07	4.04356629484243e-06	1081
MTERF	-0.14849641	9.39463716885539e-07	4.04304562628908e-06	1081
ARMCX2	-0.148547809	9.31284361089525e-07	4.01041544552126e-06	1081
KLHL18	-0.148621673	9.19650048713409e-07	3.96285566445596e-06	1081
SPTLC1	-0.148640987	9.16630931698876e-07	3.95069108238636e-06	1081
ASTN1	-0.14868419	9.09912290944036e-07	3.92425243011509e-06	1081
SLC16A7	-0.148684305	9.09894322088484e-07	3.92425243011509e-06	1081
EHBP1	-0.148716519	9.04915582919157e-07	3.90688478737814e-06	1081
ZNF626	-0.148723384	9.03857961501764e-07	3.90399198851909e-06	1081
C9orf82	-0.14874302	9.00839350583991e-07	3.89222523294616e-06	1081

SETMAR	-0.148815035	8.89851395641527e-07	3.84927128305745e-06	1081
PODN	-0.14882239	8.88736390854279e-07	3.84527375295521e-06	1081
FBN1	-0.14883433	8.86929386445308e-07	3.83827980449855e-06	1081
ALS2CR1	-0.148856102	8.83643253629738e-07	3.82488039200066e-06	1081
DUSP19	-0.14887826	8.80310920382446e-07	3.81209447358219e-06	1081
GOLGA6I	-0.14890643	8.76091835868973e-07	3.79463986961524e-06	1081
TNFRSF1!	-0.148971848	8.66369095520511e-07	3.75414184324816e-06	1081
FAM126B	-0.14897666	8.65658023306424e-07	3.75186765910239e-06	1081
GALNT4	-0.149008392	8.60982987638395e-07	3.7332118566456e-06	1081
ZFP36L1	-0.149035925	8.56946305553217e-07	3.71650878910001e-06	1081
FAM13C	-0.149107618	8.46520293652833e-07	3.67366465222669e-06	1081
SMG1	-0.149127151	8.43700931285046e-07	3.66379721076466e-06	1081
LIG4	-0.14914103	8.41703155315347e-07	3.65590988870799e-06	1081
EFHA1	-0.149143985	8.41278414686936e-07	3.65485306531557e-06	1081
MAML1	-0.149167616	8.37889062468795e-07	3.64091353827305e-06	1081
ERI2	-0.149179304	8.36217482324297e-07	3.63443391184961e-06	1081
ETAA1	-0.14920145	8.33058986662794e-07	3.62226921785495e-06	1081
LRAT	-0.149229581	8.29063583502161e-07	3.60567484664314e-06	1081
CNTN3	-0.149244643	8.26931909077442e-07	3.59873478253728e-06	1081
VPS13C	-0.149308281	8.1798339655533e-07	3.56244656508145e-06	1081
SLC24A2	-0.149311537	8.17528025992555e-07	3.56165702500433e-06	1081
ZNF142	-0.149313578	8.1724276526523e-07	3.56118440542247e-06	1081
HDHD2	-0.149348166	8.12422533429352e-07	3.54247877401175e-06	1081
GSPT2	-0.149374396	8.08785376584148e-07	3.52738285587522e-06	1081
SEMA6A	-0.149494303	7.9235676847488e-07	3.46182964788639e-06	1081
FBXO21	-0.149566959	7.82558756185142e-07	3.42040489115854e-06	1081
LRRC57	-0.14959724	7.7850973203858e-07	3.40344586630147e-06	1081
NCOA4	-0.149618178	7.757218736474e-07	3.39199417074601e-06	1081
GIPC2	-0.149634276	7.73584893905372e-07	3.38338421357441e-06	1081
IFT81	-0.149638433	7.7303389465914e-07	3.3817085359193e-06	1081
IL33	-0.149657417	7.70522891658974e-07	3.37145604962425e-06	1081
LPPR1	-0.149682085	7.67271778413026e-07	3.35868969494359e-06	1081
LRRN3	-0.149687983	7.66496392968019e-07	3.35602474165197e-06	1081

CCDC39	-0.149693177	7.6581418577489e-07	3.35376668965982e-06	1081
MDM4	-0.149747489	7.58715387271119e-07	3.32629411895031e-06	1081
HIVEP1	-0.149789168	7.53310894754557e-07	3.3033191064063e-06	1081
SOSTDC1	-0.149813351	7.50191978737996e-07	3.29035867878879e-06	1081
TXNDC16	-0.149820301	7.49297958689192e-07	3.28715317460666e-06	1081
TMEM35	-0.149821935	7.49087909502744e-07	3.28694749225284e-06	1081
EML5	-0.149832134	7.47778128782345e-07	3.28191512076696e-06	1081
RSL24D1	-0.149897013	7.39497812305682e-07	3.24769640917767e-06	1081
TSPAN11	-0.149954003	7.3229714763289e-07	3.2188797815982e-06	1081
RBM25	-0.150009325	7.25371744848733e-07	3.19143368758686e-06	1081
AK3	-0.150031662	7.22593352460632e-07	3.18038957511895e-06	1081
THAP2	-0.150066001	7.1834215695498e-07	3.16236948248647e-06	1081
TMEM20(	-0.150078173	7.16841106032721e-07	3.15645116525228e-06	1081
SHPK	-0.150113975	7.1244328372488e-07	3.13845833165049e-06	1081
RPL23AP(	-0.150121379	7.11536984823396e-07	3.13515147840492e-06	1081
KIAA0895	-0.150140072	7.09253944319963e-07	3.12645967359423e-06	1081
PCDHB3	-0.150141397	7.09092301821227e-07	3.12643125852235e-06	1081
ERCC8	-0.150168763	7.057634614462e-07	3.11243540517375e-06	1081
AKD1	-0.150208783	7.00922303912353e-07	3.0931171549429e-06	1081
ZBTB40	-0.15036641	6.82163363650991e-07	3.01562013621883e-06	1081
KIAA1797	-0.150380931	6.8045973235865e-07	3.00940972741273e-06	1081
RECK	-0.150406023	6.77525620776787e-07	2.99709126713842e-06	1081
GLIS3	-0.15046267	6.70946209344794e-07	2.96929072654896e-06	1081
FAM188A	-0.150495419	6.67170592719747e-07	2.95387947040424e-06	1081
NUP153	-0.150499955	6.66649223859584e-07	2.95221996365164e-06	1081
RGPD6	-0.150506645	6.65881109576779e-07	2.94946678813197e-06	1081
BTBD9	-0.150609333	6.54196228163807e-07	2.89898438547292e-06	1081
FBXO34	-0.150611123	6.53994198710273e-07	2.89872676194025e-06	1081
TUBGCP3	-0.150613026	6.5377962097062e-07	2.89841339446592e-06	1081
ARSJ	-0.15065074	6.4954052502441e-07	2.88025399001029e-06	1081
SOX9	-0.150651638	6.49439917588233e-07	2.88025399001029e-06	1081
RUFY2	-0.150669494	6.47442403555562e-07	2.8722147587815e-06	1081
LIMS1	-0.150670686	6.47309259604566e-07	2.8722147587815e-06	1081

TNNI1	-0.150686078	6.45592461164821e-07	2.86526991191128e-06	1081
PCNP	-0.150789081	6.34215942683401e-07	2.81850433826541e-06	1081
PLCB4	-0.150797875	6.33253699635597e-07	2.8148490245276e-06	1081
DIP2B	-0.15083391	6.29325173263559e-07	2.79862154865218e-06	1081
C1orf55	-0.150834705	6.29238796384175e-07	2.79862154865218e-06	1081
ZNF643	-0.15085957	6.26542107283103e-07	2.78809161723395e-06	1081
PCDHGB7	-0.150862577	6.26216641475691e-07	2.78725900188418e-06	1081
ZNF354B	-0.150863342	6.26133995869491e-07	2.78725900188418e-06	1081
PM20D2	-0.150882848	6.24027571339912e-07	2.77935782105738e-06	1081
CALD1	-0.150906695	6.21461626997658e-07	2.76915383230874e-06	1081
SPOPL	-0.150928868	6.19084957530863e-07	2.75978456947538e-06	1081
JAM2	-0.150944185	6.17448330673119e-07	2.75309796843957e-06	1081
ZNF528	-0.150954076	6.16393606858432e-07	2.74900358870116e-06	1081
KCNS2	-0.151005171	6.10972920839846e-07	2.72663923135106e-06	1081
ATP8B1	-0.151030772	6.08274206065616e-07	2.71519696015773e-06	1081
LRP12	-0.15107237	6.03913513664217e-07	2.69632928474416e-06	1081
AGFG1	-0.151077143	6.03415164718357e-07	2.69529900072091e-06	1081
SNX9	-0.151133026	5.97609065247787e-07	2.67114147313438e-06	1081
RGS5	-0.151150008	5.95855339776513e-07	2.66389387922722e-06	1081
RHBDD1	-0.151168697	5.93931074682494e-07	2.65647013753971e-06	1081
NAB1	-0.151182431	5.92520803995519e-07	2.65075096524311e-06	1081
HS6ST2	-0.151225654	5.88103441627518e-07	2.63274307368586e-06	1081
MTHFR	-0.15127781	5.82815359031092e-07	2.61023019290381e-06	1081
SFMBT2	-0.151284711	5.82119111395498e-07	2.60769168313593e-06	1081
LRRC49	-0.151346264	5.75944254235595e-07	2.58175278183713e-06	1081
ANKRD36	-0.151360037	5.7457117513524e-07	2.5761710044735e-06	1081
HOXA9	-0.151373311	5.73250869083481e-07	2.57139584896164e-06	1081
CREBL2	-0.151387132	5.71879315019429e-07	2.5663339282995e-06	1081
TMEM133	-0.151398297	5.70773648825695e-07	2.56199535552443e-06	1081
HS2ST1	-0.151435502	5.671039017774e-07	2.54665806984078e-06	1081
PTPN9	-0.151435973	5.67057631295083e-07	2.54665806984078e-06	1081
DENND2C	-0.151439454	5.66715459550787e-07	2.54604882530121e-06	1081
CTTNBP2	-0.151467847	5.63932024158056e-07	2.53410899546376e-06	1081

DMTF1	-0.151473627	5.63367019525276e-07	2.53213489699614e-06	1081
TARBP1	-0.151518071	5.5904072866949e-07	2.51549597477035e-06	1081
RBMS2	-0.151551937	5.5576562246887e-07	2.50243520167965e-06	1081
SAMD5	-0.151554469	5.55521466184776e-07	2.50189580511789e-06	1081
NHSL2	-0.15156002	5.54986643840853e-07	2.5000460510228e-06	1081
MSH3	-0.151572494	5.53786639096469e-07	2.49519835486432e-06	1081
ZC3H7B	-0.151594298	5.5169493571159e-07	2.4863298612774e-06	1081
NEO1	-0.151606566	5.50521432148403e-07	2.48159638635703e-06	1081
LOC15476	-0.151619068	5.49327966070475e-07	2.47677078703888e-06	1081
SRPK2	-0.151619772	5.49260887092763e-07	2.47677078703888e-06	1081
RXRA	-0.151658311	5.45598072816775e-07	2.46160653457871e-06	1081
FOXJ2	-0.151682475	5.43313493654198e-07	2.45294718280229e-06	1081
HPGD	-0.151709148	5.40802414646267e-07	2.44215750796885e-06	1081
SPHAR	-0.151739934	5.37917968596206e-07	2.42967656443286e-06	1081
RNF146	-0.15177498	5.34652422020185e-07	2.41655217446637e-06	1081
RPGR	-0.151793961	5.3289177566212e-07	2.40967560509841e-06	1081
POC1B	-0.151805573	5.31817463298509e-07	2.40535761072035e-06	1081
CA3	-0.151811361	5.31282702091107e-07	2.40347856133513e-06	1081
SPTLC2	-0.151868614	5.2602115415216e-07	2.38128003379669e-06	1081
C12orf49	-0.151871998	5.25711770583556e-07	2.38041438939216e-06	1081
FDXACB1	-0.151877468	5.25211983460312e-07	2.37868601771762e-06	1081
ECM2	-0.151945067	5.19073264575272e-07	2.35194127639875e-06	1081
ALS2CR4	-0.151949558	5.18667885764628e-07	2.35116214192809e-06	1081
HINT3	-0.151997971	5.14317341856886e-07	2.33196553043146e-06	1081
PLD5	-0.152038973	5.10660171612198e-07	2.31590480799814e-06	1081
MRI1	-0.152088396	5.06285261699421e-07	2.29709833264298e-06	1081
RPL17	-0.152091268	5.06032160756787e-07	2.29646719496406e-06	1081
UBN1	-0.152092046	5.05963597855741e-07	2.29646719496406e-06	1081
JAG1	-0.152109265	5.04448844815024e-07	2.29134655666261e-06	1081
TRMT61B	-0.152152566	5.00658729950232e-07	2.27515680461255e-06	1081
SLC40A1	-0.152156259	5.00336828353785e-07	2.27420699620645e-06	1081
C19orf55	-0.15219908	4.9661806136393e-07	2.25934300952492e-06	1081
ESYT2	-0.152203454	4.96239726643231e-07	2.25813175812692e-06	1081

C14orf49	-0.152276487	4.89963188854467e-07	2.23057817841203e-06	1081
TWF1	-0.152294828	4.88398956568647e-07	2.22496539576558e-06	1081
SLC25A46	-0.152295737	4.88321574591191e-07	2.22496539576558e-06	1081
SCN4A	-0.15233012	4.85402630885215e-07	2.21281647413049e-06	1081
TAL1	-0.152345445	4.84107107784835e-07	2.20741006933579e-06	1081
RBMX	-0.152405953	4.79024139445325e-07	2.18621234461397e-06	1081
PTPRM	-0.152417752	4.78038983405031e-07	2.18221058705968e-06	1081
C11orf42	-0.152449383	4.75407570734472e-07	2.17118238776829e-06	1081
MAPKSP1	-0.152457559	4.74729683932197e-07	2.16906996661695e-06	1081
C10orf72	-0.15245796	4.74696439837438e-07	2.16906996661695e-06	1081
FBXW7	-0.152459898	4.74535962683508e-07	2.16906996661695e-06	1081
TRIM32	-0.152460969	4.74447226726736e-07	2.16906996661695e-06	1081
ZNF669	-0.152466806	4.73964149488009e-07	2.16830597291159e-06	1081
PDS5A	-0.152504109	4.70887760421731e-07	2.15640689558894e-06	1081
ELMOD2	-0.152508649	4.70514679269917e-07	2.1551883160283e-06	1081
RPS4X	-0.152515143	4.69981540711515e-07	2.15323587392165e-06	1081
FAM38B	-0.152523014	4.69336094342016e-07	2.15076788455867e-06	1081
CNTNAP3	-0.152542818	4.67715838848282e-07	2.1438306197039e-06	1081
TADA1	-0.152568785	4.65599619725106e-07	2.13461637218076e-06	1081
ZNF415	-0.152591451	4.63759898746311e-07	2.12666586848269e-06	1081
TAS2R31	-0.152638343	4.59976072099799e-07	2.11075580238051e-06	1081
GARNL3	-0.15268175	4.56499960048205e-07	2.09575927419578e-06	1081
SDPR	-0.152689877	4.55851993928007e-07	2.09326154950529e-06	1081
HTR1B	-0.152696962	4.55287727469448e-07	2.09114712035386e-06	1081
MOSPD2	-0.152698812	4.55140583540526e-07	2.09094801720043e-06	1081
ABI2	-0.152787673	4.48123405757619e-07	2.06059027824406e-06	1081
RBBP6	-0.152789521	4.47978569582913e-07	2.0603945854447e-06	1081
KLHL31	-0.1528101	4.46368891917206e-07	2.05392903784196e-06	1081
C17orf39	-0.152815225	4.45968875611122e-07	2.05255723079416e-06	1081
HP1BP3	-0.152855429	4.42842989982777e-07	2.03956836607294e-06	1081
MAB21L1	-0.152858208	4.4262767366733e-07	2.03933263498661e-06	1081
SFRS11	-0.152870127	4.41705481333302e-07	2.03572567409274e-06	1081
USP8	-0.152892484	4.39980632475776e-07	2.02824023826648e-06	1081

TRMT5	-0.152898794	4.39494934853948e-07	2.02646497199194e-06	1081
EFNB3	-0.152944028	4.36028618246445e-07	2.01278563578704e-06	1081
SP3	-0.152949698	4.35595944303939e-07	2.01124920880194e-06	1081
CPEB4	-0.152951917	4.35426732723439e-07	2.01092882409759e-06	1081
ZNF765	-0.152952511	4.3538147066513e-07	2.01092882409759e-06	1081
MEF2D	-0.153028817	4.29601934841048e-07	1.98676101408928e-06	1081
SLC17A7	-0.153105741	4.23850409428739e-07	1.96106258565502e-06	1081
HOXA3	-0.153147929	4.20727609556939e-07	1.94706126683311e-06	1081
THBS4	-0.15315879	4.19927260264319e-07	1.94380391958288e-06	1081
SLAIN2	-0.153188821	4.17721932048624e-07	1.93404006461033e-06	1081
UTP20	-0.15320734	4.16367595840855e-07	1.9282126938423e-06	1081
PWWP2A	-0.153237254	4.14188736447105e-07	1.91856336990732e-06	1081
RABGEF1	-0.153258459	4.12650888176326e-07	1.91187951755108e-06	1081
PAK3	-0.153273566	4.11558705268495e-07	1.90725790605793e-06	1081
CCL16	-0.153339485	4.0682524814057e-07	1.88575578090009e-06	1081
GHR	-0.153374914	4.04302957727846e-07	1.87449553128365e-06	1081
RBPMS	-0.153421942	4.00978106214706e-07	1.85950827571253e-06	1081
ZNF187	-0.153571916	3.90550162997121e-07	1.81323646775225e-06	1081
STK32A	-0.153578915	3.90069929744142e-07	1.81142432796121e-06	1081
TSPAN9	-0.153580581	3.89955730540897e-07	1.81131154986082e-06	1081
SEC1	-0.153611349	3.87851984296293e-07	1.80195530988211e-06	1081
SYNE2	-0.153681507	3.83095846541523e-07	1.7815018071512e-06	1081
ZNF596	-0.153681629	3.83087598181034e-07	1.7815018071512e-06	1081
CIC	-0.153725761	3.80124765936273e-07	1.76931917971031e-06	1081
PCYOX1	-0.153747412	3.78679301586501e-07	1.76340604032826e-06	1081
NCRNA0(	-0.153776227	3.76763678331153e-07	1.75529701664687e-06	1081
BCAR3	-0.153806352	3.74771061787433e-07	1.74722588282986e-06	1081
PPP2CB	-0.153811965	3.74400895246438e-07	1.74630841276673e-06	1081
ZNF562	-0.153818339	3.73980978584421e-07	1.74475377804149e-06	1081
CDH8	-0.153835328	3.72863990338833e-07	1.73994558382576e-06	1081
SLC25A1:	-0.153848119	3.7202509576702e-07	1.73643316826381e-06	1081
WWC1	-0.153850852	3.71846060737246e-07	1.73599974358095e-06	1081
BHLHB9	-0.153854494	3.71607651871925e-07	1.73528886114046e-06	1081

RNF186	-0.153860988	3.7118296108991e-07	1.73370757040488e-06	1081
LOC10012	-0.15393838	3.66157022577203e-07	1.71301282392423e-06	1081
SPOCK1	-0.153942582	3.65886031153772e-07	1.71214264752444e-06	1081
MAP4K3	-0.154031405	3.60202595399102e-07	1.68672275321128e-06	1081
YTHDC1	-0.154064763	3.58090255841395e-07	1.67722115878282e-06	1081
SLC26A3	-0.154111152	3.55172499079965e-07	1.66432891229725e-06	1081
SYNPO	-0.154143744	3.5313631635741e-07	1.65517242741276e-06	1081
LOC10013	-0.15415757	3.52275932682249e-07	1.65152400835092e-06	1081
ZKSCAN3	-0.154166614	3.51714190363571e-07	1.64927429349956e-06	1081
DSC2	-0.154200647	3.49608228002851e-07	1.63978061772233e-06	1081
MAP2K4	-0.154208068	3.4915058608588e-07	1.63801549993015e-06	1081
TRAM2	-0.154218176	3.48528243958936e-07	1.63579818027908e-06	1081
GPD1L	-0.154218769	3.4849175462407e-07	1.63579818027908e-06	1081
MYOM3	-0.154264642	3.45680931117625e-07	1.62362843491829e-06	1081
KLF5	-0.154412821	3.36750019254056e-07	1.58352687625419e-06	1081
SLC25A16	-0.154433629	3.35513852161592e-07	1.57845085282468e-06	1081
PER3	-0.154496484	3.31806282527086e-07	1.56137294125395e-06	1081
ZNF543	-0.154500181	3.31589400080784e-07	1.56071693098771e-06	1081
BMP5	-0.154516984	3.30605575560716e-07	1.55644994617215e-06	1081
CCDC144	-0.15458276	3.26781360777658e-07	1.5398855000856e-06	1081
PHF17	-0.154620225	3.24622287661844e-07	1.53114399085644e-06	1081
LRRC4C	-0.154632989	3.23889804989405e-07	1.52804686686454e-06	1081
ITGB3	-0.154640112	3.23481761046437e-07	1.52683694383329e-06	1081
CHST3	-0.154691292	3.20564397182898e-07	1.51377632003035e-06	1081
PPP1R12A	-0.154696898	3.2024640574802e-07	1.51262927169844e-06	1081
TIMP3	-0.154710841	3.19456765246113e-07	1.50960744449518e-06	1081
PDGFRA	-0.154827997	3.12895731351227e-07	1.47929699790436e-06	1081
ANTXR1	-0.154865145	3.10842626854395e-07	1.47062581446261e-06	1081
CPEB1	-0.154902833	3.08772901920638e-07	1.46117691078019e-06	1081
GBAS	-0.15499209	3.03924134903486e-07	1.43992278871842e-06	1081
DNAH7	-0.155102769	2.98013478519561e-07	1.41358171056665e-06	1081
TRIM58	-0.155157718	2.95120349458395e-07	1.40117827948135e-06	1081
KDM5A	-0.155163195	2.94833441601629e-07	1.40014608228779e-06	1081

PEG3	-0.155177515	2.94084640182371e-07	1.39691937667387e-06	1081
SNX16	-0.155185202	2.93683454960014e-07	1.39534273588903e-06	1081
FRZB	-0.155219148	2.91918039857962e-07	1.38793696316702e-06	1081
RBM7	-0.155249702	2.90337804790049e-07	1.38140176605941e-06	1081
EXPH5	-0.155283265	2.88611397789053e-07	1.37351207381537e-06	1081
LMAN1	-0.155353177	2.85047104463384e-07	1.35751156487349e-06	1081
HNMT	-0.155359435	2.84730112081285e-07	1.3563225603872e-06	1081
SLC35D1	-0.155391416	2.83115534472717e-07	1.34926956279936e-06	1081
NEDD9	-0.155410599	2.82151340236867e-07	1.34531094652584e-06	1081
SUFU	-0.155419444	2.81707834866046e-07	1.34351428346981e-06	1081
CCDC15	-0.15542172	2.81593780286641e-07	1.34328835043201e-06	1081
TBC1D4	-0.155462226	2.79572043663614e-07	1.33427595820979e-06	1081
SGMS2	-0.155481594	2.78610326076564e-07	1.33000118929203e-06	1081
C9orf109	-0.155502362	2.77582591541752e-07	1.32572340128227e-06	1081
BAHCC1	-0.155513669	2.770245927863e-07	1.32359660109979e-06	1081
SSTR1	-0.155515239	2.76947182585655e-07	1.32359660109979e-06	1081
ZNF37B	-0.155540398	2.75709808594102e-07	1.31802897345234e-06	1081
TNR	-0.155627373	2.71473124938629e-07	1.29900857527047e-06	1081
GALNT7	-0.155636693	2.71022875260235e-07	1.29716222906093e-06	1081
MPRIP	-0.155662704	2.69770099067057e-07	1.29178004414211e-06	1081
FAM35A	-0.155705072	2.67741457597412e-07	1.28267578199759e-06	1081
PANK3	-0.15571236	2.67393984769027e-07	1.28131584756709e-06	1081
OMD	-0.155747601	2.65719902994677e-07	1.27359682270468e-06	1081
FZD8	-0.1557686	2.64727184844364e-07	1.26944278473928e-06	1081
LOC65350	-0.155782836	2.64056225907901e-07	1.26672457259807e-06	1081
VWA2	-0.155814729	2.62559031141746e-07	1.26024581423647e-06	1081
DUSP16	-0.155821455	2.6224431214139e-07	1.25903519258539e-06	1081
ZNF396	-0.155887025	2.59195337924034e-07	1.24469370261732e-06	1081
RNF11	-0.155944831	2.5653576701793e-07	1.23250966529363e-06	1081
PIAS1	-0.155968919	2.55435300705781e-07	1.22751529883539e-06	1081
EPHA3	-0.155990102	2.54471318307192e-07	1.22346651725499e-06	1081
ZNF181	-0.155992009	2.54384692924603e-07	1.22334200023064e-06	1081
DTX4	-0.156029943	2.52667754390372e-07	1.21537533720011e-06	1081

HEG1	-0.156031339	2.52604779675578e-07	1.21536261919382e-06	1081
LRRC58	-0.156062525	2.51201941877279e-07	1.20890184403196e-06	1081
HEY2	-0.156063635	2.51152151717846e-07	1.20890184403196e-06	1081
ARHGAP2	-0.156093415	2.49819847248346e-07	1.20282524445935e-06	1081
SNAP91	-0.156102873	2.49398160906644e-07	1.20108198696255e-06	1081
LGTN	-0.156120822	2.48599748032558e-07	1.1975231764983e-06	1081
FAM106C	-0.156148088	2.47391627056042e-07	1.19227376245071e-06	1081
RBBP5	-0.156156495	2.47020270067566e-07	1.19076892570259e-06	1081
CTBP2	-0.156188218	2.45623778345332e-07	1.18545544196615e-06	1081
ZNF429	-0.15621819	2.44311393346647e-07	1.17968672554367e-06	1081
PPTC7	-0.1562393	2.43391149700523e-07	1.17580688506404e-06	1081
GALNTL2	-0.15625208	2.42835600660289e-07	1.17340445557724e-06	1081
TMEM117	-0.156266646	2.42203962440266e-07	1.17063311500939e-06	1081
METTL7A	-0.156312566	2.40223022992312e-07	1.16161613014405e-06	1081
UBR1	-0.156316957	2.4003442426737e-07	1.16098282757891e-06	1081
TMEM30A	-0.156381838	2.37264194445913e-07	1.14868714181998e-06	1081
IGSF10	-0.15640279	2.36376259169597e-07	1.14493862490299e-06	1081
ETV3L	-0.156402861	2.36373243387078e-07	1.14493862490299e-06	1081
INHBB	-0.156409898	2.36075724451283e-07	1.14403306929784e-06	1081
ARHGAP2	-0.156481386	2.33073748015434e-07	1.1303010721644e-06	1081
EBF3	-0.15653437	2.30872608117879e-07	1.12043572405075e-06	1081
CDC42BP	-0.156615906	2.27524400101302e-07	1.10471897807682e-06	1081
PIAS3	-0.156625186	2.27146318058802e-07	1.10314912663804e-06	1081
BMP2K	-0.156631273	2.26898669707725e-07	1.1022121295544e-06	1081
FAM10A4	-0.156647499	2.262397091263e-07	1.09927615541469e-06	1081
MUM1L1	-0.156673368	2.25192997496437e-07	1.09471837224077e-06	1081
MAK	-0.156685157	2.24717542481894e-07	1.09267074421862e-06	1081
NRP1	-0.156704214	2.23950997343731e-07	1.08920638374927e-06	1081
C11orf57	-0.156782036	2.2084684383933e-07	1.07488757408632e-06	1081
NOTCH4	-0.156909475	2.15853080011978e-07	1.05236212411455e-06	1081
DOCK7	-0.156913981	2.15678512571069e-07	1.05176558599472e-06	1081
ISLR2	-0.156917095	2.15557945796443e-07	1.05143215933882e-06	1081
ZBTB24	-0.15695382	2.14141050204968e-07	1.04481405537027e-06	1081

ACSL3	-0.156954979	2.14096492563992e-07	1.04481405537027e-06	1081
KRCC1	-0.156968865	2.13563235144636e-07	1.04271240232397e-06	1081
AHI1	-0.156975724	2.13300271071617e-07	1.04168096017884e-06	1081
LOC15869	-0.157088092	2.09036840086915e-07	1.02160289751356e-06	1081
ZNF25	-0.157125237	2.07645687305305e-07	1.01505032049633e-06	1081
GUSBP1	-0.15722503	2.03952297091133e-07	9.97721958450917e-07	1081
FIGN	-0.157290829	2.01551821666916e-07	9.86218471576395e-07	1081
ZNF828	-0.157309719	2.00867718758783e-07	9.83109862583985e-07	1081
GPR153	-0.157358801	1.99100689018929e-07	9.75409382365351e-07	1081
COG5	-0.157380262	1.98332801821957e-07	9.71883797787235e-07	1081
TSSK4	-0.157381465	1.98289830815605e-07	9.71883797787235e-07	1081
TMEM2	-0.157403673	1.97498363003129e-07	9.68265885300083e-07	1081
AASDH	-0.157419635	1.96931413326092e-07	9.65721353810645e-07	1081
DTWD1	-0.157435637	1.96364566105475e-07	9.63176085754759e-07	1081
TRIM44	-0.157438049	1.96279282119029e-07	9.6299224020649e-07	1081
MCPH1	-0.15744524	1.9602516885228e-07	9.61979787217825e-07	1081
RCOR3	-0.157467033	1.95257036274087e-07	9.58443712412642e-07	1081
CLK4	-0.157476676	1.94918082774322e-07	9.57013106870272e-07	1081
LOC64676	-0.157489573	1.94465628188265e-07	9.55024397818769e-07	1081
C10orf107	-0.157510615	1.93729643828195e-07	9.5164195925847e-07	1081
CD46	-0.157602827	1.90535795115715e-07	9.37095603663592e-07	1081
SGSM2	-0.157614568	1.90132825664163e-07	9.35570535663059e-07	1081
MAP1B	-0.157621781	1.89885639539895e-07	9.34582508803122e-07	1081
CAT	-0.157653985	1.88785911388446e-07	9.29814457557146e-07	1081
KLHL11	-0.157655648	1.88729281644568e-07	9.29799799151337e-07	1081
METT5D1	-0.15770943	1.86906869890196e-07	9.21272056260826e-07	1081
AFTPH	-0.157744508	1.857273988985e-07	9.15906597505578e-07	1081
DHX9	-0.157749721	1.85552756958375e-07	9.15269414526556e-07	1081
ZNF559	-0.157762987	1.85108980405155e-07	9.13494094078937e-07	1081
RAPGEF6	-0.157784556	1.84389624923879e-07	9.1020068465855e-07	1081
RPL32P3	-0.157803522	1.8375931628658e-07	9.07534058983365e-07	1081
SLC16A9	-0.157845696	1.82365188706483e-07	9.01522406212071e-07	1081
LYST	-0.157915155	1.80091347697146e-07	8.90510603671822e-07	1081

KLF9	-0.158000254	1.77342813362047e-07	8.77350435947553e-07	1081
TMEM233	-0.158017192	1.76800576976178e-07	8.74882737210785e-07	1081
EPS8	-0.158025338	1.76540391596922e-07	8.73809874378379e-07	1081
C9orf129	-0.158025461	1.76536477696589e-07	8.73809874378379e-07	1081
PIWIL2	-0.158139561	1.72930612180318e-07	8.57417470433792e-07	1081
C1orf95	-0.1581438	1.72798045427565e-07	8.56971104169939e-07	1081
NT5DC1	-0.15815234	1.72531225218734e-07	8.55858540268753e-07	1081
ERBB3	-0.158179159	1.71695920462024e-07	8.51924708794945e-07	1081
SNX18	-0.158195613	1.71185374335367e-07	8.49600730718394e-07	1081
PDLIM5	-0.158263336	1.69099434911295e-07	8.40283205793795e-07	1081
LYSMD3	-0.158264677	1.69058388401026e-07	8.40283205793795e-07	1081
PYROXD1	-0.158316423	1.67481476163637e-07	8.33271014402684e-07	1081
FOXO1	-0.158366705	1.65962794366146e-07	8.25919093998518e-07	1081
TAS2R10	-0.158375758	1.65690765978661e-07	8.24769083429732e-07	1081
FEZ1	-0.158440542	1.63756757463638e-07	8.15343519304247e-07	1081
PYGO1	-0.15844248	1.6369922621435e-07	8.15258569119429e-07	1081
EDNRA	-0.158449717	1.63484609432378e-07	8.14391062565593e-07	1081
DIP2A	-0.158488247	1.62346526416392e-07	8.0892178448138e-07	1081
TMEM67	-0.158518086	1.61470441854627e-07	8.04755579208675e-07	1081
CCDC121	-0.15852569	1.61247895738488e-07	8.03845300581996e-07	1081
KCNJ16	-0.158534073	1.61002927885684e-07	8.0282276788542e-07	1081
PIK3R1	-0.158535691	1.60955671492434e-07	8.02785838627155e-07	1081
LOC28317	-0.158536818	1.60922779570514e-07	8.02785838627155e-07	1081
LPAR4	-0.15870252	1.56155732374327e-07	7.79617652709e-07	1081
VPS53	-0.158713097	1.55856134131884e-07	7.78314779892612e-07	1081
RAB14	-0.158766793	1.54343654258231e-07	7.71144076148825e-07	1081
UNC5B	-0.158825907	1.52694924068476e-07	7.63285172545771e-07	1081
PPM1A	-0.158856894	1.51837503106539e-07	7.59187515532696e-07	1081
CCDC75	-0.158857662	1.518163093238e-07	7.59187515532696e-07	1081
NSD1	-0.158870936	1.51450487559572e-07	7.57816709361047e-07	1081
MAML2	-0.158880805	1.51179056445329e-07	7.56834515927225e-07	1081
METTL9	-0.158894618	1.50799916601897e-07	7.55311864730286e-07	1081
ZNF322A	-0.158952334	1.49225737061064e-07	7.47799122660481e-07	1081

CDH13	-0.158973645	1.48648495318951e-07	7.4527723698364e-07	1081
CLVS2	-0.15900814	1.47718738193791e-07	7.41353757078705e-07	1081
POGK	-0.159059876	1.46334772057886e-07	7.34591074783483e-07	1081
ASXL1	-0.159074966	1.45933464836603e-07	7.32759134878704e-07	1081
RPP14	-0.159079094	1.4582388411513e-07	7.3239145986021e-07	1081
ENPP3	-0.159094306	1.45420697142906e-07	7.30548614449836e-07	1081
DSP	-0.159118478	1.4478227614074e-07	7.27522811887057e-07	1081
SFRS18	-0.159138009	1.44268383119208e-07	7.2512140168075e-07	1081
SLC14A1	-0.159192868	1.42834405434838e-07	7.18093111426206e-07	1081
TC2N	-0.159197166	1.42722626970964e-07	7.17710264685491e-07	1081
CYTH3	-0.159201578	1.42607994502239e-07	7.17312871222873e-07	1081
C21orf34	-0.159211151	1.4235956756951e-07	7.16242130041903e-07	1081
UBXN2B	-0.159231781	1.41825592637758e-07	7.13733840541502e-07	1081
GNPTAB	-0.159242999	1.41536050304796e-07	7.12632775153741e-07	1081
LRFN5	-0.159267786	1.40898314539836e-07	7.09599136601249e-07	1081
PDZRN3	-0.159312029	1.39766867722343e-07	7.0407690679335e-07	1081
ADCY6	-0.159340663	1.3903930792747e-07	7.00587008053748e-07	1081
TRHDE	-0.159343383	1.38970377173527e-07	7.00414873195071e-07	1081
CYB5RL	-0.159375143	1.38168027465464e-07	6.96545273596537e-07	1081
CHL1	-0.159402183	1.37488453720557e-07	6.93292841101533e-07	1081
SYPL1	-0.159407945	1.37344059905366e-07	6.92738128891736e-07	1081
ZNF483	-0.159431337	1.36759351277143e-07	6.89963868573821e-07	1081
ATP8A2	-0.159482194	1.35496440406668e-07	6.84275706190106e-07	1081
ZNF609	-0.15958954	1.32867645138399e-07	6.71673453278056e-07	1081
SEPT7	-0.15959439	1.32750044247207e-07	6.71247399939754e-07	1081
SPDYE1	-0.159602645	1.32550107235531e-07	6.70404697529445e-07	1081
WWC2	-0.159635956	1.31746283723495e-07	6.665065006554e-07	1081
FZD7	-0.159660444	1.31158367580184e-07	6.63698898493548e-07	1081
TEK	-0.159672246	1.30875936618177e-07	6.62436116375169e-07	1081
ZMIZ1	-0.159686712	1.30530545395357e-07	6.60853942445203e-07	1081
ABLIM1	-0.159727305	1.29566026366e-07	6.56300628901954e-07	1081
SATB1	-0.159744496	1.2915964043072e-07	6.54571309805499e-07	1081
GSK3B	-0.159748806	1.29057946198522e-07	6.54220514889085e-07	1081

PRICKLE	-0.159758615	1.28826793957963e-07	6.53213129696239e-07	1081
SH2D4A	-0.159761089	1.28768556960057e-07	6.53082220533825e-07	1081
TAOK1	-0.159781803	1.28281963286066e-07	6.50778179400101e-07	1081
FRK	-0.159812527	1.27563474995924e-07	6.47949118455091e-07	1081
RPRD1A	-0.159832085	1.27108128646031e-07	6.45799054621514e-07	1081
MLLT4	-0.15986428	1.26361999153733e-07	6.42170149584248e-07	1081
ADAMTS	-0.159875815	1.26095697913955e-07	6.40978509835131e-07	1081
CD93	-0.159915825	1.2517622578849e-07	6.36625869353477e-07	1081
PPIL4	-0.159964139	1.24074524728105e-07	6.311821466282e-07	1081
TCF7L2	-0.159973523	1.23861637787658e-07	6.30417557663562e-07	1081
BMI1	-0.160033329	1.22513123789815e-07	6.24027023703118e-07	1081
ZNF136	-0.160063092	1.21847313888013e-07	6.20792650044013e-07	1081
PLEKHA1	-0.160066916	1.21762029107807e-07	6.20672084103433e-07	1081
TMEM151	-0.160067828	1.21741708094346e-07	6.20672084103433e-07	1081
B3GALT1	-0.160074148	1.21600894141376e-07	6.20164560121015e-07	1081
NFIA	-0.160075998	1.21559705877918e-07	6.2011149022807e-07	1081
C6	-0.160079479	1.21482243560564e-07	6.19873302058652e-07	1081
DZIP1	-0.160151271	1.19895193322429e-07	6.12551019396485e-07	1081
TTLL11	-0.160155438	1.19803701587966e-07	6.12238855527545e-07	1081
PPP1R13E	-0.16023674	1.18031934558017e-07	6.03490690779504e-07	1081
CDK5RAI	-0.160380167	1.14967842704427e-07	5.88421542500171e-07	1081
MSL2	-0.160397029	1.14612702756985e-07	5.86752959857553e-07	1081
RPL7L1	-0.160409002	1.14361185525868e-07	5.85763051720978e-07	1081
PRND	-0.160435034	1.13816153054834e-07	5.83267973362916e-07	1081
RAI1	-0.160514904	1.12159516826335e-07	5.7536375514808e-07	1081
GRIN2A	-0.16054995	1.11439988371789e-07	5.719639657961e-07	1081
TSHZ3	-0.160557908	1.11277224925408e-07	5.71274132549016e-07	1081
PIGK	-0.160698012	1.08449075132425e-07	5.58320117184437e-07	1081
ANKRD57	-0.160699462	1.08420163897599e-07	5.58313957494156e-07	1081
ITGBL1	-0.160719985	1.08011875978767e-07	5.56638332461567e-07	1081
ZDBF2	-0.160750263	1.07412204135127e-07	5.53831290581556e-07	1081
TAC1	-0.160754978	1.07319126655687e-07	5.53493038013007e-07	1081
ZFPM2	-0.160770307	1.07017015215922e-07	5.52076253911589e-07	1081

IGF2	-0.160776151	1.06902043476129e-07	5.51624402107227e-07	1081
CX3CR1	-0.160781534	1.06796270568008e-07	5.51219797743409e-07	1081
RIF1	-0.160904848	1.04400439602091e-07	5.39406733979002e-07	1081
ARGLU1	-0.160924646	1.04020673421857e-07	5.37582469492894e-07	1081
ARMCX5	-0.160943232	1.03665366845383e-07	5.35883709289258e-07	1081
TTC19	-0.160970852	1.03139515472593e-07	5.33576152849357e-07	1081
LMBRD2	-0.160973372	1.03091667872209e-07	5.33465617591999e-07	1081
PHC3	-0.160980336	1.02959545710617e-07	5.32918820231342e-07	1081
NIPAL2	-0.161010817	1.02383182591053e-07	5.30071758750133e-07	1081
GDF10	-0.161020748	1.02196091339725e-07	5.29239141398141e-07	1081
HOXA2	-0.161115027	1.00436196032639e-07	5.20794638115192e-07	1081
STAT5B	-0.161126539	1.00223320741139e-07	5.19824612855367e-07	1081
DNAJB14	-0.16115249	9.97450397525998e-08	5.17610465176745e-07	1081
RAB6C	-0.161192226	9.90169668938189e-08	5.14097112906181e-07	1081
HIP1	-0.161201873	9.88409893124252e-08	5.13315733358805e-07	1081
USP47	-0.161227621	9.83727727778124e-08	5.11147667683526e-07	1081
ZFYVE20	-0.161229688	9.83352741832732e-08	5.11084648715696e-07	1081
BTBD3	-0.161438126	9.46244081581144e-08	4.92687697685504e-07	1081
FLJ16779	-0.161484608	9.38155382033347e-08	4.89108182480895e-07	1081
TECPR2	-0.161529466	9.30412722990048e-08	4.85197108584896e-07	1081
POM121C	-0.161579183	9.21903436815501e-08	4.8100866963606e-07	1081
CCNY	-0.161589981	9.20065311642965e-08	4.80173981944236e-07	1081
C5orf42	-0.161591147	9.1986706350105e-08	4.80173981944236e-07	1081
MYCBP2	-0.161684734	9.04086667916183e-08	4.72201864795735e-07	1081
RHPN2	-0.161712387	8.99474375299774e-08	4.70158570067824e-07	1081
BAG5	-0.161715635	8.98934018372976e-08	4.69998074231082e-07	1081
SBF2	-0.161749923	8.93249742408073e-08	4.67268659070647e-07	1081
ZCCHC14	-0.161760668	8.91475725081818e-08	4.66461778747356e-07	1081
PIAS2	-0.161780604	8.88192976359637e-08	4.6498564211967e-07	1081
ZNF146	-0.161840039	8.78475537114733e-08	4.60137537576087e-07	1081
PCYT1B	-0.161890139	8.70364345152658e-08	4.56007535321204e-07	1081
PLN	-0.161912925	8.66699234029408e-08	4.54441855010995e-07	1081
EPB41L4A	-0.161936813	8.62872934369165e-08	4.52671230803824e-07	1081

DDX46	-0.162026079	8.48718701597405e-08	4.45477807287121e-07	1081
PVRL1	-0.162067371	8.42247514851004e-08	4.42196408305277e-07	1081
MATR3	-0.162137816	8.31317452826448e-08	4.37027403110354e-07	1081
ZNF254	-0.162146363	8.30000655759488e-08	4.36449052734922e-07	1081
CREBZF	-0.162190154	8.23285796842216e-08	4.33144225055796e-07	1081
RNF217	-0.162198311	8.22040802311066e-08	4.32602193379217e-07	1081
LOC1001!	-0.162228533	8.17444036779566e-08	4.30408001069638e-07	1081
KL	-0.162261508	8.12456734178578e-08	4.28005776935865e-07	1081
GLI2	-0.162348073	7.9950405664999e-08	4.21402648383413e-07	1081
ZZEF1	-0.162433493	7.86918656682453e-08	4.14877684869616e-07	1081
TMEM30I	-0.162439045	7.86107282430235e-08	4.14666960056483e-07	1081
DNAJC16	-0.162464742	7.8236253555189e-08	4.12799719190488e-07	1081
HERPUD2	-0.162476132	7.80708259678059e-08	4.12034788871221e-07	1081
C4orf31	-0.162476354	7.80675994373384e-08	4.12034788871221e-07	1081
CHD9	-0.16247806	7.80428547239927e-08	4.12034788871221e-07	1081
CLCN6	-0.162489348	7.78792973542375e-08	4.11486185627536e-07	1081
NAALAD2	-0.162534208	7.72325614388606e-08	4.08466776105499e-07	1081
FRRS1	-0.16257025	7.67167301131203e-08	4.0605846771645e-07	1081
ZNF131	-0.162583887	7.65224168525158e-08	4.05136422468838e-07	1081
RNF128	-0.162632835	7.5828898550317e-08	4.01675824690017e-07	1081
TTC23L	-0.162655137	7.55149368785486e-08	4.00117938826502e-07	1081
ROCK2	-0.162747114	7.42333288694749e-08	3.93637907363931e-07	1081
ITPR1	-0.1628371	7.29998308096315e-08	3.87402948277141e-07	1081
RELN	-0.162845169	7.28902037148413e-08	3.86923097189849e-07	1081
NEFM	-0.162849165	7.28359688317758e-08	3.86806233549044e-07	1081
BBX	-0.162849363	7.28332804082465e-08	3.86806233549044e-07	1081
RFX7	-0.162966873	7.12558123900013e-08	3.78946235637956e-07	1081
GRIA4	-0.162993612	7.09015023539063e-08	3.77161543416806e-07	1081
CHIC1	-0.16300438	7.07593047990019e-08	3.76504541779158e-07	1081
DHX15	-0.163020959	7.05409046148901e-08	3.75441617824825e-07	1081
SLC26A2	-0.163056937	7.00691950303276e-08	3.73128187651586e-07	1081
ADAMTS	-0.16310363	6.94615464032327e-08	3.70283898463383e-07	1081
EGR1	-0.163114074	6.9326331427566e-08	3.69660917577638e-07	1081

EHHADH	-0.163148306	6.88849246451746e-08	3.67404502773906e-07	1081
ZNF277	-0.163179872	6.84803128637493e-08	3.65536805151094e-07	1081
PLEKHA6	-0.16322391	6.79196645138678e-08	3.62640244270307e-07	1081
VWA5A	-0.163247069	6.76266028723627e-08	3.61171239359424e-07	1081
BMPR1A	-0.163254943	6.75272406144525e-08	3.60736213783651e-07	1081
CALCRL	-0.163266052	6.7387304756601e-08	3.60177461266682e-07	1081
NF1	-0.163342963	6.64261259804445e-08	3.55325095028161e-07	1081
UGGT2	-0.163349632	6.63434086460712e-08	3.54976883711847e-07	1081
HMG20A	-0.163372838	6.60563544150551e-08	3.53534872394072e-07	1081
BNC2	-0.16337848	6.59867483465946e-08	3.53256190656962e-07	1081
WASF3	-0.163411412	6.5581869564337e-08	3.51182020832953e-07	1081
NUP54	-0.163416521	6.55192729300712e-08	3.50940109858092e-07	1081
CHPT1	-0.163483702	6.47014972201175e-08	3.46744256850031e-07	1081
PELI2	-0.163496704	6.45443710534629e-08	3.46086333476713e-07	1081
ALMS1	-0.163663478	6.25614282833087e-08	3.3563248276092e-07	1081
RUNDC3B	-0.163671977	6.24619728849078e-08	3.35188184274499e-07	1081
STK38L	-0.163680125	6.23667581655198e-08	3.34766411735784e-07	1081
PHC1	-0.163691397	6.22352794320655e-08	3.34149707931492e-07	1081
ZNF843	-0.163732081	6.17629675458747e-08	3.31790661656439e-07	1081
ZBTB8A	-0.163738331	6.16907082645248e-08	3.31490882365658e-07	1081
PHF8	-0.163770814	6.13165170856107e-08	3.29656054627603e-07	1081
RAB3GAP	-0.163839255	6.05352738338392e-08	3.25716637655633e-07	1081
NEK7	-0.163855473	6.03515579184091e-08	3.24901692748891e-07	1081
ZMYM4	-0.163866004	6.02325603456838e-08	3.24347748774071e-07	1081
TDRD3	-0.163896254	5.9891985542968e-08	3.2260001303826e-07	1081
CAMTA2	-0.163939325	5.9410293592864e-08	3.20091030871421e-07	1081
TBCK	-0.163984051	5.89140537343958e-08	3.17672273147592e-07	1081
SNCA	-0.163990665	5.88410101979954e-08	3.17363360224529e-07	1081
CRY2	-0.164027368	5.84372643513957e-08	3.15270136678861e-07	1081
PDE1A	-0.164037038	5.83313493542973e-08	3.14783025111792e-07	1081
CUL4A	-0.164048506	5.82059671312642e-08	3.14190570165948e-07	1081
RNF219	-0.164052663	5.81605763402889e-08	3.14029699912924e-07	1081
C5orf51	-0.164086477	5.77926898285433e-08	3.12210709733442e-07	1081

CELF1	-0.164123536	5.73920732817388e-08	3.10296112791366e-07	1081
CSGALNA	-0.164131917	5.73018486461284e-08	3.09891474087586e-07	1081
RBM47	-0.164132973	5.72904897850703e-08	3.09891474087586e-07	1081
UBR3	-0.164134823	5.72705956772365e-08	3.09888839623403e-07	1081
ZNF625	-0.164135146	5.7267126541116e-08	3.09888839623403e-07	1081
POLK	-0.164171937	5.68728935290368e-08	3.07902295120249e-07	1081
DSG1	-0.164173626	5.68548601280908e-08	3.07887407871072e-07	1081
RFTN2	-0.164188271	5.66987202259578e-08	3.07124420261339e-07	1081
ITGA6	-0.164193755	5.66403602337191e-08	3.06890816812338e-07	1081
F2R	-0.164202699	5.65452915862755e-08	3.06458138016013e-07	1081
CXADR	-0.16427786	5.57525179977316e-08	3.02324219398197e-07	1081
LCORL	-0.164308506	5.54323695121897e-08	3.00669112499478e-07	1081
RGPD5	-0.164424001	5.42417367622308e-08	2.94369554707742e-07	1081
ANKRD6	-0.164459484	5.38809459131021e-08	2.9256917935834e-07	1081
TENC1	-0.164480482	5.36685353642801e-08	2.91572989458852e-07	1081
NR2F1	-0.164480644	5.36668923945298e-08	2.91572989458852e-07	1081
NMNAT1	-0.164507418	5.33972345661091e-08	2.90333951507225e-07	1081
KCTD3	-0.16451688	5.33022446940941e-08	2.89895712570876e-07	1081
GAS7	-0.164557453	5.28968074231333e-08	2.8784607929201e-07	1081
NLGN4X	-0.164572008	5.27520819136003e-08	2.87136095690213e-07	1081
ARID1A	-0.164592117	5.2552774514748e-08	2.86128552053945e-07	1081
PAX6	-0.164617354	5.23036729831743e-08	2.84883243741892e-07	1081
C1QL3	-0.16469233	5.15703217599645e-08	2.81159440285382e-07	1081
IFT88	-0.164728571	5.12194315516164e-08	2.79473306773378e-07	1081
TBX18	-0.164733177	5.11749956887135e-08	2.79306499092152e-07	1081
MACF1	-0.164781489	5.07112082470783e-08	2.7692526162575e-07	1081
NCOR1	-0.164783826	5.06888777271119e-08	2.76878373593457e-07	1081
TCN1	-0.164833554	5.02159416644737e-08	2.74443880854808e-07	1081
KIAA1644	-0.164872999	4.98438451384209e-08	2.72484195471775e-07	1081
EFCAB6	-0.164933059	4.92824018070541e-08	2.69488052226684e-07	1081
PAFAH1B	-0.164970748	4.89332167290345e-08	2.67869470382174e-07	1081
ZNF532	-0.164996833	4.86929388947731e-08	2.6677162198401e-07	1081
RPS15AP1	-0.165100986	4.77449035615357e-08	2.61791258096662e-07	1081

KIAA1191	-0.165164475	4.71757965126701e-08	2.58811661423676e-07	1081
CACHD1	-0.16523782	4.65265130448687e-08	2.55527973088572e-07	1081
FAM35B2	-0.165291763	4.60545164435167e-08	2.5307371351736e-07	1081
PAPD4	-0.165295639	4.60207826388835e-08	2.52957344136509e-07	1081
NIPAL1	-0.165375009	4.53352038602304e-08	2.49325056446722e-07	1081
STAG3L2	-0.165454751	4.46563758387438e-08	2.45658845240714e-07	1081
EFHC1	-0.165475765	4.44791366938261e-08	2.44750671591676e-07	1081
FAM200A	-0.165494118	4.43248970935136e-08	2.43968593428643e-07	1081
MGC1638	-0.165495245	4.43154369716665e-08	2.43968593428643e-07	1081
CREB3L2	-0.165495269	4.43152371236404e-08	2.43968593428643e-07	1081
ZNF286A	-0.165514947	4.41504732877587e-08	2.43274421329841e-07	1081
WDR48	-0.165520724	4.4102211658434e-08	2.4307498053602e-07	1081
DBX2	-0.165563866	4.37434308221153e-08	2.41295567883766e-07	1081
NAP1L2	-0.165573538	4.36633829140494e-08	2.40919980499459e-07	1081
CDH17	-0.165599184	4.3451816100188e-08	2.39884032704381e-07	1081
ACAD8	-0.165726808	4.2413675846663e-08	2.34281190000281e-07	1081
C4orf12	-0.165727254	4.24100894408678e-08	2.34281190000281e-07	1081
NUMB	-0.165755569	4.21830517123495e-08	2.33135137653026e-07	1081
FILIP1	-0.165888943	4.11293865238165e-08	2.27374174402383e-07	1081
MCM3AP.	-0.1659546	4.06200943957037e-08	2.24620313368502e-07	1081
C5orf35	-0.165956551	4.06050549063289e-08	2.24598800408565e-07	1081
CLDN19	-0.165972906	4.04791916012081e-08	2.23964107334891e-07	1081
FLNB	-0.165978512	4.04361369275926e-08	2.23787356705042e-07	1081
C14orf139	-0.165982095	4.04086421743065e-08	2.23696646496676e-07	1081
PPFIBP2	-0.166056458	3.98420674953871e-08	2.20681454411486e-07	1081
PRTG	-0.166089123	3.95956284433272e-08	2.19437121042868e-07	1081
MAP3K13	-0.166107207	3.94598305578241e-08	2.18744712874894e-07	1081
TRAK2	-0.166108253	3.94519887031313e-08	2.18744712874894e-07	1081
ZNF248	-0.166140026	3.9214537403808e-08	2.17504640969084e-07	1081
PCSK5	-0.166156273	3.90936476765603e-08	2.16893839835943e-07	1081
TBC1D13	-0.166190207	3.8842326160049e-08	2.15558859640272e-07	1081
TRDMT1	-0.166284574	3.81516157206811e-08	2.11842419705932e-07	1081
KCNMB4	-0.166305055	3.80032882086373e-08	2.1107698951282e-07	1081

FBXO38	-0.166317298	3.79148877357844e-08	2.10644074307054e-07	1081
CDC40	-0.166417619	3.7197978393329e-08	2.06832259103951e-07	1081
SPRY4	-0.166424828	3.71469706342407e-08	2.06605666324346e-07	1081
POT1	-0.166456526	3.69234942021145e-08	2.05476185276684e-07	1081
POLH	-0.166526659	3.64336603756525e-08	2.02862379288977e-07	1081
DKK2	-0.166547534	3.62890783544096e-08	2.02169104936278e-07	1081
PDCL	-0.166560464	3.61998036168509e-08	2.01839203947263e-07	1081
GREB1L	-0.166586624	3.60198326698776e-08	2.01058334478992e-07	1081
N4BP2	-0.166624886	3.57581724336249e-08	1.99763833520625e-07	1081
ERG	-0.166643645	3.5630554988323e-08	1.99106111023514e-07	1081
TBRG1	-0.166648197	3.55996529170015e-08	1.98988625974749e-07	1081
RABGAP1	-0.166656214	3.55452981528193e-08	1.98739947623798e-07	1081
RSBN1L	-0.16667053	3.54484254244083e-08	1.98363480604085e-07	1081
B3GAT2	-0.16672945	3.50524316326897e-08	1.96311157976239e-07	1081
ZBTB11	-0.166736458	3.50056216260352e-08	1.96103517145851e-07	1081
IPP	-0.166750918	3.49092145840703e-08	1.95617838051766e-07	1081
MEIS2	-0.166764239	3.48206349992958e-08	1.95175762955151e-07	1081
NHS	-0.166768053	3.47953125107992e-08	1.95088107578639e-07	1081
FBXW8	-0.166811143	3.45104696781314e-08	1.93598833658022e-07	1081
GPR176	-0.166813142	3.44973084786784e-08	1.93598833658022e-07	1081
ZNF121	-0.166878442	3.40701014792122e-08	1.91394923117326e-07	1081
NPY2R	-0.166884468	3.40309369007397e-08	1.91228235387838e-07	1081
ARHGEF9	-0.166912888	3.38468118755721e-08	1.90246658826284e-07	1081
NUCKS1	-0.166913136	3.38452134389784e-08	1.90246658826284e-07	1081
TTC17	-0.166982078	3.34025863029619e-08	1.87854578747395e-07	1081
DOCK5	-0.167009163	3.32302308071319e-08	1.86989664695439e-07	1081
IRS4	-0.167040675	3.30307924366593e-08	1.85919338819922e-07	1081
FAM129A	-0.167106527	3.26177495350414e-08	1.83851305087691e-07	1081
CCDC111	-0.16710759	3.2611122102393e-08	1.83851305087691e-07	1081
CBLB	-0.167114281	3.25694442957516e-08	1.83681818403672e-07	1081
SNX27	-0.167161281	3.22781295154322e-08	1.82140873694224e-07	1081
TMEM220	-0.167192421	3.20865121858711e-08	1.81110335663876e-07	1081
ZNF776	-0.167213944	3.19547133395379e-08	1.80416956341085e-07	1081

PCDHGB6	-0.16723878	3.18032815302611e-08	1.79612309062828e-07	1081
LDB2	-0.1673708	3.10099096771383e-08	1.75420025825421e-07	1081
RNF169	-0.167441511	3.05928949923529e-08	1.7321356650392e-07	1081
TRIM66	-0.167486183	3.03322485473429e-08	1.7193110494829e-07	1081
ALDH1A3	-0.167515181	3.01642116536614e-08	1.71026750285114e-07	1081
C9orf125	-0.167531834	3.00681165132416e-08	1.70529900664204e-07	1081
NR1D2	-0.167596792	2.96961098402068e-08	1.68562449346567e-07	1081
PRPF8	-0.167604295	2.96534312197817e-08	1.68367635829341e-07	1081
ZNF611	-0.16764353	2.94312128193301e-08	1.67200164197802e-07	1081
RBM5	-0.16770689	2.90757529098776e-08	1.6536731856846e-07	1081
SUMF1	-0.167786113	2.86371571042643e-08	1.6296483894503e-07	1081
TGFB2	-0.167851409	2.82804848545337e-08	1.61071633416619e-07	1081
IFT172	-0.167869288	2.81835756309315e-08	1.60565082320451e-07	1081
ZNF304	-0.167900004	2.80178371754357e-08	1.59756391140433e-07	1081
NCOA1	-0.167945846	2.77722382836464e-08	1.58490577967155e-07	1081
PTGIS	-0.167948698	2.77570290211888e-08	1.58490577967155e-07	1081
USP54	-0.168015013	2.74056168112593e-08	1.56709097548345e-07	1081
EHMT1	-0.168021303	2.73725127297757e-08	1.56564244446715e-07	1081
KBTBD3	-0.168042968	2.72587742129622e-08	1.56090962626528e-07	1081
FAM164A	-0.168181399	2.65427910697666e-08	1.52164065481061e-07	1081
SPON1	-0.16818327	2.65332440580992e-08	1.52152633518477e-07	1081
C15orf33	-0.168194376	2.64766151889174e-08	1.51914387063726e-07	1081
PMEPA1	-0.168201713	2.64392695306684e-08	1.51743328973024e-07	1081
CBLL1	-0.168237692	2.62568823014134e-08	1.50739496711876e-07	1081
psiTPTE22	-0.168274021	2.60739497985776e-08	1.49731960858708e-07	1081
SMCR7L	-0.168319018	2.5849087721348e-08	1.48482997475494e-07	1081
TBC1D16	-0.168378908	2.55527174116544e-08	1.46822445025037e-07	1081
ARID4A	-0.16840216	2.54385383364657e-08	1.46208089811156e-07	1081
TSC22D2	-0.168405084	2.54242158360455e-08	1.46167473749183e-07	1081
PHF10	-0.168477874	2.5070170086395e-08	1.44226107882518e-07	1081
ATF6	-0.168501383	2.49568495224684e-08	1.43685548336703e-07	1081
TET3	-0.168538048	2.47811003096707e-08	1.4271448420192e-07	1081
ABCG2	-0.168549104	2.47283436550818e-08	1.42451382022197e-07	1081

ETV1	-0.168587146	2.45476334246628e-08	1.41450822465627e-07	1081
VKORC1L	-0.16864264	2.42863197440549e-08	1.40105358317293e-07	1081
TMTC4	-0.168738629	2.38406744526754e-08	1.37692198064549e-07	1081
DGKB	-0.168761205	2.37370150174809e-08	1.37132826936379e-07	1081
ZNF519	-0.168767958	2.37060960885198e-08	1.36993489874708e-07	1081
AVPR1A	-0.168768555	2.37033655648029e-08	1.36993489874708e-07	1081
ANKRD56	-0.168781211	2.36455235814624e-08	1.36761146295883e-07	1081
SEL1L	-0.168820735	2.34657787157536e-08	1.3576051471248e-07	1081
ETFDH	-0.168859351	2.32914409331867e-08	1.34790599712452e-07	1081
PCGF3	-0.168877427	2.321026363779e-08	1.34359414075655e-07	1081
ABCC9	-0.168898283	2.31169471983237e-08	1.33857689367701e-07	1081
SPIN3	-0.168929313	2.297877782969e-08	1.33095882512681e-07	1081
PELI1	-0.168959599	2.28446929825682e-08	1.32395379785338e-07	1081
IQSEC1	-0.168964616	2.2822552623235e-08	1.32305128804337e-07	1081
ZNF763	-0.168966518	2.28141662765465e-08	1.32294582510371e-07	1081
CWF19L2	-0.168980806	2.27512554456471e-08	1.32043803213069e-07	1081
RNF138P1	-0.169013571	2.26076220800083e-08	1.31247996196474e-07	1081
NRF1	-0.169018496	2.25861075471341e-08	1.31160892631022e-07	1081
JHDM1D	-0.169039507	2.2494550380402e-08	1.30666873533218e-07	1081
ELK3	-0.169060374	2.24039745128668e-08	1.3017827128979e-07	1081
CDC42BP	-0.169070735	2.23591296089776e-08	1.29992688592454e-07	1081
REL	-0.169096237	2.22491310011628e-08	1.29390515016866e-07	1081
DOC2B	-0.169104668	2.22128798096752e-08	1.29216997911033e-07	1081
FXC1	-0.169109761	2.21910094530561e-08	1.29127061072159e-07	1081
FLJ40852	-0.169110035	2.21898343739701e-08	1.29127061072159e-07	1081
SETD5	-0.16918005	2.18912952339133e-08	1.27530405577554e-07	1081
CASP8AP	-0.169191693	2.18420286741964e-08	1.27280204698202e-07	1081
LZTS1	-0.169218715	2.17281021867527e-08	1.26726293732523e-07	1081
KBTBD2	-0.169302421	2.13788354134665e-08	1.24833808522981e-07	1081
NOS1AP	-0.169323282	2.12926402619585e-08	1.24366552066441e-07	1081
ODZ3	-0.169334542	2.12462609072331e-08	1.24131649065026e-07	1081
LOC33875	-0.169368837	2.11055898538834e-08	1.23381342892188e-07	1081
EYA3	-0.169483731	2.06408756320842e-08	1.20839999886177e-07	1081

TCEAL7	-0.169492734	2.06048807458438e-08	1.20699425014546e-07	1081
ZDHHC17	-0.169551798	2.03702553118245e-08	1.19463986392054e-07	1081
EHF	-0.169594312	2.02029786364367e-08	1.18551996688324e-07	1081
C1orf58	-0.169601572	2.01745495039341e-08	1.18419667761291e-07	1081
RBM3	-0.16963436	2.00466267114242e-08	1.1773740381972e-07	1081
ZNF283	-0.169648749	1.99907328009491e-08	1.17443368992453e-07	1081
C10orf12	-0.169668991	1.99123637312055e-08	1.17128713485486e-07	1081
USP49	-0.16971396	1.97393282259682e-08	1.16169845957311e-07	1081
C7orf60	-0.169718808	1.97207603034019e-08	1.16128242125703e-07	1081
ZNF780B	-0.169747257	1.96121437683309e-08	1.15610769426727e-07	1081
LOC92973	-0.169750752	1.95988375145631e-08	1.1557921010857e-07	1081
ZMYM1	-0.169767092	1.95367577679721e-08	1.15246847799648e-07	1081
REV1	-0.169816089	1.93517397745729e-08	1.14222325742388e-07	1081
LOXHD1	-0.169893034	1.90646082061853e-08	1.12560531158735e-07	1081
MOBKL1A	-0.170004731	1.86551369294215e-08	1.1020754646428e-07	1081
MTUS1	-0.170009087	1.8639343047184e-08	1.10146543175571e-07	1081
TFAM	-0.170012162	1.86282014694326e-08	1.10113004284542e-07	1081
ZBTB38	-0.170022554	1.85905927948745e-08	1.09922950353022e-07	1081
MEOX2	-0.170070534	1.84179157135809e-08	1.08933914283643e-07	1081
ZNF317	-0.170086143	1.83620738065916e-08	1.08635529172919e-07	1081
LDOC1L	-0.170127249	1.82158033541986e-08	1.07928634873627e-07	1081
ACVR2A	-0.170133866	1.81923639217558e-08	1.07821468433001e-07	1081
IFT80	-0.170135271	1.81873912657335e-08	1.07821468433001e-07	1081
CDO1	-0.170139087	1.81738921950182e-08	1.07775407203015e-07	1081
ZHX2	-0.170139625	1.81719897740278e-08	1.07775407203015e-07	1081
PAPOLG	-0.170169282	1.80674028378897e-08	1.07207019195667e-07	1081
NBPF15	-0.170180523	1.80279178597145e-08	1.07004244338229e-07	1081
PLXNB1	-0.170201398	1.7954807339954e-08	1.06601707593096e-07	1081
IQUB	-0.170226357	1.78677720275088e-08	1.06116234520685e-07	1081
ZRANB1	-0.170231487	1.78499338527889e-08	1.06041556315079e-07	1081
TRAF6	-0.170277435	1.76909311576239e-08	1.05221083014566e-07	1081
NUP133	-0.170323227	1.75338363264165e-08	1.04348340560018e-07	1081
SNCAIP	-0.170439934	1.71395460127957e-08	1.02122494654767e-07	1081

STOX2	-0.170475866	1.70198943760796e-08	1.01439577575776e-07	1081
ZNF493	-0.170560201	1.67422307131876e-08	9.99029140157478e-08	1081
SUGT1L1	-0.170566722	1.67209470334252e-08	9.98054749595114e-08	1081
B3GALTL	-0.170567653	1.67179088725896e-08	9.98054749595114e-08	1081
ABCB7	-0.170640764	1.64810958994438e-08	9.85197854285741e-08	1081
KIAA0114	-0.170678875	1.63589433011753e-08	9.78186146637509e-08	1081
PKNOX1	-0.170725579	1.62104461141309e-08	9.70170638648746e-08	1081
KCNAB1	-0.170782163	1.60322830324538e-08	9.60078304663442e-08	1081
DOCK4	-0.170833905	1.58710330484505e-08	9.5098739072289e-08	1081
CXXC4	-0.170858056	1.57963062579621e-08	9.46791400079279e-08	1081
SYDE2	-0.170859805	1.57909065324101e-08	9.46749440760124e-08	1081
TM9SF2	-0.170869458	1.57611450518143e-08	9.45246403896396e-08	1081
RSPH3	-0.170888264	1.57033186234783e-08	9.42339450908463e-08	1081
ZNF318	-0.170908908	1.56400767656606e-08	9.38824035888657e-08	1081
CCDC50	-0.17092751	1.5583303296981e-08	9.35694917787429e-08	1081
IL6ST	-0.170947532	1.55224194672654e-08	9.32317054764641e-08	1081
NBPF16	-0.170954033	1.55026993470913e-08	9.31410314187757e-08	1081
TWISTNB	-0.170959398	1.54864464116721e-08	9.30711405021283e-08	1081
C14orf101	-0.170959868	1.54850212538339e-08	9.30711405021283e-08	1081
C17orf57	-0.170977825	1.54307413604699e-08	9.28471579171642e-08	1081
SHANK1	-0.170995445	1.53776557636102e-08	9.25553855267185e-08	1081
CNKSR2	-0.171006586	1.53441841855498e-08	9.23815273215486e-08	1081
PRKCA	-0.171017602	1.53111543851525e-08	9.22102257365913e-08	1081
MAN1A2	-0.171027498	1.52815435600673e-08	9.20594183664942e-08	1081
TNNI3K	-0.171087755	1.51024379251568e-08	9.10348928791995e-08	1081
DUSP4	-0.171173438	1.48512568815159e-08	8.96549505178718e-08	1081
RPRD2	-0.171188423	1.48077442765489e-08	8.94190672814982e-08	1081
FAM8A1	-0.171224859	1.47024634270356e-08	8.88365704072082e-08	1081
ARFGEF2	-0.171295273	1.45010594288992e-08	8.77248775360881e-08	1081
NOV	-0.171331951	1.43972116801614e-08	8.71598777508103e-08	1081
NTN4	-0.171364622	1.43053183424036e-08	8.66969428422744e-08	1081
IRF6	-0.171373768	1.42796947512107e-08	8.65676950837015e-08	1081
RBAK	-0.171459536	1.40415677207504e-08	8.52010185947342e-08	1081

FBXL13	-0.171463867	1.40296446943194e-08	8.51543212916733e-08	1081
C14orf45	-0.17151339	1.38940185935189e-08	8.43819730378169e-08	1081
ALS2	-0.171523443	1.38666431557536e-08	8.42411116925984e-08	1081
KIAA1432	-0.171556238	1.37777014320592e-08	8.37512961221581e-08	1081
REV3L	-0.171556704	1.37764411857187e-08	8.37512961221581e-08	1081
TYW3	-0.171559206	1.3769678994092e-08	8.37512961221581e-08	1081
ZNF708	-0.171559737	1.37682460243649e-08	8.37512961221581e-08	1081
ZNF487	-0.171593224	1.36780575832225e-08	8.32460634483436e-08	1081
LRRN1	-0.171648286	1.35310078155694e-08	8.24008925165195e-08	1081
PPM1L	-0.17165247	1.35198973537316e-08	8.23581288753925e-08	1081
SCN3B	-0.171692418	1.3414258001381e-08	8.18135717341264e-08	1081
GOLGA4	-0.171750796	1.32613174530508e-08	8.09052816752601e-08	1081
GPATCH8	-0.171754861	1.32507329471273e-08	8.08868629118949e-08	1081
C8orf83	-0.171760213	1.32368067293834e-08	8.08291820440827e-08	1081
TMF1	-0.171791761	1.31550092344752e-08	8.0378423120565e-08	1081
C18orf1	-0.17183487	1.30440299422909e-08	7.97729153574533e-08	1081
FAM76B	-0.171858405	1.29838267040113e-08	7.9428845718891e-08	1081
NMBR	-0.171872372	1.29482241843285e-08	7.92351081996653e-08	1081
NHLRC2	-0.171891268	1.29002116111947e-08	7.89652880302389e-08	1081
YTHDF3	-0.17195071	1.27502952455604e-08	7.80950737980586e-08	1081
CYFIP1	-0.172046518	1.25122119643774e-08	7.66834530034628e-08	1081
RBM41	-0.172057269	1.24857664774836e-08	7.65635798012454e-08	1081
ARID4B	-0.172112447	1.23508889159349e-08	7.57639029267686e-08	1081
RERGL	-0.172150579	1.22585069171373e-08	7.52889090993084e-08	1081
GPRASP1	-0.17216835	1.22156830204535e-08	7.50487753726856e-08	1081
GORAB	-0.172179276	1.21894256165685e-08	7.49103047729626e-08	1081
PTPRB	-0.172193415	1.21555280058152e-08	7.47247823244271e-08	1081
SLC30A9	-0.172221193	1.20891992166648e-08	7.43624177770111e-08	1081
PPP1R9A	-0.172227072	1.20752040751597e-08	7.42990183549458e-08	1081
SPTAN1	-0.17223882	1.20472894039316e-08	7.41952445864266e-08	1081
LOC64129	-0.172301191	1.19001314681656e-08	7.33562265686032e-08	1081
ZNF441	-0.172421995	1.16200475958219e-08	7.16735636306896e-08	1081
ARSD	-0.172511004	1.14177853869116e-08	7.04475609860133e-08	1081

ARSH	-0.172528279	1.13789292935667e-08	7.02508521663811e-08	1081
HIPK2	-0.172533652	1.13668696483693e-08	7.01979120375229e-08	1081
HECTD2	-0.172551479	1.13269466527276e-08	6.99728121187358e-08	1081
ZXDA	-0.172580489	1.12622681512214e-08	6.95945987442809e-08	1081
SEC16B	-0.172662584	1.10811760837491e-08	6.85175850850598e-08	1081
PGBD4	-0.172675981	1.10518935366939e-08	6.83575054641383e-08	1081
DKFZP58(	-0.172751899	1.08873662637092e-08	6.74019647764048e-08	1081
MORC3	-0.172859749	1.06577203171398e-08	6.60208412634626e-08	1081
ENTPD7	-0.172948072	1.04731582297456e-08	6.49174684732998e-08	1081
MANEA	-0.17308552	1.01921004897498e-08	6.32142439550523e-08	1081
IPW	-0.173113876	1.01350355366115e-08	6.28796707376159e-08	1081
RBM27	-0.173119442	1.01238708462567e-08	6.28297529876281e-08	1081
SACM1L	-0.17315807	1.00467136887706e-08	6.23701224222754e-08	1081
VCAN	-0.173167755	1.00274586604955e-08	6.22697764228368e-08	1081
ANXA3	-0.173197588	9.96837095558125e-09	6.19219342892952e-08	1081
MUT	-0.173267516	9.831192323861e-09	6.10886395324429e-08	1081
SH3RF2	-0.173434961	9.51011649274772e-09	5.91848306291019e-08	1081
BNIP3L	-0.173467161	9.44955136068923e-09	5.88442696015717e-08	1081
TMEM26	-0.173470582	9.44313762322932e-09	5.88225131168691e-08	1081
CUL3	-0.173483643	9.4186942005509e-09	5.87065577568372e-08	1081
ABHD13	-0.17353128	9.33006089901494e-09	5.81901166596458e-08	1081
UBE2G1	-0.173657343	9.09939695999718e-09	5.68218697331504e-08	1081
RCHY1	-0.173689393	9.04164174861982e-09	5.64962385316211e-08	1081
LATS2	-0.173718183	8.99006472865113e-09	5.62088311479444e-08	1081
LOC6479?	-0.173783032	8.87493348073859e-09	5.55234580650556e-08	1081
ESRRG	-0.173800633	8.84393179019923e-09	5.53638924529408e-08	1081
KIAA146?	-0.173916222	8.64294019030239e-09	5.41730025306912e-08	1081
ARL15	-0.173941739	8.59917273982873e-09	5.39154481306722e-08	1081
PRKD1	-0.173948353	8.58786182847577e-09	5.38612940643351e-08	1081
ATP1A2	-0.173971853	8.54779448634931e-09	5.36434018465753e-08	1081
NBPF7	-0.174058687	8.4013116034054e-09	5.27405491588039e-08	1081
LIFR	-0.174126429	8.28873011323803e-09	5.20824916192078e-08	1081
PLSCR4	-0.174206564	8.15744250262063e-09	5.13215737711719e-08	1081

ARRDC3	-0.174238286	8.10603206121779e-09	5.10459568218919e-08	1081
RASEF	-0.174258927	8.07274820312293e-09	5.08522553320548e-08	1081
SNAP23	-0.174270814	8.05364070571568e-09	5.07477610311674e-08	1081
TRIM4	-0.174276194	8.04500845809454e-09	5.07092288449044e-08	1081
CAMSAP1	-0.174349316	7.92855396172688e-09	5.00064870253563e-08	1081
OTUD4	-0.174357903	7.9149865309169e-09	4.99365498482058e-08	1081
IPO8	-0.1744433	7.78127658823945e-09	4.91391275454808e-08	1081
BTBD8	-0.174469261	7.7410652041243e-09	4.89005200806159e-08	1081
FEM1C	-0.174575986	7.57786690947657e-09	4.7914666946455e-08	1081
CYYR1	-0.174595897	7.54779193653312e-09	4.77544813321167e-08	1081
ARHGAP4	-0.174655519	7.4584261867271e-09	4.72187289539967e-08	1081
GOLGB1	-0.174660454	7.45107553564641e-09	4.71870219005334e-08	1081
ASXL3	-0.174680234	7.42168386651781e-09	4.7030456587292e-08	1081
SLC11A2	-0.174691411	7.40512547494714e-09	4.69402934842071e-08	1081
NUDT11	-0.174693868	7.40148956419509e-09	4.69320136199906e-08	1081
MBNL3	-0.17472166	7.36049166766548e-09	4.67014502819279e-08	1081
PCDH18	-0.174783666	7.26981421200643e-09	4.61697374845112e-08	1081
CHDH	-0.1748009	7.24480394381383e-09	4.60399291634478e-08	1081
LOC14818	-0.174832061	7.19979669178744e-09	4.57683510116939e-08	1081
LMBRD1	-0.174859267	7.16072275712848e-09	4.55343307898842e-08	1081
FAM149B	-0.174862703	7.15580208530754e-09	4.551740859126e-08	1081
TMEM184	-0.174877457	7.13471334995639e-09	4.53975996319872e-08	1081
CHST9	-0.17487802	7.13390878838852e-09	4.53975996319872e-08	1081
ALX4	-0.174882409	7.12764751903112e-09	4.53813082398489e-08	1081
EIF2A	-0.17489508	7.10960095242217e-09	4.52807180482278e-08	1081
FAM131B	-0.174950901	7.03062612108012e-09	4.47918922230104e-08	1081
OTUD7B	-0.174956746	7.02240508217747e-09	4.47536698451329e-08	1081
ARHGEF3	-0.175064496	6.87253337031292e-09	4.38262693083108e-08	1081
RPL10A	-0.17510234	6.82063648856932e-09	4.35228768014662e-08	1081
CNOT7	-0.175152564	6.75234829810782e-09	4.3100778347713e-08	1081
PERP	-0.175153338	6.75130173722891e-09	4.3100778347713e-08	1081
SLC5A3	-0.175202031	6.68574034263665e-09	4.27162192205567e-08	1081
LRRC8A	-0.175229459	6.64908337094979e-09	4.25089763591823e-08	1081

MYLK	-0.175267561	6.59848365388365e-09	4.21988740341226e-08	1081
SH3BP4	-0.175314889	6.53615439325903e-09	4.1826820283419e-08	1081
LPHN3	-0.175346952	6.4942530667687e-09	4.15718868859407e-08	1081
DOPEY1	-0.175349134	6.49141093886764e-09	4.15669018955781e-08	1081
ERMAP	-0.175370792	6.46326722326273e-09	4.13998468084667e-08	1081
MYO5C	-0.175389102	6.43956586485103e-09	4.12742775524734e-08	1081
LARS	-0.17547142	6.33405104662294e-09	4.06367701701335e-08	1081
RCBTB1	-0.175480386	6.32266116823681e-09	4.05766196986718e-08	1081
SRCAP	-0.17550705	6.28890280371687e-09	4.03857019384368e-08	1081
KATNAL1	-0.175553502	6.23051229347875e-09	4.00234917576943e-08	1081
RPL4	-0.175587558	6.18803691191226e-09	3.97633185296563e-08	1081
RALGAPA	-0.175689322	6.06278672569597e-09	3.89833509700432e-08	1081
RICTOR	-0.175727468	6.01647472350087e-09	3.8697919318303e-08	1081
SLC25A24	-0.175793034	5.93767427339441e-09	3.82154786701375e-08	1081
CGGBP1	-0.175807947	5.91989138659246e-09	3.81132029347731e-08	1081
RIOK3	-0.175856364	5.86251276078494e-09	3.77800126570738e-08	1081
ZMYM5	-0.175880956	5.83357615808788e-09	3.76055653454977e-08	1081
ANKRD12	-0.175938997	5.76583076691072e-09	3.71926547548564e-08	1081
CCDC158	-0.175995145	5.70102242334006e-09	3.68099668968544e-08	1081
LRIG1	-0.176007489	5.68687031116885e-09	3.67303630710153e-08	1081
MICAL2	-0.176046056	5.64287118894665e-09	3.64578704622612e-08	1081
ZNF24	-0.17610898	5.57179447530491e-09	3.60333225377263e-08	1081
TAS2R3	-0.176125767	5.55298023472019e-09	3.59347211141786e-08	1081
PRRG3	-0.176176816	5.49614291382732e-09	3.55897778846196e-08	1081
SLC35E1	-0.176242209	5.42416214318691e-09	3.51349666799036e-08	1081
PRO0611	-0.176339226	5.31905464754589e-09	3.44652157847578e-08	1081
RASL11B	-0.176351348	5.30606231653286e-09	3.4392093103782e-08	1081
ZDHHC21	-0.176386119	5.26896353837927e-09	3.41736221766421e-08	1081
FAM126A	-0.176387711	5.26727130279659e-09	3.41736221766421e-08	1081
SNRK	-0.176403436	5.25058168438505e-09	3.40873245349458e-08	1081
CPEB3	-0.176411178	5.24238356760663e-09	3.40450731687413e-08	1081
SCN9A	-0.176411877	5.24164425226504e-09	3.40450731687413e-08	1081
FKTN	-0.176414975	5.23836790243341e-09	3.40409423853294e-08	1081

AGPAT5	-0.176465243	5.18548027496815e-09	3.37256806890433e-08	1081
LRIG3	-0.176537295	5.11057737592165e-09	3.32641619508697e-08	1081
RPL6	-0.176572602	5.07425916618466e-09	3.30385584846231e-08	1081
INPP4B	-0.176594967	5.05138230000831e-09	3.29107685749248e-08	1081
HSPG2	-0.176600699	5.04553597638573e-09	3.28939554188643e-08	1081
PDZD2	-0.176631038	5.01469775826974e-09	3.2714082364101e-08	1081
STMN2	-0.176638992	5.00664363157333e-09	3.26721204917541e-08	1081
SLC35B4	-0.176640395	5.00522416725714e-09	3.26721204917541e-08	1081
MNT	-0.176738183	4.90725076567424e-09	3.2085870390947e-08	1081
ANAPC1	-0.17673898	4.90645956094516e-09	3.2085870390947e-08	1081
ZNF417	-0.176764404	4.88129806612917e-09	3.19369111861553e-08	1081
GALNT1	-0.176780944	4.86499655902038e-09	3.18499031122744e-08	1081
PTPN3	-0.176872983	4.77524433446381e-09	3.12836738594385e-08	1081
XIRP2	-0.176883345	4.76524068742241e-09	3.1228293314289e-08	1081
C4orf34	-0.176923502	4.72666626786566e-09	3.09956679577323e-08	1081
PROL1	-0.176932283	4.71827174158566e-09	3.0960776623532e-08	1081
SNX12	-0.176932998	4.71758882580167e-09	3.0960776623532e-08	1081
PTPLB	-0.177039252	4.61716766647777e-09	3.03368697459865e-08	1081
DDAH1	-0.177099931	4.56075512673303e-09	3.00053599046496e-08	1081
KIAA1409	-0.17718421	4.48351136398186e-09	2.95068070654736e-08	1081
SBNO1	-0.177307291	4.37298412250369e-09	2.88359296719597e-08	1081
OGN	-0.177331995	4.35112106968349e-09	2.87011571541499e-08	1081
INTS6	-0.177349843	4.33539134156129e-09	2.86067666478062e-08	1081
ARFIP1	-0.177388194	4.30178043510891e-09	2.84035945150013e-08	1081
PARK2	-0.177398179	4.29307023290685e-09	2.83553769973471e-08	1081
MIER3	-0.177415548	4.27796135435853e-09	2.82648512573147e-08	1081
WDR20	-0.17743051	4.26498704083432e-09	2.82068824483281e-08	1081
RPL22	-0.177434167	4.26182159316464e-09	2.81952039390154e-08	1081
ABL1	-0.177434901	4.26118646480866e-09	2.81952039390154e-08	1081
HEATR5A	-0.177449089	4.24893008233318e-09	2.81468913214738e-08	1081
SLC38A11	-0.177566688	4.14864736246133e-09	2.75006584787047e-08	1081
MFAP3	-0.177637295	4.0895462294136e-09	2.71535625548903e-08	1081
TOPORS	-0.177650945	4.07821587606507e-09	2.70961935433149e-08	1081

DNAH5	-0.177653362	4.07621190750357e-09	2.70918142120289e-08	1081
EMP1	-0.177662023	4.06904213977465e-09	2.70530870976107e-08	1081
CCDC122	-0.177704456	4.03408974884346e-09	2.68384207366088e-08	1081
WAPAL	-0.177709937	4.02959608406296e-09	2.68173812730255e-08	1081
SF3B1	-0.177786821	3.96707569371583e-09	2.64187569751753e-08	1081
P2RY1	-0.177791791	3.96306742530613e-09	2.64007914294947e-08	1081
SORCS2	-0.177797614	3.95837471869568e-09	2.63869817035488e-08	1081
HNRNPH2	-0.177832936	3.93002968218846e-09	2.62327527991009e-08	1081
BRPF3	-0.178053273	3.75761466722195e-09	2.51235139300319e-08	1081
FAM173B	-0.178070821	3.74420365987945e-09	2.50587982485952e-08	1081
TBC1D8B	-0.1780756	3.74055959099252e-09	2.50427294651194e-08	1081
ZNF264	-0.178189601	3.65464331577033e-09	2.44756614349047e-08	1081
ZNF599	-0.178233119	3.62235532633175e-09	2.42755648865446e-08	1081
PCDHB12	-0.178257943	3.60406136197673e-09	2.41610037061635e-08	1081
PDK4	-0.178294218	3.5774896171024e-09	2.3990854972213e-08	1081
ARIH1	-0.178347861	3.53854493075416e-09	2.37375916183958e-08	1081
SEMA6D	-0.178396205	3.5038007609876e-09	2.35201820493486e-08	1081
TMEM47	-0.178438752	3.47349717853103e-09	2.33400936162467e-08	1081
SOBP	-0.178441789	3.47134402053823e-09	2.33334085064206e-08	1081
MPP7	-0.178442658	3.47072827184003e-09	2.33334085064206e-08	1081
FAM66A	-0.178480326	3.44413308330675e-09	2.31737010565179e-08	1081
NFIC	-0.178521844	3.4150490692236e-09	2.30010576728551e-08	1081
TPT1	-0.178557746	3.39009198079278e-09	2.28558912158871e-08	1081
DAAM1	-0.178600772	3.36041577358727e-09	2.26709898723763e-08	1081
TRIP11	-0.178605263	3.35733288946873e-09	2.26577792490277e-08	1081
C7	-0.178647753	3.32830110276006e-09	2.24693785908517e-08	1081
IPO7	-0.178712228	3.28471226949774e-09	2.2197426591423e-08	1081
ARL6IP5	-0.178735174	3.26933400363754e-09	2.21009172829793e-08	1081
SCAPER	-0.178784516	3.23650159647257e-09	2.1893661739738e-08	1081
SVEP1	-0.178784639	3.23642050318293e-09	2.1893661739738e-08	1081
ZNF709	-0.178793023	3.23087376918723e-09	2.18702795968672e-08	1081
ZFP3	-0.178798301	3.22738664637891e-09	2.18613665068269e-08	1081
CLOCK	-0.178894781	3.16428575765419e-09	2.14628069319676e-08	1081

BRD1	-0.178906353	3.15679844776955e-09	2.14264503808348e-08	1081
ZNF22	-0.178913785	3.1519988459727e-09	2.14010841766498e-08	1081
ARHGAP2	-0.179050606	3.06489658224428e-09	2.08237239963949e-08	1081
ETNK1	-0.179052725	3.06356574282311e-09	2.08217044160498e-08	1081
ITFG2	-0.179167492	2.99234246983425e-09	2.03651145455442e-08	1081
LRCH3	-0.179247411	2.94369897154388e-09	2.00543847757022e-08	1081
ZNF45	-0.17926433	2.93350021414503e-09	1.99916650250175e-08	1081
TTC37	-0.179274136	2.92760463522428e-09	1.99582387061229e-08	1081
KPNA5	-0.179289809	2.9182063695793e-09	1.99076421656536e-08	1081
FKBP14	-0.179307602	2.90757175223534e-09	1.98418133295328e-08	1081
RSC1A1	-0.179318082	2.90132573521962e-09	1.98058986567263e-08	1081
KRIT1	-0.179422848	2.83959874170278e-09	1.93910903903737e-08	1081
KLHDC1	-0.179427789	2.83671971475068e-09	1.93779988652602e-08	1081
CP110	-0.179441623	2.82867280155341e-09	1.93322959235899e-08	1081
FCER1A	-0.179448718	2.82455461444169e-09	1.93145460651486e-08	1081
C18orf16	-0.179482645	2.8049420204481e-09	1.91869463503997e-08	1081
SOCS2	-0.179499156	2.79544527053995e-09	1.91349796041547e-08	1081
TMEM43	-0.179520016	2.78349174633176e-09	1.90725990577732e-08	1081
PARVA	-0.1795255	2.78035755684657e-09	1.90576056422845e-08	1081
ATP1A1	-0.179568162	2.75609194221069e-09	1.89048801341717e-08	1081
MSMP	-0.179616546	2.72882080682936e-09	1.87426168269954e-08	1081
ZNF829	-0.179624467	2.72438113243175e-09	1.87185054272979e-08	1081
PPL	-0.179649985	2.71012668241184e-09	1.86332771389715e-08	1081
ZNF594	-0.179724854	2.66872212212206e-09	1.83799682564611e-08	1081
COL25A1	-0.179757277	2.65098264724736e-09	1.82640374243496e-08	1081
ADAM22	-0.179764238	2.64718936283428e-09	1.82441429060201e-08	1081
ANKRD3(	-0.179816672	2.61878364739017e-09	1.80607314538429e-08	1081
ARHGAP1	-0.179833317	2.60982838452723e-09	1.80051345227059e-08	1081
WNT5A	-0.179845059	2.60352875553829e-09	1.79739845031936e-08	1081
LIMD1	-0.180031849	2.50528555149563e-09	1.73432912147352e-08	1081
XIAP	-0.180045521	2.49823781458822e-09	1.73004471553385e-08	1081
TAS2R5	-0.180056788	2.49244489034908e-09	1.72662662710049e-08	1081
KDM3A	-0.180057975	2.49183492228849e-09	1.72662662710049e-08	1081

TSLP	-0.180134737	2.45272105998549e-09	1.70027755517576e-08	1081
SLC35A3	-0.180141483	2.44931223906162e-09	1.69908385178707e-08	1081
KLF13	-0.180144774	2.44765078559306e-09	1.69851619275825e-08	1081
PEX1	-0.180145356	2.44735742523945e-09	1.69851619275825e-08	1081
KLRA1	-0.18017614	2.43187233999897e-09	1.68873037881004e-08	1081
ITGA10	-0.180228543	2.40573231262861e-09	1.67230771007258e-08	1081
IP6K1	-0.180244484	2.39783439481937e-09	1.66796871145153e-08	1081
PHLDB2	-0.180281745	2.37947279626246e-09	1.6557678577101e-08	1081
CXorf23	-0.180302916	2.3691011731773e-09	1.649690395218e-08	1081
SUCLA2	-0.180308061	2.36658720006358e-09	1.64850965232644e-08	1081
ZNF260	-0.180337499	2.3522531523599e-09	1.63909165528503e-08	1081
MED12	-0.180409097	2.31774215052539e-09	1.61672145506697e-08	1081
SLITRK3	-0.180446493	2.29991310125362e-09	1.60484064512484e-08	1081
PTPN13	-0.180453347	2.29665974709886e-09	1.60312580059967e-08	1081
STON1	-0.180465329	2.29098329520739e-09	1.60027248550461e-08	1081
NARG2	-0.180476104	2.28589027238238e-09	1.5972688011496e-08	1081
PDE10A	-0.180586036	2.23455433089295e-09	1.56302420124439e-08	1081
FAM160A	-0.180602067	2.22716293769248e-09	1.5583951851273e-08	1081
EEA1	-0.180619301	2.21924286786987e-09	1.55393283188177e-08	1081
DNM3	-0.180705967	2.17982828852892e-09	1.52792765735613e-08	1081
ZNF204P	-0.180707262	2.17924465092783e-09	1.52792765735613e-08	1081
PACRG	-0.180774137	2.14930510857564e-09	1.50810698057319e-08	1081
NEK5	-0.180813042	2.13207206351338e-09	1.49757990653686e-08	1081
PNMA2	-0.180831765	2.12382654476708e-09	1.49230853660038e-08	1081
GNE	-0.180885837	2.10018743060709e-09	1.47724426639595e-08	1081
GRB10	-0.180890741	2.09805642609493e-09	1.4762607999889e-08	1081
SPIN4	-0.180935395	2.07874655925591e-09	1.4636962403429e-08	1081
CAPN7	-0.18093849	2.07741476583645e-09	1.46326994607606e-08	1081
AR	-0.180957615	2.06920290470307e-09	1.45799554093191e-08	1081
EGFR	-0.180977685	2.0606190112039e-09	1.45296359750446e-08	1081
ZNF644	-0.181121064	2.00029667534313e-09	1.41339798403323e-08	1081
SVIL	-0.181146123	1.98993161365975e-09	1.40706115679802e-08	1081
ALG13	-0.181154999	1.98627264360831e-09	1.40496707884443e-08	1081

GALNT10	-0.181163897	1.98261142554398e-09	1.4028699391494e-08	1081
ITPR2	-0.181218922	1.96011593476882e-09	1.38841545379458e-08	1081
ZNF383	-0.181252488	1.94651542885569e-09	1.38023770905659e-08	1081
TTLL5	-0.181295756	1.92911912852279e-09	1.36934830317448e-08	1081
SHE	-0.181306093	1.92498519318313e-09	1.36689554870194e-08	1081
SLC44A1	-0.181337321	1.91254987079663e-09	1.35950307505992e-08	1081
MRE11A	-0.181393145	1.89051455892328e-09	1.3447886931324e-08	1081
AHR	-0.181420412	1.87984115391913e-09	1.33814134437106e-08	1081
RPS6KA2	-0.181442479	1.87124644535468e-09	1.33249415488406e-08	1081
HGSNAT	-0.181456663	1.86574229907144e-09	1.32904450547362e-08	1081
ZNF333	-0.181543179	1.83250832887283e-09	1.30629441914873e-08	1081
HERC1	-0.181565967	1.82385116432487e-09	1.30104396973529e-08	1081
GUCY1A2	-0.181642536	1.79505228849621e-09	1.28186204720865e-08	1081
PDE1C	-0.181686027	1.77889205419011e-09	1.27077235573262e-08	1081
URB1	-0.181726811	1.76386657740759e-09	1.26138062484472e-08	1081
FBXL17	-0.181776391	1.74576627530124e-09	1.24976764804348e-08	1081
MYOCD	-0.181785391	1.74250016155577e-09	1.24787293830576e-08	1081
APP	-0.181787557	1.74171522686665e-09	1.24775438283175e-08	1081
ZNF234	-0.181795761	1.7387443623588e-09	1.24606919885159e-08	1081
PPM1F	-0.181873757	1.71074605804898e-09	1.22731407903834e-08	1081
RAG1	-0.181977148	1.67430683426725e-09	1.2024567264283e-08	1081
ARID2	-0.181989908	1.66986244242964e-09	1.19969254289391e-08	1081
FAM160B	-0.182006278	1.66417728902589e-09	1.19603465884505e-08	1081
HIPK1	-0.182039056	1.6528507254908e-09	1.18831826784483e-08	1081
ZNF10	-0.182039516	1.65269227508955e-09	1.18831826784483e-08	1081
LAMC1	-0.182081893	1.63816094028216e-09	1.17901936913126e-08	1081
AOC3	-0.18209894	1.63235076047422e-09	1.17525754359375e-08	1081
FOXO4	-0.182116759	1.6262987164087e-09	1.17131882881849e-08	1081
NBPF1	-0.182130075	1.62179041118134e-09	1.16890761478527e-08	1081
SEPT11	-0.182134297	1.62036347532884e-09	1.16829714425553e-08	1081
DCHS1	-0.182139142	1.61872739113264e-09	1.16753538468912e-08	1081
FYCO1	-0.182208248	1.59556785008177e-09	1.15165583446425e-08	1081
ZBTB1	-0.182243398	1.58391180946891e-09	1.14488350921246e-08	1081

GFM1	-0.182301978	1.56467020639708e-09	1.13138123861698e-08	1081
LRIG2	-0.182316164	1.56004494788722e-09	1.12884717942486e-08	1081
NBLA0030	-0.182353914	1.5478016309458e-09	1.12039036490849e-08	1081
KANK1	-0.182363482	1.54471330702207e-09	1.11855677821565e-08	1081
ZNF793	-0.182384777	1.53786108737883e-09	1.11399538314443e-08	1081
AP1AR	-0.182407133	1.53069953610521e-09	1.10920655233236e-08	1081
DGKH	-0.182435642	1.521614020164e-09	1.10341664637163e-08	1081
KIAA1468	-0.182440942	1.51993091908241e-09	1.10259302718456e-08	1081
KIAA1244	-0.182533347	1.49087279952558e-09	1.08307365836433e-08	1081
RNF144B	-0.182574164	1.47821021003553e-09	1.07503913264317e-08	1081
CITED2	-0.182705984	1.43802680512231e-09	1.04732646381738e-08	1081
PLA2G12A	-0.182717427	1.43458910367054e-09	1.04520063267425e-08	1081
PTPN11	-0.182723335	1.4328172794586e-09	1.04428741297734e-08	1081
RBMS3	-0.182772123	1.41826761391717e-09	1.03443161051272e-08	1081
TGFBRAP	-0.18278373	1.41482731672427e-09	1.03267015563081e-08	1081
LRRC40	-0.182800062	1.41000033338562e-09	1.02989328194537e-08	1081
GLUD1	-0.18297013	1.36067985915888e-09	9.96035456495478e-09	1081
UBE4A	-0.183028486	1.3441463639358e-09	9.84648309144972e-09	1081
GULP1	-0.183072499	1.33180608402452e-09	9.7596338896595e-09	1081
MAP4K5	-0.18308031	1.32962770040697e-09	9.74721616619303e-09	1081
WDR19	-0.183106861	1.32224869756574e-09	9.69665089641858e-09	1081
DLG2	-0.183117054	1.31942626087346e-09	9.67947633841801e-09	1081
NOL8	-0.18311812	1.31913144520967e-09	9.67947633841801e-09	1081
VCL	-0.183136044	1.314184045173e-09	9.6550830014625e-09	1081
ZNF570	-0.183149853	1.31038470331081e-09	9.63068217737917e-09	1081
ZNF189	-0.183149934	1.31036232491822e-09	9.63068217737917e-09	1081
SLC38A2	-0.183250106	1.28312027058837e-09	9.44063471548675e-09	1081
ZSCAN20	-0.183323431	1.26352949678398e-09	9.30329009967589e-09	1081
GK5	-0.183386709	1.24685729046089e-09	9.18389035332164e-09	1081
LRBA	-0.183415993	1.2392143958997e-09	9.13093416437438e-09	1081
SCN3A	-0.183450807	1.23018763625427e-09	9.07770327192027e-09	1081
PKD1	-0.183471541	1.22484199340137e-09	9.04156905718967e-09	1081
ERBB2IP	-0.183560529	1.20215487476772e-09	8.87734968922131e-09	1081

C22orf29	-0.183728868	1.16035115348739e-09	8.58123127276196e-09	1081
PRMT10	-0.183747532	1.15580433436223e-09	8.55074488274958e-09	1081
KCTD1	-0.183748705	1.155519229501e-09	8.55074488274958e-09	1081
GORASP1	-0.18381362	1.13984363160718e-09	8.43886437292417e-09	1081
C2orf67	-0.183872472	1.12581140840221e-09	8.34417616713118e-09	1081
ZBTB16	-0.183902822	1.11864067887247e-09	8.29408041070512e-09	1081
WDR27	-0.18395513	1.10638635248225e-09	8.21732301718113e-09	1081
DDR2	-0.184033432	1.08828648193234e-09	8.09583869221822e-09	1081
ZNF529	-0.184050771	1.0843174118421e-09	8.06929230201663e-09	1081
ARHGAP:	-0.184095435	1.0741588289373e-09	7.99721330771184e-09	1081
CRBN	-0.184142622	1.06352684549584e-09	7.92921846873192e-09	1081
ARHGEF3	-0.18432434	1.02353234763666e-09	7.65085682491301e-09	1081
ZNF567	-0.184339767	1.02020507720169e-09	7.63164919429188e-09	1081
ANGEL2	-0.18440494	1.00626536017347e-09	7.53016927217481e-09	1081
C9orf41	-0.184411972	1.00477237823305e-09	7.52179099201219e-09	1081
CPS1	-0.18442582	1.00183855789926e-09	7.50261626352438e-09	1081
SNX29	-0.184457335	9.95193065484207e-10	7.45839445840006e-09	1081
UHMK1	-0.184503479	9.85540297828265e-10	7.38880137690748e-09	1081
PIK3R4	-0.184524907	9.81088800464209e-10	7.35816600348157e-09	1081
PROS1	-0.184612343	9.63127048937446e-10	7.22883546976336e-09	1081
GATSL1	-0.184619617	9.61647171632329e-10	7.22041829017266e-09	1081
GUCY1A3	-0.184683381	9.48769869351359e-10	7.12904476616304e-09	1081
MBTD1	-0.184700532	9.45334939120047e-10	7.10588520469155e-09	1081
PACSIN2	-0.184722903	9.40872787456682e-10	7.07762595344094e-09	1081
C1orf69	-0.184770197	9.31506652513689e-10	7.01502860369655e-09	1081
MGA	-0.184835328	9.18756803503905e-10	6.92159903013694e-09	1081
CASZ1	-0.184839456	9.17954481371304e-10	6.91814179843805e-09	1081
MYLK4	-0.184988133	8.89507589489572e-10	6.70877214162016e-09	1081
CCDC85A	-0.184989698	8.89212771300539e-10	6.70877214162016e-09	1081
RASSF9	-0.185033694	8.80963352876004e-10	6.65180162806863e-09	1081
ZNF585A	-0.185043071	8.79214788529701e-10	6.6410880820888e-09	1081
ADAMTS:	-0.185057178	8.76590575486291e-10	6.62750988284561e-09	1081
OCLM	-0.185159147	8.57847625436984e-10	6.49674451670227e-09	1081

PVRL3	-0.185302539	8.32149766940667e-10	6.31161786710081e-09	1081
RLIM	-0.18531988	8.29093305423324e-10	6.29080400668657e-09	1081
MAVS	-0.185461093	8.04607334052846e-10	6.11422661052229e-09	1081
SUV420H	-0.185514869	7.95469078868189e-10	6.04934865753102e-09	1081
BIRC6	-0.185534637	7.92135371274086e-10	6.02627154619202e-09	1081
MAMDC2	-0.185571707	7.85920492930176e-10	5.98124984135943e-09	1081
FUBP3	-0.185614331	7.78833125318585e-10	5.93179331173645e-09	1081
RPL3	-0.185644434	7.73865176501598e-10	5.89618531793672e-09	1081
7-Mar	-0.185663293	7.70768689382522e-10	5.87481469830151e-09	1081
SKIV2L2	-0.185690989	7.66243087140979e-10	5.84474327544681e-09	1081
RANBP3L	-0.185723674	7.60935414876204e-10	5.8086562836988e-09	1081
CDK13	-0.185767532	7.53869740730883e-10	5.75690141282169e-09	1081
PPP4R2	-0.185778605	7.52095980538739e-10	5.74553414029309e-09	1081
KIAA0494	-0.185813226	7.4657623407838e-10	5.70553043835697e-09	1081
LRRC16A	-0.185830047	7.43908584146989e-10	5.68730111105924e-09	1081
DDX3X	-0.185914561	7.30645894044632e-10	5.59227261988188e-09	1081
ENAH	-0.185964318	7.22945537314617e-10	5.53543817909653e-09	1081
STK32B	-0.186006327	7.16505912882834e-10	5.48821734411585e-09	1081
ZFR	-0.186075351	7.06046165734945e-10	5.41221461519424e-09	1081
RNF150	-0.186104151	7.01725997367546e-10	5.38114587627302e-09	1081
MPHOSPH	-0.18610501	7.01597481607462e-10	5.38114587627302e-09	1081
WDR78	-0.186105775	7.01483092531841e-10	5.38114587627302e-09	1081
ARHGAP6	-0.186109345	7.00949587438011e-10	5.38114587627302e-09	1081
KRBA2	-0.186112451	7.00485657255226e-10	5.37982598757397e-09	1081
GMCL1L	-0.186130871	6.97740796464094e-10	5.36078884239862e-09	1081
TAB2	-0.186131889	6.97589405449364e-10	5.36078884239862e-09	1081
WWTR1	-0.186175912	6.91073130557783e-10	5.31564269380929e-09	1081
ZBTB4	-0.186255986	6.79372162242182e-10	5.22963401160441e-09	1081
BHLHE41	-0.186266896	6.7779293081851e-10	5.2194719385852e-09	1081
SLC30A4	-0.186291555	6.74236764301718e-10	5.19804807380716e-09	1081
TSPYL1	-0.186316248	6.70693789157267e-10	5.17271301017348e-09	1081
ACADM	-0.186337415	6.67671179268806e-10	5.15334709056326e-09	1081
KDM5B	-0.186341629	6.67071045534877e-10	5.15068846772713e-09	1081

PLD2	-0.186392458	6.59873322940968e-10	5.09706598567707e-09	1081
SENP6	-0.18642584	6.55187315175757e-10	5.06281107181267e-09	1081
PPFIBP1	-0.18643925	6.53314134619875e-10	5.05027369221695e-09	1081
CCNG1	-0.186544062	6.38851448627099e-10	4.94226667918315e-09	1081
NUPL1	-0.186561978	6.36410671360189e-10	4.92527582579755e-09	1081
LUZP2	-0.186579342	6.34053781873527e-10	4.90892138195319e-09	1081
DLG1	-0.18663783	6.26177459354161e-10	4.84980581264497e-09	1081
LOC28636	-0.186664538	6.22612587842776e-10	4.82590634170555e-09	1081
APOOL	-0.186665241	6.22518966456167e-10	4.82590634170555e-09	1081
LEPROT	-0.186685414	6.19839838043951e-10	4.80811456965553e-09	1081
FER	-0.186718543	6.1546444091095e-10	4.77767581012552e-09	1081
ERMP1	-0.186743254	6.12220470141186e-10	4.75633681874053e-09	1081
RSF1	-0.186784529	6.06838984408554e-10	4.71816724851807e-09	1081
NBPF3	-0.186838899	5.99820667580037e-10	4.66720252931628e-09	1081
PJA2	-0.186839761	5.99709986628878e-10	4.66720252931628e-09	1081
JAM3	-0.186869854	5.95860092887734e-10	4.63996968350344e-09	1081
CYTSA	-0.186896593	5.92459565792553e-10	4.61527376368561e-09	1081
LOC10012	-0.186915401	5.90079103834188e-10	4.60028775028627e-09	1081
TAS2R4	-0.186925867	5.8875843690681e-10	4.59176876170642e-09	1081
TSC22D1	-0.186930719	5.88147034328326e-10	4.58877691965303e-09	1081
BICC1	-0.186945494	5.86289438726642e-10	4.57605608025889e-09	1081
LUC7L2	-0.186952009	5.85472149565381e-10	4.57144823759481e-09	1081
CHRM3	-0.186989007	5.80851862517629e-10	4.53889091172135e-09	1081
KCTD10	-0.187024449	5.76459094242534e-10	4.50806228785553e-09	1081
PIK3C2G	-0.187030036	5.75769707758555e-10	4.50441971370722e-09	1081
RAB4A	-0.187061491	5.71902771154758e-10	4.47590572063427e-09	1081
TMEM106	-0.187080117	5.69624879506827e-10	4.45981080360087e-09	1081
XPR1	-0.187123832	5.64313548217628e-10	4.42338382445296e-09	1081
TBCEL	-0.187135582	5.62894156827162e-10	4.41397539481634e-09	1081
MYOM1	-0.187171056	5.58630035079437e-10	4.38565941413689e-09	1081
ZNF431	-0.187226427	5.52037009630942e-10	4.34054689537727e-09	1081
ZNF70	-0.187264416	5.47557623302347e-10	4.30900617154923e-09	1081
CLDN8	-0.187293574	5.44143538560829e-10	4.28696581318259e-09	1081

MYO5B	-0.187333753	5.3947298177766e-10	4.25183224487909e-09	1081
RBM33	-0.187348956	5.37716024966278e-10	4.24130357202258e-09	1081
SPATA13	-0.187360979	5.36330395595142e-10	4.23368958435115e-09	1081
FAM171B	-0.187437104	5.27637970559049e-10	4.16833996741648e-09	1081
CEP192	-0.187456333	5.25464021052547e-10	4.15279431310457e-09	1081
FOXC2	-0.187508342	5.19627913238846e-10	4.10828269709441e-09	1081
NUAK1	-0.187522844	5.18011953230201e-10	4.09711456530129e-09	1081
GLCE	-0.187536777	5.16463904945648e-10	4.08808069356781e-09	1081
ATF2	-0.187545288	5.15520500246475e-10	4.08382244493324e-09	1081
RGP1	-0.187573045	5.12455449888134e-10	4.0611388819813e-09	1081
ZNF69	-0.187603676	5.09093745298152e-10	4.03926486767675e-09	1081
KIAA0232	-0.187655237	5.03483369479382e-10	3.99947652924375e-09	1081
UBE2Q2P	-0.187669664	5.01924429454552e-10	3.98866573229269e-09	1081
DYM	-0.187679772	5.00834881117745e-10	3.98157801109589e-09	1081
CSNK1G3	-0.187732337	4.95206342828944e-10	3.9383860151161e-09	1081
GUSBL1	-0.18777366	4.90824965487733e-10	3.90662541673267e-09	1081
ACADL	-0.187821195	4.85831574801969e-10	3.86993953119243e-09	1081
ZNF774	-0.187844046	4.83448766016938e-10	3.85248235419747e-09	1081
IKZF5	-0.187892289	4.7845567811728e-10	3.81722361808816e-09	1081
NEDD4L	-0.187902223	4.77433719571416e-10	3.81208968718438e-09	1081
HEATR1	-0.187902557	4.77399430848299e-10	3.81208968718438e-09	1081
LUZP1	-0.187958691	4.71665190172345e-10	3.76901834828318e-09	1081
GNPDA2	-0.187988089	4.68688871864831e-10	3.75118685884665e-09	1081
PTPRF	-0.188128635	4.54711123304388e-10	3.64510767169395e-09	1081
SORBS2	-0.1881506	4.52563654788556e-10	3.62933711214788e-09	1081
APAF1	-0.188151792	4.52447407568905e-10	3.62933711214788e-09	1081
PCCA	-0.188161937	4.51459135771054e-10	3.62336425900712e-09	1081
ZNF740	-0.188165632	4.51099696187259e-10	3.62192243112488e-09	1081
CACNA2I	-0.188181868	4.49523732192779e-10	3.61070796851018e-09	1081
MAGEE2	-0.188184571	4.49261858633856e-10	3.61004393385681e-09	1081
AQR	-0.188221223	4.4572579044399e-10	3.58305907761141e-09	1081
PDE5A	-0.188341959	4.34268450672151e-10	3.49514100630862e-09	1081
ZNF223	-0.188363168	4.32285666012202e-10	3.48057343797594e-09	1081

FRY	-0.188372088	4.31454440998125e-10	3.47526977765183e-09	1081
NCRNA0(	-0.188484895	4.2107519062076e-10	3.39302388602208e-09	1081
ZNF214	-0.188654541	4.05923112905421e-10	3.27879755793092e-09	1081
CNTN1	-0.188701162	4.01853072475309e-10	3.252442806354e-09	1081
NRIP2	-0.188725372	3.99755196986105e-10	3.2367638437641e-09	1081
CCDC76	-0.188769552	3.95954447700577e-10	3.20727878927548e-09	1081
NAALAD	-0.188798223	3.93506840591277e-10	3.19001823086973e-09	1081
FHOD3	-0.188800158	3.93342134330219e-10	3.18996670534713e-09	1081
PCDHB4	-0.18881308	3.92244225874869e-10	3.18234391069241e-09	1081
YPEL1	-0.188934008	3.82113619512542e-10	3.10265169894404e-09	1081
MAPKBP	-0.188999769	3.76712143136512e-10	3.06002666269558e-09	1081
CNOT6L	-0.189035362	3.73819635938306e-10	3.03898166504325e-09	1081
C1QTNF7	-0.189068533	3.71143579480122e-10	3.0196637353098e-09	1081
LOC7283	-0.189142184	3.6526823156484e-10	2.97738622150646e-09	1081
RC3H1	-0.189152139	3.64481085478475e-10	2.97266051294084e-09	1081
RMND5A	-0.189318029	3.51605041726545e-10	2.87346189273073e-09	1081
PCDHGA	-0.189336779	3.50177858632862e-10	2.86295980607103e-09	1081
RPL7	-0.189346957	3.49405510252093e-10	2.85896588303347e-09	1081
GRINL1A	-0.189498586	3.38094450371215e-10	2.77204424205459e-09	1081
FAM175A	-0.189515245	3.36873699539563e-10	2.7631598848634e-09	1081
CRYBG3	-0.189521266	3.36433574182183e-10	2.76067387042773e-09	1081
FMO5	-0.189568959	3.32966919057153e-10	2.73668648894588e-09	1081
ZCCHC6	-0.189657785	3.26602954935395e-10	2.68547613354022e-09	1081
SLC35E2	-0.189673493	3.25490011480358e-10	2.67741783637069e-09	1081
GXYLT2	-0.189703023	3.23407625827085e-10	2.66137525420205e-09	1081
SGK196	-0.189713826	3.22649132946208e-10	2.65621854646562e-09	1081
ZNF197	-0.189772882	3.1853305310807e-10	2.6255517000254e-09	1081
ZNF555	-0.189781896	3.17909320182228e-10	2.62148311709823e-09	1081
SULT1C4	-0.18979129	3.17260502308676e-10	2.61720426658816e-09	1081
SNRNP48	-0.189797373	3.16841146274625e-10	2.61481560495794e-09	1081
ZNF721	-0.189804411	3.1635653530508e-10	2.61188623103313e-09	1081
ALPK3	-0.189843499	3.13678392795083e-10	2.59083690974045e-09	1081
FAM47E	-0.189893813	3.10263529790109e-10	2.56368285792525e-09	1081

NTRK3	-0.189930492	3.07797034073434e-10	2.54539049729447e-09	1081
BDP1	-0.189947904	3.06632802935313e-10	2.53680403085498e-09	1081
KIAA0753	-0.189958173	3.05948205944535e-10	2.53218020080224e-09	1081
SON	-0.190012624	3.02343067797059e-10	2.50543031705954e-09	1081
LOC90834	-0.190113085	2.95799998923608e-10	2.45423845894402e-09	1081
HSN2	-0.190145935	2.93690550237349e-10	2.43975098331192e-09	1081
CYBRD1	-0.190185489	2.91170136188439e-10	2.41981121844724e-09	1081
ZNF845	-0.190200774	2.90201815565197e-10	2.41275921360334e-09	1081
BRWD3	-0.190219194	2.89039060941245e-10	2.40507719234258e-09	1081
KDSR	-0.19022559	2.88636361439995e-10	2.40271880215235e-09	1081
CRKL	-0.190283859	2.84992952715595e-10	2.37533431214549e-09	1081
PCDHB11	-0.190381539	2.78985575885266e-10	2.33008475381787e-09	1081
ZNF577	-0.1904409	2.75395417808442e-10	2.30296417258243e-09	1081
ZNF711	-0.190469874	2.7365943587675e-10	2.29034870616416e-09	1081
PDSS2	-0.190480126	2.73047786949764e-10	2.28617941317664e-09	1081
ZNF558	-0.190488532	2.72547218824915e-10	2.28293709905527e-09	1081
MN1	-0.190522359	2.70541970369376e-10	2.26708319180161e-09	1081
VAMP4	-0.190567447	2.67891540570702e-10	2.24580735946599e-09	1081
LPHN2	-0.190587654	2.66711908170352e-10	2.23684903833961e-09	1081
MYST3	-0.190740799	2.57935114439005e-10	2.16504286682239e-09	1081
COG6	-0.190741217	2.57911530172217e-10	2.16504286682239e-09	1081
PIKFYVE	-0.190807356	2.54209071101733e-10	2.13732960657112e-09	1081
PKN2	-0.190879026	2.5025555051719e-10	2.10584714501621e-09	1081
FGF14	-0.190890334	2.49637267062131e-10	2.10152225029947e-09	1081
RUNX1T1	-0.190913303	2.48385962753556e-10	2.09186255002943e-09	1081
KIAA1267	-0.190915189	2.48283492831048e-10	2.09186255002943e-09	1081
SFRS12	-0.190924835	2.4776002894966e-10	2.08921129476387e-09	1081
RNF141	-0.190957355	2.46003255867569e-10	2.07613556323929e-09	1081
ADAM10	-0.190972091	2.45211174631574e-10	2.07031815295602e-09	1081
FAM120B	-0.190995553	2.43955216893534e-10	2.06057771250325e-09	1081
DNMBP	-0.190996834	2.43886807567174e-10	2.06057771250325e-09	1081
NAV3	-0.191014243	2.42959175362766e-10	2.05474919718007e-09	1081
PCDHB15	-0.191051733	2.40973222174641e-10	2.04052356482057e-09	1081

TRIM23	-0.191078753	2.39551707412616e-10	2.02933942213084e-09	1081
HTR1F	-0.191141746	2.36269368593489e-10	2.00237544396964e-09	1081
ZNF608	-0.191146209	2.36038509335202e-10	2.00126084619429e-09	1081
GLI3	-0.191164051	2.3511770513162e-10	1.99429312415852e-09	1081
TSPYL4	-0.19118223	2.34183129275518e-10	1.98828972147085e-09	1081
GLUD2	-0.191195812	2.33487236837498e-10	1.98380446482134e-09	1081
C6orf155	-0.191197507	2.33400526904842e-10	1.98380446482134e-09	1081
HOXA5	-0.191200363	2.33254506754878e-10	1.98350022734361e-09	1081
WEE1	-0.191296515	2.28390128894912e-10	1.9429557206875e-09	1081
PRPF4B	-0.19130149	2.28141142069025e-10	1.94165750189291e-09	1081
IQGAP2	-0.191460439	2.20324308728019e-10	1.87671594051837e-09	1081
ATF1	-0.191535508	2.16724026565703e-10	1.84917641472515e-09	1081
DAG1	-0.191595864	2.1387102628571e-10	1.82560670530747e-09	1081
SHQ1	-0.191598343	2.13754630033912e-10	1.82538661383347e-09	1081
RASA2	-0.191604308	2.13474795242891e-10	1.8237700382392e-09	1081
OR2L13	-0.191612478	2.13092105525369e-10	1.82127300204012e-09	1081
GOLIM4	-0.191616106	2.12922404319011e-10	1.82059500636947e-09	1081
DCUN1D4	-0.191623827	2.12561650811982e-10	1.81905456907705e-09	1081
ZNF862	-0.191671417	2.10351268394264e-10	1.80090365567465e-09	1081
ATP1B1	-0.191693581	2.0932946574415e-10	1.79368017329472e-09	1081
TOB2	-0.191730219	2.0765101981444e-10	1.78005523155825e-09	1081
BMS1P4	-0.191767933	2.05937001334802e-10	1.76686579722726e-09	1081
UBE3A	-0.191813113	2.03901858403768e-10	1.75164304372875e-09	1081
SCN2B	-0.191859172	2.01847295050763e-10	1.7362142437223e-09	1081
ADAMTS	-0.191912763	1.99482150767211e-10	1.7166031299468e-09	1081
NAP1L3	-0.191953969	1.97682019051974e-10	1.70183943324873e-09	1081
THSD7A	-0.191972602	1.96873202871116e-10	1.69560097128629e-09	1081
KLF8	-0.192015348	1.95029922923137e-10	1.68044388250068e-09	1081
C6orf120	-0.192150889	1.89295857461794e-10	1.63453281121639e-09	1081
MLL2	-0.192338674	1.81622558401804e-10	1.56962095195382e-09	1081
ZNF790	-0.192410636	1.78763190164645e-10	1.54623635288397e-09	1081
ZNF718	-0.192499062	1.75309743680555e-10	1.51766858033725e-09	1081
EP400	-0.192585283	1.72005155610812e-10	1.49098272796033e-09	1081

SPRY1	-0.192585863	1.71983139538032e-10	1.49098272796033e-09	1081
C5orf36	-0.192592783	1.71720578406169e-10	1.48979804134034e-09	1081
AFF4	-0.192593597	1.71689743768321e-10	1.48979804134034e-09	1081
OR2C1	-0.192615318	1.70868336128856e-10	1.4836821686706e-09	1081
NBEA	-0.192622881	1.70583241268258e-10	1.48184536237562e-09	1081
ZNF436	-0.192656737	1.69312626455759e-10	1.47334896758154e-09	1081
HIF1AN	-0.192698299	1.67765468822163e-10	1.46051658142717e-09	1081
UCP1	-0.192702633	1.67604943069281e-10	1.45974992569419e-09	1081
ALKBH8	-0.192724819	1.66785466370749e-10	1.45324101212748e-09	1081
ZNF660	-0.192762241	1.65412093432549e-10	1.44189814893929e-09	1081
RCOR1	-0.192763748	1.65357048186444e-10	1.44189814893929e-09	1081
RLF	-0.192776517	1.64891117841112e-10	1.4386018055042e-09	1081
BCL9	-0.192839679	1.62605139257325e-10	1.41927232683657e-09	1081
KANK2	-0.192917748	1.59822351920677e-10	1.39679881971455e-09	1081
TRRAP	-0.192948885	1.58725469244224e-10	1.38781448694657e-09	1081
ZNF284	-0.192958223	1.58397957690207e-10	1.38555226125454e-09	1081
ZEB1	-0.192976519	1.57758171278523e-10	1.3811553065649e-09	1081
KIAA1919	-0.193041743	1.55497769119876e-10	1.36255004737708e-09	1081
PROX1	-0.193061039	1.54835150346918e-10	1.35792132544478e-09	1081
ATG2B	-0.193104655	1.53347480870368e-10	1.34663687974436e-09	1081
CRTAC1	-0.193109946	1.53167969724041e-10	1.34564707810327e-09	1081
PHF16	-0.193217896	1.4954982534247e-10	1.31616021878773e-09	1081
SIAE	-0.193317649	1.46280692169161e-10	1.28963874999902e-09	1081
ST6GAL2	-0.193517738	1.39932174524374e-10	1.23474973972559e-09	1081
CDC73	-0.193532736	1.39467276668259e-10	1.23118680476866e-09	1081
ZNF624	-0.193556746	1.38726217497237e-10	1.22518178495477e-09	1081
LNX2	-0.193558135	1.38683453535173e-10	1.22518178495477e-09	1081
ZNF454	-0.193632003	1.36428185104219e-10	1.20753329917596e-09	1081
ENTPD4	-0.193745396	1.33035652604422e-10	1.17854143435184e-09	1081
LTBP2	-0.193811102	1.31107667317905e-10	1.16248413649613e-09	1081
FOXN3	-0.193812819	1.31057640634153e-10	1.16248413649613e-09	1081
MKL2	-0.193818314	1.30897700035834e-10	1.16164500758673e-09	1081
PDCD6IP	-0.193820723	1.30827646324381e-10	1.16153500890465e-09	1081

OPCML	-0.193846845	1.30070329651393e-10	1.15532045450939e-09	1081
FLJ42709	-0.194009904	1.25438857869998e-10	1.11565818622124e-09	1081
NCOA2	-0.194010286	1.25428205819048e-10	1.11565818622124e-09	1081
WDR11	-0.19403026	1.24872094474349e-10	1.11159891435518e-09	1081
C22orf30	-0.194030557	1.2486386733384e-10	1.11159891435518e-09	1081
RPL5	-0.194059065	1.24074387871462e-10	1.10547480923069e-09	1081
C11orf95	-0.194080773	1.23476479928966e-10	1.10063437529603e-09	1081
FAM59A	-0.194114729	1.22546866299811e-10	1.09331559858711e-09	1081
GABRB2	-0.194244242	1.19063545546253e-10	1.06412383541671e-09	1081
TMEM181	-0.194258745	1.18679535927231e-10	1.0616337705391e-09	1081
TPCN1	-0.194446845	1.138073947411e-10	1.01986208499086e-09	1081
EXTL3	-0.194475259	1.13088583246157e-10	1.01432302292691e-09	1081
ZCCHC24	-0.194486953	1.12794053467028e-10	1.01213194079879e-09	1081
ZSCAN12	-0.194587733	1.10286551979846e-10	9.90513860737404e-10	1081
AFF2	-0.194737304	1.06665106848129e-10	9.6055814816967e-10	1081
EVI5	-0.194757926	1.06174989254012e-10	9.57000070927101e-10	1081
WDFY3	-0.194878091	1.03362540610961e-10	9.32484720379673e-10	1081
PHLPP1	-0.194944581	1.01837730897173e-10	9.19964613867059e-10	1081
MTMR10	-0.195137947	9.75270358062923e-11	8.82210209392797e-10	1081
TNS1	-0.195154958	9.71564440994015e-11	8.79252725239192e-10	1081
RAP1GAP	-0.195159592	9.7055730880014e-11	8.78736044304667e-10	1081
ATRN	-0.195189955	9.63983680041432e-11	8.73176764138249e-10	1081
PCM1	-0.195297329	9.41083360370905e-11	8.54354407150603e-10	1081
ABCA8	-0.195315057	9.37353769531288e-11	8.51352195095031e-10	1081
YLPM1	-0.195353267	9.29364285168572e-11	8.45238985314712e-10	1081
MAPK1	-0.195363672	9.27200379855633e-11	8.43651836142355e-10	1081
DSC1	-0.195423143	9.1492538966573e-11	8.3323562273129e-10	1081
SLC30A5	-0.195432174	9.13075447735611e-11	8.31926951362907e-10	1081
FAM115A	-0.195475237	9.04303737618734e-11	8.24680796483902e-10	1081
ERN1	-0.195491218	9.01069426718834e-11	8.22103423969697e-10	1081
C5	-0.195575203	8.84257185249806e-11	8.07862176728224e-10	1081
CTDSPL	-0.195601704	8.79016057719502e-11	8.0343822517057e-10	1081
DDI2	-0.195682931	8.63139733876394e-11	7.90361360860907e-10	1081

KIAA1737	-0.195712153	8.57496985116588e-11	7.85551467265742e-10	1081
C17orf85	-0.195714043	8.57133225304548e-11	7.85551467265742e-10	1081
SCAMP1	-0.195875594	8.26594225604316e-11	7.57930845461946e-10	1081
HRNR	-0.19588253	8.2530725354786e-11	7.57440301718525e-10	1081
ZNF491	-0.195904735	8.21199882417365e-11	7.54014203796619e-10	1081
C4orf41	-0.19599218	8.05218014893661e-11	7.39949848174353e-10	1081
ATM	-0.19605651	7.93655022288919e-11	7.30387410873014e-10	1081
DYNC1I2	-0.196070821	7.9110490495916e-11	7.28373323144528e-10	1081
ZNF175	-0.19608429	7.88711953026391e-11	7.26502162492759e-10	1081
TBC1D12	-0.196093283	7.87118299060108e-11	7.25365879897799e-10	1081
ZNF496	-0.196110889	7.84007290009669e-11	7.23160570386666e-10	1081
KIAA0947	-0.19612959	7.80716007441776e-11	7.2045460237813e-10	1081
SP4	-0.19617064	7.7353846490304e-11	7.14485666000538e-10	1081
TUBE1	-0.196289814	7.53064594927663e-11	6.96213229225231e-10	1081
EDIL3	-0.196365462	7.403438835495e-11	6.84767104412519e-10	1081
TFCP2	-0.196370334	7.39531871226716e-11	6.84644740159108e-10	1081
ZDHHC3	-0.196406759	7.33488387794466e-11	6.79674497337604e-10	1081
RPL15	-0.19641253	7.32535213153792e-11	6.79103629497614e-10	1081
AMMECR	-0.196434963	7.28841889441659e-11	6.75990785580213e-10	1081
FAT3	-0.196542295	7.11421287567577e-11	6.6044155935709e-10	1081
GCC2	-0.196545387	7.10925520373519e-11	6.60285597414686e-10	1081
FUT10	-0.196577191	7.05845529082327e-11	6.55869842406065e-10	1081
IPMK	-0.196610148	7.00618860050525e-11	6.51313656470596e-10	1081
MAN1A1	-0.196629577	6.97555343512909e-11	6.48765115192408e-10	1081
FLJ42627	-0.196634697	6.96750155417984e-11	6.48315560318489e-10	1081
VPRBP	-0.196662041	6.92465646330638e-11	6.45222962318719e-10	1081
FEM1B	-0.196665771	6.91883170833911e-11	6.4497855050667e-10	1081
PCDH11X	-0.196691451	6.87885891612762e-11	6.41846284693797e-10	1081
C11orf61	-0.196865475	6.61386857882815e-11	6.17693011221572e-10	1081
RHOJ	-0.196920716	6.53185651101357e-11	6.10883237764013e-10	1081
ZNF665	-0.196946064	6.49455624609562e-11	6.07676895390601e-10	1081
DSEL	-0.196972872	6.4553359367286e-11	6.04568770085531e-10	1081
PPP1R3B	-0.197109498	6.25901207114586e-11	5.86454875224341e-10	1081

USP12	-0.197141565	6.21378528614279e-11	5.82759332352637e-10	1081
ZNF678	-0.197152757	6.19807577924359e-11	5.81556760935547e-10	1081
TTC33	-0.197373868	5.89555022026813e-11	5.54203729292121e-10	1081
SLC24A1	-0.197483992	5.75030690187366e-11	5.40802672914309e-10	1081
ZNF782	-0.197557013	5.65593188685332e-11	5.32424055423645e-10	1081
ZNF518A	-0.197596094	5.60604537117719e-11	5.28221627700489e-10	1081
SHANK2	-0.197612397	5.58536250035466e-11	5.26519080812563e-10	1081
NLGN1	-0.197660681	5.52454226440288e-11	5.21762324971383e-10	1081
FAM20B	-0.197682615	5.49712743571954e-11	5.19660404470061e-10	1081
SNORD11	-0.197706297	5.46767715731813e-11	5.17119043822412e-10	1081
PHF3	-0.197754759	5.40789053228165e-11	5.11945276188035e-10	1081
SFRS2IP	-0.197760168	5.40125720004036e-11	5.11557716477729e-10	1081
6-Mar	-0.197799419	5.35336033722506e-11	5.07259849451547e-10	1081
RANBP6	-0.197842303	5.30150375991603e-11	5.0258255644004e-10	1081
PGM5P2	-0.197881246	5.25483934728456e-11	4.9839330814994e-10	1081
PRLR	-0.197930197	5.19675073264959e-11	4.93116078705727e-10	1081
C14orf118	-0.197971239	5.1485306744164e-11	4.88770737210737e-10	1081
N4BP2L2	-0.198075138	5.02840695788748e-11	4.77591976268945e-10	1081
C14orf43	-0.198085439	5.01664769963374e-11	4.76699848627933e-10	1081
OPHN1	-0.198092633	5.00845059670856e-11	4.7614552746906e-10	1081
BAT2L2	-0.198118029	4.97961977705551e-11	4.73851867778853e-10	1081
UBL3	-0.198123739	4.973160749195e-11	4.73460885125393e-10	1081
CASD1	-0.198180223	4.90969771544571e-11	4.6764000225841e-10	1081
NEDD4	-0.198234512	4.84944781553903e-11	4.62119802478873e-10	1081
EPC2	-0.198319501	4.7565738357036e-11	4.53913689816433e-10	1081
TNFRSF1	-0.19835167	4.72187572028413e-11	4.50816049218596e-10	1081
PDE8B	-0.198392749	4.67792693366277e-11	4.46831854332084e-10	1081
C6orf170	-0.198424423	4.64431301958168e-11	4.43831526468088e-10	1081
MLEC	-0.198435582	4.63252622132728e-11	4.4291523838936e-10	1081
SLC2A13	-0.198494057	4.57124172677712e-11	4.37263364605532e-10	1081
KIAA077	-0.198517052	4.54735856578476e-11	4.35599326237442e-10	1081
PARD6G	-0.198576788	4.48588598654292e-11	4.30734857954753e-10	1081
ZC3H14	-0.198758443	4.30389417945853e-11	4.13851781599962e-10	1081

ZNF202	-0.198842619	4.22202783923691e-11	4.06367658009688e-10	1081
BCHE	-0.198920401	4.14773385686214e-11	3.99789945198506e-10	1081
AASS	-0.198967891	4.10300245351737e-11	3.95857205105879e-10	1081
ODZ4	-0.19916589	3.92153307995471e-11	3.78893447940948e-10	1081
CCNT2	-0.199250134	3.84672261553827e-11	3.72022213586262e-10	1081
HOXA7	-0.199269483	3.82973833703311e-11	3.70735602112119e-10	1081
PCDHGA1	-0.19942235	3.69810384734523e-11	3.58682243643571e-10	1081
NDFIP2	-0.199439013	3.68402598815065e-11	3.57488938012017e-10	1081
C5orf33	-0.199503855	3.62973805295652e-11	3.52730695015963e-10	1081
KGFLP2	-0.199663197	3.49963163910081e-11	3.41239493560919e-10	1081
RHOBTB1	-0.199665623	3.49768587201517e-11	3.41214924415233e-10	1081
PARD3B	-0.199670739	3.49358718270506e-11	3.4107011703281e-10	1081
MASP2	-0.199698498	3.47143008294599e-11	3.39146261013322e-10	1081
SMAD1	-0.199701605	3.46895862052504e-11	3.3906924507752e-10	1081
LOC44120	-0.199707909	3.46394905184796e-11	3.38908468428738e-10	1081
C12orf53	-0.199824778	3.37235375733275e-11	3.30428338723095e-10	1081
TNRC6A	-0.199869372	3.33803061086056e-11	3.27224460612098e-10	1081
ZNF471	-0.199968478	3.26296901933736e-11	3.20333873755122e-10	1081
SPTB	-0.199993055	3.24461167247734e-11	3.18686992403978e-10	1081
TNS3	-0.200133935	3.14131481696048e-11	3.08993100525727e-10	1081
TSHZ2	-0.200145327	3.13310351555518e-11	3.08335956623639e-10	1081
CTTNBP2	-0.200152486	3.1279543290493e-11	3.07979667442318e-10	1081
IGSF3	-0.200215499	3.08298612596026e-11	3.04146696902397e-10	1081
SPEN	-0.200220805	3.07922890107544e-11	3.039248712012e-10	1081
ZNF84	-0.200239698	3.06588553876643e-11	3.02756196953185e-10	1081
SH3PXD2	-0.200243841	3.06296694102499e-11	3.02616326762867e-10	1081
SNORD11	-0.200350998	2.98841938943591e-11	2.95686191552978e-10	1081
TRPM7	-0.200371446	2.97439692895704e-11	2.94443371665059e-10	1081
GNAL	-0.200503233	2.88555233892554e-11	2.86210989008641e-10	1081
SAV1	-0.200556976	2.85007104527693e-11	2.82970336161182e-10	1081
UBE4B	-0.200577341	2.83673777015853e-11	2.82063585290442e-10	1081
KCTD16	-0.200578053	2.83627221764471e-11	2.82063585290442e-10	1081
CCNT1	-0.20058085	2.83444643995585e-11	2.82063585290442e-10	1081

PLCXD3	-0.20060569	2.81827847035857e-11	2.80643696418059e-10	1081
LRP2BP	-0.200759228	2.72032435424661e-11	2.7129175305098e-10	1081
KBTBD6	-0.200981993	2.58409693952496e-11	2.58473847302534e-10	1081
MYO6	-0.201113096	2.50706560363367e-11	2.51267843707464e-10	1081
HERC4	-0.201244768	2.43196012467908e-11	2.43983250556076e-10	1081
GPR125	-0.201267073	2.41945745747056e-11	2.42849877831313e-10	1081
BBS9	-0.201272142	2.41662432360297e-11	2.42686425717756e-10	1081
ZNF227	-0.201286345	2.40870508679647e-11	2.42011790391596e-10	1081
ROCK1	-0.201298024	2.40221204606078e-11	2.41479848642188e-10	1081
APBA1	-0.20130083	2.40065446655821e-11	2.4144375551081e-10	1081
PLEKHA7	-0.201325003	2.38727745915343e-11	2.40458522073229e-10	1081
EDEM3	-0.201342831	2.37745895936382e-11	2.39709262944866e-10	1081
NR3C2	-0.201357303	2.36951721141708e-11	2.39028163365033e-10	1081
PRKAA2	-0.201372838	2.36102174092743e-11	2.38290495846609e-10	1081
CLMN	-0.201421331	2.33469235655592e-11	2.35751265778541e-10	1081
USPL1	-0.201437418	2.32602149031811e-11	2.34993495097585e-10	1081
UBE2Q2	-0.201449495	2.31953258201292e-11	2.34455513620924e-10	1081
HMGXB4	-0.201450031	2.31924536230203e-11	2.34455513620924e-10	1081
ZNF470	-0.201640451	2.21925701531681e-11	2.2477090283337e-10	1081
SLMAP	-0.201682958	2.19751918911419e-11	2.22681207568941e-10	1081
KIT	-0.201696595	2.1905894789517e-11	2.22090714914354e-10	1081
MACROD	-0.201724517	2.17646756118312e-11	2.2088130488682e-10	1081
CRIM1	-0.20178391	2.14672388287594e-11	2.1808246404708e-10	1081
CNTN4	-0.201814291	2.13166295893401e-11	2.16824985910941e-10	1081
PHF20	-0.201875855	2.10146033603332e-11	2.14132111630709e-10	1081
ZNF605	-0.201876179	2.10130256658282e-11	2.14132111630709e-10	1081
NPHP3	-0.20198048	2.0510890632744e-11	2.09423158538585e-10	1081
MAP3K9	-0.201993102	2.04509262851483e-11	2.08916790068109e-10	1081
ROR1	-0.202030463	2.02744352444506e-11	2.07258338764836e-10	1081
SENP7	-0.202069214	2.00929490134283e-11	2.05677061928614e-10	1081
AKAP13	-0.202089342	1.99993139421983e-11	2.04822663632732e-10	1081
NOTCH2	-0.20215731	1.96862594895893e-11	2.01719072949021e-10	1081
INO80D	-0.202312964	1.89872717040439e-11	1.95152341060186e-10	1081

ADNP2	-0.202332606	1.89008081941203e-11	1.94362828519935e-10	1081
8-Mar	-0.202377436	1.87049088161618e-11	1.92544909607347e-10	1081
C5orf53	-0.202398256	1.861460865851e-11	1.91713339174685e-10	1081
ALG10B	-0.202424819	1.85000149391317e-11	1.90630588720617e-10	1081
BBS10	-0.202511362	1.81314326566526e-11	1.86928204129102e-10	1081
ZFP28	-0.202514134	1.8119746591188e-11	1.86903376896816e-10	1081
RASA1	-0.202518322	1.81021028915214e-11	1.86817040343084e-10	1081
AGAP1	-0.202549047	1.7973187300162e-11	1.8572616056332e-10	1081
KLHL4	-0.202625965	1.76543753197878e-11	1.82570529166902e-10	1081
CACNA1I	-0.202651394	1.7550197994001e-11	1.81586409136697e-10	1081
ANKS1A	-0.202738067	1.71996107348495e-11	1.78050441034709e-10	1081
PER2	-0.202761697	1.71052249871069e-11	1.7725553362411e-10	1081
PCDH1	-0.202782172	1.70238482542039e-11	1.76503048420451e-10	1081
SH3BGRL	-0.202821292	1.68694229271622e-11	1.74992031342782e-10	1081
ZNF141	-0.202859094	1.67214985485681e-11	1.73636385701497e-10	1081
NPAT	-0.20286183	1.67108422337873e-11	1.73615222691927e-10	1081
LEPR	-0.202881394	1.6634835789905e-11	1.72914740447696e-10	1081
CGNL1	-0.202967291	1.63050931136777e-11	1.69662242135866e-10	1081
SEMA3C	-0.202991588	1.62129871504821e-11	1.68791021264322e-10	1081
AQP4	-0.203129854	1.56984539779723e-11	1.63772840697178e-10	1081
MON2	-0.203132299	1.5689498659475e-11	1.63764223054468e-10	1081
RANBP2	-0.203187934	1.54871122287043e-11	1.61819437680108e-10	1081
GON4L	-0.203192703	1.54698818357109e-11	1.61723284681056e-10	1081
APC	-0.203221644	1.53657283215298e-11	1.60717859313197e-10	1081
SLC38A1	-0.203558083	1.42041230748095e-11	1.4949950853816e-10	1081
ZBTB20	-0.203560344	1.41966134782619e-11	1.49498577375633e-10	1081
SERINC5	-0.203566109	1.41774883017082e-11	1.49375262467527e-10	1081
AKT3	-0.203569784	1.4165308703805e-11	1.49325036022058e-10	1081
PHIP	-0.203583219	1.41208708285359e-11	1.48934525047568e-10	1081
DCAF17	-0.203767186	1.35259199290022e-11	1.43184265354571e-10	1081
TROVE2	-0.203773226	1.35068084649323e-11	1.43057127511073e-10	1081
ZNF391	-0.203778046	1.34915750319197e-11	1.42970951613899e-10	1081
KCTD20	-0.203794404	1.34400029510995e-11	1.42499399710474e-10	1081

C7orf63	-0.203828655	1.33326490054033e-11	1.41435605167904e-10	1081
CTDSPL2	-0.20385163	1.32611035447499e-11	1.40750753903576e-10	1081
SSPN	-0.203905195	1.30957632805362e-11	1.39142484855698e-10	1081
CYTL1	-0.203915838	1.30631517339406e-11	1.38869230438118e-10	1081
PRDM2	-0.203963127	1.29192086651589e-11	1.37411540950173e-10	1081
SGCD	-0.204004486	1.2794590691956e-11	1.36157965921529e-10	1081
C9orf80	-0.204081867	1.25645841441319e-11	1.33922511948961e-10	1081
ZBED4	-0.204103475	1.25010802201535e-11	1.33386790802433e-10	1081
ZNF521	-0.204399863	1.16611010482183e-11	1.24622217833612e-10	1081
CENPC1	-0.204403885	1.16500907774165e-11	1.24570636258522e-10	1081
RBM26	-0.204449353	1.15263288672247e-11	1.23493462526339e-10	1081
GTF3C4	-0.204465523	1.14826257340142e-11	1.23106703252643e-10	1081
NFAT5	-0.204498423	1.13942048021902e-11	1.22413564858292e-10	1081
BAZ2B	-0.204506719	1.13720143917832e-11	1.22246120556282e-10	1081
TIRAP	-0.204512735	1.13559486410419e-11	1.22203838340699e-10	1081
NIPSNAP1	-0.204532716	1.13027515966072e-11	1.21696382102433e-10	1081
SLC1A7	-0.204541933	1.12782920666933e-11	1.21497964536651e-10	1081
DMD	-0.204548144	1.12618400663327e-11	1.21385643732623e-10	1081
TPP2	-0.204598367	1.11296686310257e-11	1.2002525405354e-10	1081
C22orf23	-0.204644989	1.10083313399459e-11	1.18780307896738e-10	1081
RNF2	-0.204718118	1.08206087272719e-11	1.16817343414198e-10	1081
PTPN14	-0.204746739	1.07479948385089e-11	1.16095633255636e-10	1081
SGK223	-0.204762691	1.07077298602865e-11	1.15722756456798e-10	1081
SFRS2B	-0.204791252	1.06360063911042e-11	1.15009312264516e-10	1081
LOC10012	-0.204865017	1.04529345343185e-11	1.13212024835401e-10	1081
EPM2A	-0.204878534	1.04197233871149e-11	1.12913032616154e-10	1081
FCF1	-0.204898201	1.03715858655879e-11	1.1245188227248e-10	1081
GTF2A1	-0.204954595	1.02347565270115e-11	1.11087914998193e-10	1081
ZBED3	-0.204961929	1.02170920168722e-11	1.11044864078093e-10	1081
MPP5	-0.205022821	1.00715795133735e-11	1.09671334755086e-10	1081
ZNF268	-0.205093017	9.90634766127111e-12	1.08033620038763e-10	1081
TRPC1	-0.205110116	9.86650429552976e-12	1.07729392430053e-10	1081
PURB	-0.205188692	9.68540796355725e-12	1.05866816834433e-10	1081

NFRKB	-0.205218887	9.61668480840568e-12	1.05229829150099e-10	1081
MPZL3	-0.205237653	9.57421492441331e-12	1.04822043289297e-10	1081
LOC10019	-0.205304617	9.42415610844501e-12	1.03400642876977e-10	1081
PCDHGA5	-0.205332677	9.36196272355574e-12	1.02777514477401e-10	1081
SEC24B	-0.205445074	9.11684290840399e-12	1.00199663395917e-10	1081
PLDN	-0.205490203	9.02020225826552e-12	9.92419303619655e-11	1081
MEGF8	-0.205525472	8.94537246591775e-12	9.84724198502258e-11	1081
MAGI3	-0.20561421	8.75977705936306e-12	9.64820715477686e-11	1081
SERINC1	-0.20564349	8.69936801669757e-12	9.58691294837924e-11	1081
MYBPC1	-0.205749091	8.4848673468175e-12	9.36076958935589e-11	1081
FLJ13197	-0.205817075	8.34952304356895e-12	9.2165009157642e-11	1081
IQGAP1	-0.2058561	8.2727859062428e-12	9.13680219743757e-11	1081
LOC10027	-0.205871022	8.24362762164914e-12	9.10959289293044e-11	1081
FAM73A	-0.205878654	8.22875398943258e-12	9.09814759149942e-11	1081
PTAR1	-0.205908522	8.17079366472173e-12	9.03902462250517e-11	1081
KLHL8	-0.20592523	8.13854519915312e-12	9.00829632071097e-11	1081
DEPDC6	-0.205961158	8.0696224447355e-12	8.93691831496408e-11	1081
DIS3	-0.205961651	8.06867999580012e-12	8.93691831496408e-11	1081
ZBTB37	-0.205967689	8.05715579750469e-12	8.93293360158129e-11	1081
SLK	-0.205998131	7.99929168527217e-12	8.87855267216572e-11	1081
PKP4	-0.20609838	7.81159676894509e-12	8.69417772985629e-11	1081
NIPAL3	-0.206110563	7.78908418265084e-12	8.67391381202328e-11	1081
C5orf41	-0.20613115	7.75118219376136e-12	8.63648038126784e-11	1081
BACH1	-0.206150239	7.71620108096389e-12	8.60226180276799e-11	1081
TLK1	-0.206245508	7.54391425761468e-12	8.42417698002482e-11	1081
PABPC4L	-0.206352592	7.35475078893136e-12	8.22205630649402e-11	1081
ADCYAP1	-0.206479922	7.1358576404766e-12	7.99954658694497e-11	1081
MYH10	-0.206484396	7.12828356458627e-12	7.99550514524445e-11	1081
PURG	-0.206538523	7.03727150996327e-12	7.91103987545815e-11	1081
RNMT	-0.206541656	7.03203963962505e-12	7.90957222446939e-11	1081
ZNF527	-0.206555846	7.00838461022048e-12	7.88736916049674e-11	1081
ZNF667	-0.2066505	6.85258283585419e-12	7.72065331254378e-11	1081
TFAP2B	-0.20669518	6.78022161694582e-12	7.6434003622481e-11	1081

ZNF551	-0.206765557	6.66775670922695e-12	7.52082636659446e-11	1081
IPO5	-0.206824123	6.57555898386924e-12	7.42514774271558e-11	1081
DPY19L4	-0.206825645	6.57318010904622e-12	7.42514774271558e-11	1081
CALCOC(	-0.206900061	6.45787096700213e-12	7.30043830697295e-11	1081
EIF4EBP2	-0.206945282	6.38876823524858e-12	7.23043461230801e-11	1081
MBNL2	-0.206954018	6.37550334275885e-12	7.21947806857094e-11	1081
BAI3	-0.206987138	6.32545453805364e-12	7.16683248982512e-11	1081
RGPD3	-0.207068302	6.20442370476906e-12	7.04158397366607e-11	1081
ZMAT1	-0.207123871	6.12287126554257e-12	6.96079241785299e-11	1081
YPEL2	-0.207141928	6.09659751986857e-12	6.93483664809442e-11	1081
SHROOM	-0.20719651	6.01784673725987e-12	6.86075396276741e-11	1081
PCDHGA(	-0.207210803	5.99739022701086e-12	6.84130385748209e-11	1081
SWAP70	-0.207225521	5.97639595452128e-12	6.82508483581809e-11	1081
NBAS	-0.20734312	5.81121398711175e-12	6.64397876108775e-11	1081
KLHL28	-0.207356437	5.79279377287353e-12	6.63044491787144e-11	1081
TNPO1	-0.207378797	5.76199164997968e-12	6.60269179686238e-11	1081
ZNF236	-0.207560654	5.51735909521954e-12	6.33678443404775e-11	1081
CD302	-0.207743147	5.28209879454483e-12	6.07697773935498e-11	1081
FBXO3	-0.207773933	5.24339169217903e-12	6.03934394733828e-11	1081
INADL	-0.207781822	5.2335171290513e-12	6.03141891102623e-11	1081
ITGA2	-0.207836158	5.16600035971604e-12	5.95701644227131e-11	1081
ZNF844	-0.207859051	5.13781062504403e-12	5.93470155054541e-11	1081
SC5DL	-0.207900541	5.08710285697116e-12	5.87950011782468e-11	1081
SLC25A4(	-0.207928348	5.05339335904258e-12	5.84389260722806e-11	1081
ZNF627	-0.207930726	5.05052126418309e-12	5.84389260722806e-11	1081
ERCC6	-0.207930984	5.05020947599551e-12	5.84389260722806e-11	1081
ZNF641	-0.208012471	4.95273837524257e-12	5.74397896196095e-11	1081
XYLT1	-0.208093186	4.85800722385353e-12	5.64386133359454e-11	1081
C12orf26	-0.208103452	4.84608667550613e-12	5.63326117011373e-11	1081
EXOC6B	-0.208122276	4.82430225679767e-12	5.61117603713563e-11	1081
ZNF160	-0.208282603	4.64260932672585e-12	5.41548146420917e-11	1081
TOR1AIP2	-0.208326718	4.59380173382127e-12	5.361653298252e-11	1081
ACER2	-0.208334269	4.58549792666417e-12	5.35506410044347e-11	1081

KDM6B	-0.208349647	4.56863222643931e-12	5.33846265670649e-11	1081
COG3	-0.208353012	4.56495044620222e-12	5.33725634003156e-11	1081
TAF1L	-0.20837979	4.53574945754944e-12	5.30927790949061e-11	1081
NCRNA00	-0.208455016	4.45469317968306e-12	5.22046504390432e-11	1081
ZMAT3	-0.208487031	4.42062878406203e-12	5.18356035244061e-11	1081
ZNF785	-0.208566515	4.33715505661547e-12	5.09754892739316e-11	1081
ZNF813	-0.208596283	4.30629195635558e-12	5.06422950734285e-11	1081
KIAA1217	-0.208649276	4.25187952368171e-12	5.00608492136576e-11	1081
CEP350	-0.208694892	4.20558252269366e-12	4.95447134033121e-11	1081
ZNF619	-0.208715316	4.18501287470439e-12	4.93312371918782e-11	1081
MECP2	-0.208804557	4.09628815234338e-12	4.83419594780066e-11	1081
JRK	-0.208961505	3.94469421031316e-12	4.6607545376398e-11	1081
ZFHX4	-0.20906711	3.8457974840405e-12	4.54924194456816e-11	1081
USP46	-0.209157789	3.76281832757749e-12	4.45370007103693e-11	1081
LANCL1	-0.209220992	3.70602225450151e-12	4.3890545747756e-11	1081
PGM5	-0.209250913	3.67942764841049e-12	4.36012176336643e-11	1081
ZFP14	-0.209275989	3.65728359649821e-12	4.33643190414693e-11	1081
FARP1	-0.209295949	3.63974980954751e-12	4.31818374047907e-11	1081
ZNF720	-0.209333113	3.60732414641568e-12	4.2822360005624e-11	1081
SMCR8	-0.20934236	3.5993003745322e-12	4.2752303092542e-11	1081
PIK3C3	-0.209348653	3.59384967897214e-12	4.27127444146866e-11	1081
C8orf79	-0.20941148	3.53987223614366e-12	4.20960603288749e-11	1081
TRAK1	-0.209446591	3.51005375347835e-12	4.18155132252048e-11	1081
MXI1	-0.209549987	3.42366372139159e-12	4.08588303717023e-11	1081
DSG2	-0.209739706	3.27053104611994e-12	3.91240189572958e-11	1081
C9orf102	-0.209744067	3.26709104149343e-12	3.91060897390881e-11	1081
CAMSAP1	-0.209794492	3.22757243179085e-12	3.86560324842013e-11	1081
MYH11	-0.209819114	3.20844682505031e-12	3.84498282514208e-11	1081
MTX3	-0.209864571	3.17342716898256e-12	3.80754558184358e-11	1081
INSR	-0.209870758	3.16868954964545e-12	3.80412699508984e-11	1081
ZNF426	-0.209872818	3.16711382611032e-12	3.80412699508984e-11	1081
POLI	-0.209885701	3.15727666977875e-12	3.79494859860937e-11	1081
STEAP2	-0.210218069	2.91357592317179e-12	3.51250670091536e-11	1081

WDR31	-0.210221459	2.9111876838172e-12	3.51172909523937e-11	1081
PHF2	-0.210307932	2.85091324441427e-12	3.44520979656422e-11	1081
PLEKHA5	-0.21038588	2.79762948876541e-12	3.38487964271347e-11	1081
STXBP5	-0.210416817	2.77675365139022e-12	3.36366219526494e-11	1081
TAF1	-0.210451605	2.75346020120645e-12	3.33946151434702e-11	1081
MTMR6	-0.210477988	2.73592307082632e-12	3.32419603509025e-11	1081
ZNF510	-0.210488246	2.72913401219565e-12	3.31995197316917e-11	1081
PRICKLE2	-0.210600982	2.65559859211551e-12	3.23636017169794e-11	1081
RWDD4A	-0.210622034	2.64208362545098e-12	3.22183865827542e-11	1081
ENTPD5	-0.210674172	2.60890130772375e-12	3.18523132388454e-11	1081
ZNF81	-0.210683896	2.60275756145423e-12	3.17965743332295e-11	1081
EP300	-0.210803946	2.52807052508366e-12	3.09404500168958e-11	1081
PPP1R1B	-0.210887174	2.47752732728556e-12	3.03402966615001e-11	1081
CHSY1	-0.210944382	2.44336140207462e-12	2.99583173735808e-11	1081
GATAD2B	-0.2109591	2.43464643220957e-12	2.98696421296357e-11	1081
PEX26	-0.211085914	2.36080568773552e-12	2.89813714682706e-11	1081
AFF1	-0.211163191	2.31688938665668e-12	2.85117511876597e-11	1081
TEDDM1	-0.21139343	2.19073521187687e-12	2.7025328134268e-11	1081
EFNB2	-0.211484244	2.14284838174698e-12	2.64832396627564e-11	1081
WDR82	-0.211492251	2.1386752993066e-12	2.64478906719039e-11	1081
NIPBL	-0.211607894	2.07929133650197e-12	2.57926871760051e-11	1081
GIGYF2	-0.211641818	2.06217899022736e-12	2.56119579273305e-11	1081
LAMA2	-0.211869454	1.9508710608254e-12	2.42744271280591e-11	1081
C9orf5	-0.211899663	1.9365488753797e-12	2.41126505165308e-11	1081
C1orf26	-0.211945342	1.9150884701389e-12	2.38734265042996e-11	1081
LMTK2	-0.211952889	1.9115650494883e-12	2.38442587751962e-11	1081
MEX3C	-0.211974901	1.90132572791098e-12	2.37312309719745e-11	1081
DCAF16	-0.212107653	1.840700411057e-12	2.3017324506979e-11	1081
IREB2	-0.212114482	1.83763338131019e-12	2.29932450102446e-11	1081
ZXDC	-0.212116074	1.8369194678353e-12	2.29932450102446e-11	1081
SMYD4	-0.212125971	1.83248529324482e-12	2.29573484032443e-11	1081
SPTBN1	-0.212128701	1.83126395458498e-12	2.29563238115211e-11	1081
IMPG1	-0.212197917	1.8005645148637e-12	2.26278054597188e-11	1081

LONRF1	-0.212312282	1.75094154199437e-12	2.20316785530772e-11	1081
FAM108B	-0.212383243	1.7208279493009e-12	2.16934161693783e-11	1081
FZD1	-0.212513395	1.66690837921723e-12	2.10532095920571e-11	1081
PACRGL	-0.212552321	1.6511061037889e-12	2.08667079428026e-11	1081
C14orf135	-0.212587966	1.6367646094175e-12	2.07244645233913e-11	1081
QSER1	-0.212658004	1.6089398769344e-12	2.03977934681206e-11	1081
STARD13	-0.212703303	1.59119094496652e-12	2.02109341654164e-11	1081
ZNF292	-0.212773619	1.56401879296215e-12	1.9878333491623e-11	1081
IRGQ	-0.21277456	1.56365849298293e-12	1.9878333491623e-11	1081
KIAA1632	-0.2127906	1.55752528575596e-12	1.98208129384421e-11	1081
PDPR	-0.212791685	1.55711147018102e-12	1.98208129384421e-11	1081
ZC3H11A	-0.212876922	1.52492252511753e-12	1.94427621952485e-11	1081
GPR81	-0.212958686	1.4946584795872e-12	1.90931484282081e-11	1081
ASPH	-0.213064715	1.45628773256983e-12	1.86266135699169e-11	1081
GTF2IRD2	-0.213066644	1.45559840211838e-12	1.86266135699169e-11	1081
SAP30L	-0.213076959	1.45191946077171e-12	1.85943531705316e-11	1081
PUM2	-0.213146139	1.42747784432698e-12	1.83046092768727e-11	1081
METT10D	-0.213151378	1.42564357870118e-12	1.82927324158824e-11	1081
ZNF221	-0.213194013	1.41080049152302e-12	1.81138151062659e-11	1081
AEBP2	-0.213276628	1.38246856975011e-12	1.77840544940076e-11	1081
PLXNA4	-0.213292785	1.37699300448036e-12	1.77249355113462e-11	1081
STAG1	-0.213533662	1.2978398408733e-12	1.67488684140888e-11	1081
EYS	-0.213547762	1.29334744225021e-12	1.67015924513656e-11	1081
OGT	-0.213604918	1.27529178321032e-12	1.6497330133436e-11	1081
CTNND1	-0.213613581	1.27257658242688e-12	1.64756139158031e-11	1081
TSTD2	-0.213644903	1.26280736571642e-12	1.63807175675192e-11	1081
CYB561D	-0.21373478	1.23518133400675e-12	1.60326855499781e-11	1081
HECTD1	-0.213813208	1.21155893628873e-12	1.57362055264581e-11	1081
ZNF585B	-0.213890141	1.18881684659481e-12	1.5460758795773e-11	1081
LRCH1	-0.213931095	1.1768817334318e-12	1.53253280672162e-11	1081
ZC3HAV1	-0.21393636	1.17535596808399e-12	1.53153596229314e-11	1081
ZNF451	-0.213953492	1.17040435569152e-12	1.52607092203273e-11	1081
ZNF180	-0.214315445	1.07044322266839e-12	1.40117470569556e-11	1081

SHISA6	-0.21437628	1.05448470567257e-12	1.3811829906225e-11	1081
AKAP12	-0.214483511	1.0269206315689e-12	1.34770789074629e-11	1081
RNF111	-0.214484729	1.0266118095953e-12	1.34770789074629e-11	1081
LPP	-0.214507528	1.02084445449667e-12	1.34148150918692e-11	1081
RUNDC2/	-0.214569411	1.00535052322355e-12	1.32198343931712e-11	1081
VPS24	-0.214585834	1.00127722603143e-12	1.31748724483365e-11	1081
MAP3K7	-0.214621228	9.92554025648101e-13	1.30771751776854e-11	1081
ZYG11B	-0.214693806	9.74898840459145e-13	1.28698146402683e-11	1081
LOC28373	-0.21482996	9.42604413452481e-13	1.24761931070961e-11	1081
ZNF697	-0.214900243	9.26346636892114e-13	1.22690683761944e-11	1081
ZFYVE16	-0.214902228	9.25891470506615e-13	1.22690683761944e-11	1081
CHM	-0.21492	9.21826201216093e-13	1.22333259706839e-11	1081
TPR	-0.214928741	9.19833231390346e-13	1.22149244867228e-11	1081
DCBLD2	-0.214929265	9.19713925566008e-13	1.22149244867228e-11	1081
HERC2	-0.214931794	9.19138042810433e-13	1.22149244867228e-11	1081
ITGA9	-0.21503927	8.9499263858656e-13	1.19243562859301e-11	1081
FAM13B	-0.2150989	8.81865471177248e-13	1.17728163796326e-11	1081
SORBS1	-0.215108402	8.79791229031029e-13	1.17529139978979e-11	1081
STRN	-0.215120174	8.77228133008231e-13	1.17264503911419e-11	1081
INTU	-0.215140034	8.72920331739813e-13	1.16766136008622e-11	1081
SESN1	-0.215213511	8.57162597401287e-13	1.14734488535873e-11	1081
PREX2	-0.215269334	8.45377400913998e-13	1.13322936402524e-11	1081
RTTN	-0.215290193	8.41014804985869e-13	1.12873039616525e-11	1081
ZNF514	-0.215303215	8.38302373027369e-13	1.12659114774092e-11	1081
HCG11	-0.215362252	8.26112428890439e-13	1.11243548663088e-11	1081
CUL5	-0.215399059	8.18600489755699e-13	1.10379564030312e-11	1081
C2orf86	-0.215478093	8.02696264897569e-13	1.08380135766498e-11	1081
LZTFL1	-0.215491355	8.00057407804487e-13	1.08096287593705e-11	1081
REST	-0.215507026	7.96950062577567e-13	1.07748718192115e-11	1081
TNKS	-0.215657801	7.67651982814119e-13	1.04137031608016e-11	1081
C1orf107	-0.215669108	7.65497922419855e-13	1.04055031357274e-11	1081
CDC14C	-0.215714503	7.56909196021966e-13	1.02957027372468e-11	1081
MAPK8	-0.215742156	7.51723762366781e-13	1.02459236758314e-11	1081

DSTYK	-0.215745785	7.51045770593548e-13	1.02436134384611e-11	1081
FAM168A	-0.215762137	7.47998489884317e-13	1.02089631292138e-11	1081
PAR-SN	-0.215776692	7.45296276357325e-13	1.01789786354022e-11	1081
EIF2C4	-0.215818893	7.37515520474401e-13	1.00795455630643e-11	1081
TGFBR1	-0.215830489	7.35391610264556e-13	1.00573414723554e-11	1081
SMC5	-0.215937929	7.15994950629036e-13	9.81205325198771e-12	1081
TJP1	-0.216036103	6.98710237334366e-13	9.58170029346549e-12	1081
DICER1	-0.216115153	6.85090452879878e-13	9.40773495110098e-12	1081
SLC10A6	-0.216124147	6.83557323504392e-13	9.39308477625919e-12	1081
THSD7B	-0.216213744	6.68467974080894e-13	9.21086685216115e-12	1081
RGS7BP	-0.216217662	6.67815738215423e-13	9.20817799202581e-12	1081
C5orf24	-0.216310292	6.52573574176643e-13	9.02891116194263e-12	1081
HLF	-0.216325712	6.50069464346448e-13	9.0004462950235e-12	1081
GPR107	-0.216360724	6.44418954692145e-13	8.92834927254007e-12	1081
UBN2	-0.216454809	6.29471911831836e-13	8.72726198475728e-12	1081
KIAA1549	-0.21664899	5.99689785078455e-13	8.34305988978279e-12	1081
PRSS35	-0.216694109	5.92969978558884e-13	8.260982170172e-12	1081
UTP14C	-0.216741456	5.85997895191364e-13	8.16950006825608e-12	1081
SMARCC	-0.216776134	5.80942318131633e-13	8.1064145298542e-12	1081
NSUN7	-0.216784836	5.7968041631581e-13	8.09823993528572e-12	1081
FGD4	-0.216971655	5.53228378169876e-13	7.75019866358286e-12	1081
TCF12	-0.216982215	5.51769033945366e-13	7.73513374309631e-12	1081
JMJD1C	-0.216991277	5.50519709076312e-13	7.7229941081771e-12	1081
PSD3	-0.217055652	5.41724287226974e-13	7.6102062525714e-12	1081
SMARCA	-0.217067364	5.40139000987293e-13	7.59323110599373e-12	1081
ZFP2	-0.217151422	5.2889383600444e-13	7.44624750736992e-12	1081
RAPGEF5	-0.217171206	5.26280719288327e-13	7.4191218264964e-12	1081
C16orf52	-0.217246152	5.16495859312379e-13	7.30674795354485e-12	1081
KDM3B	-0.217247422	5.163316309383e-13	7.30674795354485e-12	1081
RAB27B	-0.217273782	5.12933744117685e-13	7.27167507054944e-12	1081
ANKRD17	-0.21731864	5.07201881869156e-13	7.19548021848883e-12	1081
SPRY2	-0.217321995	5.06775646479138e-13	7.19449992834548e-12	1081
ATE1	-0.217348911	5.03369397061273e-13	7.15622900762127e-12	1081

ATXN1L	-0.217365251	5.01312377014238e-13	7.137058540602e-12	1081
ZNF727	-0.21736781	5.00990976477327e-13	7.137058540602e-12	1081
SP1	-0.217402016	4.96714394229192e-13	7.08662285534495e-12	1081
CA5B	-0.217450491	4.90715097322343e-13	7.00599265454188e-12	1081
EMX2OS	-0.217482142	4.86836213280912e-13	6.95554291953473e-12	1081
NOTCH3	-0.21752873	4.81181572942983e-13	6.8796329218853e-12	1081
DHX33	-0.21758897	4.73965278474495e-13	6.79091787686252e-12	1081
DPP8	-0.217708019	4.60014875806434e-13	6.60513162731333e-12	1081
KIAA1958	-0.217732887	4.5715192604037e-13	6.56870581318349e-12	1081
KIAA1147	-0.217774215	4.52432868569053e-13	6.51018581237398e-12	1081
ZNF423	-0.217826119	4.46573628580691e-13	6.44428778492694e-12	1081
LOC28402	-0.217863147	4.42439275952353e-13	6.38920373767753e-12	1081
TNKS2	-0.217946749	4.33242228987858e-13	6.27438152621164e-12	1081
EIF3A	-0.218056505	4.21452097982474e-13	6.11242081631168e-12	1081
CBX6	-0.218198814	4.06632525414551e-13	5.9017379138877e-12	1081
HMCN1	-0.21825871	4.00549096262756e-13	5.82183372598356e-12	1081
DUSP6	-0.218274003	3.9901017430456e-13	5.81204624827575e-12	1081
ZNF347	-0.218293523	3.97054348439288e-13	5.78774229327746e-12	1081
PDZD8	-0.218549851	3.72227229615563e-13	5.44950402660285e-12	1081
SEPT2	-0.218672518	3.60891076200445e-13	5.29123051678164e-12	1081
DTWD2	-0.218732088	3.55508803740707e-13	5.2199160724173e-12	1081
ZNF573	-0.218745535	3.54304751988228e-13	5.20603153085547e-12	1081
ZBTB34	-0.21894912	3.36557203100988e-13	4.98159063664174e-12	1081
RNF38	-0.219070442	3.26398711341933e-13	4.83833851360062e-12	1081
TTC28	-0.219214115	3.14757152140475e-13	4.66920679666412e-12	1081
C10orf118	-0.219255042	3.11516089836423e-13	4.62453325700423e-12	1081
ZNF345	-0.219374613	3.0223348368081e-13	4.49335315774902e-12	1081
ZADH2	-0.219392099	3.00898973941371e-13	4.48012552110045e-12	1081
C15orf28	-0.219488312	2.93658666854254e-13	4.37555757675958e-12	1081
ARL10	-0.219506166	2.92334073411186e-13	4.36227400656915e-12	1081
FRMD6	-0.219606223	2.8501832256319e-13	4.25625953153111e-12	1081
LCOR	-0.219638531	2.82694672500579e-13	4.22469152635323e-12	1081
ARHGEF3	-0.219687714	2.79192940449988e-13	4.18166675491822e-12	1081

BRD3	-0.219843609	2.68372493555512e-13	4.02259217460997e-12	1081
ITGA8	-0.219859378	2.67301153251414e-13	4.00951729877121e-12	1081
ZNF225	-0.219863193	2.67042588174207e-13	4.00862365034978e-12	1081
CHD2	-0.219929881	2.62562408323108e-13	3.94431000422745e-12	1081
FAM179B	-0.220037419	2.55492640189187e-13	3.84958806029257e-12	1081
KIAA0754	-0.220082853	2.52562105633083e-13	3.81113379623854e-12	1081
ASXL2	-0.220121765	2.50078535226588e-13	3.78215622533005e-12	1081
ZNF561	-0.220185135	2.46085056451946e-13	3.72682471280711e-12	1081
KIAA2026	-0.220363331	2.3518770019529e-13	3.57035133416286e-12	1081
RGPD4	-0.22040536	2.32687281463937e-13	3.53505677608674e-12	1081
GTF2IRD2	-0.220497663	2.27287119692698e-13	3.46084582479924e-12	1081
ABL2	-0.220518044	2.26111355035194e-13	3.44712437850429e-12	1081
NAV2	-0.220715368	2.1503251611756e-13	3.28916479665016e-12	1081
C11orf54	-0.220783821	2.11315056874702e-13	3.23475822244747e-12	1081
KIAA1671	-0.220923637	2.03916858235866e-13	3.12433034364895e-12	1081
PREPL	-0.221114008	1.9425074733323e-13	2.98488276508613e-12	1081
ZNF12	-0.221297005	1.8538341768477e-13	2.86154728780604e-12	1081
SACS	-0.22135711	1.82558541981088e-13	2.82460969908527e-12	1081
PALLD	-0.221523363	1.74962988165059e-13	2.72172154176457e-12	1081
KIAA1109	-0.221887788	1.59381918595183e-13	2.48125869404944e-12	1081
EBF2	-0.221914241	1.58305457640677e-13	2.46640637600265e-12	1081
ZBTB26	-0.221963156	1.56333721846496e-13	2.43945997412677e-12	1081
KIAA0831	-0.222225434	1.46165515621263e-13	2.28433228253712e-12	1081
ZNF836	-0.222281913	1.44062326542462e-13	2.25496159145135e-12	1081
XPO4	-0.222316499	1.42789114673336e-13	2.23677038498783e-12	1081
ZNF654	-0.222331766	1.42230589118324e-13	2.22975503329855e-12	1081
CCDC132	-0.222351784	1.41501530519559e-13	2.22005321831505e-12	1081
NBPF14	-0.222354504	1.41402757855878e-13	2.22005321831505e-12	1081
PRKG1	-0.222421154	1.39003303989213e-13	2.18426018632036e-12	1081
ZNF354C	-0.222432397	1.38602501415658e-13	2.17966228807059e-12	1081
CLN8	-0.222436107	1.38470490918256e-13	2.17928753089708e-12	1081
PFAS	-0.222526247	1.35300808189202e-13	2.13440468361117e-12	1081
TLN2	-0.222543384	1.34706289484664e-13	2.126691380618e-12	1081

SERTAD4	-0.222658977	1.30762686475663e-13	2.07255257203165e-12	1081
TSHZ1	-0.222763516	1.27293875885182e-13	2.02075266328368e-12	1081
RGMB	-0.222784656	1.26603477662402e-13	2.01296531768673e-12	1081
NEU3	-0.222799466	1.26121980644504e-13	2.00689360196172e-12	1081
TEAD1	-0.22294691	1.21425414197744e-13	1.93674977752458e-12	1081
SRFBP1	-0.222999935	1.19778701553724e-13	1.91199836988889e-12	1081
VPS13D	-0.223075428	1.17471979886694e-13	1.87815320223608e-12	1081
FAT1	-0.223106164	1.16545383733942e-13	1.86481870954746e-12	1081
NKAIN2	-0.22315355	1.15130847253001e-13	1.8436493783082e-12	1081
DIP2C	-0.223183803	1.14236597939198e-13	1.83224224959008e-12	1081
RIMS3	-0.223220405	1.13163781157209e-13	1.81938098277094e-12	1081
C3orf63	-0.223241316	1.12555306547971e-13	1.81104365048632e-12	1081
LOC64376	-0.223350292	1.09435927063957e-13	1.76366940056273e-12	1081
UTRN	-0.223353107	1.09356483589537e-13	1.76366940056273e-12	1081
SKI	-0.223381954	1.08545647727883e-13	1.75212505887677e-12	1081
ARHGAP1	-0.223400545	1.08026205044186e-13	1.74513865325993e-12	1081
WRN	-0.223493854	1.05455753248901e-13	1.70635032064186e-12	1081
LIMCH1	-0.223604079	1.02496581133612e-13	1.66247473988455e-12	1081
ATP7A	-0.223686395	1.00339971372458e-13	1.63143561202434e-12	1081
TOM1L2	-0.223780165	9.79375003006286e-14	1.59494821629439e-12	1081
TRIM13	-0.223971874	9.32001401388406e-14	1.52272248426354e-12	1081
SMAD2	-0.224041487	9.15361999187582e-14	1.49918434744991e-12	1081
STAG2	-0.224110967	8.99044943869403e-14	1.47606034182959e-12	1081
CDKN2AI	-0.224208652	8.76586387706303e-14	1.44036156446521e-12	1081
DPY19L3	-0.224210696	8.76122336864102e-14	1.44036156446521e-12	1081
SEMA3E	-0.224216609	8.74781384359178e-14	1.43987212037591e-12	1081
FAM117B	-0.224253908	8.66369047935827e-14	1.42940249555014e-12	1081
ZNF420	-0.224322237	8.51163944717882e-14	1.40546702183129e-12	1081
ZNF169	-0.224332658	8.48868098716878e-14	1.40282591047182e-12	1081
GALNT11	-0.224481854	8.16657799687998e-14	1.35070372534604e-12	1081
SCYL3	-0.224489801	8.14976249963107e-14	1.34903011959793e-12	1081
CSDE1	-0.224660423	7.79680689527391e-14	1.29486129353086e-12	1081
RBM15B	-0.224689556	7.73806038913516e-14	1.28616523547135e-12	1081

PIK3C2A	-0.224784265	7.55006476840485e-14	1.25595420940971e-12	1081
KIAA0556	-0.224872889	7.37821163572055e-14	1.22838077191397e-12	1081
DCP2	-0.224897781	7.33063856065061e-14	1.22146992393967e-12	1081
GRLF1	-0.225100513	6.95422480039022e-14	1.16356194853705e-12	1081
ALDH6A1	-0.225112446	6.9326690027713e-14	1.16091951006507e-12	1081
ZC3H7A	-0.225381821	6.46317530600874e-14	1.08500555449622e-12	1081
DOK6	-0.225439504	6.36677445618389e-14	1.06971368990679e-12	1081
FLRT2	-0.225445955	6.35608027429399e-14	1.06880832325252e-12	1081
SETBP1	-0.225498973	6.26886632070001e-14	1.05590561898413e-12	1081
RAPGEF4	-0.225838654	5.73731448509152e-14	9.70429893385128e-13	1081
ZNF507	-0.225852028	5.71731734097362e-14	9.6786014986482e-13	1081
ITGAV	-0.225883099	5.67112481784999e-14	9.60847850761884e-13	1081
BMPR2	-0.225883788	5.67010460103977e-14	9.60847850761884e-13	1081
TMCO3	-0.225925471	5.60871968286042e-14	9.51875804643835e-13	1081
ZNF498	-0.226026476	5.46266778137206e-14	9.28653522833249e-13	1081
NAPEPLD	-0.226101249	5.35695961869094e-14	9.12222751635917e-13	1081
DENND4C	-0.226215265	5.1996267484111e-14	8.88434952050396e-13	1081
C9orf45	-0.226240386	5.16557649225352e-14	8.83366200648956e-13	1081
SECISBP2	-0.226310937	5.07111431163274e-14	8.68687056189129e-13	1081
LOC10012	-0.226345473	5.0254942191679e-14	8.62338850469654e-13	1081
DMXL1	-0.226384963	4.97382260792917e-14	8.54199969622618e-13	1081
TMC5	-0.226565975	4.7435767083061e-14	8.16746605032704e-13	1081
GRIP1	-0.226615596	4.68230755811675e-14	8.08915632073758e-13	1081
DGKI	-0.226861459	4.39001340727964e-14	7.59766495615536e-13	1081
IPO11	-0.227013483	4.21832399549353e-14	7.31939163559149e-13	1081
MAML3	-0.227112141	4.11045041013542e-14	7.13836409587742e-13	1081
FCHO2	-0.227162598	4.05633227874113e-14	7.0626459598306e-13	1081
KIAA0247	-0.227207976	4.00825904782414e-14	6.99102844315302e-13	1081
ZMYM2	-0.227357556	3.85372336070087e-14	6.7448529193153e-13	1081
KCNIP4	-0.227481646	3.72997470401915e-14	6.55674872709126e-13	1081
TMTC2	-0.227690085	3.53083558905899e-14	6.22398756286777e-13	1081
EPC1	-0.227727669	3.49605675577937e-14	6.17248583218015e-13	1081
ARL13B	-0.227736242	3.48817117429983e-14	6.16396564090088e-13	1081

LIMA1	-0.227784226	3.44435402729974e-14	6.09187988410476e-13	1081
RAD54L2	-0.227851984	3.38339952934525e-14	5.98933071341477e-13	1081
SGK269	-0.227875518	3.36247683799956e-14	5.96277252654058e-13	1081
FAM161B	-0.227963348	3.2855108718153e-14	5.84086433520499e-13	1081
ZNF649	-0.228032714	3.22594996799094e-14	5.74088004462699e-13	1081
ZNF281	-0.228149893	3.1277343344388e-14	5.58088646299996e-13	1081
ATRX	-0.228273176	3.02757357762131e-14	5.41175418998947e-13	1081
DOCK1	-0.228316776	2.99291000079103e-14	5.35454457956797e-13	1081
MLL3	-0.228365498	2.95463541550338e-14	5.29076715069472e-13	1081
CDK14	-0.228432175	2.90303428156971e-14	5.20299160162116e-13	1081
ZNF430	-0.22853007	2.82887378594729e-14	5.07911429749627e-13	1081
KIAA2018	-0.228705992	2.70025156474842e-14	4.86117674458061e-13	1081
KCTD18	-0.228707442	2.69921582522706e-14	4.86117674458061e-13	1081
TOR1AIP1	-0.228801762	2.63266405430954e-14	4.74798723133981e-13	1081
ZNF770	-0.228845257	2.60251939307596e-14	4.70626150570155e-13	1081
HACE1	-0.229252764	2.3360698737649e-14	4.24730393564927e-13	1081
PCDHB19	-0.229469115	2.20569235390335e-14	4.03942476994392e-13	1081
KIAA1328	-0.229471116	2.20452081146911e-14	4.03942476994392e-13	1081
CHD3	-0.229510607	2.18151880212057e-14	4.00243135416383e-13	1081
FRMD4B	-0.229521846	2.17501538834897e-14	3.99413719218688e-13	1081
DCP1A	-0.230049063	1.89044401966179e-14	3.49706104463606e-13	1081
WDR37	-0.230167335	1.83181455494052e-14	3.39796539680264e-13	1081
ZDHHC20	-0.230370477	1.73525530163824e-14	3.22776713310272e-13	1081
ANK3	-0.230379539	1.73106640169463e-14	3.22295126267452e-13	1081
LOC10017	-0.230382718	1.72959916006427e-14	3.22295126267452e-13	1081
SNORD11	-0.230611418	1.62719733860802e-14	3.04362956232669e-13	1081
EEF1A1P9	-0.23065489	1.60841727019273e-14	3.01409915423559e-13	1081
NKTR	-0.230682921	1.59642122444165e-14	2.99440461511891e-13	1081
KLHL24	-0.230807204	1.54428274137198e-14	2.89930809179297e-13	1081
RNF144A	-0.230945144	1.488373146725e-14	2.79955901407798e-13	1081
WASL	-0.231063784	1.4418804880274e-14	2.72227576675838e-13	1081
FAM101B	-0.231088372	1.4324248394542e-14	2.70696044941885e-13	1081
MMP16	-0.231109073	1.42451142045127e-14	2.69453357417754e-13	1081

USP34	-0.231121802	1.41966682135428e-14	2.68789361994192e-13	1081
BMS1P5	-0.23120011	1.39021704483351e-14	2.63461169973387e-13	1081
SMARCA2	-0.231252796	1.37074191915388e-14	2.60015027884697e-13	1081
SEPT10	-0.231394198	1.31978841039097e-14	2.51058900163608e-13	1081
GNA11	-0.231479955	1.28979763868163e-14	2.45585760219674e-13	1081
SLC16A12	-0.231590666	1.25206890133824e-14	2.38853485013815e-13	1081
COL14A1	-0.231605963	1.24694179292322e-14	2.38100876004153e-13	1081
KIAA1009	-0.231895385	1.15373524884715e-14	2.21774776603299e-13	1081
UACA	-0.231900099	1.15227533128525e-14	2.21705697695716e-13	1081
NEK1	-0.231931397	1.1426278886481e-14	2.20059644520229e-13	1081
TANC1	-0.231935614	1.14133413987701e-14	2.20020825338013e-13	1081
PPIP5K2	-0.231954762	1.13547756064792e-14	2.19101489073299e-13	1081
PTPRG	-0.232060137	1.10377232090953e-14	2.13392451100983e-13	1081
FAT4	-0.232116736	1.08710361793888e-14	2.10574061378641e-13	1081
CLASP2	-0.232270435	1.04307654896143e-14	2.02240395368894e-13	1081
LEMD3	-0.232295597	1.03603779950285e-14	2.01141774416583e-13	1081
SPRED1	-0.232334367	1.02528343129539e-14	1.99366165284224e-13	1081
SETD2	-0.23262185	9.48890587509049e-15	1.85226752765211e-13	1081
GAB1	-0.23263049	9.46683659414429e-15	1.8509283851402e-13	1081
FAM116A	-0.232688126	9.32088787615274e-15	1.82477440490862e-13	1081
DGKD	-0.232739834	9.19183545925585e-15	1.80125997399522e-13	1081
ETV3	-0.232816569	9.00354948133877e-15	1.76608085980107e-13	1081
ZBTB41	-0.232947863	8.69014262027299e-15	1.70959885825585e-13	1081
MGAT5	-0.232962756	8.65527758408197e-15	1.70440436883022e-13	1081
ZNF800	-0.233230351	8.05173318255686e-15	1.59805088633111e-13	1081
RNF125	-0.233463697	7.55933601519089e-15	1.50625938700317e-13	1081
KIF13B	-0.233492457	7.5007356133823e-15	1.49606256367907e-13	1081
SCN4B	-0.233640125	7.20680963309653e-15	1.43886204220743e-13	1081
DBT	-0.23364198	7.20319145163234e-15	1.43886204220743e-13	1081
KLHL9	-0.233709204	7.07322832938243e-15	1.41499686887199e-13	1081
TNRC6B	-0.233801536	6.89847428163498e-15	1.3869947330867e-13	1081
MDN1	-0.233816435	6.87067571247264e-15	1.385483105383e-13	1081
KIF27	-0.233951071	6.6244137547229e-15	1.33984754105314e-13	1081

SLC12A6	-0.233959647	6.60902451596357e-15	1.33807838064408e-13	1081
PDE11A	-0.23402291	6.49658447320003e-15	1.31663676270236e-13	1081
ZNF33A	-0.234028347	6.48700839017204e-15	1.31601998006058e-13	1081
EEF2K	-0.234220787	6.15689040336375e-15	1.25410067922915e-13	1081
ANO6	-0.234298592	6.0281592028617e-15	1.22912213706122e-13	1081
RERE	-0.234320803	5.99189833307258e-15	1.22296648348275e-13	1081
PDE3A	-0.234338985	5.96237462427843e-15	1.21817481547758e-13	1081
NBPF10	-0.234750149	5.33163776754853e-15	1.09597798803332e-13	1081
ATXN1	-0.234861964	5.17177006420304e-15	1.06420130687814e-13	1081
ST8SIA6	-0.234922008	5.08787739048317e-15	1.05015666015659e-13	1081
ZNF699	-0.234962178	5.03250024306126e-15	1.04085952152432e-13	1081
FZD4	-0.235011959	4.96469591652394e-15	1.02894855183513e-13	1081
CDK19	-0.23503435	4.93449143992304e-15	1.02374181315396e-13	1081
ANKRD28	-0.235109365	4.83460976160626e-15	1.0040537489439e-13	1081
ANKRD36	-0.235220125	4.69075863518363e-15	9.77200958694667e-14	1081
ZNF148	-0.23526199	4.63748813025839e-15	9.6710350294053e-14	1081
CCNG2	-0.235319819	4.5648831708495e-15	9.52948927220344e-14	1081
ZZZ3	-0.235508075	4.33617770455978e-15	9.10868611661698e-14	1081
BOD1L	-0.235525565	4.31551003156281e-15	9.0747337772268e-14	1081
FERMT2	-0.235555255	4.28064830263606e-15	9.01083177184989e-14	1081
PAFAH1B	-0.23561923	4.20646841617723e-15	8.86394416777096e-14	1081
SASH1	-0.235753038	4.05537779404702e-15	8.5634785808257e-14	1081
KIAA1704	-0.235782889	4.02240600173562e-15	8.51169841438699e-14	1081
CTNNB1	-0.236089465	3.69867127403277e-15	7.87629310944927e-14	1081
BTAF1	-0.236204521	3.58390727790413e-15	7.63998011781786e-14	1081
ZNF397	-0.236208154	3.58034093530732e-15	7.63998011781786e-14	1081
TRIO	-0.236220191	3.56854987398509e-15	7.62337616239976e-14	1081
ADAMTS	-0.236226599	3.56228853846129e-15	7.618078833047e-14	1081
MAP3K1	-0.23633501	3.45798196691532e-15	7.40287425329534e-14	1081
PIBF1	-0.236480436	3.32276521542454e-15	7.12855221136607e-14	1081
HIPK3	-0.236569522	3.24251500825364e-15	6.96380222188375e-14	1081
GTF2I	-0.236602863	3.2129732737626e-15	6.90772108857498e-14	1081
DDX6	-0.23680658	3.03813588183085e-15	6.56687203213331e-14	1081

FLG	-0.237014822	2.86908865572698e-15	6.2215060247169e-14	1081
MKLN1	-0.237037524	2.8512280636041e-15	6.18943850660609e-14	1081
ZFP161	-0.237225851	2.70722093622436e-15	5.89588819029618e-14	1081
ZNF432	-0.237267321	2.67648455790102e-15	5.83525772931993e-14	1081
KIAA0564	-0.237370643	2.60138988278987e-15	5.69001076968533e-14	1081
ZNF614	-0.237446267	2.54774269706659e-15	5.58693335657623e-14	1081
H6PD	-0.237477237	2.52608838929435e-15	5.54940573635057e-14	1081
SNX13	-0.237616883	2.43067349650553e-15	5.35732139902669e-14	1081
MEGF9	-0.237740329	2.34928456557199e-15	5.20069643664261e-14	1081
RAPH1	-0.23778613	2.31977455104785e-15	5.15235483250925e-14	1081
RASSF6	-0.238117875	2.11664100233496e-15	4.72200808328215e-14	1081
TMX3	-0.23824065	2.04598733664153e-15	4.57451885645324e-14	1081
TTBK2	-0.238553122	1.876463348079e-15	4.22361498849738e-14	1081
SIRT1	-0.238788524	1.75794990040197e-15	3.97016824479794e-14	1081
CDKL5	-0.238804986	1.74994318072128e-15	3.95652136651293e-14	1081
RAPGEF2	-0.238827047	1.73926974192812e-15	3.94123610249067e-14	1081
USP53	-0.238876003	1.71581135013132e-15	3.89684550714716e-14	1081
KIAA1712	-0.239014239	1.65124056125774e-15	3.75867131147314e-14	1081
GSTCD	-0.239092586	1.61571132066768e-15	3.68195752882923e-14	1081
SRRM2	-0.239107541	1.60901546756101e-15	3.67085125640051e-14	1081
RBM16	-0.239667165	1.37715185775314e-15	3.17059704850708e-14	1081
DST	-0.239670321	1.37594242285239e-15	3.17059704850708e-14	1081
ZNF704	-0.239722655	1.35603745137946e-15	3.12913796770208e-14	1081
SHROOM	-0.239908648	1.28756133051399e-15	2.98480126619151e-14	1081
VHL	-0.239934439	1.27833845962251e-15	2.96683505404327e-14	1081
FLJ10213	-0.240021554	1.24766438596429e-15	2.89898489679938e-14	1081
FOXP2	-0.240057022	1.23538423425909e-15	2.87376621237291e-14	1081
PDGFD	-0.240121924	1.21322008843819e-15	2.82547036781356e-14	1081
LOC20003	-0.240176807	1.19478304290794e-15	2.78575282400237e-14	1081
HMBOX1	-0.240201952	1.18642833379959e-15	2.76947842229347e-14	1081
ZNF717	-0.240262096	1.1666771972097e-15	2.72969943528332e-14	1081
ZNF445	-0.240278553	1.16132946745905e-15	2.72034675836775e-14	1081
SH3RF1	-0.240451169	1.10666952802157e-15	2.59532685005756e-14	1081

MSRB3	-0.240634566	1.05136801344655e-15	2.47138957186472e-14	1081
RPS6KA5	-0.240667919	1.04160696468952e-15	2.45130517566242e-14	1081
OTUD3	-0.240847167	9.90656608722131e-16	2.33412600967337e-14	1081
PAN3	-0.240868479	9.84764156851653e-16	2.32295947772559e-14	1081
RSBN1	-0.241020346	9.43763102939678e-16	2.23146804092956e-14	1081
MTR	-0.24112807	9.15702510318988e-16	2.170214949456e-14	1081
ZNF791	-0.241180158	9.02430291268955e-16	2.1412789420039e-14	1081
MTMR3	-0.241226217	8.90852099721917e-16	2.11629900340779e-14	1081
ARHGEF7	-0.241264778	8.81271175383579e-16	2.09601036931549e-14	1081
ZNF568	-0.241405954	8.47051792618322e-16	2.02178416614883e-14	1081
GTF2IRD2	-0.241414181	8.45098434553847e-16	2.01951458648722e-14	1081
FAM63B	-0.241449959	8.36655194930607e-16	2.00171245865524e-14	1081
TAB3	-0.241559458	8.11326929384693e-16	1.94573583243508e-14	1081
TOP2B	-0.241907922	7.35640385122679e-16	1.77691553456791e-14	1081
PAR5	-0.242142456	6.88656455397299e-16	1.67144389084079e-14	1081
FAM122A	-0.242200491	6.77493819150454e-16	1.64633449780288e-14	1081
OSBPL8	-0.242365583	6.46704014992953e-16	1.57531467739214e-14	1081
GNAQ	-0.242411811	6.3833198291615e-16	1.55770761358263e-14	1081
HOXA6	-0.242416199	6.37542907358455e-16	1.55770761358263e-14	1081
DOCK9	-0.242542776	6.15188685295009e-16	1.5094976936989e-14	1081
BCKDHB	-0.242559411	6.1230894899118e-16	1.50426387529601e-14	1081
SLC25A36	-0.242664464	5.94425866779995e-16	1.46345330489549e-14	1081
LOC65062	-0.242832781	5.66839385473881e-16	1.40110176937072e-14	1081
PIGN	-0.242925464	5.52190776046456e-16	1.36657041565797e-14	1081
C4orf36	-0.243241212	5.05028637268575e-16	1.25447618961473e-14	1081
RUNX1	-0.243270849	5.00811006865734e-16	1.24553552263089e-14	1081
NAA16	-0.24342061	4.80022643110547e-16	1.19678912691361e-14	1081
PDS5B	-0.243517885	4.6697731237702e-16	1.16725535234116e-14	1081
KIAA0427	-0.243819334	4.28726932327646e-16	1.07555467643094e-14	1081
GMCL1	-0.244394759	3.64077160364189e-16	9.21398793409119e-15	1081
CLCN3	-0.244424922	3.60966774545095e-16	9.1582817042959e-15	1081
VPS4B	-0.244449235	3.58478765268281e-16	9.10662638881402e-15	1081
JMY	-0.244573731	3.46000751830135e-16	8.81186491228581e-15	1081

SOCS7	-0.244603834	3.43048326479157e-16	8.75881943843171e-15	1081
ZNF280D	-0.244851616	3.19670273236968e-16	8.19307589613068e-15	1081
KREMEN	-0.244876764	3.17387125165021e-16	8.14492182987179e-15	1081
MTMR9	-0.244893527	3.15874205661321e-16	8.11643606256036e-15	1081
TMEM132	-0.245053156	3.01817716775945e-16	7.79502295442488e-15	1081
KBTBD7	-0.245196718	2.89702979584982e-16	7.51080832100265e-15	1081
EFHA2	-0.24535765	2.76690973869816e-16	7.21078870453745e-15	1081
ZKSCAN1	-0.245725082	2.49100185200987e-16	6.51704315697907e-15	1081
WDR36	-0.245754371	2.47021223726051e-16	6.47105663454005e-15	1081
KLHL2	-0.245899151	2.36992773959414e-16	6.22453641644379e-15	1081
ARHGAP3	-0.24597437	2.31941922047549e-16	6.09983031285624e-15	1081
ZNF749	-0.246009225	2.29637521792726e-16	6.04712140720845e-15	1081
GNG12	-0.246084465	2.24739691590871e-16	5.93365804337891e-15	1081
FNBP1L	-0.246091706	2.24273756164604e-16	5.92912705765874e-15	1081
KIAA0430	-0.246241335	2.14856358864069e-16	5.69510703857457e-15	1081
ZNF616	-0.246326763	2.09655374486292e-16	5.57190965570759e-15	1081
BCLAF1	-0.246340224	2.08847281564984e-16	5.55776550479076e-15	1081
ZC3H13	-0.246639259	1.91664762174508e-16	5.1208045543839e-15	1081
SPATA6	-0.246709058	1.87858929429436e-16	5.02578769369985e-15	1081
IMPACT	-0.246757111	1.85282171373339e-16	4.96344327435627e-15	1081
KLHDC10	-0.247387223	1.54547060732193e-16	4.17899401134232e-15	1081
RNF160	-0.247391849	1.54341121828923e-16	4.17899401134232e-15	1081
ANKRD50	-0.247466939	1.51035619938233e-16	4.1005560157085e-15	1081
ZNF395	-0.248335581	1.17512807499753e-16	3.23401025557722e-15	1081
CDC14B	-0.248443579	1.13894955232469e-16	3.14303270295628e-15	1081
SETD7	-0.248454539	1.1353400782266e-16	3.13736980464676e-15	1081
ZNF397OS	-0.248539249	1.1078186230842e-16	3.07260082950273e-15	1081
ZNF41	-0.248634104	1.0777803486429e-16	2.99887916069216e-15	1081
BAT2L1	-0.24868249	1.06276802839855e-16	2.9611980542308e-15	1081
ARHGEF1	-0.248773483	1.03509224715729e-16	2.88807940706144e-15	1081
ZNF615	-0.248874876	1.00508836343774e-16	2.808253131963e-15	1081
TSPAN6	-0.249068671	9.50105186113287e-17	2.66201237472214e-15	1081
ATP2B1	-0.249642178	8.04162758604142e-17	2.26255010783246e-15	1081

CBL	-0.249808056	7.66233429759687e-17	2.16187289110769e-15	1081
ZNF827	-0.249811564	7.65450553509757e-17	2.16187289110769e-15	1081
CSRNP3	-0.249820871	7.63377557874671e-17	2.15986529541928e-15	1081
FBXL18	-0.249937386	7.37888188298942e-17	2.09068320018034e-15	1081
KIDINS22	-0.250014637	7.2145260695153e-17	2.05277722698285e-15	1081
PLA2R1	-0.250127671	6.98051681146188e-17	1.98900298680197e-15	1081
KLF7	-0.250313183	6.61255921789339e-17	1.88682727258445e-15	1081
KIAA143(	-0.250474594	6.30799371120178e-17	1.80760360330242e-15	1081
PLEKHM:	-0.250823813	5.69554946932252e-17	1.64615271247492e-15	1081
KLHL20	-0.250854096	5.64528641524544e-17	1.63396975337815e-15	1081
PRKAR2A	-0.250871936	5.61588095556287e-17	1.62779743668797e-15	1081
ZC3H6	-0.25113693	5.19645646846249e-17	1.51275456007481e-15	1081
SCN7A	-0.251204919	5.09391707895566e-17	1.48720231239944e-15	1081
ARHGEF1	-0.251676886	4.43502268410326e-17	1.30809259775792e-15	1081
ZMYND1	-0.252130334	3.88134621560236e-17	1.15154226087348e-15	1081
C17orf103	-0.252202864	3.79934122987355e-17	1.12960904213803e-15	1081
GNRHR	-0.252609027	3.3706946316719e-17	1.01498719514246e-15	1081
FBXW2	-0.252641029	3.33902117875654e-17	1.00695481506064e-15	1081
THUMPD	-0.252716125	3.26584335348199e-17	9.86363033821511e-16	1081
MTMR12	-0.252758648	3.22511007577565e-17	9.7778168563904e-16	1081
ZHX3	-0.252778696	3.20607936356286e-17	9.74154883544101e-16	1081
FBXO30	-0.25282589	3.16171643837767e-17	9.6212655062112e-16	1081
GPRASP2	-0.252865733	3.12473467077128e-17	9.54397478766365e-16	1081
ZNF33B	-0.252913991	3.08051255252938e-17	9.43114367335932e-16	1081
CRK	-0.253009364	2.99492119331195e-17	9.18964673699652e-16	1081
LYRM7	-0.253357634	2.70180685690049e-17	8.31396752017449e-16	1081
RAB11FIF	-0.253435724	2.64006903945682e-17	8.14459277180054e-16	1081
MLL5	-0.253489654	2.59824508354711e-17	8.02786000123567e-16	1081
MYSM1	-0.253492092	2.59637014236669e-17	8.02786000123567e-16	1081
CREB1	-0.253652377	2.47596673985958e-17	7.68541602071975e-16	1081
ZNF407	-0.25367076	2.4625150275391e-17	7.67915870429956e-16	1081
EEF2	-0.253680177	2.45565170452029e-17	7.6696284631878e-16	1081
AKAP9	-0.254136106	2.14497433224504e-17	6.79410501935161e-16	1081

FOXO3	-0.254317099	2.03268191148458e-17	6.44856332391445e-16	1081
KIAA0355	-0.254391859	1.98800778678092e-17	6.3267641176464e-16	1081
SCAI	-0.254423906	1.96915485163416e-17	6.27668108958387e-16	1081
NUP43	-0.254459057	1.94867906996077e-17	6.22511640582772e-16	1081
AKAP11	-0.254469074	1.94288201492731e-17	6.2232781312909e-16	1081
VPS13A	-0.254500972	1.92453648348423e-17	6.18337918018975e-16	1081
MYO9A	-0.254686539	1.82114645948479e-17	5.86991926821137e-16	1081
COL4A3B	-0.254761961	1.78070608489093e-17	5.75799744464331e-16	1081
ZFP112	-0.254807632	1.75664960076203e-17	5.68934183397929e-16	1081
KLF12	-0.255400259	1.47206742728326e-17	4.79851105544035e-16	1081
ARNT	-0.255445259	1.45241651588902e-17	4.74982641438056e-16	1081
ZNF192	-0.255838904	1.29117857822816e-17	4.25708550874081e-16	1081
C20orf194	-0.256132905	1.18238924920216e-17	3.92409084434556e-16	1081
SMAD9	-0.256179521	1.16599040238977e-17	3.88054959777586e-16	1081
ELF2	-0.256306773	1.12235754086889e-17	3.74956760544011e-16	1081
WDR35	-0.256371674	1.10072783153882e-17	3.68341564225077e-16	1081
CLDN12	-0.256950727	9.25061300872373e-18	3.12673823927415e-16	1081
PGAP1	-0.257009654	9.08815439620368e-18	3.07698941700039e-16	1081
MBLAC2	-0.257035922	9.01664365742853e-18	3.05791728078952e-16	1081
NBEAL1	-0.257213928	8.54645420151781e-18	2.92803265118327e-16	1081
KIAA1012	-0.25726648	8.41232122446138e-18	2.88698826348849e-16	1081
RYK	-0.257389044	8.10750023554628e-18	2.78712614752696e-16	1081
PKP2	-0.257465489	7.92292104836823e-18	2.72832896614321e-16	1081
FOXO3B	-0.257744278	7.28419694299579e-18	2.51267375713442e-16	1081
TSC1	-0.257927973	6.89136535423833e-18	2.38944156731723e-16	1081
RASAL2	-0.258102154	6.53819843373153e-18	2.27089668012968e-16	1081
ZFP37	-0.258206205	6.33580084290497e-18	2.20439910155994e-16	1081
HEATR5B	-0.25824067	6.2701334578045e-18	2.1853259257348e-16	1081
RYBP	-0.258464197	5.8602221059749e-18	2.05311607521503e-16	1081
RFX3	-0.259000967	4.98067014431281e-18	1.76027368521371e-16	1081
SMAD5	-0.259539323	4.2294919400456e-18	1.5106935307131e-16	1081
ATP9B	-0.259912182	3.77587870313951e-18	1.36073482065734e-16	1081
LRP4	-0.259937156	3.74727121415621e-18	1.35284549478812e-16	1081

DYNC2H1	-0.260096592	3.56960828662767e-18	1.30035730441436e-16	1081
TCP11L2	-0.260682542	2.98518146250133e-18	1.09738103215491e-16	1081
CREBBP	-0.260948268	2.75227873372106e-18	1.01546987345807e-16	1081
ERC1	-0.261149068	2.58825469084953e-18	9.56704417379152e-17	1081
TET2	-0.261289838	2.47905203791979e-18	9.18023957792173e-17	1081
CBX5	-0.261690742	2.19233160788354e-18	8.16349727186949e-17	1081
ARID1B	-0.261766124	2.14219517107743e-18	8.006404772051e-17	1081
FNIP2	-0.262288711	1.82438964307966e-18	6.86959427286725e-17	1081
SLC39A10	-0.262295674	1.82048613405129e-18	6.86773280345755e-17	1081
ALS2CR8	-0.262573405	1.67131484620421e-18	6.32869127383153e-17	1081
SPRED2	-0.262589487	1.66305639001337e-18	6.30927890335581e-17	1081
RAB33B	-0.262769721	1.573210545154e-18	6.00233455153926e-17	1081
TBC1D5	-0.264095472	1.04422418144559e-18	4.0925867967357e-17	1081
AMOTL1	-0.264149791	1.02678516298663e-18	4.03208325699137e-17	1081
MPDZ	-0.264421809	9.43682301802728e-19	3.73469220993575e-17	1081
ZBTB44	-0.264580922	8.98185804170913e-19	3.57588004447096e-17	1081
SNX30	-0.264690949	8.67996825960614e-19	3.46940397995567e-17	1081
GABPA	-0.265045681	7.77321065147049e-19	3.13182657147746e-17	1081
AHNAK	-0.265093506	7.6583398821232e-19	3.09172859569883e-17	1081
PBRM1	-0.265649953	6.43884368442953e-19	2.63104474691345e-17	1081
WASF2	-0.265817732	6.11027176084814e-19	2.51081493273562e-17	1081
ZNF621	-0.265992725	5.78519337020783e-19	2.39307434174203e-17	1081
SLC12A2	-0.266068188	5.65033043192105e-19	2.34692590826906e-17	1081
GOPC	-0.266189266	5.44039745268616e-19	2.26908502452097e-17	1081
VGLL4	-0.266689964	4.650966199828e-19	1.94385298953392e-17	1081
MCM9	-0.266873126	4.39136694626243e-19	1.83917021065398e-17	1081
PDPK1	-0.266911459	4.33887413463904e-19	1.82097123838132e-17	1081
DIXDC1	-0.267220579	3.93758330764305e-19	1.65946894837802e-17	1081
VPS36	-0.267740818	3.34327995088701e-19	1.42089397912698e-17	1081
PUM1	-0.267853386	3.22683743029043e-19	1.37721694985595e-17	1081
SIK2	-0.267872366	3.20760197573608e-19	1.37191383866674e-17	1081
RPL23AP5	-0.268055733	3.02750015070422e-19	1.30597410141192e-17	1081
C9orf110	-0.268190577	2.90145947125992e-19	1.25698711932325e-17	1081

CCNI	-0.268202279	2.89076852329329e-19	1.25505456684792e-17	1081
ZNF713	-0.268465808	2.66004721901796e-19	1.16240024353833e-17	1081
YAP1	-0.268566393	2.57685988806018e-19	1.13095517309308e-17	1081
PCDHB18	-0.268594522	2.55405845807762e-19	1.12585355881781e-17	1081
FOXK1	-0.268831806	2.36947727947575e-19	1.04907955593492e-17	1081
RNF180	-0.269470727	1.9353911017989e-19	8.62576410303955e-18	1081
IL17RD	-0.269504134	1.91499274298842e-19	8.55377578880303e-18	1081
SLC23A2	-0.269852015	1.71478548731294e-19	7.6936199647927e-18	1081
LATS1	-0.270062686	1.6037389942155e-19	7.24379417902942e-18	1081
TGOLN2	-0.270152917	1.55837634899236e-19	7.05471720234858e-18	1081
SH3D19	-0.270701589	1.30856987215802e-19	5.9640588404125e-18	1081
PPP1R12E	-0.270864948	1.24214523556917e-19	5.68704903875932e-18	1081
OSBPL1A	-0.271516527	1.00873548569774e-19	4.65010900672335e-18	1081
ZC3H12B	-0.271896021	8.93337663674527e-20	4.12758881530352e-18	1081
LSM11	-0.272296149	7.85768098182238e-20	3.66418943006509e-18	1081
APPL2	-0.272699187	6.90357176304785e-20	3.23424309689765e-18	1081
ARID5B	-0.273143883	5.98318685866305e-20	2.81615185205063e-18	1081
SLIT2	-0.273254927	5.77294434351012e-20	2.7299522018782e-18	1081
AFAP1	-0.273697312	5.00536940002934e-20	2.37254509561391e-18	1081
TTC3	-0.27401302	4.52009557370848e-20	2.14757842764994e-18	1081
APPL1	-0.274121509	4.36430545037874e-20	2.07846178009172e-18	1081
POGZ	-0.274551155	3.79771507230673e-20	1.82154690789569e-18	1081
TNFRSF1(	-0.274969949	3.31551791604645e-20	1.59787340714726e-18	1081
CTDSP2	-0.275622416	2.68208352433616e-20	1.30194150837956e-18	1081
KCTD7	-0.278083319	1.19931715777902e-20	5.95079905011292e-19	1081
MED23	-0.280142881	6.07666666197584e-21	3.07573994737446e-19	1081
KIAA024(	-0.280253649	5.85752835493656e-21	2.97228989194451e-19	1081
NDST1	-0.280481778	5.43050306156668e-21	2.7625627316985e-19	1081
GPAM	-0.281776368	3.52940920986647e-21	1.8230756034041e-19	1081
LRP6	-0.282264218	2.99863944296165e-21	1.56091967903004e-19	1081
RREB1	-0.283729191	1.8345814132093e-21	9.70016865330742e-20	1081
SORL1	-0.284252344	1.53824691822917e-21	8.21962444767286e-20	1081
PRKAA1	-0.285127251	1.14474221152682e-21	6.19914834709889e-20	1081

ZFP106	-0.28599149	8.54095929313969e-22	4.71390753315888e-20	1081
DDX17	-0.286317938	7.6443965311066e-22	4.2306694538226e-20	1081
HOOK3	-0.286630205	6.87404815691932e-22	3.81481267551349e-20	1081
CRAMP1I	-0.286743433	6.61410808268379e-22	3.68069633496312e-20	1081
SMAD4	-0.287189501	5.68124113844699e-22	3.17912785372263e-20	1081
MAGI1	-0.287296297	5.47794239678887e-22	3.08249021182435e-20	1081
MAP3K4	-0.287912689	4.43754389207721e-22	2.504042624815e-20	1081
EEF1A1	-0.288105019	4.154833125607e-22	2.35772150184093e-20	1081
THRB	-0.288788237	3.28713301589056e-22	1.88122996037259e-20	1081
SPIN1	-0.289145827	2.90708578922957e-22	1.67802989180601e-20	1081
CEP68	-0.289472361	2.59815845237062e-22	1.50835452515868e-20	1081
ACVR2B	-0.291210038	1.42539930490576e-22	8.49546420039249e-21	1081
ASB1	-0.294204356	5.01633089924507e-23	3.1480992512552e-21	1081
FBXL3	-0.294545379	4.45028295451814e-23	2.81037461187361e-21	1081
BICD2	-0.294907881	3.91774838399568e-23	2.48185664137085e-21	1081
NALCN	-0.295353975	3.34820836175864e-23	2.14125896659136e-21	1081
HBP1	-0.295731564	2.93069685317438e-23	1.88021936647127e-21	1081
SOCS5	-0.297173869	1.75886105977159e-23	1.1617133130852e-21	1081
RPS3A	-0.297357922	1.64757094534919e-23	1.09178673335722e-21	1081
SOCS6	-0.297750238	1.43306773359419e-23	9.59107956586545e-22	1081
ASH1L	-0.29815197	1.2420470411791e-23	8.39632135723254e-22	1081
PRDM10	-0.298286336	1.18395765266635e-23	8.05771179492016e-22	1081
MLL	-0.300055851	6.28577418183143e-24	4.41208783599283e-22	1081
PTPN21	-0.300303016	5.7517196058843e-24	4.05134235876011e-22	1081
LCA5	-0.302289008	2.809282360061e-24	2.02842269331286e-22	1081
SOS2	-0.304191897	1.4065009548703e-24	1.03032588130408e-22	1081
KIRREL	-0.305389765	9.07522970448659e-25	6.74614399988495e-23	1081
SEMA5A	-0.30564318	8.26968016648044e-25	6.17010025754624e-23	1081
RBM9	-0.307675498	3.9111254920684e-25	2.96201590367361e-23	1081
PRKAB2	-0.316193638	1.58958548223861e-26	1.29644532549379e-24	1081
CYP20A1	-0.316898652	1.21369580565649e-26	9.93898455485776e-25	1081
TBC1D14	-0.31720922	1.07744049134328e-26	8.89550766315998e-25	1081
CCDC93	-0.318785517	5.87437795040732e-27	4.99322125784622e-25	1081

SHPRH	-0.319669741	4.17348388508314e-27	3.62391520969827e-25	1081
USP22	-0.321409552	2.12288617419657e-27	1.86749091612183e-25	1081
EIF3L	-0.323198076	1.0546744705762e-27	9.40106956183962e-26	1081
KIAA1143	-0.324291956	6.8595485159396e-28	6.16900021667872e-26	1081
ATF7	-0.324970896	5.24750827423595e-28	4.78330561920739e-26	1081
EIF4B	-0.327448029	1.96315680956059e-28	1.83943227574875e-26	1081
NR2C2	-0.32915107	9.93320298652671e-29	9.4388855737538e-27	1081
HDAC4	-0.33087098	4.97028691138133e-29	4.88421608925741e-27	1081
SH3PXD2	-0.334755477	1.02355866058491e-29	1.03097946087416e-27	1081
MIB1	-0.339761572	1.29138109152085e-30	1.36920379414145e-28	1081
CMTM4	-0.346916739	6.2673497114907e-32	7.2980208056636e-30	1081
BTBD7	-0.347321111	5.26985469023421e-32	6.20825863946013e-30	1081
C7orf41	-0.350651332	1.2519110541514e-32	1.52846958702303e-30	1081
UNC119B	-0.35779665	5.40363264509284e-34	6.97796023303816e-32	1081
TMEM168	-0.395753997	7.49885439433117e-42	1.28020696418476e-39	1081
FAM168B	-0.403848579	1.15141822728147e-43	2.10866547168956e-41	1081
FAM172A	-0.442082947	6.13394865628281e-53	1.34313473566106e-50	1081

Table S3 KEGG pathway enrichment analysis of IFI6

Category	Term	Term	Count	%	PValue	Genes	List Total	Pop Hits	Pop Total	Fold Enrichmer	Bonferroni	Benjamini	FDR
KEGG_PATHW	hsa05160	Hepatitis C	10	55.55555556	4.33E-14	RSAD2, OAS1, STAT1, OAS2, MX2, OAS3, MX1, IRF7, IFIT1, IRF9	13	157	8465	41.47476727	1.52E-12	1.52E-12	1.26E-12
KEGG_PATHW	hsa05164	Influenza A	9	50	1.09E-11	RSAD2, OAS1, STAT1, OAS2, MX2, OAS3, MX1, IRF7, IRF9	13	171	8465	34.27125506	3.81E-10	1.90E-10	1.58E-10
KEGG_PATHW	hsa05162	Measles	8	44.44444444	2.05E-10	OAS1, STAT1, OAS2, MX2, OAS3, MX1, IRF7, IRF9	13	139	8465	37.47648035	7.16E-09	2.39E-09	1.98E-09
KEGG_PATHW	hsa05171	Coronavirus disease - COVID-19	8	44.44444444	7.48E-09	OAS1, STAT1, OAS2, MX2, OAS3, MX1, ISG15, IRF9	13	232	8465	22.4535809	2.62E-07	6.55E-08	5.42E-08
KEGG_PATHW	hsa05169	Epstein-Barr virus infection	7	38.88888889	1.41E-07	OAS1, STAT1, OAS2, OAS3, IRF7, ISG15, IRF9	13	202	8465	22.56473724	4.92E-06	9.84E-07	8.16E-07
KEGG_PATHW	hsa04621	NOD-like receptor signaling pathway	6	33.33333333	3.39E-06	OAS1, STAT1, OAS2, OAS3, IRF7, IRF9	13	186	8465	21.00496278	1.19E-04	1.98E-05	1.64E-05
KEGG_PATHW	hsa05165	Human papillomavirus infection	6	33.33333333	5.60E-05	STAT1, MX2, MX1, ISG15, IRF9, OASL	13	331	8465	11.80339298	0.001957733	2.80E-04	2.32E-04
KEGG_PATHW	hsa05168	Herpes simplex virus 1 infection	6	33.33333333	4.40E-04	OAS1, STAT1, OAS2, OAS3, IRF7, IRF9	13	512	8465	7.630709135	0.015300488	0.001926919	0.00159659
KEGG_PATHW	hsa05167	Kaposi sarcoma-associated herpesvirus infection	3	16.66666667	0.029652949	STAT1, IRF7, IRF9	13	194	8465	10.06938937	0.651303042	0.115317024	0.095548392
KEGG_PATHW	hsa03250	Viral life cycle - HIV-1	2	11.11111111	0.085795901	MX2, MX1	13	63	8465	20.67155067	0.956697722	0.300285654	0.248808113
KEGG_PATHW	hsa04622	RIG-I-like receptor signaling pathway	2	11.11111111	0.096193705	IRF7, ISG15	13	71	8465	18.34236186	0.970983992	0.306070879	0.253601586

Table S4 The correlation analysis between IFI6 and immune cells (correlation coefficients)

	IFI6	B.cells.na	B.cells.me	Plasma.ce	T.cells.CI	T.cells.CI	T.cells.CI	T.cells.CI	T.cells.fo	T.cells.re	T.cells.ga	NK.cells.r	NK.cells.a	Monocytes	Macrophage	Macrophage	Macrophage	Dendritic.	Dendritic.	Mast.cells	Mast.cells	Eosinophi	Neutrophi	P.value	Correlatio	RMSE
IFI6	1	-0.240462	-0.014852	-0.167202	0.03668529	-0.016471	-0.1068	0.17882770	0.17672008	0.29448720	-0.020073	0.04047840	-0.025708	-0.037206	0.08675367	0.35223729	-0.089215	-0.074007	0.11404179	-0.139924	-0.106506	-0.072099	0.00709908	0.04587728	-0.120299	0.122253054040071
B.cells.na	-0.240462	1	-0.113143	0.48229962	0.08786320	0.01367168	0.15780567	-0.101008	-0.030459	-0.065354	-0.041719	-0.159175	-0.02091	-0.07741	-0.305289	-0.063028	-0.313803	-0.026521	-0.096556	-0.041433	0.03278589	-0.028102	-0.065131	-0.377681	0.43377807	-0.428582
B.cells.me	-0.014852	-0.113143	1	-0.026712	0.01520124	0.03233604	-0.067247	0.03520714	0.02845198	0.14716527	-0.035432	0.07494767	0.03049609	0.01272221	0.02277071	-0.09747	-0.061511	0.01337921	0.00331747	0.02552708	-0.029494	0.01758090	-0.035455	0.01957574	0.01077124	0.000292296806206077
Plasma.ce	-0.167202	0.48229962	-0.026712	1	0.03831181	-0.00621	-0.060257	-0.040697	-0.0661	-0.047071	-0.053153	-0.094873	0.06248986	-0.035341	-0.211126	-0.14504	-0.19235	-0.058114	-0.065417	-0.017635	-0.033703	-0.034353	-0.056789	-0.306427	0.48233905	-0.462979
T.cells.CI	0.03668529	0.08786320	0.01520124	0.03831181	1	-0.013869	-0.034001	0.38934899	0.25256041	0.24007145	-0.023278	-0.150789	0.07950754	-0.01848	-0.393741	0.24015740	-0.345033	0.04933133	-0.074567	-0.125984	-0.101898	-0.048598	-0.128053	0.00312741	-0.110346	0.0979061739424938
T.cells.CI	-0.016471	0.01367168	0.03233604	-0.00621	-0.013869	1	-0.044684	-0.0162	-0.047844	-0.022012	-0.014408	0.04185725	0.01326533	0.17434877	-0.038175	-0.038199	0.06082060	0.03500420	-0.013664	-0.002798	-0.008088	0.03013022	-0.008427	-0.017399	0.02405837	-0.023377
T.cells.CI	-0.1068	0.15780567	-0.067247	-0.060257	-0.034001	-0.044684	1	-0.044877	-0.173841	-0.250448	0.03039430	-0.00831	-0.134896	0.09580461	-0.446342	0.15088184	-0.099885	0.06268266	-0.064604	-0.009883	0.00032453	-0.011877	-0.029749	0.07969775	-0.160867	0.106715115070746
T.cells.CI	0.17882770	-0.101008	0.03520714	-0.040697	0.38934899	-0.0162	-0.044877	1	0.15362679	0.07561858	0.08590749	0.14099425	-0.142316	-0.120907	-0.117953	0.28618480	-0.218435	0.01176849	0.00246630	-0.242353	-0.080261	-0.033345	-0.025362	0.06441371	-0.115941	0.107804093800376
T.cells.fo	0.17672008	-0.030459	0.02845198	-0.0661	0.25256041	-0.047844	-0.173841	0.15362679	1	0.21252661	-0.018834	-0.139017	0.11961456	-0.100504	-0.036572	0.34976135	-0.346965	-0.009002	0.14903698	-0.297161	-0.056062	-0.048409	-0.111978	0.06178839	-0.175289	0.182524502237585
T.cells.re	0.29448720	-0.065354	0.14716527	-0.047071	0.24007145	-0.022012	-0.250448	0.07561858	0.21252661	1	-0.098445	0.00176462	0.08704876	-0.170858	0.07856068	0.21766702	-0.360525	0.06210327	-0.081292	-0.152669	-0.113852	-0.079904	-0.15931	-0.008716	-0.02418	0.0366893479786955
T.cells.ga	-0.020073	-0.041719	-0.035432	-0.053153	-0.023278	-0.014408	0.03039430	0.08590749	-0.018834	-0.098445	1	-0.05214	0.02070512	-0.086455	-0.113498	0.03217364	0.13392799	-0.017607	-0.017173	-0.042932	0.07185597	-0.00495	0.06604093	0.02156729	-0.06009	0.0570111308710069
NK.cells.r	0.04047840	-0.159175	0.07494767	-0.094873	-0.150789	0.04185725	-0.00831	0.14099425	-0.139017	0.00176462	-0.05214	1	-0.262279	-0.001736	0.17612778	-0.039025	0.01913748	-0.075196	0.00759252	-0.12566	0.01034147	-0.024343	0.03415378	0.05918694	-0.017203	0.0366358477874278
NK.cells.a	-0.025708	-0.02091	0.03049609	0.06248986	0.07950754	0.01326533	-0.134896	-0.142316	0.11961456	0.08704876	0.02070512	-0.262279	1	0.02175455	-0.171743	-0.060614	-0.019356	0.00920229	0.04059135	0.12709700	0.11039975	0.04560636	0.04330140	0.05853645	-0.056682	0.0513237495407205
Monocytes	-0.037206	-0.07741	0.01272221	-0.035341	-0.01848	0.17434877	0.09580461	-0.120907	-0.100504	-0.170858	-0.086455	-0.001736	0.02175455	1	-0.335079	-0.136739	0.23629534	0.05692375	0.12555091	0.13602225	0.02000718	0.14232236	0.03319555	0.08651856	-0.094981	0.0278448433566933
Macrophage	0.08675367	-0.305289	0.02277071	-0.211126	-0.393741	-0.038175	-0.446342	-0.117953	-0.036572	0.07856068	-0.113498	0.17612778	-0.171743	-0.335079	1	-0.187877	-0.112877	-0.23389	-0.00308	-0.244666	-0.018062	-0.038272	-0.036833	0.01134064	0.08674258	0.0370218011967145
Macrophage	0.35223729	-0.063028	-0.09747	-0.14504	0.24015740	-0.038199	0.15088184	0.28618480	0.34976135	0.21766702	0.03217364	-0.039025	-0.060614	-0.136739	-0.187877	1	-0.347091	0.04641851	-0.149604	-0.192291	-0.146329	-0.133162	-0.127001	0.05316673	-0.227121	0.210680506694956
Macrophage	-0.089215	-0.313803	-0.061511	-0.19235	-0.345033	0.06082060	-0.099885	-0.218435	-0.346965	-0.360525	0.13392799	0.01913748	-0.019356	0.23629534	-0.112877	-0.347091	1	-0.115908	-0.021049	0.14184232	0.10812556	0.09826340	0.20621207	0.13352657	-0.089659	0.0370642720559718
Dendritic.	-0.074007	-0.026521	0.01337921	-0.058114	0.04933133	0.03500420	0.06268266	0.01176849	-0.009002	0.06210327	-0.017607	-0.075196	0.00920229	0.05692375	-0.23389	0.04641851	-0.115908	1	0.00553513	0.07159112	-0.013471	0.01283836	-0.045062	-0.019216	-0.032117	-0.020246
Dendritic.	0.11404179	-0.096556	0.00331747	-0.065417	-0.074567	-0.013664	-0.064604	0.00246630	0.14903698	-0.081292	-0.017173	0.00759252	0.04059135	0.12555091	-0.00308	-0.149604	-0.021049	0.00553513	1	-0.111857	0.01716477	0.06322577	0.09397335	0.09572644	-0.071054	0.0771752400463624
Mast.cells	-0.139924	-0.041433	0.02552708	-0.017635	-0.125984	-0.002798	-0.009883	-0.242353	-0.297161	-0.152669	-0.042932	-0.12566	0.12709700	0.13602225	-0.244666	-0.192291	0.14184232	0.07159112	-0.111857	1	-0.185186	0.00253280	0.01756173	-0.009743	-0.032432	-0.045342
Mast.cells	-0.106506	0.03278589	-0.029494	-0.033703	-0.101898	-0.008088	0.00032453	-0.080261	-0.056062	-0.113852	0.07185597	0.01034147	0.11039975	0.02000718	-0.018062	-0.146329	0.10812556	-0.013471	0.01716477	-0.185186	1	0.09467942	0.04537326	0.00271038	0.03001248	-0.032144
Eosinophi	-0.072099	-0.028102	0.01758090	-0.034353	-0.048598	0.03013022	-0.011877	-0.033345	-0.048409	-0.079904	-0.00495	-0.024343	0.04560636	0.14232236	-0.038272	-0.133162	0.09826340	0.01283836	0.06322577	0.00253280	0.09467942	1	0.08300200	0.06992087	-0.040695	0.0305782982977568
Neutrophi	0.00709908	-0.065131	-0.035455	-0.056789	-0.128053	-0.008427	-0.029749	-0.025362	-0.111978	-0.15931	0.06604093	0.03415378	0.04330140	0.03319555	-0.036833	-0.127001	0.20621207	-0.045062	0.09397335	0.01756173	0.04537326	0.08300200	1	0.09880339	-0.047998	0.0324425658118826
P.value	0.04587728	-0.377681	0.01957574	-0.306427	0.00312741	-0.017399	0.07969775	0.06441371	0.06178839	-0.008716	0.02156729	0.05918694	0.05853645	0.08651856	0.01134064	0.05316673	0.13352657	-0.019216	0.09572644	-0.009743	0.00271038	0.06992087	0.09880339	1	-0.66768	0.625540862095903
Correlatio	-0.120299	0.43377807	0.01077124	0.48233905	-0.110346	0.02405837	-0.160867	-0.115941	-0.175289	-0.02418	-0.06009	-0.017203	-0.056682	-0.094981	0.08674258	-0.227121	-0.089659	-0.032117	-0.071054	-0.032432	0.03001248	-0.040695	-0.047998	-0.66768	1	-0.971089
RMSE	0.12225305	-0.428582	0.00029229	-0.462979	0.09790617	-0.023377	0.10671511	0.10780409	0.18252450	0.03668934	0.05701113	0.03663584	0.05132374	0.02784484	0.03702180	0.21068050	0.03706427	-0.020246	0.07717524	-0.045342	-0.032144	0.03057829	0.03244256	0.62554086	-0.971089	1

Table S4 The correlation analysis between IFI6 and immune cells (adjusted P-values)

	IFI6	B.cells.na	B.cells.me	Plasma.ce	T.cells.C	T.cells.C	T.cells.C	T.cells.C	T.cells.f	T.cells.r	T.cells.g	NK.cells.	NK.cells.	Monocytes	Macrophag	Macrophag	Macrophag	Dendritic.	Dendritic.	Mast.cell	Mast.cell	Eosinophi	Neutrophi	P.value	Correlati	RMSE
IFI6	0	1.3824920	0.6033863	3.8623672	0.1992711	0.5644990	0.0001793	2.8627559	4.6490732	5.9438216	0.4825480	0.1566417	0.3684580	0.1929593	0.0023637	3.9773602	0.0017669	0.0095361	6.2787662	8.7190408	0.0001869	0.0115635	0.8038877	0.1083690	2.4084204	1.76794941846434e-05
B.cells.na	1.3824920	0	7.1775517	2.0060608	0.0020749	0.6324805	2.7816138	0.0003968	0.2865734	0.0221120	0.1443147	2.1009075	0.4644755	0.0066926	7.3542285	0.0273255	2.0198472	0.3534947	0.0007109	0.1470850	0.2513353	0.3255323	0.0225717	7.4753074	2.0604979	6.05543858177499e-56
B.cells.me	0.6033863	7.1775517	0	0.3500398	0.5949001	0.2579018	0.0185299	0.2179949	0.3195363	2.2689359	0.2150659	0.0086584	0.2859908	0.6563031	0.4256890	0.0006320	0.0312706	0.6397798	0.9076200	0.3718322	0.3021335	0.5385531	0.2147691	0.4934719	0.7063418	0.991842471880404
Plasma.ce	3.8623672	2.0060608	0.3500398	0	0.1800553	0.8280435	0.0348906	0.1544142	0.0206333	0.0994782	0.0628117	0.0008809	0.0286736	0.2162427	8.0652516	3.3916436	1.1086621	0.0419030	0.0219839	0.5373018	0.2383182	0.2293779	0.0468118	4.5794966	1.9459667	3.72788822515393e-66
T.cells.C	0.1992711	0.0020749	0.5949001	0.1800553	0	0.6275868	0.2341905	1.1791555	2.7033736	1.5643159	0.4154522	1.1283174	0.0053450	0.5179715	9.7043788	1.5224082	1.3440605	0.0842412	0.0090043	9.6719164	0.0003521	0.0889599	6.8713537	0.9128911	0.0001081	0.000597233181964089
T.cells.C	0.5644990	0.6324805	0.2579018	0.8280435	0.6275868	0	0.1178725	0.5709225	0.0940388	0.4412763	0.6142750	0.1429895	0.6426313	7.9663378	0.1816172	0.1813392	0.0332220	0.2206610	0.6326822	0.9220449	0.7772439	0.2918136	0.7681757	0.5427661	0.3999843	0.413476240533422
T.cells.C	0.0001793	2.7816138	0.0185299	0.0348906	0.2341905	0.1178725	0	0.1162970	8.9318406	5.4569387	0.2876028	0.7712983	2.1355790	0.0007827	4.5356054	1.1080682	0.0004607	0.0281845	0.0236896	0.7295650	0.9909426	0.6778040	0.2979717	0.0052358	1.4802777	0.000181521036669545
T.cells.C	2.8627559	0.0003968	0.2179949	0.1544142	1.1791555	0.5709225	0.1162970	0	6.4532301	0.0080772	0.0026082	7.1753537	5.6296408	2.1883856	3.4684539	1.5331938	1.0413020	0.6805892	0.9312534	7.5803015	0.0049240	0.2433414	0.3749322	0.0241055	4.7177929	0.000155661651846357
T.cells.f	4.6490732	0.2865734	0.3195363	0.0206333	2.7033736	0.0940388	8.9318406	6.4532301	0	5.4800122	0.5100062	1.0271306	2.6806101	0.0004244	0.2006665	1.3474141	5.2750798	0.7528483	1.5849797	2.0387714	0.0497010	0.0902164	8.5233245	0.0305153	6.4405381	1.20576117748383e-10
T.cells.r	5.9438216	0.0221120	2.2689359	0.0994782	1.5643159	0.4412763	5.4569387	0.0080772	5.4800122	0	0.0005567	0.9507824	0.0022835	1.7365616	0.0059197	1.2955896	6.1811568	0.0296764	0.0043966	7.8015692	6.4588552	0.0051198	2.0433462	0.7604657	0.3976034	0.199221388050358
T.cells.g	0.4825480	0.1443147	0.2150659	0.0628117	0.4154522	0.6142750	0.2876028	0.0026082	0.5100062	0.0005567	0	0.0679990	0.4688735	0.0024476	6.8083555	0.2603012	2.5284253	0.5379587	0.5480303	0.1329979	0.0118468	0.8625472	0.0207478	0.4505605	0.0353979	0.0459566782193446
NK.cells.	0.1566417	2.1009075	0.0086584	0.0008809	1.1283174	0.1429895	0.7712983	7.1753537	1.0271306	0.9507824	0.0679990	0	9.8095261	0.9515821	5.3221633	0.1720801	0.5032000	0.0084387	0.7905630	1.0198479	0.7175465	0.3944352	0.2320873	0.0382569	0.5473305	0.199877809965467
NK.cells.	0.3684580	0.4644755	0.2859908	0.0286736	0.0053450	0.6426313	2.1355790	5.6296408	2.6806101	0.0022835	0.4688735	9.8095261	0	0.4466371	1.4274096	0.0338255	0.4983301	0.7475362	0.1554875	8.0524603	0.0001072	0.1104719	0.1296884	0.0404348	0.0472296	0.0724285138359287
Monocytes	0.1929593	0.0066926	0.6563031	0.2162427	0.5179715	7.9663378	0.0007827	2.1883856	0.0004244	1.7365616	0.0024476	0.9515821	0.4466371	0	1.4983184	1.5433872	5.1036830	0.0462913	1.0382606	1.7521146	0.4839943	5.6233344	0.2454577	0.0024295	0.0008690	0.329976317059044
Macrophag	0.0023637	7.3542285	0.4256890	8.0652516	9.7043788	0.1816172	4.5356054	3.4684539	0.2006665	0.0059197	6.8083555	5.3221633	1.4274096	1.4983184	0	3.3386843	7.4654376	1.0725802	0.9142040	3.6086334	0.5274946	0.1805081	0.1974692	0.6915942	0.0023668	0.195177583997325
Macrophag	3.9773602	0.0273255	0.0006320	3.3916436	1.5224082	0.1813392	1.1080682	1.5331938	1.3474141	1.2955896	0.2603012	0.1720801	0.0338255	1.5433872	3.3386843	0	4.9627393	0.1042630	1.4205272	1.1249861	2.6594958	2.8874968	8.1812483	0.0627424	8.2940679	9.11661660358565e-14
Macrophag	0.0017669	2.0198472	0.0312706	1.1086621	1.3440605	0.0332220	0.0004607	1.0413020	5.2750798	6.1811568	2.5284253	0.5032000	0.4983301	5.1036830	7.4654376	4.9627393	0	4.7415023	0.4615152	6.1427649	0.0001487	0.0005701	3.0650702	2.7108825	0.0016752	0.194665350470663
Dendritic.	0.0095361	0.3534947	0.6397798	0.0419030	0.0842412	0.2206610	0.0281845	0.6805892	0.7528483	0.0296764	0.5379587	0.0084387	0.7475362	0.0462913	1.0725802	0.1042630	4.7415023	0	0.8464792	0.0121632	0.6374806	0.6533695	0.1147954	0.5014589	0.2611396	0.478784968958549
Dendritic.	6.2787662	0.0007109	0.9076200	0.0219839	0.0090043	0.6326822	0.0236896	0.9312534	1.5849797	0.0043966	0.5480303	0.7905630	0.1554875	1.0382606	0.9142040	1.4205272	0.4615152	0.8464792	0	8.6760624	0.5482106	0.0268451	0.0009865	0.0007905	0.0128274	0.00686109531843134
Mast.cell	8.7190408	0.1470850	0.3718322	0.5373018	9.6719164	0.9220449	0.7295650	7.5803015	2.0387714	7.8015692	0.1329979	1.0198479	8.0524603	1.7521146	3.6086334	1.1249861	6.1427649	0.0121632	8.6760624	0	6.3981534	0.9294046	0.5389961	0.7332538	0.2564914	0.112558256341814
Mast.cell	0.0001869	0.2513353	0.3021335	0.2383182	0.0003521	0.7772439	0.9909426	0.0049240	0.0497010	6.4588552	0.0118468	0.7175465	0.0001072	0.4839943	0.5274946	2.6594958	0.0001487	0.6374806	0.5482106	6.3981534	0	0.0009027	0.1123068	0.9244692	0.2937042	0.260746680176147
Eosinophi	0.0115635	0.3255323	0.5385531	0.2293779	0.0889599	0.2918136	0.6778040	0.2433414	0.0902164	0.0051198	0.8625472	0.3944352	0.1104719	5.6233344	0.1805081	2.8874968	0.0005701	0.6533695	0.0268451	0.9294046	0.0009027	0	0.0036338	0.0143361	0.1544296	0.284693493648264
Neutrophi	0.8038877	0.0225717	0.2147691	0.0468118	6.8713537	0.7681757	0.2979717	0.3749322	8.5233245	2.0433462	0.0207478	0.2320873	0.1296884	0.2454577	0.1974692	8.1812483	3.0650702	0.1147954	0.0009865	0.5389961	0.1123068	0.0036338	0	0.0005312	0.0929851	0.256336258169479
P.value	0.1083690	7.4753074	0.4934719	4.5794966	0.9128911	0.5427661	0.0052358	0.0241055	0.0305153	0.7604657	0.4505605	0.0382569	0.0404348	0.0024295	0.6915942	0.0627424	2.7108825	0.5014589	0.0007905	0.7332538	0.9244692	0.0143361	0.0005312	0	4.5571759	4.12456405675574e-134
Correlati	2.4084204	2.0604979	0.7063418	1.9459667	0.0001081	0.3999843	1.4802777	4.7177929	6.4405381	0.3976034	0.0353979	0.5473305	0.0472296	0.0008690	0.0023668	8.2940679	0.0016752	0.2611396	0.0128274	0.2564914	0.2937042	0.1544296	0.0929851	4.5571759	0	0
RMSE	1.7679494	6.0554385	0.9918424	3.7278882	0.0005972	0.4134762	0.0001815	0.0001556	1.2057611	0.1992213	0.0459566	0.1998778	0.0724285	0.3299763	0.1951775	9.1166166	0.1946653	0.4787849	0.0068610	0.1125582	0.2607466	0.2846934	0.2563362	4.1245640	0	0
